# Ultra-high density intra-specific genetic linkage maps accelerate identification of functionally relevant molecular tags governing important agronomic traits in chickpea

**DOI:** 10.1038/srep09468

**Published:** 2015-05-05

**Authors:** Alice Kujur, Hari D. Upadhyaya, Tanima Shree, Deepak Bajaj, Shouvik Das, Maneesha S. Saxena, Saurabh Badoni, Vinod Kumar, Shailesh Tripathi, C. L. L. Gowda, Shivali Sharma, Sube Singh, Akhilesh K. Tyagi, Swarup K. Parida

**Affiliations:** 1 National Institute of Plant Genome Research (NIPGR), Aruna Asaf Ali Marg, New Delhi 110067, India; 2 International Crops Research Institute for the Semi-Arid Tropics (ICRISAT), Patancheru 502324, Telangana, India; 3 National Research Centre on Plant Biotechnology (NRCPB), New Delhi 110012, India; 4 Division of Genetics, Indian Agricultural Research Institute (IARI), New Delhi 110012, India

## Abstract

We discovered 26785 and 16573 high-quality SNPs differentiating two parental genotypes of a RIL mapping population using reference *desi* and *kabuli* genome-based GBS assay. Of these, 3625 and 2177 SNPs have been integrated into eight *desi* and *kabuli* chromosomes, respectively in order to construct ultra-high density (0.20–0.37 cM) intra-specific chickpea genetic linkage maps. One of these constructed high-resolution genetic map has potential to identify 33 major genomic regions harbouring 35 robust QTLs (PVE: 17.9–39.7%) associated with three agronomic traits, which were mapped within <1 cM mean marker intervals on *desi* chromosomes. The extended LD (linkage disequilibrium) decay (~15 cM) in chromosomes of genetic maps have encouraged us to use a rapid integrated approach (comparative QTL mapping, QTL-region specific haplotype/LD-based trait association analysis, expression profiling and gene haplotype-based association mapping) rather than a traditional QTL map-based cloning method to narrow-down one major seed weight (SW) robust QTL region. It delineated favourable natural allelic variants and superior haplotype-containing one seed-specific candidate embryo defective gene regulating SW in chickpea. The ultra-high-resolution genetic maps, QTLs/genes and alleles/haplotypes-related genomic information generated and integrated strategy for rapid QTL/gene identification developed have potential to expedite genomics-assisted breeding applications in crop plants, including chickpea for their genetic enhancement.

Chickpea (*Cicer arietinum* L.), the second most grown legume crop world-wide is an annual self-pollinated and diploid (2n = 2x = 16) pulse species with a genome size of ~740 Mb^1^,^2^. A member of Fabaceae family, this diploid food legume is a major source of human dietary protein packed with essential amino acids^1^. Chickpea is broadly categorized into two cultivars- *Kabuli* and *Desi*- types basing upon their plant characteristics and diverse gene pools-based geographical distribution. They are known to enrich the soil nutritional status and fertility by symbiotic nitrogen fixation. The seed and pod traits are considered the most prominent characteristics of chickpea defining its economic value as a diet for human race. Consequently, they draw major interest of researchers for yield and product quality improvement towards generation of high-yielding genetically tailored chickpea cultivars via genomics-assisted breeding^3^.

A large genome with a narrow genetic base, chickpea requires numerous informative and genome-wide well-distributed SNP (single nucleotide polymorphism) markers for construction of ultra-high density genetic linkage map (≤1 cM average map-density^4^,^5^) in order to identify and fine-map the useful trait-influencing candidate genes/QTLs (quantitative trait loci) for marker-assisted genetic enhancement. A number of high-resolution inter-specific genetic linkage maps (with mean map-density: 0.59–1.7 cM) have been generated in chickpea by genotyping of large-scale SNP markers in its diverse mapping populations^6^,^7^,^8^,^9^,^10^. Few recent efforts have also been made to construct SNP marker-based high-density genetic linkage maps (with map density: 1.74–3.68 cM) by utilizing diverse advanced generation *desi* and *kabuli* intra-specific mapping populations^11^,^12^,^13^,^14^. Indeed, all these studies have utilized high-throughput genotyping assays like Illumina GoldenGate/Infinium and Competitive Allele Specific PCR (KASPar) for large-scale validation and genotyping of SNP markers (~1000 SNPs till now) in intra- and inter-specific chickpea mapping populations to construct high-density genetic linkage maps. Aside chickpea^8^,^9^, similar efforts have also been undertaken to construct comprehensive high-density genetic linkage map (average inter-marker distance ≤1 cM) by deploying aforesaid strategies in diverse legume species (soybean, *Medicago*, pigeonpea, common bean and mung bean)^15^,^16^,^17^,^18^,^19^,^20^,^21^,^22^,^23^,^24^,^25^. However, the need of prior information regarding SNP loci and their high-quality flanking sequence regions for genotyping of SNPs largely limits the use of these assays in both discovery and validation of SNPs at genome-wide scale for constructing efficient high-resolution genetic linkage maps in chickpea. It thus implicates the necessity of a suitable modern and fast advanced approach/assay for simultaneous large-scale genome-wide discovery and high-throughput genotyping of SNPs in diverse mapping populations to generate required ultra-high resolution genetic linkage maps in chickpea. Numerous past reports have established the essentiality of high-density linkage maps as references for more effective assembly and orientation of the high-quality sequenced scaffolds into the pseudomolecules corresponding the chromosomes of crop plant genomes^15^,^22^,^23^,^25^,^26^,^27^,^28^,^29^,^30^,^31^,^32^,^33^,^34^,^35^,^36^,^37^,^38^. Therefore, it is indispensable to generate ultra-high density genetic linkage maps enriched with a greater number of SNP markers for efficient anchoring of assembled scaffolds into the chickpea chromosomes. This is expected to overall greatly facilitate the chickpea genome assembly and sequencing efforts in near future.

Genotyping-By-Sequencing (GBS) is a simple, rapid and cost-effective next-generation sequencing (NGS)-based assay having potential for simultaneous large-scale discovery and genotyping of SNPs in diverse crop plants at a genome-wide scale^39^,^40^. The high-barcoded multiplexing capacity (pooling up to 384-barcoded accessions in a single sequencing lane) at methylation-sensitive RE (restriction endonuclease)-sites (like *APe*KI) have enhanced the efficiency of GBS assay in fast discovery and genotyping of genome-wide SNPs in larger mapping populations. This in turn made the GBS assay much more advantageous over other available high-throughput traditional genotyping assays for constructing ultra-high density genetic linkage maps and genome-wide high-resolution (major and minor effects QTLs) gene/QTL mapping in small diploid as well as large genome crop species, including rice, wheat, barley, sorghum, *Medicago* and soybean^24^,^41^,^42^,^43^,^44^,^45^,^46^,^47^,^48^,^49^,^50^. Considering its utility in many high-throughput marker-based genotyping applications in genomics-assisted breeding, the use of GBS assay for generating ultra-high resolution genome maps and molecular mapping of robust major as well as minor genes/QTLs with sub-optimal use of resources in a crop species like chickpea assumes significance. Nevertheless, very limited reports are available with regard to the use of GBS approach in discovery and genotyping of genome-wide SNPs in intra- and inter-specific mapping populations for constructing ultra-high resolution genetic linkage maps and fine mapping of QTLs regulating important agronomic traits in chickpea vis-à-vis other crop plants^22^,^45^,^50^,^51^.

Over the past few years, a number of QTLs associated with important agronomic traits, including yield component and stress tolerance traits have been identified and mapped on low/high-resolution intra- as well as inter-specific genetic linkage maps of chickpea^3^,^13^,^14^,^20^,^52^,^53^,^54^,^55^,^56^,^57^,^58^,^59^,^60^,^61^,^62^,^63^,^64^,^65^,^66^,^67^,^68^,^69^,^70^,^71^,^73^,^74^,^75^,^76^,^77^. However, very limited number of these QTLs could either be successfully validated in diverse genetic backgrounds of accessions across geographical locations/years or fine-mapped through map-based cloning for their utilization in marker-assisted genetic improvement of chickpea^13^,^14^,^76^. To expedite such process of identification and mapping of high-resolution and robust major and minor effect QTLs at genome-wide scale, the construction of SNP marker-based ultra-high density intra- and inter-specific genetic linkage maps using GBS assay at present is anticipated as an attractive approach for chickpea genetic enhancement.

Keeping all above in view, we discovered 26785 and 16573 genome-wide SNPs showing differentiation between parental genotypes [ICC 12299 (*desi*) and ICC 8261 (*kabuli*)] by their genotyping in a 275 F_7_ RIL (recombinant inbred line) mapping population (ICC 12299 × ICC 8261) using reference *desi* and *kabuli* genome-based GBS assay, respectively. A selected 3625 and 2177 high-quality SNPs of these, were integrated into eight *desi* and *kabuli* chromosomes, respectively to construct ultra-high density intra-specific genetic linkage maps in chickpea. These high resolution genetic maps were further used as a reference to identify and map the novel major genomic regions underlying robust QTLs associated with three important agronomic traits in chickpea. To delineate functionally relevant candidate gene(s) regulating seed weight, one of the strong trait-associated major genomic region harbouring robust QTL has been narrowed down by integrating comparative *desi* and *kabuli* genome-based QTL mapping with QTL region-specific haplotype (linkage disequilibrium)-based high-resolution trait association analysis, differential gene expression profiling and gene haplotype-based association mapping in chickpea.

## Results

### Discovery and large-scale validation of GBS-based genome-wide SNPs

**Figure 1:**
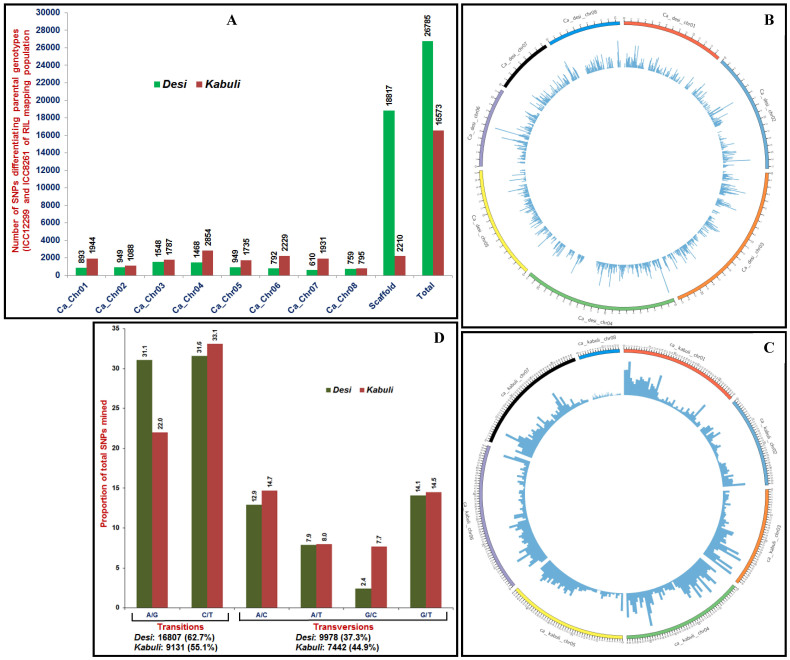
(A) Relative distribution and frequency of 43358 SNPs discovered in eight chromosomes and scaffolds of *desi* and *kabuli* chickpea using reference genome-based GBS assay. Distribution of 22331 SNPs physically mapped on eight *desi* (B) and *kabuli* (C) chromosomes are depicted in the Circos circular ideogram. The innermost circles display the distribution of SNPs identified through GBS assay, while outermost circles represent the eight chickpea chromosomes coded with different colours. (D) Proportionate distribution of transition and transversion SNPs identified using the reference *desi* and *kabuli* genomes-based GBS assays.

We generated an average of 207.3 million high-quality sequence reads (ranged from 1.9 to 8.3 with a mean of 3.4 million reads per individual) (Fig. S1) by sequencing of each 3 × 96-plex (288-plex) *Ape*KI libraries made from 275 RIL mapping individuals and two parental genotypes (ICC 12299 × ICC 8261) using GBS assay. However, 84.5 and 82.6% of sequence reads of these, were mapped to unique physical location of *desi* and *kabuli* reference genomes, respectively. It identified 43358, including 26785 and 16573 high-quality SNPs [with read-depth ≥10, SNP base quality ≥20, <10% missing data in each individual, 5% minor allele frequency (MAF) and 100% reproducibility] differentiating two parental genotypes using the reference *desi* and *kabuli* genome-based GBS assay, respectively (Fig. 1A). Of these, 22331, including 7968 and 14363 SNPs were physically mapped on eight chromosomes of *desi* and *kabuli* genomes with average map-densities of 15.6 and 24.2 kb, respectively (Table S1, Fig. 1A, B, C). A higher proportion of SNPs were mapped on *desi* and *kabuli* chromosomes 3 (19.4%, 1548 SNPs) and 4 (19.9%, 2854), respectively. The *desi* chromosomes 7 and 8 (13.9 kb each) and *kabuli* chromosome 4 (17.2 kb) had maximum mean map-density (Table S1). The remaining 21027 SNPs, including 18817 and 2210 SNPs were physically mapped on scaffolds of *desi* and *kabuli* genomes, respectively (Fig. 1A). The A/G and C/T transitions (62.7 and 55.1% of total SNPs identified in *desi* and *kabuli*, respectively) were more abundant compared with that of transversions (Fig. 1D). Detail characteristics of 43358 SNPs discovered in our study are provided in the Table S2. Of these, 16573 SNPs discovered by *kabuli* genome-based GBS assay have been submitted to NCBI dbSNP (http://www.ncbi.nlm.nih.gov/SNP) with SNP submission (SS) accession numbers 1067289484 to 1067306056 for unrestricted use [NCBI db SNP Build (B142) year 2014 (http://www.ncbi.nlm.nih.gov/SNP/snp_viewTable.cgi?handle=NIPGR)].

Four hundred-eighty SNPs mined by reference *desi* and *kabuli* genome-based GBS assay were targeted for their validation through resequencing of PCR amplicons (384 SNPs) and MALDI-TOF mass array (96 SNPs). It validated 437 (91%) SNPs successfully in parental genotypes and selected mapping individuals of a RIL population (ICC 12299 × ICC 8261) (Fig. S2A, B). Remarkably, all these validated SNPs revealed expected homozygous and heterozygous alleles discrimination in RILs as detected through our GBS assay.

### Construction of ultra-high density intra-specific genetic linkage maps

The genotyping information of 4448 and 2689 high-quality SNPs (MAF ≥ 20%) discovered by reference *desi* and *kabuli* genome-based GBS assay, respectively (Table S3) in parental genotypes and 275 individuals of a RIL mapping population (ICC 12299 × ICC 8261) were used to construct ultra-high density intra-specific genetic linkage maps in chickpea. The linkage analysis of SNPs showing significant Mendelian segregation ratio (1:1) mapped 3625 and 2177 SNPs on eight *desi* and *kabuli* linkage groups (LGs) (LG1 to LG8) of intra-specific genetic maps, respectively according to their physical positions (bp) on corresponding chromosomes (Table 1, Fig. S3, S4, S5). Considering significant variations in the genome assembly and differences in sequence coverage and length (bp) of chromosomal pseudomolecules between *desi* and *kabuli* genomes^78^, we preferred to construct two genetic maps separately by using *desi* and *kabuli* genome-based high-quality GBS-SNPs and their corresponding physical positions on eight chromosomes as reference. The constructed *desi* and *kabuli* SNP-based ultra-high density intra-specific genetic maps comprising of eight LGs covered a total map length of 714.089 and 798.467 cM with mean inter-marker distances of 0.20 and 0.37 cM, respectively (Table 1). Maximum numbers of SNPs were mapped on *desi* LG1 (736 SNPs) and *kabuli* LG3 (463), while *desi* LG8 (281) and *kabuli* LG5 (104) contained minimum numbers of SNPs (Table 1). The map length spanned by each LG of *desi* ranged from 85.662 cM (386 SNPs) in LG2 to 97.782 cM (574) in LG4, while for *kabuli*, it varied from 81.444 cM (292 SNPs) in LG5 to 120.359 cM (386) in LG2 (Table 1). In *desi*, most saturated genetic map was LG1 (average inter-SNP marker distance: 0.12 cM), whereas LG5 and LG8 were least saturated (0.32 cM). In *kabuli*, LG3 (mean inter-SNP marker distance: 0.18 cM) and LG5 (0.78 cM) had most and least saturated genetic maps, respectively (Table 1).

### Estimation of genome-wide LD patterns in chickpea

The genome-wide LD estimates (r^2^) and extent of LD decay using all possible pair-combinations of 3625 and 2177 SNPs genetically mapped on eight *desi* and *kabuli* LGs of intra-specific genetic maps, respectively were determined. The average LD estimates in eight LGs of *desi* genome (r^2^: 0.62) was almost comparable with that of *kabuli* genome (0.59) (Table S4). The LG4 of both *desi* (r^2^: 0.78) and *kabuli* (0.72) genetic maps had highest LD estimates. Maximum proportion of SNP-pairs showed significant LD (P < 0.0001) on *desi* LG4 (42.8%) and *kabuli* LG2 (34.6%) (Table S4). We determined the LD decay of 3625 and 2177 SNP-pairs by pooling the r^2^ estimates across eight *desi* and *kabuli* LGs and plotting their average r^2^ against the 5 cM equal intervals of genetic distance (maximum up to 120 cM). A decreasing trend of LD decay (r^2^ < 0.3) was observed with increase in the genetic distance (cM) of SNP markers mapped on the *desi* and *kabuli* LGs (Fig. S6). Remarkably, a rapid LD decay was observed at the genetic distance of 5 cM in both *desi* and *kabuli* genomes. The *desi* and *kabuli* LGs overall sustained a significant level of LD up to a genetic distance of 10 cM. A significant LD decay (r^2^ < 0.1) was observed near about 15 cM genetic distance (Fig. S6) in LGs of these two genomes.

### Identification and mapping of high-resolution trait-governing QTLs

A significant difference of three quantitative agronomic traits, namely pod number/plant (PN) (51.1–141.2 with 79–80% heritability), seed number/plant (SN) (60.6–231.6 with 77–81%) and 100-seed weight SW (7.1–43.7 g with 88–90%, Fig. S7) in 275 RIL mapping individuals and parental genotypes across three years based on ANOVA was observed (Table S5). We observed bi-directional transgressive segregation of these traits beyond that of parental genotypes in RILs (Fig. S8). The normal frequency distribution of all three quantitative traits in RILs and parental genotypes was evident. The coefficient of variation (CV) ranged from 0.14 for PN to 0.33 in SW (Table S5). The Pearson's correlation coefficient estimated among three pair-wise combinations of quantitative traits indicated significant (P < 10^−2^) negative correlation of PN and SN with SW (r = −0.42 to −0.51) as well as positive correlation between PN and SN (0.95) (Fig. S9).

For genetic/QTL mapping, the genotyping data of 3625 parental polymorphic *desi*-genome based SNPs mapped on an ultra-high density intra-specific genetic linkage map was integrated with phenotyping data of 275 RIL mapping population. It identified and mapped 33 genomic regions harbouring 35 major and significant (LOD: 7.25–14.5) QTLs associated with PN, SN and SW on seven LGs (except LG3) of chickpea (Table 2, Fig. S10, Fig. S11). The proportion of phenotypic variance explained (PVE) by individual QTL varied from 17.9–39.7%. Collectively, all these QTLs revealed 37.3% PVE. All these identified QTLs (considered as robust QTL) showing consistent phenotypic expression and major effects on traits individually with PVE of > 10% each, were validated across two geographical locations as well as years/seasons in field. A maximum number of QTLs (nine QTLs) were mapped on LG1 followed by LGs 2, 6 and 7 (5 QTLs each) and minimum (3 QTLs) on LG5 (Table 2). The genomic regions (from 0.015 cM on LG4 to 1.978 cM on LG1) harbouring the QTLs covered with 106 SNP markers were mapped on seven LGs.

For PN, 16 major genomic regions underlying 17 robust QTLs (PVE: 20.7–35% and LD: 7.25–14.0) were identified and mapped on six LGs. Collectively, all these QTLs showed 36.4% PVE. The PN QTL regions covered with 57 SNP markers on LGs (0.015 cm on LG4 to 1.978 cM on LG1) showed mostly the positive additive gene effects indicating the effective contributions of ICC 12299 alleles at these loci for increasing pod number in chickpea. For SN, eight major genomic regions harbouring eight robust QTLs explaining 21.7–38.1% PVE (LOD: 7.44–12.6) were identified. These SN QTLs covered with 24 SNPs (0.072 cM on LG2 to 1.083 cM on LG5) were mapped on four LGs (Table 2 and Fig. S10). The combined PVE estimated for all these QTLs was 35.8%. The QTLs showed positive additive gene effects for increasing seed number with large allelic contribution from ICC 12299. For SW, nine major genomic regions harbouring 10 robust QTLs covered with 27 SNP markers (0.115 cM on LG1 to 1.07 cM on LG5) were mapped on six LGs explaining 23.2–39.7% PVE (LOD: 7.4–14.5) (Table 2 and Fig. S10). The combined PVE for all these QTLs was 39.4%. These QTLs showed positive additive gene effects for increasing seed weight with allelic contributions from ICC 8261.

### Structural and functional annotation of SNPs mapped on intra-specific genetic linkage maps

**Figure 2:**
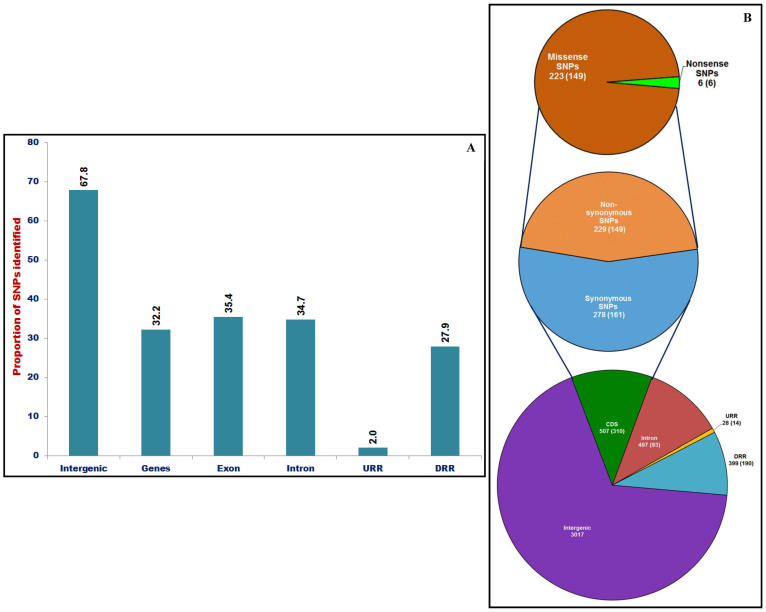
(A) Proportionate distribution of 4448 SNPs identified in different coding and non-coding sequence components and intergenic regions of 635 genes annotated from *desi* chickpea genome. (B) Number of SNPs, including synonymous and non-synonymous (missense and nonsense) SNPs annotated in the coding (CDS) as well as non-coding (intronic and regulatory) sequences of genes and intergenic regions of *desi* genome. The URR (upstream regulatory region) and DRR (downstream regulatory region) of genes were defined as 2000-bp upstream sequences from the translation start codon (ATG) and 1000-bp downstream sequences from the stop codon, respectively.

The structural annotation of 4448 and 2689, including 3625 and 2177 high-quality *desi* and *kabuli* GBS-SNPs mapped on intra-specific genetic maps revealed the presence of 3017 and 1349 SNPs in the intergenic regions, while 1431 and 1340 SNPs in the different sequence components of 635 and 760 genes, respectively (Table S6A and B). The detailed annotation of SNPs within genes identified maximum frequency (35.4%, 507 SNPs in *desi* and 49.4%, 662 SNPs in *kabuli*) of SNPs in the exons and minimum in the 1000-bp downstream regulatory regions (DRR) (27.9%, 399 and 17.7%, 238) (Fig. 2A and S12A). Remarkably, 278 and 229 coding SNPs-containing *desi* genes, while 352 and 328 coding SNPs-carrying *kabuli* genes showed synonymous and non-synonymous (missense and nonsense) substitutions, respectively (Fig. 2B and S12B). The non-synonymous SNPs identified in *desi* and *kabuli* comprised of 223 and 323 missense SNPs in the 149 and 234 genes showing amino acid substitutions as well as six and five nonsense SNPs in the six and five genes, respectively culminating into premature termination codons introduced by nucleotide replacements (Fig. 2B and S12B). Maximum (5.3 SNPs/gene) and minimum (1.6 SNPs/gene) average frequency of SNPs was observed in the non-coding intronic and coding sequence components of *desi* genes, respectively. In contrast, we observed comparable average SNP frequency between intronic (1.7 SNPs/gene) and coding (1.9) sequence components of *kabuli* genes.

The functional annotation of 635 *desi* and 760 *kabuli* genes with SNPs detected a higher proportion of SNPs belonging to growth, development and metabolism-related proteins (58 and 63% in *desi* and *kabuli*, respectively) followed by transcription factors (16 and 18%) (Fig. S13A). The KOG-based determination of putative functions (excluding unknown and general functions) for SNPs-carrying genes revealed their maximum correspondence to transcription (K, 25% in *desi* and 31% in *kabuli*) (Fig. S13B). The GO enrichment analysis of SNPs-carrying *desi* and *kabuli* genes showed a significant (P ≤ 5.9 × 10^−3^) overrepresentation/enrichment of GO terms in the genes associated with transport (15.4%) belonging to biological process (Fig. S13C).

### QTL region-specific haplotype (LD)-based trait association mapping

**Figure 3:**
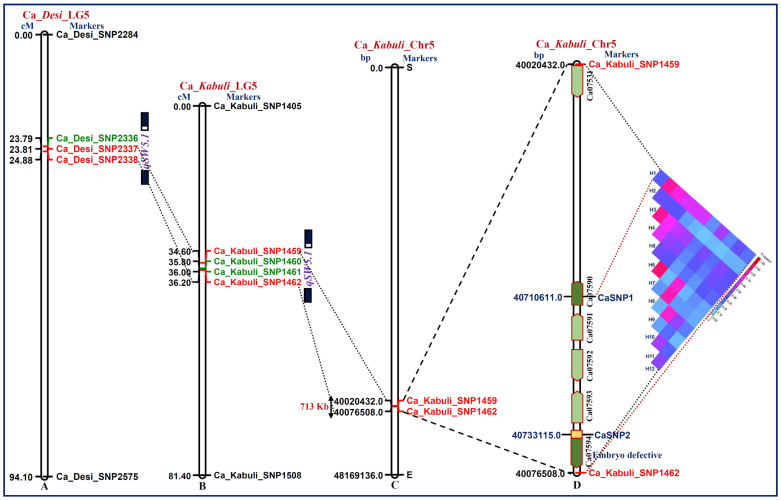
Integration of *desi* (A) and *kabuli* (B) genetic maps with *kabuli* physical (C) map of one major genomic region harbouring robust SW QTL (*qSW5.1*) mapped target QTL on 713 kb sequence interval of chromosome 5. This QTL was further narrowed down to about 22.5 kb sequenced region on chromosome 5 by integrating QTL mapping with QTL region-specific haplotype (LD)-based high-resolution trait association analysis (D). The 22.5 kb genomic region between the markers CaSNP1 and CaSNP2 contained five candidate protein-coding genes, of which one of the regulatory SNP-carrying embryo defective *kabuli* gene (Ca07594) showed strong association with SW in chickpea (D). The genetic (cM) and physical (bp) distance and identity of the markers mapped on the LGs/chromosomes are indicated on the left and right side of the LGs/chromosomes, respectively.

One major and robust (LOD: 14.5 and R^2^: 39.7%) QTL (*qSW5.1*) region [Ca_*Desi*_SNP2337 (23.805 cM)-Ca_*Desi*_SNP2338 (24.875 cM)] governing SW mapped on *desi* LG5 of intra-specific genetic map was selected for fine mapping through QTL target-specific haplotype (LD)-based high-resolution trait association mapping (Fig. S10 and Fig. 3A). The genomic region underlying such a major QTL was defined by integrating the genetic linkage map information with that of physical map of *desi* genome. The target QTL interval genetically mapped on *desi* LG5 was compared and correlated with that mapped on *kabuli* LGs of a genetic map (based on QTL mapping). In *kabuli*, the SW trait-influencing QTL region spanned with 1.6 cM genetic interval between the markers Ca_*Kabuli*_SNP1459 (34.6 cM) and Ca_*Kabuli*_SNP1462 (36.2 cM) was mapped on LG5 (Fig. 3B). The integration of genetic linkage map information with physical map of *kabuli* genome, the 1.6 cM QTL interval corresponded to 713 kb genomic region (40020.4–40733.4 kb) on chromosome 5 (Fig. 3C).

The targeted resequencing of this 713 kb *qSW5.1* QTL region in parental genotypes (ICC 12299 and ICC 8261) and 10 of each homozygous low and high seed weight RIL mapping individuals detected 804 high-quality SNPs (mean SNP density: 1/886.8 bp). It includes 435 intergenic SNPs and 369 SNPs derived from the diverse coding and non-coding sequence components of 64 genes. The decay of LD over longer genetic distance (~15 cM, Fig. S6) particularly at the target SW QTL interval mapped on *kabuli* LG5 gave us clue to narrow-down this QTL region of interest using SNP haplotype (LD)-based high-resolution trait association mapping. The genotyping of 804 SNPs identified and mapped on the 713 kb *qSW5.1* QTL region in 244 accessions belonging to an SW-specific association panel (Table S7) enabled to constitute 12 haplotypes (Fig. 3D). The SNP haplotype-based genotyping information were further correlated with their SW-specific robust field phenotyping data (SW: 5.9–57.6 g) for QTL region-specific trait association analysis. The integration of GLM and MLM analysis with EMMA and false discovery rate (FDR) correction based on multiple-comparisons (minimizing the confounding effect of population structure) identified one best haplotype (H1) between two SNPs (CaSNP1 and CaSNP2) in two genes (Ca07590 and Ca07594) showing strong association (P: 2–4 × 10^−6^ and R^2^: 37.6–40.3%) with SW in contrast to any other SNP combinations. This haplotype region covered a maximum of 22.5 kb (40710.6–40733.1 kb) physical distance between CaSNP1 and CaSNP2 at *qSW5.1* QTL interval. The structural and functional annotation of this 22.5 kb sequenced short QTL region with *kabuli* genome annotation database identified five protein-coding candidate genes (Fig. 3D). The comprehensive analysis of strong SW-associated haplotypes-constituting two genic SNPs detected one regulatory SNP (CaSNP2: C/T) in the URR (upstream regulatory region) of an embryo defective protein-coding gene showing strong association (P: 2.0 × 10^−5^ and R^2^ = 39.8%) with SW.

### Expression profiling of SW-associated genes

To infer the differential regulatory gene expression patterns, the expression profiling of five protein-coding candidate genes annotated in the 22.5 kb delineated SW-specific robust QTL interval (*qSW5.1*) was performed. The gene-based primers were amplified using the RNA isolated from three different vegetative and reproductive tissues and two seed developmental stages of low and high SW parental genotypes (ICC 12299 and ICC 8261) of a RIL mapping population through quantitative RT-PCR assay (Fig. S14). One regulatory SNP-carrying embryo defective gene of these, constituting strong SW-associated haplotypes at *qSW5.1* QTL interval revealed seed-specific expression (as compared to vegetative and reproductive tissues) as well as pronounced down-regulation (>7-fold) in parental genotypes during seed development (Fig. S14). Henceforth, this gene localized in a major SW-influencing QTL interval (*qSW5.1*) was selected as target candidate for understanding its significance in seed weight regulation through high-resolution gene haplotype-specific association/LD mapping in chickpea.

### Haplotype-based association mapping in a strong SW-associated gene

**Figure 4:**
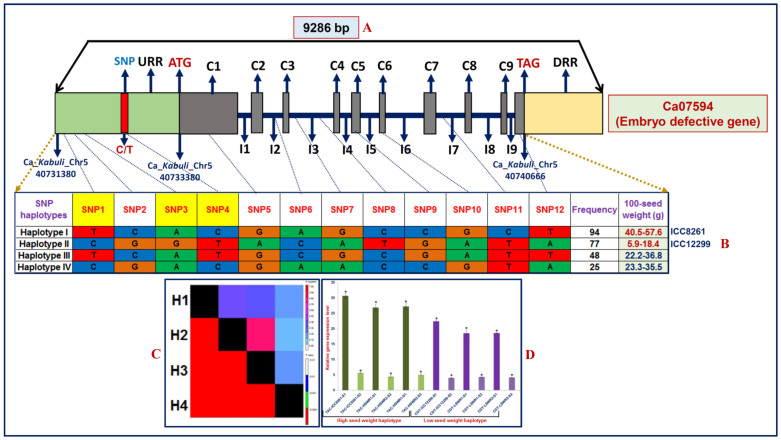
The molecular haplotyping, LD mapping and gene haplotype-specific trait association analysis in an embryo defective gene validating its strong association potential for SW in chickpea. The genotyping of 12 SNPs, including one regulatory SNP (C/T) in the URR of this gene (A) among 244 association panel constituted four haplotypes (B). The four SNP haplotype marker-based genotyping information produced higher LD estimates covering the entire 9286 bp sequenced region of gene (C). A significant correlation of parental genotypes and RIL mapping individuals representing high (haplotype I: TAC) and low (haplotype II: CGT) SW haplotypes [constituted by three SNPs (shaded with yellow colour in B)] in URR of gene with their seed-specific diverse transcript expression (D) during seed development was evident. A superior favorable high SW-regulating haplotype (TAC) with decreased transcript expression was identified in the URR of gene (D).

The sequencing of 9286 bp cloned amplicon covering the entire coding and non-coding regions (including 2 kb URR) of a strong SW-influencing embryo defective gene (Fig. 4A) (validated by QTL mapping, QTL region-specific association analysis and differential expression profiling) among 244 accessions belonging to a SW-specific association panel identified 12 SNP loci (Fig. 4B). It includes four regulatory SNPs in the URR of this gene. The gene-based haplotype analysis combining the genotyping data of 12 SNPs constituted a maximum of four haplotypes among accessions (Fig. 4B).

The association analysis using the four SNP marker-based haplotypes in an embryo defective gene with SW (5.9 to 57.6 g)-specific multi-location replicated field phenotyping data of 244 accessions (association panel) revealed its strong association with SW (P: 2.5 × 10^−7^ and R^2^: 44.7%). The haplotype-pair-based LD estimation produced a significant high degree of LD (r^2^ > 0.70 and P < 0.0001) across entire 9286 bp sequenced gene (Fig. 4C), which increased its overall potential for trait association. The 94 high SW (40–57.6 g) accessions represented by single haplotype group 1 (TAC) in the gene differentiated distinctly from another haplotype group 2 (CGT) consisting 77 accessions of low SW (5.9–18.4 g) (Fig. 4B) with higher phenotypic variance (43.4–44.7% at P < 2.0 × 10^−6^) and thus strong association potential of this gene for SW in chickpea is expected. The differential expression profiling using these high (TAC) and low (CGT) SW-specific haplotypes constituted by three SNPs in URR of an embryo defective gene revealed significant down-regulated expression (~6 times) specifically in two seed developmental stages of low and high SW parental genotypes and homozygous RIL mapping individuals as compared to leaf. It inferred that favourable natural allelic variants and superior haplotype (TAC) constituted in the URR of an embryo defective gene involved in decreased transcript expression and thus have significance in regulation of seed development and consequently seed weight in chickpea.

## Discussion

We utilized NGS-based 3 × 96-plex GBS assay for large-scale validation and high-throughput genotyping of genome-wide SNPs in a 275 RIL mapping population (ICC 12299 × ICC 8261) to construct ultra-high density intra-specific genetic linkage maps in chickpea. The reference *desi* and *kabuli* genome-based GBS assay identified 26785 and 16573 high-quality SNPs (5% MAF) differentiating the two parental genotypes (ICC 12299 and ICC 8261) of a RIL mapping population with 100% reproducibility, respectively. Notably, 22331, including 7968 and 14363 SNPs of these, showed extensive genome coverage across eight *desi* and *kabuli* chromosomes with mean densities of 1/15.6 and 1/24.2 kb, respectively. A high experimental validation success rate (91%) of 480 randomly selected SNPs from a total of 43358 GBS-based SNPs by amplicon resequencing and MALDI-TOF mass array genotyping assay was evident. A number of studies in the past have accessed the potential of diverse NGS-based assay for efficient mining and genotyping of non-erroneous SNPs through experimental validation of randomly chosen smaller set (~1–2%) of SNPs from a whole genome SNP dataset in diverse crop plants, including chickpea^12^,^22^,^42^,^45^,^50^,^51^,^79^,^80^,^81^,^82^. The correspondence of on an average of 39% (16910 SNPs) *desi* and *kabuli* genome-derived GBS-SNPs with that of SNP allelic information available in various chickpea genotypes^8^,^9^,^12^,^22^,^37^,^51^ *in silico* based on their congruent physical positions was clearly evident. These findings overall suggest the robustness and utility of GBS assay for rapid large-scale mining and high-throughput genotyping of valid and high-quality SNPs at a genome-wide scale in chickpea. The advantages of GBS-assay in simultaneous genome-wide discovery and genotyping of SNPs and their ability to expedite various large-scale genotyping applications have been demonstrated in many crop plants^24^,^40^,^41^,^42^,^43^,^44^,^45^,^46^,^47^,^48^,^49^,^50^. Collectively, a large number of 43358 high-quality reference *desi* and *kabuli* genome-based GBS-SNPs (61% novel SNPs) developed by us (submitted to NCBI SNPdb for unrestricted use) could serve as a valuable genomic resource for their immense use in genomics-assisted breeding applications of chickpea.

An ultra-high density 3625 and 2177 SNP marker-based intra-specific genetic linkage maps (ICC 12299 × ICC 8261) with eight *desi* (mean inter-marker distance 0.20 cM) and *kabuli* (0.37 cM) LGs constructed in our study are highly saturated compared to the previously reported intra- and inter-specific genetic linkage maps (0.50–8.01 cM) in chickpea^6^,^7^,^8^,^9^,^10^,^22^,^51^. Remarkably, the currently generated genetic linkage maps had much lower marker map-density and thus highly saturated in contrast to latest available high-density intra-specific genetic linkage maps (0.50–3.68 cM) in chickpea^11^,^12^,^13^,^14^,^22^,^51^. Beside chickpea, the efficacy of different high-throughput genome-wide SNP discovery and genotyping approaches, including GBS assay for construction of ultra-high density genetic linkage maps have been well demonstrated in several other crop plants^4^,^15^,^16^,^17^,^18^,^19^,^20^,^21^,^22^,^23^,^24^,^25^,^43^. The ultra-high density genetic linkage maps have been successfully deployed as a reference for effective assembly, precise orientation and anchoring of high-quality scaffolds into pseudomolecules of the chromosomes arising out of whole genome sequencing efforts (finished and/or in progress) in many plant species^15^,^22^,^23^,^25^,^26^,^27^,^28^,^29^,^30^,^31^,^32^,^33^,^34^,^35^,^36^,^37^,^38^. Therefore, ultra-high density intra-specific genetic linkage maps constructed in our study can serve as a reference for efficient generation of integrated genetic, physical and genome map, including identification and fine-mapping of major as well as minor QTLs regulating important agronomic traits in chickpea.

A significant LD estimates across eight *desi* (0.62) and *kabuli* (0.59) LGs of constructed intra-specific genetic maps using mapped SNP markers reflected direct correlation of LD patterns with the density of SNPs required to cover the genomes. In spite of large differences in SNP marker map density between *desi* (0.20 cM) and *kabuli* (0.37) LGs of genetic maps, we observed higher LD estimates and extended LD decay (~15 cM) in LGs of both *desi* and *kabuli* genomes. This is much higher than the LD decay estimated in other self- and cross-pollinated crop plants^83^,^84^,^85^,^86^,^87^,^88^,^89^,^90^. It could be due to sequential bottlenecks during chickpea domestication resulting in its narrow genetic base in contrast to other crop plants^36^,^37^,^74^,^91^,^92^,^93^. Overall, the genome-wide LD patterns and constructed LD maps gave an approximate perception regarding the SNP density required to identify the functionally relevant trait-regulatory robust genes/QTLs in a large chickpea genome with narrow genetic base using genetic (fine-mapping and map-based cloning) and association mapping.

Our study utilized ‘α-lattice’ designs in multi-location experimental field trials to efficiently grow and comprehensively phenotype the 275 RIL mapping individuals (ICC 12299 × ICC 8261) along with their parental genotypes for three important yield component traits (PN, SN and SW) in chickpea. The ‘α-lattice’ designs are more preferred than the commonly adopted ‘randomized complete block design (RCBD)’, since they are less prone to experimental errors, cost-effective and provide more precise (~35% higher) phenotyping information in field experiments aside high reproducibility. The ‘α-lattice’ designs are also associated with smaller coefficient of variation and error mean square, which collectively gives them an edge over the traditionally used RCBD designs specifically when phenotyping of yield component trait variables was conducted for very large numbers of diverse crop genotypes in field experiments with a smaller plot size^94^,^95^,^96^.

Thirty-three major genomic regions harbouring 35 robust QTLs (LOD: 7.25–14.5 and PVE: 17.9–39.7%) associated with three important agronomic traits (SW, PN and SN) were identified and mapped on seven *desi* LGs of an ultra-high density genetic map, which were well validated in multiple geographical locations/environments across years in field. The reliability and validity of the identified PN, SN and SW QTLs were determined by correlating/comparing their underlying genomic regions with that of earlier QTL mapping studies^13^,^52^,^59^,^64^,^69^,^74^,^77^,^97^,^98^,^99^,^100^,^101^ based on correspondence of physical positions of markers covering the target QTL intervals. This analysis identified one of each PN (*qPN8.1*) and SW (*qSW4.1*) QTLs in line with previous QTL mapping studies using diverse intra- and inter-specific mapping populations of chickpea^13^,^59^,^64^,^69^,^97^,^98^,^101^. This analysis indicated novelty and population-specific characteristics of our identified 33 robust QTLs for SW, PN and SN traits in chickpea. Several QTL mapping studies in crop plants, including legumes have effectively utilized ultra-high density genetic linkage maps derived from different intra- and inter-specific mapping populations as foundation for rapid molecular mapping as well as fine mapping/map-based cloning of major genes harbouring robust QTLs governing multiple yield contributing traits (like pod and seed number and seed weight) to accelerate genomics-assisted crop improvement^20^,^21^,^48^,^102^,^103^,^104^,^105^. The use of ultra-high density genetic linkage map as a reference in the present study, significantly narrowed down the marker intervals in the QTL regions to 0.015–1.978 cM (mean < 1 cM), which is lower than that reported (1–3 cM) so far in diverse QTL mapping studies of chickpea^3^,^12^,^13^,^14^,^20^,^22^,^51^,^72^,^73^,^74^,^75^,^76^. It further authenticates the use of SNP markers that are tightly linked to the QTLs for subsequent validation in diverse genetic backgrounds along with fine mapping/map-based cloning of QTLs controlling three agronomic traits in chickpea. Moreover, SNPs localized in the QTL region mapped on eight LGs of an ultra-high density intra-specific genetic linkage map derived from the diverse coding and non-coding sequence components of genes could certainly expedite the process of validation and/positional cloning of QTLs governing three agronomic traits in chickpea. The required inputs obtained from these analyses will eventually help us to identify potential candidate genes underlying the QTLs for marker-assisted genetic enhancement of chickpea. The structural and functional annotation of SNPs mapped on eight *desi* and *kabuli* LGs, including SW, PN and SN QTL intervals of ultra-high density intra-specific genetic maps will enable us to select functional gene-based SNPs (like non-synonymous coding and regulatory SNPs) for establishing marker-trait linkages and identifying genes/QTLs associated with important qualitative and quantitative traits through genetic and association mapping in chickpea.

One strong SW-associated major genomic region (LOD: 14.5, PVE: 38.7%) harbouring a robust QTL (*qSW5.1*) mapped on *desi* LG5 of an intra-specific genetic map was compared/correlated with that of integrated genetic and physical maps of *kabuli* to fine map the major QTL region. The extended LD decay (~15 cM) in *desi* and *kabuli* LGs of genetic maps suggested that high-resolution haplotype (LD)-based trait association mapping at target QTL interval could be an attractive alternative approach for narrowing-down the QTL regions to candidate gene(s) regulating three said agronomic traits in chickpea. Henceforth, we combined haplotype (LD)-based high-resolution trait association mapping at target SW-specific QTL region with differential gene expression profiling (during seed development in low and high seed weight parental genotypes), which delineated one regulatory SNP-containing embryo defective protein-coding gene governing seed weight in chickpea. Such haplotype-specific association analysis in presence of high-resolution significant LD at major QTL region have utility to scale-down the QTLs to specific candidate gene(s) regulating important agronomic traits in crop plants^106^,^107^,^108^,^109^.

The strong trait association potential of an embryo defective gene with SW has been ascertained by higher contribution of a significant superior haplotype (TAC) identified in the URR of this gene with 44.7% SW phenotypic variation in a constituted 244 association panel. This was further supported by the seed-specific expression and significant down-regulated expression of gene haplotype transcript during seed development in contrasting SW parental genotypes and RIL mapping individuals. The favourable natural allelic variants and optimal superior haplotype identified in an embryo defective gene will be helpful in dissection of gene regulatory networks underlying this complex quantitative SW trait in chickpea. This identified gene could eventually be used as a potential candidate in marker-assisted genetic enhancement of chickpea for increasing seed weight and higher yield. The embryo defective chickpea gene ortholog in *Arabidopsis* is known to control the normal embryo development, which implies the role of this vital gene in natural growth and seed development of crop plants^110^. Considering the availability of very limited information regarding embryo defective gene in plants, a further comprehensive molecular characterization and functional validation of this gene in over-expression/knock-down transgenics is required to ascertain its definite involvement in regulating seed development and seed weight of chickpea.

## Methods

### Generation of an intra-specific chickpea mapping population

An intra-specific F_7_ RIL mapping population (ICC 12299 × ICC 8261) comprising of 275 individuals derived from the crosses between two parental chickpea genotypes ICC 12299 and ICC 8261 was generated using a single seed descent method. The chickpea genotype ICC 12299 originated from Nepal is a low 100-seed weight (9 g) and high pod (103) and seed (158) number-containing *desi* (*C. arietinum*) landrace. In contrast, the genotype ICC 8261 (originated from Turkey) is a high 100-seed weight (30 g) and low pod (64) and seed (73) number-containing *kabuli* (*C. arietinum*) landrace.

### Phenotyping of mapping population for agronomic traits

The RIL mapping individuals along with their parental genotypes were grown in the field according to ‘alpha (α)-lattice’ design for three consecutive years (2011–2013) with at least two replications during crop growing season at two diverse geographical locations of India. These RILs were phenotyped individually for three important quantitative agronomic traits, namely PN, SN and SW. The PN and SN was estimated by counting the average number of fully formed pods and seeds per plant (from 10–15 representative plants from each RILs at maturity), respectively. The SW (g) was measured by taking the average weight (g) of 100-matured seeds at 10% moisture content by selecting 10–15 representative plants from each RILs. The mean, standard deviation, coefficient of variation, broad-sense heritability, frequency distribution, correlation coefficient and analysis of variance (ANOVA) of three agronomic traits in RIL mapping population were analyzed using SPSS v17.0 and following the methods of Kujur *et al.*^74^,^99^ and Saxena *et al.*^77^.

### Genome-wide discovery and genotyping of SNPs using GBS assay

The genomic DNA isolated from 275 RILs, including two parental accessions (used as biological replicates) were digested with *Ape*KI, ligated to adapters containing one of 288 unique barcodes, constructed 288-plex GBS libraries and pooled together following the detailed procedures of Elshire *et al.*^40^ and Spindel *et al.*^45^. All these libraries were sequenced by Illumina TrueSeq V3.0 single end sequencing chemistry with read lengths of 100-bp using HiSeq2000 (Illumina Inc., San Diego, CA, USA) NGS platform. The FASTQ raw sequence reads were processed for high-quality sequence filtering and mapping through TASSEL-GBS. The sequence reads with a minimum *phred* Q-score of 10 across the first 72-bp nucleotide sequences were considered as high-quality, which were further rechecked for their quality using FASTQC v0.10.1. The retained good quality sequences were de-multiplexed using unique barcodes attached to each mapping individuals. The sequence reads of each individuals were aligned and mapped to reference draft *desi* (ICC 4958^37^) and *kabuli* (CDC Frontier^36^) chickpea genome sequences separately using Burrows-Wheeler alignment tool (BWA) with default parameters. The missing SNP allele data in individuals were imputed using the haplotype probability method of fastPHASE. A maximum likelihood statistical model and a catalog was used to identify and genotype valid and high-quality SNPs (minimum sequence read depth: 10 with SNP base quality ≥20) in 275 RIL mapping individuals along with two parental genotypes.

### Large-scale validation of SNPs

To validate the genome-wide SNPs identified through GBS assay, the primers designed from the 200-bp sequences flanking either-side of 384 selected SNPs were PCR amplified using the genomic DNA of two parental chickpea genotypes and selected RIL mapping individuals. The amplified PCR products was resequenced, aligned the high-quality sequences and the SNPs were detected among individuals (following the methods^74^,^111^). A selected 96 SNPs identified by reference genome-based GBS assay were validated and genotyped in parental genotypes and representative mapping individuals using MALDI-TOF (matrix-assisted laser desorption ionization-time of flight) mass array SNP genotyping assay (http://www.sequenom.com) following the methods of Pandit *et al.*^112^ and Saxena *et al.*^77^,^111^.

### Construction of intra-specific genetic linkage maps

The genotyping data of reference *desi* and *kabuli* genome-based high-quality GBS-SNPs (MAF ≥ 0.2) showing differentiation between parental genotypes (ICC 12299 and ICC 8261) were analysed in 275 RIL mapping individuals using the χ^2^-test (p < 0.05). The SNP genotyping data showing goodness-of-fit to the expected Mendelian 1:1 segregation ratio were used further for linkage analysis using MAPMAKER/EXP3.0 and JoinMap 4.1 at higher LOD (logarithm of odds) threshold (>10.0) with Kosambi mapping function. The SNPs were integrated into eight *desi* and *kabuli* LGs of intra-specific genetic maps based on their centimorgan (cM) genetic distance using the methods of Kujur *et al.*^74^,^99^ and Saxena *et al.*^77^. The marker ordering was performed using the RECORD algorithm implemented in RECORD_WIN. The best marker ordered genetic linkage map with shortest map distance (cM) was preferred. The LGs with genetically mapped SNPs were designated and numbered (LG1 to LG8) based on their corresponding physical positions (bp) on the chromosomes of chickpea genomes as determined in our study.

### Determination of LD patterns

To determine genome-wide LD patterns in chickpea, the LD estimates (significant *P*-value ≤ 10^−3^) as average squared-allele frequency correlations (r^2^) among a pairs of SNP loci that are genetically mapped on eight *desi* and *kabuli* LGs of intra-specific genetic maps were analysed using the sliding window approach of TASSEL v5.0. The decay of LD with the genetic distance was measured by combining the r^2^ values of SNP-pairs mapped in a uniform genetic intervals of 5 cM (maximum up to 120 cM) across eight *desi* and *kabuli* LGs of genetic maps. The graph was plotted between pooled r^2^ and genetic distance (cM) based on nonlinear regression model considering the r^2^ value = 1 at marker genetic distance of 0 cM^74^,^99^,^101^,^113^ to determine the trend of LD decay in *desi* and *kabuli* genomes.

### Structural and functional annotation of genome-wide SNPs

The reference *desi* and *kabuli* genome-based SNPs mapped on the eight LGs of intra-specific genetic maps were annotated in diverse intragenic and intergenic sequence components of genomes (chromosomes/pseudomolecules) using the genome annotation information (GFF) of *desi*^37^ and *kabuli*^36^. The structural and functional annotation of SNPs, including synonymous and non-synonymous coding and regulatory SNPs were performed using the customized perl scripts and single-nucleotide polymorphism effect predictor (SnpEff v3.1h). The SNPs were plotted individually based on their unique physical positions (bp) on eight chromosomes (pseudomolecules) of *desi* and *kabuli* genomes using Circos v0.67-pre3. The functional annotation of SNP-containing genes was performed according to *desi* and *kabuli* genome annotation and PFAM database v27.0. The genes with SNPs were BLAST searched against the KOGnitor NCBI database. Gene Ontology (GO) enrichment analysis of genes with SNPs was performed using the BiNGO plugin of Cytoscape v2.6 based on Benjamini and Hochberg false discovery rate correction at 5% significance level.

### QTL mapping

For identification and mapping of QTLs, the reference *desi* genome-based high-quality GBS SNP genotyping data and genetic linkage map information of SNPs mapped on eight *desi* LGs of intra-specific genetic map were correlated with multi-location/years replicated field phenotyping data (PN, SN and SW) of RIL mapping individuals and parental genotypes using single marker analysis, interval mapping and composite interval mapping functions of QTL Cartographer v2.5 and MapQTL v6.0. To identify and map the novel genomic regions harbouring the major QTLs associated with three agronomic traits (PN, SN and SW) on LGs, the LOD threshold score of ≥5.0 at 1000 permutations was considered significant (p < 0.05). The significant major trait-influencing QTLs, which were validated across multiple environments (locations)/years were considered as “robust QTLs” controlling agronomic traits (as per Ref. 77). The positional genetic effects, phenotypic variation explained (PVE %) by QTLs and their additive effect (evaluated by parental origin of favourable alleles) on traits were evaluated at significant LOD (p ≤ 0.05).

### QTL-region targeted resequencing and association analysis

A selected strong SW major genomic region underlying robust QTL was sequenced in parental genotypes (ICC 12299 and ICC 8261) along with 10 of each homozygous low and high SW RIL mapping individuals using the multiplexed amplicons sequencing protocol (following manufacturer's instructions) of TruSeq Custom Amplicon v1.5 in Illumina MiSeq next-generation sequencer. The high-quality amplicon sequence reads (> 90% bases covered at 0.5× mean coverage) were mapped to reference *desi* and *kabuli* genomes and SNPs were detected among parental genotypes and mapping individuals adopting the methods of Agarwal *et al.*^80^, Jain *et al.*^82^ and Saxena *et al.*^77^. The sequenced genomic region underlying SW-specific QTL was structurally and functionally annotated (following aforementioned methods). The SNPs showing differentiation between low and high SW parental genotypes and mapping individuals at QTL region of interest were genotyped in 244 chickpea accessions (211 mini-core) belonging to an SW-specific association panel (Table S7) using the MALDI-TOF SNP genotyping assay (following Refs. 77, 111). The SNP genotyping data among accessions were used to constitute haplotypes at target QTL interval. The replicated multi-location/years SW field phenotyping data, population structure (K = 2) statistics and kinship matrix of 244 accessions were obtained from Kujur *et al.*^99^. The association analysis was performed using general linear model (GLM) and mixed linear model (MLM at optimum level of compression with P3D method) of TASSEL v5.0 and mixed model approach of EMMA adopting the detailed procedures of Kujur *et al.*^74^,^99^, Saxena *et al.*^77^ and Thudi *et al.*^101^. To eliminate the confounding effect of population structure and correct the false discovery rate (FDR) based on multiple comparisons, the Bonferroni correction of P-value was performed for each trait-associated SNPs at 5% significance level. By integrating the results of GLM and MLM with EMMA and FDR correction, the SNPs showing strong association (R^2^ = correlation potential of significant SNPs with traits) with SW at significant cut-off P ≤ 10^−4^ were selected.

### Differential gene expression profiling

The SNPs-carrying genes annotated at the short delineated major genomic region underlying the robust SW-specific QTL were selected to infer their differential regulation during seed development through expression profiling. The RNA isolated from three different vegetative and reproductive tissues (leaf, root and flower bud) and two seed developmental stages^74^,^99^,^100^ of high (ICC 8261) and low (ICC 12299) SW parental genotypes was amplified using the gene-specific primers through quantitative RT-PCR assay (following Refs. 74, 100).

### Gene haplotype-based LD mapping

To perform high-resolution haplotype-based association/LD mapping, the fragments covering the entire coding and non-coding sequences, including 2 kb upstream regulatory region (URR) of one strong SW-associated gene (validated by QTL mapping, association mapping and differential expression profiling) were amplified using the genomic DNA of 244 SW-specific association panel. The cloning and sequencing of amplicons, SNP mining, haplotype constitution and LD pattern determination of a gene including, its association potential with SW were estimated using the methods of Kujur *et al.*^74^,^99^, Saxena *et al.*^77^ and Bajaj *et al.*^100^. To determine the potential of diverse haplotypes constituted in the gene for regulating SW, the differential expression profiling in two seed developmental stages of parental genotypes and homozygous RIL mapping individuals representing the low and high SW haplotype groups was performed using the gene haplotype-specific primers.

## Supplementary Material

Supplementary Information Supplementary information

## Figures and Tables

**Table 1:** Detailed characteristics of constructed ultra-high density intra-specific genetic linkage maps of *desi* and *kabuli* chickpea

Linkage groups (LGs)	SNPs mapped		Map length covered (cM)		Average inter-marker distance (cM)	
	*Desi*	*Kabuli*	*Desi*	*Kabuli*	*Desi*	*Kabuli*
CaLG01	736	343	86.076	91.704	0.12	0.27
CaLG02	386	199	85.662	120.359	0.22	0.60
CaLG03	587	463	85.993	82.124	0.15	0.18
CaLG04	574	399	97.782	105.127	0.17	0.26
CaLG05	292	104	94.069	81.444	0.32	0.78
CaLG06	430	285	87.601	110.009	0.20	0.38
CaLG07	339	194	87.821	105.184	0.26	0.54
CaLG08	281	190	89.085	102.516	0.32	0.54
**Total**	**3625**	**2177**	**714.089**	**798.467**	**0.20**	**0.37**

**Table 2:** Significant and robust QTLs regulating three agronomic traits identified and mapped on an ultra-high density intra-specific genetic linkage map of chickpea

Linkage groups (LGs)	QTL identity	LGs	Marker intervals with genetic positions (cM)	Peak LOD score	Phenotypic variance explained (PVE R^2^%) by QTLs	Traits associated	Additive effect
*Ca*_*Desi*_LG01	*qSW1.1*	LG_1	Ca_Desi_SNP_1 (0) to Ca_Desi_SNP_2 (0.146)	13.5	29.8	SW	4.5
	*qPN1.1*	LG_1	Ca_Desi_SNP_15 (5.54) to Ca_Desi_SNP_17 (7.518)	8.2	22.4	PN	3.2
	*qPN1.2*	LG_1	Ca_Desi_SNP_40 (13.733) to Ca_Desi_SNP_42 (14.48)	8.31	22.8	PN	2.8
	*qPN1.3*	LG_1	Ca_Desi_SNP_250 (34.293) to Ca_Desi_SNP_252 (34.453)	8.11	22	PN	1.9
	*qSN1.1*	LG_1	Ca_Desi_SNP_486 (54.514) to Ca_Desi_SNP_488 (54.597)	10.95	29.6	SN	2.7
	*qSW1.2*	LG_1	Ca_Desi_SNP_513 (56.043) to Ca_Desi_SNP_515 (56.158)	13	28.2	SW	3.2
	*qPN1.4*	LG_1	Ca_Desi_SNP_591 (61.419) to Ca_Desi_SNP_595 (61.738)	14	35	PN	5.1
	*qSW1.3*	LG_1	Ca_Desi_SNP_636 (64.655) to Ca_Desi_SNP_638 (64.817)	7.39	23.2	SW	4.5
	*qPN1.5*	LG_1	Ca_Desi_SNP_655 (67.106) to Ca_Desi_SNP_657 (67.22)	7.25	22.6	PN	3.8
*Ca*_*Desi*_LG02	*qSN2.1*	LG_2	Ca_Desi_SNP_752 (9.224) to Ca_Desi_SNP_754 (10.101)	8.91	25.4	SN	2.5
	*qSN2.2*	LG_2	Ca_Desi_SNP_902 (39.18) to Ca_Desi_SNP_904 (39.252)	8.42	23.3	SN	2.8
	*qPN2.1*	LG_2	Ca_Desi_SNP_946 (48.4) to Ca_Desi_SNP_948 (48.982)	10.56	28	PN	3.4
	*qPN2.2*	LG_2	Ca_Desi_SNP_1012 (58.324) to Ca_Desi_SNP_1014 (58.377)	8.12	22	PN	4.7
	*qSN2.3*	LG_2	Ca_Desi_SNP_1113 (80.057) to Ca_Desi_SNP_1115 (81.06)	10.72	32.5	SN	4.2
*Ca*_*Desi*_LG04	*qPN4.1*	LG_4	Ca_Desi_SNP_1842 (42.717) to Ca_Desi_SNP_1844 (43.31)	9.16	22.2	PN	3.8
	*qPN4.2*	LG_4	Ca_Desi_SNP_2011 (51.285) to Ca_Desi_SNP_2013 (51.308)	8.32	22	PN	3.4
	*qPN4.3*	LG_4	Ca_Desi_SNP_2023 (51.702) to Ca_Desi_SNP_2025 (51.717)	9.74	24.7	PN	4.3
	*qSW4.1*	LG_4	Ca_Desi_SNP_2088 (56.304) to Ca_Desi_SNP_2090 (56.442)	9.02	21.6	SW	2.6
*Ca*_*Desi*_LG05	*qSN5.1*	LG_5	Ca_Desi_SNP_2336 (23.792) to Ca_Desi_SNP_2338 (24.875)	12.6	38.1	SN	6.7
	*qSW5.1*	LG_5	Ca_Desi_SNP_2337 (23.805) to Ca_Desi_SNP_2338 (24.875)	14.5	39.7	SW	8.9
	*qSN5.2*	LG_5	Ca_Desi_SNP_2557 (76.802) to Ca_Desi_SNP_2559 (77.188)	10.5	24.5	SN	1.9
*Ca*_*Desi*_LG06	*qSW6.1*	LG_6	Ca_Desi_SNP_2608 (10.981) to Ca_Desi_SNP_2610 (11.552)	7.8	17.9	SW	2.6
	*qSW6.2*	LG_6	Ca_Desi_SNP_2902 (56.733) to Ca_Desi_SNP_2904 (57.25)	8.37	21.8	SW	3.5
	*qPN6.1*	LG_6	Ca_Desi_SNP_2903 (56.755) to Ca_Desi_SNP_2907 (57.422)	7.86	20.7	PN	3.8
	*qSW6.3*	LG_6	Ca_Desi_SNP_2931 (62.038) to Ca_Desi_SNP_2933 (62.381)	11.62	25.7	SW	4.6
	*qPN6.2*	LG_6	Ca_Desi_SNP_2993 (77.157) to Ca_Desi_SNP_2995 (78.929)	9.22	26.4	PN	5.2
*Ca*_*Desi*_LG07	*qSW7.1*	LG_7	Ca_Desi_SNP_3006 (0) to Ca_Desi_SNP_3007 (1.014)	8.28	22.7	SW	3.6
	*qPN7.1*	LG_7	Ca_Desi_SNP_3089 (24.832) to Ca_Desi_SNP_3091 (24.848)	8.35	22.5	PN	4.3
	*qPN7.2*	LG_7	Ca_Desi_SNP_3157 (34.458) to Ca_Desi_SNP_3159 (34.858)	8.28	22.1	PN	3.9
	*qPN7.3*	LG_7	Ca_Desi_SNP_3230 (44.792) to Ca_Desi_SNP_3232 (45.282)	9.65	24.3	PN	4.5
	*qPN7.4*	LG_7	Ca_Desi_SNP_3269 (54.679) to Ca_Desi_SNP_3271 (54.792)	10.45	27.6	PN	5.1
*Ca*_*Desi*_LG08	*qSW8.1*	LG_8	Ca_Desi_SNP_3387 (19.508) to Ca_Desi_SNP_3389 (19.651)	9.06	21.7	SW	3.9
	*qSN8.1*	LG_8	Ca_Desi_SNP_3394 (20.582) to Ca_Desi_SNP_3396 (21.005)	7.44	21.7	SN	2.8
	*qSN8.2*	LG_8	Ca_Desi_SNP_3536 (54.055) to Ca_Desi_SNP_3538 (54.218)	8.45	27.6	SN	4.6
	*qPN8.1*	LG_8	Ca_Desi_SNP_3555 (57.041) to Ca_Desi_SNP_3561 (58.066)	9.21	22.4	PN	5.1

## References

[b1] Jukanti A. K. *et al.* Nutritional quality and health benefits of chickpea (*Cicer arietinum* L.): a review. Br. J. Nutr. 108, S11–26 (2012). 22916806 10.1017/S0007114512000797

[b2] Arumuganathan K. & Earle E. D. Nuclear DNA content of some important plant species. Plant Mol. Biol. Rep. 9, 208–219 (1991).

[b3] Kumar A., Choudhary A. K., Solanki R. K. & Pratap A. Towards marker-assisted selection in pulses: a review. Plant Breed. 130, 297–313 (2011).

[b4] Yu H. *et al.* Gains in QTL detection using an ultra-high density SNP map based on population sequencing relative to traditional RFLP/SSR markers. PLoS One 6, e17595 (2011). 21390234 10.1371/journal.pone.0017595 PMC3048400

[b5] Truco M. J. *et al.* An ultra high-density, transcript-based, genetic map of lettuce. G3 (Bethesda) 3, 617–631 (2013). 23550116 10.1534/g3.112.004929 PMC3618349

[b6] Nayak S. N. *et al.* Integration of novel SSR gene-based SNP marker loci in the chickpea genetic map and establishment of new anchor points with *Medicago truncatula* genome. Theor. Appl. Genet. 120, 1415–1441 (2010). 20098978 10.1007/s00122-010-1265-1 PMC2854349

[b7] Gujaria N. *et al.* Development and use of genic molecular markers (GMMs) for construction of a transcript map of chickpea (*Cicer arietinum* L.). Theor. Appl. Genet. 122, 1577–1589 (2011). 21384113 10.1007/s00122-011-1556-1 PMC3082040

[b8] Gaur R. *et al.* High-throughput SNP discovery and genotyping for constructing a saturated linkage map of chickpea (*Cicer arietinum* L.). DNA Res. 19, 357–373 (2012). 22864163 10.1093/dnares/dss018 PMC3473369

[b9] Hiremath P. J. *et al.* Large-scale development of cost-effective SNP marker assays for diversity assessment and genetic mapping in chickpea and comparative mapping in legumes. Plant Biotechnol. J. 10, 716–732 (2012). 22703242 10.1111/j.1467-7652.2012.00710.x PMC3465799

[b10] Roorkiwal M. *et al.* Single nucleotide polymorphism genotyping for breeding genetics and applications in chickpea and pigeonpea using the BeadXpress platform. Plant Genome 6, 1–10 (2013).

[b11] Choudhary S., Gaur R. & Gupta S. EST-derived genic molecular markers, development and utilization for generating an advanced transcript map of chickpea. Theor. Appl. Genet. 124, 1449–1462 (2012). 22301907 10.1007/s00122-012-1800-3

[b12] Stephens A. *et al.* Genetic marker discovery, intraspecific linkage map construction and quantitative trait locus analysis of *Ascochyta* blight resistance in chickpea (*Cicer arietinum* L.). Mol. Breed. 33, 297–313 (2014).

[b13] Varshney R. K. *et al.* Genetic dissection of drought tolerance in chickpea (*Cicer arietinum* L.). Theor. Appl. Genet. 127, 445–462 (2014a). 24326458 10.1007/s00122-013-2230-6 PMC3910274

[b14] Varshney R. K. *et al.* Marker-assisted backcrossing to introgress resistance to *Fusarium* wilt race 1 and *Ascochyta* blight in C214, an elite cultivar of chickpea. Plant Genome 7, 1–11 (2014b).

[b15] Hyten D. L. *et al.* High density integrated genetic linkage map of soybean and the development of a 1536 universal soy linkage panel for quantitative trait locus mapping. Crop Sci. 50, 960–968 (2010a).

[b16] Antanaviciute L. *et al.* Development of a dense SNP-based linkage map of an apple rootstock progeny using the *Malus* Infinium whole genome genotyping array. BMC Genomics 13, 203 (2012). 22631220 10.1186/1471-2164-13-203 PMC3410780

[b17] Saxena R. K. *et al.* Large-scale development of cost-effective single-nucleotide polymorphism marker assays for genetic mapping in pigeonpea and comparative mapping in legumes. DNA Res. 19, 449–461 (2012). 23103470 10.1093/dnares/dss025 PMC3514856

[b18] Sim S. C. *et al.* Development of a large SNP genotyping array and generation of high-density genetic maps in tomato. PLoS One 7, e40563 (2012). 22802968 10.1371/journal.pone.0040563 PMC3393668

[b19] Akond M. *et al.* SNP-based genetic linkage map of soybean using the SoySNP6K Illumina Infinium Bead chip genotyping array. J. Plant Genome Sci. 3, 80–89 (2013).

[b20] Varshney R. K. *et al.* Achievements and prospects of genomics-assisted breeding in three legume crops of the semi-arid tropics**. Biotech. Advances 31, 1120–1134 (2013a). 10.1016/j.biotechadv.2013.01.001 23313999

[b21] Bohra A. *et al.* Genomics-assisted breeding in four major pulse crops of developing countries: present status and prospects. Theor. Appl. Genet. 127, 1263–1291 (2014). 24710822 10.1007/s00122-014-2301-3 PMC4035543

[b22] Deokar A. A. *et al.* Genome-wide SNP identification in chickpea for use in development of a high density genetic map and improvement of chickpea reference genome assembly. BMC Genomics 15, 708 (2014). 25150411 10.1186/1471-2164-15-708 PMC4158123

[b23] Kang Y. J. *et al.* Genome sequence of mungbean and insights into evolution within *Vigna* species. Nat. Commun. 5, 5443 (2014). 25384727 10.1038/ncomms6443 PMC4241982

[b24] Li X. *et al.* A saturated genetic linkage map of autotetraploid alfalfa (*Medicago sativa* L.) developed using genotyping-by-sequencing is highly syntenous with the *Medicago truncatula* genome. G3 (Bethesda) 4, 1971–1979 (2014). 25147192 10.1534/g3.114.012245 PMC4199703

[b25] Schmutz J. *et al.* A reference genome for common bean and genome-wide analysis of dual domestications. Nat. Genet. 46, 707–713 (2014). 24908249 10.1038/ng.3008 PMC7048698

[b26] Velasco R. *et al.* A high quality draft consensus sequence of the genome of a heterozygous grapevine variety. PLoS One 2, e1326 (2007). 18094749 10.1371/journal.pone.0001326 PMC2147077

[b27] Velasco R. *et al.* The genome of the domesticated apple (*Malus* x *domestica* Borkh.). Nat. Genet. 42, 833–839 (2010). 20802477 10.1038/ng.654

[b28] Jaillon O. *et al.* The grapevine genome sequence suggests ancestral hexaploidization in major angiosperm phyla. Nature 449, 463–467 (2007). 17721507 10.1038/nature06148

[b29] Huang S. *et al.* The genome of the cucumber, *Cucumis sativus* L. Nat. Genet. 41, 1275–1281 (2009). 19881527 10.1038/ng.475

[b30] Muchero W. *et al.* A consensus genetic map of cowpea [*Vigna unguiculata* (L) Walp.] and synteny based on EST-derived SNPs. Proc. Natl. Acad. Sci. USA 106, 18159–18164 (2009). 19826088 10.1073/pnas.0905886106 PMC2761239

[b31] Hyten D. L. *et al.* High-throughput SNP discovery through deep resequencing of a reduced representation library to anchor and orient scaffolds in the soybean whole genome sequence. BMC Genomics 11, 38 (2010b). 20078886 10.1186/1471-2164-11-38 PMC2817691

[b32] Huo N. *et al.* Comparison of a high-density genetic linkage map to genome features in the model grass *Brachypodium distachyon*. Theor. Appl. Genet. 123, 455–464 (2011). 21597976 10.1007/s00122-011-1598-4

[b33] Mayer K. F. *et al.* A physical, genetic and functional sequence assembly of the barley genome. Nature 491, 711–716 (2012). 23075845 10.1038/nature11543

[b34] Ren Y. *et al.* A high resolution genetic map anchoring scaffolds of the sequenced watermelon genome. PLoS One 7, e29453 (2012). 22247776 10.1371/journal.pone.0029453 PMC3256148

[b35] Varshney R. K. *et al.* Draft genome sequence of pigeonpea (*Cajanus cajan*), an orphan legume crop of resource-poor farmers. Nat. Biotechnol. 30, 83–89 (2012). 10.1038/nbt.2022 22057054

[b36] Varshney R. K. *et al.* Draft genome sequence of chickpea (*Cicer arietinum*) provides a resource for trait improvement. Nat. Biotechnol. 31, 240–246 (2013c). 23354103 10.1038/nbt.2491

[b37] Jain M. *et al.* A draft genome sequence of the pulse crop chickpea (*Cicer arietinum* L.). Plant J. 74, 715–729 (2013). 23489434 10.1111/tpj.12173

[b38] Michael T. P. & Jackson S. The first 50 plant genomes. Plant Genome 6, 10.3835/plantgenome2013.03.0001in (2013).

[b39] Schnable P. S. *et al.* The B73 maize genome: complexity, diversity and dynamics. Science 326, 1112–1115 (2009). 19965430 10.1126/science.1178534

[b40] Elshire R. J. *et al.* A robust, simple genotyping-by-sequencing (GBS) approach for high diversity species. PLoS One 6, e19379 (2011). 21573248 10.1371/journal.pone.0019379 PMC3087801

[b41] Poland J. A., Brown P. J., Sorrells M. E. & Jannink J. L. Development of high-density genetic maps for barley and wheat using a novel two-enzyme genotyping-by-sequencing approach. PLoS One 7, e32253 (2012). 22389690 10.1371/journal.pone.0032253 PMC3289635

[b42] Sonah H. *et al.* An improved genotyping-by-sequencing (GBS) approach offering increased versatility and efficiency of SNP discovery and genotyping. PLoS One 8, e54603 (2013). 23372741 10.1371/journal.pone.0054603 PMC3553054

[b43] Thurber C. S., Ma J. M., Higgins R. H. & Brown P. J. Retrospective genomic analysis of sorghum adaptation to temperate-zone grain production. Genome Biol. 14, R68 (2013). 23803286 10.1186/gb-2013-14-6-r68 PMC3706989

[b44] Morris G. P. *et al.* Population genomic and genome-wide association studies of agroclimatic traits in sorghum. Proc. Natl. Acad. Sci. USA 110, 453–458 (2013). 23267105 10.1073/pnas.1215985110 PMC3545811

[b45] Spindel J. *et al.* Bridging the genotyping gap: using genotyping by sequencing (GBS) to add high-density SNP markers and new value to traditional bi-parental mapping and breeding populations. Theor. Appl. Genet. 126, 2699–2716 (2013). 23918062 10.1007/s00122-013-2166-x

[b46] Varala K., Swaminathan K., Li Y. & Hudson M. E. Rapid genotyping of soybean cultivars using high throughput sequencing. PLoS One 6, e24811 (2011). 21949759 10.1371/journal.pone.0024811 PMC3176760

[b47] Bastien M., Sonah H. & Belzile F. Genome-wide association mapping of *Sclerotinia sclerotiorum* resistance in soybean with a genotyping-by-sequencing approach. Plant Genome 7, 1–13 (2014).

[b48] He J. *et al.* Genotyping-by-sequencing (GBS), an ultimate marker assisted selection (MAS) tool to accelerate plant breeding. Front. Plant. Sci. 5, 484 (2014). 25324846 10.3389/fpls.2014.00484 PMC4179701

[b49] Jarquín D. *et al.* Genotyping-by-sequencing for genomic prediction in a soybean breeding population. BMC Genomics 15, 740 (2014). 25174348 10.1186/1471-2164-15-740 PMC4176594

[b50] Sonah H., O'Donoughue L., Cober E., Rajcan I. & Belzile F. Identification of loci governing eight agronomic traits using a GBS-GWAS approach and validation by QTL mapping in soybean. Plant Biotechnol. J. 13, 211–221 (2014). 25213593 10.1111/pbi.12249

[b51] Jaganathan D. *et al.* Genotyping-by-sequencing based intra-specific genetic map refines a “QTL-hotspot” region for drought tolerance in chickpea. Mol. Genet. Genomics 10.1007/s00438-014-0932-3 (2014). PMC4361754 25344290

[b52] Cho S. *et al.* Mapping genes for double podding and other morphological traits in chickpea. Euphytica 125, 285–292 (2002).

[b53] Rakshit S. *et al.* DAF marker tightly linked to a major locus for *Ascochyta* blight resistance in chickpea (*Cicer arietinum* L.). Euphytica 132, 23–30 (2003).

[b54] Chandra S. *et al.* Identifying QTL-linked markers in marker-deficient crops. Proceedings of the 4th International Crop Science Congress Brisbane, Australia, 26 September-1 October 2004., Fisher T., ed. (ed.), The Regional Institute Ltd (Publisher), Gosford New South Wales, Australia. (2004).

[b55] Sharma K. D., Winter P., Kahl G. & Muehlbauer F. J. Molecular mapping of *Fusarium oxysporum* f. sp. *ciceris* race 3 resistance gene in chickpea. Theor. Appl. Genet. 108, 1243–1248 (2004). 14689189 10.1007/s00122-003-1561-0

[b56] Sharma K. D., Chen W. & Muehlbauer F. J. Genetics of chickpea resistance to five races of *Fusarium* wilt and a concise set of race differentials for *Fusarium oxysporum* f.sp. *ciceris*. Plant Dis. 89, 385–390 (2005). 10.1094/PD-89-0385 30795454

[b57] Cobos M. J. *et al.* Linkage map of chickpea (*Cicer arietinum* L.) based on populations from *kabuli* x *desi* crosses: Location of genes for resistance to *Fusarium* wilt race 0. Theor. Appl. Genet. 110, 1347–1353 (2005). 15806343 10.1007/s00122-005-1980-1

[b58] Cobos M. J. *et al.* A new QTL for *Ascochyta* blight resistance in an RIL population derived from an inter-specific cross in chickpea. Euphytica 149, 105–111 (2006).

[b59] Cobos M. J. *et al.* Genetic analysis of agronomic traits in a wide cross of chickpea. Field Crops Res. 111, 130–136 (2009).

[b60] Iruela M. *et al.* Detection of two quantitative trait loci for resistance to *Ascochyta* blight in an intra-specific cross of chickpea (*Cicer arietinum* L.): development of SCAR markers associated with resistance. Theor. Appl. Genet. 112, 278–287 (2006). 16328235 10.1007/s00122-005-0126-9

[b61] Iruela M. *et al.* Validation of a QTL for resistance to *Ascochyta* blight linked to resistance to *Fusarium* wilt race 5 in chickpea (*Cicer arietinum* L.). Eur. J. Plant Pathol. 119, 29–37 (2007).

[b62] Iruela M. *et al.* The marker SCK13603 associated with resistance to *Ascochyta* blight in chickpea is located in a region of a putative retrotransposon. Plant Cell Rep. 28, 53–60 (2009). 18815788 10.1007/s00299-008-0609-7

[b63] Lichtenzveig J., Bonfil D. J., Zhang H. B., Shtienberg D. & Abbo S. Mapping quantitative trait loci in chickpea associated with time to flowering and resistance to *Didymella rabiei* the causal agent of *Ascochyta* blight. Theor. Appl. Genet. 113, 1357–1369 (2006). 17016689 10.1007/s00122-006-0390-3

[b64] Radhika P. *et al.* Development of an integrated intraspecific map of chickpea (*Cicer arietinum* L.) using two recombinant inbred line populations. Theor. Appl. Genet. 115, 209–216 (2007). 17503013 10.1007/s00122-007-0556-7

[b65] Tara'n B., Warkentin T. D., Tullu A. & Vanderberg A. Genetic mapping of *Ascochyta* blight resistance in chickpea (*Cicer arietinum*) using a simple sequence repeat linkage map. Genome 50, 26–34 (2007). 17546068 10.1139/g06-137

[b66] Madrid E. *et al.* Mechanism and molecular markers associated with rust resistance in a chickpea interspecific cross (*Cicer arietinum* x *Cicer reticulatum*). Eur. J. Plant Pathol. 121, 43–53 (2008).

[b67] Anbessa Y., Taran B., Warkentin T. D., Tullu A. & Vandenberg A. Genetic analyses and conservation of QTL for *Ascochyta* blight resistance in chickpea (*Cicer arietinum* L.). Theor. Appl. Genet. 119, 757–765 (2009). 19517090 10.1007/s00122-009-1086-2

[b68] Gowda S. J. M., Radhika P., Kadoo N. Y., Mhase L. B. & Gupta V. S. Molecular mapping of wilt resistance genes in chickpea. Mol. Breed. 24, 177–183 (2009).

[b69] Gowda C. L. L., Upadhyaya H. D., Dronavalli N. & Singh S. Identification of large-seeded high-yielding stable *kabuli* chickpea germplasm lines for use in crop improvement. Crop Sci. 5, 198–209 (2011).

[b70] Aryamanesh N., Nelson M. N., Yan G., Clarke H. J. & Siddique K. H. M. Mapping a major gene for growth habit and QTLs for *Ascochyta* blight resistance and flowering time in a population between chickpea and *Cicer reticulatum*. Euphytica 173, 307–319 (2010).

[b71] Anuradha C. *et al.* Mapping QTL for resistance to botrytis grey mould in chickpea. Euphytica 182, 1–9 (2011).

[b72] Rehman A. U. *et al.* Mapping QTL associated with traits affecting grain yield in chickpea (*Cicer arietinum* L.) under terminal drought stress. Crop Sci. 51, 450–463 (2011).

[b73] Vadez V. *et al.* Assessment of ICCV 2 × JG 62 chickpea progenies shows sensitivity of reproduction to salt stress and reveals QTLs for seed yield and yield components. Mol. Breed. 30, 9–21 (2012).

[b74] Kujur A. *et al.* Functionally relevant microsatellite markers from chickpea transcription factor genes for efficient genotyping applications and trait association mapping. DNA Res. 20, 355–374 (2013). 23633531 10.1093/dnares/dst015 PMC3738162

[b75] Sabbavarapu M. M. *et al.* Molecular mapping of QTLs for resistance to *Fusarium* wilt (race 1) and *Ascochyta* blight in chickpea (*Cicer arietinum* L.). Euphytica 193, 121–133 (2013).

[b76] Varshney R. K. *et al.* Fast-track introgression of “QTL-hotspot” for root traits and other drought tolerance trait in JG 11, an elite and leading variety of chickpea (*Cicer arietinum L.*). Plant Genome 6, 1–26. (2013b).

[b77] Saxena M. S. *et al.* An Integrated genomic approach for rapid delineation of candidate genes regulating agro-morphological traits in chickpea. DNA Res. 21, 695–710 (2014a). 25335477 10.1093/dnares/dsu031 PMC4263302

[b78] Ruperao P. *et al.* A chromosomal genomics approach to assess and validate the *desi* and *kabuli* draft chickpea genome assemblies. Plant Biotechnol. J. 12, 778–786. (2014). 24702794 10.1111/pbi.12182

[b79] Hiremath P. J. *et al.* Large-scale transcriptome analysis in chickpea (*Cicer arietinum* L.), an orphan legume crop of the semi-arid tropics of Asia and Africa. Plant Biotechnol. J. 9, 922–931 (2011). 21615673 10.1111/j.1467-7652.2011.00625.x PMC3437486

[b80] Agarwal G. *et al.* Comparative analysis of *kabuli* chickpea transcriptome with *desi* wild chickpea provides a rich resource for development of functional markers. PLoS One 7, e52443 (2012). 23300670 10.1371/journal.pone.0052443 PMC3531472

[b81] Jhanwar S. *et al.* Transcriptome sequencing of wild chickpea as a rich resource for marker development. Plant Biotechnol. J. 10, 690–702 (2012). 22672127 10.1111/j.1467-7652.2012.00712.x

[b82] Jain M., Moharana K. C., Shankar R., Kumari R. & Garg R. Genome-wide discovery of DNA polymorphisms in rice cultivars with contrasting drought and salinity stress response and their functional relevance. Plant Biotechnol. J. 12, 253–264 (2014). 24460890 10.1111/pbi.12133

[b83] Mather K. A. *et al.* Extent of linkage disequilibrium in rice (*Oryza sativa* L.). Genetics 177, 2223–2232 (2007). 17947413 10.1534/genetics.107.079616 PMC2219496

[b84] Atwell S. *et al.* Genome-wide association study of 107 phenotypes in *Arabidopsis thaliana* inbred lines. Nature 465, 627–631 (2010). 20336072 10.1038/nature08800 PMC3023908

[b85] Branca A. *et al.* Whole-genome nucleotide diversity, recombination, and linkage disequilibrium in the model legume *Medicago truncatula*. Proc. Natl. Acad. Sci. USA 108, E864–E870 (2011). 21949378 10.1073/pnas.1104032108 PMC3198318

[b86] Lam H. M. *et al.* Resequencing of 31 wild and cultivated soybean genomes identifies patterns of genetic diversity and selection. Nat. Genet. 42, 1053–1059 (2010). 21076406 10.1038/ng.715

[b87] Zhao K. *et al.* Genome-wide association mapping reveals a rich genetic architecture of complex traits in *Oryza sativa*. Nat. Commun. 2, 467 (2011). 21915109 10.1038/ncomms1467 PMC3195253

[b88] Riedelsheimer C. *et al.* Genome-wide association mapping of leaf metabolic profiles for dissecting complex traits in maize. Proc. Natl. Acad. Sci. USA 109, 8872–8877 (2012). 22615396 10.1073/pnas.1120813109 PMC3384205

[b89] Sakiroglu M. *et al.* Patterns of linkage disequilibrium and association mapping in diploid alfalfa (*M. sativa L.)*. Theor. Appl. Genet. 125, 577–590 (2012). 22476875 10.1007/s00122-012-1854-2 PMC3397135

[b90] Xiao Y. *et al.* Genetic structure and linkage disequilibrium pattern of a rapeseed (*Brassica napus* L.) association mapping panel revealed by microsatellites. Theor. Appl. Genet. 125, 437–447 (2012). 22437490 10.1007/s00122-012-1843-5

[b91] Abbo S., Berger J. & Turner N. C. Evolution of cultivated chickpea: four bottlenecks limit diversity and constrain adaptation. Funct. Plant Biol. 30, 1081–1087 (2003). 10.1071/FP03084 32689090

[b92] Berger J. D., Buck R., Henzell J. M. & Turner N. C. Evolution in the genus *Cicer* vernalisation response and low temperature pod set in chickpea (*C. arietinum L.*) and its annual wild relatives. Aust. J. Agric. Res. 56, 1191–1200 (2005).

[b93] Toker C. A note on the evolution of *kabuli* chickpeas as shown by induced mutations in *Cicer reticulatum* Ladizinsky. Genet. Resour. Crop Evol. 56, 7–12 (2009).

[b94] Masood A. A., Mujahid Y., Khan M. I. & Abid S. Improving precision of agricultural field experiments. J. Sust. Develop. 3, 11–13 (2006).

[b95] Abd El-Mohsen A. A. & Abo-Hegazy S. R. Comparing the relative efficiency of two experimental designs in wheat field trials. Sci. Res. Review J. 1, 101–109 (2013).

[b96] Abd El-Shafi *et al.* Efficiency of classical complete and incomplete block designs in yield trial on bread wheat genotypes. Res. J. Agri. Biolo. Sci. 10, 17–23 (2014).

[b97] Cobos M. J. *et al.* Population derived from a *kabuli* × *desi* cross. Ann. Appl. Biol. 151, 33–42 (2007).

[b98] Hossain S., Ford R., McNeil D., Pittock C. & Panozzo J. F. Inheritance of seed size in chickpea (*Cicer arietinum* L.) and identification of QTL based on 100-seed weight and seed size index. Aust. J. Crop. Sci. 4, 126–135 (2010).

[b99] Kujur A. *et al.* An efficient and cost-effective approach for genic microsatellite marker-based large-scale trait association mapping: identification of candidate genes for seed weight in chickpea**. Mol. Breed. 34, 241–265 (2014).

[b100] Bajaj D. *et al.* Genome-wide conserved non-coding microsatellite (CNMS) marker-based integrative genetical genomics for quantitative dissection of seed weight in chickpea. J. Exp. Bot. 66 1271–1290 (2015). 25504138 10.1093/jxb/eru478 PMC4339591

[b101] Thudi M. *et al.* Genetic dissection of drought and heat tolerance in chickpea through genome-wide and candidate gene-based association mapping approaches. PLoS One 9, e96758 (2014). 24801366 10.1371/journal.pone.0096758 PMC4011848

[b102] Pathan S. M. *et al.* Genetic mapping and confirmation of quantitative trait loci for seed protein and oil contents and seed weight in soybean. Crop Sci. 53, 765–774 (2012).

[b103] Kato S. *et al.* A major and stable QTL associated with seed weight in soybean across multiple environments and genetic backgrounds. Theor. Appl. Genet. 127, 1365–1374 (2014). 24718925 10.1007/s00122-014-2304-0

[b104] Liu Y.-L. *et al.* Identification of quantitative trait loci underlying plant height and seed weight in soybean. Plant Genome 6, 1–11 (2014).

[b105] Yuste-Lisbona F. J. *et al.* Genetic analysis of single-locus and epistatic QTLs for seed traits in an adapted × nuña RIL population of common bean (*Phaseolus vulgaris* L.). Theor. Appl. Genet. 127, 897–912 (2014). 24441949 10.1007/s00122-014-2265-3

[b106] Lu Y. *et al.* Joint linkage-linkage disequilibrium mapping is a powerful approach to detecting quantitative trait loci underlying drought tolerance in maize. Proc. Natl. Acad. Sci. USA 107, 19585–19590 (2010). 20974948 10.1073/pnas.1006105107 PMC2984198

[b107] Famoso A. N. *et al.* Genetic architecture of aluminum tolerance in rice (*Oryza sativa*) determined through genome-wide association analysis and QTL mapping. PLoS Genet. 7, e1002221 (2011). 21829395 10.1371/journal.pgen.1002221 PMC3150440

[b108] Li S. *et al.* Natural variation in *PTB1* regulates rice seed setting rate by controlling pollen tube growth. Nat. Commun. 4, 2793 (2013). 24240868 10.1038/ncomms3793

[b109] Zhang Z. *et al.* QTL analysis of kernel-related traits in maize using an immortalized F_2_ population. PLoS One 9, e89645 (2014). 24586932 10.1371/journal.pone.0089645 PMC3938492

[b110] Tzafrir I. *et al.* Identification of genes required for embryo development in *Arabidopsis*. Plant Physiol. 135, 1206–1220 (2004). 15266054 10.1104/pp.104.045179 PMC519041

[b111] Saxena M. S. *et al.* Natural allelic diversity, genetic structure and linkage disequilibrium pattern in wild chickpea. PLoS One 9, e107484 (2014b). 25222488 10.1371/journal.pone.0107484 PMC4164632

[b112] Pandit A. *et al.* Combining QTL mapping and transcriptome profiling of bulked RILs for identification of functional polymorphism for salt tolerance genes in rice (*Oryza sativa* L.). Mol. Genet. Genomics 284, 121–136 (2010). 20602115 10.1007/s00438-010-0551-6

[b113] Yan W. G. *et al.* Association mapping of stigma and spikelet characteristics in rice (*Oryza sativa* L.). Mol. Breed. 24, 277–292 (2009). 20234878 10.1007/s11032-009-9290-y PMC2837221

