# Supplementary material for: Ultra-high density intra-specific genetic linkage maps accelerate identification of functionally relevant molecular tags governing important agronomic traits in chickpea
Source: Sci Rep. 2015 May 5;5:9468. doi: 10.1038/srep09468 (PMC5386344; doi:10.1038/srep09468)
Supplement: Supplementary Information [file srep09468-s1.pdf]

Ultra-high density intra-specific genetic linkage maps accelerate identification of functionally relevant molecular tags governing important agronomic traits in chickpea

Alice Kujur[1a], Hari D. Upadhyaya[2a], Tanima Shree[1], Deepak Bajaj[1], Shouvik Das[1], Maneesha S. Saxena[1], Saurabh Badoni[1], Vinod Kumar[3], Shailesh Tripathi[4], C.L.L. Gowda[2], Shivali Sharma[2], Sube Singh[2], Akhilesh K. Tyagi[1], Swarup K. Parida[1*]

[1]National Institute of Plant Genome Research (NIPGR), Aruna Asaf Ali Marg, New Delhi 110067, India

[2]International Crops Research Institute for the Semi-Arid Tropics (ICRISAT), Patancheru 502324, Andhra Pradesh, India

[3]National Research Centre on Plant Biotechnology (NRCPB), New Delhi 110012, India

[4]Division of Genetics, Indian Agricultural Research Institute (IARI), New Delhi 110012, India

*Corresponding author

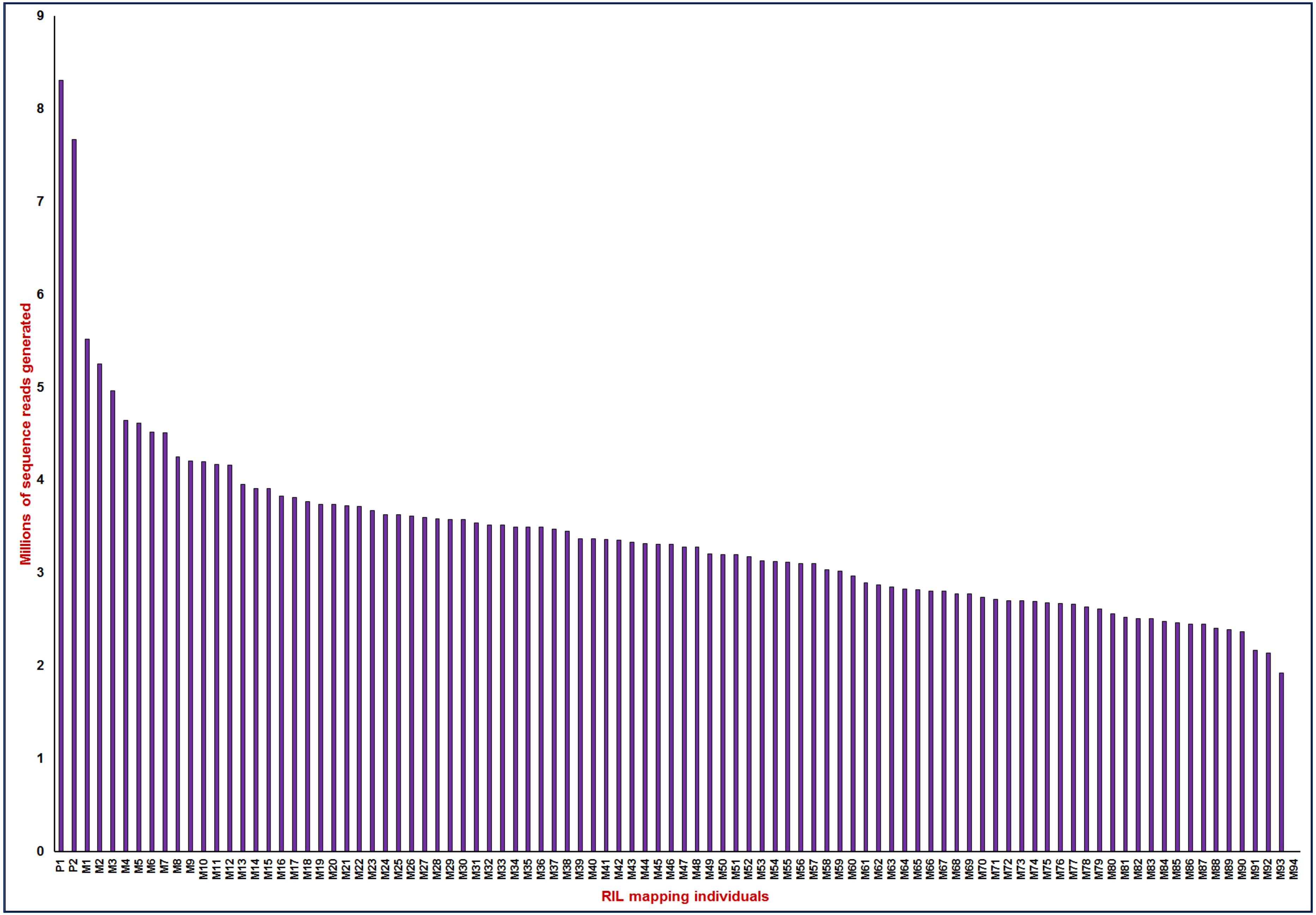

Figure S1: Frequency distribution of millions sequence reads generated from a representative set of 96 individuals [including two mapping parental genotypes (P1 and P2) and 93 RIL mapping individuals (M1-M93)] using a 288-plex GBS assay. P1: ICC 12299 and P2: ICC 8261. M94: Negative control (without genomic DNA) blank sample.

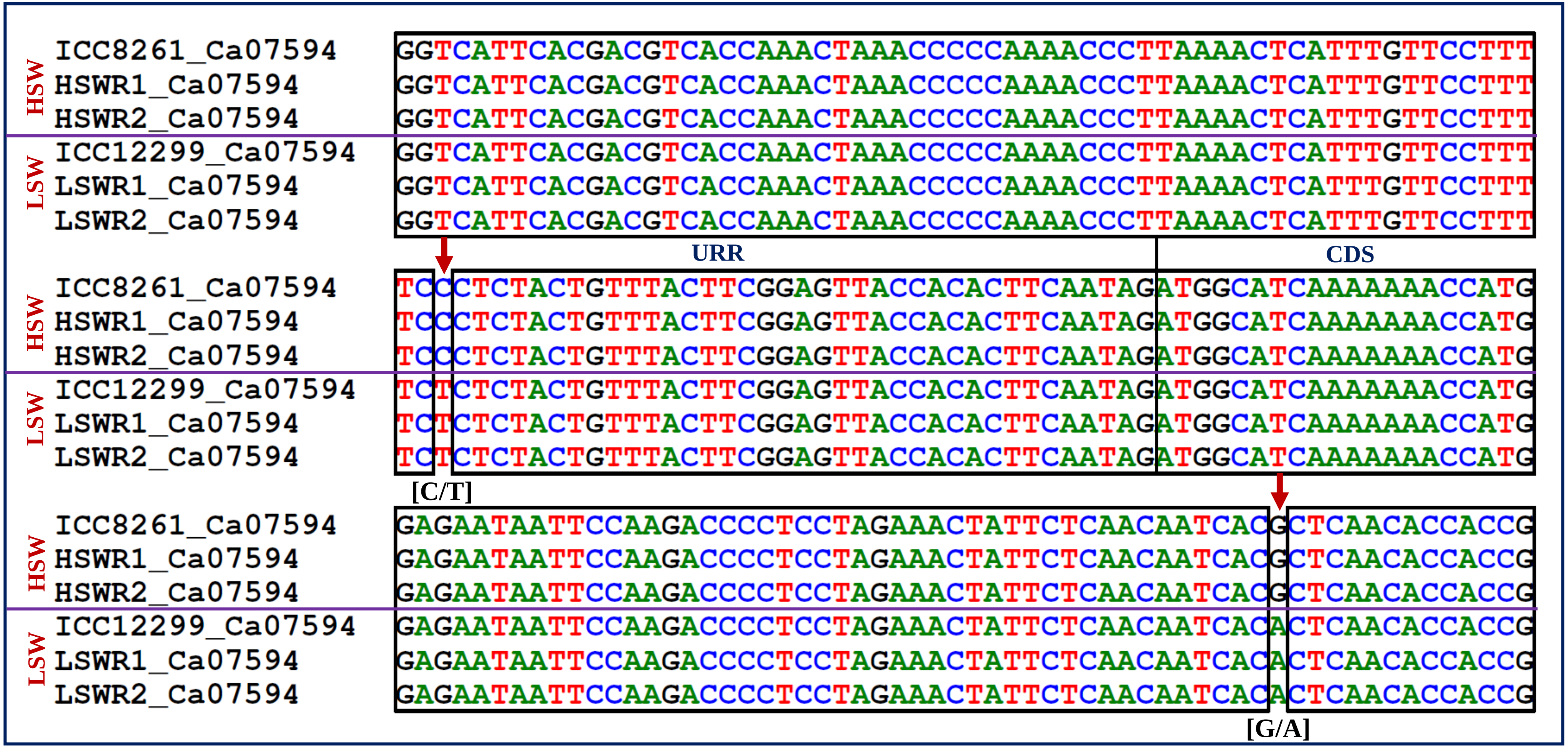

Figure S2A: A representative figure showing the validation of two GBS-based SNP loci (C/T and G/A) in the upstream regulatory and coding sequences (CDS) of a *kabuli* embryo defective protein coding gene (Ca07594) by the multiple alignment of amplicon resequences generated from high and low seed weight mapping parental genotypes (ICC 8261 and ICC 12299) and selective homozygous RIL mapping individuals. The two validated SNP loci showing differentiation between high and low seed weight parental genotypes and RIL mapping individuals are highlighted. HSWR: High Seed Weight RILs and LSWR: Low Seed Weight RILs.

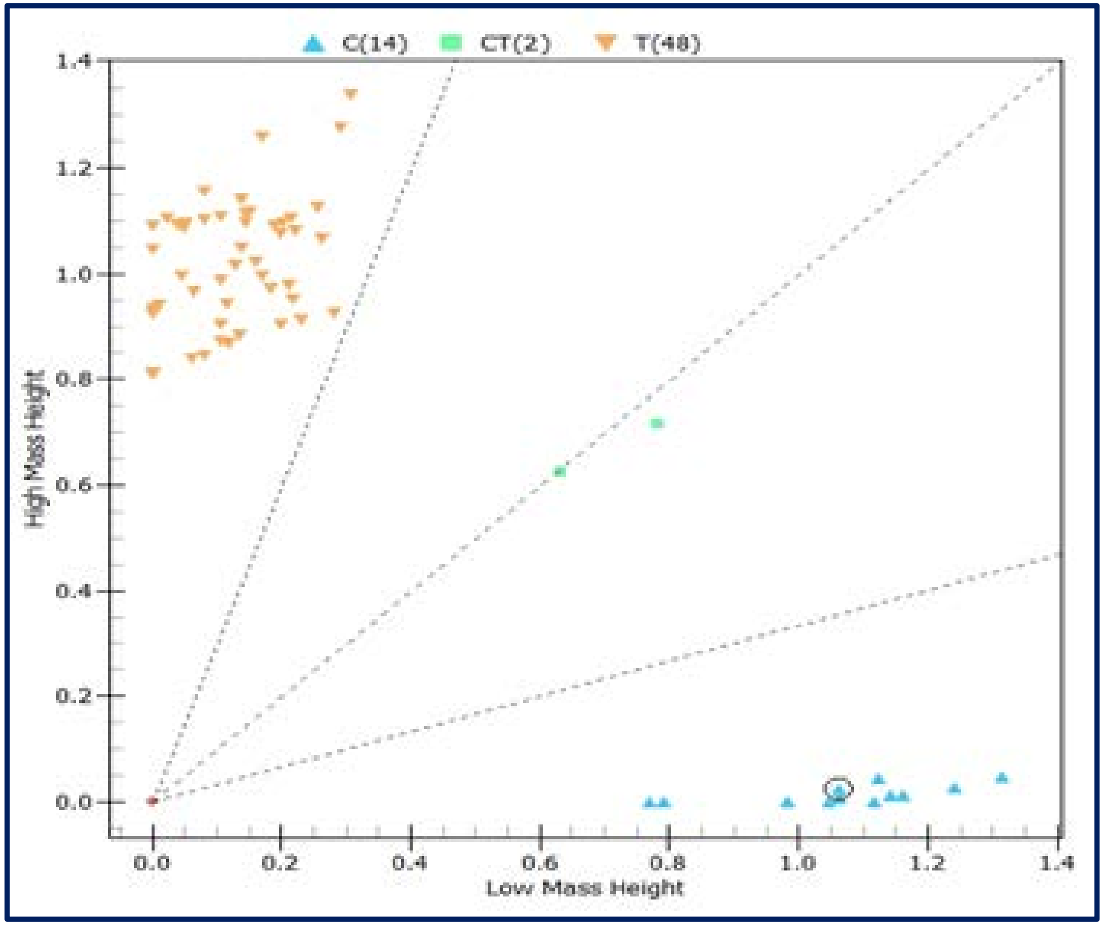

Figure S2B: Call cluster plots for one representative SNP locus (C/T) validating the GBS-based genotyping information generated in mapping parental genotypes (ICC 12299 and ICC 8261) and selected RIL mapping individuals using a MALDI-TOF mass array. Distinct differentiation of homozygous and heterozygous SNPs based on the mass differences of corresponding alleles are evident.

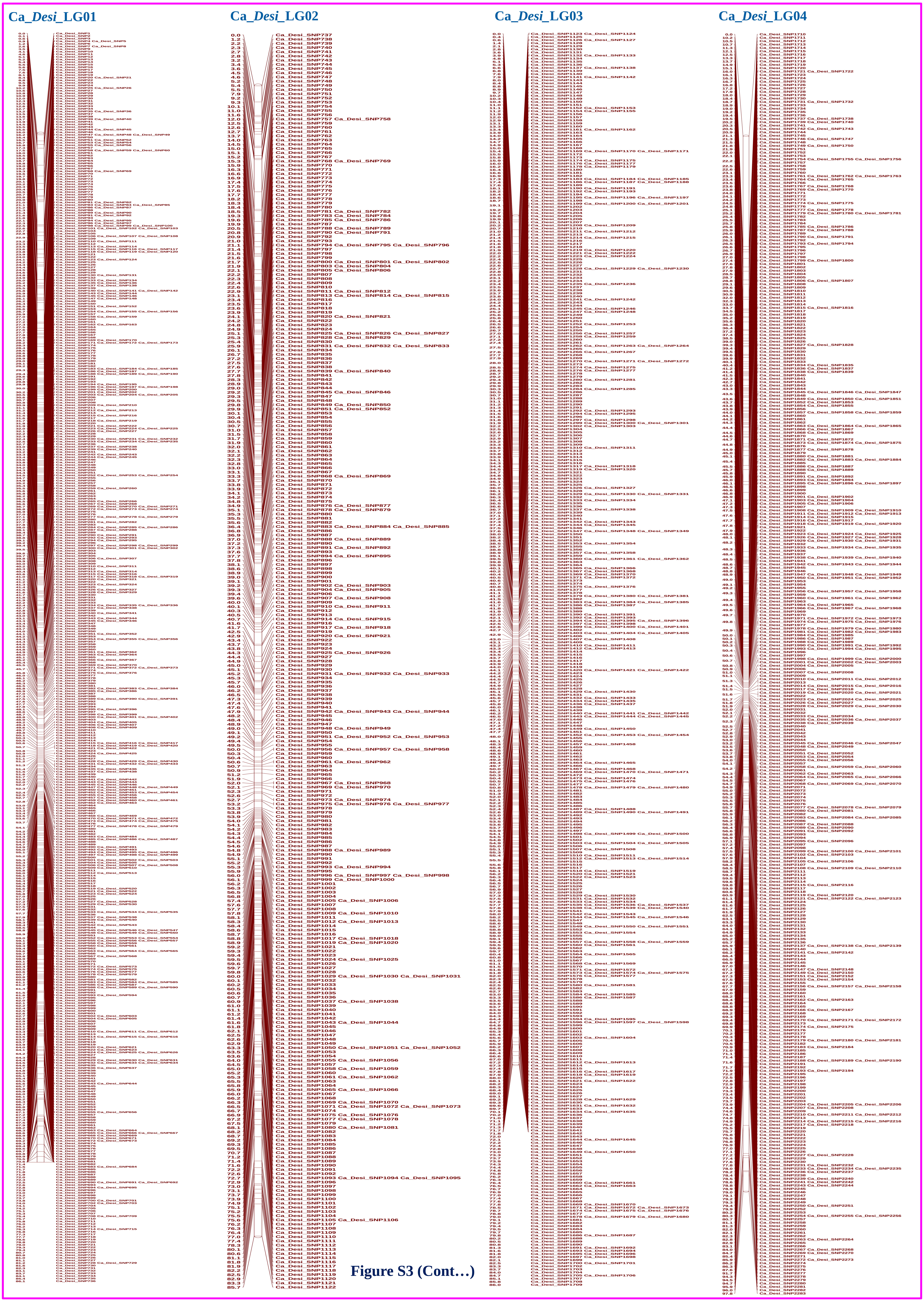

Figure S3 (Cont…)

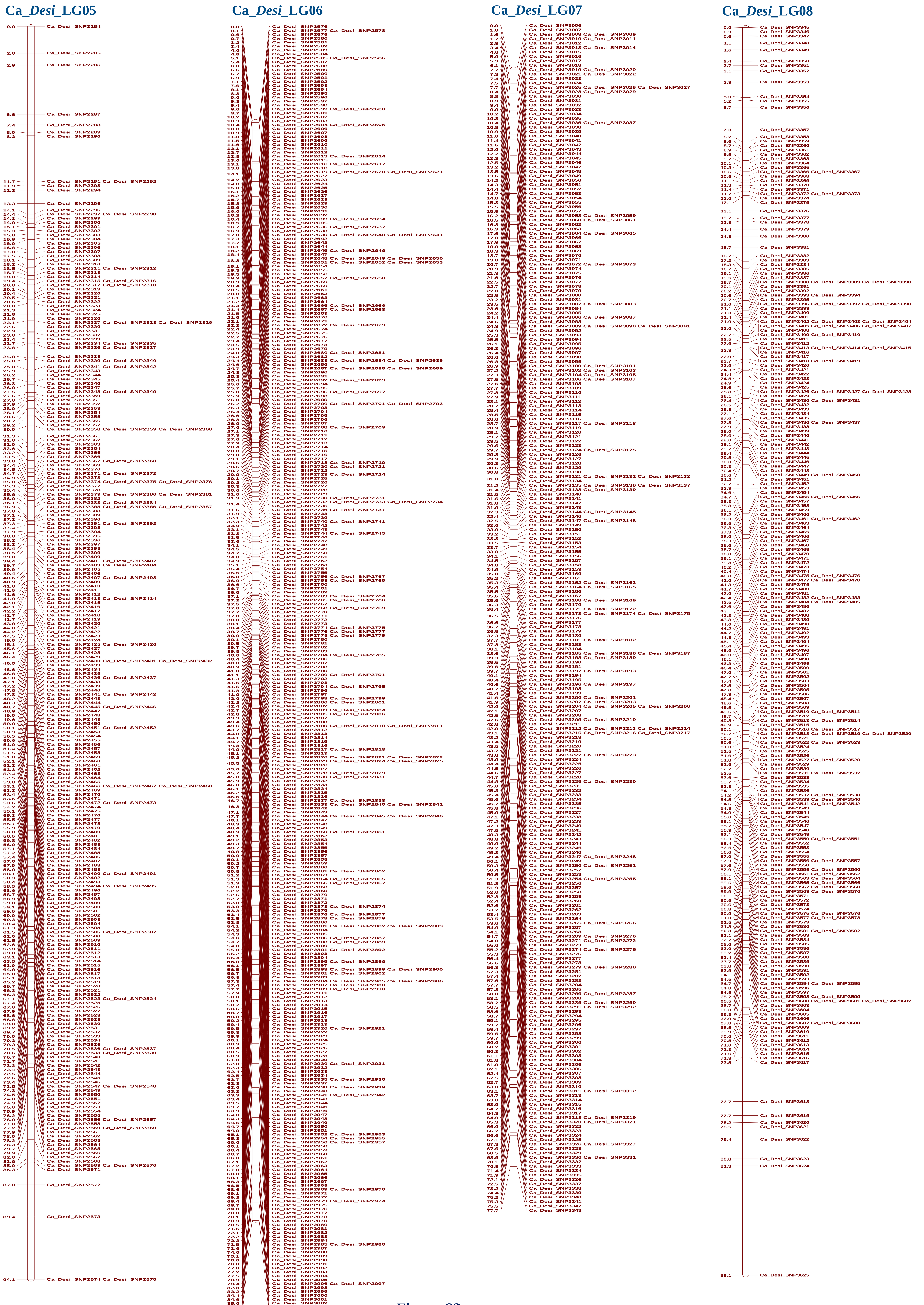

Figure S3

Figure S3. A 3625 SNP markers-based ultra-high density intra-specific genetic linkage map (ICC 12299 x ICC 8261) constructed using reference *desi* genome-based GBS assay. The genetic distance (cM) and identity of the SNP loci integrated on the eight LGs are indicated on the left and right side of each LGs, respectively. The identity of SNP loci mentioned on the right side of chromosomes that corresponds to the marker IDs as mentioned details in the Table S3 and S6.

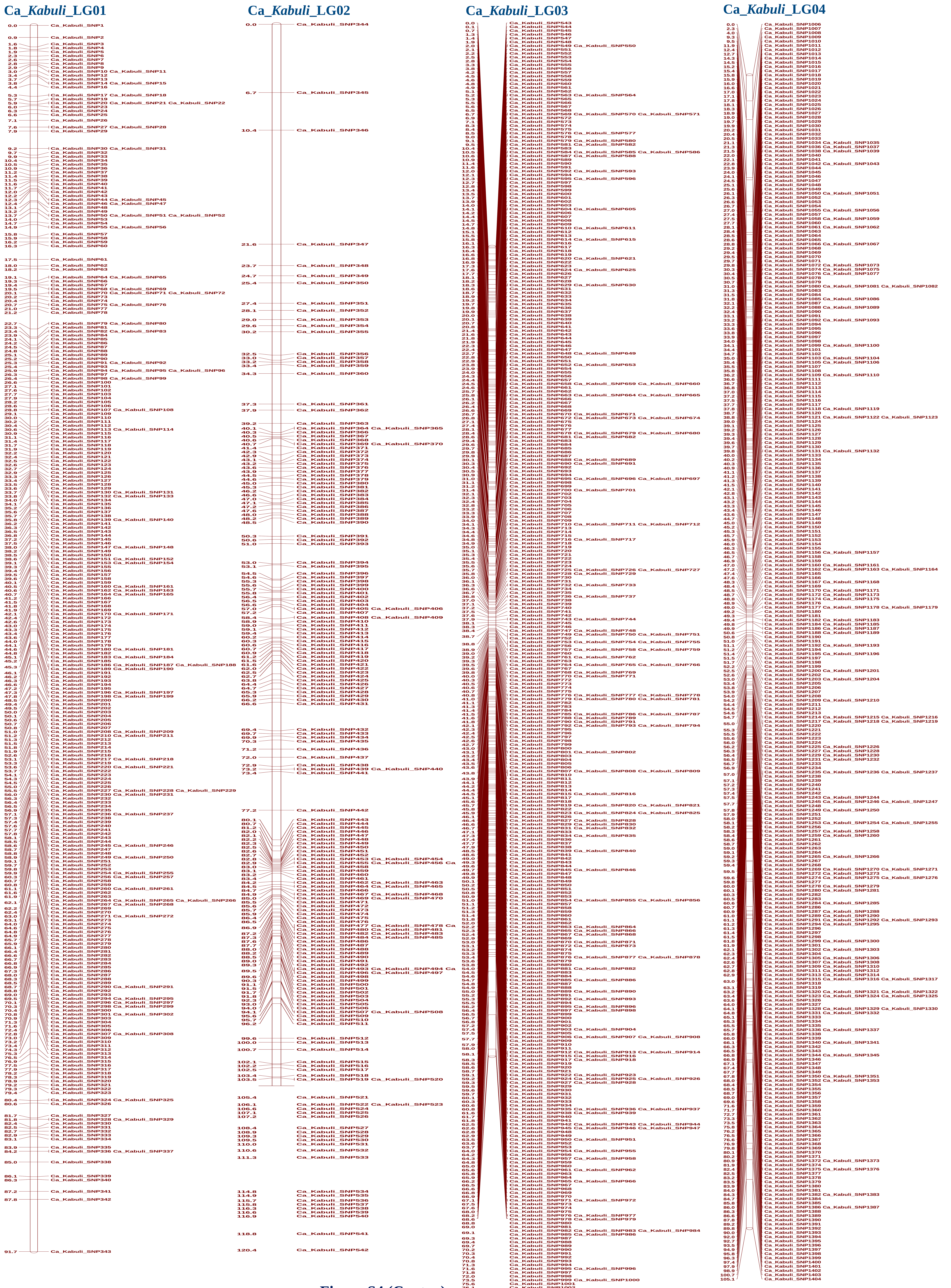

Figure S4 (Cont…)

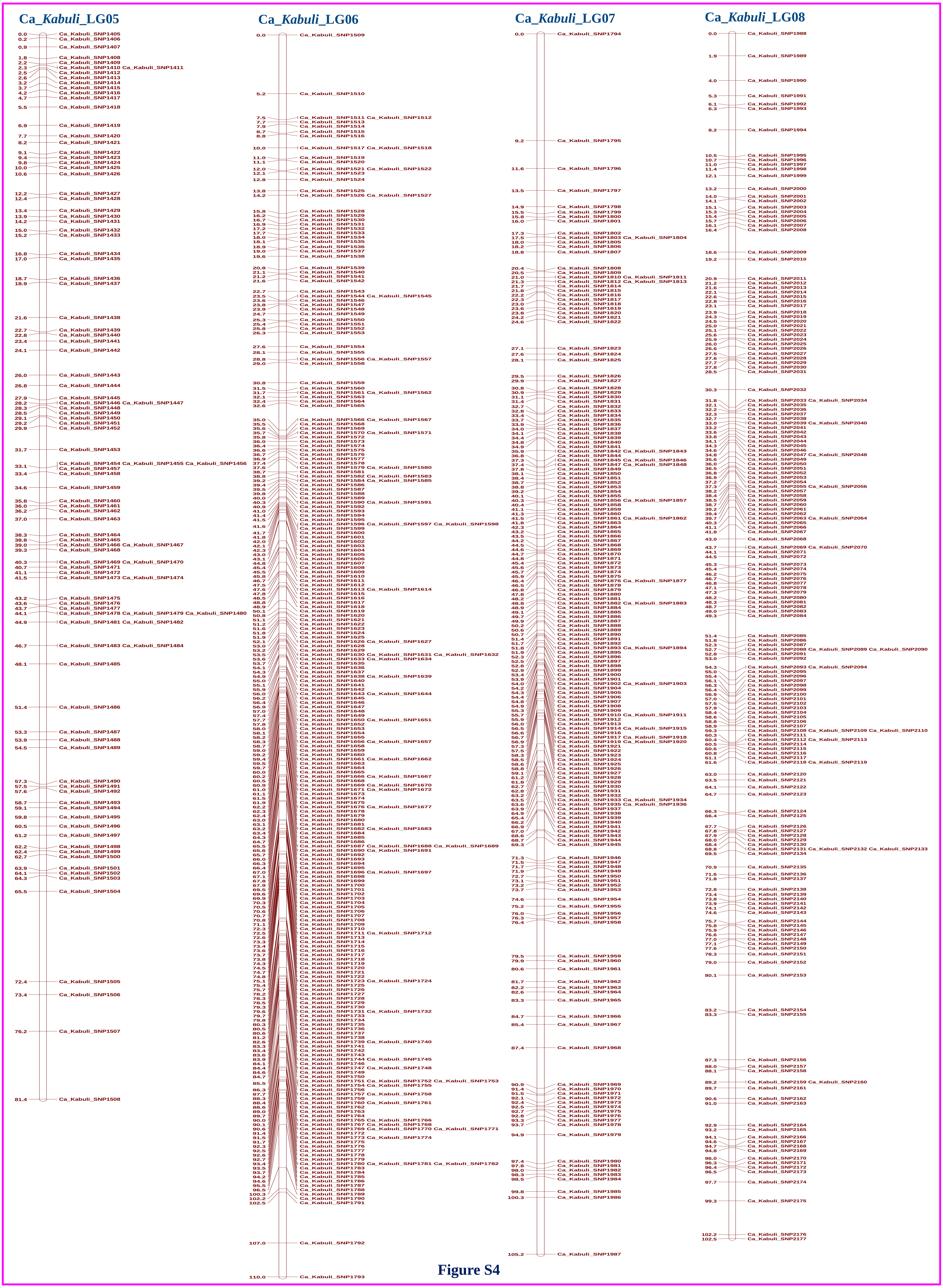

Figure S4

Figure S4: A 3625 SNP markers-based ultra-high density intra-specific genetic linkage map (ICC 12299 x ICC 8261) constructed using reference *kabuli* genome-based GBS assay. The genetic distance (cM) and identity of the SNP loci integrated on the eight LGs are indicated on the left and right side of each LGs, respectively. The identity of SNP loci mentioned on the right side of chromosomes that corresponds to the marker IDs as mentioned details in the Table S3 and S6.

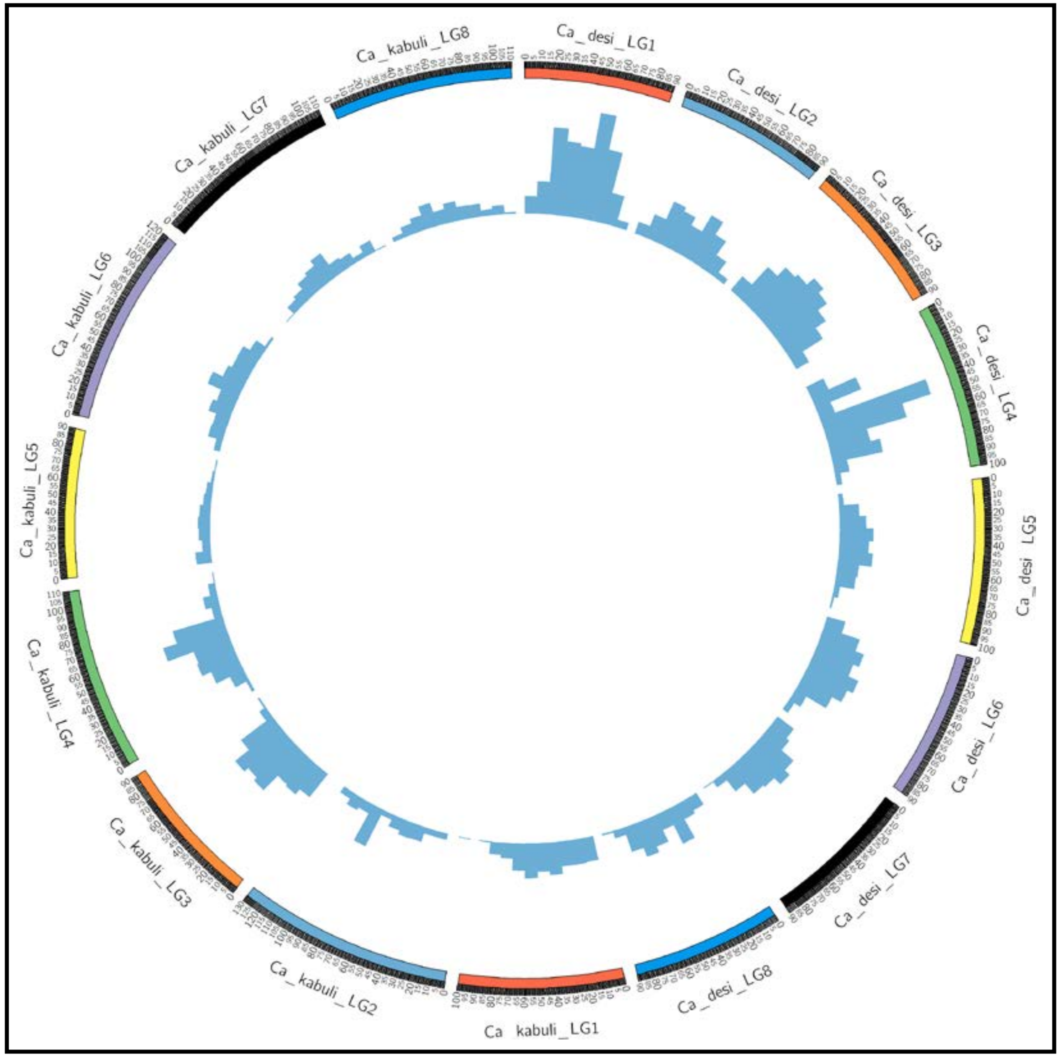

Figure S5: Distribution of 3625 and 2177 SNP markers mapped on eight *desi* and *kabuli* LGs of ultra-high density intra-specific genetic linkage maps (ICC 12299 x ICC 8261), respectively are depicted in the Circos circular ideogram. The innermost circles display the distribution of SNPs genetically mapped on eight LGs, while outermost circles represent the eight *desi* and *kabuli* chickpea LGs coded with different colours.

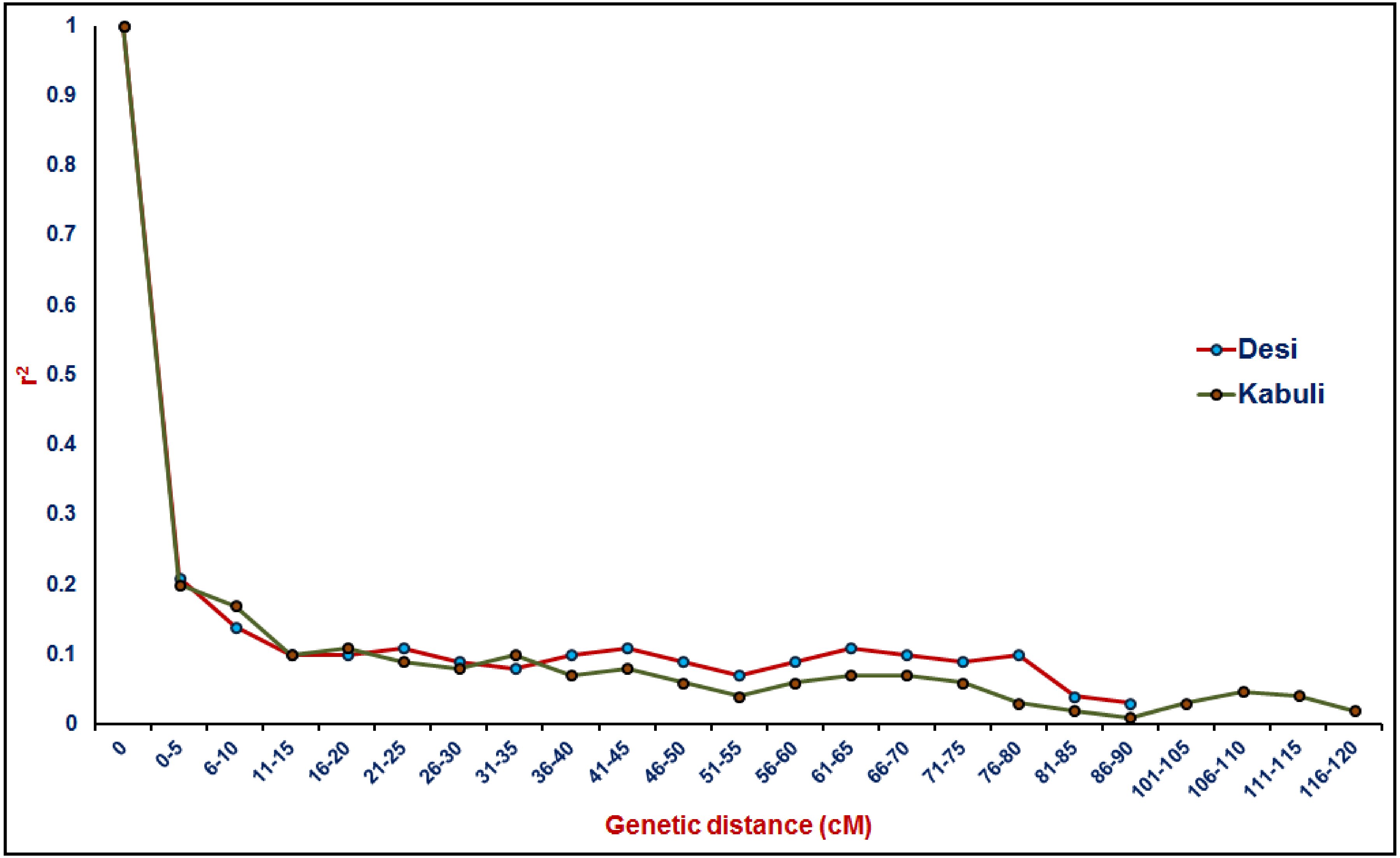

Figure S6: Genome-wide LD decay (mean r^2) estimated using 3625 and 2689 SNPs that are mapped on eight *desi* and *kabuli* LGs of ultra-high density intra-specific genetic maps, respectively. For LD decay, the r^2 value of the SNP marker genetic distance of 0 cM is considered 1. The plotted curved lines indicate the mean r^2 values among markers spaced with uniform 5 cM physical intervals from 0 to 120 cM.

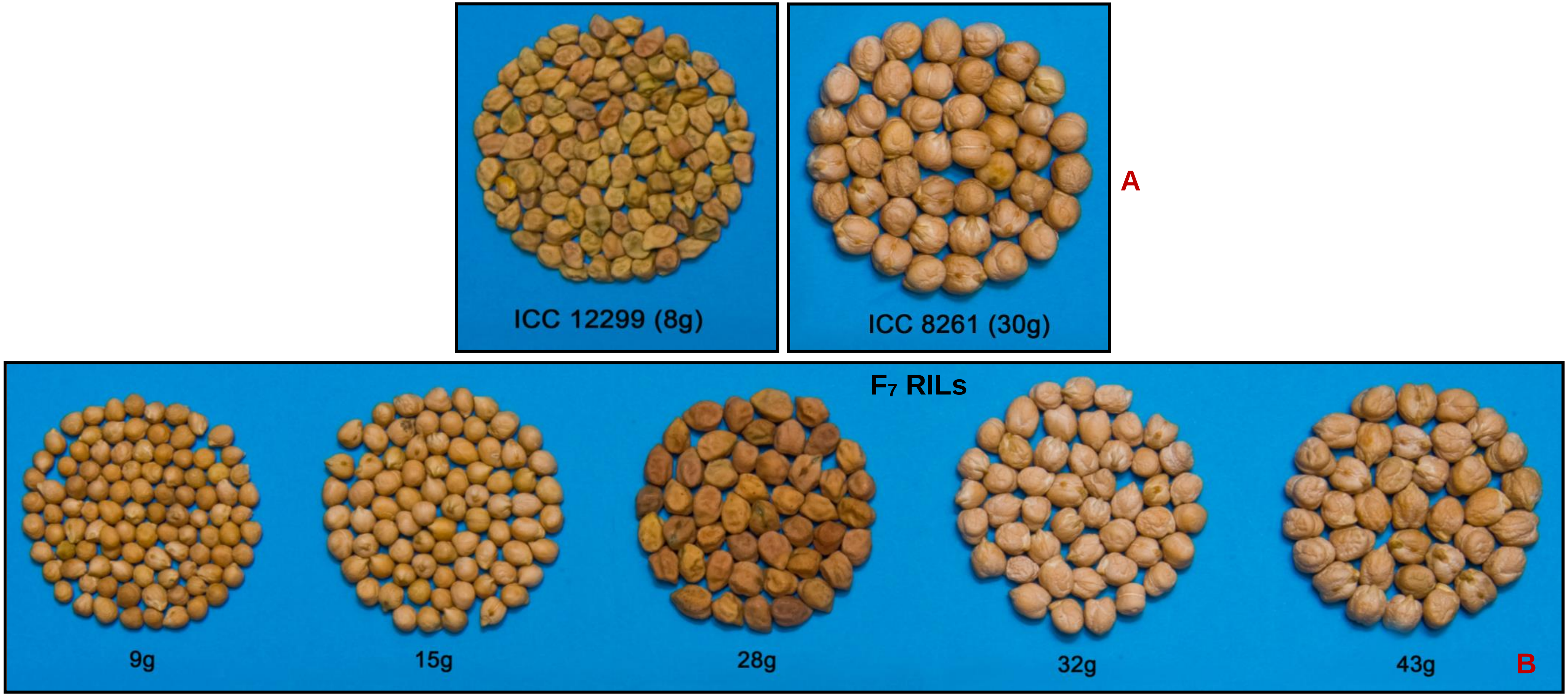

Figure S7: A snapshot depicting a wider phenotypic trait variation of 100-seed weight (9-43 g) among two parental genotypes (A) (ICC 12299 x ICC 8261) of an intra-specific RIL mapping population (ICC 12299 x ICC 8261) and five selected RIL mapping individuals (B).

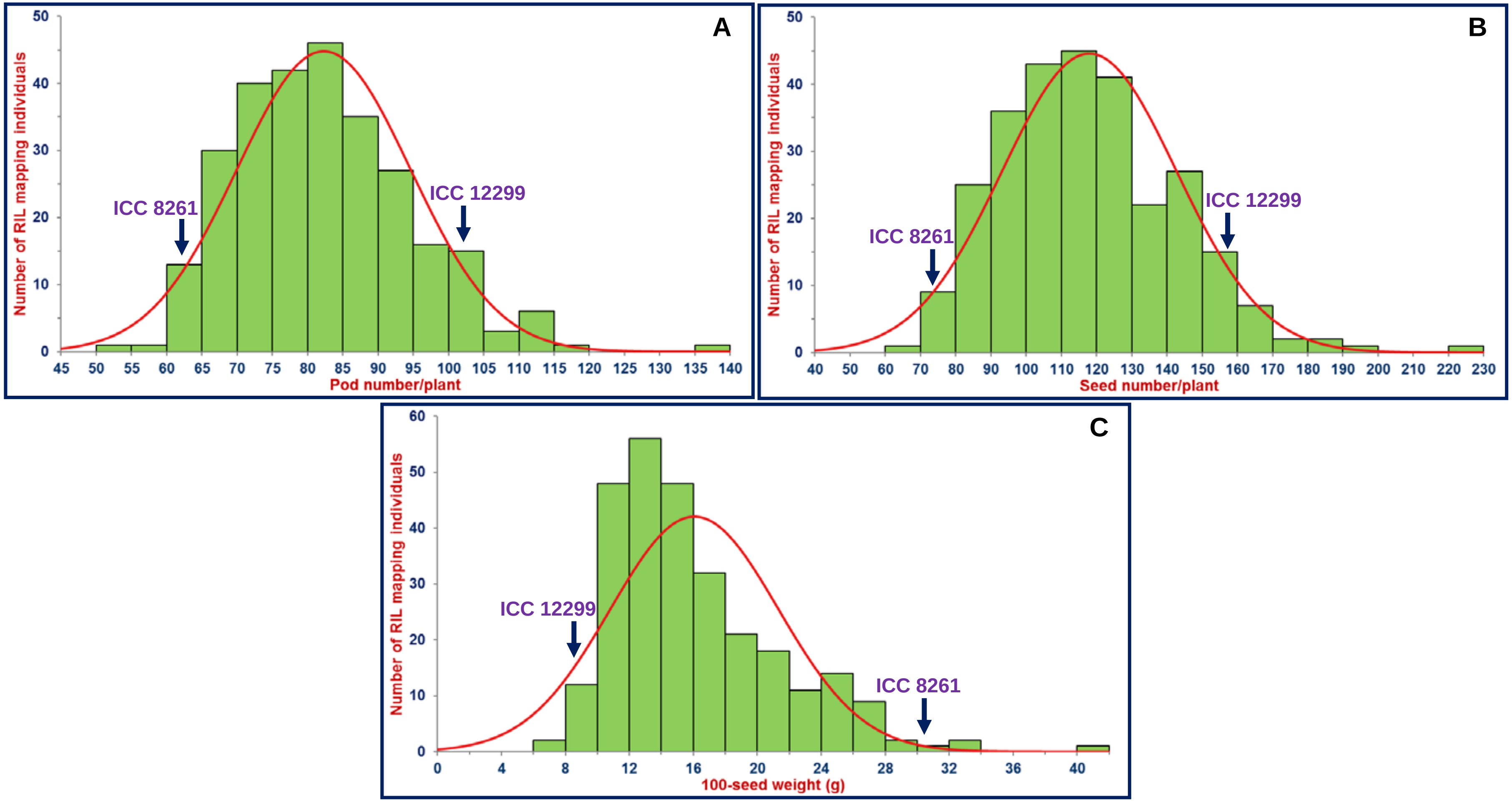

Figure S8: Frequency distribution of three quantitative agronomic traits, namely PN (A), SN (B) and SW (C) in 275 mapping individuals and parental genotypes of a F_7 RIL population (ICC 12299 x ICC 8261) depicted bi-directional transgressive segregation including goodness of fit to the normal distribution.

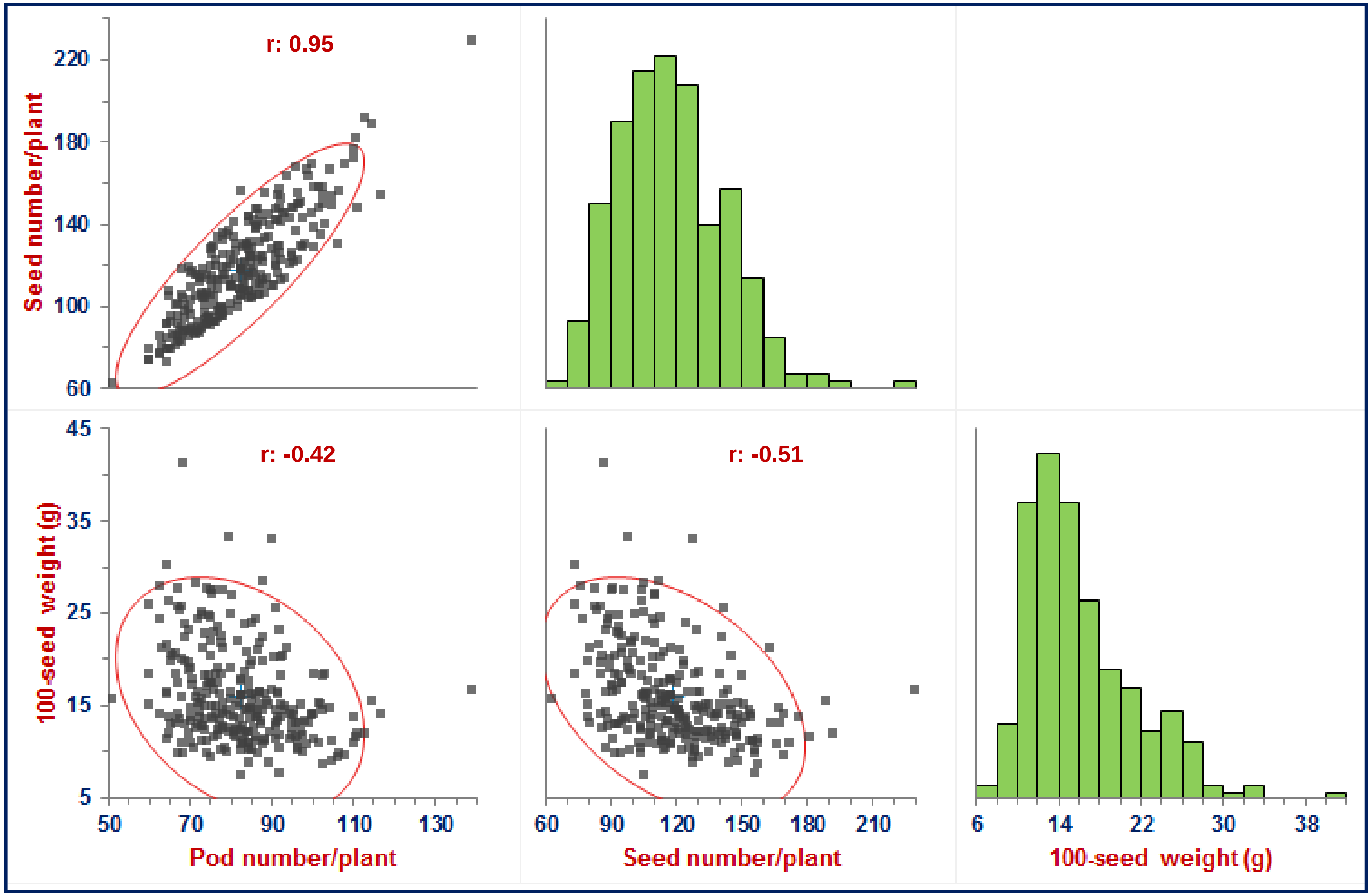

Figure S9: Pearson's correlation coefficients (r) estimated among three quantitative agronomic traits (PN, SN and SW) in a RIL mapping population (ICC 12299 x ICC 8261).

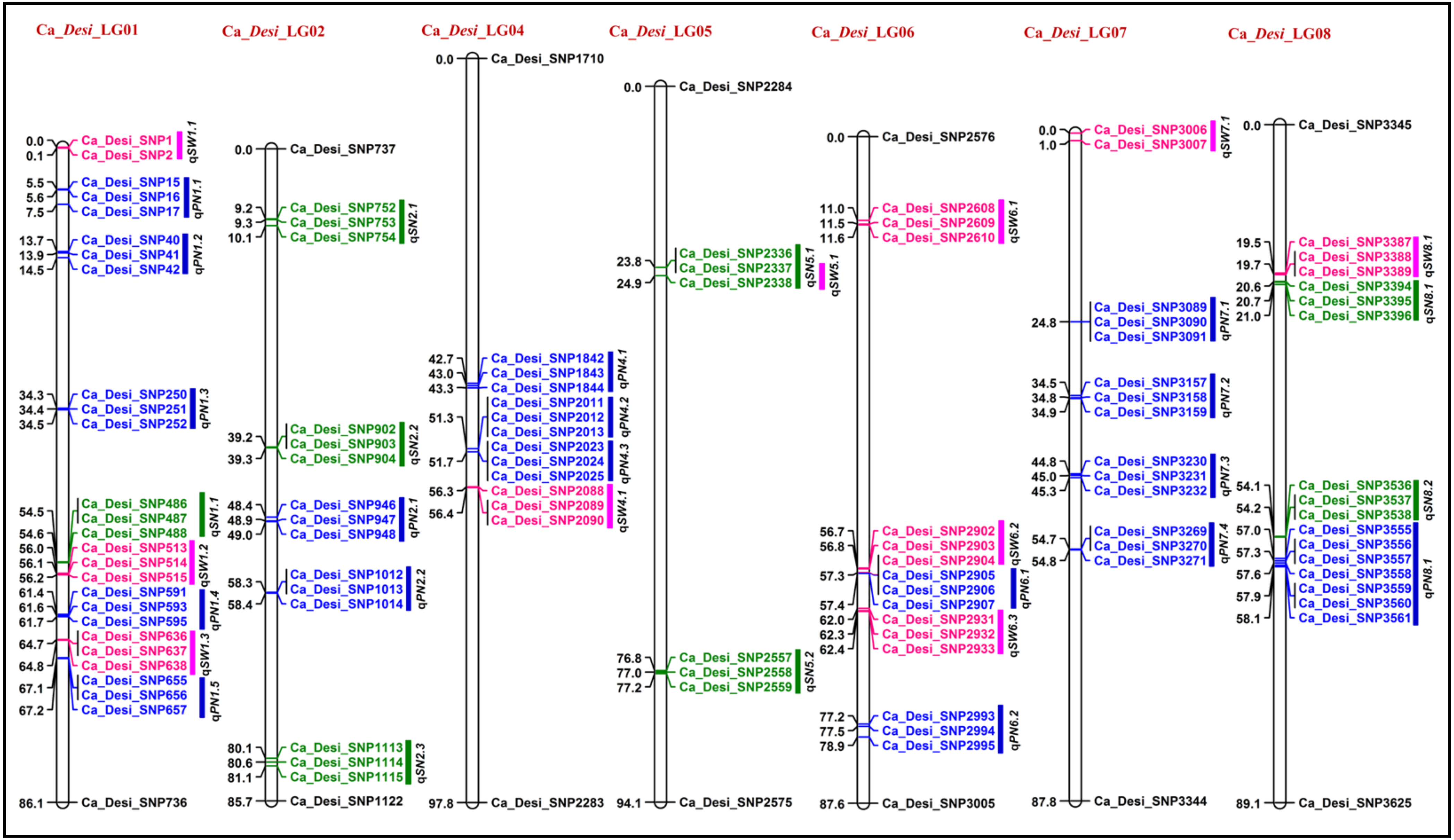

Figure S10: Thirty-three major genomic regions harbouring 35 significant robust QTLs (PVE 17.9-39.7%) associated with PN, SN and SW identified and mapped on seven *desi* LGs of an ultra-high density intra-specific genetic map (ICC 12299 x ICC 8261) with a significant LOD >5.0 ($p < 0.05$). The genetic distance (cM) and identity of the SNP loci integrated on the LGs are indicated on the left and right side of the LGs, respectively. Blue, green and pink lines represent the robust QTLs governing PN, SN and SW mapped on seven LGs and validated across geographical locations as well as years/seasons in field.

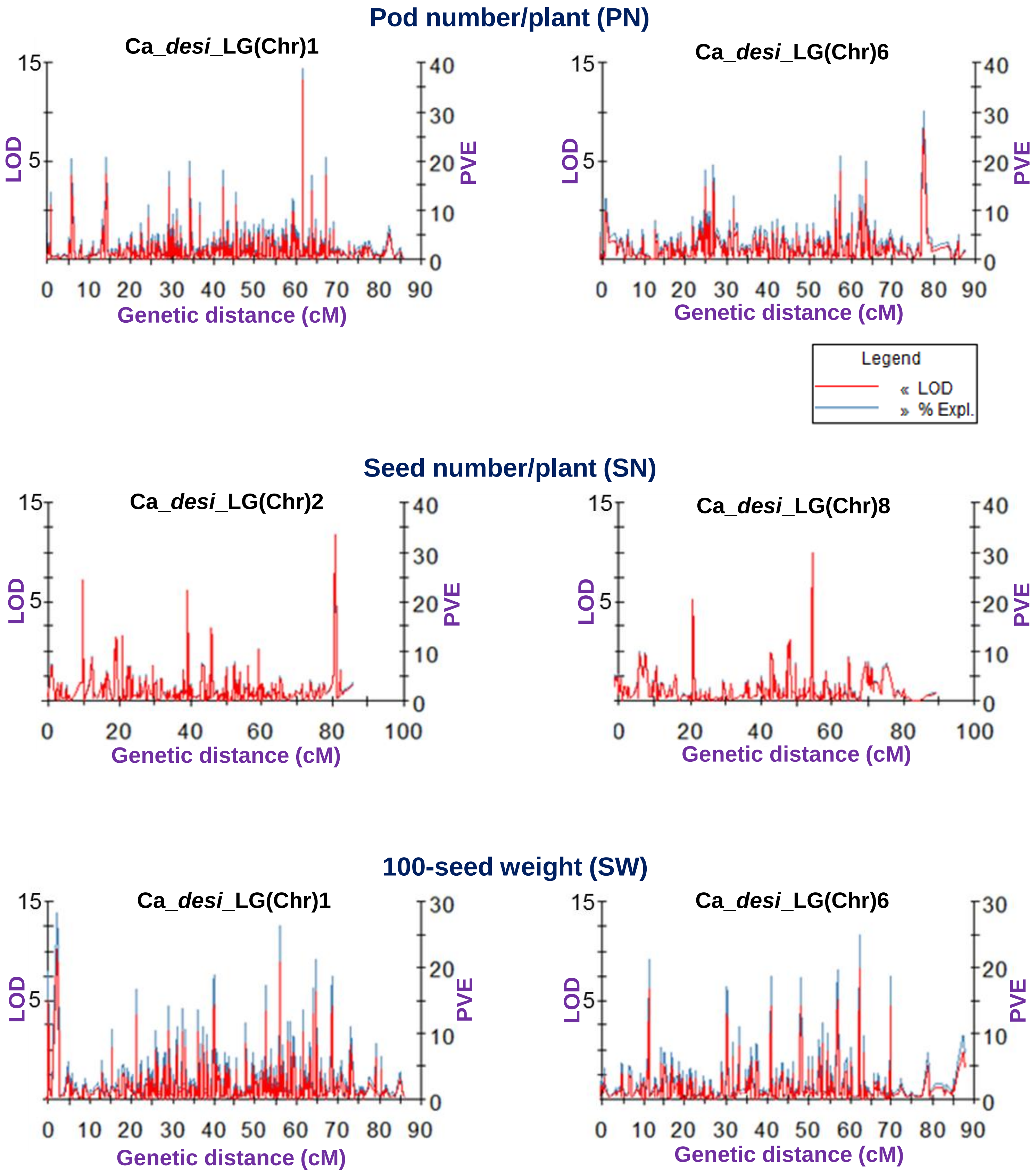

Figure S11: A representative figure depicting the PVE and peak LOD scores of SNP markers at major genomic regions harbouring the robust PN, SN and SW QTLs that were identified and mapped on *desi* LGs of an ultra-high density intra-specific genetic linkage map (ICC 12299 x ICC 8261). The x-axis indicates the genetic distance (cM) among SNP loci mapped at significant QTL regions of a linkage map, while y-axis shows the LOD and PVE estimates of SNPs covering the target QTL interval.

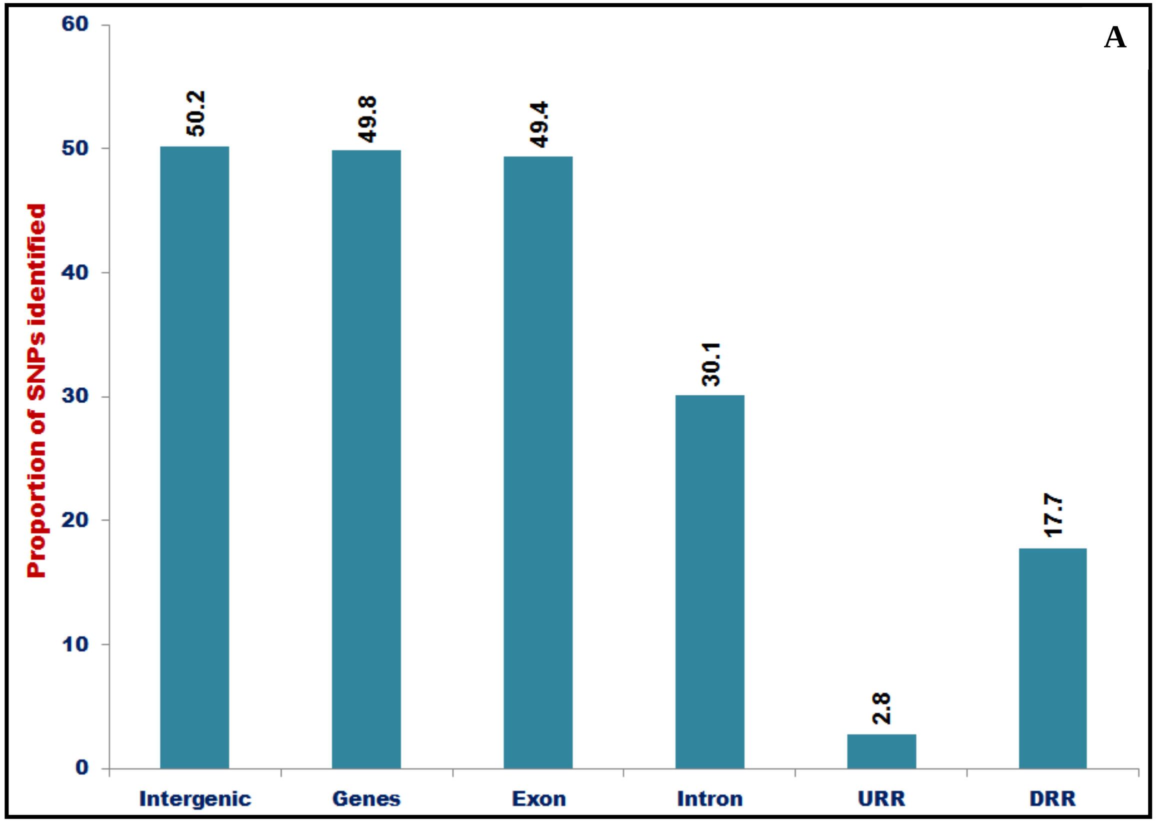

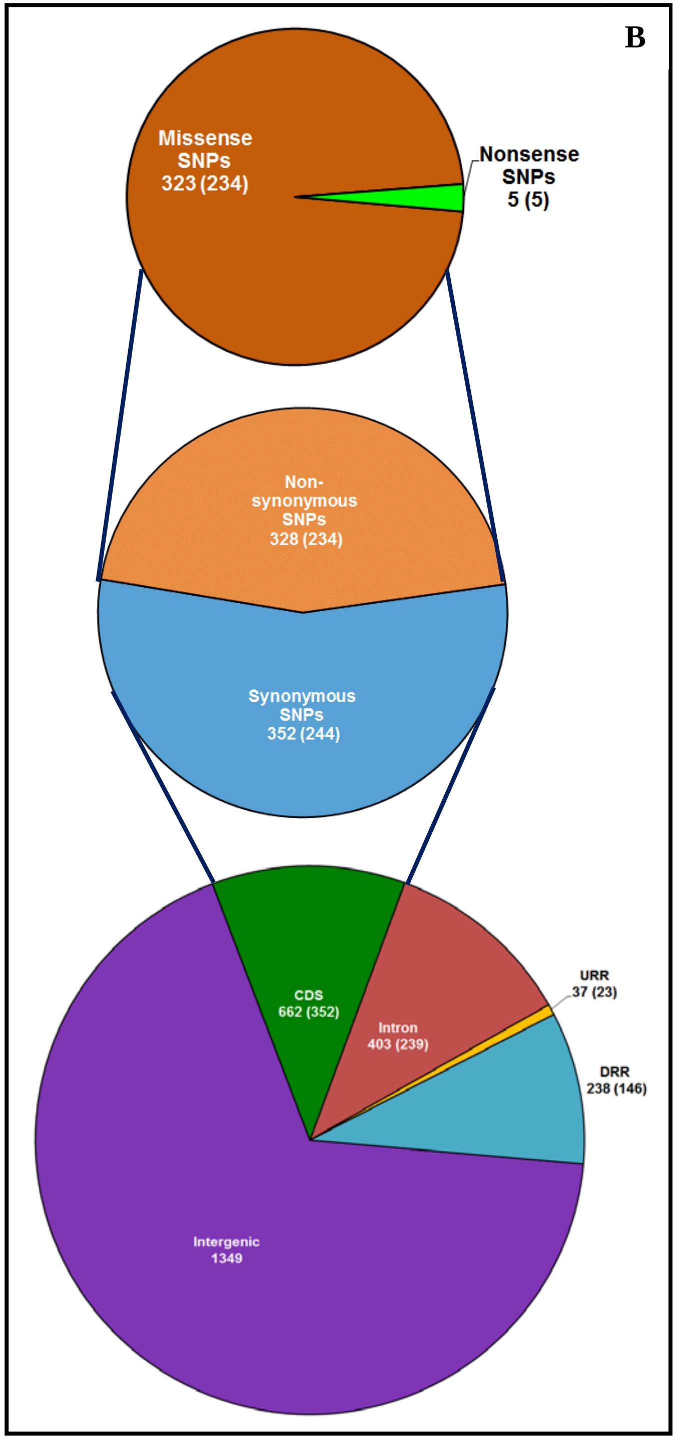

Figure S12: (A) Proportionate distribution of 2177 SNPs identified in different coding and non-coding sequence components and intergenic regions of 760 genes annotated from *kabuli* chickpea genome. (B) Number of SNPs, including synonymous and non-synonymous (missense and nonsense) SNPs annotated in the coding (CDS) as well as non-coding (intronic and regulatory) sequences of genes and intergenic regions of *kabuli* chickpea genome. The URR (upstream regulatory region) and DRR (downstream regulatory region) of genes were defined as 2000-bp upstream sequences from the translation start codon (ATG) and 1000-bp downstream sequences from the stop codon, respectively.

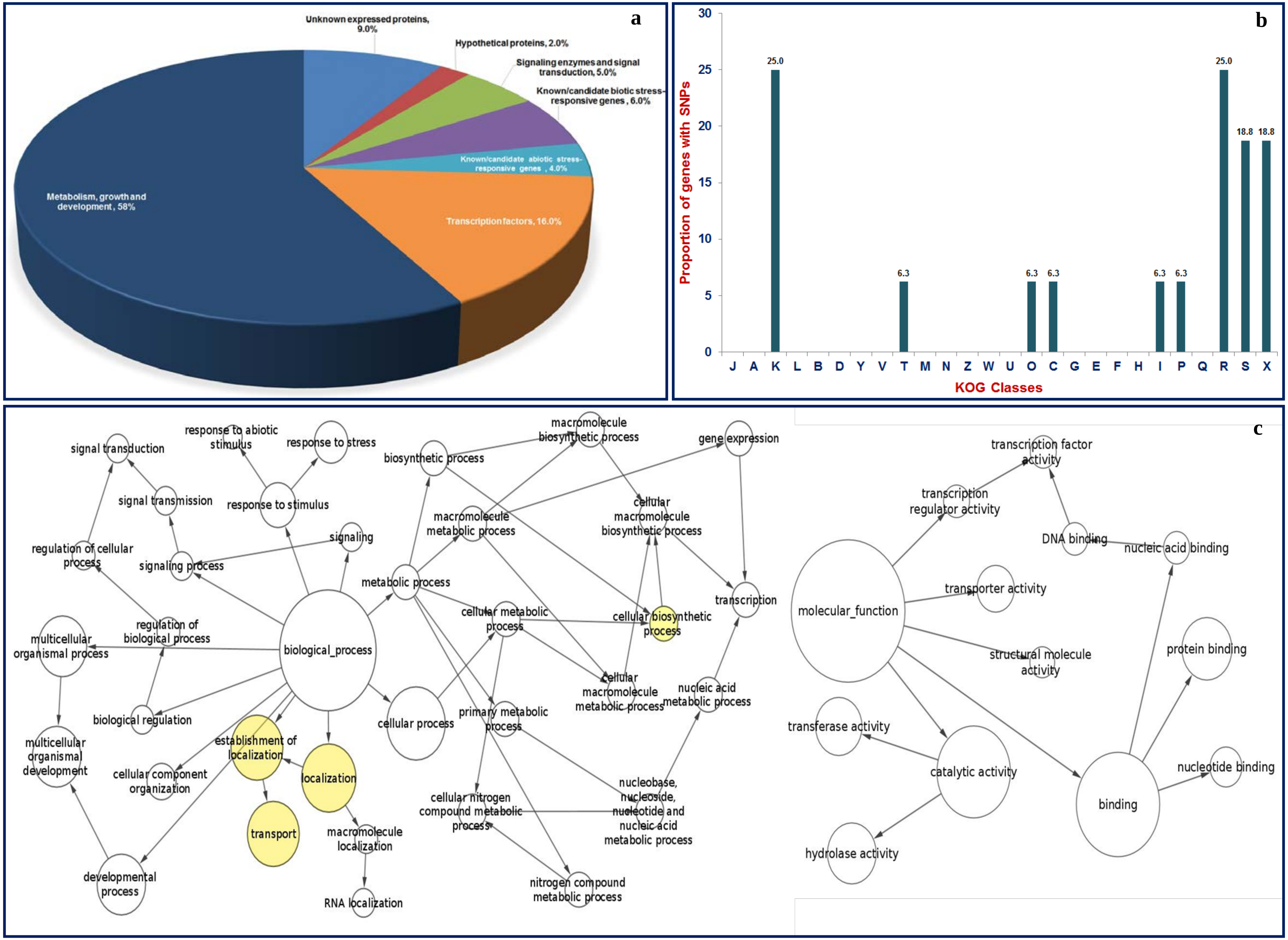

Figure S13: (a) Functional annotation of SNP-containing 635 *desi* chickpea genes. (b) KOG-based putative function determination of *desi* genes identified with SNPs. The diverse KOG classes are defined as K: Transcription, T: Signal transduction mechanisms, O: Post-translational modification, protein turnover and chaperones, C: Energy production and conversion, I: Lipid transport and metabolism, P: Inorganic ion transport and metabolism, R: General function prediction only, S: Function unknown and X: Unnamed proteins. (c) Gene ontology (GO) enrichment analysis of the SNPs-carrying *desi* genes. The number of genes in each category is directly proportional to the node size. The nodes were colour shaded (white and yellow) according to the significance level (P value <0.05).

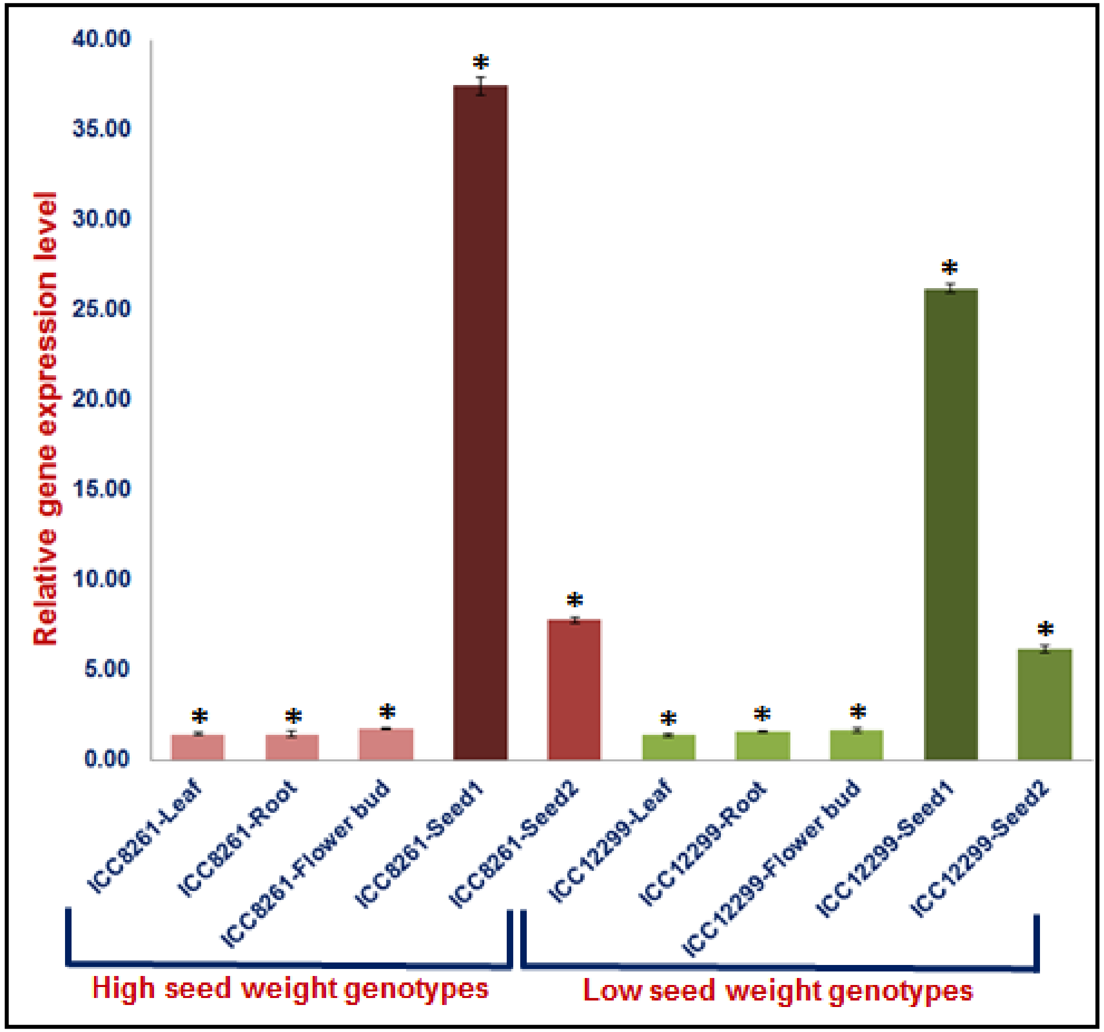

Figure S14: Differential expression profiling of a strong SW-associated embryo defective gene in three different vegetative (leaf and root) and reproductive (flower bud) tissues, as well as seed developmental stages of high (ICC 8261) and low (ICC 12299) seed weight parental genotypes of a RIL mapping population (ICC 12299 x ICC 8261) using quantitative RT-PCR assay. The *elongation factor-1 alpha* gene was used as the internal control in the RT-PCR. The bars indicate the standard error. *Significance at $p \leq 0.001$.

Table S1: Genomic distribution of SNPs physically mapped on eight *desi* and *kabuli* chickpea chromosomes

Chromosomes	Size (Mb) of chromosomes (pseudomolecules)		Number (%) of SNPs mapped		Average map density (kb)	
	Desi	***Kabuli***	***Desi***	***Kabuli***	***Desi***	***Kabuli***
Ca_Chr01	14.79	48.36	893 (11.2)	1944 (13.5)	16.6	24.9
Ca_Chr02	17.30	36.63	949 (11.9)	1088 (7.6)	18.2	33.7
Ca_Chr03	23.38	39.99	1548 (19.4)	1787 (12.4)	15.1	22.4
Ca_Chr04	22.09	49.19	1468 (18.4)	2854 (19.9)	15.0	17.2
Ca_Chr05	16.30	48.17	949 (11.9)	1735 (12.1)	17.2	27.8
Ca_Chr06	11.48	59.46	792 (9.9)	2229 (15.5)	14.5	26.7
Ca_Chr07	8.46	48.96	610 (7.7)	1931 (13.4)	13.9	25.4
Ca_Chr08	10.57	16.48	759 (9.5)	795 (5.5)	13.9	20.7
**Total**	**124.37**	**347.24**	**7968**	**14363**	**15.6**	**24.2**

Ca_Chr: *Cicer arietinum* Chromosome

Table S2: 43358 SNPs identified using reference *desi* and *kabuli* genome-based GBS assay showing differentiation between the parental genotypes (ICC 12299 and ICC 8261) of a RIL mapping population (ICC 12299 x ICC 8261)				
**SNP IDs**	**Chromosomes**	**Physical positions (bp)**	**SNPs**	**NCBI SNPdb accession IDs (NCBI_ss#)**
Ca_Kabuli_NC_021160.1_548	Ca_kabuli_chr01	548	[G/C]	1067289484
Ca_Kabuli_NC_021160.1_578	Ca_kabuli_chr01	578	[G/A]	1067289485
Ca_Kabuli_NC_021160.1_579	Ca_kabuli_chr01	579	[C/T]	1067289486
Ca_Kabuli_NC_021160.1_586	Ca_kabuli_chr01	586	[G/C]	1067289487
Ca_Kabuli_NC_021160.1_824	Ca_kabuli_chr01	824	[A/G]	1067289488
Ca_Kabuli_NC_021160.1_66934	Ca_kabuli_chr01	66934	[A/G]	1067289489
Ca_Kabuli_NC_021160.1_66944	Ca_kabuli_chr01	66944	[T/C]	1067289490
Ca_Kabuli_NC_021160.1_128752	Ca_kabuli_chr01	128752	[A/G]	1067289491
Ca_Kabuli_NC_021160.1_128768	Ca_kabuli_chr01	128768	[T/C]	1067289492
Ca_Kabuli_NC_021160.1_175476	Ca_kabuli_chr01	175476	[T/G]	1067289493
Ca_Kabuli_NC_021160.1_175489	Ca_kabuli_chr01	175489	[A/T]	1067289494
Ca_Kabuli_NC_021160.1_175529	Ca_kabuli_chr01	175529	[T/C]	1067289495
Ca_Kabuli_NC_021160.1_175544	Ca_kabuli_chr01	175544	[T/A]	1067289496
Ca_Kabuli_NC_021160.1_177441	Ca_kabuli_chr01	177441	[A/G]	1067289497
Ca_Kabuli_NC_021160.1_177449	Ca_kabuli_chr01	177449	[T/A]	1067289498
Ca_Kabuli_NC_021160.1_177513	Ca_kabuli_chr01	177513	[A/C]	1067289499
Ca_Kabuli_NC_021160.1_208931	Ca_kabuli_chr01	208931	[T/G]	1067289500
Ca_Kabuli_NC_021160.1_208998	Ca_kabuli_chr01	208998	[A/C]	1067289501
Ca_Kabuli_NC_021160.1_215042	Ca_kabuli_chr01	215042	[C/T]	1067289502
Ca_Kabuli_NC_021160.1_215048	Ca_kabuli_chr01	215048	[C/T]	1067289503
Ca_Kabuli_NC_021160.1_215053	Ca_kabuli_chr01	215053	[G/T]	1067289504
Ca_Kabuli_NC_021160.1_215058	Ca_kabuli_chr01	215058	[C/T]	1067289505
Ca_Kabuli_NC_021160.1_314255	Ca_kabuli_chr01	314255	[A/C]	1067289506
Ca_Kabuli_NC_021160.1_347520	Ca_kabuli_chr01	347520	[T/C]	1067289507
Ca_Kabuli_NC_021160.1_347541	Ca_kabuli_chr01	347541	[T/G]	1067289508
Ca_Kabuli_NC_021160.1_347544	Ca_kabuli_chr01	347544	[A/G]	1067289509
Ca_Kabuli_NC_021160.1_347572	Ca_kabuli_chr01	347572	[T/C]	1067289510
Ca_Kabuli_NC_021160.1_347579	Ca_kabuli_chr01	347579	[A/C]	1067289511
Ca_Kabuli_NC_021160.1_347581	Ca_kabuli_chr01	347581	[T/G]	1067289512
Ca_Kabuli_NC_021160.1_347600	Ca_kabuli_chr01	347600	[C/A]	1067289513
Ca_Kabuli_NC_021160.1_347620	Ca_kabuli_chr01	347620	[A/C]	1067289514

SNP IDs	Chromosomes	Physical positions (bp)	SNPs	NCBI SNPdb accession IDs (NCBI_ss#)
Ca_Kabuli_NC_021160.1_406585	Ca_kabuli_chr01	406585	[T/A]	1067289515
Ca_Kabuli_NC_021160.1_406609	Ca_kabuli_chr01	406609	[A/C]	1067289516
Ca_Kabuli_NC_021160.1_411808	Ca_kabuli_chr01	411808	[A/C]	1067289517
Ca_Kabuli_NC_021160.1_411816	Ca_kabuli_chr01	411816	[A/C]	1067289518
Ca_Kabuli_NC_021160.1_412550	Ca_kabuli_chr01	412550	[T/C]	1067289519
Ca_Kabuli_NC_021160.1_435273	Ca_kabuli_chr01	435273	[T/G]	1067289520
Ca_Kabuli_NC_021160.1_435274	Ca_kabuli_chr01	435274	[G/T]	1067289521
Ca_Kabuli_NC_021160.1_435275	Ca_kabuli_chr01	435275	[T/G]	1067289522
Ca_Kabuli_NC_021160.1_439736	Ca_kabuli_chr01	439736	[C/A]	1067289523
Ca_Kabuli_NC_021160.1_446443	Ca_kabuli_chr01	446443	[A/C]	1067289524
Ca_Kabuli_NC_021160.1_446450	Ca_kabuli_chr01	446450	[A/C]	1067289525
Ca_Kabuli_NC_021160.1_456630	Ca_kabuli_chr01	456630	[A/G]	1067289526
Ca_Kabuli_NC_021160.1_460015	Ca_kabuli_chr01	460015	[A/G]	1067289527
Ca_Kabuli_NC_021160.1_460017	Ca_kabuli_chr01	460017	[A/G]	1067289528
Ca_Kabuli_NC_021160.1_462814	Ca_kabuli_chr01	462814	[C/A]	1067289529
Ca_Kabuli_NC_021160.1_469512	Ca_kabuli_chr01	469512	[A/G]	1067289530
Ca_Kabuli_NC_021160.1_501991	Ca_kabuli_chr01	501991	[A/C]	1067289531
Ca_Kabuli_NC_021160.1_515995	Ca_kabuli_chr01	515995	[A/C]	1067289532
Ca_Kabuli_NC_021160.1_550453	Ca_kabuli_chr01	550453	[A/C]	1067289533
Ca_Kabuli_NC_021160.1_567381	Ca_kabuli_chr01	567381	[A/G]	1067289534
Ca_Kabuli_NC_021160.1_570997	Ca_kabuli_chr01	570997	[G/A]	1067289535
Ca_Kabuli_NC_021160.1_571002	Ca_kabuli_chr01	571002	[A/G]	1067289536
Ca_Kabuli_NC_021160.1_581039	Ca_kabuli_chr01	581039	[A/C]	1067289537
Ca_Kabuli_NC_021160.1_581154	Ca_kabuli_chr01	581154	[T/G]	1067289538
Ca_Kabuli_NC_021160.1_581199	Ca_kabuli_chr01	581199	[A/G]	1067289539
Ca_Kabuli_NC_021160.1_581365	Ca_kabuli_chr01	581365	[A/G]	1067289540
Ca_Kabuli_NC_021160.1_591765	Ca_kabuli_chr01	591765	[T/A]	1067289541
Ca_Kabuli_NC_021160.1_645358	Ca_kabuli_chr01	645358	[T/C]	1067289542
Ca_Kabuli_NC_021160.1_662573	Ca_kabuli_chr01	662573	[A/G]	1067289543
Ca_Kabuli_NC_021160.1_662611	Ca_kabuli_chr01	662611	[T/G]	1067289544
Ca_Kabuli_NC_021160.1_671345	Ca_kabuli_chr01	671345	[T/G]	1067289545
Ca_Kabuli_NC_021160.1_685641	Ca_kabuli_chr01	685641	[A/G]	1067289546
Ca_Kabuli_NC_021160.1_685650	Ca_kabuli_chr01	685650	[A/G]	1067289547
Ca_Kabuli_NC_021160.1_685679	Ca_kabuli_chr01	685679	[A/G]	1067289548

SNP IDs	Chromosomes	Physical positions (bp)	SNPs	NCBI SNPdb accession IDs (NCBI_ss#)
Ca_Kabuli_NC_021160.1_685686	Ca_kabuli_chr01	685686	[T/C]	1067289549
Ca_Kabuli_NC_021160.1_696914	Ca_kabuli_chr01	696914	[T/G]	1067289550
Ca_Kabuli_NC_021160.1_704102	Ca_kabuli_chr01	704102	[T/C]	1067289551
Ca_Kabuli_NC_021160.1_706166	Ca_kabuli_chr01	706166	[T/C]	1067289552
Ca_Kabuli_NC_021160.1_706190	Ca_kabuli_chr01	706190	[T/A]	1067289553
Ca_Kabuli_NC_021160.1_720109	Ca_kabuli_chr01	720109	[G/T]	1067289554
Ca_Kabuli_NC_021160.1_741535	Ca_kabuli_chr01	741535	[C/T]	1067289555
Ca_Kabuli_NC_021160.1_742033	Ca_kabuli_chr01	742033	[T/G]	1067289556
Ca_Kabuli_NC_021160.1_746096	Ca_kabuli_chr01	746096	[T/C]	1067289557
Ca_Kabuli_NC_021160.1_750693	Ca_kabuli_chr01	750693	[C/T]	1067289558
Ca_Kabuli_NC_021160.1_777240	Ca_kabuli_chr01	777240	[G/A]	1067289559
Ca_Kabuli_NC_021160.1_777273	Ca_kabuli_chr01	777273	[C/T]	1067289560
Ca_Kabuli_NC_021160.1_777556	Ca_kabuli_chr01	777556	[C/G]	1067289561
Ca_Kabuli_NC_021160.1_777557	Ca_kabuli_chr01	777557	[T/G]	1067289562
Ca_Kabuli_NC_021160.1_790696	Ca_kabuli_chr01	790696	[G/T]	1067289563
Ca_Kabuli_NC_021160.1_866835	Ca_kabuli_chr01	866835	[T/G]	1067289564
Ca_Kabuli_NC_021160.1_866889	Ca_kabuli_chr01	866889	[A/C]	1067289565
Ca_Kabuli_NC_021160.1_872327	Ca_kabuli_chr01	872327	[T/G]	1067289566
Ca_Kabuli_NC_021160.1_872372	Ca_kabuli_chr01	872372	[A/T]	1067289567
Ca_Kabuli_NC_021160.1_872379	Ca_kabuli_chr01	872379	[A/G]	1067289568
Ca_Kabuli_NC_021160.1_881379	Ca_kabuli_chr01	881379	[T/C]	1067289569
Ca_Kabuli_NC_021160.1_881484	Ca_kabuli_chr01	881484	[T/G]	1067289570
Ca_Kabuli_NC_021160.1_881491	Ca_kabuli_chr01	881491	[T/G]	1067289571
Ca_Kabuli_NC_021160.1_881514	Ca_kabuli_chr01	881514	[T/G]	1067289572
Ca_Kabuli_NC_021160.1_881567	Ca_kabuli_chr01	881567	[T/A]	1067289573
Ca_Kabuli_NC_021160.1_881603	Ca_kabuli_chr01	881603	[C/A]	1067289574
Ca_Kabuli_NC_021160.1_881741	Ca_kabuli_chr01	881741	[A/G]	1067289575
Ca_Kabuli_NC_021160.1_910457	Ca_kabuli_chr01	910457	[T/A]	1067289576
Ca_Kabuli_NC_021160.1_910624	Ca_kabuli_chr01	910624	[A/C]	1067289577
Ca_Kabuli_NC_021160.1_929474	Ca_kabuli_chr01	929474	[A/G]	1067289578
Ca_Kabuli_NC_021160.1_929497	Ca_kabuli_chr01	929497	[A/C]	1067289579
Ca_Kabuli_NC_021160.1_929504	Ca_kabuli_chr01	929504	[T/C]	1067289580
Ca_Kabuli_NC_021160.1_929517	Ca_kabuli_chr01	929517	[T/G]	1067289581
Ca_Kabuli_NC_021160.1_929533	Ca_kabuli_chr01	929533	[T/C]	1067289582

SNP IDs	Chromosomes	Physical positions (bp)	SNPs	NCBI SNPdb accession IDs (NCBI_ss#)
Ca_Kabuli_NC_021160.1_929582	Ca_kabuli_chr01	929582	[T/G]	1067289583
Ca_Kabuli_NC_021160.1_930042	Ca_kabuli_chr01	930042	[A/C]	1067289584
Ca_Kabuli_NC_021160.1_996350	Ca_kabuli_chr01	996350	[G/T]	1067289585
Ca_Kabuli_NC_021160.1_996382	Ca_kabuli_chr01	996382	[A/T]	1067289586
Ca_Kabuli_NC_021160.1_1007741	Ca_kabuli_chr01	1007741	[A/C]	1067289587
Ca_Kabuli_NC_021160.1_1018924	Ca_kabuli_chr01	1018924	[T/C]	1067289588
Ca_Kabuli_NC_021160.1_1018934	Ca_kabuli_chr01	1018934	[A/G]	1067289589
Ca_Kabuli_NC_021160.1_1018971	Ca_kabuli_chr01	1018971	[T/A]	1067289590
Ca_Kabuli_NC_021160.1_1023523	Ca_kabuli_chr01	1023523	[T/C]	1067289591
Ca_Kabuli_NC_021160.1_1023539	Ca_kabuli_chr01	1023539	[A/C]	1067289592
Ca_Kabuli_NC_021160.1_1026178	Ca_kabuli_chr01	1026178	[A/G]	1067289593
Ca_Kabuli_NC_021160.1_1085254	Ca_kabuli_chr01	1085254	[A/C]	1067289594
Ca_Kabuli_NC_021160.1_1085263	Ca_kabuli_chr01	1085263	[A/C]	1067289595
Ca_Kabuli_NC_021160.1_1085323	Ca_kabuli_chr01	1085323	[A/T]	1067289596
Ca_Kabuli_NC_021160.1_1092487	Ca_kabuli_chr01	1092487	[A/T]	1067289597
Ca_Kabuli_NC_021160.1_1092494	Ca_kabuli_chr01	1092494	[T/C]	1067289598
Ca_Kabuli_NC_021160.1_1092495	Ca_kabuli_chr01	1092495	[C/T]	1067289599
Ca_Kabuli_NC_021160.1_1092566	Ca_kabuli_chr01	1092566	[A/C]	1067289600
Ca_Kabuli_NC_021160.1_1110408	Ca_kabuli_chr01	1110408	[T/C]	1067289601
Ca_Kabuli_NC_021160.1_1110435	Ca_kabuli_chr01	1110435	[T/G]	1067289602
Ca_Kabuli_NC_021160.1_1110440	Ca_kabuli_chr01	1110440	[A/G]	1067289603
Ca_Kabuli_NC_021160.1_1115360	Ca_kabuli_chr01	1115360	[A/C]	1067289604
Ca_Kabuli_NC_021160.1_1115425	Ca_kabuli_chr01	1115425	[A/G]	1067289605
Ca_Kabuli_NC_021160.1_1116012	Ca_kabuli_chr01	1116012	[A/C]	1067289606
Ca_Kabuli_NC_021160.1_1116018	Ca_kabuli_chr01	1116018	[T/C]	1067289607
Ca_Kabuli_NC_021160.1_1116051	Ca_kabuli_chr01	1116051	[A/G]	1067289608
Ca_Kabuli_NC_021160.1_1117444	Ca_kabuli_chr01	1117444	[G/T]	1067289609
Ca_Kabuli_NC_021160.1_1117450	Ca_kabuli_chr01	1117450	[G/T]	1067289610
Ca_Kabuli_NC_021160.1_1117455	Ca_kabuli_chr01	1117455	[G/A]	1067289611
Ca_Kabuli_NC_021160.1_1117458	Ca_kabuli_chr01	1117458	[A/G]	1067289612
Ca_Kabuli_NC_021160.1_1117461	Ca_kabuli_chr01	1117461	[C/G]	1067289613
Ca_Kabuli_NC_021160.1_1117632	Ca_kabuli_chr01	1117632	[T/G]	1067289614
Ca_Kabuli_NC_021160.1_1140008	Ca_kabuli_chr01	1140008	[T/C]	1067289615
Ca_Kabuli_NC_021160.1_1140062	Ca_kabuli_chr01	1140062	[A/G]	1067289616

SNP IDs	Chromosomes	Physical positions (bp)	SNPs	NCBI SNPdb accession IDs (NCBI_ss#)
Ca_Kabuli_NC_021160.1_1140071	Ca_kabuli_chr01	1140071	[A/G]	1067289617
Ca_Kabuli_NC_021160.1_1140313	Ca_kabuli_chr01	1140313	[A/C]	1067289618
Ca_Kabuli_NC_021160.1_1140950	Ca_kabuli_chr01	1140950	[A/C]	1067289619
Ca_Kabuli_NC_021160.1_1150972	Ca_kabuli_chr01	1150972	[A/C]	1067289620
Ca_Kabuli_NC_021160.1_1150996	Ca_kabuli_chr01	1150996	[A/C]	1067289621
Ca_Kabuli_NC_021160.1_1159421	Ca_kabuli_chr01	1159421	[A/C]	1067289622
Ca_Kabuli_NC_021160.1_1159493	Ca_kabuli_chr01	1159493	[T/C]	1067289623
Ca_Kabuli_NC_021160.1_1159524	Ca_kabuli_chr01	1159524	[A/G]	1067289624
Ca_Kabuli_NC_021160.1_1159534	Ca_kabuli_chr01	1159534	[A/G]	1067289625
Ca_Kabuli_NC_021160.1_1292470	Ca_kabuli_chr01	1292470	[C/A]	1067289626
Ca_Kabuli_NC_021160.1_1292471	Ca_kabuli_chr01	1292471	[A/T]	1067289627
Ca_Kabuli_NC_021160.1_1292472	Ca_kabuli_chr01	1292472	[A/C]	1067289628
Ca_Kabuli_NC_021160.1_1292473	Ca_kabuli_chr01	1292473	[G/T]	1067289629
Ca_Kabuli_NC_021160.1_1295534	Ca_kabuli_chr01	1295534	[T/C]	1067289630
Ca_Kabuli_NC_021160.1_1295549	Ca_kabuli_chr01	1295549	[T/G]	1067289631
Ca_Kabuli_NC_021160.1_1335478	Ca_kabuli_chr01	1335478	[T/A]	1067289632
Ca_Kabuli_NC_021160.1_1335528	Ca_kabuli_chr01	1335528	[T/C]	1067289633
Ca_Kabuli_NC_021160.1_1344050	Ca_kabuli_chr01	1344050	[A/T]	1067289634
Ca_Kabuli_NC_021160.1_1344076	Ca_kabuli_chr01	1344076	[T/C]	1067289635
Ca_Kabuli_NC_021160.1_1344082	Ca_kabuli_chr01	1344082	[T/C]	1067289636
Ca_Kabuli_NC_021160.1_1344095	Ca_kabuli_chr01	1344095	[A/T]	1067289637
Ca_Kabuli_NC_021160.1_1430182	Ca_kabuli_chr01	1430182	[A/G]	1067289638
Ca_Kabuli_NC_021160.1_1434713	Ca_kabuli_chr01	1434713	[T/G]	1067289639
Ca_Kabuli_NC_021160.1_1434724	Ca_kabuli_chr01	1434724	[T/C]	1067289640
Ca_Kabuli_NC_021160.1_1449812	Ca_kabuli_chr01	1449812	[T/A]	1067289641
Ca_Kabuli_NC_021160.1_1449954	Ca_kabuli_chr01	1449954	[T/G]	1067289642
Ca_Kabuli_NC_021160.1_1449972	Ca_kabuli_chr01	1449972	[A/C]	1067289643
Ca_Kabuli_NC_021160.1_1449987	Ca_kabuli_chr01	1449987	[T/C]	1067289644
Ca_Kabuli_NC_021160.1_1461574	Ca_kabuli_chr01	1461574	[T/G]	1067289645
Ca_Kabuli_NC_021160.1_1527423	Ca_kabuli_chr01	1527423	[T/C]	1067289646
Ca_Kabuli_NC_021160.1_1527513	Ca_kabuli_chr01	1527513	[A/G]	1067289647
Ca_Kabuli_NC_021160.1_1528325	Ca_kabuli_chr01	1528325	[A/G]	1067289648
Ca_Kabuli_NC_021160.1_1546603	Ca_kabuli_chr01	1546603	[T/G]	1067289649
Ca_Kabuli_NC_021160.1_1546615	Ca_kabuli_chr01	1546615	[T/G]	1067289650

SNP IDs	Chromosomes	Physical positions (bp)	SNPs	NCBI SNPdb accession IDs (NCBI_ss#)
Ca_Kabuli_NC_021160.1_1546631	Ca_kabuli_chr01	1546631	[T/G]	1067289651
Ca_Kabuli_NC_021160.1_1546688	Ca_kabuli_chr01	1546688	[A/C]	1067289652
Ca_Kabuli_NC_021160.1_1547809	Ca_kabuli_chr01	1547809	[T/A]	1067289653
Ca_Kabuli_NC_021160.1_1650119	Ca_kabuli_chr01	1650119	[T/C]	1067289654
Ca_Kabuli_NC_021160.1_1650130	Ca_kabuli_chr01	1650130	[T/C]	1067289655
Ca_Kabuli_NC_021160.1_1650141	Ca_kabuli_chr01	1650141	[T/C]	1067289656
Ca_Kabuli_NC_021160.1_1732879	Ca_kabuli_chr01	1732879	[G/C]	1067289657
Ca_Kabuli_NC_021160.1_1769544	Ca_kabuli_chr01	1769544	[T/C]	1067289658
Ca_Kabuli_NC_021160.1_1769581	Ca_kabuli_chr01	1769581	[A/C]	1067289659
Ca_Kabuli_NC_021160.1_1782760	Ca_kabuli_chr01	1782760	[G/A]	1067289660
Ca_Kabuli_NC_021160.1_1782793	Ca_kabuli_chr01	1782793	[G/T]	1067289661
Ca_Kabuli_NC_021160.1_1783207	Ca_kabuli_chr01	1783207	[G/A]	1067289662
Ca_Kabuli_NC_021160.1_1825840	Ca_kabuli_chr01	1825840	[G/A]	1067289663
Ca_Kabuli_NC_021160.1_1858156	Ca_kabuli_chr01	1858156	[T/C]	1067289664
Ca_Kabuli_NC_021160.1_1858159	Ca_kabuli_chr01	1858159	[A/G]	1067289665
Ca_Kabuli_NC_021160.1_1858161	Ca_kabuli_chr01	1858161	[A/C]	1067289666
Ca_Kabuli_NC_021160.1_1858177	Ca_kabuli_chr01	1858177	[T/C]	1067289667
Ca_Kabuli_NC_021160.1_1858202	Ca_kabuli_chr01	1858202	[T/C]	1067289668
Ca_Kabuli_NC_021160.1_1858226	Ca_kabuli_chr01	1858226	[G/A]	1067289669
Ca_Kabuli_NC_021160.1_1858509	Ca_kabuli_chr01	1858509	[C/T]	1067289670
Ca_Kabuli_NC_021160.1_1858717	Ca_kabuli_chr01	1858717	[A/G]	1067289671
Ca_Kabuli_NC_021160.1_1861696	Ca_kabuli_chr01	1861696	[C/T]	1067289672
Ca_Kabuli_NC_021160.1_1861707	Ca_kabuli_chr01	1861707	[T/G]	1067289673
Ca_Kabuli_NC_021160.1_1861837	Ca_kabuli_chr01	1861837	[T/G]	1067289674
Ca_Kabuli_NC_021160.1_1861887	Ca_kabuli_chr01	1861887	[G/A]	1067289675
Ca_Kabuli_NC_021160.1_1904230	Ca_kabuli_chr01	1904230	[G/T]	1067289676
Ca_Kabuli_NC_021160.1_1904238	Ca_kabuli_chr01	1904238	[A/G]	1067289677
Ca_Kabuli_NC_021160.1_1904281	Ca_kabuli_chr01	1904281	[T/A]	1067289678
Ca_Kabuli_NC_021160.1_1904334	Ca_kabuli_chr01	1904334	[T/G]	1067289679
Ca_Kabuli_NC_021160.1_1935519	Ca_kabuli_chr01	1935519	[T/A]	1067289680
Ca_Kabuli_NC_021160.1_1935557	Ca_kabuli_chr01	1935557	[A/T]	1067289681
Ca_Kabuli_NC_021160.1_1962690	Ca_kabuli_chr01	1962690	[A/C]	1067289682
Ca_Kabuli_NC_021160.1_1968750	Ca_kabuli_chr01	1968750	[T/G]	1067289683
Ca_Kabuli_NC_021160.1_1968823	Ca_kabuli_chr01	1968823	[G/A]	1067289684

SNP IDs	Chromosomes	Physical positions (bp)	SNPs	NCBI SNPdb accession IDs (NCBI_ss#)
Ca_Kabuli_NC_021160.1_1968826	Ca_kabuli_chr01	1968826	[G/A]	1067289685
Ca_Kabuli_NC_021160.1_1968828	Ca_kabuli_chr01	1968828	[G/A]	1067289686
Ca_Kabuli_NC_021160.1_1968837	Ca_kabuli_chr01	1968837	[A/G]	1067289687
Ca_Kabuli_NC_021160.1_1971936	Ca_kabuli_chr01	1971936	[T/G]	1067289688
Ca_Kabuli_NC_021160.1_1971964	Ca_kabuli_chr01	1971964	[A/C]	1067289689
Ca_Kabuli_NC_021160.1_1971975	Ca_kabuli_chr01	1971975	[A/T]	1067289690
Ca_Kabuli_NC_021160.1_2002932	Ca_kabuli_chr01	2002932	[A/G]	1067289691
Ca_Kabuli_NC_021160.1_2002944	Ca_kabuli_chr01	2002944	[A/G]	1067289692
Ca_Kabuli_NC_021160.1_2020960	Ca_kabuli_chr01	2020960	[T/C]	1067289693
Ca_Kabuli_NC_021160.1_2041027	Ca_kabuli_chr01	2041027	[C/G]	1067289694
Ca_Kabuli_NC_021160.1_2042071	Ca_kabuli_chr01	2042071	[C/G]	1067289695
Ca_Kabuli_NC_021160.1_2044359	Ca_kabuli_chr01	2044359	[C/T]	1067289696
Ca_Kabuli_NC_021160.1_2050368	Ca_kabuli_chr01	2050368	[T/C]	1067289697
Ca_Kabuli_NC_021160.1_2050386	Ca_kabuli_chr01	2050386	[T/G]	1067289698
Ca_Kabuli_NC_021160.1_2053744	Ca_kabuli_chr01	2053744	[C/A]	1067289699
Ca_Kabuli_NC_021160.1_2053784	Ca_kabuli_chr01	2053784	[C/A]	1067289700
Ca_Kabuli_NC_021160.1_2053787	Ca_kabuli_chr01	2053787	[C/A]	1067289701
Ca_Kabuli_NC_021160.1_2053788	Ca_kabuli_chr01	2053788	[A/G]	1067289702
Ca_Kabuli_NC_021160.1_2053819	Ca_kabuli_chr01	2053819	[C/A]	1067289703
Ca_Kabuli_NC_021160.1_2053830	Ca_kabuli_chr01	2053830	[A/G]	1067289704
Ca_Kabuli_NC_021160.1_2053833	Ca_kabuli_chr01	2053833	[T/G]	1067289705
Ca_Kabuli_NC_021160.1_2053856	Ca_kabuli_chr01	2053856	[T/G]	1067289706
Ca_Kabuli_NC_021160.1_2072627	Ca_kabuli_chr01	2072627	[A/T]	1067289707
Ca_Kabuli_NC_021160.1_2074894	Ca_kabuli_chr01	2074894	[G/C]	1067289708
Ca_Kabuli_NC_021160.1_2074900	Ca_kabuli_chr01	2074900	[G/A]	1067289709
Ca_Kabuli_NC_021160.1_2074902	Ca_kabuli_chr01	2074902	[A/G]	1067289710
Ca_Kabuli_NC_021160.1_2074904	Ca_kabuli_chr01	2074904	[A/T]	1067289711
Ca_Kabuli_NC_021160.1_2075012	Ca_kabuli_chr01	2075012	[C/A]	1067289712
Ca_Kabuli_NC_021160.1_2075013	Ca_kabuli_chr01	2075013	[A/G]	1067289713
Ca_Kabuli_NC_021160.1_2075015	Ca_kabuli_chr01	2075015	[A/G]	1067289714
Ca_Kabuli_NC_021160.1_2082760	Ca_kabuli_chr01	2082760	[C/G]	1067289715
Ca_Kabuli_NC_021160.1_2083003	Ca_kabuli_chr01	2083003	[G/C]	1067289716
Ca_Kabuli_NC_021160.1_2102245	Ca_kabuli_chr01	2102245	[G/A]	1067289717
Ca_Kabuli_NC_021160.1_2178832	Ca_kabuli_chr01	2178832	[C/A]	1067289718

SNP IDs	Chromosomes	Physical positions (bp)	SNPs	NCBI SNPdb accession IDs (NCBI_ss#)
Ca_Kabuli_NC_021160.1_2178833	Ca_kabuli_chr01	2178833	[T/G]	1067289719
Ca_Kabuli_NC_021160.1_2188439	Ca_kabuli_chr01	2188439	[T/G]	1067289720
Ca_Kabuli_NC_021160.1_2188498	Ca_kabuli_chr01	2188498	[A/G]	1067289721
Ca_Kabuli_NC_021160.1_2220109	Ca_kabuli_chr01	2220109	[C/T]	1067289722
Ca_Kabuli_NC_021160.1_2232774	Ca_kabuli_chr01	2232774	[A/T]	1067289723
Ca_Kabuli_NC_021160.1_2232775	Ca_kabuli_chr01	2232775	[A/T]	1067289724
Ca_Kabuli_NC_021160.1_2232777	Ca_kabuli_chr01	2232777	[T/G]	1067289725
Ca_Kabuli_NC_021160.1_2232778	Ca_kabuli_chr01	2232778	[T/A]	1067289726
Ca_Kabuli_NC_021160.1_2252270	Ca_kabuli_chr01	2252270	[C/T]	1067289727
Ca_Kabuli_NC_021160.1_2252473	Ca_kabuli_chr01	2252473	[G/A]	1067289728
Ca_Kabuli_NC_021160.1_2276929	Ca_kabuli_chr01	2276929	[C/T]	1067289729
Ca_Kabuli_NC_021160.1_2287391	Ca_kabuli_chr01	2287391	[T/A]	1067289730
Ca_Kabuli_NC_021160.1_2287408	Ca_kabuli_chr01	2287408	[A/T]	1067289731
Ca_Kabuli_NC_021160.1_2287427	Ca_kabuli_chr01	2287427	[C/A]	1067289732
Ca_Kabuli_NC_021160.1_2287439	Ca_kabuli_chr01	2287439	[T/A]	1067289733
Ca_Kabuli_NC_021160.1_2288179	Ca_kabuli_chr01	2288179	[A/T]	1067289734
Ca_Kabuli_NC_021160.1_2288186	Ca_kabuli_chr01	2288186	[A/C]	1067289735
Ca_Kabuli_NC_021160.1_2288207	Ca_kabuli_chr01	2288207	[T/G]	1067289736
Ca_Kabuli_NC_021160.1_2290092	Ca_kabuli_chr01	2290092	[A/G]	1067289737
Ca_Kabuli_NC_021160.1_2305216	Ca_kabuli_chr01	2305216	[T/G]	1067289738
Ca_Kabuli_NC_021160.1_2305246	Ca_kabuli_chr01	2305246	[T/A]	1067289739
Ca_Kabuli_NC_021160.1_2305477	Ca_kabuli_chr01	2305477	[C/T]	1067289740
Ca_Kabuli_NC_021160.1_2305786	Ca_kabuli_chr01	2305786	[A/T]	1067289741
Ca_Kabuli_NC_021160.1_2305837	Ca_kabuli_chr01	2305837	[G/T]	1067289742
Ca_Kabuli_NC_021160.1_2306002	Ca_kabuli_chr01	2306002	[G/A]	1067289743
Ca_Kabuli_NC_021160.1_2306323	Ca_kabuli_chr01	2306323	[C/T]	1067289744
Ca_Kabuli_NC_021160.1_2306340	Ca_kabuli_chr01	2306340	[T/C]	1067289745
Ca_Kabuli_NC_021160.1_2313615	Ca_kabuli_chr01	2313615	[A/G]	1067289746
Ca_Kabuli_NC_021160.1_2366246	Ca_kabuli_chr01	2366246	[A/G]	1067289747
Ca_Kabuli_NC_021160.1_2366251	Ca_kabuli_chr01	2366251	[A/G]	1067289748
Ca_Kabuli_NC_021160.1_2373480	Ca_kabuli_chr01	2373480	[T/G]	1067289749
Ca_Kabuli_NC_021160.1_2387858	Ca_kabuli_chr01	2387858	[T/C]	1067289750
Ca_Kabuli_NC_021160.1_2387861	Ca_kabuli_chr01	2387861	[A/G]	1067289751
Ca_Kabuli_NC_021160.1_2387876	Ca_kabuli_chr01	2387876	[A/C]	1067289752

SNP IDs	Chromosomes	Physical positions (bp)	SNPs	NCBI SNPdb accession IDs (NCBI_ss#)
Ca_Kabuli_NC_021160.1_2395804	Ca_kabuli_chr01	2395804	[A/T]	1067289753
Ca_Kabuli_NC_021160.1_2400287	Ca_kabuli_chr01	2400287	[C/A]	1067289754
Ca_Kabuli_NC_021160.1_2439915	Ca_kabuli_chr01	2439915	[G/A]	1067289755
Ca_Kabuli_NC_021160.1_2439917	Ca_kabuli_chr01	2439917	[A/G]	1067289756
Ca_Kabuli_NC_021160.1_2439970	Ca_kabuli_chr01	2439970	[C/A]	1067289757
Ca_Kabuli_NC_021160.1_2439971	Ca_kabuli_chr01	2439971	[A/C]	1067289758
Ca_Kabuli_NC_021160.1_2439972	Ca_kabuli_chr01	2439972	[A/C]	1067289759
Ca_Kabuli_NC_021160.1_2452204	Ca_kabuli_chr01	2452204	[C/G]	1067289760
Ca_Kabuli_NC_021160.1_2457898	Ca_kabuli_chr01	2457898	[A/G]	1067289761
Ca_Kabuli_NC_021160.1_2473309	Ca_kabuli_chr01	2473309	[T/C]	1067289762
Ca_Kabuli_NC_021160.1_2481257	Ca_kabuli_chr01	2481257	[A/T]	1067289763
Ca_Kabuli_NC_021160.1_2510835	Ca_kabuli_chr01	2510835	[T/A]	1067289764
Ca_Kabuli_NC_021160.1_2510854	Ca_kabuli_chr01	2510854	[A/C]	1067289765
Ca_Kabuli_NC_021160.1_2513880	Ca_kabuli_chr01	2513880	[A/G]	1067289766
Ca_Kabuli_NC_021160.1_2513902	Ca_kabuli_chr01	2513902	[T/C]	1067289767
Ca_Kabuli_NC_021160.1_2538961	Ca_kabuli_chr01	2538961	[T/C]	1067289768
Ca_Kabuli_NC_021160.1_2539571	Ca_kabuli_chr01	2539571	[T/G]	1067289769
Ca_Kabuli_NC_021160.1_2542639	Ca_kabuli_chr01	2542639	[T/C]	1067289770
Ca_Kabuli_NC_021160.1_2542648	Ca_kabuli_chr01	2542648	[G/T]	1067289771
Ca_Kabuli_NC_021160.1_2542652	Ca_kabuli_chr01	2542652	[G/A]	1067289772
Ca_Kabuli_NC_021160.1_2542653	Ca_kabuli_chr01	2542653	[C/A]	1067289773
Ca_Kabuli_NC_021160.1_2542658	Ca_kabuli_chr01	2542658	[G/A]	1067289774
Ca_Kabuli_NC_021160.1_2542705	Ca_kabuli_chr01	2542705	[T/A]	1067289775
Ca_Kabuli_NC_021160.1_2542727	Ca_kabuli_chr01	2542727	[T/A]	1067289776
Ca_Kabuli_NC_021160.1_2638990	Ca_kabuli_chr01	2638990	[A/T]	1067289777
Ca_Kabuli_NC_021160.1_2638991	Ca_kabuli_chr01	2638991	[T/G]	1067289778
Ca_Kabuli_NC_021160.1_2638992	Ca_kabuli_chr01	2638992	[C/G]	1067289779
Ca_Kabuli_NC_021160.1_2638993	Ca_kabuli_chr01	2638993	[T/G]	1067289780
Ca_Kabuli_NC_021160.1_2649712	Ca_kabuli_chr01	2649712	[A/G]	1067289781
Ca_Kabuli_NC_021160.1_2649739	Ca_kabuli_chr01	2649739	[A/T]	1067289782
Ca_Kabuli_NC_021160.1_2649792	Ca_kabuli_chr01	2649792	[A/G]	1067289783
Ca_Kabuli_NC_021160.1_2649872	Ca_kabuli_chr01	2649872	[A/C]	1067289784
Ca_Kabuli_NC_021160.1_2649905	Ca_kabuli_chr01	2649905	[T/C]	1067289785
Ca_Kabuli_NC_021160.1_2649916	Ca_kabuli_chr01	2649916	[T/C]	1067289786

SNP IDs	Chromosomes	Physical positions (bp)	SNPs	NCBI SNPdb accession IDs (NCBI_ss#)
Ca_Kabuli_NC_021160.1_2649963	Ca_kabuli_chr01	2649963	[A/G]	1067289787
Ca_Kabuli_NC_021160.1_2671432	Ca_kabuli_chr01	2671432	[T/G]	1067289788
Ca_Kabuli_NC_021160.1_2733653	Ca_kabuli_chr01	2733653	[T/G]	1067289789
Ca_Kabuli_NC_021160.1_2733669	Ca_kabuli_chr01	2733669	[T/G]	1067289790
Ca_Kabuli_NC_021160.1_2733701	Ca_kabuli_chr01	2733701	[G/T]	1067289791
Ca_Kabuli_NC_021160.1_2739513	Ca_kabuli_chr01	2739513	[A/G]	1067289792
Ca_Kabuli_NC_021160.1_2739522	Ca_kabuli_chr01	2739522	[A/G]	1067289793
Ca_Kabuli_NC_021160.1_2783500	Ca_kabuli_chr01	2783500	[T/A]	1067289794
Ca_Kabuli_NC_021160.1_2786629	Ca_kabuli_chr01	2786629	[A/C]	1067289795
Ca_Kabuli_NC_021160.1_2786970	Ca_kabuli_chr01	2786970	[T/A]	1067289796
Ca_Kabuli_NC_021160.1_2787024	Ca_kabuli_chr01	2787024	[A/G]	1067289797
Ca_Kabuli_NC_021160.1_2787040	Ca_kabuli_chr01	2787040	[A/G]	1067289798
Ca_Kabuli_NC_021160.1_2808035	Ca_kabuli_chr01	2808035	[C/A]	1067289799
Ca_Kabuli_NC_021160.1_2808039	Ca_kabuli_chr01	2808039	[A/G]	1067289800
Ca_Kabuli_NC_021160.1_2808079	Ca_kabuli_chr01	2808079	[T/G]	1067289801
Ca_Kabuli_NC_021160.1_2855971	Ca_kabuli_chr01	2855971	[T/C]	1067289802
Ca_Kabuli_NC_021160.1_2855972	Ca_kabuli_chr01	2855972	[G/T]	1067289803
Ca_Kabuli_NC_021160.1_2883189	Ca_kabuli_chr01	2883189	[G/C]	1067289804
Ca_Kabuli_NC_021160.1_2883194	Ca_kabuli_chr01	2883194	[T/C]	1067289805
Ca_Kabuli_NC_021160.1_2883201	Ca_kabuli_chr01	2883201	[G/A]	1067289806
Ca_Kabuli_NC_021160.1_2883663	Ca_kabuli_chr01	2883663	[C/A]	1067289807
Ca_Kabuli_NC_021160.1_2883664	Ca_kabuli_chr01	2883664	[A/G]	1067289808
Ca_Kabuli_NC_021160.1_2883684	Ca_kabuli_chr01	2883684	[G/T]	1067289809
Ca_Kabuli_NC_021160.1_2883716	Ca_kabuli_chr01	2883716	[A/C]	1067289810
Ca_Kabuli_NC_021160.1_2883717	Ca_kabuli_chr01	2883717	[C/A]	1067289811
Ca_Kabuli_NC_021160.1_2885508	Ca_kabuli_chr01	2885508	[C/T]	1067289812
Ca_Kabuli_NC_021160.1_2885525	Ca_kabuli_chr01	2885525	[C/T]	1067289813
Ca_Kabuli_NC_021160.1_2885987	Ca_kabuli_chr01	2885987	[A/T]	1067289814
Ca_Kabuli_NC_021160.1_2885988	Ca_kabuli_chr01	2885988	[G/C]	1067289815
Ca_Kabuli_NC_021160.1_2885997	Ca_kabuli_chr01	2885997	[A/G]	1067289816
Ca_Kabuli_NC_021160.1_2886005	Ca_kabuli_chr01	2886005	[A/G]	1067289817
Ca_Kabuli_NC_021160.1_2886442	Ca_kabuli_chr01	2886442	[A/G]	1067289818
Ca_Kabuli_NC_021160.1_2888375	Ca_kabuli_chr01	2888375	[A/C]	1067289819
Ca_Kabuli_NC_021160.1_2936921	Ca_kabuli_chr01	2936921	[A/C]	1067289820

SNP IDs	Chromosomes	Physical positions (bp)	SNPs	NCBI SNPdb accession IDs (NCBI_ss#)
Ca_Kabuli_NC_021160.1_2948213	Ca_kabuli_chr01	2948213	[A/C]	1067289821
Ca_Kabuli_NC_021160.1_2948247	Ca_kabuli_chr01	2948247	[A/G]	1067289822
Ca_Kabuli_NC_021160.1_2948248	Ca_kabuli_chr01	2948248	[A/G]	1067289823
Ca_Kabuli_NC_021160.1_2948249	Ca_kabuli_chr01	2948249	[C/A]	1067289824
Ca_Kabuli_NC_021160.1_2948253	Ca_kabuli_chr01	2948253	[C/T]	1067289825
Ca_Kabuli_NC_021160.1_2948258	Ca_kabuli_chr01	2948258	[C/A]	1067289826
Ca_Kabuli_NC_021160.1_2948260	Ca_kabuli_chr01	2948260	[G/A]	1067289827
Ca_Kabuli_NC_021160.1_2997728	Ca_kabuli_chr01	2997728	[T/G]	1067289828
Ca_Kabuli_NC_021160.1_3006195	Ca_kabuli_chr01	3006195	[T/A]	1067289829
Ca_Kabuli_NC_021160.1_3009393	Ca_kabuli_chr01	3009393	[A/C]	1067289830
Ca_Kabuli_NC_021160.1_3032090	Ca_kabuli_chr01	3032090	[T/G]	1067289831
Ca_Kabuli_NC_021160.1_3034364	Ca_kabuli_chr01	3034364	[A/C]	1067289832
Ca_Kabuli_NC_021160.1_3051969	Ca_kabuli_chr01	3051969	[C/A]	1067289833
Ca_Kabuli_NC_021160.1_3052183	Ca_kabuli_chr01	3052183	[T/A]	1067289834
Ca_Kabuli_NC_021160.1_3052591	Ca_kabuli_chr01	3052591	[A/T]	1067289835
Ca_Kabuli_NC_021160.1_3052594	Ca_kabuli_chr01	3052594	[C/T]	1067289836
Ca_Kabuli_NC_021160.1_3052634	Ca_kabuli_chr01	3052634	[T/G]	1067289837
Ca_Kabuli_NC_021160.1_3052640	Ca_kabuli_chr01	3052640	[A/G]	1067289838
Ca_Kabuli_NC_021160.1_3081902	Ca_kabuli_chr01	3081902	[A/C]	1067289839
Ca_Kabuli_NC_021160.1_3143867	Ca_kabuli_chr01	3143867	[T/G]	1067289840
Ca_Kabuli_NC_021160.1_3154736	Ca_kabuli_chr01	3154736	[C/A]	1067289841
Ca_Kabuli_NC_021160.1_3154739	Ca_kabuli_chr01	3154739	[C/T]	1067289842
Ca_Kabuli_NC_021160.1_3154806	Ca_kabuli_chr01	3154806	[A/C]	1067289843
Ca_Kabuli_NC_021160.1_3230872	Ca_kabuli_chr01	3230872	[A/G]	1067289844
Ca_Kabuli_NC_021160.1_3235705	Ca_kabuli_chr01	3235705	[A/C]	1067289845
Ca_Kabuli_NC_021160.1_3235729	Ca_kabuli_chr01	3235729	[T/A]	1067289846
Ca_Kabuli_NC_021160.1_3260240	Ca_kabuli_chr01	3260240	[G/A]	1067289847
Ca_Kabuli_NC_021160.1_3260243	Ca_kabuli_chr01	3260243	[C/A]	1067289848
Ca_Kabuli_NC_021160.1_3260252	Ca_kabuli_chr01	3260252	[A/G]	1067289849
Ca_Kabuli_NC_021160.1_3260253	Ca_kabuli_chr01	3260253	[A/G]	1067289850
Ca_Kabuli_NC_021160.1_3298109	Ca_kabuli_chr01	3298109	[T/A]	1067289851
Ca_Kabuli_NC_021160.1_3298206	Ca_kabuli_chr01	3298206	[A/C]	1067289852
Ca_Kabuli_NC_021160.1_3298295	Ca_kabuli_chr01	3298295	[T/G]	1067289853
Ca_Kabuli_NC_021160.1_3298317	Ca_kabuli_chr01	3298317	[T/A]	1067289854

SNP IDs	Chromosomes	Physical positions (bp)	SNPs	NCBI SNPdb accession IDs (NCBI_ss#)
Ca_Kabuli_NC_021160.1_3349259	Ca_kabuli_chr01	3349259	[C/G]	1067289855
Ca_Kabuli_NC_021160.1_3349819	Ca_kabuli_chr01	3349819	[T/C]	1067289856
Ca_Kabuli_NC_021160.1_3364890	Ca_kabuli_chr01	3364890	[T/C]	1067289857
Ca_Kabuli_NC_021160.1_3405838	Ca_kabuli_chr01	3405838	[G/T]	1067289858
Ca_Kabuli_NC_021160.1_3481995	Ca_kabuli_chr01	3481995	[T/A]	1067289859
Ca_Kabuli_NC_021160.1_3503132	Ca_kabuli_chr01	3503132	[A/C]	1067289860
Ca_Kabuli_NC_021160.1_3503209	Ca_kabuli_chr01	3503209	[T/C]	1067289861
Ca_Kabuli_NC_021160.1_3503231	Ca_kabuli_chr01	3503231	[T/G]	1067289862
Ca_Kabuli_NC_021160.1_3503244	Ca_kabuli_chr01	3503244	[A/C]	1067289863
Ca_Kabuli_NC_021160.1_3503249	Ca_kabuli_chr01	3503249	[A/T]	1067289864
Ca_Kabuli_NC_021160.1_3544578	Ca_kabuli_chr01	3544578	[T/A]	1067289865
Ca_Kabuli_NC_021160.1_3544912	Ca_kabuli_chr01	3544912	[A/C]	1067289866
Ca_Kabuli_NC_021160.1_3546461	Ca_kabuli_chr01	3546461	[C/T]	1067289867
Ca_Kabuli_NC_021160.1_3546462	Ca_kabuli_chr01	3546462	[G/C]	1067289868
Ca_Kabuli_NC_021160.1_3546463	Ca_kabuli_chr01	3546463	[G/T]	1067289869
Ca_Kabuli_NC_021160.1_3607175	Ca_kabuli_chr01	3607175	[A/G]	1067289870
Ca_Kabuli_NC_021160.1_3611230	Ca_kabuli_chr01	3611230	[A/G]	1067289871
Ca_Kabuli_NC_021160.1_3711498	Ca_kabuli_chr01	3711498	[T/G]	1067289872
Ca_Kabuli_NC_021160.1_3711500	Ca_kabuli_chr01	3711500	[A/G]	1067289873
Ca_Kabuli_NC_021160.1_3711510	Ca_kabuli_chr01	3711510	[T/A]	1067289874
Ca_Kabuli_NC_021160.1_3711516	Ca_kabuli_chr01	3711516	[T/G]	1067289875
Ca_Kabuli_NC_021160.1_3711525	Ca_kabuli_chr01	3711525	[T/G]	1067289876
Ca_Kabuli_NC_021160.1_3711551	Ca_kabuli_chr01	3711551	[A/C]	1067289877
Ca_Kabuli_NC_021160.1_3736950	Ca_kabuli_chr01	3736950	[G/A]	1067289878
Ca_Kabuli_NC_021160.1_3736953	Ca_kabuli_chr01	3736953	[G/A]	1067289879
Ca_Kabuli_NC_021160.1_3736956	Ca_kabuli_chr01	3736956	[T/G]	1067289880
Ca_Kabuli_NC_021160.1_3852265	Ca_kabuli_chr01	3852265	[A/G]	1067289881
Ca_Kabuli_NC_021160.1_3853187	Ca_kabuli_chr01	3853187	[A/C]	1067289882
Ca_Kabuli_NC_021160.1_3853190	Ca_kabuli_chr01	3853190	[A/G]	1067289883
Ca_Kabuli_NC_021160.1_3853392	Ca_kabuli_chr01	3853392	[T/C]	1067289884
Ca_Kabuli_NC_021160.1_3853397	Ca_kabuli_chr01	3853397	[T/G]	1067289885
Ca_Kabuli_NC_021160.1_3853462	Ca_kabuli_chr01	3853462	[A/G]	1067289886
Ca_Kabuli_NC_021160.1_3853547	Ca_kabuli_chr01	3853547	[T/C]	1067289887
Ca_Kabuli_NC_021160.1_3909375	Ca_kabuli_chr01	3909375	[A/G]	1067289888

SNP IDs	Chromosomes	Physical positions (bp)	SNPs	NCBI SNPdb accession IDs (NCBI_ss#)
Ca_Kabuli_NC_021160.1_3911111	Ca_kabuli_chr01	3911111	[T/G]	1067289889
Ca_Kabuli_NC_021160.1_3911134	Ca_kabuli_chr01	3911134	[A/G]	1067289890
Ca_Kabuli_NC_021160.1_3911408	Ca_kabuli_chr01	3911408	[A/G]	1067289891
Ca_Kabuli_NC_021160.1_3927976	Ca_kabuli_chr01	3927976	[A/G]	1067289892
Ca_Kabuli_NC_021160.1_3940980	Ca_kabuli_chr01	3940980	[T/G]	1067289893
Ca_Kabuli_NC_021160.1_3941139	Ca_kabuli_chr01	3941139	[T/A]	1067289894
Ca_Kabuli_NC_021160.1_3941141	Ca_kabuli_chr01	3941141	[T/C]	1067289895
Ca_Kabuli_NC_021160.1_3941142	Ca_kabuli_chr01	3941142	[G/T]	1067289896
Ca_Kabuli_NC_021160.1_3952796	Ca_kabuli_chr01	3952796	[T/G]	1067289897
Ca_Kabuli_NC_021160.1_3952823	Ca_kabuli_chr01	3952823	[T/G]	1067289898
Ca_Kabuli_NC_021160.1_3972668	Ca_kabuli_chr01	3972668	[A/C]	1067289899
Ca_Kabuli_NC_021160.1_4017064	Ca_kabuli_chr01	4017064	[A/T]	1067289900
Ca_Kabuli_NC_021160.1_4017077	Ca_kabuli_chr01	4017077	[T/G]	1067289901
Ca_Kabuli_NC_021160.1_4030790	Ca_kabuli_chr01	4030790	[A/C]	1067289902
Ca_Kabuli_NC_021160.1_4187752	Ca_kabuli_chr01	4187752	[T/G]	1067289903
Ca_Kabuli_NC_021160.1_4187762	Ca_kabuli_chr01	4187762	[A/T]	1067289904
Ca_Kabuli_NC_021160.1_4189333	Ca_kabuli_chr01	4189333	[T/C]	1067289905
Ca_Kabuli_NC_021160.1_4293010	Ca_kabuli_chr01	4293010	[A/C]	1067289906
Ca_Kabuli_NC_021160.1_4302705	Ca_kabuli_chr01	4302705	[C/A]	1067289907
Ca_Kabuli_NC_021160.1_4302706	Ca_kabuli_chr01	4302706	[A/G]	1067289908
Ca_Kabuli_NC_021160.1_4302770	Ca_kabuli_chr01	4302770	[A/C]	1067289909
Ca_Kabuli_NC_021160.1_4320391	Ca_kabuli_chr01	4320391	[A/G]	1067289910
Ca_Kabuli_NC_021160.1_4320404	Ca_kabuli_chr01	4320404	[A/G]	1067289911
Ca_Kabuli_NC_021160.1_4372894	Ca_kabuli_chr01	4372894	[T/C]	1067289912
Ca_Kabuli_NC_021160.1_4375775	Ca_kabuli_chr01	4375775	[A/G]	1067289913
Ca_Kabuli_NC_021160.1_4386125	Ca_kabuli_chr01	4386125	[A/C]	1067289914
Ca_Kabuli_NC_021160.1_4386145	Ca_kabuli_chr01	4386145	[A/T]	1067289915
Ca_Kabuli_NC_021160.1_4386162	Ca_kabuli_chr01	4386162	[A/C]	1067289916
Ca_Kabuli_NC_021160.1_4423934	Ca_kabuli_chr01	4423934	[A/G]	1067289917
Ca_Kabuli_NC_021160.1_4423978	Ca_kabuli_chr01	4423978	[A/G]	1067289918
Ca_Kabuli_NC_021160.1_4423984	Ca_kabuli_chr01	4423984	[A/C]	1067289919
Ca_Kabuli_NC_021160.1_4429013	Ca_kabuli_chr01	4429013	[T/C]	1067289920
Ca_Kabuli_NC_021160.1_4429024	Ca_kabuli_chr01	4429024	[T/C]	1067289921
Ca_Kabuli_NC_021160.1_4456639	Ca_kabuli_chr01	4456639	[T/C]	1067289922

SNP IDs	Chromosomes	Physical positions (bp)	SNPs	NCBI SNPdb accession IDs (NCBI_ss#)
Ca_Kabuli_NC_021160.1_4456648	Ca_kabuli_chr01	4456648	[T/A]	1067289923
Ca_Kabuli_NC_021160.1_4466616	Ca_kabuli_chr01	4466616	[T/G]	1067289924
Ca_Kabuli_NC_021160.1_4569479	Ca_kabuli_chr01	4569479	[A/C]	1067289925
Ca_Kabuli_NC_021160.1_4569555	Ca_kabuli_chr01	4569555	[T/G]	1067289926
Ca_Kabuli_NC_021160.1_4569576	Ca_kabuli_chr01	4569576	[T/G]	1067289927
Ca_Kabuli_NC_021160.1_4569615	Ca_kabuli_chr01	4569615	[T/A]	1067289928
Ca_Kabuli_NC_021160.1_4569653	Ca_kabuli_chr01	4569653	[T/C]	1067289929
Ca_Kabuli_NC_021160.1_4580855	Ca_kabuli_chr01	4580855	[T/C]	1067289930
Ca_Kabuli_NC_021160.1_4580881	Ca_kabuli_chr01	4580881	[A/G]	1067289931
Ca_Kabuli_NC_021160.1_4626197	Ca_kabuli_chr01	4626197	[A/C]	1067289932
Ca_Kabuli_NC_021160.1_4631461	Ca_kabuli_chr01	4631461	[A/C]	1067289933
Ca_Kabuli_NC_021160.1_4737620	Ca_kabuli_chr01	4737620	[G/C]	1067289934
Ca_Kabuli_NC_021160.1_4737621	Ca_kabuli_chr01	4737621	[G/T]	1067289935
Ca_Kabuli_NC_021160.1_4781690	Ca_kabuli_chr01	4781690	[A/C]	1067289936
Ca_Kabuli_NC_021160.1_4781714	Ca_kabuli_chr01	4781714	[A/T]	1067289937
Ca_Kabuli_NC_021160.1_4827194	Ca_kabuli_chr01	4827194	[A/C]	1067289938
Ca_Kabuli_NC_021160.1_4827200	Ca_kabuli_chr01	4827200	[A/C]	1067289939
Ca_Kabuli_NC_021160.1_4827202	Ca_kabuli_chr01	4827202	[T/C]	1067289940
Ca_Kabuli_NC_021160.1_4899835	Ca_kabuli_chr01	4899835	[T/G]	1067289941
Ca_Kabuli_NC_021160.1_4899882	Ca_kabuli_chr01	4899882	[A/C]	1067289942
Ca_Kabuli_NC_021160.1_4902036	Ca_kabuli_chr01	4902036	[C/T]	1067289943
Ca_Kabuli_NC_021160.1_4906830	Ca_kabuli_chr01	4906830	[T/C]	1067289944
Ca_Kabuli_NC_021160.1_4906831	Ca_kabuli_chr01	4906831	[G/T]	1067289945
Ca_Kabuli_NC_021160.1_4908732	Ca_kabuli_chr01	4908732	[A/T]	1067289946
Ca_Kabuli_NC_021160.1_4908733	Ca_kabuli_chr01	4908733	[C/A]	1067289947
Ca_Kabuli_NC_021160.1_4948887	Ca_kabuli_chr01	4948887	[T/G]	1067289948
Ca_Kabuli_NC_021160.1_4950486	Ca_kabuli_chr01	4950486	[A/G]	1067289949
Ca_Kabuli_NC_021160.1_4950743	Ca_kabuli_chr01	4950743	[T/G]	1067289950
Ca_Kabuli_NC_021160.1_4996269	Ca_kabuli_chr01	4996269	[A/C]	1067289951
Ca_Kabuli_NC_021160.1_4996279	Ca_kabuli_chr01	4996279	[G/C]	1067289952
Ca_Kabuli_NC_021160.1_4996282	Ca_kabuli_chr01	4996282	[A/T]	1067289953
Ca_Kabuli_NC_021160.1_4996302	Ca_kabuli_chr01	4996302	[T/C]	1067289954
Ca_Kabuli_NC_021160.1_4996818	Ca_kabuli_chr01	4996818	[T/G]	1067289955
Ca_Kabuli_NC_021160.1_4996834	Ca_kabuli_chr01	4996834	[A/G]	1067289956

SNP IDs	Chromosomes	Physical positions (bp)	SNPs	NCBI SNPdb accession IDs (NCBI_ss#)
Ca_Kabuli_NC_021160.1_5068304	Ca_kabuli_chr01	5068304	[T/C]	1067289957
Ca_Kabuli_NC_021160.1_5068311	Ca_kabuli_chr01	5068311	[T/C]	1067289958
Ca_Kabuli_NC_021160.1_5068341	Ca_kabuli_chr01	5068341	[A/C]	1067289959
Ca_Kabuli_NC_021160.1_5071992	Ca_kabuli_chr01	5071992	[T/A]	1067289960
Ca_Kabuli_NC_021160.1_5087503	Ca_kabuli_chr01	5087503	[T/G]	1067289961
Ca_Kabuli_NC_021160.1_5094543	Ca_kabuli_chr01	5094543	[A/C]	1067289962
Ca_Kabuli_NC_021160.1_5094544	Ca_kabuli_chr01	5094544	[G/T]	1067289963
Ca_Kabuli_NC_021160.1_5107995	Ca_kabuli_chr01	5107995	[A/C]	1067289964
Ca_Kabuli_NC_021160.1_5108029	Ca_kabuli_chr01	5108029	[A/G]	1067289965
Ca_Kabuli_NC_021160.1_5164105	Ca_kabuli_chr01	5164105	[T/G]	1067289966
Ca_Kabuli_NC_021160.1_5164186	Ca_kabuli_chr01	5164186	[G/A]	1067289967
Ca_Kabuli_NC_021160.1_5164198	Ca_kabuli_chr01	5164198	[C/A]	1067289968
Ca_Kabuli_NC_021160.1_5249782	Ca_kabuli_chr01	5249782	[T/C]	1067289969
Ca_Kabuli_NC_021160.1_5249783	Ca_kabuli_chr01	5249783	[C/T]	1067289970
Ca_Kabuli_NC_021160.1_5249784	Ca_kabuli_chr01	5249784	[T/C]	1067289971
Ca_Kabuli_NC_021160.1_5249798	Ca_kabuli_chr01	5249798	[T/C]	1067289972
Ca_Kabuli_NC_021160.1_5249808	Ca_kabuli_chr01	5249808	[T/C]	1067289973
Ca_Kabuli_NC_021160.1_5249822	Ca_kabuli_chr01	5249822	[T/A]	1067289974
Ca_Kabuli_NC_021160.1_5249868	Ca_kabuli_chr01	5249868	[G/A]	1067289975
Ca_Kabuli_NC_021160.1_5288463	Ca_kabuli_chr01	5288463	[A/C]	1067289976
Ca_Kabuli_NC_021160.1_5288475	Ca_kabuli_chr01	5288475	[A/C]	1067289977
Ca_Kabuli_NC_021160.1_5288478	Ca_kabuli_chr01	5288478	[A/C]	1067289978
Ca_Kabuli_NC_021160.1_5363160	Ca_kabuli_chr01	5363160	[A/C]	1067289979
Ca_Kabuli_NC_021160.1_5430636	Ca_kabuli_chr01	5430636	[T/C]	1067289980
Ca_Kabuli_NC_021160.1_5430640	Ca_kabuli_chr01	5430640	[A/T]	1067289981
Ca_Kabuli_NC_021160.1_5430645	Ca_kabuli_chr01	5430645	[A/C]	1067289982
Ca_Kabuli_NC_021160.1_5430652	Ca_kabuli_chr01	5430652	[T/C]	1067289983
Ca_Kabuli_NC_021160.1_5430676	Ca_kabuli_chr01	5430676	[T/A]	1067289984
Ca_Kabuli_NC_021160.1_5487600	Ca_kabuli_chr01	5487600	[A/C]	1067289985
Ca_Kabuli_NC_021160.1_5489570	Ca_kabuli_chr01	5489570	[T/G]	1067289986
Ca_Kabuli_NC_021160.1_5498288	Ca_kabuli_chr01	5498288	[T/G]	1067289987
Ca_Kabuli_NC_021160.1_5498291	Ca_kabuli_chr01	5498291	[T/G]	1067289988
Ca_Kabuli_NC_021160.1_5567072	Ca_kabuli_chr01	5567072	[C/T]	1067289989
Ca_Kabuli_NC_021160.1_5576985	Ca_kabuli_chr01	5576985	[A/G]	1067289990

SNP IDs	Chromosomes	Physical positions (bp)	SNPs	NCBI SNPdb accession IDs (NCBI_ss#)
Ca_Kabuli_NC_021160.1_5666149	Ca_kabuli_chr01	5666149	[A/C]	1067289991
Ca_Kabuli_NC_021160.1_5666152	Ca_kabuli_chr01	5666152	[A/G]	1067289992
Ca_Kabuli_NC_021160.1_5666237	Ca_kabuli_chr01	5666237	[T/G]	1067289993
Ca_Kabuli_NC_021160.1_5666260	Ca_kabuli_chr01	5666260	[T/G]	1067289994
Ca_Kabuli_NC_021160.1_5666263	Ca_kabuli_chr01	5666263	[T/G]	1067289995
Ca_Kabuli_NC_021160.1_5749429	Ca_kabuli_chr01	5749429	[A/G]	1067289996
Ca_Kabuli_NC_021160.1_5749438	Ca_kabuli_chr01	5749438	[T/G]	1067289997
Ca_Kabuli_NC_021160.1_5749452	Ca_kabuli_chr01	5749452	[C/T]	1067289998
Ca_Kabuli_NC_021160.1_5793815	Ca_kabuli_chr01	5793815	[G/A]	1067289999
Ca_Kabuli_NC_021160.1_5862586	Ca_kabuli_chr01	5862586	[T/C]	1067290000
Ca_Kabuli_NC_021160.1_5933308	Ca_kabuli_chr01	5933308	[T/G]	1067290001
Ca_Kabuli_NC_021160.1_5978264	Ca_kabuli_chr01	5978264	[A/C]	1067290002
Ca_Kabuli_NC_021160.1_5978287	Ca_kabuli_chr01	5978287	[C/T]	1067290003
Ca_Kabuli_NC_021160.1_6011790	Ca_kabuli_chr01	6011790	[A/G]	1067290004
Ca_Kabuli_NC_021160.1_6088245	Ca_kabuli_chr01	6088245	[T/C]	1067290005
Ca_Kabuli_NC_021160.1_6097569	Ca_kabuli_chr01	6097569	[G/A]	1067290006
Ca_Kabuli_NC_021160.1_6097599	Ca_kabuli_chr01	6097599	[A/C]	1067290007
Ca_Kabuli_NC_021160.1_6224010	Ca_kabuli_chr01	6224010	[G/C]	1067290008
Ca_Kabuli_NC_021160.1_6224015	Ca_kabuli_chr01	6224015	[T/C]	1067290009
Ca_Kabuli_NC_021160.1_6257582	Ca_kabuli_chr01	6257582	[T/C]	1067290010
Ca_Kabuli_NC_021160.1_6257712	Ca_kabuli_chr01	6257712	[T/A]	1067290011
Ca_Kabuli_NC_021160.1_6259026	Ca_kabuli_chr01	6259026	[C/T]	1067290012
Ca_Kabuli_NC_021160.1_6259419	Ca_kabuli_chr01	6259419	[G/T]	1067290013
Ca_Kabuli_NC_021160.1_6259466	Ca_kabuli_chr01	6259466	[T/A]	1067290014
Ca_Kabuli_NC_021160.1_6268630	Ca_kabuli_chr01	6268630	[A/G]	1067290015
Ca_Kabuli_NC_021160.1_6268776	Ca_kabuli_chr01	6268776	[A/C]	1067290016
Ca_Kabuli_NC_021160.1_6268787	Ca_kabuli_chr01	6268787	[G/A]	1067290017
Ca_Kabuli_NC_021160.1_6279597	Ca_kabuli_chr01	6279597	[A/C]	1067290018
Ca_Kabuli_NC_021160.1_6296986	Ca_kabuli_chr01	6296986	[A/C]	1067290019
Ca_Kabuli_NC_021160.1_6304919	Ca_kabuli_chr01	6304919	[C/A]	1067290020
Ca_Kabuli_NC_021160.1_6315397	Ca_kabuli_chr01	6315397	[G/C]	1067290021
Ca_Kabuli_NC_021160.1_6315399	Ca_kabuli_chr01	6315399	[G/T]	1067290022
Ca_Kabuli_NC_021160.1_6329022	Ca_kabuli_chr01	6329022	[T/C]	1067290023
Ca_Kabuli_NC_021160.1_6329054	Ca_kabuli_chr01	6329054	[A/G]	1067290024

SNP IDs	Chromosomes	Physical positions (bp)	SNPs	NCBI SNPdb accession IDs (NCBI_ss#)
Ca_Kabuli_NC_021160.1_6329089	Ca_kabuli_chr01	6329089	[A/G]	1067290025
Ca_Kabuli_NC_021160.1_6329096	Ca_kabuli_chr01	6329096	[C/T]	1067290026
Ca_Kabuli_NC_021160.1_6329121	Ca_kabuli_chr01	6329121	[G/A]	1067290027
Ca_Kabuli_NC_021160.1_6329786	Ca_kabuli_chr01	6329786	[T/G]	1067290028
Ca_Kabuli_NC_021160.1_6329847	Ca_kabuli_chr01	6329847	[G/A]	1067290029
Ca_Kabuli_NC_021160.1_6331686	Ca_kabuli_chr01	6331686	[A/G]	1067290030
Ca_Kabuli_NC_021160.1_6332104	Ca_kabuli_chr01	6332104	[G/A]	1067290031
Ca_Kabuli_NC_021160.1_6332153	Ca_kabuli_chr01	6332153	[T/C]	1067290032
Ca_Kabuli_NC_021160.1_6390541	Ca_kabuli_chr01	6390541	[T/A]	1067290033
Ca_Kabuli_NC_021160.1_6391016	Ca_kabuli_chr01	6391016	[A/G]	1067290034
Ca_Kabuli_NC_021160.1_6433592	Ca_kabuli_chr01	6433592	[G/T]	1067290035
Ca_Kabuli_NC_021160.1_6433838	Ca_kabuli_chr01	6433838	[C/T]	1067290036
Ca_Kabuli_NC_021160.1_6433919	Ca_kabuli_chr01	6433919	[A/C]	1067290037
Ca_Kabuli_NC_021160.1_6433934	Ca_kabuli_chr01	6433934	[T/A]	1067290038
Ca_Kabuli_NC_021160.1_6435319	Ca_kabuli_chr01	6435319	[T/C]	1067290039
Ca_Kabuli_NC_021160.1_6435331	Ca_kabuli_chr01	6435331	[T/C]	1067290040
Ca_Kabuli_NC_021160.1_6435499	Ca_kabuli_chr01	6435499	[C/T]	1067290041
Ca_Kabuli_NC_021160.1_6437643	Ca_kabuli_chr01	6437643	[A/G]	1067290042
Ca_Kabuli_NC_021160.1_6478765	Ca_kabuli_chr01	6478765	[G/A]	1067290043
Ca_Kabuli_NC_021160.1_6478794	Ca_kabuli_chr01	6478794	[G/A]	1067290044
Ca_Kabuli_NC_021160.1_6520998	Ca_kabuli_chr01	6520998	[T/C]	1067290045
Ca_Kabuli_NC_021160.1_6591483	Ca_kabuli_chr01	6591483	[T/G]	1067290046
Ca_Kabuli_NC_021160.1_6591493	Ca_kabuli_chr01	6591493	[A/T]	1067290047
Ca_Kabuli_NC_021160.1_6591527	Ca_kabuli_chr01	6591527	[T/G]	1067290048
Ca_Kabuli_NC_021160.1_6621746	Ca_kabuli_chr01	6621746	[T/C]	1067290049
Ca_Kabuli_NC_021160.1_6621752	Ca_kabuli_chr01	6621752	[T/G]	1067290050
Ca_Kabuli_NC_021160.1_6621759	Ca_kabuli_chr01	6621759	[G/A]	1067290051
Ca_Kabuli_NC_021160.1_6635359	Ca_kabuli_chr01	6635359	[A/C]	1067290052
Ca_Kabuli_NC_021160.1_6635791	Ca_kabuli_chr01	6635791	[A/G]	1067290053
Ca_Kabuli_NC_021160.1_6635809	Ca_kabuli_chr01	6635809	[A/G]	1067290054
Ca_Kabuli_NC_021160.1_6678449	Ca_kabuli_chr01	6678449	[T/G]	1067290055
Ca_Kabuli_NC_021160.1_6678554	Ca_kabuli_chr01	6678554	[T/G]	1067290056
Ca_Kabuli_NC_021160.1_6678612	Ca_kabuli_chr01	6678612	[A/G]	1067290057
Ca_Kabuli_NC_021160.1_6734885	Ca_kabuli_chr01	6734885	[A/G]	1067290058

SNP IDs	Chromosomes	Physical positions (bp)	SNPs	NCBI SNPdb accession IDs (NCBI_ss#)
Ca_Kabuli_NC_021160.1_6761335	Ca_kabuli_chr01	6761335	[C/A]	1067290059
Ca_Kabuli_NC_021160.1_6803365	Ca_kabuli_chr01	6803365	[A/G]	1067290060
Ca_Kabuli_NC_021160.1_6803370	Ca_kabuli_chr01	6803370	[T/C]	1067290061
Ca_Kabuli_NC_021160.1_6848555	Ca_kabuli_chr01	6848555	[A/C]	1067290062
Ca_Kabuli_NC_021160.1_6879106	Ca_kabuli_chr01	6879106	[T/C]	1067290063
Ca_Kabuli_NC_021160.1_6879157	Ca_kabuli_chr01	6879157	[A/C]	1067290064
Ca_Kabuli_NC_021160.1_6880831	Ca_kabuli_chr01	6880831	[A/C]	1067290065
Ca_Kabuli_NC_021160.1_6880849	Ca_kabuli_chr01	6880849	[A/G]	1067290066
Ca_Kabuli_NC_021160.1_6880903	Ca_kabuli_chr01	6880903	[A/T]	1067290067
Ca_Kabuli_NC_021160.1_6881779	Ca_kabuli_chr01	6881779	[A/G]	1067290068
Ca_Kabuli_NC_021160.1_6911785	Ca_kabuli_chr01	6911785	[T/C]	1067290069
Ca_Kabuli_NC_021160.1_6952381	Ca_kabuli_chr01	6952381	[T/G]	1067290070
Ca_Kabuli_NC_021160.1_7066766	Ca_kabuli_chr01	7066766	[A/G]	1067290071
Ca_Kabuli_NC_021160.1_7066768	Ca_kabuli_chr01	7066768	[A/C]	1067290072
Ca_Kabuli_NC_021160.1_7066781	Ca_kabuli_chr01	7066781	[A/G]	1067290073
Ca_Kabuli_NC_021160.1_7066793	Ca_kabuli_chr01	7066793	[A/C]	1067290074
Ca_Kabuli_NC_021160.1_7084295	Ca_kabuli_chr01	7084295	[T/C]	1067290075
Ca_Kabuli_NC_021160.1_7084306	Ca_kabuli_chr01	7084306	[A/C]	1067290076
Ca_Kabuli_NC_021160.1_7111622	Ca_kabuli_chr01	7111622	[A/G]	1067290077
Ca_Kabuli_NC_021160.1_7160640	Ca_kabuli_chr01	7160640	[T/G]	1067290078
Ca_Kabuli_NC_021160.1_7160645	Ca_kabuli_chr01	7160645	[T/C]	1067290079
Ca_Kabuli_NC_021160.1_7160664	Ca_kabuli_chr01	7160664	[A/G]	1067290080
Ca_Kabuli_NC_021160.1_7183663	Ca_kabuli_chr01	7183663	[A/G]	1067290081
Ca_Kabuli_NC_021160.1_7231728	Ca_kabuli_chr01	7231728	[T/C]	1067290082
Ca_Kabuli_NC_021160.1_7327934	Ca_kabuli_chr01	7327934	[G/T]	1067290083
Ca_Kabuli_NC_021160.1_7330067	Ca_kabuli_chr01	7330067	[A/G]	1067290084
Ca_Kabuli_NC_021160.1_7439391	Ca_kabuli_chr01	7439391	[A/C]	1067290085
Ca_Kabuli_NC_021160.1_7440335	Ca_kabuli_chr01	7440335	[T/C]	1067290086
Ca_Kabuli_NC_021160.1_7440343	Ca_kabuli_chr01	7440343	[A/G]	1067290087
Ca_Kabuli_NC_021160.1_7440354	Ca_kabuli_chr01	7440354	[A/G]	1067290088
Ca_Kabuli_NC_021160.1_7486081	Ca_kabuli_chr01	7486081	[T/G]	1067290089
Ca_Kabuli_NC_021160.1_7486104	Ca_kabuli_chr01	7486104	[A/C]	1067290090
Ca_Kabuli_NC_021160.1_7486109	Ca_kabuli_chr01	7486109	[T/G]	1067290091
Ca_Kabuli_NC_021160.1_7486195	Ca_kabuli_chr01	7486195	[T/C]	1067290092

SNP IDs	Chromosomes	Physical positions (bp)	SNPs	NCBI SNPdb accession IDs (NCBI_ss#)
Ca_Kabuli_NC_021160.1_7486199	Ca_kabuli_chr01	7486199	[A/G]	1067290093
Ca_Kabuli_NC_021160.1_7486207	Ca_kabuli_chr01	7486207	[A/G]	1067290094
Ca_Kabuli_NC_021160.1_7486222	Ca_kabuli_chr01	7486222	[T/G]	1067290095
Ca_Kabuli_NC_021160.1_7500494	Ca_kabuli_chr01	7500494	[T/G]	1067290096
Ca_Kabuli_NC_021160.1_7500497	Ca_kabuli_chr01	7500497	[T/G]	1067290097
Ca_Kabuli_NC_021160.1_7500514	Ca_kabuli_chr01	7500514	[C/A]	1067290098
Ca_Kabuli_NC_021160.1_7500515	Ca_kabuli_chr01	7500515	[C/G]	1067290099
Ca_Kabuli_NC_021160.1_7500516	Ca_kabuli_chr01	7500516	[G/A]	1067290100
Ca_Kabuli_NC_021160.1_7500627	Ca_kabuli_chr01	7500627	[T/C]	1067290101
Ca_Kabuli_NC_021160.1_7504924	Ca_kabuli_chr01	7504924	[T/A]	1067290102
Ca_Kabuli_NC_021160.1_7509839	Ca_kabuli_chr01	7509839	[A/T]	1067290103
Ca_Kabuli_NC_021160.1_7554807	Ca_kabuli_chr01	7554807	[A/C]	1067290104
Ca_Kabuli_NC_021160.1_7554818	Ca_kabuli_chr01	7554818	[A/G]	1067290105
Ca_Kabuli_NC_021160.1_7554828	Ca_kabuli_chr01	7554828	[A/G]	1067290106
Ca_Kabuli_NC_021160.1_7565585	Ca_kabuli_chr01	7565585	[C/G]	1067290107
Ca_Kabuli_NC_021160.1_7565676	Ca_kabuli_chr01	7565676	[T/G]	1067290108
Ca_Kabuli_NC_021160.1_7604966	Ca_kabuli_chr01	7604966	[A/C]	1067290109
Ca_Kabuli_NC_021160.1_7678588	Ca_kabuli_chr01	7678588	[G/A]	1067290110
Ca_Kabuli_NC_021160.1_7679328	Ca_kabuli_chr01	7679328	[A/G]	1067290111
Ca_Kabuli_NC_021160.1_7690613	Ca_kabuli_chr01	7690613	[T/G]	1067290112
Ca_Kabuli_NC_021160.1_7690620	Ca_kabuli_chr01	7690620	[T/G]	1067290113
Ca_Kabuli_NC_021160.1_7811388	Ca_kabuli_chr01	7811388	[C/T]	1067290114
Ca_Kabuli_NC_021160.1_7811444	Ca_kabuli_chr01	7811444	[G/T]	1067290115
Ca_Kabuli_NC_021160.1_7897505	Ca_kabuli_chr01	7897505	[T/G]	1067290116
Ca_Kabuli_NC_021160.1_7897545	Ca_kabuli_chr01	7897545	[A/G]	1067290117
Ca_Kabuli_NC_021160.1_7915141	Ca_kabuli_chr01	7915141	[G/A]	1067290118
Ca_Kabuli_NC_021160.1_7916143	Ca_kabuli_chr01	7916143	[T/G]	1067290119
Ca_Kabuli_NC_021160.1_7916152	Ca_kabuli_chr01	7916152	[G/A]	1067290120
Ca_Kabuli_NC_021160.1_7916540	Ca_kabuli_chr01	7916540	[A/T]	1067290121
Ca_Kabuli_NC_021160.1_7916564	Ca_kabuli_chr01	7916564	[A/G]	1067290122
Ca_Kabuli_NC_021160.1_7916572	Ca_kabuli_chr01	7916572	[T/C]	1067290123
Ca_Kabuli_NC_021160.1_7916583	Ca_kabuli_chr01	7916583	[T/G]	1067290124
Ca_Kabuli_NC_021160.1_7916636	Ca_kabuli_chr01	7916636	[T/C]	1067290125
Ca_Kabuli_NC_021160.1_7925666	Ca_kabuli_chr01	7925666	[A/C]	1067290126

SNP IDs	Chromosomes	Physical positions (bp)	SNPs	NCBI SNPdb accession IDs (NCBI_ss#)
Ca_Kabuli_NC_021160.1_7925774	Ca_kabuli_chr01	7925774	[C/T]	1067290127
Ca_Kabuli_NC_021160.1_7925780	Ca_kabuli_chr01	7925780	[A/C]	1067290128
Ca_Kabuli_NC_021160.1_7934892	Ca_kabuli_chr01	7934892	[G/T]	1067290129
Ca_Kabuli_NC_021160.1_7935389	Ca_kabuli_chr01	7935389	[A/T]	1067290130
Ca_Kabuli_NC_021160.1_7935401	Ca_kabuli_chr01	7935401	[T/G]	1067290131
Ca_Kabuli_NC_021160.1_7969141	Ca_kabuli_chr01	7969141	[A/C]	1067290132
Ca_Kabuli_NC_021160.1_7969189	Ca_kabuli_chr01	7969189	[T/G]	1067290133
Ca_Kabuli_NC_021160.1_7969218	Ca_kabuli_chr01	7969218	[T/C]	1067290134
Ca_Kabuli_NC_021160.1_7971308	Ca_kabuli_chr01	7971308	[G/A]	1067290135
Ca_Kabuli_NC_021160.1_8036666	Ca_kabuli_chr01	8036666	[C/T]	1067290136
Ca_Kabuli_NC_021160.1_8036903	Ca_kabuli_chr01	8036903	[A/T]	1067290137
Ca_Kabuli_NC_021160.1_8036917	Ca_kabuli_chr01	8036917	[T/G]	1067290138
Ca_Kabuli_NC_021160.1_8045800	Ca_kabuli_chr01	8045800	[A/C]	1067290139
Ca_Kabuli_NC_021160.1_8045827	Ca_kabuli_chr01	8045827	[T/G]	1067290140
Ca_Kabuli_NC_021160.1_8072470	Ca_kabuli_chr01	8072470	[T/G]	1067290141
Ca_Kabuli_NC_021160.1_8075742	Ca_kabuli_chr01	8075742	[G/T]	1067290142
Ca_Kabuli_NC_021160.1_8100906	Ca_kabuli_chr01	8100906	[C/T]	1067290143
Ca_Kabuli_NC_021160.1_8108014	Ca_kabuli_chr01	8108014	[C/T]	1067290144
Ca_Kabuli_NC_021160.1_8108207	Ca_kabuli_chr01	8108207	[T/A]	1067290145
Ca_Kabuli_NC_021160.1_8108211	Ca_kabuli_chr01	8108211	[A/C]	1067290146
Ca_Kabuli_NC_021160.1_8108226	Ca_kabuli_chr01	8108226	[A/G]	1067290147
Ca_Kabuli_NC_021160.1_8108233	Ca_kabuli_chr01	8108233	[T/G]	1067290148
Ca_Kabuli_NC_021160.1_8112247	Ca_kabuli_chr01	8112247	[A/G]	1067290149
Ca_Kabuli_NC_021160.1_8112280	Ca_kabuli_chr01	8112280	[T/A]	1067290150
Ca_Kabuli_NC_021160.1_8112294	Ca_kabuli_chr01	8112294	[T/G]	1067290151
Ca_Kabuli_NC_021160.1_8112343	Ca_kabuli_chr01	8112343	[C/T]	1067290152
Ca_Kabuli_NC_021160.1_8113173	Ca_kabuli_chr01	8113173	[T/C]	1067290153
Ca_Kabuli_NC_021160.1_8113179	Ca_kabuli_chr01	8113179	[T/A]	1067290154
Ca_Kabuli_NC_021160.1_8113260	Ca_kabuli_chr01	8113260	[A/G]	1067290155
Ca_Kabuli_NC_021160.1_8113398	Ca_kabuli_chr01	8113398	[C/G]	1067290156
Ca_Kabuli_NC_021160.1_8113403	Ca_kabuli_chr01	8113403	[A/T]	1067290157
Ca_Kabuli_NC_021160.1_8113449	Ca_kabuli_chr01	8113449	[T/A]	1067290158
Ca_Kabuli_NC_021160.1_8113502	Ca_kabuli_chr01	8113502	[T/G]	1067290159
Ca_Kabuli_NC_021160.1_8131096	Ca_kabuli_chr01	8131096	[A/C]	1067290160

SNP IDs	Chromosomes	Physical positions (bp)	SNPs	NCBI SNPdb accession IDs (NCBI_ss#)
Ca_Kabuli_NC_021160.1_8131157	Ca_kabuli_chr01	8131157	[T/G]	1067290161
Ca_Kabuli_NC_021160.1_8133516	Ca_kabuli_chr01	8133516	[G/A]	1067290162
Ca_Kabuli_NC_021160.1_8231238	Ca_kabuli_chr01	8231238	[A/C]	1067290163
Ca_Kabuli_NC_021160.1_8238567	Ca_kabuli_chr01	8238567	[A/G]	1067290164
Ca_Kabuli_NC_021160.1_8238575	Ca_kabuli_chr01	8238575	[A/G]	1067290165
Ca_Kabuli_NC_021160.1_8238577	Ca_kabuli_chr01	8238577	[G/C]	1067290166
Ca_Kabuli_NC_021160.1_8238584	Ca_kabuli_chr01	8238584	[A/T]	1067290167
Ca_Kabuli_NC_021160.1_8238598	Ca_kabuli_chr01	8238598	[T/C]	1067290168
Ca_Kabuli_NC_021160.1_8238621	Ca_kabuli_chr01	8238621	[T/C]	1067290169
Ca_Kabuli_NC_021160.1_8389189	Ca_kabuli_chr01	8389189	[C/A]	1067290170
Ca_Kabuli_NC_021160.1_8389216	Ca_kabuli_chr01	8389216	[T/C]	1067290171
Ca_Kabuli_NC_021160.1_8389256	Ca_kabuli_chr01	8389256	[A/C]	1067290172
Ca_Kabuli_NC_021160.1_8465699	Ca_kabuli_chr01	8465699	[A/G]	1067290173
Ca_Kabuli_NC_021160.1_8465711	Ca_kabuli_chr01	8465711	[T/C]	1067290174
Ca_Kabuli_NC_021160.1_8542017	Ca_kabuli_chr01	8542017	[T/A]	1067290175
Ca_Kabuli_NC_021160.1_8669467	Ca_kabuli_chr01	8669467	[C/T]	1067290176
Ca_Kabuli_NC_021160.1_8677266	Ca_kabuli_chr01	8677266	[A/T]	1067290177
Ca_Kabuli_NC_021160.1_8682168	Ca_kabuli_chr01	8682168	[T/C]	1067290178
Ca_Kabuli_NC_021160.1_8682190	Ca_kabuli_chr01	8682190	[T/G]	1067290179
Ca_Kabuli_NC_021160.1_8692680	Ca_kabuli_chr01	8692680	[T/G]	1067290180
Ca_Kabuli_NC_021160.1_8697279	Ca_kabuli_chr01	8697279	[G/T]	1067290181
Ca_Kabuli_NC_021160.1_8737370	Ca_kabuli_chr01	8737370	[A/G]	1067290182
Ca_Kabuli_NC_021160.1_8738746	Ca_kabuli_chr01	8738746	[T/G]	1067290183
Ca_Kabuli_NC_021160.1_8738752	Ca_kabuli_chr01	8738752	[A/G]	1067290184
Ca_Kabuli_NC_021160.1_8776194	Ca_kabuli_chr01	8776194	[A/C]	1067290185
Ca_Kabuli_NC_021160.1_8807347	Ca_kabuli_chr01	8807347	[A/T]	1067290186
Ca_Kabuli_NC_021160.1_8822309	Ca_kabuli_chr01	8822309	[T/G]	1067290187
Ca_Kabuli_NC_021160.1_8822364	Ca_kabuli_chr01	8822364	[A/T]	1067290188
Ca_Kabuli_NC_021160.1_8822383	Ca_kabuli_chr01	8822383	[T/C]	1067290189
Ca_Kabuli_NC_021160.1_8824843	Ca_kabuli_chr01	8824843	[A/G]	1067290190
Ca_Kabuli_NC_021160.1_8871047	Ca_kabuli_chr01	8871047	[T/C]	1067290191
Ca_Kabuli_NC_021160.1_8871080	Ca_kabuli_chr01	8871080	[T/C]	1067290192
Ca_Kabuli_NC_021160.1_8871092	Ca_kabuli_chr01	8871092	[T/C]	1067290193
Ca_Kabuli_NC_021160.1_8891438	Ca_kabuli_chr01	8891438	[T/G]	1067290194

SNP IDs	Chromosomes	Physical positions (bp)	SNPs	NCBI SNPdb accession IDs (NCBI_ss#)
Ca_Kabuli_NC_021160.1_8891458	Ca_kabuli_chr01	8891458	[T/G]	1067290195
Ca_Kabuli_NC_021160.1_8891473	Ca_kabuli_chr01	8891473	[A/T]	1067290196
Ca_Kabuli_NC_021160.1_8891485	Ca_kabuli_chr01	8891485	[T/G]	1067290197
Ca_Kabuli_NC_021160.1_8894567	Ca_kabuli_chr01	8894567	[A/T]	1067290198
Ca_Kabuli_NC_021160.1_8894568	Ca_kabuli_chr01	8894568	[T/C]	1067290199
Ca_Kabuli_NC_021160.1_8894569	Ca_kabuli_chr01	8894569	[G/T]	1067290200
Ca_Kabuli_NC_021160.1_8897947	Ca_kabuli_chr01	8897947	[A/T]	1067290201
Ca_Kabuli_NC_021160.1_8897963	Ca_kabuli_chr01	8897963	[T/C]	1067290202
Ca_Kabuli_NC_021160.1_8901692	Ca_kabuli_chr01	8901692	[A/C]	1067290203
Ca_Kabuli_NC_021160.1_8941054	Ca_kabuli_chr01	8941054	[A/G]	1067290204
Ca_Kabuli_NC_021160.1_8942144	Ca_kabuli_chr01	8942144	[A/C]	1067290205
Ca_Kabuli_NC_021160.1_8949732	Ca_kabuli_chr01	8949732	[T/A]	1067290206
Ca_Kabuli_NC_021160.1_8949771	Ca_kabuli_chr01	8949771	[A/T]	1067290207
Ca_Kabuli_NC_021160.1_8971649	Ca_kabuli_chr01	8971649	[A/G]	1067290208
Ca_Kabuli_NC_021160.1_8971684	Ca_kabuli_chr01	8971684	[A/C]	1067290209
Ca_Kabuli_NC_021160.1_8971710	Ca_kabuli_chr01	8971710	[A/C]	1067290210
Ca_Kabuli_NC_021160.1_8971786	Ca_kabuli_chr01	8971786	[A/G]	1067290211
Ca_Kabuli_NC_021160.1_8971801	Ca_kabuli_chr01	8971801	[A/C]	1067290212
Ca_Kabuli_NC_021160.1_8972032	Ca_kabuli_chr01	8972032	[C/G]	1067290213
Ca_Kabuli_NC_021160.1_8976243	Ca_kabuli_chr01	8976243	[A/C]	1067290214
Ca_Kabuli_NC_021160.1_8980198	Ca_kabuli_chr01	8980198	[A/C]	1067290215
Ca_Kabuli_NC_021160.1_9007413	Ca_kabuli_chr01	9007413	[A/C]	1067290216
Ca_Kabuli_NC_021160.1_9031488	Ca_kabuli_chr01	9031488	[T/A]	1067290217
Ca_Kabuli_NC_021160.1_9031497	Ca_kabuli_chr01	9031497	[G/A]	1067290218
Ca_Kabuli_NC_021160.1_9032161	Ca_kabuli_chr01	9032161	[T/C]	1067290219
Ca_Kabuli_NC_021160.1_9032196	Ca_kabuli_chr01	9032196	[T/G]	1067290220
Ca_Kabuli_NC_021160.1_9064167	Ca_kabuli_chr01	9064167	[T/C]	1067290221
Ca_Kabuli_NC_021160.1_9064170	Ca_kabuli_chr01	9064170	[T/C]	1067290222
Ca_Kabuli_NC_021160.1_9064225	Ca_kabuli_chr01	9064225	[A/G]	1067290223
Ca_Kabuli_NC_021160.1_9085302	Ca_kabuli_chr01	9085302	[A/C]	1067290224
Ca_Kabuli_NC_021160.1_9097860	Ca_kabuli_chr01	9097860	[T/C]	1067290225
Ca_Kabuli_NC_021160.1_9097864	Ca_kabuli_chr01	9097864	[T/C]	1067290226
Ca_Kabuli_NC_021160.1_9097878	Ca_kabuli_chr01	9097878	[A/G]	1067290227
Ca_Kabuli_NC_021160.1_9097879	Ca_kabuli_chr01	9097879	[C/A]	1067290228

SNP IDs	Chromosomes	Physical positions (bp)	SNPs	NCBI SNPdb accession IDs (NCBI_ss#)
Ca_Kabuli_NC_021160.1_9097881	Ca_kabuli_chr01	9097881	[C/G]	1067290229
Ca_Kabuli_NC_021160.1_9097885	Ca_kabuli_chr01	9097885	[A/G]	1067290230
Ca_Kabuli_NC_021160.1_9097999	Ca_kabuli_chr01	9097999	[A/C]	1067290231
Ca_Kabuli_NC_021160.1_9098048	Ca_kabuli_chr01	9098048	[A/C]	1067290232
Ca_Kabuli_NC_021160.1_9105452	Ca_kabuli_chr01	9105452	[T/G]	1067290233
Ca_Kabuli_NC_021160.1_9199585	Ca_kabuli_chr01	9199585	[T/C]	1067290234
Ca_Kabuli_NC_021160.1_9199591	Ca_kabuli_chr01	9199591	[T/C]	1067290235
Ca_Kabuli_NC_021160.1_9270090	Ca_kabuli_chr01	9270090	[T/G]	1067290236
Ca_Kabuli_NC_021160.1_9384194	Ca_kabuli_chr01	9384194	[T/C]	1067290237
Ca_Kabuli_NC_021160.1_9384265	Ca_kabuli_chr01	9384265	[A/T]	1067290238
Ca_Kabuli_NC_021160.1_9433023	Ca_kabuli_chr01	9433023	[G/A]	1067290239
Ca_Kabuli_NC_021160.1_9468055	Ca_kabuli_chr01	9468055	[A/G]	1067290240
Ca_Kabuli_NC_021160.1_9468064	Ca_kabuli_chr01	9468064	[C/T]	1067290241
Ca_Kabuli_NC_021160.1_9484646	Ca_kabuli_chr01	9484646	[T/C]	1067290242
Ca_Kabuli_NC_021160.1_9497704	Ca_kabuli_chr01	9497704	[T/A]	1067290243
Ca_Kabuli_NC_021160.1_9497705	Ca_kabuli_chr01	9497705	[C/A]	1067290244
Ca_Kabuli_NC_021160.1_9497706	Ca_kabuli_chr01	9497706	[T/G]	1067290245
Ca_Kabuli_NC_021160.1_9500436	Ca_kabuli_chr01	9500436	[T/C]	1067290246
Ca_Kabuli_NC_021160.1_9500455	Ca_kabuli_chr01	9500455	[A/C]	1067290247
Ca_Kabuli_NC_021160.1_9534606	Ca_kabuli_chr01	9534606	[A/C]	1067290248
Ca_Kabuli_NC_021160.1_9535634	Ca_kabuli_chr01	9535634	[A/G]	1067290249
Ca_Kabuli_NC_021160.1_9570032	Ca_kabuli_chr01	9570032	[A/G]	1067290250
Ca_Kabuli_NC_021160.1_9570058	Ca_kabuli_chr01	9570058	[A/G]	1067290251
Ca_Kabuli_NC_021160.1_9596690	Ca_kabuli_chr01	9596690	[C/A]	1067290252
Ca_Kabuli_NC_021160.1_9596691	Ca_kabuli_chr01	9596691	[T/G]	1067290253
Ca_Kabuli_NC_021160.1_9603920	Ca_kabuli_chr01	9603920	[T/A]	1067290254
Ca_Kabuli_NC_021160.1_9705108	Ca_kabuli_chr01	9705108	[T/G]	1067290255
Ca_Kabuli_NC_021160.1_9705127	Ca_kabuli_chr01	9705127	[C/T]	1067290256
Ca_Kabuli_NC_021160.1_9705151	Ca_kabuli_chr01	9705151	[A/G]	1067290257
Ca_Kabuli_NC_021160.1_9705183	Ca_kabuli_chr01	9705183	[T/G]	1067290258
Ca_Kabuli_NC_021160.1_9705197	Ca_kabuli_chr01	9705197	[T/G]	1067290259
Ca_Kabuli_NC_021160.1_9705199	Ca_kabuli_chr01	9705199	[A/G]	1067290260
Ca_Kabuli_NC_021160.1_9705217	Ca_kabuli_chr01	9705217	[A/G]	1067290261
Ca_Kabuli_NC_021160.1_9705244	Ca_kabuli_chr01	9705244	[A/G]	1067290262

SNP IDs	Chromosomes	Physical positions (bp)	SNPs	NCBI SNPdb accession IDs (NCBI_ss#)
Ca_Kabuli_NC_021160.1_9745090	Ca_kabuli_chr01	9745090	[C/A]	1067290263
Ca_Kabuli_NC_021160.1_9745091	Ca_kabuli_chr01	9745091	[A/G]	1067290264
Ca_Kabuli_NC_021160.1_9752908	Ca_kabuli_chr01	9752908	[T/G]	1067290265
Ca_Kabuli_NC_021160.1_9756950	Ca_kabuli_chr01	9756950	[T/A]	1067290266
Ca_Kabuli_NC_021160.1_9757008	Ca_kabuli_chr01	9757008	[A/C]	1067290267
Ca_Kabuli_NC_021160.1_9779385	Ca_kabuli_chr01	9779385	[T/A]	1067290268
Ca_Kabuli_NC_021160.1_9797297	Ca_kabuli_chr01	9797297	[T/C]	1067290269
Ca_Kabuli_NC_021160.1_9797352	Ca_kabuli_chr01	9797352	[G/T]	1067290270
Ca_Kabuli_NC_021160.1_9797353	Ca_kabuli_chr01	9797353	[T/C]	1067290271
Ca_Kabuli_NC_021160.1_9801542	Ca_kabuli_chr01	9801542	[T/G]	1067290272
Ca_Kabuli_NC_021160.1_9807759	Ca_kabuli_chr01	9807759	[T/G]	1067290273
Ca_Kabuli_NC_021160.1_9808455	Ca_kabuli_chr01	9808455	[A/C]	1067290274
Ca_Kabuli_NC_021160.1_9808476	Ca_kabuli_chr01	9808476	[G/A]	1067290275
Ca_Kabuli_NC_021160.1_9810246	Ca_kabuli_chr01	9810246	[T/C]	1067290276
Ca_Kabuli_NC_021160.1_9810284	Ca_kabuli_chr01	9810284	[A/G]	1067290277
Ca_Kabuli_NC_021160.1_9810295	Ca_kabuli_chr01	9810295	[T/C]	1067290278
Ca_Kabuli_NC_021160.1_9816351	Ca_kabuli_chr01	9816351	[T/C]	1067290279
Ca_Kabuli_NC_021160.1_9896510	Ca_kabuli_chr01	9896510	[A/G]	1067290280
Ca_Kabuli_NC_021160.1_9921381	Ca_kabuli_chr01	9921381	[C/A]	1067290281
Ca_Kabuli_NC_021160.1_9921382	Ca_kabuli_chr01	9921382	[A/G]	1067290282
Ca_Kabuli_NC_021160.1_9921383	Ca_kabuli_chr01	9921383	[G/A]	1067290283
Ca_Kabuli_NC_021160.1_9932701	Ca_kabuli_chr01	9932701	[T/C]	1067290284
Ca_Kabuli_NC_021160.1_9952513	Ca_kabuli_chr01	9952513	[A/G]	1067290285
Ca_Kabuli_NC_021160.1_9952565	Ca_kabuli_chr01	9952565	[T/G]	1067290286
Ca_Kabuli_NC_021160.1_10093170	Ca_kabuli_chr01	10093170	[T/A]	1067290287
Ca_Kabuli_NC_021160.1_10155527	Ca_kabuli_chr01	10155527	[T/G]	1067290288
Ca_Kabuli_NC_021160.1_10155547	Ca_kabuli_chr01	10155547	[T/C]	1067290289
Ca_Kabuli_NC_021160.1_10186582	Ca_kabuli_chr01	10186582	[T/G]	1067290290
Ca_Kabuli_NC_021160.1_10186667	Ca_kabuli_chr01	10186667	[T/G]	1067290291
Ca_Kabuli_NC_021160.1_10269116	Ca_kabuli_chr01	10269116	[C/T]	1067290292
Ca_Kabuli_NC_021160.1_10269117	Ca_kabuli_chr01	10269117	[A/C]	1067290293
Ca_Kabuli_NC_021160.1_10269118	Ca_kabuli_chr01	10269118	[G/T]	1067290294
Ca_Kabuli_NC_021160.1_10289771	Ca_kabuli_chr01	10289771	[T/G]	1067290295
Ca_Kabuli_NC_021160.1_10289805	Ca_kabuli_chr01	10289805	[T/G]	1067290296

SNP IDs	Chromosomes	Physical positions (bp)	SNPs	NCBI SNPdb accession IDs (NCBI_ss#)
Ca_Kabuli_NC_021160.1_10289825	Ca_kabuli_chr01	10289825	[A/G]	1067290297
Ca_Kabuli_NC_021160.1_10298355	Ca_kabuli_chr01	10298355	[A/T]	1067290298
Ca_Kabuli_NC_021160.1_10298363	Ca_kabuli_chr01	10298363	[T/C]	1067290299
Ca_Kabuli_NC_021160.1_10339063	Ca_kabuli_chr01	10339063	[G/T]	1067290300
Ca_Kabuli_NC_021160.1_10364978	Ca_kabuli_chr01	10364978	[T/A]	1067290301
Ca_Kabuli_NC_021160.1_10364981	Ca_kabuli_chr01	10364981	[C/A]	1067290302
Ca_Kabuli_NC_021160.1_10364986	Ca_kabuli_chr01	10364986	[C/G]	1067290303
Ca_Kabuli_NC_021160.1_10364992	Ca_kabuli_chr01	10364992	[C/A]	1067290304
Ca_Kabuli_NC_021160.1_10364999	Ca_kabuli_chr01	10364999	[G/A]	1067290305
Ca_Kabuli_NC_021160.1_10366346	Ca_kabuli_chr01	10366346	[T/C]	1067290306
Ca_Kabuli_NC_021160.1_10366380	Ca_kabuli_chr01	10366380	[A/T]	1067290307
Ca_Kabuli_NC_021160.1_10410393	Ca_kabuli_chr01	10410393	[T/A]	1067290308
Ca_Kabuli_NC_021160.1_10440427	Ca_kabuli_chr01	10440427	[A/G]	1067290309
Ca_Kabuli_NC_021160.1_10460268	Ca_kabuli_chr01	10460268	[A/G]	1067290310
Ca_Kabuli_NC_021160.1_10482467	Ca_kabuli_chr01	10482467	[A/G]	1067290311
Ca_Kabuli_NC_021160.1_10486212	Ca_kabuli_chr01	10486212	[T/G]	1067290312
Ca_Kabuli_NC_021160.1_10528100	Ca_kabuli_chr01	10528100	[A/C]	1067290313
Ca_Kabuli_NC_021160.1_10672358	Ca_kabuli_chr01	10672358	[C/A]	1067290314
Ca_Kabuli_NC_021160.1_10672359	Ca_kabuli_chr01	10672359	[A/G]	1067290315
Ca_Kabuli_NC_021160.1_10672360	Ca_kabuli_chr01	10672360	[T/A]	1067290316
Ca_Kabuli_NC_021160.1_10697416	Ca_kabuli_chr01	10697416	[T/G]	1067290317
Ca_Kabuli_NC_021160.1_10698016	Ca_kabuli_chr01	10698016	[T/A]	1067290318
Ca_Kabuli_NC_021160.1_10712205	Ca_kabuli_chr01	10712205	[T/G]	1067290319
Ca_Kabuli_NC_021160.1_10715409	Ca_kabuli_chr01	10715409	[A/C]	1067290320
Ca_Kabuli_NC_021160.1_10715410	Ca_kabuli_chr01	10715410	[T/A]	1067290321
Ca_Kabuli_NC_021160.1_10715411	Ca_kabuli_chr01	10715411	[C/A]	1067290322
Ca_Kabuli_NC_021160.1_10715412	Ca_kabuli_chr01	10715412	[T/G]	1067290323
Ca_Kabuli_NC_021160.1_10739566	Ca_kabuli_chr01	10739566	[C/A]	1067290324
Ca_Kabuli_NC_021160.1_10739567	Ca_kabuli_chr01	10739567	[A/T]	1067290325
Ca_Kabuli_NC_021160.1_10806321	Ca_kabuli_chr01	10806321	[A/C]	1067290326
Ca_Kabuli_NC_021160.1_10861266	Ca_kabuli_chr01	10861266	[A/G]	1067290327
Ca_Kabuli_NC_021160.1_10861285	Ca_kabuli_chr01	10861285	[A/G]	1067290328
Ca_Kabuli_NC_021160.1_10861326	Ca_kabuli_chr01	10861326	[T/G]	1067290329
Ca_Kabuli_NC_021160.1_10861328	Ca_kabuli_chr01	10861328	[T/G]	1067290330

SNP IDs	Chromosomes	Physical positions (bp)	SNPs	NCBI SNPdb accession IDs (NCBI_ss#)
Ca_Kabuli_NC_021160.1_10863032	Ca_kabuli_chr01	10863032	[T/A]	1067290331
Ca_Kabuli_NC_021160.1_10863037	Ca_kabuli_chr01	10863037	[T/C]	1067290332
Ca_Kabuli_NC_021160.1_10863079	Ca_kabuli_chr01	10863079	[A/G]	1067290333
Ca_Kabuli_NC_021160.1_10863094	Ca_kabuli_chr01	10863094	[T/C]	1067290334
Ca_Kabuli_NC_021160.1_10888044	Ca_kabuli_chr01	10888044	[A/G]	1067290335
Ca_Kabuli_NC_021160.1_10888047	Ca_kabuli_chr01	10888047	[C/T]	1067290336
Ca_Kabuli_NC_021160.1_10895661	Ca_kabuli_chr01	10895661	[T/C]	1067290337
Ca_Kabuli_NC_021160.1_10895664	Ca_kabuli_chr01	10895664	[T/C]	1067290338
Ca_Kabuli_NC_021160.1_10895673	Ca_kabuli_chr01	10895673	[T/A]	1067290339
Ca_Kabuli_NC_021160.1_10896314	Ca_kabuli_chr01	10896314	[A/G]	1067290340
Ca_Kabuli_NC_021160.1_10896328	Ca_kabuli_chr01	10896328	[G/A]	1067290341
Ca_Kabuli_NC_021160.1_10896363	Ca_kabuli_chr01	10896363	[G/T]	1067290342
Ca_Kabuli_NC_021160.1_10899031	Ca_kabuli_chr01	10899031	[T/C]	1067290343
Ca_Kabuli_NC_021160.1_10899032	Ca_kabuli_chr01	10899032	[G/T]	1067290344
Ca_Kabuli_NC_021160.1_10914064	Ca_kabuli_chr01	10914064	[T/C]	1067290345
Ca_Kabuli_NC_021160.1_10914094	Ca_kabuli_chr01	10914094	[T/C]	1067290346
Ca_Kabuli_NC_021160.1_10953405	Ca_kabuli_chr01	10953405	[A/G]	1067290347
Ca_Kabuli_NC_021160.1_10963892	Ca_kabuli_chr01	10963892	[A/C]	1067290348
Ca_Kabuli_NC_021160.1_10963897	Ca_kabuli_chr01	10963897	[A/T]	1067290349
Ca_Kabuli_NC_021160.1_10963917	Ca_kabuli_chr01	10963917	[A/C]	1067290350
Ca_Kabuli_NC_021160.1_10963954	Ca_kabuli_chr01	10963954	[T/C]	1067290351
Ca_Kabuli_NC_021160.1_11045048	Ca_kabuli_chr01	11045048	[T/C]	1067290352
Ca_Kabuli_NC_021160.1_11045064	Ca_kabuli_chr01	11045064	[T/G]	1067290353
Ca_Kabuli_NC_021160.1_11046645	Ca_kabuli_chr01	11046645	[A/T]	1067290354
Ca_Kabuli_NC_021160.1_11053473	Ca_kabuli_chr01	11053473	[A/G]	1067290355
Ca_Kabuli_NC_021160.1_11096731	Ca_kabuli_chr01	11096731	[T/C]	1067290356
Ca_Kabuli_NC_021160.1_11096787	Ca_kabuli_chr01	11096787	[T/G]	1067290357
Ca_Kabuli_NC_021160.1_11100632	Ca_kabuli_chr01	11100632	[T/C]	1067290358
Ca_Kabuli_NC_021160.1_11100676	Ca_kabuli_chr01	11100676	[A/G]	1067290359
Ca_Kabuli_NC_021160.1_11105572	Ca_kabuli_chr01	11105572	[A/C]	1067290360
Ca_Kabuli_NC_021160.1_11111108	Ca_kabuli_chr01	11111108	[A/C]	1067290361
Ca_Kabuli_NC_021160.1_11172819	Ca_kabuli_chr01	11172819	[T/G]	1067290362
Ca_Kabuli_NC_021160.1_11191087	Ca_kabuli_chr01	11191087	[A/T]	1067290363
Ca_Kabuli_NC_021160.1_11193223	Ca_kabuli_chr01	11193223	[A/T]	1067290364

SNP IDs	Chromosomes	Physical positions (bp)	SNPs	NCBI SNPdb accession IDs (NCBI_ss#)
Ca_Kabuli_NC_021160.1_11193251	Ca_kabuli_chr01	11193251	[T/A]	1067290365
Ca_Kabuli_NC_021160.1_11193266	Ca_kabuli_chr01	11193266	[T/G]	1067290366
Ca_Kabuli_NC_021160.1_11230109	Ca_kabuli_chr01	11230109	[T/C]	1067290367
Ca_Kabuli_NC_021160.1_11230377	Ca_kabuli_chr01	11230377	[A/C]	1067290368
Ca_Kabuli_NC_021160.1_11231022	Ca_kabuli_chr01	11231022	[T/G]	1067290369
Ca_Kabuli_NC_021160.1_11231081	Ca_kabuli_chr01	11231081	[A/G]	1067290370
Ca_Kabuli_NC_021160.1_11231094	Ca_kabuli_chr01	11231094	[A/G]	1067290371
Ca_Kabuli_NC_021160.1_11277751	Ca_kabuli_chr01	11277751	[A/C]	1067290372
Ca_Kabuli_NC_021160.1_11277808	Ca_kabuli_chr01	11277808	[A/G]	1067290373
Ca_Kabuli_NC_021160.1_11277812	Ca_kabuli_chr01	11277812	[T/C]	1067290374
Ca_Kabuli_NC_021160.1_11277832	Ca_kabuli_chr01	11277832	[A/C]	1067290375
Ca_Kabuli_NC_021160.1_11277884	Ca_kabuli_chr01	11277884	[A/T]	1067290376
Ca_Kabuli_NC_021160.1_11277913	Ca_kabuli_chr01	11277913	[T/A]	1067290377
Ca_Kabuli_NC_021160.1_11277945	Ca_kabuli_chr01	11277945	[A/G]	1067290378
Ca_Kabuli_NC_021160.1_11277953	Ca_kabuli_chr01	11277953	[T/G]	1067290379
Ca_Kabuli_NC_021160.1_11277961	Ca_kabuli_chr01	11277961	[A/G]	1067290380
Ca_Kabuli_NC_021160.1_11285295	Ca_kabuli_chr01	11285295	[A/G]	1067290381
Ca_Kabuli_NC_021160.1_11336814	Ca_kabuli_chr01	11336814	[T/G]	1067290382
Ca_Kabuli_NC_021160.1_11336858	Ca_kabuli_chr01	11336858	[A/C]	1067290383
Ca_Kabuli_NC_021160.1_11340761	Ca_kabuli_chr01	11340761	[T/C]	1067290384
Ca_Kabuli_NC_021160.1_11342365	Ca_kabuli_chr01	11342365	[A/G]	1067290385
Ca_Kabuli_NC_021160.1_11361625	Ca_kabuli_chr01	11361625	[A/C]	1067290386
Ca_Kabuli_NC_021160.1_11361687	Ca_kabuli_chr01	11361687	[T/A]	1067290387
Ca_Kabuli_NC_021160.1_11374655	Ca_kabuli_chr01	11374655	[T/A]	1067290388
Ca_Kabuli_NC_021160.1_11374733	Ca_kabuli_chr01	11374733	[A/G]	1067290389
Ca_Kabuli_NC_021160.1_11405118	Ca_kabuli_chr01	11405118	[G/A]	1067290390
Ca_Kabuli_NC_021160.1_11405133	Ca_kabuli_chr01	11405133	[A/G]	1067290391
Ca_Kabuli_NC_021160.1_11405155	Ca_kabuli_chr01	11405155	[T/C]	1067290392
Ca_Kabuli_NC_021160.1_11405163	Ca_kabuli_chr01	11405163	[T/C]	1067290393
Ca_Kabuli_NC_021160.1_11487640	Ca_kabuli_chr01	11487640	[C/A]	1067290394
Ca_Kabuli_NC_021160.1_11487664	Ca_kabuli_chr01	11487664	[C/T]	1067290395
Ca_Kabuli_NC_021160.1_11502160	Ca_kabuli_chr01	11502160	[G/T]	1067290396
Ca_Kabuli_NC_021160.1_11537936	Ca_kabuli_chr01	11537936	[T/C]	1067290397
Ca_Kabuli_NC_021160.1_11599706	Ca_kabuli_chr01	11599706	[T/G]	1067290398

SNP IDs	Chromosomes	Physical positions (bp)	SNPs	NCBI SNPdb accession IDs (NCBI_ss#)
Ca_Kabuli_NC_021160.1_11669101	Ca_kabuli_chr01	11669101	[A/G]	1067290399
Ca_Kabuli_NC_021160.1_11669721	Ca_kabuli_chr01	11669721	[T/G]	1067290400
Ca_Kabuli_NC_021160.1_11669794	Ca_kabuli_chr01	11669794	[G/T]	1067290401
Ca_Kabuli_NC_021160.1_11676536	Ca_kabuli_chr01	11676536	[A/T]	1067290402
Ca_Kabuli_NC_021160.1_11676582	Ca_kabuli_chr01	11676582	[T/C]	1067290403
Ca_Kabuli_NC_021160.1_11676658	Ca_kabuli_chr01	11676658	[A/C]	1067290404
Ca_Kabuli_NC_021160.1_11703765	Ca_kabuli_chr01	11703765	[A/C]	1067290405
Ca_Kabuli_NC_021160.1_11706510	Ca_kabuli_chr01	11706510	[T/G]	1067290406
Ca_Kabuli_NC_021160.1_11706577	Ca_kabuli_chr01	11706577	[T/C]	1067290407
Ca_Kabuli_NC_021160.1_11706597	Ca_kabuli_chr01	11706597	[A/G]	1067290408
Ca_Kabuli_NC_021160.1_11746090	Ca_kabuli_chr01	11746090	[T/C]	1067290409
Ca_Kabuli_NC_021160.1_11746122	Ca_kabuli_chr01	11746122	[T/C]	1067290410
Ca_Kabuli_NC_021160.1_11749976	Ca_kabuli_chr01	11749976	[A/C]	1067290411
Ca_Kabuli_NC_021160.1_11749979	Ca_kabuli_chr01	11749979	[A/C]	1067290412
Ca_Kabuli_NC_021160.1_11955897	Ca_kabuli_chr01	11955897	[T/G]	1067290413
Ca_Kabuli_NC_021160.1_11970674	Ca_kabuli_chr01	11970674	[A/C]	1067290414
Ca_Kabuli_NC_021160.1_11972242	Ca_kabuli_chr01	11972242	[T/C]	1067290415
Ca_Kabuli_NC_021160.1_11972251	Ca_kabuli_chr01	11972251	[G/A]	1067290416
Ca_Kabuli_NC_021160.1_12013222	Ca_kabuli_chr01	12013222	[A/T]	1067290417
Ca_Kabuli_NC_021160.1_12131395	Ca_kabuli_chr01	12131395	[T/C]	1067290418
Ca_Kabuli_NC_021160.1_12159607	Ca_kabuli_chr01	12159607	[A/C]	1067290419
Ca_Kabuli_NC_021160.1_12169598	Ca_kabuli_chr01	12169598	[G/A]	1067290420
Ca_Kabuli_NC_021160.1_12189933	Ca_kabuli_chr01	12189933	[A/C]	1067290421
Ca_Kabuli_NC_021160.1_12326230	Ca_kabuli_chr01	12326230	[A/G]	1067290422
Ca_Kabuli_NC_021160.1_12326232	Ca_kabuli_chr01	12326232	[T/G]	1067290423
Ca_Kabuli_NC_021160.1_12326242	Ca_kabuli_chr01	12326242	[G/T]	1067290424
Ca_Kabuli_NC_021160.1_12326264	Ca_kabuli_chr01	12326264	[T/G]	1067290425
Ca_Kabuli_NC_021160.1_12337445	Ca_kabuli_chr01	12337445	[T/C]	1067290426
Ca_Kabuli_NC_021160.1_12340978	Ca_kabuli_chr01	12340978	[A/C]	1067290427
Ca_Kabuli_NC_021160.1_12341006	Ca_kabuli_chr01	12341006	[T/G]	1067290428
Ca_Kabuli_NC_021160.1_12341059	Ca_kabuli_chr01	12341059	[A/C]	1067290429
Ca_Kabuli_NC_021160.1_12341081	Ca_kabuli_chr01	12341081	[T/C]	1067290430
Ca_Kabuli_NC_021160.1_12341147	Ca_kabuli_chr01	12341147	[T/G]	1067290431
Ca_Kabuli_NC_021160.1_12372201	Ca_kabuli_chr01	12372201	[C/T]	1067290432

SNP IDs	Chromosomes	Physical positions (bp)	SNPs	NCBI SNPdb accession IDs (NCBI_ss#)
Ca_Kabuli_NC_021160.1_12390750	Ca_kabuli_chr01	12390750	[T/C]	1067290433
Ca_Kabuli_NC_021160.1_12390781	Ca_kabuli_chr01	12390781	[A/G]	1067290434
Ca_Kabuli_NC_021160.1_12412979	Ca_kabuli_chr01	12412979	[A/T]	1067290435
Ca_Kabuli_NC_021160.1_12413012	Ca_kabuli_chr01	12413012	[G/T]	1067290436
Ca_Kabuli_NC_021160.1_12413016	Ca_kabuli_chr01	12413016	[T/A]	1067290437
Ca_Kabuli_NC_021160.1_12413080	Ca_kabuli_chr01	12413080	[C/T]	1067290438
Ca_Kabuli_NC_021160.1_12434148	Ca_kabuli_chr01	12434148	[T/G]	1067290439
Ca_Kabuli_NC_021160.1_12439871	Ca_kabuli_chr01	12439871	[T/G]	1067290440
Ca_Kabuli_NC_021160.1_12517452	Ca_kabuli_chr01	12517452	[T/C]	1067290441
Ca_Kabuli_NC_021160.1_12517473	Ca_kabuli_chr01	12517473	[A/G]	1067290442
Ca_Kabuli_NC_021160.1_12561061	Ca_kabuli_chr01	12561061	[A/G]	1067290443
Ca_Kabuli_NC_021160.1_12561084	Ca_kabuli_chr01	12561084	[T/A]	1067290444
Ca_Kabuli_NC_021160.1_12561121	Ca_kabuli_chr01	12561121	[T/A]	1067290445
Ca_Kabuli_NC_021160.1_12575980	Ca_kabuli_chr01	12575980	[T/C]	1067290446
Ca_Kabuli_NC_021160.1_12588797	Ca_kabuli_chr01	12588797	[A/C]	1067290447
Ca_Kabuli_NC_021160.1_12640616	Ca_kabuli_chr01	12640616	[T/C]	1067290448
Ca_Kabuli_NC_021160.1_12640627	Ca_kabuli_chr01	12640627	[T/C]	1067290449
Ca_Kabuli_NC_021160.1_12640721	Ca_kabuli_chr01	12640721	[G/T]	1067290450
Ca_Kabuli_NC_021160.1_12641452	Ca_kabuli_chr01	12641452	[A/C]	1067290451
Ca_Kabuli_NC_021160.1_12642126	Ca_kabuli_chr01	12642126	[T/A]	1067290452
Ca_Kabuli_NC_021160.1_12642147	Ca_kabuli_chr01	12642147	[A/C]	1067290453
Ca_Kabuli_NC_021160.1_12688435	Ca_kabuli_chr01	12688435	[A/C]	1067290454
Ca_Kabuli_NC_021160.1_12704408	Ca_kabuli_chr01	12704408	[T/A]	1067290455
Ca_Kabuli_NC_021160.1_12704473	Ca_kabuli_chr01	12704473	[A/G]	1067290456
Ca_Kabuli_NC_021160.1_12713237	Ca_kabuli_chr01	12713237	[T/C]	1067290457
Ca_Kabuli_NC_021160.1_12736318	Ca_kabuli_chr01	12736318	[T/G]	1067290458
Ca_Kabuli_NC_021160.1_12736332	Ca_kabuli_chr01	12736332	[A/G]	1067290459
Ca_Kabuli_NC_021160.1_12736575	Ca_kabuli_chr01	12736575	[T/G]	1067290460
Ca_Kabuli_NC_021160.1_12772559	Ca_kabuli_chr01	12772559	[G/C]	1067290461
Ca_Kabuli_NC_021160.1_12773810	Ca_kabuli_chr01	12773810	[A/C]	1067290462
Ca_Kabuli_NC_021160.1_12800435	Ca_kabuli_chr01	12800435	[C/A]	1067290463
Ca_Kabuli_NC_021160.1_12800436	Ca_kabuli_chr01	12800436	[T/G]	1067290464
Ca_Kabuli_NC_021160.1_12800607	Ca_kabuli_chr01	12800607	[T/A]	1067290465
Ca_Kabuli_NC_021160.1_12800610	Ca_kabuli_chr01	12800610	[A/C]	1067290466

SNP IDs	Chromosomes	Physical positions (bp)	SNPs	NCBI SNPdb accession IDs (NCBI_ss#)
Ca_Kabuli_NC_021160.1_12824152	Ca_kabuli_chr01	12824152	[A/C]	1067290467
Ca_Kabuli_NC_021160.1_12844395	Ca_kabuli_chr01	12844395	[A/G]	1067290468
Ca_Kabuli_NC_021160.1_12880420	Ca_kabuli_chr01	12880420	[T/A]	1067290469
Ca_Kabuli_NC_021160.1_12948219	Ca_kabuli_chr01	12948219	[A/G]	1067290470
Ca_Kabuli_NC_021160.1_12948253	Ca_kabuli_chr01	12948253	[T/A]	1067290471
Ca_Kabuli_NC_021160.1_12990248	Ca_kabuli_chr01	12990248	[T/C]	1067290472
Ca_Kabuli_NC_021160.1_12990448	Ca_kabuli_chr01	12990448	[T/C]	1067290473
Ca_Kabuli_NC_021160.1_12990476	Ca_kabuli_chr01	12990476	[T/C]	1067290474
Ca_Kabuli_NC_021160.1_12990489	Ca_kabuli_chr01	12990489	[T/C]	1067290475
Ca_Kabuli_NC_021160.1_12990780	Ca_kabuli_chr01	12990780	[A/T]	1067290476
Ca_Kabuli_NC_021160.1_12990784	Ca_kabuli_chr01	12990784	[T/C]	1067290477
Ca_Kabuli_NC_021160.1_12990819	Ca_kabuli_chr01	12990819	[A/G]	1067290478
Ca_Kabuli_NC_021160.1_12991618	Ca_kabuli_chr01	12991618	[T/C]	1067290479
Ca_Kabuli_NC_021160.1_12991624	Ca_kabuli_chr01	12991624	[A/G]	1067290480
Ca_Kabuli_NC_021160.1_12991635	Ca_kabuli_chr01	12991635	[A/G]	1067290481
Ca_Kabuli_NC_021160.1_12991638	Ca_kabuli_chr01	12991638	[A/C]	1067290482
Ca_Kabuli_NC_021160.1_12991923	Ca_kabuli_chr01	12991923	[T/C]	1067290483
Ca_Kabuli_NC_021160.1_12991944	Ca_kabuli_chr01	12991944	[A/G]	1067290484
Ca_Kabuli_NC_021160.1_12991962	Ca_kabuli_chr01	12991962	[T/A]	1067290485
Ca_Kabuli_NC_021160.1_12992439	Ca_kabuli_chr01	12992439	[A/C]	1067290486
Ca_Kabuli_NC_021160.1_12992445	Ca_kabuli_chr01	12992445	[A/C]	1067290487
Ca_Kabuli_NC_021160.1_12992448	Ca_kabuli_chr01	12992448	[T/C]	1067290488
Ca_Kabuli_NC_021160.1_12992456	Ca_kabuli_chr01	12992456	[T/C]	1067290489
Ca_Kabuli_NC_021160.1_12993764	Ca_kabuli_chr01	12993764	[T/C]	1067290490
Ca_Kabuli_NC_021160.1_12993785	Ca_kabuli_chr01	12993785	[T/C]	1067290491
Ca_Kabuli_NC_021160.1_12993883	Ca_kabuli_chr01	12993883	[A/G]	1067290492
Ca_Kabuli_NC_021160.1_12993887	Ca_kabuli_chr01	12993887	[A/G]	1067290493
Ca_Kabuli_NC_021160.1_12993898	Ca_kabuli_chr01	12993898	[T/C]	1067290494
Ca_Kabuli_NC_021160.1_12993909	Ca_kabuli_chr01	12993909	[A/G]	1067290495
Ca_Kabuli_NC_021160.1_12993953	Ca_kabuli_chr01	12993953	[T/A]	1067290496
Ca_Kabuli_NC_021160.1_12994024	Ca_kabuli_chr01	12994024	[T/C]	1067290497
Ca_Kabuli_NC_021160.1_12994053	Ca_kabuli_chr01	12994053	[T/C]	1067290498
Ca_Kabuli_NC_021160.1_12994470	Ca_kabuli_chr01	12994470	[A/T]	1067290499
Ca_Kabuli_NC_021160.1_13034730	Ca_kabuli_chr01	13034730	[A/G]	1067290500

SNP IDs	Chromosomes	Physical positions (bp)	SNPs	NCBI SNPdb accession IDs (NCBI_ss#)
Ca_Kabuli_NC_021160.1_13034733	Ca_kabuli_chr01	13034733	[A/C]	1067290501
Ca_Kabuli_NC_021160.1_13034756	Ca_kabuli_chr01	13034756	[T/C]	1067290502
Ca_Kabuli_NC_021160.1_13034785	Ca_kabuli_chr01	13034785	[G/A]	1067290503
Ca_Kabuli_NC_021160.1_13034819	Ca_kabuli_chr01	13034819	[A/G]	1067290504
Ca_Kabuli_NC_021160.1_13034821	Ca_kabuli_chr01	13034821	[T/C]	1067290505
Ca_Kabuli_NC_021160.1_13040711	Ca_kabuli_chr01	13040711	[A/T]	1067290506
Ca_Kabuli_NC_021160.1_13112887	Ca_kabuli_chr01	13112887	[T/A]	1067290507
Ca_Kabuli_NC_021160.1_13112892	Ca_kabuli_chr01	13112892	[T/G]	1067290508
Ca_Kabuli_NC_021160.1_13156427	Ca_kabuli_chr01	13156427	[A/C]	1067290509
Ca_Kabuli_NC_021160.1_13158171	Ca_kabuli_chr01	13158171	[T/A]	1067290510
Ca_Kabuli_NC_021160.1_13203052	Ca_kabuli_chr01	13203052	[A/C]	1067290511
Ca_Kabuli_NC_021160.1_13262992	Ca_kabuli_chr01	13262992	[T/G]	1067290512
Ca_Kabuli_NC_021160.1_13263007	Ca_kabuli_chr01	13263007	[A/C]	1067290513
Ca_Kabuli_NC_021160.1_13267650	Ca_kabuli_chr01	13267650	[A/C]	1067290514
Ca_Kabuli_NC_021160.1_13365552	Ca_kabuli_chr01	13365552	[T/C]	1067290515
Ca_Kabuli_NC_021160.1_13365564	Ca_kabuli_chr01	13365564	[T/G]	1067290516
Ca_Kabuli_NC_021160.1_13404600	Ca_kabuli_chr01	13404600	[T/G]	1067290517
Ca_Kabuli_NC_021160.1_13501332	Ca_kabuli_chr01	13501332	[A/C]	1067290518
Ca_Kabuli_NC_021160.1_13549635	Ca_kabuli_chr01	13549635	[T/C]	1067290519
Ca_Kabuli_NC_021160.1_13593876	Ca_kabuli_chr01	13593876	[T/C]	1067290520
Ca_Kabuli_NC_021160.1_13596442	Ca_kabuli_chr01	13596442	[T/C]	1067290521
Ca_Kabuli_NC_021160.1_13607447	Ca_kabuli_chr01	13607447	[A/C]	1067290522
Ca_Kabuli_NC_021160.1_13607463	Ca_kabuli_chr01	13607463	[A/G]	1067290523
Ca_Kabuli_NC_021160.1_13607468	Ca_kabuli_chr01	13607468	[A/G]	1067290524
Ca_Kabuli_NC_021160.1_13637683	Ca_kabuli_chr01	13637683	[A/C]	1067290525
Ca_Kabuli_NC_021160.1_13637714	Ca_kabuli_chr01	13637714	[A/G]	1067290526
Ca_Kabuli_NC_021160.1_13641192	Ca_kabuli_chr01	13641192	[T/C]	1067290527
Ca_Kabuli_NC_021160.1_13668866	Ca_kabuli_chr01	13668866	[T/G]	1067290528
Ca_Kabuli_NC_021160.1_13679996	Ca_kabuli_chr01	13679996	[A/G]	1067290529
Ca_Kabuli_NC_021160.1_13680008	Ca_kabuli_chr01	13680008	[G/C]	1067290530
Ca_Kabuli_NC_021160.1_13759589	Ca_kabuli_chr01	13759589	[G/C]	1067290531
Ca_Kabuli_NC_021160.1_13828672	Ca_kabuli_chr01	13828672	[A/C]	1067290532
Ca_Kabuli_NC_021160.1_13880760	Ca_kabuli_chr01	13880760	[T/C]	1067290533
Ca_Kabuli_NC_021160.1_13880810	Ca_kabuli_chr01	13880810	[A/G]	1067290534

SNP IDs	Chromosomes	Physical positions (bp)	SNPs	NCBI SNPdb accession IDs (NCBI_ss#)
Ca_Kabuli_NC_021160.1_14004185	Ca_kabuli_chr01	14004185	[A/C]	1067290535
Ca_Kabuli_NC_021160.1_14015757	Ca_kabuli_chr01	14015757	[T/C]	1067290536
Ca_Kabuli_NC_021160.1_14022915	Ca_kabuli_chr01	14022915	[A/G]	1067290537
Ca_Kabuli_NC_021160.1_14022932	Ca_kabuli_chr01	14022932	[A/C]	1067290538
Ca_Kabuli_NC_021160.1_14023047	Ca_kabuli_chr01	14023047	[A/C]	1067290539
Ca_Kabuli_NC_021160.1_14026370	Ca_kabuli_chr01	14026370	[T/G]	1067290540
Ca_Kabuli_NC_021160.1_14047891	Ca_kabuli_chr01	14047891	[G/A]	1067290541
Ca_Kabuli_NC_021160.1_14047892	Ca_kabuli_chr01	14047892	[A/T]	1067290542
Ca_Kabuli_NC_021160.1_14047896	Ca_kabuli_chr01	14047896	[T/G]	1067290543
Ca_Kabuli_NC_021160.1_14047901	Ca_kabuli_chr01	14047901	[T/C]	1067290544
Ca_Kabuli_NC_021160.1_14076966	Ca_kabuli_chr01	14076966	[T/A]	1067290545
Ca_Kabuli_NC_021160.1_14181789	Ca_kabuli_chr01	14181789	[T/G]	1067290546
Ca_Kabuli_NC_021160.1_14181834	Ca_kabuli_chr01	14181834	[A/C]	1067290547
Ca_Kabuli_NC_021160.1_14207277	Ca_kabuli_chr01	14207277	[G/A]	1067290548
Ca_Kabuli_NC_021160.1_14240580	Ca_kabuli_chr01	14240580	[A/G]	1067290549
Ca_Kabuli_NC_021160.1_14240590	Ca_kabuli_chr01	14240590	[T/G]	1067290550
Ca_Kabuli_NC_021160.1_14244545	Ca_kabuli_chr01	14244545	[T/C]	1067290551
Ca_Kabuli_NC_021160.1_14259438	Ca_kabuli_chr01	14259438	[A/C]	1067290552
Ca_Kabuli_NC_021160.1_14282344	Ca_kabuli_chr01	14282344	[A/G]	1067290553
Ca_Kabuli_NC_021160.1_14282377	Ca_kabuli_chr01	14282377	[A/G]	1067290554
Ca_Kabuli_NC_021160.1_14296994	Ca_kabuli_chr01	14296994	[A/C]	1067290555
Ca_Kabuli_NC_021160.1_14296995	Ca_kabuli_chr01	14296995	[G/T]	1067290556
Ca_Kabuli_NC_021160.1_14296996	Ca_kabuli_chr01	14296996	[A/C]	1067290557
Ca_Kabuli_NC_021160.1_14296997	Ca_kabuli_chr01	14296997	[T/A]	1067290558
Ca_Kabuli_NC_021160.1_14390428	Ca_kabuli_chr01	14390428	[C/A]	1067290559
Ca_Kabuli_NC_021160.1_14390429	Ca_kabuli_chr01	14390429	[T/G]	1067290560
Ca_Kabuli_NC_021160.1_14390430	Ca_kabuli_chr01	14390430	[T/A]	1067290561
Ca_Kabuli_NC_021160.1_14390431	Ca_kabuli_chr01	14390431	[A/T]	1067290562
Ca_Kabuli_NC_021160.1_14405466	Ca_kabuli_chr01	14405466	[C/A]	1067290563
Ca_Kabuli_NC_021160.1_14409571	Ca_kabuli_chr01	14409571	[A/C]	1067290564
Ca_Kabuli_NC_021160.1_14409604	Ca_kabuli_chr01	14409604	[A/C]	1067290565
Ca_Kabuli_NC_021160.1_14410147	Ca_kabuli_chr01	14410147	[A/C]	1067290566
Ca_Kabuli_NC_021160.1_14410862	Ca_kabuli_chr01	14410862	[T/C]	1067290567
Ca_Kabuli_NC_021160.1_14411513	Ca_kabuli_chr01	14411513	[T/G]	1067290568

SNP IDs	Chromosomes	Physical positions (bp)	SNPs	NCBI SNPdb accession IDs (NCBI_ss#)
Ca_Kabuli_NC_021160.1_14411531	Ca_kabuli_chr01	14411531	[T/A]	1067290569
Ca_Kabuli_NC_021160.1_14489206	Ca_kabuli_chr01	14489206	[C/A]	1067290570
Ca_Kabuli_NC_021160.1_14489208	Ca_kabuli_chr01	14489208	[T/A]	1067290571
Ca_Kabuli_NC_021160.1_14489210	Ca_kabuli_chr01	14489210	[A/C]	1067290572
Ca_Kabuli_NC_021160.1_14489212	Ca_kabuli_chr01	14489212	[T/G]	1067290573
Ca_Kabuli_NC_021160.1_14583058	Ca_kabuli_chr01	14583058	[A/G]	1067290574
Ca_Kabuli_NC_021160.1_14583073	Ca_kabuli_chr01	14583073	[A/C]	1067290575
Ca_Kabuli_NC_021160.1_14583076	Ca_kabuli_chr01	14583076	[A/G]	1067290576
Ca_Kabuli_NC_021160.1_14617743	Ca_kabuli_chr01	14617743	[T/G]	1067290577
Ca_Kabuli_NC_021160.1_14617751	Ca_kabuli_chr01	14617751	[T/A]	1067290578
Ca_Kabuli_NC_021160.1_14617832	Ca_kabuli_chr01	14617832	[T/A]	1067290579
Ca_Kabuli_NC_021160.1_14617834	Ca_kabuli_chr01	14617834	[G/T]	1067290580
Ca_Kabuli_NC_021160.1_14617869	Ca_kabuli_chr01	14617869	[A/C]	1067290581
Ca_Kabuli_NC_021160.1_14623270	Ca_kabuli_chr01	14623270	[C/T]	1067290582
Ca_Kabuli_NC_021160.1_14623271	Ca_kabuli_chr01	14623271	[T/C]	1067290583
Ca_Kabuli_NC_021160.1_14623279	Ca_kabuli_chr01	14623279	[T/G]	1067290584
Ca_Kabuli_NC_021160.1_14631966	Ca_kabuli_chr01	14631966	[T/C]	1067290585
Ca_Kabuli_NC_021160.1_14631995	Ca_kabuli_chr01	14631995	[A/C]	1067290586
Ca_Kabuli_NC_021160.1_14801393	Ca_kabuli_chr01	14801393	[T/G]	1067290587
Ca_Kabuli_NC_021160.1_14801397	Ca_kabuli_chr01	14801397	[T/C]	1067290588
Ca_Kabuli_NC_021160.1_14801430	Ca_kabuli_chr01	14801430	[A/C]	1067290589
Ca_Kabuli_NC_021160.1_14801444	Ca_kabuli_chr01	14801444	[A/C]	1067290590
Ca_Kabuli_NC_021160.1_14801466	Ca_kabuli_chr01	14801466	[A/G]	1067290591
Ca_Kabuli_NC_021160.1_14884927	Ca_kabuli_chr01	14884927	[A/G]	1067290592
Ca_Kabuli_NC_021160.1_14884951	Ca_kabuli_chr01	14884951	[T/A]	1067290593
Ca_Kabuli_NC_021160.1_14911564	Ca_kabuli_chr01	14911564	[A/C]	1067290594
Ca_Kabuli_NC_021160.1_14960025	Ca_kabuli_chr01	14960025	[C/T]	1067290595
Ca_Kabuli_NC_021160.1_15008621	Ca_kabuli_chr01	15008621	[C/A]	1067290596
Ca_Kabuli_NC_021160.1_15008622	Ca_kabuli_chr01	15008622	[A/G]	1067290597
Ca_Kabuli_NC_021160.1_15021857	Ca_kabuli_chr01	15021857	[A/C]	1067290598
Ca_Kabuli_NC_021160.1_15104356	Ca_kabuli_chr01	15104356	[T/G]	1067290599
Ca_Kabuli_NC_021160.1_15108435	Ca_kabuli_chr01	15108435	[T/A]	1067290600
Ca_Kabuli_NC_021160.1_15140980	Ca_kabuli_chr01	15140980	[T/G]	1067290601
Ca_Kabuli_NC_021160.1_15144307	Ca_kabuli_chr01	15144307	[T/C]	1067290602

SNP IDs	Chromosomes	Physical positions (bp)	SNPs	NCBI SNPdb accession IDs (NCBI_ss#)
Ca_Kabuli_NC_021160.1_15146092	Ca_kabuli_chr01	15146092	[T/C]	1067290603
Ca_Kabuli_NC_021160.1_15146099	Ca_kabuli_chr01	15146099	[A/G]	1067290604
Ca_Kabuli_NC_021160.1_15146104	Ca_kabuli_chr01	15146104	[A/G]	1067290605
Ca_Kabuli_NC_021160.1_15154171	Ca_kabuli_chr01	15154171	[T/G]	1067290606
Ca_Kabuli_NC_021160.1_15167660	Ca_kabuli_chr01	15167660	[A/C]	1067290607
Ca_Kabuli_NC_021160.1_15196079	Ca_kabuli_chr01	15196079	[T/C]	1067290608
Ca_Kabuli_NC_021160.1_15196091	Ca_kabuli_chr01	15196091	[A/C]	1067290609
Ca_Kabuli_NC_021160.1_15233627	Ca_kabuli_chr01	15233627	[C/A]	1067290610
Ca_Kabuli_NC_021160.1_15233628	Ca_kabuli_chr01	15233628	[C/T]	1067290611
Ca_Kabuli_NC_021160.1_15233629	Ca_kabuli_chr01	15233629	[G/C]	1067290612
Ca_Kabuli_NC_021160.1_15233630	Ca_kabuli_chr01	15233630	[G/T]	1067290613
Ca_Kabuli_NC_021160.1_15253222	Ca_kabuli_chr01	15253222	[T/C]	1067290614
Ca_Kabuli_NC_021160.1_15253233	Ca_kabuli_chr01	15253233	[C/A]	1067290615
Ca_Kabuli_NC_021160.1_15253238	Ca_kabuli_chr01	15253238	[G/C]	1067290616
Ca_Kabuli_NC_021160.1_15253243	Ca_kabuli_chr01	15253243	[T/C]	1067290617
Ca_Kabuli_NC_021160.1_15253253	Ca_kabuli_chr01	15253253	[A/G]	1067290618
Ca_Kabuli_NC_021160.1_15253257	Ca_kabuli_chr01	15253257	[A/G]	1067290619
Ca_Kabuli_NC_021160.1_15253261	Ca_kabuli_chr01	15253261	[A/G]	1067290620
Ca_Kabuli_NC_021160.1_15285011	Ca_kabuli_chr01	15285011	[G/T]	1067290621
Ca_Kabuli_NC_021160.1_15291788	Ca_kabuli_chr01	15291788	[T/C]	1067290622
Ca_Kabuli_NC_021160.1_15291832	Ca_kabuli_chr01	15291832	[T/A]	1067290623
Ca_Kabuli_NC_021160.1_15291847	Ca_kabuli_chr01	15291847	[A/G]	1067290624
Ca_Kabuli_NC_021160.1_15373744	Ca_kabuli_chr01	15373744	[C/A]	1067290625
Ca_Kabuli_NC_021160.1_15373745	Ca_kabuli_chr01	15373745	[C/G]	1067290626
Ca_Kabuli_NC_021160.1_15373746	Ca_kabuli_chr01	15373746	[T/A]	1067290627
Ca_Kabuli_NC_021160.1_15493528	Ca_kabuli_chr01	15493528	[T/G]	1067290628
Ca_Kabuli_NC_021160.1_15645681	Ca_kabuli_chr01	15645681	[G/A]	1067290629
Ca_Kabuli_NC_021160.1_15645682	Ca_kabuli_chr01	15645682	[A/T]	1067290630
Ca_Kabuli_NC_021160.1_15645684	Ca_kabuli_chr01	15645684	[C/T]	1067290631
Ca_Kabuli_NC_021160.1_15645685	Ca_kabuli_chr01	15645685	[T/G]	1067290632
Ca_Kabuli_NC_021160.1_15683176	Ca_kabuli_chr01	15683176	[T/C]	1067290633
Ca_Kabuli_NC_021160.1_15799075	Ca_kabuli_chr01	15799075	[T/G]	1067290634
Ca_Kabuli_NC_021160.1_15936187	Ca_kabuli_chr01	15936187	[A/G]	1067290635
Ca_Kabuli_NC_021160.1_15938115	Ca_kabuli_chr01	15938115	[C/A]	1067290636

SNP IDs	Chromosomes	Physical positions (bp)	SNPs	NCBI SNPdb accession IDs (NCBI_ss#)
Ca_Kabuli_NC_021160.1_15938116	Ca_kabuli_chr01	15938116	[T/G]	1067290637
Ca_Kabuli_NC_021160.1_15959271	Ca_kabuli_chr01	15959271	[A/G]	1067290638
Ca_Kabuli_NC_021160.1_15959355	Ca_kabuli_chr01	15959355	[T/C]	1067290639
Ca_Kabuli_NC_021160.1_15959392	Ca_kabuli_chr01	15959392	[A/C]	1067290640
Ca_Kabuli_NC_021160.1_15959435	Ca_kabuli_chr01	15959435	[G/A]	1067290641
Ca_Kabuli_NC_021160.1_15960425	Ca_kabuli_chr01	15960425	[A/C]	1067290642
Ca_Kabuli_NC_021160.1_15960455	Ca_kabuli_chr01	15960455	[A/G]	1067290643
Ca_Kabuli_NC_021160.1_15960476	Ca_kabuli_chr01	15960476	[T/G]	1067290644
Ca_Kabuli_NC_021160.1_15964019	Ca_kabuli_chr01	15964019	[A/T]	1067290645
Ca_Kabuli_NC_021160.1_15964060	Ca_kabuli_chr01	15964060	[T/A]	1067290646
Ca_Kabuli_NC_021160.1_15965278	Ca_kabuli_chr01	15965278	[A/G]	1067290647
Ca_Kabuli_NC_021160.1_15965367	Ca_kabuli_chr01	15965367	[A/C]	1067290648
Ca_Kabuli_NC_021160.1_16061265	Ca_kabuli_chr01	16061265	[T/G]	1067290649
Ca_Kabuli_NC_021160.1_16114593	Ca_kabuli_chr01	16114593	[A/G]	1067290650
Ca_Kabuli_NC_021160.1_16228094	Ca_kabuli_chr01	16228094	[G/T]	1067290651
Ca_Kabuli_NC_021160.1_16376274	Ca_kabuli_chr01	16376274	[T/A]	1067290652
Ca_Kabuli_NC_021160.1_16385973	Ca_kabuli_chr01	16385973	[T/A]	1067290653
Ca_Kabuli_NC_021160.1_16385979	Ca_kabuli_chr01	16385979	[T/A]	1067290654
Ca_Kabuli_NC_021160.1_16385986	Ca_kabuli_chr01	16385986	[A/C]	1067290655
Ca_Kabuli_NC_021160.1_16386014	Ca_kabuli_chr01	16386014	[A/T]	1067290656
Ca_Kabuli_NC_021160.1_16386016	Ca_kabuli_chr01	16386016	[T/C]	1067290657
Ca_Kabuli_NC_021160.1_16386040	Ca_kabuli_chr01	16386040	[A/C]	1067290658
Ca_Kabuli_NC_021160.1_16386050	Ca_kabuli_chr01	16386050	[T/C]	1067290659
Ca_Kabuli_NC_021160.1_16386063	Ca_kabuli_chr01	16386063	[A/G]	1067290660
Ca_Kabuli_NC_021160.1_16386076	Ca_kabuli_chr01	16386076	[A/C]	1067290661
Ca_Kabuli_NC_021160.1_16419482	Ca_kabuli_chr01	16419482	[A/T]	1067290662
Ca_Kabuli_NC_021160.1_16467635	Ca_kabuli_chr01	16467635	[G/T]	1067290663
Ca_Kabuli_NC_021160.1_16509078	Ca_kabuli_chr01	16509078	[T/C]	1067290664
Ca_Kabuli_NC_021160.1_16511324	Ca_kabuli_chr01	16511324	[T/G]	1067290665
Ca_Kabuli_NC_021160.1_16513357	Ca_kabuli_chr01	16513357	[A/G]	1067290666
Ca_Kabuli_NC_021160.1_16526502	Ca_kabuli_chr01	16526502	[T/A]	1067290667
Ca_Kabuli_NC_021160.1_16526608	Ca_kabuli_chr01	16526608	[A/T]	1067290668
Ca_Kabuli_NC_021160.1_16526710	Ca_kabuli_chr01	16526710	[C/A]	1067290669
Ca_Kabuli_NC_021160.1_16527348	Ca_kabuli_chr01	16527348	[C/T]	1067290670

SNP IDs	Chromosomes	Physical positions (bp)	SNPs	NCBI SNPdb accession IDs (NCBI_ss#)
Ca_Kabuli_NC_021160.1_16527372	Ca_kabuli_chr01	16527372	[A/C]	1067290671
Ca_Kabuli_NC_021160.1_16552357	Ca_kabuli_chr01	16552357	[T/C]	1067290672
Ca_Kabuli_NC_021160.1_16589738	Ca_kabuli_chr01	16589738	[A/G]	1067290673
Ca_Kabuli_NC_021160.1_16589752	Ca_kabuli_chr01	16589752	[A/C]	1067290674
Ca_Kabuli_NC_021160.1_16609370	Ca_kabuli_chr01	16609370	[A/G]	1067290675
Ca_Kabuli_NC_021160.1_16609385	Ca_kabuli_chr01	16609385	[T/C]	1067290676
Ca_Kabuli_NC_021160.1_16609399	Ca_kabuli_chr01	16609399	[A/C]	1067290677
Ca_Kabuli_NC_021160.1_16609410	Ca_kabuli_chr01	16609410	[T/G]	1067290678
Ca_Kabuli_NC_021160.1_16609413	Ca_kabuli_chr01	16609413	[T/C]	1067290679
Ca_Kabuli_NC_021160.1_16614308	Ca_kabuli_chr01	16614308	[A/C]	1067290680
Ca_Kabuli_NC_021160.1_16614769	Ca_kabuli_chr01	16614769	[A/C]	1067290681
Ca_Kabuli_NC_021160.1_16636571	Ca_kabuli_chr01	16636571	[A/C]	1067290682
Ca_Kabuli_NC_021160.1_16636574	Ca_kabuli_chr01	16636574	[A/T]	1067290683
Ca_Kabuli_NC_021160.1_16883186	Ca_kabuli_chr01	16883186	[A/G]	1067290684
Ca_Kabuli_NC_021160.1_16901797	Ca_kabuli_chr01	16901797	[T/G]	1067290685
Ca_Kabuli_NC_021160.1_16908580	Ca_kabuli_chr01	16908580	[T/G]	1067290686
Ca_Kabuli_NC_021160.1_16923207	Ca_kabuli_chr01	16923207	[A/T]	1067290687
Ca_Kabuli_NC_021160.1_16926191	Ca_kabuli_chr01	16926191	[T/G]	1067290688
Ca_Kabuli_NC_021160.1_16933366	Ca_kabuli_chr01	16933366	[A/T]	1067290689
Ca_Kabuli_NC_021160.1_16947273	Ca_kabuli_chr01	16947273	[T/C]	1067290690
Ca_Kabuli_NC_021160.1_16947309	Ca_kabuli_chr01	16947309	[T/G]	1067290691
Ca_Kabuli_NC_021160.1_16947321	Ca_kabuli_chr01	16947321	[T/C]	1067290692
Ca_Kabuli_NC_021160.1_16985736	Ca_kabuli_chr01	16985736	[A/C]	1067290693
Ca_Kabuli_NC_021160.1_17027471	Ca_kabuli_chr01	17027471	[T/C]	1067290694
Ca_Kabuli_NC_021160.1_17027509	Ca_kabuli_chr01	17027509	[T/C]	1067290695
Ca_Kabuli_NC_021160.1_17027523	Ca_kabuli_chr01	17027523	[A/C]	1067290696
Ca_Kabuli_NC_021160.1_17027527	Ca_kabuli_chr01	17027527	[A/G]	1067290697
Ca_Kabuli_NC_021160.1_17027531	Ca_kabuli_chr01	17027531	[A/G]	1067290698
Ca_Kabuli_NC_021160.1_17092346	Ca_kabuli_chr01	17092346	[T/G]	1067290699
Ca_Kabuli_NC_021160.1_17093633	Ca_kabuli_chr01	17093633	[G/A]	1067290700
Ca_Kabuli_NC_021160.1_17093640	Ca_kabuli_chr01	17093640	[T/G]	1067290701
Ca_Kabuli_NC_021160.1_17167022	Ca_kabuli_chr01	17167022	[T/A]	1067290702
Ca_Kabuli_NC_021160.1_17170729	Ca_kabuli_chr01	17170729	[A/C]	1067290703
Ca_Kabuli_NC_021160.1_17182813	Ca_kabuli_chr01	17182813	[A/T]	1067290704

SNP IDs	Chromosomes	Physical positions (bp)	SNPs	NCBI SNPdb accession IDs (NCBI_ss#)
Ca_Kabuli_NC_021160.1_17182831	Ca_kabuli_chr01	17182831	[A/G]	1067290705
Ca_Kabuli_NC_021160.1_17316493	Ca_kabuli_chr01	17316493	[C/A]	1067290706
Ca_Kabuli_NC_021160.1_17328742	Ca_kabuli_chr01	17328742	[T/G]	1067290707
Ca_Kabuli_NC_021160.1_17333012	Ca_kabuli_chr01	17333012	[A/C]	1067290708
Ca_Kabuli_NC_021160.1_17333045	Ca_kabuli_chr01	17333045	[A/C]	1067290709
Ca_Kabuli_NC_021160.1_17333054	Ca_kabuli_chr01	17333054	[A/G]	1067290710
Ca_Kabuli_NC_021160.1_17349988	Ca_kabuli_chr01	17349988	[T/A]	1067290711
Ca_Kabuli_NC_021160.1_17359380	Ca_kabuli_chr01	17359380	[A/G]	1067290712
Ca_Kabuli_NC_021160.1_17359685	Ca_kabuli_chr01	17359685	[T/C]	1067290713
Ca_Kabuli_NC_021160.1_17359686	Ca_kabuli_chr01	17359686	[T/C]	1067290714
Ca_Kabuli_NC_021160.1_17359714	Ca_kabuli_chr01	17359714	[A/C]	1067290715
Ca_Kabuli_NC_021160.1_17359716	Ca_kabuli_chr01	17359716	[A/C]	1067290716
Ca_Kabuli_NC_021160.1_17359743	Ca_kabuli_chr01	17359743	[A/G]	1067290717
Ca_Kabuli_NC_021160.1_17363280	Ca_kabuli_chr01	17363280	[A/C]	1067290718
Ca_Kabuli_NC_021160.1_17363352	Ca_kabuli_chr01	17363352	[T/C]	1067290719
Ca_Kabuli_NC_021160.1_17363360	Ca_kabuli_chr01	17363360	[A/G]	1067290720
Ca_Kabuli_NC_021160.1_17391874	Ca_kabuli_chr01	17391874	[T/C]	1067290721
Ca_Kabuli_NC_021160.1_17391875	Ca_kabuli_chr01	17391875	[C/A]	1067290722
Ca_Kabuli_NC_021160.1_17391876	Ca_kabuli_chr01	17391876	[T/G]	1067290723
Ca_Kabuli_NC_021160.1_17400763	Ca_kabuli_chr01	17400763	[T/A]	1067290724
Ca_Kabuli_NC_021160.1_17400780	Ca_kabuli_chr01	17400780	[A/G]	1067290725
Ca_Kabuli_NC_021160.1_17400793	Ca_kabuli_chr01	17400793	[A/G]	1067290726
Ca_Kabuli_NC_021160.1_17400854	Ca_kabuli_chr01	17400854	[T/G]	1067290727
Ca_Kabuli_NC_021160.1_17400906	Ca_kabuli_chr01	17400906	[T/C]	1067290728
Ca_Kabuli_NC_021160.1_17400915	Ca_kabuli_chr01	17400915	[A/G]	1067290729
Ca_Kabuli_NC_021160.1_17404449	Ca_kabuli_chr01	17404449	[T/C]	1067290730
Ca_Kabuli_NC_021160.1_17404456	Ca_kabuli_chr01	17404456	[T/G]	1067290731
Ca_Kabuli_NC_021160.1_17404507	Ca_kabuli_chr01	17404507	[T/A]	1067290732
Ca_Kabuli_NC_021160.1_17404509	Ca_kabuli_chr01	17404509	[A/G]	1067290733
Ca_Kabuli_NC_021160.1_17404560	Ca_kabuli_chr01	17404560	[C/T]	1067290734
Ca_Kabuli_NC_021160.1_17404569	Ca_kabuli_chr01	17404569	[T/C]	1067290735
Ca_Kabuli_NC_021160.1_17404571	Ca_kabuli_chr01	17404571	[A/G]	1067290736
Ca_Kabuli_NC_021160.1_17404587	Ca_kabuli_chr01	17404587	[T/C]	1067290737
Ca_Kabuli_NC_021160.1_17404600	Ca_kabuli_chr01	17404600	[C/A]	1067290738

SNP IDs	Chromosomes	Physical positions (bp)	SNPs	NCBI SNPdb accession IDs (NCBI_ss#)
Ca_Kabuli_NC_021160.1_17437379	Ca_kabuli_chr01	17437379	[T/G]	1067290739
Ca_Kabuli_NC_021160.1_17437391	Ca_kabuli_chr01	17437391	[T/G]	1067290740
Ca_Kabuli_NC_021160.1_17458109	Ca_kabuli_chr01	17458109	[T/G]	1067290741
Ca_Kabuli_NC_021160.1_17458129	Ca_kabuli_chr01	17458129	[T/A]	1067290742
Ca_Kabuli_NC_021160.1_17462446	Ca_kabuli_chr01	17462446	[C/T]	1067290743
Ca_Kabuli_NC_021160.1_17462461	Ca_kabuli_chr01	17462461	[G/A]	1067290744
Ca_Kabuli_NC_021160.1_17462468	Ca_kabuli_chr01	17462468	[T/C]	1067290745
Ca_Kabuli_NC_021160.1_17462494	Ca_kabuli_chr01	17462494	[A/T]	1067290746
Ca_Kabuli_NC_021160.1_17462593	Ca_kabuli_chr01	17462593	[A/G]	1067290747
Ca_Kabuli_NC_021160.1_17522357	Ca_kabuli_chr01	17522357	[G/C]	1067290748
Ca_Kabuli_NC_021160.1_17522359	Ca_kabuli_chr01	17522359	[T/C]	1067290749
Ca_Kabuli_NC_021160.1_17522489	Ca_kabuli_chr01	17522489	[T/C]	1067290750
Ca_Kabuli_NC_021160.1_17522490	Ca_kabuli_chr01	17522490	[C/T]	1067290751
Ca_Kabuli_NC_021160.1_17522492	Ca_kabuli_chr01	17522492	[C/T]	1067290752
Ca_Kabuli_NC_021160.1_17522499	Ca_kabuli_chr01	17522499	[T/C]	1067290753
Ca_Kabuli_NC_021160.1_17523917	Ca_kabuli_chr01	17523917	[G/T]	1067290754
Ca_Kabuli_NC_021160.1_17523919	Ca_kabuli_chr01	17523919	[T/C]	1067290755
Ca_Kabuli_NC_021160.1_17523921	Ca_kabuli_chr01	17523921	[C/T]	1067290756
Ca_Kabuli_NC_021160.1_17523925	Ca_kabuli_chr01	17523925	[G/C]	1067290757
Ca_Kabuli_NC_021160.1_17558878	Ca_kabuli_chr01	17558878	[A/G]	1067290758
Ca_Kabuli_NC_021160.1_17558882	Ca_kabuli_chr01	17558882	[C/A]	1067290759
Ca_Kabuli_NC_021160.1_17558883	Ca_kabuli_chr01	17558883	[T/G]	1067290760
Ca_Kabuli_NC_021160.1_17558896	Ca_kabuli_chr01	17558896	[A/G]	1067290761
Ca_Kabuli_NC_021160.1_17558960	Ca_kabuli_chr01	17558960	[A/G]	1067290762
Ca_Kabuli_NC_021160.1_17558994	Ca_kabuli_chr01	17558994	[A/C]	1067290763
Ca_Kabuli_NC_021160.1_17571776	Ca_kabuli_chr01	17571776	[T/C]	1067290764
Ca_Kabuli_NC_021160.1_17571793	Ca_kabuli_chr01	17571793	[T/G]	1067290765
Ca_Kabuli_NC_021160.1_17571806	Ca_kabuli_chr01	17571806	[A/G]	1067290766
Ca_Kabuli_NC_021160.1_17576835	Ca_kabuli_chr01	17576835	[A/C]	1067290767
Ca_Kabuli_NC_021160.1_17590658	Ca_kabuli_chr01	17590658	[A/G]	1067290768
Ca_Kabuli_NC_021160.1_17653519	Ca_kabuli_chr01	17653519	[T/C]	1067290769
Ca_Kabuli_NC_021160.1_17653537	Ca_kabuli_chr01	17653537	[A/G]	1067290770
Ca_Kabuli_NC_021160.1_17653549	Ca_kabuli_chr01	17653549	[T/G]	1067290771
Ca_Kabuli_NC_021160.1_17653552	Ca_kabuli_chr01	17653552	[T/G]	1067290772

SNP IDs	Chromosomes	Physical positions (bp)	SNPs	NCBI SNPdb accession IDs (NCBI_ss#)
Ca_Kabuli_NC_021160.1_17653553	Ca_kabuli_chr01	17653553	[A/C]	1067290773
Ca_Kabuli_NC_021160.1_17653557	Ca_kabuli_chr01	17653557	[A/C]	1067290774
Ca_Kabuli_NC_021160.1_17706867	Ca_kabuli_chr01	17706867	[T/C]	1067290775
Ca_Kabuli_NC_021160.1_17706889	Ca_kabuli_chr01	17706889	[A/G]	1067290776
Ca_Kabuli_NC_021160.1_17759272	Ca_kabuli_chr01	17759272	[A/G]	1067290777
Ca_Kabuli_NC_021160.1_17761284	Ca_kabuli_chr01	17761284	[A/G]	1067290778
Ca_Kabuli_NC_021160.1_17761393	Ca_kabuli_chr01	17761393	[A/G]	1067290779
Ca_Kabuli_NC_021160.1_17763486	Ca_kabuli_chr01	17763486	[T/G]	1067290780
Ca_Kabuli_NC_021160.1_17763492	Ca_kabuli_chr01	17763492	[T/G]	1067290781
Ca_Kabuli_NC_021160.1_17763498	Ca_kabuli_chr01	17763498	[T/C]	1067290782
Ca_Kabuli_NC_021160.1_17778537	Ca_kabuli_chr01	17778537	[T/G]	1067290783
Ca_Kabuli_NC_021160.1_17799607	Ca_kabuli_chr01	17799607	[C/A]	1067290784
Ca_Kabuli_NC_021160.1_17799615	Ca_kabuli_chr01	17799615	[A/G]	1067290785
Ca_Kabuli_NC_021160.1_17799621	Ca_kabuli_chr01	17799621	[A/G]	1067290786
Ca_Kabuli_NC_021160.1_17799635	Ca_kabuli_chr01	17799635	[A/G]	1067290787
Ca_Kabuli_NC_021160.1_17820791	Ca_kabuli_chr01	17820791	[A/G]	1067290788
Ca_Kabuli_NC_021160.1_17866910	Ca_kabuli_chr01	17866910	[T/G]	1067290789
Ca_Kabuli_NC_021160.1_17866946	Ca_kabuli_chr01	17866946	[C/G]	1067290790
Ca_Kabuli_NC_021160.1_17867617	Ca_kabuli_chr01	17867617	[A/C]	1067290791
Ca_Kabuli_NC_021160.1_17961745	Ca_kabuli_chr01	17961745	[C/A]	1067290792
Ca_Kabuli_NC_021160.1_17961746	Ca_kabuli_chr01	17961746	[A/G]	1067290793
Ca_Kabuli_NC_021160.1_17983266	Ca_kabuli_chr01	17983266	[A/G]	1067290794
Ca_Kabuli_NC_021160.1_17983298	Ca_kabuli_chr01	17983298	[T/G]	1067290795
Ca_Kabuli_NC_021160.1_17983306	Ca_kabuli_chr01	17983306	[T/G]	1067290796
Ca_Kabuli_NC_021160.1_17983310	Ca_kabuli_chr01	17983310	[T/G]	1067290797
Ca_Kabuli_NC_021160.1_18015870	Ca_kabuli_chr01	18015870	[T/G]	1067290798
Ca_Kabuli_NC_021160.1_18017331	Ca_kabuli_chr01	18017331	[A/G]	1067290799
Ca_Kabuli_NC_021160.1_18068251	Ca_kabuli_chr01	18068251	[A/C]	1067290800
Ca_Kabuli_NC_021160.1_18085174	Ca_kabuli_chr01	18085174	[T/G]	1067290801
Ca_Kabuli_NC_021160.1_18092984	Ca_kabuli_chr01	18092984	[T/C]	1067290802
Ca_Kabuli_NC_021160.1_18093009	Ca_kabuli_chr01	18093009	[T/C]	1067290803
Ca_Kabuli_NC_021160.1_18093020	Ca_kabuli_chr01	18093020	[T/G]	1067290804
Ca_Kabuli_NC_021160.1_18098525	Ca_kabuli_chr01	18098525	[T/C]	1067290805
Ca_Kabuli_NC_021160.1_18098574	Ca_kabuli_chr01	18098574	[A/T]	1067290806

SNP IDs	Chromosomes	Physical positions (bp)	SNPs	NCBI SNPdb accession IDs (NCBI_ss#)
Ca_Kabuli_NC_021160.1_18137644	Ca_kabuli_chr01	18137644	[T/C]	1067290807
Ca_Kabuli_NC_021160.1_18137673	Ca_kabuli_chr01	18137673	[A/G]	1067290808
Ca_Kabuli_NC_021160.1_18156778	Ca_kabuli_chr01	18156778	[T/C]	1067290809
Ca_Kabuli_NC_021160.1_18156837	Ca_kabuli_chr01	18156837	[A/C]	1067290810
Ca_Kabuli_NC_021160.1_18172904	Ca_kabuli_chr01	18172904	[T/C]	1067290811
Ca_Kabuli_NC_021160.1_18172944	Ca_kabuli_chr01	18172944	[T/G]	1067290812
Ca_Kabuli_NC_021160.1_18371385	Ca_kabuli_chr01	18371385	[C/A]	1067290813
Ca_Kabuli_NC_021160.1_18460647	Ca_kabuli_chr01	18460647	[A/C]	1067290814
Ca_Kabuli_NC_021160.1_18503993	Ca_kabuli_chr01	18503993	[A/C]	1067290815
Ca_Kabuli_NC_021160.1_18504001	Ca_kabuli_chr01	18504001	[A/C]	1067290816
Ca_Kabuli_NC_021160.1_18745989	Ca_kabuli_chr01	18745989	[T/G]	1067290817
Ca_Kabuli_NC_021160.1_18748594	Ca_kabuli_chr01	18748594	[C/A]	1067290818
Ca_Kabuli_NC_021160.1_18748595	Ca_kabuli_chr01	18748595	[C/G]	1067290819
Ca_Kabuli_NC_021160.1_18748596	Ca_kabuli_chr01	18748596	[G/A]	1067290820
Ca_Kabuli_NC_021160.1_18764083	Ca_kabuli_chr01	18764083	[T/A]	1067290821
Ca_Kabuli_NC_021160.1_18764113	Ca_kabuli_chr01	18764113	[A/G]	1067290822
Ca_Kabuli_NC_021160.1_18765875	Ca_kabuli_chr01	18765875	[A/G]	1067290823
Ca_Kabuli_NC_021160.1_18765881	Ca_kabuli_chr01	18765881	[T/C]	1067290824
Ca_Kabuli_NC_021160.1_18771081	Ca_kabuli_chr01	18771081	[T/G]	1067290825
Ca_Kabuli_NC_021160.1_18784103	Ca_kabuli_chr01	18784103	[A/C]	1067290826
Ca_Kabuli_NC_021160.1_18784153	Ca_kabuli_chr01	18784153	[A/G]	1067290827
Ca_Kabuli_NC_021160.1_18784172	Ca_kabuli_chr01	18784172	[A/C]	1067290828
Ca_Kabuli_NC_021160.1_18797833	Ca_kabuli_chr01	18797833	[C/A]	1067290829
Ca_Kabuli_NC_021160.1_18835334	Ca_kabuli_chr01	18835334	[T/C]	1067290830
Ca_Kabuli_NC_021160.1_19165710	Ca_kabuli_chr01	19165710	[C/A]	1067290831
Ca_Kabuli_NC_021160.1_19165714	Ca_kabuli_chr01	19165714	[T/A]	1067290832
Ca_Kabuli_NC_021160.1_19165715	Ca_kabuli_chr01	19165715	[C/A]	1067290833
Ca_Kabuli_NC_021160.1_19209701	Ca_kabuli_chr01	19209701	[T/A]	1067290834
Ca_Kabuli_NC_021160.1_19252764	Ca_kabuli_chr01	19252764	[T/G]	1067290835
Ca_Kabuli_NC_021160.1_19252846	Ca_kabuli_chr01	19252846	[A/G]	1067290836
Ca_Kabuli_NC_021160.1_19252879	Ca_kabuli_chr01	19252879	[A/G]	1067290837
Ca_Kabuli_NC_021160.1_19254161	Ca_kabuli_chr01	19254161	[A/G]	1067290838
Ca_Kabuli_NC_021160.1_19359812	Ca_kabuli_chr01	19359812	[A/C]	1067290839
Ca_Kabuli_NC_021160.1_19360048	Ca_kabuli_chr01	19360048	[A/G]	1067290840

SNP IDs	Chromosomes	Physical positions (bp)	SNPs	NCBI SNPdb accession IDs (NCBI_ss#)
Ca_Kabuli_NC_021160.1_19526652	Ca_kabuli_chr01	19526652	[G/T]	1067290841
Ca_Kabuli_NC_021160.1_19539805	Ca_kabuli_chr01	19539805	[T/G]	1067290842
Ca_Kabuli_NC_021160.1_19876064	Ca_kabuli_chr01	19876064	[T/G]	1067290843
Ca_Kabuli_NC_021160.1_19902827	Ca_kabuli_chr01	19902827	[T/A]	1067290844
Ca_Kabuli_NC_021160.1_20005201	Ca_kabuli_chr01	20005201	[T/A]	1067290845
Ca_Kabuli_NC_021160.1_20056368	Ca_kabuli_chr01	20056368	[A/C]	1067290846
Ca_Kabuli_NC_021160.1_20070148	Ca_kabuli_chr01	20070148	[A/G]	1067290847
Ca_Kabuli_NC_021160.1_20070159	Ca_kabuli_chr01	20070159	[T/G]	1067290848
Ca_Kabuli_NC_021160.1_20070197	Ca_kabuli_chr01	20070197	[T/A]	1067290849
Ca_Kabuli_NC_021160.1_20091291	Ca_kabuli_chr01	20091291	[T/G]	1067290850
Ca_Kabuli_NC_021160.1_20092567	Ca_kabuli_chr01	20092567	[T/C]	1067290851
Ca_Kabuli_NC_021160.1_20107182	Ca_kabuli_chr01	20107182	[G/A]	1067290852
Ca_Kabuli_NC_021160.1_20113713	Ca_kabuli_chr01	20113713	[T/C]	1067290853
Ca_Kabuli_NC_021160.1_21739090	Ca_kabuli_chr01	21739090	[T/C]	1067290854
Ca_Kabuli_NC_021160.1_21739123	Ca_kabuli_chr01	21739123	[A/G]	1067290855
Ca_Kabuli_NC_021160.1_21739125	Ca_kabuli_chr01	21739125	[T/C]	1067290856
Ca_Kabuli_NC_021160.1_21739126	Ca_kabuli_chr01	21739126	[G/T]	1067290857
Ca_Kabuli_NC_021160.1_21739129	Ca_kabuli_chr01	21739129	[C/T]	1067290858
Ca_Kabuli_NC_021160.1_21739425	Ca_kabuli_chr01	21739425	[A/T]	1067290859
Ca_Kabuli_NC_021160.1_21749813	Ca_kabuli_chr01	21749813	[G/A]	1067290860
Ca_Kabuli_NC_021160.1_21822951	Ca_kabuli_chr01	21822951	[A/G]	1067290861
Ca_Kabuli_NC_021160.1_21822958	Ca_kabuli_chr01	21822958	[A/G]	1067290862
Ca_Kabuli_NC_021160.1_21917899	Ca_kabuli_chr01	21917899	[A/G]	1067290863
Ca_Kabuli_NC_021160.1_22369879	Ca_kabuli_chr01	22369879	[T/C]	1067290864
Ca_Kabuli_NC_021160.1_22369882	Ca_kabuli_chr01	22369882	[A/G]	1067290865
Ca_Kabuli_NC_021160.1_22369908	Ca_kabuli_chr01	22369908	[A/T]	1067290866
Ca_Kabuli_NC_021160.1_22433014	Ca_kabuli_chr01	22433014	[T/G]	1067290867
Ca_Kabuli_NC_021160.1_22433020	Ca_kabuli_chr01	22433020	[A/C]	1067290868
Ca_Kabuli_NC_021160.1_22433050	Ca_kabuli_chr01	22433050	[A/G]	1067290869
Ca_Kabuli_NC_021160.1_22465218	Ca_kabuli_chr01	22465218	[T/C]	1067290870
Ca_Kabuli_NC_021160.1_22465224	Ca_kabuli_chr01	22465224	[A/C]	1067290871
Ca_Kabuli_NC_021160.1_22465269	Ca_kabuli_chr01	22465269	[A/G]	1067290872
Ca_Kabuli_NC_021160.1_22465279	Ca_kabuli_chr01	22465279	[T/A]	1067290873
Ca_Kabuli_NC_021160.1_22465582	Ca_kabuli_chr01	22465582	[A/T]	1067290874

SNP IDs	Chromosomes	Physical positions (bp)	SNPs	NCBI SNPdb accession IDs (NCBI_ss#)
Ca_Kabuli_NC_021160.1_22465603	Ca_kabuli_chr01	22465603	[A/C]	1067290875
Ca_Kabuli_NC_021160.1_22514655	Ca_kabuli_chr01	22514655	[G/T]	1067290876
Ca_Kabuli_NC_021160.1_22744673	Ca_kabuli_chr01	22744673	[A/C]	1067290877
Ca_Kabuli_NC_021160.1_22744691	Ca_kabuli_chr01	22744691	[A/G]	1067290878
Ca_Kabuli_NC_021160.1_23147204	Ca_kabuli_chr01	23147204	[T/C]	1067290879
Ca_Kabuli_NC_021160.1_23148736	Ca_kabuli_chr01	23148736	[A/C]	1067290880
Ca_Kabuli_NC_021160.1_23148858	Ca_kabuli_chr01	23148858	[A/G]	1067290881
Ca_Kabuli_NC_021160.1_23149053	Ca_kabuli_chr01	23149053	[G/A]	1067290882
Ca_Kabuli_NC_021160.1_23149096	Ca_kabuli_chr01	23149096	[A/G]	1067290883
Ca_Kabuli_NC_021160.1_23155387	Ca_kabuli_chr01	23155387	[T/C]	1067290884
Ca_Kabuli_NC_021160.1_23155450	Ca_kabuli_chr01	23155450	[A/G]	1067290885
Ca_Kabuli_NC_021160.1_23558999	Ca_kabuli_chr01	23558999	[A/T]	1067290886
Ca_Kabuli_NC_021160.1_23602532	Ca_kabuli_chr01	23602532	[A/C]	1067290887
Ca_Kabuli_NC_021160.1_23885558	Ca_kabuli_chr01	23885558	[T/G]	1067290888
Ca_Kabuli_NC_021160.1_23885577	Ca_kabuli_chr01	23885577	[C/G]	1067290889
Ca_Kabuli_NC_021160.1_24070339	Ca_kabuli_chr01	24070339	[T/A]	1067290890
Ca_Kabuli_NC_021160.1_24070476	Ca_kabuli_chr01	24070476	[A/G]	1067290891
Ca_Kabuli_NC_021160.1_24070485	Ca_kabuli_chr01	24070485	[A/G]	1067290892
Ca_Kabuli_NC_021160.1_24070557	Ca_kabuli_chr01	24070557	[T/C]	1067290893
Ca_Kabuli_NC_021160.1_24070595	Ca_kabuli_chr01	24070595	[T/C]	1067290894
Ca_Kabuli_NC_021160.1_24070851	Ca_kabuli_chr01	24070851	[T/C]	1067290895
Ca_Kabuli_NC_021160.1_24070917	Ca_kabuli_chr01	24070917	[A/G]	1067290896
Ca_Kabuli_NC_021160.1_24070982	Ca_kabuli_chr01	24070982	[T/C]	1067290897
Ca_Kabuli_NC_021160.1_24072521	Ca_kabuli_chr01	24072521	[T/G]	1067290898
Ca_Kabuli_NC_021160.1_24247479	Ca_kabuli_chr01	24247479	[A/C]	1067290899
Ca_Kabuli_NC_021160.1_24247497	Ca_kabuli_chr01	24247497	[T/C]	1067290900
Ca_Kabuli_NC_021160.1_24247576	Ca_kabuli_chr01	24247576	[A/T]	1067290901
Ca_Kabuli_NC_021160.1_24247596	Ca_kabuli_chr01	24247596	[T/C]	1067290902
Ca_Kabuli_NC_021160.1_24450415	Ca_kabuli_chr01	24450415	[T/C]	1067290903
Ca_Kabuli_NC_021160.1_24450483	Ca_kabuli_chr01	24450483	[T/G]	1067290904
Ca_Kabuli_NC_021160.1_24450511	Ca_kabuli_chr01	24450511	[A/G]	1067290905
Ca_Kabuli_NC_021160.1_24505912	Ca_kabuli_chr01	24505912	[T/G]	1067290906
Ca_Kabuli_NC_021160.1_24560013	Ca_kabuli_chr01	24560013	[A/C]	1067290907
Ca_Kabuli_NC_021160.1_24658542	Ca_kabuli_chr01	24658542	[T/G]	1067290908

SNP IDs	Chromosomes	Physical positions (bp)	SNPs	NCBI SNPdb accession IDs (NCBI_ss#)
Ca_Kabuli_NC_021160.1_24658546	Ca_kabuli_chr01	24658546	[G/A]	1067290909
Ca_Kabuli_NC_021160.1_24845112	Ca_kabuli_chr01	24845112	[A/C]	1067290910
Ca_Kabuli_NC_021160.1_24845135	Ca_kabuli_chr01	24845135	[T/G]	1067290911
Ca_Kabuli_NC_021160.1_24868038	Ca_kabuli_chr01	24868038	[T/A]	1067290912
Ca_Kabuli_NC_021160.1_24983868	Ca_kabuli_chr01	24983868	[A/T]	1067290913
Ca_Kabuli_NC_021160.1_24983895	Ca_kabuli_chr01	24983895	[T/C]	1067290914
Ca_Kabuli_NC_021160.1_24984524	Ca_kabuli_chr01	24984524	[T/A]	1067290915
Ca_Kabuli_NC_021160.1_24984532	Ca_kabuli_chr01	24984532	[A/T]	1067290916
Ca_Kabuli_NC_021160.1_24984538	Ca_kabuli_chr01	24984538	[A/C]	1067290917
Ca_Kabuli_NC_021160.1_24984614	Ca_kabuli_chr01	24984614	[A/G]	1067290918
Ca_Kabuli_NC_021160.1_25060354	Ca_kabuli_chr01	25060354	[A/C]	1067290919
Ca_Kabuli_NC_021160.1_25176176	Ca_kabuli_chr01	25176176	[T/C]	1067290920
Ca_Kabuli_NC_021160.1_25176210	Ca_kabuli_chr01	25176210	[A/G]	1067290921
Ca_Kabuli_NC_021160.1_25176218	Ca_kabuli_chr01	25176218	[A/G]	1067290922
Ca_Kabuli_NC_021160.1_25664814	Ca_kabuli_chr01	25664814	[T/C]	1067290923
Ca_Kabuli_NC_021160.1_25733402	Ca_kabuli_chr01	25733402	[T/C]	1067290924
Ca_Kabuli_NC_021160.1_25733416	Ca_kabuli_chr01	25733416	[T/G]	1067290925
Ca_Kabuli_NC_021160.1_25733432	Ca_kabuli_chr01	25733432	[A/G]	1067290926
Ca_Kabuli_NC_021160.1_25843908	Ca_kabuli_chr01	25843908	[A/G]	1067290927
Ca_Kabuli_NC_021160.1_25987757	Ca_kabuli_chr01	25987757	[A/T]	1067290928
Ca_Kabuli_NC_021160.1_25987803	Ca_kabuli_chr01	25987803	[A/C]	1067290929
Ca_Kabuli_NC_021160.1_26150856	Ca_kabuli_chr01	26150856	[A/C]	1067290930
Ca_Kabuli_NC_021160.1_26326715	Ca_kabuli_chr01	26326715	[T/A]	1067290931
Ca_Kabuli_NC_021160.1_26579621	Ca_kabuli_chr01	26579621	[A/C]	1067290932
Ca_Kabuli_NC_021160.1_26613090	Ca_kabuli_chr01	26613090	[T/C]	1067290933
Ca_Kabuli_NC_021160.1_26769028	Ca_kabuli_chr01	26769028	[T/G]	1067290934
Ca_Kabuli_NC_021160.1_26769098	Ca_kabuli_chr01	26769098	[A/C]	1067290935
Ca_Kabuli_NC_021160.1_26807402	Ca_kabuli_chr01	26807402	[C/A]	1067290936
Ca_Kabuli_NC_021160.1_26807403	Ca_kabuli_chr01	26807403	[T/C]	1067290937
Ca_Kabuli_NC_021160.1_26807421	Ca_kabuli_chr01	26807421	[A/C]	1067290938
Ca_Kabuli_NC_021160.1_26921411	Ca_kabuli_chr01	26921411	[A/G]	1067290939
Ca_Kabuli_NC_021160.1_26921421	Ca_kabuli_chr01	26921421	[T/C]	1067290940
Ca_Kabuli_NC_021160.1_26922011	Ca_kabuli_chr01	26922011	[T/G]	1067290941
Ca_Kabuli_NC_021160.1_26922027	Ca_kabuli_chr01	26922027	[T/G]	1067290942

SNP IDs	Chromosomes	Physical positions (bp)	SNPs	NCBI SNPdb accession IDs (NCBI_ss#)
Ca_Kabuli_NC_021160.1_26922042	Ca_kabuli_chr01	26922042	[A/C]	1067290943
Ca_Kabuli_NC_021160.1_26950435	Ca_kabuli_chr01	26950435	[A/C]	1067290944
Ca_Kabuli_NC_021160.1_26974025	Ca_kabuli_chr01	26974025	[T/G]	1067290945
Ca_Kabuli_NC_021160.1_26974090	Ca_kabuli_chr01	26974090	[A/G]	1067290946
Ca_Kabuli_NC_021160.1_27012643	Ca_kabuli_chr01	27012643	[A/T]	1067290947
Ca_Kabuli_NC_021160.1_27012644	Ca_kabuli_chr01	27012644	[A/C]	1067290948
Ca_Kabuli_NC_021160.1_27012645	Ca_kabuli_chr01	27012645	[G/T]	1067290949
Ca_Kabuli_NC_021160.1_27017559	Ca_kabuli_chr01	27017559	[G/A]	1067290950
Ca_Kabuli_NC_021160.1_27017560	Ca_kabuli_chr01	27017560	[A/G]	1067290951
Ca_Kabuli_NC_021160.1_27017570	Ca_kabuli_chr01	27017570	[A/G]	1067290952
Ca_Kabuli_NC_021160.1_27017606	Ca_kabuli_chr01	27017606	[C/T]	1067290953
Ca_Kabuli_NC_021160.1_27252494	Ca_kabuli_chr01	27252494	[T/C]	1067290954
Ca_Kabuli_NC_021160.1_27252495	Ca_kabuli_chr01	27252495	[A/T]	1067290955
Ca_Kabuli_NC_021160.1_27618900	Ca_kabuli_chr01	27618900	[T/G]	1067290956
Ca_Kabuli_NC_021160.1_27745471	Ca_kabuli_chr01	27745471	[C/T]	1067290957
Ca_Kabuli_NC_021160.1_27831051	Ca_kabuli_chr01	27831051	[G/T]	1067290958
Ca_Kabuli_NC_021160.1_27831071	Ca_kabuli_chr01	27831071	[G/A]	1067290959
Ca_Kabuli_NC_021160.1_28065106	Ca_kabuli_chr01	28065106	[A/T]	1067290960
Ca_Kabuli_NC_021160.1_28065140	Ca_kabuli_chr01	28065140	[C/T]	1067290961
Ca_Kabuli_NC_021160.1_28073495	Ca_kabuli_chr01	28073495	[A/G]	1067290962
Ca_Kabuli_NC_021160.1_28074557	Ca_kabuli_chr01	28074557	[T/A]	1067290963
Ca_Kabuli_NC_021160.1_28082758	Ca_kabuli_chr01	28082758	[C/T]	1067290964
Ca_Kabuli_NC_021160.1_28088369	Ca_kabuli_chr01	28088369	[C/A]	1067290965
Ca_Kabuli_NC_021160.1_28088501	Ca_kabuli_chr01	28088501	[T/C]	1067290966
Ca_Kabuli_NC_021160.1_28207010	Ca_kabuli_chr01	28207010	[A/T]	1067290967
Ca_Kabuli_NC_021160.1_28208813	Ca_kabuli_chr01	28208813	[T/C]	1067290968
Ca_Kabuli_NC_021160.1_28209985	Ca_kabuli_chr01	28209985	[A/G]	1067290969
Ca_Kabuli_NC_021160.1_28343923	Ca_kabuli_chr01	28343923	[T/G]	1067290970
Ca_Kabuli_NC_021160.1_28377349	Ca_kabuli_chr01	28377349	[C/A]	1067290971
Ca_Kabuli_NC_021160.1_28379428	Ca_kabuli_chr01	28379428	[A/C]	1067290972
Ca_Kabuli_NC_021160.1_28379447	Ca_kabuli_chr01	28379447	[T/A]	1067290973
Ca_Kabuli_NC_021160.1_28404490	Ca_kabuli_chr01	28404490	[A/T]	1067290974
Ca_Kabuli_NC_021160.1_28404500	Ca_kabuli_chr01	28404500	[T/C]	1067290975
Ca_Kabuli_NC_021160.1_28404515	Ca_kabuli_chr01	28404515	[A/C]	1067290976

SNP IDs	Chromosomes	Physical positions (bp)	SNPs	NCBI SNPdb accession IDs (NCBI_ss#)
Ca_Kabuli_NC_021160.1_28404524	Ca_kabuli_chr01	28404524	[A/C]	1067290977
Ca_Kabuli_NC_021160.1_28472701	Ca_kabuli_chr01	28472701	[T/G]	1067290978
Ca_Kabuli_NC_021160.1_28472705	Ca_kabuli_chr01	28472705	[G/A]	1067290979
Ca_Kabuli_NC_021160.1_28473940	Ca_kabuli_chr01	28473940	[T/C]	1067290980
Ca_Kabuli_NC_021160.1_28656143	Ca_kabuli_chr01	28656143	[T/G]	1067290981
Ca_Kabuli_NC_021160.1_28656167	Ca_kabuli_chr01	28656167	[C/G]	1067290982
Ca_Kabuli_NC_021160.1_28656179	Ca_kabuli_chr01	28656179	[A/G]	1067290983
Ca_Kabuli_NC_021160.1_28684536	Ca_kabuli_chr01	28684536	[A/C]	1067290984
Ca_Kabuli_NC_021160.1_28684574	Ca_kabuli_chr01	28684574	[A/C]	1067290985
Ca_Kabuli_NC_021160.1_28684627	Ca_kabuli_chr01	28684627	[T/G]	1067290986
Ca_Kabuli_NC_021160.1_28684637	Ca_kabuli_chr01	28684637	[A/G]	1067290987
Ca_Kabuli_NC_021160.1_28788675	Ca_kabuli_chr01	28788675	[T/G]	1067290988
Ca_Kabuli_NC_021160.1_28790140	Ca_kabuli_chr01	28790140	[A/C]	1067290989
Ca_Kabuli_NC_021160.1_28832901	Ca_kabuli_chr01	28832901	[A/C]	1067290990
Ca_Kabuli_NC_021160.1_28849194	Ca_kabuli_chr01	28849194	[T/C]	1067290991
Ca_Kabuli_NC_021160.1_29056015	Ca_kabuli_chr01	29056015	[A/G]	1067290992
Ca_Kabuli_NC_021160.1_29138250	Ca_kabuli_chr01	29138250	[A/G]	1067290993
Ca_Kabuli_NC_021160.1_29171283	Ca_kabuli_chr01	29171283	[T/G]	1067290994
Ca_Kabuli_NC_021160.1_29171293	Ca_kabuli_chr01	29171293	[A/G]	1067290995
Ca_Kabuli_NC_021160.1_29215364	Ca_kabuli_chr01	29215364	[C/A]	1067290996
Ca_Kabuli_NC_021160.1_30226559	Ca_kabuli_chr01	30226559	[T/C]	1067290997
Ca_Kabuli_NC_021160.1_30227916	Ca_kabuli_chr01	30227916	[T/G]	1067290998
Ca_Kabuli_NC_021160.1_30227929	Ca_kabuli_chr01	30227929	[A/G]	1067290999
Ca_Kabuli_NC_021160.1_30232233	Ca_kabuli_chr01	30232233	[T/C]	1067291000
Ca_Kabuli_NC_021160.1_30637112	Ca_kabuli_chr01	30637112	[A/T]	1067291001
Ca_Kabuli_NC_021160.1_30641066	Ca_kabuli_chr01	30641066	[A/C]	1067291002
Ca_Kabuli_NC_021160.1_30990317	Ca_kabuli_chr01	30990317	[G/A]	1067291003
Ca_Kabuli_NC_021160.1_31091042	Ca_kabuli_chr01	31091042	[A/G]	1067291004
Ca_Kabuli_NC_021160.1_31091093	Ca_kabuli_chr01	31091093	[A/G]	1067291005
Ca_Kabuli_NC_021160.1_31091097	Ca_kabuli_chr01	31091097	[C/G]	1067291006
Ca_Kabuli_NC_021160.1_31136007	Ca_kabuli_chr01	31136007	[C/T]	1067291007
Ca_Kabuli_NC_021160.1_31393408	Ca_kabuli_chr01	31393408	[A/T]	1067291008
Ca_Kabuli_NC_021160.1_31528988	Ca_kabuli_chr01	31528988	[C/A]	1067291009
Ca_Kabuli_NC_021160.1_31528989	Ca_kabuli_chr01	31528989	[T/G]	1067291010

SNP IDs	Chromosomes	Physical positions (bp)	SNPs	NCBI SNPdb accession IDs (NCBI_ss#)
Ca_Kabuli_NC_021160.1_31528990	Ca_kabuli_chr01	31528990	[T/A]	1067291011
Ca_Kabuli_NC_021160.1_31528991	Ca_kabuli_chr01	31528991	[G/T]	1067291012
Ca_Kabuli_NC_021160.1_31535245	Ca_kabuli_chr01	31535245	[T/A]	1067291013
Ca_Kabuli_NC_021160.1_31535330	Ca_kabuli_chr01	31535330	[T/G]	1067291014
Ca_Kabuli_NC_021160.1_31535331	Ca_kabuli_chr01	31535331	[A/T]	1067291015
Ca_Kabuli_NC_021160.1_31535332	Ca_kabuli_chr01	31535332	[A/T]	1067291016
Ca_Kabuli_NC_021160.1_31590263	Ca_kabuli_chr01	31590263	[C/G]	1067291017
Ca_Kabuli_NC_021160.1_31640687	Ca_kabuli_chr01	31640687	[T/C]	1067291018
Ca_Kabuli_NC_021160.1_32081147	Ca_kabuli_chr01	32081147	[C/A]	1067291019
Ca_Kabuli_NC_021160.1_32280752	Ca_kabuli_chr01	32280752	[A/T]	1067291020
Ca_Kabuli_NC_021160.1_32286505	Ca_kabuli_chr01	32286505	[G/A]	1067291021
Ca_Kabuli_NC_021160.1_32286506	Ca_kabuli_chr01	32286506	[A/C]	1067291022
Ca_Kabuli_NC_021160.1_32467929	Ca_kabuli_chr01	32467929	[G/A]	1067291023
Ca_Kabuli_NC_021160.1_32467954	Ca_kabuli_chr01	32467954	[A/C]	1067291024
Ca_Kabuli_NC_021160.1_33186572	Ca_kabuli_chr01	33186572	[A/G]	1067291025
Ca_Kabuli_NC_021160.1_33186629	Ca_kabuli_chr01	33186629	[T/C]	1067291026
Ca_Kabuli_NC_021160.1_33372256	Ca_kabuli_chr01	33372256	[C/T]	1067291027
Ca_Kabuli_NC_021160.1_33695857	Ca_kabuli_chr01	33695857	[A/G]	1067291028
Ca_Kabuli_NC_021160.1_33696064	Ca_kabuli_chr01	33696064	[T/A]	1067291029
Ca_Kabuli_NC_021160.1_33696065	Ca_kabuli_chr01	33696065	[C/T]	1067291030
Ca_Kabuli_NC_021160.1_33696066	Ca_kabuli_chr01	33696066	[T/G]	1067291031
Ca_Kabuli_NC_021160.1_34161921	Ca_kabuli_chr01	34161921	[G/A]	1067291032
Ca_Kabuli_NC_021160.1_34272001	Ca_kabuli_chr01	34272001	[C/G]	1067291033
Ca_Kabuli_NC_021160.1_34272024	Ca_kabuli_chr01	34272024	[A/G]	1067291034
Ca_Kabuli_NC_021160.1_34272025	Ca_kabuli_chr01	34272025	[A/G]	1067291035
Ca_Kabuli_NC_021160.1_34309435	Ca_kabuli_chr01	34309435	[A/G]	1067291036
Ca_Kabuli_NC_021160.1_34324590	Ca_kabuli_chr01	34324590	[A/T]	1067291037
Ca_Kabuli_NC_021160.1_34324635	Ca_kabuli_chr01	34324635	[A/T]	1067291038
Ca_Kabuli_NC_021160.1_34324641	Ca_kabuli_chr01	34324641	[A/G]	1067291039
Ca_Kabuli_NC_021160.1_34355775	Ca_kabuli_chr01	34355775	[T/G]	1067291040
Ca_Kabuli_NC_021160.1_34403069	Ca_kabuli_chr01	34403069	[T/C]	1067291041
Ca_Kabuli_NC_021160.1_34476088	Ca_kabuli_chr01	34476088	[T/C]	1067291042
Ca_Kabuli_NC_021160.1_34476185	Ca_kabuli_chr01	34476185	[T/A]	1067291043
Ca_Kabuli_NC_021160.1_34513857	Ca_kabuli_chr01	34513857	[T/C]	1067291044

SNP IDs	Chromosomes	Physical positions (bp)	SNPs	NCBI SNPdb accession IDs (NCBI_ss#)
Ca_Kabuli_NC_021160.1_34727251	Ca_kabuli_chr01	34727251	[T/G]	1067291045
Ca_Kabuli_NC_021160.1_34727756	Ca_kabuli_chr01	34727756	[T/C]	1067291046
Ca_Kabuli_NC_021160.1_34867480	Ca_kabuli_chr01	34867480	[T/G]	1067291047
Ca_Kabuli_NC_021160.1_34911011	Ca_kabuli_chr01	34911011	[T/C]	1067291048
Ca_Kabuli_NC_021160.1_34911033	Ca_kabuli_chr01	34911033	[T/G]	1067291049
Ca_Kabuli_NC_021160.1_34949071	Ca_kabuli_chr01	34949071	[T/C]	1067291050
Ca_Kabuli_NC_021160.1_34950909	Ca_kabuli_chr01	34950909	[T/G]	1067291051
Ca_Kabuli_NC_021160.1_34975381	Ca_kabuli_chr01	34975381	[A/G]	1067291052
Ca_Kabuli_NC_021160.1_34975385	Ca_kabuli_chr01	34975385	[T/C]	1067291053
Ca_Kabuli_NC_021160.1_34975398	Ca_kabuli_chr01	34975398	[C/T]	1067291054
Ca_Kabuli_NC_021160.1_34979124	Ca_kabuli_chr01	34979124	[T/G]	1067291055
Ca_Kabuli_NC_021160.1_35041275	Ca_kabuli_chr01	35041275	[C/T]	1067291056
Ca_Kabuli_NC_021160.1_35041283	Ca_kabuli_chr01	35041283	[T/C]	1067291057
Ca_Kabuli_NC_021160.1_35260260	Ca_kabuli_chr01	35260260	[A/G]	1067291058
Ca_Kabuli_NC_021160.1_35260363	Ca_kabuli_chr01	35260363	[G/A]	1067291059
Ca_Kabuli_NC_021160.1_35260396	Ca_kabuli_chr01	35260396	[T/G]	1067291060
Ca_Kabuli_NC_021160.1_35708846	Ca_kabuli_chr01	35708846	[T/C]	1067291061
Ca_Kabuli_NC_021160.1_35708882	Ca_kabuli_chr01	35708882	[A/C]	1067291062
Ca_Kabuli_NC_021160.1_35708891	Ca_kabuli_chr01	35708891	[A/T]	1067291063
Ca_Kabuli_NC_021160.1_36094441	Ca_kabuli_chr01	36094441	[T/C]	1067291064
Ca_Kabuli_NC_021160.1_36362260	Ca_kabuli_chr01	36362260	[T/A]	1067291065
Ca_Kabuli_NC_021160.1_36362281	Ca_kabuli_chr01	36362281	[T/C]	1067291066
Ca_Kabuli_NC_021160.1_36362302	Ca_kabuli_chr01	36362302	[T/C]	1067291067
Ca_Kabuli_NC_021160.1_36362304	Ca_kabuli_chr01	36362304	[G/T]	1067291068
Ca_Kabuli_NC_021160.1_36362341	Ca_kabuli_chr01	36362341	[T/C]	1067291069
Ca_Kabuli_NC_021160.1_36408325	Ca_kabuli_chr01	36408325	[T/A]	1067291070
Ca_Kabuli_NC_021160.1_36408358	Ca_kabuli_chr01	36408358	[A/C]	1067291071
Ca_Kabuli_NC_021160.1_36633911	Ca_kabuli_chr01	36633911	[A/G]	1067291072
Ca_Kabuli_NC_021160.1_36633917	Ca_kabuli_chr01	36633917	[A/G]	1067291073
Ca_Kabuli_NC_021160.1_36633956	Ca_kabuli_chr01	36633956	[A/G]	1067291074
Ca_Kabuli_NC_021160.1_36679586	Ca_kabuli_chr01	36679586	[A/G]	1067291075
Ca_Kabuli_NC_021160.1_37355995	Ca_kabuli_chr01	37355995	[T/G]	1067291076
Ca_Kabuli_NC_021160.1_37356085	Ca_kabuli_chr01	37356085	[T/C]	1067291077
Ca_Kabuli_NC_021160.1_37356089	Ca_kabuli_chr01	37356089	[T/G]	1067291078

SNP IDs	Chromosomes	Physical positions (bp)	SNPs	NCBI SNPdb accession IDs (NCBI_ss#)
Ca_Kabuli_NC_021160.1_37356115	Ca_kabuli_chr01	37356115	[T/C]	1067291079
Ca_Kabuli_NC_021160.1_37608107	Ca_kabuli_chr01	37608107	[T/A]	1067291080
Ca_Kabuli_NC_021160.1_37608120	Ca_kabuli_chr01	37608120	[A/G]	1067291081
Ca_Kabuli_NC_021160.1_37648716	Ca_kabuli_chr01	37648716	[A/C]	1067291082
Ca_Kabuli_NC_021160.1_37648729	Ca_kabuli_chr01	37648729	[A/G]	1067291083
Ca_Kabuli_NC_021160.1_37654055	Ca_kabuli_chr01	37654055	[T/C]	1067291084
Ca_Kabuli_NC_021160.1_37658162	Ca_kabuli_chr01	37658162	[G/C]	1067291085
Ca_Kabuli_NC_021160.1_37658166	Ca_kabuli_chr01	37658166	[A/T]	1067291086
Ca_Kabuli_NC_021160.1_37690416	Ca_kabuli_chr01	37690416	[G/A]	1067291087
Ca_Kabuli_NC_021160.1_37690465	Ca_kabuli_chr01	37690465	[T/C]	1067291088
Ca_Kabuli_NC_021160.1_37690497	Ca_kabuli_chr01	37690497	[A/T]	1067291089
Ca_Kabuli_NC_021160.1_37690498	Ca_kabuli_chr01	37690498	[C/A]	1067291090
Ca_Kabuli_NC_021160.1_37722095	Ca_kabuli_chr01	37722095	[G/A]	1067291091
Ca_Kabuli_NC_021160.1_37722143	Ca_kabuli_chr01	37722143	[G/C]	1067291092
Ca_Kabuli_NC_021160.1_37732133	Ca_kabuli_chr01	37732133	[C/T]	1067291093
Ca_Kabuli_NC_021160.1_37732150	Ca_kabuli_chr01	37732150	[T/C]	1067291094
Ca_Kabuli_NC_021160.1_37768299	Ca_kabuli_chr01	37768299	[A/G]	1067291095
Ca_Kabuli_NC_021160.1_37768333	Ca_kabuli_chr01	37768333	[C/T]	1067291096
Ca_Kabuli_NC_021160.1_37771605	Ca_kabuli_chr01	37771605	[A/G]	1067291097
Ca_Kabuli_NC_021160.1_37792731	Ca_kabuli_chr01	37792731	[G/A]	1067291098
Ca_Kabuli_NC_021160.1_37862342	Ca_kabuli_chr01	37862342	[T/G]	1067291099
Ca_Kabuli_NC_021160.1_37862352	Ca_kabuli_chr01	37862352	[T/G]	1067291100
Ca_Kabuli_NC_021160.1_37862383	Ca_kabuli_chr01	37862383	[A/T]	1067291101
Ca_Kabuli_NC_021160.1_37863217	Ca_kabuli_chr01	37863217	[G/T]	1067291102
Ca_Kabuli_NC_021160.1_37864681	Ca_kabuli_chr01	37864681	[A/G]	1067291103
Ca_Kabuli_NC_021160.1_37864684	Ca_kabuli_chr01	37864684	[T/C]	1067291104
Ca_Kabuli_NC_021160.1_37869004	Ca_kabuli_chr01	37869004	[G/C]	1067291105
Ca_Kabuli_NC_021160.1_37869082	Ca_kabuli_chr01	37869082	[C/G]	1067291106
Ca_Kabuli_NC_021160.1_37869136	Ca_kabuli_chr01	37869136	[G/T]	1067291107
Ca_Kabuli_NC_021160.1_37870666	Ca_kabuli_chr01	37870666	[T/C]	1067291108
Ca_Kabuli_NC_021160.1_37870835	Ca_kabuli_chr01	37870835	[G/A]	1067291109
Ca_Kabuli_NC_021160.1_37892139	Ca_kabuli_chr01	37892139	[T/A]	1067291110
Ca_Kabuli_NC_021160.1_38111568	Ca_kabuli_chr01	38111568	[A/G]	1067291111
Ca_Kabuli_NC_021160.1_38193253	Ca_kabuli_chr01	38193253	[A/T]	1067291112

SNP IDs	Chromosomes	Physical positions (bp)	SNPs	NCBI SNPdb accession IDs (NCBI_ss#)
Ca_Kabuli_NC_021160.1_38252610	Ca_kabuli_chr01	38252610	[T/G]	1067291113
Ca_Kabuli_NC_021160.1_38440513	Ca_kabuli_chr01	38440513	[A/G]	1067291114
Ca_Kabuli_NC_021160.1_38451588	Ca_kabuli_chr01	38451588	[A/C]	1067291115
Ca_Kabuli_NC_021160.1_38508170	Ca_kabuli_chr01	38508170	[T/C]	1067291116
Ca_Kabuli_NC_021160.1_38508171	Ca_kabuli_chr01	38508171	[G/T]	1067291117
Ca_Kabuli_NC_021160.1_38508280	Ca_kabuli_chr01	38508280	[A/G]	1067291118
Ca_Kabuli_NC_021160.1_38508295	Ca_kabuli_chr01	38508295	[A/G]	1067291119
Ca_Kabuli_NC_021160.1_38596501	Ca_kabuli_chr01	38596501	[T/C]	1067291120
Ca_Kabuli_NC_021160.1_38678724	Ca_kabuli_chr01	38678724	[T/C]	1067291121
Ca_Kabuli_NC_021160.1_38811851	Ca_kabuli_chr01	38811851	[C/A]	1067291122
Ca_Kabuli_NC_021160.1_38811939	Ca_kabuli_chr01	38811939	[A/G]	1067291123
Ca_Kabuli_NC_021160.1_38811946	Ca_kabuli_chr01	38811946	[C/A]	1067291124
Ca_Kabuli_NC_021160.1_38811964	Ca_kabuli_chr01	38811964	[C/T]	1067291125
Ca_Kabuli_NC_021160.1_38811965	Ca_kabuli_chr01	38811965	[A/G]	1067291126
Ca_Kabuli_NC_021160.1_38817471	Ca_kabuli_chr01	38817471	[A/C]	1067291127
Ca_Kabuli_NC_021160.1_38877670	Ca_kabuli_chr01	38877670	[T/C]	1067291128
Ca_Kabuli_NC_021160.1_38877710	Ca_kabuli_chr01	38877710	[T/A]	1067291129
Ca_Kabuli_NC_021160.1_38877711	Ca_kabuli_chr01	38877711	[A/T]	1067291130
Ca_Kabuli_NC_021160.1_38884980	Ca_kabuli_chr01	38884980	[G/C]	1067291131
Ca_Kabuli_NC_021160.1_39107150	Ca_kabuli_chr01	39107150	[G/C]	1067291132
Ca_Kabuli_NC_021160.1_39436182	Ca_kabuli_chr01	39436182	[T/C]	1067291133
Ca_Kabuli_NC_021160.1_39439352	Ca_kabuli_chr01	39439352	[T/A]	1067291134
Ca_Kabuli_NC_021160.1_39439823	Ca_kabuli_chr01	39439823	[T/C]	1067291135
Ca_Kabuli_NC_021160.1_39587402	Ca_kabuli_chr01	39587402	[G/A]	1067291136
Ca_Kabuli_NC_021160.1_39587534	Ca_kabuli_chr01	39587534	[A/G]	1067291137
Ca_Kabuli_NC_021160.1_39616074	Ca_kabuli_chr01	39616074	[T/A]	1067291138
Ca_Kabuli_NC_021160.1_39616101	Ca_kabuli_chr01	39616101	[T/G]	1067291139
Ca_Kabuli_NC_021160.1_39616113	Ca_kabuli_chr01	39616113	[A/G]	1067291140
Ca_Kabuli_NC_021160.1_39616118	Ca_kabuli_chr01	39616118	[T/G]	1067291141
Ca_Kabuli_NC_021160.1_39663380	Ca_kabuli_chr01	39663380	[C/T]	1067291142
Ca_Kabuli_NC_021160.1_39663432	Ca_kabuli_chr01	39663432	[C/G]	1067291143
Ca_Kabuli_NC_021160.1_39663435	Ca_kabuli_chr01	39663435	[T/C]	1067291144
Ca_Kabuli_NC_021160.1_39663465	Ca_kabuli_chr01	39663465	[T/A]	1067291145
Ca_Kabuli_NC_021160.1_39663484	Ca_kabuli_chr01	39663484	[A/C]	1067291146

SNP IDs	Chromosomes	Physical positions (bp)	SNPs	NCBI SNPdb accession IDs (NCBI_ss#)
Ca_Kabuli_NC_021160.1_39663824	Ca_kabuli_chr01	39663824	[G/C]	1067291147
Ca_Kabuli_NC_021160.1_39720383	Ca_kabuli_chr01	39720383	[A/T]	1067291148
Ca_Kabuli_NC_021160.1_39746552	Ca_kabuli_chr01	39746552	[A/G]	1067291149
Ca_Kabuli_NC_021160.1_40025764	Ca_kabuli_chr01	40025764	[G/A]	1067291150
Ca_Kabuli_NC_021160.1_40136941	Ca_kabuli_chr01	40136941	[T/G]	1067291151
Ca_Kabuli_NC_021160.1_40137856	Ca_kabuli_chr01	40137856	[A/T]	1067291152
Ca_Kabuli_NC_021160.1_40137893	Ca_kabuli_chr01	40137893	[T/G]	1067291153
Ca_Kabuli_NC_021160.1_40137902	Ca_kabuli_chr01	40137902	[A/G]	1067291154
Ca_Kabuli_NC_021160.1_40199841	Ca_kabuli_chr01	40199841	[T/A]	1067291155
Ca_Kabuli_NC_021160.1_40199917	Ca_kabuli_chr01	40199917	[T/C]	1067291156
Ca_Kabuli_NC_021160.1_40199920	Ca_kabuli_chr01	40199920	[C/G]	1067291157
Ca_Kabuli_NC_021160.1_40199922	Ca_kabuli_chr01	40199922	[C/A]	1067291158
Ca_Kabuli_NC_021160.1_40214167	Ca_kabuli_chr01	40214167	[C/T]	1067291159
Ca_Kabuli_NC_021160.1_40224804	Ca_kabuli_chr01	40224804	[A/G]	1067291160
Ca_Kabuli_NC_021160.1_40224834	Ca_kabuli_chr01	40224834	[C/G]	1067291161
Ca_Kabuli_NC_021160.1_40224835	Ca_kabuli_chr01	40224835	[T/A]	1067291162
Ca_Kabuli_NC_021160.1_40335668	Ca_kabuli_chr01	40335668	[C/T]	1067291163
Ca_Kabuli_NC_021160.1_40336759	Ca_kabuli_chr01	40336759	[C/T]	1067291164
Ca_Kabuli_NC_021160.1_40339065	Ca_kabuli_chr01	40339065	[T/C]	1067291165
Ca_Kabuli_NC_021160.1_40343216	Ca_kabuli_chr01	40343216	[G/T]	1067291166
Ca_Kabuli_NC_021160.1_40346280	Ca_kabuli_chr01	40346280	[A/C]	1067291167
Ca_Kabuli_NC_021160.1_40358747	Ca_kabuli_chr01	40358747	[T/G]	1067291168
Ca_Kabuli_NC_021160.1_40359876	Ca_kabuli_chr01	40359876	[T/A]	1067291169
Ca_Kabuli_NC_021160.1_40365171	Ca_kabuli_chr01	40365171	[G/A]	1067291170
Ca_Kabuli_NC_021160.1_40365226	Ca_kabuli_chr01	40365226	[G/T]	1067291171
Ca_Kabuli_NC_021160.1_40365231	Ca_kabuli_chr01	40365231	[C/A]	1067291172
Ca_Kabuli_NC_021160.1_40367634	Ca_kabuli_chr01	40367634	[T/C]	1067291173
Ca_Kabuli_NC_021160.1_40367940	Ca_kabuli_chr01	40367940	[G/A]	1067291174
Ca_Kabuli_NC_021160.1_40368805	Ca_kabuli_chr01	40368805	[A/T]	1067291175
Ca_Kabuli_NC_021160.1_40368847	Ca_kabuli_chr01	40368847	[T/C]	1067291176
Ca_Kabuli_NC_021160.1_40419760	Ca_kabuli_chr01	40419760	[C/T]	1067291177
Ca_Kabuli_NC_021160.1_40497408	Ca_kabuli_chr01	40497408	[T/C]	1067291178
Ca_Kabuli_NC_021160.1_40497413	Ca_kabuli_chr01	40497413	[A/C]	1067291179
Ca_Kabuli_NC_021160.1_40567085	Ca_kabuli_chr01	40567085	[T/C]	1067291180

SNP IDs	Chromosomes	Physical positions (bp)	SNPs	NCBI SNPdb accession IDs (NCBI_ss#)
Ca_Kabuli_NC_021160.1_40570753	Ca_kabuli_chr01	40570753	[A/C]	1067291181
Ca_Kabuli_NC_021160.1_40570789	Ca_kabuli_chr01	40570789	[A/G]	1067291182
Ca_Kabuli_NC_021160.1_40572132	Ca_kabuli_chr01	40572132	[T/C]	1067291183
Ca_Kabuli_NC_021160.1_40617637	Ca_kabuli_chr01	40617637	[A/C]	1067291184
Ca_Kabuli_NC_021160.1_40745827	Ca_kabuli_chr01	40745827	[G/C]	1067291185
Ca_Kabuli_NC_021160.1_40746094	Ca_kabuli_chr01	40746094	[T/G]	1067291186
Ca_Kabuli_NC_021160.1_41075343	Ca_kabuli_chr01	41075343	[T/G]	1067291187
Ca_Kabuli_NC_021160.1_41125525	Ca_kabuli_chr01	41125525	[T/G]	1067291188
Ca_Kabuli_NC_021160.1_41169162	Ca_kabuli_chr01	41169162	[G/A]	1067291189
Ca_Kabuli_NC_021160.1_41179697	Ca_kabuli_chr01	41179697	[T/G]	1067291190
Ca_Kabuli_NC_021160.1_41185412	Ca_kabuli_chr01	41185412	[A/C]	1067291191
Ca_Kabuli_NC_021160.1_41339399	Ca_kabuli_chr01	41339399	[T/G]	1067291192
Ca_Kabuli_NC_021160.1_41344968	Ca_kabuli_chr01	41344968	[A/G]	1067291193
Ca_Kabuli_NC_021160.1_41437183	Ca_kabuli_chr01	41437183	[A/C]	1067291194
Ca_Kabuli_NC_021160.1_41437428	Ca_kabuli_chr01	41437428	[G/C]	1067291195
Ca_Kabuli_NC_021160.1_41437429	Ca_kabuli_chr01	41437429	[C/G]	1067291196
Ca_Kabuli_NC_021160.1_41437430	Ca_kabuli_chr01	41437430	[A/C]	1067291197
Ca_Kabuli_NC_021160.1_41463514	Ca_kabuli_chr01	41463514	[T/C]	1067291198
Ca_Kabuli_NC_021160.1_41463532	Ca_kabuli_chr01	41463532	[A/G]	1067291199
Ca_Kabuli_NC_021160.1_41730114	Ca_kabuli_chr01	41730114	[A/C]	1067291200
Ca_Kabuli_NC_021160.1_41777827	Ca_kabuli_chr01	41777827	[T/G]	1067291201
Ca_Kabuli_NC_021160.1_41782803	Ca_kabuli_chr01	41782803	[T/G]	1067291202
Ca_Kabuli_NC_021160.1_41795347	Ca_kabuli_chr01	41795347	[A/G]	1067291203
Ca_Kabuli_NC_021160.1_41795349	Ca_kabuli_chr01	41795349	[T/G]	1067291204
Ca_Kabuli_NC_021160.1_41822418	Ca_kabuli_chr01	41822418	[A/C]	1067291205
Ca_Kabuli_NC_021160.1_41866970	Ca_kabuli_chr01	41866970	[T/C]	1067291206
Ca_Kabuli_NC_021160.1_41867002	Ca_kabuli_chr01	41867002	[T/G]	1067291207
Ca_Kabuli_NC_021160.1_41897246	Ca_kabuli_chr01	41897246	[C/T]	1067291208
Ca_Kabuli_NC_021160.1_41927994	Ca_kabuli_chr01	41927994	[T/C]	1067291209
Ca_Kabuli_NC_021160.1_41966344	Ca_kabuli_chr01	41966344	[T/C]	1067291210
Ca_Kabuli_NC_021160.1_41981278	Ca_kabuli_chr01	41981278	[C/T]	1067291211
Ca_Kabuli_NC_021160.1_41981286	Ca_kabuli_chr01	41981286	[G/C]	1067291212
Ca_Kabuli_NC_021160.1_42189352	Ca_kabuli_chr01	42189352	[T/G]	1067291213
Ca_Kabuli_NC_021160.1_42253413	Ca_kabuli_chr01	42253413	[A/C]	1067291214

SNP IDs	Chromosomes	Physical positions (bp)	SNPs	NCBI SNPdb accession IDs (NCBI_ss#)
Ca_Kabuli_NC_021160.1_42271245	Ca_kabuli_chr01	42271245	[C/G]	1067291215
Ca_Kabuli_NC_021160.1_42298514	Ca_kabuli_chr01	42298514	[T/G]	1067291216
Ca_Kabuli_NC_021160.1_42298524	Ca_kabuli_chr01	42298524	[T/C]	1067291217
Ca_Kabuli_NC_021160.1_42298531	Ca_kabuli_chr01	42298531	[G/A]	1067291218
Ca_Kabuli_NC_021160.1_42298544	Ca_kabuli_chr01	42298544	[A/G]	1067291219
Ca_Kabuli_NC_021160.1_42439795	Ca_kabuli_chr01	42439795	[A/C]	1067291220
Ca_Kabuli_NC_021160.1_42450096	Ca_kabuli_chr01	42450096	[T/G]	1067291221
Ca_Kabuli_NC_021160.1_42450112	Ca_kabuli_chr01	42450112	[T/A]	1067291222
Ca_Kabuli_NC_021160.1_42450120	Ca_kabuli_chr01	42450120	[T/C]	1067291223
Ca_Kabuli_NC_021160.1_42450147	Ca_kabuli_chr01	42450147	[T/C]	1067291224
Ca_Kabuli_NC_021160.1_42450658	Ca_kabuli_chr01	42450658	[T/G]	1067291225
Ca_Kabuli_NC_021160.1_42450714	Ca_kabuli_chr01	42450714	[T/G]	1067291226
Ca_Kabuli_NC_021160.1_42450812	Ca_kabuli_chr01	42450812	[T/C]	1067291227
Ca_Kabuli_NC_021160.1_42450822	Ca_kabuli_chr01	42450822	[A/G]	1067291228
Ca_Kabuli_NC_021160.1_42450834	Ca_kabuli_chr01	42450834	[T/G]	1067291229
Ca_Kabuli_NC_021160.1_42450901	Ca_kabuli_chr01	42450901	[T/G]	1067291230
Ca_Kabuli_NC_021160.1_42450930	Ca_kabuli_chr01	42450930	[A/T]	1067291231
Ca_Kabuli_NC_021160.1_42450936	Ca_kabuli_chr01	42450936	[T/G]	1067291232
Ca_Kabuli_NC_021160.1_42450967	Ca_kabuli_chr01	42450967	[T/C]	1067291233
Ca_Kabuli_NC_021160.1_42450979	Ca_kabuli_chr01	42450979	[T/G]	1067291234
Ca_Kabuli_NC_021160.1_42450982	Ca_kabuli_chr01	42450982	[T/G]	1067291235
Ca_Kabuli_NC_021160.1_42451008	Ca_kabuli_chr01	42451008	[A/T]	1067291236
Ca_Kabuli_NC_021160.1_42451015	Ca_kabuli_chr01	42451015	[A/G]	1067291237
Ca_Kabuli_NC_021160.1_42451027	Ca_kabuli_chr01	42451027	[A/G]	1067291238
Ca_Kabuli_NC_021160.1_42451298	Ca_kabuli_chr01	42451298	[T/C]	1067291239
Ca_Kabuli_NC_021160.1_42451338	Ca_kabuli_chr01	42451338	[T/C]	1067291240
Ca_Kabuli_NC_021160.1_42451374	Ca_kabuli_chr01	42451374	[A/G]	1067291241
Ca_Kabuli_NC_021160.1_42451376	Ca_kabuli_chr01	42451376	[T/C]	1067291242
Ca_Kabuli_NC_021160.1_42451456	Ca_kabuli_chr01	42451456	[T/A]	1067291243
Ca_Kabuli_NC_021160.1_42451459	Ca_kabuli_chr01	42451459	[T/G]	1067291244
Ca_Kabuli_NC_021160.1_42451502	Ca_kabuli_chr01	42451502	[T/C]	1067291245
Ca_Kabuli_NC_021160.1_42451504	Ca_kabuli_chr01	42451504	[A/G]	1067291246
Ca_Kabuli_NC_021160.1_42451528	Ca_kabuli_chr01	42451528	[T/C]	1067291247
Ca_Kabuli_NC_021160.1_42451535	Ca_kabuli_chr01	42451535	[T/C]	1067291248

SNP IDs	Chromosomes	Physical positions (bp)	SNPs	NCBI SNPdb accession IDs (NCBI_ss#)
Ca_Kabuli_NC_021160.1_42451540	Ca_kabuli_chr01	42451540	[T/C]	1067291249
Ca_Kabuli_NC_021160.1_42451619	Ca_kabuli_chr01	42451619	[T/C]	1067291250
Ca_Kabuli_NC_021160.1_42451624	Ca_kabuli_chr01	42451624	[T/C]	1067291251
Ca_Kabuli_NC_021160.1_42451629	Ca_kabuli_chr01	42451629	[A/G]	1067291252
Ca_Kabuli_NC_021160.1_42451653	Ca_kabuli_chr01	42451653	[A/C]	1067291253
Ca_Kabuli_NC_021160.1_42451664	Ca_kabuli_chr01	42451664	[A/C]	1067291254
Ca_Kabuli_NC_021160.1_42451680	Ca_kabuli_chr01	42451680	[T/C]	1067291255
Ca_Kabuli_NC_021160.1_42451708	Ca_kabuli_chr01	42451708	[T/C]	1067291256
Ca_Kabuli_NC_021160.1_42451717	Ca_kabuli_chr01	42451717	[G/A]	1067291257
Ca_Kabuli_NC_021160.1_42451778	Ca_kabuli_chr01	42451778	[T/C]	1067291258
Ca_Kabuli_NC_021160.1_42451780	Ca_kabuli_chr01	42451780	[T/C]	1067291259
Ca_Kabuli_NC_021160.1_42451783	Ca_kabuli_chr01	42451783	[T/A]	1067291260
Ca_Kabuli_NC_021160.1_42451795	Ca_kabuli_chr01	42451795	[T/A]	1067291261
Ca_Kabuli_NC_021160.1_42451815	Ca_kabuli_chr01	42451815	[T/C]	1067291262
Ca_Kabuli_NC_021160.1_42451818	Ca_kabuli_chr01	42451818	[T/G]	1067291263
Ca_Kabuli_NC_021160.1_42451831	Ca_kabuli_chr01	42451831	[A/G]	1067291264
Ca_Kabuli_NC_021160.1_42451855	Ca_kabuli_chr01	42451855	[T/C]	1067291265
Ca_Kabuli_NC_021160.1_42451862	Ca_kabuli_chr01	42451862	[T/C]	1067291266
Ca_Kabuli_NC_021160.1_42451882	Ca_kabuli_chr01	42451882	[A/T]	1067291267
Ca_Kabuli_NC_021160.1_42451966	Ca_kabuli_chr01	42451966	[A/C]	1067291268
Ca_Kabuli_NC_021160.1_42451986	Ca_kabuli_chr01	42451986	[T/C]	1067291269
Ca_Kabuli_NC_021160.1_42452026	Ca_kabuli_chr01	42452026	[T/G]	1067291270
Ca_Kabuli_NC_021160.1_42452035	Ca_kabuli_chr01	42452035	[T/C]	1067291271
Ca_Kabuli_NC_021160.1_42452110	Ca_kabuli_chr01	42452110	[T/C]	1067291272
Ca_Kabuli_NC_021160.1_42452191	Ca_kabuli_chr01	42452191	[T/G]	1067291273
Ca_Kabuli_NC_021160.1_42452552	Ca_kabuli_chr01	42452552	[T/C]	1067291274
Ca_Kabuli_NC_021160.1_42452623	Ca_kabuli_chr01	42452623	[T/C]	1067291275
Ca_Kabuli_NC_021160.1_42452638	Ca_kabuli_chr01	42452638	[T/G]	1067291276
Ca_Kabuli_NC_021160.1_42452642	Ca_kabuli_chr01	42452642	[A/G]	1067291277
Ca_Kabuli_NC_021160.1_42452647	Ca_kabuli_chr01	42452647	[T/G]	1067291278
Ca_Kabuli_NC_021160.1_42452648	Ca_kabuli_chr01	42452648	[A/G]	1067291279
Ca_Kabuli_NC_021160.1_42452664	Ca_kabuli_chr01	42452664	[A/C]	1067291280
Ca_Kabuli_NC_021160.1_42452675	Ca_kabuli_chr01	42452675	[A/G]	1067291281
Ca_Kabuli_NC_021160.1_42452683	Ca_kabuli_chr01	42452683	[A/G]	1067291282

SNP IDs	Chromosomes	Physical positions (bp)	SNPs	NCBI SNPdb accession IDs (NCBI_ss#)
Ca_Kabuli_NC_021160.1_42452722	Ca_kabuli_chr01	42452722	[T/C]	1067291283
Ca_Kabuli_NC_021160.1_42452748	Ca_kabuli_chr01	42452748	[A/C]	1067291284
Ca_Kabuli_NC_021160.1_42452803	Ca_kabuli_chr01	42452803	[T/C]	1067291285
Ca_Kabuli_NC_021160.1_42452810	Ca_kabuli_chr01	42452810	[A/G]	1067291286
Ca_Kabuli_NC_021160.1_42452826	Ca_kabuli_chr01	42452826	[A/C]	1067291287
Ca_Kabuli_NC_021160.1_42452832	Ca_kabuli_chr01	42452832	[T/G]	1067291288
Ca_Kabuli_NC_021160.1_42452837	Ca_kabuli_chr01	42452837	[A/C]	1067291289
Ca_Kabuli_NC_021160.1_42452872	Ca_kabuli_chr01	42452872	[T/G]	1067291290
Ca_Kabuli_NC_021160.1_42452882	Ca_kabuli_chr01	42452882	[A/G]	1067291291
Ca_Kabuli_NC_021160.1_42452885	Ca_kabuli_chr01	42452885	[A/G]	1067291292
Ca_Kabuli_NC_021160.1_42452959	Ca_kabuli_chr01	42452959	[T/G]	1067291293
Ca_Kabuli_NC_021160.1_42452962	Ca_kabuli_chr01	42452962	[A/C]	1067291294
Ca_Kabuli_NC_021160.1_42452976	Ca_kabuli_chr01	42452976	[A/C]	1067291295
Ca_Kabuli_NC_021160.1_42453025	Ca_kabuli_chr01	42453025	[T/C]	1067291296
Ca_Kabuli_NC_021160.1_42453051	Ca_kabuli_chr01	42453051	[A/C]	1067291297
Ca_Kabuli_NC_021160.1_42453070	Ca_kabuli_chr01	42453070	[A/G]	1067291298
Ca_Kabuli_NC_021160.1_42453083	Ca_kabuli_chr01	42453083	[A/G]	1067291299
Ca_Kabuli_NC_021160.1_42860146	Ca_kabuli_chr01	42860146	[T/C]	1067291300
Ca_Kabuli_NC_021160.1_43053356	Ca_kabuli_chr01	43053356	[C/G]	1067291301
Ca_Kabuli_NC_021160.1_43053372	Ca_kabuli_chr01	43053372	[T/G]	1067291302
Ca_Kabuli_NC_021160.1_43053545	Ca_kabuli_chr01	43053545	[T/G]	1067291303
Ca_Kabuli_NC_021160.1_43303530	Ca_kabuli_chr01	43303530	[A/T]	1067291304
Ca_Kabuli_NC_021160.1_43304549	Ca_kabuli_chr01	43304549	[A/G]	1067291305
Ca_Kabuli_NC_021160.1_43304559	Ca_kabuli_chr01	43304559	[T/G]	1067291306
Ca_Kabuli_NC_021160.1_43304608	Ca_kabuli_chr01	43304608	[T/A]	1067291307
Ca_Kabuli_NC_021160.1_43305961	Ca_kabuli_chr01	43305961	[T/G]	1067291308
Ca_Kabuli_NC_021160.1_43543945	Ca_kabuli_chr01	43543945	[A/C]	1067291309
Ca_Kabuli_NC_021160.1_43543958	Ca_kabuli_chr01	43543958	[T/C]	1067291310
Ca_Kabuli_NC_021160.1_43543961	Ca_kabuli_chr01	43543961	[A/C]	1067291311
Ca_Kabuli_NC_021160.1_43543972	Ca_kabuli_chr01	43543972	[T/C]	1067291312
Ca_Kabuli_NC_021160.1_43725046	Ca_kabuli_chr01	43725046	[T/G]	1067291313
Ca_Kabuli_NC_021160.1_43727856	Ca_kabuli_chr01	43727856	[T/A]	1067291314
Ca_Kabuli_NC_021160.1_43753049	Ca_kabuli_chr01	43753049	[A/C]	1067291315
Ca_Kabuli_NC_021160.1_43754076	Ca_kabuli_chr01	43754076	[T/G]	1067291316

SNP IDs	Chromosomes	Physical positions (bp)	SNPs	NCBI SNPdb accession IDs (NCBI_ss#)
Ca_Kabuli_NC_021160.1_43782277	Ca_kabuli_chr01	43782277	[T/G]	1067291317
Ca_Kabuli_NC_021160.1_43924558	Ca_kabuli_chr01	43924558	[T/G]	1067291318
Ca_Kabuli_NC_021160.1_43924562	Ca_kabuli_chr01	43924562	[A/G]	1067291319
Ca_Kabuli_NC_021160.1_43929264	Ca_kabuli_chr01	43929264	[A/G]	1067291320
Ca_Kabuli_NC_021160.1_43933984	Ca_kabuli_chr01	43933984	[C/T]	1067291321
Ca_Kabuli_NC_021160.1_43934070	Ca_kabuli_chr01	43934070	[T/C]	1067291322
Ca_Kabuli_NC_021160.1_43994989	Ca_kabuli_chr01	43994989	[T/C]	1067291323
Ca_Kabuli_NC_021160.1_44096017	Ca_kabuli_chr01	44096017	[T/C]	1067291324
Ca_Kabuli_NC_021160.1_44096052	Ca_kabuli_chr01	44096052	[G/C]	1067291325
Ca_Kabuli_NC_021160.1_44096065	Ca_kabuli_chr01	44096065	[A/G]	1067291326
Ca_Kabuli_NC_021160.1_44096071	Ca_kabuli_chr01	44096071	[A/C]	1067291327
Ca_Kabuli_NC_021160.1_44096088	Ca_kabuli_chr01	44096088	[T/G]	1067291328
Ca_Kabuli_NC_021160.1_44444216	Ca_kabuli_chr01	44444216	[T/G]	1067291329
Ca_Kabuli_NC_021160.1_44444218	Ca_kabuli_chr01	44444218	[A/G]	1067291330
Ca_Kabuli_NC_021160.1_44518201	Ca_kabuli_chr01	44518201	[C/A]	1067291331
Ca_Kabuli_NC_021160.1_44518202	Ca_kabuli_chr01	44518202	[C/G]	1067291332
Ca_Kabuli_NC_021160.1_44518203	Ca_kabuli_chr01	44518203	[T/A]	1067291333
Ca_Kabuli_NC_021160.1_44862154	Ca_kabuli_chr01	44862154	[T/G]	1067291334
Ca_Kabuli_NC_021160.1_45044113	Ca_kabuli_chr01	45044113	[A/G]	1067291335
Ca_Kabuli_NC_021160.1_45074532	Ca_kabuli_chr01	45074532	[T/C]	1067291336
Ca_Kabuli_NC_021160.1_45088864	Ca_kabuli_chr01	45088864	[T/C]	1067291337
Ca_Kabuli_NC_021160.1_45088871	Ca_kabuli_chr01	45088871	[A/G]	1067291338
Ca_Kabuli_NC_021160.1_45088948	Ca_kabuli_chr01	45088948	[A/G]	1067291339
Ca_Kabuli_NC_021160.1_45484919	Ca_kabuli_chr01	45484919	[A/G]	1067291340
Ca_Kabuli_NC_021160.1_45484925	Ca_kabuli_chr01	45484925	[A/C]	1067291341
Ca_Kabuli_NC_021160.1_45484951	Ca_kabuli_chr01	45484951	[T/C]	1067291342
Ca_Kabuli_NC_021160.1_45826522	Ca_kabuli_chr01	45826522	[A/T]	1067291343
Ca_Kabuli_NC_021160.1_45826559	Ca_kabuli_chr01	45826559	[T/C]	1067291344
Ca_Kabuli_NC_021160.1_45826625	Ca_kabuli_chr01	45826625	[A/G]	1067291345
Ca_Kabuli_NC_021160.1_46437023	Ca_kabuli_chr01	46437023	[T/G]	1067291346
Ca_Kabuli_NC_021160.1_46456567	Ca_kabuli_chr01	46456567	[G/A]	1067291347
Ca_Kabuli_NC_021160.1_46523221	Ca_kabuli_chr01	46523221	[T/C]	1067291348
Ca_Kabuli_NC_021160.1_46717322	Ca_kabuli_chr01	46717322	[G/A]	1067291349
Ca_Kabuli_NC_021160.1_46736579	Ca_kabuli_chr01	46736579	[A/C]	1067291350

SNP IDs	Chromosomes	Physical positions (bp)	SNPs	NCBI SNPdb accession IDs (NCBI_ss#)
Ca_Kabuli_NC_021160.1_46737758	Ca_kabuli_chr01	46737758	[T/C]	1067291351
Ca_Kabuli_NC_021160.1_46737770	Ca_kabuli_chr01	46737770	[G/A]	1067291352
Ca_Kabuli_NC_021160.1_46737781	Ca_kabuli_chr01	46737781	[A/G]	1067291353
Ca_Kabuli_NC_021160.1_46764836	Ca_kabuli_chr01	46764836	[T/C]	1067291354
Ca_Kabuli_NC_021160.1_46779325	Ca_kabuli_chr01	46779325	[T/G]	1067291355
Ca_Kabuli_NC_021160.1_46779336	Ca_kabuli_chr01	46779336	[T/A]	1067291356
Ca_Kabuli_NC_021160.1_46779362	Ca_kabuli_chr01	46779362	[A/C]	1067291357
Ca_Kabuli_NC_021160.1_46779764	Ca_kabuli_chr01	46779764	[G/A]	1067291358
Ca_Kabuli_NC_021160.1_46793271	Ca_kabuli_chr01	46793271	[A/T]	1067291359
Ca_Kabuli_NC_021160.1_46793284	Ca_kabuli_chr01	46793284	[T/C]	1067291360
Ca_Kabuli_NC_021160.1_46830882	Ca_kabuli_chr01	46830882	[T/C]	1067291361
Ca_Kabuli_NC_021160.1_46830911	Ca_kabuli_chr01	46830911	[A/G]	1067291362
Ca_Kabuli_NC_021160.1_46871009	Ca_kabuli_chr01	46871009	[A/C]	1067291363
Ca_Kabuli_NC_021160.1_46941841	Ca_kabuli_chr01	46941841	[T/C]	1067291364
Ca_Kabuli_NC_021160.1_46968694	Ca_kabuli_chr01	46968694	[T/C]	1067291365
Ca_Kabuli_NC_021160.1_46968740	Ca_kabuli_chr01	46968740	[T/C]	1067291366
Ca_Kabuli_NC_021160.1_46969221	Ca_kabuli_chr01	46969221	[T/G]	1067291367
Ca_Kabuli_NC_021160.1_46969302	Ca_kabuli_chr01	46969302	[A/G]	1067291368
Ca_Kabuli_NC_021160.1_47045613	Ca_kabuli_chr01	47045613	[C/T]	1067291369
Ca_Kabuli_NC_021160.1_47045650	Ca_kabuli_chr01	47045650	[A/G]	1067291370
Ca_Kabuli_NC_021160.1_47203105	Ca_kabuli_chr01	47203105	[A/G]	1067291371
Ca_Kabuli_NC_021160.1_47203115	Ca_kabuli_chr01	47203115	[A/C]	1067291372
Ca_Kabuli_NC_021160.1_47203136	Ca_kabuli_chr01	47203136	[T/C]	1067291373
Ca_Kabuli_NC_021160.1_47203161	Ca_kabuli_chr01	47203161	[T/A]	1067291374
Ca_Kabuli_NC_021160.1_47203165	Ca_kabuli_chr01	47203165	[T/C]	1067291375
Ca_Kabuli_NC_021160.1_47468656	Ca_kabuli_chr01	47468656	[C/A]	1067291376
Ca_Kabuli_NC_021160.1_47755803	Ca_kabuli_chr01	47755803	[T/A]	1067291377
Ca_Kabuli_NC_021160.1_47889964	Ca_kabuli_chr01	47889964	[T/G]	1067291378
Ca_Kabuli_NC_021160.1_47889984	Ca_kabuli_chr01	47889984	[T/G]	1067291379
Ca_Kabuli_NC_021160.1_47889995	Ca_kabuli_chr01	47889995	[T/G]	1067291380
Ca_Kabuli_NC_021160.1_47890812	Ca_kabuli_chr01	47890812	[A/G]	1067291381
Ca_Kabuli_NC_021160.1_47896376	Ca_kabuli_chr01	47896376	[A/T]	1067291382
Ca_Kabuli_NC_021160.1_47896398	Ca_kabuli_chr01	47896398	[T/G]	1067291383
Ca_Kabuli_NC_021160.1_47901296	Ca_kabuli_chr01	47901296	[T/G]	1067291384

SNP IDs	Chromosomes	Physical positions (bp)	SNPs	NCBI SNPdb accession IDs (NCBI_ss#)
Ca_Kabuli_NC_021160.1_47947941	Ca_kabuli_chr01	47947941	[C/A]	1067291385
Ca_Kabuli_NC_021160.1_47947964	Ca_kabuli_chr01	47947964	[A/C]	1067291386
Ca_Kabuli_NC_021160.1_47979058	Ca_kabuli_chr01	47979058	[C/A]	1067291387
Ca_Kabuli_NC_021160.1_48002634	Ca_kabuli_chr01	48002634	[A/C]	1067291388
Ca_Kabuli_NC_021160.1_48026686	Ca_kabuli_chr01	48026686	[T/C]	1067291389
Ca_Kabuli_NC_021160.1_48027623	Ca_kabuli_chr01	48027623	[T/G]	1067291390
Ca_Kabuli_NC_021160.1_48027987	Ca_kabuli_chr01	48027987	[T/G]	1067291391
Ca_Kabuli_NC_021160.1_48027996	Ca_kabuli_chr01	48027996	[T/G]	1067291392
Ca_Kabuli_NC_021160.1_48028119	Ca_kabuli_chr01	48028119	[A/T]	1067291393
Ca_Kabuli_NC_021160.1_48028129	Ca_kabuli_chr01	48028129	[T/G]	1067291394
Ca_Kabuli_NC_021160.1_48028131	Ca_kabuli_chr01	48028131	[A/G]	1067291395
Ca_Kabuli_NC_021160.1_48028136	Ca_kabuli_chr01	48028136	[G/A]	1067291396
Ca_Kabuli_NC_021160.1_48028139	Ca_kabuli_chr01	48028139	[G/A]	1067291397
Ca_Kabuli_NC_021160.1_48028191	Ca_kabuli_chr01	48028191	[G/T]	1067291398
Ca_Kabuli_NC_021160.1_48028282	Ca_kabuli_chr01	48028282	[T/G]	1067291399
Ca_Kabuli_NC_021160.1_48028382	Ca_kabuli_chr01	48028382	[T/C]	1067291400
Ca_Kabuli_NC_021160.1_48028943	Ca_kabuli_chr01	48028943	[T/G]	1067291401
Ca_Kabuli_NC_021160.1_48028958	Ca_kabuli_chr01	48028958	[T/G]	1067291402
Ca_Kabuli_NC_021160.1_48028982	Ca_kabuli_chr01	48028982	[A/G]	1067291403
Ca_Kabuli_NC_021160.1_48049250	Ca_kabuli_chr01	48049250	[A/C]	1067291404
Ca_Kabuli_NC_021160.1_48049256	Ca_kabuli_chr01	48049256	[A/C]	1067291405
Ca_Kabuli_NC_021160.1_48049275	Ca_kabuli_chr01	48049275	[T/C]	1067291406
Ca_Kabuli_NC_021160.1_48132202	Ca_kabuli_chr01	48132202	[T/G]	1067291407
Ca_Kabuli_NC_021160.1_48132210	Ca_kabuli_chr01	48132210	[T/C]	1067291408
Ca_Kabuli_NC_021160.1_48198820	Ca_kabuli_chr01	48198820	[A/T]	1067291409
Ca_Kabuli_NC_021160.1_48198825	Ca_kabuli_chr01	48198825	[A/C]	1067291410
Ca_Kabuli_NC_021160.1_48198899	Ca_kabuli_chr01	48198899	[A/C]	1067291411
Ca_Kabuli_NC_021160.1_48198921	Ca_kabuli_chr01	48198921	[T/C]	1067291412
Ca_Kabuli_NC_021160.1_48198926	Ca_kabuli_chr01	48198926	[T/A]	1067291413
Ca_Kabuli_NC_021160.1_48198934	Ca_kabuli_chr01	48198934	[T/A]	1067291414
Ca_Kabuli_NC_021160.1_48265598	Ca_kabuli_chr01	48265598	[T/C]	1067291415
Ca_Kabuli_NC_021160.1_48265618	Ca_kabuli_chr01	48265618	[C/T]	1067291416
Ca_Kabuli_NC_021160.1_48265652	Ca_kabuli_chr01	48265652	[T/C]	1067291417
Ca_Kabuli_NC_021160.1_48273236	Ca_kabuli_chr01	48273236	[T/C]	1067291418

SNP IDs	Chromosomes	Physical positions (bp)	SNPs	NCBI SNPdb accession IDs (NCBI_ss#)
Ca_Kabuli_NC_021160.1_48273253	Ca_kabuli_chr01	48273253	[A/C]	1067291419
Ca_Kabuli_NC_021160.1_48273267	Ca_kabuli_chr01	48273267	[G/A]	1067291420
Ca_Kabuli_NC_021160.1_48273272	Ca_kabuli_chr01	48273272	[A/C]	1067291421
Ca_Kabuli_NC_021160.1_48273277	Ca_kabuli_chr01	48273277	[A/G]	1067291422
Ca_Kabuli_NC_021160.1_48273344	Ca_kabuli_chr01	48273344	[T/G]	1067291423
Ca_Kabuli_NC_021160.1_48273389	Ca_kabuli_chr01	48273389	[A/C]	1067291424
Ca_Kabuli_NC_021160.1_48273411	Ca_kabuli_chr01	48273411	[A/G]	1067291425
Ca_Kabuli_NC_021160.1_48338891	Ca_kabuli_chr01	48338891	[T/G]	1067291426
Ca_Kabuli_NC_021160.1_48338900	Ca_kabuli_chr01	48338900	[A/C]	1067291427
Ca_Kabuli_NC_021161.1_104226	Ca_kabuli_chr02	104226	[T/C]	1067291428
Ca_Kabuli_NC_021161.1_106736	Ca_kabuli_chr02	106736	[A/C]	1067291429
Ca_Kabuli_NC_021161.1_113402	Ca_kabuli_chr02	113402	[T/C]	1067291430
Ca_Kabuli_NC_021161.1_113411	Ca_kabuli_chr02	113411	[G/C]	1067291431
Ca_Kabuli_NC_021161.1_113464	Ca_kabuli_chr02	113464	[T/C]	1067291432
Ca_Kabuli_NC_021161.1_188971	Ca_kabuli_chr02	188971	[T/G]	1067291433
Ca_Kabuli_NC_021161.1_233551	Ca_kabuli_chr02	233551	[T/G]	1067291434
Ca_Kabuli_NC_021161.1_451206	Ca_kabuli_chr02	451206	[T/C]	1067291435
Ca_Kabuli_NC_021161.1_451304	Ca_kabuli_chr02	451304	[T/G]	1067291436
Ca_Kabuli_NC_021161.1_487221	Ca_kabuli_chr02	487221	[C/A]	1067291437
Ca_Kabuli_NC_021161.1_543389	Ca_kabuli_chr02	543389	[A/C]	1067291438
Ca_Kabuli_NC_021161.1_737212	Ca_kabuli_chr02	737212	[T/C]	1067291439
Ca_Kabuli_NC_021161.1_737222	Ca_kabuli_chr02	737222	[A/C]	1067291440
Ca_Kabuli_NC_021161.1_737224	Ca_kabuli_chr02	737224	[T/C]	1067291441
Ca_Kabuli_NC_021161.1_737237	Ca_kabuli_chr02	737237	[T/C]	1067291442
Ca_Kabuli_NC_021161.1_737253	Ca_kabuli_chr02	737253	[T/C]	1067291443
Ca_Kabuli_NC_021161.1_737773	Ca_kabuli_chr02	737773	[T/C]	1067291444
Ca_Kabuli_NC_021161.1_737776	Ca_kabuli_chr02	737776	[T/C]	1067291445
Ca_Kabuli_NC_021161.1_737786	Ca_kabuli_chr02	737786	[A/G]	1067291446
Ca_Kabuli_NC_021161.1_737825	Ca_kabuli_chr02	737825	[A/T]	1067291447
Ca_Kabuli_NC_021161.1_737828	Ca_kabuli_chr02	737828	[A/G]	1067291448
Ca_Kabuli_NC_021161.1_740087	Ca_kabuli_chr02	740087	[T/C]	1067291449
Ca_Kabuli_NC_021161.1_740088	Ca_kabuli_chr02	740088	[C/T]	1067291450
Ca_Kabuli_NC_021161.1_1190385	Ca_kabuli_chr02	1190385	[G/T]	1067291451
Ca_Kabuli_NC_021161.1_1190394	Ca_kabuli_chr02	1190394	[C/A]	1067291452

SNP IDs	Chromosomes	Physical positions (bp)	SNPs	NCBI SNPdb accession IDs (NCBI_ss#)
Ca_Kabuli_NC_021161.1_1198086	Ca_kabuli_chr02	1198086	[A/G]	1067291453
Ca_Kabuli_NC_021161.1_1234985	Ca_kabuli_chr02	1234985	[A/G]	1067291454
Ca_Kabuli_NC_021161.1_1242088	Ca_kabuli_chr02	1242088	[T/A]	1067291455
Ca_Kabuli_NC_021161.1_1314335	Ca_kabuli_chr02	1314335	[A/G]	1067291456
Ca_Kabuli_NC_021161.1_1565538	Ca_kabuli_chr02	1565538	[A/C]	1067291457
Ca_Kabuli_NC_021161.1_1565539	Ca_kabuli_chr02	1565539	[G/A]	1067291458
Ca_Kabuli_NC_021161.1_1565540	Ca_kabuli_chr02	1565540	[A/C]	1067291459
Ca_Kabuli_NC_021161.1_1565541	Ca_kabuli_chr02	1565541	[T/C]	1067291460
Ca_Kabuli_NC_021161.1_1573716	Ca_kabuli_chr02	1573716	[T/C]	1067291461
Ca_Kabuli_NC_021161.1_1584393	Ca_kabuli_chr02	1584393	[T/G]	1067291462
Ca_Kabuli_NC_021161.1_1584412	Ca_kabuli_chr02	1584412	[C/A]	1067291463
Ca_Kabuli_NC_021161.1_1584413	Ca_kabuli_chr02	1584413	[A/G]	1067291464
Ca_Kabuli_NC_021161.1_1610606	Ca_kabuli_chr02	1610606	[T/C]	1067291465
Ca_Kabuli_NC_021161.1_1624937	Ca_kabuli_chr02	1624937	[A/C]	1067291466
Ca_Kabuli_NC_021161.1_1671279	Ca_kabuli_chr02	1671279	[T/C]	1067291467
Ca_Kabuli_NC_021161.1_1704218	Ca_kabuli_chr02	1704218	[A/C]	1067291468
Ca_Kabuli_NC_021161.1_1704229	Ca_kabuli_chr02	1704229	[T/C]	1067291469
Ca_Kabuli_NC_021161.1_1713310	Ca_kabuli_chr02	1713310	[T/G]	1067291470
Ca_Kabuli_NC_021161.1_1728819	Ca_kabuli_chr02	1728819	[G/C]	1067291471
Ca_Kabuli_NC_021161.1_1728820	Ca_kabuli_chr02	1728820	[G/C]	1067291472
Ca_Kabuli_NC_021161.1_1801332	Ca_kabuli_chr02	1801332	[T/C]	1067291473
Ca_Kabuli_NC_021161.1_1803466	Ca_kabuli_chr02	1803466	[T/G]	1067291474
Ca_Kabuli_NC_021161.1_1804162	Ca_kabuli_chr02	1804162	[T/C]	1067291475
Ca_Kabuli_NC_021161.1_1804184	Ca_kabuli_chr02	1804184	[A/C]	1067291476
Ca_Kabuli_NC_021161.1_1819997	Ca_kabuli_chr02	1819997	[T/G]	1067291477
Ca_Kabuli_NC_021161.1_1828324	Ca_kabuli_chr02	1828324	[T/A]	1067291478
Ca_Kabuli_NC_021161.1_1828333	Ca_kabuli_chr02	1828333	[T/G]	1067291479
Ca_Kabuli_NC_021161.1_1828346	Ca_kabuli_chr02	1828346	[T/G]	1067291480
Ca_Kabuli_NC_021161.1_1828360	Ca_kabuli_chr02	1828360	[A/G]	1067291481
Ca_Kabuli_NC_021161.1_1828388	Ca_kabuli_chr02	1828388	[A/C]	1067291482
Ca_Kabuli_NC_021161.1_1865072	Ca_kabuli_chr02	1865072	[T/C]	1067291483
Ca_Kabuli_NC_021161.1_1865197	Ca_kabuli_chr02	1865197	[A/G]	1067291484
Ca_Kabuli_NC_021161.1_1882667	Ca_kabuli_chr02	1882667	[T/A]	1067291485
Ca_Kabuli_NC_021161.1_1882676	Ca_kabuli_chr02	1882676	[T/A]	1067291486

SNP IDs	Chromosomes	Physical positions (bp)	SNPs	NCBI SNPdb accession IDs (NCBI_ss#)
Ca_Kabuli_NC_021161.1_1884640	Ca_kabuli_chr02	1884640	[T/C]	1067291487
Ca_Kabuli_NC_021161.1_1884649	Ca_kabuli_chr02	1884649	[T/G]	1067291488
Ca_Kabuli_NC_021161.1_1884670	Ca_kabuli_chr02	1884670	[T/G]	1067291489
Ca_Kabuli_NC_021161.1_1884694	Ca_kabuli_chr02	1884694	[A/G]	1067291490
Ca_Kabuli_NC_021161.1_1901253	Ca_kabuli_chr02	1901253	[A/T]	1067291491
Ca_Kabuli_NC_021161.1_1953645	Ca_kabuli_chr02	1953645	[A/G]	1067291492
Ca_Kabuli_NC_021161.1_2003544	Ca_kabuli_chr02	2003544	[T/C]	1067291493
Ca_Kabuli_NC_021161.1_2003584	Ca_kabuli_chr02	2003584	[A/C]	1067291494
Ca_Kabuli_NC_021161.1_2078923	Ca_kabuli_chr02	2078923	[T/A]	1067291495
Ca_Kabuli_NC_021161.1_2080305	Ca_kabuli_chr02	2080305	[A/G]	1067291496
Ca_Kabuli_NC_021161.1_2080312	Ca_kabuli_chr02	2080312	[A/G]	1067291497
Ca_Kabuli_NC_021161.1_2080356	Ca_kabuli_chr02	2080356	[T/G]	1067291498
Ca_Kabuli_NC_021161.1_2080358	Ca_kabuli_chr02	2080358	[T/A]	1067291499
Ca_Kabuli_NC_021161.1_2080361	Ca_kabuli_chr02	2080361	[A/G]	1067291500
Ca_Kabuli_NC_021161.1_2080413	Ca_kabuli_chr02	2080413	[T/G]	1067291501
Ca_Kabuli_NC_021161.1_2080418	Ca_kabuli_chr02	2080418	[T/G]	1067291502
Ca_Kabuli_NC_021161.1_2085354	Ca_kabuli_chr02	2085354	[T/C]	1067291503
Ca_Kabuli_NC_021161.1_2085406	Ca_kabuli_chr02	2085406	[T/A]	1067291504
Ca_Kabuli_NC_021161.1_2086795	Ca_kabuli_chr02	2086795	[T/G]	1067291505
Ca_Kabuli_NC_021161.1_2227452	Ca_kabuli_chr02	2227452	[A/G]	1067291506
Ca_Kabuli_NC_021161.1_2227506	Ca_kabuli_chr02	2227506	[T/C]	1067291507
Ca_Kabuli_NC_021161.1_2312002	Ca_kabuli_chr02	2312002	[T/C]	1067291508
Ca_Kabuli_NC_021161.1_2312049	Ca_kabuli_chr02	2312049	[T/C]	1067291509
Ca_Kabuli_NC_021161.1_2312071	Ca_kabuli_chr02	2312071	[T/C]	1067291510
Ca_Kabuli_NC_021161.1_2416367	Ca_kabuli_chr02	2416367	[T/G]	1067291511
Ca_Kabuli_NC_021161.1_2420820	Ca_kabuli_chr02	2420820	[T/C]	1067291512
Ca_Kabuli_NC_021161.1_2420851	Ca_kabuli_chr02	2420851	[A/G]	1067291513
Ca_Kabuli_NC_021161.1_2424862	Ca_kabuli_chr02	2424862	[T/C]	1067291514
Ca_Kabuli_NC_021161.1_2577056	Ca_kabuli_chr02	2577056	[A/T]	1067291515
Ca_Kabuli_NC_021161.1_2577077	Ca_kabuli_chr02	2577077	[T/G]	1067291516
Ca_Kabuli_NC_021161.1_2578333	Ca_kabuli_chr02	2578333	[A/C]	1067291517
Ca_Kabuli_NC_021161.1_2578341	Ca_kabuli_chr02	2578341	[T/C]	1067291518
Ca_Kabuli_NC_021161.1_2578373	Ca_kabuli_chr02	2578373	[T/C]	1067291519
Ca_Kabuli_NC_021161.1_2579095	Ca_kabuli_chr02	2579095	[T/C]	1067291520

SNP IDs	Chromosomes	Physical positions (bp)	SNPs	NCBI SNPdb accession IDs (NCBI_ss#)
Ca_Kabuli_NC_021161.1_2579106	Ca_kabuli_chr02	2579106	[T/C]	1067291521
Ca_Kabuli_NC_021161.1_2697827	Ca_kabuli_chr02	2697827	[T/C]	1067291522
Ca_Kabuli_NC_021161.1_2697829	Ca_kabuli_chr02	2697829	[A/G]	1067291523
Ca_Kabuli_NC_021161.1_2697860	Ca_kabuli_chr02	2697860	[T/G]	1067291524
Ca_Kabuli_NC_021161.1_2762971	Ca_kabuli_chr02	2762971	[C/G]	1067291525
Ca_Kabuli_NC_021161.1_2762973	Ca_kabuli_chr02	2762973	[G/A]	1067291526
Ca_Kabuli_NC_021161.1_2762974	Ca_kabuli_chr02	2762974	[G/A]	1067291527
Ca_Kabuli_NC_021161.1_2762977	Ca_kabuli_chr02	2762977	[A/G]	1067291528
Ca_Kabuli_NC_021161.1_2965854	Ca_kabuli_chr02	2965854	[T/C]	1067291529
Ca_Kabuli_NC_021161.1_2965856	Ca_kabuli_chr02	2965856	[T/C]	1067291530
Ca_Kabuli_NC_021161.1_2965861	Ca_kabuli_chr02	2965861	[T/C]	1067291531
Ca_Kabuli_NC_021161.1_2965874	Ca_kabuli_chr02	2965874	[T/C]	1067291532
Ca_Kabuli_NC_021161.1_2965903	Ca_kabuli_chr02	2965903	[T/G]	1067291533
Ca_Kabuli_NC_021161.1_3044461	Ca_kabuli_chr02	3044461	[T/C]	1067291534
Ca_Kabuli_NC_021161.1_3074493	Ca_kabuli_chr02	3074493	[T/G]	1067291535
Ca_Kabuli_NC_021161.1_3074499	Ca_kabuli_chr02	3074499	[A/G]	1067291536
Ca_Kabuli_NC_021161.1_3074517	Ca_kabuli_chr02	3074517	[C/A]	1067291537
Ca_Kabuli_NC_021161.1_3074518	Ca_kabuli_chr02	3074518	[T/C]	1067291538
Ca_Kabuli_NC_021161.1_3099939	Ca_kabuli_chr02	3099939	[A/G]	1067291539
Ca_Kabuli_NC_021161.1_3099967	Ca_kabuli_chr02	3099967	[A/T]	1067291540
Ca_Kabuli_NC_021161.1_3145552	Ca_kabuli_chr02	3145552	[A/G]	1067291541
Ca_Kabuli_NC_021161.1_3145555	Ca_kabuli_chr02	3145555	[T/C]	1067291542
Ca_Kabuli_NC_021161.1_3145568	Ca_kabuli_chr02	3145568	[A/G]	1067291543
Ca_Kabuli_NC_021161.1_3151157	Ca_kabuli_chr02	3151157	[T/A]	1067291544
Ca_Kabuli_NC_021161.1_3164292	Ca_kabuli_chr02	3164292	[T/A]	1067291545
Ca_Kabuli_NC_021161.1_3164298	Ca_kabuli_chr02	3164298	[A/T]	1067291546
Ca_Kabuli_NC_021161.1_3450843	Ca_kabuli_chr02	3450843	[T/C]	1067291547
Ca_Kabuli_NC_021161.1_3450856	Ca_kabuli_chr02	3450856	[A/C]	1067291548
Ca_Kabuli_NC_021161.1_3450886	Ca_kabuli_chr02	3450886	[A/C]	1067291549
Ca_Kabuli_NC_021161.1_3450889	Ca_kabuli_chr02	3450889	[A/C]	1067291550
Ca_Kabuli_NC_021161.1_3450898	Ca_kabuli_chr02	3450898	[A/C]	1067291551
Ca_Kabuli_NC_021161.1_3480904	Ca_kabuli_chr02	3480904	[A/G]	1067291552
Ca_Kabuli_NC_021161.1_3482714	Ca_kabuli_chr02	3482714	[A/C]	1067291553
Ca_Kabuli_NC_021161.1_3548778	Ca_kabuli_chr02	3548778	[C/T]	1067291554

SNP IDs	Chromosomes	Physical positions (bp)	SNPs	NCBI SNPdb accession IDs (NCBI_ss#)
Ca_Kabuli_NC_021161.1_3548789	Ca_kabuli_chr02	3548789	[T/G]	1067291555
Ca_Kabuli_NC_021161.1_3548818	Ca_kabuli_chr02	3548818	[A/G]	1067291556
Ca_Kabuli_NC_021161.1_3663621	Ca_kabuli_chr02	3663621	[A/T]	1067291557
Ca_Kabuli_NC_021161.1_3688767	Ca_kabuli_chr02	3688767	[T/G]	1067291558
Ca_Kabuli_NC_021161.1_3706508	Ca_kabuli_chr02	3706508	[C/T]	1067291559
Ca_Kabuli_NC_021161.1_3789782	Ca_kabuli_chr02	3789782	[C/T]	1067291560
Ca_Kabuli_NC_021161.1_3789793	Ca_kabuli_chr02	3789793	[A/G]	1067291561
Ca_Kabuli_NC_021161.1_3808452	Ca_kabuli_chr02	3808452	[T/C]	1067291562
Ca_Kabuli_NC_021161.1_3808473	Ca_kabuli_chr02	3808473	[A/T]	1067291563
Ca_Kabuli_NC_021161.1_3808490	Ca_kabuli_chr02	3808490	[C/T]	1067291564
Ca_Kabuli_NC_021161.1_3808491	Ca_kabuli_chr02	3808491	[C/T]	1067291565
Ca_Kabuli_NC_021161.1_3856590	Ca_kabuli_chr02	3856590	[A/T]	1067291566
Ca_Kabuli_NC_021161.1_3870877	Ca_kabuli_chr02	3870877	[A/G]	1067291567
Ca_Kabuli_NC_021161.1_3904829	Ca_kabuli_chr02	3904829	[A/C]	1067291568
Ca_Kabuli_NC_021161.1_3908947	Ca_kabuli_chr02	3908947	[G/C]	1067291569
Ca_Kabuli_NC_021161.1_3908953	Ca_kabuli_chr02	3908953	[A/C]	1067291570
Ca_Kabuli_NC_021161.1_3915501	Ca_kabuli_chr02	3915501	[T/C]	1067291571
Ca_Kabuli_NC_021161.1_3915506	Ca_kabuli_chr02	3915506	[A/T]	1067291572
Ca_Kabuli_NC_021161.1_3915511	Ca_kabuli_chr02	3915511	[T/C]	1067291573
Ca_Kabuli_NC_021161.1_3915512	Ca_kabuli_chr02	3915512	[A/G]	1067291574
Ca_Kabuli_NC_021161.1_3915517	Ca_kabuli_chr02	3915517	[T/C]	1067291575
Ca_Kabuli_NC_021161.1_3915524	Ca_kabuli_chr02	3915524	[A/G]	1067291576
Ca_Kabuli_NC_021161.1_3930249	Ca_kabuli_chr02	3930249	[T/C]	1067291577
Ca_Kabuli_NC_021161.1_3930269	Ca_kabuli_chr02	3930269	[T/A]	1067291578
Ca_Kabuli_NC_021161.1_3930290	Ca_kabuli_chr02	3930290	[A/G]	1067291579
Ca_Kabuli_NC_021161.1_3941385	Ca_kabuli_chr02	3941385	[A/T]	1067291580
Ca_Kabuli_NC_021161.1_3941386	Ca_kabuli_chr02	3941386	[G/A]	1067291581
Ca_Kabuli_NC_021161.1_3941387	Ca_kabuli_chr02	3941387	[T/G]	1067291582
Ca_Kabuli_NC_021161.1_4360467	Ca_kabuli_chr02	4360467	[A/G]	1067291583
Ca_Kabuli_NC_021161.1_4360507	Ca_kabuli_chr02	4360507	[T/G]	1067291584
Ca_Kabuli_NC_021161.1_4433263	Ca_kabuli_chr02	4433263	[T/C]	1067291585
Ca_Kabuli_NC_021161.1_4433272	Ca_kabuli_chr02	4433272	[A/C]	1067291586
Ca_Kabuli_NC_021161.1_4433290	Ca_kabuli_chr02	4433290	[T/C]	1067291587
Ca_Kabuli_NC_021161.1_4576979	Ca_kabuli_chr02	4576979	[A/T]	1067291588

SNP IDs	Chromosomes	Physical positions (bp)	SNPs	NCBI SNPdb accession IDs (NCBI_ss#)
Ca_Kabuli_NC_021161.1_4966188	Ca_kabuli_chr02	4966188	[T/C]	1067291589
Ca_Kabuli_NC_021161.1_4994596	Ca_kabuli_chr02	4994596	[T/C]	1067291590
Ca_Kabuli_NC_021161.1_4994635	Ca_kabuli_chr02	4994635	[T/G]	1067291591
Ca_Kabuli_NC_021161.1_5079638	Ca_kabuli_chr02	5079638	[A/C]	1067291592
Ca_Kabuli_NC_021161.1_5101163	Ca_kabuli_chr02	5101163	[T/G]	1067291593
Ca_Kabuli_NC_021161.1_5119812	Ca_kabuli_chr02	5119812	[G/T]	1067291594
Ca_Kabuli_NC_021161.1_5209689	Ca_kabuli_chr02	5209689	[A/G]	1067291595
Ca_Kabuli_NC_021161.1_5247387	Ca_kabuli_chr02	5247387	[T/G]	1067291596
Ca_Kabuli_NC_021161.1_5259270	Ca_kabuli_chr02	5259270	[A/C]	1067291597
Ca_Kabuli_NC_021161.1_5298384	Ca_kabuli_chr02	5298384	[A/C]	1067291598
Ca_Kabuli_NC_021161.1_5299261	Ca_kabuli_chr02	5299261	[A/G]	1067291599
Ca_Kabuli_NC_021161.1_5299270	Ca_kabuli_chr02	5299270	[T/C]	1067291600
Ca_Kabuli_NC_021161.1_5299272	Ca_kabuli_chr02	5299272	[T/C]	1067291601
Ca_Kabuli_NC_021161.1_5342942	Ca_kabuli_chr02	5342942	[A/C]	1067291602
Ca_Kabuli_NC_021161.1_5359143	Ca_kabuli_chr02	5359143	[T/G]	1067291603
Ca_Kabuli_NC_021161.1_5417026	Ca_kabuli_chr02	5417026	[T/G]	1067291604
Ca_Kabuli_NC_021161.1_5417046	Ca_kabuli_chr02	5417046	[A/G]	1067291605
Ca_Kabuli_NC_021161.1_5417051	Ca_kabuli_chr02	5417051	[T/G]	1067291606
Ca_Kabuli_NC_021161.1_5417088	Ca_kabuli_chr02	5417088	[A/T]	1067291607
Ca_Kabuli_NC_021161.1_5444255	Ca_kabuli_chr02	5444255	[A/G]	1067291608
Ca_Kabuli_NC_021161.1_5444287	Ca_kabuli_chr02	5444287	[A/C]	1067291609
Ca_Kabuli_NC_021161.1_5449788	Ca_kabuli_chr02	5449788	[T/A]	1067291610
Ca_Kabuli_NC_021161.1_5449794	Ca_kabuli_chr02	5449794	[A/G]	1067291611
Ca_Kabuli_NC_021161.1_5451160	Ca_kabuli_chr02	5451160	[A/C]	1067291612
Ca_Kabuli_NC_021161.1_5451165	Ca_kabuli_chr02	5451165	[A/C]	1067291613
Ca_Kabuli_NC_021161.1_5451190	Ca_kabuli_chr02	5451190	[A/G]	1067291614
Ca_Kabuli_NC_021161.1_5462998	Ca_kabuli_chr02	5462998	[T/G]	1067291615
Ca_Kabuli_NC_021161.1_5472554	Ca_kabuli_chr02	5472554	[A/G]	1067291616
Ca_Kabuli_NC_021161.1_5472556	Ca_kabuli_chr02	5472556	[T/G]	1067291617
Ca_Kabuli_NC_021161.1_5564516	Ca_kabuli_chr02	5564516	[A/C]	1067291618
Ca_Kabuli_NC_021161.1_5601080	Ca_kabuli_chr02	5601080	[A/G]	1067291619
Ca_Kabuli_NC_021161.1_5601093	Ca_kabuli_chr02	5601093	[G/T]	1067291620
Ca_Kabuli_NC_021161.1_5601099	Ca_kabuli_chr02	5601099	[A/G]	1067291621
Ca_Kabuli_NC_021161.1_5601105	Ca_kabuli_chr02	5601105	[A/G]	1067291622

SNP IDs	Chromosomes	Physical positions (bp)	SNPs	NCBI SNPdb accession IDs (NCBI_ss#)
Ca_Kabuli_NC_021161.1_5627158	Ca_kabuli_chr02	5627158	[G/T]	1067291623
Ca_Kabuli_NC_021161.1_5661608	Ca_kabuli_chr02	5661608	[T/C]	1067291624
Ca_Kabuli_NC_021161.1_5661664	Ca_kabuli_chr02	5661664	[T/C]	1067291625
Ca_Kabuli_NC_021161.1_5829447	Ca_kabuli_chr02	5829447	[T/C]	1067291626
Ca_Kabuli_NC_021161.1_5829454	Ca_kabuli_chr02	5829454	[A/C]	1067291627
Ca_Kabuli_NC_021161.1_5866786	Ca_kabuli_chr02	5866786	[T/C]	1067291628
Ca_Kabuli_NC_021161.1_5866794	Ca_kabuli_chr02	5866794	[C/A]	1067291629
Ca_Kabuli_NC_021161.1_5866795	Ca_kabuli_chr02	5866795	[A/G]	1067291630
Ca_Kabuli_NC_021161.1_5938046	Ca_kabuli_chr02	5938046	[A/G]	1067291631
Ca_Kabuli_NC_021161.1_6052668	Ca_kabuli_chr02	6052668	[A/C]	1067291632
Ca_Kabuli_NC_021161.1_6054862	Ca_kabuli_chr02	6054862	[A/G]	1067291633
Ca_Kabuli_NC_021161.1_6054880	Ca_kabuli_chr02	6054880	[A/G]	1067291634
Ca_Kabuli_NC_021161.1_6054890	Ca_kabuli_chr02	6054890	[T/C]	1067291635
Ca_Kabuli_NC_021161.1_6130262	Ca_kabuli_chr02	6130262	[A/G]	1067291636
Ca_Kabuli_NC_021161.1_6130275	Ca_kabuli_chr02	6130275	[T/G]	1067291637
Ca_Kabuli_NC_021161.1_6130277	Ca_kabuli_chr02	6130277	[A/T]	1067291638
Ca_Kabuli_NC_021161.1_6130289	Ca_kabuli_chr02	6130289	[A/G]	1067291639
Ca_Kabuli_NC_021161.1_6130302	Ca_kabuli_chr02	6130302	[T/A]	1067291640
Ca_Kabuli_NC_021161.1_6162613	Ca_kabuli_chr02	6162613	[A/C]	1067291641
Ca_Kabuli_NC_021161.1_6246589	Ca_kabuli_chr02	6246589	[A/G]	1067291642
Ca_Kabuli_NC_021161.1_6256294	Ca_kabuli_chr02	6256294	[T/C]	1067291643
Ca_Kabuli_NC_021161.1_6257163	Ca_kabuli_chr02	6257163	[T/G]	1067291644
Ca_Kabuli_NC_021161.1_6321964	Ca_kabuli_chr02	6321964	[G/A]	1067291645
Ca_Kabuli_NC_021161.1_6367684	Ca_kabuli_chr02	6367684	[T/G]	1067291646
Ca_Kabuli_NC_021161.1_6367704	Ca_kabuli_chr02	6367704	[A/C]	1067291647
Ca_Kabuli_NC_021161.1_6392257	Ca_kabuli_chr02	6392257	[T/C]	1067291648
Ca_Kabuli_NC_021161.1_6392296	Ca_kabuli_chr02	6392296	[T/C]	1067291649
Ca_Kabuli_NC_021161.1_6396845	Ca_kabuli_chr02	6396845	[T/C]	1067291650
Ca_Kabuli_NC_021161.1_6396873	Ca_kabuli_chr02	6396873	[A/G]	1067291651
Ca_Kabuli_NC_021161.1_6396886	Ca_kabuli_chr02	6396886	[A/G]	1067291652
Ca_Kabuli_NC_021161.1_6397711	Ca_kabuli_chr02	6397711	[T/G]	1067291653
Ca_Kabuli_NC_021161.1_6408374	Ca_kabuli_chr02	6408374	[C/G]	1067291654
Ca_Kabuli_NC_021161.1_6408390	Ca_kabuli_chr02	6408390	[T/A]	1067291655
Ca_Kabuli_NC_021161.1_6412020	Ca_kabuli_chr02	6412020	[C/T]	1067291656

SNP IDs	Chromosomes	Physical positions (bp)	SNPs	NCBI SNPdb accession IDs (NCBI_ss#)
Ca_Kabuli_NC_021161.1_6412028	Ca_kabuli_chr02	6412028	[A/G]	1067291657
Ca_Kabuli_NC_021161.1_6728716	Ca_kabuli_chr02	6728716	[A/T]	1067291658
Ca_Kabuli_NC_021161.1_6750401	Ca_kabuli_chr02	6750401	[T/G]	1067291659
Ca_Kabuli_NC_021161.1_6750409	Ca_kabuli_chr02	6750409	[T/A]	1067291660
Ca_Kabuli_NC_021161.1_6750412	Ca_kabuli_chr02	6750412	[A/C]	1067291661
Ca_Kabuli_NC_021161.1_6750437	Ca_kabuli_chr02	6750437	[A/G]	1067291662
Ca_Kabuli_NC_021161.1_6936812	Ca_kabuli_chr02	6936812	[A/G]	1067291663
Ca_Kabuli_NC_021161.1_6936821	Ca_kabuli_chr02	6936821	[T/C]	1067291664
Ca_Kabuli_NC_021161.1_6936827	Ca_kabuli_chr02	6936827	[T/C]	1067291665
Ca_Kabuli_NC_021161.1_7046939	Ca_kabuli_chr02	7046939	[T/A]	1067291666
Ca_Kabuli_NC_021161.1_7046942	Ca_kabuli_chr02	7046942	[T/C]	1067291667
Ca_Kabuli_NC_021161.1_7047004	Ca_kabuli_chr02	7047004	[T/G]	1067291668
Ca_Kabuli_NC_021161.1_7047015	Ca_kabuli_chr02	7047015	[C/G]	1067291669
Ca_Kabuli_NC_021161.1_7047141	Ca_kabuli_chr02	7047141	[A/G]	1067291670
Ca_Kabuli_NC_021161.1_7047143	Ca_kabuli_chr02	7047143	[T/A]	1067291671
Ca_Kabuli_NC_021161.1_7047182	Ca_kabuli_chr02	7047182	[A/C]	1067291672
Ca_Kabuli_NC_021161.1_7047186	Ca_kabuli_chr02	7047186	[A/C]	1067291673
Ca_Kabuli_NC_021161.1_7047212	Ca_kabuli_chr02	7047212	[A/C]	1067291674
Ca_Kabuli_NC_021161.1_7051422	Ca_kabuli_chr02	7051422	[A/G]	1067291675
Ca_Kabuli_NC_021161.1_7051424	Ca_kabuli_chr02	7051424	[G/A]	1067291676
Ca_Kabuli_NC_021161.1_7051426	Ca_kabuli_chr02	7051426	[A/C]	1067291677
Ca_Kabuli_NC_021161.1_7051427	Ca_kabuli_chr02	7051427	[C/G]	1067291678
Ca_Kabuli_NC_021161.1_7083705	Ca_kabuli_chr02	7083705	[T/C]	1067291679
Ca_Kabuli_NC_021161.1_7125951	Ca_kabuli_chr02	7125951	[T/G]	1067291680
Ca_Kabuli_NC_021161.1_7125959	Ca_kabuli_chr02	7125959	[C/A]	1067291681
Ca_Kabuli_NC_021161.1_7125970	Ca_kabuli_chr02	7125970	[G/A]	1067291682
Ca_Kabuli_NC_021161.1_7125992	Ca_kabuli_chr02	7125992	[T/G]	1067291683
Ca_Kabuli_NC_021161.1_7126029	Ca_kabuli_chr02	7126029	[A/C]	1067291684
Ca_Kabuli_NC_021161.1_7126046	Ca_kabuli_chr02	7126046	[A/C]	1067291685
Ca_Kabuli_NC_021161.1_7570375	Ca_kabuli_chr02	7570375	[T/G]	1067291686
Ca_Kabuli_NC_021161.1_7570376	Ca_kabuli_chr02	7570376	[T/G]	1067291687
Ca_Kabuli_NC_021161.1_7570377	Ca_kabuli_chr02	7570377	[G/T]	1067291688
Ca_Kabuli_NC_021161.1_7570378	Ca_kabuli_chr02	7570378	[G/A]	1067291689
Ca_Kabuli_NC_021161.1_7570379	Ca_kabuli_chr02	7570379	[A/G]	1067291690

SNP IDs	Chromosomes	Physical positions (bp)	SNPs	NCBI SNPdb accession IDs (NCBI_ss#)
Ca_Kabuli_NC_021161.1_7616802	Ca_kabuli_chr02	7616802	[G/A]	1067291691
Ca_Kabuli_NC_021161.1_7616806	Ca_kabuli_chr02	7616806	[T/G]	1067291692
Ca_Kabuli_NC_021161.1_7616890	Ca_kabuli_chr02	7616890	[T/G]	1067291693
Ca_Kabuli_NC_021161.1_7668523	Ca_kabuli_chr02	7668523	[A/C]	1067291694
Ca_Kabuli_NC_021161.1_7679627	Ca_kabuli_chr02	7679627	[A/T]	1067291695
Ca_Kabuli_NC_021161.1_7679693	Ca_kabuli_chr02	7679693	[C/G]	1067291696
Ca_Kabuli_NC_021161.1_7701199	Ca_kabuli_chr02	7701199	[T/G]	1067291697
Ca_Kabuli_NC_021161.1_7701201	Ca_kabuli_chr02	7701201	[T/G]	1067291698
Ca_Kabuli_NC_021161.1_7701203	Ca_kabuli_chr02	7701203	[T/G]	1067291699
Ca_Kabuli_NC_021161.1_7701206	Ca_kabuli_chr02	7701206	[T/A]	1067291700
Ca_Kabuli_NC_021161.1_7701208	Ca_kabuli_chr02	7701208	[A/T]	1067291701
Ca_Kabuli_NC_021161.1_7701216	Ca_kabuli_chr02	7701216	[T/G]	1067291702
Ca_Kabuli_NC_021161.1_7711924	Ca_kabuli_chr02	7711924	[T/A]	1067291703
Ca_Kabuli_NC_021161.1_7712233	Ca_kabuli_chr02	7712233	[T/G]	1067291704
Ca_Kabuli_NC_021161.1_7712249	Ca_kabuli_chr02	7712249	[T/G]	1067291705
Ca_Kabuli_NC_021161.1_7714220	Ca_kabuli_chr02	7714220	[G/A]	1067291706
Ca_Kabuli_NC_021161.1_7714332	Ca_kabuli_chr02	7714332	[A/T]	1067291707
Ca_Kabuli_NC_021161.1_7714392	Ca_kabuli_chr02	7714392	[G/A]	1067291708
Ca_Kabuli_NC_021161.1_7715048	Ca_kabuli_chr02	7715048	[C/T]	1067291709
Ca_Kabuli_NC_021161.1_7715055	Ca_kabuli_chr02	7715055	[T/C]	1067291710
Ca_Kabuli_NC_021161.1_7715065	Ca_kabuli_chr02	7715065	[C/T]	1067291711
Ca_Kabuli_NC_021161.1_7715074	Ca_kabuli_chr02	7715074	[T/A]	1067291712
Ca_Kabuli_NC_021161.1_7753419	Ca_kabuli_chr02	7753419	[G/C]	1067291713
Ca_Kabuli_NC_021161.1_7753425	Ca_kabuli_chr02	7753425	[T/C]	1067291714
Ca_Kabuli_NC_021161.1_7753440	Ca_kabuli_chr02	7753440	[C/T]	1067291715
Ca_Kabuli_NC_021161.1_7753668	Ca_kabuli_chr02	7753668	[A/T]	1067291716
Ca_Kabuli_NC_021161.1_7852704	Ca_kabuli_chr02	7852704	[T/C]	1067291717
Ca_Kabuli_NC_021161.1_7852705	Ca_kabuli_chr02	7852705	[A/G]	1067291718
Ca_Kabuli_NC_021161.1_7852713	Ca_kabuli_chr02	7852713	[G/A]	1067291719
Ca_Kabuli_NC_021161.1_7861570	Ca_kabuli_chr02	7861570	[A/C]	1067291720
Ca_Kabuli_NC_021161.1_7870840	Ca_kabuli_chr02	7870840	[A/T]	1067291721
Ca_Kabuli_NC_021161.1_7870843	Ca_kabuli_chr02	7870843	[A/C]	1067291722
Ca_Kabuli_NC_021161.1_7870943	Ca_kabuli_chr02	7870943	[T/G]	1067291723
Ca_Kabuli_NC_021161.1_7871084	Ca_kabuli_chr02	7871084	[T/A]	1067291724

SNP IDs	Chromosomes	Physical positions (bp)	SNPs	NCBI SNPdb accession IDs (NCBI_ss#)
Ca_Kabuli_NC_021161.1_7871118	Ca_kabuli_chr02	7871118	[A/G]	1067291725
Ca_Kabuli_NC_021161.1_7872709	Ca_kabuli_chr02	7872709	[A/C]	1067291726
Ca_Kabuli_NC_021161.1_7872719	Ca_kabuli_chr02	7872719	[A/C]	1067291727
Ca_Kabuli_NC_021161.1_8200027	Ca_kabuli_chr02	8200027	[A/G]	1067291728
Ca_Kabuli_NC_021161.1_8201025	Ca_kabuli_chr02	8201025	[A/C]	1067291729
Ca_Kabuli_NC_021161.1_8261979	Ca_kabuli_chr02	8261979	[T/A]	1067291730
Ca_Kabuli_NC_021161.1_8261989	Ca_kabuli_chr02	8261989	[T/G]	1067291731
Ca_Kabuli_NC_021161.1_8337534	Ca_kabuli_chr02	8337534	[A/C]	1067291732
Ca_Kabuli_NC_021161.1_8360347	Ca_kabuli_chr02	8360347	[T/A]	1067291733
Ca_Kabuli_NC_021161.1_8360348	Ca_kabuli_chr02	8360348	[C/A]	1067291734
Ca_Kabuli_NC_021161.1_8360349	Ca_kabuli_chr02	8360349	[T/G]	1067291735
Ca_Kabuli_NC_021161.1_8375893	Ca_kabuli_chr02	8375893	[T/C]	1067291736
Ca_Kabuli_NC_021161.1_8375895	Ca_kabuli_chr02	8375895	[A/G]	1067291737
Ca_Kabuli_NC_021161.1_8516215	Ca_kabuli_chr02	8516215	[T/A]	1067291738
Ca_Kabuli_NC_021161.1_8516235	Ca_kabuli_chr02	8516235	[A/C]	1067291739
Ca_Kabuli_NC_021161.1_8516269	Ca_kabuli_chr02	8516269	[A/G]	1067291740
Ca_Kabuli_NC_021161.1_8516291	Ca_kabuli_chr02	8516291	[T/G]	1067291741
Ca_Kabuli_NC_021161.1_8600511	Ca_kabuli_chr02	8600511	[A/T]	1067291742
Ca_Kabuli_NC_021161.1_8634831	Ca_kabuli_chr02	8634831	[A/G]	1067291743
Ca_Kabuli_NC_021161.1_8733429	Ca_kabuli_chr02	8733429	[A/T]	1067291744
Ca_Kabuli_NC_021161.1_8782786	Ca_kabuli_chr02	8782786	[T/C]	1067291745
Ca_Kabuli_NC_021161.1_8782791	Ca_kabuli_chr02	8782791	[T/C]	1067291746
Ca_Kabuli_NC_021161.1_8802121	Ca_kabuli_chr02	8802121	[T/C]	1067291747
Ca_Kabuli_NC_021161.1_9459001	Ca_kabuli_chr02	9459001	[T/G]	1067291748
Ca_Kabuli_NC_021161.1_9598708	Ca_kabuli_chr02	9598708	[A/C]	1067291749
Ca_Kabuli_NC_021161.1_9621950	Ca_kabuli_chr02	9621950	[A/C]	1067291750
Ca_Kabuli_NC_021161.1_9621953	Ca_kabuli_chr02	9621953	[A/C]	1067291751
Ca_Kabuli_NC_021161.1_9621986	Ca_kabuli_chr02	9621986	[T/G]	1067291752
Ca_Kabuli_NC_021161.1_9756907	Ca_kabuli_chr02	9756907	[G/T]	1067291753
Ca_Kabuli_NC_021161.1_9756955	Ca_kabuli_chr02	9756955	[G/A]	1067291754
Ca_Kabuli_NC_021161.1_9857985	Ca_kabuli_chr02	9857985	[T/G]	1067291755
Ca_Kabuli_NC_021161.1_9906081	Ca_kabuli_chr02	9906081	[T/G]	1067291756
Ca_Kabuli_NC_021161.1_9986816	Ca_kabuli_chr02	9986816	[T/G]	1067291757
Ca_Kabuli_NC_021161.1_10023259	Ca_kabuli_chr02	10023259	[T/G]	1067291758

SNP IDs	Chromosomes	Physical positions (bp)	SNPs	NCBI SNPdb accession IDs (NCBI_ss#)
Ca_Kabuli_NC_021161.1_10033970	Ca_kabuli_chr02	10033970	[T/G]	1067291759
Ca_Kabuli_NC_021161.1_10049340	Ca_kabuli_chr02	10049340	[G/T]	1067291760
Ca_Kabuli_NC_021161.1_10067451	Ca_kabuli_chr02	10067451	[A/G]	1067291761
Ca_Kabuli_NC_021161.1_10068454	Ca_kabuli_chr02	10068454	[C/A]	1067291762
Ca_Kabuli_NC_021161.1_10068455	Ca_kabuli_chr02	10068455	[T/G]	1067291763
Ca_Kabuli_NC_021161.1_10434697	Ca_kabuli_chr02	10434697	[T/G]	1067291764
Ca_Kabuli_NC_021161.1_10637651	Ca_kabuli_chr02	10637651	[A/C]	1067291765
Ca_Kabuli_NC_021161.1_10761032	Ca_kabuli_chr02	10761032	[A/G]	1067291766
Ca_Kabuli_NC_021161.1_10863335	Ca_kabuli_chr02	10863335	[T/C]	1067291767
Ca_Kabuli_NC_021161.1_10863413	Ca_kabuli_chr02	10863413	[A/C]	1067291768
Ca_Kabuli_NC_021161.1_10943245	Ca_kabuli_chr02	10943245	[A/C]	1067291769
Ca_Kabuli_NC_021161.1_10943421	Ca_kabuli_chr02	10943421	[A/T]	1067291770
Ca_Kabuli_NC_021161.1_11018696	Ca_kabuli_chr02	11018696	[T/C]	1067291771
Ca_Kabuli_NC_021161.1_11121406	Ca_kabuli_chr02	11121406	[T/G]	1067291772
Ca_Kabuli_NC_021161.1_11121441	Ca_kabuli_chr02	11121441	[A/C]	1067291773
Ca_Kabuli_NC_021161.1_11246842	Ca_kabuli_chr02	11246842	[G/A]	1067291774
Ca_Kabuli_NC_021161.1_11353931	Ca_kabuli_chr02	11353931	[T/C]	1067291775
Ca_Kabuli_NC_021161.1_11353934	Ca_kabuli_chr02	11353934	[A/T]	1067291776
Ca_Kabuli_NC_021161.1_11441401	Ca_kabuli_chr02	11441401	[T/C]	1067291777
Ca_Kabuli_NC_021161.1_11441435	Ca_kabuli_chr02	11441435	[A/G]	1067291778
Ca_Kabuli_NC_021161.1_11456773	Ca_kabuli_chr02	11456773	[A/C]	1067291779
Ca_Kabuli_NC_021161.1_11456976	Ca_kabuli_chr02	11456976	[T/A]	1067291780
Ca_Kabuli_NC_021161.1_11456995	Ca_kabuli_chr02	11456995	[T/G]	1067291781
Ca_Kabuli_NC_021161.1_11457021	Ca_kabuli_chr02	11457021	[T/C]	1067291782
Ca_Kabuli_NC_021161.1_11682390	Ca_kabuli_chr02	11682390	[T/C]	1067291783
Ca_Kabuli_NC_021161.1_11682433	Ca_kabuli_chr02	11682433	[A/G]	1067291784
Ca_Kabuli_NC_021161.1_11922886	Ca_kabuli_chr02	11922886	[T/C]	1067291785
Ca_Kabuli_NC_021161.1_11922903	Ca_kabuli_chr02	11922903	[C/G]	1067291786
Ca_Kabuli_NC_021161.1_11922922	Ca_kabuli_chr02	11922922	[A/G]	1067291787
Ca_Kabuli_NC_021161.1_12006214	Ca_kabuli_chr02	12006214	[A/C]	1067291788
Ca_Kabuli_NC_021161.1_12006236	Ca_kabuli_chr02	12006236	[T/A]	1067291789
Ca_Kabuli_NC_021161.1_12006243	Ca_kabuli_chr02	12006243	[A/C]	1067291790
Ca_Kabuli_NC_021161.1_12006260	Ca_kabuli_chr02	12006260	[T/A]	1067291791
Ca_Kabuli_NC_021161.1_12154019	Ca_kabuli_chr02	12154019	[T/G]	1067291792

SNP IDs	Chromosomes	Physical positions (bp)	SNPs	NCBI SNPdb accession IDs (NCBI_ss#)
Ca_Kabuli_NC_021161.1_12154074	Ca_kabuli_chr02	12154074	[A/C]	1067291793
Ca_Kabuli_NC_021161.1_12274519	Ca_kabuli_chr02	12274519	[T/C]	1067291794
Ca_Kabuli_NC_021161.1_12278521	Ca_kabuli_chr02	12278521	[A/T]	1067291795
Ca_Kabuli_NC_021161.1_12298536	Ca_kabuli_chr02	12298536	[T/A]	1067291796
Ca_Kabuli_NC_021161.1_12515511	Ca_kabuli_chr02	12515511	[T/G]	1067291797
Ca_Kabuli_NC_021161.1_12517618	Ca_kabuli_chr02	12517618	[A/G]	1067291798
Ca_Kabuli_NC_021161.1_12517629	Ca_kabuli_chr02	12517629	[A/G]	1067291799
Ca_Kabuli_NC_021161.1_12517654	Ca_kabuli_chr02	12517654	[A/G]	1067291800
Ca_Kabuli_NC_021161.1_12517665	Ca_kabuli_chr02	12517665	[A/G]	1067291801
Ca_Kabuli_NC_021161.1_12517674	Ca_kabuli_chr02	12517674	[A/G]	1067291802
Ca_Kabuli_NC_021161.1_12518273	Ca_kabuli_chr02	12518273	[T/G]	1067291803
Ca_Kabuli_NC_021161.1_12519390	Ca_kabuli_chr02	12519390	[T/C]	1067291804
Ca_Kabuli_NC_021161.1_12519400	Ca_kabuli_chr02	12519400	[A/C]	1067291805
Ca_Kabuli_NC_021161.1_12519406	Ca_kabuli_chr02	12519406	[A/G]	1067291806
Ca_Kabuli_NC_021161.1_12519410	Ca_kabuli_chr02	12519410	[A/G]	1067291807
Ca_Kabuli_NC_021161.1_12519430	Ca_kabuli_chr02	12519430	[T/A]	1067291808
Ca_Kabuli_NC_021161.1_12737860	Ca_kabuli_chr02	12737860	[A/C]	1067291809
Ca_Kabuli_NC_021161.1_12792699	Ca_kabuli_chr02	12792699	[T/G]	1067291810
Ca_Kabuli_NC_021161.1_12792706	Ca_kabuli_chr02	12792706	[T/C]	1067291811
Ca_Kabuli_NC_021161.1_12794446	Ca_kabuli_chr02	12794446	[T/A]	1067291812
Ca_Kabuli_NC_021161.1_12862857	Ca_kabuli_chr02	12862857	[T/C]	1067291813
Ca_Kabuli_NC_021161.1_13079528	Ca_kabuli_chr02	13079528	[T/A]	1067291814
Ca_Kabuli_NC_021161.1_13135203	Ca_kabuli_chr02	13135203	[T/A]	1067291815
Ca_Kabuli_NC_021161.1_13135230	Ca_kabuli_chr02	13135230	[A/G]	1067291816
Ca_Kabuli_NC_021161.1_13136109	Ca_kabuli_chr02	13136109	[T/C]	1067291817
Ca_Kabuli_NC_021161.1_13217756	Ca_kabuli_chr02	13217756	[A/G]	1067291818
Ca_Kabuli_NC_021161.1_13217758	Ca_kabuli_chr02	13217758	[T/C]	1067291819
Ca_Kabuli_NC_021161.1_13217809	Ca_kabuli_chr02	13217809	[T/C]	1067291820
Ca_Kabuli_NC_021161.1_13217903	Ca_kabuli_chr02	13217903	[A/C]	1067291821
Ca_Kabuli_NC_021161.1_13217904	Ca_kabuli_chr02	13217904	[G/T]	1067291822
Ca_Kabuli_NC_021161.1_13217905	Ca_kabuli_chr02	13217905	[A/C]	1067291823
Ca_Kabuli_NC_021161.1_13217906	Ca_kabuli_chr02	13217906	[T/C]	1067291824
Ca_Kabuli_NC_021161.1_13297592	Ca_kabuli_chr02	13297592	[T/G]	1067291825
Ca_Kabuli_NC_021161.1_13297594	Ca_kabuli_chr02	13297594	[T/G]	1067291826

SNP IDs	Chromosomes	Physical positions (bp)	SNPs	NCBI SNPdb accession IDs (NCBI_ss#)
Ca_Kabuli_NC_021161.1_13297596	Ca_kabuli_chr02	13297596	[G/A]	1067291827
Ca_Kabuli_NC_021161.1_13297600	Ca_kabuli_chr02	13297600	[C/T]	1067291828
Ca_Kabuli_NC_021161.1_13297603	Ca_kabuli_chr02	13297603	[A/G]	1067291829
Ca_Kabuli_NC_021161.1_13354975	Ca_kabuli_chr02	13354975	[T/G]	1067291830
Ca_Kabuli_NC_021161.1_13355140	Ca_kabuli_chr02	13355140	[C/A]	1067291831
Ca_Kabuli_NC_021161.1_13355647	Ca_kabuli_chr02	13355647	[A/C]	1067291832
Ca_Kabuli_NC_021161.1_13355677	Ca_kabuli_chr02	13355677	[T/C]	1067291833
Ca_Kabuli_NC_021161.1_13411523	Ca_kabuli_chr02	13411523	[A/C]	1067291834
Ca_Kabuli_NC_021161.1_13411540	Ca_kabuli_chr02	13411540	[T/G]	1067291835
Ca_Kabuli_NC_021161.1_13411543	Ca_kabuli_chr02	13411543	[T/G]	1067291836
Ca_Kabuli_NC_021161.1_13435061	Ca_kabuli_chr02	13435061	[A/T]	1067291837
Ca_Kabuli_NC_021161.1_13529048	Ca_kabuli_chr02	13529048	[A/G]	1067291838
Ca_Kabuli_NC_021161.1_13529086	Ca_kabuli_chr02	13529086	[T/C]	1067291839
Ca_Kabuli_NC_021161.1_13539273	Ca_kabuli_chr02	13539273	[C/A]	1067291840
Ca_Kabuli_NC_021161.1_13541139	Ca_kabuli_chr02	13541139	[T/G]	1067291841
Ca_Kabuli_NC_021161.1_13541148	Ca_kabuli_chr02	13541148	[G/T]	1067291842
Ca_Kabuli_NC_021161.1_13541156	Ca_kabuli_chr02	13541156	[A/G]	1067291843
Ca_Kabuli_NC_021161.1_13541171	Ca_kabuli_chr02	13541171	[A/G]	1067291844
Ca_Kabuli_NC_021161.1_13541195	Ca_kabuli_chr02	13541195	[A/T]	1067291845
Ca_Kabuli_NC_021161.1_13632204	Ca_kabuli_chr02	13632204	[T/G]	1067291846
Ca_Kabuli_NC_021161.1_13632221	Ca_kabuli_chr02	13632221	[T/G]	1067291847
Ca_Kabuli_NC_021161.1_13756720	Ca_kabuli_chr02	13756720	[A/G]	1067291848
Ca_Kabuli_NC_021161.1_13923504	Ca_kabuli_chr02	13923504	[A/G]	1067291849
Ca_Kabuli_NC_021161.1_13946495	Ca_kabuli_chr02	13946495	[A/G]	1067291850
Ca_Kabuli_NC_021161.1_14005994	Ca_kabuli_chr02	14005994	[T/C]	1067291851
Ca_Kabuli_NC_021161.1_14006013	Ca_kabuli_chr02	14006013	[A/G]	1067291852
Ca_Kabuli_NC_021161.1_14006027	Ca_kabuli_chr02	14006027	[A/G]	1067291853
Ca_Kabuli_NC_021161.1_14101836	Ca_kabuli_chr02	14101836	[A/T]	1067291854
Ca_Kabuli_NC_021161.1_14134347	Ca_kabuli_chr02	14134347	[G/T]	1067291855
Ca_Kabuli_NC_021161.1_14134364	Ca_kabuli_chr02	14134364	[A/G]	1067291856
Ca_Kabuli_NC_021161.1_14134371	Ca_kabuli_chr02	14134371	[A/C]	1067291857
Ca_Kabuli_NC_021161.1_14134676	Ca_kabuli_chr02	14134676	[T/C]	1067291858
Ca_Kabuli_NC_021161.1_14134681	Ca_kabuli_chr02	14134681	[C/A]	1067291859
Ca_Kabuli_NC_021161.1_14134712	Ca_kabuli_chr02	14134712	[A/G]	1067291860

SNP IDs	Chromosomes	Physical positions (bp)	SNPs	NCBI SNPdb accession IDs (NCBI_ss#)
Ca_Kabuli_NC_021161.1_14134719	Ca_kabuli_chr02	14134719	[A/G]	1067291861
Ca_Kabuli_NC_021161.1_14136036	Ca_kabuli_chr02	14136036	[A/C]	1067291862
Ca_Kabuli_NC_021161.1_14136050	Ca_kabuli_chr02	14136050	[T/C]	1067291863
Ca_Kabuli_NC_021161.1_14136061	Ca_kabuli_chr02	14136061	[A/C]	1067291864
Ca_Kabuli_NC_021161.1_14136223	Ca_kabuli_chr02	14136223	[A/C]	1067291865
Ca_Kabuli_NC_021161.1_14136229	Ca_kabuli_chr02	14136229	[A/C]	1067291866
Ca_Kabuli_NC_021161.1_14188615	Ca_kabuli_chr02	14188615	[T/G]	1067291867
Ca_Kabuli_NC_021161.1_15201537	Ca_kabuli_chr02	15201537	[G/T]	1067291868
Ca_Kabuli_NC_021161.1_15363526	Ca_kabuli_chr02	15363526	[G/A]	1067291869
Ca_Kabuli_NC_021161.1_15363570	Ca_kabuli_chr02	15363570	[C/A]	1067291870
Ca_Kabuli_NC_021161.1_15363572	Ca_kabuli_chr02	15363572	[T/A]	1067291871
Ca_Kabuli_NC_021161.1_15389426	Ca_kabuli_chr02	15389426	[T/C]	1067291872
Ca_Kabuli_NC_021161.1_15389455	Ca_kabuli_chr02	15389455	[A/G]	1067291873
Ca_Kabuli_NC_021161.1_15390723	Ca_kabuli_chr02	15390723	[A/C]	1067291874
Ca_Kabuli_NC_021161.1_15610615	Ca_kabuli_chr02	15610615	[A/T]	1067291875
Ca_Kabuli_NC_021161.1_15821481	Ca_kabuli_chr02	15821481	[G/A]	1067291876
Ca_Kabuli_NC_021161.1_15821500	Ca_kabuli_chr02	15821500	[A/C]	1067291877
Ca_Kabuli_NC_021161.1_15821568	Ca_kabuli_chr02	15821568	[T/G]	1067291878
Ca_Kabuli_NC_021161.1_16448465	Ca_kabuli_chr02	16448465	[A/T]	1067291879
Ca_Kabuli_NC_021161.1_16448473	Ca_kabuli_chr02	16448473	[T/G]	1067291880
Ca_Kabuli_NC_021161.1_16448478	Ca_kabuli_chr02	16448478	[A/T]	1067291881
Ca_Kabuli_NC_021161.1_16552569	Ca_kabuli_chr02	16552569	[T/C]	1067291882
Ca_Kabuli_NC_021161.1_16563121	Ca_kabuli_chr02	16563121	[A/T]	1067291883
Ca_Kabuli_NC_021161.1_16563123	Ca_kabuli_chr02	16563123	[C/T]	1067291884
Ca_Kabuli_NC_021161.1_16563125	Ca_kabuli_chr02	16563125	[A/T]	1067291885
Ca_Kabuli_NC_021161.1_16563133	Ca_kabuli_chr02	16563133	[A/T]	1067291886
Ca_Kabuli_NC_021161.1_16692101	Ca_kabuli_chr02	16692101	[T/C]	1067291887
Ca_Kabuli_NC_021161.1_16692126	Ca_kabuli_chr02	16692126	[A/G]	1067291888
Ca_Kabuli_NC_021161.1_16692134	Ca_kabuli_chr02	16692134	[T/C]	1067291889
Ca_Kabuli_NC_021161.1_16692137	Ca_kabuli_chr02	16692137	[A/C]	1067291890
Ca_Kabuli_NC_021161.1_17010035	Ca_kabuli_chr02	17010035	[C/A]	1067291891
Ca_Kabuli_NC_021161.1_17010037	Ca_kabuli_chr02	17010037	[G/A]	1067291892
Ca_Kabuli_NC_021161.1_17010038	Ca_kabuli_chr02	17010038	[C/T]	1067291893
Ca_Kabuli_NC_021161.1_17010039	Ca_kabuli_chr02	17010039	[A/C]	1067291894

SNP IDs	Chromosomes	Physical positions (bp)	SNPs	NCBI SNPdb accession IDs (NCBI_ss#)
Ca_Kabuli_NC_021161.1_17029096	Ca_kabuli_chr02	17029096	[G/C]	1067291895
Ca_Kabuli_NC_021161.1_17185183	Ca_kabuli_chr02	17185183	[A/T]	1067291896
Ca_Kabuli_NC_021161.1_17185186	Ca_kabuli_chr02	17185186	[A/G]	1067291897
Ca_Kabuli_NC_021161.1_17185200	Ca_kabuli_chr02	17185200	[A/C]	1067291898
Ca_Kabuli_NC_021161.1_17204167	Ca_kabuli_chr02	17204167	[C/T]	1067291899
Ca_Kabuli_NC_021161.1_17427646	Ca_kabuli_chr02	17427646	[T/G]	1067291900
Ca_Kabuli_NC_021161.1_17427679	Ca_kabuli_chr02	17427679	[T/G]	1067291901
Ca_Kabuli_NC_021161.1_17681915	Ca_kabuli_chr02	17681915	[C/T]	1067291902
Ca_Kabuli_NC_021161.1_17910565	Ca_kabuli_chr02	17910565	[T/G]	1067291903
Ca_Kabuli_NC_021161.1_18457266	Ca_kabuli_chr02	18457266	[A/G]	1067291904
Ca_Kabuli_NC_021161.1_18631374	Ca_kabuli_chr02	18631374	[T/G]	1067291905
Ca_Kabuli_NC_021161.1_18631385	Ca_kabuli_chr02	18631385	[T/G]	1067291906
Ca_Kabuli_NC_021161.1_18631471	Ca_kabuli_chr02	18631471	[T/A]	1067291907
Ca_Kabuli_NC_021161.1_18631478	Ca_kabuli_chr02	18631478	[A/C]	1067291908
Ca_Kabuli_NC_021161.1_18671571	Ca_kabuli_chr02	18671571	[C/T]	1067291909
Ca_Kabuli_NC_021161.1_18671572	Ca_kabuli_chr02	18671572	[C/T]	1067291910
Ca_Kabuli_NC_021161.1_18671574	Ca_kabuli_chr02	18671574	[G/A]	1067291911
Ca_Kabuli_NC_021161.1_18712473	Ca_kabuli_chr02	18712473	[C/T]	1067291912
Ca_Kabuli_NC_021161.1_19204733	Ca_kabuli_chr02	19204733	[C/T]	1067291913
Ca_Kabuli_NC_021161.1_19204759	Ca_kabuli_chr02	19204759	[T/A]	1067291914
Ca_Kabuli_NC_021161.1_19901544	Ca_kabuli_chr02	19901544	[A/C]	1067291915
Ca_Kabuli_NC_021161.1_19901593	Ca_kabuli_chr02	19901593	[G/A]	1067291916
Ca_Kabuli_NC_021161.1_19901595	Ca_kabuli_chr02	19901595	[T/C]	1067291917
Ca_Kabuli_NC_021161.1_20042847	Ca_kabuli_chr02	20042847	[T/C]	1067291918
Ca_Kabuli_NC_021161.1_20812096	Ca_kabuli_chr02	20812096	[G/A]	1067291919
Ca_Kabuli_NC_021161.1_20829560	Ca_kabuli_chr02	20829560	[T/G]	1067291920
Ca_Kabuli_NC_021161.1_20829582	Ca_kabuli_chr02	20829582	[T/A]	1067291921
Ca_Kabuli_NC_021161.1_21159158	Ca_kabuli_chr02	21159158	[A/T]	1067291922
Ca_Kabuli_NC_021161.1_21159254	Ca_kabuli_chr02	21159254	[C/T]	1067291923
Ca_Kabuli_NC_021161.1_21159275	Ca_kabuli_chr02	21159275	[G/A]	1067291924
Ca_Kabuli_NC_021161.1_21764792	Ca_kabuli_chr02	21764792	[T/C]	1067291925
Ca_Kabuli_NC_021161.1_21849874	Ca_kabuli_chr02	21849874	[A/T]	1067291926
Ca_Kabuli_NC_021161.1_21904437	Ca_kabuli_chr02	21904437	[T/C]	1067291927
Ca_Kabuli_NC_021161.1_22104801	Ca_kabuli_chr02	22104801	[G/A]	1067291928

SNP IDs	Chromosomes	Physical positions (bp)	SNPs	NCBI SNPdb accession IDs (NCBI_ss#)
Ca_Kabuli_NC_021161.1_22419663	Ca_kabuli_chr02	22419663	[T/C]	1067291929
Ca_Kabuli_NC_021161.1_22536190	Ca_kabuli_chr02	22536190	[A/G]	1067291930
Ca_Kabuli_NC_021161.1_22542135	Ca_kabuli_chr02	22542135	[T/G]	1067291931
Ca_Kabuli_NC_021161.1_22542274	Ca_kabuli_chr02	22542274	[G/T]	1067291932
Ca_Kabuli_NC_021161.1_22702701	Ca_kabuli_chr02	22702701	[A/G]	1067291933
Ca_Kabuli_NC_021161.1_22704098	Ca_kabuli_chr02	22704098	[T/G]	1067291934
Ca_Kabuli_NC_021161.1_22719678	Ca_kabuli_chr02	22719678	[T/A]	1067291935
Ca_Kabuli_NC_021161.1_22770275	Ca_kabuli_chr02	22770275	[A/C]	1067291936
Ca_Kabuli_NC_021161.1_22770306	Ca_kabuli_chr02	22770306	[T/C]	1067291937
Ca_Kabuli_NC_021161.1_22818416	Ca_kabuli_chr02	22818416	[T/C]	1067291938
Ca_Kabuli_NC_021161.1_22849526	Ca_kabuli_chr02	22849526	[C/G]	1067291939
Ca_Kabuli_NC_021161.1_23127798	Ca_kabuli_chr02	23127798	[T/C]	1067291940
Ca_Kabuli_NC_021161.1_23127810	Ca_kabuli_chr02	23127810	[A/C]	1067291941
Ca_Kabuli_NC_021161.1_23141573	Ca_kabuli_chr02	23141573	[A/C]	1067291942
Ca_Kabuli_NC_021161.1_23181177	Ca_kabuli_chr02	23181177	[A/C]	1067291943
Ca_Kabuli_NC_021161.1_23182319	Ca_kabuli_chr02	23182319	[A/G]	1067291944
Ca_Kabuli_NC_021161.1_23217505	Ca_kabuli_chr02	23217505	[A/T]	1067291945
Ca_Kabuli_NC_021161.1_23217508	Ca_kabuli_chr02	23217508	[A/C]	1067291946
Ca_Kabuli_NC_021161.1_23217524	Ca_kabuli_chr02	23217524	[A/C]	1067291947
Ca_Kabuli_NC_021161.1_23219939	Ca_kabuli_chr02	23219939	[C/A]	1067291948
Ca_Kabuli_NC_021161.1_23297194	Ca_kabuli_chr02	23297194	[T/G]	1067291949
Ca_Kabuli_NC_021161.1_23297203	Ca_kabuli_chr02	23297203	[T/A]	1067291950
Ca_Kabuli_NC_021161.1_23297341	Ca_kabuli_chr02	23297341	[A/C]	1067291951
Ca_Kabuli_NC_021161.1_23297458	Ca_kabuli_chr02	23297458	[A/G]	1067291952
Ca_Kabuli_NC_021161.1_23329362	Ca_kabuli_chr02	23329362	[T/C]	1067291953
Ca_Kabuli_NC_021161.1_23329385	Ca_kabuli_chr02	23329385	[T/C]	1067291954
Ca_Kabuli_NC_021161.1_23329392	Ca_kabuli_chr02	23329392	[A/C]	1067291955
Ca_Kabuli_NC_021161.1_23337832	Ca_kabuli_chr02	23337832	[C/A]	1067291956
Ca_Kabuli_NC_021161.1_23337834	Ca_kabuli_chr02	23337834	[A/C]	1067291957
Ca_Kabuli_NC_021161.1_23346218	Ca_kabuli_chr02	23346218	[G/A]	1067291958
Ca_Kabuli_NC_021161.1_23346299	Ca_kabuli_chr02	23346299	[A/C]	1067291959
Ca_Kabuli_NC_021161.1_23354434	Ca_kabuli_chr02	23354434	[A/C]	1067291960
Ca_Kabuli_NC_021161.1_23433522	Ca_kabuli_chr02	23433522	[A/G]	1067291961
Ca_Kabuli_NC_021161.1_23435792	Ca_kabuli_chr02	23435792	[A/G]	1067291962

SNP IDs	Chromosomes	Physical positions (bp)	SNPs	NCBI SNPdb accession IDs (NCBI_ss#)
Ca_Kabuli_NC_021161.1_23435886	Ca_kabuli_chr02	23435886	[A/C]	1067291963
Ca_Kabuli_NC_021161.1_23439889	Ca_kabuli_chr02	23439889	[A/G]	1067291964
Ca_Kabuli_NC_021161.1_23441701	Ca_kabuli_chr02	23441701	[T/A]	1067291965
Ca_Kabuli_NC_021161.1_23441737	Ca_kabuli_chr02	23441737	[A/C]	1067291966
Ca_Kabuli_NC_021161.1_23910254	Ca_kabuli_chr02	23910254	[T/C]	1067291967
Ca_Kabuli_NC_021161.1_23933608	Ca_kabuli_chr02	23933608	[G/C]	1067291968
Ca_Kabuli_NC_021161.1_23960995	Ca_kabuli_chr02	23960995	[T/C]	1067291969
Ca_Kabuli_NC_021161.1_24048033	Ca_kabuli_chr02	24048033	[T/G]	1067291970
Ca_Kabuli_NC_021161.1_24048084	Ca_kabuli_chr02	24048084	[A/C]	1067291971
Ca_Kabuli_NC_021161.1_24048087	Ca_kabuli_chr02	24048087	[A/C]	1067291972
Ca_Kabuli_NC_021161.1_24092328	Ca_kabuli_chr02	24092328	[T/C]	1067291973
Ca_Kabuli_NC_021161.1_24092331	Ca_kabuli_chr02	24092331	[T/C]	1067291974
Ca_Kabuli_NC_021161.1_24101339	Ca_kabuli_chr02	24101339	[A/C]	1067291975
Ca_Kabuli_NC_021161.1_24164862	Ca_kabuli_chr02	24164862	[A/C]	1067291976
Ca_Kabuli_NC_021161.1_24220954	Ca_kabuli_chr02	24220954	[A/C]	1067291977
Ca_Kabuli_NC_021161.1_24220955	Ca_kabuli_chr02	24220955	[G/T]	1067291978
Ca_Kabuli_NC_021161.1_24286153	Ca_kabuli_chr02	24286153	[C/A]	1067291979
Ca_Kabuli_NC_021161.1_24511121	Ca_kabuli_chr02	24511121	[G/A]	1067291980
Ca_Kabuli_NC_021161.1_24542913	Ca_kabuli_chr02	24542913	[T/A]	1067291981
Ca_Kabuli_NC_021161.1_24542917	Ca_kabuli_chr02	24542917	[A/C]	1067291982
Ca_Kabuli_NC_021161.1_24542934	Ca_kabuli_chr02	24542934	[A/G]	1067291983
Ca_Kabuli_NC_021161.1_24709026	Ca_kabuli_chr02	24709026	[T/G]	1067291984
Ca_Kabuli_NC_021161.1_24709295	Ca_kabuli_chr02	24709295	[G/A]	1067291985
Ca_Kabuli_NC_021161.1_24709315	Ca_kabuli_chr02	24709315	[T/G]	1067291986
Ca_Kabuli_NC_021161.1_24712004	Ca_kabuli_chr02	24712004	[T/C]	1067291987
Ca_Kabuli_NC_021161.1_24741144	Ca_kabuli_chr02	24741144	[T/A]	1067291988
Ca_Kabuli_NC_021161.1_24741159	Ca_kabuli_chr02	24741159	[T/G]	1067291989
Ca_Kabuli_NC_021161.1_24741195	Ca_kabuli_chr02	24741195	[C/T]	1067291990
Ca_Kabuli_NC_021161.1_24744708	Ca_kabuli_chr02	24744708	[T/G]	1067291991
Ca_Kabuli_NC_021161.1_24744717	Ca_kabuli_chr02	24744717	[T/G]	1067291992
Ca_Kabuli_NC_021161.1_24846391	Ca_kabuli_chr02	24846391	[A/C]	1067291993
Ca_Kabuli_NC_021161.1_24911088	Ca_kabuli_chr02	24911088	[A/G]	1067291994
Ca_Kabuli_NC_021161.1_24911135	Ca_kabuli_chr02	24911135	[A/G]	1067291995
Ca_Kabuli_NC_021161.1_25141453	Ca_kabuli_chr02	25141453	[T/C]	1067291996

SNP IDs	Chromosomes	Physical positions (bp)	SNPs	NCBI SNPdb accession IDs (NCBI_ss#)
Ca_Kabuli_NC_021161.1_25141456	Ca_kabuli_chr02	25141456	[T/C]	1067291997
Ca_Kabuli_NC_021161.1_25141465	Ca_kabuli_chr02	25141465	[T/C]	1067291998
Ca_Kabuli_NC_021161.1_25141474	Ca_kabuli_chr02	25141474	[T/C]	1067291999
Ca_Kabuli_NC_021161.1_25141487	Ca_kabuli_chr02	25141487	[A/G]	1067292000
Ca_Kabuli_NC_021161.1_25141493	Ca_kabuli_chr02	25141493	[A/T]	1067292001
Ca_Kabuli_NC_021161.1_25141504	Ca_kabuli_chr02	25141504	[T/C]	1067292002
Ca_Kabuli_NC_021161.1_25141506	Ca_kabuli_chr02	25141506	[A/T]	1067292003
Ca_Kabuli_NC_021161.1_25249607	Ca_kabuli_chr02	25249607	[T/C]	1067292004
Ca_Kabuli_NC_021161.1_25249615	Ca_kabuli_chr02	25249615	[T/G]	1067292005
Ca_Kabuli_NC_021161.1_25249618	Ca_kabuli_chr02	25249618	[A/G]	1067292006
Ca_Kabuli_NC_021161.1_25249635	Ca_kabuli_chr02	25249635	[A/C]	1067292007
Ca_Kabuli_NC_021161.1_25317252	Ca_kabuli_chr02	25317252	[T/G]	1067292008
Ca_Kabuli_NC_021161.1_25321120	Ca_kabuli_chr02	25321120	[T/G]	1067292009
Ca_Kabuli_NC_021161.1_25321812	Ca_kabuli_chr02	25321812	[T/A]	1067292010
Ca_Kabuli_NC_021161.1_25321837	Ca_kabuli_chr02	25321837	[A/C]	1067292011
Ca_Kabuli_NC_021161.1_25349105	Ca_kabuli_chr02	25349105	[G/T]	1067292012
Ca_Kabuli_NC_021161.1_25508879	Ca_kabuli_chr02	25508879	[A/C]	1067292013
Ca_Kabuli_NC_021161.1_25508914	Ca_kabuli_chr02	25508914	[A/C]	1067292014
Ca_Kabuli_NC_021161.1_25508941	Ca_kabuli_chr02	25508941	[A/T]	1067292015
Ca_Kabuli_NC_021161.1_25508973	Ca_kabuli_chr02	25508973	[T/C]	1067292016
Ca_Kabuli_NC_021161.1_25725665	Ca_kabuli_chr02	25725665	[G/T]	1067292017
Ca_Kabuli_NC_021161.1_25725688	Ca_kabuli_chr02	25725688	[G/A]	1067292018
Ca_Kabuli_NC_021161.1_25725692	Ca_kabuli_chr02	25725692	[C/T]	1067292019
Ca_Kabuli_NC_021161.1_25941062	Ca_kabuli_chr02	25941062	[A/C]	1067292020
Ca_Kabuli_NC_021161.1_25941072	Ca_kabuli_chr02	25941072	[T/C]	1067292021
Ca_Kabuli_NC_021161.1_25984147	Ca_kabuli_chr02	25984147	[A/G]	1067292022
Ca_Kabuli_NC_021161.1_25984184	Ca_kabuli_chr02	25984184	[A/G]	1067292023
Ca_Kabuli_NC_021161.1_25984208	Ca_kabuli_chr02	25984208	[T/C]	1067292024
Ca_Kabuli_NC_021161.1_26025852	Ca_kabuli_chr02	26025852	[A/G]	1067292025
Ca_Kabuli_NC_021161.1_26025885	Ca_kabuli_chr02	26025885	[A/G]	1067292026
Ca_Kabuli_NC_021161.1_26035673	Ca_kabuli_chr02	26035673	[C/T]	1067292027
Ca_Kabuli_NC_021161.1_26035674	Ca_kabuli_chr02	26035674	[G/C]	1067292028
Ca_Kabuli_NC_021161.1_26035675	Ca_kabuli_chr02	26035675	[G/T]	1067292029
Ca_Kabuli_NC_021161.1_26051628	Ca_kabuli_chr02	26051628	[A/T]	1067292030

SNP IDs	Chromosomes	Physical positions (bp)	SNPs	NCBI SNPdb accession IDs (NCBI_ss#)
Ca_Kabuli_NC_021161.1_26051650	Ca_kabuli_chr02	26051650	[G/T]	1067292031
Ca_Kabuli_NC_021161.1_26051657	Ca_kabuli_chr02	26051657	[A/T]	1067292032
Ca_Kabuli_NC_021161.1_26051659	Ca_kabuli_chr02	26051659	[T/G]	1067292033
Ca_Kabuli_NC_021161.1_26061758	Ca_kabuli_chr02	26061758	[T/A]	1067292034
Ca_Kabuli_NC_021161.1_26092916	Ca_kabuli_chr02	26092916	[C/A]	1067292035
Ca_Kabuli_NC_021161.1_26092997	Ca_kabuli_chr02	26092997	[A/C]	1067292036
Ca_Kabuli_NC_021161.1_26121429	Ca_kabuli_chr02	26121429	[A/G]	1067292037
Ca_Kabuli_NC_021161.1_26121431	Ca_kabuli_chr02	26121431	[G/T]	1067292038
Ca_Kabuli_NC_021161.1_26121448	Ca_kabuli_chr02	26121448	[C/T]	1067292039
Ca_Kabuli_NC_021161.1_26121519	Ca_kabuli_chr02	26121519	[T/G]	1067292040
Ca_Kabuli_NC_021161.1_26417225	Ca_kabuli_chr02	26417225	[C/A]	1067292041
Ca_Kabuli_NC_021161.1_26417226	Ca_kabuli_chr02	26417226	[T/G]	1067292042
Ca_Kabuli_NC_021161.1_26457480	Ca_kabuli_chr02	26457480	[G/A]	1067292043
Ca_Kabuli_NC_021161.1_26706735	Ca_kabuli_chr02	26706735	[A/C]	1067292044
Ca_Kabuli_NC_021161.1_26706740	Ca_kabuli_chr02	26706740	[C/A]	1067292045
Ca_Kabuli_NC_021161.1_26706752	Ca_kabuli_chr02	26706752	[C/T]	1067292046
Ca_Kabuli_NC_021161.1_26706782	Ca_kabuli_chr02	26706782	[G/A]	1067292047
Ca_Kabuli_NC_021161.1_26832618	Ca_kabuli_chr02	26832618	[G/T]	1067292048
Ca_Kabuli_NC_021161.1_26871568	Ca_kabuli_chr02	26871568	[C/A]	1067292049
Ca_Kabuli_NC_021161.1_26871569	Ca_kabuli_chr02	26871569	[T/G]	1067292050
Ca_Kabuli_NC_021161.1_26871570	Ca_kabuli_chr02	26871570	[T/A]	1067292051
Ca_Kabuli_NC_021161.1_26871571	Ca_kabuli_chr02	26871571	[C/T]	1067292052
Ca_Kabuli_NC_021161.1_26875498	Ca_kabuli_chr02	26875498	[A/T]	1067292053
Ca_Kabuli_NC_021161.1_27056842	Ca_kabuli_chr02	27056842	[T/C]	1067292054
Ca_Kabuli_NC_021161.1_27056855	Ca_kabuli_chr02	27056855	[T/G]	1067292055
Ca_Kabuli_NC_021161.1_27057554	Ca_kabuli_chr02	27057554	[T/G]	1067292056
Ca_Kabuli_NC_021161.1_27063609	Ca_kabuli_chr02	27063609	[A/C]	1067292057
Ca_Kabuli_NC_021161.1_27063610	Ca_kabuli_chr02	27063610	[G/T]	1067292058
Ca_Kabuli_NC_021161.1_27063723	Ca_kabuli_chr02	27063723	[T/A]	1067292059
Ca_Kabuli_NC_021161.1_27091401	Ca_kabuli_chr02	27091401	[A/G]	1067292060
Ca_Kabuli_NC_021161.1_27091403	Ca_kabuli_chr02	27091403	[T/C]	1067292061
Ca_Kabuli_NC_021161.1_27130718	Ca_kabuli_chr02	27130718	[T/G]	1067292062
Ca_Kabuli_NC_021161.1_27130753	Ca_kabuli_chr02	27130753	[A/C]	1067292063
Ca_Kabuli_NC_021161.1_27172568	Ca_kabuli_chr02	27172568	[G/A]	1067292064

SNP IDs	Chromosomes	Physical positions (bp)	SNPs	NCBI SNPdb accession IDs (NCBI_ss#)
Ca_Kabuli_NC_021161.1_27172580	Ca_kabuli_chr02	27172580	[G/A]	1067292065
Ca_Kabuli_NC_021161.1_27172582	Ca_kabuli_chr02	27172582	[A/G]	1067292066
Ca_Kabuli_NC_021161.1_27183708	Ca_kabuli_chr02	27183708	[A/C]	1067292067
Ca_Kabuli_NC_021161.1_27254655	Ca_kabuli_chr02	27254655	[C/T]	1067292068
Ca_Kabuli_NC_021161.1_27336078	Ca_kabuli_chr02	27336078	[T/C]	1067292069
Ca_Kabuli_NC_021161.1_27373969	Ca_kabuli_chr02	27373969	[A/T]	1067292070
Ca_Kabuli_NC_021161.1_27374109	Ca_kabuli_chr02	27374109	[A/C]	1067292071
Ca_Kabuli_NC_021161.1_27623253	Ca_kabuli_chr02	27623253	[A/C]	1067292072
Ca_Kabuli_NC_021161.1_27693643	Ca_kabuli_chr02	27693643	[T/G]	1067292073
Ca_Kabuli_NC_021161.1_27693652	Ca_kabuli_chr02	27693652	[T/C]	1067292074
Ca_Kabuli_NC_021161.1_27693703	Ca_kabuli_chr02	27693703	[A/G]	1067292075
Ca_Kabuli_NC_021161.1_27693707	Ca_kabuli_chr02	27693707	[T/C]	1067292076
Ca_Kabuli_NC_021161.1_27703354	Ca_kabuli_chr02	27703354	[A/G]	1067292077
Ca_Kabuli_NC_021161.1_27757565	Ca_kabuli_chr02	27757565	[G/T]	1067292078
Ca_Kabuli_NC_021161.1_27757573	Ca_kabuli_chr02	27757573	[T/C]	1067292079
Ca_Kabuli_NC_021161.1_27757800	Ca_kabuli_chr02	27757800	[T/C]	1067292080
Ca_Kabuli_NC_021161.1_27758034	Ca_kabuli_chr02	27758034	[T/C]	1067292081
Ca_Kabuli_NC_021161.1_27766605	Ca_kabuli_chr02	27766605	[A/C]	1067292082
Ca_Kabuli_NC_021161.1_27835859	Ca_kabuli_chr02	27835859	[G/T]	1067292083
Ca_Kabuli_NC_021161.1_27847700	Ca_kabuli_chr02	27847700	[A/C]	1067292084
Ca_Kabuli_NC_021161.1_27899966	Ca_kabuli_chr02	27899966	[A/T]	1067292085
Ca_Kabuli_NC_021161.1_27928698	Ca_kabuli_chr02	27928698	[A/T]	1067292086
Ca_Kabuli_NC_021161.1_27987041	Ca_kabuli_chr02	27987041	[A/T]	1067292087
Ca_Kabuli_NC_021161.1_27987046	Ca_kabuli_chr02	27987046	[A/G]	1067292088
Ca_Kabuli_NC_021161.1_27987048	Ca_kabuli_chr02	27987048	[T/C]	1067292089
Ca_Kabuli_NC_021161.1_28018175	Ca_kabuli_chr02	28018175	[G/C]	1067292090
Ca_Kabuli_NC_021161.1_28022987	Ca_kabuli_chr02	28022987	[T/G]	1067292091
Ca_Kabuli_NC_021161.1_28022995	Ca_kabuli_chr02	28022995	[T/A]	1067292092
Ca_Kabuli_NC_021161.1_28022999	Ca_kabuli_chr02	28022999	[A/C]	1067292093
Ca_Kabuli_NC_021161.1_28023007	Ca_kabuli_chr02	28023007	[T/C]	1067292094
Ca_Kabuli_NC_021161.1_28060919	Ca_kabuli_chr02	28060919	[T/G]	1067292095
Ca_Kabuli_NC_021161.1_28097382	Ca_kabuli_chr02	28097382	[G/T]	1067292096
Ca_Kabuli_NC_021161.1_28097386	Ca_kabuli_chr02	28097386	[C/A]	1067292097
Ca_Kabuli_NC_021161.1_28097391	Ca_kabuli_chr02	28097391	[A/G]	1067292098

SNP IDs	Chromosomes	Physical positions (bp)	SNPs	NCBI SNPdb accession IDs (NCBI_ss#)
Ca_Kabuli_NC_021161.1_28097395	Ca_kabuli_chr02	28097395	[T/C]	1067292099
Ca_Kabuli_NC_021161.1_28101712	Ca_kabuli_chr02	28101712	[T/C]	1067292100
Ca_Kabuli_NC_021161.1_28101727	Ca_kabuli_chr02	28101727	[A/G]	1067292101
Ca_Kabuli_NC_021161.1_28101732	Ca_kabuli_chr02	28101732	[T/C]	1067292102
Ca_Kabuli_NC_021161.1_28101741	Ca_kabuli_chr02	28101741	[A/C]	1067292103
Ca_Kabuli_NC_021161.1_28101828	Ca_kabuli_chr02	28101828	[T/C]	1067292104
Ca_Kabuli_NC_021161.1_28101831	Ca_kabuli_chr02	28101831	[T/G]	1067292105
Ca_Kabuli_NC_021161.1_28101852	Ca_kabuli_chr02	28101852	[A/C]	1067292106
Ca_Kabuli_NC_021161.1_28228930	Ca_kabuli_chr02	28228930	[A/C]	1067292107
Ca_Kabuli_NC_021161.1_28228939	Ca_kabuli_chr02	28228939	[A/C]	1067292108
Ca_Kabuli_NC_021161.1_28229093	Ca_kabuli_chr02	28229093	[A/C]	1067292109
Ca_Kabuli_NC_021161.1_28665207	Ca_kabuli_chr02	28665207	[A/T]	1067292110
Ca_Kabuli_NC_021161.1_28665211	Ca_kabuli_chr02	28665211	[T/C]	1067292111
Ca_Kabuli_NC_021161.1_28665212	Ca_kabuli_chr02	28665212	[G/T]	1067292112
Ca_Kabuli_NC_021161.1_28665239	Ca_kabuli_chr02	28665239	[G/T]	1067292113
Ca_Kabuli_NC_021161.1_28897971	Ca_kabuli_chr02	28897971	[T/G]	1067292114
Ca_Kabuli_NC_021161.1_28897976	Ca_kabuli_chr02	28897976	[T/G]	1067292115
Ca_Kabuli_NC_021161.1_28949900	Ca_kabuli_chr02	28949900	[C/T]	1067292116
Ca_Kabuli_NC_021161.1_28949901	Ca_kabuli_chr02	28949901	[T/C]	1067292117
Ca_Kabuli_NC_021161.1_29336266	Ca_kabuli_chr02	29336266	[A/G]	1067292118
Ca_Kabuli_NC_021161.1_29710344	Ca_kabuli_chr02	29710344	[A/G]	1067292119
Ca_Kabuli_NC_021161.1_29710346	Ca_kabuli_chr02	29710346	[T/C]	1067292120
Ca_Kabuli_NC_021161.1_29711375	Ca_kabuli_chr02	29711375	[T/G]	1067292121
Ca_Kabuli_NC_021161.1_29711387	Ca_kabuli_chr02	29711387	[A/C]	1067292122
Ca_Kabuli_NC_021161.1_29712020	Ca_kabuli_chr02	29712020	[A/G]	1067292123
Ca_Kabuli_NC_021161.1_29712050	Ca_kabuli_chr02	29712050	[A/G]	1067292124
Ca_Kabuli_NC_021161.1_29712056	Ca_kabuli_chr02	29712056	[A/G]	1067292125
Ca_Kabuli_NC_021161.1_29798328	Ca_kabuli_chr02	29798328	[A/C]	1067292126
Ca_Kabuli_NC_021161.1_29948418	Ca_kabuli_chr02	29948418	[A/G]	1067292127
Ca_Kabuli_NC_021161.1_29999079	Ca_kabuli_chr02	29999079	[A/T]	1067292128
Ca_Kabuli_NC_021161.1_30004243	Ca_kabuli_chr02	30004243	[A/C]	1067292129
Ca_Kabuli_NC_021161.1_30091140	Ca_kabuli_chr02	30091140	[C/T]	1067292130
Ca_Kabuli_NC_021161.1_30091146	Ca_kabuli_chr02	30091146	[A/T]	1067292131
Ca_Kabuli_NC_021161.1_30091161	Ca_kabuli_chr02	30091161	[A/C]	1067292132

SNP IDs	Chromosomes	Physical positions (bp)	SNPs	NCBI SNPdb accession IDs (NCBI_ss#)
Ca_Kabuli_NC_021161.1_30091173	Ca_kabuli_chr02	30091173	[C/T]	1067292133
Ca_Kabuli_NC_021161.1_30091263	Ca_kabuli_chr02	30091263	[A/C]	1067292134
Ca_Kabuli_NC_021161.1_30133224	Ca_kabuli_chr02	30133224	[A/G]	1067292135
Ca_Kabuli_NC_021161.1_30335130	Ca_kabuli_chr02	30335130	[T/G]	1067292136
Ca_Kabuli_NC_021161.1_30335160	Ca_kabuli_chr02	30335160	[T/C]	1067292137
Ca_Kabuli_NC_021161.1_30335194	Ca_kabuli_chr02	30335194	[A/G]	1067292138
Ca_Kabuli_NC_021161.1_30335212	Ca_kabuli_chr02	30335212	[T/A]	1067292139
Ca_Kabuli_NC_021161.1_30389175	Ca_kabuli_chr02	30389175	[A/C]	1067292140
Ca_Kabuli_NC_021161.1_30408063	Ca_kabuli_chr02	30408063	[A/T]	1067292141
Ca_Kabuli_NC_021161.1_30408087	Ca_kabuli_chr02	30408087	[A/G]	1067292142
Ca_Kabuli_NC_021161.1_30422750	Ca_kabuli_chr02	30422750	[T/G]	1067292143
Ca_Kabuli_NC_021161.1_30422768	Ca_kabuli_chr02	30422768	[A/G]	1067292144
Ca_Kabuli_NC_021161.1_30422772	Ca_kabuli_chr02	30422772	[A/C]	1067292145
Ca_Kabuli_NC_021161.1_30423017	Ca_kabuli_chr02	30423017	[A/C]	1067292146
Ca_Kabuli_NC_021161.1_30423031	Ca_kabuli_chr02	30423031	[T/C]	1067292147
Ca_Kabuli_NC_021161.1_30702078	Ca_kabuli_chr02	30702078	[T/G]	1067292148
Ca_Kabuli_NC_021161.1_30702097	Ca_kabuli_chr02	30702097	[T/G]	1067292149
Ca_Kabuli_NC_021161.1_30867559	Ca_kabuli_chr02	30867559	[T/A]	1067292150
Ca_Kabuli_NC_021161.1_30867590	Ca_kabuli_chr02	30867590	[A/T]	1067292151
Ca_Kabuli_NC_021161.1_30867592	Ca_kabuli_chr02	30867592	[T/C]	1067292152
Ca_Kabuli_NC_021161.1_30867595	Ca_kabuli_chr02	30867595	[A/C]	1067292153
Ca_Kabuli_NC_021161.1_30868458	Ca_kabuli_chr02	30868458	[T/G]	1067292154
Ca_Kabuli_NC_021161.1_30868461	Ca_kabuli_chr02	30868461	[A/G]	1067292155
Ca_Kabuli_NC_021161.1_30880119	Ca_kabuli_chr02	30880119	[A/C]	1067292156
Ca_Kabuli_NC_021161.1_30903543	Ca_kabuli_chr02	30903543	[G/A]	1067292157
Ca_Kabuli_NC_021161.1_30903557	Ca_kabuli_chr02	30903557	[G/A]	1067292158
Ca_Kabuli_NC_021161.1_30903566	Ca_kabuli_chr02	30903566	[T/A]	1067292159
Ca_Kabuli_NC_021161.1_30907564	Ca_kabuli_chr02	30907564	[A/C]	1067292160
Ca_Kabuli_NC_021161.1_30907567	Ca_kabuli_chr02	30907567	[A/C]	1067292161
Ca_Kabuli_NC_021161.1_30918734	Ca_kabuli_chr02	30918734	[G/C]	1067292162
Ca_Kabuli_NC_021161.1_30920461	Ca_kabuli_chr02	30920461	[C/T]	1067292163
Ca_Kabuli_NC_021161.1_30921092	Ca_kabuli_chr02	30921092	[C/A]	1067292164
Ca_Kabuli_NC_021161.1_30921994	Ca_kabuli_chr02	30921994	[T/C]	1067292165
Ca_Kabuli_NC_021161.1_30925755	Ca_kabuli_chr02	30925755	[A/T]	1067292166

SNP IDs	Chromosomes	Physical positions (bp)	SNPs	NCBI SNPdb accession IDs (NCBI_ss#)
Ca_Kabuli_NC_021161.1_30926690	Ca_kabuli_chr02	30926690	[T/G]	1067292167
Ca_Kabuli_NC_021161.1_30928725	Ca_kabuli_chr02	30928725	[A/G]	1067292168
Ca_Kabuli_NC_021161.1_30950434	Ca_kabuli_chr02	30950434	[A/T]	1067292169
Ca_Kabuli_NC_021161.1_30950703	Ca_kabuli_chr02	30950703	[C/T]	1067292170
Ca_Kabuli_NC_021161.1_30951724	Ca_kabuli_chr02	30951724	[T/A]	1067292171
Ca_Kabuli_NC_021161.1_30954305	Ca_kabuli_chr02	30954305	[T/G]	1067292172
Ca_Kabuli_NC_021161.1_30954317	Ca_kabuli_chr02	30954317	[G/A]	1067292173
Ca_Kabuli_NC_021161.1_30977522	Ca_kabuli_chr02	30977522	[A/T]	1067292174
Ca_Kabuli_NC_021161.1_30977528	Ca_kabuli_chr02	30977528	[C/T]	1067292175
Ca_Kabuli_NC_021161.1_30977572	Ca_kabuli_chr02	30977572	[A/G]	1067292176
Ca_Kabuli_NC_021161.1_30997935	Ca_kabuli_chr02	30997935	[T/G]	1067292177
Ca_Kabuli_NC_021161.1_31005845	Ca_kabuli_chr02	31005845	[C/T]	1067292178
Ca_Kabuli_NC_021161.1_31005847	Ca_kabuli_chr02	31005847	[T/G]	1067292179
Ca_Kabuli_NC_021161.1_31199538	Ca_kabuli_chr02	31199538	[T/A]	1067292180
Ca_Kabuli_NC_021161.1_31205503	Ca_kabuli_chr02	31205503	[A/G]	1067292181
Ca_Kabuli_NC_021161.1_31205617	Ca_kabuli_chr02	31205617	[A/T]	1067292182
Ca_Kabuli_NC_021161.1_31205639	Ca_kabuli_chr02	31205639	[T/G]	1067292183
Ca_Kabuli_NC_021161.1_31427865	Ca_kabuli_chr02	31427865	[A/G]	1067292184
Ca_Kabuli_NC_021161.1_31427883	Ca_kabuli_chr02	31427883	[A/G]	1067292185
Ca_Kabuli_NC_021161.1_31427886	Ca_kabuli_chr02	31427886	[A/G]	1067292186
Ca_Kabuli_NC_021161.1_31428417	Ca_kabuli_chr02	31428417	[T/C]	1067292187
Ca_Kabuli_NC_021161.1_31534265	Ca_kabuli_chr02	31534265	[A/C]	1067292188
Ca_Kabuli_NC_021161.1_31534283	Ca_kabuli_chr02	31534283	[G/A]	1067292189
Ca_Kabuli_NC_021161.1_31686697	Ca_kabuli_chr02	31686697	[C/A]	1067292190
Ca_Kabuli_NC_021161.1_31686714	Ca_kabuli_chr02	31686714	[C/T]	1067292191
Ca_Kabuli_NC_021161.1_31686744	Ca_kabuli_chr02	31686744	[G/C]	1067292192
Ca_Kabuli_NC_021161.1_31686751	Ca_kabuli_chr02	31686751	[A/T]	1067292193
Ca_Kabuli_NC_021161.1_31823306	Ca_kabuli_chr02	31823306	[T/A]	1067292194
Ca_Kabuli_NC_021161.1_31837712	Ca_kabuli_chr02	31837712	[A/G]	1067292195
Ca_Kabuli_NC_021161.1_31837724	Ca_kabuli_chr02	31837724	[T/C]	1067292196
Ca_Kabuli_NC_021161.1_31837731	Ca_kabuli_chr02	31837731	[A/C]	1067292197
Ca_Kabuli_NC_021161.1_31908639	Ca_kabuli_chr02	31908639	[A/C]	1067292198
Ca_Kabuli_NC_021161.1_31908666	Ca_kabuli_chr02	31908666	[A/G]	1067292199
Ca_Kabuli_NC_021161.1_31908845	Ca_kabuli_chr02	31908845	[T/G]	1067292200

SNP IDs	Chromosomes	Physical positions (bp)	SNPs	NCBI SNPdb accession IDs (NCBI_ss#)
Ca_Kabuli_NC_021161.1_31975310	Ca_kabuli_chr02	31975310	[A/G]	1067292201
Ca_Kabuli_NC_021161.1_31975322	Ca_kabuli_chr02	31975322	[A/C]	1067292202
Ca_Kabuli_NC_021161.1_31975327	Ca_kabuli_chr02	31975327	[A/G]	1067292203
Ca_Kabuli_NC_021161.1_31975343	Ca_kabuli_chr02	31975343	[T/G]	1067292204
Ca_Kabuli_NC_021161.1_32034289	Ca_kabuli_chr02	32034289	[G/A]	1067292205
Ca_Kabuli_NC_021161.1_32058615	Ca_kabuli_chr02	32058615	[T/G]	1067292206
Ca_Kabuli_NC_021161.1_32096180	Ca_kabuli_chr02	32096180	[C/G]	1067292207
Ca_Kabuli_NC_021161.1_32096186	Ca_kabuli_chr02	32096186	[T/A]	1067292208
Ca_Kabuli_NC_021161.1_32096192	Ca_kabuli_chr02	32096192	[A/C]	1067292209
Ca_Kabuli_NC_021161.1_32096226	Ca_kabuli_chr02	32096226	[A/G]	1067292210
Ca_Kabuli_NC_021161.1_32099907	Ca_kabuli_chr02	32099907	[T/C]	1067292211
Ca_Kabuli_NC_021161.1_32119094	Ca_kabuli_chr02	32119094	[T/A]	1067292212
Ca_Kabuli_NC_021161.1_32119331	Ca_kabuli_chr02	32119331	[A/T]	1067292213
Ca_Kabuli_NC_021161.1_32121949	Ca_kabuli_chr02	32121949	[C/G]	1067292214
Ca_Kabuli_NC_021161.1_32129260	Ca_kabuli_chr02	32129260	[T/A]	1067292215
Ca_Kabuli_NC_021161.1_32280969	Ca_kabuli_chr02	32280969	[T/A]	1067292216
Ca_Kabuli_NC_021161.1_32283139	Ca_kabuli_chr02	32283139	[G/T]	1067292217
Ca_Kabuli_NC_021161.1_32283172	Ca_kabuli_chr02	32283172	[A/G]	1067292218
Ca_Kabuli_NC_021161.1_32348054	Ca_kabuli_chr02	32348054	[A/T]	1067292219
Ca_Kabuli_NC_021161.1_32348870	Ca_kabuli_chr02	32348870	[C/T]	1067292220
Ca_Kabuli_NC_021161.1_32352022	Ca_kabuli_chr02	32352022	[C/T]	1067292221
Ca_Kabuli_NC_021161.1_32352044	Ca_kabuli_chr02	32352044	[T/C]	1067292222
Ca_Kabuli_NC_021161.1_32352045	Ca_kabuli_chr02	32352045	[G/A]	1067292223
Ca_Kabuli_NC_021161.1_32481983	Ca_kabuli_chr02	32481983	[T/G]	1067292224
Ca_Kabuli_NC_021161.1_32482739	Ca_kabuli_chr02	32482739	[C/T]	1067292225
Ca_Kabuli_NC_021161.1_32506300	Ca_kabuli_chr02	32506300	[T/G]	1067292226
Ca_Kabuli_NC_021161.1_32642277	Ca_kabuli_chr02	32642277	[T/C]	1067292227
Ca_Kabuli_NC_021161.1_32670089	Ca_kabuli_chr02	32670089	[C/A]	1067292228
Ca_Kabuli_NC_021161.1_32670090	Ca_kabuli_chr02	32670090	[T/G]	1067292229
Ca_Kabuli_NC_021161.1_32670181	Ca_kabuli_chr02	32670181	[T/G]	1067292230
Ca_Kabuli_NC_021161.1_32742369	Ca_kabuli_chr02	32742369	[A/G]	1067292231
Ca_Kabuli_NC_021161.1_32742428	Ca_kabuli_chr02	32742428	[A/G]	1067292232
Ca_Kabuli_NC_021161.1_32781827	Ca_kabuli_chr02	32781827	[A/C]	1067292233
Ca_Kabuli_NC_021161.1_32781914	Ca_kabuli_chr02	32781914	[A/C]	1067292234

SNP IDs	Chromosomes	Physical positions (bp)	SNPs	NCBI SNPdb accession IDs (NCBI_ss#)
Ca_Kabuli_NC_021161.1_32844731	Ca_kabuli_chr02	32844731	[C/G]	1067292235
Ca_Kabuli_NC_021161.1_32844733	Ca_kabuli_chr02	32844733	[T/G]	1067292236
Ca_Kabuli_NC_021161.1_32844735	Ca_kabuli_chr02	32844735	[C/T]	1067292237
Ca_Kabuli_NC_021161.1_32844739	Ca_kabuli_chr02	32844739	[G/C]	1067292238
Ca_Kabuli_NC_021161.1_32902901	Ca_kabuli_chr02	32902901	[A/G]	1067292239
Ca_Kabuli_NC_021161.1_32934618	Ca_kabuli_chr02	32934618	[A/C]	1067292240
Ca_Kabuli_NC_021161.1_32936857	Ca_kabuli_chr02	32936857	[T/G]	1067292241
Ca_Kabuli_NC_021161.1_32936865	Ca_kabuli_chr02	32936865	[A/C]	1067292242
Ca_Kabuli_NC_021161.1_32936925	Ca_kabuli_chr02	32936925	[T/C]	1067292243
Ca_Kabuli_NC_021161.1_32936931	Ca_kabuli_chr02	32936931	[T/A]	1067292244
Ca_Kabuli_NC_021161.1_32937000	Ca_kabuli_chr02	32937000	[A/C]	1067292245
Ca_Kabuli_NC_021161.1_32957496	Ca_kabuli_chr02	32957496	[T/C]	1067292246
Ca_Kabuli_NC_021161.1_32957534	Ca_kabuli_chr02	32957534	[T/A]	1067292247
Ca_Kabuli_NC_021161.1_33103130	Ca_kabuli_chr02	33103130	[A/G]	1067292248
Ca_Kabuli_NC_021161.1_33103134	Ca_kabuli_chr02	33103134	[T/A]	1067292249
Ca_Kabuli_NC_021161.1_33103180	Ca_kabuli_chr02	33103180	[T/A]	1067292250
Ca_Kabuli_NC_021161.1_33103378	Ca_kabuli_chr02	33103378	[T/C]	1067292251
Ca_Kabuli_NC_021161.1_33103384	Ca_kabuli_chr02	33103384	[A/G]	1067292252
Ca_Kabuli_NC_021161.1_33103403	Ca_kabuli_chr02	33103403	[A/G]	1067292253
Ca_Kabuli_NC_021161.1_33103404	Ca_kabuli_chr02	33103404	[C/A]	1067292254
Ca_Kabuli_NC_021161.1_33103418	Ca_kabuli_chr02	33103418	[G/T]	1067292255
Ca_Kabuli_NC_021161.1_33103654	Ca_kabuli_chr02	33103654	[T/G]	1067292256
Ca_Kabuli_NC_021161.1_33103780	Ca_kabuli_chr02	33103780	[T/G]	1067292257
Ca_Kabuli_NC_021161.1_33103839	Ca_kabuli_chr02	33103839	[T/G]	1067292258
Ca_Kabuli_NC_021161.1_33103873	Ca_kabuli_chr02	33103873	[T/G]	1067292259
Ca_Kabuli_NC_021161.1_33104575	Ca_kabuli_chr02	33104575	[A/C]	1067292260
Ca_Kabuli_NC_021161.1_33143206	Ca_kabuli_chr02	33143206	[C/A]	1067292261
Ca_Kabuli_NC_021161.1_33172840	Ca_kabuli_chr02	33172840	[T/C]	1067292262
Ca_Kabuli_NC_021161.1_33172934	Ca_kabuli_chr02	33172934	[A/C]	1067292263
Ca_Kabuli_NC_021161.1_33196153	Ca_kabuli_chr02	33196153	[T/C]	1067292264
Ca_Kabuli_NC_021161.1_33198774	Ca_kabuli_chr02	33198774	[T/G]	1067292265
Ca_Kabuli_NC_021161.1_33198797	Ca_kabuli_chr02	33198797	[T/G]	1067292266
Ca_Kabuli_NC_021161.1_33302708	Ca_kabuli_chr02	33302708	[T/G]	1067292267
Ca_Kabuli_NC_021161.1_33309055	Ca_kabuli_chr02	33309055	[T/C]	1067292268

SNP IDs	Chromosomes	Physical positions (bp)	SNPs	NCBI SNPdb accession IDs (NCBI_ss#)
Ca_Kabuli_NC_021161.1_33326509	Ca_kabuli_chr02	33326509	[A/T]	1067292269
Ca_Kabuli_NC_021161.1_33340390	Ca_kabuli_chr02	33340390	[A/C]	1067292270
Ca_Kabuli_NC_021161.1_33340412	Ca_kabuli_chr02	33340412	[T/G]	1067292271
Ca_Kabuli_NC_021161.1_33345575	Ca_kabuli_chr02	33345575	[A/G]	1067292272
Ca_Kabuli_NC_021161.1_33398549	Ca_kabuli_chr02	33398549	[T/C]	1067292273
Ca_Kabuli_NC_021161.1_33398550	Ca_kabuli_chr02	33398550	[G/A]	1067292274
Ca_Kabuli_NC_021161.1_33399065	Ca_kabuli_chr02	33399065	[T/C]	1067292275
Ca_Kabuli_NC_021161.1_33399066	Ca_kabuli_chr02	33399066	[G/T]	1067292276
Ca_Kabuli_NC_021161.1_33399381	Ca_kabuli_chr02	33399381	[C/T]	1067292277
Ca_Kabuli_NC_021161.1_33399393	Ca_kabuli_chr02	33399393	[C/A]	1067292278
Ca_Kabuli_NC_021161.1_33399397	Ca_kabuli_chr02	33399397	[C/T]	1067292279
Ca_Kabuli_NC_021161.1_33486730	Ca_kabuli_chr02	33486730	[T/G]	1067292280
Ca_Kabuli_NC_021161.1_33490058	Ca_kabuli_chr02	33490058	[C/A]	1067292281
Ca_Kabuli_NC_021161.1_33490962	Ca_kabuli_chr02	33490962	[A/G]	1067292282
Ca_Kabuli_NC_021161.1_33490965	Ca_kabuli_chr02	33490965	[T/G]	1067292283
Ca_Kabuli_NC_021161.1_33647899	Ca_kabuli_chr02	33647899	[A/C]	1067292284
Ca_Kabuli_NC_021161.1_33718719	Ca_kabuli_chr02	33718719	[T/C]	1067292285
Ca_Kabuli_NC_021161.1_33756537	Ca_kabuli_chr02	33756537	[G/C]	1067292286
Ca_Kabuli_NC_021161.1_33785359	Ca_kabuli_chr02	33785359	[A/C]	1067292287
Ca_Kabuli_NC_021161.1_33786074	Ca_kabuli_chr02	33786074	[A/G]	1067292288
Ca_Kabuli_NC_021161.1_33786282	Ca_kabuli_chr02	33786282	[A/C]	1067292289
Ca_Kabuli_NC_021161.1_33788911	Ca_kabuli_chr02	33788911	[A/C]	1067292290
Ca_Kabuli_NC_021161.1_33798764	Ca_kabuli_chr02	33798764	[A/G]	1067292291
Ca_Kabuli_NC_021161.1_34203159	Ca_kabuli_chr02	34203159	[T/G]	1067292292
Ca_Kabuli_NC_021161.1_34223036	Ca_kabuli_chr02	34223036	[T/G]	1067292293
Ca_Kabuli_NC_021161.1_34223049	Ca_kabuli_chr02	34223049	[T/G]	1067292294
Ca_Kabuli_NC_021161.1_34290093	Ca_kabuli_chr02	34290093	[C/A]	1067292295
Ca_Kabuli_NC_021161.1_34290094	Ca_kabuli_chr02	34290094	[T/G]	1067292296
Ca_Kabuli_NC_021161.1_34290095	Ca_kabuli_chr02	34290095	[T/A]	1067292297
Ca_Kabuli_NC_021161.1_34290096	Ca_kabuli_chr02	34290096	[C/T]	1067292298
Ca_Kabuli_NC_021161.1_34299057	Ca_kabuli_chr02	34299057	[C/T]	1067292299
Ca_Kabuli_NC_021161.1_34299079	Ca_kabuli_chr02	34299079	[A/C]	1067292300
Ca_Kabuli_NC_021161.1_34299113	Ca_kabuli_chr02	34299113	[T/C]	1067292301
Ca_Kabuli_NC_021161.1_34300027	Ca_kabuli_chr02	34300027	[A/T]	1067292302

SNP IDs	Chromosomes	Physical positions (bp)	SNPs	NCBI SNPdb accession IDs (NCBI_ss#)
Ca_Kabuli_NC_021161.1_34428931	Ca_kabuli_chr02	34428931	[T/C]	1067292303
Ca_Kabuli_NC_021161.1_34428959	Ca_kabuli_chr02	34428959	[C/A]	1067292304
Ca_Kabuli_NC_021161.1_34428963	Ca_kabuli_chr02	34428963	[A/G]	1067292305
Ca_Kabuli_NC_021161.1_34429196	Ca_kabuli_chr02	34429196	[A/C]	1067292306
Ca_Kabuli_NC_021161.1_34432572	Ca_kabuli_chr02	34432572	[A/T]	1067292307
Ca_Kabuli_NC_021161.1_34432614	Ca_kabuli_chr02	34432614	[A/G]	1067292308
Ca_Kabuli_NC_021161.1_34432615	Ca_kabuli_chr02	34432615	[A/C]	1067292309
Ca_Kabuli_NC_021161.1_34551849	Ca_kabuli_chr02	34551849	[A/C]	1067292310
Ca_Kabuli_NC_021161.1_34551879	Ca_kabuli_chr02	34551879	[A/C]	1067292311
Ca_Kabuli_NC_021161.1_34734325	Ca_kabuli_chr02	34734325	[T/G]	1067292312
Ca_Kabuli_NC_021161.1_34734460	Ca_kabuli_chr02	34734460	[T/G]	1067292313
Ca_Kabuli_NC_021161.1_34734539	Ca_kabuli_chr02	34734539	[T/G]	1067292314
Ca_Kabuli_NC_021161.1_34739789	Ca_kabuli_chr02	34739789	[A/G]	1067292315
Ca_Kabuli_NC_021161.1_34739790	Ca_kabuli_chr02	34739790	[C/A]	1067292316
Ca_Kabuli_NC_021161.1_34739791	Ca_kabuli_chr02	34739791	[C/T]	1067292317
Ca_Kabuli_NC_021161.1_34739792	Ca_kabuli_chr02	34739792	[A/C]	1067292318
Ca_Kabuli_NC_021161.1_34739793	Ca_kabuli_chr02	34739793	[G/T]	1067292319
Ca_Kabuli_NC_021161.1_34751340	Ca_kabuli_chr02	34751340	[T/G]	1067292320
Ca_Kabuli_NC_021161.1_34752118	Ca_kabuli_chr02	34752118	[T/G]	1067292321
Ca_Kabuli_NC_021161.1_34752121	Ca_kabuli_chr02	34752121	[T/C]	1067292322
Ca_Kabuli_NC_021161.1_34752174	Ca_kabuli_chr02	34752174	[A/G]	1067292323
Ca_Kabuli_NC_021161.1_34752177	Ca_kabuli_chr02	34752177	[T/C]	1067292324
Ca_Kabuli_NC_021161.1_34827344	Ca_kabuli_chr02	34827344	[A/G]	1067292325
Ca_Kabuli_NC_021161.1_34827373	Ca_kabuli_chr02	34827373	[T/A]	1067292326
Ca_Kabuli_NC_021161.1_34847978	Ca_kabuli_chr02	34847978	[A/C]	1067292327
Ca_Kabuli_NC_021161.1_34848963	Ca_kabuli_chr02	34848963	[G/C]	1067292328
Ca_Kabuli_NC_021161.1_34848964	Ca_kabuli_chr02	34848964	[G/T]	1067292329
Ca_Kabuli_NC_021161.1_34862134	Ca_kabuli_chr02	34862134	[T/G]	1067292330
Ca_Kabuli_NC_021161.1_34862139	Ca_kabuli_chr02	34862139	[A/G]	1067292331
Ca_Kabuli_NC_021161.1_34982412	Ca_kabuli_chr02	34982412	[C/T]	1067292332
Ca_Kabuli_NC_021161.1_35009235	Ca_kabuli_chr02	35009235	[T/G]	1067292333
Ca_Kabuli_NC_021161.1_35009270	Ca_kabuli_chr02	35009270	[A/C]	1067292334
Ca_Kabuli_NC_021161.1_35015348	Ca_kabuli_chr02	35015348	[T/G]	1067292335
Ca_Kabuli_NC_021161.1_35046871	Ca_kabuli_chr02	35046871	[T/G]	1067292336

SNP IDs	Chromosomes	Physical positions (bp)	SNPs	NCBI SNPdb accession IDs (NCBI_ss#)
Ca_Kabuli_NC_021161.1_35046926	Ca_kabuli_chr02	35046926	[A/G]	1067292337
Ca_Kabuli_NC_021161.1_35046934	Ca_kabuli_chr02	35046934	[A/G]	1067292338
Ca_Kabuli_NC_021161.1_35059431	Ca_kabuli_chr02	35059431	[T/C]	1067292339
Ca_Kabuli_NC_021161.1_35059446	Ca_kabuli_chr02	35059446	[A/G]	1067292340
Ca_Kabuli_NC_021161.1_35060279	Ca_kabuli_chr02	35060279	[A/C]	1067292341
Ca_Kabuli_NC_021161.1_35096553	Ca_kabuli_chr02	35096553	[T/G]	1067292342
Ca_Kabuli_NC_021161.1_35103037	Ca_kabuli_chr02	35103037	[A/C]	1067292343
Ca_Kabuli_NC_021161.1_35103042	Ca_kabuli_chr02	35103042	[T/C]	1067292344
Ca_Kabuli_NC_021161.1_35103055	Ca_kabuli_chr02	35103055	[A/G]	1067292345
Ca_Kabuli_NC_021161.1_35103065	Ca_kabuli_chr02	35103065	[A/C]	1067292346
Ca_Kabuli_NC_021161.1_35121975	Ca_kabuli_chr02	35121975	[A/T]	1067292347
Ca_Kabuli_NC_021161.1_35121976	Ca_kabuli_chr02	35121976	[T/C]	1067292348
Ca_Kabuli_NC_021161.1_35121978	Ca_kabuli_chr02	35121978	[T/C]	1067292349
Ca_Kabuli_NC_021161.1_35121998	Ca_kabuli_chr02	35121998	[A/G]	1067292350
Ca_Kabuli_NC_021161.1_35122022	Ca_kabuli_chr02	35122022	[A/G]	1067292351
Ca_Kabuli_NC_021161.1_35122026	Ca_kabuli_chr02	35122026	[T/G]	1067292352
Ca_Kabuli_NC_021161.1_35122057	Ca_kabuli_chr02	35122057	[A/C]	1067292353
Ca_Kabuli_NC_021161.1_35147373	Ca_kabuli_chr02	35147373	[A/C]	1067292354
Ca_Kabuli_NC_021161.1_35154578	Ca_kabuli_chr02	35154578	[T/G]	1067292355
Ca_Kabuli_NC_021161.1_35154655	Ca_kabuli_chr02	35154655	[A/C]	1067292356
Ca_Kabuli_NC_021161.1_35171834	Ca_kabuli_chr02	35171834	[A/C]	1067292357
Ca_Kabuli_NC_021161.1_35171853	Ca_kabuli_chr02	35171853	[T/G]	1067292358
Ca_Kabuli_NC_021161.1_35209974	Ca_kabuli_chr02	35209974	[T/A]	1067292359
Ca_Kabuli_NC_021161.1_35363488	Ca_kabuli_chr02	35363488	[T/C]	1067292360
Ca_Kabuli_NC_021161.1_35441180	Ca_kabuli_chr02	35441180	[G/A]	1067292361
Ca_Kabuli_NC_021161.1_35441200	Ca_kabuli_chr02	35441200	[T/C]	1067292362
Ca_Kabuli_NC_021161.1_35441204	Ca_kabuli_chr02	35441204	[T/C]	1067292363
Ca_Kabuli_NC_021161.1_35441210	Ca_kabuli_chr02	35441210	[T/C]	1067292364
Ca_Kabuli_NC_021161.1_35441214	Ca_kabuli_chr02	35441214	[T/C]	1067292365
Ca_Kabuli_NC_021161.1_35441216	Ca_kabuli_chr02	35441216	[G/C]	1067292366
Ca_Kabuli_NC_021161.1_35441220	Ca_kabuli_chr02	35441220	[T/C]	1067292367
Ca_Kabuli_NC_021161.1_35441225	Ca_kabuli_chr02	35441225	[T/C]	1067292368
Ca_Kabuli_NC_021161.1_35441227	Ca_kabuli_chr02	35441227	[T/C]	1067292369
Ca_Kabuli_NC_021161.1_35449653	Ca_kabuli_chr02	35449653	[A/G]	1067292370

SNP IDs	Chromosomes	Physical positions (bp)	SNPs	NCBI SNPdb accession IDs (NCBI_ss#)
Ca_Kabuli_NC_021161.1_35476121	Ca_kabuli_chr02	35476121	[A/G]	1067292371
Ca_Kabuli_NC_021161.1_35476125	Ca_kabuli_chr02	35476125	[T/C]	1067292372
Ca_Kabuli_NC_021161.1_35505884	Ca_kabuli_chr02	35505884	[T/G]	1067292373
Ca_Kabuli_NC_021161.1_35505927	Ca_kabuli_chr02	35505927	[T/C]	1067292374
Ca_Kabuli_NC_021161.1_35505948	Ca_kabuli_chr02	35505948	[A/G]	1067292375
Ca_Kabuli_NC_021161.1_35505960	Ca_kabuli_chr02	35505960	[T/C]	1067292376
Ca_Kabuli_NC_021161.1_35506245	Ca_kabuli_chr02	35506245	[A/G]	1067292377
Ca_Kabuli_NC_021161.1_35506254	Ca_kabuli_chr02	35506254	[G/A]	1067292378
Ca_Kabuli_NC_021161.1_35506256	Ca_kabuli_chr02	35506256	[G/A]	1067292379
Ca_Kabuli_NC_021161.1_35506258	Ca_kabuli_chr02	35506258	[G/A]	1067292380
Ca_Kabuli_NC_021161.1_35506259	Ca_kabuli_chr02	35506259	[G/A]	1067292381
Ca_Kabuli_NC_021161.1_35533250	Ca_kabuli_chr02	35533250	[T/G]	1067292382
Ca_Kabuli_NC_021161.1_35533271	Ca_kabuli_chr02	35533271	[T/G]	1067292383
Ca_Kabuli_NC_021161.1_35593355	Ca_kabuli_chr02	35593355	[C/G]	1067292384
Ca_Kabuli_NC_021161.1_35593363	Ca_kabuli_chr02	35593363	[C/T]	1067292385
Ca_Kabuli_NC_021161.1_35593412	Ca_kabuli_chr02	35593412	[T/G]	1067292386
Ca_Kabuli_NC_021161.1_35593713	Ca_kabuli_chr02	35593713	[T/C]	1067292387
Ca_Kabuli_NC_021161.1_35593731	Ca_kabuli_chr02	35593731	[A/G]	1067292388
Ca_Kabuli_NC_021161.1_35593749	Ca_kabuli_chr02	35593749	[T/C]	1067292389
Ca_Kabuli_NC_021161.1_35601566	Ca_kabuli_chr02	35601566	[T/C]	1067292390
Ca_Kabuli_NC_021161.1_35601571	Ca_kabuli_chr02	35601571	[A/C]	1067292391
Ca_Kabuli_NC_021161.1_35601578	Ca_kabuli_chr02	35601578	[C/T]	1067292392
Ca_Kabuli_NC_021161.1_35601657	Ca_kabuli_chr02	35601657	[T/C]	1067292393
Ca_Kabuli_NC_021161.1_35601691	Ca_kabuli_chr02	35601691	[A/G]	1067292394
Ca_Kabuli_NC_021161.1_35612587	Ca_kabuli_chr02	35612587	[C/A]	1067292395
Ca_Kabuli_NC_021161.1_35612644	Ca_kabuli_chr02	35612644	[G/A]	1067292396
Ca_Kabuli_NC_021161.1_35613135	Ca_kabuli_chr02	35613135	[T/C]	1067292397
Ca_Kabuli_NC_021161.1_35661053	Ca_kabuli_chr02	35661053	[C/T]	1067292398
Ca_Kabuli_NC_021161.1_35670478	Ca_kabuli_chr02	35670478	[A/G]	1067292399
Ca_Kabuli_NC_021161.1_35670520	Ca_kabuli_chr02	35670520	[A/T]	1067292400
Ca_Kabuli_NC_021161.1_35676127	Ca_kabuli_chr02	35676127	[G/A]	1067292401
Ca_Kabuli_NC_021161.1_35676704	Ca_kabuli_chr02	35676704	[T/A]	1067292402
Ca_Kabuli_NC_021161.1_35708845	Ca_kabuli_chr02	35708845	[A/C]	1067292403
Ca_Kabuli_NC_021161.1_35709004	Ca_kabuli_chr02	35709004	[C/T]	1067292404

SNP IDs	Chromosomes	Physical positions (bp)	SNPs	NCBI SNPdb accession IDs (NCBI_ss#)
Ca_Kabuli_NC_021161.1_35709032	Ca_kabuli_chr02	35709032	[T/C]	1067292405
Ca_Kabuli_NC_021161.1_35709129	Ca_kabuli_chr02	35709129	[C/G]	1067292406
Ca_Kabuli_NC_021161.1_35727800	Ca_kabuli_chr02	35727800	[G/A]	1067292407
Ca_Kabuli_NC_021161.1_35754653	Ca_kabuli_chr02	35754653	[C/T]	1067292408
Ca_Kabuli_NC_021161.1_35754677	Ca_kabuli_chr02	35754677	[T/A]	1067292409
Ca_Kabuli_NC_021161.1_35754679	Ca_kabuli_chr02	35754679	[A/T]	1067292410
Ca_Kabuli_NC_021161.1_35760155	Ca_kabuli_chr02	35760155	[G/A]	1067292411
Ca_Kabuli_NC_021161.1_35760165	Ca_kabuli_chr02	35760165	[A/G]	1067292412
Ca_Kabuli_NC_021161.1_35760170	Ca_kabuli_chr02	35760170	[A/G]	1067292413
Ca_Kabuli_NC_021161.1_35770311	Ca_kabuli_chr02	35770311	[A/G]	1067292414
Ca_Kabuli_NC_021161.1_35770313	Ca_kabuli_chr02	35770313	[A/G]	1067292415
Ca_Kabuli_NC_021161.1_35795410	Ca_kabuli_chr02	35795410	[T/C]	1067292416
Ca_Kabuli_NC_021161.1_35795440	Ca_kabuli_chr02	35795440	[T/C]	1067292417
Ca_Kabuli_NC_021161.1_35795510	Ca_kabuli_chr02	35795510	[C/A]	1067292418
Ca_Kabuli_NC_021161.1_35795511	Ca_kabuli_chr02	35795511	[T/G]	1067292419
Ca_Kabuli_NC_021161.1_35859086	Ca_kabuli_chr02	35859086	[A/G]	1067292420
Ca_Kabuli_NC_021161.1_35875663	Ca_kabuli_chr02	35875663	[T/G]	1067292421
Ca_Kabuli_NC_021161.1_35894728	Ca_kabuli_chr02	35894728	[T/C]	1067292422
Ca_Kabuli_NC_021161.1_35896680	Ca_kabuli_chr02	35896680	[T/G]	1067292423
Ca_Kabuli_NC_021161.1_35972953	Ca_kabuli_chr02	35972953	[C/A]	1067292424
Ca_Kabuli_NC_021161.1_35986432	Ca_kabuli_chr02	35986432	[G/C]	1067292425
Ca_Kabuli_NC_021161.1_36008104	Ca_kabuli_chr02	36008104	[T/C]	1067292426
Ca_Kabuli_NC_021161.1_36016362	Ca_kabuli_chr02	36016362	[A/G]	1067292427
Ca_Kabuli_NC_021161.1_36018141	Ca_kabuli_chr02	36018141	[T/G]	1067292428
Ca_Kabuli_NC_021161.1_36039313	Ca_kabuli_chr02	36039313	[C/T]	1067292429
Ca_Kabuli_NC_021161.1_36039387	Ca_kabuli_chr02	36039387	[T/C]	1067292430
Ca_Kabuli_NC_021161.1_36039398	Ca_kabuli_chr02	36039398	[A/C]	1067292431
Ca_Kabuli_NC_021161.1_36046593	Ca_kabuli_chr02	36046593	[G/T]	1067292432
Ca_Kabuli_NC_021161.1_36046595	Ca_kabuli_chr02	36046595	[T/G]	1067292433
Ca_Kabuli_NC_021161.1_36046720	Ca_kabuli_chr02	36046720	[T/A]	1067292434
Ca_Kabuli_NC_021161.1_36046741	Ca_kabuli_chr02	36046741	[G/A]	1067292435
Ca_Kabuli_NC_021161.1_36046767	Ca_kabuli_chr02	36046767	[T/A]	1067292436
Ca_Kabuli_NC_021161.1_36046820	Ca_kabuli_chr02	36046820	[T/A]	1067292437
Ca_Kabuli_NC_021161.1_36051329	Ca_kabuli_chr02	36051329	[T/C]	1067292438

SNP IDs	Chromosomes	Physical positions (bp)	SNPs	NCBI SNPdb accession IDs (NCBI_ss#)
Ca_Kabuli_NC_021161.1_36055099	Ca_kabuli_chr02	36055099	[T/C]	1067292439
Ca_Kabuli_NC_021161.1_36055128	Ca_kabuli_chr02	36055128	[T/A]	1067292440
Ca_Kabuli_NC_021161.1_36055150	Ca_kabuli_chr02	36055150	[A/G]	1067292441
Ca_Kabuli_NC_021161.1_36069340	Ca_kabuli_chr02	36069340	[C/T]	1067292442
Ca_Kabuli_NC_021161.1_36069379	Ca_kabuli_chr02	36069379	[A/G]	1067292443
Ca_Kabuli_NC_021161.1_36069394	Ca_kabuli_chr02	36069394	[T/C]	1067292444
Ca_Kabuli_NC_021161.1_36072247	Ca_kabuli_chr02	36072247	[A/C]	1067292445
Ca_Kabuli_NC_021161.1_36073955	Ca_kabuli_chr02	36073955	[T/C]	1067292446
Ca_Kabuli_NC_021161.1_36073971	Ca_kabuli_chr02	36073971	[A/G]	1067292447
Ca_Kabuli_NC_021161.1_36073980	Ca_kabuli_chr02	36073980	[A/C]	1067292448
Ca_Kabuli_NC_021161.1_36074024	Ca_kabuli_chr02	36074024	[T/G]	1067292449
Ca_Kabuli_NC_021161.1_36074147	Ca_kabuli_chr02	36074147	[C/T]	1067292450
Ca_Kabuli_NC_021161.1_36088577	Ca_kabuli_chr02	36088577	[T/A]	1067292451
Ca_Kabuli_NC_021161.1_36088676	Ca_kabuli_chr02	36088676	[C/T]	1067292452
Ca_Kabuli_NC_021161.1_36101377	Ca_kabuli_chr02	36101377	[T/C]	1067292453
Ca_Kabuli_NC_021161.1_36101414	Ca_kabuli_chr02	36101414	[T/C]	1067292454
Ca_Kabuli_NC_021161.1_36101450	Ca_kabuli_chr02	36101450	[T/C]	1067292455
Ca_Kabuli_NC_021161.1_36117647	Ca_kabuli_chr02	36117647	[A/G]	1067292456
Ca_Kabuli_NC_021161.1_36117695	Ca_kabuli_chr02	36117695	[A/T]	1067292457
Ca_Kabuli_NC_021161.1_36165245	Ca_kabuli_chr02	36165245	[G/T]	1067292458
Ca_Kabuli_NC_021161.1_36171226	Ca_kabuli_chr02	36171226	[T/G]	1067292459
Ca_Kabuli_NC_021161.1_36171325	Ca_kabuli_chr02	36171325	[C/A]	1067292460
Ca_Kabuli_NC_021161.1_36171326	Ca_kabuli_chr02	36171326	[T/G]	1067292461
Ca_Kabuli_NC_021161.1_36171327	Ca_kabuli_chr02	36171327	[T/A]	1067292462
Ca_Kabuli_NC_021161.1_36238770	Ca_kabuli_chr02	36238770	[C/T]	1067292463
Ca_Kabuli_NC_021161.1_36238788	Ca_kabuli_chr02	36238788	[A/C]	1067292464
Ca_Kabuli_NC_021161.1_36238792	Ca_kabuli_chr02	36238792	[A/G]	1067292465
Ca_Kabuli_NC_021161.1_36238798	Ca_kabuli_chr02	36238798	[A/G]	1067292466
Ca_Kabuli_NC_021161.1_36240619	Ca_kabuli_chr02	36240619	[T/G]	1067292467
Ca_Kabuli_NC_021161.1_36275166	Ca_kabuli_chr02	36275166	[T/A]	1067292468
Ca_Kabuli_NC_021161.1_36275167	Ca_kabuli_chr02	36275167	[G/T]	1067292469
Ca_Kabuli_NC_021161.1_36275168	Ca_kabuli_chr02	36275168	[A/C]	1067292470
Ca_Kabuli_NC_021161.1_36275169	Ca_kabuli_chr02	36275169	[G/T]	1067292471
Ca_Kabuli_NC_021161.1_36276820	Ca_kabuli_chr02	36276820	[T/C]	1067292472

SNP IDs	Chromosomes	Physical positions (bp)	SNPs	NCBI SNPdb accession IDs (NCBI_ss#)
Ca_Kabuli_NC_021161.1_36276876	Ca_kabuli_chr02	36276876	[T/C]	1067292473
Ca_Kabuli_NC_021161.1_36276883	Ca_kabuli_chr02	36276883	[T/G]	1067292474
Ca_Kabuli_NC_021161.1_36276889	Ca_kabuli_chr02	36276889	[G/A]	1067292475
Ca_Kabuli_NC_021161.1_36276891	Ca_kabuli_chr02	36276891	[T/G]	1067292476
Ca_Kabuli_NC_021161.1_36276907	Ca_kabuli_chr02	36276907	[T/G]	1067292477
Ca_Kabuli_NC_021161.1_36323595	Ca_kabuli_chr02	36323595	[T/G]	1067292478
Ca_Kabuli_NC_021161.1_36323606	Ca_kabuli_chr02	36323606	[T/G]	1067292479
Ca_Kabuli_NC_021161.1_36323618	Ca_kabuli_chr02	36323618	[G/A]	1067292480
Ca_Kabuli_NC_021161.1_36323646	Ca_kabuli_chr02	36323646	[A/G]	1067292481
Ca_Kabuli_NC_021161.1_36326190	Ca_kabuli_chr02	36326190	[T/C]	1067292482
Ca_Kabuli_NC_021161.1_36326312	Ca_kabuli_chr02	36326312	[T/C]	1067292483
Ca_Kabuli_NC_021161.1_36326346	Ca_kabuli_chr02	36326346	[T/A]	1067292484
Ca_Kabuli_NC_021161.1_36326352	Ca_kabuli_chr02	36326352	[A/G]	1067292485
Ca_Kabuli_NC_021161.1_36326413	Ca_kabuli_chr02	36326413	[A/G]	1067292486
Ca_Kabuli_NC_021161.1_36335214	Ca_kabuli_chr02	36335214	[T/G]	1067292487
Ca_Kabuli_NC_021161.1_36343886	Ca_kabuli_chr02	36343886	[T/G]	1067292488
Ca_Kabuli_NC_021161.1_36346216	Ca_kabuli_chr02	36346216	[A/C]	1067292489
Ca_Kabuli_NC_021161.1_36357168	Ca_kabuli_chr02	36357168	[T/G]	1067292490
Ca_Kabuli_NC_021161.1_36375623	Ca_kabuli_chr02	36375623	[A/C]	1067292491
Ca_Kabuli_NC_021161.1_36375627	Ca_kabuli_chr02	36375627	[A/C]	1067292492
Ca_Kabuli_NC_021161.1_36399776	Ca_kabuli_chr02	36399776	[T/C]	1067292493
Ca_Kabuli_NC_021161.1_36399778	Ca_kabuli_chr02	36399778	[T/C]	1067292494
Ca_Kabuli_NC_021161.1_36427777	Ca_kabuli_chr02	36427777	[T/C]	1067292495
Ca_Kabuli_NC_021161.1_36427779	Ca_kabuli_chr02	36427779	[T/G]	1067292496
Ca_Kabuli_NC_021161.1_36453677	Ca_kabuli_chr02	36453677	[A/C]	1067292497
Ca_Kabuli_NC_021161.1_36453707	Ca_kabuli_chr02	36453707	[T/G]	1067292498
Ca_Kabuli_NC_021161.1_36461900	Ca_kabuli_chr02	36461900	[A/T]	1067292499
Ca_Kabuli_NC_021161.1_36461939	Ca_kabuli_chr02	36461939	[T/C]	1067292500
Ca_Kabuli_NC_021161.1_36466832	Ca_kabuli_chr02	36466832	[C/A]	1067292501
Ca_Kabuli_NC_021161.1_36466982	Ca_kabuli_chr02	36466982	[T/A]	1067292502
Ca_Kabuli_NC_021161.1_36466992	Ca_kabuli_chr02	36466992	[A/T]	1067292503
Ca_Kabuli_NC_021161.1_36466995	Ca_kabuli_chr02	36466995	[A/G]	1067292504
Ca_Kabuli_NC_021161.1_36467002	Ca_kabuli_chr02	36467002	[T/C]	1067292505
Ca_Kabuli_NC_021161.1_36467100	Ca_kabuli_chr02	36467100	[A/C]	1067292506

SNP IDs	Chromosomes	Physical positions (bp)	SNPs	NCBI SNPdb accession IDs (NCBI_ss#)
Ca_Kabuli_NC_021161.1_36467109	Ca_kabuli_chr02	36467109	[A/C]	1067292507
Ca_Kabuli_NC_021161.1_36505271	Ca_kabuli_chr02	36505271	[A/C]	1067292508
Ca_Kabuli_NC_021161.1_36505764	Ca_kabuli_chr02	36505764	[T/C]	1067292509
Ca_Kabuli_NC_021161.1_36533802	Ca_kabuli_chr02	36533802	[T/G]	1067292510
Ca_Kabuli_NC_021161.1_36533883	Ca_kabuli_chr02	36533883	[A/C]	1067292511
Ca_Kabuli_NC_021161.1_36533885	Ca_kabuli_chr02	36533885	[A/C]	1067292512
Ca_Kabuli_NC_021161.1_36565919	Ca_kabuli_chr02	36565919	[A/C]	1067292513
Ca_Kabuli_NC_021161.1_36565937	Ca_kabuli_chr02	36565937	[A/G]	1067292514
Ca_Kabuli_NC_021161.1_36634330	Ca_kabuli_chr02	36634330	[A/G]	1067292515
Ca_Kabuli_NC_021162.1_17783	Ca_kabuli_chr03	17783	[T/G]	1067292516
Ca_Kabuli_NC_021162.1_17786	Ca_kabuli_chr03	17786	[T/C]	1067292517
Ca_Kabuli_NC_021162.1_17826	Ca_kabuli_chr03	17826	[A/C]	1067292518
Ca_Kabuli_NC_021162.1_55508	Ca_kabuli_chr03	55508	[A/G]	1067292519
Ca_Kabuli_NC_021162.1_55510	Ca_kabuli_chr03	55510	[A/C]	1067292520
Ca_Kabuli_NC_021162.1_57241	Ca_kabuli_chr03	57241	[T/A]	1067292521
Ca_Kabuli_NC_021162.1_57271	Ca_kabuli_chr03	57271	[T/C]	1067292522
Ca_Kabuli_NC_021162.1_72801	Ca_kabuli_chr03	72801	[T/G]	1067292523
Ca_Kabuli_NC_021162.1_171675	Ca_kabuli_chr03	171675	[A/T]	1067292524
Ca_Kabuli_NC_021162.1_202442	Ca_kabuli_chr03	202442	[T/G]	1067292525
Ca_Kabuli_NC_021162.1_228564	Ca_kabuli_chr03	228564	[A/C]	1067292526
Ca_Kabuli_NC_021162.1_419704	Ca_kabuli_chr03	419704	[A/T]	1067292527
Ca_Kabuli_NC_021162.1_559006	Ca_kabuli_chr03	559006	[T/C]	1067292528
Ca_Kabuli_NC_021162.1_559031	Ca_kabuli_chr03	559031	[A/T]	1067292529
Ca_Kabuli_NC_021162.1_559070	Ca_kabuli_chr03	559070	[A/T]	1067292530
Ca_Kabuli_NC_021162.1_638768	Ca_kabuli_chr03	638768	[A/C]	1067292531
Ca_Kabuli_NC_021162.1_654821	Ca_kabuli_chr03	654821	[A/G]	1067292532
Ca_Kabuli_NC_021162.1_654827	Ca_kabuli_chr03	654827	[T/G]	1067292533
Ca_Kabuli_NC_021162.1_654847	Ca_kabuli_chr03	654847	[A/C]	1067292534
Ca_Kabuli_NC_021162.1_654931	Ca_kabuli_chr03	654931	[A/C]	1067292535
Ca_Kabuli_NC_021162.1_726856	Ca_kabuli_chr03	726856	[G/A]	1067292536
Ca_Kabuli_NC_021162.1_726877	Ca_kabuli_chr03	726877	[A/C]	1067292537
Ca_Kabuli_NC_021162.1_726898	Ca_kabuli_chr03	726898	[T/G]	1067292538
Ca_Kabuli_NC_021162.1_774205	Ca_kabuli_chr03	774205	[C/G]	1067292539
Ca_Kabuli_NC_021162.1_779153	Ca_kabuli_chr03	779153	[T/C]	1067292540

SNP IDs	Chromosomes	Physical positions (bp)	SNPs	NCBI SNPdb accession IDs (NCBI_ss#)
Ca_Kabuli_NC_021162.1_779217	Ca_kabuli_chr03	779217	[A/G]	1067292541
Ca_Kabuli_NC_021162.1_938903	Ca_kabuli_chr03	938903	[T/A]	1067292542
Ca_Kabuli_NC_021162.1_1307829	Ca_kabuli_chr03	1307829	[T/C]	1067292543
Ca_Kabuli_NC_021162.1_1307833	Ca_kabuli_chr03	1307833	[T/G]	1067292544
Ca_Kabuli_NC_021162.1_1307848	Ca_kabuli_chr03	1307848	[A/G]	1067292545
Ca_Kabuli_NC_021162.1_1307876	Ca_kabuli_chr03	1307876	[A/C]	1067292546
Ca_Kabuli_NC_021162.1_1507162	Ca_kabuli_chr03	1507162	[G/A]	1067292547
Ca_Kabuli_NC_021162.1_1507166	Ca_kabuli_chr03	1507166	[A/T]	1067292548
Ca_Kabuli_NC_021162.1_1507167	Ca_kabuli_chr03	1507167	[C/T]	1067292549
Ca_Kabuli_NC_021162.1_1746337	Ca_kabuli_chr03	1746337	[T/G]	1067292550
Ca_Kabuli_NC_021162.1_1746346	Ca_kabuli_chr03	1746346	[A/T]	1067292551
Ca_Kabuli_NC_021162.1_1918964	Ca_kabuli_chr03	1918964	[A/C]	1067292552
Ca_Kabuli_NC_021162.1_2097376	Ca_kabuli_chr03	2097376	[T/G]	1067292553
Ca_Kabuli_NC_021162.1_2461959	Ca_kabuli_chr03	2461959	[A/G]	1067292554
Ca_Kabuli_NC_021162.1_2461960	Ca_kabuli_chr03	2461960	[A/G]	1067292555
Ca_Kabuli_NC_021162.1_2657331	Ca_kabuli_chr03	2657331	[G/A]	1067292556
Ca_Kabuli_NC_021162.1_2773414	Ca_kabuli_chr03	2773414	[C/A]	1067292557
Ca_Kabuli_NC_021162.1_2825562	Ca_kabuli_chr03	2825562	[G/A]	1067292558
Ca_Kabuli_NC_021162.1_2825574	Ca_kabuli_chr03	2825574	[T/C]	1067292559
Ca_Kabuli_NC_021162.1_2899877	Ca_kabuli_chr03	2899877	[A/C]	1067292560
Ca_Kabuli_NC_021162.1_2919136	Ca_kabuli_chr03	2919136	[T/C]	1067292561
Ca_Kabuli_NC_021162.1_2919165	Ca_kabuli_chr03	2919165	[G/A]	1067292562
Ca_Kabuli_NC_021162.1_2919167	Ca_kabuli_chr03	2919167	[T/G]	1067292563
Ca_Kabuli_NC_021162.1_2919168	Ca_kabuli_chr03	2919168	[G/T]	1067292564
Ca_Kabuli_NC_021162.1_2919169	Ca_kabuli_chr03	2919169	[T/G]	1067292565
Ca_Kabuli_NC_021162.1_2919171	Ca_kabuli_chr03	2919171	[T/G]	1067292566
Ca_Kabuli_NC_021162.1_2931012	Ca_kabuli_chr03	2931012	[A/C]	1067292567
Ca_Kabuli_NC_021162.1_2931017	Ca_kabuli_chr03	2931017	[A/C]	1067292568
Ca_Kabuli_NC_021162.1_2975130	Ca_kabuli_chr03	2975130	[A/C]	1067292569
Ca_Kabuli_NC_021162.1_2975136	Ca_kabuli_chr03	2975136	[A/G]	1067292570
Ca_Kabuli_NC_021162.1_2975145	Ca_kabuli_chr03	2975145	[C/T]	1067292571
Ca_Kabuli_NC_021162.1_2975157	Ca_kabuli_chr03	2975157	[A/G]	1067292572
Ca_Kabuli_NC_021162.1_2975166	Ca_kabuli_chr03	2975166	[A/T]	1067292573
Ca_Kabuli_NC_021162.1_2975179	Ca_kabuli_chr03	2975179	[T/G]	1067292574

SNP IDs	Chromosomes	Physical positions (bp)	SNPs	NCBI SNPdb accession IDs (NCBI_ss#)
Ca_Kabuli_NC_021162.1_2983757	Ca_kabuli_chr03	2983757	[T/G]	1067292575
Ca_Kabuli_NC_021162.1_2985176	Ca_kabuli_chr03	2985176	[T/G]	1067292576
Ca_Kabuli_NC_021162.1_3139678	Ca_kabuli_chr03	3139678	[T/G]	1067292577
Ca_Kabuli_NC_021162.1_3182276	Ca_kabuli_chr03	3182276	[T/G]	1067292578
Ca_Kabuli_NC_021162.1_3182360	Ca_kabuli_chr03	3182360	[A/C]	1067292579
Ca_Kabuli_NC_021162.1_3182403	Ca_kabuli_chr03	3182403	[G/A]	1067292580
Ca_Kabuli_NC_021162.1_3516526	Ca_kabuli_chr03	3516526	[A/C]	1067292581
Ca_Kabuli_NC_021162.1_3524264	Ca_kabuli_chr03	3524264	[T/C]	1067292582
Ca_Kabuli_NC_021162.1_3524268	Ca_kabuli_chr03	3524268	[T/G]	1067292583
Ca_Kabuli_NC_021162.1_3557377	Ca_kabuli_chr03	3557377	[T/A]	1067292584
Ca_Kabuli_NC_021162.1_3603410	Ca_kabuli_chr03	3603410	[T/C]	1067292585
Ca_Kabuli_NC_021162.1_3603423	Ca_kabuli_chr03	3603423	[T/G]	1067292586
Ca_Kabuli_NC_021162.1_3617555	Ca_kabuli_chr03	3617555	[G/A]	1067292587
Ca_Kabuli_NC_021162.1_3617576	Ca_kabuli_chr03	3617576	[T/G]	1067292588
Ca_Kabuli_NC_021162.1_3617617	Ca_kabuli_chr03	3617617	[A/C]	1067292589
Ca_Kabuli_NC_021162.1_3617635	Ca_kabuli_chr03	3617635	[A/G]	1067292590
Ca_Kabuli_NC_021162.1_3701398	Ca_kabuli_chr03	3701398	[T/A]	1067292591
Ca_Kabuli_NC_021162.1_3701401	Ca_kabuli_chr03	3701401	[A/T]	1067292592
Ca_Kabuli_NC_021162.1_3723547	Ca_kabuli_chr03	3723547	[T/C]	1067292593
Ca_Kabuli_NC_021162.1_3723555	Ca_kabuli_chr03	3723555	[A/G]	1067292594
Ca_Kabuli_NC_021162.1_3723651	Ca_kabuli_chr03	3723651	[C/G]	1067292595
Ca_Kabuli_NC_021162.1_3744880	Ca_kabuli_chr03	3744880	[T/G]	1067292596
Ca_Kabuli_NC_021162.1_3764428	Ca_kabuli_chr03	3764428	[A/G]	1067292597
Ca_Kabuli_NC_021162.1_3764549	Ca_kabuli_chr03	3764549	[A/G]	1067292598
Ca_Kabuli_NC_021162.1_4149626	Ca_kabuli_chr03	4149626	[A/G]	1067292599
Ca_Kabuli_NC_021162.1_4149639	Ca_kabuli_chr03	4149639	[T/C]	1067292600
Ca_Kabuli_NC_021162.1_4173267	Ca_kabuli_chr03	4173267	[T/G]	1067292601
Ca_Kabuli_NC_021162.1_4243649	Ca_kabuli_chr03	4243649	[G/T]	1067292602
Ca_Kabuli_NC_021162.1_4243651	Ca_kabuli_chr03	4243651	[C/T]	1067292603
Ca_Kabuli_NC_021162.1_4359042	Ca_kabuli_chr03	4359042	[T/C]	1067292604
Ca_Kabuli_NC_021162.1_4472934	Ca_kabuli_chr03	4472934	[A/C]	1067292605
Ca_Kabuli_NC_021162.1_4596782	Ca_kabuli_chr03	4596782	[A/C]	1067292606
Ca_Kabuli_NC_021162.1_4597188	Ca_kabuli_chr03	4597188	[A/G]	1067292607
Ca_Kabuli_NC_021162.1_4597224	Ca_kabuli_chr03	4597224	[C/T]	1067292608

SNP IDs	Chromosomes	Physical positions (bp)	SNPs	NCBI SNPdb accession IDs (NCBI_ss#)
Ca_Kabuli_NC_021162.1_4597225	Ca_kabuli_chr03	4597225	[T/G]	1067292609
Ca_Kabuli_NC_021162.1_4597252	Ca_kabuli_chr03	4597252	[A/G]	1067292610
Ca_Kabuli_NC_021162.1_4639221	Ca_kabuli_chr03	4639221	[T/G]	1067292611
Ca_Kabuli_NC_021162.1_4766681	Ca_kabuli_chr03	4766681	[A/C]	1067292612
Ca_Kabuli_NC_021162.1_4771806	Ca_kabuli_chr03	4771806	[A/G]	1067292613
Ca_Kabuli_NC_021162.1_4823781	Ca_kabuli_chr03	4823781	[T/G]	1067292614
Ca_Kabuli_NC_021162.1_4823784	Ca_kabuli_chr03	4823784	[T/G]	1067292615
Ca_Kabuli_NC_021162.1_4823787	Ca_kabuli_chr03	4823787	[T/G]	1067292616
Ca_Kabuli_NC_021162.1_4823818	Ca_kabuli_chr03	4823818	[C/A]	1067292617
Ca_Kabuli_NC_021162.1_4823820	Ca_kabuli_chr03	4823820	[T/A]	1067292618
Ca_Kabuli_NC_021162.1_4920818	Ca_kabuli_chr03	4920818	[T/C]	1067292619
Ca_Kabuli_NC_021162.1_4920825	Ca_kabuli_chr03	4920825	[A/G]	1067292620
Ca_Kabuli_NC_021162.1_5106054	Ca_kabuli_chr03	5106054	[A/C]	1067292621
Ca_Kabuli_NC_021162.1_5106055	Ca_kabuli_chr03	5106055	[G/A]	1067292622
Ca_Kabuli_NC_021162.1_5106056	Ca_kabuli_chr03	5106056	[A/T]	1067292623
Ca_Kabuli_NC_021162.1_5106057	Ca_kabuli_chr03	5106057	[T/G]	1067292624
Ca_Kabuli_NC_021162.1_5179256	Ca_kabuli_chr03	5179256	[T/G]	1067292625
Ca_Kabuli_NC_021162.1_5224973	Ca_kabuli_chr03	5224973	[A/G]	1067292626
Ca_Kabuli_NC_021162.1_5224976	Ca_kabuli_chr03	5224976	[A/G]	1067292627
Ca_Kabuli_NC_021162.1_5224979	Ca_kabuli_chr03	5224979	[T/G]	1067292628
Ca_Kabuli_NC_021162.1_5514227	Ca_kabuli_chr03	5514227	[A/C]	1067292629
Ca_Kabuli_NC_021162.1_5514329	Ca_kabuli_chr03	5514329	[G/C]	1067292630
Ca_Kabuli_NC_021162.1_5738698	Ca_kabuli_chr03	5738698	[T/C]	1067292631
Ca_Kabuli_NC_021162.1_5740327	Ca_kabuli_chr03	5740327	[A/G]	1067292632
Ca_Kabuli_NC_021162.1_5742330	Ca_kabuli_chr03	5742330	[A/G]	1067292633
Ca_Kabuli_NC_021162.1_5742342	Ca_kabuli_chr03	5742342	[A/C]	1067292634
Ca_Kabuli_NC_021162.1_5742351	Ca_kabuli_chr03	5742351	[T/A]	1067292635
Ca_Kabuli_NC_021162.1_6169510	Ca_kabuli_chr03	6169510	[A/C]	1067292636
Ca_Kabuli_NC_021162.1_6683590	Ca_kabuli_chr03	6683590	[T/C]	1067292637
Ca_Kabuli_NC_021162.1_6683625	Ca_kabuli_chr03	6683625	[A/G]	1067292638
Ca_Kabuli_NC_021162.1_6683651	Ca_kabuli_chr03	6683651	[T/A]	1067292639
Ca_Kabuli_NC_021162.1_6889802	Ca_kabuli_chr03	6889802	[T/G]	1067292640
Ca_Kabuli_NC_021162.1_6899409	Ca_kabuli_chr03	6899409	[A/G]	1067292641
Ca_Kabuli_NC_021162.1_6899636	Ca_kabuli_chr03	6899636	[T/A]	1067292642

SNP IDs	Chromosomes	Physical positions (bp)	SNPs	NCBI SNPdb accession IDs (NCBI_ss#)
Ca_Kabuli_NC_021162.1_6899649	Ca_kabuli_chr03	6899649	[T/G]	1067292643
Ca_Kabuli_NC_021162.1_6899667	Ca_kabuli_chr03	6899667	[T/C]	1067292644
Ca_Kabuli_NC_021162.1_6899670	Ca_kabuli_chr03	6899670	[T/C]	1067292645
Ca_Kabuli_NC_021162.1_6899681	Ca_kabuli_chr03	6899681	[T/A]	1067292646
Ca_Kabuli_NC_021162.1_7013451	Ca_kabuli_chr03	7013451	[T/C]	1067292647
Ca_Kabuli_NC_021162.1_7013458	Ca_kabuli_chr03	7013458	[T/C]	1067292648
Ca_Kabuli_NC_021162.1_7013496	Ca_kabuli_chr03	7013496	[A/T]	1067292649
Ca_Kabuli_NC_021162.1_7013629	Ca_kabuli_chr03	7013629	[A/C]	1067292650
Ca_Kabuli_NC_021162.1_7022117	Ca_kabuli_chr03	7022117	[T/A]	1067292651
Ca_Kabuli_NC_021162.1_7026895	Ca_kabuli_chr03	7026895	[C/A]	1067292652
Ca_Kabuli_NC_021162.1_7204053	Ca_kabuli_chr03	7204053	[G/A]	1067292653
Ca_Kabuli_NC_021162.1_7204071	Ca_kabuli_chr03	7204071	[T/C]	1067292654
Ca_Kabuli_NC_021162.1_7204087	Ca_kabuli_chr03	7204087	[C/T]	1067292655
Ca_Kabuli_NC_021162.1_7204088	Ca_kabuli_chr03	7204088	[G/C]	1067292656
Ca_Kabuli_NC_021162.1_7204099	Ca_kabuli_chr03	7204099	[C/T]	1067292657
Ca_Kabuli_NC_021162.1_7204105	Ca_kabuli_chr03	7204105	[G/A]	1067292658
Ca_Kabuli_NC_021162.1_7245991	Ca_kabuli_chr03	7245991	[A/G]	1067292659
Ca_Kabuli_NC_021162.1_7246022	Ca_kabuli_chr03	7246022	[T/G]	1067292660
Ca_Kabuli_NC_021162.1_7246048	Ca_kabuli_chr03	7246048	[T/C]	1067292661
Ca_Kabuli_NC_021162.1_7246052	Ca_kabuli_chr03	7246052	[T/C]	1067292662
Ca_Kabuli_NC_021162.1_7246820	Ca_kabuli_chr03	7246820	[T/G]	1067292663
Ca_Kabuli_NC_021162.1_7433676	Ca_kabuli_chr03	7433676	[T/C]	1067292664
Ca_Kabuli_NC_021162.1_7433689	Ca_kabuli_chr03	7433689	[A/G]	1067292665
Ca_Kabuli_NC_021162.1_7433694	Ca_kabuli_chr03	7433694	[T/A]	1067292666
Ca_Kabuli_NC_021162.1_7460294	Ca_kabuli_chr03	7460294	[C/A]	1067292667
Ca_Kabuli_NC_021162.1_7461841	Ca_kabuli_chr03	7461841	[A/C]	1067292668
Ca_Kabuli_NC_021162.1_7654641	Ca_kabuli_chr03	7654641	[G/A]	1067292669
Ca_Kabuli_NC_021162.1_7654644	Ca_kabuli_chr03	7654644	[A/T]	1067292670
Ca_Kabuli_NC_021162.1_7654669	Ca_kabuli_chr03	7654669	[T/A]	1067292671
Ca_Kabuli_NC_021162.1_7660001	Ca_kabuli_chr03	7660001	[C/A]	1067292672
Ca_Kabuli_NC_021162.1_7660002	Ca_kabuli_chr03	7660002	[A/G]	1067292673
Ca_Kabuli_NC_021162.1_7693883	Ca_kabuli_chr03	7693883	[T/G]	1067292674
Ca_Kabuli_NC_021162.1_7694182	Ca_kabuli_chr03	7694182	[T/C]	1067292675
Ca_Kabuli_NC_021162.1_7694212	Ca_kabuli_chr03	7694212	[A/G]	1067292676

SNP IDs	Chromosomes	Physical positions (bp)	SNPs	NCBI SNPdb accession IDs (NCBI_ss#)
Ca_Kabuli_NC_021162.1_7694220	Ca_kabuli_chr03	7694220	[T/G]	1067292677
Ca_Kabuli_NC_021162.1_7694228	Ca_kabuli_chr03	7694228	[T/G]	1067292678
Ca_Kabuli_NC_021162.1_7826991	Ca_kabuli_chr03	7826991	[G/T]	1067292679
Ca_Kabuli_NC_021162.1_8536213	Ca_kabuli_chr03	8536213	[C/T]	1067292680
Ca_Kabuli_NC_021162.1_8536275	Ca_kabuli_chr03	8536275	[C/T]	1067292681
Ca_Kabuli_NC_021162.1_8536295	Ca_kabuli_chr03	8536295	[A/T]	1067292682
Ca_Kabuli_NC_021162.1_8536421	Ca_kabuli_chr03	8536421	[A/C]	1067292683
Ca_Kabuli_NC_021162.1_8668904	Ca_kabuli_chr03	8668904	[A/C]	1067292684
Ca_Kabuli_NC_021162.1_8712519	Ca_kabuli_chr03	8712519	[A/G]	1067292685
Ca_Kabuli_NC_021162.1_8933210	Ca_kabuli_chr03	8933210	[A/C]	1067292686
Ca_Kabuli_NC_021162.1_8933221	Ca_kabuli_chr03	8933221	[T/A]	1067292687
Ca_Kabuli_NC_021162.1_8933245	Ca_kabuli_chr03	8933245	[G/T]	1067292688
Ca_Kabuli_NC_021162.1_9221402	Ca_kabuli_chr03	9221402	[T/G]	1067292689
Ca_Kabuli_NC_021162.1_9221435	Ca_kabuli_chr03	9221435	[T/G]	1067292690
Ca_Kabuli_NC_021162.1_9221892	Ca_kabuli_chr03	9221892	[T/C]	1067292691
Ca_Kabuli_NC_021162.1_9221894	Ca_kabuli_chr03	9221894	[A/T]	1067292692
Ca_Kabuli_NC_021162.1_9221932	Ca_kabuli_chr03	9221932	[T/G]	1067292693
Ca_Kabuli_NC_021162.1_9221984	Ca_kabuli_chr03	9221984	[T/A]	1067292694
Ca_Kabuli_NC_021162.1_9222083	Ca_kabuli_chr03	9222083	[A/C]	1067292695
Ca_Kabuli_NC_021162.1_9411780	Ca_kabuli_chr03	9411780	[G/A]	1067292696
Ca_Kabuli_NC_021162.1_9492468	Ca_kabuli_chr03	9492468	[A/C]	1067292697
Ca_Kabuli_NC_021162.1_9492489	Ca_kabuli_chr03	9492489	[A/C]	1067292698
Ca_Kabuli_NC_021162.1_9794311	Ca_kabuli_chr03	9794311	[T/G]	1067292699
Ca_Kabuli_NC_021162.1_9794318	Ca_kabuli_chr03	9794318	[A/G]	1067292700
Ca_Kabuli_NC_021162.1_9794320	Ca_kabuli_chr03	9794320	[T/C]	1067292701
Ca_Kabuli_NC_021162.1_9794355	Ca_kabuli_chr03	9794355	[T/A]	1067292702
Ca_Kabuli_NC_021162.1_9842188	Ca_kabuli_chr03	9842188	[T/C]	1067292703
Ca_Kabuli_NC_021162.1_9898715	Ca_kabuli_chr03	9898715	[T/A]	1067292704
Ca_Kabuli_NC_021162.1_10102706	Ca_kabuli_chr03	10102706	[A/C]	1067292705
Ca_Kabuli_NC_021162.1_10102718	Ca_kabuli_chr03	10102718	[A/C]	1067292706
Ca_Kabuli_NC_021162.1_10142131	Ca_kabuli_chr03	10142131	[T/G]	1067292707
Ca_Kabuli_NC_021162.1_10159836	Ca_kabuli_chr03	10159836	[C/A]	1067292708
Ca_Kabuli_NC_021162.1_10159849	Ca_kabuli_chr03	10159849	[T/G]	1067292709
Ca_Kabuli_NC_021162.1_10201302	Ca_kabuli_chr03	10201302	[T/C]	1067292710

SNP IDs	Chromosomes	Physical positions (bp)	SNPs	NCBI SNPdb accession IDs (NCBI_ss#)
Ca_Kabuli_NC_021162.1_10201315	Ca_kabuli_chr03	10201315	[A/C]	1067292711
Ca_Kabuli_NC_021162.1_10201322	Ca_kabuli_chr03	10201322	[T/C]	1067292712
Ca_Kabuli_NC_021162.1_10201377	Ca_kabuli_chr03	10201377	[T/G]	1067292713
Ca_Kabuli_NC_021162.1_10201628	Ca_kabuli_chr03	10201628	[A/C]	1067292714
Ca_Kabuli_NC_021162.1_10810939	Ca_kabuli_chr03	10810939	[A/G]	1067292715
Ca_Kabuli_NC_021162.1_11378171	Ca_kabuli_chr03	11378171	[T/G]	1067292716
Ca_Kabuli_NC_021162.1_11378182	Ca_kabuli_chr03	11378182	[T/C]	1067292717
Ca_Kabuli_NC_021162.1_11378192	Ca_kabuli_chr03	11378192	[A/G]	1067292718
Ca_Kabuli_NC_021162.1_11378540	Ca_kabuli_chr03	11378540	[T/G]	1067292719
Ca_Kabuli_NC_021162.1_11392951	Ca_kabuli_chr03	11392951	[A/C]	1067292720
Ca_Kabuli_NC_021162.1_11677779	Ca_kabuli_chr03	11677779	[T/A]	1067292721
Ca_Kabuli_NC_021162.1_11677780	Ca_kabuli_chr03	11677780	[C/A]	1067292722
Ca_Kabuli_NC_021162.1_11677781	Ca_kabuli_chr03	11677781	[T/G]	1067292723
Ca_Kabuli_NC_021162.1_11707759	Ca_kabuli_chr03	11707759	[A/G]	1067292724
Ca_Kabuli_NC_021162.1_11812070	Ca_kabuli_chr03	11812070	[T/G]	1067292725
Ca_Kabuli_NC_021162.1_11812168	Ca_kabuli_chr03	11812168	[T/C]	1067292726
Ca_Kabuli_NC_021162.1_11906828	Ca_kabuli_chr03	11906828	[G/A]	1067292727
Ca_Kabuli_NC_021162.1_12045674	Ca_kabuli_chr03	12045674	[A/G]	1067292728
Ca_Kabuli_NC_021162.1_12058482	Ca_kabuli_chr03	12058482	[A/C]	1067292729
Ca_Kabuli_NC_021162.1_12058588	Ca_kabuli_chr03	12058588	[A/C]	1067292730
Ca_Kabuli_NC_021162.1_12095667	Ca_kabuli_chr03	12095667	[T/A]	1067292731
Ca_Kabuli_NC_021162.1_12224519	Ca_kabuli_chr03	12224519	[T/C]	1067292732
Ca_Kabuli_NC_021162.1_12294550	Ca_kabuli_chr03	12294550	[T/C]	1067292733
Ca_Kabuli_NC_021162.1_12294581	Ca_kabuli_chr03	12294581	[A/G]	1067292734
Ca_Kabuli_NC_021162.1_12412724	Ca_kabuli_chr03	12412724	[T/G]	1067292735
Ca_Kabuli_NC_021162.1_12412765	Ca_kabuli_chr03	12412765	[A/G]	1067292736
Ca_Kabuli_NC_021162.1_12599378	Ca_kabuli_chr03	12599378	[T/G]	1067292737
Ca_Kabuli_NC_021162.1_12599387	Ca_kabuli_chr03	12599387	[A/T]	1067292738
Ca_Kabuli_NC_021162.1_12599496	Ca_kabuli_chr03	12599496	[A/G]	1067292739
Ca_Kabuli_NC_021162.1_12785073	Ca_kabuli_chr03	12785073	[T/G]	1067292740
Ca_Kabuli_NC_021162.1_12897831	Ca_kabuli_chr03	12897831	[A/C]	1067292741
Ca_Kabuli_NC_021162.1_12897853	Ca_kabuli_chr03	12897853	[T/C]	1067292742
Ca_Kabuli_NC_021162.1_12931582	Ca_kabuli_chr03	12931582	[T/A]	1067292743
Ca_Kabuli_NC_021162.1_12931626	Ca_kabuli_chr03	12931626	[T/C]	1067292744

SNP IDs	Chromosomes	Physical positions (bp)	SNPs	NCBI SNPdb accession IDs (NCBI_ss#)
Ca_Kabuli_NC_021162.1_13344037	Ca_kabuli_chr03	13344037	[A/G]	1067292745
Ca_Kabuli_NC_021162.1_13351178	Ca_kabuli_chr03	13351178	[A/T]	1067292746
Ca_Kabuli_NC_021162.1_13351186	Ca_kabuli_chr03	13351186	[A/G]	1067292747
Ca_Kabuli_NC_021162.1_13396902	Ca_kabuli_chr03	13396902	[A/C]	1067292748
Ca_Kabuli_NC_021162.1_13396908	Ca_kabuli_chr03	13396908	[C/T]	1067292749
Ca_Kabuli_NC_021162.1_13397005	Ca_kabuli_chr03	13397005	[A/T]	1067292750
Ca_Kabuli_NC_021162.1_13397024	Ca_kabuli_chr03	13397024	[A/G]	1067292751
Ca_Kabuli_NC_021162.1_13401136	Ca_kabuli_chr03	13401136	[T/G]	1067292752
Ca_Kabuli_NC_021162.1_13401272	Ca_kabuli_chr03	13401272	[A/C]	1067292753
Ca_Kabuli_NC_021162.1_13401311	Ca_kabuli_chr03	13401311	[T/C]	1067292754
Ca_Kabuli_NC_021162.1_13401328	Ca_kabuli_chr03	13401328	[T/G]	1067292755
Ca_Kabuli_NC_021162.1_13401339	Ca_kabuli_chr03	13401339	[T/G]	1067292756
Ca_Kabuli_NC_021162.1_13420618	Ca_kabuli_chr03	13420618	[T/G]	1067292757
Ca_Kabuli_NC_021162.1_13420634	Ca_kabuli_chr03	13420634	[A/G]	1067292758
Ca_Kabuli_NC_021162.1_13420739	Ca_kabuli_chr03	13420739	[T/G]	1067292759
Ca_Kabuli_NC_021162.1_13746694	Ca_kabuli_chr03	13746694	[T/C]	1067292760
Ca_Kabuli_NC_021162.1_13746781	Ca_kabuli_chr03	13746781	[A/C]	1067292761
Ca_Kabuli_NC_021162.1_13746893	Ca_kabuli_chr03	13746893	[A/G]	1067292762
Ca_Kabuli_NC_021162.1_13830248	Ca_kabuli_chr03	13830248	[T/C]	1067292763
Ca_Kabuli_NC_021162.1_13830249	Ca_kabuli_chr03	13830249	[A/G]	1067292764
Ca_Kabuli_NC_021162.1_13830253	Ca_kabuli_chr03	13830253	[A/G]	1067292765
Ca_Kabuli_NC_021162.1_14458398	Ca_kabuli_chr03	14458398	[T/C]	1067292766
Ca_Kabuli_NC_021162.1_14458415	Ca_kabuli_chr03	14458415	[T/G]	1067292767
Ca_Kabuli_NC_021162.1_14497303	Ca_kabuli_chr03	14497303	[T/G]	1067292768
Ca_Kabuli_NC_021162.1_14556858	Ca_kabuli_chr03	14556858	[T/G]	1067292769
Ca_Kabuli_NC_021162.1_14653148	Ca_kabuli_chr03	14653148	[A/C]	1067292770
Ca_Kabuli_NC_021162.1_14653297	Ca_kabuli_chr03	14653297	[A/G]	1067292771
Ca_Kabuli_NC_021162.1_14653304	Ca_kabuli_chr03	14653304	[T/G]	1067292772
Ca_Kabuli_NC_021162.1_14653315	Ca_kabuli_chr03	14653315	[C/G]	1067292773
Ca_Kabuli_NC_021162.1_14653316	Ca_kabuli_chr03	14653316	[G/C]	1067292774
Ca_Kabuli_NC_021162.1_14653322	Ca_kabuli_chr03	14653322	[A/C]	1067292775
Ca_Kabuli_NC_021162.1_14658158	Ca_kabuli_chr03	14658158	[T/C]	1067292776
Ca_Kabuli_NC_021162.1_14658170	Ca_kabuli_chr03	14658170	[A/C]	1067292777
Ca_Kabuli_NC_021162.1_14665738	Ca_kabuli_chr03	14665738	[T/C]	1067292778

SNP IDs	Chromosomes	Physical positions (bp)	SNPs	NCBI SNPdb accession IDs (NCBI_ss#)
Ca_Kabuli_NC_021162.1_14665746	Ca_kabuli_chr03	14665746	[T/G]	1067292779
Ca_Kabuli_NC_021162.1_14746564	Ca_kabuli_chr03	14746564	[T/G]	1067292780
Ca_Kabuli_NC_021162.1_14746627	Ca_kabuli_chr03	14746627	[A/G]	1067292781
Ca_Kabuli_NC_021162.1_14812960	Ca_kabuli_chr03	14812960	[A/C]	1067292782
Ca_Kabuli_NC_021162.1_14814985	Ca_kabuli_chr03	14814985	[T/C]	1067292783
Ca_Kabuli_NC_021162.1_15744994	Ca_kabuli_chr03	15744994	[T/C]	1067292784
Ca_Kabuli_NC_021162.1_15745044	Ca_kabuli_chr03	15745044	[A/C]	1067292785
Ca_Kabuli_NC_021162.1_15745095	Ca_kabuli_chr03	15745095	[G/C]	1067292786
Ca_Kabuli_NC_021162.1_15745126	Ca_kabuli_chr03	15745126	[A/C]	1067292787
Ca_Kabuli_NC_021162.1_15745152	Ca_kabuli_chr03	15745152	[A/C]	1067292788
Ca_Kabuli_NC_021162.1_15745173	Ca_kabuli_chr03	15745173	[A/G]	1067292789
Ca_Kabuli_NC_021162.1_15745199	Ca_kabuli_chr03	15745199	[T/G]	1067292790
Ca_Kabuli_NC_021162.1_15745297	Ca_kabuli_chr03	15745297	[T/C]	1067292791
Ca_Kabuli_NC_021162.1_15745336	Ca_kabuli_chr03	15745336	[A/C]	1067292792
Ca_Kabuli_NC_021162.1_15745337	Ca_kabuli_chr03	15745337	[G/T]	1067292793
Ca_Kabuli_NC_021162.1_15745349	Ca_kabuli_chr03	15745349	[A/C]	1067292794
Ca_Kabuli_NC_021162.1_15900664	Ca_kabuli_chr03	15900664	[A/C]	1067292795
Ca_Kabuli_NC_021162.1_15962245	Ca_kabuli_chr03	15962245	[A/C]	1067292796
Ca_Kabuli_NC_021162.1_15962248	Ca_kabuli_chr03	15962248	[T/C]	1067292797
Ca_Kabuli_NC_021162.1_15962250	Ca_kabuli_chr03	15962250	[A/C]	1067292798
Ca_Kabuli_NC_021162.1_16032127	Ca_kabuli_chr03	16032127	[A/G]	1067292799
Ca_Kabuli_NC_021162.1_16032139	Ca_kabuli_chr03	16032139	[A/T]	1067292800
Ca_Kabuli_NC_021162.1_16032148	Ca_kabuli_chr03	16032148	[A/C]	1067292801
Ca_Kabuli_NC_021162.1_16071260	Ca_kabuli_chr03	16071260	[T/G]	1067292802
Ca_Kabuli_NC_021162.1_16071272	Ca_kabuli_chr03	16071272	[A/C]	1067292803
Ca_Kabuli_NC_021162.1_16071317	Ca_kabuli_chr03	16071317	[T/C]	1067292804
Ca_Kabuli_NC_021162.1_16071323	Ca_kabuli_chr03	16071323	[T/C]	1067292805
Ca_Kabuli_NC_021162.1_16071333	Ca_kabuli_chr03	16071333	[T/C]	1067292806
Ca_Kabuli_NC_021162.1_16071359	Ca_kabuli_chr03	16071359	[T/C]	1067292807
Ca_Kabuli_NC_021162.1_16071381	Ca_kabuli_chr03	16071381	[T/C]	1067292808
Ca_Kabuli_NC_021162.1_16072277	Ca_kabuli_chr03	16072277	[T/C]	1067292809
Ca_Kabuli_NC_021162.1_16072279	Ca_kabuli_chr03	16072279	[A/C]	1067292810
Ca_Kabuli_NC_021162.1_16072287	Ca_kabuli_chr03	16072287	[A/G]	1067292811
Ca_Kabuli_NC_021162.1_16072290	Ca_kabuli_chr03	16072290	[T/A]	1067292812

SNP IDs	Chromosomes	Physical positions (bp)	SNPs	NCBI SNPdb accession IDs (NCBI_ss#)
Ca_Kabuli_NC_021162.1_16072305	Ca_kabuli_chr03	16072305	[A/G]	1067292813
Ca_Kabuli_NC_021162.1_16072318	Ca_kabuli_chr03	16072318	[A/G]	1067292814
Ca_Kabuli_NC_021162.1_16396606	Ca_kabuli_chr03	16396606	[T/C]	1067292815
Ca_Kabuli_NC_021162.1_16396641	Ca_kabuli_chr03	16396641	[T/C]	1067292816
Ca_Kabuli_NC_021162.1_16458098	Ca_kabuli_chr03	16458098	[T/C]	1067292817
Ca_Kabuli_NC_021162.1_16458103	Ca_kabuli_chr03	16458103	[T/G]	1067292818
Ca_Kabuli_NC_021162.1_16458107	Ca_kabuli_chr03	16458107	[T/G]	1067292819
Ca_Kabuli_NC_021162.1_16534322	Ca_kabuli_chr03	16534322	[T/A]	1067292820
Ca_Kabuli_NC_021162.1_16534353	Ca_kabuli_chr03	16534353	[A/G]	1067292821
Ca_Kabuli_NC_021162.1_16534362	Ca_kabuli_chr03	16534362	[A/G]	1067292822
Ca_Kabuli_NC_021162.1_16534366	Ca_kabuli_chr03	16534366	[T/C]	1067292823
Ca_Kabuli_NC_021162.1_16948797	Ca_kabuli_chr03	16948797	[T/C]	1067292824
Ca_Kabuli_NC_021162.1_17056557	Ca_kabuli_chr03	17056557	[G/C]	1067292825
Ca_Kabuli_NC_021162.1_17056558	Ca_kabuli_chr03	17056558	[G/T]	1067292826
Ca_Kabuli_NC_021162.1_17056566	Ca_kabuli_chr03	17056566	[A/G]	1067292827
Ca_Kabuli_NC_021162.1_17072178	Ca_kabuli_chr03	17072178	[A/G]	1067292828
Ca_Kabuli_NC_021162.1_17072181	Ca_kabuli_chr03	17072181	[A/C]	1067292829
Ca_Kabuli_NC_021162.1_17072193	Ca_kabuli_chr03	17072193	[T/C]	1067292830
Ca_Kabuli_NC_021162.1_17072194	Ca_kabuli_chr03	17072194	[C/T]	1067292831
Ca_Kabuli_NC_021162.1_17072200	Ca_kabuli_chr03	17072200	[C/A]	1067292832
Ca_Kabuli_NC_021162.1_17178219	Ca_kabuli_chr03	17178219	[T/G]	1067292833
Ca_Kabuli_NC_021162.1_17179915	Ca_kabuli_chr03	17179915	[T/G]	1067292834
Ca_Kabuli_NC_021162.1_17181173	Ca_kabuli_chr03	17181173	[T/C]	1067292835
Ca_Kabuli_NC_021162.1_17181193	Ca_kabuli_chr03	17181193	[A/G]	1067292836
Ca_Kabuli_NC_021162.1_17181201	Ca_kabuli_chr03	17181201	[T/G]	1067292837
Ca_Kabuli_NC_021162.1_17186987	Ca_kabuli_chr03	17186987	[C/A]	1067292838
Ca_Kabuli_NC_021162.1_17186988	Ca_kabuli_chr03	17186988	[T/G]	1067292839
Ca_Kabuli_NC_021162.1_17186990	Ca_kabuli_chr03	17186990	[G/T]	1067292840
Ca_Kabuli_NC_021162.1_17205756	Ca_kabuli_chr03	17205756	[C/A]	1067292841
Ca_Kabuli_NC_021162.1_17335816	Ca_kabuli_chr03	17335816	[T/A]	1067292842
Ca_Kabuli_NC_021162.1_17335817	Ca_kabuli_chr03	17335817	[A/T]	1067292843
Ca_Kabuli_NC_021162.1_17335831	Ca_kabuli_chr03	17335831	[A/G]	1067292844
Ca_Kabuli_NC_021162.1_17335839	Ca_kabuli_chr03	17335839	[T/A]	1067292845
Ca_Kabuli_NC_021162.1_17679400	Ca_kabuli_chr03	17679400	[G/C]	1067292846

SNP IDs	Chromosomes	Physical positions (bp)	SNPs	NCBI SNPdb accession IDs (NCBI_ss#)
Ca_Kabuli_NC_021162.1_17679402	Ca_kabuli_chr03	17679402	[T/G]	1067292847
Ca_Kabuli_NC_021162.1_17914677	Ca_kabuli_chr03	17914677	[T/G]	1067292848
Ca_Kabuli_NC_021162.1_17917871	Ca_kabuli_chr03	17917871	[T/G]	1067292849
Ca_Kabuli_NC_021162.1_17917890	Ca_kabuli_chr03	17917890	[T/C]	1067292850
Ca_Kabuli_NC_021162.1_17918036	Ca_kabuli_chr03	17918036	[A/C]	1067292851
Ca_Kabuli_NC_021162.1_17918037	Ca_kabuli_chr03	17918037	[G/A]	1067292852
Ca_Kabuli_NC_021162.1_17918038	Ca_kabuli_chr03	17918038	[A/C]	1067292853
Ca_Kabuli_NC_021162.1_17918039	Ca_kabuli_chr03	17918039	[T/A]	1067292854
Ca_Kabuli_NC_021162.1_17920352	Ca_kabuli_chr03	17920352	[A/G]	1067292855
Ca_Kabuli_NC_021162.1_17920362	Ca_kabuli_chr03	17920362	[A/G]	1067292856
Ca_Kabuli_NC_021162.1_17920365	Ca_kabuli_chr03	17920365	[A/C]	1067292857
Ca_Kabuli_NC_021162.1_17920367	Ca_kabuli_chr03	17920367	[A/C]	1067292858
Ca_Kabuli_NC_021162.1_17920406	Ca_kabuli_chr03	17920406	[A/G]	1067292859
Ca_Kabuli_NC_021162.1_17936579	Ca_kabuli_chr03	17936579	[T/G]	1067292860
Ca_Kabuli_NC_021162.1_17984963	Ca_kabuli_chr03	17984963	[T/G]	1067292861
Ca_Kabuli_NC_021162.1_17984974	Ca_kabuli_chr03	17984974	[A/G]	1067292862
Ca_Kabuli_NC_021162.1_17984978	Ca_kabuli_chr03	17984978	[T/G]	1067292863
Ca_Kabuli_NC_021162.1_17984981	Ca_kabuli_chr03	17984981	[T/G]	1067292864
Ca_Kabuli_NC_021162.1_18110539	Ca_kabuli_chr03	18110539	[C/A]	1067292865
Ca_Kabuli_NC_021162.1_18110584	Ca_kabuli_chr03	18110584	[A/G]	1067292866
Ca_Kabuli_NC_021162.1_18118249	Ca_kabuli_chr03	18118249	[G/T]	1067292867
Ca_Kabuli_NC_021162.1_18173457	Ca_kabuli_chr03	18173457	[A/T]	1067292868
Ca_Kabuli_NC_021162.1_18173467	Ca_kabuli_chr03	18173467	[A/G]	1067292869
Ca_Kabuli_NC_021162.1_18215380	Ca_kabuli_chr03	18215380	[G/A]	1067292870
Ca_Kabuli_NC_021162.1_18337498	Ca_kabuli_chr03	18337498	[T/G]	1067292871
Ca_Kabuli_NC_021162.1_18337501	Ca_kabuli_chr03	18337501	[A/T]	1067292872
Ca_Kabuli_NC_021162.1_18355403	Ca_kabuli_chr03	18355403	[T/G]	1067292873
Ca_Kabuli_NC_021162.1_18355409	Ca_kabuli_chr03	18355409	[A/C]	1067292874
Ca_Kabuli_NC_021162.1_18506357	Ca_kabuli_chr03	18506357	[G/A]	1067292875
Ca_Kabuli_NC_021162.1_18573699	Ca_kabuli_chr03	18573699	[A/C]	1067292876
Ca_Kabuli_NC_021162.1_18645184	Ca_kabuli_chr03	18645184	[A/C]	1067292877
Ca_Kabuli_NC_021162.1_18650824	Ca_kabuli_chr03	18650824	[T/A]	1067292878
Ca_Kabuli_NC_021162.1_18650838	Ca_kabuli_chr03	18650838	[T/C]	1067292879
Ca_Kabuli_NC_021162.1_18832490	Ca_kabuli_chr03	18832490	[T/G]	1067292880

SNP IDs	Chromosomes	Physical positions (bp)	SNPs	NCBI SNPdb accession IDs (NCBI_ss#)
Ca_Kabuli_NC_021162.1_18832514	Ca_kabuli_chr03	18832514	[A/G]	1067292881
Ca_Kabuli_NC_021162.1_18832517	Ca_kabuli_chr03	18832517	[A/G]	1067292882
Ca_Kabuli_NC_021162.1_18833450	Ca_kabuli_chr03	18833450	[T/C]	1067292883
Ca_Kabuli_NC_021162.1_18833484	Ca_kabuli_chr03	18833484	[A/G]	1067292884
Ca_Kabuli_NC_021162.1_18835241	Ca_kabuli_chr03	18835241	[A/C]	1067292885
Ca_Kabuli_NC_021162.1_18835286	Ca_kabuli_chr03	18835286	[A/G]	1067292886
Ca_Kabuli_NC_021162.1_18939339	Ca_kabuli_chr03	18939339	[T/C]	1067292887
Ca_Kabuli_NC_021162.1_18939424	Ca_kabuli_chr03	18939424	[A/C]	1067292888
Ca_Kabuli_NC_021162.1_19102331	Ca_kabuli_chr03	19102331	[C/T]	1067292889
Ca_Kabuli_NC_021162.1_19102333	Ca_kabuli_chr03	19102333	[C/G]	1067292890
Ca_Kabuli_NC_021162.1_19102337	Ca_kabuli_chr03	19102337	[C/T]	1067292891
Ca_Kabuli_NC_021162.1_19102339	Ca_kabuli_chr03	19102339	[A/C]	1067292892
Ca_Kabuli_NC_021162.1_19102345	Ca_kabuli_chr03	19102345	[A/T]	1067292893
Ca_Kabuli_NC_021162.1_19102358	Ca_kabuli_chr03	19102358	[A/G]	1067292894
Ca_Kabuli_NC_021162.1_19372802	Ca_kabuli_chr03	19372802	[A/T]	1067292895
Ca_Kabuli_NC_021162.1_19372804	Ca_kabuli_chr03	19372804	[A/C]	1067292896
Ca_Kabuli_NC_021162.1_19445920	Ca_kabuli_chr03	19445920	[T/A]	1067292897
Ca_Kabuli_NC_021162.1_19445925	Ca_kabuli_chr03	19445925	[A/G]	1067292898
Ca_Kabuli_NC_021162.1_19472565	Ca_kabuli_chr03	19472565	[T/C]	1067292899
Ca_Kabuli_NC_021162.1_19561510	Ca_kabuli_chr03	19561510	[T/G]	1067292900
Ca_Kabuli_NC_021162.1_19787136	Ca_kabuli_chr03	19787136	[T/C]	1067292901
Ca_Kabuli_NC_021162.1_19788683	Ca_kabuli_chr03	19788683	[T/G]	1067292902
Ca_Kabuli_NC_021162.1_19792757	Ca_kabuli_chr03	19792757	[T/C]	1067292903
Ca_Kabuli_NC_021162.1_19792758	Ca_kabuli_chr03	19792758	[G/T]	1067292904
Ca_Kabuli_NC_021162.1_19805016	Ca_kabuli_chr03	19805016	[T/C]	1067292905
Ca_Kabuli_NC_021162.1_19805030	Ca_kabuli_chr03	19805030	[A/C]	1067292906
Ca_Kabuli_NC_021162.1_19850575	Ca_kabuli_chr03	19850575	[A/C]	1067292907
Ca_Kabuli_NC_021162.1_19850583	Ca_kabuli_chr03	19850583	[A/C]	1067292908
Ca_Kabuli_NC_021162.1_19850645	Ca_kabuli_chr03	19850645	[A/C]	1067292909
Ca_Kabuli_NC_021162.1_19855208	Ca_kabuli_chr03	19855208	[A/C]	1067292910
Ca_Kabuli_NC_021162.1_19855230	Ca_kabuli_chr03	19855230	[T/C]	1067292911
Ca_Kabuli_NC_021162.1_19855235	Ca_kabuli_chr03	19855235	[T/C]	1067292912
Ca_Kabuli_NC_021162.1_19857584	Ca_kabuli_chr03	19857584	[T/G]	1067292913
Ca_Kabuli_NC_021162.1_19922709	Ca_kabuli_chr03	19922709	[C/A]	1067292914

SNP IDs	Chromosomes	Physical positions (bp)	SNPs	NCBI SNPdb accession IDs (NCBI_ss#)
Ca_Kabuli_NC_021162.1_19954392	Ca_kabuli_chr03	19954392	[T/A]	1067292915
Ca_Kabuli_NC_021162.1_19978334	Ca_kabuli_chr03	19978334	[A/C]	1067292916
Ca_Kabuli_NC_021162.1_19978343	Ca_kabuli_chr03	19978343	[A/C]	1067292917
Ca_Kabuli_NC_021162.1_20030427	Ca_kabuli_chr03	20030427	[T/G]	1067292918
Ca_Kabuli_NC_021162.1_20030447	Ca_kabuli_chr03	20030447	[A/T]	1067292919
Ca_Kabuli_NC_021162.1_20081473	Ca_kabuli_chr03	20081473	[A/G]	1067292920
Ca_Kabuli_NC_021162.1_20172508	Ca_kabuli_chr03	20172508	[A/T]	1067292921
Ca_Kabuli_NC_021162.1_20179054	Ca_kabuli_chr03	20179054	[A/G]	1067292922
Ca_Kabuli_NC_021162.1_20179062	Ca_kabuli_chr03	20179062	[T/G]	1067292923
Ca_Kabuli_NC_021162.1_20179729	Ca_kabuli_chr03	20179729	[T/G]	1067292924
Ca_Kabuli_NC_021162.1_20240118	Ca_kabuli_chr03	20240118	[T/G]	1067292925
Ca_Kabuli_NC_021162.1_20244346	Ca_kabuli_chr03	20244346	[A/C]	1067292926
Ca_Kabuli_NC_021162.1_20495749	Ca_kabuli_chr03	20495749	[C/A]	1067292927
Ca_Kabuli_NC_021162.1_20552836	Ca_kabuli_chr03	20552836	[T/G]	1067292928
Ca_Kabuli_NC_021162.1_20680409	Ca_kabuli_chr03	20680409	[A/T]	1067292929
Ca_Kabuli_NC_021162.1_20680438	Ca_kabuli_chr03	20680438	[T/C]	1067292930
Ca_Kabuli_NC_021162.1_20690198	Ca_kabuli_chr03	20690198	[A/T]	1067292931
Ca_Kabuli_NC_021162.1_20823116	Ca_kabuli_chr03	20823116	[A/C]	1067292932
Ca_Kabuli_NC_021162.1_20823144	Ca_kabuli_chr03	20823144	[T/C]	1067292933
Ca_Kabuli_NC_021162.1_21020225	Ca_kabuli_chr03	21020225	[T/G]	1067292934
Ca_Kabuli_NC_021162.1_21069658	Ca_kabuli_chr03	21069658	[A/C]	1067292935
Ca_Kabuli_NC_021162.1_21069672	Ca_kabuli_chr03	21069672	[A/G]	1067292936
Ca_Kabuli_NC_021162.1_21069690	Ca_kabuli_chr03	21069690	[T/A]	1067292937
Ca_Kabuli_NC_021162.1_21069905	Ca_kabuli_chr03	21069905	[T/C]	1067292938
Ca_Kabuli_NC_021162.1_21069917	Ca_kabuli_chr03	21069917	[A/G]	1067292939
Ca_Kabuli_NC_021162.1_21069935	Ca_kabuli_chr03	21069935	[T/A]	1067292940
Ca_Kabuli_NC_021162.1_21218591	Ca_kabuli_chr03	21218591	[A/T]	1067292941
Ca_Kabuli_NC_021162.1_21218610	Ca_kabuli_chr03	21218610	[A/G]	1067292942
Ca_Kabuli_NC_021162.1_21231086	Ca_kabuli_chr03	21231086	[T/G]	1067292943
Ca_Kabuli_NC_021162.1_21231111	Ca_kabuli_chr03	21231111	[A/C]	1067292944
Ca_Kabuli_NC_021162.1_21231140	Ca_kabuli_chr03	21231140	[T/C]	1067292945
Ca_Kabuli_NC_021162.1_21231160	Ca_kabuli_chr03	21231160	[A/C]	1067292946
Ca_Kabuli_NC_021162.1_21231163	Ca_kabuli_chr03	21231163	[A/C]	1067292947
Ca_Kabuli_NC_021162.1_21231172	Ca_kabuli_chr03	21231172	[T/C]	1067292948

SNP IDs	Chromosomes	Physical positions (bp)	SNPs	NCBI SNPdb accession IDs (NCBI_ss#)
Ca_Kabuli_NC_021162.1_21231187	Ca_kabuli_chr03	21231187	[T/A]	1067292949
Ca_Kabuli_NC_021162.1_21231229	Ca_kabuli_chr03	21231229	[T/C]	1067292950
Ca_Kabuli_NC_021162.1_21231232	Ca_kabuli_chr03	21231232	[T/C]	1067292951
Ca_Kabuli_NC_021162.1_21231265	Ca_kabuli_chr03	21231265	[A/C]	1067292952
Ca_Kabuli_NC_021162.1_21231277	Ca_kabuli_chr03	21231277	[A/C]	1067292953
Ca_Kabuli_NC_021162.1_21232585	Ca_kabuli_chr03	21232585	[A/C]	1067292954
Ca_Kabuli_NC_021162.1_21272962	Ca_kabuli_chr03	21272962	[A/C]	1067292955
Ca_Kabuli_NC_021162.1_21273423	Ca_kabuli_chr03	21273423	[T/A]	1067292956
Ca_Kabuli_NC_021162.1_21278656	Ca_kabuli_chr03	21278656	[T/C]	1067292957
Ca_Kabuli_NC_021162.1_21278657	Ca_kabuli_chr03	21278657	[G/T]	1067292958
Ca_Kabuli_NC_021162.1_21288619	Ca_kabuli_chr03	21288619	[T/A]	1067292959
Ca_Kabuli_NC_021162.1_21315975	Ca_kabuli_chr03	21315975	[A/C]	1067292960
Ca_Kabuli_NC_021162.1_21315978	Ca_kabuli_chr03	21315978	[A/C]	1067292961
Ca_Kabuli_NC_021162.1_21315987	Ca_kabuli_chr03	21315987	[A/T]	1067292962
Ca_Kabuli_NC_021162.1_21382890	Ca_kabuli_chr03	21382890	[A/T]	1067292963
Ca_Kabuli_NC_021162.1_21382893	Ca_kabuli_chr03	21382893	[C/T]	1067292964
Ca_Kabuli_NC_021162.1_21421225	Ca_kabuli_chr03	21421225	[T/G]	1067292965
Ca_Kabuli_NC_021162.1_21421464	Ca_kabuli_chr03	21421464	[A/G]	1067292966
Ca_Kabuli_NC_021162.1_21421539	Ca_kabuli_chr03	21421539	[A/C]	1067292967
Ca_Kabuli_NC_021162.1_21435569	Ca_kabuli_chr03	21435569	[A/T]	1067292968
Ca_Kabuli_NC_021162.1_21445329	Ca_kabuli_chr03	21445329	[T/G]	1067292969
Ca_Kabuli_NC_021162.1_21445352	Ca_kabuli_chr03	21445352	[T/G]	1067292970
Ca_Kabuli_NC_021162.1_21446288	Ca_kabuli_chr03	21446288	[A/G]	1067292971
Ca_Kabuli_NC_021162.1_21489237	Ca_kabuli_chr03	21489237	[A/C]	1067292972
Ca_Kabuli_NC_021162.1_21489262	Ca_kabuli_chr03	21489262	[A/C]	1067292973
Ca_Kabuli_NC_021162.1_21524254	Ca_kabuli_chr03	21524254	[A/C]	1067292974
Ca_Kabuli_NC_021162.1_21531470	Ca_kabuli_chr03	21531470	[A/C]	1067292975
Ca_Kabuli_NC_021162.1_21534108	Ca_kabuli_chr03	21534108	[A/T]	1067292976
Ca_Kabuli_NC_021162.1_21558364	Ca_kabuli_chr03	21558364	[T/C]	1067292977
Ca_Kabuli_NC_021162.1_21558387	Ca_kabuli_chr03	21558387	[T/A]	1067292978
Ca_Kabuli_NC_021162.1_21558393	Ca_kabuli_chr03	21558393	[A/G]	1067292979
Ca_Kabuli_NC_021162.1_21558403	Ca_kabuli_chr03	21558403	[A/G]	1067292980
Ca_Kabuli_NC_021162.1_21558424	Ca_kabuli_chr03	21558424	[T/C]	1067292981
Ca_Kabuli_NC_021162.1_21627581	Ca_kabuli_chr03	21627581	[T/G]	1067292982

SNP IDs	Chromosomes	Physical positions (bp)	SNPs	NCBI SNPdb accession IDs (NCBI_ss#)
Ca_Kabuli_NC_021162.1_21627668	Ca_kabuli_chr03	21627668	[T/G]	1067292983
Ca_Kabuli_NC_021162.1_21627698	Ca_kabuli_chr03	21627698	[T/G]	1067292984
Ca_Kabuli_NC_021162.1_21646857	Ca_kabuli_chr03	21646857	[T/C]	1067292985
Ca_Kabuli_NC_021162.1_21646881	Ca_kabuli_chr03	21646881	[A/G]	1067292986
Ca_Kabuli_NC_021162.1_21646953	Ca_kabuli_chr03	21646953	[T/C]	1067292987
Ca_Kabuli_NC_021162.1_21657205	Ca_kabuli_chr03	21657205	[C/A]	1067292988
Ca_Kabuli_NC_021162.1_21657206	Ca_kabuli_chr03	21657206	[A/G]	1067292989
Ca_Kabuli_NC_021162.1_21686530	Ca_kabuli_chr03	21686530	[T/G]	1067292990
Ca_Kabuli_NC_021162.1_21687416	Ca_kabuli_chr03	21687416	[A/T]	1067292991
Ca_Kabuli_NC_021162.1_21687443	Ca_kabuli_chr03	21687443	[T/A]	1067292992
Ca_Kabuli_NC_021162.1_21687959	Ca_kabuli_chr03	21687959	[A/C]	1067292993
Ca_Kabuli_NC_021162.1_21687960	Ca_kabuli_chr03	21687960	[A/T]	1067292994
Ca_Kabuli_NC_021162.1_21687962	Ca_kabuli_chr03	21687962	[T/G]	1067292995
Ca_Kabuli_NC_021162.1_21687964	Ca_kabuli_chr03	21687964	[A/T]	1067292996
Ca_Kabuli_NC_021162.1_21687966	Ca_kabuli_chr03	21687966	[C/A]	1067292997
Ca_Kabuli_NC_021162.1_21687969	Ca_kabuli_chr03	21687969	[G/A]	1067292998
Ca_Kabuli_NC_021162.1_21687978	Ca_kabuli_chr03	21687978	[A/G]	1067292999
Ca_Kabuli_NC_021162.1_21700173	Ca_kabuli_chr03	21700173	[T/A]	1067293000
Ca_Kabuli_NC_021162.1_21700175	Ca_kabuli_chr03	21700175	[A/G]	1067293001
Ca_Kabuli_NC_021162.1_21700206	Ca_kabuli_chr03	21700206	[A/G]	1067293002
Ca_Kabuli_NC_021162.1_21744356	Ca_kabuli_chr03	21744356	[A/C]	1067293003
Ca_Kabuli_NC_021162.1_21863769	Ca_kabuli_chr03	21863769	[C/A]	1067293004
Ca_Kabuli_NC_021162.1_22007864	Ca_kabuli_chr03	22007864	[A/G]	1067293005
Ca_Kabuli_NC_021162.1_22007902	Ca_kabuli_chr03	22007902	[T/G]	1067293006
Ca_Kabuli_NC_021162.1_22007904	Ca_kabuli_chr03	22007904	[T/G]	1067293007
Ca_Kabuli_NC_021162.1_22007924	Ca_kabuli_chr03	22007924	[T/C]	1067293008
Ca_Kabuli_NC_021162.1_22009451	Ca_kabuli_chr03	22009451	[A/C]	1067293009
Ca_Kabuli_NC_021162.1_22009465	Ca_kabuli_chr03	22009465	[T/G]	1067293010
Ca_Kabuli_NC_021162.1_22014101	Ca_kabuli_chr03	22014101	[A/G]	1067293011
Ca_Kabuli_NC_021162.1_22014121	Ca_kabuli_chr03	22014121	[T/C]	1067293012
Ca_Kabuli_NC_021162.1_22015131	Ca_kabuli_chr03	22015131	[T/G]	1067293013
Ca_Kabuli_NC_021162.1_22060236	Ca_kabuli_chr03	22060236	[A/C]	1067293014
Ca_Kabuli_NC_021162.1_22061919	Ca_kabuli_chr03	22061919	[A/C]	1067293015
Ca_Kabuli_NC_021162.1_22185338	Ca_kabuli_chr03	22185338	[A/G]	1067293016

SNP IDs	Chromosomes	Physical positions (bp)	SNPs	NCBI SNPdb accession IDs (NCBI_ss#)
Ca_Kabuli_NC_021162.1_22185431	Ca_kabuli_chr03	22185431	[T/G]	1067293017
Ca_Kabuli_NC_021162.1_22187794	Ca_kabuli_chr03	22187794	[A/C]	1067293018
Ca_Kabuli_NC_021162.1_22188162	Ca_kabuli_chr03	22188162	[A/C]	1067293019
Ca_Kabuli_NC_021162.1_22199967	Ca_kabuli_chr03	22199967	[T/A]	1067293020
Ca_Kabuli_NC_021162.1_22200730	Ca_kabuli_chr03	22200730	[A/C]	1067293021
Ca_Kabuli_NC_021162.1_22202826	Ca_kabuli_chr03	22202826	[A/C]	1067293022
Ca_Kabuli_NC_021162.1_22209591	Ca_kabuli_chr03	22209591	[T/C]	1067293023
Ca_Kabuli_NC_021162.1_22225100	Ca_kabuli_chr03	22225100	[G/T]	1067293024
Ca_Kabuli_NC_021162.1_22225125	Ca_kabuli_chr03	22225125	[G/T]	1067293025
Ca_Kabuli_NC_021162.1_22225128	Ca_kabuli_chr03	22225128	[G/T]	1067293026
Ca_Kabuli_NC_021162.1_22225134	Ca_kabuli_chr03	22225134	[A/T]	1067293027
Ca_Kabuli_NC_021162.1_22225139	Ca_kabuli_chr03	22225139	[G/T]	1067293028
Ca_Kabuli_NC_021162.1_22294715	Ca_kabuli_chr03	22294715	[T/C]	1067293029
Ca_Kabuli_NC_021162.1_22294722	Ca_kabuli_chr03	22294722	[A/T]	1067293030
Ca_Kabuli_NC_021162.1_22294727	Ca_kabuli_chr03	22294727	[T/C]	1067293031
Ca_Kabuli_NC_021162.1_22294753	Ca_kabuli_chr03	22294753	[A/G]	1067293032
Ca_Kabuli_NC_021162.1_22329341	Ca_kabuli_chr03	22329341	[T/G]	1067293033
Ca_Kabuli_NC_021162.1_22345311	Ca_kabuli_chr03	22345311	[T/C]	1067293034
Ca_Kabuli_NC_021162.1_22346166	Ca_kabuli_chr03	22346166	[A/C]	1067293035
Ca_Kabuli_NC_021162.1_22407084	Ca_kabuli_chr03	22407084	[A/C]	1067293036
Ca_Kabuli_NC_021162.1_22462445	Ca_kabuli_chr03	22462445	[C/A]	1067293037
Ca_Kabuli_NC_021162.1_22462446	Ca_kabuli_chr03	22462446	[T/G]	1067293038
Ca_Kabuli_NC_021162.1_22462448	Ca_kabuli_chr03	22462448	[G/T]	1067293039
Ca_Kabuli_NC_021162.1_22539439	Ca_kabuli_chr03	22539439	[A/C]	1067293040
Ca_Kabuli_NC_021162.1_22539555	Ca_kabuli_chr03	22539555	[A/C]	1067293041
Ca_Kabuli_NC_021162.1_22539925	Ca_kabuli_chr03	22539925	[T/C]	1067293042
Ca_Kabuli_NC_021162.1_22539942	Ca_kabuli_chr03	22539942	[A/G]	1067293043
Ca_Kabuli_NC_021162.1_22610064	Ca_kabuli_chr03	22610064	[T/C]	1067293044
Ca_Kabuli_NC_021162.1_22610071	Ca_kabuli_chr03	22610071	[T/G]	1067293045
Ca_Kabuli_NC_021162.1_22610073	Ca_kabuli_chr03	22610073	[T/G]	1067293046
Ca_Kabuli_NC_021162.1_22610112	Ca_kabuli_chr03	22610112	[T/C]	1067293047
Ca_Kabuli_NC_021162.1_22690218	Ca_kabuli_chr03	22690218	[T/C]	1067293048
Ca_Kabuli_NC_021162.1_22696727	Ca_kabuli_chr03	22696727	[A/T]	1067293049
Ca_Kabuli_NC_021162.1_22718070	Ca_kabuli_chr03	22718070	[T/C]	1067293050

**SNP IDs**	**Chromosomes**	**Physical positions (bp)**	**SNPs**	**NCBI SNPdb accession IDs (NCBI_ss#)**
Ca_Kabuli_NC_021162.1_22718091	Ca_kabuli_chr03	22718091	[T/G]	1067293051
Ca_Kabuli_NC_021162.1_22719600	Ca_kabuli_chr03	22719600	[C/A]	1067293052
Ca_Kabuli_NC_021162.1_22719603	Ca_kabuli_chr03	22719603	[A/C]	1067293053
Ca_Kabuli_NC_021162.1_22719605	Ca_kabuli_chr03	22719605	[T/G]	1067293054
Ca_Kabuli_NC_021162.1_22727382	Ca_kabuli_chr03	22727382	[T/C]	1067293055
Ca_Kabuli_NC_021162.1_22730700	Ca_kabuli_chr03	22730700	[T/G]	1067293056
Ca_Kabuli_NC_021162.1_22772522	Ca_kabuli_chr03	22772522	[A/C]	1067293057
Ca_Kabuli_NC_021162.1_22772533	Ca_kabuli_chr03	22772533	[A/G]	1067293058
Ca_Kabuli_NC_021162.1_22794680	Ca_kabuli_chr03	22794680	[A/T]	1067293059
Ca_Kabuli_NC_021162.1_22794692	Ca_kabuli_chr03	22794692	[T/A]	1067293060
Ca_Kabuli_NC_021162.1_22812683	Ca_kabuli_chr03	22812683	[T/G]	1067293061
Ca_Kabuli_NC_021162.1_22812692	Ca_kabuli_chr03	22812692	[T/G]	1067293062
Ca_Kabuli_NC_021162.1_22849461	Ca_kabuli_chr03	22849461	[A/G]	1067293063
Ca_Kabuli_NC_021162.1_22849484	Ca_kabuli_chr03	22849484	[T/C]	1067293064
Ca_Kabuli_NC_021162.1_22868707	Ca_kabuli_chr03	22868707	[A/G]	1067293065
Ca_Kabuli_NC_021162.1_22868717	Ca_kabuli_chr03	22868717	[A/C]	1067293066
Ca_Kabuli_NC_021162.1_22868721	Ca_kabuli_chr03	22868721	[A/C]	1067293067
Ca_Kabuli_NC_021162.1_22927162	Ca_kabuli_chr03	22927162	[T/G]	1067293068
Ca_Kabuli_NC_021162.1_22937333	Ca_kabuli_chr03	22937333	[C/T]	1067293069
Ca_Kabuli_NC_021162.1_22937403	Ca_kabuli_chr03	22937403	[G/T]	1067293070
Ca_Kabuli_NC_021162.1_22993218	Ca_kabuli_chr03	22993218	[A/C]	1067293071
Ca_Kabuli_NC_021162.1_22993243	Ca_kabuli_chr03	22993243	[A/C]	1067293072
Ca_Kabuli_NC_021162.1_22995324	Ca_kabuli_chr03	22995324	[A/C]	1067293073
Ca_Kabuli_NC_021162.1_23042271	Ca_kabuli_chr03	23042271	[T/A]	1067293074
Ca_Kabuli_NC_021162.1_23042281	Ca_kabuli_chr03	23042281	[A/C]	1067293075
Ca_Kabuli_NC_021162.1_23053022	Ca_kabuli_chr03	23053022	[T/G]	1067293076
Ca_Kabuli_NC_021162.1_23222201	Ca_kabuli_chr03	23222201	[C/T]	1067293077
Ca_Kabuli_NC_021162.1_23222202	Ca_kabuli_chr03	23222202	[T/C]	1067293078
Ca_Kabuli_NC_021162.1_23222203	Ca_kabuli_chr03	23222203	[T/C]	1067293079
Ca_Kabuli_NC_021162.1_23271812	Ca_kabuli_chr03	23271812	[T/C]	1067293080
Ca_Kabuli_NC_021162.1_23271823	Ca_kabuli_chr03	23271823	[A/G]	1067293081
Ca_Kabuli_NC_021162.1_23273108	Ca_kabuli_chr03	23273108	[G/T]	1067293082
Ca_Kabuli_NC_021162.1_23273222	Ca_kabuli_chr03	23273222	[T/C]	1067293083
Ca_Kabuli_NC_021162.1_23275720	Ca_kabuli_chr03	23275720	[T/A]	1067293084

SNP IDs	Chromosomes	Physical positions (bp)	SNPs	NCBI SNPdb accession IDs (NCBI_ss#)
Ca_Kabuli_NC_021162.1_23275763	Ca_kabuli_chr03	23275763	[A/C]	1067293085
Ca_Kabuli_NC_021162.1_23298846	Ca_kabuli_chr03	23298846	[A/G]	1067293086
Ca_Kabuli_NC_021162.1_23303162	Ca_kabuli_chr03	23303162	[A/G]	1067293087
Ca_Kabuli_NC_021162.1_23340881	Ca_kabuli_chr03	23340881	[T/C]	1067293088
Ca_Kabuli_NC_021162.1_23340882	Ca_kabuli_chr03	23340882	[T/C]	1067293089
Ca_Kabuli_NC_021162.1_23340883	Ca_kabuli_chr03	23340883	[T/C]	1067293090
Ca_Kabuli_NC_021162.1_23340884	Ca_kabuli_chr03	23340884	[T/C]	1067293091
Ca_Kabuli_NC_021162.1_23340885	Ca_kabuli_chr03	23340885	[T/C]	1067293092
Ca_Kabuli_NC_021162.1_23340886	Ca_kabuli_chr03	23340886	[T/A]	1067293093
Ca_Kabuli_NC_021162.1_23340887	Ca_kabuli_chr03	23340887	[A/C]	1067293094
Ca_Kabuli_NC_021162.1_23341077	Ca_kabuli_chr03	23341077	[A/C]	1067293095
Ca_Kabuli_NC_021162.1_23341373	Ca_kabuli_chr03	23341373	[A/G]	1067293096
Ca_Kabuli_NC_021162.1_23401529	Ca_kabuli_chr03	23401529	[A/C]	1067293097
Ca_Kabuli_NC_021162.1_23401530	Ca_kabuli_chr03	23401530	[G/A]	1067293098
Ca_Kabuli_NC_021162.1_23401531	Ca_kabuli_chr03	23401531	[A/C]	1067293099
Ca_Kabuli_NC_021162.1_23401532	Ca_kabuli_chr03	23401532	[T/A]	1067293100
Ca_Kabuli_NC_021162.1_23444074	Ca_kabuli_chr03	23444074	[A/C]	1067293101
Ca_Kabuli_NC_021162.1_23461331	Ca_kabuli_chr03	23461331	[T/C]	1067293102
Ca_Kabuli_NC_021162.1_23482443	Ca_kabuli_chr03	23482443	[A/G]	1067293103
Ca_Kabuli_NC_021162.1_23694570	Ca_kabuli_chr03	23694570	[A/G]	1067293104
Ca_Kabuli_NC_021162.1_23694597	Ca_kabuli_chr03	23694597	[A/G]	1067293105
Ca_Kabuli_NC_021162.1_23695129	Ca_kabuli_chr03	23695129	[A/C]	1067293106
Ca_Kabuli_NC_021162.1_23695185	Ca_kabuli_chr03	23695185	[T/C]	1067293107
Ca_Kabuli_NC_021162.1_23721066	Ca_kabuli_chr03	23721066	[A/C]	1067293108
Ca_Kabuli_NC_021162.1_23727724	Ca_kabuli_chr03	23727724	[T/G]	1067293109
Ca_Kabuli_NC_021162.1_23727749	Ca_kabuli_chr03	23727749	[T/G]	1067293110
Ca_Kabuli_NC_021162.1_23765099	Ca_kabuli_chr03	23765099	[A/C]	1067293111
Ca_Kabuli_NC_021162.1_23780028	Ca_kabuli_chr03	23780028	[T/G]	1067293112
Ca_Kabuli_NC_021162.1_23800346	Ca_kabuli_chr03	23800346	[A/T]	1067293113
Ca_Kabuli_NC_021162.1_23800411	Ca_kabuli_chr03	23800411	[T/C]	1067293114
Ca_Kabuli_NC_021162.1_23800432	Ca_kabuli_chr03	23800432	[A/T]	1067293115
Ca_Kabuli_NC_021162.1_23800459	Ca_kabuli_chr03	23800459	[A/G]	1067293116
Ca_Kabuli_NC_021162.1_23865001	Ca_kabuli_chr03	23865001	[A/C]	1067293117
Ca_Kabuli_NC_021162.1_23930065	Ca_kabuli_chr03	23930065	[T/G]	1067293118

SNP IDs	Chromosomes	Physical positions (bp)	SNPs	NCBI SNPdb accession IDs (NCBI_ss#)
Ca_Kabuli_NC_021162.1_23969850	Ca_kabuli_chr03	23969850	[T/G]	1067293119
Ca_Kabuli_NC_021162.1_23978265	Ca_kabuli_chr03	23978265	[T/A]	1067293120
Ca_Kabuli_NC_021162.1_24024538	Ca_kabuli_chr03	24024538	[A/C]	1067293121
Ca_Kabuli_NC_021162.1_24024544	Ca_kabuli_chr03	24024544	[A/C]	1067293122
Ca_Kabuli_NC_021162.1_24024548	Ca_kabuli_chr03	24024548	[T/C]	1067293123
Ca_Kabuli_NC_021162.1_24024554	Ca_kabuli_chr03	24024554	[T/C]	1067293124
Ca_Kabuli_NC_021162.1_24024661	Ca_kabuli_chr03	24024661	[C/A]	1067293125
Ca_Kabuli_NC_021162.1_24024662	Ca_kabuli_chr03	24024662	[T/G]	1067293126
Ca_Kabuli_NC_021162.1_24025826	Ca_kabuli_chr03	24025826	[A/C]	1067293127
Ca_Kabuli_NC_021162.1_24026374	Ca_kabuli_chr03	24026374	[T/C]	1067293128
Ca_Kabuli_NC_021162.1_24064895	Ca_kabuli_chr03	24064895	[A/G]	1067293129
Ca_Kabuli_NC_021162.1_24084754	Ca_kabuli_chr03	24084754	[G/T]	1067293130
Ca_Kabuli_NC_021162.1_24084757	Ca_kabuli_chr03	24084757	[A/T]	1067293131
Ca_Kabuli_NC_021162.1_24084788	Ca_kabuli_chr03	24084788	[T/C]	1067293132
Ca_Kabuli_NC_021162.1_24162196	Ca_kabuli_chr03	24162196	[T/C]	1067293133
Ca_Kabuli_NC_021162.1_24194574	Ca_kabuli_chr03	24194574	[T/C]	1067293134
Ca_Kabuli_NC_021162.1_24238308	Ca_kabuli_chr03	24238308	[T/C]	1067293135
Ca_Kabuli_NC_021162.1_24240003	Ca_kabuli_chr03	24240003	[T/C]	1067293136
Ca_Kabuli_NC_021162.1_24240011	Ca_kabuli_chr03	24240011	[T/G]	1067293137
Ca_Kabuli_NC_021162.1_24240019	Ca_kabuli_chr03	24240019	[G/A]	1067293138
Ca_Kabuli_NC_021162.1_24240022	Ca_kabuli_chr03	24240022	[T/A]	1067293139
Ca_Kabuli_NC_021162.1_24240025	Ca_kabuli_chr03	24240025	[G/A]	1067293140
Ca_Kabuli_NC_021162.1_24240028	Ca_kabuli_chr03	24240028	[G/A]	1067293141
Ca_Kabuli_NC_021162.1_24247162	Ca_kabuli_chr03	24247162	[T/C]	1067293142
Ca_Kabuli_NC_021162.1_24262412	Ca_kabuli_chr03	24262412	[C/A]	1067293143
Ca_Kabuli_NC_021162.1_24262413	Ca_kabuli_chr03	24262413	[C/G]	1067293144
Ca_Kabuli_NC_021162.1_24262414	Ca_kabuli_chr03	24262414	[T/A]	1067293145
Ca_Kabuli_NC_021162.1_24262415	Ca_kabuli_chr03	24262415	[C/T]	1067293146
Ca_Kabuli_NC_021162.1_24297032	Ca_kabuli_chr03	24297032	[T/C]	1067293147
Ca_Kabuli_NC_021162.1_24297033	Ca_kabuli_chr03	24297033	[A/C]	1067293148
Ca_Kabuli_NC_021162.1_24297866	Ca_kabuli_chr03	24297866	[A/C]	1067293149
Ca_Kabuli_NC_021162.1_24297875	Ca_kabuli_chr03	24297875	[A/G]	1067293150
Ca_Kabuli_NC_021162.1_24297887	Ca_kabuli_chr03	24297887	[T/C]	1067293151
Ca_Kabuli_NC_021162.1_24390790	Ca_kabuli_chr03	24390790	[T/G]	1067293152

SNP IDs	Chromosomes	Physical positions (bp)	SNPs	NCBI SNPdb accession IDs (NCBI_ss#)
Ca_Kabuli_NC_021162.1_24390802	Ca_kabuli_chr03	24390802	[T/G]	1067293153
Ca_Kabuli_NC_021162.1_24390832	Ca_kabuli_chr03	24390832	[T/G]	1067293154
Ca_Kabuli_NC_021162.1_24390881	Ca_kabuli_chr03	24390881	[A/T]	1067293155
Ca_Kabuli_NC_021162.1_24417498	Ca_kabuli_chr03	24417498	[A/C]	1067293156
Ca_Kabuli_NC_021162.1_24417507	Ca_kabuli_chr03	24417507	[A/G]	1067293157
Ca_Kabuli_NC_021162.1_24418066	Ca_kabuli_chr03	24418066	[T/G]	1067293158
Ca_Kabuli_NC_021162.1_24459620	Ca_kabuli_chr03	24459620	[T/A]	1067293159
Ca_Kabuli_NC_021162.1_24481380	Ca_kabuli_chr03	24481380	[T/C]	1067293160
Ca_Kabuli_NC_021162.1_24481409	Ca_kabuli_chr03	24481409	[A/G]	1067293161
Ca_Kabuli_NC_021162.1_24540205	Ca_kabuli_chr03	24540205	[T/G]	1067293162
Ca_Kabuli_NC_021162.1_24540313	Ca_kabuli_chr03	24540313	[T/G]	1067293163
Ca_Kabuli_NC_021162.1_24637837	Ca_kabuli_chr03	24637837	[A/C]	1067293164
Ca_Kabuli_NC_021162.1_24697785	Ca_kabuli_chr03	24697785	[A/T]	1067293165
Ca_Kabuli_NC_021162.1_24798127	Ca_kabuli_chr03	24798127	[A/G]	1067293166
Ca_Kabuli_NC_021162.1_24880665	Ca_kabuli_chr03	24880665	[T/G]	1067293167
Ca_Kabuli_NC_021162.1_24894262	Ca_kabuli_chr03	24894262	[G/C]	1067293168
Ca_Kabuli_NC_021162.1_24935188	Ca_kabuli_chr03	24935188	[T/C]	1067293169
Ca_Kabuli_NC_021162.1_25032514	Ca_kabuli_chr03	25032514	[A/C]	1067293170
Ca_Kabuli_NC_021162.1_25032520	Ca_kabuli_chr03	25032520	[A/G]	1067293171
Ca_Kabuli_NC_021162.1_25046405	Ca_kabuli_chr03	25046405	[T/G]	1067293172
Ca_Kabuli_NC_021162.1_25046559	Ca_kabuli_chr03	25046559	[T/A]	1067293173
Ca_Kabuli_NC_021162.1_25046562	Ca_kabuli_chr03	25046562	[T/A]	1067293174
Ca_Kabuli_NC_021162.1_25145247	Ca_kabuli_chr03	25145247	[T/C]	1067293175
Ca_Kabuli_NC_021162.1_25244223	Ca_kabuli_chr03	25244223	[A/C]	1067293176
Ca_Kabuli_NC_021162.1_25244226	Ca_kabuli_chr03	25244226	[A/C]	1067293177
Ca_Kabuli_NC_021162.1_25244244	Ca_kabuli_chr03	25244244	[A/G]	1067293178
Ca_Kabuli_NC_021162.1_25311403	Ca_kabuli_chr03	25311403	[T/A]	1067293179
Ca_Kabuli_NC_021162.1_25333190	Ca_kabuli_chr03	25333190	[T/C]	1067293180
Ca_Kabuli_NC_021162.1_25333208	Ca_kabuli_chr03	25333208	[T/A]	1067293181
Ca_Kabuli_NC_021162.1_25385388	Ca_kabuli_chr03	25385388	[T/C]	1067293182
Ca_Kabuli_NC_021162.1_25385389	Ca_kabuli_chr03	25385389	[T/G]	1067293183
Ca_Kabuli_NC_021162.1_25385394	Ca_kabuli_chr03	25385394	[C/A]	1067293184
Ca_Kabuli_NC_021162.1_25385425	Ca_kabuli_chr03	25385425	[A/C]	1067293185
Ca_Kabuli_NC_021162.1_25444137	Ca_kabuli_chr03	25444137	[A/C]	1067293186

SNP IDs	Chromosomes	Physical positions (bp)	SNPs	NCBI SNPdb accession IDs (NCBI_ss#)
Ca_Kabuli_NC_021162.1_25462227	Ca_kabuli_chr03	25462227	[A/C]	1067293187
Ca_Kabuli_NC_021162.1_25505355	Ca_kabuli_chr03	25505355	[T/G]	1067293188
Ca_Kabuli_NC_021162.1_25505442	Ca_kabuli_chr03	25505442	[A/G]	1067293189
Ca_Kabuli_NC_021162.1_25537761	Ca_kabuli_chr03	25537761	[T/A]	1067293190
Ca_Kabuli_NC_021162.1_25537766	Ca_kabuli_chr03	25537766	[T/A]	1067293191
Ca_Kabuli_NC_021162.1_25537803	Ca_kabuli_chr03	25537803	[A/G]	1067293192
Ca_Kabuli_NC_021162.1_25579982	Ca_kabuli_chr03	25579982	[C/A]	1067293193
Ca_Kabuli_NC_021162.1_25579983	Ca_kabuli_chr03	25579983	[A/G]	1067293194
Ca_Kabuli_NC_021162.1_25579984	Ca_kabuli_chr03	25579984	[T/A]	1067293195
Ca_Kabuli_NC_021162.1_25580084	Ca_kabuli_chr03	25580084	[T/G]	1067293196
Ca_Kabuli_NC_021162.1_25580104	Ca_kabuli_chr03	25580104	[A/C]	1067293197
Ca_Kabuli_NC_021162.1_25614515	Ca_kabuli_chr03	25614515	[T/G]	1067293198
Ca_Kabuli_NC_021162.1_25673614	Ca_kabuli_chr03	25673614	[A/G]	1067293199
Ca_Kabuli_NC_021162.1_25675094	Ca_kabuli_chr03	25675094	[A/T]	1067293200
Ca_Kabuli_NC_021162.1_25688342	Ca_kabuli_chr03	25688342	[T/C]	1067293201
Ca_Kabuli_NC_021162.1_25688408	Ca_kabuli_chr03	25688408	[A/C]	1067293202
Ca_Kabuli_NC_021162.1_25702286	Ca_kabuli_chr03	25702286	[T/G]	1067293203
Ca_Kabuli_NC_021162.1_25711777	Ca_kabuli_chr03	25711777	[T/G]	1067293204
Ca_Kabuli_NC_021162.1_25757728	Ca_kabuli_chr03	25757728	[T/G]	1067293205
Ca_Kabuli_NC_021162.1_25757753	Ca_kabuli_chr03	25757753	[A/G]	1067293206
Ca_Kabuli_NC_021162.1_25822560	Ca_kabuli_chr03	25822560	[T/C]	1067293207
Ca_Kabuli_NC_021162.1_25822584	Ca_kabuli_chr03	25822584	[T/A]	1067293208
Ca_Kabuli_NC_021162.1_25987849	Ca_kabuli_chr03	25987849	[A/T]	1067293209
Ca_Kabuli_NC_021162.1_26120260	Ca_kabuli_chr03	26120260	[A/T]	1067293210
Ca_Kabuli_NC_021162.1_26120261	Ca_kabuli_chr03	26120261	[T/C]	1067293211
Ca_Kabuli_NC_021162.1_26120262	Ca_kabuli_chr03	26120262	[C/G]	1067293212
Ca_Kabuli_NC_021162.1_26120263	Ca_kabuli_chr03	26120263	[T/G]	1067293213
Ca_Kabuli_NC_021162.1_26178545	Ca_kabuli_chr03	26178545	[T/C]	1067293214
Ca_Kabuli_NC_021162.1_26178553	Ca_kabuli_chr03	26178553	[T/C]	1067293215
Ca_Kabuli_NC_021162.1_26198241	Ca_kabuli_chr03	26198241	[T/A]	1067293216
Ca_Kabuli_NC_021162.1_26198286	Ca_kabuli_chr03	26198286	[A/G]	1067293217
Ca_Kabuli_NC_021162.1_26198292	Ca_kabuli_chr03	26198292	[A/G]	1067293218
Ca_Kabuli_NC_021162.1_26322681	Ca_kabuli_chr03	26322681	[T/G]	1067293219
Ca_Kabuli_NC_021162.1_26322700	Ca_kabuli_chr03	26322700	[G/A]	1067293220

SNP IDs	Chromosomes	Physical positions (bp)	SNPs	NCBI SNPdb accession IDs (NCBI_ss#)
Ca_Kabuli_NC_021162.1_26322701	Ca_kabuli_chr03	26322701	[T/G]	1067293221
Ca_Kabuli_NC_021162.1_26335843	Ca_kabuli_chr03	26335843	[T/G]	1067293222
Ca_Kabuli_NC_021162.1_26340845	Ca_kabuli_chr03	26340845	[T/C]	1067293223
Ca_Kabuli_NC_021162.1_26340848	Ca_kabuli_chr03	26340848	[T/A]	1067293224
Ca_Kabuli_NC_021162.1_26340920	Ca_kabuli_chr03	26340920	[A/C]	1067293225
Ca_Kabuli_NC_021162.1_26340924	Ca_kabuli_chr03	26340924	[A/C]	1067293226
Ca_Kabuli_NC_021162.1_26344534	Ca_kabuli_chr03	26344534	[A/G]	1067293227
Ca_Kabuli_NC_021162.1_26344535	Ca_kabuli_chr03	26344535	[A/G]	1067293228
Ca_Kabuli_NC_021162.1_26557385	Ca_kabuli_chr03	26557385	[A/G]	1067293229
Ca_Kabuli_NC_021162.1_26573634	Ca_kabuli_chr03	26573634	[A/T]	1067293230
Ca_Kabuli_NC_021162.1_26573636	Ca_kabuli_chr03	26573636	[T/C]	1067293231
Ca_Kabuli_NC_021162.1_26573647	Ca_kabuli_chr03	26573647	[T/A]	1067293232
Ca_Kabuli_NC_021162.1_26573660	Ca_kabuli_chr03	26573660	[T/C]	1067293233
Ca_Kabuli_NC_021162.1_26624198	Ca_kabuli_chr03	26624198	[T/C]	1067293234
Ca_Kabuli_NC_021162.1_26624219	Ca_kabuli_chr03	26624219	[A/C]	1067293235
Ca_Kabuli_NC_021162.1_26624244	Ca_kabuli_chr03	26624244	[T/C]	1067293236
Ca_Kabuli_NC_021162.1_26624258	Ca_kabuli_chr03	26624258	[A/G]	1067293237
Ca_Kabuli_NC_021162.1_26627140	Ca_kabuli_chr03	26627140	[T/G]	1067293238
Ca_Kabuli_NC_021162.1_26636607	Ca_kabuli_chr03	26636607	[A/C]	1067293239
Ca_Kabuli_NC_021162.1_26646079	Ca_kabuli_chr03	26646079	[T/C]	1067293240
Ca_Kabuli_NC_021162.1_26646080	Ca_kabuli_chr03	26646080	[T/G]	1067293241
Ca_Kabuli_NC_021162.1_26649150	Ca_kabuli_chr03	26649150	[A/C]	1067293242
Ca_Kabuli_NC_021162.1_26659077	Ca_kabuli_chr03	26659077	[A/C]	1067293243
Ca_Kabuli_NC_021162.1_26659082	Ca_kabuli_chr03	26659082	[T/C]	1067293244
Ca_Kabuli_NC_021162.1_26659093	Ca_kabuli_chr03	26659093	[G/C]	1067293245
Ca_Kabuli_NC_021162.1_26659097	Ca_kabuli_chr03	26659097	[A/C]	1067293246
Ca_Kabuli_NC_021162.1_26659108	Ca_kabuli_chr03	26659108	[T/C]	1067293247
Ca_Kabuli_NC_021162.1_26659111	Ca_kabuli_chr03	26659111	[T/C]	1067293248
Ca_Kabuli_NC_021162.1_26659115	Ca_kabuli_chr03	26659115	[A/G]	1067293249
Ca_Kabuli_NC_021162.1_26726839	Ca_kabuli_chr03	26726839	[T/G]	1067293250
Ca_Kabuli_NC_021162.1_26757751	Ca_kabuli_chr03	26757751	[C/A]	1067293251
Ca_Kabuli_NC_021162.1_26757752	Ca_kabuli_chr03	26757752	[A/G]	1067293252
Ca_Kabuli_NC_021162.1_26849263	Ca_kabuli_chr03	26849263	[A/C]	1067293253
Ca_Kabuli_NC_021162.1_26849303	Ca_kabuli_chr03	26849303	[T/C]	1067293254

SNP IDs	Chromosomes	Physical positions (bp)	SNPs	NCBI SNPdb accession IDs (NCBI_ss#)
Ca_Kabuli_NC_021162.1_26900231	Ca_kabuli_chr03	26900231	[T/C]	1067293255
Ca_Kabuli_NC_021162.1_26900252	Ca_kabuli_chr03	26900252	[G/A]	1067293256
Ca_Kabuli_NC_021162.1_26900271	Ca_kabuli_chr03	26900271	[T/G]	1067293257
Ca_Kabuli_NC_021162.1_26910675	Ca_kabuli_chr03	26910675	[C/A]	1067293258
Ca_Kabuli_NC_021162.1_26911144	Ca_kabuli_chr03	26911144	[T/A]	1067293259
Ca_Kabuli_NC_021162.1_26911172	Ca_kabuli_chr03	26911172	[A/G]	1067293260
Ca_Kabuli_NC_021162.1_26916665	Ca_kabuli_chr03	26916665	[A/C]	1067293261
Ca_Kabuli_NC_021162.1_26942286	Ca_kabuli_chr03	26942286	[A/C]	1067293262
Ca_Kabuli_NC_021162.1_27061234	Ca_kabuli_chr03	27061234	[T/G]	1067293263
Ca_Kabuli_NC_021162.1_27061288	Ca_kabuli_chr03	27061288	[A/G]	1067293264
Ca_Kabuli_NC_021162.1_27061317	Ca_kabuli_chr03	27061317	[A/G]	1067293265
Ca_Kabuli_NC_021162.1_27105602	Ca_kabuli_chr03	27105602	[A/C]	1067293266
Ca_Kabuli_NC_021162.1_27157795	Ca_kabuli_chr03	27157795	[A/T]	1067293267
Ca_Kabuli_NC_021162.1_27194182	Ca_kabuli_chr03	27194182	[T/G]	1067293268
Ca_Kabuli_NC_021162.1_27335334	Ca_kabuli_chr03	27335334	[A/C]	1067293269
Ca_Kabuli_NC_021162.1_27342507	Ca_kabuli_chr03	27342507	[T/A]	1067293270
Ca_Kabuli_NC_021162.1_27342792	Ca_kabuli_chr03	27342792	[C/A]	1067293271
Ca_Kabuli_NC_021162.1_27393412	Ca_kabuli_chr03	27393412	[A/T]	1067293272
Ca_Kabuli_NC_021162.1_27417562	Ca_kabuli_chr03	27417562	[G/A]	1067293273
Ca_Kabuli_NC_021162.1_27420406	Ca_kabuli_chr03	27420406	[A/C]	1067293274
Ca_Kabuli_NC_021162.1_27447278	Ca_kabuli_chr03	27447278	[T/G]	1067293275
Ca_Kabuli_NC_021162.1_27447290	Ca_kabuli_chr03	27447290	[T/G]	1067293276
Ca_Kabuli_NC_021162.1_27447386	Ca_kabuli_chr03	27447386	[A/G]	1067293277
Ca_Kabuli_NC_021162.1_27447418	Ca_kabuli_chr03	27447418	[T/G]	1067293278
Ca_Kabuli_NC_021162.1_27447427	Ca_kabuli_chr03	27447427	[T/C]	1067293279
Ca_Kabuli_NC_021162.1_27447437	Ca_kabuli_chr03	27447437	[T/G]	1067293280
Ca_Kabuli_NC_021162.1_27447448	Ca_kabuli_chr03	27447448	[A/G]	1067293281
Ca_Kabuli_NC_021162.1_27447640	Ca_kabuli_chr03	27447640	[A/G]	1067293282
Ca_Kabuli_NC_021162.1_27450765	Ca_kabuli_chr03	27450765	[A/C]	1067293283
Ca_Kabuli_NC_021162.1_27452679	Ca_kabuli_chr03	27452679	[A/C]	1067293284
Ca_Kabuli_NC_021162.1_27457005	Ca_kabuli_chr03	27457005	[A/C]	1067293285
Ca_Kabuli_NC_021162.1_27470325	Ca_kabuli_chr03	27470325	[T/C]	1067293286
Ca_Kabuli_NC_021162.1_27470503	Ca_kabuli_chr03	27470503	[A/G]	1067293287
Ca_Kabuli_NC_021162.1_27484075	Ca_kabuli_chr03	27484075	[A/G]	1067293288

SNP IDs	Chromosomes	Physical positions (bp)	SNPs	NCBI SNPdb accession IDs (NCBI_ss#)
Ca_Kabuli_NC_021162.1_27484082	Ca_kabuli_chr03	27484082	[T/A]	1067293289
Ca_Kabuli_NC_021162.1_27484086	Ca_kabuli_chr03	27484086	[T/C]	1067293290
Ca_Kabuli_NC_021162.1_27529943	Ca_kabuli_chr03	27529943	[T/G]	1067293291
Ca_Kabuli_NC_021162.1_27529950	Ca_kabuli_chr03	27529950	[T/G]	1067293292
Ca_Kabuli_NC_021162.1_27529992	Ca_kabuli_chr03	27529992	[G/A]	1067293293
Ca_Kabuli_NC_021162.1_27597269	Ca_kabuli_chr03	27597269	[A/T]	1067293294
Ca_Kabuli_NC_021162.1_27597304	Ca_kabuli_chr03	27597304	[A/G]	1067293295
Ca_Kabuli_NC_021162.1_27597411	Ca_kabuli_chr03	27597411	[A/C]	1067293296
Ca_Kabuli_NC_021162.1_27597936	Ca_kabuli_chr03	27597936	[T/G]	1067293297
Ca_Kabuli_NC_021162.1_27597961	Ca_kabuli_chr03	27597961	[G/T]	1067293298
Ca_Kabuli_NC_021162.1_27622568	Ca_kabuli_chr03	27622568	[T/G]	1067293299
Ca_Kabuli_NC_021162.1_27633010	Ca_kabuli_chr03	27633010	[T/A]	1067293300
Ca_Kabuli_NC_021162.1_27633018	Ca_kabuli_chr03	27633018	[A/G]	1067293301
Ca_Kabuli_NC_021162.1_27633033	Ca_kabuli_chr03	27633033	[A/G]	1067293302
Ca_Kabuli_NC_021162.1_27635497	Ca_kabuli_chr03	27635497	[T/G]	1067293303
Ca_Kabuli_NC_021162.1_27654688	Ca_kabuli_chr03	27654688	[G/C]	1067293304
Ca_Kabuli_NC_021162.1_27654689	Ca_kabuli_chr03	27654689	[G/T]	1067293305
Ca_Kabuli_NC_021162.1_27673719	Ca_kabuli_chr03	27673719	[A/G]	1067293306
Ca_Kabuli_NC_021162.1_27738444	Ca_kabuli_chr03	27738444	[A/C]	1067293307
Ca_Kabuli_NC_021162.1_27812554	Ca_kabuli_chr03	27812554	[A/G]	1067293308
Ca_Kabuli_NC_021162.1_27812588	Ca_kabuli_chr03	27812588	[T/G]	1067293309
Ca_Kabuli_NC_021162.1_27824684	Ca_kabuli_chr03	27824684	[A/C]	1067293310
Ca_Kabuli_NC_021162.1_27876459	Ca_kabuli_chr03	27876459	[A/G]	1067293311
Ca_Kabuli_NC_021162.1_27887405	Ca_kabuli_chr03	27887405	[T/C]	1067293312
Ca_Kabuli_NC_021162.1_27906649	Ca_kabuli_chr03	27906649	[A/T]	1067293313
Ca_Kabuli_NC_021162.1_27906656	Ca_kabuli_chr03	27906656	[G/C]	1067293314
Ca_Kabuli_NC_021162.1_27906658	Ca_kabuli_chr03	27906658	[T/G]	1067293315
Ca_Kabuli_NC_021162.1_27906660	Ca_kabuli_chr03	27906660	[A/C]	1067293316
Ca_Kabuli_NC_021162.1_27955085	Ca_kabuli_chr03	27955085	[G/A]	1067293317
Ca_Kabuli_NC_021162.1_27955095	Ca_kabuli_chr03	27955095	[T/C]	1067293318
Ca_Kabuli_NC_021162.1_27955532	Ca_kabuli_chr03	27955532	[A/C]	1067293319
Ca_Kabuli_NC_021162.1_27969031	Ca_kabuli_chr03	27969031	[A/T]	1067293320
Ca_Kabuli_NC_021162.1_28028961	Ca_kabuli_chr03	28028961	[T/G]	1067293321
Ca_Kabuli_NC_021162.1_28080234	Ca_kabuli_chr03	28080234	[T/C]	1067293322

SNP IDs	Chromosomes	Physical positions (bp)	SNPs	NCBI SNPdb accession IDs (NCBI_ss#)
Ca_Kabuli_NC_021162.1_28154628	Ca_kabuli_chr03	28154628	[A/G]	1067293323
Ca_Kabuli_NC_021162.1_28174583	Ca_kabuli_chr03	28174583	[A/T]	1067293324
Ca_Kabuli_NC_021162.1_28176369	Ca_kabuli_chr03	28176369	[T/G]	1067293325
Ca_Kabuli_NC_021162.1_28176370	Ca_kabuli_chr03	28176370	[G/C]	1067293326
Ca_Kabuli_NC_021162.1_28176375	Ca_kabuli_chr03	28176375	[A/C]	1067293327
Ca_Kabuli_NC_021162.1_28176378	Ca_kabuli_chr03	28176378	[T/A]	1067293328
Ca_Kabuli_NC_021162.1_28176380	Ca_kabuli_chr03	28176380	[A/C]	1067293329
Ca_Kabuli_NC_021162.1_28272304	Ca_kabuli_chr03	28272304	[A/G]	1067293330
Ca_Kabuli_NC_021162.1_28288214	Ca_kabuli_chr03	28288214	[A/G]	1067293331
Ca_Kabuli_NC_021162.1_28309399	Ca_kabuli_chr03	28309399	[T/C]	1067293332
Ca_Kabuli_NC_021162.1_28309431	Ca_kabuli_chr03	28309431	[A/G]	1067293333
Ca_Kabuli_NC_021162.1_28408993	Ca_kabuli_chr03	28408993	[T/C]	1067293334
Ca_Kabuli_NC_021162.1_28478128	Ca_kabuli_chr03	28478128	[A/G]	1067293335
Ca_Kabuli_NC_021162.1_28478614	Ca_kabuli_chr03	28478614	[A/C]	1067293336
Ca_Kabuli_NC_021162.1_28596286	Ca_kabuli_chr03	28596286	[T/A]	1067293337
Ca_Kabuli_NC_021162.1_28602055	Ca_kabuli_chr03	28602055	[C/A]	1067293338
Ca_Kabuli_NC_021162.1_28604025	Ca_kabuli_chr03	28604025	[A/C]	1067293339
Ca_Kabuli_NC_021162.1_28613009	Ca_kabuli_chr03	28613009	[C/A]	1067293340
Ca_Kabuli_NC_021162.1_28665366	Ca_kabuli_chr03	28665366	[A/C]	1067293341
Ca_Kabuli_NC_021162.1_28679667	Ca_kabuli_chr03	28679667	[T/G]	1067293342
Ca_Kabuli_NC_021162.1_28706204	Ca_kabuli_chr03	28706204	[A/C]	1067293343
Ca_Kabuli_NC_021162.1_28706216	Ca_kabuli_chr03	28706216	[A/C]	1067293344
Ca_Kabuli_NC_021162.1_28746492	Ca_kabuli_chr03	28746492	[T/C]	1067293345
Ca_Kabuli_NC_021162.1_28746535	Ca_kabuli_chr03	28746535	[A/G]	1067293346
Ca_Kabuli_NC_021162.1_28746547	Ca_kabuli_chr03	28746547	[A/T]	1067293347
Ca_Kabuli_NC_021162.1_28845997	Ca_kabuli_chr03	28845997	[A/G]	1067293348
Ca_Kabuli_NC_021162.1_28926587	Ca_kabuli_chr03	28926587	[T/A]	1067293349
Ca_Kabuli_NC_021162.1_28943991	Ca_kabuli_chr03	28943991	[A/T]	1067293350
Ca_Kabuli_NC_021162.1_28944017	Ca_kabuli_chr03	28944017	[A/C]	1067293351
Ca_Kabuli_NC_021162.1_28963956	Ca_kabuli_chr03	28963956	[T/G]	1067293352
Ca_Kabuli_NC_021162.1_28971120	Ca_kabuli_chr03	28971120	[T/G]	1067293353
Ca_Kabuli_NC_021162.1_28994737	Ca_kabuli_chr03	28994737	[A/C]	1067293354
Ca_Kabuli_NC_021162.1_28999814	Ca_kabuli_chr03	28999814	[A/T]	1067293355
Ca_Kabuli_NC_021162.1_29039804	Ca_kabuli_chr03	29039804	[A/C]	1067293356

SNP IDs	Chromosomes	Physical positions (bp)	SNPs	NCBI SNPdb accession IDs (NCBI_ss#)
Ca_Kabuli_NC_021162.1_29039839	Ca_kabuli_chr03	29039839	[T/A]	1067293357
Ca_Kabuli_NC_021162.1_29039846	Ca_kabuli_chr03	29039846	[T/C]	1067293358
Ca_Kabuli_NC_021162.1_29044203	Ca_kabuli_chr03	29044203	[T/C]	1067293359
Ca_Kabuli_NC_021162.1_29056416	Ca_kabuli_chr03	29056416	[A/C]	1067293360
Ca_Kabuli_NC_021162.1_29107925	Ca_kabuli_chr03	29107925	[A/T]	1067293361
Ca_Kabuli_NC_021162.1_29107926	Ca_kabuli_chr03	29107926	[A/C]	1067293362
Ca_Kabuli_NC_021162.1_29107927	Ca_kabuli_chr03	29107927	[G/T]	1067293363
Ca_Kabuli_NC_021162.1_29108886	Ca_kabuli_chr03	29108886	[A/C]	1067293364
Ca_Kabuli_NC_021162.1_29137907	Ca_kabuli_chr03	29137907	[T/G]	1067293365
Ca_Kabuli_NC_021162.1_29137962	Ca_kabuli_chr03	29137962	[A/G]	1067293366
Ca_Kabuli_NC_021162.1_29155860	Ca_kabuli_chr03	29155860	[A/T]	1067293367
Ca_Kabuli_NC_021162.1_29177819	Ca_kabuli_chr03	29177819	[T/C]	1067293368
Ca_Kabuli_NC_021162.1_29177823	Ca_kabuli_chr03	29177823	[T/C]	1067293369
Ca_Kabuli_NC_021162.1_29207307	Ca_kabuli_chr03	29207307	[A/C]	1067293370
Ca_Kabuli_NC_021162.1_29215229	Ca_kabuli_chr03	29215229	[A/G]	1067293371
Ca_Kabuli_NC_021162.1_29216108	Ca_kabuli_chr03	29216108	[T/A]	1067293372
Ca_Kabuli_NC_021162.1_29216111	Ca_kabuli_chr03	29216111	[G/T]	1067293373
Ca_Kabuli_NC_021162.1_29264741	Ca_kabuli_chr03	29264741	[G/T]	1067293374
Ca_Kabuli_NC_021162.1_29302274	Ca_kabuli_chr03	29302274	[T/G]	1067293375
Ca_Kabuli_NC_021162.1_29302301	Ca_kabuli_chr03	29302301	[A/C]	1067293376
Ca_Kabuli_NC_021162.1_29304122	Ca_kabuli_chr03	29304122	[A/G]	1067293377
Ca_Kabuli_NC_021162.1_29311320	Ca_kabuli_chr03	29311320	[A/C]	1067293378
Ca_Kabuli_NC_021162.1_29326050	Ca_kabuli_chr03	29326050	[T/G]	1067293379
Ca_Kabuli_NC_021162.1_29326060	Ca_kabuli_chr03	29326060	[T/G]	1067293380
Ca_Kabuli_NC_021162.1_29348144	Ca_kabuli_chr03	29348144	[A/C]	1067293381
Ca_Kabuli_NC_021162.1_29359041	Ca_kabuli_chr03	29359041	[A/C]	1067293382
Ca_Kabuli_NC_021162.1_29359088	Ca_kabuli_chr03	29359088	[T/A]	1067293383
Ca_Kabuli_NC_021162.1_29370197	Ca_kabuli_chr03	29370197	[A/C]	1067293384
Ca_Kabuli_NC_021162.1_29370446	Ca_kabuli_chr03	29370446	[A/C]	1067293385
Ca_Kabuli_NC_021162.1_29371503	Ca_kabuli_chr03	29371503	[T/G]	1067293386
Ca_Kabuli_NC_021162.1_29375433	Ca_kabuli_chr03	29375433	[A/C]	1067293387
Ca_Kabuli_NC_021162.1_29375449	Ca_kabuli_chr03	29375449	[T/C]	1067293388
Ca_Kabuli_NC_021162.1_29375995	Ca_kabuli_chr03	29375995	[T/C]	1067293389
Ca_Kabuli_NC_021162.1_29376023	Ca_kabuli_chr03	29376023	[A/T]	1067293390

SNP IDs	Chromosomes	Physical positions (bp)	SNPs	NCBI SNPdb accession IDs (NCBI_ss#)
Ca_Kabuli_NC_021162.1_29376043	Ca_kabuli_chr03	29376043	[T/C]	1067293391
Ca_Kabuli_NC_021162.1_29415845	Ca_kabuli_chr03	29415845	[A/C]	1067293392
Ca_Kabuli_NC_021162.1_29454570	Ca_kabuli_chr03	29454570	[T/G]	1067293393
Ca_Kabuli_NC_021162.1_29455943	Ca_kabuli_chr03	29455943	[T/G]	1067293394
Ca_Kabuli_NC_021162.1_29547542	Ca_kabuli_chr03	29547542	[T/C]	1067293395
Ca_Kabuli_NC_021162.1_29547555	Ca_kabuli_chr03	29547555	[A/G]	1067293396
Ca_Kabuli_NC_021162.1_29547570	Ca_kabuli_chr03	29547570	[A/G]	1067293397
Ca_Kabuli_NC_021162.1_29591562	Ca_kabuli_chr03	29591562	[T/C]	1067293398
Ca_Kabuli_NC_021162.1_29591680	Ca_kabuli_chr03	29591680	[T/C]	1067293399
Ca_Kabuli_NC_021162.1_29591688	Ca_kabuli_chr03	29591688	[T/G]	1067293400
Ca_Kabuli_NC_021162.1_29603771	Ca_kabuli_chr03	29603771	[A/C]	1067293401
Ca_Kabuli_NC_021162.1_29603783	Ca_kabuli_chr03	29603783	[A/T]	1067293402
Ca_Kabuli_NC_021162.1_29608246	Ca_kabuli_chr03	29608246	[T/G]	1067293403
Ca_Kabuli_NC_021162.1_29640019	Ca_kabuli_chr03	29640019	[A/T]	1067293404
Ca_Kabuli_NC_021162.1_29666126	Ca_kabuli_chr03	29666126	[A/G]	1067293405
Ca_Kabuli_NC_021162.1_29666138	Ca_kabuli_chr03	29666138	[C/T]	1067293406
Ca_Kabuli_NC_021162.1_29666633	Ca_kabuli_chr03	29666633	[C/T]	1067293407
Ca_Kabuli_NC_021162.1_29675395	Ca_kabuli_chr03	29675395	[T/C]	1067293408
Ca_Kabuli_NC_021162.1_29675397	Ca_kabuli_chr03	29675397	[A/T]	1067293409
Ca_Kabuli_NC_021162.1_29675414	Ca_kabuli_chr03	29675414	[A/G]	1067293410
Ca_Kabuli_NC_021162.1_29676483	Ca_kabuli_chr03	29676483	[G/T]	1067293411
Ca_Kabuli_NC_021162.1_29676507	Ca_kabuli_chr03	29676507	[A/G]	1067293412
Ca_Kabuli_NC_021162.1_29676564	Ca_kabuli_chr03	29676564	[A/G]	1067293413
Ca_Kabuli_NC_021162.1_29689658	Ca_kabuli_chr03	29689658	[A/G]	1067293414
Ca_Kabuli_NC_021162.1_29689666	Ca_kabuli_chr03	29689666	[T/C]	1067293415
Ca_Kabuli_NC_021162.1_29689668	Ca_kabuli_chr03	29689668	[A/T]	1067293416
Ca_Kabuli_NC_021162.1_29689677	Ca_kabuli_chr03	29689677	[G/T]	1067293417
Ca_Kabuli_NC_021162.1_29698166	Ca_kabuli_chr03	29698166	[G/T]	1067293418
Ca_Kabuli_NC_021162.1_29744834	Ca_kabuli_chr03	29744834	[T/C]	1067293419
Ca_Kabuli_NC_021162.1_29744849	Ca_kabuli_chr03	29744849	[T/G]	1067293420
Ca_Kabuli_NC_021162.1_29744861	Ca_kabuli_chr03	29744861	[A/G]	1067293421
Ca_Kabuli_NC_021162.1_29761540	Ca_kabuli_chr03	29761540	[T/C]	1067293422
Ca_Kabuli_NC_021162.1_29761550	Ca_kabuli_chr03	29761550	[A/C]	1067293423
Ca_Kabuli_NC_021162.1_29761556	Ca_kabuli_chr03	29761556	[A/T]	1067293424

SNP IDs	Chromosomes	Physical positions (bp)	SNPs	NCBI SNPdb accession IDs (NCBI_ss#)
Ca_Kabuli_NC_021162.1_29788933	Ca_kabuli_chr03	29788933	[C/T]	1067293425
Ca_Kabuli_NC_021162.1_29788974	Ca_kabuli_chr03	29788974	[C/T]	1067293426
Ca_Kabuli_NC_021162.1_29803764	Ca_kabuli_chr03	29803764	[A/G]	1067293427
Ca_Kabuli_NC_021162.1_29803779	Ca_kabuli_chr03	29803779	[G/A]	1067293428
Ca_Kabuli_NC_021162.1_29803962	Ca_kabuli_chr03	29803962	[A/G]	1067293429
Ca_Kabuli_NC_021162.1_29803967	Ca_kabuli_chr03	29803967	[T/G]	1067293430
Ca_Kabuli_NC_021162.1_29804030	Ca_kabuli_chr03	29804030	[A/G]	1067293431
Ca_Kabuli_NC_021162.1_29804058	Ca_kabuli_chr03	29804058	[A/T]	1067293432
Ca_Kabuli_NC_021162.1_29804078	Ca_kabuli_chr03	29804078	[A/G]	1067293433
Ca_Kabuli_NC_021162.1_29804107	Ca_kabuli_chr03	29804107	[C/A]	1067293434
Ca_Kabuli_NC_021162.1_29804207	Ca_kabuli_chr03	29804207	[C/G]	1067293435
Ca_Kabuli_NC_021162.1_29804223	Ca_kabuli_chr03	29804223	[A/T]	1067293436
Ca_Kabuli_NC_021162.1_29804225	Ca_kabuli_chr03	29804225	[G/A]	1067293437
Ca_Kabuli_NC_021162.1_29807091	Ca_kabuli_chr03	29807091	[C/G]	1067293438
Ca_Kabuli_NC_021162.1_29807248	Ca_kabuli_chr03	29807248	[A/G]	1067293439
Ca_Kabuli_NC_021162.1_29836313	Ca_kabuli_chr03	29836313	[T/C]	1067293440
Ca_Kabuli_NC_021162.1_29840004	Ca_kabuli_chr03	29840004	[T/C]	1067293441
Ca_Kabuli_NC_021162.1_29840012	Ca_kabuli_chr03	29840012	[A/T]	1067293442
Ca_Kabuli_NC_021162.1_29840021	Ca_kabuli_chr03	29840021	[T/C]	1067293443
Ca_Kabuli_NC_021162.1_29840022	Ca_kabuli_chr03	29840022	[C/A]	1067293444
Ca_Kabuli_NC_021162.1_29840029	Ca_kabuli_chr03	29840029	[C/G]	1067293445
Ca_Kabuli_NC_021162.1_29840046	Ca_kabuli_chr03	29840046	[T/C]	1067293446
Ca_Kabuli_NC_021162.1_29843906	Ca_kabuli_chr03	29843906	[T/C]	1067293447
Ca_Kabuli_NC_021162.1_29843912	Ca_kabuli_chr03	29843912	[T/C]	1067293448
Ca_Kabuli_NC_021162.1_29843922	Ca_kabuli_chr03	29843922	[A/C]	1067293449
Ca_Kabuli_NC_021162.1_29856537	Ca_kabuli_chr03	29856537	[T/C]	1067293450
Ca_Kabuli_NC_021162.1_29858002	Ca_kabuli_chr03	29858002	[A/G]	1067293451
Ca_Kabuli_NC_021162.1_29858046	Ca_kabuli_chr03	29858046	[C/T]	1067293452
Ca_Kabuli_NC_021162.1_29859161	Ca_kabuli_chr03	29859161	[C/A]	1067293453
Ca_Kabuli_NC_021162.1_29869303	Ca_kabuli_chr03	29869303	[T/G]	1067293454
Ca_Kabuli_NC_021162.1_29869380	Ca_kabuli_chr03	29869380	[G/A]	1067293455
Ca_Kabuli_NC_021162.1_29869381	Ca_kabuli_chr03	29869381	[G/A]	1067293456
Ca_Kabuli_NC_021162.1_29869535	Ca_kabuli_chr03	29869535	[C/T]	1067293457
Ca_Kabuli_NC_021162.1_29872672	Ca_kabuli_chr03	29872672	[A/C]	1067293458

SNP IDs	Chromosomes	Physical positions (bp)	SNPs	NCBI SNPdb accession IDs (NCBI_ss#)
Ca_Kabuli_NC_021162.1_29882784	Ca_kabuli_chr03	29882784	[A/G]	1067293459
Ca_Kabuli_NC_021162.1_29908941	Ca_kabuli_chr03	29908941	[C/G]	1067293460
Ca_Kabuli_NC_021162.1_29912456	Ca_kabuli_chr03	29912456	[T/A]	1067293461
Ca_Kabuli_NC_021162.1_29912475	Ca_kabuli_chr03	29912475	[T/G]	1067293462
Ca_Kabuli_NC_021162.1_29913183	Ca_kabuli_chr03	29913183	[C/T]	1067293463
Ca_Kabuli_NC_021162.1_29915542	Ca_kabuli_chr03	29915542	[A/C]	1067293464
Ca_Kabuli_NC_021162.1_29915545	Ca_kabuli_chr03	29915545	[A/C]	1067293465
Ca_Kabuli_NC_021162.1_29932378	Ca_kabuli_chr03	29932378	[C/G]	1067293466
Ca_Kabuli_NC_021162.1_29932502	Ca_kabuli_chr03	29932502	[G/C]	1067293467
Ca_Kabuli_NC_021162.1_29932504	Ca_kabuli_chr03	29932504	[T/A]	1067293468
Ca_Kabuli_NC_021162.1_29954498	Ca_kabuli_chr03	29954498	[G/A]	1067293469
Ca_Kabuli_NC_021162.1_29954612	Ca_kabuli_chr03	29954612	[T/G]	1067293470
Ca_Kabuli_NC_021162.1_29954648	Ca_kabuli_chr03	29954648	[A/T]	1067293471
Ca_Kabuli_NC_021162.1_29958058	Ca_kabuli_chr03	29958058	[T/C]	1067293472
Ca_Kabuli_NC_021162.1_29958817	Ca_kabuli_chr03	29958817	[T/A]	1067293473
Ca_Kabuli_NC_021162.1_29958818	Ca_kabuli_chr03	29958818	[T/G]	1067293474
Ca_Kabuli_NC_021162.1_29958891	Ca_kabuli_chr03	29958891	[G/C]	1067293475
Ca_Kabuli_NC_021162.1_29958977	Ca_kabuli_chr03	29958977	[A/C]	1067293476
Ca_Kabuli_NC_021162.1_29974527	Ca_kabuli_chr03	29974527	[T/C]	1067293477
Ca_Kabuli_NC_021162.1_29974595	Ca_kabuli_chr03	29974595	[C/T]	1067293478
Ca_Kabuli_NC_021162.1_29982638	Ca_kabuli_chr03	29982638	[A/G]	1067293479
Ca_Kabuli_NC_021162.1_30004207	Ca_kabuli_chr03	30004207	[C/T]	1067293480
Ca_Kabuli_NC_021162.1_30004211	Ca_kabuli_chr03	30004211	[C/T]	1067293481
Ca_Kabuli_NC_021162.1_30004213	Ca_kabuli_chr03	30004213	[A/T]	1067293482
Ca_Kabuli_NC_021162.1_30004311	Ca_kabuli_chr03	30004311	[T/G]	1067293483
Ca_Kabuli_NC_021162.1_30009740	Ca_kabuli_chr03	30009740	[A/G]	1067293484
Ca_Kabuli_NC_021162.1_30009749	Ca_kabuli_chr03	30009749	[T/A]	1067293485
Ca_Kabuli_NC_021162.1_30011584	Ca_kabuli_chr03	30011584	[T/C]	1067293486
Ca_Kabuli_NC_021162.1_30011669	Ca_kabuli_chr03	30011669	[A/G]	1067293487
Ca_Kabuli_NC_021162.1_30011759	Ca_kabuli_chr03	30011759	[T/C]	1067293488
Ca_Kabuli_NC_021162.1_30012090	Ca_kabuli_chr03	30012090	[G/A]	1067293489
Ca_Kabuli_NC_021162.1_30012110	Ca_kabuli_chr03	30012110	[T/G]	1067293490
Ca_Kabuli_NC_021162.1_30029868	Ca_kabuli_chr03	30029868	[A/G]	1067293491
Ca_Kabuli_NC_021162.1_30141530	Ca_kabuli_chr03	30141530	[T/C]	1067293492

SNP IDs	Chromosomes	Physical positions (bp)	SNPs	NCBI SNPdb accession IDs (NCBI_ss#)
Ca_Kabuli_NC_021162.1_30141539	Ca_kabuli_chr03	30141539	[T/G]	1067293493
Ca_Kabuli_NC_021162.1_30141557	Ca_kabuli_chr03	30141557	[T/G]	1067293494
Ca_Kabuli_NC_021162.1_30141581	Ca_kabuli_chr03	30141581	[A/C]	1067293495
Ca_Kabuli_NC_021162.1_30142292	Ca_kabuli_chr03	30142292	[T/G]	1067293496
Ca_Kabuli_NC_021162.1_30166602	Ca_kabuli_chr03	30166602	[T/C]	1067293497
Ca_Kabuli_NC_021162.1_30166615	Ca_kabuli_chr03	30166615	[A/G]	1067293498
Ca_Kabuli_NC_021162.1_30166636	Ca_kabuli_chr03	30166636	[T/G]	1067293499
Ca_Kabuli_NC_021162.1_30166640	Ca_kabuli_chr03	30166640	[A/G]	1067293500
Ca_Kabuli_NC_021162.1_30169536	Ca_kabuli_chr03	30169536	[T/C]	1067293501
Ca_Kabuli_NC_021162.1_30169571	Ca_kabuli_chr03	30169571	[A/G]	1067293502
Ca_Kabuli_NC_021162.1_30202456	Ca_kabuli_chr03	30202456	[T/C]	1067293503
Ca_Kabuli_NC_021162.1_30221802	Ca_kabuli_chr03	30221802	[T/C]	1067293504
Ca_Kabuli_NC_021162.1_30221810	Ca_kabuli_chr03	30221810	[A/T]	1067293505
Ca_Kabuli_NC_021162.1_30221826	Ca_kabuli_chr03	30221826	[A/G]	1067293506
Ca_Kabuli_NC_021162.1_30221829	Ca_kabuli_chr03	30221829	[T/C]	1067293507
Ca_Kabuli_NC_021162.1_30267655	Ca_kabuli_chr03	30267655	[A/C]	1067293508
Ca_Kabuli_NC_021162.1_30317360	Ca_kabuli_chr03	30317360	[T/C]	1067293509
Ca_Kabuli_NC_021162.1_30317409	Ca_kabuli_chr03	30317409	[T/G]	1067293510
Ca_Kabuli_NC_021162.1_30317414	Ca_kabuli_chr03	30317414	[T/C]	1067293511
Ca_Kabuli_NC_021162.1_30317438	Ca_kabuli_chr03	30317438	[A/C]	1067293512
Ca_Kabuli_NC_021162.1_30317453	Ca_kabuli_chr03	30317453	[T/C]	1067293513
Ca_Kabuli_NC_021162.1_30321216	Ca_kabuli_chr03	30321216	[T/C]	1067293514
Ca_Kabuli_NC_021162.1_30321271	Ca_kabuli_chr03	30321271	[A/C]	1067293515
Ca_Kabuli_NC_021162.1_30321289	Ca_kabuli_chr03	30321289	[T/C]	1067293516
Ca_Kabuli_NC_021162.1_30321322	Ca_kabuli_chr03	30321322	[T/G]	1067293517
Ca_Kabuli_NC_021162.1_30321326	Ca_kabuli_chr03	30321326	[T/G]	1067293518
Ca_Kabuli_NC_021162.1_30321350	Ca_kabuli_chr03	30321350	[T/C]	1067293519
Ca_Kabuli_NC_021162.1_30321364	Ca_kabuli_chr03	30321364	[A/G]	1067293520
Ca_Kabuli_NC_021162.1_30321368	Ca_kabuli_chr03	30321368	[T/G]	1067293521
Ca_Kabuli_NC_021162.1_30321380	Ca_kabuli_chr03	30321380	[T/C]	1067293522
Ca_Kabuli_NC_021162.1_30358849	Ca_kabuli_chr03	30358849	[T/G]	1067293523
Ca_Kabuli_NC_021162.1_30358873	Ca_kabuli_chr03	30358873	[T/G]	1067293524
Ca_Kabuli_NC_021162.1_30376309	Ca_kabuli_chr03	30376309	[T/C]	1067293525
Ca_Kabuli_NC_021162.1_30383534	Ca_kabuli_chr03	30383534	[T/C]	1067293526

SNP IDs	Chromosomes	Physical positions (bp)	SNPs	NCBI SNPdb accession IDs (NCBI_ss#)
Ca_Kabuli_NC_021162.1_30397271	Ca_kabuli_chr03	30397271	[C/T]	1067293527
Ca_Kabuli_NC_021162.1_30397293	Ca_kabuli_chr03	30397293	[C/T]	1067293528
Ca_Kabuli_NC_021162.1_30397295	Ca_kabuli_chr03	30397295	[A/G]	1067293529
Ca_Kabuli_NC_021162.1_30408222	Ca_kabuli_chr03	30408222	[T/A]	1067293530
Ca_Kabuli_NC_021162.1_30409084	Ca_kabuli_chr03	30409084	[A/G]	1067293531
Ca_Kabuli_NC_021162.1_30433704	Ca_kabuli_chr03	30433704	[A/G]	1067293532
Ca_Kabuli_NC_021162.1_30475732	Ca_kabuli_chr03	30475732	[T/G]	1067293533
Ca_Kabuli_NC_021162.1_30475735	Ca_kabuli_chr03	30475735	[A/G]	1067293534
Ca_Kabuli_NC_021162.1_30475744	Ca_kabuli_chr03	30475744	[T/G]	1067293535
Ca_Kabuli_NC_021162.1_30510344	Ca_kabuli_chr03	30510344	[T/G]	1067293536
Ca_Kabuli_NC_021162.1_30578369	Ca_kabuli_chr03	30578369	[A/C]	1067293537
Ca_Kabuli_NC_021162.1_30591340	Ca_kabuli_chr03	30591340	[A/C]	1067293538
Ca_Kabuli_NC_021162.1_30614369	Ca_kabuli_chr03	30614369	[C/T]	1067293539
Ca_Kabuli_NC_021162.1_30642253	Ca_kabuli_chr03	30642253	[A/G]	1067293540
Ca_Kabuli_NC_021162.1_30672187	Ca_kabuli_chr03	30672187	[T/G]	1067293541
Ca_Kabuli_NC_021162.1_30672192	Ca_kabuli_chr03	30672192	[C/G]	1067293542
Ca_Kabuli_NC_021162.1_30675504	Ca_kabuli_chr03	30675504	[A/C]	1067293543
Ca_Kabuli_NC_021162.1_30756961	Ca_kabuli_chr03	30756961	[A/C]	1067293544
Ca_Kabuli_NC_021162.1_30790251	Ca_kabuli_chr03	30790251	[A/G]	1067293545
Ca_Kabuli_NC_021162.1_30790253	Ca_kabuli_chr03	30790253	[T/G]	1067293546
Ca_Kabuli_NC_021162.1_30790267	Ca_kabuli_chr03	30790267	[T/G]	1067293547
Ca_Kabuli_NC_021162.1_30791688	Ca_kabuli_chr03	30791688	[C/T]	1067293548
Ca_Kabuli_NC_021162.1_30836998	Ca_kabuli_chr03	30836998	[T/G]	1067293549
Ca_Kabuli_NC_021162.1_30837031	Ca_kabuli_chr03	30837031	[G/A]	1067293550
Ca_Kabuli_NC_021162.1_30837130	Ca_kabuli_chr03	30837130	[A/G]	1067293551
Ca_Kabuli_NC_021162.1_30837284	Ca_kabuli_chr03	30837284	[T/C]	1067293552
Ca_Kabuli_NC_021162.1_30932016	Ca_kabuli_chr03	30932016	[T/C]	1067293553
Ca_Kabuli_NC_021162.1_30932019	Ca_kabuli_chr03	30932019	[A/C]	1067293554
Ca_Kabuli_NC_021162.1_31020202	Ca_kabuli_chr03	31020202	[T/C]	1067293555
Ca_Kabuli_NC_021162.1_31020250	Ca_kabuli_chr03	31020250	[A/G]	1067293556
Ca_Kabuli_NC_021162.1_31020278	Ca_kabuli_chr03	31020278	[T/A]	1067293557
Ca_Kabuli_NC_021162.1_31091032	Ca_kabuli_chr03	31091032	[A/C]	1067293558
Ca_Kabuli_NC_021162.1_31091037	Ca_kabuli_chr03	31091037	[A/C]	1067293559
Ca_Kabuli_NC_021162.1_31091054	Ca_kabuli_chr03	31091054	[A/G]	1067293560

SNP IDs	Chromosomes	Physical positions (bp)	SNPs	NCBI SNPdb accession IDs (NCBI_ss#)
Ca_Kabuli_NC_021162.1_31091085	Ca_kabuli_chr03	31091085	[A/C]	1067293561
Ca_Kabuli_NC_021162.1_31091141	Ca_kabuli_chr03	31091141	[A/C]	1067293562
Ca_Kabuli_NC_021162.1_31091171	Ca_kabuli_chr03	31091171	[T/A]	1067293563
Ca_Kabuli_NC_021162.1_31091890	Ca_kabuli_chr03	31091890	[A/G]	1067293564
Ca_Kabuli_NC_021162.1_31091901	Ca_kabuli_chr03	31091901	[T/C]	1067293565
Ca_Kabuli_NC_021162.1_31091908	Ca_kabuli_chr03	31091908	[A/G]	1067293566
Ca_Kabuli_NC_021162.1_31091936	Ca_kabuli_chr03	31091936	[C/G]	1067293567
Ca_Kabuli_NC_021162.1_31091960	Ca_kabuli_chr03	31091960	[T/G]	1067293568
Ca_Kabuli_NC_021162.1_31097470	Ca_kabuli_chr03	31097470	[G/C]	1067293569
Ca_Kabuli_NC_021162.1_31174333	Ca_kabuli_chr03	31174333	[A/G]	1067293570
Ca_Kabuli_NC_021162.1_31174340	Ca_kabuli_chr03	31174340	[A/G]	1067293571
Ca_Kabuli_NC_021162.1_31228545	Ca_kabuli_chr03	31228545	[G/T]	1067293572
Ca_Kabuli_NC_021162.1_31228546	Ca_kabuli_chr03	31228546	[C/T]	1067293573
Ca_Kabuli_NC_021162.1_31228552	Ca_kabuli_chr03	31228552	[C/A]	1067293574
Ca_Kabuli_NC_021162.1_31228555	Ca_kabuli_chr03	31228555	[A/T]	1067293575
Ca_Kabuli_NC_021162.1_31228557	Ca_kabuli_chr03	31228557	[G/T]	1067293576
Ca_Kabuli_NC_021162.1_31228559	Ca_kabuli_chr03	31228559	[T/C]	1067293577
Ca_Kabuli_NC_021162.1_31228561	Ca_kabuli_chr03	31228561	[C/A]	1067293578
Ca_Kabuli_NC_021162.1_31267639	Ca_kabuli_chr03	31267639	[T/A]	1067293579
Ca_Kabuli_NC_021162.1_31268122	Ca_kabuli_chr03	31268122	[T/C]	1067293580
Ca_Kabuli_NC_021162.1_31268127	Ca_kabuli_chr03	31268127	[T/C]	1067293581
Ca_Kabuli_NC_021162.1_31268138	Ca_kabuli_chr03	31268138	[A/G]	1067293582
Ca_Kabuli_NC_021162.1_31279605	Ca_kabuli_chr03	31279605	[A/G]	1067293583
Ca_Kabuli_NC_021162.1_31279606	Ca_kabuli_chr03	31279606	[A/G]	1067293584
Ca_Kabuli_NC_021162.1_31279616	Ca_kabuli_chr03	31279616	[A/T]	1067293585
Ca_Kabuli_NC_021162.1_31288191	Ca_kabuli_chr03	31288191	[T/C]	1067293586
Ca_Kabuli_NC_021162.1_31288200	Ca_kabuli_chr03	31288200	[T/C]	1067293587
Ca_Kabuli_NC_021162.1_31288208	Ca_kabuli_chr03	31288208	[T/G]	1067293588
Ca_Kabuli_NC_021162.1_31301705	Ca_kabuli_chr03	31301705	[G/A]	1067293589
Ca_Kabuli_NC_021162.1_31302060	Ca_kabuli_chr03	31302060	[C/T]	1067293590
Ca_Kabuli_NC_021162.1_31312923	Ca_kabuli_chr03	31312923	[T/G]	1067293591
Ca_Kabuli_NC_021162.1_31313019	Ca_kabuli_chr03	31313019	[T/C]	1067293592
Ca_Kabuli_NC_021162.1_31313409	Ca_kabuli_chr03	31313409	[T/C]	1067293593
Ca_Kabuli_NC_021162.1_31313641	Ca_kabuli_chr03	31313641	[G/T]	1067293594

SNP IDs	Chromosomes	Physical positions (bp)	SNPs	NCBI SNPdb accession IDs (NCBI_ss#)
Ca_Kabuli_NC_021162.1_31317512	Ca_kabuli_chr03	31317512	[A/C]	1067293595
Ca_Kabuli_NC_021162.1_31317521	Ca_kabuli_chr03	31317521	[T/G]	1067293596
Ca_Kabuli_NC_021162.1_31317600	Ca_kabuli_chr03	31317600	[A/G]	1067293597
Ca_Kabuli_NC_021162.1_31321725	Ca_kabuli_chr03	31321725	[G/A]	1067293598
Ca_Kabuli_NC_021162.1_31321877	Ca_kabuli_chr03	31321877	[A/G]	1067293599
Ca_Kabuli_NC_021162.1_31321907	Ca_kabuli_chr03	31321907	[C/T]	1067293600
Ca_Kabuli_NC_021162.1_31322012	Ca_kabuli_chr03	31322012	[A/G]	1067293601
Ca_Kabuli_NC_021162.1_31322014	Ca_kabuli_chr03	31322014	[C/T]	1067293602
Ca_Kabuli_NC_021162.1_31322336	Ca_kabuli_chr03	31322336	[T/C]	1067293603
Ca_Kabuli_NC_021162.1_31322404	Ca_kabuli_chr03	31322404	[C/T]	1067293604
Ca_Kabuli_NC_021162.1_31331685	Ca_kabuli_chr03	31331685	[A/G]	1067293605
Ca_Kabuli_NC_021162.1_31338240	Ca_kabuli_chr03	31338240	[A/C]	1067293606
Ca_Kabuli_NC_021162.1_31341330	Ca_kabuli_chr03	31341330	[A/T]	1067293607
Ca_Kabuli_NC_021162.1_31341359	Ca_kabuli_chr03	31341359	[A/C]	1067293608
Ca_Kabuli_NC_021162.1_31341373	Ca_kabuli_chr03	31341373	[T/C]	1067293609
Ca_Kabuli_NC_021162.1_31342009	Ca_kabuli_chr03	31342009	[A/C]	1067293610
Ca_Kabuli_NC_021162.1_31342024	Ca_kabuli_chr03	31342024	[A/G]	1067293611
Ca_Kabuli_NC_021162.1_31354988	Ca_kabuli_chr03	31354988	[G/C]	1067293612
Ca_Kabuli_NC_021162.1_31355001	Ca_kabuli_chr03	31355001	[C/T]	1067293613
Ca_Kabuli_NC_021162.1_31355026	Ca_kabuli_chr03	31355026	[T/C]	1067293614
Ca_Kabuli_NC_021162.1_31357728	Ca_kabuli_chr03	31357728	[T/G]	1067293615
Ca_Kabuli_NC_021162.1_31357730	Ca_kabuli_chr03	31357730	[G/T]	1067293616
Ca_Kabuli_NC_021162.1_31357732	Ca_kabuli_chr03	31357732	[G/C]	1067293617
Ca_Kabuli_NC_021162.1_31358137	Ca_kabuli_chr03	31358137	[T/G]	1067293618
Ca_Kabuli_NC_021162.1_31361550	Ca_kabuli_chr03	31361550	[A/C]	1067293619
Ca_Kabuli_NC_021162.1_31410134	Ca_kabuli_chr03	31410134	[T/G]	1067293620
Ca_Kabuli_NC_021162.1_31411720	Ca_kabuli_chr03	31411720	[A/G]	1067293621
Ca_Kabuli_NC_021162.1_31411932	Ca_kabuli_chr03	31411932	[T/G]	1067293622
Ca_Kabuli_NC_021162.1_31413589	Ca_kabuli_chr03	31413589	[T/G]	1067293623
Ca_Kabuli_NC_021162.1_31431216	Ca_kabuli_chr03	31431216	[T/C]	1067293624
Ca_Kabuli_NC_021162.1_31437752	Ca_kabuli_chr03	31437752	[A/C]	1067293625
Ca_Kabuli_NC_021162.1_31437784	Ca_kabuli_chr03	31437784	[A/G]	1067293626
Ca_Kabuli_NC_021162.1_31457191	Ca_kabuli_chr03	31457191	[T/C]	1067293627
Ca_Kabuli_NC_021162.1_31478882	Ca_kabuli_chr03	31478882	[T/C]	1067293628

SNP IDs	Chromosomes	Physical positions (bp)	SNPs	NCBI SNPdb accession IDs (NCBI_ss#)
Ca_Kabuli_NC_021162.1_31479308	Ca_kabuli_chr03	31479308	[G/A]	1067293629
Ca_Kabuli_NC_021162.1_31479392	Ca_kabuli_chr03	31479392	[T/C]	1067293630
Ca_Kabuli_NC_021162.1_31487486	Ca_kabuli_chr03	31487486	[G/T]	1067293631
Ca_Kabuli_NC_021162.1_31516662	Ca_kabuli_chr03	31516662	[T/G]	1067293632
Ca_Kabuli_NC_021162.1_31516766	Ca_kabuli_chr03	31516766	[C/T]	1067293633
Ca_Kabuli_NC_021162.1_31517201	Ca_kabuli_chr03	31517201	[G/A]	1067293634
Ca_Kabuli_NC_021162.1_31519880	Ca_kabuli_chr03	31519880	[T/C]	1067293635
Ca_Kabuli_NC_021162.1_31520119	Ca_kabuli_chr03	31520119	[T/C]	1067293636
Ca_Kabuli_NC_021162.1_31520198	Ca_kabuli_chr03	31520198	[A/G]	1067293637
Ca_Kabuli_NC_021162.1_31520225	Ca_kabuli_chr03	31520225	[T/A]	1067293638
Ca_Kabuli_NC_021162.1_31520840	Ca_kabuli_chr03	31520840	[A/G]	1067293639
Ca_Kabuli_NC_021162.1_31520906	Ca_kabuli_chr03	31520906	[T/A]	1067293640
Ca_Kabuli_NC_021162.1_31520907	Ca_kabuli_chr03	31520907	[A/T]	1067293641
Ca_Kabuli_NC_021162.1_31520908	Ca_kabuli_chr03	31520908	[G/T]	1067293642
Ca_Kabuli_NC_021162.1_31521142	Ca_kabuli_chr03	31521142	[C/T]	1067293643
Ca_Kabuli_NC_021162.1_31521155	Ca_kabuli_chr03	31521155	[A/T]	1067293644
Ca_Kabuli_NC_021162.1_31530104	Ca_kabuli_chr03	31530104	[G/A]	1067293645
Ca_Kabuli_NC_021162.1_31537963	Ca_kabuli_chr03	31537963	[A/T]	1067293646
Ca_Kabuli_NC_021162.1_31542359	Ca_kabuli_chr03	31542359	[A/C]	1067293647
Ca_Kabuli_NC_021162.1_31542386	Ca_kabuli_chr03	31542386	[T/G]	1067293648
Ca_Kabuli_NC_021162.1_31542398	Ca_kabuli_chr03	31542398	[T/G]	1067293649
Ca_Kabuli_NC_021162.1_31617723	Ca_kabuli_chr03	31617723	[T/C]	1067293650
Ca_Kabuli_NC_021162.1_31617741	Ca_kabuli_chr03	31617741	[T/C]	1067293651
Ca_Kabuli_NC_021162.1_31617743	Ca_kabuli_chr03	31617743	[T/A]	1067293652
Ca_Kabuli_NC_021162.1_31621021	Ca_kabuli_chr03	31621021	[A/T]	1067293653
Ca_Kabuli_NC_021162.1_31621033	Ca_kabuli_chr03	31621033	[T/G]	1067293654
Ca_Kabuli_NC_021162.1_31625771	Ca_kabuli_chr03	31625771	[T/C]	1067293655
Ca_Kabuli_NC_021162.1_31625792	Ca_kabuli_chr03	31625792	[T/C]	1067293656
Ca_Kabuli_NC_021162.1_31629855	Ca_kabuli_chr03	31629855	[G/T]	1067293657
Ca_Kabuli_NC_021162.1_31630195	Ca_kabuli_chr03	31630195	[C/T]	1067293658
Ca_Kabuli_NC_021162.1_31630512	Ca_kabuli_chr03	31630512	[T/A]	1067293659
Ca_Kabuli_NC_021162.1_31630555	Ca_kabuli_chr03	31630555	[T/G]	1067293660
Ca_Kabuli_NC_021162.1_31631584	Ca_kabuli_chr03	31631584	[A/G]	1067293661
Ca_Kabuli_NC_021162.1_31631622	Ca_kabuli_chr03	31631622	[A/G]	1067293662

SNP IDs	Chromosomes	Physical positions (bp)	SNPs	NCBI SNPdb accession IDs (NCBI_ss#)
Ca_Kabuli_NC_021162.1_31631644	Ca_kabuli_chr03	31631644	[C/A]	1067293663
Ca_Kabuli_NC_021162.1_31646806	Ca_kabuli_chr03	31646806	[G/T]	1067293664
Ca_Kabuli_NC_021162.1_31646807	Ca_kabuli_chr03	31646807	[T/G]	1067293665
Ca_Kabuli_NC_021162.1_31689184	Ca_kabuli_chr03	31689184	[T/C]	1067293666
Ca_Kabuli_NC_021162.1_31698429	Ca_kabuli_chr03	31698429	[C/A]	1067293667
Ca_Kabuli_NC_021162.1_31698432	Ca_kabuli_chr03	31698432	[A/C]	1067293668
Ca_Kabuli_NC_021162.1_31698469	Ca_kabuli_chr03	31698469	[A/G]	1067293669
Ca_Kabuli_NC_021162.1_31729709	Ca_kabuli_chr03	31729709	[A/C]	1067293670
Ca_Kabuli_NC_021162.1_31729713	Ca_kabuli_chr03	31729713	[A/C]	1067293671
Ca_Kabuli_NC_021162.1_31749084	Ca_kabuli_chr03	31749084	[T/C]	1067293672
Ca_Kabuli_NC_021162.1_31749113	Ca_kabuli_chr03	31749113	[A/G]	1067293673
Ca_Kabuli_NC_021162.1_31765232	Ca_kabuli_chr03	31765232	[T/C]	1067293674
Ca_Kabuli_NC_021162.1_31788112	Ca_kabuli_chr03	31788112	[A/G]	1067293675
Ca_Kabuli_NC_021162.1_31790392	Ca_kabuli_chr03	31790392	[T/C]	1067293676
Ca_Kabuli_NC_021162.1_31790393	Ca_kabuli_chr03	31790393	[G/T]	1067293677
Ca_Kabuli_NC_021162.1_31799510	Ca_kabuli_chr03	31799510	[T/G]	1067293678
Ca_Kabuli_NC_021162.1_31799518	Ca_kabuli_chr03	31799518	[G/C]	1067293679
Ca_Kabuli_NC_021162.1_31814796	Ca_kabuli_chr03	31814796	[T/A]	1067293680
Ca_Kabuli_NC_021162.1_31814841	Ca_kabuli_chr03	31814841	[A/G]	1067293681
Ca_Kabuli_NC_021162.1_31817431	Ca_kabuli_chr03	31817431	[G/T]	1067293682
Ca_Kabuli_NC_021162.1_31842500	Ca_kabuli_chr03	31842500	[T/G]	1067293683
Ca_Kabuli_NC_021162.1_31859066	Ca_kabuli_chr03	31859066	[A/C]	1067293684
Ca_Kabuli_NC_021162.1_31864865	Ca_kabuli_chr03	31864865	[A/T]	1067293685
Ca_Kabuli_NC_021162.1_31901267	Ca_kabuli_chr03	31901267	[T/G]	1067293686
Ca_Kabuli_NC_021162.1_31912607	Ca_kabuli_chr03	31912607	[T/A]	1067293687
Ca_Kabuli_NC_021162.1_31930943	Ca_kabuli_chr03	31930943	[T/C]	1067293688
Ca_Kabuli_NC_021162.1_31931009	Ca_kabuli_chr03	31931009	[T/G]	1067293689
Ca_Kabuli_NC_021162.1_31951942	Ca_kabuli_chr03	31951942	[T/G]	1067293690
Ca_Kabuli_NC_021162.1_31951994	Ca_kabuli_chr03	31951994	[A/G]	1067293691
Ca_Kabuli_NC_021162.1_31952316	Ca_kabuli_chr03	31952316	[G/T]	1067293692
Ca_Kabuli_NC_021162.1_31952326	Ca_kabuli_chr03	31952326	[C/A]	1067293693
Ca_Kabuli_NC_021162.1_31952330	Ca_kabuli_chr03	31952330	[T/C]	1067293694
Ca_Kabuli_NC_021162.1_31952333	Ca_kabuli_chr03	31952333	[T/G]	1067293695
Ca_Kabuli_NC_021162.1_31952337	Ca_kabuli_chr03	31952337	[C/T]	1067293696

SNP IDs	Chromosomes	Physical positions (bp)	SNPs	NCBI SNPdb accession IDs (NCBI_ss#)
Ca_Kabuli_NC_021162.1_31978674	Ca_kabuli_chr03	31978674	[A/C]	1067293697
Ca_Kabuli_NC_021162.1_31978693	Ca_kabuli_chr03	31978693	[A/G]	1067293698
Ca_Kabuli_NC_021162.1_32378057	Ca_kabuli_chr03	32378057	[C/A]	1067293699
Ca_Kabuli_NC_021162.1_32441088	Ca_kabuli_chr03	32441088	[T/G]	1067293700
Ca_Kabuli_NC_021162.1_32441092	Ca_kabuli_chr03	32441092	[T/C]	1067293701
Ca_Kabuli_NC_021162.1_32448969	Ca_kabuli_chr03	32448969	[T/G]	1067293702
Ca_Kabuli_NC_021162.1_32452041	Ca_kabuli_chr03	32452041	[A/C]	1067293703
Ca_Kabuli_NC_021162.1_32473005	Ca_kabuli_chr03	32473005	[A/G]	1067293704
Ca_Kabuli_NC_021162.1_32473007	Ca_kabuli_chr03	32473007	[T/C]	1067293705
Ca_Kabuli_NC_021162.1_32473062	Ca_kabuli_chr03	32473062	[T/A]	1067293706
Ca_Kabuli_NC_021162.1_32535281	Ca_kabuli_chr03	32535281	[A/G]	1067293707
Ca_Kabuli_NC_021162.1_32593710	Ca_kabuli_chr03	32593710	[A/G]	1067293708
Ca_Kabuli_NC_021162.1_32593718	Ca_kabuli_chr03	32593718	[A/G]	1067293709
Ca_Kabuli_NC_021162.1_32593731	Ca_kabuli_chr03	32593731	[A/G]	1067293710
Ca_Kabuli_NC_021162.1_32593736	Ca_kabuli_chr03	32593736	[T/C]	1067293711
Ca_Kabuli_NC_021162.1_32639819	Ca_kabuli_chr03	32639819	[T/A]	1067293712
Ca_Kabuli_NC_021162.1_32663061	Ca_kabuli_chr03	32663061	[A/G]	1067293713
Ca_Kabuli_NC_021162.1_32694474	Ca_kabuli_chr03	32694474	[A/C]	1067293714
Ca_Kabuli_NC_021162.1_32706136	Ca_kabuli_chr03	32706136	[A/G]	1067293715
Ca_Kabuli_NC_021162.1_32706248	Ca_kabuli_chr03	32706248	[T/C]	1067293716
Ca_Kabuli_NC_021162.1_32706377	Ca_kabuli_chr03	32706377	[T/C]	1067293717
Ca_Kabuli_NC_021162.1_32706412	Ca_kabuli_chr03	32706412	[A/C]	1067293718
Ca_Kabuli_NC_021162.1_32734708	Ca_kabuli_chr03	32734708	[A/C]	1067293719
Ca_Kabuli_NC_021162.1_32734718	Ca_kabuli_chr03	32734718	[A/C]	1067293720
Ca_Kabuli_NC_021162.1_32734724	Ca_kabuli_chr03	32734724	[T/C]	1067293721
Ca_Kabuli_NC_021162.1_32735238	Ca_kabuli_chr03	32735238	[A/T]	1067293722
Ca_Kabuli_NC_021162.1_32735258	Ca_kabuli_chr03	32735258	[T/C]	1067293723
Ca_Kabuli_NC_021162.1_32735266	Ca_kabuli_chr03	32735266	[A/T]	1067293724
Ca_Kabuli_NC_021162.1_32735291	Ca_kabuli_chr03	32735291	[T/A]	1067293725
Ca_Kabuli_NC_021162.1_32759265	Ca_kabuli_chr03	32759265	[T/C]	1067293726
Ca_Kabuli_NC_021162.1_32759278	Ca_kabuli_chr03	32759278	[T/G]	1067293727
Ca_Kabuli_NC_021162.1_32759292	Ca_kabuli_chr03	32759292	[A/C]	1067293728
Ca_Kabuli_NC_021162.1_32799371	Ca_kabuli_chr03	32799371	[T/A]	1067293729
Ca_Kabuli_NC_021162.1_32826988	Ca_kabuli_chr03	32826988	[T/C]	1067293730

SNP IDs	Chromosomes	Physical positions (bp)	SNPs	NCBI SNPdb accession IDs (NCBI_ss#)
Ca_Kabuli_NC_021162.1_32827079	Ca_kabuli_chr03	32827079	[T/C]	1067293731
Ca_Kabuli_NC_021162.1_32840343	Ca_kabuli_chr03	32840343	[T/C]	1067293732
Ca_Kabuli_NC_021162.1_32840345	Ca_kabuli_chr03	32840345	[A/G]	1067293733
Ca_Kabuli_NC_021162.1_32840378	Ca_kabuli_chr03	32840378	[A/G]	1067293734
Ca_Kabuli_NC_021162.1_32840387	Ca_kabuli_chr03	32840387	[A/G]	1067293735
Ca_Kabuli_NC_021162.1_32845301	Ca_kabuli_chr03	32845301	[T/G]	1067293736
Ca_Kabuli_NC_021162.1_32845448	Ca_kabuli_chr03	32845448	[A/C]	1067293737
Ca_Kabuli_NC_021162.1_32880614	Ca_kabuli_chr03	32880614	[T/C]	1067293738
Ca_Kabuli_NC_021162.1_32880623	Ca_kabuli_chr03	32880623	[T/C]	1067293739
Ca_Kabuli_NC_021162.1_32880658	Ca_kabuli_chr03	32880658	[A/G]	1067293740
Ca_Kabuli_NC_021162.1_33056013	Ca_kabuli_chr03	33056013	[T/G]	1067293741
Ca_Kabuli_NC_021162.1_33056288	Ca_kabuli_chr03	33056288	[A/T]	1067293742
Ca_Kabuli_NC_021162.1_33078629	Ca_kabuli_chr03	33078629	[T/G]	1067293743
Ca_Kabuli_NC_021162.1_33081726	Ca_kabuli_chr03	33081726	[C/A]	1067293744
Ca_Kabuli_NC_021162.1_33120876	Ca_kabuli_chr03	33120876	[T/C]	1067293745
Ca_Kabuli_NC_021162.1_33171927	Ca_kabuli_chr03	33171927	[A/G]	1067293746
Ca_Kabuli_NC_021162.1_33173947	Ca_kabuli_chr03	33173947	[A/C]	1067293747
Ca_Kabuli_NC_021162.1_33173959	Ca_kabuli_chr03	33173959	[A/T]	1067293748
Ca_Kabuli_NC_021162.1_33173997	Ca_kabuli_chr03	33173997	[T/C]	1067293749
Ca_Kabuli_NC_021162.1_33174023	Ca_kabuli_chr03	33174023	[A/C]	1067293750
Ca_Kabuli_NC_021162.1_33242163	Ca_kabuli_chr03	33242163	[T/A]	1067293751
Ca_Kabuli_NC_021162.1_33242164	Ca_kabuli_chr03	33242164	[G/T]	1067293752
Ca_Kabuli_NC_021162.1_33513756	Ca_kabuli_chr03	33513756	[T/C]	1067293753
Ca_Kabuli_NC_021162.1_33520864	Ca_kabuli_chr03	33520864	[A/T]	1067293754
Ca_Kabuli_NC_021162.1_33520890	Ca_kabuli_chr03	33520890	[A/T]	1067293755
Ca_Kabuli_NC_021162.1_33522970	Ca_kabuli_chr03	33522970	[A/G]	1067293756
Ca_Kabuli_NC_021162.1_33522999	Ca_kabuli_chr03	33522999	[A/G]	1067293757
Ca_Kabuli_NC_021162.1_33523006	Ca_kabuli_chr03	33523006	[A/T]	1067293758
Ca_Kabuli_NC_021162.1_33523032	Ca_kabuli_chr03	33523032	[T/A]	1067293759
Ca_Kabuli_NC_021162.1_33526209	Ca_kabuli_chr03	33526209	[A/G]	1067293760
Ca_Kabuli_NC_021162.1_33627826	Ca_kabuli_chr03	33627826	[A/C]	1067293761
Ca_Kabuli_NC_021162.1_33715215	Ca_kabuli_chr03	33715215	[T/G]	1067293762
Ca_Kabuli_NC_021162.1_33898279	Ca_kabuli_chr03	33898279	[C/G]	1067293763
Ca_Kabuli_NC_021162.1_33898282	Ca_kabuli_chr03	33898282	[C/T]	1067293764

SNP IDs	Chromosomes	Physical positions (bp)	SNPs	NCBI SNPdb accession IDs (NCBI_ss#)
Ca_Kabuli_NC_021162.1_33898283	Ca_kabuli_chr03	33898283	[A/G]	1067293765
Ca_Kabuli_NC_021162.1_33898285	Ca_kabuli_chr03	33898285	[A/G]	1067293766
Ca_Kabuli_NC_021162.1_33900522	Ca_kabuli_chr03	33900522	[A/C]	1067293767
Ca_Kabuli_NC_021162.1_33904394	Ca_kabuli_chr03	33904394	[T/A]	1067293768
Ca_Kabuli_NC_021162.1_34206048	Ca_kabuli_chr03	34206048	[C/A]	1067293769
Ca_Kabuli_NC_021162.1_34387300	Ca_kabuli_chr03	34387300	[A/G]	1067293770
Ca_Kabuli_NC_021162.1_34387310	Ca_kabuli_chr03	34387310	[T/A]	1067293771
Ca_Kabuli_NC_021162.1_34387322	Ca_kabuli_chr03	34387322	[T/G]	1067293772
Ca_Kabuli_NC_021162.1_34434203	Ca_kabuli_chr03	34434203	[A/C]	1067293773
Ca_Kabuli_NC_021162.1_34496893	Ca_kabuli_chr03	34496893	[A/G]	1067293774
Ca_Kabuli_NC_021162.1_34496910	Ca_kabuli_chr03	34496910	[T/C]	1067293775
Ca_Kabuli_NC_021162.1_34496927	Ca_kabuli_chr03	34496927	[A/G]	1067293776
Ca_Kabuli_NC_021162.1_34496933	Ca_kabuli_chr03	34496933	[A/C]	1067293777
Ca_Kabuli_NC_021162.1_34496941	Ca_kabuli_chr03	34496941	[T/C]	1067293778
Ca_Kabuli_NC_021162.1_34496945	Ca_kabuli_chr03	34496945	[A/C]	1067293779
Ca_Kabuli_NC_021162.1_34576560	Ca_kabuli_chr03	34576560	[A/C]	1067293780
Ca_Kabuli_NC_021162.1_34576588	Ca_kabuli_chr03	34576588	[A/G]	1067293781
Ca_Kabuli_NC_021162.1_34603704	Ca_kabuli_chr03	34603704	[A/C]	1067293782
Ca_Kabuli_NC_021162.1_34603709	Ca_kabuli_chr03	34603709	[A/C]	1067293783
Ca_Kabuli_NC_021162.1_34603724	Ca_kabuli_chr03	34603724	[A/T]	1067293784
Ca_Kabuli_NC_021162.1_34603975	Ca_kabuli_chr03	34603975	[A/C]	1067293785
Ca_Kabuli_NC_021162.1_34650253	Ca_kabuli_chr03	34650253	[T/C]	1067293786
Ca_Kabuli_NC_021162.1_34699109	Ca_kabuli_chr03	34699109	[T/G]	1067293787
Ca_Kabuli_NC_021162.1_34699114	Ca_kabuli_chr03	34699114	[C/A]	1067293788
Ca_Kabuli_NC_021162.1_34722866	Ca_kabuli_chr03	34722866	[G/A]	1067293789
Ca_Kabuli_NC_021162.1_34722868	Ca_kabuli_chr03	34722868	[T/G]	1067293790
Ca_Kabuli_NC_021162.1_34722869	Ca_kabuli_chr03	34722869	[G/A]	1067293791
Ca_Kabuli_NC_021162.1_34722876	Ca_kabuli_chr03	34722876	[T/G]	1067293792
Ca_Kabuli_NC_021162.1_34722893	Ca_kabuli_chr03	34722893	[T/G]	1067293793
Ca_Kabuli_NC_021162.1_34722942	Ca_kabuli_chr03	34722942	[T/G]	1067293794
Ca_Kabuli_NC_021162.1_34722960	Ca_kabuli_chr03	34722960	[T/A]	1067293795
Ca_Kabuli_NC_021162.1_34860356	Ca_kabuli_chr03	34860356	[A/G]	1067293796
Ca_Kabuli_NC_021162.1_34896470	Ca_kabuli_chr03	34896470	[T/G]	1067293797
Ca_Kabuli_NC_021162.1_34911630	Ca_kabuli_chr03	34911630	[A/C]	1067293798

SNP IDs	Chromosomes	Physical positions (bp)	SNPs	NCBI SNPdb accession IDs (NCBI_ss#)
Ca_Kabuli_NC_021162.1_34982544	Ca_kabuli_chr03	34982544	[C/T]	1067293799
Ca_Kabuli_NC_021162.1_35029896	Ca_kabuli_chr03	35029896	[A/G]	1067293800
Ca_Kabuli_NC_021162.1_35029902	Ca_kabuli_chr03	35029902	[T/C]	1067293801
Ca_Kabuli_NC_021162.1_35029903	Ca_kabuli_chr03	35029903	[A/T]	1067293802
Ca_Kabuli_NC_021162.1_35029931	Ca_kabuli_chr03	35029931	[A/T]	1067293803
Ca_Kabuli_NC_021162.1_35061822	Ca_kabuli_chr03	35061822	[A/G]	1067293804
Ca_Kabuli_NC_021162.1_35061823	Ca_kabuli_chr03	35061823	[A/C]	1067293805
Ca_Kabuli_NC_021162.1_35061827	Ca_kabuli_chr03	35061827	[G/T]	1067293806
Ca_Kabuli_NC_021162.1_35061829	Ca_kabuli_chr03	35061829	[T/G]	1067293807
Ca_Kabuli_NC_021162.1_35071244	Ca_kabuli_chr03	35071244	[T/C]	1067293808
Ca_Kabuli_NC_021162.1_35071852	Ca_kabuli_chr03	35071852	[T/G]	1067293809
Ca_Kabuli_NC_021162.1_35082215	Ca_kabuli_chr03	35082215	[A/C]	1067293810
Ca_Kabuli_NC_021162.1_35082253	Ca_kabuli_chr03	35082253	[T/C]	1067293811
Ca_Kabuli_NC_021162.1_35084142	Ca_kabuli_chr03	35084142	[A/G]	1067293812
Ca_Kabuli_NC_021162.1_35085576	Ca_kabuli_chr03	35085576	[T/A]	1067293813
Ca_Kabuli_NC_021162.1_35085580	Ca_kabuli_chr03	35085580	[A/G]	1067293814
Ca_Kabuli_NC_021162.1_35085618	Ca_kabuli_chr03	35085618	[T/C]	1067293815
Ca_Kabuli_NC_021162.1_35085622	Ca_kabuli_chr03	35085622	[A/G]	1067293816
Ca_Kabuli_NC_021162.1_35085659	Ca_kabuli_chr03	35085659	[A/C]	1067293817
Ca_Kabuli_NC_021162.1_35184964	Ca_kabuli_chr03	35184964	[T/C]	1067293818
Ca_Kabuli_NC_021162.1_35184973	Ca_kabuli_chr03	35184973	[A/C]	1067293819
Ca_Kabuli_NC_021162.1_35195902	Ca_kabuli_chr03	35195902	[A/G]	1067293820
Ca_Kabuli_NC_021162.1_35298513	Ca_kabuli_chr03	35298513	[C/A]	1067293821
Ca_Kabuli_NC_021162.1_35298514	Ca_kabuli_chr03	35298514	[A/G]	1067293822
Ca_Kabuli_NC_021162.1_35298515	Ca_kabuli_chr03	35298515	[T/A]	1067293823
Ca_Kabuli_NC_021162.1_35332171	Ca_kabuli_chr03	35332171	[A/C]	1067293824
Ca_Kabuli_NC_021162.1_35354403	Ca_kabuli_chr03	35354403	[A/T]	1067293825
Ca_Kabuli_NC_021162.1_35354446	Ca_kabuli_chr03	35354446	[T/C]	1067293826
Ca_Kabuli_NC_021162.1_35384915	Ca_kabuli_chr03	35384915	[T/C]	1067293827
Ca_Kabuli_NC_021162.1_35414268	Ca_kabuli_chr03	35414268	[T/A]	1067293828
Ca_Kabuli_NC_021162.1_35414306	Ca_kabuli_chr03	35414306	[T/G]	1067293829
Ca_Kabuli_NC_021162.1_35424830	Ca_kabuli_chr03	35424830	[T/C]	1067293830
Ca_Kabuli_NC_021162.1_35424833	Ca_kabuli_chr03	35424833	[T/C]	1067293831
Ca_Kabuli_NC_021162.1_35424855	Ca_kabuli_chr03	35424855	[T/A]	1067293832

SNP IDs	Chromosomes	Physical positions (bp)	SNPs	NCBI SNPdb accession IDs (NCBI_ss#)
Ca_Kabuli_NC_021162.1_35424896	Ca_kabuli_chr03	35424896	[T/A]	1067293833
Ca_Kabuli_NC_021162.1_35425042	Ca_kabuli_chr03	35425042	[G/T]	1067293834
Ca_Kabuli_NC_021162.1_35425085	Ca_kabuli_chr03	35425085	[G/C]	1067293835
Ca_Kabuli_NC_021162.1_35425110	Ca_kabuli_chr03	35425110	[T/C]	1067293836
Ca_Kabuli_NC_021162.1_35425159	Ca_kabuli_chr03	35425159	[A/G]	1067293837
Ca_Kabuli_NC_021162.1_35464889	Ca_kabuli_chr03	35464889	[T/G]	1067293838
Ca_Kabuli_NC_021162.1_35465375	Ca_kabuli_chr03	35465375	[G/C]	1067293839
Ca_Kabuli_NC_021162.1_35465376	Ca_kabuli_chr03	35465376	[T/C]	1067293840
Ca_Kabuli_NC_021162.1_35465380	Ca_kabuli_chr03	35465380	[G/T]	1067293841
Ca_Kabuli_NC_021162.1_35478610	Ca_kabuli_chr03	35478610	[G/T]	1067293842
Ca_Kabuli_NC_021162.1_35484047	Ca_kabuli_chr03	35484047	[A/T]	1067293843
Ca_Kabuli_NC_021162.1_35490904	Ca_kabuli_chr03	35490904	[T/C]	1067293844
Ca_Kabuli_NC_021162.1_35631684	Ca_kabuli_chr03	35631684	[T/G]	1067293845
Ca_Kabuli_NC_021162.1_35631690	Ca_kabuli_chr03	35631690	[T/C]	1067293846
Ca_Kabuli_NC_021162.1_35631718	Ca_kabuli_chr03	35631718	[T/G]	1067293847
Ca_Kabuli_NC_021162.1_35676141	Ca_kabuli_chr03	35676141	[T/C]	1067293848
Ca_Kabuli_NC_021162.1_35676164	Ca_kabuli_chr03	35676164	[T/G]	1067293849
Ca_Kabuli_NC_021162.1_35807358	Ca_kabuli_chr03	35807358	[A/C]	1067293850
Ca_Kabuli_NC_021162.1_35807390	Ca_kabuli_chr03	35807390	[A/C]	1067293851
Ca_Kabuli_NC_021162.1_35826134	Ca_kabuli_chr03	35826134	[A/G]	1067293852
Ca_Kabuli_NC_021162.1_35829777	Ca_kabuli_chr03	35829777	[G/C]	1067293853
Ca_Kabuli_NC_021162.1_35853464	Ca_kabuli_chr03	35853464	[A/C]	1067293854
Ca_Kabuli_NC_021162.1_35853514	Ca_kabuli_chr03	35853514	[T/A]	1067293855
Ca_Kabuli_NC_021162.1_35853624	Ca_kabuli_chr03	35853624	[T/C]	1067293856
Ca_Kabuli_NC_021162.1_35853657	Ca_kabuli_chr03	35853657	[T/G]	1067293857
Ca_Kabuli_NC_021162.1_35897932	Ca_kabuli_chr03	35897932	[T/C]	1067293858
Ca_Kabuli_NC_021162.1_35898227	Ca_kabuli_chr03	35898227	[A/C]	1067293859
Ca_Kabuli_NC_021162.1_35898233	Ca_kabuli_chr03	35898233	[A/C]	1067293860
Ca_Kabuli_NC_021162.1_35920747	Ca_kabuli_chr03	35920747	[T/C]	1067293861
Ca_Kabuli_NC_021162.1_35920780	Ca_kabuli_chr03	35920780	[T/G]	1067293862
Ca_Kabuli_NC_021162.1_35927676	Ca_kabuli_chr03	35927676	[T/G]	1067293863
Ca_Kabuli_NC_021162.1_35979412	Ca_kabuli_chr03	35979412	[G/T]	1067293864
Ca_Kabuli_NC_021162.1_35979490	Ca_kabuli_chr03	35979490	[T/C]	1067293865
Ca_Kabuli_NC_021162.1_36003699	Ca_kabuli_chr03	36003699	[A/G]	1067293866

SNP IDs	Chromosomes	Physical positions (bp)	SNPs	NCBI SNPdb accession IDs (NCBI_ss#)
Ca_Kabuli_NC_021162.1_36004668	Ca_kabuli_chr03	36004668	[G/A]	1067293867
Ca_Kabuli_NC_021162.1_36009983	Ca_kabuli_chr03	36009983	[G/C]	1067293868
Ca_Kabuli_NC_021162.1_36034934	Ca_kabuli_chr03	36034934	[C/A]	1067293869
Ca_Kabuli_NC_021162.1_36036333	Ca_kabuli_chr03	36036333	[G/C]	1067293870
Ca_Kabuli_NC_021162.1_36036341	Ca_kabuli_chr03	36036341	[T/A]	1067293871
Ca_Kabuli_NC_021162.1_36036353	Ca_kabuli_chr03	36036353	[G/A]	1067293872
Ca_Kabuli_NC_021162.1_36039133	Ca_kabuli_chr03	36039133	[T/C]	1067293873
Ca_Kabuli_NC_021162.1_36046845	Ca_kabuli_chr03	36046845	[A/C]	1067293874
Ca_Kabuli_NC_021162.1_36046937	Ca_kabuli_chr03	36046937	[A/C]	1067293875
Ca_Kabuli_NC_021162.1_36046938	Ca_kabuli_chr03	36046938	[C/G]	1067293876
Ca_Kabuli_NC_021162.1_36046951	Ca_kabuli_chr03	36046951	[C/T]	1067293877
Ca_Kabuli_NC_021162.1_36046961	Ca_kabuli_chr03	36046961	[A/C]	1067293878
Ca_Kabuli_NC_021162.1_36046966	Ca_kabuli_chr03	36046966	[C/T]	1067293879
Ca_Kabuli_NC_021162.1_36050259	Ca_kabuli_chr03	36050259	[G/T]	1067293880
Ca_Kabuli_NC_021162.1_36050319	Ca_kabuli_chr03	36050319	[C/T]	1067293881
Ca_Kabuli_NC_021162.1_36060899	Ca_kabuli_chr03	36060899	[T/G]	1067293882
Ca_Kabuli_NC_021162.1_36060930	Ca_kabuli_chr03	36060930	[A/C]	1067293883
Ca_Kabuli_NC_021162.1_36066511	Ca_kabuli_chr03	36066511	[T/G]	1067293884
Ca_Kabuli_NC_021162.1_36066583	Ca_kabuli_chr03	36066583	[C/T]	1067293885
Ca_Kabuli_NC_021162.1_36070879	Ca_kabuli_chr03	36070879	[C/G]	1067293886
Ca_Kabuli_NC_021162.1_36070972	Ca_kabuli_chr03	36070972	[A/G]	1067293887
Ca_Kabuli_NC_021162.1_36071033	Ca_kabuli_chr03	36071033	[G/A]	1067293888
Ca_Kabuli_NC_021162.1_36072770	Ca_kabuli_chr03	36072770	[A/G]	1067293889
Ca_Kabuli_NC_021162.1_36085966	Ca_kabuli_chr03	36085966	[A/G]	1067293890
Ca_Kabuli_NC_021162.1_36086013	Ca_kabuli_chr03	36086013	[A/C]	1067293891
Ca_Kabuli_NC_021162.1_36086028	Ca_kabuli_chr03	36086028	[G/T]	1067293892
Ca_Kabuli_NC_021162.1_36118120	Ca_kabuli_chr03	36118120	[C/T]	1067293893
Ca_Kabuli_NC_021162.1_36132391	Ca_kabuli_chr03	36132391	[A/G]	1067293894
Ca_Kabuli_NC_021162.1_36132432	Ca_kabuli_chr03	36132432	[A/G]	1067293895
Ca_Kabuli_NC_021162.1_36132445	Ca_kabuli_chr03	36132445	[T/G]	1067293896
Ca_Kabuli_NC_021162.1_36132586	Ca_kabuli_chr03	36132586	[G/T]	1067293897
Ca_Kabuli_NC_021162.1_36150722	Ca_kabuli_chr03	36150722	[A/G]	1067293898
Ca_Kabuli_NC_021162.1_36178343	Ca_kabuli_chr03	36178343	[A/G]	1067293899
Ca_Kabuli_NC_021162.1_36194086	Ca_kabuli_chr03	36194086	[G/C]	1067293900

SNP IDs	Chromosomes	Physical positions (bp)	SNPs	NCBI SNPdb accession IDs (NCBI_ss#)
Ca_Kabuli_NC_021162.1_36205806	Ca_kabuli_chr03	36205806	[G/T]	1067293901
Ca_Kabuli_NC_021162.1_36205819	Ca_kabuli_chr03	36205819	[G/A]	1067293902
Ca_Kabuli_NC_021162.1_36205845	Ca_kabuli_chr03	36205845	[G/A]	1067293903
Ca_Kabuli_NC_021162.1_36206037	Ca_kabuli_chr03	36206037	[A/G]	1067293904
Ca_Kabuli_NC_021162.1_36206046	Ca_kabuli_chr03	36206046	[T/C]	1067293905
Ca_Kabuli_NC_021162.1_36206085	Ca_kabuli_chr03	36206085	[T/C]	1067293906
Ca_Kabuli_NC_021162.1_36206138	Ca_kabuli_chr03	36206138	[A/G]	1067293907
Ca_Kabuli_NC_021162.1_36206615	Ca_kabuli_chr03	36206615	[C/A]	1067293908
Ca_Kabuli_NC_021162.1_36212843	Ca_kabuli_chr03	36212843	[T/A]	1067293909
Ca_Kabuli_NC_021162.1_36227565	Ca_kabuli_chr03	36227565	[T/A]	1067293910
Ca_Kabuli_NC_021162.1_36227566	Ca_kabuli_chr03	36227566	[T/A]	1067293911
Ca_Kabuli_NC_021162.1_36228032	Ca_kabuli_chr03	36228032	[G/T]	1067293912
Ca_Kabuli_NC_021162.1_36228034	Ca_kabuli_chr03	36228034	[T/G]	1067293913
Ca_Kabuli_NC_021162.1_36228121	Ca_kabuli_chr03	36228121	[T/A]	1067293914
Ca_Kabuli_NC_021162.1_36228142	Ca_kabuli_chr03	36228142	[A/G]	1067293915
Ca_Kabuli_NC_021162.1_36242492	Ca_kabuli_chr03	36242492	[A/G]	1067293916
Ca_Kabuli_NC_021162.1_36242501	Ca_kabuli_chr03	36242501	[A/G]	1067293917
Ca_Kabuli_NC_021162.1_36242504	Ca_kabuli_chr03	36242504	[C/T]	1067293918
Ca_Kabuli_NC_021162.1_36242544	Ca_kabuli_chr03	36242544	[T/A]	1067293919
Ca_Kabuli_NC_021162.1_36247792	Ca_kabuli_chr03	36247792	[T/C]	1067293920
Ca_Kabuli_NC_021162.1_36260856	Ca_kabuli_chr03	36260856	[A/G]	1067293921
Ca_Kabuli_NC_021162.1_36260890	Ca_kabuli_chr03	36260890	[A/G]	1067293922
Ca_Kabuli_NC_021162.1_36282085	Ca_kabuli_chr03	36282085	[T/A]	1067293923
Ca_Kabuli_NC_021162.1_36282088	Ca_kabuli_chr03	36282088	[T/G]	1067293924
Ca_Kabuli_NC_021162.1_36282091	Ca_kabuli_chr03	36282091	[T/G]	1067293925
Ca_Kabuli_NC_021162.1_36282094	Ca_kabuli_chr03	36282094	[T/G]	1067293926
Ca_Kabuli_NC_021162.1_36282105	Ca_kabuli_chr03	36282105	[A/T]	1067293927
Ca_Kabuli_NC_021162.1_36282123	Ca_kabuli_chr03	36282123	[A/G]	1067293928
Ca_Kabuli_NC_021162.1_36293697	Ca_kabuli_chr03	36293697	[T/C]	1067293929
Ca_Kabuli_NC_021162.1_36330750	Ca_kabuli_chr03	36330750	[A/G]	1067293930
Ca_Kabuli_NC_021162.1_36330815	Ca_kabuli_chr03	36330815	[T/C]	1067293931
Ca_Kabuli_NC_021162.1_36334653	Ca_kabuli_chr03	36334653	[A/G]	1067293932
Ca_Kabuli_NC_021162.1_36334699	Ca_kabuli_chr03	36334699	[T/G]	1067293933
Ca_Kabuli_NC_021162.1_36361339	Ca_kabuli_chr03	36361339	[C/T]	1067293934

SNP IDs	Chromosomes	Physical positions (bp)	SNPs	NCBI SNPdb accession IDs (NCBI_ss#)
Ca_Kabuli_NC_021162.1_36378294	Ca_kabuli_chr03	36378294	[G/T]	1067293935
Ca_Kabuli_NC_021162.1_36380423	Ca_kabuli_chr03	36380423	[G/A]	1067293936
Ca_Kabuli_NC_021162.1_36387718	Ca_kabuli_chr03	36387718	[T/C]	1067293937
Ca_Kabuli_NC_021162.1_36407587	Ca_kabuli_chr03	36407587	[G/A]	1067293938
Ca_Kabuli_NC_021162.1_36417921	Ca_kabuli_chr03	36417921	[G/A]	1067293939
Ca_Kabuli_NC_021162.1_36417942	Ca_kabuli_chr03	36417942	[T/C]	1067293940
Ca_Kabuli_NC_021162.1_36418025	Ca_kabuli_chr03	36418025	[A/G]	1067293941
Ca_Kabuli_NC_021162.1_36432389	Ca_kabuli_chr03	36432389	[G/A]	1067293942
Ca_Kabuli_NC_021162.1_36441575	Ca_kabuli_chr03	36441575	[A/G]	1067293943
Ca_Kabuli_NC_021162.1_36441593	Ca_kabuli_chr03	36441593	[T/A]	1067293944
Ca_Kabuli_NC_021162.1_36441594	Ca_kabuli_chr03	36441594	[G/A]	1067293945
Ca_Kabuli_NC_021162.1_36441595	Ca_kabuli_chr03	36441595	[T/C]	1067293946
Ca_Kabuli_NC_021162.1_36447525	Ca_kabuli_chr03	36447525	[C/T]	1067293947
Ca_Kabuli_NC_021162.1_36463010	Ca_kabuli_chr03	36463010	[A/T]	1067293948
Ca_Kabuli_NC_021162.1_36563350	Ca_kabuli_chr03	36563350	[C/T]	1067293949
Ca_Kabuli_NC_021162.1_36568324	Ca_kabuli_chr03	36568324	[A/C]	1067293950
Ca_Kabuli_NC_021162.1_36573324	Ca_kabuli_chr03	36573324	[T/C]	1067293951
Ca_Kabuli_NC_021162.1_36573411	Ca_kabuli_chr03	36573411	[A/C]	1067293952
Ca_Kabuli_NC_021162.1_36584095	Ca_kabuli_chr03	36584095	[A/G]	1067293953
Ca_Kabuli_NC_021162.1_36584127	Ca_kabuli_chr03	36584127	[T/G]	1067293954
Ca_Kabuli_NC_021162.1_36588765	Ca_kabuli_chr03	36588765	[A/T]	1067293955
Ca_Kabuli_NC_021162.1_36588788	Ca_kabuli_chr03	36588788	[A/G]	1067293956
Ca_Kabuli_NC_021162.1_36588914	Ca_kabuli_chr03	36588914	[A/C]	1067293957
Ca_Kabuli_NC_021162.1_36609256	Ca_kabuli_chr03	36609256	[T/G]	1067293958
Ca_Kabuli_NC_021162.1_36610509	Ca_kabuli_chr03	36610509	[A/G]	1067293959
Ca_Kabuli_NC_021162.1_36634188	Ca_kabuli_chr03	36634188	[T/G]	1067293960
Ca_Kabuli_NC_021162.1_36644077	Ca_kabuli_chr03	36644077	[A/G]	1067293961
Ca_Kabuli_NC_021162.1_36644129	Ca_kabuli_chr03	36644129	[A/G]	1067293962
Ca_Kabuli_NC_021162.1_36658776	Ca_kabuli_chr03	36658776	[A/T]	1067293963
Ca_Kabuli_NC_021162.1_36749838	Ca_kabuli_chr03	36749838	[A/C]	1067293964
Ca_Kabuli_NC_021162.1_36749851	Ca_kabuli_chr03	36749851	[A/G]	1067293965
Ca_Kabuli_NC_021162.1_36749942	Ca_kabuli_chr03	36749942	[T/C]	1067293966
Ca_Kabuli_NC_021162.1_36749980	Ca_kabuli_chr03	36749980	[A/G]	1067293967
Ca_Kabuli_NC_021162.1_36752677	Ca_kabuli_chr03	36752677	[A/C]	1067293968

SNP IDs	Chromosomes	Physical positions (bp)	SNPs	NCBI SNPdb accession IDs (NCBI_ss#)
Ca_Kabuli_NC_021162.1_36752691	Ca_kabuli_chr03	36752691	[A/T]	1067293969
Ca_Kabuli_NC_021162.1_36752752	Ca_kabuli_chr03	36752752	[T/C]	1067293970
Ca_Kabuli_NC_021162.1_36752763	Ca_kabuli_chr03	36752763	[A/G]	1067293971
Ca_Kabuli_NC_021162.1_36752765	Ca_kabuli_chr03	36752765	[T/C]	1067293972
Ca_Kabuli_NC_021162.1_36752778	Ca_kabuli_chr03	36752778	[A/G]	1067293973
Ca_Kabuli_NC_021162.1_36752828	Ca_kabuli_chr03	36752828	[A/G]	1067293974
Ca_Kabuli_NC_021162.1_36754270	Ca_kabuli_chr03	36754270	[T/G]	1067293975
Ca_Kabuli_NC_021162.1_36754366	Ca_kabuli_chr03	36754366	[A/C]	1067293976
Ca_Kabuli_NC_021162.1_36754369	Ca_kabuli_chr03	36754369	[A/C]	1067293977
Ca_Kabuli_NC_021162.1_36816705	Ca_kabuli_chr03	36816705	[T/C]	1067293978
Ca_Kabuli_NC_021162.1_36816725	Ca_kabuli_chr03	36816725	[T/C]	1067293979
Ca_Kabuli_NC_021162.1_36816752	Ca_kabuli_chr03	36816752	[A/C]	1067293980
Ca_Kabuli_NC_021162.1_36825508	Ca_kabuli_chr03	36825508	[T/C]	1067293981
Ca_Kabuli_NC_021162.1_36860990	Ca_kabuli_chr03	36860990	[A/C]	1067293982
Ca_Kabuli_NC_021162.1_36871004	Ca_kabuli_chr03	36871004	[T/A]	1067293983
Ca_Kabuli_NC_021162.1_36871092	Ca_kabuli_chr03	36871092	[T/C]	1067293984
Ca_Kabuli_NC_021162.1_36871094	Ca_kabuli_chr03	36871094	[A/G]	1067293985
Ca_Kabuli_NC_021162.1_36871099	Ca_kabuli_chr03	36871099	[A/T]	1067293986
Ca_Kabuli_NC_021162.1_36871115	Ca_kabuli_chr03	36871115	[T/G]	1067293987
Ca_Kabuli_NC_021162.1_36871128	Ca_kabuli_chr03	36871128	[T/C]	1067293988
Ca_Kabuli_NC_021162.1_36872737	Ca_kabuli_chr03	36872737	[A/C]	1067293989
Ca_Kabuli_NC_021162.1_36872747	Ca_kabuli_chr03	36872747	[A/C]	1067293990
Ca_Kabuli_NC_021162.1_36883124	Ca_kabuli_chr03	36883124	[T/C]	1067293991
Ca_Kabuli_NC_021162.1_36883176	Ca_kabuli_chr03	36883176	[A/C]	1067293992
Ca_Kabuli_NC_021162.1_36935486	Ca_kabuli_chr03	36935486	[T/A]	1067293993
Ca_Kabuli_NC_021162.1_36935487	Ca_kabuli_chr03	36935487	[A/T]	1067293994
Ca_Kabuli_NC_021162.1_36935488	Ca_kabuli_chr03	36935488	[G/C]	1067293995
Ca_Kabuli_NC_021162.1_36935489	Ca_kabuli_chr03	36935489	[G/T]	1067293996
Ca_Kabuli_NC_021162.1_36967289	Ca_kabuli_chr03	36967289	[A/C]	1067293997
Ca_Kabuli_NC_021162.1_37155895	Ca_kabuli_chr03	37155895	[A/G]	1067293998
Ca_Kabuli_NC_021162.1_37155901	Ca_kabuli_chr03	37155901	[T/C]	1067293999
Ca_Kabuli_NC_021162.1_37155955	Ca_kabuli_chr03	37155955	[T/A]	1067294000
Ca_Kabuli_NC_021162.1_37160795	Ca_kabuli_chr03	37160795	[A/C]	1067294001
Ca_Kabuli_NC_021162.1_37160804	Ca_kabuli_chr03	37160804	[A/G]	1067294002

SNP IDs	Chromosomes	Physical positions (bp)	SNPs	NCBI SNPdb accession IDs (NCBI_ss#)
Ca_Kabuli_NC_021162.1_37160944	Ca_kabuli_chr03	37160944	[A/G]	1067294003
Ca_Kabuli_NC_021162.1_37178482	Ca_kabuli_chr03	37178482	[A/T]	1067294004
Ca_Kabuli_NC_021162.1_37178516	Ca_kabuli_chr03	37178516	[T/C]	1067294005
Ca_Kabuli_NC_021162.1_37267774	Ca_kabuli_chr03	37267774	[A/T]	1067294006
Ca_Kabuli_NC_021162.1_37267875	Ca_kabuli_chr03	37267875	[C/A]	1067294007
Ca_Kabuli_NC_021162.1_37267898	Ca_kabuli_chr03	37267898	[T/G]	1067294008
Ca_Kabuli_NC_021162.1_37269067	Ca_kabuli_chr03	37269067	[C/A]	1067294009
Ca_Kabuli_NC_021162.1_37297922	Ca_kabuli_chr03	37297922	[A/G]	1067294010
Ca_Kabuli_NC_021162.1_37303602	Ca_kabuli_chr03	37303602	[T/A]	1067294011
Ca_Kabuli_NC_021162.1_37309436	Ca_kabuli_chr03	37309436	[A/G]	1067294012
Ca_Kabuli_NC_021162.1_37314823	Ca_kabuli_chr03	37314823	[A/G]	1067294013
Ca_Kabuli_NC_021162.1_37314832	Ca_kabuli_chr03	37314832	[T/A]	1067294014
Ca_Kabuli_NC_021162.1_37316815	Ca_kabuli_chr03	37316815	[C/G]	1067294015
Ca_Kabuli_NC_021162.1_37316816	Ca_kabuli_chr03	37316816	[T/C]	1067294016
Ca_Kabuli_NC_021162.1_37316817	Ca_kabuli_chr03	37316817	[G/T]	1067294017
Ca_Kabuli_NC_021162.1_37316818	Ca_kabuli_chr03	37316818	[T/G]	1067294018
Ca_Kabuli_NC_021162.1_37322763	Ca_kabuli_chr03	37322763	[A/C]	1067294019
Ca_Kabuli_NC_021162.1_37322808	Ca_kabuli_chr03	37322808	[T/C]	1067294020
Ca_Kabuli_NC_021162.1_37378323	Ca_kabuli_chr03	37378323	[A/C]	1067294021
Ca_Kabuli_NC_021162.1_37436493	Ca_kabuli_chr03	37436493	[A/G]	1067294022
Ca_Kabuli_NC_021162.1_37438110	Ca_kabuli_chr03	37438110	[T/C]	1067294023
Ca_Kabuli_NC_021162.1_37438148	Ca_kabuli_chr03	37438148	[G/A]	1067294024
Ca_Kabuli_NC_021162.1_37439576	Ca_kabuli_chr03	37439576	[T/C]	1067294025
Ca_Kabuli_NC_021162.1_37472189	Ca_kabuli_chr03	37472189	[T/C]	1067294026
Ca_Kabuli_NC_021162.1_37472240	Ca_kabuli_chr03	37472240	[A/G]	1067294027
Ca_Kabuli_NC_021162.1_37499211	Ca_kabuli_chr03	37499211	[A/G]	1067294028
Ca_Kabuli_NC_021162.1_37499253	Ca_kabuli_chr03	37499253	[A/C]	1067294029
Ca_Kabuli_NC_021162.1_37499259	Ca_kabuli_chr03	37499259	[A/G]	1067294030
Ca_Kabuli_NC_021162.1_37608935	Ca_kabuli_chr03	37608935	[C/A]	1067294031
Ca_Kabuli_NC_021162.1_37608936	Ca_kabuli_chr03	37608936	[T/G]	1067294032
Ca_Kabuli_NC_021162.1_37608937	Ca_kabuli_chr03	37608937	[T/A]	1067294033
Ca_Kabuli_NC_021162.1_37637640	Ca_kabuli_chr03	37637640	[A/T]	1067294034
Ca_Kabuli_NC_021162.1_37637641	Ca_kabuli_chr03	37637641	[A/T]	1067294035
Ca_Kabuli_NC_021162.1_37637642	Ca_kabuli_chr03	37637642	[A/T]	1067294036

SNP IDs	Chromosomes	Physical positions (bp)	SNPs	NCBI SNPdb accession IDs (NCBI_ss#)
Ca_Kabuli_NC_021162.1_37759195	Ca_kabuli_chr03	37759195	[T/A]	1067294037
Ca_Kabuli_NC_021162.1_37759428	Ca_kabuli_chr03	37759428	[G/A]	1067294038
Ca_Kabuli_NC_021162.1_37765723	Ca_kabuli_chr03	37765723	[T/C]	1067294039
Ca_Kabuli_NC_021162.1_37765738	Ca_kabuli_chr03	37765738	[C/T]	1067294040
Ca_Kabuli_NC_021162.1_37765765	Ca_kabuli_chr03	37765765	[T/G]	1067294041
Ca_Kabuli_NC_021162.1_37765767	Ca_kabuli_chr03	37765767	[A/G]	1067294042
Ca_Kabuli_NC_021162.1_37806633	Ca_kabuli_chr03	37806633	[T/C]	1067294043
Ca_Kabuli_NC_021162.1_37806647	Ca_kabuli_chr03	37806647	[C/A]	1067294044
Ca_Kabuli_NC_021162.1_37817266	Ca_kabuli_chr03	37817266	[A/C]	1067294045
Ca_Kabuli_NC_021162.1_37822882	Ca_kabuli_chr03	37822882	[A/T]	1067294046
Ca_Kabuli_NC_021162.1_37866125	Ca_kabuli_chr03	37866125	[A/G]	1067294047
Ca_Kabuli_NC_021162.1_37866133	Ca_kabuli_chr03	37866133	[A/G]	1067294048
Ca_Kabuli_NC_021162.1_37942807	Ca_kabuli_chr03	37942807	[A/G]	1067294049
Ca_Kabuli_NC_021162.1_37951112	Ca_kabuli_chr03	37951112	[C/T]	1067294050
Ca_Kabuli_NC_021162.1_37985776	Ca_kabuli_chr03	37985776	[T/A]	1067294051
Ca_Kabuli_NC_021162.1_37985947	Ca_kabuli_chr03	37985947	[A/T]	1067294052
Ca_Kabuli_NC_021162.1_37985977	Ca_kabuli_chr03	37985977	[A/G]	1067294053
Ca_Kabuli_NC_021162.1_37986028	Ca_kabuli_chr03	37986028	[G/A]	1067294054
Ca_Kabuli_NC_021162.1_38015973	Ca_kabuli_chr03	38015973	[C/G]	1067294055
Ca_Kabuli_NC_021162.1_38016234	Ca_kabuli_chr03	38016234	[T/C]	1067294056
Ca_Kabuli_NC_021162.1_38026725	Ca_kabuli_chr03	38026725	[G/A]	1067294057
Ca_Kabuli_NC_021162.1_38029092	Ca_kabuli_chr03	38029092	[T/C]	1067294058
Ca_Kabuli_NC_021162.1_38029101	Ca_kabuli_chr03	38029101	[A/C]	1067294059
Ca_Kabuli_NC_021162.1_38029131	Ca_kabuli_chr03	38029131	[A/T]	1067294060
Ca_Kabuli_NC_021162.1_38034006	Ca_kabuli_chr03	38034006	[G/C]	1067294061
Ca_Kabuli_NC_021162.1_38046362	Ca_kabuli_chr03	38046362	[A/G]	1067294062
Ca_Kabuli_NC_021162.1_38046522	Ca_kabuli_chr03	38046522	[A/G]	1067294063
Ca_Kabuli_NC_021162.1_38049996	Ca_kabuli_chr03	38049996	[A/G]	1067294064
Ca_Kabuli_NC_021162.1_38054694	Ca_kabuli_chr03	38054694	[G/C]	1067294065
Ca_Kabuli_NC_021162.1_38055488	Ca_kabuli_chr03	38055488	[T/C]	1067294066
Ca_Kabuli_NC_021162.1_38059303	Ca_kabuli_chr03	38059303	[T/C]	1067294067
Ca_Kabuli_NC_021162.1_38060814	Ca_kabuli_chr03	38060814	[A/G]	1067294068
Ca_Kabuli_NC_021162.1_38074259	Ca_kabuli_chr03	38074259	[C/A]	1067294069
Ca_Kabuli_NC_021162.1_38076428	Ca_kabuli_chr03	38076428	[T/C]	1067294070

SNP IDs	Chromosomes	Physical positions (bp)	SNPs	NCBI SNPdb accession IDs (NCBI_ss#)
Ca_Kabuli_NC_021162.1_38076432	Ca_kabuli_chr03	38076432	[A/G]	1067294071
Ca_Kabuli_NC_021162.1_38076439	Ca_kabuli_chr03	38076439	[A/G]	1067294072
Ca_Kabuli_NC_021162.1_38076449	Ca_kabuli_chr03	38076449	[T/C]	1067294073
Ca_Kabuli_NC_021162.1_38076458	Ca_kabuli_chr03	38076458	[A/G]	1067294074
Ca_Kabuli_NC_021162.1_38088503	Ca_kabuli_chr03	38088503	[A/C]	1067294075
Ca_Kabuli_NC_021162.1_38103366	Ca_kabuli_chr03	38103366	[T/G]	1067294076
Ca_Kabuli_NC_021162.1_38103367	Ca_kabuli_chr03	38103367	[T/G]	1067294077
Ca_Kabuli_NC_021162.1_38103368	Ca_kabuli_chr03	38103368	[T/A]	1067294078
Ca_Kabuli_NC_021162.1_38103369	Ca_kabuli_chr03	38103369	[T/G]	1067294079
Ca_Kabuli_NC_021162.1_38103375	Ca_kabuli_chr03	38103375	[T/C]	1067294080
Ca_Kabuli_NC_021162.1_38103524	Ca_kabuli_chr03	38103524	[C/T]	1067294081
Ca_Kabuli_NC_021162.1_38112801	Ca_kabuli_chr03	38112801	[C/T]	1067294082
Ca_Kabuli_NC_021162.1_38112802	Ca_kabuli_chr03	38112802	[T/C]	1067294083
Ca_Kabuli_NC_021162.1_38112971	Ca_kabuli_chr03	38112971	[A/G]	1067294084
Ca_Kabuli_NC_021162.1_38115564	Ca_kabuli_chr03	38115564	[A/T]	1067294085
Ca_Kabuli_NC_021162.1_38117169	Ca_kabuli_chr03	38117169	[C/T]	1067294086
Ca_Kabuli_NC_021162.1_38117584	Ca_kabuli_chr03	38117584	[G/A]	1067294087
Ca_Kabuli_NC_021162.1_38117585	Ca_kabuli_chr03	38117585	[A/C]	1067294088
Ca_Kabuli_NC_021162.1_38119199	Ca_kabuli_chr03	38119199	[T/C]	1067294089
Ca_Kabuli_NC_021162.1_38119204	Ca_kabuli_chr03	38119204	[T/G]	1067294090
Ca_Kabuli_NC_021162.1_38119236	Ca_kabuli_chr03	38119236	[T/G]	1067294091
Ca_Kabuli_NC_021162.1_38127341	Ca_kabuli_chr03	38127341	[C/T]	1067294092
Ca_Kabuli_NC_021162.1_38130410	Ca_kabuli_chr03	38130410	[A/G]	1067294093
Ca_Kabuli_NC_021162.1_38145115	Ca_kabuli_chr03	38145115	[T/C]	1067294094
Ca_Kabuli_NC_021162.1_38147699	Ca_kabuli_chr03	38147699	[T/C]	1067294095
Ca_Kabuli_NC_021162.1_38166877	Ca_kabuli_chr03	38166877	[A/C]	1067294096
Ca_Kabuli_NC_021162.1_38166921	Ca_kabuli_chr03	38166921	[A/G]	1067294097
Ca_Kabuli_NC_021162.1_38167980	Ca_kabuli_chr03	38167980	[T/A]	1067294098
Ca_Kabuli_NC_021162.1_38169101	Ca_kabuli_chr03	38169101	[A/G]	1067294099
Ca_Kabuli_NC_021162.1_38169102	Ca_kabuli_chr03	38169102	[T/G]	1067294100
Ca_Kabuli_NC_021162.1_38171434	Ca_kabuli_chr03	38171434	[A/G]	1067294101
Ca_Kabuli_NC_021162.1_38171484	Ca_kabuli_chr03	38171484	[T/C]	1067294102
Ca_Kabuli_NC_021162.1_38171485	Ca_kabuli_chr03	38171485	[A/C]	1067294103
Ca_Kabuli_NC_021162.1_38171486	Ca_kabuli_chr03	38171486	[G/A]	1067294104

SNP IDs	Chromosomes	Physical positions (bp)	SNPs	NCBI SNPdb accession IDs (NCBI_ss#)
Ca_Kabuli_NC_021162.1_38171987	Ca_kabuli_chr03	38171987	[C/A]	1067294105
Ca_Kabuli_NC_021162.1_38200748	Ca_kabuli_chr03	38200748	[T/G]	1067294106
Ca_Kabuli_NC_021162.1_38202223	Ca_kabuli_chr03	38202223	[T/C]	1067294107
Ca_Kabuli_NC_021162.1_38205470	Ca_kabuli_chr03	38205470	[A/G]	1067294108
Ca_Kabuli_NC_021162.1_38205481	Ca_kabuli_chr03	38205481	[A/T]	1067294109
Ca_Kabuli_NC_021162.1_38205491	Ca_kabuli_chr03	38205491	[C/A]	1067294110
Ca_Kabuli_NC_021162.1_38205503	Ca_kabuli_chr03	38205503	[G/C]	1067294111
Ca_Kabuli_NC_021162.1_38211280	Ca_kabuli_chr03	38211280	[T/C]	1067294112
Ca_Kabuli_NC_021162.1_38216263	Ca_kabuli_chr03	38216263	[A/T]	1067294113
Ca_Kabuli_NC_021162.1_38220857	Ca_kabuli_chr03	38220857	[T/C]	1067294114
Ca_Kabuli_NC_021162.1_38220882	Ca_kabuli_chr03	38220882	[T/G]	1067294115
Ca_Kabuli_NC_021162.1_38220946	Ca_kabuli_chr03	38220946	[T/G]	1067294116
Ca_Kabuli_NC_021162.1_38220958	Ca_kabuli_chr03	38220958	[A/G]	1067294117
Ca_Kabuli_NC_021162.1_38220982	Ca_kabuli_chr03	38220982	[T/G]	1067294118
Ca_Kabuli_NC_021162.1_38222733	Ca_kabuli_chr03	38222733	[C/G]	1067294119
Ca_Kabuli_NC_021162.1_38222747	Ca_kabuli_chr03	38222747	[G/A]	1067294120
Ca_Kabuli_NC_021162.1_38237628	Ca_kabuli_chr03	38237628	[A/G]	1067294121
Ca_Kabuli_NC_021162.1_38290472	Ca_kabuli_chr03	38290472	[T/C]	1067294122
Ca_Kabuli_NC_021162.1_38294310	Ca_kabuli_chr03	38294310	[A/G]	1067294123
Ca_Kabuli_NC_021162.1_38315599	Ca_kabuli_chr03	38315599	[A/C]	1067294124
Ca_Kabuli_NC_021162.1_38379763	Ca_kabuli_chr03	38379763	[A/C]	1067294125
Ca_Kabuli_NC_021162.1_38401004	Ca_kabuli_chr03	38401004	[A/G]	1067294126
Ca_Kabuli_NC_021162.1_38423974	Ca_kabuli_chr03	38423974	[T/C]	1067294127
Ca_Kabuli_NC_021162.1_38423983	Ca_kabuli_chr03	38423983	[A/C]	1067294128
Ca_Kabuli_NC_021162.1_38424010	Ca_kabuli_chr03	38424010	[T/C]	1067294129
Ca_Kabuli_NC_021162.1_38449908	Ca_kabuli_chr03	38449908	[A/G]	1067294130
Ca_Kabuli_NC_021162.1_38452070	Ca_kabuli_chr03	38452070	[A/C]	1067294131
Ca_Kabuli_NC_021162.1_38460864	Ca_kabuli_chr03	38460864	[T/C]	1067294132
Ca_Kabuli_NC_021162.1_38465347	Ca_kabuli_chr03	38465347	[A/T]	1067294133
Ca_Kabuli_NC_021162.1_38496218	Ca_kabuli_chr03	38496218	[A/G]	1067294134
Ca_Kabuli_NC_021162.1_38496232	Ca_kabuli_chr03	38496232	[T/G]	1067294135
Ca_Kabuli_NC_021162.1_38496235	Ca_kabuli_chr03	38496235	[T/A]	1067294136
Ca_Kabuli_NC_021162.1_38508707	Ca_kabuli_chr03	38508707	[T/C]	1067294137
Ca_Kabuli_NC_021162.1_38508723	Ca_kabuli_chr03	38508723	[A/C]	1067294138

SNP IDs	Chromosomes	Physical positions (bp)	SNPs	NCBI SNPdb accession IDs (NCBI_ss#)
Ca_Kabuli_NC_021162.1_38511390	Ca_kabuli_chr03	38511390	[T/C]	1067294139
Ca_Kabuli_NC_021162.1_38511392	Ca_kabuli_chr03	38511392	[A/G]	1067294140
Ca_Kabuli_NC_021162.1_38539721	Ca_kabuli_chr03	38539721	[T/G]	1067294141
Ca_Kabuli_NC_021162.1_38572158	Ca_kabuli_chr03	38572158	[T/C]	1067294142
Ca_Kabuli_NC_021162.1_38592068	Ca_kabuli_chr03	38592068	[T/G]	1067294143
Ca_Kabuli_NC_021162.1_38592071	Ca_kabuli_chr03	38592071	[A/G]	1067294144
Ca_Kabuli_NC_021162.1_38604320	Ca_kabuli_chr03	38604320	[T/C]	1067294145
Ca_Kabuli_NC_021162.1_38624786	Ca_kabuli_chr03	38624786	[C/T]	1067294146
Ca_Kabuli_NC_021162.1_38624799	Ca_kabuli_chr03	38624799	[T/G]	1067294147
Ca_Kabuli_NC_021162.1_38624802	Ca_kabuli_chr03	38624802	[G/T]	1067294148
Ca_Kabuli_NC_021162.1_38624805	Ca_kabuli_chr03	38624805	[T/A]	1067294149
Ca_Kabuli_NC_021162.1_38629643	Ca_kabuli_chr03	38629643	[G/A]	1067294150
Ca_Kabuli_NC_021162.1_38702928	Ca_kabuli_chr03	38702928	[A/T]	1067294151
Ca_Kabuli_NC_021162.1_38731433	Ca_kabuli_chr03	38731433	[T/C]	1067294152
Ca_Kabuli_NC_021162.1_38764360	Ca_kabuli_chr03	38764360	[A/C]	1067294153
Ca_Kabuli_NC_021162.1_38764380	Ca_kabuli_chr03	38764380	[T/C]	1067294154
Ca_Kabuli_NC_021162.1_38764384	Ca_kabuli_chr03	38764384	[T/C]	1067294155
Ca_Kabuli_NC_021162.1_38766264	Ca_kabuli_chr03	38766264	[A/G]	1067294156
Ca_Kabuli_NC_021162.1_38766265	Ca_kabuli_chr03	38766265	[G/A]	1067294157
Ca_Kabuli_NC_021162.1_38772170	Ca_kabuli_chr03	38772170	[A/C]	1067294158
Ca_Kabuli_NC_021162.1_38772184	Ca_kabuli_chr03	38772184	[A/G]	1067294159
Ca_Kabuli_NC_021162.1_38772193	Ca_kabuli_chr03	38772193	[T/C]	1067294160
Ca_Kabuli_NC_021162.1_38772196	Ca_kabuli_chr03	38772196	[T/C]	1067294161
Ca_Kabuli_NC_021162.1_38772204	Ca_kabuli_chr03	38772204	[T/G]	1067294162
Ca_Kabuli_NC_021162.1_38785851	Ca_kabuli_chr03	38785851	[T/G]	1067294163
Ca_Kabuli_NC_021162.1_38790727	Ca_kabuli_chr03	38790727	[T/C]	1067294164
Ca_Kabuli_NC_021162.1_38790740	Ca_kabuli_chr03	38790740	[A/G]	1067294165
Ca_Kabuli_NC_021162.1_38793194	Ca_kabuli_chr03	38793194	[T/C]	1067294166
Ca_Kabuli_NC_021162.1_38809117	Ca_kabuli_chr03	38809117	[T/G]	1067294167
Ca_Kabuli_NC_021162.1_38809122	Ca_kabuli_chr03	38809122	[T/G]	1067294168
Ca_Kabuli_NC_021162.1_38818172	Ca_kabuli_chr03	38818172	[T/G]	1067294169
Ca_Kabuli_NC_021162.1_38818290	Ca_kabuli_chr03	38818290	[A/C]	1067294170
Ca_Kabuli_NC_021162.1_38818305	Ca_kabuli_chr03	38818305	[T/G]	1067294171
Ca_Kabuli_NC_021162.1_38822052	Ca_kabuli_chr03	38822052	[T/A]	1067294172

SNP IDs	Chromosomes	Physical positions (bp)	SNPs	NCBI SNPdb accession IDs (NCBI_ss#)
Ca_Kabuli_NC_021162.1_38822054	Ca_kabuli_chr03	38822054	[T/A]	1067294173
Ca_Kabuli_NC_021162.1_38828026	Ca_kabuli_chr03	38828026	[T/G]	1067294174
Ca_Kabuli_NC_021162.1_38828029	Ca_kabuli_chr03	38828029	[T/C]	1067294175
Ca_Kabuli_NC_021162.1_38878722	Ca_kabuli_chr03	38878722	[A/T]	1067294176
Ca_Kabuli_NC_021162.1_38878767	Ca_kabuli_chr03	38878767	[A/G]	1067294177
Ca_Kabuli_NC_021162.1_38878783	Ca_kabuli_chr03	38878783	[T/A]	1067294178
Ca_Kabuli_NC_021162.1_38878858	Ca_kabuli_chr03	38878858	[T/G]	1067294179
Ca_Kabuli_NC_021162.1_38888421	Ca_kabuli_chr03	38888421	[A/G]	1067294180
Ca_Kabuli_NC_021162.1_38895997	Ca_kabuli_chr03	38895997	[T/C]	1067294181
Ca_Kabuli_NC_021162.1_38896031	Ca_kabuli_chr03	38896031	[T/G]	1067294182
Ca_Kabuli_NC_021162.1_38913449	Ca_kabuli_chr03	38913449	[C/T]	1067294183
Ca_Kabuli_NC_021162.1_38923357	Ca_kabuli_chr03	38923357	[A/G]	1067294184
Ca_Kabuli_NC_021162.1_38923382	Ca_kabuli_chr03	38923382	[T/C]	1067294185
Ca_Kabuli_NC_021162.1_38923390	Ca_kabuli_chr03	38923390	[A/G]	1067294186
Ca_Kabuli_NC_021162.1_38954393	Ca_kabuli_chr03	38954393	[T/G]	1067294187
Ca_Kabuli_NC_021162.1_38964923	Ca_kabuli_chr03	38964923	[T/A]	1067294188
Ca_Kabuli_NC_021162.1_38964964	Ca_kabuli_chr03	38964964	[A/G]	1067294189
Ca_Kabuli_NC_021162.1_38969280	Ca_kabuli_chr03	38969280	[T/C]	1067294190
Ca_Kabuli_NC_021162.1_38987817	Ca_kabuli_chr03	38987817	[T/G]	1067294191
Ca_Kabuli_NC_021162.1_38987846	Ca_kabuli_chr03	38987846	[A/C]	1067294192
Ca_Kabuli_NC_021162.1_38987868	Ca_kabuli_chr03	38987868	[A/C]	1067294193
Ca_Kabuli_NC_021162.1_38987909	Ca_kabuli_chr03	38987909	[A/T]	1067294194
Ca_Kabuli_NC_021162.1_38987921	Ca_kabuli_chr03	38987921	[G/C]	1067294195
Ca_Kabuli_NC_021162.1_39084932	Ca_kabuli_chr03	39084932	[A/C]	1067294196
Ca_Kabuli_NC_021162.1_39084943	Ca_kabuli_chr03	39084943	[T/A]	1067294197
Ca_Kabuli_NC_021162.1_39085112	Ca_kabuli_chr03	39085112	[A/G]	1067294198
Ca_Kabuli_NC_021162.1_39085156	Ca_kabuli_chr03	39085156	[T/G]	1067294199
Ca_Kabuli_NC_021162.1_39085188	Ca_kabuli_chr03	39085188	[T/G]	1067294200
Ca_Kabuli_NC_021162.1_39093228	Ca_kabuli_chr03	39093228	[A/T]	1067294201
Ca_Kabuli_NC_021162.1_39093229	Ca_kabuli_chr03	39093229	[A/T]	1067294202
Ca_Kabuli_NC_021162.1_39115312	Ca_kabuli_chr03	39115312	[A/C]	1067294203
Ca_Kabuli_NC_021162.1_39118486	Ca_kabuli_chr03	39118486	[T/C]	1067294204
Ca_Kabuli_NC_021162.1_39118505	Ca_kabuli_chr03	39118505	[A/C]	1067294205
Ca_Kabuli_NC_021162.1_39118508	Ca_kabuli_chr03	39118508	[T/C]	1067294206

SNP IDs	Chromosomes	Physical positions (bp)	SNPs	NCBI SNPdb accession IDs (NCBI_ss#)
Ca_Kabuli_NC_021162.1_39120063	Ca_kabuli_chr03	39120063	[T/C]	1067294207
Ca_Kabuli_NC_021162.1_39121150	Ca_kabuli_chr03	39121150	[A/C]	1067294208
Ca_Kabuli_NC_021162.1_39124757	Ca_kabuli_chr03	39124757	[A/C]	1067294209
Ca_Kabuli_NC_021162.1_39124767	Ca_kabuli_chr03	39124767	[A/C]	1067294210
Ca_Kabuli_NC_021162.1_39124782	Ca_kabuli_chr03	39124782	[A/T]	1067294211
Ca_Kabuli_NC_021162.1_39152610	Ca_kabuli_chr03	39152610	[T/C]	1067294212
Ca_Kabuli_NC_021162.1_39152618	Ca_kabuli_chr03	39152618	[T/C]	1067294213
Ca_Kabuli_NC_021162.1_39202533	Ca_kabuli_chr03	39202533	[A/G]	1067294214
Ca_Kabuli_NC_021162.1_39207111	Ca_kabuli_chr03	39207111	[C/T]	1067294215
Ca_Kabuli_NC_021162.1_39207119	Ca_kabuli_chr03	39207119	[A/T]	1067294216
Ca_Kabuli_NC_021162.1_39236691	Ca_kabuli_chr03	39236691	[A/G]	1067294217
Ca_Kabuli_NC_021162.1_39236734	Ca_kabuli_chr03	39236734	[T/A]	1067294218
Ca_Kabuli_NC_021162.1_39236796	Ca_kabuli_chr03	39236796	[A/G]	1067294219
Ca_Kabuli_NC_021162.1_39236813	Ca_kabuli_chr03	39236813	[A/G]	1067294220
Ca_Kabuli_NC_021162.1_39237231	Ca_kabuli_chr03	39237231	[A/G]	1067294221
Ca_Kabuli_NC_021162.1_39254527	Ca_kabuli_chr03	39254527	[T/G]	1067294222
Ca_Kabuli_NC_021162.1_39255720	Ca_kabuli_chr03	39255720	[T/A]	1067294223
Ca_Kabuli_NC_021162.1_39255797	Ca_kabuli_chr03	39255797	[T/C]	1067294224
Ca_Kabuli_NC_021162.1_39257226	Ca_kabuli_chr03	39257226	[A/T]	1067294225
Ca_Kabuli_NC_021162.1_39289349	Ca_kabuli_chr03	39289349	[T/A]	1067294226
Ca_Kabuli_NC_021162.1_39289350	Ca_kabuli_chr03	39289350	[A/T]	1067294227
Ca_Kabuli_NC_021162.1_39289463	Ca_kabuli_chr03	39289463	[T/G]	1067294228
Ca_Kabuli_NC_021162.1_39289489	Ca_kabuli_chr03	39289489	[T/G]	1067294229
Ca_Kabuli_NC_021162.1_39289511	Ca_kabuli_chr03	39289511	[A/G]	1067294230
Ca_Kabuli_NC_021162.1_39289940	Ca_kabuli_chr03	39289940	[T/C]	1067294231
Ca_Kabuli_NC_021162.1_39323757	Ca_kabuli_chr03	39323757	[T/C]	1067294232
Ca_Kabuli_NC_021162.1_39323759	Ca_kabuli_chr03	39323759	[T/C]	1067294233
Ca_Kabuli_NC_021162.1_39323768	Ca_kabuli_chr03	39323768	[T/C]	1067294234
Ca_Kabuli_NC_021162.1_39323772	Ca_kabuli_chr03	39323772	[A/G]	1067294235
Ca_Kabuli_NC_021162.1_39323813	Ca_kabuli_chr03	39323813	[T/A]	1067294236
Ca_Kabuli_NC_021162.1_39452574	Ca_kabuli_chr03	39452574	[A/C]	1067294237
Ca_Kabuli_NC_021162.1_39486151	Ca_kabuli_chr03	39486151	[A/C]	1067294238
Ca_Kabuli_NC_021162.1_39491576	Ca_kabuli_chr03	39491576	[T/G]	1067294239
Ca_Kabuli_NC_021162.1_39491577	Ca_kabuli_chr03	39491577	[A/T]	1067294240

SNP IDs	Chromosomes	Physical positions (bp)	SNPs	NCBI SNPdb accession IDs (NCBI_ss#)
Ca_Kabuli_NC_021162.1_39491592	Ca_kabuli_chr03	39491592	[T/C]	1067294241
Ca_Kabuli_NC_021162.1_39491595	Ca_kabuli_chr03	39491595	[A/C]	1067294242
Ca_Kabuli_NC_021162.1_39504201	Ca_kabuli_chr03	39504201	[T/C]	1067294243
Ca_Kabuli_NC_021162.1_39504225	Ca_kabuli_chr03	39504225	[C/T]	1067294244
Ca_Kabuli_NC_021162.1_39504237	Ca_kabuli_chr03	39504237	[C/A]	1067294245
Ca_Kabuli_NC_021162.1_39504242	Ca_kabuli_chr03	39504242	[A/G]	1067294246
Ca_Kabuli_NC_021162.1_39519534	Ca_kabuli_chr03	39519534	[T/G]	1067294247
Ca_Kabuli_NC_021162.1_39546930	Ca_kabuli_chr03	39546930	[T/G]	1067294248
Ca_Kabuli_NC_021162.1_39590693	Ca_kabuli_chr03	39590693	[A/C]	1067294249
Ca_Kabuli_NC_021162.1_39592298	Ca_kabuli_chr03	39592298	[A/C]	1067294250
Ca_Kabuli_NC_021162.1_39611444	Ca_kabuli_chr03	39611444	[A/G]	1067294251
Ca_Kabuli_NC_021162.1_39611446	Ca_kabuli_chr03	39611446	[T/A]	1067294252
Ca_Kabuli_NC_021162.1_39611461	Ca_kabuli_chr03	39611461	[A/G]	1067294253
Ca_Kabuli_NC_021162.1_39611468	Ca_kabuli_chr03	39611468	[T/C]	1067294254
Ca_Kabuli_NC_021162.1_39612358	Ca_kabuli_chr03	39612358	[A/G]	1067294255
Ca_Kabuli_NC_021162.1_39612568	Ca_kabuli_chr03	39612568	[A/T]	1067294256
Ca_Kabuli_NC_021162.1_39628916	Ca_kabuli_chr03	39628916	[G/A]	1067294257
Ca_Kabuli_NC_021162.1_39642781	Ca_kabuli_chr03	39642781	[A/T]	1067294258
Ca_Kabuli_NC_021162.1_39642782	Ca_kabuli_chr03	39642782	[T/C]	1067294259
Ca_Kabuli_NC_021162.1_39642783	Ca_kabuli_chr03	39642783	[G/T]	1067294260
Ca_Kabuli_NC_021162.1_39642805	Ca_kabuli_chr03	39642805	[A/G]	1067294261
Ca_Kabuli_NC_021162.1_39644723	Ca_kabuli_chr03	39644723	[A/C]	1067294262
Ca_Kabuli_NC_021162.1_39701359	Ca_kabuli_chr03	39701359	[T/C]	1067294263
Ca_Kabuli_NC_021162.1_39701373	Ca_kabuli_chr03	39701373	[T/G]	1067294264
Ca_Kabuli_NC_021162.1_39701412	Ca_kabuli_chr03	39701412	[A/C]	1067294265
Ca_Kabuli_NC_021162.1_39701553	Ca_kabuli_chr03	39701553	[A/C]	1067294266
Ca_Kabuli_NC_021162.1_39720656	Ca_kabuli_chr03	39720656	[T/G]	1067294267
Ca_Kabuli_NC_021162.1_39720772	Ca_kabuli_chr03	39720772	[T/G]	1067294268
Ca_Kabuli_NC_021162.1_39722359	Ca_kabuli_chr03	39722359	[T/G]	1067294269
Ca_Kabuli_NC_021162.1_39787520	Ca_kabuli_chr03	39787520	[A/T]	1067294270
Ca_Kabuli_NC_021162.1_39787759	Ca_kabuli_chr03	39787759	[A/C]	1067294271
Ca_Kabuli_NC_021162.1_39793492	Ca_kabuli_chr03	39793492	[A/C]	1067294272
Ca_Kabuli_NC_021162.1_39793532	Ca_kabuli_chr03	39793532	[A/T]	1067294273
Ca_Kabuli_NC_021162.1_39793549	Ca_kabuli_chr03	39793549	[T/C]	1067294274

SNP IDs	Chromosomes	Physical positions (bp)	SNPs	NCBI SNPdb accession IDs (NCBI_ss#)
Ca_Kabuli_NC_021162.1_39817064	Ca_kabuli_chr03	39817064	[T/A]	1067294275
Ca_Kabuli_NC_021162.1_39817996	Ca_kabuli_chr03	39817996	[T/C]	1067294276
Ca_Kabuli_NC_021162.1_39839763	Ca_kabuli_chr03	39839763	[T/G]	1067294277
Ca_Kabuli_NC_021162.1_39839975	Ca_kabuli_chr03	39839975	[C/A]	1067294278
Ca_Kabuli_NC_021162.1_39839979	Ca_kabuli_chr03	39839979	[G/A]	1067294279
Ca_Kabuli_NC_021162.1_39839981	Ca_kabuli_chr03	39839981	[G/T]	1067294280
Ca_Kabuli_NC_021162.1_39839997	Ca_kabuli_chr03	39839997	[G/T]	1067294281
Ca_Kabuli_NC_021162.1_39840000	Ca_kabuli_chr03	39840000	[C/T]	1067294282
Ca_Kabuli_NC_021162.1_39871160	Ca_kabuli_chr03	39871160	[A/G]	1067294283
Ca_Kabuli_NC_021162.1_39871167	Ca_kabuli_chr03	39871167	[T/G]	1067294284
Ca_Kabuli_NC_021162.1_39871219	Ca_kabuli_chr03	39871219	[A/G]	1067294285
Ca_Kabuli_NC_021162.1_39871226	Ca_kabuli_chr03	39871226	[A/C]	1067294286
Ca_Kabuli_NC_021162.1_39876167	Ca_kabuli_chr03	39876167	[A/C]	1067294287
Ca_Kabuli_NC_021162.1_39876187	Ca_kabuli_chr03	39876187	[A/C]	1067294288
Ca_Kabuli_NC_021162.1_39883317	Ca_kabuli_chr03	39883317	[T/C]	1067294289
Ca_Kabuli_NC_021162.1_39891064	Ca_kabuli_chr03	39891064	[A/G]	1067294290
Ca_Kabuli_NC_021162.1_39896359	Ca_kabuli_chr03	39896359	[A/G]	1067294291
Ca_Kabuli_NC_021162.1_39896366	Ca_kabuli_chr03	39896366	[A/C]	1067294292
Ca_Kabuli_NC_021162.1_39896379	Ca_kabuli_chr03	39896379	[A/G]	1067294293
Ca_Kabuli_NC_021162.1_39896390	Ca_kabuli_chr03	39896390	[T/G]	1067294294
Ca_Kabuli_NC_021162.1_39899805	Ca_kabuli_chr03	39899805	[T/C]	1067294295
Ca_Kabuli_NC_021162.1_39914426	Ca_kabuli_chr03	39914426	[T/C]	1067294296
Ca_Kabuli_NC_021162.1_39922275	Ca_kabuli_chr03	39922275	[T/G]	1067294297
Ca_Kabuli_NC_021162.1_39922314	Ca_kabuli_chr03	39922314	[T/C]	1067294298
Ca_Kabuli_NC_021162.1_39923370	Ca_kabuli_chr03	39923370	[T/C]	1067294299
Ca_Kabuli_NC_021162.1_39955553	Ca_kabuli_chr03	39955553	[T/G]	1067294300
Ca_Kabuli_NC_021162.1_39955735	Ca_kabuli_chr03	39955735	[A/G]	1067294301
Ca_Kabuli_NC_021162.1_39963553	Ca_kabuli_chr03	39963553	[A/G]	1067294302
Ca_Kabuli_NC_021163.1_167225	Ca_kabuli_chr04	167225	[A/C]	1067294303
Ca_Kabuli_NC_021163.1_167229	Ca_kabuli_chr04	167229	[T/G]	1067294304
Ca_Kabuli_NC_021163.1_394659	Ca_kabuli_chr04	394659	[T/G]	1067294305
Ca_Kabuli_NC_021163.1_394676	Ca_kabuli_chr04	394676	[A/C]	1067294306
Ca_Kabuli_NC_021163.1_394681	Ca_kabuli_chr04	394681	[A/T]	1067294307
Ca_Kabuli_NC_021163.1_394687	Ca_kabuli_chr04	394687	[A/C]	1067294308

SNP IDs	Chromosomes	Physical positions (bp)	SNPs	NCBI SNPdb accession IDs (NCBI_ss#)
Ca_Kabuli_NC_021163.1_394776	Ca_kabuli_chr04	394776	[A/T]	1067294309
Ca_Kabuli_NC_021163.1_402558	Ca_kabuli_chr04	402558	[T/A]	1067294310
Ca_Kabuli_NC_021163.1_402584	Ca_kabuli_chr04	402584	[A/C]	1067294311
Ca_Kabuli_NC_021163.1_411969	Ca_kabuli_chr04	411969	[A/G]	1067294312
Ca_Kabuli_NC_021163.1_455240	Ca_kabuli_chr04	455240	[A/G]	1067294313
Ca_Kabuli_NC_021163.1_455248	Ca_kabuli_chr04	455248	[T/C]	1067294314
Ca_Kabuli_NC_021163.1_479818	Ca_kabuli_chr04	479818	[A/C]	1067294315
Ca_Kabuli_NC_021163.1_479845	Ca_kabuli_chr04	479845	[A/T]	1067294316
Ca_Kabuli_NC_021163.1_479908	Ca_kabuli_chr04	479908	[A/C]	1067294317
Ca_Kabuli_NC_021163.1_479914	Ca_kabuli_chr04	479914	[A/C]	1067294318
Ca_Kabuli_NC_021163.1_479971	Ca_kabuli_chr04	479971	[A/G]	1067294319
Ca_Kabuli_NC_021163.1_479974	Ca_kabuli_chr04	479974	[A/T]	1067294320
Ca_Kabuli_NC_021163.1_479979	Ca_kabuli_chr04	479979	[T/C]	1067294321
Ca_Kabuli_NC_021163.1_480004	Ca_kabuli_chr04	480004	[A/C]	1067294322
Ca_Kabuli_NC_021163.1_480031	Ca_kabuli_chr04	480031	[T/C]	1067294323
Ca_Kabuli_NC_021163.1_480049	Ca_kabuli_chr04	480049	[A/C]	1067294324
Ca_Kabuli_NC_021163.1_480057	Ca_kabuli_chr04	480057	[A/C]	1067294325
Ca_Kabuli_NC_021163.1_480100	Ca_kabuli_chr04	480100	[A/C]	1067294326
Ca_Kabuli_NC_021163.1_480105	Ca_kabuli_chr04	480105	[T/C]	1067294327
Ca_Kabuli_NC_021163.1_480118	Ca_kabuli_chr04	480118	[A/C]	1067294328
Ca_Kabuli_NC_021163.1_480190	Ca_kabuli_chr04	480190	[A/G]	1067294329
Ca_Kabuli_NC_021163.1_480209	Ca_kabuli_chr04	480209	[A/G]	1067294330
Ca_Kabuli_NC_021163.1_480215	Ca_kabuli_chr04	480215	[T/C]	1067294331
Ca_Kabuli_NC_021163.1_480231	Ca_kabuli_chr04	480231	[T/C]	1067294332
Ca_Kabuli_NC_021163.1_480253	Ca_kabuli_chr04	480253	[A/C]	1067294333
Ca_Kabuli_NC_021163.1_485149	Ca_kabuli_chr04	485149	[T/G]	1067294334
Ca_Kabuli_NC_021163.1_488123	Ca_kabuli_chr04	488123	[A/C]	1067294335
Ca_Kabuli_NC_021163.1_504924	Ca_kabuli_chr04	504924	[A/G]	1067294336
Ca_Kabuli_NC_021163.1_590915	Ca_kabuli_chr04	590915	[T/G]	1067294337
Ca_Kabuli_NC_021163.1_595903	Ca_kabuli_chr04	595903	[T/C]	1067294338
Ca_Kabuli_NC_021163.1_595910	Ca_kabuli_chr04	595910	[A/G]	1067294339
Ca_Kabuli_NC_021163.1_615690	Ca_kabuli_chr04	615690	[A/C]	1067294340
Ca_Kabuli_NC_021163.1_615692	Ca_kabuli_chr04	615692	[C/A]	1067294341
Ca_Kabuli_NC_021163.1_615694	Ca_kabuli_chr04	615694	[A/G]	1067294342

SNP IDs	Chromosomes	Physical positions (bp)	SNPs	NCBI SNPdb accession IDs (NCBI_ss#)
Ca_Kabuli_NC_021163.1_615695	Ca_kabuli_chr04	615695	[G/T]	1067294343
Ca_Kabuli_NC_021163.1_615704	Ca_kabuli_chr04	615704	[C/T]	1067294344
Ca_Kabuli_NC_021163.1_615706	Ca_kabuli_chr04	615706	[T/G]	1067294345
Ca_Kabuli_NC_021163.1_615709	Ca_kabuli_chr04	615709	[A/C]	1067294346
Ca_Kabuli_NC_021163.1_646567	Ca_kabuli_chr04	646567	[C/T]	1067294347
Ca_Kabuli_NC_021163.1_659987	Ca_kabuli_chr04	659987	[A/C]	1067294348
Ca_Kabuli_NC_021163.1_660041	Ca_kabuli_chr04	660041	[A/C]	1067294349
Ca_Kabuli_NC_021163.1_660047	Ca_kabuli_chr04	660047	[A/G]	1067294350
Ca_Kabuli_NC_021163.1_660064	Ca_kabuli_chr04	660064	[T/C]	1067294351
Ca_Kabuli_NC_021163.1_660069	Ca_kabuli_chr04	660069	[T/A]	1067294352
Ca_Kabuli_NC_021163.1_677313	Ca_kabuli_chr04	677313	[T/C]	1067294353
Ca_Kabuli_NC_021163.1_709948	Ca_kabuli_chr04	709948	[T/G]	1067294354
Ca_Kabuli_NC_021163.1_746247	Ca_kabuli_chr04	746247	[A/C]	1067294355
Ca_Kabuli_NC_021163.1_746275	Ca_kabuli_chr04	746275	[T/C]	1067294356
Ca_Kabuli_NC_021163.1_746300	Ca_kabuli_chr04	746300	[T/A]	1067294357
Ca_Kabuli_NC_021163.1_746381	Ca_kabuli_chr04	746381	[A/C]	1067294358
Ca_Kabuli_NC_021163.1_749219	Ca_kabuli_chr04	749219	[T/C]	1067294359
Ca_Kabuli_NC_021163.1_767589	Ca_kabuli_chr04	767589	[G/T]	1067294360
Ca_Kabuli_NC_021163.1_767591	Ca_kabuli_chr04	767591	[T/C]	1067294361
Ca_Kabuli_NC_021163.1_767610	Ca_kabuli_chr04	767610	[T/C]	1067294362
Ca_Kabuli_NC_021163.1_770860	Ca_kabuli_chr04	770860	[T/C]	1067294363
Ca_Kabuli_NC_021163.1_790620	Ca_kabuli_chr04	790620	[C/A]	1067294364
Ca_Kabuli_NC_021163.1_790621	Ca_kabuli_chr04	790621	[A/G]	1067294365
Ca_Kabuli_NC_021163.1_825623	Ca_kabuli_chr04	825623	[T/G]	1067294366
Ca_Kabuli_NC_021163.1_825654	Ca_kabuli_chr04	825654	[A/G]	1067294367
Ca_Kabuli_NC_021163.1_825679	Ca_kabuli_chr04	825679	[A/G]	1067294368
Ca_Kabuli_NC_021163.1_827616	Ca_kabuli_chr04	827616	[A/G]	1067294369
Ca_Kabuli_NC_021163.1_827636	Ca_kabuli_chr04	827636	[T/C]	1067294370
Ca_Kabuli_NC_021163.1_827638	Ca_kabuli_chr04	827638	[A/G]	1067294371
Ca_Kabuli_NC_021163.1_839231	Ca_kabuli_chr04	839231	[A/C]	1067294372
Ca_Kabuli_NC_021163.1_839232	Ca_kabuli_chr04	839232	[A/G]	1067294373
Ca_Kabuli_NC_021163.1_848062	Ca_kabuli_chr04	848062	[T/C]	1067294374
Ca_Kabuli_NC_021163.1_848334	Ca_kabuli_chr04	848334	[A/G]	1067294375
Ca_Kabuli_NC_021163.1_868836	Ca_kabuli_chr04	868836	[A/G]	1067294376

SNP IDs	Chromosomes	Physical positions (bp)	SNPs	NCBI SNPdb accession IDs (NCBI_ss#)
Ca_Kabuli_NC_021163.1_868864	Ca_kabuli_chr04	868864	[T/C]	1067294377
Ca_Kabuli_NC_021163.1_875369	Ca_kabuli_chr04	875369	[T/A]	1067294378
Ca_Kabuli_NC_021163.1_875388	Ca_kabuli_chr04	875388	[A/G]	1067294379
Ca_Kabuli_NC_021163.1_875453	Ca_kabuli_chr04	875453	[A/C]	1067294380
Ca_Kabuli_NC_021163.1_877393	Ca_kabuli_chr04	877393	[C/A]	1067294381
Ca_Kabuli_NC_021163.1_877394	Ca_kabuli_chr04	877394	[T/G]	1067294382
Ca_Kabuli_NC_021163.1_877395	Ca_kabuli_chr04	877395	[T/A]	1067294383
Ca_Kabuli_NC_021163.1_877396	Ca_kabuli_chr04	877396	[A/T]	1067294384
Ca_Kabuli_NC_021163.1_921871	Ca_kabuli_chr04	921871	[A/T]	1067294385
Ca_Kabuli_NC_021163.1_921872	Ca_kabuli_chr04	921872	[T/C]	1067294386
Ca_Kabuli_NC_021163.1_921873	Ca_kabuli_chr04	921873	[G/T]	1067294387
Ca_Kabuli_NC_021163.1_929061	Ca_kabuli_chr04	929061	[A/C]	1067294388
Ca_Kabuli_NC_021163.1_951370	Ca_kabuli_chr04	951370	[T/G]	1067294389
Ca_Kabuli_NC_021163.1_999365	Ca_kabuli_chr04	999365	[A/C]	1067294390
Ca_Kabuli_NC_021163.1_1044930	Ca_kabuli_chr04	1044930	[A/C]	1067294391
Ca_Kabuli_NC_021163.1_1049374	Ca_kabuli_chr04	1049374	[T/C]	1067294392
Ca_Kabuli_NC_021163.1_1049400	Ca_kabuli_chr04	1049400	[A/G]	1067294393
Ca_Kabuli_NC_021163.1_1049406	Ca_kabuli_chr04	1049406	[T/C]	1067294394
Ca_Kabuli_NC_021163.1_1049408	Ca_kabuli_chr04	1049408	[T/C]	1067294395
Ca_Kabuli_NC_021163.1_1049432	Ca_kabuli_chr04	1049432	[T/C]	1067294396
Ca_Kabuli_NC_021163.1_1052600	Ca_kabuli_chr04	1052600	[T/C]	1067294397
Ca_Kabuli_NC_021163.1_1052606	Ca_kabuli_chr04	1052606	[T/C]	1067294398
Ca_Kabuli_NC_021163.1_1052633	Ca_kabuli_chr04	1052633	[T/C]	1067294399
Ca_Kabuli_NC_021163.1_1052642	Ca_kabuli_chr04	1052642	[T/G]	1067294400
Ca_Kabuli_NC_021163.1_1052648	Ca_kabuli_chr04	1052648	[A/G]	1067294401
Ca_Kabuli_NC_021163.1_1149922	Ca_kabuli_chr04	1149922	[C/A]	1067294402
Ca_Kabuli_NC_021163.1_1149924	Ca_kabuli_chr04	1149924	[C/A]	1067294403
Ca_Kabuli_NC_021163.1_1149925	Ca_kabuli_chr04	1149925	[C/T]	1067294404
Ca_Kabuli_NC_021163.1_1189966	Ca_kabuli_chr04	1189966	[G/T]	1067294405
Ca_Kabuli_NC_021163.1_1189985	Ca_kabuli_chr04	1189985	[A/C]	1067294406
Ca_Kabuli_NC_021163.1_1217605	Ca_kabuli_chr04	1217605	[A/C]	1067294407
Ca_Kabuli_NC_021163.1_1220348	Ca_kabuli_chr04	1220348	[A/T]	1067294408
Ca_Kabuli_NC_021163.1_1228284	Ca_kabuli_chr04	1228284	[T/C]	1067294409
Ca_Kabuli_NC_021163.1_1259915	Ca_kabuli_chr04	1259915	[T/C]	1067294410

SNP IDs	Chromosomes	Physical positions (bp)	SNPs	NCBI SNPdb accession IDs (NCBI_ss#)
Ca_Kabuli_NC_021163.1_1259950	Ca_kabuli_chr04	1259950	[A/G]	1067294411
Ca_Kabuli_NC_021163.1_1373679	Ca_kabuli_chr04	1373679	[A/T]	1067294412
Ca_Kabuli_NC_021163.1_1390447	Ca_kabuli_chr04	1390447	[T/A]	1067294413
Ca_Kabuli_NC_021163.1_1390455	Ca_kabuli_chr04	1390455	[A/G]	1067294414
Ca_Kabuli_NC_021163.1_1390513	Ca_kabuli_chr04	1390513	[A/C]	1067294415
Ca_Kabuli_NC_021163.1_1395074	Ca_kabuli_chr04	1395074	[T/G]	1067294416
Ca_Kabuli_NC_021163.1_1395113	Ca_kabuli_chr04	1395113	[A/T]	1067294417
Ca_Kabuli_NC_021163.1_1399303	Ca_kabuli_chr04	1399303	[T/A]	1067294418
Ca_Kabuli_NC_021163.1_1474768	Ca_kabuli_chr04	1474768	[T/C]	1067294419
Ca_Kabuli_NC_021163.1_1474782	Ca_kabuli_chr04	1474782	[A/G]	1067294420
Ca_Kabuli_NC_021163.1_1474879	Ca_kabuli_chr04	1474879	[A/T]	1067294421
Ca_Kabuli_NC_021163.1_1474916	Ca_kabuli_chr04	1474916	[T/C]	1067294422
Ca_Kabuli_NC_021163.1_1489043	Ca_kabuli_chr04	1489043	[G/C]	1067294423
Ca_Kabuli_NC_021163.1_1493821	Ca_kabuli_chr04	1493821	[T/C]	1067294424
Ca_Kabuli_NC_021163.1_1493835	Ca_kabuli_chr04	1493835	[T/G]	1067294425
Ca_Kabuli_NC_021163.1_1513737	Ca_kabuli_chr04	1513737	[A/G]	1067294426
Ca_Kabuli_NC_021163.1_1526320	Ca_kabuli_chr04	1526320	[T/G]	1067294427
Ca_Kabuli_NC_021163.1_1526322	Ca_kabuli_chr04	1526322	[G/A]	1067294428
Ca_Kabuli_NC_021163.1_1657205	Ca_kabuli_chr04	1657205	[A/G]	1067294429
Ca_Kabuli_NC_021163.1_1657224	Ca_kabuli_chr04	1657224	[T/G]	1067294430
Ca_Kabuli_NC_021163.1_1657254	Ca_kabuli_chr04	1657254	[T/C]	1067294431
Ca_Kabuli_NC_021163.1_1657258	Ca_kabuli_chr04	1657258	[A/G]	1067294432
Ca_Kabuli_NC_021163.1_1687365	Ca_kabuli_chr04	1687365	[T/C]	1067294433
Ca_Kabuli_NC_021163.1_1687391	Ca_kabuli_chr04	1687391	[A/G]	1067294434
Ca_Kabuli_NC_021163.1_1708370	Ca_kabuli_chr04	1708370	[T/C]	1067294435
Ca_Kabuli_NC_021163.1_1710449	Ca_kabuli_chr04	1710449	[T/A]	1067294436
Ca_Kabuli_NC_021163.1_1711775	Ca_kabuli_chr04	1711775	[A/G]	1067294437
Ca_Kabuli_NC_021163.1_1711813	Ca_kabuli_chr04	1711813	[T/C]	1067294438
Ca_Kabuli_NC_021163.1_1711822	Ca_kabuli_chr04	1711822	[T/C]	1067294439
Ca_Kabuli_NC_021163.1_1711838	Ca_kabuli_chr04	1711838	[T/C]	1067294440
Ca_Kabuli_NC_021163.1_1718415	Ca_kabuli_chr04	1718415	[T/C]	1067294441
Ca_Kabuli_NC_021163.1_1718483	Ca_kabuli_chr04	1718483	[A/G]	1067294442
Ca_Kabuli_NC_021163.1_1755063	Ca_kabuli_chr04	1755063	[G/A]	1067294443
Ca_Kabuli_NC_021163.1_1773978	Ca_kabuli_chr04	1773978	[A/C]	1067294444

SNP IDs	Chromosomes	Physical positions (bp)	SNPs	NCBI SNPdb accession IDs (NCBI_ss#)
Ca_Kabuli_NC_021163.1_1785677	Ca_kabuli_chr04	1785677	[A/C]	1067294445
Ca_Kabuli_NC_021163.1_1813891	Ca_kabuli_chr04	1813891	[A/C]	1067294446
Ca_Kabuli_NC_021163.1_1813907	Ca_kabuli_chr04	1813907	[T/C]	1067294447
Ca_Kabuli_NC_021163.1_1813924	Ca_kabuli_chr04	1813924	[T/A]	1067294448
Ca_Kabuli_NC_021163.1_1816071	Ca_kabuli_chr04	1816071	[T/A]	1067294449
Ca_Kabuli_NC_021163.1_1816718	Ca_kabuli_chr04	1816718	[A/G]	1067294450
Ca_Kabuli_NC_021163.1_1843971	Ca_kabuli_chr04	1843971	[T/G]	1067294451
Ca_Kabuli_NC_021163.1_1988657	Ca_kabuli_chr04	1988657	[T/A]	1067294452
Ca_Kabuli_NC_021163.1_1988667	Ca_kabuli_chr04	1988667	[A/T]	1067294453
Ca_Kabuli_NC_021163.1_1988678	Ca_kabuli_chr04	1988678	[T/C]	1067294454
Ca_Kabuli_NC_021163.1_2015452	Ca_kabuli_chr04	2015452	[A/C]	1067294455
Ca_Kabuli_NC_021163.1_2015473	Ca_kabuli_chr04	2015473	[C/T]	1067294456
Ca_Kabuli_NC_021163.1_2015480	Ca_kabuli_chr04	2015480	[A/T]	1067294457
Ca_Kabuli_NC_021163.1_2015482	Ca_kabuli_chr04	2015482	[T/C]	1067294458
Ca_Kabuli_NC_021163.1_2025198	Ca_kabuli_chr04	2025198	[T/G]	1067294459
Ca_Kabuli_NC_021163.1_2032449	Ca_kabuli_chr04	2032449	[G/A]	1067294460
Ca_Kabuli_NC_021163.1_2032475	Ca_kabuli_chr04	2032475	[G/A]	1067294461
Ca_Kabuli_NC_021163.1_2032480	Ca_kabuli_chr04	2032480	[C/T]	1067294462
Ca_Kabuli_NC_021163.1_2032481	Ca_kabuli_chr04	2032481	[T/G]	1067294463
Ca_Kabuli_NC_021163.1_2032773	Ca_kabuli_chr04	2032773	[T/A]	1067294464
Ca_Kabuli_NC_021163.1_2034266	Ca_kabuli_chr04	2034266	[C/T]	1067294465
Ca_Kabuli_NC_021163.1_2034304	Ca_kabuli_chr04	2034304	[G/A]	1067294466
Ca_Kabuli_NC_021163.1_2034429	Ca_kabuli_chr04	2034429	[C/T]	1067294467
Ca_Kabuli_NC_021163.1_2035841	Ca_kabuli_chr04	2035841	[C/T]	1067294468
Ca_Kabuli_NC_021163.1_2035894	Ca_kabuli_chr04	2035894	[C/A]	1067294469
Ca_Kabuli_NC_021163.1_2051581	Ca_kabuli_chr04	2051581	[A/C]	1067294470
Ca_Kabuli_NC_021163.1_2059046	Ca_kabuli_chr04	2059046	[T/A]	1067294471
Ca_Kabuli_NC_021163.1_2059070	Ca_kabuli_chr04	2059070	[T/A]	1067294472
Ca_Kabuli_NC_021163.1_2059080	Ca_kabuli_chr04	2059080	[A/G]	1067294473
Ca_Kabuli_NC_021163.1_2059082	Ca_kabuli_chr04	2059082	[T/C]	1067294474
Ca_Kabuli_NC_021163.1_2060248	Ca_kabuli_chr04	2060248	[T/C]	1067294475
Ca_Kabuli_NC_021163.1_2060283	Ca_kabuli_chr04	2060283	[A/G]	1067294476
Ca_Kabuli_NC_021163.1_2085185	Ca_kabuli_chr04	2085185	[A/C]	1067294477
Ca_Kabuli_NC_021163.1_2107404	Ca_kabuli_chr04	2107404	[T/G]	1067294478

SNP IDs	Chromosomes	Physical positions (bp)	SNPs	NCBI SNPdb accession IDs (NCBI_ss#)
Ca_Kabuli_NC_021163.1_2107516	Ca_kabuli_chr04	2107516	[G/T]	1067294479
Ca_Kabuli_NC_021163.1_2113227	Ca_kabuli_chr04	2113227	[C/T]	1067294480
Ca_Kabuli_NC_021163.1_2125779	Ca_kabuli_chr04	2125779	[A/T]	1067294481
Ca_Kabuli_NC_021163.1_2125792	Ca_kabuli_chr04	2125792	[T/G]	1067294482
Ca_Kabuli_NC_021163.1_2213688	Ca_kabuli_chr04	2213688	[A/C]	1067294483
Ca_Kabuli_NC_021163.1_2238501	Ca_kabuli_chr04	2238501	[A/G]	1067294484
Ca_Kabuli_NC_021163.1_2238519	Ca_kabuli_chr04	2238519	[A/G]	1067294485
Ca_Kabuli_NC_021163.1_2240156	Ca_kabuli_chr04	2240156	[T/C]	1067294486
Ca_Kabuli_NC_021163.1_2256939	Ca_kabuli_chr04	2256939	[T/C]	1067294487
Ca_Kabuli_NC_021163.1_2258450	Ca_kabuli_chr04	2258450	[A/G]	1067294488
Ca_Kabuli_NC_021163.1_2258453	Ca_kabuli_chr04	2258453	[T/A]	1067294489
Ca_Kabuli_NC_021163.1_2270375	Ca_kabuli_chr04	2270375	[A/C]	1067294490
Ca_Kabuli_NC_021163.1_2311915	Ca_kabuli_chr04	2311915	[T/C]	1067294491
Ca_Kabuli_NC_021163.1_2313927	Ca_kabuli_chr04	2313927	[T/A]	1067294492
Ca_Kabuli_NC_021163.1_2314051	Ca_kabuli_chr04	2314051	[A/G]	1067294493
Ca_Kabuli_NC_021163.1_2396821	Ca_kabuli_chr04	2396821	[T/G]	1067294494
Ca_Kabuli_NC_021163.1_2396873	Ca_kabuli_chr04	2396873	[A/C]	1067294495
Ca_Kabuli_NC_021163.1_2450534	Ca_kabuli_chr04	2450534	[T/A]	1067294496
Ca_Kabuli_NC_021163.1_2545456	Ca_kabuli_chr04	2545456	[A/T]	1067294497
Ca_Kabuli_NC_021163.1_2575939	Ca_kabuli_chr04	2575939	[T/C]	1067294498
Ca_Kabuli_NC_021163.1_2609880	Ca_kabuli_chr04	2609880	[T/C]	1067294499
Ca_Kabuli_NC_021163.1_2609893	Ca_kabuli_chr04	2609893	[T/C]	1067294500
Ca_Kabuli_NC_021163.1_2609926	Ca_kabuli_chr04	2609926	[C/A]	1067294501
Ca_Kabuli_NC_021163.1_2609927	Ca_kabuli_chr04	2609927	[T/G]	1067294502
Ca_Kabuli_NC_021163.1_2609928	Ca_kabuli_chr04	2609928	[C/A]	1067294503
Ca_Kabuli_NC_021163.1_2609930	Ca_kabuli_chr04	2609930	[T/C]	1067294504
Ca_Kabuli_NC_021163.1_2630862	Ca_kabuli_chr04	2630862	[T/C]	1067294505
Ca_Kabuli_NC_021163.1_2630886	Ca_kabuli_chr04	2630886	[T/C]	1067294506
Ca_Kabuli_NC_021163.1_2632382	Ca_kabuli_chr04	2632382	[T/G]	1067294507
Ca_Kabuli_NC_021163.1_2632386	Ca_kabuli_chr04	2632386	[T/G]	1067294508
Ca_Kabuli_NC_021163.1_2886775	Ca_kabuli_chr04	2886775	[T/G]	1067294509
Ca_Kabuli_NC_021163.1_2887609	Ca_kabuli_chr04	2887609	[T/G]	1067294510
Ca_Kabuli_NC_021163.1_2948204	Ca_kabuli_chr04	2948204	[A/G]	1067294511
Ca_Kabuli_NC_021163.1_3002801	Ca_kabuli_chr04	3002801	[T/C]	1067294512

SNP IDs	Chromosomes	Physical positions (bp)	SNPs	NCBI SNPdb accession IDs (NCBI_ss#)
Ca_Kabuli_NC_021163.1_3002810	Ca_kabuli_chr04	3002810	[T/A]	1067294513
Ca_Kabuli_NC_021163.1_3002826	Ca_kabuli_chr04	3002826	[A/C]	1067294514
Ca_Kabuli_NC_021163.1_3037899	Ca_kabuli_chr04	3037899	[A/C]	1067294515
Ca_Kabuli_NC_021163.1_3037905	Ca_kabuli_chr04	3037905	[A/C]	1067294516
Ca_Kabuli_NC_021163.1_3038006	Ca_kabuli_chr04	3038006	[A/G]	1067294517
Ca_Kabuli_NC_021163.1_3038141	Ca_kabuli_chr04	3038141	[A/C]	1067294518
Ca_Kabuli_NC_021163.1_3144608	Ca_kabuli_chr04	3144608	[T/C]	1067294519
Ca_Kabuli_NC_021163.1_3164076	Ca_kabuli_chr04	3164076	[A/G]	1067294520
Ca_Kabuli_NC_021163.1_3164094	Ca_kabuli_chr04	3164094	[T/G]	1067294521
Ca_Kabuli_NC_021163.1_3226079	Ca_kabuli_chr04	3226079	[T/C]	1067294522
Ca_Kabuli_NC_021163.1_3226091	Ca_kabuli_chr04	3226091	[T/G]	1067294523
Ca_Kabuli_NC_021163.1_3226123	Ca_kabuli_chr04	3226123	[T/G]	1067294524
Ca_Kabuli_NC_021163.1_3245405	Ca_kabuli_chr04	3245405	[C/T]	1067294525
Ca_Kabuli_NC_021163.1_3245647	Ca_kabuli_chr04	3245647	[A/G]	1067294526
Ca_Kabuli_NC_021163.1_3280217	Ca_kabuli_chr04	3280217	[G/A]	1067294527
Ca_Kabuli_NC_021163.1_3284239	Ca_kabuli_chr04	3284239	[T/G]	1067294528
Ca_Kabuli_NC_021163.1_3284312	Ca_kabuli_chr04	3284312	[G/A]	1067294529
Ca_Kabuli_NC_021163.1_3284956	Ca_kabuli_chr04	3284956	[T/A]	1067294530
Ca_Kabuli_NC_021163.1_3290298	Ca_kabuli_chr04	3290298	[G/T]	1067294531
Ca_Kabuli_NC_021163.1_3307908	Ca_kabuli_chr04	3307908	[A/T]	1067294532
Ca_Kabuli_NC_021163.1_3353586	Ca_kabuli_chr04	3353586	[A/G]	1067294533
Ca_Kabuli_NC_021163.1_3369440	Ca_kabuli_chr04	3369440	[G/A]	1067294534
Ca_Kabuli_NC_021163.1_3369444	Ca_kabuli_chr04	3369444	[G/A]	1067294535
Ca_Kabuli_NC_021163.1_3400828	Ca_kabuli_chr04	3400828	[C/G]	1067294536
Ca_Kabuli_NC_021163.1_3427522	Ca_kabuli_chr04	3427522	[G/T]	1067294537
Ca_Kabuli_NC_021163.1_3428588	Ca_kabuli_chr04	3428588	[T/G]	1067294538
Ca_Kabuli_NC_021163.1_3428929	Ca_kabuli_chr04	3428929	[T/C]	1067294539
Ca_Kabuli_NC_021163.1_3429035	Ca_kabuli_chr04	3429035	[G/A]	1067294540
Ca_Kabuli_NC_021163.1_3429335	Ca_kabuli_chr04	3429335	[T/C]	1067294541
Ca_Kabuli_NC_021163.1_3453350	Ca_kabuli_chr04	3453350	[A/T]	1067294542
Ca_Kabuli_NC_021163.1_3453989	Ca_kabuli_chr04	3453989	[A/G]	1067294543
Ca_Kabuli_NC_021163.1_3456454	Ca_kabuli_chr04	3456454	[A/G]	1067294544
Ca_Kabuli_NC_021163.1_3542115	Ca_kabuli_chr04	3542115	[A/G]	1067294545
Ca_Kabuli_NC_021163.1_3542150	Ca_kabuli_chr04	3542150	[A/G]	1067294546

SNP IDs	Chromosomes	Physical positions (bp)	SNPs	NCBI SNPdb accession IDs (NCBI_ss#)
Ca_Kabuli_NC_021163.1_3642099	Ca_kabuli_chr04	3642099	[A/C]	1067294547
Ca_Kabuli_NC_021163.1_3642100	Ca_kabuli_chr04	3642100	[T/A]	1067294548
Ca_Kabuli_NC_021163.1_3699203	Ca_kabuli_chr04	3699203	[T/G]	1067294549
Ca_Kabuli_NC_021163.1_3719218	Ca_kabuli_chr04	3719218	[A/G]	1067294550
Ca_Kabuli_NC_021163.1_3735377	Ca_kabuli_chr04	3735377	[T/C]	1067294551
Ca_Kabuli_NC_021163.1_3741522	Ca_kabuli_chr04	3741522	[T/G]	1067294552
Ca_Kabuli_NC_021163.1_3741539	Ca_kabuli_chr04	3741539	[T/G]	1067294553
Ca_Kabuli_NC_021163.1_3741562	Ca_kabuli_chr04	3741562	[T/G]	1067294554
Ca_Kabuli_NC_021163.1_3762538	Ca_kabuli_chr04	3762538	[A/C]	1067294555
Ca_Kabuli_NC_021163.1_3762551	Ca_kabuli_chr04	3762551	[C/G]	1067294556
Ca_Kabuli_NC_021163.1_3762559	Ca_kabuli_chr04	3762559	[T/G]	1067294557
Ca_Kabuli_NC_021163.1_3762569	Ca_kabuli_chr04	3762569	[G/C]	1067294558
Ca_Kabuli_NC_021163.1_3857904	Ca_kabuli_chr04	3857904	[G/C]	1067294559
Ca_Kabuli_NC_021163.1_3857947	Ca_kabuli_chr04	3857947	[T/C]	1067294560
Ca_Kabuli_NC_021163.1_3857951	Ca_kabuli_chr04	3857951	[T/C]	1067294561
Ca_Kabuli_NC_021163.1_3887025	Ca_kabuli_chr04	3887025	[A/C]	1067294562
Ca_Kabuli_NC_021163.1_3888185	Ca_kabuli_chr04	3888185	[A/G]	1067294563
Ca_Kabuli_NC_021163.1_3888244	Ca_kabuli_chr04	3888244	[T/G]	1067294564
Ca_Kabuli_NC_021163.1_3890552	Ca_kabuli_chr04	3890552	[T/C]	1067294565
Ca_Kabuli_NC_021163.1_3890741	Ca_kabuli_chr04	3890741	[A/G]	1067294566
Ca_Kabuli_NC_021163.1_3890777	Ca_kabuli_chr04	3890777	[A/G]	1067294567
Ca_Kabuli_NC_021163.1_3890779	Ca_kabuli_chr04	3890779	[A/G]	1067294568
Ca_Kabuli_NC_021163.1_3890784	Ca_kabuli_chr04	3890784	[T/C]	1067294569
Ca_Kabuli_NC_021163.1_3945255	Ca_kabuli_chr04	3945255	[A/C]	1067294570
Ca_Kabuli_NC_021163.1_3990334	Ca_kabuli_chr04	3990334	[G/A]	1067294571
Ca_Kabuli_NC_021163.1_3990682	Ca_kabuli_chr04	3990682	[G/A]	1067294572
Ca_Kabuli_NC_021163.1_3990867	Ca_kabuli_chr04	3990867	[A/G]	1067294573
Ca_Kabuli_NC_021163.1_4006205	Ca_kabuli_chr04	4006205	[A/G]	1067294574
Ca_Kabuli_NC_021163.1_4006206	Ca_kabuli_chr04	4006206	[G/A]	1067294575
Ca_Kabuli_NC_021163.1_4027829	Ca_kabuli_chr04	4027829	[T/A]	1067294576
Ca_Kabuli_NC_021163.1_4027849	Ca_kabuli_chr04	4027849	[T/A]	1067294577
Ca_Kabuli_NC_021163.1_4027882	Ca_kabuli_chr04	4027882	[A/C]	1067294578
Ca_Kabuli_NC_021163.1_4037453	Ca_kabuli_chr04	4037453	[A/G]	1067294579
Ca_Kabuli_NC_021163.1_4037486	Ca_kabuli_chr04	4037486	[A/G]	1067294580

SNP IDs	Chromosomes	Physical positions (bp)	SNPs	NCBI SNPdb accession IDs (NCBI_ss#)
Ca_Kabuli_NC_021163.1_4097989	Ca_kabuli_chr04	4097989	[G/A]	1067294581
Ca_Kabuli_NC_021163.1_4098041	Ca_kabuli_chr04	4098041	[G/A]	1067294582
Ca_Kabuli_NC_021163.1_4098049	Ca_kabuli_chr04	4098049	[T/G]	1067294583
Ca_Kabuli_NC_021163.1_4141183	Ca_kabuli_chr04	4141183	[T/G]	1067294584
Ca_Kabuli_NC_021163.1_4154507	Ca_kabuli_chr04	4154507	[T/G]	1067294585
Ca_Kabuli_NC_021163.1_4219919	Ca_kabuli_chr04	4219919	[T/A]	1067294586
Ca_Kabuli_NC_021163.1_4219935	Ca_kabuli_chr04	4219935	[A/C]	1067294587
Ca_Kabuli_NC_021163.1_4219958	Ca_kabuli_chr04	4219958	[T/G]	1067294588
Ca_Kabuli_NC_021163.1_4229392	Ca_kabuli_chr04	4229392	[A/T]	1067294589
Ca_Kabuli_NC_021163.1_4245016	Ca_kabuli_chr04	4245016	[G/C]	1067294590
Ca_Kabuli_NC_021163.1_4251974	Ca_kabuli_chr04	4251974	[C/A]	1067294591
Ca_Kabuli_NC_021163.1_4251975	Ca_kabuli_chr04	4251975	[T/G]	1067294592
Ca_Kabuli_NC_021163.1_4251976	Ca_kabuli_chr04	4251976	[C/A]	1067294593
Ca_Kabuli_NC_021163.1_4252110	Ca_kabuli_chr04	4252110	[T/G]	1067294594
Ca_Kabuli_NC_021163.1_4252144	Ca_kabuli_chr04	4252144	[T/G]	1067294595
Ca_Kabuli_NC_021163.1_4255810	Ca_kabuli_chr04	4255810	[T/G]	1067294596
Ca_Kabuli_NC_021163.1_4255935	Ca_kabuli_chr04	4255935	[T/G]	1067294597
Ca_Kabuli_NC_021163.1_4255963	Ca_kabuli_chr04	4255963	[T/C]	1067294598
Ca_Kabuli_NC_021163.1_4255972	Ca_kabuli_chr04	4255972	[A/T]	1067294599
Ca_Kabuli_NC_021163.1_4256030	Ca_kabuli_chr04	4256030	[T/G]	1067294600
Ca_Kabuli_NC_021163.1_4257158	Ca_kabuli_chr04	4257158	[T/G]	1067294601
Ca_Kabuli_NC_021163.1_4257182	Ca_kabuli_chr04	4257182	[T/G]	1067294602
Ca_Kabuli_NC_021163.1_4305814	Ca_kabuli_chr04	4305814	[T/C]	1067294603
Ca_Kabuli_NC_021163.1_4305819	Ca_kabuli_chr04	4305819	[A/G]	1067294604
Ca_Kabuli_NC_021163.1_4305821	Ca_kabuli_chr04	4305821	[A/G]	1067294605
Ca_Kabuli_NC_021163.1_4305832	Ca_kabuli_chr04	4305832	[A/G]	1067294606
Ca_Kabuli_NC_021163.1_4305880	Ca_kabuli_chr04	4305880	[T/C]	1067294607
Ca_Kabuli_NC_021163.1_4305883	Ca_kabuli_chr04	4305883	[A/G]	1067294608
Ca_Kabuli_NC_021163.1_4309183	Ca_kabuli_chr04	4309183	[A/G]	1067294609
Ca_Kabuli_NC_021163.1_4309184	Ca_kabuli_chr04	4309184	[A/T]	1067294610
Ca_Kabuli_NC_021163.1_4309185	Ca_kabuli_chr04	4309185	[T/A]	1067294611
Ca_Kabuli_NC_021163.1_4309245	Ca_kabuli_chr04	4309245	[A/T]	1067294612
Ca_Kabuli_NC_021163.1_4309263	Ca_kabuli_chr04	4309263	[T/C]	1067294613
Ca_Kabuli_NC_021163.1_4309269	Ca_kabuli_chr04	4309269	[T/C]	1067294614

SNP IDs	Chromosomes	Physical positions (bp)	SNPs	NCBI SNPdb accession IDs (NCBI_ss#)
Ca_Kabuli_NC_021163.1_4309286	Ca_kabuli_chr04	4309286	[T/A]	1067294615
Ca_Kabuli_NC_021163.1_4309296	Ca_kabuli_chr04	4309296	[T/C]	1067294616
Ca_Kabuli_NC_021163.1_4309304	Ca_kabuli_chr04	4309304	[T/A]	1067294617
Ca_Kabuli_NC_021163.1_4309307	Ca_kabuli_chr04	4309307	[G/T]	1067294618
Ca_Kabuli_NC_021163.1_4320567	Ca_kabuli_chr04	4320567	[T/A]	1067294619
Ca_Kabuli_NC_021163.1_4325394	Ca_kabuli_chr04	4325394	[T/G]	1067294620
Ca_Kabuli_NC_021163.1_4343571	Ca_kabuli_chr04	4343571	[T/G]	1067294621
Ca_Kabuli_NC_021163.1_4375250	Ca_kabuli_chr04	4375250	[A/T]	1067294622
Ca_Kabuli_NC_021163.1_4375271	Ca_kabuli_chr04	4375271	[T/C]	1067294623
Ca_Kabuli_NC_021163.1_4389287	Ca_kabuli_chr04	4389287	[T/C]	1067294624
Ca_Kabuli_NC_021163.1_4403125	Ca_kabuli_chr04	4403125	[T/C]	1067294625
Ca_Kabuli_NC_021163.1_4403140	Ca_kabuli_chr04	4403140	[A/C]	1067294626
Ca_Kabuli_NC_021163.1_4403177	Ca_kabuli_chr04	4403177	[G/C]	1067294627
Ca_Kabuli_NC_021163.1_4403178	Ca_kabuli_chr04	4403178	[A/C]	1067294628
Ca_Kabuli_NC_021163.1_4403213	Ca_kabuli_chr04	4403213	[A/C]	1067294629
Ca_Kabuli_NC_021163.1_4403248	Ca_kabuli_chr04	4403248	[A/G]	1067294630
Ca_Kabuli_NC_021163.1_4426493	Ca_kabuli_chr04	4426493	[T/G]	1067294631
Ca_Kabuli_NC_021163.1_4426534	Ca_kabuli_chr04	4426534	[T/C]	1067294632
Ca_Kabuli_NC_021163.1_4452213	Ca_kabuli_chr04	4452213	[T/G]	1067294633
Ca_Kabuli_NC_021163.1_4452245	Ca_kabuli_chr04	4452245	[C/A]	1067294634
Ca_Kabuli_NC_021163.1_4452246	Ca_kabuli_chr04	4452246	[A/C]	1067294635
Ca_Kabuli_NC_021163.1_4452247	Ca_kabuli_chr04	4452247	[A/G]	1067294636
Ca_Kabuli_NC_021163.1_4452252	Ca_kabuli_chr04	4452252	[G/A]	1067294637
Ca_Kabuli_NC_021163.1_4452335	Ca_kabuli_chr04	4452335	[A/G]	1067294638
Ca_Kabuli_NC_021163.1_4452337	Ca_kabuli_chr04	4452337	[T/G]	1067294639
Ca_Kabuli_NC_021163.1_4453889	Ca_kabuli_chr04	4453889	[T/G]	1067294640
Ca_Kabuli_NC_021163.1_4462406	Ca_kabuli_chr04	4462406	[A/C]	1067294641
Ca_Kabuli_NC_021163.1_4462408	Ca_kabuli_chr04	4462408	[C/T]	1067294642
Ca_Kabuli_NC_021163.1_4476250	Ca_kabuli_chr04	4476250	[A/T]	1067294643
Ca_Kabuli_NC_021163.1_4476258	Ca_kabuli_chr04	4476258	[T/G]	1067294644
Ca_Kabuli_NC_021163.1_4498780	Ca_kabuli_chr04	4498780	[C/T]	1067294645
Ca_Kabuli_NC_021163.1_4535558	Ca_kabuli_chr04	4535558	[A/C]	1067294646
Ca_Kabuli_NC_021163.1_4536205	Ca_kabuli_chr04	4536205	[G/A]	1067294647
Ca_Kabuli_NC_021163.1_4536270	Ca_kabuli_chr04	4536270	[T/G]	1067294648

SNP IDs	Chromosomes	Physical positions (bp)	SNPs	NCBI SNPdb accession IDs (NCBI_ss#)
Ca_Kabuli_NC_021163.1_4536328	Ca_kabuli_chr04	4536328	[G/T]	1067294649
Ca_Kabuli_NC_021163.1_4567258	Ca_kabuli_chr04	4567258	[A/G]	1067294650
Ca_Kabuli_NC_021163.1_4567284	Ca_kabuli_chr04	4567284	[T/C]	1067294651
Ca_Kabuli_NC_021163.1_4567299	Ca_kabuli_chr04	4567299	[A/G]	1067294652
Ca_Kabuli_NC_021163.1_4605188	Ca_kabuli_chr04	4605188	[A/G]	1067294653
Ca_Kabuli_NC_021163.1_4605235	Ca_kabuli_chr04	4605235	[A/C]	1067294654
Ca_Kabuli_NC_021163.1_4605290	Ca_kabuli_chr04	4605290	[A/G]	1067294655
Ca_Kabuli_NC_021163.1_4605293	Ca_kabuli_chr04	4605293	[T/G]	1067294656
Ca_Kabuli_NC_021163.1_4605356	Ca_kabuli_chr04	4605356	[A/T]	1067294657
Ca_Kabuli_NC_021163.1_4622091	Ca_kabuli_chr04	4622091	[T/C]	1067294658
Ca_Kabuli_NC_021163.1_4622094	Ca_kabuli_chr04	4622094	[A/C]	1067294659
Ca_Kabuli_NC_021163.1_4622921	Ca_kabuli_chr04	4622921	[T/A]	1067294660
Ca_Kabuli_NC_021163.1_4633934	Ca_kabuli_chr04	4633934	[T/C]	1067294661
Ca_Kabuli_NC_021163.1_4633959	Ca_kabuli_chr04	4633959	[T/C]	1067294662
Ca_Kabuli_NC_021163.1_4635998	Ca_kabuli_chr04	4635998	[T/G]	1067294663
Ca_Kabuli_NC_021163.1_4636518	Ca_kabuli_chr04	4636518	[T/G]	1067294664
Ca_Kabuli_NC_021163.1_4636555	Ca_kabuli_chr04	4636555	[A/C]	1067294665
Ca_Kabuli_NC_021163.1_4675176	Ca_kabuli_chr04	4675176	[T/G]	1067294666
Ca_Kabuli_NC_021163.1_4675182	Ca_kabuli_chr04	4675182	[T/G]	1067294667
Ca_Kabuli_NC_021163.1_4679252	Ca_kabuli_chr04	4679252	[A/C]	1067294668
Ca_Kabuli_NC_021163.1_4689116	Ca_kabuli_chr04	4689116	[T/C]	1067294669
Ca_Kabuli_NC_021163.1_4689148	Ca_kabuli_chr04	4689148	[A/G]	1067294670
Ca_Kabuli_NC_021163.1_4822685	Ca_kabuli_chr04	4822685	[T/C]	1067294671
Ca_Kabuli_NC_021163.1_4890259	Ca_kabuli_chr04	4890259	[T/C]	1067294672
Ca_Kabuli_NC_021163.1_4890299	Ca_kabuli_chr04	4890299	[T/C]	1067294673
Ca_Kabuli_NC_021163.1_4897950	Ca_kabuli_chr04	4897950	[A/C]	1067294674
Ca_Kabuli_NC_021163.1_4911083	Ca_kabuli_chr04	4911083	[T/C]	1067294675
Ca_Kabuli_NC_021163.1_4911130	Ca_kabuli_chr04	4911130	[T/C]	1067294676
Ca_Kabuli_NC_021163.1_4911160	Ca_kabuli_chr04	4911160	[A/C]	1067294677
Ca_Kabuli_NC_021163.1_4920706	Ca_kabuli_chr04	4920706	[T/C]	1067294678
Ca_Kabuli_NC_021163.1_4920712	Ca_kabuli_chr04	4920712	[A/T]	1067294679
Ca_Kabuli_NC_021163.1_4928157	Ca_kabuli_chr04	4928157	[A/G]	1067294680
Ca_Kabuli_NC_021163.1_4928180	Ca_kabuli_chr04	4928180	[A/T]	1067294681
Ca_Kabuli_NC_021163.1_4928192	Ca_kabuli_chr04	4928192	[T/A]	1067294682

SNP IDs	Chromosomes	Physical positions (bp)	SNPs	NCBI SNPdb accession IDs (NCBI_ss#)
Ca_Kabuli_NC_021163.1_4928764	Ca_kabuli_chr04	4928764	[T/C]	1067294683
Ca_Kabuli_NC_021163.1_4928770	Ca_kabuli_chr04	4928770	[A/T]	1067294684
Ca_Kabuli_NC_021163.1_4951176	Ca_kabuli_chr04	4951176	[A/G]	1067294685
Ca_Kabuli_NC_021163.1_4951189	Ca_kabuli_chr04	4951189	[G/C]	1067294686
Ca_Kabuli_NC_021163.1_4951194	Ca_kabuli_chr04	4951194	[G/C]	1067294687
Ca_Kabuli_NC_021163.1_4951205	Ca_kabuli_chr04	4951205	[A/C]	1067294688
Ca_Kabuli_NC_021163.1_4953602	Ca_kabuli_chr04	4953602	[T/G]	1067294689
Ca_Kabuli_NC_021163.1_4953623	Ca_kabuli_chr04	4953623	[T/G]	1067294690
Ca_Kabuli_NC_021163.1_4953681	Ca_kabuli_chr04	4953681	[T/C]	1067294691
Ca_Kabuli_NC_021163.1_4953690	Ca_kabuli_chr04	4953690	[A/G]	1067294692
Ca_Kabuli_NC_021163.1_4953697	Ca_kabuli_chr04	4953697	[A/C]	1067294693
Ca_Kabuli_NC_021163.1_5017018	Ca_kabuli_chr04	5017018	[A/C]	1067294694
Ca_Kabuli_NC_021163.1_5017088	Ca_kabuli_chr04	5017088	[A/G]	1067294695
Ca_Kabuli_NC_021163.1_5017090	Ca_kabuli_chr04	5017090	[G/C]	1067294696
Ca_Kabuli_NC_021163.1_5017120	Ca_kabuli_chr04	5017120	[A/C]	1067294697
Ca_Kabuli_NC_021163.1_5086698	Ca_kabuli_chr04	5086698	[A/G]	1067294698
Ca_Kabuli_NC_021163.1_5086729	Ca_kabuli_chr04	5086729	[A/G]	1067294699
Ca_Kabuli_NC_021163.1_5086736	Ca_kabuli_chr04	5086736	[T/C]	1067294700
Ca_Kabuli_NC_021163.1_5092361	Ca_kabuli_chr04	5092361	[C/A]	1067294701
Ca_Kabuli_NC_021163.1_5101729	Ca_kabuli_chr04	5101729	[A/G]	1067294702
Ca_Kabuli_NC_021163.1_5155611	Ca_kabuli_chr04	5155611	[A/C]	1067294703
Ca_Kabuli_NC_021163.1_5155621	Ca_kabuli_chr04	5155621	[A/G]	1067294704
Ca_Kabuli_NC_021163.1_5186399	Ca_kabuli_chr04	5186399	[T/C]	1067294705
Ca_Kabuli_NC_021163.1_5186403	Ca_kabuli_chr04	5186403	[T/C]	1067294706
Ca_Kabuli_NC_021163.1_5186412	Ca_kabuli_chr04	5186412	[A/G]	1067294707
Ca_Kabuli_NC_021163.1_5207834	Ca_kabuli_chr04	5207834	[A/G]	1067294708
Ca_Kabuli_NC_021163.1_5207847	Ca_kabuli_chr04	5207847	[A/G]	1067294709
Ca_Kabuli_NC_021163.1_5207850	Ca_kabuli_chr04	5207850	[A/G]	1067294710
Ca_Kabuli_NC_021163.1_5207854	Ca_kabuli_chr04	5207854	[A/G]	1067294711
Ca_Kabuli_NC_021163.1_5207859	Ca_kabuli_chr04	5207859	[T/C]	1067294712
Ca_Kabuli_NC_021163.1_5223159	Ca_kabuli_chr04	5223159	[A/T]	1067294713
Ca_Kabuli_NC_021163.1_5232361	Ca_kabuli_chr04	5232361	[T/A]	1067294714
Ca_Kabuli_NC_021163.1_5232391	Ca_kabuli_chr04	5232391	[C/A]	1067294715
Ca_Kabuli_NC_021163.1_5232392	Ca_kabuli_chr04	5232392	[G/C]	1067294716

SNP IDs	Chromosomes	Physical positions (bp)	SNPs	NCBI SNPdb accession IDs (NCBI_ss#)
Ca_Kabuli_NC_021163.1_5232399	Ca_kabuli_chr04	5232399	[A/G]	1067294717
Ca_Kabuli_NC_021163.1_5232401	Ca_kabuli_chr04	5232401	[T/C]	1067294718
Ca_Kabuli_NC_021163.1_5233608	Ca_kabuli_chr04	5233608	[A/C]	1067294719
Ca_Kabuli_NC_021163.1_5233609	Ca_kabuli_chr04	5233609	[C/A]	1067294720
Ca_Kabuli_NC_021163.1_5233635	Ca_kabuli_chr04	5233635	[A/C]	1067294721
Ca_Kabuli_NC_021163.1_5233688	Ca_kabuli_chr04	5233688	[A/C]	1067294722
Ca_Kabuli_NC_021163.1_5233719	Ca_kabuli_chr04	5233719	[T/C]	1067294723
Ca_Kabuli_NC_021163.1_5272594	Ca_kabuli_chr04	5272594	[A/C]	1067294724
Ca_Kabuli_NC_021163.1_5274012	Ca_kabuli_chr04	5274012	[T/C]	1067294725
Ca_Kabuli_NC_021163.1_5282090	Ca_kabuli_chr04	5282090	[A/C]	1067294726
Ca_Kabuli_NC_021163.1_5310888	Ca_kabuli_chr04	5310888	[T/C]	1067294727
Ca_Kabuli_NC_021163.1_5321389	Ca_kabuli_chr04	5321389	[T/A]	1067294728
Ca_Kabuli_NC_021163.1_5321414	Ca_kabuli_chr04	5321414	[A/G]	1067294729
Ca_Kabuli_NC_021163.1_5321423	Ca_kabuli_chr04	5321423	[T/C]	1067294730
Ca_Kabuli_NC_021163.1_5321435	Ca_kabuli_chr04	5321435	[T/C]	1067294731
Ca_Kabuli_NC_021163.1_5345499	Ca_kabuli_chr04	5345499	[T/A]	1067294732
Ca_Kabuli_NC_021163.1_5347480	Ca_kabuli_chr04	5347480	[A/C]	1067294733
Ca_Kabuli_NC_021163.1_5347481	Ca_kabuli_chr04	5347481	[G/T]	1067294734
Ca_Kabuli_NC_021163.1_5376927	Ca_kabuli_chr04	5376927	[A/T]	1067294735
Ca_Kabuli_NC_021163.1_5376928	Ca_kabuli_chr04	5376928	[A/C]	1067294736
Ca_Kabuli_NC_021163.1_5376929	Ca_kabuli_chr04	5376929	[G/T]	1067294737
Ca_Kabuli_NC_021163.1_5377171	Ca_kabuli_chr04	5377171	[T/G]	1067294738
Ca_Kabuli_NC_021163.1_5390317	Ca_kabuli_chr04	5390317	[G/A]	1067294739
Ca_Kabuli_NC_021163.1_5390321	Ca_kabuli_chr04	5390321	[T/C]	1067294740
Ca_Kabuli_NC_021163.1_5390428	Ca_kabuli_chr04	5390428	[A/C]	1067294741
Ca_Kabuli_NC_021163.1_5401291	Ca_kabuli_chr04	5401291	[T/G]	1067294742
Ca_Kabuli_NC_021163.1_5401310	Ca_kabuli_chr04	5401310	[T/G]	1067294743
Ca_Kabuli_NC_021163.1_5405992	Ca_kabuli_chr04	5405992	[T/G]	1067294744
Ca_Kabuli_NC_021163.1_5406164	Ca_kabuli_chr04	5406164	[A/C]	1067294745
Ca_Kabuli_NC_021163.1_5408156	Ca_kabuli_chr04	5408156	[A/C]	1067294746
Ca_Kabuli_NC_021163.1_5458449	Ca_kabuli_chr04	5458449	[A/G]	1067294747
Ca_Kabuli_NC_021163.1_5528418	Ca_kabuli_chr04	5528418	[T/G]	1067294748
Ca_Kabuli_NC_021163.1_5528421	Ca_kabuli_chr04	5528421	[T/G]	1067294749
Ca_Kabuli_NC_021163.1_5528426	Ca_kabuli_chr04	5528426	[A/G]	1067294750

SNP IDs	Chromosomes	Physical positions (bp)	SNPs	NCBI SNPdb accession IDs (NCBI_ss#)
Ca_Kabuli_NC_021163.1_5575091	Ca_kabuli_chr04	5575091	[A/T]	1067294751
Ca_Kabuli_NC_021163.1_5575822	Ca_kabuli_chr04	5575822	[A/T]	1067294752
Ca_Kabuli_NC_021163.1_5595089	Ca_kabuli_chr04	5595089	[A/C]	1067294753
Ca_Kabuli_NC_021163.1_5595101	Ca_kabuli_chr04	5595101	[A/G]	1067294754
Ca_Kabuli_NC_021163.1_5613386	Ca_kabuli_chr04	5613386	[A/T]	1067294755
Ca_Kabuli_NC_021163.1_5630336	Ca_kabuli_chr04	5630336	[G/A]	1067294756
Ca_Kabuli_NC_021163.1_5630342	Ca_kabuli_chr04	5630342	[A/T]	1067294757
Ca_Kabuli_NC_021163.1_5647745	Ca_kabuli_chr04	5647745	[T/G]	1067294758
Ca_Kabuli_NC_021163.1_5647748	Ca_kabuli_chr04	5647748	[T/C]	1067294759
Ca_Kabuli_NC_021163.1_5647760	Ca_kabuli_chr04	5647760	[T/G]	1067294760
Ca_Kabuli_NC_021163.1_5701888	Ca_kabuli_chr04	5701888	[C/T]	1067294761
Ca_Kabuli_NC_021163.1_5701900	Ca_kabuli_chr04	5701900	[G/T]	1067294762
Ca_Kabuli_NC_021163.1_5701902	Ca_kabuli_chr04	5701902	[C/T]	1067294763
Ca_Kabuli_NC_021163.1_5701905	Ca_kabuli_chr04	5701905	[C/T]	1067294764
Ca_Kabuli_NC_021163.1_5701907	Ca_kabuli_chr04	5701907	[G/T]	1067294765
Ca_Kabuli_NC_021163.1_5701913	Ca_kabuli_chr04	5701913	[T/C]	1067294766
Ca_Kabuli_NC_021163.1_5701919	Ca_kabuli_chr04	5701919	[A/T]	1067294767
Ca_Kabuli_NC_021163.1_5710437	Ca_kabuli_chr04	5710437	[T/A]	1067294768
Ca_Kabuli_NC_021163.1_5710447	Ca_kabuli_chr04	5710447	[T/G]	1067294769
Ca_Kabuli_NC_021163.1_5747426	Ca_kabuli_chr04	5747426	[A/C]	1067294770
Ca_Kabuli_NC_021163.1_5779788	Ca_kabuli_chr04	5779788	[A/T]	1067294771
Ca_Kabuli_NC_021163.1_5779789	Ca_kabuli_chr04	5779789	[A/C]	1067294772
Ca_Kabuli_NC_021163.1_5779790	Ca_kabuli_chr04	5779790	[C/A]	1067294773
Ca_Kabuli_NC_021163.1_5779959	Ca_kabuli_chr04	5779959	[A/G]	1067294774
Ca_Kabuli_NC_021163.1_5841468	Ca_kabuli_chr04	5841468	[A/C]	1067294775
Ca_Kabuli_NC_021163.1_5900290	Ca_kabuli_chr04	5900290	[C/T]	1067294776
Ca_Kabuli_NC_021163.1_5907409	Ca_kabuli_chr04	5907409	[T/G]	1067294777
Ca_Kabuli_NC_021163.1_5907465	Ca_kabuli_chr04	5907465	[C/A]	1067294778
Ca_Kabuli_NC_021163.1_5907470	Ca_kabuli_chr04	5907470	[G/C]	1067294779
Ca_Kabuli_NC_021163.1_5907472	Ca_kabuli_chr04	5907472	[C/A]	1067294780
Ca_Kabuli_NC_021163.1_5919776	Ca_kabuli_chr04	5919776	[T/G]	1067294781
Ca_Kabuli_NC_021163.1_5920631	Ca_kabuli_chr04	5920631	[A/C]	1067294782
Ca_Kabuli_NC_021163.1_5920659	Ca_kabuli_chr04	5920659	[T/G]	1067294783
Ca_Kabuli_NC_021163.1_5975388	Ca_kabuli_chr04	5975388	[T/A]	1067294784

SNP IDs	Chromosomes	Physical positions (bp)	SNPs	NCBI SNPdb accession IDs (NCBI_ss#)
Ca_Kabuli_NC_021163.1_5997952	Ca_kabuli_chr04	5997952	[A/C]	1067294785
Ca_Kabuli_NC_021163.1_5997958	Ca_kabuli_chr04	5997958	[A/C]	1067294786
Ca_Kabuli_NC_021163.1_5997964	Ca_kabuli_chr04	5997964	[A/C]	1067294787
Ca_Kabuli_NC_021163.1_6006828	Ca_kabuli_chr04	6006828	[T/G]	1067294788
Ca_Kabuli_NC_021163.1_6006836	Ca_kabuli_chr04	6006836	[A/G]	1067294789
Ca_Kabuli_NC_021163.1_6006843	Ca_kabuli_chr04	6006843	[T/G]	1067294790
Ca_Kabuli_NC_021163.1_6040583	Ca_kabuli_chr04	6040583	[A/C]	1067294791
Ca_Kabuli_NC_021163.1_6041154	Ca_kabuli_chr04	6041154	[A/C]	1067294792
Ca_Kabuli_NC_021163.1_6076570	Ca_kabuli_chr04	6076570	[T/G]	1067294793
Ca_Kabuli_NC_021163.1_6076597	Ca_kabuli_chr04	6076597	[A/T]	1067294794
Ca_Kabuli_NC_021163.1_6083434	Ca_kabuli_chr04	6083434	[T/G]	1067294795
Ca_Kabuli_NC_021163.1_6102249	Ca_kabuli_chr04	6102249	[T/G]	1067294796
Ca_Kabuli_NC_021163.1_6205353	Ca_kabuli_chr04	6205353	[A/G]	1067294797
Ca_Kabuli_NC_021163.1_6264855	Ca_kabuli_chr04	6264855	[T/A]	1067294798
Ca_Kabuli_NC_021163.1_6281228	Ca_kabuli_chr04	6281228	[A/C]	1067294799
Ca_Kabuli_NC_021163.1_6338691	Ca_kabuli_chr04	6338691	[C/A]	1067294800
Ca_Kabuli_NC_021163.1_6338692	Ca_kabuli_chr04	6338692	[T/G]	1067294801
Ca_Kabuli_NC_021163.1_6389134	Ca_kabuli_chr04	6389134	[T/C]	1067294802
Ca_Kabuli_NC_021163.1_6389180	Ca_kabuli_chr04	6389180	[A/G]	1067294803
Ca_Kabuli_NC_021163.1_6389193	Ca_kabuli_chr04	6389193	[A/G]	1067294804
Ca_Kabuli_NC_021163.1_6389195	Ca_kabuli_chr04	6389195	[A/G]	1067294805
Ca_Kabuli_NC_021163.1_6389203	Ca_kabuli_chr04	6389203	[A/C]	1067294806
Ca_Kabuli_NC_021163.1_6391444	Ca_kabuli_chr04	6391444	[A/C]	1067294807
Ca_Kabuli_NC_021163.1_6408516	Ca_kabuli_chr04	6408516	[A/G]	1067294808
Ca_Kabuli_NC_021163.1_6408553	Ca_kabuli_chr04	6408553	[A/G]	1067294809
Ca_Kabuli_NC_021163.1_6411034	Ca_kabuli_chr04	6411034	[T/C]	1067294810
Ca_Kabuli_NC_021163.1_6411039	Ca_kabuli_chr04	6411039	[G/A]	1067294811
Ca_Kabuli_NC_021163.1_6415015	Ca_kabuli_chr04	6415015	[T/G]	1067294812
Ca_Kabuli_NC_021163.1_6415032	Ca_kabuli_chr04	6415032	[A/C]	1067294813
Ca_Kabuli_NC_021163.1_6456474	Ca_kabuli_chr04	6456474	[T/C]	1067294814
Ca_Kabuli_NC_021163.1_6461026	Ca_kabuli_chr04	6461026	[G/C]	1067294815
Ca_Kabuli_NC_021163.1_6462846	Ca_kabuli_chr04	6462846	[C/A]	1067294816
Ca_Kabuli_NC_021163.1_6462847	Ca_kabuli_chr04	6462847	[T/G]	1067294817
Ca_Kabuli_NC_021163.1_6507498	Ca_kabuli_chr04	6507498	[T/G]	1067294818

SNP IDs	Chromosomes	Physical positions (bp)	SNPs	NCBI SNPdb accession IDs (NCBI_ss#)
Ca_Kabuli_NC_021163.1_6507522	Ca_kabuli_chr04	6507522	[A/C]	1067294819
Ca_Kabuli_NC_021163.1_6514899	Ca_kabuli_chr04	6514899	[T/C]	1067294820
Ca_Kabuli_NC_021163.1_6514900	Ca_kabuli_chr04	6514900	[G/T]	1067294821
Ca_Kabuli_NC_021163.1_6544317	Ca_kabuli_chr04	6544317	[A/C]	1067294822
Ca_Kabuli_NC_021163.1_6544397	Ca_kabuli_chr04	6544397	[T/A]	1067294823
Ca_Kabuli_NC_021163.1_6544399	Ca_kabuli_chr04	6544399	[A/T]	1067294824
Ca_Kabuli_NC_021163.1_6559477	Ca_kabuli_chr04	6559477	[T/C]	1067294825
Ca_Kabuli_NC_021163.1_6562602	Ca_kabuli_chr04	6562602	[T/C]	1067294826
Ca_Kabuli_NC_021163.1_6562628	Ca_kabuli_chr04	6562628	[T/G]	1067294827
Ca_Kabuli_NC_021163.1_6567319	Ca_kabuli_chr04	6567319	[T/A]	1067294828
Ca_Kabuli_NC_021163.1_6621474	Ca_kabuli_chr04	6621474	[A/T]	1067294829
Ca_Kabuli_NC_021163.1_6622339	Ca_kabuli_chr04	6622339	[T/C]	1067294830
Ca_Kabuli_NC_021163.1_6622368	Ca_kabuli_chr04	6622368	[A/G]	1067294831
Ca_Kabuli_NC_021163.1_6635780	Ca_kabuli_chr04	6635780	[T/G]	1067294832
Ca_Kabuli_NC_021163.1_6664462	Ca_kabuli_chr04	6664462	[A/C]	1067294833
Ca_Kabuli_NC_021163.1_6666908	Ca_kabuli_chr04	6666908	[T/G]	1067294834
Ca_Kabuli_NC_021163.1_6666916	Ca_kabuli_chr04	6666916	[A/C]	1067294835
Ca_Kabuli_NC_021163.1_6749909	Ca_kabuli_chr04	6749909	[T/C]	1067294836
Ca_Kabuli_NC_021163.1_6761719	Ca_kabuli_chr04	6761719	[T/A]	1067294837
Ca_Kabuli_NC_021163.1_6761759	Ca_kabuli_chr04	6761759	[A/C]	1067294838
Ca_Kabuli_NC_021163.1_6761763	Ca_kabuli_chr04	6761763	[T/C]	1067294839
Ca_Kabuli_NC_021163.1_6816250	Ca_kabuli_chr04	6816250	[A/T]	1067294840
Ca_Kabuli_NC_021163.1_6851628	Ca_kabuli_chr04	6851628	[G/T]	1067294841
Ca_Kabuli_NC_021163.1_6854067	Ca_kabuli_chr04	6854067	[A/C]	1067294842
Ca_Kabuli_NC_021163.1_6854127	Ca_kabuli_chr04	6854127	[T/G]	1067294843
Ca_Kabuli_NC_021163.1_6854135	Ca_kabuli_chr04	6854135	[T/A]	1067294844
Ca_Kabuli_NC_021163.1_6854162	Ca_kabuli_chr04	6854162	[A/C]	1067294845
Ca_Kabuli_NC_021163.1_6854187	Ca_kabuli_chr04	6854187	[A/G]	1067294846
Ca_Kabuli_NC_021163.1_6854533	Ca_kabuli_chr04	6854533	[A/C]	1067294847
Ca_Kabuli_NC_021163.1_6889964	Ca_kabuli_chr04	6889964	[T/G]	1067294848
Ca_Kabuli_NC_021163.1_6889967	Ca_kabuli_chr04	6889967	[A/T]	1067294849
Ca_Kabuli_NC_021163.1_6889974	Ca_kabuli_chr04	6889974	[T/G]	1067294850
Ca_Kabuli_NC_021163.1_6889978	Ca_kabuli_chr04	6889978	[G/A]	1067294851
Ca_Kabuli_NC_021163.1_6889981	Ca_kabuli_chr04	6889981	[T/G]	1067294852

SNP IDs	Chromosomes	Physical positions (bp)	SNPs	NCBI SNPdb accession IDs (NCBI_ss#)
Ca_Kabuli_NC_021163.1_6918370	Ca_kabuli_chr04	6918370	[T/C]	1067294853
Ca_Kabuli_NC_021163.1_6952860	Ca_kabuli_chr04	6952860	[T/G]	1067294854
Ca_Kabuli_NC_021163.1_6952876	Ca_kabuli_chr04	6952876	[T/G]	1067294855
Ca_Kabuli_NC_021163.1_7040253	Ca_kabuli_chr04	7040253	[A/C]	1067294856
Ca_Kabuli_NC_021163.1_7040258	Ca_kabuli_chr04	7040258	[A/C]	1067294857
Ca_Kabuli_NC_021163.1_7068169	Ca_kabuli_chr04	7068169	[C/A]	1067294858
Ca_Kabuli_NC_021163.1_7068170	Ca_kabuli_chr04	7068170	[C/G]	1067294859
Ca_Kabuli_NC_021163.1_7068171	Ca_kabuli_chr04	7068171	[G/A]	1067294860
Ca_Kabuli_NC_021163.1_7069673	Ca_kabuli_chr04	7069673	[A/G]	1067294861
Ca_Kabuli_NC_021163.1_7069678	Ca_kabuli_chr04	7069678	[C/T]	1067294862
Ca_Kabuli_NC_021163.1_7069713	Ca_kabuli_chr04	7069713	[C/T]	1067294863
Ca_Kabuli_NC_021163.1_7142438	Ca_kabuli_chr04	7142438	[T/G]	1067294864
Ca_Kabuli_NC_021163.1_7143222	Ca_kabuli_chr04	7143222	[T/C]	1067294865
Ca_Kabuli_NC_021163.1_7143229	Ca_kabuli_chr04	7143229	[C/T]	1067294866
Ca_Kabuli_NC_021163.1_7143232	Ca_kabuli_chr04	7143232	[C/T]	1067294867
Ca_Kabuli_NC_021163.1_7143234	Ca_kabuli_chr04	7143234	[C/T]	1067294868
Ca_Kabuli_NC_021163.1_7143254	Ca_kabuli_chr04	7143254	[A/C]	1067294869
Ca_Kabuli_NC_021163.1_7155819	Ca_kabuli_chr04	7155819	[A/G]	1067294870
Ca_Kabuli_NC_021163.1_7155857	Ca_kabuli_chr04	7155857	[T/G]	1067294871
Ca_Kabuli_NC_021163.1_7155864	Ca_kabuli_chr04	7155864	[A/G]	1067294872
Ca_Kabuli_NC_021163.1_7163978	Ca_kabuli_chr04	7163978	[T/C]	1067294873
Ca_Kabuli_NC_021163.1_7163980	Ca_kabuli_chr04	7163980	[A/C]	1067294874
Ca_Kabuli_NC_021163.1_7163982	Ca_kabuli_chr04	7163982	[C/T]	1067294875
Ca_Kabuli_NC_021163.1_7164004	Ca_kabuli_chr04	7164004	[A/C]	1067294876
Ca_Kabuli_NC_021163.1_7187783	Ca_kabuli_chr04	7187783	[T/A]	1067294877
Ca_Kabuli_NC_021163.1_7350212	Ca_kabuli_chr04	7350212	[A/T]	1067294878
Ca_Kabuli_NC_021163.1_7395128	Ca_kabuli_chr04	7395128	[A/G]	1067294879
Ca_Kabuli_NC_021163.1_7417035	Ca_kabuli_chr04	7417035	[T/G]	1067294880
Ca_Kabuli_NC_021163.1_7417042	Ca_kabuli_chr04	7417042	[T/G]	1067294881
Ca_Kabuli_NC_021163.1_7523665	Ca_kabuli_chr04	7523665	[T/C]	1067294882
Ca_Kabuli_NC_021163.1_7523666	Ca_kabuli_chr04	7523666	[A/T]	1067294883
Ca_Kabuli_NC_021163.1_7523689	Ca_kabuli_chr04	7523689	[A/C]	1067294884
Ca_Kabuli_NC_021163.1_7548603	Ca_kabuli_chr04	7548603	[A/C]	1067294885
Ca_Kabuli_NC_021163.1_7548632	Ca_kabuli_chr04	7548632	[G/T]	1067294886

SNP IDs	Chromosomes	Physical positions (bp)	SNPs	NCBI SNPdb accession IDs (NCBI_ss#)
Ca_Kabuli_NC_021163.1_7548635	Ca_kabuli_chr04	7548635	[C/G]	1067294887
Ca_Kabuli_NC_021163.1_7548636	Ca_kabuli_chr04	7548636	[G/C]	1067294888
Ca_Kabuli_NC_021163.1_7548639	Ca_kabuli_chr04	7548639	[T/G]	1067294889
Ca_Kabuli_NC_021163.1_7548640	Ca_kabuli_chr04	7548640	[A/C]	1067294890
Ca_Kabuli_NC_021163.1_7561479	Ca_kabuli_chr04	7561479	[A/G]	1067294891
Ca_Kabuli_NC_021163.1_7561491	Ca_kabuli_chr04	7561491	[A/G]	1067294892
Ca_Kabuli_NC_021163.1_7561495	Ca_kabuli_chr04	7561495	[A/G]	1067294893
Ca_Kabuli_NC_021163.1_7561515	Ca_kabuli_chr04	7561515	[T/G]	1067294894
Ca_Kabuli_NC_021163.1_7587496	Ca_kabuli_chr04	7587496	[T/C]	1067294895
Ca_Kabuli_NC_021163.1_7739201	Ca_kabuli_chr04	7739201	[T/G]	1067294896
Ca_Kabuli_NC_021163.1_7771941	Ca_kabuli_chr04	7771941	[A/G]	1067294897
Ca_Kabuli_NC_021163.1_7771948	Ca_kabuli_chr04	7771948	[T/C]	1067294898
Ca_Kabuli_NC_021163.1_7771956	Ca_kabuli_chr04	7771956	[A/G]	1067294899
Ca_Kabuli_NC_021163.1_7806626	Ca_kabuli_chr04	7806626	[A/C]	1067294900
Ca_Kabuli_NC_021163.1_7867676	Ca_kabuli_chr04	7867676	[A/T]	1067294901
Ca_Kabuli_NC_021163.1_7883714	Ca_kabuli_chr04	7883714	[A/C]	1067294902
Ca_Kabuli_NC_021163.1_7898356	Ca_kabuli_chr04	7898356	[T/C]	1067294903
Ca_Kabuli_NC_021163.1_7898383	Ca_kabuli_chr04	7898383	[T/C]	1067294904
Ca_Kabuli_NC_021163.1_7898392	Ca_kabuli_chr04	7898392	[A/G]	1067294905
Ca_Kabuli_NC_021163.1_7920411	Ca_kabuli_chr04	7920411	[G/A]	1067294906
Ca_Kabuli_NC_021163.1_8025364	Ca_kabuli_chr04	8025364	[A/C]	1067294907
Ca_Kabuli_NC_021163.1_8050741	Ca_kabuli_chr04	8050741	[A/T]	1067294908
Ca_Kabuli_NC_021163.1_8063576	Ca_kabuli_chr04	8063576	[T/A]	1067294909
Ca_Kabuli_NC_021163.1_8063608	Ca_kabuli_chr04	8063608	[A/G]	1067294910
Ca_Kabuli_NC_021163.1_8115068	Ca_kabuli_chr04	8115068	[T/G]	1067294911
Ca_Kabuli_NC_021163.1_8115233	Ca_kabuli_chr04	8115233	[A/C]	1067294912
Ca_Kabuli_NC_021163.1_8116598	Ca_kabuli_chr04	8116598	[T/C]	1067294913
Ca_Kabuli_NC_021163.1_8116604	Ca_kabuli_chr04	8116604	[T/G]	1067294914
Ca_Kabuli_NC_021163.1_8116611	Ca_kabuli_chr04	8116611	[A/G]	1067294915
Ca_Kabuli_NC_021163.1_8127200	Ca_kabuli_chr04	8127200	[T/C]	1067294916
Ca_Kabuli_NC_021163.1_8127204	Ca_kabuli_chr04	8127204	[T/C]	1067294917
Ca_Kabuli_NC_021163.1_8127255	Ca_kabuli_chr04	8127255	[A/G]	1067294918
Ca_Kabuli_NC_021163.1_8129337	Ca_kabuli_chr04	8129337	[T/C]	1067294919
Ca_Kabuli_NC_021163.1_8129348	Ca_kabuli_chr04	8129348	[A/T]	1067294920

SNP IDs	Chromosomes	Physical positions (bp)	SNPs	NCBI SNPdb accession IDs (NCBI_ss#)
Ca_Kabuli_NC_021163.1_8129397	Ca_kabuli_chr04	8129397	[A/G]	1067294921
Ca_Kabuli_NC_021163.1_8186648	Ca_kabuli_chr04	8186648	[A/C]	1067294922
Ca_Kabuli_NC_021163.1_8186655	Ca_kabuli_chr04	8186655	[A/C]	1067294923
Ca_Kabuli_NC_021163.1_8186677	Ca_kabuli_chr04	8186677	[A/C]	1067294924
Ca_Kabuli_NC_021163.1_8194139	Ca_kabuli_chr04	8194139	[T/C]	1067294925
Ca_Kabuli_NC_021163.1_8236340	Ca_kabuli_chr04	8236340	[T/G]	1067294926
Ca_Kabuli_NC_021163.1_8236402	Ca_kabuli_chr04	8236402	[A/C]	1067294927
Ca_Kabuli_NC_021163.1_8237305	Ca_kabuli_chr04	8237305	[A/G]	1067294928
Ca_Kabuli_NC_021163.1_8245769	Ca_kabuli_chr04	8245769	[A/G]	1067294929
Ca_Kabuli_NC_021163.1_8268995	Ca_kabuli_chr04	8268995	[T/C]	1067294930
Ca_Kabuli_NC_021163.1_8290396	Ca_kabuli_chr04	8290396	[A/C]	1067294931
Ca_Kabuli_NC_021163.1_8302146	Ca_kabuli_chr04	8302146	[A/G]	1067294932
Ca_Kabuli_NC_021163.1_8302168	Ca_kabuli_chr04	8302168	[T/C]	1067294933
Ca_Kabuli_NC_021163.1_8302198	Ca_kabuli_chr04	8302198	[A/C]	1067294934
Ca_Kabuli_NC_021163.1_8304444	Ca_kabuli_chr04	8304444	[A/C]	1067294935
Ca_Kabuli_NC_021163.1_8356201	Ca_kabuli_chr04	8356201	[A/C]	1067294936
Ca_Kabuli_NC_021163.1_8361398	Ca_kabuli_chr04	8361398	[A/C]	1067294937
Ca_Kabuli_NC_021163.1_8361399	Ca_kabuli_chr04	8361399	[G/A]	1067294938
Ca_Kabuli_NC_021163.1_8367480	Ca_kabuli_chr04	8367480	[T/G]	1067294939
Ca_Kabuli_NC_021163.1_8369566	Ca_kabuli_chr04	8369566	[A/C]	1067294940
Ca_Kabuli_NC_021163.1_8404188	Ca_kabuli_chr04	8404188	[A/T]	1067294941
Ca_Kabuli_NC_021163.1_8471002	Ca_kabuli_chr04	8471002	[A/G]	1067294942
Ca_Kabuli_NC_021163.1_8473138	Ca_kabuli_chr04	8473138	[T/G]	1067294943
Ca_Kabuli_NC_021163.1_8475634	Ca_kabuli_chr04	8475634	[A/C]	1067294944
Ca_Kabuli_NC_021163.1_8496747	Ca_kabuli_chr04	8496747	[A/G]	1067294945
Ca_Kabuli_NC_021163.1_8533012	Ca_kabuli_chr04	8533012	[T/C]	1067294946
Ca_Kabuli_NC_021163.1_8533017	Ca_kabuli_chr04	8533017	[T/C]	1067294947
Ca_Kabuli_NC_021163.1_8596243	Ca_kabuli_chr04	8596243	[T/G]	1067294948
Ca_Kabuli_NC_021163.1_8596268	Ca_kabuli_chr04	8596268	[T/G]	1067294949
Ca_Kabuli_NC_021163.1_8620826	Ca_kabuli_chr04	8620826	[T/C]	1067294950
Ca_Kabuli_NC_021163.1_8627547	Ca_kabuli_chr04	8627547	[A/G]	1067294951
Ca_Kabuli_NC_021163.1_8656398	Ca_kabuli_chr04	8656398	[A/G]	1067294952
Ca_Kabuli_NC_021163.1_8656635	Ca_kabuli_chr04	8656635	[T/C]	1067294953
Ca_Kabuli_NC_021163.1_8659774	Ca_kabuli_chr04	8659774	[G/A]	1067294954

SNP IDs	Chromosomes	Physical positions (bp)	SNPs	NCBI SNPdb accession IDs (NCBI_ss#)
Ca_Kabuli_NC_021163.1_8660115	Ca_kabuli_chr04	8660115	[T/A]	1067294955
Ca_Kabuli_NC_021163.1_8660123	Ca_kabuli_chr04	8660123	[A/G]	1067294956
Ca_Kabuli_NC_021163.1_8660125	Ca_kabuli_chr04	8660125	[A/C]	1067294957
Ca_Kabuli_NC_021163.1_8660131	Ca_kabuli_chr04	8660131	[T/C]	1067294958
Ca_Kabuli_NC_021163.1_8660144	Ca_kabuli_chr04	8660144	[T/C]	1067294959
Ca_Kabuli_NC_021163.1_8660196	Ca_kabuli_chr04	8660196	[T/C]	1067294960
Ca_Kabuli_NC_021163.1_8660198	Ca_kabuli_chr04	8660198	[T/C]	1067294961
Ca_Kabuli_NC_021163.1_8660205	Ca_kabuli_chr04	8660205	[A/G]	1067294962
Ca_Kabuli_NC_021163.1_8660252	Ca_kabuli_chr04	8660252	[A/G]	1067294963
Ca_Kabuli_NC_021163.1_8661559	Ca_kabuli_chr04	8661559	[G/A]	1067294964
Ca_Kabuli_NC_021163.1_8661588	Ca_kabuli_chr04	8661588	[T/C]	1067294965
Ca_Kabuli_NC_021163.1_8661607	Ca_kabuli_chr04	8661607	[T/C]	1067294966
Ca_Kabuli_NC_021163.1_8662340	Ca_kabuli_chr04	8662340	[G/C]	1067294967
Ca_Kabuli_NC_021163.1_8662348	Ca_kabuli_chr04	8662348	[C/A]	1067294968
Ca_Kabuli_NC_021163.1_8662350	Ca_kabuli_chr04	8662350	[G/A]	1067294969
Ca_Kabuli_NC_021163.1_8668945	Ca_kabuli_chr04	8668945	[C/T]	1067294970
Ca_Kabuli_NC_021163.1_8669513	Ca_kabuli_chr04	8669513	[A/G]	1067294971
Ca_Kabuli_NC_021163.1_8669515	Ca_kabuli_chr04	8669515	[T/C]	1067294972
Ca_Kabuli_NC_021163.1_8669560	Ca_kabuli_chr04	8669560	[A/C]	1067294973
Ca_Kabuli_NC_021163.1_8677598	Ca_kabuli_chr04	8677598	[C/T]	1067294974
Ca_Kabuli_NC_021163.1_8681906	Ca_kabuli_chr04	8681906	[A/T]	1067294975
Ca_Kabuli_NC_021163.1_8681949	Ca_kabuli_chr04	8681949	[A/G]	1067294976
Ca_Kabuli_NC_021163.1_8682191	Ca_kabuli_chr04	8682191	[T/C]	1067294977
Ca_Kabuli_NC_021163.1_8683428	Ca_kabuli_chr04	8683428	[C/T]	1067294978
Ca_Kabuli_NC_021163.1_8683435	Ca_kabuli_chr04	8683435	[T/C]	1067294979
Ca_Kabuli_NC_021163.1_8683887	Ca_kabuli_chr04	8683887	[G/A]	1067294980
Ca_Kabuli_NC_021163.1_8683953	Ca_kabuli_chr04	8683953	[C/G]	1067294981
Ca_Kabuli_NC_021163.1_8684184	Ca_kabuli_chr04	8684184	[G/A]	1067294982
Ca_Kabuli_NC_021163.1_8684185	Ca_kabuli_chr04	8684185	[G/A]	1067294983
Ca_Kabuli_NC_021163.1_8684220	Ca_kabuli_chr04	8684220	[A/G]	1067294984
Ca_Kabuli_NC_021163.1_8685875	Ca_kabuli_chr04	8685875	[G/A]	1067294985
Ca_Kabuli_NC_021163.1_8686216	Ca_kabuli_chr04	8686216	[A/T]	1067294986
Ca_Kabuli_NC_021163.1_8692150	Ca_kabuli_chr04	8692150	[C/T]	1067294987
Ca_Kabuli_NC_021163.1_8692321	Ca_kabuli_chr04	8692321	[C/T]	1067294988

SNP IDs	Chromosomes	Physical positions (bp)	SNPs	NCBI SNPdb accession IDs (NCBI_ss#)
Ca_Kabuli_NC_021163.1_8695562	Ca_kabuli_chr04	8695562	[T/C]	1067294989
Ca_Kabuli_NC_021163.1_8696395	Ca_kabuli_chr04	8696395	[G/C]	1067294990
Ca_Kabuli_NC_021163.1_8697219	Ca_kabuli_chr04	8697219	[C/T]	1067294991
Ca_Kabuli_NC_021163.1_8697220	Ca_kabuli_chr04	8697220	[A/T]	1067294992
Ca_Kabuli_NC_021163.1_8697226	Ca_kabuli_chr04	8697226	[A/G]	1067294993
Ca_Kabuli_NC_021163.1_8751611	Ca_kabuli_chr04	8751611	[T/C]	1067294994
Ca_Kabuli_NC_021163.1_8751618	Ca_kabuli_chr04	8751618	[A/C]	1067294995
Ca_Kabuli_NC_021163.1_8751626	Ca_kabuli_chr04	8751626	[A/C]	1067294996
Ca_Kabuli_NC_021163.1_8751629	Ca_kabuli_chr04	8751629	[T/C]	1067294997
Ca_Kabuli_NC_021163.1_8751638	Ca_kabuli_chr04	8751638	[A/C]	1067294998
Ca_Kabuli_NC_021163.1_8751642	Ca_kabuli_chr04	8751642	[A/C]	1067294999
Ca_Kabuli_NC_021163.1_8752822	Ca_kabuli_chr04	8752822	[T/C]	1067295000
Ca_Kabuli_NC_021163.1_8776321	Ca_kabuli_chr04	8776321	[C/A]	1067295001
Ca_Kabuli_NC_021163.1_8776322	Ca_kabuli_chr04	8776322	[T/G]	1067295002
Ca_Kabuli_NC_021163.1_8776323	Ca_kabuli_chr04	8776323	[T/A]	1067295003
Ca_Kabuli_NC_021163.1_8776324	Ca_kabuli_chr04	8776324	[C/T]	1067295004
Ca_Kabuli_NC_021163.1_8777418	Ca_kabuli_chr04	8777418	[T/C]	1067295005
Ca_Kabuli_NC_021163.1_8782609	Ca_kabuli_chr04	8782609	[C/T]	1067295006
Ca_Kabuli_NC_021163.1_8783887	Ca_kabuli_chr04	8783887	[G/A]	1067295007
Ca_Kabuli_NC_021163.1_8791636	Ca_kabuli_chr04	8791636	[A/C]	1067295008
Ca_Kabuli_NC_021163.1_8791651	Ca_kabuli_chr04	8791651	[A/C]	1067295009
Ca_Kabuli_NC_021163.1_8805116	Ca_kabuli_chr04	8805116	[C/T]	1067295010
Ca_Kabuli_NC_021163.1_8805504	Ca_kabuli_chr04	8805504	[G/A]	1067295011
Ca_Kabuli_NC_021163.1_8805667	Ca_kabuli_chr04	8805667	[T/C]	1067295012
Ca_Kabuli_NC_021163.1_8805839	Ca_kabuli_chr04	8805839	[A/G]	1067295013
Ca_Kabuli_NC_021163.1_8805867	Ca_kabuli_chr04	8805867	[C/A]	1067295014
Ca_Kabuli_NC_021163.1_8806078	Ca_kabuli_chr04	8806078	[A/G]	1067295015
Ca_Kabuli_NC_021163.1_8806108	Ca_kabuli_chr04	8806108	[T/C]	1067295016
Ca_Kabuli_NC_021163.1_8806137	Ca_kabuli_chr04	8806137	[T/C]	1067295017
Ca_Kabuli_NC_021163.1_8809411	Ca_kabuli_chr04	8809411	[A/C]	1067295018
Ca_Kabuli_NC_021163.1_8844835	Ca_kabuli_chr04	8844835	[A/G]	1067295019
Ca_Kabuli_NC_021163.1_8845593	Ca_kabuli_chr04	8845593	[T/G]	1067295020
Ca_Kabuli_NC_021163.1_8845663	Ca_kabuli_chr04	8845663	[A/G]	1067295021
Ca_Kabuli_NC_021163.1_8848673	Ca_kabuli_chr04	8848673	[T/C]	1067295022

SNP IDs	Chromosomes	Physical positions (bp)	SNPs	NCBI SNPdb accession IDs (NCBI_ss#)
Ca_Kabuli_NC_021163.1_8848689	Ca_kabuli_chr04	8848689	[A/C]	1067295023
Ca_Kabuli_NC_021163.1_8849693	Ca_kabuli_chr04	8849693	[A/T]	1067295024
Ca_Kabuli_NC_021163.1_8852420	Ca_kabuli_chr04	8852420	[C/T]	1067295025
Ca_Kabuli_NC_021163.1_8865427	Ca_kabuli_chr04	8865427	[A/G]	1067295026
Ca_Kabuli_NC_021163.1_8919168	Ca_kabuli_chr04	8919168	[A/T]	1067295027
Ca_Kabuli_NC_021163.1_8919205	Ca_kabuli_chr04	8919205	[A/T]	1067295028
Ca_Kabuli_NC_021163.1_8919209	Ca_kabuli_chr04	8919209	[A/G]	1067295029
Ca_Kabuli_NC_021163.1_8919223	Ca_kabuli_chr04	8919223	[T/C]	1067295030
Ca_Kabuli_NC_021163.1_8919308	Ca_kabuli_chr04	8919308	[A/C]	1067295031
Ca_Kabuli_NC_021163.1_8919332	Ca_kabuli_chr04	8919332	[A/G]	1067295032
Ca_Kabuli_NC_021163.1_8919427	Ca_kabuli_chr04	8919427	[A/C]	1067295033
Ca_Kabuli_NC_021163.1_8922803	Ca_kabuli_chr04	8922803	[A/C]	1067295034
Ca_Kabuli_NC_021163.1_8926390	Ca_kabuli_chr04	8926390	[C/A]	1067295035
Ca_Kabuli_NC_021163.1_8952820	Ca_kabuli_chr04	8952820	[T/G]	1067295036
Ca_Kabuli_NC_021163.1_8986026	Ca_kabuli_chr04	8986026	[T/A]	1067295037
Ca_Kabuli_NC_021163.1_8986274	Ca_kabuli_chr04	8986274	[A/C]	1067295038
Ca_Kabuli_NC_021163.1_9075516	Ca_kabuli_chr04	9075516	[T/C]	1067295039
Ca_Kabuli_NC_021163.1_9075538	Ca_kabuli_chr04	9075538	[T/G]	1067295040
Ca_Kabuli_NC_021163.1_9075543	Ca_kabuli_chr04	9075543	[T/G]	1067295041
Ca_Kabuli_NC_021163.1_9075565	Ca_kabuli_chr04	9075565	[A/G]	1067295042
Ca_Kabuli_NC_021163.1_9075592	Ca_kabuli_chr04	9075592	[A/G]	1067295043
Ca_Kabuli_NC_021163.1_9096853	Ca_kabuli_chr04	9096853	[A/C]	1067295044
Ca_Kabuli_NC_021163.1_9099191	Ca_kabuli_chr04	9099191	[T/G]	1067295045
Ca_Kabuli_NC_021163.1_9115267	Ca_kabuli_chr04	9115267	[T/C]	1067295046
Ca_Kabuli_NC_021163.1_9115502	Ca_kabuli_chr04	9115502	[T/C]	1067295047
Ca_Kabuli_NC_021163.1_9115563	Ca_kabuli_chr04	9115563	[A/G]	1067295048
Ca_Kabuli_NC_021163.1_9187861	Ca_kabuli_chr04	9187861	[T/C]	1067295049
Ca_Kabuli_NC_021163.1_9243289	Ca_kabuli_chr04	9243289	[A/C]	1067295050
Ca_Kabuli_NC_021163.1_9243310	Ca_kabuli_chr04	9243310	[A/T]	1067295051
Ca_Kabuli_NC_021163.1_9250479	Ca_kabuli_chr04	9250479	[A/G]	1067295052
Ca_Kabuli_NC_021163.1_9250487	Ca_kabuli_chr04	9250487	[A/C]	1067295053
Ca_Kabuli_NC_021163.1_9325185	Ca_kabuli_chr04	9325185	[T/A]	1067295054
Ca_Kabuli_NC_021163.1_9325208	Ca_kabuli_chr04	9325208	[A/C]	1067295055
Ca_Kabuli_NC_021163.1_9325219	Ca_kabuli_chr04	9325219	[T/C]	1067295056

SNP IDs	Chromosomes	Physical positions (bp)	SNPs	NCBI SNPdb accession IDs (NCBI_ss#)
Ca_Kabuli_NC_021163.1_9331930	Ca_kabuli_chr04	9331930	[A/T]	1067295057
Ca_Kabuli_NC_021163.1_9331931	Ca_kabuli_chr04	9331931	[T/A]	1067295058
Ca_Kabuli_NC_021163.1_9404962	Ca_kabuli_chr04	9404962	[A/C]	1067295059
Ca_Kabuli_NC_021163.1_9451895	Ca_kabuli_chr04	9451895	[T/A]	1067295060
Ca_Kabuli_NC_021163.1_9451905	Ca_kabuli_chr04	9451905	[T/G]	1067295061
Ca_Kabuli_NC_021163.1_9498227	Ca_kabuli_chr04	9498227	[A/C]	1067295062
Ca_Kabuli_NC_021163.1_9498228	Ca_kabuli_chr04	9498228	[C/T]	1067295063
Ca_Kabuli_NC_021163.1_9541066	Ca_kabuli_chr04	9541066	[A/C]	1067295064
Ca_Kabuli_NC_021163.1_9541081	Ca_kabuli_chr04	9541081	[T/G]	1067295065
Ca_Kabuli_NC_021163.1_9541086	Ca_kabuli_chr04	9541086	[T/C]	1067295066
Ca_Kabuli_NC_021163.1_9580315	Ca_kabuli_chr04	9580315	[T/C]	1067295067
Ca_Kabuli_NC_021163.1_9580349	Ca_kabuli_chr04	9580349	[T/C]	1067295068
Ca_Kabuli_NC_021163.1_9580351	Ca_kabuli_chr04	9580351	[A/T]	1067295069
Ca_Kabuli_NC_021163.1_9580421	Ca_kabuli_chr04	9580421	[A/G]	1067295070
Ca_Kabuli_NC_021163.1_9611920	Ca_kabuli_chr04	9611920	[T/A]	1067295071
Ca_Kabuli_NC_021163.1_9661566	Ca_kabuli_chr04	9661566	[T/A]	1067295072
Ca_Kabuli_NC_021163.1_9803227	Ca_kabuli_chr04	9803227	[C/T]	1067295073
Ca_Kabuli_NC_021163.1_9803230	Ca_kabuli_chr04	9803230	[C/T]	1067295074
Ca_Kabuli_NC_021163.1_9823199	Ca_kabuli_chr04	9823199	[T/C]	1067295075
Ca_Kabuli_NC_021163.1_9840885	Ca_kabuli_chr04	9840885	[T/C]	1067295076
Ca_Kabuli_NC_021163.1_9908004	Ca_kabuli_chr04	9908004	[A/T]	1067295077
Ca_Kabuli_NC_021163.1_9908049	Ca_kabuli_chr04	9908049	[A/T]	1067295078
Ca_Kabuli_NC_021163.1_9908096	Ca_kabuli_chr04	9908096	[T/G]	1067295079
Ca_Kabuli_NC_021163.1_9908280	Ca_kabuli_chr04	9908280	[T/C]	1067295080
Ca_Kabuli_NC_021163.1_9908289	Ca_kabuli_chr04	9908289	[T/C]	1067295081
Ca_Kabuli_NC_021163.1_9908305	Ca_kabuli_chr04	9908305	[A/T]	1067295082
Ca_Kabuli_NC_021163.1_9908336	Ca_kabuli_chr04	9908336	[A/G]	1067295083
Ca_Kabuli_NC_021163.1_9908346	Ca_kabuli_chr04	9908346	[A/G]	1067295084
Ca_Kabuli_NC_021163.1_9976026	Ca_kabuli_chr04	9976026	[A/G]	1067295085
Ca_Kabuli_NC_021163.1_9980947	Ca_kabuli_chr04	9980947	[A/G]	1067295086
Ca_Kabuli_NC_021163.1_9980957	Ca_kabuli_chr04	9980957	[A/G]	1067295087
Ca_Kabuli_NC_021163.1_9980959	Ca_kabuli_chr04	9980959	[A/C]	1067295088
Ca_Kabuli_NC_021163.1_9980970	Ca_kabuli_chr04	9980970	[T/A]	1067295089
Ca_Kabuli_NC_021163.1_10007142	Ca_kabuli_chr04	10007142	[A/G]	1067295090

SNP IDs	Chromosomes	Physical positions (bp)	SNPs	NCBI SNPdb accession IDs (NCBI_ss#)
Ca_Kabuli_NC_021163.1_10007190	Ca_kabuli_chr04	10007190	[T/A]	1067295091
Ca_Kabuli_NC_021163.1_10024207	Ca_kabuli_chr04	10024207	[T/A]	1067295092
Ca_Kabuli_NC_021163.1_10024246	Ca_kabuli_chr04	10024246	[A/G]	1067295093
Ca_Kabuli_NC_021163.1_10026849	Ca_kabuli_chr04	10026849	[A/C]	1067295094
Ca_Kabuli_NC_021163.1_10026868	Ca_kabuli_chr04	10026868	[A/G]	1067295095
Ca_Kabuli_NC_021163.1_10049947	Ca_kabuli_chr04	10049947	[T/C]	1067295096
Ca_Kabuli_NC_021163.1_10049951	Ca_kabuli_chr04	10049951	[A/C]	1067295097
Ca_Kabuli_NC_021163.1_10049963	Ca_kabuli_chr04	10049963	[A/C]	1067295098
Ca_Kabuli_NC_021163.1_10049980	Ca_kabuli_chr04	10049980	[A/G]	1067295099
Ca_Kabuli_NC_021163.1_10053932	Ca_kabuli_chr04	10053932	[A/T]	1067295100
Ca_Kabuli_NC_021163.1_10053933	Ca_kabuli_chr04	10053933	[G/C]	1067295101
Ca_Kabuli_NC_021163.1_10053934	Ca_kabuli_chr04	10053934	[G/T]	1067295102
Ca_Kabuli_NC_021163.1_10082251	Ca_kabuli_chr04	10082251	[T/C]	1067295103
Ca_Kabuli_NC_021163.1_10151639	Ca_kabuli_chr04	10151639	[T/G]	1067295104
Ca_Kabuli_NC_021163.1_10151684	Ca_kabuli_chr04	10151684	[A/C]	1067295105
Ca_Kabuli_NC_021163.1_10151688	Ca_kabuli_chr04	10151688	[A/C]	1067295106
Ca_Kabuli_NC_021163.1_10152130	Ca_kabuli_chr04	10152130	[T/G]	1067295107
Ca_Kabuli_NC_021163.1_10152160	Ca_kabuli_chr04	10152160	[A/C]	1067295108
Ca_Kabuli_NC_021163.1_10184474	Ca_kabuli_chr04	10184474	[T/G]	1067295109
Ca_Kabuli_NC_021163.1_10200521	Ca_kabuli_chr04	10200521	[C/A]	1067295110
Ca_Kabuli_NC_021163.1_10200523	Ca_kabuli_chr04	10200523	[A/C]	1067295111
Ca_Kabuli_NC_021163.1_10205223	Ca_kabuli_chr04	10205223	[T/C]	1067295112
Ca_Kabuli_NC_021163.1_10236946	Ca_kabuli_chr04	10236946	[T/C]	1067295113
Ca_Kabuli_NC_021163.1_10236955	Ca_kabuli_chr04	10236955	[T/C]	1067295114
Ca_Kabuli_NC_021163.1_10236977	Ca_kabuli_chr04	10236977	[A/C]	1067295115
Ca_Kabuli_NC_021163.1_10236987	Ca_kabuli_chr04	10236987	[A/C]	1067295116
Ca_Kabuli_NC_021163.1_10305241	Ca_kabuli_chr04	10305241	[T/A]	1067295117
Ca_Kabuli_NC_021163.1_10305261	Ca_kabuli_chr04	10305261	[A/C]	1067295118
Ca_Kabuli_NC_021163.1_10355491	Ca_kabuli_chr04	10355491	[T/C]	1067295119
Ca_Kabuli_NC_021163.1_10355514	Ca_kabuli_chr04	10355514	[A/T]	1067295120
Ca_Kabuli_NC_021163.1_10355527	Ca_kabuli_chr04	10355527	[T/A]	1067295121
Ca_Kabuli_NC_021163.1_10355547	Ca_kabuli_chr04	10355547	[A/T]	1067295122
Ca_Kabuli_NC_021163.1_10355549	Ca_kabuli_chr04	10355549	[A/C]	1067295123
Ca_Kabuli_NC_021163.1_10419430	Ca_kabuli_chr04	10419430	[T/C]	1067295124

SNP IDs	Chromosomes	Physical positions (bp)	SNPs	NCBI SNPdb accession IDs (NCBI_ss#)
Ca_Kabuli_NC_021163.1_10420290	Ca_kabuli_chr04	10420290	[T/C]	1067295125
Ca_Kabuli_NC_021163.1_10422406	Ca_kabuli_chr04	10422406	[T/C]	1067295126
Ca_Kabuli_NC_021163.1_10430193	Ca_kabuli_chr04	10430193	[A/G]	1067295127
Ca_Kabuli_NC_021163.1_10430603	Ca_kabuli_chr04	10430603	[G/A]	1067295128
Ca_Kabuli_NC_021163.1_10430613	Ca_kabuli_chr04	10430613	[C/A]	1067295129
Ca_Kabuli_NC_021163.1_10430617	Ca_kabuli_chr04	10430617	[A/C]	1067295130
Ca_Kabuli_NC_021163.1_10430622	Ca_kabuli_chr04	10430622	[A/C]	1067295131
Ca_Kabuli_NC_021163.1_10434774	Ca_kabuli_chr04	10434774	[T/A]	1067295132
Ca_Kabuli_NC_021163.1_10475828	Ca_kabuli_chr04	10475828	[A/T]	1067295133
Ca_Kabuli_NC_021163.1_10475829	Ca_kabuli_chr04	10475829	[A/C]	1067295134
Ca_Kabuli_NC_021163.1_10475830	Ca_kabuli_chr04	10475830	[G/T]	1067295135
Ca_Kabuli_NC_021163.1_10547652	Ca_kabuli_chr04	10547652	[A/T]	1067295136
Ca_Kabuli_NC_021163.1_10547674	Ca_kabuli_chr04	10547674	[A/T]	1067295137
Ca_Kabuli_NC_021163.1_10595816	Ca_kabuli_chr04	10595816	[A/C]	1067295138
Ca_Kabuli_NC_021163.1_10595824	Ca_kabuli_chr04	10595824	[A/C]	1067295139
Ca_Kabuli_NC_021163.1_10601686	Ca_kabuli_chr04	10601686	[A/C]	1067295140
Ca_Kabuli_NC_021163.1_10643322	Ca_kabuli_chr04	10643322	[G/A]	1067295141
Ca_Kabuli_NC_021163.1_10643323	Ca_kabuli_chr04	10643323	[A/G]	1067295142
Ca_Kabuli_NC_021163.1_10643425	Ca_kabuli_chr04	10643425	[A/T]	1067295143
Ca_Kabuli_NC_021163.1_10645995	Ca_kabuli_chr04	10645995	[A/T]	1067295144
Ca_Kabuli_NC_021163.1_10917698	Ca_kabuli_chr04	10917698	[A/G]	1067295145
Ca_Kabuli_NC_021163.1_10917706	Ca_kabuli_chr04	10917706	[A/T]	1067295146
Ca_Kabuli_NC_021163.1_10917734	Ca_kabuli_chr04	10917734	[A/G]	1067295147
Ca_Kabuli_NC_021163.1_10950829	Ca_kabuli_chr04	10950829	[A/C]	1067295148
Ca_Kabuli_NC_021163.1_10971334	Ca_kabuli_chr04	10971334	[A/T]	1067295149
Ca_Kabuli_NC_021163.1_10991873	Ca_kabuli_chr04	10991873	[A/C]	1067295150
Ca_Kabuli_NC_021163.1_11040063	Ca_kabuli_chr04	11040063	[T/C]	1067295151
Ca_Kabuli_NC_021163.1_11040087	Ca_kabuli_chr04	11040087	[T/G]	1067295152
Ca_Kabuli_NC_021163.1_11041726	Ca_kabuli_chr04	11041726	[A/G]	1067295153
Ca_Kabuli_NC_021163.1_11041734	Ca_kabuli_chr04	11041734	[T/C]	1067295154
Ca_Kabuli_NC_021163.1_11041765	Ca_kabuli_chr04	11041765	[T/C]	1067295155
Ca_Kabuli_NC_021163.1_11051314	Ca_kabuli_chr04	11051314	[A/C]	1067295156
Ca_Kabuli_NC_021163.1_11051318	Ca_kabuli_chr04	11051318	[T/C]	1067295157
Ca_Kabuli_NC_021163.1_11051330	Ca_kabuli_chr04	11051330	[A/G]	1067295158

SNP IDs	Chromosomes	Physical positions (bp)	SNPs	NCBI SNPdb accession IDs (NCBI_ss#)
Ca_Kabuli_NC_021163.1_11051337	Ca_kabuli_chr04	11051337	[A/C]	1067295159
Ca_Kabuli_NC_021163.1_11051349	Ca_kabuli_chr04	11051349	[A/C]	1067295160
Ca_Kabuli_NC_021163.1_11059414	Ca_kabuli_chr04	11059414	[T/G]	1067295161
Ca_Kabuli_NC_021163.1_11111630	Ca_kabuli_chr04	11111630	[T/G]	1067295162
Ca_Kabuli_NC_021163.1_11111662	Ca_kabuli_chr04	11111662	[T/G]	1067295163
Ca_Kabuli_NC_021163.1_11113204	Ca_kabuli_chr04	11113204	[T/G]	1067295164
Ca_Kabuli_NC_021163.1_11113225	Ca_kabuli_chr04	11113225	[G/T]	1067295165
Ca_Kabuli_NC_021163.1_11114238	Ca_kabuli_chr04	11114238	[T/C]	1067295166
Ca_Kabuli_NC_021163.1_11114250	Ca_kabuli_chr04	11114250	[T/C]	1067295167
Ca_Kabuli_NC_021163.1_11142264	Ca_kabuli_chr04	11142264	[A/G]	1067295168
Ca_Kabuli_NC_021163.1_11196786	Ca_kabuli_chr04	11196786	[A/C]	1067295169
Ca_Kabuli_NC_021163.1_11212154	Ca_kabuli_chr04	11212154	[T/C]	1067295170
Ca_Kabuli_NC_021163.1_11212351	Ca_kabuli_chr04	11212351	[C/T]	1067295171
Ca_Kabuli_NC_021163.1_11230372	Ca_kabuli_chr04	11230372	[A/C]	1067295172
Ca_Kabuli_NC_021163.1_11230387	Ca_kabuli_chr04	11230387	[A/C]	1067295173
Ca_Kabuli_NC_021163.1_11230431	Ca_kabuli_chr04	11230431	[A/C]	1067295174
Ca_Kabuli_NC_021163.1_11230443	Ca_kabuli_chr04	11230443	[A/G]	1067295175
Ca_Kabuli_NC_021163.1_11231056	Ca_kabuli_chr04	11231056	[T/C]	1067295176
Ca_Kabuli_NC_021163.1_11231079	Ca_kabuli_chr04	11231079	[A/G]	1067295177
Ca_Kabuli_NC_021163.1_11231087	Ca_kabuli_chr04	11231087	[T/C]	1067295178
Ca_Kabuli_NC_021163.1_11231129	Ca_kabuli_chr04	11231129	[T/A]	1067295179
Ca_Kabuli_NC_021163.1_11231131	Ca_kabuli_chr04	11231131	[A/G]	1067295180
Ca_Kabuli_NC_021163.1_11231135	Ca_kabuli_chr04	11231135	[T/C]	1067295181
Ca_Kabuli_NC_021163.1_11231137	Ca_kabuli_chr04	11231137	[G/A]	1067295182
Ca_Kabuli_NC_021163.1_11231142	Ca_kabuli_chr04	11231142	[G/A]	1067295183
Ca_Kabuli_NC_021163.1_11231162	Ca_kabuli_chr04	11231162	[A/G]	1067295184
Ca_Kabuli_NC_021163.1_11231185	Ca_kabuli_chr04	11231185	[T/C]	1067295185
Ca_Kabuli_NC_021163.1_11243872	Ca_kabuli_chr04	11243872	[T/C]	1067295186
Ca_Kabuli_NC_021163.1_11243888	Ca_kabuli_chr04	11243888	[G/A]	1067295187
Ca_Kabuli_NC_021163.1_11244334	Ca_kabuli_chr04	11244334	[G/T]	1067295188
Ca_Kabuli_NC_021163.1_11244395	Ca_kabuli_chr04	11244395	[T/G]	1067295189
Ca_Kabuli_NC_021163.1_11246093	Ca_kabuli_chr04	11246093	[A/G]	1067295190
Ca_Kabuli_NC_021163.1_11246164	Ca_kabuli_chr04	11246164	[A/G]	1067295191
Ca_Kabuli_NC_021163.1_11246173	Ca_kabuli_chr04	11246173	[T/C]	1067295192

SNP IDs	Chromosomes	Physical positions (bp)	SNPs	NCBI SNPdb accession IDs (NCBI_ss#)
Ca_Kabuli_NC_021163.1_11248605	Ca_kabuli_chr04	11248605	[A/T]	1067295193
Ca_Kabuli_NC_021163.1_11257588	Ca_kabuli_chr04	11257588	[C/T]	1067295194
Ca_Kabuli_NC_021163.1_11272888	Ca_kabuli_chr04	11272888	[C/T]	1067295195
Ca_Kabuli_NC_021163.1_11272904	Ca_kabuli_chr04	11272904	[C/A]	1067295196
Ca_Kabuli_NC_021163.1_11273328	Ca_kabuli_chr04	11273328	[T/C]	1067295197
Ca_Kabuli_NC_021163.1_11273405	Ca_kabuli_chr04	11273405	[T/A]	1067295198
Ca_Kabuli_NC_021163.1_11274281	Ca_kabuli_chr04	11274281	[C/T]	1067295199
Ca_Kabuli_NC_021163.1_11276140	Ca_kabuli_chr04	11276140	[A/G]	1067295200
Ca_Kabuli_NC_021163.1_11276166	Ca_kabuli_chr04	11276166	[A/G]	1067295201
Ca_Kabuli_NC_021163.1_11276186	Ca_kabuli_chr04	11276186	[T/C]	1067295202
Ca_Kabuli_NC_021163.1_11276225	Ca_kabuli_chr04	11276225	[T/C]	1067295203
Ca_Kabuli_NC_021163.1_11276413	Ca_kabuli_chr04	11276413	[C/G]	1067295204
Ca_Kabuli_NC_021163.1_11277138	Ca_kabuli_chr04	11277138	[G/A]	1067295205
Ca_Kabuli_NC_021163.1_11277513	Ca_kabuli_chr04	11277513	[T/C]	1067295206
Ca_Kabuli_NC_021163.1_11277574	Ca_kabuli_chr04	11277574	[C/G]	1067295207
Ca_Kabuli_NC_021163.1_11305651	Ca_kabuli_chr04	11305651	[T/A]	1067295208
Ca_Kabuli_NC_021163.1_11328227	Ca_kabuli_chr04	11328227	[G/T]	1067295209
Ca_Kabuli_NC_021163.1_11333394	Ca_kabuli_chr04	11333394	[G/C]	1067295210
Ca_Kabuli_NC_021163.1_11343230	Ca_kabuli_chr04	11343230	[C/G]	1067295211
Ca_Kabuli_NC_021163.1_11343257	Ca_kabuli_chr04	11343257	[C/T]	1067295212
Ca_Kabuli_NC_021163.1_11398889	Ca_kabuli_chr04	11398889	[G/A]	1067295213
Ca_Kabuli_NC_021163.1_11410315	Ca_kabuli_chr04	11410315	[A/C]	1067295214
Ca_Kabuli_NC_021163.1_11414502	Ca_kabuli_chr04	11414502	[A/C]	1067295215
Ca_Kabuli_NC_021163.1_11414537	Ca_kabuli_chr04	11414537	[A/T]	1067295216
Ca_Kabuli_NC_021163.1_11435253	Ca_kabuli_chr04	11435253	[G/T]	1067295217
Ca_Kabuli_NC_021163.1_11435255	Ca_kabuli_chr04	11435255	[A/G]	1067295218
Ca_Kabuli_NC_021163.1_11435380	Ca_kabuli_chr04	11435380	[T/C]	1067295219
Ca_Kabuli_NC_021163.1_11435651	Ca_kabuli_chr04	11435651	[A/G]	1067295220
Ca_Kabuli_NC_021163.1_11440109	Ca_kabuli_chr04	11440109	[T/C]	1067295221
Ca_Kabuli_NC_021163.1_11441604	Ca_kabuli_chr04	11441604	[A/T]	1067295222
Ca_Kabuli_NC_021163.1_11441673	Ca_kabuli_chr04	11441673	[A/G]	1067295223
Ca_Kabuli_NC_021163.1_11441735	Ca_kabuli_chr04	11441735	[T/G]	1067295224
Ca_Kabuli_NC_021163.1_11465057	Ca_kabuli_chr04	11465057	[G/C]	1067295225
Ca_Kabuli_NC_021163.1_11465075	Ca_kabuli_chr04	11465075	[C/T]	1067295226

SNP IDs	Chromosomes	Physical positions (bp)	SNPs	NCBI SNPdb accession IDs (NCBI_ss#)
Ca_Kabuli_NC_021163.1_11490100	Ca_kabuli_chr04	11490100	[T/C]	1067295227
Ca_Kabuli_NC_021163.1_11490155	Ca_kabuli_chr04	11490155	[A/G]	1067295228
Ca_Kabuli_NC_021163.1_11490159	Ca_kabuli_chr04	11490159	[A/G]	1067295229
Ca_Kabuli_NC_021163.1_11490192	Ca_kabuli_chr04	11490192	[A/G]	1067295230
Ca_Kabuli_NC_021163.1_11490496	Ca_kabuli_chr04	11490496	[G/A]	1067295231
Ca_Kabuli_NC_021163.1_11491021	Ca_kabuli_chr04	11491021	[G/A]	1067295232
Ca_Kabuli_NC_021163.1_11491864	Ca_kabuli_chr04	11491864	[C/A]	1067295233
Ca_Kabuli_NC_021163.1_11517517	Ca_kabuli_chr04	11517517	[A/C]	1067295234
Ca_Kabuli_NC_021163.1_11517565	Ca_kabuli_chr04	11517565	[A/T]	1067295235
Ca_Kabuli_NC_021163.1_11547141	Ca_kabuli_chr04	11547141	[A/C]	1067295236
Ca_Kabuli_NC_021163.1_11645662	Ca_kabuli_chr04	11645662	[A/G]	1067295237
Ca_Kabuli_NC_021163.1_11645683	Ca_kabuli_chr04	11645683	[A/G]	1067295238
Ca_Kabuli_NC_021163.1_11645733	Ca_kabuli_chr04	11645733	[A/G]	1067295239
Ca_Kabuli_NC_021163.1_11645828	Ca_kabuli_chr04	11645828	[T/G]	1067295240
Ca_Kabuli_NC_021163.1_11646345	Ca_kabuli_chr04	11646345	[A/C]	1067295241
Ca_Kabuli_NC_021163.1_11648201	Ca_kabuli_chr04	11648201	[A/C]	1067295242
Ca_Kabuli_NC_021163.1_11745632	Ca_kabuli_chr04	11745632	[A/G]	1067295243
Ca_Kabuli_NC_021163.1_11771986	Ca_kabuli_chr04	11771986	[A/T]	1067295244
Ca_Kabuli_NC_021163.1_11772008	Ca_kabuli_chr04	11772008	[A/C]	1067295245
Ca_Kabuli_NC_021163.1_11772025	Ca_kabuli_chr04	11772025	[T/G]	1067295246
Ca_Kabuli_NC_021163.1_11825176	Ca_kabuli_chr04	11825176	[T/G]	1067295247
Ca_Kabuli_NC_021163.1_11923283	Ca_kabuli_chr04	11923283	[A/G]	1067295248
Ca_Kabuli_NC_021163.1_11923288	Ca_kabuli_chr04	11923288	[A/G]	1067295249
Ca_Kabuli_NC_021163.1_11951124	Ca_kabuli_chr04	11951124	[T/G]	1067295250
Ca_Kabuli_NC_021163.1_11951399	Ca_kabuli_chr04	11951399	[T/C]	1067295251
Ca_Kabuli_NC_021163.1_11951415	Ca_kabuli_chr04	11951415	[A/C]	1067295252
Ca_Kabuli_NC_021163.1_11985461	Ca_kabuli_chr04	11985461	[A/C]	1067295253
Ca_Kabuli_NC_021163.1_11985676	Ca_kabuli_chr04	11985676	[T/C]	1067295254
Ca_Kabuli_NC_021163.1_11985713	Ca_kabuli_chr04	11985713	[T/C]	1067295255
Ca_Kabuli_NC_021163.1_11985720	Ca_kabuli_chr04	11985720	[A/G]	1067295256
Ca_Kabuli_NC_021163.1_12004205	Ca_kabuli_chr04	12004205	[A/C]	1067295257
Ca_Kabuli_NC_021163.1_12004209	Ca_kabuli_chr04	12004209	[C/T]	1067295258
Ca_Kabuli_NC_021163.1_12156799	Ca_kabuli_chr04	12156799	[T/C]	1067295259
Ca_Kabuli_NC_021163.1_12156812	Ca_kabuli_chr04	12156812	[T/C]	1067295260

SNP IDs	Chromosomes	Physical positions (bp)	SNPs	NCBI SNPdb accession IDs (NCBI_ss#)
Ca_Kabuli_NC_021163.1_12156821	Ca_kabuli_chr04	12156821	[A/G]	1067295261
Ca_Kabuli_NC_021163.1_12156835	Ca_kabuli_chr04	12156835	[G/A]	1067295262
Ca_Kabuli_NC_021163.1_12156840	Ca_kabuli_chr04	12156840	[T/G]	1067295263
Ca_Kabuli_NC_021163.1_12156855	Ca_kabuli_chr04	12156855	[T/A]	1067295264
Ca_Kabuli_NC_021163.1_12162780	Ca_kabuli_chr04	12162780	[A/G]	1067295265
Ca_Kabuli_NC_021163.1_12162794	Ca_kabuli_chr04	12162794	[T/C]	1067295266
Ca_Kabuli_NC_021163.1_12162806	Ca_kabuli_chr04	12162806	[A/G]	1067295267
Ca_Kabuli_NC_021163.1_12167568	Ca_kabuli_chr04	12167568	[A/G]	1067295268
Ca_Kabuli_NC_021163.1_12170567	Ca_kabuli_chr04	12170567	[C/A]	1067295269
Ca_Kabuli_NC_021163.1_12170568	Ca_kabuli_chr04	12170568	[T/G]	1067295270
Ca_Kabuli_NC_021163.1_12175299	Ca_kabuli_chr04	12175299	[A/C]	1067295271
Ca_Kabuli_NC_021163.1_12175301	Ca_kabuli_chr04	12175301	[A/T]	1067295272
Ca_Kabuli_NC_021163.1_12175302	Ca_kabuli_chr04	12175302	[G/T]	1067295273
Ca_Kabuli_NC_021163.1_12249389	Ca_kabuli_chr04	12249389	[T/C]	1067295274
Ca_Kabuli_NC_021163.1_12249407	Ca_kabuli_chr04	12249407	[A/G]	1067295275
Ca_Kabuli_NC_021163.1_12249474	Ca_kabuli_chr04	12249474	[T/G]	1067295276
Ca_Kabuli_NC_021163.1_12305192	Ca_kabuli_chr04	12305192	[T/A]	1067295277
Ca_Kabuli_NC_021163.1_12305208	Ca_kabuli_chr04	12305208	[C/A]	1067295278
Ca_Kabuli_NC_021163.1_12305216	Ca_kabuli_chr04	12305216	[A/C]	1067295279
Ca_Kabuli_NC_021163.1_12305234	Ca_kabuli_chr04	12305234	[A/C]	1067295280
Ca_Kabuli_NC_021163.1_12316617	Ca_kabuli_chr04	12316617	[G/A]	1067295281
Ca_Kabuli_NC_021163.1_12431831	Ca_kabuli_chr04	12431831	[T/G]	1067295282
Ca_Kabuli_NC_021163.1_12431864	Ca_kabuli_chr04	12431864	[T/G]	1067295283
Ca_Kabuli_NC_021163.1_12462635	Ca_kabuli_chr04	12462635	[A/G]	1067295284
Ca_Kabuli_NC_021163.1_12462644	Ca_kabuli_chr04	12462644	[C/T]	1067295285
Ca_Kabuli_NC_021163.1_12484911	Ca_kabuli_chr04	12484911	[A/T]	1067295286
Ca_Kabuli_NC_021163.1_12490280	Ca_kabuli_chr04	12490280	[G/A]	1067295287
Ca_Kabuli_NC_021163.1_12506450	Ca_kabuli_chr04	12506450	[T/A]	1067295288
Ca_Kabuli_NC_021163.1_12525328	Ca_kabuli_chr04	12525328	[G/A]	1067295289
Ca_Kabuli_NC_021163.1_12525639	Ca_kabuli_chr04	12525639	[T/A]	1067295290
Ca_Kabuli_NC_021163.1_12526606	Ca_kabuli_chr04	12526606	[G/T]	1067295291
Ca_Kabuli_NC_021163.1_12533089	Ca_kabuli_chr04	12533089	[A/G]	1067295292
Ca_Kabuli_NC_021163.1_12534847	Ca_kabuli_chr04	12534847	[C/T]	1067295293
Ca_Kabuli_NC_021163.1_12558541	Ca_kabuli_chr04	12558541	[C/T]	1067295294

SNP IDs	Chromosomes	Physical positions (bp)	SNPs	NCBI SNPdb accession IDs (NCBI_ss#)
Ca_Kabuli_NC_021163.1_12558680	Ca_kabuli_chr04	12558680	[C/T]	1067295295
Ca_Kabuli_NC_021163.1_12568599	Ca_kabuli_chr04	12568599	[A/G]	1067295296
Ca_Kabuli_NC_021163.1_12568602	Ca_kabuli_chr04	12568602	[C/T]	1067295297
Ca_Kabuli_NC_021163.1_12568707	Ca_kabuli_chr04	12568707	[A/G]	1067295298
Ca_Kabuli_NC_021163.1_12568845	Ca_kabuli_chr04	12568845	[G/T]	1067295299
Ca_Kabuli_NC_021163.1_12568905	Ca_kabuli_chr04	12568905	[G/A]	1067295300
Ca_Kabuli_NC_021163.1_12578606	Ca_kabuli_chr04	12578606	[A/T]	1067295301
Ca_Kabuli_NC_021163.1_12578627	Ca_kabuli_chr04	12578627	[T/C]	1067295302
Ca_Kabuli_NC_021163.1_12581371	Ca_kabuli_chr04	12581371	[G/T]	1067295303
Ca_Kabuli_NC_021163.1_12581403	Ca_kabuli_chr04	12581403	[A/G]	1067295304
Ca_Kabuli_NC_021163.1_12581430	Ca_kabuli_chr04	12581430	[A/C]	1067295305
Ca_Kabuli_NC_021163.1_12581457	Ca_kabuli_chr04	12581457	[A/G]	1067295306
Ca_Kabuli_NC_021163.1_12581461	Ca_kabuli_chr04	12581461	[A/G]	1067295307
Ca_Kabuli_NC_021163.1_12581711	Ca_kabuli_chr04	12581711	[T/G]	1067295308
Ca_Kabuli_NC_021163.1_12609670	Ca_kabuli_chr04	12609670	[A/C]	1067295309
Ca_Kabuli_NC_021163.1_12609673	Ca_kabuli_chr04	12609673	[C/G]	1067295310
Ca_Kabuli_NC_021163.1_12661547	Ca_kabuli_chr04	12661547	[A/G]	1067295311
Ca_Kabuli_NC_021163.1_12661585	Ca_kabuli_chr04	12661585	[A/G]	1067295312
Ca_Kabuli_NC_021163.1_12706307	Ca_kabuli_chr04	12706307	[T/G]	1067295313
Ca_Kabuli_NC_021163.1_12740322	Ca_kabuli_chr04	12740322	[T/G]	1067295314
Ca_Kabuli_NC_021163.1_12744974	Ca_kabuli_chr04	12744974	[T/C]	1067295315
Ca_Kabuli_NC_021163.1_12744984	Ca_kabuli_chr04	12744984	[T/G]	1067295316
Ca_Kabuli_NC_021163.1_12745003	Ca_kabuli_chr04	12745003	[T/G]	1067295317
Ca_Kabuli_NC_021163.1_12745010	Ca_kabuli_chr04	12745010	[A/G]	1067295318
Ca_Kabuli_NC_021163.1_12854305	Ca_kabuli_chr04	12854305	[T/G]	1067295319
Ca_Kabuli_NC_021163.1_12854341	Ca_kabuli_chr04	12854341	[T/C]	1067295320
Ca_Kabuli_NC_021163.1_12869088	Ca_kabuli_chr04	12869088	[A/T]	1067295321
Ca_Kabuli_NC_021163.1_12879612	Ca_kabuli_chr04	12879612	[T/G]	1067295322
Ca_Kabuli_NC_021163.1_12879635	Ca_kabuli_chr04	12879635	[T/G]	1067295323
Ca_Kabuli_NC_021163.1_12879671	Ca_kabuli_chr04	12879671	[A/C]	1067295324
Ca_Kabuli_NC_021163.1_12949212	Ca_kabuli_chr04	12949212	[A/G]	1067295325
Ca_Kabuli_NC_021163.1_12973591	Ca_kabuli_chr04	12973591	[A/T]	1067295326
Ca_Kabuli_NC_021163.1_13018762	Ca_kabuli_chr04	13018762	[T/G]	1067295327
Ca_Kabuli_NC_021163.1_13018807	Ca_kabuli_chr04	13018807	[A/G]	1067295328

SNP IDs	Chromosomes	Physical positions (bp)	SNPs	NCBI SNPdb accession IDs (NCBI_ss#)
Ca_Kabuli_NC_021163.1_13063677	Ca_kabuli_chr04	13063677	[T/C]	1067295329
Ca_Kabuli_NC_021163.1_13063682	Ca_kabuli_chr04	13063682	[T/C]	1067295330
Ca_Kabuli_NC_021163.1_13063695	Ca_kabuli_chr04	13063695	[T/C]	1067295331
Ca_Kabuli_NC_021163.1_13063715	Ca_kabuli_chr04	13063715	[G/A]	1067295332
Ca_Kabuli_NC_021163.1_13063722	Ca_kabuli_chr04	13063722	[A/C]	1067295333
Ca_Kabuli_NC_021163.1_13063788	Ca_kabuli_chr04	13063788	[T/G]	1067295334
Ca_Kabuli_NC_021163.1_13063818	Ca_kabuli_chr04	13063818	[A/T]	1067295335
Ca_Kabuli_NC_021163.1_13071325	Ca_kabuli_chr04	13071325	[T/G]	1067295336
Ca_Kabuli_NC_021163.1_13072102	Ca_kabuli_chr04	13072102	[T/C]	1067295337
Ca_Kabuli_NC_021163.1_13072107	Ca_kabuli_chr04	13072107	[T/C]	1067295338
Ca_Kabuli_NC_021163.1_13072147	Ca_kabuli_chr04	13072147	[A/C]	1067295339
Ca_Kabuli_NC_021163.1_13072243	Ca_kabuli_chr04	13072243	[A/T]	1067295340
Ca_Kabuli_NC_021163.1_13103650	Ca_kabuli_chr04	13103650	[T/G]	1067295341
Ca_Kabuli_NC_021163.1_13282984	Ca_kabuli_chr04	13282984	[A/C]	1067295342
Ca_Kabuli_NC_021163.1_13283002	Ca_kabuli_chr04	13283002	[T/G]	1067295343
Ca_Kabuli_NC_021163.1_13283013	Ca_kabuli_chr04	13283013	[C/A]	1067295344
Ca_Kabuli_NC_021163.1_13296229	Ca_kabuli_chr04	13296229	[A/G]	1067295345
Ca_Kabuli_NC_021163.1_13296553	Ca_kabuli_chr04	13296553	[T/G]	1067295346
Ca_Kabuli_NC_021163.1_13297343	Ca_kabuli_chr04	13297343	[T/A]	1067295347
Ca_Kabuli_NC_021163.1_13297360	Ca_kabuli_chr04	13297360	[A/G]	1067295348
Ca_Kabuli_NC_021163.1_13376268	Ca_kabuli_chr04	13376268	[A/T]	1067295349
Ca_Kabuli_NC_021163.1_13376279	Ca_kabuli_chr04	13376279	[T/C]	1067295350
Ca_Kabuli_NC_021163.1_13376289	Ca_kabuli_chr04	13376289	[A/C]	1067295351
Ca_Kabuli_NC_021163.1_13376308	Ca_kabuli_chr04	13376308	[A/C]	1067295352
Ca_Kabuli_NC_021163.1_13376323	Ca_kabuli_chr04	13376323	[A/C]	1067295353
Ca_Kabuli_NC_021163.1_13376456	Ca_kabuli_chr04	13376456	[A/G]	1067295354
Ca_Kabuli_NC_021163.1_13406799	Ca_kabuli_chr04	13406799	[A/C]	1067295355
Ca_Kabuli_NC_021163.1_13414172	Ca_kabuli_chr04	13414172	[T/G]	1067295356
Ca_Kabuli_NC_021163.1_13462251	Ca_kabuli_chr04	13462251	[T/C]	1067295357
Ca_Kabuli_NC_021163.1_13462253	Ca_kabuli_chr04	13462253	[C/T]	1067295358
Ca_Kabuli_NC_021163.1_13500661	Ca_kabuli_chr04	13500661	[A/C]	1067295359
Ca_Kabuli_NC_021163.1_13500667	Ca_kabuli_chr04	13500667	[C/T]	1067295360
Ca_Kabuli_NC_021163.1_13500833	Ca_kabuli_chr04	13500833	[A/C]	1067295361
Ca_Kabuli_NC_021163.1_13500834	Ca_kabuli_chr04	13500834	[G/C]	1067295362

SNP IDs	Chromosomes	Physical positions (bp)	SNPs	NCBI SNPdb accession IDs (NCBI_ss#)
Ca_Kabuli_NC_021163.1_13500835	Ca_kabuli_chr04	13500835	[A/C]	1067295363
Ca_Kabuli_NC_021163.1_13566612	Ca_kabuli_chr04	13566612	[G/C]	1067295364
Ca_Kabuli_NC_021163.1_13566613	Ca_kabuli_chr04	13566613	[G/T]	1067295365
Ca_Kabuli_NC_021163.1_13587985	Ca_kabuli_chr04	13587985	[G/T]	1067295366
Ca_Kabuli_NC_021163.1_13588874	Ca_kabuli_chr04	13588874	[T/G]	1067295367
Ca_Kabuli_NC_021163.1_13588928	Ca_kabuli_chr04	13588928	[C/T]	1067295368
Ca_Kabuli_NC_021163.1_13588950	Ca_kabuli_chr04	13588950	[A/T]	1067295369
Ca_Kabuli_NC_021163.1_13588951	Ca_kabuli_chr04	13588951	[T/C]	1067295370
Ca_Kabuli_NC_021163.1_13588952	Ca_kabuli_chr04	13588952	[G/T]	1067295371
Ca_Kabuli_NC_021163.1_13588956	Ca_kabuli_chr04	13588956	[T/C]	1067295372
Ca_Kabuli_NC_021163.1_13589022	Ca_kabuli_chr04	13589022	[A/G]	1067295373
Ca_Kabuli_NC_021163.1_13589070	Ca_kabuli_chr04	13589070	[T/C]	1067295374
Ca_Kabuli_NC_021163.1_13589074	Ca_kabuli_chr04	13589074	[A/G]	1067295375
Ca_Kabuli_NC_021163.1_13641148	Ca_kabuli_chr04	13641148	[A/C]	1067295376
Ca_Kabuli_NC_021163.1_13643245	Ca_kabuli_chr04	13643245	[A/G]	1067295377
Ca_Kabuli_NC_021163.1_13646680	Ca_kabuli_chr04	13646680	[A/C]	1067295378
Ca_Kabuli_NC_021163.1_13646739	Ca_kabuli_chr04	13646739	[T/A]	1067295379
Ca_Kabuli_NC_021163.1_13646753	Ca_kabuli_chr04	13646753	[A/G]	1067295380
Ca_Kabuli_NC_021163.1_13646756	Ca_kabuli_chr04	13646756	[A/C]	1067295381
Ca_Kabuli_NC_021163.1_13646762	Ca_kabuli_chr04	13646762	[A/G]	1067295382
Ca_Kabuli_NC_021163.1_13646773	Ca_kabuli_chr04	13646773	[A/G]	1067295383
Ca_Kabuli_NC_021163.1_13660846	Ca_kabuli_chr04	13660846	[A/G]	1067295384
Ca_Kabuli_NC_021163.1_13687456	Ca_kabuli_chr04	13687456	[A/G]	1067295385
Ca_Kabuli_NC_021163.1_13687728	Ca_kabuli_chr04	13687728	[T/C]	1067295386
Ca_Kabuli_NC_021163.1_13697850	Ca_kabuli_chr04	13697850	[A/T]	1067295387
Ca_Kabuli_NC_021163.1_13697853	Ca_kabuli_chr04	13697853	[G/A]	1067295388
Ca_Kabuli_NC_021163.1_13697894	Ca_kabuli_chr04	13697894	[T/A]	1067295389
Ca_Kabuli_NC_021163.1_13699663	Ca_kabuli_chr04	13699663	[A/G]	1067295390
Ca_Kabuli_NC_021163.1_13702641	Ca_kabuli_chr04	13702641	[A/G]	1067295391
Ca_Kabuli_NC_021163.1_13704352	Ca_kabuli_chr04	13704352	[A/G]	1067295392
Ca_Kabuli_NC_021163.1_13713367	Ca_kabuli_chr04	13713367	[G/C]	1067295393
Ca_Kabuli_NC_021163.1_13713380	Ca_kabuli_chr04	13713380	[G/T]	1067295394
Ca_Kabuli_NC_021163.1_13717261	Ca_kabuli_chr04	13717261	[G/A]	1067295395
Ca_Kabuli_NC_021163.1_13724666	Ca_kabuli_chr04	13724666	[C/G]	1067295396

SNP IDs	Chromosomes	Physical positions (bp)	SNPs	NCBI SNPdb accession IDs (NCBI_ss#)
Ca_Kabuli_NC_021163.1_13726718	Ca_kabuli_chr04	13726718	[T/G]	1067295397
Ca_Kabuli_NC_021163.1_13742737	Ca_kabuli_chr04	13742737	[A/G]	1067295398
Ca_Kabuli_NC_021163.1_13742744	Ca_kabuli_chr04	13742744	[A/C]	1067295399
Ca_Kabuli_NC_021163.1_13742754	Ca_kabuli_chr04	13742754	[A/T]	1067295400
Ca_Kabuli_NC_021163.1_13758029	Ca_kabuli_chr04	13758029	[T/A]	1067295401
Ca_Kabuli_NC_021163.1_13773428	Ca_kabuli_chr04	13773428	[T/C]	1067295402
Ca_Kabuli_NC_021163.1_13787353	Ca_kabuli_chr04	13787353	[C/T]	1067295403
Ca_Kabuli_NC_021163.1_13787448	Ca_kabuli_chr04	13787448	[C/A]	1067295404
Ca_Kabuli_NC_021163.1_13787649	Ca_kabuli_chr04	13787649	[C/T]	1067295405
Ca_Kabuli_NC_021163.1_13787770	Ca_kabuli_chr04	13787770	[C/A]	1067295406
Ca_Kabuli_NC_021163.1_13787775	Ca_kabuli_chr04	13787775	[G/A]	1067295407
Ca_Kabuli_NC_021163.1_13838126	Ca_kabuli_chr04	13838126	[T/A]	1067295408
Ca_Kabuli_NC_021163.1_13838172	Ca_kabuli_chr04	13838172	[A/C]	1067295409
Ca_Kabuli_NC_021163.1_13838222	Ca_kabuli_chr04	13838222	[A/T]	1067295410
Ca_Kabuli_NC_021163.1_13838240	Ca_kabuli_chr04	13838240	[T/G]	1067295411
Ca_Kabuli_NC_021163.1_13838278	Ca_kabuli_chr04	13838278	[T/G]	1067295412
Ca_Kabuli_NC_021163.1_13838625	Ca_kabuli_chr04	13838625	[T/A]	1067295413
Ca_Kabuli_NC_021163.1_13838656	Ca_kabuli_chr04	13838656	[A/C]	1067295414
Ca_Kabuli_NC_021163.1_13838784	Ca_kabuli_chr04	13838784	[T/C]	1067295415
Ca_Kabuli_NC_021163.1_13838796	Ca_kabuli_chr04	13838796	[G/A]	1067295416
Ca_Kabuli_NC_021163.1_13838932	Ca_kabuli_chr04	13838932	[T/G]	1067295417
Ca_Kabuli_NC_021163.1_13839288	Ca_kabuli_chr04	13839288	[A/G]	1067295418
Ca_Kabuli_NC_021163.1_13839294	Ca_kabuli_chr04	13839294	[G/A]	1067295419
Ca_Kabuli_NC_021163.1_13839804	Ca_kabuli_chr04	13839804	[T/G]	1067295420
Ca_Kabuli_NC_021163.1_13839825	Ca_kabuli_chr04	13839825	[A/C]	1067295421
Ca_Kabuli_NC_021163.1_13839840	Ca_kabuli_chr04	13839840	[A/C]	1067295422
Ca_Kabuli_NC_021163.1_13839935	Ca_kabuli_chr04	13839935	[T/G]	1067295423
Ca_Kabuli_NC_021163.1_13839938	Ca_kabuli_chr04	13839938	[T/A]	1067295424
Ca_Kabuli_NC_021163.1_13840191	Ca_kabuli_chr04	13840191	[A/T]	1067295425
Ca_Kabuli_NC_021163.1_13840227	Ca_kabuli_chr04	13840227	[A/C]	1067295426
Ca_Kabuli_NC_021163.1_13840230	Ca_kabuli_chr04	13840230	[T/C]	1067295427
Ca_Kabuli_NC_021163.1_13840233	Ca_kabuli_chr04	13840233	[A/G]	1067295428
Ca_Kabuli_NC_021163.1_13840244	Ca_kabuli_chr04	13840244	[A/G]	1067295429
Ca_Kabuli_NC_021163.1_13840288	Ca_kabuli_chr04	13840288	[A/G]	1067295430

SNP IDs	Chromosomes	Physical positions (bp)	SNPs	NCBI SNPdb accession IDs (NCBI_ss#)
Ca_Kabuli_NC_021163.1_13840484	Ca_kabuli_chr04	13840484	[C/T]	1067295431
Ca_Kabuli_NC_021163.1_13841279	Ca_kabuli_chr04	13841279	[A/C]	1067295432
Ca_Kabuli_NC_021163.1_13841340	Ca_kabuli_chr04	13841340	[T/G]	1067295433
Ca_Kabuli_NC_021163.1_13845581	Ca_kabuli_chr04	13845581	[T/A]	1067295434
Ca_Kabuli_NC_021163.1_13845620	Ca_kabuli_chr04	13845620	[C/A]	1067295435
Ca_Kabuli_NC_021163.1_13858972	Ca_kabuli_chr04	13858972	[G/A]	1067295436
Ca_Kabuli_NC_021163.1_13873036	Ca_kabuli_chr04	13873036	[A/G]	1067295437
Ca_Kabuli_NC_021163.1_13873042	Ca_kabuli_chr04	13873042	[A/G]	1067295438
Ca_Kabuli_NC_021163.1_13884235	Ca_kabuli_chr04	13884235	[T/G]	1067295439
Ca_Kabuli_NC_021163.1_13941139	Ca_kabuli_chr04	13941139	[T/C]	1067295440
Ca_Kabuli_NC_021163.1_13941148	Ca_kabuli_chr04	13941148	[T/C]	1067295441
Ca_Kabuli_NC_021163.1_13941162	Ca_kabuli_chr04	13941162	[C/A]	1067295442
Ca_Kabuli_NC_021163.1_13941176	Ca_kabuli_chr04	13941176	[A/T]	1067295443
Ca_Kabuli_NC_021163.1_13949558	Ca_kabuli_chr04	13949558	[C/T]	1067295444
Ca_Kabuli_NC_021163.1_13949567	Ca_kabuli_chr04	13949567	[A/T]	1067295445
Ca_Kabuli_NC_021163.1_13970609	Ca_kabuli_chr04	13970609	[T/A]	1067295446
Ca_Kabuli_NC_021163.1_13970649	Ca_kabuli_chr04	13970649	[A/C]	1067295447
Ca_Kabuli_NC_021163.1_13970661	Ca_kabuli_chr04	13970661	[T/C]	1067295448
Ca_Kabuli_NC_021163.1_14056922	Ca_kabuli_chr04	14056922	[A/C]	1067295449
Ca_Kabuli_NC_021163.1_14056927	Ca_kabuli_chr04	14056927	[T/G]	1067295450
Ca_Kabuli_NC_021163.1_14082077	Ca_kabuli_chr04	14082077	[T/C]	1067295451
Ca_Kabuli_NC_021163.1_14082094	Ca_kabuli_chr04	14082094	[T/A]	1067295452
Ca_Kabuli_NC_021163.1_14104005	Ca_kabuli_chr04	14104005	[T/A]	1067295453
Ca_Kabuli_NC_021163.1_14104032	Ca_kabuli_chr04	14104032	[T/C]	1067295454
Ca_Kabuli_NC_021163.1_14104076	Ca_kabuli_chr04	14104076	[A/G]	1067295455
Ca_Kabuli_NC_021163.1_14104131	Ca_kabuli_chr04	14104131	[T/G]	1067295456
Ca_Kabuli_NC_021163.1_14132752	Ca_kabuli_chr04	14132752	[T/A]	1067295457
Ca_Kabuli_NC_021163.1_14132760	Ca_kabuli_chr04	14132760	[C/A]	1067295458
Ca_Kabuli_NC_021163.1_14181644	Ca_kabuli_chr04	14181644	[A/C]	1067295459
Ca_Kabuli_NC_021163.1_14199386	Ca_kabuli_chr04	14199386	[A/C]	1067295460
Ca_Kabuli_NC_021163.1_14206908	Ca_kabuli_chr04	14206908	[C/T]	1067295461
Ca_Kabuli_NC_021163.1_14206957	Ca_kabuli_chr04	14206957	[G/C]	1067295462
Ca_Kabuli_NC_021163.1_14440413	Ca_kabuli_chr04	14440413	[A/C]	1067295463
Ca_Kabuli_NC_021163.1_14440418	Ca_kabuli_chr04	14440418	[A/C]	1067295464

SNP IDs	Chromosomes	Physical positions (bp)	SNPs	NCBI SNPdb accession IDs (NCBI_ss#)
Ca_Kabuli_NC_021163.1_14448716	Ca_kabuli_chr04	14448716	[T/A]	1067295465
Ca_Kabuli_NC_021163.1_14453927	Ca_kabuli_chr04	14453927	[T/C]	1067295466
Ca_Kabuli_NC_021163.1_14453933	Ca_kabuli_chr04	14453933	[T/C]	1067295467
Ca_Kabuli_NC_021163.1_14453944	Ca_kabuli_chr04	14453944	[A/G]	1067295468
Ca_Kabuli_NC_021163.1_14453958	Ca_kabuli_chr04	14453958	[A/G]	1067295469
Ca_Kabuli_NC_021163.1_14453970	Ca_kabuli_chr04	14453970	[A/G]	1067295470
Ca_Kabuli_NC_021163.1_14562403	Ca_kabuli_chr04	14562403	[C/A]	1067295471
Ca_Kabuli_NC_021163.1_14562412	Ca_kabuli_chr04	14562412	[C/A]	1067295472
Ca_Kabuli_NC_021163.1_14562427	Ca_kabuli_chr04	14562427	[A/C]	1067295473
Ca_Kabuli_NC_021163.1_14656292	Ca_kabuli_chr04	14656292	[G/T]	1067295474
Ca_Kabuli_NC_021163.1_14656293	Ca_kabuli_chr04	14656293	[G/C]	1067295475
Ca_Kabuli_NC_021163.1_14656294	Ca_kabuli_chr04	14656294	[G/T]	1067295476
Ca_Kabuli_NC_021163.1_14657763	Ca_kabuli_chr04	14657763	[A/G]	1067295477
Ca_Kabuli_NC_021163.1_14685587	Ca_kabuli_chr04	14685587	[A/C]	1067295478
Ca_Kabuli_NC_021163.1_14685600	Ca_kabuli_chr04	14685600	[A/C]	1067295479
Ca_Kabuli_NC_021163.1_14698743	Ca_kabuli_chr04	14698743	[T/G]	1067295480
Ca_Kabuli_NC_021163.1_14699239	Ca_kabuli_chr04	14699239	[A/C]	1067295481
Ca_Kabuli_NC_021163.1_14699270	Ca_kabuli_chr04	14699270	[T/C]	1067295482
Ca_Kabuli_NC_021163.1_14699274	Ca_kabuli_chr04	14699274	[T/C]	1067295483
Ca_Kabuli_NC_021163.1_14706369	Ca_kabuli_chr04	14706369	[T/G]	1067295484
Ca_Kabuli_NC_021163.1_14706386	Ca_kabuli_chr04	14706386	[T/G]	1067295485
Ca_Kabuli_NC_021163.1_14777481	Ca_kabuli_chr04	14777481	[T/G]	1067295486
Ca_Kabuli_NC_021163.1_14785531	Ca_kabuli_chr04	14785531	[T/C]	1067295487
Ca_Kabuli_NC_021163.1_14785557	Ca_kabuli_chr04	14785557	[A/C]	1067295488
Ca_Kabuli_NC_021163.1_14786100	Ca_kabuli_chr04	14786100	[T/C]	1067295489
Ca_Kabuli_NC_021163.1_14786128	Ca_kabuli_chr04	14786128	[T/G]	1067295490
Ca_Kabuli_NC_021163.1_14786141	Ca_kabuli_chr04	14786141	[T/C]	1067295491
Ca_Kabuli_NC_021163.1_14818654	Ca_kabuli_chr04	14818654	[T/G]	1067295492
Ca_Kabuli_NC_021163.1_14831250	Ca_kabuli_chr04	14831250	[T/G]	1067295493
Ca_Kabuli_NC_021163.1_14831252	Ca_kabuli_chr04	14831252	[T/A]	1067295494
Ca_Kabuli_NC_021163.1_14960465	Ca_kabuli_chr04	14960465	[G/A]	1067295495
Ca_Kabuli_NC_021163.1_14960467	Ca_kabuli_chr04	14960467	[T/G]	1067295496
Ca_Kabuli_NC_021163.1_14985691	Ca_kabuli_chr04	14985691	[A/C]	1067295497
Ca_Kabuli_NC_021163.1_14985692	Ca_kabuli_chr04	14985692	[C/A]	1067295498

SNP IDs	Chromosomes	Physical positions (bp)	SNPs	NCBI SNPdb accession IDs (NCBI_ss#)
Ca_Kabuli_NC_021163.1_14985694	Ca_kabuli_chr04	14985694	[C/A]	1067295499
Ca_Kabuli_NC_021163.1_14985695	Ca_kabuli_chr04	14985695	[A/C]	1067295500
Ca_Kabuli_NC_021163.1_15022229	Ca_kabuli_chr04	15022229	[T/C]	1067295501
Ca_Kabuli_NC_021163.1_15022234	Ca_kabuli_chr04	15022234	[C/G]	1067295502
Ca_Kabuli_NC_021163.1_15022256	Ca_kabuli_chr04	15022256	[T/G]	1067295503
Ca_Kabuli_NC_021163.1_15022269	Ca_kabuli_chr04	15022269	[T/A]	1067295504
Ca_Kabuli_NC_021163.1_15022270	Ca_kabuli_chr04	15022270	[T/A]	1067295505
Ca_Kabuli_NC_021163.1_15036349	Ca_kabuli_chr04	15036349	[A/C]	1067295506
Ca_Kabuli_NC_021163.1_15039822	Ca_kabuli_chr04	15039822	[C/A]	1067295507
Ca_Kabuli_NC_021163.1_15039994	Ca_kabuli_chr04	15039994	[A/T]	1067295508
Ca_Kabuli_NC_021163.1_15109654	Ca_kabuli_chr04	15109654	[T/G]	1067295509
Ca_Kabuli_NC_021163.1_15109738	Ca_kabuli_chr04	15109738	[G/T]	1067295510
Ca_Kabuli_NC_021163.1_15143547	Ca_kabuli_chr04	15143547	[T/A]	1067295511
Ca_Kabuli_NC_021163.1_15177151	Ca_kabuli_chr04	15177151	[T/G]	1067295512
Ca_Kabuli_NC_021163.1_15190651	Ca_kabuli_chr04	15190651	[T/C]	1067295513
Ca_Kabuli_NC_021163.1_15190655	Ca_kabuli_chr04	15190655	[A/G]	1067295514
Ca_Kabuli_NC_021163.1_15190663	Ca_kabuli_chr04	15190663	[T/C]	1067295515
Ca_Kabuli_NC_021163.1_15240690	Ca_kabuli_chr04	15240690	[A/C]	1067295516
Ca_Kabuli_NC_021163.1_15273133	Ca_kabuli_chr04	15273133	[T/C]	1067295517
Ca_Kabuli_NC_021163.1_15273138	Ca_kabuli_chr04	15273138	[A/G]	1067295518
Ca_Kabuli_NC_021163.1_15273145	Ca_kabuli_chr04	15273145	[T/C]	1067295519
Ca_Kabuli_NC_021163.1_15273159	Ca_kabuli_chr04	15273159	[T/C]	1067295520
Ca_Kabuli_NC_021163.1_15273171	Ca_kabuli_chr04	15273171	[A/G]	1067295521
Ca_Kabuli_NC_021163.1_15299038	Ca_kabuli_chr04	15299038	[T/G]	1067295522
Ca_Kabuli_NC_021163.1_15299210	Ca_kabuli_chr04	15299210	[T/C]	1067295523
Ca_Kabuli_NC_021163.1_15299223	Ca_kabuli_chr04	15299223	[A/G]	1067295524
Ca_Kabuli_NC_021163.1_15389690	Ca_kabuli_chr04	15389690	[A/G]	1067295525
Ca_Kabuli_NC_021163.1_15389702	Ca_kabuli_chr04	15389702	[T/C]	1067295526
Ca_Kabuli_NC_021163.1_15389711	Ca_kabuli_chr04	15389711	[A/G]	1067295527
Ca_Kabuli_NC_021163.1_15389726	Ca_kabuli_chr04	15389726	[A/T]	1067295528
Ca_Kabuli_NC_021163.1_15402086	Ca_kabuli_chr04	15402086	[T/G]	1067295529
Ca_Kabuli_NC_021163.1_15402093	Ca_kabuli_chr04	15402093	[A/T]	1067295530
Ca_Kabuli_NC_021163.1_15402097	Ca_kabuli_chr04	15402097	[T/G]	1067295531
Ca_Kabuli_NC_021163.1_15447013	Ca_kabuli_chr04	15447013	[A/G]	1067295532

SNP IDs	Chromosomes	Physical positions (bp)	SNPs	NCBI SNPdb accession IDs (NCBI_ss#)
Ca_Kabuli_NC_021163.1_15452440	Ca_kabuli_chr04	15452440	[T/G]	1067295533
Ca_Kabuli_NC_021163.1_15542631	Ca_kabuli_chr04	15542631	[T/G]	1067295534
Ca_Kabuli_NC_021163.1_15552657	Ca_kabuli_chr04	15552657	[T/C]	1067295535
Ca_Kabuli_NC_021163.1_15651804	Ca_kabuli_chr04	15651804	[C/A]	1067295536
Ca_Kabuli_NC_021163.1_15697080	Ca_kabuli_chr04	15697080	[T/C]	1067295537
Ca_Kabuli_NC_021163.1_15697119	Ca_kabuli_chr04	15697119	[A/C]	1067295538
Ca_Kabuli_NC_021163.1_15697125	Ca_kabuli_chr04	15697125	[A/C]	1067295539
Ca_Kabuli_NC_021163.1_15697146	Ca_kabuli_chr04	15697146	[A/G]	1067295540
Ca_Kabuli_NC_021163.1_15697413	Ca_kabuli_chr04	15697413	[A/G]	1067295541
Ca_Kabuli_NC_021163.1_15697418	Ca_kabuli_chr04	15697418	[A/G]	1067295542
Ca_Kabuli_NC_021163.1_15697805	Ca_kabuli_chr04	15697805	[A/C]	1067295543
Ca_Kabuli_NC_021163.1_15698219	Ca_kabuli_chr04	15698219	[T/C]	1067295544
Ca_Kabuli_NC_021163.1_15698342	Ca_kabuli_chr04	15698342	[A/C]	1067295545
Ca_Kabuli_NC_021163.1_15700605	Ca_kabuli_chr04	15700605	[A/T]	1067295546
Ca_Kabuli_NC_021163.1_15703805	Ca_kabuli_chr04	15703805	[A/T]	1067295547
Ca_Kabuli_NC_021163.1_15743441	Ca_kabuli_chr04	15743441	[T/C]	1067295548
Ca_Kabuli_NC_021163.1_15743459	Ca_kabuli_chr04	15743459	[A/T]	1067295549
Ca_Kabuli_NC_021163.1_15744703	Ca_kabuli_chr04	15744703	[T/G]	1067295550
Ca_Kabuli_NC_021163.1_15744732	Ca_kabuli_chr04	15744732	[A/C]	1067295551
Ca_Kabuli_NC_021163.1_15821266	Ca_kabuli_chr04	15821266	[A/C]	1067295552
Ca_Kabuli_NC_021163.1_15821270	Ca_kabuli_chr04	15821270	[A/C]	1067295553
Ca_Kabuli_NC_021163.1_15900593	Ca_kabuli_chr04	15900593	[A/G]	1067295554
Ca_Kabuli_NC_021163.1_15900602	Ca_kabuli_chr04	15900602	[G/A]	1067295555
Ca_Kabuli_NC_021163.1_15900604	Ca_kabuli_chr04	15900604	[G/A]	1067295556
Ca_Kabuli_NC_021163.1_15900605	Ca_kabuli_chr04	15900605	[T/G]	1067295557
Ca_Kabuli_NC_021163.1_15916534	Ca_kabuli_chr04	15916534	[G/A]	1067295558
Ca_Kabuli_NC_021163.1_15926160	Ca_kabuli_chr04	15926160	[G/A]	1067295559
Ca_Kabuli_NC_021163.1_15934462	Ca_kabuli_chr04	15934462	[G/A]	1067295560
Ca_Kabuli_NC_021163.1_15934607	Ca_kabuli_chr04	15934607	[T/A]	1067295561
Ca_Kabuli_NC_021163.1_15934901	Ca_kabuli_chr04	15934901	[G/T]	1067295562
Ca_Kabuli_NC_021163.1_15942084	Ca_kabuli_chr04	15942084	[A/G]	1067295563
Ca_Kabuli_NC_021163.1_15942490	Ca_kabuli_chr04	15942490	[A/G]	1067295564
Ca_Kabuli_NC_021163.1_15942542	Ca_kabuli_chr04	15942542	[A/C]	1067295565
Ca_Kabuli_NC_021163.1_15957070	Ca_kabuli_chr04	15957070	[A/T]	1067295566

SNP IDs	Chromosomes	Physical positions (bp)	SNPs	NCBI SNPdb accession IDs (NCBI_ss#)
Ca_Kabuli_NC_021163.1_15962223	Ca_kabuli_chr04	15962223	[A/G]	1067295567
Ca_Kabuli_NC_021163.1_15962674	Ca_kabuli_chr04	15962674	[G/A]	1067295568
Ca_Kabuli_NC_021163.1_16027227	Ca_kabuli_chr04	16027227	[A/G]	1067295569
Ca_Kabuli_NC_021163.1_16027259	Ca_kabuli_chr04	16027259	[A/C]	1067295570
Ca_Kabuli_NC_021163.1_16027281	Ca_kabuli_chr04	16027281	[A/G]	1067295571
Ca_Kabuli_NC_021163.1_16046928	Ca_kabuli_chr04	16046928	[C/T]	1067295572
Ca_Kabuli_NC_021163.1_16149795	Ca_kabuli_chr04	16149795	[T/C]	1067295573
Ca_Kabuli_NC_021163.1_16149998	Ca_kabuli_chr04	16149998	[C/T]	1067295574
Ca_Kabuli_NC_021163.1_16150042	Ca_kabuli_chr04	16150042	[T/G]	1067295575
Ca_Kabuli_NC_021163.1_16278671	Ca_kabuli_chr04	16278671	[A/T]	1067295576
Ca_Kabuli_NC_021163.1_16361657	Ca_kabuli_chr04	16361657	[A/T]	1067295577
Ca_Kabuli_NC_021163.1_16446946	Ca_kabuli_chr04	16446946	[T/A]	1067295578
Ca_Kabuli_NC_021163.1_16446962	Ca_kabuli_chr04	16446962	[A/G]	1067295579
Ca_Kabuli_NC_021163.1_16447212	Ca_kabuli_chr04	16447212	[T/C]	1067295580
Ca_Kabuli_NC_021163.1_16447213	Ca_kabuli_chr04	16447213	[C/T]	1067295581
Ca_Kabuli_NC_021163.1_16447214	Ca_kabuli_chr04	16447214	[C/T]	1067295582
Ca_Kabuli_NC_021163.1_16448174	Ca_kabuli_chr04	16448174	[T/C]	1067295583
Ca_Kabuli_NC_021163.1_16448200	Ca_kabuli_chr04	16448200	[T/G]	1067295584
Ca_Kabuli_NC_021163.1_16457949	Ca_kabuli_chr04	16457949	[T/A]	1067295585
Ca_Kabuli_NC_021163.1_16457966	Ca_kabuli_chr04	16457966	[T/G]	1067295586
Ca_Kabuli_NC_021163.1_16457971	Ca_kabuli_chr04	16457971	[A/C]	1067295587
Ca_Kabuli_NC_021163.1_16457977	Ca_kabuli_chr04	16457977	[A/T]	1067295588
Ca_Kabuli_NC_021163.1_16500750	Ca_kabuli_chr04	16500750	[T/G]	1067295589
Ca_Kabuli_NC_021163.1_16500752	Ca_kabuli_chr04	16500752	[T/A]	1067295590
Ca_Kabuli_NC_021163.1_16500755	Ca_kabuli_chr04	16500755	[T/C]	1067295591
Ca_Kabuli_NC_021163.1_16501487	Ca_kabuli_chr04	16501487	[T/A]	1067295592
Ca_Kabuli_NC_021163.1_16517910	Ca_kabuli_chr04	16517910	[T/G]	1067295593
Ca_Kabuli_NC_021163.1_16532045	Ca_kabuli_chr04	16532045	[A/G]	1067295594
Ca_Kabuli_NC_021163.1_16536720	Ca_kabuli_chr04	16536720	[T/A]	1067295595
Ca_Kabuli_NC_021163.1_16537984	Ca_kabuli_chr04	16537984	[A/C]	1067295596
Ca_Kabuli_NC_021163.1_16586896	Ca_kabuli_chr04	16586896	[A/T]	1067295597
Ca_Kabuli_NC_021163.1_16622385	Ca_kabuli_chr04	16622385	[T/A]	1067295598
Ca_Kabuli_NC_021163.1_16622409	Ca_kabuli_chr04	16622409	[T/G]	1067295599
Ca_Kabuli_NC_021163.1_16622418	Ca_kabuli_chr04	16622418	[A/C]	1067295600

SNP IDs	Chromosomes	Physical positions (bp)	SNPs	NCBI SNPdb accession IDs (NCBI_ss#)
Ca_Kabuli_NC_021163.1_16622467	Ca_kabuli_chr04	16622467	[A/G]	1067295601
Ca_Kabuli_NC_021163.1_16622499	Ca_kabuli_chr04	16622499	[A/G]	1067295602
Ca_Kabuli_NC_021163.1_16622505	Ca_kabuli_chr04	16622505	[T/A]	1067295603
Ca_Kabuli_NC_021163.1_16622509	Ca_kabuli_chr04	16622509	[C/G]	1067295604
Ca_Kabuli_NC_021163.1_16622512	Ca_kabuli_chr04	16622512	[A/G]	1067295605
Ca_Kabuli_NC_021163.1_16622521	Ca_kabuli_chr04	16622521	[A/C]	1067295606
Ca_Kabuli_NC_021163.1_16622550	Ca_kabuli_chr04	16622550	[T/C]	1067295607
Ca_Kabuli_NC_021163.1_16622568	Ca_kabuli_chr04	16622568	[T/A]	1067295608
Ca_Kabuli_NC_021163.1_16622571	Ca_kabuli_chr04	16622571	[T/C]	1067295609
Ca_Kabuli_NC_021163.1_16628556	Ca_kabuli_chr04	16628556	[A/G]	1067295610
Ca_Kabuli_NC_021163.1_16628559	Ca_kabuli_chr04	16628559	[T/G]	1067295611
Ca_Kabuli_NC_021163.1_16628605	Ca_kabuli_chr04	16628605	[G/A]	1067295612
Ca_Kabuli_NC_021163.1_16629643	Ca_kabuli_chr04	16629643	[T/C]	1067295613
Ca_Kabuli_NC_021163.1_16629646	Ca_kabuli_chr04	16629646	[T/C]	1067295614
Ca_Kabuli_NC_021163.1_16630376	Ca_kabuli_chr04	16630376	[A/C]	1067295615
Ca_Kabuli_NC_021163.1_16670183	Ca_kabuli_chr04	16670183	[A/G]	1067295616
Ca_Kabuli_NC_021163.1_16670198	Ca_kabuli_chr04	16670198	[A/G]	1067295617
Ca_Kabuli_NC_021163.1_16754113	Ca_kabuli_chr04	16754113	[C/A]	1067295618
Ca_Kabuli_NC_021163.1_16773172	Ca_kabuli_chr04	16773172	[A/C]	1067295619
Ca_Kabuli_NC_021163.1_16782993	Ca_kabuli_chr04	16782993	[C/A]	1067295620
Ca_Kabuli_NC_021163.1_16783500	Ca_kabuli_chr04	16783500	[A/C]	1067295621
Ca_Kabuli_NC_021163.1_16789488	Ca_kabuli_chr04	16789488	[T/G]	1067295622
Ca_Kabuli_NC_021163.1_16789494	Ca_kabuli_chr04	16789494	[T/G]	1067295623
Ca_Kabuli_NC_021163.1_16789497	Ca_kabuli_chr04	16789497	[T/G]	1067295624
Ca_Kabuli_NC_021163.1_16789500	Ca_kabuli_chr04	16789500	[T/G]	1067295625
Ca_Kabuli_NC_021163.1_16789518	Ca_kabuli_chr04	16789518	[G/T]	1067295626
Ca_Kabuli_NC_021163.1_16830347	Ca_kabuli_chr04	16830347	[T/G]	1067295627
Ca_Kabuli_NC_021163.1_16830372	Ca_kabuli_chr04	16830372	[A/T]	1067295628
Ca_Kabuli_NC_021163.1_16830398	Ca_kabuli_chr04	16830398	[T/C]	1067295629
Ca_Kabuli_NC_021163.1_16846275	Ca_kabuli_chr04	16846275	[A/T]	1067295630
Ca_Kabuli_NC_021163.1_16847692	Ca_kabuli_chr04	16847692	[A/C]	1067295631
Ca_Kabuli_NC_021163.1_16847697	Ca_kabuli_chr04	16847697	[A/C]	1067295632
Ca_Kabuli_NC_021163.1_16847711	Ca_kabuli_chr04	16847711	[A/G]	1067295633
Ca_Kabuli_NC_021163.1_16847739	Ca_kabuli_chr04	16847739	[T/C]	1067295634

SNP IDs	Chromosomes	Physical positions (bp)	SNPs	NCBI SNPdb accession IDs (NCBI_ss#)
Ca_Kabuli_NC_021163.1_16866627	Ca_kabuli_chr04	16866627	[T/G]	1067295635
Ca_Kabuli_NC_021163.1_16872519	Ca_kabuli_chr04	16872519	[T/G]	1067295636
Ca_Kabuli_NC_021163.1_16889591	Ca_kabuli_chr04	16889591	[T/A]	1067295637
Ca_Kabuli_NC_021163.1_16926491	Ca_kabuli_chr04	16926491	[A/G]	1067295638
Ca_Kabuli_NC_021163.1_16926503	Ca_kabuli_chr04	16926503	[A/G]	1067295639
Ca_Kabuli_NC_021163.1_16926506	Ca_kabuli_chr04	16926506	[A/C]	1067295640
Ca_Kabuli_NC_021163.1_16926515	Ca_kabuli_chr04	16926515	[A/C]	1067295641
Ca_Kabuli_NC_021163.1_16926524	Ca_kabuli_chr04	16926524	[T/C]	1067295642
Ca_Kabuli_NC_021163.1_16926596	Ca_kabuli_chr04	16926596	[T/C]	1067295643
Ca_Kabuli_NC_021163.1_16926635	Ca_kabuli_chr04	16926635	[A/G]	1067295644
Ca_Kabuli_NC_021163.1_16926652	Ca_kabuli_chr04	16926652	[T/C]	1067295645
Ca_Kabuli_NC_021163.1_16926656	Ca_kabuli_chr04	16926656	[A/G]	1067295646
Ca_Kabuli_NC_021163.1_16931381	Ca_kabuli_chr04	16931381	[T/G]	1067295647
Ca_Kabuli_NC_021163.1_16932273	Ca_kabuli_chr04	16932273	[A/C]	1067295648
Ca_Kabuli_NC_021163.1_16932276	Ca_kabuli_chr04	16932276	[C/T]	1067295649
Ca_Kabuli_NC_021163.1_17003904	Ca_kabuli_chr04	17003904	[A/T]	1067295650
Ca_Kabuli_NC_021163.1_17028391	Ca_kabuli_chr04	17028391	[T/G]	1067295651
Ca_Kabuli_NC_021163.1_17109263	Ca_kabuli_chr04	17109263	[A/G]	1067295652
Ca_Kabuli_NC_021163.1_17109297	Ca_kabuli_chr04	17109297	[A/G]	1067295653
Ca_Kabuli_NC_021163.1_17115127	Ca_kabuli_chr04	17115127	[A/C]	1067295654
Ca_Kabuli_NC_021163.1_17148049	Ca_kabuli_chr04	17148049	[C/G]	1067295655
Ca_Kabuli_NC_021163.1_17148050	Ca_kabuli_chr04	17148050	[C/A]	1067295656
Ca_Kabuli_NC_021163.1_17148052	Ca_kabuli_chr04	17148052	[G/C]	1067295657
Ca_Kabuli_NC_021163.1_17148053	Ca_kabuli_chr04	17148053	[G/T]	1067295658
Ca_Kabuli_NC_021163.1_17202598	Ca_kabuli_chr04	17202598	[A/T]	1067295659
Ca_Kabuli_NC_021163.1_17202605	Ca_kabuli_chr04	17202605	[A/G]	1067295660
Ca_Kabuli_NC_021163.1_17203994	Ca_kabuli_chr04	17203994	[A/C]	1067295661
Ca_Kabuli_NC_021163.1_17203997	Ca_kabuli_chr04	17203997	[A/C]	1067295662
Ca_Kabuli_NC_021163.1_17204002	Ca_kabuli_chr04	17204002	[T/C]	1067295663
Ca_Kabuli_NC_021163.1_17228576	Ca_kabuli_chr04	17228576	[T/C]	1067295664
Ca_Kabuli_NC_021163.1_17228599	Ca_kabuli_chr04	17228599	[A/T]	1067295665
Ca_Kabuli_NC_021163.1_17264493	Ca_kabuli_chr04	17264493	[C/A]	1067295666
Ca_Kabuli_NC_021163.1_17274890	Ca_kabuli_chr04	17274890	[A/G]	1067295667
Ca_Kabuli_NC_021163.1_17274893	Ca_kabuli_chr04	17274893	[T/C]	1067295668

SNP IDs	Chromosomes	Physical positions (bp)	SNPs	NCBI SNPdb accession IDs (NCBI_ss#)
Ca_Kabuli_NC_021163.1_17274913	Ca_kabuli_chr04	17274913	[C/A]	1067295669
Ca_Kabuli_NC_021163.1_17275870	Ca_kabuli_chr04	17275870	[T/C]	1067295670
Ca_Kabuli_NC_021163.1_17278732	Ca_kabuli_chr04	17278732	[C/T]	1067295671
Ca_Kabuli_NC_021163.1_17278734	Ca_kabuli_chr04	17278734	[T/C]	1067295672
Ca_Kabuli_NC_021163.1_17303693	Ca_kabuli_chr04	17303693	[A/C]	1067295673
Ca_Kabuli_NC_021163.1_17305899	Ca_kabuli_chr04	17305899	[T/G]	1067295674
Ca_Kabuli_NC_021163.1_17306028	Ca_kabuli_chr04	17306028	[T/C]	1067295675
Ca_Kabuli_NC_021163.1_17306043	Ca_kabuli_chr04	17306043	[A/G]	1067295676
Ca_Kabuli_NC_021163.1_17309658	Ca_kabuli_chr04	17309658	[A/C]	1067295677
Ca_Kabuli_NC_021163.1_17309659	Ca_kabuli_chr04	17309659	[C/T]	1067295678
Ca_Kabuli_NC_021163.1_17396352	Ca_kabuli_chr04	17396352	[T/G]	1067295679
Ca_Kabuli_NC_021163.1_17401841	Ca_kabuli_chr04	17401841	[T/G]	1067295680
Ca_Kabuli_NC_021163.1_17408093	Ca_kabuli_chr04	17408093	[A/G]	1067295681
Ca_Kabuli_NC_021163.1_17427162	Ca_kabuli_chr04	17427162	[T/C]	1067295682
Ca_Kabuli_NC_021163.1_17427181	Ca_kabuli_chr04	17427181	[T/G]	1067295683
Ca_Kabuli_NC_021163.1_17429446	Ca_kabuli_chr04	17429446	[C/A]	1067295684
Ca_Kabuli_NC_021163.1_17446750	Ca_kabuli_chr04	17446750	[T/G]	1067295685
Ca_Kabuli_NC_021163.1_17449395	Ca_kabuli_chr04	17449395	[A/T]	1067295686
Ca_Kabuli_NC_021163.1_17449417	Ca_kabuli_chr04	17449417	[T/C]	1067295687
Ca_Kabuli_NC_021163.1_17451460	Ca_kabuli_chr04	17451460	[T/G]	1067295688
Ca_Kabuli_NC_021163.1_17473015	Ca_kabuli_chr04	17473015	[T/C]	1067295689
Ca_Kabuli_NC_021163.1_17473018	Ca_kabuli_chr04	17473018	[C/T]	1067295690
Ca_Kabuli_NC_021163.1_17473210	Ca_kabuli_chr04	17473210	[A/C]	1067295691
Ca_Kabuli_NC_021163.1_17473225	Ca_kabuli_chr04	17473225	[A/G]	1067295692
Ca_Kabuli_NC_021163.1_17473312	Ca_kabuli_chr04	17473312	[C/A]	1067295693
Ca_Kabuli_NC_021163.1_17494744	Ca_kabuli_chr04	17494744	[A/C]	1067295694
Ca_Kabuli_NC_021163.1_17494756	Ca_kabuli_chr04	17494756	[T/A]	1067295695
Ca_Kabuli_NC_021163.1_17494770	Ca_kabuli_chr04	17494770	[G/A]	1067295696
Ca_Kabuli_NC_021163.1_17494771	Ca_kabuli_chr04	17494771	[C/T]	1067295697
Ca_Kabuli_NC_021163.1_17494772	Ca_kabuli_chr04	17494772	[G/C]	1067295698
Ca_Kabuli_NC_021163.1_17494773	Ca_kabuli_chr04	17494773	[G/T]	1067295699
Ca_Kabuli_NC_021163.1_17497238	Ca_kabuli_chr04	17497238	[A/C]	1067295700
Ca_Kabuli_NC_021163.1_17595267	Ca_kabuli_chr04	17595267	[A/G]	1067295701
Ca_Kabuli_NC_021163.1_17595297	Ca_kabuli_chr04	17595297	[A/G]	1067295702

SNP IDs	Chromosomes	Physical positions (bp)	SNPs	NCBI SNPdb accession IDs (NCBI_ss#)
Ca_Kabuli_NC_021163.1_17621574	Ca_kabuli_chr04	17621574	[T/G]	1067295703
Ca_Kabuli_NC_021163.1_17621583	Ca_kabuli_chr04	17621583	[A/G]	1067295704
Ca_Kabuli_NC_021163.1_17621619	Ca_kabuli_chr04	17621619	[T/G]	1067295705
Ca_Kabuli_NC_021163.1_17622943	Ca_kabuli_chr04	17622943	[T/G]	1067295706
Ca_Kabuli_NC_021163.1_17622952	Ca_kabuli_chr04	17622952	[T/C]	1067295707
Ca_Kabuli_NC_021163.1_17622969	Ca_kabuli_chr04	17622969	[T/G]	1067295708
Ca_Kabuli_NC_021163.1_17622979	Ca_kabuli_chr04	17622979	[A/G]	1067295709
Ca_Kabuli_NC_021163.1_17647678	Ca_kabuli_chr04	17647678	[T/C]	1067295710
Ca_Kabuli_NC_021163.1_17647684	Ca_kabuli_chr04	17647684	[T/G]	1067295711
Ca_Kabuli_NC_021163.1_17647689	Ca_kabuli_chr04	17647689	[G/T]	1067295712
Ca_Kabuli_NC_021163.1_17647693	Ca_kabuli_chr04	17647693	[T/G]	1067295713
Ca_Kabuli_NC_021163.1_17647716	Ca_kabuli_chr04	17647716	[T/G]	1067295714
Ca_Kabuli_NC_021163.1_17652717	Ca_kabuli_chr04	17652717	[T/G]	1067295715
Ca_Kabuli_NC_021163.1_17685071	Ca_kabuli_chr04	17685071	[A/T]	1067295716
Ca_Kabuli_NC_021163.1_17685098	Ca_kabuli_chr04	17685098	[T/C]	1067295717
Ca_Kabuli_NC_021163.1_17718922	Ca_kabuli_chr04	17718922	[T/G]	1067295718
Ca_Kabuli_NC_021163.1_17751940	Ca_kabuli_chr04	17751940	[T/A]	1067295719
Ca_Kabuli_NC_021163.1_17771707	Ca_kabuli_chr04	17771707	[T/C]	1067295720
Ca_Kabuli_NC_021163.1_17839101	Ca_kabuli_chr04	17839101	[A/C]	1067295721
Ca_Kabuli_NC_021163.1_17839132	Ca_kabuli_chr04	17839132	[A/G]	1067295722
Ca_Kabuli_NC_021163.1_17847854	Ca_kabuli_chr04	17847854	[T/G]	1067295723
Ca_Kabuli_NC_021163.1_17858133	Ca_kabuli_chr04	17858133	[C/T]	1067295724
Ca_Kabuli_NC_021163.1_17858140	Ca_kabuli_chr04	17858140	[A/C]	1067295725
Ca_Kabuli_NC_021163.1_17886842	Ca_kabuli_chr04	17886842	[A/C]	1067295726
Ca_Kabuli_NC_021163.1_17913708	Ca_kabuli_chr04	17913708	[A/T]	1067295727
Ca_Kabuli_NC_021163.1_17913711	Ca_kabuli_chr04	17913711	[T/C]	1067295728
Ca_Kabuli_NC_021163.1_17913719	Ca_kabuli_chr04	17913719	[G/C]	1067295729
Ca_Kabuli_NC_021163.1_17913726	Ca_kabuli_chr04	17913726	[G/A]	1067295730
Ca_Kabuli_NC_021163.1_17913731	Ca_kabuli_chr04	17913731	[A/C]	1067295731
Ca_Kabuli_NC_021163.1_17914220	Ca_kabuli_chr04	17914220	[T/A]	1067295732
Ca_Kabuli_NC_021163.1_17914257	Ca_kabuli_chr04	17914257	[A/C]	1067295733
Ca_Kabuli_NC_021163.1_17914277	Ca_kabuli_chr04	17914277	[A/C]	1067295734
Ca_Kabuli_NC_021163.1_17920556	Ca_kabuli_chr04	17920556	[A/G]	1067295735
Ca_Kabuli_NC_021163.1_17920572	Ca_kabuli_chr04	17920572	[T/C]	1067295736

SNP IDs	Chromosomes	Physical positions (bp)	SNPs	NCBI SNPdb accession IDs (NCBI_ss#)
Ca_Kabuli_NC_021163.1_17920613	Ca_kabuli_chr04	17920613	[T/G]	1067295737
Ca_Kabuli_NC_021163.1_17920629	Ca_kabuli_chr04	17920629	[T/C]	1067295738
Ca_Kabuli_NC_021163.1_17920771	Ca_kabuli_chr04	17920771	[A/C]	1067295739
Ca_Kabuli_NC_021163.1_17953824	Ca_kabuli_chr04	17953824	[T/C]	1067295740
Ca_Kabuli_NC_021163.1_17953846	Ca_kabuli_chr04	17953846	[T/C]	1067295741
Ca_Kabuli_NC_021163.1_17990878	Ca_kabuli_chr04	17990878	[G/T]	1067295742
Ca_Kabuli_NC_021163.1_17990879	Ca_kabuli_chr04	17990879	[G/C]	1067295743
Ca_Kabuli_NC_021163.1_17990880	Ca_kabuli_chr04	17990880	[G/T]	1067295744
Ca_Kabuli_NC_021163.1_17992406	Ca_kabuli_chr04	17992406	[T/G]	1067295745
Ca_Kabuli_NC_021163.1_17992532	Ca_kabuli_chr04	17992532	[A/T]	1067295746
Ca_Kabuli_NC_021163.1_17992540	Ca_kabuli_chr04	17992540	[T/A]	1067295747
Ca_Kabuli_NC_021163.1_17992553	Ca_kabuli_chr04	17992553	[T/C]	1067295748
Ca_Kabuli_NC_021163.1_18013299	Ca_kabuli_chr04	18013299	[G/A]	1067295749
Ca_Kabuli_NC_021163.1_18070194	Ca_kabuli_chr04	18070194	[T/C]	1067295750
Ca_Kabuli_NC_021163.1_18071074	Ca_kabuli_chr04	18071074	[A/C]	1067295751
Ca_Kabuli_NC_021163.1_18071095	Ca_kabuli_chr04	18071095	[A/C]	1067295752
Ca_Kabuli_NC_021163.1_18071100	Ca_kabuli_chr04	18071100	[A/C]	1067295753
Ca_Kabuli_NC_021163.1_18071129	Ca_kabuli_chr04	18071129	[T/C]	1067295754
Ca_Kabuli_NC_021163.1_18071135	Ca_kabuli_chr04	18071135	[T/G]	1067295755
Ca_Kabuli_NC_021163.1_18071138	Ca_kabuli_chr04	18071138	[T/C]	1067295756
Ca_Kabuli_NC_021163.1_18071152	Ca_kabuli_chr04	18071152	[T/C]	1067295757
Ca_Kabuli_NC_021163.1_18089508	Ca_kabuli_chr04	18089508	[A/C]	1067295758
Ca_Kabuli_NC_021163.1_18117799	Ca_kabuli_chr04	18117799	[G/T]	1067295759
Ca_Kabuli_NC_021163.1_18167607	Ca_kabuli_chr04	18167607	[A/T]	1067295760
Ca_Kabuli_NC_021163.1_18167620	Ca_kabuli_chr04	18167620	[T/C]	1067295761
Ca_Kabuli_NC_021163.1_18167660	Ca_kabuli_chr04	18167660	[A/C]	1067295762
Ca_Kabuli_NC_021163.1_18167679	Ca_kabuli_chr04	18167679	[T/C]	1067295763
Ca_Kabuli_NC_021163.1_18168367	Ca_kabuli_chr04	18168367	[A/C]	1067295764
Ca_Kabuli_NC_021163.1_18168378	Ca_kabuli_chr04	18168378	[G/A]	1067295765
Ca_Kabuli_NC_021163.1_18168379	Ca_kabuli_chr04	18168379	[C/G]	1067295766
Ca_Kabuli_NC_021163.1_18168381	Ca_kabuli_chr04	18168381	[C/G]	1067295767
Ca_Kabuli_NC_021163.1_18168385	Ca_kabuli_chr04	18168385	[G/T]	1067295768
Ca_Kabuli_NC_021163.1_18168387	Ca_kabuli_chr04	18168387	[C/T]	1067295769
Ca_Kabuli_NC_021163.1_18171478	Ca_kabuli_chr04	18171478	[A/C]	1067295770

SNP IDs	Chromosomes	Physical positions (bp)	SNPs	NCBI SNPdb accession IDs (NCBI_ss#)
Ca_Kabuli_NC_021163.1_18171483	Ca_kabuli_chr04	18171483	[A/T]	1067295771
Ca_Kabuli_NC_021163.1_18171498	Ca_kabuli_chr04	18171498	[A/C]	1067295772
Ca_Kabuli_NC_021163.1_18171505	Ca_kabuli_chr04	18171505	[A/G]	1067295773
Ca_Kabuli_NC_021163.1_18171522	Ca_kabuli_chr04	18171522	[T/G]	1067295774
Ca_Kabuli_NC_021163.1_18171534	Ca_kabuli_chr04	18171534	[A/G]	1067295775
Ca_Kabuli_NC_021163.1_18171542	Ca_kabuli_chr04	18171542	[A/G]	1067295776
Ca_Kabuli_NC_021163.1_18427444	Ca_kabuli_chr04	18427444	[A/G]	1067295777
Ca_Kabuli_NC_021163.1_18433965	Ca_kabuli_chr04	18433965	[A/G]	1067295778
Ca_Kabuli_NC_021163.1_18433977	Ca_kabuli_chr04	18433977	[T/C]	1067295779
Ca_Kabuli_NC_021163.1_18433981	Ca_kabuli_chr04	18433981	[T/C]	1067295780
Ca_Kabuli_NC_021163.1_18433987	Ca_kabuli_chr04	18433987	[A/G]	1067295781
Ca_Kabuli_NC_021163.1_18435345	Ca_kabuli_chr04	18435345	[T/A]	1067295782
Ca_Kabuli_NC_021163.1_18435353	Ca_kabuli_chr04	18435353	[T/A]	1067295783
Ca_Kabuli_NC_021163.1_18485909	Ca_kabuli_chr04	18485909	[T/G]	1067295784
Ca_Kabuli_NC_021163.1_18493810	Ca_kabuli_chr04	18493810	[T/G]	1067295785
Ca_Kabuli_NC_021163.1_18505268	Ca_kabuli_chr04	18505268	[T/C]	1067295786
Ca_Kabuli_NC_021163.1_18505312	Ca_kabuli_chr04	18505312	[A/G]	1067295787
Ca_Kabuli_NC_021163.1_18523370	Ca_kabuli_chr04	18523370	[A/G]	1067295788
Ca_Kabuli_NC_021163.1_18523398	Ca_kabuli_chr04	18523398	[G/T]	1067295789
Ca_Kabuli_NC_021163.1_18526794	Ca_kabuli_chr04	18526794	[A/G]	1067295790
Ca_Kabuli_NC_021163.1_18585411	Ca_kabuli_chr04	18585411	[A/G]	1067295791
Ca_Kabuli_NC_021163.1_18585421	Ca_kabuli_chr04	18585421	[A/G]	1067295792
Ca_Kabuli_NC_021163.1_18592828	Ca_kabuli_chr04	18592828	[T/C]	1067295793
Ca_Kabuli_NC_021163.1_18628594	Ca_kabuli_chr04	18628594	[T/A]	1067295794
Ca_Kabuli_NC_021163.1_18633561	Ca_kabuli_chr04	18633561	[T/G]	1067295795
Ca_Kabuli_NC_021163.1_18719393	Ca_kabuli_chr04	18719393	[T/C]	1067295796
Ca_Kabuli_NC_021163.1_18719417	Ca_kabuli_chr04	18719417	[A/G]	1067295797
Ca_Kabuli_NC_021163.1_18719419	Ca_kabuli_chr04	18719419	[C/G]	1067295798
Ca_Kabuli_NC_021163.1_18719438	Ca_kabuli_chr04	18719438	[A/G]	1067295799
Ca_Kabuli_NC_021163.1_18719451	Ca_kabuli_chr04	18719451	[A/G]	1067295800
Ca_Kabuli_NC_021163.1_18719461	Ca_kabuli_chr04	18719461	[A/G]	1067295801
Ca_Kabuli_NC_021163.1_18719463	Ca_kabuli_chr04	18719463	[A/G]	1067295802
Ca_Kabuli_NC_021163.1_18720247	Ca_kabuli_chr04	18720247	[A/G]	1067295803
Ca_Kabuli_NC_021163.1_18720318	Ca_kabuli_chr04	18720318	[A/C]	1067295804

SNP IDs	Chromosomes	Physical positions (bp)	SNPs	NCBI SNPdb accession IDs (NCBI_ss#)
Ca_Kabuli_NC_021163.1_18723809	Ca_kabuli_chr04	18723809	[A/G]	1067295805
Ca_Kabuli_NC_021163.1_18727628	Ca_kabuli_chr04	18727628	[T/C]	1067295806
Ca_Kabuli_NC_021163.1_18727657	Ca_kabuli_chr04	18727657	[T/G]	1067295807
Ca_Kabuli_NC_021163.1_18747915	Ca_kabuli_chr04	18747915	[A/G]	1067295808
Ca_Kabuli_NC_021163.1_18752357	Ca_kabuli_chr04	18752357	[A/G]	1067295809
Ca_Kabuli_NC_021163.1_18855201	Ca_kabuli_chr04	18855201	[T/C]	1067295810
Ca_Kabuli_NC_021163.1_18867076	Ca_kabuli_chr04	18867076	[A/C]	1067295811
Ca_Kabuli_NC_021163.1_18867117	Ca_kabuli_chr04	18867117	[T/G]	1067295812
Ca_Kabuli_NC_021163.1_18875677	Ca_kabuli_chr04	18875677	[A/C]	1067295813
Ca_Kabuli_NC_021163.1_18886200	Ca_kabuli_chr04	18886200	[A/C]	1067295814
Ca_Kabuli_NC_021163.1_18912194	Ca_kabuli_chr04	18912194	[A/C]	1067295815
Ca_Kabuli_NC_021163.1_18923641	Ca_kabuli_chr04	18923641	[G/T]	1067295816
Ca_Kabuli_NC_021163.1_18943307	Ca_kabuli_chr04	18943307	[A/C]	1067295817
Ca_Kabuli_NC_021163.1_18958392	Ca_kabuli_chr04	18958392	[A/T]	1067295818
Ca_Kabuli_NC_021163.1_18958409	Ca_kabuli_chr04	18958409	[G/A]	1067295819
Ca_Kabuli_NC_021163.1_18959763	Ca_kabuli_chr04	18959763	[A/C]	1067295820
Ca_Kabuli_NC_021163.1_18994533	Ca_kabuli_chr04	18994533	[G/A]	1067295821
Ca_Kabuli_NC_021163.1_18994535	Ca_kabuli_chr04	18994535	[A/C]	1067295822
Ca_Kabuli_NC_021163.1_18994536	Ca_kabuli_chr04	18994536	[G/T]	1067295823
Ca_Kabuli_NC_021163.1_19056752	Ca_kabuli_chr04	19056752	[A/C]	1067295824
Ca_Kabuli_NC_021163.1_19056787	Ca_kabuli_chr04	19056787	[A/G]	1067295825
Ca_Kabuli_NC_021163.1_19057199	Ca_kabuli_chr04	19057199	[T/G]	1067295826
Ca_Kabuli_NC_021163.1_19057215	Ca_kabuli_chr04	19057215	[T/A]	1067295827
Ca_Kabuli_NC_021163.1_19061732	Ca_kabuli_chr04	19061732	[T/G]	1067295828
Ca_Kabuli_NC_021163.1_19061738	Ca_kabuli_chr04	19061738	[A/G]	1067295829
Ca_Kabuli_NC_021163.1_19152663	Ca_kabuli_chr04	19152663	[C/T]	1067295830
Ca_Kabuli_NC_021163.1_19152665	Ca_kabuli_chr04	19152665	[A/C]	1067295831
Ca_Kabuli_NC_021163.1_19166053	Ca_kabuli_chr04	19166053	[T/G]	1067295832
Ca_Kabuli_NC_021163.1_19207681	Ca_kabuli_chr04	19207681	[T/C]	1067295833
Ca_Kabuli_NC_021163.1_19211864	Ca_kabuli_chr04	19211864	[A/C]	1067295834
Ca_Kabuli_NC_021163.1_19227621	Ca_kabuli_chr04	19227621	[T/G]	1067295835
Ca_Kabuli_NC_021163.1_19227624	Ca_kabuli_chr04	19227624	[T/C]	1067295836
Ca_Kabuli_NC_021163.1_19263967	Ca_kabuli_chr04	19263967	[T/G]	1067295837
Ca_Kabuli_NC_021163.1_19540579	Ca_kabuli_chr04	19540579	[T/G]	1067295838

SNP IDs	Chromosomes	Physical positions (bp)	SNPs	NCBI SNPdb accession IDs (NCBI_ss#)
Ca_Kabuli_NC_021163.1_19593695	Ca_kabuli_chr04	19593695	[C/A]	1067295839
Ca_Kabuli_NC_021163.1_19593696	Ca_kabuli_chr04	19593696	[C/G]	1067295840
Ca_Kabuli_NC_021163.1_19593699	Ca_kabuli_chr04	19593699	[A/C]	1067295841
Ca_Kabuli_NC_021163.1_19593700	Ca_kabuli_chr04	19593700	[T/G]	1067295842
Ca_Kabuli_NC_021163.1_19660464	Ca_kabuli_chr04	19660464	[A/C]	1067295843
Ca_Kabuli_NC_021163.1_19687982	Ca_kabuli_chr04	19687982	[T/C]	1067295844
Ca_Kabuli_NC_021163.1_19712368	Ca_kabuli_chr04	19712368	[A/C]	1067295845
Ca_Kabuli_NC_021163.1_19712457	Ca_kabuli_chr04	19712457	[A/T]	1067295846
Ca_Kabuli_NC_021163.1_19712931	Ca_kabuli_chr04	19712931	[G/A]	1067295847
Ca_Kabuli_NC_021163.1_19733301	Ca_kabuli_chr04	19733301	[A/C]	1067295848
Ca_Kabuli_NC_021163.1_19738361	Ca_kabuli_chr04	19738361	[C/G]	1067295849
Ca_Kabuli_NC_021163.1_19738371	Ca_kabuli_chr04	19738371	[A/G]	1067295850
Ca_Kabuli_NC_021163.1_19738402	Ca_kabuli_chr04	19738402	[A/C]	1067295851
Ca_Kabuli_NC_021163.1_19755453	Ca_kabuli_chr04	19755453	[T/C]	1067295852
Ca_Kabuli_NC_021163.1_19755467	Ca_kabuli_chr04	19755467	[T/C]	1067295853
Ca_Kabuli_NC_021163.1_19765190	Ca_kabuli_chr04	19765190	[T/C]	1067295854
Ca_Kabuli_NC_021163.1_19765194	Ca_kabuli_chr04	19765194	[T/G]	1067295855
Ca_Kabuli_NC_021163.1_19765202	Ca_kabuli_chr04	19765202	[A/T]	1067295856
Ca_Kabuli_NC_021163.1_19765230	Ca_kabuli_chr04	19765230	[A/G]	1067295857
Ca_Kabuli_NC_021163.1_19765242	Ca_kabuli_chr04	19765242	[C/T]	1067295858
Ca_Kabuli_NC_021163.1_19869636	Ca_kabuli_chr04	19869636	[A/C]	1067295859
Ca_Kabuli_NC_021163.1_19869708	Ca_kabuli_chr04	19869708	[A/G]	1067295860
Ca_Kabuli_NC_021163.1_19869717	Ca_kabuli_chr04	19869717	[T/G]	1067295861
Ca_Kabuli_NC_021163.1_19869722	Ca_kabuli_chr04	19869722	[T/G]	1067295862
Ca_Kabuli_NC_021163.1_19869742	Ca_kabuli_chr04	19869742	[A/C]	1067295863
Ca_Kabuli_NC_021163.1_19870269	Ca_kabuli_chr04	19870269	[A/G]	1067295864
Ca_Kabuli_NC_021163.1_20161601	Ca_kabuli_chr04	20161601	[A/G]	1067295865
Ca_Kabuli_NC_021163.1_20193277	Ca_kabuli_chr04	20193277	[T/G]	1067295866
Ca_Kabuli_NC_021163.1_20193298	Ca_kabuli_chr04	20193298	[T/G]	1067295867
Ca_Kabuli_NC_021163.1_20215275	Ca_kabuli_chr04	20215275	[A/G]	1067295868
Ca_Kabuli_NC_021163.1_20229504	Ca_kabuli_chr04	20229504	[A/C]	1067295869
Ca_Kabuli_NC_021163.1_20229510	Ca_kabuli_chr04	20229510	[T/C]	1067295870
Ca_Kabuli_NC_021163.1_20229520	Ca_kabuli_chr04	20229520	[A/G]	1067295871
Ca_Kabuli_NC_021163.1_20398685	Ca_kabuli_chr04	20398685	[A/C]	1067295872

SNP IDs	Chromosomes	Physical positions (bp)	SNPs	NCBI SNPdb accession IDs (NCBI_ss#)
Ca_Kabuli_NC_021163.1_20398714	Ca_kabuli_chr04	20398714	[A/T]	1067295873
Ca_Kabuli_NC_021163.1_20435986	Ca_kabuli_chr04	20435986	[T/C]	1067295874
Ca_Kabuli_NC_021163.1_20435998	Ca_kabuli_chr04	20435998	[A/G]	1067295875
Ca_Kabuli_NC_021163.1_20436025	Ca_kabuli_chr04	20436025	[T/G]	1067295876
Ca_Kabuli_NC_021163.1_20473194	Ca_kabuli_chr04	20473194	[A/C]	1067295877
Ca_Kabuli_NC_021163.1_20599115	Ca_kabuli_chr04	20599115	[C/T]	1067295878
Ca_Kabuli_NC_021163.1_20668758	Ca_kabuli_chr04	20668758	[T/G]	1067295879
Ca_Kabuli_NC_021163.1_20668802	Ca_kabuli_chr04	20668802	[A/C]	1067295880
Ca_Kabuli_NC_021163.1_20669172	Ca_kabuli_chr04	20669172	[A/C]	1067295881
Ca_Kabuli_NC_021163.1_20669340	Ca_kabuli_chr04	20669340	[A/G]	1067295882
Ca_Kabuli_NC_021163.1_20669365	Ca_kabuli_chr04	20669365	[A/C]	1067295883
Ca_Kabuli_NC_021163.1_20669387	Ca_kabuli_chr04	20669387	[A/G]	1067295884
Ca_Kabuli_NC_021163.1_20669520	Ca_kabuli_chr04	20669520	[A/C]	1067295885
Ca_Kabuli_NC_021163.1_20696943	Ca_kabuli_chr04	20696943	[A/G]	1067295886
Ca_Kabuli_NC_021163.1_20697620	Ca_kabuli_chr04	20697620	[C/T]	1067295887
Ca_Kabuli_NC_021163.1_20697689	Ca_kabuli_chr04	20697689	[A/T]	1067295888
Ca_Kabuli_NC_021163.1_20745125	Ca_kabuli_chr04	20745125	[A/C]	1067295889
Ca_Kabuli_NC_021163.1_20764801	Ca_kabuli_chr04	20764801	[T/C]	1067295890
Ca_Kabuli_NC_021163.1_20764818	Ca_kabuli_chr04	20764818	[A/G]	1067295891
Ca_Kabuli_NC_021163.1_20764913	Ca_kabuli_chr04	20764913	[T/G]	1067295892
Ca_Kabuli_NC_021163.1_20905105	Ca_kabuli_chr04	20905105	[A/G]	1067295893
Ca_Kabuli_NC_021163.1_20907409	Ca_kabuli_chr04	20907409	[T/C]	1067295894
Ca_Kabuli_NC_021163.1_20907435	Ca_kabuli_chr04	20907435	[A/C]	1067295895
Ca_Kabuli_NC_021163.1_21385323	Ca_kabuli_chr04	21385323	[T/G]	1067295896
Ca_Kabuli_NC_021163.1_21409377	Ca_kabuli_chr04	21409377	[T/G]	1067295897
Ca_Kabuli_NC_021163.1_21593215	Ca_kabuli_chr04	21593215	[G/A]	1067295898
Ca_Kabuli_NC_021163.1_21830594	Ca_kabuli_chr04	21830594	[A/G]	1067295899
Ca_Kabuli_NC_021163.1_22079563	Ca_kabuli_chr04	22079563	[T/C]	1067295900
Ca_Kabuli_NC_021163.1_22079657	Ca_kabuli_chr04	22079657	[A/C]	1067295901
Ca_Kabuli_NC_021163.1_22099567	Ca_kabuli_chr04	22099567	[A/T]	1067295902
Ca_Kabuli_NC_021163.1_22108956	Ca_kabuli_chr04	22108956	[A/G]	1067295903
Ca_Kabuli_NC_021163.1_22108961	Ca_kabuli_chr04	22108961	[T/G]	1067295904
Ca_Kabuli_NC_021163.1_22108978	Ca_kabuli_chr04	22108978	[T/A]	1067295905
Ca_Kabuli_NC_021163.1_22124986	Ca_kabuli_chr04	22124986	[C/A]	1067295906

SNP IDs	Chromosomes	Physical positions (bp)	SNPs	NCBI SNPdb accession IDs (NCBI_ss#)
Ca_Kabuli_NC_021163.1_22124987	Ca_kabuli_chr04	22124987	[T/G]	1067295907
Ca_Kabuli_NC_021163.1_22124988	Ca_kabuli_chr04	22124988	[G/A]	1067295908
Ca_Kabuli_NC_021163.1_22238028	Ca_kabuli_chr04	22238028	[A/C]	1067295909
Ca_Kabuli_NC_021163.1_22266672	Ca_kabuli_chr04	22266672	[A/T]	1067295910
Ca_Kabuli_NC_021163.1_22266678	Ca_kabuli_chr04	22266678	[C/A]	1067295911
Ca_Kabuli_NC_021163.1_22289520	Ca_kabuli_chr04	22289520	[T/C]	1067295912
Ca_Kabuli_NC_021163.1_22289561	Ca_kabuli_chr04	22289561	[A/C]	1067295913
Ca_Kabuli_NC_021163.1_22293448	Ca_kabuli_chr04	22293448	[T/G]	1067295914
Ca_Kabuli_NC_021163.1_22293455	Ca_kabuli_chr04	22293455	[T/A]	1067295915
Ca_Kabuli_NC_021163.1_22295491	Ca_kabuli_chr04	22295491	[A/C]	1067295916
Ca_Kabuli_NC_021163.1_22375189	Ca_kabuli_chr04	22375189	[T/G]	1067295917
Ca_Kabuli_NC_021163.1_22375612	Ca_kabuli_chr04	22375612	[A/C]	1067295918
Ca_Kabuli_NC_021163.1_22695001	Ca_kabuli_chr04	22695001	[A/G]	1067295919
Ca_Kabuli_NC_021163.1_22827207	Ca_kabuli_chr04	22827207	[T/C]	1067295920
Ca_Kabuli_NC_021163.1_22850596	Ca_kabuli_chr04	22850596	[C/G]	1067295921
Ca_Kabuli_NC_021163.1_22850617	Ca_kabuli_chr04	22850617	[T/C]	1067295922
Ca_Kabuli_NC_021163.1_22850630	Ca_kabuli_chr04	22850630	[C/A]	1067295923
Ca_Kabuli_NC_021163.1_22850637	Ca_kabuli_chr04	22850637	[T/G]	1067295924
Ca_Kabuli_NC_021163.1_22850643	Ca_kabuli_chr04	22850643	[T/A]	1067295925
Ca_Kabuli_NC_021163.1_22860484	Ca_kabuli_chr04	22860484	[T/G]	1067295926
Ca_Kabuli_NC_021163.1_22860656	Ca_kabuli_chr04	22860656	[C/A]	1067295927
Ca_Kabuli_NC_021163.1_22864624	Ca_kabuli_chr04	22864624	[A/G]	1067295928
Ca_Kabuli_NC_021163.1_22865436	Ca_kabuli_chr04	22865436	[A/T]	1067295929
Ca_Kabuli_NC_021163.1_22904068	Ca_kabuli_chr04	22904068	[T/C]	1067295930
Ca_Kabuli_NC_021163.1_22908336	Ca_kabuli_chr04	22908336	[A/T]	1067295931
Ca_Kabuli_NC_021163.1_22908350	Ca_kabuli_chr04	22908350	[T/A]	1067295932
Ca_Kabuli_NC_021163.1_22908360	Ca_kabuli_chr04	22908360	[A/G]	1067295933
Ca_Kabuli_NC_021163.1_22971678	Ca_kabuli_chr04	22971678	[A/C]	1067295934
Ca_Kabuli_NC_021163.1_22971687	Ca_kabuli_chr04	22971687	[A/G]	1067295935
Ca_Kabuli_NC_021163.1_22971797	Ca_kabuli_chr04	22971797	[T/C]	1067295936
Ca_Kabuli_NC_021163.1_22971853	Ca_kabuli_chr04	22971853	[A/C]	1067295937
Ca_Kabuli_NC_021163.1_23104495	Ca_kabuli_chr04	23104495	[T/G]	1067295938
Ca_Kabuli_NC_021163.1_23104498	Ca_kabuli_chr04	23104498	[T/G]	1067295939
Ca_Kabuli_NC_021163.1_23107182	Ca_kabuli_chr04	23107182	[A/G]	1067295940

SNP IDs	Chromosomes	Physical positions (bp)	SNPs	NCBI SNPdb accession IDs (NCBI_ss#)
Ca_Kabuli_NC_021163.1_23157619	Ca_kabuli_chr04	23157619	[T/G]	1067295941
Ca_Kabuli_NC_021163.1_23157635	Ca_kabuli_chr04	23157635	[T/C]	1067295942
Ca_Kabuli_NC_021163.1_23157638	Ca_kabuli_chr04	23157638	[T/C]	1067295943
Ca_Kabuli_NC_021163.1_23157671	Ca_kabuli_chr04	23157671	[A/G]	1067295944
Ca_Kabuli_NC_021163.1_23194502	Ca_kabuli_chr04	23194502	[T/G]	1067295945
Ca_Kabuli_NC_021163.1_23195429	Ca_kabuli_chr04	23195429	[G/T]	1067295946
Ca_Kabuli_NC_021163.1_23195437	Ca_kabuli_chr04	23195437	[T/C]	1067295947
Ca_Kabuli_NC_021163.1_23195447	Ca_kabuli_chr04	23195447	[G/T]	1067295948
Ca_Kabuli_NC_021163.1_23203064	Ca_kabuli_chr04	23203064	[A/C]	1067295949
Ca_Kabuli_NC_021163.1_23203077	Ca_kabuli_chr04	23203077	[A/G]	1067295950
Ca_Kabuli_NC_021163.1_23267869	Ca_kabuli_chr04	23267869	[A/C]	1067295951
Ca_Kabuli_NC_021163.1_23267883	Ca_kabuli_chr04	23267883	[A/C]	1067295952
Ca_Kabuli_NC_021163.1_23267886	Ca_kabuli_chr04	23267886	[T/A]	1067295953
Ca_Kabuli_NC_021163.1_23267891	Ca_kabuli_chr04	23267891	[C/A]	1067295954
Ca_Kabuli_NC_021163.1_23267915	Ca_kabuli_chr04	23267915	[A/C]	1067295955
Ca_Kabuli_NC_021163.1_23314676	Ca_kabuli_chr04	23314676	[A/C]	1067295956
Ca_Kabuli_NC_021163.1_23331556	Ca_kabuli_chr04	23331556	[A/C]	1067295957
Ca_Kabuli_NC_021163.1_23333583	Ca_kabuli_chr04	23333583	[A/C]	1067295958
Ca_Kabuli_NC_021163.1_23337322	Ca_kabuli_chr04	23337322	[T/C]	1067295959
Ca_Kabuli_NC_021163.1_23337349	Ca_kabuli_chr04	23337349	[T/C]	1067295960
Ca_Kabuli_NC_021163.1_23337430	Ca_kabuli_chr04	23337430	[A/C]	1067295961
Ca_Kabuli_NC_021163.1_23449152	Ca_kabuli_chr04	23449152	[A/C]	1067295962
Ca_Kabuli_NC_021163.1_23449214	Ca_kabuli_chr04	23449214	[A/C]	1067295963
Ca_Kabuli_NC_021163.1_23449298	Ca_kabuli_chr04	23449298	[A/G]	1067295964
Ca_Kabuli_NC_021163.1_23634799	Ca_kabuli_chr04	23634799	[A/C]	1067295965
Ca_Kabuli_NC_021163.1_23671430	Ca_kabuli_chr04	23671430	[T/C]	1067295966
Ca_Kabuli_NC_021163.1_23731599	Ca_kabuli_chr04	23731599	[T/A]	1067295967
Ca_Kabuli_NC_021163.1_23872475	Ca_kabuli_chr04	23872475	[T/G]	1067295968
Ca_Kabuli_NC_021163.1_23921817	Ca_kabuli_chr04	23921817	[T/G]	1067295969
Ca_Kabuli_NC_021163.1_24240537	Ca_kabuli_chr04	24240537	[A/C]	1067295970
Ca_Kabuli_NC_021163.1_24240556	Ca_kabuli_chr04	24240556	[T/A]	1067295971
Ca_Kabuli_NC_021163.1_24261779	Ca_kabuli_chr04	24261779	[A/C]	1067295972
Ca_Kabuli_NC_021163.1_24533026	Ca_kabuli_chr04	24533026	[A/G]	1067295973
Ca_Kabuli_NC_021163.1_24586826	Ca_kabuli_chr04	24586826	[T/G]	1067295974

SNP IDs	Chromosomes	Physical positions (bp)	SNPs	NCBI SNPdb accession IDs (NCBI_ss#)
Ca_Kabuli_NC_021163.1_24591218	Ca_kabuli_chr04	24591218	[A/C]	1067295975
Ca_Kabuli_NC_021163.1_24591231	Ca_kabuli_chr04	24591231	[A/T]	1067295976
Ca_Kabuli_NC_021163.1_24591243	Ca_kabuli_chr04	24591243	[A/G]	1067295977
Ca_Kabuli_NC_021163.1_24615928	Ca_kabuli_chr04	24615928	[A/G]	1067295978
Ca_Kabuli_NC_021163.1_24615942	Ca_kabuli_chr04	24615942	[T/C]	1067295979
Ca_Kabuli_NC_021163.1_24615969	Ca_kabuli_chr04	24615969	[A/G]	1067295980
Ca_Kabuli_NC_021163.1_24615973	Ca_kabuli_chr04	24615973	[A/C]	1067295981
Ca_Kabuli_NC_021163.1_24615990	Ca_kabuli_chr04	24615990	[T/C]	1067295982
Ca_Kabuli_NC_021163.1_24713674	Ca_kabuli_chr04	24713674	[A/T]	1067295983
Ca_Kabuli_NC_021163.1_24713717	Ca_kabuli_chr04	24713717	[T/C]	1067295984
Ca_Kabuli_NC_021163.1_24713725	Ca_kabuli_chr04	24713725	[A/G]	1067295985
Ca_Kabuli_NC_021163.1_24713734	Ca_kabuli_chr04	24713734	[T/A]	1067295986
Ca_Kabuli_NC_021163.1_24719395	Ca_kabuli_chr04	24719395	[T/C]	1067295987
Ca_Kabuli_NC_021163.1_24719409	Ca_kabuli_chr04	24719409	[T/G]	1067295988
Ca_Kabuli_NC_021163.1_24719466	Ca_kabuli_chr04	24719466	[T/G]	1067295989
Ca_Kabuli_NC_021163.1_24719561	Ca_kabuli_chr04	24719561	[A/G]	1067295990
Ca_Kabuli_NC_021163.1_24729157	Ca_kabuli_chr04	24729157	[T/C]	1067295991
Ca_Kabuli_NC_021163.1_24729192	Ca_kabuli_chr04	24729192	[C/A]	1067295992
Ca_Kabuli_NC_021163.1_24729195	Ca_kabuli_chr04	24729195	[G/A]	1067295993
Ca_Kabuli_NC_021163.1_24729198	Ca_kabuli_chr04	24729198	[A/C]	1067295994
Ca_Kabuli_NC_021163.1_24729294	Ca_kabuli_chr04	24729294	[A/C]	1067295995
Ca_Kabuli_NC_021163.1_24771768	Ca_kabuli_chr04	24771768	[A/C]	1067295996
Ca_Kabuli_NC_021163.1_24771771	Ca_kabuli_chr04	24771771	[A/C]	1067295997
Ca_Kabuli_NC_021163.1_24856201	Ca_kabuli_chr04	24856201	[A/G]	1067295998
Ca_Kabuli_NC_021163.1_24857501	Ca_kabuli_chr04	24857501	[A/G]	1067295999
Ca_Kabuli_NC_021163.1_24861477	Ca_kabuli_chr04	24861477	[T/G]	1067296000
Ca_Kabuli_NC_021163.1_25251375	Ca_kabuli_chr04	25251375	[T/C]	1067296001
Ca_Kabuli_NC_021163.1_25251379	Ca_kabuli_chr04	25251379	[A/G]	1067296002
Ca_Kabuli_NC_021163.1_25386077	Ca_kabuli_chr04	25386077	[G/A]	1067296003
Ca_Kabuli_NC_021163.1_25386310	Ca_kabuli_chr04	25386310	[A/T]	1067296004
Ca_Kabuli_NC_021163.1_25386321	Ca_kabuli_chr04	25386321	[T/C]	1067296005
Ca_Kabuli_NC_021163.1_25513681	Ca_kabuli_chr04	25513681	[A/G]	1067296006
Ca_Kabuli_NC_021163.1_25513690	Ca_kabuli_chr04	25513690	[T/C]	1067296007
Ca_Kabuli_NC_021163.1_25650720	Ca_kabuli_chr04	25650720	[A/T]	1067296008

SNP IDs	Chromosomes	Physical positions (bp)	SNPs	NCBI SNPdb accession IDs (NCBI_ss#)
Ca_Kabuli_NC_021163.1_25657291	Ca_kabuli_chr04	25657291	[T/C]	1067296009
Ca_Kabuli_NC_021163.1_25700553	Ca_kabuli_chr04	25700553	[T/G]	1067296010
Ca_Kabuli_NC_021163.1_25729905	Ca_kabuli_chr04	25729905	[C/A]	1067296011
Ca_Kabuli_NC_021163.1_25817794	Ca_kabuli_chr04	25817794	[T/C]	1067296012
Ca_Kabuli_NC_021163.1_25818698	Ca_kabuli_chr04	25818698	[A/G]	1067296013
Ca_Kabuli_NC_021163.1_25818711	Ca_kabuli_chr04	25818711	[T/C]	1067296014
Ca_Kabuli_NC_021163.1_25818719	Ca_kabuli_chr04	25818719	[T/C]	1067296015
Ca_Kabuli_NC_021163.1_25818731	Ca_kabuli_chr04	25818731	[C/G]	1067296016
Ca_Kabuli_NC_021163.1_25818733	Ca_kabuli_chr04	25818733	[T/C]	1067296017
Ca_Kabuli_NC_021163.1_25818741	Ca_kabuli_chr04	25818741	[T/G]	1067296018
Ca_Kabuli_NC_021163.1_25843911	Ca_kabuli_chr04	25843911	[T/C]	1067296019
Ca_Kabuli_NC_021163.1_25843927	Ca_kabuli_chr04	25843927	[A/G]	1067296020
Ca_Kabuli_NC_021163.1_25843931	Ca_kabuli_chr04	25843931	[T/C]	1067296021
Ca_Kabuli_NC_021163.1_25843937	Ca_kabuli_chr04	25843937	[T/A]	1067296022
Ca_Kabuli_NC_021163.1_25855756	Ca_kabuli_chr04	25855756	[C/A]	1067296023
Ca_Kabuli_NC_021163.1_25985095	Ca_kabuli_chr04	25985095	[T/C]	1067296024
Ca_Kabuli_NC_021163.1_26002991	Ca_kabuli_chr04	26002991	[A/C]	1067296025
Ca_Kabuli_NC_021163.1_26089832	Ca_kabuli_chr04	26089832	[C/T]	1067296026
Ca_Kabuli_NC_021163.1_26219483	Ca_kabuli_chr04	26219483	[T/G]	1067296027
Ca_Kabuli_NC_021163.1_26221325	Ca_kabuli_chr04	26221325	[C/T]	1067296028
Ca_Kabuli_NC_021163.1_26221326	Ca_kabuli_chr04	26221326	[C/T]	1067296029
Ca_Kabuli_NC_021163.1_26221327	Ca_kabuli_chr04	26221327	[G/T]	1067296030
Ca_Kabuli_NC_021163.1_26221336	Ca_kabuli_chr04	26221336	[A/C]	1067296031
Ca_Kabuli_NC_021163.1_26221338	Ca_kabuli_chr04	26221338	[A/C]	1067296032
Ca_Kabuli_NC_021163.1_26529578	Ca_kabuli_chr04	26529578	[A/G]	1067296033
Ca_Kabuli_NC_021163.1_26530930	Ca_kabuli_chr04	26530930	[A/C]	1067296034
Ca_Kabuli_NC_021163.1_26532751	Ca_kabuli_chr04	26532751	[T/C]	1067296035
Ca_Kabuli_NC_021163.1_26532763	Ca_kabuli_chr04	26532763	[T/G]	1067296036
Ca_Kabuli_NC_021163.1_26611657	Ca_kabuli_chr04	26611657	[A/C]	1067296037
Ca_Kabuli_NC_021163.1_26612521	Ca_kabuli_chr04	26612521	[T/A]	1067296038
Ca_Kabuli_NC_021163.1_26612597	Ca_kabuli_chr04	26612597	[G/T]	1067296039
Ca_Kabuli_NC_021163.1_26645875	Ca_kabuli_chr04	26645875	[A/G]	1067296040
Ca_Kabuli_NC_021163.1_26660664	Ca_kabuli_chr04	26660664	[C/T]	1067296041
Ca_Kabuli_NC_021163.1_26764829	Ca_kabuli_chr04	26764829	[T/G]	1067296042

SNP IDs	Chromosomes	Physical positions (bp)	SNPs	NCBI SNPdb accession IDs (NCBI_ss#)
Ca_Kabuli_NC_021163.1_26764860	Ca_kabuli_chr04	26764860	[T/C]	1067296043
Ca_Kabuli_NC_021163.1_26764869	Ca_kabuli_chr04	26764869	[A/C]	1067296044
Ca_Kabuli_NC_021163.1_26834518	Ca_kabuli_chr04	26834518	[T/C]	1067296045
Ca_Kabuli_NC_021163.1_26869917	Ca_kabuli_chr04	26869917	[A/C]	1067296046
Ca_Kabuli_NC_021163.1_26941637	Ca_kabuli_chr04	26941637	[A/T]	1067296047
Ca_Kabuli_NC_021163.1_26989112	Ca_kabuli_chr04	26989112	[T/G]	1067296048
Ca_Kabuli_NC_021163.1_26989197	Ca_kabuli_chr04	26989197	[A/C]	1067296049
Ca_Kabuli_NC_021163.1_27142314	Ca_kabuli_chr04	27142314	[G/A]	1067296050
Ca_Kabuli_NC_021163.1_27493041	Ca_kabuli_chr04	27493041	[A/G]	1067296051
Ca_Kabuli_NC_021163.1_27493045	Ca_kabuli_chr04	27493045	[T/G]	1067296052
Ca_Kabuli_NC_021163.1_27493051	Ca_kabuli_chr04	27493051	[A/G]	1067296053
Ca_Kabuli_NC_021163.1_27617813	Ca_kabuli_chr04	27617813	[A/C]	1067296054
Ca_Kabuli_NC_021163.1_27669925	Ca_kabuli_chr04	27669925	[T/G]	1067296055
Ca_Kabuli_NC_021163.1_27774461	Ca_kabuli_chr04	27774461	[T/C]	1067296056
Ca_Kabuli_NC_021163.1_27777836	Ca_kabuli_chr04	27777836	[T/C]	1067296057
Ca_Kabuli_NC_021163.1_27777882	Ca_kabuli_chr04	27777882	[T/C]	1067296058
Ca_Kabuli_NC_021163.1_27786230	Ca_kabuli_chr04	27786230	[A/C]	1067296059
Ca_Kabuli_NC_021163.1_27971105	Ca_kabuli_chr04	27971105	[T/A]	1067296060
Ca_Kabuli_NC_021163.1_28060949	Ca_kabuli_chr04	28060949	[T/G]	1067296061
Ca_Kabuli_NC_021163.1_28060964	Ca_kabuli_chr04	28060964	[C/G]	1067296062
Ca_Kabuli_NC_021163.1_28060969	Ca_kabuli_chr04	28060969	[A/G]	1067296063
Ca_Kabuli_NC_021163.1_28092574	Ca_kabuli_chr04	28092574	[T/A]	1067296064
Ca_Kabuli_NC_021163.1_28092616	Ca_kabuli_chr04	28092616	[A/G]	1067296065
Ca_Kabuli_NC_021163.1_28092687	Ca_kabuli_chr04	28092687	[A/G]	1067296066
Ca_Kabuli_NC_021163.1_28092694	Ca_kabuli_chr04	28092694	[T/C]	1067296067
Ca_Kabuli_NC_021163.1_28092705	Ca_kabuli_chr04	28092705	[G/T]	1067296068
Ca_Kabuli_NC_021163.1_28092707	Ca_kabuli_chr04	28092707	[A/G]	1067296069
Ca_Kabuli_NC_021163.1_28092712	Ca_kabuli_chr04	28092712	[T/G]	1067296070
Ca_Kabuli_NC_021163.1_28092719	Ca_kabuli_chr04	28092719	[G/T]	1067296071
Ca_Kabuli_NC_021163.1_28092727	Ca_kabuli_chr04	28092727	[T/A]	1067296072
Ca_Kabuli_NC_021163.1_28246738	Ca_kabuli_chr04	28246738	[C/T]	1067296073
Ca_Kabuli_NC_021163.1_28246744	Ca_kabuli_chr04	28246744	[C/T]	1067296074
Ca_Kabuli_NC_021163.1_28246755	Ca_kabuli_chr04	28246755	[C/T]	1067296075
Ca_Kabuli_NC_021163.1_28246771	Ca_kabuli_chr04	28246771	[A/G]	1067296076

SNP IDs	Chromosomes	Physical positions (bp)	SNPs	NCBI SNPdb accession IDs (NCBI_ss#)
Ca_Kabuli_NC_021163.1_28246772	Ca_kabuli_chr04	28246772	[G/T]	1067296077
Ca_Kabuli_NC_021163.1_28299145	Ca_kabuli_chr04	28299145	[T/G]	1067296078
Ca_Kabuli_NC_021163.1_28299148	Ca_kabuli_chr04	28299148	[T/G]	1067296079
Ca_Kabuli_NC_021163.1_28344258	Ca_kabuli_chr04	28344258	[C/G]	1067296080
Ca_Kabuli_NC_021163.1_28431767	Ca_kabuli_chr04	28431767	[A/G]	1067296081
Ca_Kabuli_NC_021163.1_28516385	Ca_kabuli_chr04	28516385	[T/A]	1067296082
Ca_Kabuli_NC_021163.1_28517090	Ca_kabuli_chr04	28517090	[G/T]	1067296083
Ca_Kabuli_NC_021163.1_28669356	Ca_kabuli_chr04	28669356	[C/A]	1067296084
Ca_Kabuli_NC_021163.1_28669368	Ca_kabuli_chr04	28669368	[C/A]	1067296085
Ca_Kabuli_NC_021163.1_28669413	Ca_kabuli_chr04	28669413	[A/G]	1067296086
Ca_Kabuli_NC_021163.1_28790027	Ca_kabuli_chr04	28790027	[T/C]	1067296087
Ca_Kabuli_NC_021163.1_28790030	Ca_kabuli_chr04	28790030	[A/C]	1067296088
Ca_Kabuli_NC_021163.1_28790033	Ca_kabuli_chr04	28790033	[C/T]	1067296089
Ca_Kabuli_NC_021163.1_28790034	Ca_kabuli_chr04	28790034	[T/C]	1067296090
Ca_Kabuli_NC_021163.1_28790036	Ca_kabuli_chr04	28790036	[T/C]	1067296091
Ca_Kabuli_NC_021163.1_28790047	Ca_kabuli_chr04	28790047	[T/C]	1067296092
Ca_Kabuli_NC_021163.1_28790066	Ca_kabuli_chr04	28790066	[A/G]	1067296093
Ca_Kabuli_NC_021163.1_28790085	Ca_kabuli_chr04	28790085	[A/G]	1067296094
Ca_Kabuli_NC_021163.1_28863988	Ca_kabuli_chr04	28863988	[T/G]	1067296095
Ca_Kabuli_NC_021163.1_28867193	Ca_kabuli_chr04	28867193	[A/G]	1067296096
Ca_Kabuli_NC_021163.1_28940492	Ca_kabuli_chr04	28940492	[T/G]	1067296097
Ca_Kabuli_NC_021163.1_28947438	Ca_kabuli_chr04	28947438	[T/C]	1067296098
Ca_Kabuli_NC_021163.1_28947455	Ca_kabuli_chr04	28947455	[A/T]	1067296099
Ca_Kabuli_NC_021163.1_28947498	Ca_kabuli_chr04	28947498	[G/C]	1067296100
Ca_Kabuli_NC_021163.1_29092466	Ca_kabuli_chr04	29092466	[A/G]	1067296101
Ca_Kabuli_NC_021163.1_29092487	Ca_kabuli_chr04	29092487	[T/C]	1067296102
Ca_Kabuli_NC_021163.1_29092521	Ca_kabuli_chr04	29092521	[T/C]	1067296103
Ca_Kabuli_NC_021163.1_29318025	Ca_kabuli_chr04	29318025	[A/G]	1067296104
Ca_Kabuli_NC_021163.1_29318150	Ca_kabuli_chr04	29318150	[G/A]	1067296105
Ca_Kabuli_NC_021163.1_29323082	Ca_kabuli_chr04	29323082	[T/C]	1067296106
Ca_Kabuli_NC_021163.1_29476239	Ca_kabuli_chr04	29476239	[C/T]	1067296107
Ca_Kabuli_NC_021163.1_29524038	Ca_kabuli_chr04	29524038	[A/C]	1067296108
Ca_Kabuli_NC_021163.1_29524039	Ca_kabuli_chr04	29524039	[G/T]	1067296109
Ca_Kabuli_NC_021163.1_29524040	Ca_kabuli_chr04	29524040	[A/C]	1067296110

SNP IDs	Chromosomes	Physical positions (bp)	SNPs	NCBI SNPdb accession IDs (NCBI_ss#)
Ca_Kabuli_NC_021163.1_29671606	Ca_kabuli_chr04	29671606	[G/A]	1067296111
Ca_Kabuli_NC_021163.1_29675337	Ca_kabuli_chr04	29675337	[C/T]	1067296112
Ca_Kabuli_NC_021163.1_29680412	Ca_kabuli_chr04	29680412	[C/T]	1067296113
Ca_Kabuli_NC_021163.1_29680875	Ca_kabuli_chr04	29680875	[G/C]	1067296114
Ca_Kabuli_NC_021163.1_29682902	Ca_kabuli_chr04	29682902	[T/C]	1067296115
Ca_Kabuli_NC_021163.1_29682905	Ca_kabuli_chr04	29682905	[C/T]	1067296116
Ca_Kabuli_NC_021163.1_29682915	Ca_kabuli_chr04	29682915	[G/A]	1067296117
Ca_Kabuli_NC_021163.1_29682948	Ca_kabuli_chr04	29682948	[T/A]	1067296118
Ca_Kabuli_NC_021163.1_29689175	Ca_kabuli_chr04	29689175	[A/T]	1067296119
Ca_Kabuli_NC_021163.1_29923380	Ca_kabuli_chr04	29923380	[A/G]	1067296120
Ca_Kabuli_NC_021163.1_29958122	Ca_kabuli_chr04	29958122	[G/T]	1067296121
Ca_Kabuli_NC_021163.1_29972164	Ca_kabuli_chr04	29972164	[G/A]	1067296122
Ca_Kabuli_NC_021163.1_29972199	Ca_kabuli_chr04	29972199	[A/T]	1067296123
Ca_Kabuli_NC_021163.1_30131105	Ca_kabuli_chr04	30131105	[T/C]	1067296124
Ca_Kabuli_NC_021163.1_30151880	Ca_kabuli_chr04	30151880	[C/G]	1067296125
Ca_Kabuli_NC_021163.1_30153505	Ca_kabuli_chr04	30153505	[G/C]	1067296126
Ca_Kabuli_NC_021163.1_30153519	Ca_kabuli_chr04	30153519	[A/C]	1067296127
Ca_Kabuli_NC_021163.1_30153522	Ca_kabuli_chr04	30153522	[A/C]	1067296128
Ca_Kabuli_NC_021163.1_30153525	Ca_kabuli_chr04	30153525	[A/C]	1067296129
Ca_Kabuli_NC_021163.1_30258044	Ca_kabuli_chr04	30258044	[T/A]	1067296130
Ca_Kabuli_NC_021163.1_30258045	Ca_kabuli_chr04	30258045	[T/A]	1067296131
Ca_Kabuli_NC_021163.1_30258056	Ca_kabuli_chr04	30258056	[G/A]	1067296132
Ca_Kabuli_NC_021163.1_30258565	Ca_kabuli_chr04	30258565	[A/G]	1067296133
Ca_Kabuli_NC_021163.1_30258588	Ca_kabuli_chr04	30258588	[T/C]	1067296134
Ca_Kabuli_NC_021163.1_30258593	Ca_kabuli_chr04	30258593	[T/C]	1067296135
Ca_Kabuli_NC_021163.1_30258595	Ca_kabuli_chr04	30258595	[A/G]	1067296136
Ca_Kabuli_NC_021163.1_30258596	Ca_kabuli_chr04	30258596	[G/C]	1067296137
Ca_Kabuli_NC_021163.1_30258604	Ca_kabuli_chr04	30258604	[C/A]	1067296138
Ca_Kabuli_NC_021163.1_30258607	Ca_kabuli_chr04	30258607	[C/A]	1067296139
Ca_Kabuli_NC_021163.1_30258611	Ca_kabuli_chr04	30258611	[A/C]	1067296140
Ca_Kabuli_NC_021163.1_30258621	Ca_kabuli_chr04	30258621	[T/G]	1067296141
Ca_Kabuli_NC_021163.1_30259993	Ca_kabuli_chr04	30259993	[T/G]	1067296142
Ca_Kabuli_NC_021163.1_30259999	Ca_kabuli_chr04	30259999	[T/G]	1067296143
Ca_Kabuli_NC_021163.1_30260494	Ca_kabuli_chr04	30260494	[A/G]	1067296144

SNP IDs	Chromosomes	Physical positions (bp)	SNPs	NCBI SNPdb accession IDs (NCBI_ss#)
Ca_Kabuli_NC_021163.1_30260593	Ca_kabuli_chr04	30260593	[A/G]	1067296145
Ca_Kabuli_NC_021163.1_30260626	Ca_kabuli_chr04	30260626	[A/G]	1067296146
Ca_Kabuli_NC_021163.1_30261173	Ca_kabuli_chr04	30261173	[A/G]	1067296147
Ca_Kabuli_NC_021163.1_30299995	Ca_kabuli_chr04	30299995	[T/A]	1067296148
Ca_Kabuli_NC_021163.1_30300334	Ca_kabuli_chr04	30300334	[C/T]	1067296149
Ca_Kabuli_NC_021163.1_30301019	Ca_kabuli_chr04	30301019	[T/C]	1067296150
Ca_Kabuli_NC_021163.1_30301039	Ca_kabuli_chr04	30301039	[A/G]	1067296151
Ca_Kabuli_NC_021163.1_30302554	Ca_kabuli_chr04	30302554	[A/G]	1067296152
Ca_Kabuli_NC_021163.1_30308656	Ca_kabuli_chr04	30308656	[C/A]	1067296153
Ca_Kabuli_NC_021163.1_30308668	Ca_kabuli_chr04	30308668	[A/C]	1067296154
Ca_Kabuli_NC_021163.1_30308684	Ca_kabuli_chr04	30308684	[T/C]	1067296155
Ca_Kabuli_NC_021163.1_30308699	Ca_kabuli_chr04	30308699	[T/G]	1067296156
Ca_Kabuli_NC_021163.1_30308712	Ca_kabuli_chr04	30308712	[T/A]	1067296157
Ca_Kabuli_NC_021163.1_30308759	Ca_kabuli_chr04	30308759	[T/G]	1067296158
Ca_Kabuli_NC_021163.1_30334005	Ca_kabuli_chr04	30334005	[G/A]	1067296159
Ca_Kabuli_NC_021163.1_30463074	Ca_kabuli_chr04	30463074	[G/T]	1067296160
Ca_Kabuli_NC_021163.1_30463076	Ca_kabuli_chr04	30463076	[A/G]	1067296161
Ca_Kabuli_NC_021163.1_30463117	Ca_kabuli_chr04	30463117	[A/C]	1067296162
Ca_Kabuli_NC_021163.1_30580071	Ca_kabuli_chr04	30580071	[A/G]	1067296163
Ca_Kabuli_NC_021163.1_30580097	Ca_kabuli_chr04	30580097	[C/A]	1067296164
Ca_Kabuli_NC_021163.1_30580108	Ca_kabuli_chr04	30580108	[A/G]	1067296165
Ca_Kabuli_NC_021163.1_30599220	Ca_kabuli_chr04	30599220	[A/C]	1067296166
Ca_Kabuli_NC_021163.1_30603949	Ca_kabuli_chr04	30603949	[C/A]	1067296167
Ca_Kabuli_NC_021163.1_30603950	Ca_kabuli_chr04	30603950	[A/G]	1067296168
Ca_Kabuli_NC_021163.1_30603951	Ca_kabuli_chr04	30603951	[T/A]	1067296169
Ca_Kabuli_NC_021163.1_30668893	Ca_kabuli_chr04	30668893	[C/A]	1067296170
Ca_Kabuli_NC_021163.1_30668957	Ca_kabuli_chr04	30668957	[G/A]	1067296171
Ca_Kabuli_NC_021163.1_30668958	Ca_kabuli_chr04	30668958	[A/G]	1067296172
Ca_Kabuli_NC_021163.1_30674579	Ca_kabuli_chr04	30674579	[A/C]	1067296173
Ca_Kabuli_NC_021163.1_30676612	Ca_kabuli_chr04	30676612	[T/C]	1067296174
Ca_Kabuli_NC_021163.1_30676655	Ca_kabuli_chr04	30676655	[A/G]	1067296175
Ca_Kabuli_NC_021163.1_30676669	Ca_kabuli_chr04	30676669	[A/G]	1067296176
Ca_Kabuli_NC_021163.1_30852711	Ca_kabuli_chr04	30852711	[G/A]	1067296177
Ca_Kabuli_NC_021163.1_31142124	Ca_kabuli_chr04	31142124	[G/A]	1067296178

SNP IDs	Chromosomes	Physical positions (bp)	SNPs	NCBI SNPdb accession IDs (NCBI_ss#)
Ca_Kabuli_NC_021163.1_31142126	Ca_kabuli_chr04	31142126	[G/A]	1067296179
Ca_Kabuli_NC_021163.1_31221198	Ca_kabuli_chr04	31221198	[A/G]	1067296180
Ca_Kabuli_NC_021163.1_31221205	Ca_kabuli_chr04	31221205	[T/A]	1067296181
Ca_Kabuli_NC_021163.1_31221206	Ca_kabuli_chr04	31221206	[A/T]	1067296182
Ca_Kabuli_NC_021163.1_31221226	Ca_kabuli_chr04	31221226	[T/G]	1067296183
Ca_Kabuli_NC_021163.1_31221264	Ca_kabuli_chr04	31221264	[T/G]	1067296184
Ca_Kabuli_NC_021163.1_31224001	Ca_kabuli_chr04	31224001	[G/A]	1067296185
Ca_Kabuli_NC_021163.1_31224341	Ca_kabuli_chr04	31224341	[C/G]	1067296186
Ca_Kabuli_NC_021163.1_31263205	Ca_kabuli_chr04	31263205	[T/A]	1067296187
Ca_Kabuli_NC_021163.1_31594126	Ca_kabuli_chr04	31594126	[A/C]	1067296188
Ca_Kabuli_NC_021163.1_31600685	Ca_kabuli_chr04	31600685	[A/C]	1067296189
Ca_Kabuli_NC_021163.1_31843049	Ca_kabuli_chr04	31843049	[A/C]	1067296190
Ca_Kabuli_NC_021163.1_31843050	Ca_kabuli_chr04	31843050	[G/T]	1067296191
Ca_Kabuli_NC_021163.1_31849494	Ca_kabuli_chr04	31849494	[T/G]	1067296192
Ca_Kabuli_NC_021163.1_31849509	Ca_kabuli_chr04	31849509	[A/C]	1067296193
Ca_Kabuli_NC_021163.1_31884008	Ca_kabuli_chr04	31884008	[A/T]	1067296194
Ca_Kabuli_NC_021163.1_31987824	Ca_kabuli_chr04	31987824	[A/G]	1067296195
Ca_Kabuli_NC_021163.1_31987833	Ca_kabuli_chr04	31987833	[T/C]	1067296196
Ca_Kabuli_NC_021163.1_31987866	Ca_kabuli_chr04	31987866	[A/T]	1067296197
Ca_Kabuli_NC_021163.1_31987877	Ca_kabuli_chr04	31987877	[T/C]	1067296198
Ca_Kabuli_NC_021163.1_32042494	Ca_kabuli_chr04	32042494	[T/C]	1067296199
Ca_Kabuli_NC_021163.1_32042503	Ca_kabuli_chr04	32042503	[A/G]	1067296200
Ca_Kabuli_NC_021163.1_32042517	Ca_kabuli_chr04	32042517	[C/G]	1067296201
Ca_Kabuli_NC_021163.1_32042593	Ca_kabuli_chr04	32042593	[T/G]	1067296202
Ca_Kabuli_NC_021163.1_32042619	Ca_kabuli_chr04	32042619	[T/G]	1067296203
Ca_Kabuli_NC_021163.1_32068987	Ca_kabuli_chr04	32068987	[A/G]	1067296204
Ca_Kabuli_NC_021163.1_32115072	Ca_kabuli_chr04	32115072	[T/G]	1067296205
Ca_Kabuli_NC_021163.1_32115091	Ca_kabuli_chr04	32115091	[T/G]	1067296206
Ca_Kabuli_NC_021163.1_32128327	Ca_kabuli_chr04	32128327	[T/G]	1067296207
Ca_Kabuli_NC_021163.1_32213012	Ca_kabuli_chr04	32213012	[T/G]	1067296208
Ca_Kabuli_NC_021163.1_32421887	Ca_kabuli_chr04	32421887	[T/C]	1067296209
Ca_Kabuli_NC_021163.1_32604583	Ca_kabuli_chr04	32604583	[A/T]	1067296210
Ca_Kabuli_NC_021163.1_32639136	Ca_kabuli_chr04	32639136	[A/C]	1067296211
Ca_Kabuli_NC_021163.1_32639138	Ca_kabuli_chr04	32639138	[T/C]	1067296212

SNP IDs	Chromosomes	Physical positions (bp)	SNPs	NCBI SNPdb accession IDs (NCBI_ss#)
Ca_Kabuli_NC_021163.1_32639172	Ca_kabuli_chr04	32639172	[A/C]	1067296213
Ca_Kabuli_NC_021163.1_32639301	Ca_kabuli_chr04	32639301	[A/C]	1067296214
Ca_Kabuli_NC_021163.1_32779855	Ca_kabuli_chr04	32779855	[T/C]	1067296215
Ca_Kabuli_NC_021163.1_32793291	Ca_kabuli_chr04	32793291	[T/A]	1067296216
Ca_Kabuli_NC_021163.1_32835192	Ca_kabuli_chr04	32835192	[C/A]	1067296217
Ca_Kabuli_NC_021163.1_32853834	Ca_kabuli_chr04	32853834	[T/C]	1067296218
Ca_Kabuli_NC_021163.1_32853838	Ca_kabuli_chr04	32853838	[G/T]	1067296219
Ca_Kabuli_NC_021163.1_33276089	Ca_kabuli_chr04	33276089	[C/T]	1067296220
Ca_Kabuli_NC_021163.1_33316940	Ca_kabuli_chr04	33316940	[A/C]	1067296221
Ca_Kabuli_NC_021163.1_33920345	Ca_kabuli_chr04	33920345	[A/T]	1067296222
Ca_Kabuli_NC_021163.1_33920355	Ca_kabuli_chr04	33920355	[A/G]	1067296223
Ca_Kabuli_NC_021163.1_33920443	Ca_kabuli_chr04	33920443	[A/C]	1067296224
Ca_Kabuli_NC_021163.1_33929609	Ca_kabuli_chr04	33929609	[A/G]	1067296225
Ca_Kabuli_NC_021163.1_33929613	Ca_kabuli_chr04	33929613	[G/C]	1067296226
Ca_Kabuli_NC_021163.1_33929633	Ca_kabuli_chr04	33929633	[T/G]	1067296227
Ca_Kabuli_NC_021163.1_33929643	Ca_kabuli_chr04	33929643	[T/C]	1067296228
Ca_Kabuli_NC_021163.1_33929876	Ca_kabuli_chr04	33929876	[A/T]	1067296229
Ca_Kabuli_NC_021163.1_33929902	Ca_kabuli_chr04	33929902	[T/C]	1067296230
Ca_Kabuli_NC_021163.1_33934513	Ca_kabuli_chr04	33934513	[A/C]	1067296231
Ca_Kabuli_NC_021163.1_34222778	Ca_kabuli_chr04	34222778	[T/G]	1067296232
Ca_Kabuli_NC_021163.1_34314413	Ca_kabuli_chr04	34314413	[T/G]	1067296233
Ca_Kabuli_NC_021163.1_34314462	Ca_kabuli_chr04	34314462	[A/G]	1067296234
Ca_Kabuli_NC_021163.1_34317134	Ca_kabuli_chr04	34317134	[C/T]	1067296235
Ca_Kabuli_NC_021163.1_34354537	Ca_kabuli_chr04	34354537	[T/C]	1067296236
Ca_Kabuli_NC_021163.1_34354562	Ca_kabuli_chr04	34354562	[A/G]	1067296237
Ca_Kabuli_NC_021163.1_34487028	Ca_kabuli_chr04	34487028	[T/C]	1067296238
Ca_Kabuli_NC_021163.1_34487061	Ca_kabuli_chr04	34487061	[A/G]	1067296239
Ca_Kabuli_NC_021163.1_34517809	Ca_kabuli_chr04	34517809	[T/C]	1067296240
Ca_Kabuli_NC_021163.1_34744388	Ca_kabuli_chr04	34744388	[A/G]	1067296241
Ca_Kabuli_NC_021163.1_34751509	Ca_kabuli_chr04	34751509	[G/A]	1067296242
Ca_Kabuli_NC_021163.1_34798446	Ca_kabuli_chr04	34798446	[A/T]	1067296243
Ca_Kabuli_NC_021163.1_34798494	Ca_kabuli_chr04	34798494	[A/G]	1067296244
Ca_Kabuli_NC_021163.1_34798572	Ca_kabuli_chr04	34798572	[A/G]	1067296245
Ca_Kabuli_NC_021163.1_34798602	Ca_kabuli_chr04	34798602	[A/G]	1067296246

SNP IDs	Chromosomes	Physical positions (bp)	SNPs	NCBI SNPdb accession IDs (NCBI_ss#)
Ca_Kabuli_NC_021163.1_34798657	Ca_kabuli_chr04	34798657	[A/C]	1067296247
Ca_Kabuli_NC_021163.1_34915986	Ca_kabuli_chr04	34915986	[C/T]	1067296248
Ca_Kabuli_NC_021163.1_34917020	Ca_kabuli_chr04	34917020	[A/G]	1067296249
Ca_Kabuli_NC_021163.1_34917022	Ca_kabuli_chr04	34917022	[T/C]	1067296250
Ca_Kabuli_NC_021163.1_34924595	Ca_kabuli_chr04	34924595	[A/C]	1067296251
Ca_Kabuli_NC_021163.1_35141281	Ca_kabuli_chr04	35141281	[A/T]	1067296252
Ca_Kabuli_NC_021163.1_35168915	Ca_kabuli_chr04	35168915	[C/T]	1067296253
Ca_Kabuli_NC_021163.1_35168939	Ca_kabuli_chr04	35168939	[C/T]	1067296254
Ca_Kabuli_NC_021163.1_35170261	Ca_kabuli_chr04	35170261	[A/C]	1067296255
Ca_Kabuli_NC_021163.1_35170263	Ca_kabuli_chr04	35170263	[A/C]	1067296256
Ca_Kabuli_NC_021163.1_35170563	Ca_kabuli_chr04	35170563	[A/T]	1067296257
Ca_Kabuli_NC_021163.1_35171719	Ca_kabuli_chr04	35171719	[C/T]	1067296258
Ca_Kabuli_NC_021163.1_35228143	Ca_kabuli_chr04	35228143	[A/G]	1067296259
Ca_Kabuli_NC_021163.1_35228155	Ca_kabuli_chr04	35228155	[T/A]	1067296260
Ca_Kabuli_NC_021163.1_35229659	Ca_kabuli_chr04	35229659	[C/T]	1067296261
Ca_Kabuli_NC_021163.1_35229966	Ca_kabuli_chr04	35229966	[C/T]	1067296262
Ca_Kabuli_NC_021163.1_35231891	Ca_kabuli_chr04	35231891	[C/T]	1067296263
Ca_Kabuli_NC_021163.1_35257027	Ca_kabuli_chr04	35257027	[G/T]	1067296264
Ca_Kabuli_NC_021163.1_35257072	Ca_kabuli_chr04	35257072	[G/A]	1067296265
Ca_Kabuli_NC_021163.1_35298965	Ca_kabuli_chr04	35298965	[A/G]	1067296266
Ca_Kabuli_NC_021163.1_35298969	Ca_kabuli_chr04	35298969	[T/C]	1067296267
Ca_Kabuli_NC_021163.1_35298973	Ca_kabuli_chr04	35298973	[A/G]	1067296268
Ca_Kabuli_NC_021163.1_35298985	Ca_kabuli_chr04	35298985	[A/G]	1067296269
Ca_Kabuli_NC_021163.1_35298992	Ca_kabuli_chr04	35298992	[T/C]	1067296270
Ca_Kabuli_NC_021163.1_35299005	Ca_kabuli_chr04	35299005	[T/C]	1067296271
Ca_Kabuli_NC_021163.1_35299046	Ca_kabuli_chr04	35299046	[C/T]	1067296272
Ca_Kabuli_NC_021163.1_35302598	Ca_kabuli_chr04	35302598	[T/C]	1067296273
Ca_Kabuli_NC_021163.1_35303983	Ca_kabuli_chr04	35303983	[A/G]	1067296274
Ca_Kabuli_NC_021163.1_35304102	Ca_kabuli_chr04	35304102	[C/T]	1067296275
Ca_Kabuli_NC_021163.1_35309647	Ca_kabuli_chr04	35309647	[T/G]	1067296276
Ca_Kabuli_NC_021163.1_35327989	Ca_kabuli_chr04	35327989	[G/A]	1067296277
Ca_Kabuli_NC_021163.1_35328213	Ca_kabuli_chr04	35328213	[C/T]	1067296278
Ca_Kabuli_NC_021163.1_35328336	Ca_kabuli_chr04	35328336	[C/A]	1067296279
Ca_Kabuli_NC_021163.1_35328337	Ca_kabuli_chr04	35328337	[A/G]	1067296280

SNP IDs	Chromosomes	Physical positions (bp)	SNPs	NCBI SNPdb accession IDs (NCBI_ss#)
Ca_Kabuli_NC_021163.1_35346388	Ca_kabuli_chr04	35346388	[A/G]	1067296281
Ca_Kabuli_NC_021163.1_35346407	Ca_kabuli_chr04	35346407	[A/G]	1067296282
Ca_Kabuli_NC_021163.1_35346558	Ca_kabuli_chr04	35346558	[T/G]	1067296283
Ca_Kabuli_NC_021163.1_35346857	Ca_kabuli_chr04	35346857	[G/A]	1067296284
Ca_Kabuli_NC_021163.1_35347408	Ca_kabuli_chr04	35347408	[A/G]	1067296285
Ca_Kabuli_NC_021163.1_35381267	Ca_kabuli_chr04	35381267	[C/T]	1067296286
Ca_Kabuli_NC_021163.1_35381289	Ca_kabuli_chr04	35381289	[G/T]	1067296287
Ca_Kabuli_NC_021163.1_35393506	Ca_kabuli_chr04	35393506	[G/T]	1067296288
Ca_Kabuli_NC_021163.1_35393510	Ca_kabuli_chr04	35393510	[T/C]	1067296289
Ca_Kabuli_NC_021163.1_35393527	Ca_kabuli_chr04	35393527	[A/G]	1067296290
Ca_Kabuli_NC_021163.1_35393530	Ca_kabuli_chr04	35393530	[C/A]	1067296291
Ca_Kabuli_NC_021163.1_35393532	Ca_kabuli_chr04	35393532	[T/C]	1067296292
Ca_Kabuli_NC_021163.1_35393534	Ca_kabuli_chr04	35393534	[A/G]	1067296293
Ca_Kabuli_NC_021163.1_35393544	Ca_kabuli_chr04	35393544	[A/G]	1067296294
Ca_Kabuli_NC_021163.1_35393551	Ca_kabuli_chr04	35393551	[A/G]	1067296295
Ca_Kabuli_NC_021163.1_35393565	Ca_kabuli_chr04	35393565	[C/G]	1067296296
Ca_Kabuli_NC_021163.1_35416662	Ca_kabuli_chr04	35416662	[T/C]	1067296297
Ca_Kabuli_NC_021163.1_35455095	Ca_kabuli_chr04	35455095	[A/C]	1067296298
Ca_Kabuli_NC_021163.1_35512265	Ca_kabuli_chr04	35512265	[G/A]	1067296299
Ca_Kabuli_NC_021163.1_35512303	Ca_kabuli_chr04	35512303	[A/G]	1067296300
Ca_Kabuli_NC_021163.1_35517792	Ca_kabuli_chr04	35517792	[C/T]	1067296301
Ca_Kabuli_NC_021163.1_35517793	Ca_kabuli_chr04	35517793	[T/C]	1067296302
Ca_Kabuli_NC_021163.1_35530456	Ca_kabuli_chr04	35530456	[G/A]	1067296303
Ca_Kabuli_NC_021163.1_35574088	Ca_kabuli_chr04	35574088	[G/C]	1067296304
Ca_Kabuli_NC_021163.1_35574338	Ca_kabuli_chr04	35574338	[T/A]	1067296305
Ca_Kabuli_NC_021163.1_35634712	Ca_kabuli_chr04	35634712	[A/G]	1067296306
Ca_Kabuli_NC_021163.1_35634751	Ca_kabuli_chr04	35634751	[A/G]	1067296307
Ca_Kabuli_NC_021163.1_35634873	Ca_kabuli_chr04	35634873	[T/G]	1067296308
Ca_Kabuli_NC_021163.1_35648681	Ca_kabuli_chr04	35648681	[C/T]	1067296309
Ca_Kabuli_NC_021163.1_35652742	Ca_kabuli_chr04	35652742	[T/C]	1067296310
Ca_Kabuli_NC_021163.1_35961593	Ca_kabuli_chr04	35961593	[A/C]	1067296311
Ca_Kabuli_NC_021163.1_35961617	Ca_kabuli_chr04	35961617	[A/G]	1067296312
Ca_Kabuli_NC_021163.1_35977422	Ca_kabuli_chr04	35977422	[G/A]	1067296313
Ca_Kabuli_NC_021163.1_35982209	Ca_kabuli_chr04	35982209	[A/C]	1067296314

SNP IDs	Chromosomes	Physical positions (bp)	SNPs	NCBI SNPdb accession IDs (NCBI_ss#)
Ca_Kabuli_NC_021163.1_36153995	Ca_kabuli_chr04	36153995	[A/G]	1067296315
Ca_Kabuli_NC_021163.1_36154744	Ca_kabuli_chr04	36154744	[T/C]	1067296316
Ca_Kabuli_NC_021163.1_36154786	Ca_kabuli_chr04	36154786	[T/A]	1067296317
Ca_Kabuli_NC_021163.1_36154836	Ca_kabuli_chr04	36154836	[T/G]	1067296318
Ca_Kabuli_NC_021163.1_36154844	Ca_kabuli_chr04	36154844	[T/G]	1067296319
Ca_Kabuli_NC_021163.1_36184787	Ca_kabuli_chr04	36184787	[C/A]	1067296320
Ca_Kabuli_NC_021163.1_36184788	Ca_kabuli_chr04	36184788	[A/G]	1067296321
Ca_Kabuli_NC_021163.1_36276466	Ca_kabuli_chr04	36276466	[T/G]	1067296322
Ca_Kabuli_NC_021163.1_36276514	Ca_kabuli_chr04	36276514	[A/C]	1067296323
Ca_Kabuli_NC_021163.1_36448985	Ca_kabuli_chr04	36448985	[A/C]	1067296324
Ca_Kabuli_NC_021163.1_36464945	Ca_kabuli_chr04	36464945	[T/G]	1067296325
Ca_Kabuli_NC_021163.1_36464998	Ca_kabuli_chr04	36464998	[A/G]	1067296326
Ca_Kabuli_NC_021163.1_36466198	Ca_kabuli_chr04	36466198	[G/A]	1067296327
Ca_Kabuli_NC_021163.1_36466237	Ca_kabuli_chr04	36466237	[G/A]	1067296328
Ca_Kabuli_NC_021163.1_36467980	Ca_kabuli_chr04	36467980	[A/C]	1067296329
Ca_Kabuli_NC_021163.1_36489013	Ca_kabuli_chr04	36489013	[A/G]	1067296330
Ca_Kabuli_NC_021163.1_36537074	Ca_kabuli_chr04	36537074	[T/G]	1067296331
Ca_Kabuli_NC_021163.1_36537161	Ca_kabuli_chr04	36537161	[A/T]	1067296332
Ca_Kabuli_NC_021163.1_36537194	Ca_kabuli_chr04	36537194	[T/G]	1067296333
Ca_Kabuli_NC_021163.1_36537228	Ca_kabuli_chr04	36537228	[T/C]	1067296334
Ca_Kabuli_NC_021163.1_36537249	Ca_kabuli_chr04	36537249	[A/G]	1067296335
Ca_Kabuli_NC_021163.1_36537255	Ca_kabuli_chr04	36537255	[A/G]	1067296336
Ca_Kabuli_NC_021163.1_36541109	Ca_kabuli_chr04	36541109	[A/T]	1067296337
Ca_Kabuli_NC_021163.1_36639382	Ca_kabuli_chr04	36639382	[C/T]	1067296338
Ca_Kabuli_NC_021163.1_36639442	Ca_kabuli_chr04	36639442	[G/A]	1067296339
Ca_Kabuli_NC_021163.1_36645009	Ca_kabuli_chr04	36645009	[T/C]	1067296340
Ca_Kabuli_NC_021163.1_36670226	Ca_kabuli_chr04	36670226	[G/A]	1067296341
Ca_Kabuli_NC_021163.1_36673677	Ca_kabuli_chr04	36673677	[C/A]	1067296342
Ca_Kabuli_NC_021163.1_36677713	Ca_kabuli_chr04	36677713	[C/G]	1067296343
Ca_Kabuli_NC_021163.1_36712806	Ca_kabuli_chr04	36712806	[C/T]	1067296344
Ca_Kabuli_NC_021163.1_36738213	Ca_kabuli_chr04	36738213	[C/G]	1067296345
Ca_Kabuli_NC_021163.1_36738233	Ca_kabuli_chr04	36738233	[A/G]	1067296346
Ca_Kabuli_NC_021163.1_36738433	Ca_kabuli_chr04	36738433	[T/A]	1067296347
Ca_Kabuli_NC_021163.1_36753781	Ca_kabuli_chr04	36753781	[C/T]	1067296348

SNP IDs	Chromosomes	Physical positions (bp)	SNPs	NCBI SNPdb accession IDs (NCBI_ss#)
Ca_Kabuli_NC_021163.1_36753800	Ca_kabuli_chr04	36753800	[A/G]	1067296349
Ca_Kabuli_NC_021163.1_36753988	Ca_kabuli_chr04	36753988	[A/G]	1067296350
Ca_Kabuli_NC_021163.1_36754016	Ca_kabuli_chr04	36754016	[C/T]	1067296351
Ca_Kabuli_NC_021163.1_36754377	Ca_kabuli_chr04	36754377	[T/A]	1067296352
Ca_Kabuli_NC_021163.1_36754378	Ca_kabuli_chr04	36754378	[C/T]	1067296353
Ca_Kabuli_NC_021163.1_36754542	Ca_kabuli_chr04	36754542	[A/T]	1067296354
Ca_Kabuli_NC_021163.1_36756913	Ca_kabuli_chr04	36756913	[A/G]	1067296355
Ca_Kabuli_NC_021163.1_36799668	Ca_kabuli_chr04	36799668	[T/G]	1067296356
Ca_Kabuli_NC_021163.1_36799701	Ca_kabuli_chr04	36799701	[A/G]	1067296357
Ca_Kabuli_NC_021163.1_36831273	Ca_kabuli_chr04	36831273	[C/T]	1067296358
Ca_Kabuli_NC_021163.1_36831477	Ca_kabuli_chr04	36831477	[G/C]	1067296359
Ca_Kabuli_NC_021163.1_36831478	Ca_kabuli_chr04	36831478	[G/T]	1067296360
Ca_Kabuli_NC_021163.1_36834689	Ca_kabuli_chr04	36834689	[T/A]	1067296361
Ca_Kabuli_NC_021163.1_36844197	Ca_kabuli_chr04	36844197	[A/G]	1067296362
Ca_Kabuli_NC_021163.1_36934733	Ca_kabuli_chr04	36934733	[A/G]	1067296363
Ca_Kabuli_NC_021163.1_36975360	Ca_kabuli_chr04	36975360	[G/A]	1067296364
Ca_Kabuli_NC_021163.1_36975361	Ca_kabuli_chr04	36975361	[C/A]	1067296365
Ca_Kabuli_NC_021163.1_36977253	Ca_kabuli_chr04	36977253	[A/G]	1067296366
Ca_Kabuli_NC_021163.1_36979926	Ca_kabuli_chr04	36979926	[G/T]	1067296367
Ca_Kabuli_NC_021163.1_36984727	Ca_kabuli_chr04	36984727	[G/C]	1067296368
Ca_Kabuli_NC_021163.1_36984795	Ca_kabuli_chr04	36984795	[A/T]	1067296369
Ca_Kabuli_NC_021163.1_37006188	Ca_kabuli_chr04	37006188	[A/G]	1067296370
Ca_Kabuli_NC_021163.1_37010914	Ca_kabuli_chr04	37010914	[A/G]	1067296371
Ca_Kabuli_NC_021163.1_37010940	Ca_kabuli_chr04	37010940	[G/A]	1067296372
Ca_Kabuli_NC_021163.1_37010957	Ca_kabuli_chr04	37010957	[A/G]	1067296373
Ca_Kabuli_NC_021163.1_37186531	Ca_kabuli_chr04	37186531	[C/T]	1067296374
Ca_Kabuli_NC_021163.1_37241235	Ca_kabuli_chr04	37241235	[G/A]	1067296375
Ca_Kabuli_NC_021163.1_37241491	Ca_kabuli_chr04	37241491	[G/A]	1067296376
Ca_Kabuli_NC_021163.1_37243069	Ca_kabuli_chr04	37243069	[T/C]	1067296377
Ca_Kabuli_NC_021163.1_37243095	Ca_kabuli_chr04	37243095	[T/A]	1067296378
Ca_Kabuli_NC_021163.1_37243121	Ca_kabuli_chr04	37243121	[A/G]	1067296379
Ca_Kabuli_NC_021163.1_37243129	Ca_kabuli_chr04	37243129	[A/C]	1067296380
Ca_Kabuli_NC_021163.1_37273166	Ca_kabuli_chr04	37273166	[T/C]	1067296381
Ca_Kabuli_NC_021163.1_37293773	Ca_kabuli_chr04	37293773	[T/A]	1067296382

SNP IDs	Chromosomes	Physical positions (bp)	SNPs	NCBI SNPdb accession IDs (NCBI_ss#)
Ca_Kabuli_NC_021163.1_37316713	Ca_kabuli_chr04	37316713	[A/T]	1067296383
Ca_Kabuli_NC_021163.1_37316734	Ca_kabuli_chr04	37316734	[A/C]	1067296384
Ca_Kabuli_NC_021163.1_37316738	Ca_kabuli_chr04	37316738	[C/T]	1067296385
Ca_Kabuli_NC_021163.1_37329491	Ca_kabuli_chr04	37329491	[T/C]	1067296386
Ca_Kabuli_NC_021163.1_37330335	Ca_kabuli_chr04	37330335	[G/T]	1067296387
Ca_Kabuli_NC_021163.1_37330337	Ca_kabuli_chr04	37330337	[G/T]	1067296388
Ca_Kabuli_NC_021163.1_37330358	Ca_kabuli_chr04	37330358	[A/C]	1067296389
Ca_Kabuli_NC_021163.1_37348360	Ca_kabuli_chr04	37348360	[A/C]	1067296390
Ca_Kabuli_NC_021163.1_37349160	Ca_kabuli_chr04	37349160	[A/T]	1067296391
Ca_Kabuli_NC_021163.1_37349161	Ca_kabuli_chr04	37349161	[T/C]	1067296392
Ca_Kabuli_NC_021163.1_37349162	Ca_kabuli_chr04	37349162	[G/T]	1067296393
Ca_Kabuli_NC_021163.1_37349212	Ca_kabuli_chr04	37349212	[C/A]	1067296394
Ca_Kabuli_NC_021163.1_37356889	Ca_kabuli_chr04	37356889	[C/T]	1067296395
Ca_Kabuli_NC_021163.1_37356892	Ca_kabuli_chr04	37356892	[T/C]	1067296396
Ca_Kabuli_NC_021163.1_37356919	Ca_kabuli_chr04	37356919	[T/A]	1067296397
Ca_Kabuli_NC_021163.1_37356951	Ca_kabuli_chr04	37356951	[C/T]	1067296398
Ca_Kabuli_NC_021163.1_37359987	Ca_kabuli_chr04	37359987	[A/T]	1067296399
Ca_Kabuli_NC_021163.1_37360011	Ca_kabuli_chr04	37360011	[G/T]	1067296400
Ca_Kabuli_NC_021163.1_37364980	Ca_kabuli_chr04	37364980	[A/G]	1067296401
Ca_Kabuli_NC_021163.1_37371411	Ca_kabuli_chr04	37371411	[C/T]	1067296402
Ca_Kabuli_NC_021163.1_37433440	Ca_kabuli_chr04	37433440	[G/T]	1067296403
Ca_Kabuli_NC_021163.1_37437877	Ca_kabuli_chr04	37437877	[A/C]	1067296404
Ca_Kabuli_NC_021163.1_37440652	Ca_kabuli_chr04	37440652	[G/A]	1067296405
Ca_Kabuli_NC_021163.1_37449545	Ca_kabuli_chr04	37449545	[C/T]	1067296406
Ca_Kabuli_NC_021163.1_37450206	Ca_kabuli_chr04	37450206	[C/A]	1067296407
Ca_Kabuli_NC_021163.1_37450294	Ca_kabuli_chr04	37450294	[C/T]	1067296408
Ca_Kabuli_NC_021163.1_37450306	Ca_kabuli_chr04	37450306	[A/C]	1067296409
Ca_Kabuli_NC_021163.1_37450326	Ca_kabuli_chr04	37450326	[T/A]	1067296410
Ca_Kabuli_NC_021163.1_37453497	Ca_kabuli_chr04	37453497	[C/G]	1067296411
Ca_Kabuli_NC_021163.1_37453540	Ca_kabuli_chr04	37453540	[T/C]	1067296412
Ca_Kabuli_NC_021163.1_37453578	Ca_kabuli_chr04	37453578	[A/G]	1067296413
Ca_Kabuli_NC_021163.1_37453643	Ca_kabuli_chr04	37453643	[G/C]	1067296414
Ca_Kabuli_NC_021163.1_37454296	Ca_kabuli_chr04	37454296	[A/G]	1067296415
Ca_Kabuli_NC_021163.1_37454374	Ca_kabuli_chr04	37454374	[C/T]	1067296416

SNP IDs	Chromosomes	Physical positions (bp)	SNPs	NCBI SNPdb accession IDs (NCBI_ss#)
Ca_Kabuli_NC_021163.1_37454636	Ca_kabuli_chr04	37454636	[A/G]	1067296417
Ca_Kabuli_NC_021163.1_37454675	Ca_kabuli_chr04	37454675	[T/G]	1067296418
Ca_Kabuli_NC_021163.1_37455038	Ca_kabuli_chr04	37455038	[A/C]	1067296419
Ca_Kabuli_NC_021163.1_37549862	Ca_kabuli_chr04	37549862	[T/G]	1067296420
Ca_Kabuli_NC_021163.1_37549883	Ca_kabuli_chr04	37549883	[T/C]	1067296421
Ca_Kabuli_NC_021163.1_37550161	Ca_kabuli_chr04	37550161	[T/G]	1067296422
Ca_Kabuli_NC_021163.1_37570104	Ca_kabuli_chr04	37570104	[G/T]	1067296423
Ca_Kabuli_NC_021163.1_37574409	Ca_kabuli_chr04	37574409	[G/A]	1067296424
Ca_Kabuli_NC_021163.1_37576578	Ca_kabuli_chr04	37576578	[A/C]	1067296425
Ca_Kabuli_NC_021163.1_37630404	Ca_kabuli_chr04	37630404	[A/G]	1067296426
Ca_Kabuli_NC_021163.1_37630445	Ca_kabuli_chr04	37630445	[A/G]	1067296427
Ca_Kabuli_NC_021163.1_37632954	Ca_kabuli_chr04	37632954	[A/T]	1067296428
Ca_Kabuli_NC_021163.1_37632972	Ca_kabuli_chr04	37632972	[C/T]	1067296429
Ca_Kabuli_NC_021163.1_37632973	Ca_kabuli_chr04	37632973	[G/A]	1067296430
Ca_Kabuli_NC_021163.1_37633044	Ca_kabuli_chr04	37633044	[C/T]	1067296431
Ca_Kabuli_NC_021163.1_37650745	Ca_kabuli_chr04	37650745	[A/G]	1067296432
Ca_Kabuli_NC_021163.1_37650783	Ca_kabuli_chr04	37650783	[C/A]	1067296433
Ca_Kabuli_NC_021163.1_37688124	Ca_kabuli_chr04	37688124	[G/T]	1067296434
Ca_Kabuli_NC_021163.1_37688361	Ca_kabuli_chr04	37688361	[G/T]	1067296435
Ca_Kabuli_NC_021163.1_37690030	Ca_kabuli_chr04	37690030	[A/G]	1067296436
Ca_Kabuli_NC_021163.1_37690366	Ca_kabuli_chr04	37690366	[A/G]	1067296437
Ca_Kabuli_NC_021163.1_37698067	Ca_kabuli_chr04	37698067	[T/C]	1067296438
Ca_Kabuli_NC_021163.1_37698508	Ca_kabuli_chr04	37698508	[T/G]	1067296439
Ca_Kabuli_NC_021163.1_37698673	Ca_kabuli_chr04	37698673	[C/A]	1067296440
Ca_Kabuli_NC_021163.1_37699173	Ca_kabuli_chr04	37699173	[A/C]	1067296441
Ca_Kabuli_NC_021163.1_37699981	Ca_kabuli_chr04	37699981	[G/T]	1067296442
Ca_Kabuli_NC_021163.1_37706480	Ca_kabuli_chr04	37706480	[T/A]	1067296443
Ca_Kabuli_NC_021163.1_37706496	Ca_kabuli_chr04	37706496	[G/A]	1067296444
Ca_Kabuli_NC_021163.1_37706498	Ca_kabuli_chr04	37706498	[T/G]	1067296445
Ca_Kabuli_NC_021163.1_37708217	Ca_kabuli_chr04	37708217	[A/C]	1067296446
Ca_Kabuli_NC_021163.1_37708220	Ca_kabuli_chr04	37708220	[A/C]	1067296447
Ca_Kabuli_NC_021163.1_37708249	Ca_kabuli_chr04	37708249	[G/T]	1067296448
Ca_Kabuli_NC_021163.1_37708256	Ca_kabuli_chr04	37708256	[A/G]	1067296449
Ca_Kabuli_NC_021163.1_37723097	Ca_kabuli_chr04	37723097	[T/G]	1067296450

SNP IDs	Chromosomes	Physical positions (bp)	SNPs	NCBI SNPdb accession IDs (NCBI_ss#)
Ca_Kabuli_NC_021163.1_37754127	Ca_kabuli_chr04	37754127	[C/T]	1067296451
Ca_Kabuli_NC_021163.1_37754128	Ca_kabuli_chr04	37754128	[T/C]	1067296452
Ca_Kabuli_NC_021163.1_37754129	Ca_kabuli_chr04	37754129	[G/T]	1067296453
Ca_Kabuli_NC_021163.1_37797651	Ca_kabuli_chr04	37797651	[C/G]	1067296454
Ca_Kabuli_NC_021163.1_37797676	Ca_kabuli_chr04	37797676	[T/C]	1067296455
Ca_Kabuli_NC_021163.1_37797966	Ca_kabuli_chr04	37797966	[T/G]	1067296456
Ca_Kabuli_NC_021163.1_37806689	Ca_kabuli_chr04	37806689	[T/C]	1067296457
Ca_Kabuli_NC_021163.1_37858969	Ca_kabuli_chr04	37858969	[T/G]	1067296458
Ca_Kabuli_NC_021163.1_37859003	Ca_kabuli_chr04	37859003	[T/C]	1067296459
Ca_Kabuli_NC_021163.1_37859011	Ca_kabuli_chr04	37859011	[A/G]	1067296460
Ca_Kabuli_NC_021163.1_37897351	Ca_kabuli_chr04	37897351	[C/A]	1067296461
Ca_Kabuli_NC_021163.1_37898485	Ca_kabuli_chr04	37898485	[G/T]	1067296462
Ca_Kabuli_NC_021163.1_37899646	Ca_kabuli_chr04	37899646	[A/G]	1067296463
Ca_Kabuli_NC_021163.1_37899651	Ca_kabuli_chr04	37899651	[T/C]	1067296464
Ca_Kabuli_NC_021163.1_38008062	Ca_kabuli_chr04	38008062	[A/T]	1067296465
Ca_Kabuli_NC_021163.1_38008082	Ca_kabuli_chr04	38008082	[C/T]	1067296466
Ca_Kabuli_NC_021163.1_38008205	Ca_kabuli_chr04	38008205	[G/A]	1067296467
Ca_Kabuli_NC_021163.1_38008227	Ca_kabuli_chr04	38008227	[G/T]	1067296468
Ca_Kabuli_NC_021163.1_38008527	Ca_kabuli_chr04	38008527	[A/T]	1067296469
Ca_Kabuli_NC_021163.1_38008567	Ca_kabuli_chr04	38008567	[C/A]	1067296470
Ca_Kabuli_NC_021163.1_38029136	Ca_kabuli_chr04	38029136	[G/T]	1067296471
Ca_Kabuli_NC_021163.1_38029208	Ca_kabuli_chr04	38029208	[G/A]	1067296472
Ca_Kabuli_NC_021163.1_38029215	Ca_kabuli_chr04	38029215	[A/T]	1067296473
Ca_Kabuli_NC_021163.1_38029358	Ca_kabuli_chr04	38029358	[A/G]	1067296474
Ca_Kabuli_NC_021163.1_38029380	Ca_kabuli_chr04	38029380	[A/G]	1067296475
Ca_Kabuli_NC_021163.1_38038959	Ca_kabuli_chr04	38038959	[G/A]	1067296476
Ca_Kabuli_NC_021163.1_38039046	Ca_kabuli_chr04	38039046	[T/C]	1067296477
Ca_Kabuli_NC_021163.1_38039251	Ca_kabuli_chr04	38039251	[G/C]	1067296478
Ca_Kabuli_NC_021163.1_38039289	Ca_kabuli_chr04	38039289	[G/A]	1067296479
Ca_Kabuli_NC_021163.1_38060338	Ca_kabuli_chr04	38060338	[T/A]	1067296480
Ca_Kabuli_NC_021163.1_38060368	Ca_kabuli_chr04	38060368	[G/C]	1067296481
Ca_Kabuli_NC_021163.1_38153183	Ca_kabuli_chr04	38153183	[A/C]	1067296482
Ca_Kabuli_NC_021163.1_38153198	Ca_kabuli_chr04	38153198	[T/G]	1067296483
Ca_Kabuli_NC_021163.1_38153216	Ca_kabuli_chr04	38153216	[C/T]	1067296484

SNP IDs	Chromosomes	Physical positions (bp)	SNPs	NCBI SNPdb accession IDs (NCBI_ss#)
Ca_Kabuli_NC_021163.1_38157210	Ca_kabuli_chr04	38157210	[T/G]	1067296485
Ca_Kabuli_NC_021163.1_38157279	Ca_kabuli_chr04	38157279	[T/A]	1067296486
Ca_Kabuli_NC_021163.1_38172943	Ca_kabuli_chr04	38172943	[A/T]	1067296487
Ca_Kabuli_NC_021163.1_38179583	Ca_kabuli_chr04	38179583	[A/C]	1067296488
Ca_Kabuli_NC_021163.1_38181292	Ca_kabuli_chr04	38181292	[G/A]	1067296489
Ca_Kabuli_NC_021163.1_38181893	Ca_kabuli_chr04	38181893	[G/T]	1067296490
Ca_Kabuli_NC_021163.1_38194159	Ca_kabuli_chr04	38194159	[T/C]	1067296491
Ca_Kabuli_NC_021163.1_38195346	Ca_kabuli_chr04	38195346	[A/G]	1067296492
Ca_Kabuli_NC_021163.1_38215267	Ca_kabuli_chr04	38215267	[A/G]	1067296493
Ca_Kabuli_NC_021163.1_38226374	Ca_kabuli_chr04	38226374	[C/A]	1067296494
Ca_Kabuli_NC_021163.1_38246261	Ca_kabuli_chr04	38246261	[A/C]	1067296495
Ca_Kabuli_NC_021163.1_38246303	Ca_kabuli_chr04	38246303	[A/T]	1067296496
Ca_Kabuli_NC_021163.1_38246306	Ca_kabuli_chr04	38246306	[G/T]	1067296497
Ca_Kabuli_NC_021163.1_38246739	Ca_kabuli_chr04	38246739	[T/G]	1067296498
Ca_Kabuli_NC_021163.1_38246753	Ca_kabuli_chr04	38246753	[A/G]	1067296499
Ca_Kabuli_NC_021163.1_38246775	Ca_kabuli_chr04	38246775	[C/A]	1067296500
Ca_Kabuli_NC_021163.1_38288823	Ca_kabuli_chr04	38288823	[T/G]	1067296501
Ca_Kabuli_NC_021163.1_38289052	Ca_kabuli_chr04	38289052	[A/G]	1067296502
Ca_Kabuli_NC_021163.1_38289066	Ca_kabuli_chr04	38289066	[A/G]	1067296503
Ca_Kabuli_NC_021163.1_38302165	Ca_kabuli_chr04	38302165	[A/T]	1067296504
Ca_Kabuli_NC_021163.1_38302413	Ca_kabuli_chr04	38302413	[G/A]	1067296505
Ca_Kabuli_NC_021163.1_38302445	Ca_kabuli_chr04	38302445	[G/A]	1067296506
Ca_Kabuli_NC_021163.1_38323250	Ca_kabuli_chr04	38323250	[T/A]	1067296507
Ca_Kabuli_NC_021163.1_38323321	Ca_kabuli_chr04	38323321	[T/G]	1067296508
Ca_Kabuli_NC_021163.1_38323345	Ca_kabuli_chr04	38323345	[T/A]	1067296509
Ca_Kabuli_NC_021163.1_38343796	Ca_kabuli_chr04	38343796	[T/C]	1067296510
Ca_Kabuli_NC_021163.1_38358096	Ca_kabuli_chr04	38358096	[G/T]	1067296511
Ca_Kabuli_NC_021163.1_38358129	Ca_kabuli_chr04	38358129	[G/A]	1067296512
Ca_Kabuli_NC_021163.1_38358146	Ca_kabuli_chr04	38358146	[A/C]	1067296513
Ca_Kabuli_NC_021163.1_38358398	Ca_kabuli_chr04	38358398	[A/G]	1067296514
Ca_Kabuli_NC_021163.1_38358672	Ca_kabuli_chr04	38358672	[T/G]	1067296515
Ca_Kabuli_NC_021163.1_38358798	Ca_kabuli_chr04	38358798	[T/A]	1067296516
Ca_Kabuli_NC_021163.1_38358829	Ca_kabuli_chr04	38358829	[A/G]	1067296517
Ca_Kabuli_NC_021163.1_38358849	Ca_kabuli_chr04	38358849	[T/C]	1067296518

SNP IDs	Chromosomes	Physical positions (bp)	SNPs	NCBI SNPdb accession IDs (NCBI_ss#)
Ca_Kabuli_NC_021163.1_38359493	Ca_kabuli_chr04	38359493	[T/G]	1067296519
Ca_Kabuli_NC_021163.1_38362132	Ca_kabuli_chr04	38362132	[A/T]	1067296520
Ca_Kabuli_NC_021163.1_38363041	Ca_kabuli_chr04	38363041	[C/T]	1067296521
Ca_Kabuli_NC_021163.1_38370768	Ca_kabuli_chr04	38370768	[A/C]	1067296522
Ca_Kabuli_NC_021163.1_38370848	Ca_kabuli_chr04	38370848	[A/G]	1067296523
Ca_Kabuli_NC_021163.1_38370926	Ca_kabuli_chr04	38370926	[T/C]	1067296524
Ca_Kabuli_NC_021163.1_38370939	Ca_kabuli_chr04	38370939	[C/A]	1067296525
Ca_Kabuli_NC_021163.1_38370941	Ca_kabuli_chr04	38370941	[A/G]	1067296526
Ca_Kabuli_NC_021163.1_38381635	Ca_kabuli_chr04	38381635	[C/T]	1067296527
Ca_Kabuli_NC_021163.1_38381646	Ca_kabuli_chr04	38381646	[G/A]	1067296528
Ca_Kabuli_NC_021163.1_38382829	Ca_kabuli_chr04	38382829	[A/C]	1067296529
Ca_Kabuli_NC_021163.1_38382947	Ca_kabuli_chr04	38382947	[C/A]	1067296530
Ca_Kabuli_NC_021163.1_38405153	Ca_kabuli_chr04	38405153	[C/T]	1067296531
Ca_Kabuli_NC_021163.1_38421833	Ca_kabuli_chr04	38421833	[A/G]	1067296532
Ca_Kabuli_NC_021163.1_38422037	Ca_kabuli_chr04	38422037	[T/C]	1067296533
Ca_Kabuli_NC_021163.1_38425720	Ca_kabuli_chr04	38425720	[T/A]	1067296534
Ca_Kabuli_NC_021163.1_38474093	Ca_kabuli_chr04	38474093	[C/T]	1067296535
Ca_Kabuli_NC_021163.1_38474108	Ca_kabuli_chr04	38474108	[T/C]	1067296536
Ca_Kabuli_NC_021163.1_38474123	Ca_kabuli_chr04	38474123	[A/T]	1067296537
Ca_Kabuli_NC_021163.1_38474149	Ca_kabuli_chr04	38474149	[T/C]	1067296538
Ca_Kabuli_NC_021163.1_38475310	Ca_kabuli_chr04	38475310	[A/C]	1067296539
Ca_Kabuli_NC_021163.1_38475425	Ca_kabuli_chr04	38475425	[G/A]	1067296540
Ca_Kabuli_NC_021163.1_38475449	Ca_kabuli_chr04	38475449	[T/A]	1067296541
Ca_Kabuli_NC_021163.1_38475547	Ca_kabuli_chr04	38475547	[C/T]	1067296542
Ca_Kabuli_NC_021163.1_38534421	Ca_kabuli_chr04	38534421	[T/C]	1067296543
Ca_Kabuli_NC_021163.1_38536170	Ca_kabuli_chr04	38536170	[A/G]	1067296544
Ca_Kabuli_NC_021163.1_38536332	Ca_kabuli_chr04	38536332	[A/C]	1067296545
Ca_Kabuli_NC_021163.1_38538925	Ca_kabuli_chr04	38538925	[G/T]	1067296546
Ca_Kabuli_NC_021163.1_38540247	Ca_kabuli_chr04	38540247	[G/A]	1067296547
Ca_Kabuli_NC_021163.1_38592107	Ca_kabuli_chr04	38592107	[T/G]	1067296548
Ca_Kabuli_NC_021163.1_38592115	Ca_kabuli_chr04	38592115	[A/G]	1067296549
Ca_Kabuli_NC_021163.1_38659536	Ca_kabuli_chr04	38659536	[T/A]	1067296550
Ca_Kabuli_NC_021163.1_38659625	Ca_kabuli_chr04	38659625	[A/T]	1067296551
Ca_Kabuli_NC_021163.1_38659636	Ca_kabuli_chr04	38659636	[T/G]	1067296552

SNP IDs	Chromosomes	Physical positions (bp)	SNPs	NCBI SNPdb accession IDs (NCBI_ss#)
Ca_Kabuli_NC_021163.1_38668599	Ca_kabuli_chr04	38668599	[A/T]	1067296553
Ca_Kabuli_NC_021163.1_38673440	Ca_kabuli_chr04	38673440	[A/G]	1067296554
Ca_Kabuli_NC_021163.1_38673445	Ca_kabuli_chr04	38673445	[A/G]	1067296555
Ca_Kabuli_NC_021163.1_38673456	Ca_kabuli_chr04	38673456	[T/C]	1067296556
Ca_Kabuli_NC_021163.1_38673464	Ca_kabuli_chr04	38673464	[A/G]	1067296557
Ca_Kabuli_NC_021163.1_38673483	Ca_kabuli_chr04	38673483	[A/G]	1067296558
Ca_Kabuli_NC_021163.1_38673501	Ca_kabuli_chr04	38673501	[T/C]	1067296559
Ca_Kabuli_NC_021163.1_38679967	Ca_kabuli_chr04	38679967	[T/C]	1067296560
Ca_Kabuli_NC_021163.1_38679991	Ca_kabuli_chr04	38679991	[T/C]	1067296561
Ca_Kabuli_NC_021163.1_38680297	Ca_kabuli_chr04	38680297	[A/C]	1067296562
Ca_Kabuli_NC_021163.1_38680306	Ca_kabuli_chr04	38680306	[A/C]	1067296563
Ca_Kabuli_NC_021163.1_38721160	Ca_kabuli_chr04	38721160	[A/G]	1067296564
Ca_Kabuli_NC_021163.1_38721207	Ca_kabuli_chr04	38721207	[A/C]	1067296565
Ca_Kabuli_NC_021163.1_38724356	Ca_kabuli_chr04	38724356	[C/A]	1067296566
Ca_Kabuli_NC_021163.1_38756507	Ca_kabuli_chr04	38756507	[T/C]	1067296567
Ca_Kabuli_NC_021163.1_38756510	Ca_kabuli_chr04	38756510	[C/G]	1067296568
Ca_Kabuli_NC_021163.1_38759544	Ca_kabuli_chr04	38759544	[T/G]	1067296569
Ca_Kabuli_NC_021163.1_38770348	Ca_kabuli_chr04	38770348	[T/A]	1067296570
Ca_Kabuli_NC_021163.1_38770450	Ca_kabuli_chr04	38770450	[C/G]	1067296571
Ca_Kabuli_NC_021163.1_38785451	Ca_kabuli_chr04	38785451	[A/C]	1067296572
Ca_Kabuli_NC_021163.1_38829961	Ca_kabuli_chr04	38829961	[A/T]	1067296573
Ca_Kabuli_NC_021163.1_38829998	Ca_kabuli_chr04	38829998	[C/G]	1067296574
Ca_Kabuli_NC_021163.1_38833182	Ca_kabuli_chr04	38833182	[T/G]	1067296575
Ca_Kabuli_NC_021163.1_38833280	Ca_kabuli_chr04	38833280	[T/C]	1067296576
Ca_Kabuli_NC_021163.1_38833304	Ca_kabuli_chr04	38833304	[C/A]	1067296577
Ca_Kabuli_NC_021163.1_38833521	Ca_kabuli_chr04	38833521	[G/A]	1067296578
Ca_Kabuli_NC_021163.1_38834474	Ca_kabuli_chr04	38834474	[G/A]	1067296579
Ca_Kabuli_NC_021163.1_38834477	Ca_kabuli_chr04	38834477	[G/A]	1067296580
Ca_Kabuli_NC_021163.1_38836356	Ca_kabuli_chr04	38836356	[T/C]	1067296581
Ca_Kabuli_NC_021163.1_38836381	Ca_kabuli_chr04	38836381	[T/A]	1067296582
Ca_Kabuli_NC_021163.1_38836398	Ca_kabuli_chr04	38836398	[T/C]	1067296583
Ca_Kabuli_NC_021163.1_38836436	Ca_kabuli_chr04	38836436	[T/G]	1067296584
Ca_Kabuli_NC_021163.1_38836454	Ca_kabuli_chr04	38836454	[A/C]	1067296585
Ca_Kabuli_NC_021163.1_38836455	Ca_kabuli_chr04	38836455	[A/C]	1067296586

SNP IDs	Chromosomes	Physical positions (bp)	SNPs	NCBI SNPdb accession IDs (NCBI_ss#)
Ca_Kabuli_NC_021163.1_38836457	Ca_kabuli_chr04	38836457	[A/T]	1067296587
Ca_Kabuli_NC_021163.1_38836649	Ca_kabuli_chr04	38836649	[T/C]	1067296588
Ca_Kabuli_NC_021163.1_38888779	Ca_kabuli_chr04	38888779	[T/C]	1067296589
Ca_Kabuli_NC_021163.1_38888791	Ca_kabuli_chr04	38888791	[C/G]	1067296590
Ca_Kabuli_NC_021163.1_38888936	Ca_kabuli_chr04	38888936	[T/G]	1067296591
Ca_Kabuli_NC_021163.1_38888996	Ca_kabuli_chr04	38888996	[T/C]	1067296592
Ca_Kabuli_NC_021163.1_38918394	Ca_kabuli_chr04	38918394	[T/A]	1067296593
Ca_Kabuli_NC_021163.1_38918424	Ca_kabuli_chr04	38918424	[A/G]	1067296594
Ca_Kabuli_NC_021163.1_38918724	Ca_kabuli_chr04	38918724	[C/A]	1067296595
Ca_Kabuli_NC_021163.1_38928945	Ca_kabuli_chr04	38928945	[G/C]	1067296596
Ca_Kabuli_NC_021163.1_38929639	Ca_kabuli_chr04	38929639	[T/A]	1067296597
Ca_Kabuli_NC_021163.1_38929681	Ca_kabuli_chr04	38929681	[A/G]	1067296598
Ca_Kabuli_NC_021163.1_38948380	Ca_kabuli_chr04	38948380	[A/G]	1067296599
Ca_Kabuli_NC_021163.1_38948391	Ca_kabuli_chr04	38948391	[A/C]	1067296600
Ca_Kabuli_NC_021163.1_38948405	Ca_kabuli_chr04	38948405	[C/A]	1067296601
Ca_Kabuli_NC_021163.1_38948407	Ca_kabuli_chr04	38948407	[G/C]	1067296602
Ca_Kabuli_NC_021163.1_38976822	Ca_kabuli_chr04	38976822	[C/A]	1067296603
Ca_Kabuli_NC_021163.1_38976835	Ca_kabuli_chr04	38976835	[G/T]	1067296604
Ca_Kabuli_NC_021163.1_38978405	Ca_kabuli_chr04	38978405	[A/G]	1067296605
Ca_Kabuli_NC_021163.1_38978417	Ca_kabuli_chr04	38978417	[T/A]	1067296606
Ca_Kabuli_NC_021163.1_38978427	Ca_kabuli_chr04	38978427	[A/G]	1067296607
Ca_Kabuli_NC_021163.1_38978435	Ca_kabuli_chr04	38978435	[A/G]	1067296608
Ca_Kabuli_NC_021163.1_38978466	Ca_kabuli_chr04	38978466	[T/A]	1067296609
Ca_Kabuli_NC_021163.1_38979793	Ca_kabuli_chr04	38979793	[T/A]	1067296610
Ca_Kabuli_NC_021163.1_39033052	Ca_kabuli_chr04	39033052	[A/G]	1067296611
Ca_Kabuli_NC_021163.1_39033073	Ca_kabuli_chr04	39033073	[G/C]	1067296612
Ca_Kabuli_NC_021163.1_39033314	Ca_kabuli_chr04	39033314	[C/G]	1067296613
Ca_Kabuli_NC_021163.1_39033838	Ca_kabuli_chr04	39033838	[C/A]	1067296614
Ca_Kabuli_NC_021163.1_39042794	Ca_kabuli_chr04	39042794	[A/G]	1067296615
Ca_Kabuli_NC_021163.1_39042938	Ca_kabuli_chr04	39042938	[T/G]	1067296616
Ca_Kabuli_NC_021163.1_39047261	Ca_kabuli_chr04	39047261	[C/T]	1067296617
Ca_Kabuli_NC_021163.1_39052494	Ca_kabuli_chr04	39052494	[A/G]	1067296618
Ca_Kabuli_NC_021163.1_39081951	Ca_kabuli_chr04	39081951	[A/G]	1067296619
Ca_Kabuli_NC_021163.1_39085061	Ca_kabuli_chr04	39085061	[A/G]	1067296620

SNP IDs	Chromosomes	Physical positions (bp)	SNPs	NCBI SNPdb accession IDs (NCBI_ss#)
Ca_Kabuli_NC_021163.1_39085068	Ca_kabuli_chr04	39085068	[A/G]	1067296621
Ca_Kabuli_NC_021163.1_39086017	Ca_kabuli_chr04	39086017	[A/T]	1067296622
Ca_Kabuli_NC_021163.1_39086269	Ca_kabuli_chr04	39086269	[G/A]	1067296623
Ca_Kabuli_NC_021163.1_39110692	Ca_kabuli_chr04	39110692	[A/G]	1067296624
Ca_Kabuli_NC_021163.1_39116656	Ca_kabuli_chr04	39116656	[A/G]	1067296625
Ca_Kabuli_NC_021163.1_39116795	Ca_kabuli_chr04	39116795	[C/G]	1067296626
Ca_Kabuli_NC_021163.1_39136826	Ca_kabuli_chr04	39136826	[C/T]	1067296627
Ca_Kabuli_NC_021163.1_39635719	Ca_kabuli_chr04	39635719	[G/T]	1067296628
Ca_Kabuli_NC_021163.1_39635734	Ca_kabuli_chr04	39635734	[A/C]	1067296629
Ca_Kabuli_NC_021163.1_39655306	Ca_kabuli_chr04	39655306	[G/A]	1067296630
Ca_Kabuli_NC_021163.1_39715083	Ca_kabuli_chr04	39715083	[A/G]	1067296631
Ca_Kabuli_NC_021163.1_39715112	Ca_kabuli_chr04	39715112	[T/C]	1067296632
Ca_Kabuli_NC_021163.1_39718739	Ca_kabuli_chr04	39718739	[A/C]	1067296633
Ca_Kabuli_NC_021163.1_39719808	Ca_kabuli_chr04	39719808	[A/C]	1067296634
Ca_Kabuli_NC_021163.1_39803289	Ca_kabuli_chr04	39803289	[A/C]	1067296635
Ca_Kabuli_NC_021163.1_39849639	Ca_kabuli_chr04	39849639	[T/G]	1067296636
Ca_Kabuli_NC_021163.1_40010143	Ca_kabuli_chr04	40010143	[A/C]	1067296637
Ca_Kabuli_NC_021163.1_40010165	Ca_kabuli_chr04	40010165	[A/C]	1067296638
Ca_Kabuli_NC_021163.1_40010180	Ca_kabuli_chr04	40010180	[A/C]	1067296639
Ca_Kabuli_NC_021163.1_40061970	Ca_kabuli_chr04	40061970	[A/C]	1067296640
Ca_Kabuli_NC_021163.1_40159151	Ca_kabuli_chr04	40159151	[A/C]	1067296641
Ca_Kabuli_NC_021163.1_40179388	Ca_kabuli_chr04	40179388	[C/A]	1067296642
Ca_Kabuli_NC_021163.1_40179389	Ca_kabuli_chr04	40179389	[A/G]	1067296643
Ca_Kabuli_NC_021163.1_40349066	Ca_kabuli_chr04	40349066	[A/C]	1067296644
Ca_Kabuli_NC_021163.1_40349094	Ca_kabuli_chr04	40349094	[T/C]	1067296645
Ca_Kabuli_NC_021163.1_40349101	Ca_kabuli_chr04	40349101	[T/C]	1067296646
Ca_Kabuli_NC_021163.1_40349123	Ca_kabuli_chr04	40349123	[T/C]	1067296647
Ca_Kabuli_NC_021163.1_40349143	Ca_kabuli_chr04	40349143	[A/C]	1067296648
Ca_Kabuli_NC_021163.1_40349162	Ca_kabuli_chr04	40349162	[C/G]	1067296649
Ca_Kabuli_NC_021163.1_40349163	Ca_kabuli_chr04	40349163	[A/G]	1067296650
Ca_Kabuli_NC_021163.1_40349186	Ca_kabuli_chr04	40349186	[A/C]	1067296651
Ca_Kabuli_NC_021163.1_40368254	Ca_kabuli_chr04	40368254	[T/G]	1067296652
Ca_Kabuli_NC_021163.1_40375385	Ca_kabuli_chr04	40375385	[A/G]	1067296653
Ca_Kabuli_NC_021163.1_40387949	Ca_kabuli_chr04	40387949	[T/C]	1067296654

SNP IDs	Chromosomes	Physical positions (bp)	SNPs	NCBI SNPdb accession IDs (NCBI_ss#)
Ca_Kabuli_NC_021163.1_40387974	Ca_kabuli_chr04	40387974	[T/C]	1067296655
Ca_Kabuli_NC_021163.1_40429302	Ca_kabuli_chr04	40429302	[T/C]	1067296656
Ca_Kabuli_NC_021163.1_40435006	Ca_kabuli_chr04	40435006	[C/T]	1067296657
Ca_Kabuli_NC_021163.1_40472031	Ca_kabuli_chr04	40472031	[T/C]	1067296658
Ca_Kabuli_NC_021163.1_40481316	Ca_kabuli_chr04	40481316	[T/G]	1067296659
Ca_Kabuli_NC_021163.1_40481319	Ca_kabuli_chr04	40481319	[A/G]	1067296660
Ca_Kabuli_NC_021163.1_40481321	Ca_kabuli_chr04	40481321	[C/T]	1067296661
Ca_Kabuli_NC_021163.1_40481324	Ca_kabuli_chr04	40481324	[C/T]	1067296662
Ca_Kabuli_NC_021163.1_40513601	Ca_kabuli_chr04	40513601	[A/C]	1067296663
Ca_Kabuli_NC_021163.1_40513718	Ca_kabuli_chr04	40513718	[T/G]	1067296664
Ca_Kabuli_NC_021163.1_40513788	Ca_kabuli_chr04	40513788	[A/C]	1067296665
Ca_Kabuli_NC_021163.1_40597195	Ca_kabuli_chr04	40597195	[T/G]	1067296666
Ca_Kabuli_NC_021163.1_40795107	Ca_kabuli_chr04	40795107	[T/C]	1067296667
Ca_Kabuli_NC_021163.1_40801814	Ca_kabuli_chr04	40801814	[A/C]	1067296668
Ca_Kabuli_NC_021163.1_40801824	Ca_kabuli_chr04	40801824	[A/G]	1067296669
Ca_Kabuli_NC_021163.1_40805737	Ca_kabuli_chr04	40805737	[T/C]	1067296670
Ca_Kabuli_NC_021163.1_40805740	Ca_kabuli_chr04	40805740	[C/A]	1067296671
Ca_Kabuli_NC_021163.1_40805952	Ca_kabuli_chr04	40805952	[A/C]	1067296672
Ca_Kabuli_NC_021163.1_40805972	Ca_kabuli_chr04	40805972	[G/A]	1067296673
Ca_Kabuli_NC_021163.1_40816988	Ca_kabuli_chr04	40816988	[T/C]	1067296674
Ca_Kabuli_NC_021163.1_40839857	Ca_kabuli_chr04	40839857	[T/G]	1067296675
Ca_Kabuli_NC_021163.1_40913750	Ca_kabuli_chr04	40913750	[T/C]	1067296676
Ca_Kabuli_NC_021163.1_40913786	Ca_kabuli_chr04	40913786	[A/G]	1067296677
Ca_Kabuli_NC_021163.1_40913801	Ca_kabuli_chr04	40913801	[T/G]	1067296678
Ca_Kabuli_NC_021163.1_40941094	Ca_kabuli_chr04	40941094	[A/T]	1067296679
Ca_Kabuli_NC_021163.1_40941283	Ca_kabuli_chr04	40941283	[A/G]	1067296680
Ca_Kabuli_NC_021163.1_41069987	Ca_kabuli_chr04	41069987	[T/C]	1067296681
Ca_Kabuli_NC_021163.1_41094084	Ca_kabuli_chr04	41094084	[T/G]	1067296682
Ca_Kabuli_NC_021163.1_41094127	Ca_kabuli_chr04	41094127	[T/C]	1067296683
Ca_Kabuli_NC_021163.1_41094150	Ca_kabuli_chr04	41094150	[T/G]	1067296684
Ca_Kabuli_NC_021163.1_41094179	Ca_kabuli_chr04	41094179	[A/G]	1067296685
Ca_Kabuli_NC_021163.1_41094186	Ca_kabuli_chr04	41094186	[A/C]	1067296686
Ca_Kabuli_NC_021163.1_41094723	Ca_kabuli_chr04	41094723	[A/C]	1067296687
Ca_Kabuli_NC_021163.1_41094730	Ca_kabuli_chr04	41094730	[A/C]	1067296688

SNP IDs	Chromosomes	Physical positions (bp)	SNPs	NCBI SNPdb accession IDs (NCBI_ss#)
Ca_Kabuli_NC_021163.1_41094736	Ca_kabuli_chr04	41094736	[A/T]	1067296689
Ca_Kabuli_NC_021163.1_41094904	Ca_kabuli_chr04	41094904	[A/C]	1067296690
Ca_Kabuli_NC_021163.1_41119721	Ca_kabuli_chr04	41119721	[T/G]	1067296691
Ca_Kabuli_NC_021163.1_41119725	Ca_kabuli_chr04	41119725	[A/G]	1067296692
Ca_Kabuli_NC_021163.1_41139450	Ca_kabuli_chr04	41139450	[A/G]	1067296693
Ca_Kabuli_NC_021163.1_41139466	Ca_kabuli_chr04	41139466	[T/C]	1067296694
Ca_Kabuli_NC_021163.1_41139484	Ca_kabuli_chr04	41139484	[A/G]	1067296695
Ca_Kabuli_NC_021163.1_41139606	Ca_kabuli_chr04	41139606	[A/C]	1067296696
Ca_Kabuli_NC_021163.1_41142249	Ca_kabuli_chr04	41142249	[T/C]	1067296697
Ca_Kabuli_NC_021163.1_41145896	Ca_kabuli_chr04	41145896	[T/A]	1067296698
Ca_Kabuli_NC_021163.1_41145941	Ca_kabuli_chr04	41145941	[A/G]	1067296699
Ca_Kabuli_NC_021163.1_41145944	Ca_kabuli_chr04	41145944	[A/C]	1067296700
Ca_Kabuli_NC_021163.1_41207338	Ca_kabuli_chr04	41207338	[T/C]	1067296701
Ca_Kabuli_NC_021163.1_41207368	Ca_kabuli_chr04	41207368	[A/C]	1067296702
Ca_Kabuli_NC_021163.1_41229597	Ca_kabuli_chr04	41229597	[T/A]	1067296703
Ca_Kabuli_NC_021163.1_41280087	Ca_kabuli_chr04	41280087	[A/G]	1067296704
Ca_Kabuli_NC_021163.1_41285837	Ca_kabuli_chr04	41285837	[A/T]	1067296705
Ca_Kabuli_NC_021163.1_41285842	Ca_kabuli_chr04	41285842	[G/T]	1067296706
Ca_Kabuli_NC_021163.1_41289867	Ca_kabuli_chr04	41289867	[A/C]	1067296707
Ca_Kabuli_NC_021163.1_41328938	Ca_kabuli_chr04	41328938	[T/C]	1067296708
Ca_Kabuli_NC_021163.1_41339625	Ca_kabuli_chr04	41339625	[G/C]	1067296709
Ca_Kabuli_NC_021163.1_41344843	Ca_kabuli_chr04	41344843	[T/A]	1067296710
Ca_Kabuli_NC_021163.1_41344947	Ca_kabuli_chr04	41344947	[A/C]	1067296711
Ca_Kabuli_NC_021163.1_41344958	Ca_kabuli_chr04	41344958	[A/G]	1067296712
Ca_Kabuli_NC_021163.1_41427086	Ca_kabuli_chr04	41427086	[C/A]	1067296713
Ca_Kabuli_NC_021163.1_41445209	Ca_kabuli_chr04	41445209	[A/G]	1067296714
Ca_Kabuli_NC_021163.1_41445212	Ca_kabuli_chr04	41445212	[A/G]	1067296715
Ca_Kabuli_NC_021163.1_41455142	Ca_kabuli_chr04	41455142	[A/C]	1067296716
Ca_Kabuli_NC_021163.1_41568682	Ca_kabuli_chr04	41568682	[A/T]	1067296717
Ca_Kabuli_NC_021163.1_41571837	Ca_kabuli_chr04	41571837	[A/T]	1067296718
Ca_Kabuli_NC_021163.1_41571840	Ca_kabuli_chr04	41571840	[A/G]	1067296719
Ca_Kabuli_NC_021163.1_41571849	Ca_kabuli_chr04	41571849	[A/T]	1067296720
Ca_Kabuli_NC_021163.1_41571864	Ca_kabuli_chr04	41571864	[T/C]	1067296721
Ca_Kabuli_NC_021163.1_41571876	Ca_kabuli_chr04	41571876	[T/G]	1067296722

SNP IDs	Chromosomes	Physical positions (bp)	SNPs	NCBI SNPdb accession IDs (NCBI_ss#)
Ca_Kabuli_NC_021163.1_41571904	Ca_kabuli_chr04	41571904	[T/C]	1067296723
Ca_Kabuli_NC_021163.1_41577452	Ca_kabuli_chr04	41577452	[T/G]	1067296724
Ca_Kabuli_NC_021163.1_41580613	Ca_kabuli_chr04	41580613	[T/C]	1067296725
Ca_Kabuli_NC_021163.1_41600485	Ca_kabuli_chr04	41600485	[A/C]	1067296726
Ca_Kabuli_NC_021163.1_41700764	Ca_kabuli_chr04	41700764	[A/C]	1067296727
Ca_Kabuli_NC_021163.1_41702759	Ca_kabuli_chr04	41702759	[T/G]	1067296728
Ca_Kabuli_NC_021163.1_41749243	Ca_kabuli_chr04	41749243	[A/C]	1067296729
Ca_Kabuli_NC_021163.1_41751117	Ca_kabuli_chr04	41751117	[T/C]	1067296730
Ca_Kabuli_NC_021163.1_41772742	Ca_kabuli_chr04	41772742	[A/G]	1067296731
Ca_Kabuli_NC_021163.1_41772809	Ca_kabuli_chr04	41772809	[A/T]	1067296732
Ca_Kabuli_NC_021163.1_41790245	Ca_kabuli_chr04	41790245	[A/C]	1067296733
Ca_Kabuli_NC_021163.1_41790247	Ca_kabuli_chr04	41790247	[C/T]	1067296734
Ca_Kabuli_NC_021163.1_41864886	Ca_kabuli_chr04	41864886	[A/C]	1067296735
Ca_Kabuli_NC_021163.1_41864916	Ca_kabuli_chr04	41864916	[A/C]	1067296736
Ca_Kabuli_NC_021163.1_41865096	Ca_kabuli_chr04	41865096	[T/C]	1067296737
Ca_Kabuli_NC_021163.1_41955962	Ca_kabuli_chr04	41955962	[T/C]	1067296738
Ca_Kabuli_NC_021163.1_41969260	Ca_kabuli_chr04	41969260	[C/G]	1067296739
Ca_Kabuli_NC_021163.1_41969261	Ca_kabuli_chr04	41969261	[T/G]	1067296740
Ca_Kabuli_NC_021163.1_41973674	Ca_kabuli_chr04	41973674	[A/G]	1067296741
Ca_Kabuli_NC_021163.1_41973694	Ca_kabuli_chr04	41973694	[T/C]	1067296742
Ca_Kabuli_NC_021163.1_41973700	Ca_kabuli_chr04	41973700	[T/G]	1067296743
Ca_Kabuli_NC_021163.1_41973708	Ca_kabuli_chr04	41973708	[A/G]	1067296744
Ca_Kabuli_NC_021163.1_41973711	Ca_kabuli_chr04	41973711	[G/T]	1067296745
Ca_Kabuli_NC_021163.1_42001659	Ca_kabuli_chr04	42001659	[G/A]	1067296746
Ca_Kabuli_NC_021163.1_42038742	Ca_kabuli_chr04	42038742	[A/T]	1067296747
Ca_Kabuli_NC_021163.1_42038745	Ca_kabuli_chr04	42038745	[A/T]	1067296748
Ca_Kabuli_NC_021163.1_42089384	Ca_kabuli_chr04	42089384	[T/G]	1067296749
Ca_Kabuli_NC_021163.1_42089391	Ca_kabuli_chr04	42089391	[T/G]	1067296750
Ca_Kabuli_NC_021163.1_42143779	Ca_kabuli_chr04	42143779	[G/A]	1067296751
Ca_Kabuli_NC_021163.1_42143836	Ca_kabuli_chr04	42143836	[G/A]	1067296752
Ca_Kabuli_NC_021163.1_42246426	Ca_kabuli_chr04	42246426	[T/A]	1067296753
Ca_Kabuli_NC_021163.1_42246450	Ca_kabuli_chr04	42246450	[C/T]	1067296754
Ca_Kabuli_NC_021163.1_42246458	Ca_kabuli_chr04	42246458	[C/T]	1067296755
Ca_Kabuli_NC_021163.1_42246467	Ca_kabuli_chr04	42246467	[G/T]	1067296756

SNP IDs	Chromosomes	Physical positions (bp)	SNPs	NCBI SNPdb accession IDs (NCBI_ss#)
Ca_Kabuli_NC_021163.1_42246640	Ca_kabuli_chr04	42246640	[A/G]	1067296757
Ca_Kabuli_NC_021163.1_42246664	Ca_kabuli_chr04	42246664	[A/C]	1067296758
Ca_Kabuli_NC_021163.1_42246701	Ca_kabuli_chr04	42246701	[T/C]	1067296759
Ca_Kabuli_NC_021163.1_42496219	Ca_kabuli_chr04	42496219	[T/G]	1067296760
Ca_Kabuli_NC_021163.1_42499678	Ca_kabuli_chr04	42499678	[T/G]	1067296761
Ca_Kabuli_NC_021163.1_42504525	Ca_kabuli_chr04	42504525	[A/G]	1067296762
Ca_Kabuli_NC_021163.1_42592198	Ca_kabuli_chr04	42592198	[G/A]	1067296763
Ca_Kabuli_NC_021163.1_42592210	Ca_kabuli_chr04	42592210	[C/G]	1067296764
Ca_Kabuli_NC_021163.1_42592212	Ca_kabuli_chr04	42592212	[A/G]	1067296765
Ca_Kabuli_NC_021163.1_42592218	Ca_kabuli_chr04	42592218	[C/T]	1067296766
Ca_Kabuli_NC_021163.1_42592232	Ca_kabuli_chr04	42592232	[G/A]	1067296767
Ca_Kabuli_NC_021163.1_43319788	Ca_kabuli_chr04	43319788	[T/G]	1067296768
Ca_Kabuli_NC_021163.1_43319793	Ca_kabuli_chr04	43319793	[T/C]	1067296769
Ca_Kabuli_NC_021163.1_43379488	Ca_kabuli_chr04	43379488	[A/G]	1067296770
Ca_Kabuli_NC_021163.1_43431772	Ca_kabuli_chr04	43431772	[A/T]	1067296771
Ca_Kabuli_NC_021163.1_43497610	Ca_kabuli_chr04	43497610	[C/T]	1067296772
Ca_Kabuli_NC_021163.1_43497613	Ca_kabuli_chr04	43497613	[T/C]	1067296773
Ca_Kabuli_NC_021163.1_43519154	Ca_kabuli_chr04	43519154	[A/G]	1067296774
Ca_Kabuli_NC_021163.1_43519190	Ca_kabuli_chr04	43519190	[T/C]	1067296775
Ca_Kabuli_NC_021163.1_43519197	Ca_kabuli_chr04	43519197	[T/C]	1067296776
Ca_Kabuli_NC_021163.1_43519217	Ca_kabuli_chr04	43519217	[T/C]	1067296777
Ca_Kabuli_NC_021163.1_43571877	Ca_kabuli_chr04	43571877	[C/T]	1067296778
Ca_Kabuli_NC_021163.1_43662160	Ca_kabuli_chr04	43662160	[T/C]	1067296779
Ca_Kabuli_NC_021163.1_43676592	Ca_kabuli_chr04	43676592	[A/C]	1067296780
Ca_Kabuli_NC_021163.1_43723198	Ca_kabuli_chr04	43723198	[G/T]	1067296781
Ca_Kabuli_NC_021163.1_43723199	Ca_kabuli_chr04	43723199	[C/G]	1067296782
Ca_Kabuli_NC_021163.1_43723203	Ca_kabuli_chr04	43723203	[A/C]	1067296783
Ca_Kabuli_NC_021163.1_43723205	Ca_kabuli_chr04	43723205	[T/A]	1067296784
Ca_Kabuli_NC_021163.1_43723223	Ca_kabuli_chr04	43723223	[A/G]	1067296785
Ca_Kabuli_NC_021163.1_43726334	Ca_kabuli_chr04	43726334	[T/C]	1067296786
Ca_Kabuli_NC_021163.1_43726342	Ca_kabuli_chr04	43726342	[T/C]	1067296787
Ca_Kabuli_NC_021163.1_43726378	Ca_kabuli_chr04	43726378	[T/C]	1067296788
Ca_Kabuli_NC_021163.1_43726405	Ca_kabuli_chr04	43726405	[A/G]	1067296789
Ca_Kabuli_NC_021163.1_43729057	Ca_kabuli_chr04	43729057	[A/G]	1067296790

SNP IDs	Chromosomes	Physical positions (bp)	SNPs	NCBI SNPdb accession IDs (NCBI_ss#)
Ca_Kabuli_NC_021163.1_43729060	Ca_kabuli_chr04	43729060	[A/T]	1067296791
Ca_Kabuli_NC_021163.1_43730681	Ca_kabuli_chr04	43730681	[A/G]	1067296792
Ca_Kabuli_NC_021163.1_43840662	Ca_kabuli_chr04	43840662	[T/G]	1067296793
Ca_Kabuli_NC_021163.1_43840663	Ca_kabuli_chr04	43840663	[A/T]	1067296794
Ca_Kabuli_NC_021163.1_43840673	Ca_kabuli_chr04	43840673	[T/G]	1067296795
Ca_Kabuli_NC_021163.1_43840689	Ca_kabuli_chr04	43840689	[T/C]	1067296796
Ca_Kabuli_NC_021163.1_43859940	Ca_kabuli_chr04	43859940	[A/G]	1067296797
Ca_Kabuli_NC_021163.1_43889033	Ca_kabuli_chr04	43889033	[A/T]	1067296798
Ca_Kabuli_NC_021163.1_43922411	Ca_kabuli_chr04	43922411	[A/C]	1067296799
Ca_Kabuli_NC_021163.1_43922418	Ca_kabuli_chr04	43922418	[A/C]	1067296800
Ca_Kabuli_NC_021163.1_43922440	Ca_kabuli_chr04	43922440	[A/C]	1067296801
Ca_Kabuli_NC_021163.1_43942501	Ca_kabuli_chr04	43942501	[T/C]	1067296802
Ca_Kabuli_NC_021163.1_43945389	Ca_kabuli_chr04	43945389	[T/C]	1067296803
Ca_Kabuli_NC_021163.1_43945395	Ca_kabuli_chr04	43945395	[A/C]	1067296804
Ca_Kabuli_NC_021163.1_43979186	Ca_kabuli_chr04	43979186	[T/C]	1067296805
Ca_Kabuli_NC_021163.1_43979187	Ca_kabuli_chr04	43979187	[G/T]	1067296806
Ca_Kabuli_NC_021163.1_44059060	Ca_kabuli_chr04	44059060	[C/T]	1067296807
Ca_Kabuli_NC_021163.1_44067747	Ca_kabuli_chr04	44067747	[T/A]	1067296808
Ca_Kabuli_NC_021163.1_44067748	Ca_kabuli_chr04	44067748	[A/C]	1067296809
Ca_Kabuli_NC_021163.1_44090715	Ca_kabuli_chr04	44090715	[A/T]	1067296810
Ca_Kabuli_NC_021163.1_44239776	Ca_kabuli_chr04	44239776	[A/T]	1067296811
Ca_Kabuli_NC_021163.1_44239872	Ca_kabuli_chr04	44239872	[T/C]	1067296812
Ca_Kabuli_NC_021163.1_44239889	Ca_kabuli_chr04	44239889	[A/G]	1067296813
Ca_Kabuli_NC_021163.1_44250230	Ca_kabuli_chr04	44250230	[A/G]	1067296814
Ca_Kabuli_NC_021163.1_44260893	Ca_kabuli_chr04	44260893	[A/C]	1067296815
Ca_Kabuli_NC_021163.1_44261160	Ca_kabuli_chr04	44261160	[T/A]	1067296816
Ca_Kabuli_NC_021163.1_44261172	Ca_kabuli_chr04	44261172	[A/C]	1067296817
Ca_Kabuli_NC_021163.1_44261181	Ca_kabuli_chr04	44261181	[A/C]	1067296818
Ca_Kabuli_NC_021163.1_44261184	Ca_kabuli_chr04	44261184	[A/C]	1067296819
Ca_Kabuli_NC_021163.1_44261928	Ca_kabuli_chr04	44261928	[A/C]	1067296820
Ca_Kabuli_NC_021163.1_44261934	Ca_kabuli_chr04	44261934	[A/C]	1067296821
Ca_Kabuli_NC_021163.1_44261940	Ca_kabuli_chr04	44261940	[A/C]	1067296822
Ca_Kabuli_NC_021163.1_44261967	Ca_kabuli_chr04	44261967	[A/C]	1067296823
Ca_Kabuli_NC_021163.1_44261970	Ca_kabuli_chr04	44261970	[T/C]	1067296824

SNP IDs	Chromosomes	Physical positions (bp)	SNPs	NCBI SNPdb accession IDs (NCBI_ss#)
Ca_Kabuli_NC_021163.1_44353354	Ca_kabuli_chr04	44353354	[C/T]	1067296825
Ca_Kabuli_NC_021163.1_44354390	Ca_kabuli_chr04	44354390	[T/C]	1067296826
Ca_Kabuli_NC_021163.1_44354436	Ca_kabuli_chr04	44354436	[A/T]	1067296827
Ca_Kabuli_NC_021163.1_44363092	Ca_kabuli_chr04	44363092	[A/T]	1067296828
Ca_Kabuli_NC_021163.1_44366719	Ca_kabuli_chr04	44366719	[T/A]	1067296829
Ca_Kabuli_NC_021163.1_44367755	Ca_kabuli_chr04	44367755	[A/C]	1067296830
Ca_Kabuli_NC_021163.1_44367782	Ca_kabuli_chr04	44367782	[C/A]	1067296831
Ca_Kabuli_NC_021163.1_44372986	Ca_kabuli_chr04	44372986	[C/T]	1067296832
Ca_Kabuli_NC_021163.1_44372998	Ca_kabuli_chr04	44372998	[C/T]	1067296833
Ca_Kabuli_NC_021163.1_44373020	Ca_kabuli_chr04	44373020	[G/C]	1067296834
Ca_Kabuli_NC_021163.1_44373412	Ca_kabuli_chr04	44373412	[C/T]	1067296835
Ca_Kabuli_NC_021163.1_44385456	Ca_kabuli_chr04	44385456	[A/G]	1067296836
Ca_Kabuli_NC_021163.1_44385457	Ca_kabuli_chr04	44385457	[C/T]	1067296837
Ca_Kabuli_NC_021163.1_44388391	Ca_kabuli_chr04	44388391	[G/C]	1067296838
Ca_Kabuli_NC_021163.1_44411260	Ca_kabuli_chr04	44411260	[A/T]	1067296839
Ca_Kabuli_NC_021163.1_44411345	Ca_kabuli_chr04	44411345	[C/T]	1067296840
Ca_Kabuli_NC_021163.1_44411391	Ca_kabuli_chr04	44411391	[G/C]	1067296841
Ca_Kabuli_NC_021163.1_44431319	Ca_kabuli_chr04	44431319	[A/C]	1067296842
Ca_Kabuli_NC_021163.1_44431436	Ca_kabuli_chr04	44431436	[T/C]	1067296843
Ca_Kabuli_NC_021163.1_44431501	Ca_kabuli_chr04	44431501	[T/C]	1067296844
Ca_Kabuli_NC_021163.1_44433095	Ca_kabuli_chr04	44433095	[A/G]	1067296845
Ca_Kabuli_NC_021163.1_44439492	Ca_kabuli_chr04	44439492	[G/T]	1067296846
Ca_Kabuli_NC_021163.1_44439570	Ca_kabuli_chr04	44439570	[C/T]	1067296847
Ca_Kabuli_NC_021163.1_44446575	Ca_kabuli_chr04	44446575	[G/A]	1067296848
Ca_Kabuli_NC_021163.1_44451062	Ca_kabuli_chr04	44451062	[C/T]	1067296849
Ca_Kabuli_NC_021163.1_44451765	Ca_kabuli_chr04	44451765	[G/A]	1067296850
Ca_Kabuli_NC_021163.1_44453175	Ca_kabuli_chr04	44453175	[C/A]	1067296851
Ca_Kabuli_NC_021163.1_44453192	Ca_kabuli_chr04	44453192	[A/G]	1067296852
Ca_Kabuli_NC_021163.1_44453216	Ca_kabuli_chr04	44453216	[A/G]	1067296853
Ca_Kabuli_NC_021163.1_44458789	Ca_kabuli_chr04	44458789	[T/C]	1067296854
Ca_Kabuli_NC_021163.1_44472237	Ca_kabuli_chr04	44472237	[T/A]	1067296855
Ca_Kabuli_NC_021163.1_44472269	Ca_kabuli_chr04	44472269	[T/G]	1067296856
Ca_Kabuli_NC_021163.1_44534136	Ca_kabuli_chr04	44534136	[C/A]	1067296857
Ca_Kabuli_NC_021163.1_44534137	Ca_kabuli_chr04	44534137	[T/G]	1067296858

SNP IDs	Chromosomes	Physical positions (bp)	SNPs	NCBI SNPdb accession IDs (NCBI_ss#)
Ca_Kabuli_NC_021163.1_44534146	Ca_kabuli_chr04	44534146	[T/A]	1067296859
Ca_Kabuli_NC_021163.1_44536606	Ca_kabuli_chr04	44536606	[A/C]	1067296860
Ca_Kabuli_NC_021163.1_44558549	Ca_kabuli_chr04	44558549	[C/T]	1067296861
Ca_Kabuli_NC_021163.1_44590652	Ca_kabuli_chr04	44590652	[C/T]	1067296862
Ca_Kabuli_NC_021163.1_44590678	Ca_kabuli_chr04	44590678	[G/A]	1067296863
Ca_Kabuli_NC_021163.1_44590697	Ca_kabuli_chr04	44590697	[A/C]	1067296864
Ca_Kabuli_NC_021163.1_44591405	Ca_kabuli_chr04	44591405	[G/T]	1067296865
Ca_Kabuli_NC_021163.1_44634703	Ca_kabuli_chr04	44634703	[G/A]	1067296866
Ca_Kabuli_NC_021163.1_44655132	Ca_kabuli_chr04	44655132	[T/G]	1067296867
Ca_Kabuli_NC_021163.1_44655248	Ca_kabuli_chr04	44655248	[C/T]	1067296868
Ca_Kabuli_NC_021163.1_44731563	Ca_kabuli_chr04	44731563	[T/C]	1067296869
Ca_Kabuli_NC_021163.1_44754355	Ca_kabuli_chr04	44754355	[C/T]	1067296870
Ca_Kabuli_NC_021163.1_44754426	Ca_kabuli_chr04	44754426	[A/C]	1067296871
Ca_Kabuli_NC_021163.1_44756435	Ca_kabuli_chr04	44756435	[T/A]	1067296872
Ca_Kabuli_NC_021163.1_44802374	Ca_kabuli_chr04	44802374	[A/G]	1067296873
Ca_Kabuli_NC_021163.1_44803694	Ca_kabuli_chr04	44803694	[T/A]	1067296874
Ca_Kabuli_NC_021163.1_44811433	Ca_kabuli_chr04	44811433	[T/C]	1067296875
Ca_Kabuli_NC_021163.1_44811530	Ca_kabuli_chr04	44811530	[T/C]	1067296876
Ca_Kabuli_NC_021163.1_44811534	Ca_kabuli_chr04	44811534	[A/C]	1067296877
Ca_Kabuli_NC_021163.1_44814981	Ca_kabuli_chr04	44814981	[T/C]	1067296878
Ca_Kabuli_NC_021163.1_44814982	Ca_kabuli_chr04	44814982	[A/G]	1067296879
Ca_Kabuli_NC_021163.1_44824103	Ca_kabuli_chr04	44824103	[T/G]	1067296880
Ca_Kabuli_NC_021163.1_44824105	Ca_kabuli_chr04	44824105	[G/T]	1067296881
Ca_Kabuli_NC_021163.1_44824342	Ca_kabuli_chr04	44824342	[A/C]	1067296882
Ca_Kabuli_NC_021163.1_44824360	Ca_kabuli_chr04	44824360	[A/G]	1067296883
Ca_Kabuli_NC_021163.1_44832121	Ca_kabuli_chr04	44832121	[G/C]	1067296884
Ca_Kabuli_NC_021163.1_44832144	Ca_kabuli_chr04	44832144	[T/C]	1067296885
Ca_Kabuli_NC_021163.1_44849384	Ca_kabuli_chr04	44849384	[G/A]	1067296886
Ca_Kabuli_NC_021163.1_44871398	Ca_kabuli_chr04	44871398	[G/A]	1067296887
Ca_Kabuli_NC_021163.1_44871469	Ca_kabuli_chr04	44871469	[A/G]	1067296888
Ca_Kabuli_NC_021163.1_44871550	Ca_kabuli_chr04	44871550	[A/C]	1067296889
Ca_Kabuli_NC_021163.1_44906255	Ca_kabuli_chr04	44906255	[A/G]	1067296890
Ca_Kabuli_NC_021163.1_44952217	Ca_kabuli_chr04	44952217	[T/C]	1067296891
Ca_Kabuli_NC_021163.1_44952223	Ca_kabuli_chr04	44952223	[C/G]	1067296892

SNP IDs	Chromosomes	Physical positions (bp)	SNPs	NCBI SNPdb accession IDs (NCBI_ss#)
Ca_Kabuli_NC_021163.1_44952225	Ca_kabuli_chr04	44952225	[A/G]	1067296893
Ca_Kabuli_NC_021163.1_45019764	Ca_kabuli_chr04	45019764	[C/T]	1067296894
Ca_Kabuli_NC_021163.1_45019778	Ca_kabuli_chr04	45019778	[C/T]	1067296895
Ca_Kabuli_NC_021163.1_45019805	Ca_kabuli_chr04	45019805	[C/A]	1067296896
Ca_Kabuli_NC_021163.1_45019807	Ca_kabuli_chr04	45019807	[C/T]	1067296897
Ca_Kabuli_NC_021163.1_45019822	Ca_kabuli_chr04	45019822	[G/A]	1067296898
Ca_Kabuli_NC_021163.1_45022550	Ca_kabuli_chr04	45022550	[A/T]	1067296899
Ca_Kabuli_NC_021163.1_45022634	Ca_kabuli_chr04	45022634	[G/C]	1067296900
Ca_Kabuli_NC_021163.1_45033480	Ca_kabuli_chr04	45033480	[C/T]	1067296901
Ca_Kabuli_NC_021163.1_45035589	Ca_kabuli_chr04	45035589	[G/T]	1067296902
Ca_Kabuli_NC_021163.1_45067525	Ca_kabuli_chr04	45067525	[A/T]	1067296903
Ca_Kabuli_NC_021163.1_45089570	Ca_kabuli_chr04	45089570	[C/T]	1067296904
Ca_Kabuli_NC_021163.1_45104683	Ca_kabuli_chr04	45104683	[A/G]	1067296905
Ca_Kabuli_NC_021163.1_45104696	Ca_kabuli_chr04	45104696	[A/G]	1067296906
Ca_Kabuli_NC_021163.1_45104707	Ca_kabuli_chr04	45104707	[T/C]	1067296907
Ca_Kabuli_NC_021163.1_45104714	Ca_kabuli_chr04	45104714	[T/C]	1067296908
Ca_Kabuli_NC_021163.1_45104726	Ca_kabuli_chr04	45104726	[A/G]	1067296909
Ca_Kabuli_NC_021163.1_45104767	Ca_kabuli_chr04	45104767	[G/A]	1067296910
Ca_Kabuli_NC_021163.1_45104779	Ca_kabuli_chr04	45104779	[C/A]	1067296911
Ca_Kabuli_NC_021163.1_45109319	Ca_kabuli_chr04	45109319	[A/G]	1067296912
Ca_Kabuli_NC_021163.1_45109345	Ca_kabuli_chr04	45109345	[T/A]	1067296913
Ca_Kabuli_NC_021163.1_45125568	Ca_kabuli_chr04	45125568	[T/A]	1067296914
Ca_Kabuli_NC_021163.1_45126517	Ca_kabuli_chr04	45126517	[A/G]	1067296915
Ca_Kabuli_NC_021163.1_45135282	Ca_kabuli_chr04	45135282	[A/T]	1067296916
Ca_Kabuli_NC_021163.1_45135296	Ca_kabuli_chr04	45135296	[T/A]	1067296917
Ca_Kabuli_NC_021163.1_45136480	Ca_kabuli_chr04	45136480	[T/C]	1067296918
Ca_Kabuli_NC_021163.1_45152327	Ca_kabuli_chr04	45152327	[G/T]	1067296919
Ca_Kabuli_NC_021163.1_45152342	Ca_kabuli_chr04	45152342	[C/A]	1067296920
Ca_Kabuli_NC_021163.1_45152343	Ca_kabuli_chr04	45152343	[T/G]	1067296921
Ca_Kabuli_NC_021163.1_45152344	Ca_kabuli_chr04	45152344	[C/A]	1067296922
Ca_Kabuli_NC_021163.1_45153110	Ca_kabuli_chr04	45153110	[C/T]	1067296923
Ca_Kabuli_NC_021163.1_45158491	Ca_kabuli_chr04	45158491	[T/C]	1067296924
Ca_Kabuli_NC_021163.1_45158497	Ca_kabuli_chr04	45158497	[T/C]	1067296925
Ca_Kabuli_NC_021163.1_45159504	Ca_kabuli_chr04	45159504	[G/A]	1067296926

SNP IDs	Chromosomes	Physical positions (bp)	SNPs	NCBI SNPdb accession IDs (NCBI_ss#)
Ca_Kabuli_NC_021163.1_45165191	Ca_kabuli_chr04	45165191	[T/C]	1067296927
Ca_Kabuli_NC_021163.1_45165327	Ca_kabuli_chr04	45165327	[T/C]	1067296928
Ca_Kabuli_NC_021163.1_45165807	Ca_kabuli_chr04	45165807	[C/T]	1067296929
Ca_Kabuli_NC_021163.1_45166848	Ca_kabuli_chr04	45166848	[A/T]	1067296930
Ca_Kabuli_NC_021163.1_45200660	Ca_kabuli_chr04	45200660	[T/C]	1067296931
Ca_Kabuli_NC_021163.1_45200661	Ca_kabuli_chr04	45200661	[G/T]	1067296932
Ca_Kabuli_NC_021163.1_45204033	Ca_kabuli_chr04	45204033	[G/A]	1067296933
Ca_Kabuli_NC_021163.1_45204213	Ca_kabuli_chr04	45204213	[A/G]	1067296934
Ca_Kabuli_NC_021163.1_45206130	Ca_kabuli_chr04	45206130	[T/G]	1067296935
Ca_Kabuli_NC_021163.1_45206175	Ca_kabuli_chr04	45206175	[C/A]	1067296936
Ca_Kabuli_NC_021163.1_45206176	Ca_kabuli_chr04	45206176	[T/G]	1067296937
Ca_Kabuli_NC_021163.1_45206177	Ca_kabuli_chr04	45206177	[T/A]	1067296938
Ca_Kabuli_NC_021163.1_45220422	Ca_kabuli_chr04	45220422	[C/T]	1067296939
Ca_Kabuli_NC_021163.1_45220432	Ca_kabuli_chr04	45220432	[T/G]	1067296940
Ca_Kabuli_NC_021163.1_45220438	Ca_kabuli_chr04	45220438	[T/G]	1067296941
Ca_Kabuli_NC_021163.1_45220448	Ca_kabuli_chr04	45220448	[G/A]	1067296942
Ca_Kabuli_NC_021163.1_45220498	Ca_kabuli_chr04	45220498	[A/C]	1067296943
Ca_Kabuli_NC_021163.1_45220580	Ca_kabuli_chr04	45220580	[A/C]	1067296944
Ca_Kabuli_NC_021163.1_45220599	Ca_kabuli_chr04	45220599	[T/G]	1067296945
Ca_Kabuli_NC_021163.1_45220607	Ca_kabuli_chr04	45220607	[A/C]	1067296946
Ca_Kabuli_NC_021163.1_45220691	Ca_kabuli_chr04	45220691	[A/G]	1067296947
Ca_Kabuli_NC_021163.1_45220696	Ca_kabuli_chr04	45220696	[A/C]	1067296948
Ca_Kabuli_NC_021163.1_45220879	Ca_kabuli_chr04	45220879	[T/C]	1067296949
Ca_Kabuli_NC_021163.1_45220916	Ca_kabuli_chr04	45220916	[T/C]	1067296950
Ca_Kabuli_NC_021163.1_45225059	Ca_kabuli_chr04	45225059	[G/A]	1067296951
Ca_Kabuli_NC_021163.1_45225077	Ca_kabuli_chr04	45225077	[A/T]	1067296952
Ca_Kabuli_NC_021163.1_45225093	Ca_kabuli_chr04	45225093	[G/A]	1067296953
Ca_Kabuli_NC_021163.1_45225165	Ca_kabuli_chr04	45225165	[T/C]	1067296954
Ca_Kabuli_NC_021163.1_45231839	Ca_kabuli_chr04	45231839	[T/C]	1067296955
Ca_Kabuli_NC_021163.1_45256989	Ca_kabuli_chr04	45256989	[A/G]	1067296956
Ca_Kabuli_NC_021163.1_45335231	Ca_kabuli_chr04	45335231	[G/A]	1067296957
Ca_Kabuli_NC_021163.1_45335241	Ca_kabuli_chr04	45335241	[T/G]	1067296958
Ca_Kabuli_NC_021163.1_45347134	Ca_kabuli_chr04	45347134	[G/C]	1067296959
Ca_Kabuli_NC_021163.1_45347135	Ca_kabuli_chr04	45347135	[G/T]	1067296960

SNP IDs	Chromosomes	Physical positions (bp)	SNPs	NCBI SNPdb accession IDs (NCBI_ss#)
Ca_Kabuli_NC_021163.1_45535905	Ca_kabuli_chr04	45535905	[T/G]	1067296961
Ca_Kabuli_NC_021163.1_45612147	Ca_kabuli_chr04	45612147	[A/G]	1067296962
Ca_Kabuli_NC_021163.1_45751621	Ca_kabuli_chr04	45751621	[T/C]	1067296963
Ca_Kabuli_NC_021163.1_45751646	Ca_kabuli_chr04	45751646	[T/C]	1067296964
Ca_Kabuli_NC_021163.1_45752844	Ca_kabuli_chr04	45752844	[G/A]	1067296965
Ca_Kabuli_NC_021163.1_45752850	Ca_kabuli_chr04	45752850	[T/G]	1067296966
Ca_Kabuli_NC_021163.1_45752869	Ca_kabuli_chr04	45752869	[G/T]	1067296967
Ca_Kabuli_NC_021163.1_45752874	Ca_kabuli_chr04	45752874	[A/T]	1067296968
Ca_Kabuli_NC_021163.1_45918958	Ca_kabuli_chr04	45918958	[A/C]	1067296969
Ca_Kabuli_NC_021163.1_45935599	Ca_kabuli_chr04	45935599	[A/C]	1067296970
Ca_Kabuli_NC_021163.1_45942471	Ca_kabuli_chr04	45942471	[C/A]	1067296971
Ca_Kabuli_NC_021163.1_45942473	Ca_kabuli_chr04	45942473	[T/C]	1067296972
Ca_Kabuli_NC_021163.1_46134225	Ca_kabuli_chr04	46134225	[T/C]	1067296973
Ca_Kabuli_NC_021163.1_46134238	Ca_kabuli_chr04	46134238	[A/G]	1067296974
Ca_Kabuli_NC_021163.1_46134253	Ca_kabuli_chr04	46134253	[T/C]	1067296975
Ca_Kabuli_NC_021163.1_46134348	Ca_kabuli_chr04	46134348	[T/C]	1067296976
Ca_Kabuli_NC_021163.1_46140738	Ca_kabuli_chr04	46140738	[A/G]	1067296977
Ca_Kabuli_NC_021163.1_46150755	Ca_kabuli_chr04	46150755	[A/C]	1067296978
Ca_Kabuli_NC_021163.1_46263193	Ca_kabuli_chr04	46263193	[T/G]	1067296979
Ca_Kabuli_NC_021163.1_46263200	Ca_kabuli_chr04	46263200	[A/T]	1067296980
Ca_Kabuli_NC_021163.1_46371703	Ca_kabuli_chr04	46371703	[A/G]	1067296981
Ca_Kabuli_NC_021163.1_46371764	Ca_kabuli_chr04	46371764	[T/C]	1067296982
Ca_Kabuli_NC_021163.1_46392528	Ca_kabuli_chr04	46392528	[T/C]	1067296983
Ca_Kabuli_NC_021163.1_46392555	Ca_kabuli_chr04	46392555	[T/G]	1067296984
Ca_Kabuli_NC_021163.1_46392588	Ca_kabuli_chr04	46392588	[G/A]	1067296985
Ca_Kabuli_NC_021163.1_46402114	Ca_kabuli_chr04	46402114	[A/C]	1067296986
Ca_Kabuli_NC_021163.1_46430297	Ca_kabuli_chr04	46430297	[A/G]	1067296987
Ca_Kabuli_NC_021163.1_46430331	Ca_kabuli_chr04	46430331	[T/G]	1067296988
Ca_Kabuli_NC_021163.1_46430344	Ca_kabuli_chr04	46430344	[T/G]	1067296989
Ca_Kabuli_NC_021163.1_46434287	Ca_kabuli_chr04	46434287	[A/C]	1067296990
Ca_Kabuli_NC_021163.1_46463324	Ca_kabuli_chr04	46463324	[T/A]	1067296991
Ca_Kabuli_NC_021163.1_46521568	Ca_kabuli_chr04	46521568	[T/G]	1067296992
Ca_Kabuli_NC_021163.1_46521576	Ca_kabuli_chr04	46521576	[A/C]	1067296993
Ca_Kabuli_NC_021163.1_46521581	Ca_kabuli_chr04	46521581	[A/C]	1067296994

SNP IDs	Chromosomes	Physical positions (bp)	SNPs	NCBI SNPdb accession IDs (NCBI_ss#)
Ca_Kabuli_NC_021163.1_46521590	Ca_kabuli_chr04	46521590	[A/C]	1067296995
Ca_Kabuli_NC_021163.1_46534847	Ca_kabuli_chr04	46534847	[T/C]	1067296996
Ca_Kabuli_NC_021163.1_46534848	Ca_kabuli_chr04	46534848	[G/T]	1067296997
Ca_Kabuli_NC_021163.1_46605136	Ca_kabuli_chr04	46605136	[A/T]	1067296998
Ca_Kabuli_NC_021163.1_46605150	Ca_kabuli_chr04	46605150	[A/G]	1067296999
Ca_Kabuli_NC_021163.1_46605158	Ca_kabuli_chr04	46605158	[T/C]	1067297000
Ca_Kabuli_NC_021163.1_46612214	Ca_kabuli_chr04	46612214	[C/T]	1067297001
Ca_Kabuli_NC_021163.1_46612228	Ca_kabuli_chr04	46612228	[A/C]	1067297002
Ca_Kabuli_NC_021163.1_46612240	Ca_kabuli_chr04	46612240	[A/C]	1067297003
Ca_Kabuli_NC_021163.1_46613012	Ca_kabuli_chr04	46613012	[A/C]	1067297004
Ca_Kabuli_NC_021163.1_46613068	Ca_kabuli_chr04	46613068	[G/T]	1067297005
Ca_Kabuli_NC_021163.1_46613075	Ca_kabuli_chr04	46613075	[A/T]	1067297006
Ca_Kabuli_NC_021163.1_46613089	Ca_kabuli_chr04	46613089	[A/G]	1067297007
Ca_Kabuli_NC_021163.1_46613097	Ca_kabuli_chr04	46613097	[T/C]	1067297008
Ca_Kabuli_NC_021163.1_46613098	Ca_kabuli_chr04	46613098	[T/C]	1067297009
Ca_Kabuli_NC_021163.1_46651499	Ca_kabuli_chr04	46651499	[A/C]	1067297010
Ca_Kabuli_NC_021163.1_46713873	Ca_kabuli_chr04	46713873	[A/G]	1067297011
Ca_Kabuli_NC_021163.1_46713886	Ca_kabuli_chr04	46713886	[T/G]	1067297012
Ca_Kabuli_NC_021163.1_46713939	Ca_kabuli_chr04	46713939	[A/T]	1067297013
Ca_Kabuli_NC_021163.1_46713940	Ca_kabuli_chr04	46713940	[A/C]	1067297014
Ca_Kabuli_NC_021163.1_46713941	Ca_kabuli_chr04	46713941	[G/T]	1067297015
Ca_Kabuli_NC_021163.1_46713948	Ca_kabuli_chr04	46713948	[T/G]	1067297016
Ca_Kabuli_NC_021163.1_46714984	Ca_kabuli_chr04	46714984	[T/G]	1067297017
Ca_Kabuli_NC_021163.1_46715661	Ca_kabuli_chr04	46715661	[T/G]	1067297018
Ca_Kabuli_NC_021163.1_46717150	Ca_kabuli_chr04	46717150	[A/G]	1067297019
Ca_Kabuli_NC_021163.1_46717170	Ca_kabuli_chr04	46717170	[A/G]	1067297020
Ca_Kabuli_NC_021163.1_46717200	Ca_kabuli_chr04	46717200	[C/T]	1067297021
Ca_Kabuli_NC_021163.1_46733965	Ca_kabuli_chr04	46733965	[A/G]	1067297022
Ca_Kabuli_NC_021163.1_47223533	Ca_kabuli_chr04	47223533	[T/A]	1067297023
Ca_Kabuli_NC_021163.1_47223627	Ca_kabuli_chr04	47223627	[C/G]	1067297024
Ca_Kabuli_NC_021163.1_47223630	Ca_kabuli_chr04	47223630	[A/G]	1067297025
Ca_Kabuli_NC_021163.1_47223646	Ca_kabuli_chr04	47223646	[T/C]	1067297026
Ca_Kabuli_NC_021163.1_47223674	Ca_kabuli_chr04	47223674	[A/C]	1067297027
Ca_Kabuli_NC_021163.1_47296161	Ca_kabuli_chr04	47296161	[G/A]	1067297028

SNP IDs	Chromosomes	Physical positions (bp)	SNPs	NCBI SNPdb accession IDs (NCBI_ss#)
Ca_Kabuli_NC_021163.1_47296163	Ca_kabuli_chr04	47296163	[G/A]	1067297029
Ca_Kabuli_NC_021163.1_47296164	Ca_kabuli_chr04	47296164	[A/G]	1067297030
Ca_Kabuli_NC_021163.1_47345557	Ca_kabuli_chr04	47345557	[T/A]	1067297031
Ca_Kabuli_NC_021163.1_47427865	Ca_kabuli_chr04	47427865	[A/C]	1067297032
Ca_Kabuli_NC_021163.1_47446366	Ca_kabuli_chr04	47446366	[A/T]	1067297033
Ca_Kabuli_NC_021163.1_47505073	Ca_kabuli_chr04	47505073	[A/C]	1067297034
Ca_Kabuli_NC_021163.1_47592455	Ca_kabuli_chr04	47592455	[T/C]	1067297035
Ca_Kabuli_NC_021163.1_47612353	Ca_kabuli_chr04	47612353	[T/C]	1067297036
Ca_Kabuli_NC_021163.1_47612384	Ca_kabuli_chr04	47612384	[T/G]	1067297037
Ca_Kabuli_NC_021163.1_47612455	Ca_kabuli_chr04	47612455	[A/C]	1067297038
Ca_Kabuli_NC_021163.1_47622133	Ca_kabuli_chr04	47622133	[A/G]	1067297039
Ca_Kabuli_NC_021163.1_47622154	Ca_kabuli_chr04	47622154	[T/G]	1067297040
Ca_Kabuli_NC_021163.1_47675390	Ca_kabuli_chr04	47675390	[A/C]	1067297041
Ca_Kabuli_NC_021163.1_47675686	Ca_kabuli_chr04	47675686	[A/C]	1067297042
Ca_Kabuli_NC_021163.1_47675839	Ca_kabuli_chr04	47675839	[T/C]	1067297043
Ca_Kabuli_NC_021163.1_47675845	Ca_kabuli_chr04	47675845	[A/G]	1067297044
Ca_Kabuli_NC_021163.1_47675867	Ca_kabuli_chr04	47675867	[A/G]	1067297045
Ca_Kabuli_NC_021163.1_47675880	Ca_kabuli_chr04	47675880	[C/A]	1067297046
Ca_Kabuli_NC_021163.1_47697192	Ca_kabuli_chr04	47697192	[A/G]	1067297047
Ca_Kabuli_NC_021163.1_47697195	Ca_kabuli_chr04	47697195	[T/C]	1067297048
Ca_Kabuli_NC_021163.1_47697219	Ca_kabuli_chr04	47697219	[T/G]	1067297049
Ca_Kabuli_NC_021163.1_47798598	Ca_kabuli_chr04	47798598	[A/C]	1067297050
Ca_Kabuli_NC_021163.1_47798604	Ca_kabuli_chr04	47798604	[A/C]	1067297051
Ca_Kabuli_NC_021163.1_47899055	Ca_kabuli_chr04	47899055	[T/G]	1067297052
Ca_Kabuli_NC_021163.1_48011333	Ca_kabuli_chr04	48011333	[A/C]	1067297053
Ca_Kabuli_NC_021163.1_48011418	Ca_kabuli_chr04	48011418	[T/C]	1067297054
Ca_Kabuli_NC_021163.1_48011422	Ca_kabuli_chr04	48011422	[T/C]	1067297055
Ca_Kabuli_NC_021163.1_48011440	Ca_kabuli_chr04	48011440	[T/A]	1067297056
Ca_Kabuli_NC_021163.1_48011455	Ca_kabuli_chr04	48011455	[A/G]	1067297057
Ca_Kabuli_NC_021163.1_48097268	Ca_kabuli_chr04	48097268	[G/A]	1067297058
Ca_Kabuli_NC_021163.1_48097271	Ca_kabuli_chr04	48097271	[A/C]	1067297059
Ca_Kabuli_NC_021163.1_48103174	Ca_kabuli_chr04	48103174	[C/G]	1067297060
Ca_Kabuli_NC_021163.1_48103176	Ca_kabuli_chr04	48103176	[G/C]	1067297061
Ca_Kabuli_NC_021163.1_48123659	Ca_kabuli_chr04	48123659	[T/G]	1067297062

SNP IDs	Chromosomes	Physical positions (bp)	SNPs	NCBI SNPdb accession IDs (NCBI_ss#)
Ca_Kabuli_NC_021163.1_48228110	Ca_kabuli_chr04	48228110	[C/A]	1067297063
Ca_Kabuli_NC_021163.1_48238802	Ca_kabuli_chr04	48238802	[T/C]	1067297064
Ca_Kabuli_NC_021163.1_48238836	Ca_kabuli_chr04	48238836	[T/C]	1067297065
Ca_Kabuli_NC_021163.1_48248272	Ca_kabuli_chr04	48248272	[C/T]	1067297066
Ca_Kabuli_NC_021163.1_48248286	Ca_kabuli_chr04	48248286	[G/A]	1067297067
Ca_Kabuli_NC_021163.1_48248319	Ca_kabuli_chr04	48248319	[T/G]	1067297068
Ca_Kabuli_NC_021163.1_48248663	Ca_kabuli_chr04	48248663	[A/G]	1067297069
Ca_Kabuli_NC_021163.1_48263603	Ca_kabuli_chr04	48263603	[A/G]	1067297070
Ca_Kabuli_NC_021163.1_48267662	Ca_kabuli_chr04	48267662	[C/T]	1067297071
Ca_Kabuli_NC_021163.1_48268253	Ca_kabuli_chr04	48268253	[T/C]	1067297072
Ca_Kabuli_NC_021163.1_48268316	Ca_kabuli_chr04	48268316	[A/C]	1067297073
Ca_Kabuli_NC_021163.1_48268319	Ca_kabuli_chr04	48268319	[A/C]	1067297074
Ca_Kabuli_NC_021163.1_48301151	Ca_kabuli_chr04	48301151	[T/C]	1067297075
Ca_Kabuli_NC_021163.1_48305147	Ca_kabuli_chr04	48305147	[C/G]	1067297076
Ca_Kabuli_NC_021163.1_48322502	Ca_kabuli_chr04	48322502	[T/C]	1067297077
Ca_Kabuli_NC_021163.1_48345604	Ca_kabuli_chr04	48345604	[G/A]	1067297078
Ca_Kabuli_NC_021163.1_48345608	Ca_kabuli_chr04	48345608	[T/G]	1067297079
Ca_Kabuli_NC_021163.1_48355358	Ca_kabuli_chr04	48355358	[T/A]	1067297080
Ca_Kabuli_NC_021163.1_48355468	Ca_kabuli_chr04	48355468	[T/G]	1067297081
Ca_Kabuli_NC_021163.1_48358019	Ca_kabuli_chr04	48358019	[T/G]	1067297082
Ca_Kabuli_NC_021163.1_48360127	Ca_kabuli_chr04	48360127	[C/G]	1067297083
Ca_Kabuli_NC_021163.1_48361612	Ca_kabuli_chr04	48361612	[G/T]	1067297084
Ca_Kabuli_NC_021163.1_48363106	Ca_kabuli_chr04	48363106	[T/C]	1067297085
Ca_Kabuli_NC_021163.1_48366219	Ca_kabuli_chr04	48366219	[G/A]	1067297086
Ca_Kabuli_NC_021163.1_48382603	Ca_kabuli_chr04	48382603	[C/T]	1067297087
Ca_Kabuli_NC_021163.1_48382666	Ca_kabuli_chr04	48382666	[C/G]	1067297088
Ca_Kabuli_NC_021163.1_48384226	Ca_kabuli_chr04	48384226	[T/C]	1067297089
Ca_Kabuli_NC_021163.1_48384244	Ca_kabuli_chr04	48384244	[A/G]	1067297090
Ca_Kabuli_NC_021163.1_48384246	Ca_kabuli_chr04	48384246	[T/A]	1067297091
Ca_Kabuli_NC_021163.1_48384247	Ca_kabuli_chr04	48384247	[A/C]	1067297092
Ca_Kabuli_NC_021163.1_48454970	Ca_kabuli_chr04	48454970	[A/G]	1067297093
Ca_Kabuli_NC_021163.1_48454986	Ca_kabuli_chr04	48454986	[T/A]	1067297094
Ca_Kabuli_NC_021163.1_48454989	Ca_kabuli_chr04	48454989	[A/G]	1067297095
Ca_Kabuli_NC_021163.1_48455002	Ca_kabuli_chr04	48455002	[A/G]	1067297096

SNP IDs	Chromosomes	Physical positions (bp)	SNPs	NCBI SNPdb accession IDs (NCBI_ss#)
Ca_Kabuli_NC_021163.1_48455054	Ca_kabuli_chr04	48455054	[A/T]	1067297097
Ca_Kabuli_NC_021163.1_48455326	Ca_kabuli_chr04	48455326	[T/C]	1067297098
Ca_Kabuli_NC_021163.1_48455626	Ca_kabuli_chr04	48455626	[T/G]	1067297099
Ca_Kabuli_NC_021163.1_48455657	Ca_kabuli_chr04	48455657	[T/G]	1067297100
Ca_Kabuli_NC_021163.1_48455692	Ca_kabuli_chr04	48455692	[A/T]	1067297101
Ca_Kabuli_NC_021163.1_48457876	Ca_kabuli_chr04	48457876	[C/T]	1067297102
Ca_Kabuli_NC_021163.1_48464448	Ca_kabuli_chr04	48464448	[T/G]	1067297103
Ca_Kabuli_NC_021163.1_48465107	Ca_kabuli_chr04	48465107	[A/G]	1067297104
Ca_Kabuli_NC_021163.1_48470477	Ca_kabuli_chr04	48470477	[G/A]	1067297105
Ca_Kabuli_NC_021163.1_48475447	Ca_kabuli_chr04	48475447	[C/T]	1067297106
Ca_Kabuli_NC_021163.1_48475461	Ca_kabuli_chr04	48475461	[G/A]	1067297107
Ca_Kabuli_NC_021163.1_48477876	Ca_kabuli_chr04	48477876	[T/A]	1067297108
Ca_Kabuli_NC_021163.1_48478018	Ca_kabuli_chr04	48478018	[G/A]	1067297109
Ca_Kabuli_NC_021163.1_48478058	Ca_kabuli_chr04	48478058	[T/C]	1067297110
Ca_Kabuli_NC_021163.1_48478444	Ca_kabuli_chr04	48478444	[A/C]	1067297111
Ca_Kabuli_NC_021163.1_48484455	Ca_kabuli_chr04	48484455	[A/G]	1067297112
Ca_Kabuli_NC_021163.1_48496892	Ca_kabuli_chr04	48496892	[A/G]	1067297113
Ca_Kabuli_NC_021163.1_48496921	Ca_kabuli_chr04	48496921	[A/G]	1067297114
Ca_Kabuli_NC_021163.1_48496922	Ca_kabuli_chr04	48496922	[G/A]	1067297115
Ca_Kabuli_NC_021163.1_48496923	Ca_kabuli_chr04	48496923	[A/G]	1067297116
Ca_Kabuli_NC_021163.1_48497704	Ca_kabuli_chr04	48497704	[A/C]	1067297117
Ca_Kabuli_NC_021163.1_48497710	Ca_kabuli_chr04	48497710	[T/G]	1067297118
Ca_Kabuli_NC_021163.1_48497718	Ca_kabuli_chr04	48497718	[T/A]	1067297119
Ca_Kabuli_NC_021163.1_48497722	Ca_kabuli_chr04	48497722	[T/C]	1067297120
Ca_Kabuli_NC_021163.1_48497839	Ca_kabuli_chr04	48497839	[A/G]	1067297121
Ca_Kabuli_NC_021163.1_48497926	Ca_kabuli_chr04	48497926	[A/T]	1067297122
Ca_Kabuli_NC_021163.1_48498133	Ca_kabuli_chr04	48498133	[C/T]	1067297123
Ca_Kabuli_NC_021163.1_48498166	Ca_kabuli_chr04	48498166	[A/G]	1067297124
Ca_Kabuli_NC_021163.1_48498181	Ca_kabuli_chr04	48498181	[G/C]	1067297125
Ca_Kabuli_NC_021163.1_48498334	Ca_kabuli_chr04	48498334	[G/T]	1067297126
Ca_Kabuli_NC_021163.1_48498368	Ca_kabuli_chr04	48498368	[C/T]	1067297127
Ca_Kabuli_NC_021163.1_48501021	Ca_kabuli_chr04	48501021	[A/T]	1067297128
Ca_Kabuli_NC_021163.1_48501484	Ca_kabuli_chr04	48501484	[G/T]	1067297129
Ca_Kabuli_NC_021163.1_48501662	Ca_kabuli_chr04	48501662	[A/T]	1067297130

SNP IDs	Chromosomes	Physical positions (bp)	SNPs	NCBI SNPdb accession IDs (NCBI_ss#)
Ca_Kabuli_NC_021163.1_48502687	Ca_kabuli_chr04	48502687	[A/G]	1067297131
Ca_Kabuli_NC_021163.1_48502714	Ca_kabuli_chr04	48502714	[C/T]	1067297132
Ca_Kabuli_NC_021163.1_48502718	Ca_kabuli_chr04	48502718	[C/G]	1067297133
Ca_Kabuli_NC_021163.1_48644169	Ca_kabuli_chr04	48644169	[T/G]	1067297134
Ca_Kabuli_NC_021163.1_48644179	Ca_kabuli_chr04	48644179	[T/G]	1067297135
Ca_Kabuli_NC_021163.1_48644216	Ca_kabuli_chr04	48644216	[A/T]	1067297136
Ca_Kabuli_NC_021163.1_48678293	Ca_kabuli_chr04	48678293	[A/G]	1067297137
Ca_Kabuli_NC_021163.1_48678296	Ca_kabuli_chr04	48678296	[T/C]	1067297138
Ca_Kabuli_NC_021163.1_48678302	Ca_kabuli_chr04	48678302	[A/G]	1067297139
Ca_Kabuli_NC_021163.1_48678319	Ca_kabuli_chr04	48678319	[T/C]	1067297140
Ca_Kabuli_NC_021163.1_48678328	Ca_kabuli_chr04	48678328	[A/G]	1067297141
Ca_Kabuli_NC_021163.1_48678692	Ca_kabuli_chr04	48678692	[T/C]	1067297142
Ca_Kabuli_NC_021163.1_48681589	Ca_kabuli_chr04	48681589	[T/A]	1067297143
Ca_Kabuli_NC_021163.1_48702117	Ca_kabuli_chr04	48702117	[T/G]	1067297144
Ca_Kabuli_NC_021163.1_48765850	Ca_kabuli_chr04	48765850	[A/G]	1067297145
Ca_Kabuli_NC_021163.1_48765858	Ca_kabuli_chr04	48765858	[T/A]	1067297146
Ca_Kabuli_NC_021163.1_48932337	Ca_kabuli_chr04	48932337	[A/T]	1067297147
Ca_Kabuli_NC_021163.1_48936860	Ca_kabuli_chr04	48936860	[G/C]	1067297148
Ca_Kabuli_NC_021163.1_48936868	Ca_kabuli_chr04	48936868	[T/G]	1067297149
Ca_Kabuli_NC_021163.1_48936869	Ca_kabuli_chr04	48936869	[A/T]	1067297150
Ca_Kabuli_NC_021163.1_48936870	Ca_kabuli_chr04	48936870	[T/C]	1067297151
Ca_Kabuli_NC_021163.1_48936871	Ca_kabuli_chr04	48936871	[G/T]	1067297152
Ca_Kabuli_NC_021163.1_49012669	Ca_kabuli_chr04	49012669	[T/G]	1067297153
Ca_Kabuli_NC_021163.1_49012793	Ca_kabuli_chr04	49012793	[T/G]	1067297154
Ca_Kabuli_NC_021163.1_49108401	Ca_kabuli_chr04	49108401	[T/A]	1067297155
Ca_Kabuli_NC_021163.1_49108429	Ca_kabuli_chr04	49108429	[A/C]	1067297156
Ca_Kabuli_NC_021164.1_23820	Ca_kabuli_chr05	23820	[T/G]	1067297157
Ca_Kabuli_NC_021164.1_23877	Ca_kabuli_chr05	23877	[A/C]	1067297158
Ca_Kabuli_NC_021164.1_23889	Ca_kabuli_chr05	23889	[A/C]	1067297159
Ca_Kabuli_NC_021164.1_80027	Ca_kabuli_chr05	80027	[C/A]	1067297160
Ca_Kabuli_NC_021164.1_80124	Ca_kabuli_chr05	80124	[A/C]	1067297161
Ca_Kabuli_NC_021164.1_131600	Ca_kabuli_chr05	131600	[A/T]	1067297162
Ca_Kabuli_NC_021164.1_154162	Ca_kabuli_chr05	154162	[T/C]	1067297163
Ca_Kabuli_NC_021164.1_175603	Ca_kabuli_chr05	175603	[A/G]	1067297164

SNP IDs	Chromosomes	Physical positions (bp)	SNPs	NCBI SNPdb accession IDs (NCBI_ss#)
Ca_Kabuli_NC_021164.1_175629	Ca_kabuli_chr05	175629	[T/A]	1067297165
Ca_Kabuli_NC_021164.1_210586	Ca_kabuli_chr05	210586	[A/T]	1067297166
Ca_Kabuli_NC_021164.1_222629	Ca_kabuli_chr05	222629	[T/G]	1067297167
Ca_Kabuli_NC_021164.1_222637	Ca_kabuli_chr05	222637	[C/T]	1067297168
Ca_Kabuli_NC_021164.1_222639	Ca_kabuli_chr05	222639	[C/T]	1067297169
Ca_Kabuli_NC_021164.1_222641	Ca_kabuli_chr05	222641	[C/T]	1067297170
Ca_Kabuli_NC_021164.1_222651	Ca_kabuli_chr05	222651	[A/C]	1067297171
Ca_Kabuli_NC_021164.1_222657	Ca_kabuli_chr05	222657	[G/A]	1067297172
Ca_Kabuli_NC_021164.1_223401	Ca_kabuli_chr05	223401	[T/A]	1067297173
Ca_Kabuli_NC_021164.1_234405	Ca_kabuli_chr05	234405	[A/G]	1067297174
Ca_Kabuli_NC_021164.1_234410	Ca_kabuli_chr05	234410	[A/C]	1067297175
Ca_Kabuli_NC_021164.1_235777	Ca_kabuli_chr05	235777	[T/C]	1067297176
Ca_Kabuli_NC_021164.1_235807	Ca_kabuli_chr05	235807	[T/G]	1067297177
Ca_Kabuli_NC_021164.1_235820	Ca_kabuli_chr05	235820	[T/G]	1067297178
Ca_Kabuli_NC_021164.1_245863	Ca_kabuli_chr05	245863	[A/T]	1067297179
Ca_Kabuli_NC_021164.1_254262	Ca_kabuli_chr05	254262	[T/C]	1067297180
Ca_Kabuli_NC_021164.1_254272	Ca_kabuli_chr05	254272	[T/C]	1067297181
Ca_Kabuli_NC_021164.1_255245	Ca_kabuli_chr05	255245	[T/G]	1067297182
Ca_Kabuli_NC_021164.1_263342	Ca_kabuli_chr05	263342	[A/G]	1067297183
Ca_Kabuli_NC_021164.1_263345	Ca_kabuli_chr05	263345	[T/C]	1067297184
Ca_Kabuli_NC_021164.1_263350	Ca_kabuli_chr05	263350	[T/C]	1067297185
Ca_Kabuli_NC_021164.1_263356	Ca_kabuli_chr05	263356	[A/G]	1067297186
Ca_Kabuli_NC_021164.1_272745	Ca_kabuli_chr05	272745	[A/C]	1067297187
Ca_Kabuli_NC_021164.1_272863	Ca_kabuli_chr05	272863	[T/A]	1067297188
Ca_Kabuli_NC_021164.1_273752	Ca_kabuli_chr05	273752	[T/A]	1067297189
Ca_Kabuli_NC_021164.1_273772	Ca_kabuli_chr05	273772	[A/C]	1067297190
Ca_Kabuli_NC_021164.1_276413	Ca_kabuli_chr05	276413	[G/A]	1067297191
Ca_Kabuli_NC_021164.1_282870	Ca_kabuli_chr05	282870	[A/C]	1067297192
Ca_Kabuli_NC_021164.1_303757	Ca_kabuli_chr05	303757	[T/C]	1067297193
Ca_Kabuli_NC_021164.1_303762	Ca_kabuli_chr05	303762	[T/C]	1067297194
Ca_Kabuli_NC_021164.1_306319	Ca_kabuli_chr05	306319	[A/T]	1067297195
Ca_Kabuli_NC_021164.1_306350	Ca_kabuli_chr05	306350	[T/A]	1067297196
Ca_Kabuli_NC_021164.1_306359	Ca_kabuli_chr05	306359	[T/A]	1067297197
Ca_Kabuli_NC_021164.1_306362	Ca_kabuli_chr05	306362	[T/G]	1067297198

SNP IDs	Chromosomes	Physical positions (bp)	SNPs	NCBI SNPdb accession IDs (NCBI_ss#)
Ca_Kabuli_NC_021164.1_321517	Ca_kabuli_chr05	321517	[A/C]	1067297199
Ca_Kabuli_NC_021164.1_321520	Ca_kabuli_chr05	321520	[A/C]	1067297200
Ca_Kabuli_NC_021164.1_322011	Ca_kabuli_chr05	322011	[A/G]	1067297201
Ca_Kabuli_NC_021164.1_335086	Ca_kabuli_chr05	335086	[T/G]	1067297202
Ca_Kabuli_NC_021164.1_335124	Ca_kabuli_chr05	335124	[A/G]	1067297203
Ca_Kabuli_NC_021164.1_335147	Ca_kabuli_chr05	335147	[A/T]	1067297204
Ca_Kabuli_NC_021164.1_343574	Ca_kabuli_chr05	343574	[A/G]	1067297205
Ca_Kabuli_NC_021164.1_438390	Ca_kabuli_chr05	438390	[T/C]	1067297206
Ca_Kabuli_NC_021164.1_438411	Ca_kabuli_chr05	438411	[G/C]	1067297207
Ca_Kabuli_NC_021164.1_438428	Ca_kabuli_chr05	438428	[T/G]	1067297208
Ca_Kabuli_NC_021164.1_438431	Ca_kabuli_chr05	438431	[T/C]	1067297209
Ca_Kabuli_NC_021164.1_438438	Ca_kabuli_chr05	438438	[G/A]	1067297210
Ca_Kabuli_NC_021164.1_438442	Ca_kabuli_chr05	438442	[T/C]	1067297211
Ca_Kabuli_NC_021164.1_438445	Ca_kabuli_chr05	438445	[A/C]	1067297212
Ca_Kabuli_NC_021164.1_438448	Ca_kabuli_chr05	438448	[A/C]	1067297213
Ca_Kabuli_NC_021164.1_438453	Ca_kabuli_chr05	438453	[T/C]	1067297214
Ca_Kabuli_NC_021164.1_443282	Ca_kabuli_chr05	443282	[T/C]	1067297215
Ca_Kabuli_NC_021164.1_443305	Ca_kabuli_chr05	443305	[A/C]	1067297216
Ca_Kabuli_NC_021164.1_443383	Ca_kabuli_chr05	443383	[T/C]	1067297217
Ca_Kabuli_NC_021164.1_443487	Ca_kabuli_chr05	443487	[A/C]	1067297218
Ca_Kabuli_NC_021164.1_469339	Ca_kabuli_chr05	469339	[T/A]	1067297219
Ca_Kabuli_NC_021164.1_473545	Ca_kabuli_chr05	473545	[A/G]	1067297220
Ca_Kabuli_NC_021164.1_473563	Ca_kabuli_chr05	473563	[T/C]	1067297221
Ca_Kabuli_NC_021164.1_473585	Ca_kabuli_chr05	473585	[A/C]	1067297222
Ca_Kabuli_NC_021164.1_473603	Ca_kabuli_chr05	473603	[T/A]	1067297223
Ca_Kabuli_NC_021164.1_473663	Ca_kabuli_chr05	473663	[A/T]	1067297224
Ca_Kabuli_NC_021164.1_473668	Ca_kabuli_chr05	473668	[A/T]	1067297225
Ca_Kabuli_NC_021164.1_560662	Ca_kabuli_chr05	560662	[G/A]	1067297226
Ca_Kabuli_NC_021164.1_805760	Ca_kabuli_chr05	805760	[T/C]	1067297227
Ca_Kabuli_NC_021164.1_805764	Ca_kabuli_chr05	805764	[C/G]	1067297228
Ca_Kabuli_NC_021164.1_805767	Ca_kabuli_chr05	805767	[T/C]	1067297229
Ca_Kabuli_NC_021164.1_1252328	Ca_kabuli_chr05	1252328	[A/G]	1067297230
Ca_Kabuli_NC_021164.1_1252330	Ca_kabuli_chr05	1252330	[A/G]	1067297231
Ca_Kabuli_NC_021164.1_1280977	Ca_kabuli_chr05	1280977	[T/G]	1067297232

SNP IDs	Chromosomes	Physical positions (bp)	SNPs	NCBI SNPdb accession IDs (NCBI_ss#)
Ca_Kabuli_NC_021164.1_2380032	Ca_kabuli_chr05	2380032	[C/T]	1067297233
Ca_Kabuli_NC_021164.1_2613443	Ca_kabuli_chr05	2613443	[T/C]	1067297234
Ca_Kabuli_NC_021164.1_2700479	Ca_kabuli_chr05	2700479	[T/A]	1067297235
Ca_Kabuli_NC_021164.1_2700484	Ca_kabuli_chr05	2700484	[A/C]	1067297236
Ca_Kabuli_NC_021164.1_2839097	Ca_kabuli_chr05	2839097	[A/C]	1067297237
Ca_Kabuli_NC_021164.1_2855067	Ca_kabuli_chr05	2855067	[T/G]	1067297238
Ca_Kabuli_NC_021164.1_2948530	Ca_kabuli_chr05	2948530	[G/T]	1067297239
Ca_Kabuli_NC_021164.1_2948531	Ca_kabuli_chr05	2948531	[T/C]	1067297240
Ca_Kabuli_NC_021164.1_2948616	Ca_kabuli_chr05	2948616	[G/A]	1067297241
Ca_Kabuli_NC_021164.1_2948618	Ca_kabuli_chr05	2948618	[C/T]	1067297242
Ca_Kabuli_NC_021164.1_3040267	Ca_kabuli_chr05	3040267	[T/G]	1067297243
Ca_Kabuli_NC_021164.1_3040277	Ca_kabuli_chr05	3040277	[T/G]	1067297244
Ca_Kabuli_NC_021164.1_3040328	Ca_kabuli_chr05	3040328	[G/A]	1067297245
Ca_Kabuli_NC_021164.1_3040334	Ca_kabuli_chr05	3040334	[A/T]	1067297246
Ca_Kabuli_NC_021164.1_3043038	Ca_kabuli_chr05	3043038	[A/C]	1067297247
Ca_Kabuli_NC_021164.1_3043039	Ca_kabuli_chr05	3043039	[T/G]	1067297248
Ca_Kabuli_NC_021164.1_3043042	Ca_kabuli_chr05	3043042	[T/C]	1067297249
Ca_Kabuli_NC_021164.1_3043043	Ca_kabuli_chr05	3043043	[G/T]	1067297250
Ca_Kabuli_NC_021164.1_3137441	Ca_kabuli_chr05	3137441	[A/G]	1067297251
Ca_Kabuli_NC_021164.1_3137454	Ca_kabuli_chr05	3137454	[T/C]	1067297252
Ca_Kabuli_NC_021164.1_3137466	Ca_kabuli_chr05	3137466	[T/C]	1067297253
Ca_Kabuli_NC_021164.1_3137480	Ca_kabuli_chr05	3137480	[A/C]	1067297254
Ca_Kabuli_NC_021164.1_3276362	Ca_kabuli_chr05	3276362	[G/A]	1067297255
Ca_Kabuli_NC_021164.1_3365078	Ca_kabuli_chr05	3365078	[T/A]	1067297256
Ca_Kabuli_NC_021164.1_3481146	Ca_kabuli_chr05	3481146	[T/A]	1067297257
Ca_Kabuli_NC_021164.1_3481151	Ca_kabuli_chr05	3481151	[G/A]	1067297258
Ca_Kabuli_NC_021164.1_3643160	Ca_kabuli_chr05	3643160	[C/T]	1067297259
Ca_Kabuli_NC_021164.1_3654489	Ca_kabuli_chr05	3654489	[A/C]	1067297260
Ca_Kabuli_NC_021164.1_3965094	Ca_kabuli_chr05	3965094	[T/C]	1067297261
Ca_Kabuli_NC_021164.1_3965136	Ca_kabuli_chr05	3965136	[T/G]	1067297262
Ca_Kabuli_NC_021164.1_3965148	Ca_kabuli_chr05	3965148	[A/G]	1067297263
Ca_Kabuli_NC_021164.1_3965169	Ca_kabuli_chr05	3965169	[A/C]	1067297264
Ca_Kabuli_NC_021164.1_4200281	Ca_kabuli_chr05	4200281	[T/G]	1067297265
Ca_Kabuli_NC_021164.1_4890586	Ca_kabuli_chr05	4890586	[G/A]	1067297266

SNP IDs	Chromosomes	Physical positions (bp)	SNPs	NCBI SNPdb accession IDs (NCBI_ss#)
Ca_Kabuli_NC_021164.1_4990103	Ca_kabuli_chr05	4990103	[G/T]	1067297267
Ca_Kabuli_NC_021164.1_5053307	Ca_kabuli_chr05	5053307	[A/G]	1067297268
Ca_Kabuli_NC_021164.1_5131288	Ca_kabuli_chr05	5131288	[T/A]	1067297269
Ca_Kabuli_NC_021164.1_5145576	Ca_kabuli_chr05	5145576	[A/C]	1067297270
Ca_Kabuli_NC_021164.1_5145586	Ca_kabuli_chr05	5145586	[A/G]	1067297271
Ca_Kabuli_NC_021164.1_5404185	Ca_kabuli_chr05	5404185	[A/C]	1067297272
Ca_Kabuli_NC_021164.1_5404195	Ca_kabuli_chr05	5404195	[A/G]	1067297273
Ca_Kabuli_NC_021164.1_5404199	Ca_kabuli_chr05	5404199	[T/C]	1067297274
Ca_Kabuli_NC_021164.1_5404239	Ca_kabuli_chr05	5404239	[A/G]	1067297275
Ca_Kabuli_NC_021164.1_6300288	Ca_kabuli_chr05	6300288	[A/G]	1067297276
Ca_Kabuli_NC_021164.1_6300317	Ca_kabuli_chr05	6300317	[A/G]	1067297277
Ca_Kabuli_NC_021164.1_6316813	Ca_kabuli_chr05	6316813	[A/C]	1067297278
Ca_Kabuli_NC_021164.1_6442437	Ca_kabuli_chr05	6442437	[A/C]	1067297279
Ca_Kabuli_NC_021164.1_6740218	Ca_kabuli_chr05	6740218	[G/T]	1067297280
Ca_Kabuli_NC_021164.1_7047168	Ca_kabuli_chr05	7047168	[T/C]	1067297281
Ca_Kabuli_NC_021164.1_7047186	Ca_kabuli_chr05	7047186	[A/G]	1067297282
Ca_Kabuli_NC_021164.1_7047194	Ca_kabuli_chr05	7047194	[T/C]	1067297283
Ca_Kabuli_NC_021164.1_7047246	Ca_kabuli_chr05	7047246	[A/G]	1067297284
Ca_Kabuli_NC_021164.1_7130712	Ca_kabuli_chr05	7130712	[A/G]	1067297285
Ca_Kabuli_NC_021164.1_7274305	Ca_kabuli_chr05	7274305	[T/G]	1067297286
Ca_Kabuli_NC_021164.1_7335132	Ca_kabuli_chr05	7335132	[T/C]	1067297287
Ca_Kabuli_NC_021164.1_7335168	Ca_kabuli_chr05	7335168	[A/G]	1067297288
Ca_Kabuli_NC_021164.1_7467768	Ca_kabuli_chr05	7467768	[A/C]	1067297289
Ca_Kabuli_NC_021164.1_7467769	Ca_kabuli_chr05	7467769	[G/T]	1067297290
Ca_Kabuli_NC_021164.1_7471749	Ca_kabuli_chr05	7471749	[T/C]	1067297291
Ca_Kabuli_NC_021164.1_7471751	Ca_kabuli_chr05	7471751	[T/C]	1067297292
Ca_Kabuli_NC_021164.1_7638130	Ca_kabuli_chr05	7638130	[A/C]	1067297293
Ca_Kabuli_NC_021164.1_7775298	Ca_kabuli_chr05	7775298	[A/T]	1067297294
Ca_Kabuli_NC_021164.1_7775497	Ca_kabuli_chr05	7775497	[G/C]	1067297295
Ca_Kabuli_NC_021164.1_7775516	Ca_kabuli_chr05	7775516	[A/G]	1067297296
Ca_Kabuli_NC_021164.1_7788986	Ca_kabuli_chr05	7788986	[T/G]	1067297297
Ca_Kabuli_NC_021164.1_7848643	Ca_kabuli_chr05	7848643	[A/T]	1067297298
Ca_Kabuli_NC_021164.1_7848672	Ca_kabuli_chr05	7848672	[T/C]	1067297299
Ca_Kabuli_NC_021164.1_8020717	Ca_kabuli_chr05	8020717	[A/G]	1067297300

SNP IDs	Chromosomes	Physical positions (bp)	SNPs	NCBI SNPdb accession IDs (NCBI_ss#)
Ca_Kabuli_NC_021164.1_8020741	Ca_kabuli_chr05	8020741	[T/A]	1067297301
Ca_Kabuli_NC_021164.1_8535592	Ca_kabuli_chr05	8535592	[A/C]	1067297302
Ca_Kabuli_NC_021164.1_8535619	Ca_kabuli_chr05	8535619	[A/C]	1067297303
Ca_Kabuli_NC_021164.1_8944478	Ca_kabuli_chr05	8944478	[T/C]	1067297304
Ca_Kabuli_NC_021164.1_8944488	Ca_kabuli_chr05	8944488	[T/G]	1067297305
Ca_Kabuli_NC_021164.1_8944508	Ca_kabuli_chr05	8944508	[A/G]	1067297306
Ca_Kabuli_NC_021164.1_8946173	Ca_kabuli_chr05	8946173	[T/G]	1067297307
Ca_Kabuli_NC_021164.1_9077441	Ca_kabuli_chr05	9077441	[A/T]	1067297308
Ca_Kabuli_NC_021164.1_9077447	Ca_kabuli_chr05	9077447	[A/G]	1067297309
Ca_Kabuli_NC_021164.1_9077481	Ca_kabuli_chr05	9077481	[G/A]	1067297310
Ca_Kabuli_NC_021164.1_9077499	Ca_kabuli_chr05	9077499	[T/A]	1067297311
Ca_Kabuli_NC_021164.1_9120091	Ca_kabuli_chr05	9120091	[T/G]	1067297312
Ca_Kabuli_NC_021164.1_9121639	Ca_kabuli_chr05	9121639	[T/C]	1067297313
Ca_Kabuli_NC_021164.1_9127099	Ca_kabuli_chr05	9127099	[A/C]	1067297314
Ca_Kabuli_NC_021164.1_9127126	Ca_kabuli_chr05	9127126	[A/T]	1067297315
Ca_Kabuli_NC_021164.1_9444595	Ca_kabuli_chr05	9444595	[T/G]	1067297316
Ca_Kabuli_NC_021164.1_9483764	Ca_kabuli_chr05	9483764	[A/C]	1067297317
Ca_Kabuli_NC_021164.1_9483785	Ca_kabuli_chr05	9483785	[T/A]	1067297318
Ca_Kabuli_NC_021164.1_9519382	Ca_kabuli_chr05	9519382	[C/A]	1067297319
Ca_Kabuli_NC_021164.1_9609752	Ca_kabuli_chr05	9609752	[T/A]	1067297320
Ca_Kabuli_NC_021164.1_9611022	Ca_kabuli_chr05	9611022	[A/C]	1067297321
Ca_Kabuli_NC_021164.1_9639391	Ca_kabuli_chr05	9639391	[A/C]	1067297322
Ca_Kabuli_NC_021164.1_9783650	Ca_kabuli_chr05	9783650	[T/G]	1067297323
Ca_Kabuli_NC_021164.1_9851952	Ca_kabuli_chr05	9851952	[C/A]	1067297324
Ca_Kabuli_NC_021164.1_9868969	Ca_kabuli_chr05	9868969	[C/G]	1067297325
Ca_Kabuli_NC_021164.1_9869001	Ca_kabuli_chr05	9869001	[A/G]	1067297326
Ca_Kabuli_NC_021164.1_10004421	Ca_kabuli_chr05	10004421	[T/G]	1067297327
Ca_Kabuli_NC_021164.1_10004424	Ca_kabuli_chr05	10004424	[T/G]	1067297328
Ca_Kabuli_NC_021164.1_10004427	Ca_kabuli_chr05	10004427	[G/T]	1067297329
Ca_Kabuli_NC_021164.1_10004430	Ca_kabuli_chr05	10004430	[T/G]	1067297330
Ca_Kabuli_NC_021164.1_10012365	Ca_kabuli_chr05	10012365	[A/C]	1067297331
Ca_Kabuli_NC_021164.1_10293383	Ca_kabuli_chr05	10293383	[A/T]	1067297332
Ca_Kabuli_NC_021164.1_10293384	Ca_kabuli_chr05	10293384	[T/C]	1067297333
Ca_Kabuli_NC_021164.1_10293386	Ca_kabuli_chr05	10293386	[T/C]	1067297334

SNP IDs	Chromosomes	Physical positions (bp)	SNPs	NCBI SNPdb accession IDs (NCBI_ss#)
Ca_Kabuli_NC_021164.1_10293388	Ca_kabuli_chr05	10293388	[A/T]	1067297335
Ca_Kabuli_NC_021164.1_10293434	Ca_kabuli_chr05	10293434	[T/C]	1067297336
Ca_Kabuli_NC_021164.1_10293445	Ca_kabuli_chr05	10293445	[T/A]	1067297337
Ca_Kabuli_NC_021164.1_10318805	Ca_kabuli_chr05	10318805	[A/T]	1067297338
Ca_Kabuli_NC_021164.1_10510124	Ca_kabuli_chr05	10510124	[T/G]	1067297339
Ca_Kabuli_NC_021164.1_10510126	Ca_kabuli_chr05	10510126	[T/G]	1067297340
Ca_Kabuli_NC_021164.1_10544854	Ca_kabuli_chr05	10544854	[T/A]	1067297341
Ca_Kabuli_NC_021164.1_10919414	Ca_kabuli_chr05	10919414	[C/A]	1067297342
Ca_Kabuli_NC_021164.1_10919423	Ca_kabuli_chr05	10919423	[G/A]	1067297343
Ca_Kabuli_NC_021164.1_10919431	Ca_kabuli_chr05	10919431	[G/A]	1067297344
Ca_Kabuli_NC_021164.1_10919455	Ca_kabuli_chr05	10919455	[A/T]	1067297345
Ca_Kabuli_NC_021164.1_10928968	Ca_kabuli_chr05	10928968	[T/G]	1067297346
Ca_Kabuli_NC_021164.1_10929610	Ca_kabuli_chr05	10929610	[T/G]	1067297347
Ca_Kabuli_NC_021164.1_11110859	Ca_kabuli_chr05	11110859	[T/C]	1067297348
Ca_Kabuli_NC_021164.1_11112101	Ca_kabuli_chr05	11112101	[T/G]	1067297349
Ca_Kabuli_NC_021164.1_11208382	Ca_kabuli_chr05	11208382	[G/A]	1067297350
Ca_Kabuli_NC_021164.1_11208390	Ca_kabuli_chr05	11208390	[C/A]	1067297351
Ca_Kabuli_NC_021164.1_11356511	Ca_kabuli_chr05	11356511	[G/A]	1067297352
Ca_Kabuli_NC_021164.1_11385791	Ca_kabuli_chr05	11385791	[T/A]	1067297353
Ca_Kabuli_NC_021164.1_11385793	Ca_kabuli_chr05	11385793	[A/G]	1067297354
Ca_Kabuli_NC_021164.1_11484424	Ca_kabuli_chr05	11484424	[A/G]	1067297355
Ca_Kabuli_NC_021164.1_11614171	Ca_kabuli_chr05	11614171	[T/C]	1067297356
Ca_Kabuli_NC_021164.1_11614191	Ca_kabuli_chr05	11614191	[T/C]	1067297357
Ca_Kabuli_NC_021164.1_11614193	Ca_kabuli_chr05	11614193	[T/C]	1067297358
Ca_Kabuli_NC_021164.1_11614203	Ca_kabuli_chr05	11614203	[A/T]	1067297359
Ca_Kabuli_NC_021164.1_11614206	Ca_kabuli_chr05	11614206	[T/C]	1067297360
Ca_Kabuli_NC_021164.1_11614211	Ca_kabuli_chr05	11614211	[T/C]	1067297361
Ca_Kabuli_NC_021164.1_11699180	Ca_kabuli_chr05	11699180	[A/C]	1067297362
Ca_Kabuli_NC_021164.1_11745910	Ca_kabuli_chr05	11745910	[G/A]	1067297363
Ca_Kabuli_NC_021164.1_11745911	Ca_kabuli_chr05	11745911	[T/A]	1067297364
Ca_Kabuli_NC_021164.1_11745916	Ca_kabuli_chr05	11745916	[A/G]	1067297365
Ca_Kabuli_NC_021164.1_11745922	Ca_kabuli_chr05	11745922	[A/G]	1067297366
Ca_Kabuli_NC_021164.1_11779651	Ca_kabuli_chr05	11779651	[A/G]	1067297367
Ca_Kabuli_NC_021164.1_11779703	Ca_kabuli_chr05	11779703	[A/G]	1067297368

SNP IDs	Chromosomes	Physical positions (bp)	SNPs	NCBI SNPdb accession IDs (NCBI_ss#)
Ca_Kabuli_NC_021164.1_11845508	Ca_kabuli_chr05	11845508	[A/G]	1067297369
Ca_Kabuli_NC_021164.1_11845514	Ca_kabuli_chr05	11845514	[A/C]	1067297370
Ca_Kabuli_NC_021164.1_11847809	Ca_kabuli_chr05	11847809	[T/A]	1067297371
Ca_Kabuli_NC_021164.1_11847835	Ca_kabuli_chr05	11847835	[T/G]	1067297372
Ca_Kabuli_NC_021164.1_11848040	Ca_kabuli_chr05	11848040	[A/G]	1067297373
Ca_Kabuli_NC_021164.1_11848092	Ca_kabuli_chr05	11848092	[A/T]	1067297374
Ca_Kabuli_NC_021164.1_11864912	Ca_kabuli_chr05	11864912	[A/C]	1067297375
Ca_Kabuli_NC_021164.1_11870832	Ca_kabuli_chr05	11870832	[T/C]	1067297376
Ca_Kabuli_NC_021164.1_11870849	Ca_kabuli_chr05	11870849	[A/G]	1067297377
Ca_Kabuli_NC_021164.1_11983308	Ca_kabuli_chr05	11983308	[T/G]	1067297378
Ca_Kabuli_NC_021164.1_11996101	Ca_kabuli_chr05	11996101	[A/C]	1067297379
Ca_Kabuli_NC_021164.1_12082036	Ca_kabuli_chr05	12082036	[A/G]	1067297380
Ca_Kabuli_NC_021164.1_12082066	Ca_kabuli_chr05	12082066	[A/G]	1067297381
Ca_Kabuli_NC_021164.1_12162166	Ca_kabuli_chr05	12162166	[G/T]	1067297382
Ca_Kabuli_NC_021164.1_12410192	Ca_kabuli_chr05	12410192	[T/C]	1067297383
Ca_Kabuli_NC_021164.1_12427951	Ca_kabuli_chr05	12427951	[A/T]	1067297384
Ca_Kabuli_NC_021164.1_12435649	Ca_kabuli_chr05	12435649	[C/A]	1067297385
Ca_Kabuli_NC_021164.1_12435650	Ca_kabuli_chr05	12435650	[T/G]	1067297386
Ca_Kabuli_NC_021164.1_12435651	Ca_kabuli_chr05	12435651	[T/A]	1067297387
Ca_Kabuli_NC_021164.1_12485979	Ca_kabuli_chr05	12485979	[T/A]	1067297388
Ca_Kabuli_NC_021164.1_12553443	Ca_kabuli_chr05	12553443	[A/C]	1067297389
Ca_Kabuli_NC_021164.1_12553623	Ca_kabuli_chr05	12553623	[A/C]	1067297390
Ca_Kabuli_NC_021164.1_12553701	Ca_kabuli_chr05	12553701	[A/G]	1067297391
Ca_Kabuli_NC_021164.1_12553723	Ca_kabuli_chr05	12553723	[A/G]	1067297392
Ca_Kabuli_NC_021164.1_12590478	Ca_kabuli_chr05	12590478	[C/T]	1067297393
Ca_Kabuli_NC_021164.1_12590512	Ca_kabuli_chr05	12590512	[G/A]	1067297394
Ca_Kabuli_NC_021164.1_13981962	Ca_kabuli_chr05	13981962	[T/G]	1067297395
Ca_Kabuli_NC_021164.1_14168818	Ca_kabuli_chr05	14168818	[T/G]	1067297396
Ca_Kabuli_NC_021164.1_15401236	Ca_kabuli_chr05	15401236	[T/C]	1067297397
Ca_Kabuli_NC_021164.1_15476287	Ca_kabuli_chr05	15476287	[T/G]	1067297398
Ca_Kabuli_NC_021164.1_15476661	Ca_kabuli_chr05	15476661	[T/G]	1067297399
Ca_Kabuli_NC_021164.1_15500774	Ca_kabuli_chr05	15500774	[T/G]	1067297400
Ca_Kabuli_NC_021164.1_15500785	Ca_kabuli_chr05	15500785	[T/A]	1067297401
Ca_Kabuli_NC_021164.1_15777671	Ca_kabuli_chr05	15777671	[T/C]	1067297402

SNP IDs	Chromosomes	Physical positions (bp)	SNPs	NCBI SNPdb accession IDs (NCBI_ss#)
Ca_Kabuli_NC_021164.1_15777675	Ca_kabuli_chr05	15777675	[A/G]	1067297403
Ca_Kabuli_NC_021164.1_15777680	Ca_kabuli_chr05	15777680	[A/C]	1067297404
Ca_Kabuli_NC_021164.1_15777696	Ca_kabuli_chr05	15777696	[A/G]	1067297405
Ca_Kabuli_NC_021164.1_15777707	Ca_kabuli_chr05	15777707	[A/G]	1067297406
Ca_Kabuli_NC_021164.1_15777723	Ca_kabuli_chr05	15777723	[T/G]	1067297407
Ca_Kabuli_NC_021164.1_15777729	Ca_kabuli_chr05	15777729	[A/G]	1067297408
Ca_Kabuli_NC_021164.1_15777737	Ca_kabuli_chr05	15777737	[A/G]	1067297409
Ca_Kabuli_NC_021164.1_16353372	Ca_kabuli_chr05	16353372	[T/G]	1067297410
Ca_Kabuli_NC_021164.1_16591550	Ca_kabuli_chr05	16591550	[A/C]	1067297411
Ca_Kabuli_NC_021164.1_16594090	Ca_kabuli_chr05	16594090	[T/C]	1067297412
Ca_Kabuli_NC_021164.1_16594092	Ca_kabuli_chr05	16594092	[A/G]	1067297413
Ca_Kabuli_NC_021164.1_16676418	Ca_kabuli_chr05	16676418	[T/G]	1067297414
Ca_Kabuli_NC_021164.1_16676438	Ca_kabuli_chr05	16676438	[T/C]	1067297415
Ca_Kabuli_NC_021164.1_16767050	Ca_kabuli_chr05	16767050	[T/A]	1067297416
Ca_Kabuli_NC_021164.1_16772243	Ca_kabuli_chr05	16772243	[A/G]	1067297417
Ca_Kabuli_NC_021164.1_16929891	Ca_kabuli_chr05	16929891	[T/G]	1067297418
Ca_Kabuli_NC_021164.1_16929894	Ca_kabuli_chr05	16929894	[C/G]	1067297419
Ca_Kabuli_NC_021164.1_16929896	Ca_kabuli_chr05	16929896	[A/G]	1067297420
Ca_Kabuli_NC_021164.1_16929897	Ca_kabuli_chr05	16929897	[A/G]	1067297421
Ca_Kabuli_NC_021164.1_16929903	Ca_kabuli_chr05	16929903	[C/G]	1067297422
Ca_Kabuli_NC_021164.1_17043790	Ca_kabuli_chr05	17043790	[A/T]	1067297423
Ca_Kabuli_NC_021164.1_17043811	Ca_kabuli_chr05	17043811	[G/C]	1067297424
Ca_Kabuli_NC_021164.1_17043824	Ca_kabuli_chr05	17043824	[A/T]	1067297425
Ca_Kabuli_NC_021164.1_17043827	Ca_kabuli_chr05	17043827	[G/T]	1067297426
Ca_Kabuli_NC_021164.1_17494697	Ca_kabuli_chr05	17494697	[C/T]	1067297427
Ca_Kabuli_NC_021164.1_17494699	Ca_kabuli_chr05	17494699	[C/A]	1067297428
Ca_Kabuli_NC_021164.1_17494700	Ca_kabuli_chr05	17494700	[T/C]	1067297429
Ca_Kabuli_NC_021164.1_17494702	Ca_kabuli_chr05	17494702	[G/A]	1067297430
Ca_Kabuli_NC_021164.1_17494705	Ca_kabuli_chr05	17494705	[A/T]	1067297431
Ca_Kabuli_NC_021164.1_17494710	Ca_kabuli_chr05	17494710	[C/T]	1067297432
Ca_Kabuli_NC_021164.1_17494714	Ca_kabuli_chr05	17494714	[A/G]	1067297433
Ca_Kabuli_NC_021164.1_17494725	Ca_kabuli_chr05	17494725	[C/T]	1067297434
Ca_Kabuli_NC_021164.1_18149854	Ca_kabuli_chr05	18149854	[T/A]	1067297435
Ca_Kabuli_NC_021164.1_18150640	Ca_kabuli_chr05	18150640	[A/T]	1067297436

SNP IDs	Chromosomes	Physical positions (bp)	SNPs	NCBI SNPdb accession IDs (NCBI_ss#)
Ca_Kabuli_NC_021164.1_18150658	Ca_kabuli_chr05	18150658	[A/G]	1067297437
Ca_Kabuli_NC_021164.1_18296460	Ca_kabuli_chr05	18296460	[A/C]	1067297438
Ca_Kabuli_NC_021164.1_18543600	Ca_kabuli_chr05	18543600	[A/T]	1067297439
Ca_Kabuli_NC_021164.1_18543616	Ca_kabuli_chr05	18543616	[A/G]	1067297440
Ca_Kabuli_NC_021164.1_18732773	Ca_kabuli_chr05	18732773	[A/C]	1067297441
Ca_Kabuli_NC_021164.1_18889146	Ca_kabuli_chr05	18889146	[G/T]	1067297442
Ca_Kabuli_NC_021164.1_18971088	Ca_kabuli_chr05	18971088	[C/A]	1067297443
Ca_Kabuli_NC_021164.1_18971091	Ca_kabuli_chr05	18971091	[T/G]	1067297444
Ca_Kabuli_NC_021164.1_19716362	Ca_kabuli_chr05	19716362	[G/A]	1067297445
Ca_Kabuli_NC_021164.1_19719893	Ca_kabuli_chr05	19719893	[T/C]	1067297446
Ca_Kabuli_NC_021164.1_19720404	Ca_kabuli_chr05	19720404	[C/T]	1067297447
Ca_Kabuli_NC_021164.1_19859837	Ca_kabuli_chr05	19859837	[T/G]	1067297448
Ca_Kabuli_NC_021164.1_19886380	Ca_kabuli_chr05	19886380	[G/C]	1067297449
Ca_Kabuli_NC_021164.1_19886396	Ca_kabuli_chr05	19886396	[A/C]	1067297450
Ca_Kabuli_NC_021164.1_19886405	Ca_kabuli_chr05	19886405	[T/C]	1067297451
Ca_Kabuli_NC_021164.1_19886414	Ca_kabuli_chr05	19886414	[A/C]	1067297452
Ca_Kabuli_NC_021164.1_19886958	Ca_kabuli_chr05	19886958	[T/C]	1067297453
Ca_Kabuli_NC_021164.1_19886967	Ca_kabuli_chr05	19886967	[A/G]	1067297454
Ca_Kabuli_NC_021164.1_19886970	Ca_kabuli_chr05	19886970	[T/C]	1067297455
Ca_Kabuli_NC_021164.1_19886973	Ca_kabuli_chr05	19886973	[A/T]	1067297456
Ca_Kabuli_NC_021164.1_20354222	Ca_kabuli_chr05	20354222	[A/T]	1067297457
Ca_Kabuli_NC_021164.1_20354249	Ca_kabuli_chr05	20354249	[A/G]	1067297458
Ca_Kabuli_NC_021164.1_20354258	Ca_kabuli_chr05	20354258	[T/A]	1067297459
Ca_Kabuli_NC_021164.1_20370644	Ca_kabuli_chr05	20370644	[G/C]	1067297460
Ca_Kabuli_NC_021164.1_20443993	Ca_kabuli_chr05	20443993	[A/G]	1067297461
Ca_Kabuli_NC_021164.1_20443995	Ca_kabuli_chr05	20443995	[A/G]	1067297462
Ca_Kabuli_NC_021164.1_20443996	Ca_kabuli_chr05	20443996	[A/C]	1067297463
Ca_Kabuli_NC_021164.1_20460859	Ca_kabuli_chr05	20460859	[T/G]	1067297464
Ca_Kabuli_NC_021164.1_20460873	Ca_kabuli_chr05	20460873	[A/G]	1067297465
Ca_Kabuli_NC_021164.1_20460882	Ca_kabuli_chr05	20460882	[A/G]	1067297466
Ca_Kabuli_NC_021164.1_20546402	Ca_kabuli_chr05	20546402	[T/G]	1067297467
Ca_Kabuli_NC_021164.1_20645027	Ca_kabuli_chr05	20645027	[A/G]	1067297468
Ca_Kabuli_NC_021164.1_20645036	Ca_kabuli_chr05	20645036	[A/G]	1067297469
Ca_Kabuli_NC_021164.1_21033841	Ca_kabuli_chr05	21033841	[A/C]	1067297470

SNP IDs	Chromosomes	Physical positions (bp)	SNPs	NCBI SNPdb accession IDs (NCBI_ss#)
Ca_Kabuli_NC_021164.1_21033852	Ca_kabuli_chr05	21033852	[A/G]	1067297471
Ca_Kabuli_NC_021164.1_21033919	Ca_kabuli_chr05	21033919	[A/C]	1067297472
Ca_Kabuli_NC_021164.1_21033922	Ca_kabuli_chr05	21033922	[C/T]	1067297473
Ca_Kabuli_NC_021164.1_21033923	Ca_kabuli_chr05	21033923	[C/T]	1067297474
Ca_Kabuli_NC_021164.1_21033926	Ca_kabuli_chr05	21033926	[C/T]	1067297475
Ca_Kabuli_NC_021164.1_21033969	Ca_kabuli_chr05	21033969	[A/C]	1067297476
Ca_Kabuli_NC_021164.1_21095660	Ca_kabuli_chr05	21095660	[T/C]	1067297477
Ca_Kabuli_NC_021164.1_21163371	Ca_kabuli_chr05	21163371	[A/G]	1067297478
Ca_Kabuli_NC_021164.1_21163435	Ca_kabuli_chr05	21163435	[A/G]	1067297479
Ca_Kabuli_NC_021164.1_21163447	Ca_kabuli_chr05	21163447	[A/C]	1067297480
Ca_Kabuli_NC_021164.1_21163453	Ca_kabuli_chr05	21163453	[A/T]	1067297481
Ca_Kabuli_NC_021164.1_21166082	Ca_kabuli_chr05	21166082	[A/T]	1067297482
Ca_Kabuli_NC_021164.1_21188830	Ca_kabuli_chr05	21188830	[T/A]	1067297483
Ca_Kabuli_NC_021164.1_21231054	Ca_kabuli_chr05	21231054	[G/C]	1067297484
Ca_Kabuli_NC_021164.1_21231055	Ca_kabuli_chr05	21231055	[G/T]	1067297485
Ca_Kabuli_NC_021164.1_21495508	Ca_kabuli_chr05	21495508	[G/A]	1067297486
Ca_Kabuli_NC_021164.1_21770485	Ca_kabuli_chr05	21770485	[T/C]	1067297487
Ca_Kabuli_NC_021164.1_21770503	Ca_kabuli_chr05	21770503	[T/C]	1067297488
Ca_Kabuli_NC_021164.1_21933944	Ca_kabuli_chr05	21933944	[A/C]	1067297489
Ca_Kabuli_NC_021164.1_21933954	Ca_kabuli_chr05	21933954	[A/C]	1067297490
Ca_Kabuli_NC_021164.1_21933957	Ca_kabuli_chr05	21933957	[G/C]	1067297491
Ca_Kabuli_NC_021164.1_22046195	Ca_kabuli_chr05	22046195	[A/C]	1067297492
Ca_Kabuli_NC_021164.1_22065859	Ca_kabuli_chr05	22065859	[G/A]	1067297493
Ca_Kabuli_NC_021164.1_22116956	Ca_kabuli_chr05	22116956	[T/G]	1067297494
Ca_Kabuli_NC_021164.1_22116961	Ca_kabuli_chr05	22116961	[T/G]	1067297495
Ca_Kabuli_NC_021164.1_22296799	Ca_kabuli_chr05	22296799	[G/A]	1067297496
Ca_Kabuli_NC_021164.1_22373590	Ca_kabuli_chr05	22373590	[T/G]	1067297497
Ca_Kabuli_NC_021164.1_22430965	Ca_kabuli_chr05	22430965	[A/G]	1067297498
Ca_Kabuli_NC_021164.1_22430967	Ca_kabuli_chr05	22430967	[A/G]	1067297499
Ca_Kabuli_NC_021164.1_22431041	Ca_kabuli_chr05	22431041	[T/G]	1067297500
Ca_Kabuli_NC_021164.1_22446077	Ca_kabuli_chr05	22446077	[A/C]	1067297501
Ca_Kabuli_NC_021164.1_22484728	Ca_kabuli_chr05	22484728	[T/G]	1067297502
Ca_Kabuli_NC_021164.1_22627341	Ca_kabuli_chr05	22627341	[A/C]	1067297503
Ca_Kabuli_NC_021164.1_22695359	Ca_kabuli_chr05	22695359	[T/G]	1067297504

SNP IDs	Chromosomes	Physical positions (bp)	SNPs	NCBI SNPdb accession IDs (NCBI_ss#)
Ca_Kabuli_NC_021164.1_22695584	Ca_kabuli_chr05	22695584	[A/C]	1067297505
Ca_Kabuli_NC_021164.1_22695590	Ca_kabuli_chr05	22695590	[C/T]	1067297506
Ca_Kabuli_NC_021164.1_22695666	Ca_kabuli_chr05	22695666	[G/T]	1067297507
Ca_Kabuli_NC_021164.1_22763707	Ca_kabuli_chr05	22763707	[A/G]	1067297508
Ca_Kabuli_NC_021164.1_22763708	Ca_kabuli_chr05	22763708	[T/C]	1067297509
Ca_Kabuli_NC_021164.1_22763710	Ca_kabuli_chr05	22763710	[A/G]	1067297510
Ca_Kabuli_NC_021164.1_22763712	Ca_kabuli_chr05	22763712	[A/C]	1067297511
Ca_Kabuli_NC_021164.1_22763716	Ca_kabuli_chr05	22763716	[A/C]	1067297512
Ca_Kabuli_NC_021164.1_22763726	Ca_kabuli_chr05	22763726	[T/C]	1067297513
Ca_Kabuli_NC_021164.1_23063384	Ca_kabuli_chr05	23063384	[T/C]	1067297514
Ca_Kabuli_NC_021164.1_23151994	Ca_kabuli_chr05	23151994	[A/C]	1067297515
Ca_Kabuli_NC_021164.1_23152020	Ca_kabuli_chr05	23152020	[T/A]	1067297516
Ca_Kabuli_NC_021164.1_23157944	Ca_kabuli_chr05	23157944	[A/C]	1067297517
Ca_Kabuli_NC_021164.1_23161972	Ca_kabuli_chr05	23161972	[T/G]	1067297518
Ca_Kabuli_NC_021164.1_23161993	Ca_kabuli_chr05	23161993	[T/C]	1067297519
Ca_Kabuli_NC_021164.1_23318702	Ca_kabuli_chr05	23318702	[T/A]	1067297520
Ca_Kabuli_NC_021164.1_23440279	Ca_kabuli_chr05	23440279	[A/G]	1067297521
Ca_Kabuli_NC_021164.1_23440287	Ca_kabuli_chr05	23440287	[T/G]	1067297522
Ca_Kabuli_NC_021164.1_23449134	Ca_kabuli_chr05	23449134	[A/C]	1067297523
Ca_Kabuli_NC_021164.1_23661904	Ca_kabuli_chr05	23661904	[G/C]	1067297524
Ca_Kabuli_NC_021164.1_23663999	Ca_kabuli_chr05	23663999	[A/C]	1067297525
Ca_Kabuli_NC_021164.1_23669836	Ca_kabuli_chr05	23669836	[G/T]	1067297526
Ca_Kabuli_NC_021164.1_23673391	Ca_kabuli_chr05	23673391	[T/C]	1067297527
Ca_Kabuli_NC_021164.1_23673912	Ca_kabuli_chr05	23673912	[T/C]	1067297528
Ca_Kabuli_NC_021164.1_23674378	Ca_kabuli_chr05	23674378	[T/A]	1067297529
Ca_Kabuli_NC_021164.1_23674385	Ca_kabuli_chr05	23674385	[C/A]	1067297530
Ca_Kabuli_NC_021164.1_23674574	Ca_kabuli_chr05	23674574	[A/G]	1067297531
Ca_Kabuli_NC_021164.1_23674577	Ca_kabuli_chr05	23674577	[A/C]	1067297532
Ca_Kabuli_NC_021164.1_23674580	Ca_kabuli_chr05	23674580	[A/C]	1067297533
Ca_Kabuli_NC_021164.1_23683507	Ca_kabuli_chr05	23683507	[A/C]	1067297534
Ca_Kabuli_NC_021164.1_23896621	Ca_kabuli_chr05	23896621	[G/C]	1067297535
Ca_Kabuli_NC_021164.1_23896642	Ca_kabuli_chr05	23896642	[T/C]	1067297536
Ca_Kabuli_NC_021164.1_23896645	Ca_kabuli_chr05	23896645	[A/G]	1067297537
Ca_Kabuli_NC_021164.1_23896648	Ca_kabuli_chr05	23896648	[A/G]	1067297538

SNP IDs	Chromosomes	Physical positions (bp)	SNPs	NCBI SNPdb accession IDs (NCBI_ss#)
Ca_Kabuli_NC_021164.1_23896654	Ca_kabuli_chr05	23896654	[A/G]	1067297539
Ca_Kabuli_NC_021164.1_23896659	Ca_kabuli_chr05	23896659	[A/G]	1067297540
Ca_Kabuli_NC_021164.1_23900520	Ca_kabuli_chr05	23900520	[T/G]	1067297541
Ca_Kabuli_NC_021164.1_23930960	Ca_kabuli_chr05	23930960	[T/A]	1067297542
Ca_Kabuli_NC_021164.1_24090523	Ca_kabuli_chr05	24090523	[A/C]	1067297543
Ca_Kabuli_NC_021164.1_24346246	Ca_kabuli_chr05	24346246	[C/A]	1067297544
Ca_Kabuli_NC_021164.1_24346247	Ca_kabuli_chr05	24346247	[C/T]	1067297545
Ca_Kabuli_NC_021164.1_24346248	Ca_kabuli_chr05	24346248	[A/C]	1067297546
Ca_Kabuli_NC_021164.1_24346249	Ca_kabuli_chr05	24346249	[G/T]	1067297547
Ca_Kabuli_NC_021164.1_24453018	Ca_kabuli_chr05	24453018	[T/C]	1067297548
Ca_Kabuli_NC_021164.1_24453047	Ca_kabuli_chr05	24453047	[T/C]	1067297549
Ca_Kabuli_NC_021164.1_24455862	Ca_kabuli_chr05	24455862	[T/C]	1067297550
Ca_Kabuli_NC_021164.1_24456308	Ca_kabuli_chr05	24456308	[A/C]	1067297551
Ca_Kabuli_NC_021164.1_24487926	Ca_kabuli_chr05	24487926	[T/G]	1067297552
Ca_Kabuli_NC_021164.1_24497690	Ca_kabuli_chr05	24497690	[T/G]	1067297553
Ca_Kabuli_NC_021164.1_24500906	Ca_kabuli_chr05	24500906	[T/C]	1067297554
Ca_Kabuli_NC_021164.1_24539412	Ca_kabuli_chr05	24539412	[A/T]	1067297555
Ca_Kabuli_NC_021164.1_24596240	Ca_kabuli_chr05	24596240	[T/G]	1067297556
Ca_Kabuli_NC_021164.1_24596258	Ca_kabuli_chr05	24596258	[A/G]	1067297557
Ca_Kabuli_NC_021164.1_24811305	Ca_kabuli_chr05	24811305	[T/G]	1067297558
Ca_Kabuli_NC_021164.1_24822626	Ca_kabuli_chr05	24822626	[A/C]	1067297559
Ca_Kabuli_NC_021164.1_24822630	Ca_kabuli_chr05	24822630	[A/C]	1067297560
Ca_Kabuli_NC_021164.1_24822635	Ca_kabuli_chr05	24822635	[A/C]	1067297561
Ca_Kabuli_NC_021164.1_24822644	Ca_kabuli_chr05	24822644	[T/C]	1067297562
Ca_Kabuli_NC_021164.1_24822651	Ca_kabuli_chr05	24822651	[T/G]	1067297563
Ca_Kabuli_NC_021164.1_24822652	Ca_kabuli_chr05	24822652	[T/C]	1067297564
Ca_Kabuli_NC_021164.1_24822654	Ca_kabuli_chr05	24822654	[C/T]	1067297565
Ca_Kabuli_NC_021164.1_24822656	Ca_kabuli_chr05	24822656	[C/T]	1067297566
Ca_Kabuli_NC_021164.1_24822660	Ca_kabuli_chr05	24822660	[C/T]	1067297567
Ca_Kabuli_NC_021164.1_24822664	Ca_kabuli_chr05	24822664	[C/T]	1067297568
Ca_Kabuli_NC_021164.1_24843049	Ca_kabuli_chr05	24843049	[A/C]	1067297569
Ca_Kabuli_NC_021164.1_24843053	Ca_kabuli_chr05	24843053	[A/T]	1067297570
Ca_Kabuli_NC_021164.1_24843085	Ca_kabuli_chr05	24843085	[A/T]	1067297571
Ca_Kabuli_NC_021164.1_24985735	Ca_kabuli_chr05	24985735	[T/G]	1067297572

SNP IDs	Chromosomes	Physical positions (bp)	SNPs	NCBI SNPdb accession IDs (NCBI_ss#)
Ca_Kabuli_NC_021164.1_24985904	Ca_kabuli_chr05	24985904	[T/G]	1067297573
Ca_Kabuli_NC_021164.1_25006128	Ca_kabuli_chr05	25006128	[T/G]	1067297574
Ca_Kabuli_NC_021164.1_25006227	Ca_kabuli_chr05	25006227	[A/C]	1067297575
Ca_Kabuli_NC_021164.1_25120905	Ca_kabuli_chr05	25120905	[T/A]	1067297576
Ca_Kabuli_NC_021164.1_25120950	Ca_kabuli_chr05	25120950	[A/C]	1067297577
Ca_Kabuli_NC_021164.1_25333468	Ca_kabuli_chr05	25333468	[T/A]	1067297578
Ca_Kabuli_NC_021164.1_25400510	Ca_kabuli_chr05	25400510	[T/C]	1067297579
Ca_Kabuli_NC_021164.1_25400568	Ca_kabuli_chr05	25400568	[A/G]	1067297580
Ca_Kabuli_NC_021164.1_25435023	Ca_kabuli_chr05	25435023	[A/T]	1067297581
Ca_Kabuli_NC_021164.1_25437861	Ca_kabuli_chr05	25437861	[T/A]	1067297582
Ca_Kabuli_NC_021164.1_25437871	Ca_kabuli_chr05	25437871	[A/G]	1067297583
Ca_Kabuli_NC_021164.1_25437888	Ca_kabuli_chr05	25437888	[A/G]	1067297584
Ca_Kabuli_NC_021164.1_25457957	Ca_kabuli_chr05	25457957	[T/G]	1067297585
Ca_Kabuli_NC_021164.1_25679023	Ca_kabuli_chr05	25679023	[C/T]	1067297586
Ca_Kabuli_NC_021164.1_25679048	Ca_kabuli_chr05	25679048	[T/C]	1067297587
Ca_Kabuli_NC_021164.1_25697491	Ca_kabuli_chr05	25697491	[G/A]	1067297588
Ca_Kabuli_NC_021164.1_25697492	Ca_kabuli_chr05	25697492	[A/C]	1067297589
Ca_Kabuli_NC_021164.1_25697494	Ca_kabuli_chr05	25697494	[G/A]	1067297590
Ca_Kabuli_NC_021164.1_25697512	Ca_kabuli_chr05	25697512	[T/C]	1067297591
Ca_Kabuli_NC_021164.1_25747850	Ca_kabuli_chr05	25747850	[T/A]	1067297592
Ca_Kabuli_NC_021164.1_26095637	Ca_kabuli_chr05	26095637	[T/A]	1067297593
Ca_Kabuli_NC_021164.1_26095693	Ca_kabuli_chr05	26095693	[A/C]	1067297594
Ca_Kabuli_NC_021164.1_26124304	Ca_kabuli_chr05	26124304	[A/G]	1067297595
Ca_Kabuli_NC_021164.1_26201244	Ca_kabuli_chr05	26201244	[T/C]	1067297596
Ca_Kabuli_NC_021164.1_26290961	Ca_kabuli_chr05	26290961	[T/G]	1067297597
Ca_Kabuli_NC_021164.1_26290999	Ca_kabuli_chr05	26290999	[T/G]	1067297598
Ca_Kabuli_NC_021164.1_26319766	Ca_kabuli_chr05	26319766	[T/G]	1067297599
Ca_Kabuli_NC_021164.1_26325980	Ca_kabuli_chr05	26325980	[A/C]	1067297600
Ca_Kabuli_NC_021164.1_26384675	Ca_kabuli_chr05	26384675	[A/C]	1067297601
Ca_Kabuli_NC_021164.1_26474873	Ca_kabuli_chr05	26474873	[A/C]	1067297602
Ca_Kabuli_NC_021164.1_26474935	Ca_kabuli_chr05	26474935	[T/G]	1067297603
Ca_Kabuli_NC_021164.1_26474963	Ca_kabuli_chr05	26474963	[C/T]	1067297604
Ca_Kabuli_NC_021164.1_26474966	Ca_kabuli_chr05	26474966	[G/A]	1067297605
Ca_Kabuli_NC_021164.1_26474971	Ca_kabuli_chr05	26474971	[A/C]	1067297606

SNP IDs	Chromosomes	Physical positions (bp)	SNPs	NCBI SNPdb accession IDs (NCBI_ss#)
Ca_Kabuli_NC_021164.1_26475111	Ca_kabuli_chr05	26475111	[A/C]	1067297607
Ca_Kabuli_NC_021164.1_26569427	Ca_kabuli_chr05	26569427	[A/G]	1067297608
Ca_Kabuli_NC_021164.1_26605894	Ca_kabuli_chr05	26605894	[T/G]	1067297609
Ca_Kabuli_NC_021164.1_26605966	Ca_kabuli_chr05	26605966	[A/G]	1067297610
Ca_Kabuli_NC_021164.1_26626832	Ca_kabuli_chr05	26626832	[T/G]	1067297611
Ca_Kabuli_NC_021164.1_26626855	Ca_kabuli_chr05	26626855	[T/A]	1067297612
Ca_Kabuli_NC_021164.1_26696323	Ca_kabuli_chr05	26696323	[T/C]	1067297613
Ca_Kabuli_NC_021164.1_26696378	Ca_kabuli_chr05	26696378	[A/G]	1067297614
Ca_Kabuli_NC_021164.1_26696533	Ca_kabuli_chr05	26696533	[C/T]	1067297615
Ca_Kabuli_NC_021164.1_26987679	Ca_kabuli_chr05	26987679	[G/A]	1067297616
Ca_Kabuli_NC_021164.1_26987707	Ca_kabuli_chr05	26987707	[C/T]	1067297617
Ca_Kabuli_NC_021164.1_26997313	Ca_kabuli_chr05	26997313	[T/C]	1067297618
Ca_Kabuli_NC_021164.1_26997335	Ca_kabuli_chr05	26997335	[T/G]	1067297619
Ca_Kabuli_NC_021164.1_26997539	Ca_kabuli_chr05	26997539	[A/C]	1067297620
Ca_Kabuli_NC_021164.1_26997543	Ca_kabuli_chr05	26997543	[A/T]	1067297621
Ca_Kabuli_NC_021164.1_26999390	Ca_kabuli_chr05	26999390	[A/C]	1067297622
Ca_Kabuli_NC_021164.1_26999761	Ca_kabuli_chr05	26999761	[T/C]	1067297623
Ca_Kabuli_NC_021164.1_27000075	Ca_kabuli_chr05	27000075	[A/G]	1067297624
Ca_Kabuli_NC_021164.1_27000090	Ca_kabuli_chr05	27000090	[A/G]	1067297625
Ca_Kabuli_NC_021164.1_27000227	Ca_kabuli_chr05	27000227	[A/C]	1067297626
Ca_Kabuli_NC_021164.1_27000258	Ca_kabuli_chr05	27000258	[G/A]	1067297627
Ca_Kabuli_NC_021164.1_27000259	Ca_kabuli_chr05	27000259	[G/A]	1067297628
Ca_Kabuli_NC_021164.1_27000994	Ca_kabuli_chr05	27000994	[A/T]	1067297629
Ca_Kabuli_NC_021164.1_27001020	Ca_kabuli_chr05	27001020	[A/C]	1067297630
Ca_Kabuli_NC_021164.1_27012109	Ca_kabuli_chr05	27012109	[A/T]	1067297631
Ca_Kabuli_NC_021164.1_27150310	Ca_kabuli_chr05	27150310	[A/C]	1067297632
Ca_Kabuli_NC_021164.1_27150550	Ca_kabuli_chr05	27150550	[G/A]	1067297633
Ca_Kabuli_NC_021164.1_27181850	Ca_kabuli_chr05	27181850	[A/T]	1067297634
Ca_Kabuli_NC_021164.1_27181851	Ca_kabuli_chr05	27181851	[T/C]	1067297635
Ca_Kabuli_NC_021164.1_27282826	Ca_kabuli_chr05	27282826	[A/G]	1067297636
Ca_Kabuli_NC_021164.1_27282828	Ca_kabuli_chr05	27282828	[G/T]	1067297637
Ca_Kabuli_NC_021164.1_27333995	Ca_kabuli_chr05	27333995	[A/G]	1067297638
Ca_Kabuli_NC_021164.1_27334054	Ca_kabuli_chr05	27334054	[A/T]	1067297639
Ca_Kabuli_NC_021164.1_27334057	Ca_kabuli_chr05	27334057	[G/C]	1067297640

SNP IDs	Chromosomes	Physical positions (bp)	SNPs	NCBI SNPdb accession IDs (NCBI_ss#)
Ca_Kabuli_NC_021164.1_27361511	Ca_kabuli_chr05	27361511	[A/C]	1067297641
Ca_Kabuli_NC_021164.1_27361517	Ca_kabuli_chr05	27361517	[A/C]	1067297642
Ca_Kabuli_NC_021164.1_27361523	Ca_kabuli_chr05	27361523	[A/C]	1067297643
Ca_Kabuli_NC_021164.1_27361526	Ca_kabuli_chr05	27361526	[C/T]	1067297644
Ca_Kabuli_NC_021164.1_27361530	Ca_kabuli_chr05	27361530	[A/C]	1067297645
Ca_Kabuli_NC_021164.1_27361534	Ca_kabuli_chr05	27361534	[T/C]	1067297646
Ca_Kabuli_NC_021164.1_27361540	Ca_kabuli_chr05	27361540	[A/C]	1067297647
Ca_Kabuli_NC_021164.1_27361581	Ca_kabuli_chr05	27361581	[A/T]	1067297648
Ca_Kabuli_NC_021164.1_27361584	Ca_kabuli_chr05	27361584	[G/C]	1067297649
Ca_Kabuli_NC_021164.1_27361587	Ca_kabuli_chr05	27361587	[G/T]	1067297650
Ca_Kabuli_NC_021164.1_27361605	Ca_kabuli_chr05	27361605	[A/T]	1067297651
Ca_Kabuli_NC_021164.1_27361668	Ca_kabuli_chr05	27361668	[T/G]	1067297652
Ca_Kabuli_NC_021164.1_27362312	Ca_kabuli_chr05	27362312	[A/G]	1067297653
Ca_Kabuli_NC_021164.1_27362537	Ca_kabuli_chr05	27362537	[T/C]	1067297654
Ca_Kabuli_NC_021164.1_27362547	Ca_kabuli_chr05	27362547	[A/C]	1067297655
Ca_Kabuli_NC_021164.1_27362562	Ca_kabuli_chr05	27362562	[A/G]	1067297656
Ca_Kabuli_NC_021164.1_27362645	Ca_kabuli_chr05	27362645	[T/G]	1067297657
Ca_Kabuli_NC_021164.1_27555618	Ca_kabuli_chr05	27555618	[T/G]	1067297658
Ca_Kabuli_NC_021164.1_27555710	Ca_kabuli_chr05	27555710	[G/A]	1067297659
Ca_Kabuli_NC_021164.1_27555713	Ca_kabuli_chr05	27555713	[T/G]	1067297660
Ca_Kabuli_NC_021164.1_27626966	Ca_kabuli_chr05	27626966	[T/C]	1067297661
Ca_Kabuli_NC_021164.1_27626978	Ca_kabuli_chr05	27626978	[T/A]	1067297662
Ca_Kabuli_NC_021164.1_27686976	Ca_kabuli_chr05	27686976	[T/G]	1067297663
Ca_Kabuli_NC_021164.1_27692771	Ca_kabuli_chr05	27692771	[A/C]	1067297664
Ca_Kabuli_NC_021164.1_27692802	Ca_kabuli_chr05	27692802	[C/T]	1067297665
Ca_Kabuli_NC_021164.1_27692804	Ca_kabuli_chr05	27692804	[C/T]	1067297666
Ca_Kabuli_NC_021164.1_27694255	Ca_kabuli_chr05	27694255	[A/C]	1067297667
Ca_Kabuli_NC_021164.1_27749043	Ca_kabuli_chr05	27749043	[T/C]	1067297668
Ca_Kabuli_NC_021164.1_27749044	Ca_kabuli_chr05	27749044	[C/G]	1067297669
Ca_Kabuli_NC_021164.1_27749080	Ca_kabuli_chr05	27749080	[A/G]	1067297670
Ca_Kabuli_NC_021164.1_27749082	Ca_kabuli_chr05	27749082	[T/C]	1067297671
Ca_Kabuli_NC_021164.1_27750009	Ca_kabuli_chr05	27750009	[T/G]	1067297672
Ca_Kabuli_NC_021164.1_27750191	Ca_kabuli_chr05	27750191	[A/G]	1067297673
Ca_Kabuli_NC_021164.1_27750231	Ca_kabuli_chr05	27750231	[T/G]	1067297674

SNP IDs	Chromosomes	Physical positions (bp)	SNPs	NCBI SNPdb accession IDs (NCBI_ss#)
Ca_Kabuli_NC_021164.1_27850742	Ca_kabuli_chr05	27850742	[A/G]	1067297675
Ca_Kabuli_NC_021164.1_27874379	Ca_kabuli_chr05	27874379	[A/G]	1067297676
Ca_Kabuli_NC_021164.1_27875320	Ca_kabuli_chr05	27875320	[A/C]	1067297677
Ca_Kabuli_NC_021164.1_27884193	Ca_kabuli_chr05	27884193	[C/A]	1067297678
Ca_Kabuli_NC_021164.1_27884194	Ca_kabuli_chr05	27884194	[T/G]	1067297679
Ca_Kabuli_NC_021164.1_27884195	Ca_kabuli_chr05	27884195	[C/A]	1067297680
Ca_Kabuli_NC_021164.1_27884196	Ca_kabuli_chr05	27884196	[C/T]	1067297681
Ca_Kabuli_NC_021164.1_27884877	Ca_kabuli_chr05	27884877	[T/G]	1067297682
Ca_Kabuli_NC_021164.1_27884979	Ca_kabuli_chr05	27884979	[T/G]	1067297683
Ca_Kabuli_NC_021164.1_27932008	Ca_kabuli_chr05	27932008	[C/G]	1067297684
Ca_Kabuli_NC_021164.1_27932017	Ca_kabuli_chr05	27932017	[G/A]	1067297685
Ca_Kabuli_NC_021164.1_27932030	Ca_kabuli_chr05	27932030	[A/C]	1067297686
Ca_Kabuli_NC_021164.1_27974642	Ca_kabuli_chr05	27974642	[T/C]	1067297687
Ca_Kabuli_NC_021164.1_27974679	Ca_kabuli_chr05	27974679	[A/G]	1067297688
Ca_Kabuli_NC_021164.1_28116898	Ca_kabuli_chr05	28116898	[A/G]	1067297689
Ca_Kabuli_NC_021164.1_28162044	Ca_kabuli_chr05	28162044	[T/G]	1067297690
Ca_Kabuli_NC_021164.1_28162046	Ca_kabuli_chr05	28162046	[A/G]	1067297691
Ca_Kabuli_NC_021164.1_28239522	Ca_kabuli_chr05	28239522	[A/G]	1067297692
Ca_Kabuli_NC_021164.1_28239791	Ca_kabuli_chr05	28239791	[T/C]	1067297693
Ca_Kabuli_NC_021164.1_28335375	Ca_kabuli_chr05	28335375	[T/C]	1067297694
Ca_Kabuli_NC_021164.1_28335434	Ca_kabuli_chr05	28335434	[A/C]	1067297695
Ca_Kabuli_NC_021164.1_28335436	Ca_kabuli_chr05	28335436	[C/A]	1067297696
Ca_Kabuli_NC_021164.1_28335440	Ca_kabuli_chr05	28335440	[A/G]	1067297697
Ca_Kabuli_NC_021164.1_28335447	Ca_kabuli_chr05	28335447	[A/C]	1067297698
Ca_Kabuli_NC_021164.1_28335455	Ca_kabuli_chr05	28335455	[A/C]	1067297699
Ca_Kabuli_NC_021164.1_28360495	Ca_kabuli_chr05	28360495	[T/C]	1067297700
Ca_Kabuli_NC_021164.1_28360722	Ca_kabuli_chr05	28360722	[T/C]	1067297701
Ca_Kabuli_NC_021164.1_28376836	Ca_kabuli_chr05	28376836	[C/G]	1067297702
Ca_Kabuli_NC_021164.1_28541649	Ca_kabuli_chr05	28541649	[A/C]	1067297703
Ca_Kabuli_NC_021164.1_28541690	Ca_kabuli_chr05	28541690	[T/C]	1067297704
Ca_Kabuli_NC_021164.1_28559836	Ca_kabuli_chr05	28559836	[T/G]	1067297705
Ca_Kabuli_NC_021164.1_28559861	Ca_kabuli_chr05	28559861	[T/A]	1067297706
Ca_Kabuli_NC_021164.1_28562767	Ca_kabuli_chr05	28562767	[T/C]	1067297707
Ca_Kabuli_NC_021164.1_28581814	Ca_kabuli_chr05	28581814	[G/A]	1067297708

SNP IDs	Chromosomes	Physical positions (bp)	SNPs	NCBI SNPdb accession IDs (NCBI_ss#)
Ca_Kabuli_NC_021164.1_28581815	Ca_kabuli_chr05	28581815	[G/C]	1067297709
Ca_Kabuli_NC_021164.1_28581827	Ca_kabuli_chr05	28581827	[C/T]	1067297710
Ca_Kabuli_NC_021164.1_28582763	Ca_kabuli_chr05	28582763	[G/A]	1067297711
Ca_Kabuli_NC_021164.1_28582806	Ca_kabuli_chr05	28582806	[C/T]	1067297712
Ca_Kabuli_NC_021164.1_28690037	Ca_kabuli_chr05	28690037	[T/G]	1067297713
Ca_Kabuli_NC_021164.1_28717362	Ca_kabuli_chr05	28717362	[T/C]	1067297714
Ca_Kabuli_NC_021164.1_28717384	Ca_kabuli_chr05	28717384	[T/C]	1067297715
Ca_Kabuli_NC_021164.1_28721236	Ca_kabuli_chr05	28721236	[T/C]	1067297716
Ca_Kabuli_NC_021164.1_28721237	Ca_kabuli_chr05	28721237	[G/A]	1067297717
Ca_Kabuli_NC_021164.1_28721383	Ca_kabuli_chr05	28721383	[A/C]	1067297718
Ca_Kabuli_NC_021164.1_28721386	Ca_kabuli_chr05	28721386	[T/C]	1067297719
Ca_Kabuli_NC_021164.1_28721761	Ca_kabuli_chr05	28721761	[T/C]	1067297720
Ca_Kabuli_NC_021164.1_28721764	Ca_kabuli_chr05	28721764	[C/A]	1067297721
Ca_Kabuli_NC_021164.1_28746115	Ca_kabuli_chr05	28746115	[A/T]	1067297722
Ca_Kabuli_NC_021164.1_28746139	Ca_kabuli_chr05	28746139	[A/C]	1067297723
Ca_Kabuli_NC_021164.1_28746142	Ca_kabuli_chr05	28746142	[A/C]	1067297724
Ca_Kabuli_NC_021164.1_28748061	Ca_kabuli_chr05	28748061	[T/C]	1067297725
Ca_Kabuli_NC_021164.1_28757467	Ca_kabuli_chr05	28757467	[T/A]	1067297726
Ca_Kabuli_NC_021164.1_28757485	Ca_kabuli_chr05	28757485	[C/A]	1067297727
Ca_Kabuli_NC_021164.1_28757498	Ca_kabuli_chr05	28757498	[T/A]	1067297728
Ca_Kabuli_NC_021164.1_28757507	Ca_kabuli_chr05	28757507	[T/A]	1067297729
Ca_Kabuli_NC_021164.1_28757939	Ca_kabuli_chr05	28757939	[T/A]	1067297730
Ca_Kabuli_NC_021164.1_28757959	Ca_kabuli_chr05	28757959	[A/G]	1067297731
Ca_Kabuli_NC_021164.1_28758723	Ca_kabuli_chr05	28758723	[G/A]	1067297732
Ca_Kabuli_NC_021164.1_28813280	Ca_kabuli_chr05	28813280	[T/A]	1067297733
Ca_Kabuli_NC_021164.1_28817832	Ca_kabuli_chr05	28817832	[G/A]	1067297734
Ca_Kabuli_NC_021164.1_28842766	Ca_kabuli_chr05	28842766	[T/G]	1067297735
Ca_Kabuli_NC_021164.1_28854006	Ca_kabuli_chr05	28854006	[A/G]	1067297736
Ca_Kabuli_NC_021164.1_28854486	Ca_kabuli_chr05	28854486	[T/C]	1067297737
Ca_Kabuli_NC_021164.1_29007876	Ca_kabuli_chr05	29007876	[T/A]	1067297738
Ca_Kabuli_NC_021164.1_29007879	Ca_kabuli_chr05	29007879	[T/C]	1067297739
Ca_Kabuli_NC_021164.1_29007882	Ca_kabuli_chr05	29007882	[A/C]	1067297740
Ca_Kabuli_NC_021164.1_29007884	Ca_kabuli_chr05	29007884	[A/C]	1067297741
Ca_Kabuli_NC_021164.1_29007886	Ca_kabuli_chr05	29007886	[A/C]	1067297742

SNP IDs	Chromosomes	Physical positions (bp)	SNPs	NCBI SNPdb accession IDs (NCBI_ss#)
Ca_Kabuli_NC_021164.1_29079813	Ca_kabuli_chr05	29079813	[T/G]	1067297743
Ca_Kabuli_NC_021164.1_29079958	Ca_kabuli_chr05	29079958	[T/G]	1067297744
Ca_Kabuli_NC_021164.1_29084345	Ca_kabuli_chr05	29084345	[T/C]	1067297745
Ca_Kabuli_NC_021164.1_29222560	Ca_kabuli_chr05	29222560	[A/C]	1067297746
Ca_Kabuli_NC_021164.1_29262600	Ca_kabuli_chr05	29262600	[A/C]	1067297747
Ca_Kabuli_NC_021164.1_29272193	Ca_kabuli_chr05	29272193	[T/A]	1067297748
Ca_Kabuli_NC_021164.1_29274443	Ca_kabuli_chr05	29274443	[T/C]	1067297749
Ca_Kabuli_NC_021164.1_29274461	Ca_kabuli_chr05	29274461	[T/C]	1067297750
Ca_Kabuli_NC_021164.1_29274518	Ca_kabuli_chr05	29274518	[A/G]	1067297751
Ca_Kabuli_NC_021164.1_29274520	Ca_kabuli_chr05	29274520	[A/C]	1067297752
Ca_Kabuli_NC_021164.1_29437139	Ca_kabuli_chr05	29437139	[T/C]	1067297753
Ca_Kabuli_NC_021164.1_29437146	Ca_kabuli_chr05	29437146	[C/A]	1067297754
Ca_Kabuli_NC_021164.1_29437162	Ca_kabuli_chr05	29437162	[C/A]	1067297755
Ca_Kabuli_NC_021164.1_29437166	Ca_kabuli_chr05	29437166	[T/C]	1067297756
Ca_Kabuli_NC_021164.1_29437171	Ca_kabuli_chr05	29437171	[T/A]	1067297757
Ca_Kabuli_NC_021164.1_29531093	Ca_kabuli_chr05	29531093	[T/C]	1067297758
Ca_Kabuli_NC_021164.1_29531158	Ca_kabuli_chr05	29531158	[A/C]	1067297759
Ca_Kabuli_NC_021164.1_29608675	Ca_kabuli_chr05	29608675	[A/C]	1067297760
Ca_Kabuli_NC_021164.1_29641509	Ca_kabuli_chr05	29641509	[A/G]	1067297761
Ca_Kabuli_NC_021164.1_29641606	Ca_kabuli_chr05	29641606	[T/C]	1067297762
Ca_Kabuli_NC_021164.1_29641621	Ca_kabuli_chr05	29641621	[A/C]	1067297763
Ca_Kabuli_NC_021164.1_29641644	Ca_kabuli_chr05	29641644	[A/G]	1067297764
Ca_Kabuli_NC_021164.1_29658220	Ca_kabuli_chr05	29658220	[T/C]	1067297765
Ca_Kabuli_NC_021164.1_29658239	Ca_kabuli_chr05	29658239	[A/C]	1067297766
Ca_Kabuli_NC_021164.1_29772723	Ca_kabuli_chr05	29772723	[T/A]	1067297767
Ca_Kabuli_NC_021164.1_29772735	Ca_kabuli_chr05	29772735	[A/C]	1067297768
Ca_Kabuli_NC_021164.1_29792542	Ca_kabuli_chr05	29792542	[T/C]	1067297769
Ca_Kabuli_NC_021164.1_29792610	Ca_kabuli_chr05	29792610	[A/G]	1067297770
Ca_Kabuli_NC_021164.1_29795513	Ca_kabuli_chr05	29795513	[T/G]	1067297771
Ca_Kabuli_NC_021164.1_29809678	Ca_kabuli_chr05	29809678	[A/G]	1067297772
Ca_Kabuli_NC_021164.1_29809734	Ca_kabuli_chr05	29809734	[A/C]	1067297773
Ca_Kabuli_NC_021164.1_29886174	Ca_kabuli_chr05	29886174	[G/A]	1067297774
Ca_Kabuli_NC_021164.1_29886177	Ca_kabuli_chr05	29886177	[G/A]	1067297775
Ca_Kabuli_NC_021164.1_29886203	Ca_kabuli_chr05	29886203	[T/A]	1067297776

SNP IDs	Chromosomes	Physical positions (bp)	SNPs	NCBI SNPdb accession IDs (NCBI_ss#)
Ca_Kabuli_NC_021164.1_29968829	Ca_kabuli_chr05	29968829	[A/G]	1067297777
Ca_Kabuli_NC_021164.1_29968837	Ca_kabuli_chr05	29968837	[A/G]	1067297778
Ca_Kabuli_NC_021164.1_29974494	Ca_kabuli_chr05	29974494	[T/G]	1067297779
Ca_Kabuli_NC_021164.1_29974583	Ca_kabuli_chr05	29974583	[G/T]	1067297780
Ca_Kabuli_NC_021164.1_29989693	Ca_kabuli_chr05	29989693	[C/A]	1067297781
Ca_Kabuli_NC_021164.1_29989694	Ca_kabuli_chr05	29989694	[C/G]	1067297782
Ca_Kabuli_NC_021164.1_29989696	Ca_kabuli_chr05	29989696	[A/T]	1067297783
Ca_Kabuli_NC_021164.1_29999465	Ca_kabuli_chr05	29999465	[G/T]	1067297784
Ca_Kabuli_NC_021164.1_30035763	Ca_kabuli_chr05	30035763	[A/C]	1067297785
Ca_Kabuli_NC_021164.1_30053486	Ca_kabuli_chr05	30053486	[A/T]	1067297786
Ca_Kabuli_NC_021164.1_30124077	Ca_kabuli_chr05	30124077	[G/C]	1067297787
Ca_Kabuli_NC_021164.1_30124086	Ca_kabuli_chr05	30124086	[C/G]	1067297788
Ca_Kabuli_NC_021164.1_30138508	Ca_kabuli_chr05	30138508	[T/A]	1067297789
Ca_Kabuli_NC_021164.1_30179608	Ca_kabuli_chr05	30179608	[A/C]	1067297790
Ca_Kabuli_NC_021164.1_30191349	Ca_kabuli_chr05	30191349	[T/G]	1067297791
Ca_Kabuli_NC_021164.1_30286879	Ca_kabuli_chr05	30286879	[A/C]	1067297792
Ca_Kabuli_NC_021164.1_30286888	Ca_kabuli_chr05	30286888	[T/C]	1067297793
Ca_Kabuli_NC_021164.1_30286894	Ca_kabuli_chr05	30286894	[T/A]	1067297794
Ca_Kabuli_NC_021164.1_30286913	Ca_kabuli_chr05	30286913	[T/C]	1067297795
Ca_Kabuli_NC_021164.1_30358986	Ca_kabuli_chr05	30358986	[A/G]	1067297796
Ca_Kabuli_NC_021164.1_30359007	Ca_kabuli_chr05	30359007	[T/C]	1067297797
Ca_Kabuli_NC_021164.1_30359016	Ca_kabuli_chr05	30359016	[T/C]	1067297798
Ca_Kabuli_NC_021164.1_30359040	Ca_kabuli_chr05	30359040	[A/C]	1067297799
Ca_Kabuli_NC_021164.1_30359044	Ca_kabuli_chr05	30359044	[A/G]	1067297800
Ca_Kabuli_NC_021164.1_30366765	Ca_kabuli_chr05	30366765	[A/G]	1067297801
Ca_Kabuli_NC_021164.1_30366771	Ca_kabuli_chr05	30366771	[A/T]	1067297802
Ca_Kabuli_NC_021164.1_30366787	Ca_kabuli_chr05	30366787	[A/C]	1067297803
Ca_Kabuli_NC_021164.1_30366796	Ca_kabuli_chr05	30366796	[C/A]	1067297804
Ca_Kabuli_NC_021164.1_30366836	Ca_kabuli_chr05	30366836	[A/G]	1067297805
Ca_Kabuli_NC_021164.1_30367691	Ca_kabuli_chr05	30367691	[A/C]	1067297806
Ca_Kabuli_NC_021164.1_30367710	Ca_kabuli_chr05	30367710	[T/G]	1067297807
Ca_Kabuli_NC_021164.1_30373415	Ca_kabuli_chr05	30373415	[T/C]	1067297808
Ca_Kabuli_NC_021164.1_30373837	Ca_kabuli_chr05	30373837	[A/C]	1067297809
Ca_Kabuli_NC_021164.1_30376781	Ca_kabuli_chr05	30376781	[A/G]	1067297810

SNP IDs	Chromosomes	Physical positions (bp)	SNPs	NCBI SNPdb accession IDs (NCBI_ss#)
Ca_Kabuli_NC_021164.1_30377476	Ca_kabuli_chr05	30377476	[A/G]	1067297811
Ca_Kabuli_NC_021164.1_30378223	Ca_kabuli_chr05	30378223	[T/C]	1067297812
Ca_Kabuli_NC_021164.1_30420884	Ca_kabuli_chr05	30420884	[T/A]	1067297813
Ca_Kabuli_NC_021164.1_30420906	Ca_kabuli_chr05	30420906	[T/C]	1067297814
Ca_Kabuli_NC_021164.1_30420912	Ca_kabuli_chr05	30420912	[A/G]	1067297815
Ca_Kabuli_NC_021164.1_30422331	Ca_kabuli_chr05	30422331	[G/T]	1067297816
Ca_Kabuli_NC_021164.1_30438909	Ca_kabuli_chr05	30438909	[A/C]	1067297817
Ca_Kabuli_NC_021164.1_30438950	Ca_kabuli_chr05	30438950	[T/A]	1067297818
Ca_Kabuli_NC_021164.1_30627756	Ca_kabuli_chr05	30627756	[G/T]	1067297819
Ca_Kabuli_NC_021164.1_30635500	Ca_kabuli_chr05	30635500	[A/C]	1067297820
Ca_Kabuli_NC_021164.1_30635525	Ca_kabuli_chr05	30635525	[T/C]	1067297821
Ca_Kabuli_NC_021164.1_30635537	Ca_kabuli_chr05	30635537	[A/G]	1067297822
Ca_Kabuli_NC_021164.1_30690864	Ca_kabuli_chr05	30690864	[T/C]	1067297823
Ca_Kabuli_NC_021164.1_30745585	Ca_kabuli_chr05	30745585	[T/G]	1067297824
Ca_Kabuli_NC_021164.1_30748790	Ca_kabuli_chr05	30748790	[A/T]	1067297825
Ca_Kabuli_NC_021164.1_30748799	Ca_kabuli_chr05	30748799	[A/T]	1067297826
Ca_Kabuli_NC_021164.1_30811243	Ca_kabuli_chr05	30811243	[G/C]	1067297827
Ca_Kabuli_NC_021164.1_30811244	Ca_kabuli_chr05	30811244	[T/A]	1067297828
Ca_Kabuli_NC_021164.1_30811245	Ca_kabuli_chr05	30811245	[C/T]	1067297829
Ca_Kabuli_NC_021164.1_30816644	Ca_kabuli_chr05	30816644	[T/G]	1067297830
Ca_Kabuli_NC_021164.1_30863959	Ca_kabuli_chr05	30863959	[T/G]	1067297831
Ca_Kabuli_NC_021164.1_30863984	Ca_kabuli_chr05	30863984	[A/C]	1067297832
Ca_Kabuli_NC_021164.1_30890443	Ca_kabuli_chr05	30890443	[A/G]	1067297833
Ca_Kabuli_NC_021164.1_30890446	Ca_kabuli_chr05	30890446	[T/C]	1067297834
Ca_Kabuli_NC_021164.1_30890472	Ca_kabuli_chr05	30890472	[A/T]	1067297835
Ca_Kabuli_NC_021164.1_30894313	Ca_kabuli_chr05	30894313	[A/G]	1067297836
Ca_Kabuli_NC_021164.1_30903096	Ca_kabuli_chr05	30903096	[A/C]	1067297837
Ca_Kabuli_NC_021164.1_30903110	Ca_kabuli_chr05	30903110	[T/G]	1067297838
Ca_Kabuli_NC_021164.1_30903128	Ca_kabuli_chr05	30903128	[A/G]	1067297839
Ca_Kabuli_NC_021164.1_30903134	Ca_kabuli_chr05	30903134	[A/G]	1067297840
Ca_Kabuli_NC_021164.1_30939087	Ca_kabuli_chr05	30939087	[A/G]	1067297841
Ca_Kabuli_NC_021164.1_30939325	Ca_kabuli_chr05	30939325	[G/T]	1067297842
Ca_Kabuli_NC_021164.1_30976615	Ca_kabuli_chr05	30976615	[A/T]	1067297843
Ca_Kabuli_NC_021164.1_30976616	Ca_kabuli_chr05	30976616	[T/C]	1067297844

SNP IDs	Chromosomes	Physical positions (bp)	SNPs	NCBI SNPdb accession IDs (NCBI_ss#)
Ca_Kabuli_NC_021164.1_30976617	Ca_kabuli_chr05	30976617	[G/T]	1067297845
Ca_Kabuli_NC_021164.1_30976732	Ca_kabuli_chr05	30976732	[A/C]	1067297846
Ca_Kabuli_NC_021164.1_30993661	Ca_kabuli_chr05	30993661	[A/T]	1067297847
Ca_Kabuli_NC_021164.1_30993664	Ca_kabuli_chr05	30993664	[A/G]	1067297848
Ca_Kabuli_NC_021164.1_30993670	Ca_kabuli_chr05	30993670	[A/G]	1067297849
Ca_Kabuli_NC_021164.1_30993679	Ca_kabuli_chr05	30993679	[T/A]	1067297850
Ca_Kabuli_NC_021164.1_30993682	Ca_kabuli_chr05	30993682	[G/A]	1067297851
Ca_Kabuli_NC_021164.1_30993711	Ca_kabuli_chr05	30993711	[A/G]	1067297852
Ca_Kabuli_NC_021164.1_31036716	Ca_kabuli_chr05	31036716	[T/A]	1067297853
Ca_Kabuli_NC_021164.1_31036742	Ca_kabuli_chr05	31036742	[A/G]	1067297854
Ca_Kabuli_NC_021164.1_31036760	Ca_kabuli_chr05	31036760	[G/T]	1067297855
Ca_Kabuli_NC_021164.1_31036770	Ca_kabuli_chr05	31036770	[A/T]	1067297856
Ca_Kabuli_NC_021164.1_31057528	Ca_kabuli_chr05	31057528	[T/G]	1067297857
Ca_Kabuli_NC_021164.1_31064311	Ca_kabuli_chr05	31064311	[A/G]	1067297858
Ca_Kabuli_NC_021164.1_31064326	Ca_kabuli_chr05	31064326	[T/G]	1067297859
Ca_Kabuli_NC_021164.1_31082143	Ca_kabuli_chr05	31082143	[T/A]	1067297860
Ca_Kabuli_NC_021164.1_31082144	Ca_kabuli_chr05	31082144	[C/T]	1067297861
Ca_Kabuli_NC_021164.1_31082145	Ca_kabuli_chr05	31082145	[G/C]	1067297862
Ca_Kabuli_NC_021164.1_31082146	Ca_kabuli_chr05	31082146	[G/T]	1067297863
Ca_Kabuli_NC_021164.1_31131830	Ca_kabuli_chr05	31131830	[T/C]	1067297864
Ca_Kabuli_NC_021164.1_31132450	Ca_kabuli_chr05	31132450	[T/A]	1067297865
Ca_Kabuli_NC_021164.1_31138669	Ca_kabuli_chr05	31138669	[C/G]	1067297866
Ca_Kabuli_NC_021164.1_31141664	Ca_kabuli_chr05	31141664	[A/C]	1067297867
Ca_Kabuli_NC_021164.1_31141695	Ca_kabuli_chr05	31141695	[A/C]	1067297868
Ca_Kabuli_NC_021164.1_31165033	Ca_kabuli_chr05	31165033	[A/C]	1067297869
Ca_Kabuli_NC_021164.1_31171529	Ca_kabuli_chr05	31171529	[T/C]	1067297870
Ca_Kabuli_NC_021164.1_31171533	Ca_kabuli_chr05	31171533	[A/C]	1067297871
Ca_Kabuli_NC_021164.1_31176695	Ca_kabuli_chr05	31176695	[T/C]	1067297872
Ca_Kabuli_NC_021164.1_31176697	Ca_kabuli_chr05	31176697	[T/C]	1067297873
Ca_Kabuli_NC_021164.1_31176731	Ca_kabuli_chr05	31176731	[A/G]	1067297874
Ca_Kabuli_NC_021164.1_31176750	Ca_kabuli_chr05	31176750	[T/C]	1067297875
Ca_Kabuli_NC_021164.1_31176817	Ca_kabuli_chr05	31176817	[T/A]	1067297876
Ca_Kabuli_NC_021164.1_31181392	Ca_kabuli_chr05	31181392	[T/G]	1067297877
Ca_Kabuli_NC_021164.1_31192391	Ca_kabuli_chr05	31192391	[A/T]	1067297878

SNP IDs	Chromosomes	Physical positions (bp)	SNPs	NCBI SNPdb accession IDs (NCBI_ss#)
Ca_Kabuli_NC_021164.1_31192429	Ca_kabuli_chr05	31192429	[T/C]	1067297879
Ca_Kabuli_NC_021164.1_31192432	Ca_kabuli_chr05	31192432	[T/C]	1067297880
Ca_Kabuli_NC_021164.1_31215125	Ca_kabuli_chr05	31215125	[A/G]	1067297881
Ca_Kabuli_NC_021164.1_31303773	Ca_kabuli_chr05	31303773	[T/G]	1067297882
Ca_Kabuli_NC_021164.1_31306171	Ca_kabuli_chr05	31306171	[C/A]	1067297883
Ca_Kabuli_NC_021164.1_31306172	Ca_kabuli_chr05	31306172	[A/G]	1067297884
Ca_Kabuli_NC_021164.1_31313858	Ca_kabuli_chr05	31313858	[T/G]	1067297885
Ca_Kabuli_NC_021164.1_31314012	Ca_kabuli_chr05	31314012	[A/C]	1067297886
Ca_Kabuli_NC_021164.1_31330704	Ca_kabuli_chr05	31330704	[T/A]	1067297887
Ca_Kabuli_NC_021164.1_31330710	Ca_kabuli_chr05	31330710	[A/G]	1067297888
Ca_Kabuli_NC_021164.1_31330757	Ca_kabuli_chr05	31330757	[A/G]	1067297889
Ca_Kabuli_NC_021164.1_31371625	Ca_kabuli_chr05	31371625	[C/A]	1067297890
Ca_Kabuli_NC_021164.1_31371626	Ca_kabuli_chr05	31371626	[T/G]	1067297891
Ca_Kabuli_NC_021164.1_31403645	Ca_kabuli_chr05	31403645	[G/A]	1067297892
Ca_Kabuli_NC_021164.1_31403647	Ca_kabuli_chr05	31403647	[G/A]	1067297893
Ca_Kabuli_NC_021164.1_31403655	Ca_kabuli_chr05	31403655	[T/G]	1067297894
Ca_Kabuli_NC_021164.1_31403667	Ca_kabuli_chr05	31403667	[T/G]	1067297895
Ca_Kabuli_NC_021164.1_31521962	Ca_kabuli_chr05	31521962	[T/A]	1067297896
Ca_Kabuli_NC_021164.1_31622168	Ca_kabuli_chr05	31622168	[T/C]	1067297897
Ca_Kabuli_NC_021164.1_31622185	Ca_kabuli_chr05	31622185	[A/G]	1067297898
Ca_Kabuli_NC_021164.1_31683378	Ca_kabuli_chr05	31683378	[A/C]	1067297899
Ca_Kabuli_NC_021164.1_31687858	Ca_kabuli_chr05	31687858	[T/C]	1067297900
Ca_Kabuli_NC_021164.1_31696257	Ca_kabuli_chr05	31696257	[T/G]	1067297901
Ca_Kabuli_NC_021164.1_31708574	Ca_kabuli_chr05	31708574	[T/C]	1067297902
Ca_Kabuli_NC_021164.1_31708618	Ca_kabuli_chr05	31708618	[A/G]	1067297903
Ca_Kabuli_NC_021164.1_31710625	Ca_kabuli_chr05	31710625	[T/G]	1067297904
Ca_Kabuli_NC_021164.1_31756559	Ca_kabuli_chr05	31756559	[T/G]	1067297905
Ca_Kabuli_NC_021164.1_31864516	Ca_kabuli_chr05	31864516	[A/G]	1067297906
Ca_Kabuli_NC_021164.1_31864519	Ca_kabuli_chr05	31864519	[T/C]	1067297907
Ca_Kabuli_NC_021164.1_31864523	Ca_kabuli_chr05	31864523	[A/G]	1067297908
Ca_Kabuli_NC_021164.1_31880926	Ca_kabuli_chr05	31880926	[T/C]	1067297909
Ca_Kabuli_NC_021164.1_31935643	Ca_kabuli_chr05	31935643	[T/C]	1067297910
Ca_Kabuli_NC_021164.1_31935709	Ca_kabuli_chr05	31935709	[A/C]	1067297911
Ca_Kabuli_NC_021164.1_32000346	Ca_kabuli_chr05	32000346	[T/G]	1067297912

SNP IDs	Chromosomes	Physical positions (bp)	SNPs	NCBI SNPdb accession IDs (NCBI_ss#)
Ca_Kabuli_NC_021164.1_32011769	Ca_kabuli_chr05	32011769	[A/G]	1067297913
Ca_Kabuli_NC_021164.1_32011781	Ca_kabuli_chr05	32011781	[T/G]	1067297914
Ca_Kabuli_NC_021164.1_32011792	Ca_kabuli_chr05	32011792	[T/G]	1067297915
Ca_Kabuli_NC_021164.1_32015983	Ca_kabuli_chr05	32015983	[A/T]	1067297916
Ca_Kabuli_NC_021164.1_32067558	Ca_kabuli_chr05	32067558	[C/T]	1067297917
Ca_Kabuli_NC_021164.1_32149964	Ca_kabuli_chr05	32149964	[C/T]	1067297918
Ca_Kabuli_NC_021164.1_32149976	Ca_kabuli_chr05	32149976	[G/A]	1067297919
Ca_Kabuli_NC_021164.1_32149978	Ca_kabuli_chr05	32149978	[T/C]	1067297920
Ca_Kabuli_NC_021164.1_32149981	Ca_kabuli_chr05	32149981	[C/A]	1067297921
Ca_Kabuli_NC_021164.1_32171616	Ca_kabuli_chr05	32171616	[T/G]	1067297922
Ca_Kabuli_NC_021164.1_32171618	Ca_kabuli_chr05	32171618	[T/G]	1067297923
Ca_Kabuli_NC_021164.1_32171632	Ca_kabuli_chr05	32171632	[G/A]	1067297924
Ca_Kabuli_NC_021164.1_32188164	Ca_kabuli_chr05	32188164	[A/T]	1067297925
Ca_Kabuli_NC_021164.1_32188177	Ca_kabuli_chr05	32188177	[A/T]	1067297926
Ca_Kabuli_NC_021164.1_32188182	Ca_kabuli_chr05	32188182	[A/C]	1067297927
Ca_Kabuli_NC_021164.1_32193751	Ca_kabuli_chr05	32193751	[A/G]	1067297928
Ca_Kabuli_NC_021164.1_32204865	Ca_kabuli_chr05	32204865	[A/T]	1067297929
Ca_Kabuli_NC_021164.1_32204898	Ca_kabuli_chr05	32204898	[T/G]	1067297930
Ca_Kabuli_NC_021164.1_32204907	Ca_kabuli_chr05	32204907	[T/C]	1067297931
Ca_Kabuli_NC_021164.1_32229542	Ca_kabuli_chr05	32229542	[A/C]	1067297932
Ca_Kabuli_NC_021164.1_32346662	Ca_kabuli_chr05	32346662	[T/A]	1067297933
Ca_Kabuli_NC_021164.1_32362334	Ca_kabuli_chr05	32362334	[A/G]	1067297934
Ca_Kabuli_NC_021164.1_32362361	Ca_kabuli_chr05	32362361	[A/T]	1067297935
Ca_Kabuli_NC_021164.1_32368469	Ca_kabuli_chr05	32368469	[T/G]	1067297936
Ca_Kabuli_NC_021164.1_32383734	Ca_kabuli_chr05	32383734	[T/G]	1067297937
Ca_Kabuli_NC_021164.1_32383735	Ca_kabuli_chr05	32383735	[T/C]	1067297938
Ca_Kabuli_NC_021164.1_32383737	Ca_kabuli_chr05	32383737	[T/C]	1067297939
Ca_Kabuli_NC_021164.1_32399307	Ca_kabuli_chr05	32399307	[A/T]	1067297940
Ca_Kabuli_NC_021164.1_32459857	Ca_kabuli_chr05	32459857	[A/T]	1067297941
Ca_Kabuli_NC_021164.1_32481257	Ca_kabuli_chr05	32481257	[T/C]	1067297942
Ca_Kabuli_NC_021164.1_32554169	Ca_kabuli_chr05	32554169	[T/G]	1067297943
Ca_Kabuli_NC_021164.1_32598189	Ca_kabuli_chr05	32598189	[A/G]	1067297944
Ca_Kabuli_NC_021164.1_32598217	Ca_kabuli_chr05	32598217	[T/C]	1067297945
Ca_Kabuli_NC_021164.1_32794257	Ca_kabuli_chr05	32794257	[A/C]	1067297946

SNP IDs	Chromosomes	Physical positions (bp)	SNPs	NCBI SNPdb accession IDs (NCBI_ss#)
Ca_Kabuli_NC_021164.1_32794266	Ca_kabuli_chr05	32794266	[T/C]	1067297947
Ca_Kabuli_NC_021164.1_32839329	Ca_kabuli_chr05	32839329	[A/C]	1067297948
Ca_Kabuli_NC_021164.1_32839356	Ca_kabuli_chr05	32839356	[A/G]	1067297949
Ca_Kabuli_NC_021164.1_32840541	Ca_kabuli_chr05	32840541	[T/G]	1067297950
Ca_Kabuli_NC_021164.1_32968793	Ca_kabuli_chr05	32968793	[A/C]	1067297951
Ca_Kabuli_NC_021164.1_32978975	Ca_kabuli_chr05	32978975	[A/C]	1067297952
Ca_Kabuli_NC_021164.1_32998113	Ca_kabuli_chr05	32998113	[G/A]	1067297953
Ca_Kabuli_NC_021164.1_32998127	Ca_kabuli_chr05	32998127	[A/C]	1067297954
Ca_Kabuli_NC_021164.1_32998133	Ca_kabuli_chr05	32998133	[A/G]	1067297955
Ca_Kabuli_NC_021164.1_33107719	Ca_kabuli_chr05	33107719	[T/C]	1067297956
Ca_Kabuli_NC_021164.1_33142987	Ca_kabuli_chr05	33142987	[A/C]	1067297957
Ca_Kabuli_NC_021164.1_33143008	Ca_kabuli_chr05	33143008	[A/T]	1067297958
Ca_Kabuli_NC_021164.1_33255835	Ca_kabuli_chr05	33255835	[A/G]	1067297959
Ca_Kabuli_NC_021164.1_33255848	Ca_kabuli_chr05	33255848	[A/G]	1067297960
Ca_Kabuli_NC_021164.1_33256200	Ca_kabuli_chr05	33256200	[T/A]	1067297961
Ca_Kabuli_NC_021164.1_33256287	Ca_kabuli_chr05	33256287	[T/G]	1067297962
Ca_Kabuli_NC_021164.1_33256367	Ca_kabuli_chr05	33256367	[A/C]	1067297963
Ca_Kabuli_NC_021164.1_33256447	Ca_kabuli_chr05	33256447	[A/G]	1067297964
Ca_Kabuli_NC_021164.1_33256468	Ca_kabuli_chr05	33256468	[T/C]	1067297965
Ca_Kabuli_NC_021164.1_33431968	Ca_kabuli_chr05	33431968	[T/C]	1067297966
Ca_Kabuli_NC_021164.1_33432041	Ca_kabuli_chr05	33432041	[A/C]	1067297967
Ca_Kabuli_NC_021164.1_33578410	Ca_kabuli_chr05	33578410	[T/C]	1067297968
Ca_Kabuli_NC_021164.1_33578415	Ca_kabuli_chr05	33578415	[T/C]	1067297969
Ca_Kabuli_NC_021164.1_33578455	Ca_kabuli_chr05	33578455	[A/G]	1067297970
Ca_Kabuli_NC_021164.1_33600199	Ca_kabuli_chr05	33600199	[A/C]	1067297971
Ca_Kabuli_NC_021164.1_33600232	Ca_kabuli_chr05	33600232	[A/T]	1067297972
Ca_Kabuli_NC_021164.1_33600234	Ca_kabuli_chr05	33600234	[A/C]	1067297973
Ca_Kabuli_NC_021164.1_33600274	Ca_kabuli_chr05	33600274	[C/A]	1067297974
Ca_Kabuli_NC_021164.1_33600774	Ca_kabuli_chr05	33600774	[A/C]	1067297975
Ca_Kabuli_NC_021164.1_33704718	Ca_kabuli_chr05	33704718	[G/T]	1067297976
Ca_Kabuli_NC_021164.1_33752912	Ca_kabuli_chr05	33752912	[A/G]	1067297977
Ca_Kabuli_NC_021164.1_33754233	Ca_kabuli_chr05	33754233	[G/T]	1067297978
Ca_Kabuli_NC_021164.1_33754236	Ca_kabuli_chr05	33754236	[A/G]	1067297979
Ca_Kabuli_NC_021164.1_33754240	Ca_kabuli_chr05	33754240	[A/G]	1067297980

SNP IDs	Chromosomes	Physical positions (bp)	SNPs	NCBI SNPdb accession IDs (NCBI_ss#)
Ca_Kabuli_NC_021164.1_33754245	Ca_kabuli_chr05	33754245	[C/T]	1067297981
Ca_Kabuli_NC_021164.1_33754246	Ca_kabuli_chr05	33754246	[A/C]	1067297982
Ca_Kabuli_NC_021164.1_33754384	Ca_kabuli_chr05	33754384	[T/C]	1067297983
Ca_Kabuli_NC_021164.1_33754427	Ca_kabuli_chr05	33754427	[T/C]	1067297984
Ca_Kabuli_NC_021164.1_33754441	Ca_kabuli_chr05	33754441	[T/C]	1067297985
Ca_Kabuli_NC_021164.1_33812176	Ca_kabuli_chr05	33812176	[A/G]	1067297986
Ca_Kabuli_NC_021164.1_33812187	Ca_kabuli_chr05	33812187	[A/G]	1067297987
Ca_Kabuli_NC_021164.1_33812200	Ca_kabuli_chr05	33812200	[G/A]	1067297988
Ca_Kabuli_NC_021164.1_33937365	Ca_kabuli_chr05	33937365	[A/G]	1067297989
Ca_Kabuli_NC_021164.1_33949406	Ca_kabuli_chr05	33949406	[A/C]	1067297990
Ca_Kabuli_NC_021164.1_33949411	Ca_kabuli_chr05	33949411	[A/G]	1067297991
Ca_Kabuli_NC_021164.1_33949424	Ca_kabuli_chr05	33949424	[A/C]	1067297992
Ca_Kabuli_NC_021164.1_33985810	Ca_kabuli_chr05	33985810	[A/C]	1067297993
Ca_Kabuli_NC_021164.1_33986108	Ca_kabuli_chr05	33986108	[A/C]	1067297994
Ca_Kabuli_NC_021164.1_33986111	Ca_kabuli_chr05	33986111	[A/T]	1067297995
Ca_Kabuli_NC_021164.1_33986115	Ca_kabuli_chr05	33986115	[T/G]	1067297996
Ca_Kabuli_NC_021164.1_33986184	Ca_kabuli_chr05	33986184	[T/A]	1067297997
Ca_Kabuli_NC_021164.1_33986237	Ca_kabuli_chr05	33986237	[A/G]	1067297998
Ca_Kabuli_NC_021164.1_33986240	Ca_kabuli_chr05	33986240	[A/T]	1067297999
Ca_Kabuli_NC_021164.1_33990236	Ca_kabuli_chr05	33990236	[T/G]	1067298000
Ca_Kabuli_NC_021164.1_33990241	Ca_kabuli_chr05	33990241	[A/C]	1067298001
Ca_Kabuli_NC_021164.1_33996555	Ca_kabuli_chr05	33996555	[T/C]	1067298002
Ca_Kabuli_NC_021164.1_33996559	Ca_kabuli_chr05	33996559	[A/G]	1067298003
Ca_Kabuli_NC_021164.1_33996588	Ca_kabuli_chr05	33996588	[A/G]	1067298004
Ca_Kabuli_NC_021164.1_33996606	Ca_kabuli_chr05	33996606	[G/A]	1067298005
Ca_Kabuli_NC_021164.1_34022356	Ca_kabuli_chr05	34022356	[T/G]	1067298006
Ca_Kabuli_NC_021164.1_34041827	Ca_kabuli_chr05	34041827	[A/C]	1067298007
Ca_Kabuli_NC_021164.1_34041947	Ca_kabuli_chr05	34041947	[A/C]	1067298008
Ca_Kabuli_NC_021164.1_34073361	Ca_kabuli_chr05	34073361	[A/C]	1067298009
Ca_Kabuli_NC_021164.1_34108161	Ca_kabuli_chr05	34108161	[T/C]	1067298010
Ca_Kabuli_NC_021164.1_34126909	Ca_kabuli_chr05	34126909	[A/C]	1067298011
Ca_Kabuli_NC_021164.1_34126915	Ca_kabuli_chr05	34126915	[A/C]	1067298012
Ca_Kabuli_NC_021164.1_34189654	Ca_kabuli_chr05	34189654	[A/C]	1067298013
Ca_Kabuli_NC_021164.1_34189660	Ca_kabuli_chr05	34189660	[A/G]	1067298014

SNP IDs	Chromosomes	Physical positions (bp)	SNPs	NCBI SNPdb accession IDs (NCBI_ss#)
Ca_Kabuli_NC_021164.1_34201939	Ca_kabuli_chr05	34201939	[A/G]	1067298015
Ca_Kabuli_NC_021164.1_34201947	Ca_kabuli_chr05	34201947	[T/G]	1067298016
Ca_Kabuli_NC_021164.1_34202000	Ca_kabuli_chr05	34202000	[T/C]	1067298017
Ca_Kabuli_NC_021164.1_34240610	Ca_kabuli_chr05	34240610	[A/C]	1067298018
Ca_Kabuli_NC_021164.1_34241341	Ca_kabuli_chr05	34241341	[A/C]	1067298019
Ca_Kabuli_NC_021164.1_34243816	Ca_kabuli_chr05	34243816	[A/C]	1067298020
Ca_Kabuli_NC_021164.1_34243842	Ca_kabuli_chr05	34243842	[A/G]	1067298021
Ca_Kabuli_NC_021164.1_34243881	Ca_kabuli_chr05	34243881	[T/G]	1067298022
Ca_Kabuli_NC_021164.1_34244774	Ca_kabuli_chr05	34244774	[T/C]	1067298023
Ca_Kabuli_NC_021164.1_34244775	Ca_kabuli_chr05	34244775	[G/T]	1067298024
Ca_Kabuli_NC_021164.1_34246019	Ca_kabuli_chr05	34246019	[T/A]	1067298025
Ca_Kabuli_NC_021164.1_34246020	Ca_kabuli_chr05	34246020	[C/A]	1067298026
Ca_Kabuli_NC_021164.1_34246021	Ca_kabuli_chr05	34246021	[T/G]	1067298027
Ca_Kabuli_NC_021164.1_34279544	Ca_kabuli_chr05	34279544	[A/G]	1067298028
Ca_Kabuli_NC_021164.1_34289094	Ca_kabuli_chr05	34289094	[T/G]	1067298029
Ca_Kabuli_NC_021164.1_34413128	Ca_kabuli_chr05	34413128	[A/G]	1067298030
Ca_Kabuli_NC_021164.1_34413135	Ca_kabuli_chr05	34413135	[A/C]	1067298031
Ca_Kabuli_NC_021164.1_34438983	Ca_kabuli_chr05	34438983	[T/G]	1067298032
Ca_Kabuli_NC_021164.1_34441597	Ca_kabuli_chr05	34441597	[T/C]	1067298033
Ca_Kabuli_NC_021164.1_34486754	Ca_kabuli_chr05	34486754	[T/C]	1067298034
Ca_Kabuli_NC_021164.1_34510722	Ca_kabuli_chr05	34510722	[A/G]	1067298035
Ca_Kabuli_NC_021164.1_34511338	Ca_kabuli_chr05	34511338	[T/G]	1067298036
Ca_Kabuli_NC_021164.1_34511353	Ca_kabuli_chr05	34511353	[A/C]	1067298037
Ca_Kabuli_NC_021164.1_34511645	Ca_kabuli_chr05	34511645	[T/C]	1067298038
Ca_Kabuli_NC_021164.1_34511657	Ca_kabuli_chr05	34511657	[T/C]	1067298039
Ca_Kabuli_NC_021164.1_34511691	Ca_kabuli_chr05	34511691	[T/A]	1067298040
Ca_Kabuli_NC_021164.1_34521694	Ca_kabuli_chr05	34521694	[A/G]	1067298041
Ca_Kabuli_NC_021164.1_34527336	Ca_kabuli_chr05	34527336	[A/G]	1067298042
Ca_Kabuli_NC_021164.1_34527366	Ca_kabuli_chr05	34527366	[T/G]	1067298043
Ca_Kabuli_NC_021164.1_34530237	Ca_kabuli_chr05	34530237	[T/G]	1067298044
Ca_Kabuli_NC_021164.1_34530367	Ca_kabuli_chr05	34530367	[C/A]	1067298045
Ca_Kabuli_NC_021164.1_34531127	Ca_kabuli_chr05	34531127	[A/T]	1067298046
Ca_Kabuli_NC_021164.1_34531150	Ca_kabuli_chr05	34531150	[A/G]	1067298047
Ca_Kabuli_NC_021164.1_34572312	Ca_kabuli_chr05	34572312	[A/G]	1067298048

SNP IDs	Chromosomes	Physical positions (bp)	SNPs	NCBI SNPdb accession IDs (NCBI_ss#)
Ca_Kabuli_NC_021164.1_34578038	Ca_kabuli_chr05	34578038	[T/G]	1067298049
Ca_Kabuli_NC_021164.1_34578053	Ca_kabuli_chr05	34578053	[A/C]	1067298050
Ca_Kabuli_NC_021164.1_34578057	Ca_kabuli_chr05	34578057	[T/C]	1067298051
Ca_Kabuli_NC_021164.1_34578635	Ca_kabuli_chr05	34578635	[T/A]	1067298052
Ca_Kabuli_NC_021164.1_34629032	Ca_kabuli_chr05	34629032	[T/G]	1067298053
Ca_Kabuli_NC_021164.1_34688480	Ca_kabuli_chr05	34688480	[T/G]	1067298054
Ca_Kabuli_NC_021164.1_34688489	Ca_kabuli_chr05	34688489	[A/C]	1067298055
Ca_Kabuli_NC_021164.1_34691434	Ca_kabuli_chr05	34691434	[T/C]	1067298056
Ca_Kabuli_NC_021164.1_34699658	Ca_kabuli_chr05	34699658	[A/C]	1067298057
Ca_Kabuli_NC_021164.1_34732777	Ca_kabuli_chr05	34732777	[A/C]	1067298058
Ca_Kabuli_NC_021164.1_34806999	Ca_kabuli_chr05	34806999	[A/G]	1067298059
Ca_Kabuli_NC_021164.1_34845399	Ca_kabuli_chr05	34845399	[G/T]	1067298060
Ca_Kabuli_NC_021164.1_34845467	Ca_kabuli_chr05	34845467	[A/G]	1067298061
Ca_Kabuli_NC_021164.1_34845479	Ca_kabuli_chr05	34845479	[T/G]	1067298062
Ca_Kabuli_NC_021164.1_34845512	Ca_kabuli_chr05	34845512	[A/C]	1067298063
Ca_Kabuli_NC_021164.1_34845521	Ca_kabuli_chr05	34845521	[A/C]	1067298064
Ca_Kabuli_NC_021164.1_34845530	Ca_kabuli_chr05	34845530	[A/G]	1067298065
Ca_Kabuli_NC_021164.1_34857308	Ca_kabuli_chr05	34857308	[G/T]	1067298066
Ca_Kabuli_NC_021164.1_34882224	Ca_kabuli_chr05	34882224	[T/G]	1067298067
Ca_Kabuli_NC_021164.1_34882230	Ca_kabuli_chr05	34882230	[T/G]	1067298068
Ca_Kabuli_NC_021164.1_34932304	Ca_kabuli_chr05	34932304	[A/G]	1067298069
Ca_Kabuli_NC_021164.1_34975470	Ca_kabuli_chr05	34975470	[T/C]	1067298070
Ca_Kabuli_NC_021164.1_34989692	Ca_kabuli_chr05	34989692	[A/G]	1067298071
Ca_Kabuli_NC_021164.1_35126956	Ca_kabuli_chr05	35126956	[T/A]	1067298072
Ca_Kabuli_NC_021164.1_35126957	Ca_kabuli_chr05	35126957	[A/T]	1067298073
Ca_Kabuli_NC_021164.1_35126977	Ca_kabuli_chr05	35126977	[A/C]	1067298074
Ca_Kabuli_NC_021164.1_35209505	Ca_kabuli_chr05	35209505	[T/G]	1067298075
Ca_Kabuli_NC_021164.1_35226127	Ca_kabuli_chr05	35226127	[A/C]	1067298076
Ca_Kabuli_NC_021164.1_35226132	Ca_kabuli_chr05	35226132	[A/C]	1067298077
Ca_Kabuli_NC_021164.1_35240014	Ca_kabuli_chr05	35240014	[A/C]	1067298078
Ca_Kabuli_NC_021164.1_35296146	Ca_kabuli_chr05	35296146	[T/C]	1067298079
Ca_Kabuli_NC_021164.1_35317721	Ca_kabuli_chr05	35317721	[A/C]	1067298080
Ca_Kabuli_NC_021164.1_35340062	Ca_kabuli_chr05	35340062	[A/G]	1067298081
Ca_Kabuli_NC_021164.1_35431496	Ca_kabuli_chr05	35431496	[A/C]	1067298082

SNP IDs	Chromosomes	Physical positions (bp)	SNPs	NCBI SNPdb accession IDs (NCBI_ss#)
Ca_Kabuli_NC_021164.1_35475543	Ca_kabuli_chr05	35475543	[A/G]	1067298083
Ca_Kabuli_NC_021164.1_35525244	Ca_kabuli_chr05	35525244	[T/G]	1067298084
Ca_Kabuli_NC_021164.1_35536233	Ca_kabuli_chr05	35536233	[A/G]	1067298085
Ca_Kabuli_NC_021164.1_35540350	Ca_kabuli_chr05	35540350	[A/G]	1067298086
Ca_Kabuli_NC_021164.1_35554377	Ca_kabuli_chr05	35554377	[T/A]	1067298087
Ca_Kabuli_NC_021164.1_35688754	Ca_kabuli_chr05	35688754	[T/G]	1067298088
Ca_Kabuli_NC_021164.1_35715560	Ca_kabuli_chr05	35715560	[A/G]	1067298089
Ca_Kabuli_NC_021164.1_35715571	Ca_kabuli_chr05	35715571	[A/C]	1067298090
Ca_Kabuli_NC_021164.1_35773032	Ca_kabuli_chr05	35773032	[T/G]	1067298091
Ca_Kabuli_NC_021164.1_35773034	Ca_kabuli_chr05	35773034	[A/T]	1067298092
Ca_Kabuli_NC_021164.1_35773044	Ca_kabuli_chr05	35773044	[A/G]	1067298093
Ca_Kabuli_NC_021164.1_35773046	Ca_kabuli_chr05	35773046	[A/G]	1067298094
Ca_Kabuli_NC_021164.1_35789265	Ca_kabuli_chr05	35789265	[T/C]	1067298095
Ca_Kabuli_NC_021164.1_35845092	Ca_kabuli_chr05	35845092	[A/G]	1067298096
Ca_Kabuli_NC_021164.1_35845097	Ca_kabuli_chr05	35845097	[G/T]	1067298097
Ca_Kabuli_NC_021164.1_35845099	Ca_kabuli_chr05	35845099	[C/T]	1067298098
Ca_Kabuli_NC_021164.1_35846367	Ca_kabuli_chr05	35846367	[A/C]	1067298099
Ca_Kabuli_NC_021164.1_35868836	Ca_kabuli_chr05	35868836	[A/G]	1067298100
Ca_Kabuli_NC_021164.1_35868859	Ca_kabuli_chr05	35868859	[A/G]	1067298101
Ca_Kabuli_NC_021164.1_35877446	Ca_kabuli_chr05	35877446	[T/A]	1067298102
Ca_Kabuli_NC_021164.1_35877448	Ca_kabuli_chr05	35877448	[A/T]	1067298103
Ca_Kabuli_NC_021164.1_35877479	Ca_kabuli_chr05	35877479	[T/A]	1067298104
Ca_Kabuli_NC_021164.1_35877482	Ca_kabuli_chr05	35877482	[A/G]	1067298105
Ca_Kabuli_NC_021164.1_35877502	Ca_kabuli_chr05	35877502	[A/G]	1067298106
Ca_Kabuli_NC_021164.1_35889383	Ca_kabuli_chr05	35889383	[T/C]	1067298107
Ca_Kabuli_NC_021164.1_35937861	Ca_kabuli_chr05	35937861	[C/A]	1067298108
Ca_Kabuli_NC_021164.1_35937882	Ca_kabuli_chr05	35937882	[A/G]	1067298109
Ca_Kabuli_NC_021164.1_35937883	Ca_kabuli_chr05	35937883	[T/A]	1067298110
Ca_Kabuli_NC_021164.1_35937886	Ca_kabuli_chr05	35937886	[A/G]	1067298111
Ca_Kabuli_NC_021164.1_35951837	Ca_kabuli_chr05	35951837	[A/G]	1067298112
Ca_Kabuli_NC_021164.1_35951855	Ca_kabuli_chr05	35951855	[A/G]	1067298113
Ca_Kabuli_NC_021164.1_35951893	Ca_kabuli_chr05	35951893	[A/G]	1067298114
Ca_Kabuli_NC_021164.1_35956521	Ca_kabuli_chr05	35956521	[T/G]	1067298115
Ca_Kabuli_NC_021164.1_36012677	Ca_kabuli_chr05	36012677	[T/C]	1067298116

SNP IDs	Chromosomes	Physical positions (bp)	SNPs	NCBI SNPdb accession IDs (NCBI_ss#)
Ca_Kabuli_NC_021164.1_36039387	Ca_kabuli_chr05	36039387	[A/C]	1067298117
Ca_Kabuli_NC_021164.1_36039430	Ca_kabuli_chr05	36039430	[T/C]	1067298118
Ca_Kabuli_NC_021164.1_36201080	Ca_kabuli_chr05	36201080	[G/A]	1067298119
Ca_Kabuli_NC_021164.1_36201823	Ca_kabuli_chr05	36201823	[C/A]	1067298120
Ca_Kabuli_NC_021164.1_36201842	Ca_kabuli_chr05	36201842	[A/G]	1067298121
Ca_Kabuli_NC_021164.1_36217832	Ca_kabuli_chr05	36217832	[T/C]	1067298122
Ca_Kabuli_NC_021164.1_36217838	Ca_kabuli_chr05	36217838	[G/C]	1067298123
Ca_Kabuli_NC_021164.1_36225904	Ca_kabuli_chr05	36225904	[T/C]	1067298124
Ca_Kabuli_NC_021164.1_36292584	Ca_kabuli_chr05	36292584	[T/G]	1067298125
Ca_Kabuli_NC_021164.1_36295956	Ca_kabuli_chr05	36295956	[T/A]	1067298126
Ca_Kabuli_NC_021164.1_36397258	Ca_kabuli_chr05	36397258	[T/G]	1067298127
Ca_Kabuli_NC_021164.1_36407458	Ca_kabuli_chr05	36407458	[A/G]	1067298128
Ca_Kabuli_NC_021164.1_36407481	Ca_kabuli_chr05	36407481	[A/C]	1067298129
Ca_Kabuli_NC_021164.1_36408734	Ca_kabuli_chr05	36408734	[A/C]	1067298130
Ca_Kabuli_NC_021164.1_36408745	Ca_kabuli_chr05	36408745	[A/C]	1067298131
Ca_Kabuli_NC_021164.1_36427186	Ca_kabuli_chr05	36427186	[A/G]	1067298132
Ca_Kabuli_NC_021164.1_36430731	Ca_kabuli_chr05	36430731	[A/C]	1067298133
Ca_Kabuli_NC_021164.1_36440951	Ca_kabuli_chr05	36440951	[A/C]	1067298134
Ca_Kabuli_NC_021164.1_36440954	Ca_kabuli_chr05	36440954	[A/C]	1067298135
Ca_Kabuli_NC_021164.1_36440984	Ca_kabuli_chr05	36440984	[A/C]	1067298136
Ca_Kabuli_NC_021164.1_36441067	Ca_kabuli_chr05	36441067	[A/C]	1067298137
Ca_Kabuli_NC_021164.1_36441113	Ca_kabuli_chr05	36441113	[A/G]	1067298138
Ca_Kabuli_NC_021164.1_36566265	Ca_kabuli_chr05	36566265	[A/C]	1067298139
Ca_Kabuli_NC_021164.1_36613606	Ca_kabuli_chr05	36613606	[T/G]	1067298140
Ca_Kabuli_NC_021164.1_36613688	Ca_kabuli_chr05	36613688	[G/A]	1067298141
Ca_Kabuli_NC_021164.1_36619876	Ca_kabuli_chr05	36619876	[A/G]	1067298142
Ca_Kabuli_NC_021164.1_36671454	Ca_kabuli_chr05	36671454	[T/G]	1067298143
Ca_Kabuli_NC_021164.1_36671524	Ca_kabuli_chr05	36671524	[C/G]	1067298144
Ca_Kabuli_NC_021164.1_36672025	Ca_kabuli_chr05	36672025	[T/G]	1067298145
Ca_Kabuli_NC_021164.1_36673942	Ca_kabuli_chr05	36673942	[T/G]	1067298146
Ca_Kabuli_NC_021164.1_36751851	Ca_kabuli_chr05	36751851	[A/T]	1067298147
Ca_Kabuli_NC_021164.1_36757372	Ca_kabuli_chr05	36757372	[T/C]	1067298148
Ca_Kabuli_NC_021164.1_36757432	Ca_kabuli_chr05	36757432	[A/G]	1067298149
Ca_Kabuli_NC_021164.1_36757434	Ca_kabuli_chr05	36757434	[T/C]	1067298150

SNP IDs	Chromosomes	Physical positions (bp)	SNPs	NCBI SNPdb accession IDs (NCBI_ss#)
Ca_Kabuli_NC_021164.1_36793966	Ca_kabuli_chr05	36793966	[T/C]	1067298151
Ca_Kabuli_NC_021164.1_36793979	Ca_kabuli_chr05	36793979	[A/T]	1067298152
Ca_Kabuli_NC_021164.1_36799793	Ca_kabuli_chr05	36799793	[A/C]	1067298153
Ca_Kabuli_NC_021164.1_36799795	Ca_kabuli_chr05	36799795	[C/G]	1067298154
Ca_Kabuli_NC_021164.1_36799801	Ca_kabuli_chr05	36799801	[A/T]	1067298155
Ca_Kabuli_NC_021164.1_36799839	Ca_kabuli_chr05	36799839	[A/G]	1067298156
Ca_Kabuli_NC_021164.1_36800094	Ca_kabuli_chr05	36800094	[T/C]	1067298157
Ca_Kabuli_NC_021164.1_36800095	Ca_kabuli_chr05	36800095	[A/C]	1067298158
Ca_Kabuli_NC_021164.1_36802444	Ca_kabuli_chr05	36802444	[T/C]	1067298159
Ca_Kabuli_NC_021164.1_36802465	Ca_kabuli_chr05	36802465	[A/G]	1067298160
Ca_Kabuli_NC_021164.1_36908355	Ca_kabuli_chr05	36908355	[T/G]	1067298161
Ca_Kabuli_NC_021164.1_36908363	Ca_kabuli_chr05	36908363	[A/G]	1067298162
Ca_Kabuli_NC_021164.1_36978251	Ca_kabuli_chr05	36978251	[A/G]	1067298163
Ca_Kabuli_NC_021164.1_36978252	Ca_kabuli_chr05	36978252	[G/A]	1067298164
Ca_Kabuli_NC_021164.1_36990631	Ca_kabuli_chr05	36990631	[T/A]	1067298165
Ca_Kabuli_NC_021164.1_36999074	Ca_kabuli_chr05	36999074	[G/C]	1067298166
Ca_Kabuli_NC_021164.1_37004113	Ca_kabuli_chr05	37004113	[T/G]	1067298167
Ca_Kabuli_NC_021164.1_37012135	Ca_kabuli_chr05	37012135	[T/C]	1067298168
Ca_Kabuli_NC_021164.1_37012147	Ca_kabuli_chr05	37012147	[T/C]	1067298169
Ca_Kabuli_NC_021164.1_37012168	Ca_kabuli_chr05	37012168	[C/T]	1067298170
Ca_Kabuli_NC_021164.1_37012371	Ca_kabuli_chr05	37012371	[A/T]	1067298171
Ca_Kabuli_NC_021164.1_37016833	Ca_kabuli_chr05	37016833	[C/T]	1067298172
Ca_Kabuli_NC_021164.1_37039246	Ca_kabuli_chr05	37039246	[A/T]	1067298173
Ca_Kabuli_NC_021164.1_37039531	Ca_kabuli_chr05	37039531	[A/C]	1067298174
Ca_Kabuli_NC_021164.1_37040045	Ca_kabuli_chr05	37040045	[G/A]	1067298175
Ca_Kabuli_NC_021164.1_37042470	Ca_kabuli_chr05	37042470	[C/T]	1067298176
Ca_Kabuli_NC_021164.1_37067293	Ca_kabuli_chr05	37067293	[A/T]	1067298177
Ca_Kabuli_NC_021164.1_37082797	Ca_kabuli_chr05	37082797	[G/T]	1067298178
Ca_Kabuli_NC_021164.1_37083275	Ca_kabuli_chr05	37083275	[G/T]	1067298179
Ca_Kabuli_NC_021164.1_37083276	Ca_kabuli_chr05	37083276	[T/G]	1067298180
Ca_Kabuli_NC_021164.1_37113106	Ca_kabuli_chr05	37113106	[T/C]	1067298181
Ca_Kabuli_NC_021164.1_37113154	Ca_kabuli_chr05	37113154	[A/T]	1067298182
Ca_Kabuli_NC_021164.1_37147247	Ca_kabuli_chr05	37147247	[T/G]	1067298183
Ca_Kabuli_NC_021164.1_37147250	Ca_kabuli_chr05	37147250	[T/C]	1067298184

SNP IDs	Chromosomes	Physical positions (bp)	SNPs	NCBI SNPdb accession IDs (NCBI_ss#)
Ca_Kabuli_NC_021164.1_37147300	Ca_kabuli_chr05	37147300	[A/C]	1067298185
Ca_Kabuli_NC_021164.1_37147373	Ca_kabuli_chr05	37147373	[A/C]	1067298186
Ca_Kabuli_NC_021164.1_37147439	Ca_kabuli_chr05	37147439	[T/G]	1067298187
Ca_Kabuli_NC_021164.1_37147447	Ca_kabuli_chr05	37147447	[C/A]	1067298188
Ca_Kabuli_NC_021164.1_37147486	Ca_kabuli_chr05	37147486	[A/C]	1067298189
Ca_Kabuli_NC_021164.1_37147616	Ca_kabuli_chr05	37147616	[A/G]	1067298190
Ca_Kabuli_NC_021164.1_37147631	Ca_kabuli_chr05	37147631	[T/G]	1067298191
Ca_Kabuli_NC_021164.1_37147697	Ca_kabuli_chr05	37147697	[T/G]	1067298192
Ca_Kabuli_NC_021164.1_37167100	Ca_kabuli_chr05	37167100	[A/C]	1067298193
Ca_Kabuli_NC_021164.1_37172184	Ca_kabuli_chr05	37172184	[T/A]	1067298194
Ca_Kabuli_NC_021164.1_37172188	Ca_kabuli_chr05	37172188	[T/G]	1067298195
Ca_Kabuli_NC_021164.1_37172189	Ca_kabuli_chr05	37172189	[T/C]	1067298196
Ca_Kabuli_NC_021164.1_37174786	Ca_kabuli_chr05	37174786	[T/C]	1067298197
Ca_Kabuli_NC_021164.1_37176168	Ca_kabuli_chr05	37176168	[T/G]	1067298198
Ca_Kabuli_NC_021164.1_37176215	Ca_kabuli_chr05	37176215	[A/G]	1067298199
Ca_Kabuli_NC_021164.1_37197258	Ca_kabuli_chr05	37197258	[A/G]	1067298200
Ca_Kabuli_NC_021164.1_37197281	Ca_kabuli_chr05	37197281	[A/C]	1067298201
Ca_Kabuli_NC_021164.1_37207839	Ca_kabuli_chr05	37207839	[T/G]	1067298202
Ca_Kabuli_NC_021164.1_37207841	Ca_kabuli_chr05	37207841	[T/C]	1067298203
Ca_Kabuli_NC_021164.1_37207850	Ca_kabuli_chr05	37207850	[C/G]	1067298204
Ca_Kabuli_NC_021164.1_37207855	Ca_kabuli_chr05	37207855	[G/T]	1067298205
Ca_Kabuli_NC_021164.1_37207856	Ca_kabuli_chr05	37207856	[T/G]	1067298206
Ca_Kabuli_NC_021164.1_37207857	Ca_kabuli_chr05	37207857	[T/C]	1067298207
Ca_Kabuli_NC_021164.1_37207864	Ca_kabuli_chr05	37207864	[A/C]	1067298208
Ca_Kabuli_NC_021164.1_37207892	Ca_kabuli_chr05	37207892	[A/T]	1067298209
Ca_Kabuli_NC_021164.1_37274550	Ca_kabuli_chr05	37274550	[T/C]	1067298210
Ca_Kabuli_NC_021164.1_37274583	Ca_kabuli_chr05	37274583	[C/T]	1067298211
Ca_Kabuli_NC_021164.1_37310070	Ca_kabuli_chr05	37310070	[T/C]	1067298212
Ca_Kabuli_NC_021164.1_37332182	Ca_kabuli_chr05	37332182	[G/A]	1067298213
Ca_Kabuli_NC_021164.1_37332467	Ca_kabuli_chr05	37332467	[T/C]	1067298214
Ca_Kabuli_NC_021164.1_37363511	Ca_kabuli_chr05	37363511	[T/C]	1067298215
Ca_Kabuli_NC_021164.1_37462538	Ca_kabuli_chr05	37462538	[T/C]	1067298216
Ca_Kabuli_NC_021164.1_37462550	Ca_kabuli_chr05	37462550	[T/C]	1067298217
Ca_Kabuli_NC_021164.1_37579009	Ca_kabuli_chr05	37579009	[T/G]	1067298218

SNP IDs	Chromosomes	Physical positions (bp)	SNPs	NCBI SNPdb accession IDs (NCBI_ss#)
Ca_Kabuli_NC_021164.1_37594911	Ca_kabuli_chr05	37594911	[A/T]	1067298219
Ca_Kabuli_NC_021164.1_37834567	Ca_kabuli_chr05	37834567	[A/G]	1067298220
Ca_Kabuli_NC_021164.1_37869762	Ca_kabuli_chr05	37869762	[A/G]	1067298221
Ca_Kabuli_NC_021164.1_37886452	Ca_kabuli_chr05	37886452	[A/C]	1067298222
Ca_Kabuli_NC_021164.1_37911668	Ca_kabuli_chr05	37911668	[A/C]	1067298223
Ca_Kabuli_NC_021164.1_37911750	Ca_kabuli_chr05	37911750	[T/G]	1067298224
Ca_Kabuli_NC_021164.1_37911759	Ca_kabuli_chr05	37911759	[T/C]	1067298225
Ca_Kabuli_NC_021164.1_37911765	Ca_kabuli_chr05	37911765	[A/G]	1067298226
Ca_Kabuli_NC_021164.1_37911827	Ca_kabuli_chr05	37911827	[A/G]	1067298227
Ca_Kabuli_NC_021164.1_37911839	Ca_kabuli_chr05	37911839	[T/G]	1067298228
Ca_Kabuli_NC_021164.1_37922171	Ca_kabuli_chr05	37922171	[A/C]	1067298229
Ca_Kabuli_NC_021164.1_37980181	Ca_kabuli_chr05	37980181	[T/A]	1067298230
Ca_Kabuli_NC_021164.1_37980227	Ca_kabuli_chr05	37980227	[A/C]	1067298231
Ca_Kabuli_NC_021164.1_38057581	Ca_kabuli_chr05	38057581	[A/G]	1067298232
Ca_Kabuli_NC_021164.1_38080355	Ca_kabuli_chr05	38080355	[A/G]	1067298233
Ca_Kabuli_NC_021164.1_38110217	Ca_kabuli_chr05	38110217	[A/T]	1067298234
Ca_Kabuli_NC_021164.1_38113821	Ca_kabuli_chr05	38113821	[T/A]	1067298235
Ca_Kabuli_NC_021164.1_38180943	Ca_kabuli_chr05	38180943	[T/G]	1067298236
Ca_Kabuli_NC_021164.1_38180987	Ca_kabuli_chr05	38180987	[A/C]	1067298237
Ca_Kabuli_NC_021164.1_38180999	Ca_kabuli_chr05	38180999	[A/C]	1067298238
Ca_Kabuli_NC_021164.1_38182372	Ca_kabuli_chr05	38182372	[T/A]	1067298239
Ca_Kabuli_NC_021164.1_38248865	Ca_kabuli_chr05	38248865	[A/T]	1067298240
Ca_Kabuli_NC_021164.1_38248881	Ca_kabuli_chr05	38248881	[T/A]	1067298241
Ca_Kabuli_NC_021164.1_38248889	Ca_kabuli_chr05	38248889	[T/C]	1067298242
Ca_Kabuli_NC_021164.1_38288096	Ca_kabuli_chr05	38288096	[A/G]	1067298243
Ca_Kabuli_NC_021164.1_38288136	Ca_kabuli_chr05	38288136	[A/G]	1067298244
Ca_Kabuli_NC_021164.1_38288156	Ca_kabuli_chr05	38288156	[T/A]	1067298245
Ca_Kabuli_NC_021164.1_38307037	Ca_kabuli_chr05	38307037	[T/C]	1067298246
Ca_Kabuli_NC_021164.1_38307090	Ca_kabuli_chr05	38307090	[A/C]	1067298247
Ca_Kabuli_NC_021164.1_38307107	Ca_kabuli_chr05	38307107	[A/T]	1067298248
Ca_Kabuli_NC_021164.1_38335108	Ca_kabuli_chr05	38335108	[A/G]	1067298249
Ca_Kabuli_NC_021164.1_38347184	Ca_kabuli_chr05	38347184	[T/G]	1067298250
Ca_Kabuli_NC_021164.1_38362659	Ca_kabuli_chr05	38362659	[T/G]	1067298251
Ca_Kabuli_NC_021164.1_38362774	Ca_kabuli_chr05	38362774	[A/C]	1067298252

SNP IDs	Chromosomes	Physical positions (bp)	SNPs	NCBI SNPdb accession IDs (NCBI_ss#)
Ca_Kabuli_NC_021164.1_38367973	Ca_kabuli_chr05	38367973	[A/G]	1067298253
Ca_Kabuli_NC_021164.1_38453278	Ca_kabuli_chr05	38453278	[G/T]	1067298254
Ca_Kabuli_NC_021164.1_38458823	Ca_kabuli_chr05	38458823	[A/T]	1067298255
Ca_Kabuli_NC_021164.1_38483382	Ca_kabuli_chr05	38483382	[G/T]	1067298256
Ca_Kabuli_NC_021164.1_38483386	Ca_kabuli_chr05	38483386	[T/G]	1067298257
Ca_Kabuli_NC_021164.1_38483396	Ca_kabuli_chr05	38483396	[G/T]	1067298258
Ca_Kabuli_NC_021164.1_38509657	Ca_kabuli_chr05	38509657	[A/C]	1067298259
Ca_Kabuli_NC_021164.1_38538823	Ca_kabuli_chr05	38538823	[T/G]	1067298260
Ca_Kabuli_NC_021164.1_38540373	Ca_kabuli_chr05	38540373	[T/G]	1067298261
Ca_Kabuli_NC_021164.1_38540400	Ca_kabuli_chr05	38540400	[T/C]	1067298262
Ca_Kabuli_NC_021164.1_38551427	Ca_kabuli_chr05	38551427	[A/G]	1067298263
Ca_Kabuli_NC_021164.1_38558828	Ca_kabuli_chr05	38558828	[T/C]	1067298264
Ca_Kabuli_NC_021164.1_38559028	Ca_kabuli_chr05	38559028	[A/C]	1067298265
Ca_Kabuli_NC_021164.1_38559048	Ca_kabuli_chr05	38559048	[A/T]	1067298266
Ca_Kabuli_NC_021164.1_38559049	Ca_kabuli_chr05	38559049	[T/C]	1067298267
Ca_Kabuli_NC_021164.1_38559051	Ca_kabuli_chr05	38559051	[T/C]	1067298268
Ca_Kabuli_NC_021164.1_38559053	Ca_kabuli_chr05	38559053	[A/G]	1067298269
Ca_Kabuli_NC_021164.1_38559084	Ca_kabuli_chr05	38559084	[T/C]	1067298270
Ca_Kabuli_NC_021164.1_38559088	Ca_kabuli_chr05	38559088	[T/C]	1067298271
Ca_Kabuli_NC_021164.1_38559110	Ca_kabuli_chr05	38559110	[T/C]	1067298272
Ca_Kabuli_NC_021164.1_38559115	Ca_kabuli_chr05	38559115	[T/C]	1067298273
Ca_Kabuli_NC_021164.1_38590941	Ca_kabuli_chr05	38590941	[C/A]	1067298274
Ca_Kabuli_NC_021164.1_38591095	Ca_kabuli_chr05	38591095	[T/C]	1067298275
Ca_Kabuli_NC_021164.1_38737288	Ca_kabuli_chr05	38737288	[T/G]	1067298276
Ca_Kabuli_NC_021164.1_38783604	Ca_kabuli_chr05	38783604	[C/A]	1067298277
Ca_Kabuli_NC_021164.1_38783651	Ca_kabuli_chr05	38783651	[A/G]	1067298278
Ca_Kabuli_NC_021164.1_38783858	Ca_kabuli_chr05	38783858	[A/C]	1067298279
Ca_Kabuli_NC_021164.1_38783952	Ca_kabuli_chr05	38783952	[T/A]	1067298280
Ca_Kabuli_NC_021164.1_38783964	Ca_kabuli_chr05	38783964	[A/C]	1067298281
Ca_Kabuli_NC_021164.1_38783990	Ca_kabuli_chr05	38783990	[A/G]	1067298282
Ca_Kabuli_NC_021164.1_38784144	Ca_kabuli_chr05	38784144	[T/G]	1067298283
Ca_Kabuli_NC_021164.1_38820117	Ca_kabuli_chr05	38820117	[A/T]	1067298284
Ca_Kabuli_NC_021164.1_38820152	Ca_kabuli_chr05	38820152	[T/G]	1067298285
Ca_Kabuli_NC_021164.1_38857770	Ca_kabuli_chr05	38857770	[G/A]	1067298286

SNP IDs	Chromosomes	Physical positions (bp)	SNPs	NCBI SNPdb accession IDs (NCBI_ss#)
Ca_Kabuli_NC_021164.1_38863750	Ca_kabuli_chr05	38863750	[T/C]	1067298287
Ca_Kabuli_NC_021164.1_38878823	Ca_kabuli_chr05	38878823	[T/C]	1067298288
Ca_Kabuli_NC_021164.1_38878843	Ca_kabuli_chr05	38878843	[T/C]	1067298289
Ca_Kabuli_NC_021164.1_38878870	Ca_kabuli_chr05	38878870	[A/G]	1067298290
Ca_Kabuli_NC_021164.1_38878890	Ca_kabuli_chr05	38878890	[A/G]	1067298291
Ca_Kabuli_NC_021164.1_38898933	Ca_kabuli_chr05	38898933	[T/C]	1067298292
Ca_Kabuli_NC_021164.1_38909711	Ca_kabuli_chr05	38909711	[T/G]	1067298293
Ca_Kabuli_NC_021164.1_38909720	Ca_kabuli_chr05	38909720	[T/C]	1067298294
Ca_Kabuli_NC_021164.1_38984704	Ca_kabuli_chr05	38984704	[A/G]	1067298295
Ca_Kabuli_NC_021164.1_38984707	Ca_kabuli_chr05	38984707	[T/A]	1067298296
Ca_Kabuli_NC_021164.1_38984743	Ca_kabuli_chr05	38984743	[A/G]	1067298297
Ca_Kabuli_NC_021164.1_38984746	Ca_kabuli_chr05	38984746	[A/G]	1067298298
Ca_Kabuli_NC_021164.1_39038254	Ca_kabuli_chr05	39038254	[A/T]	1067298299
Ca_Kabuli_NC_021164.1_39038305	Ca_kabuli_chr05	39038305	[T/A]	1067298300
Ca_Kabuli_NC_021164.1_39038327	Ca_kabuli_chr05	39038327	[A/G]	1067298301
Ca_Kabuli_NC_021164.1_39038357	Ca_kabuli_chr05	39038357	[T/C]	1067298302
Ca_Kabuli_NC_021164.1_39134875	Ca_kabuli_chr05	39134875	[T/G]	1067298303
Ca_Kabuli_NC_021164.1_39137124	Ca_kabuli_chr05	39137124	[A/G]	1067298304
Ca_Kabuli_NC_021164.1_39137135	Ca_kabuli_chr05	39137135	[T/G]	1067298305
Ca_Kabuli_NC_021164.1_39137174	Ca_kabuli_chr05	39137174	[A/C]	1067298306
Ca_Kabuli_NC_021164.1_39137175	Ca_kabuli_chr05	39137175	[G/T]	1067298307
Ca_Kabuli_NC_021164.1_39175009	Ca_kabuli_chr05	39175009	[A/T]	1067298308
Ca_Kabuli_NC_021164.1_39176406	Ca_kabuli_chr05	39176406	[A/C]	1067298309
Ca_Kabuli_NC_021164.1_39194838	Ca_kabuli_chr05	39194838	[T/G]	1067298310
Ca_Kabuli_NC_021164.1_39243641	Ca_kabuli_chr05	39243641	[A/T]	1067298311
Ca_Kabuli_NC_021164.1_39243661	Ca_kabuli_chr05	39243661	[A/G]	1067298312
Ca_Kabuli_NC_021164.1_39282613	Ca_kabuli_chr05	39282613	[A/G]	1067298313
Ca_Kabuli_NC_021164.1_39282658	Ca_kabuli_chr05	39282658	[A/C]	1067298314
Ca_Kabuli_NC_021164.1_39364695	Ca_kabuli_chr05	39364695	[A/C]	1067298315
Ca_Kabuli_NC_021164.1_39364706	Ca_kabuli_chr05	39364706	[T/A]	1067298316
Ca_Kabuli_NC_021164.1_39364717	Ca_kabuli_chr05	39364717	[T/A]	1067298317
Ca_Kabuli_NC_021164.1_39410329	Ca_kabuli_chr05	39410329	[T/G]	1067298318
Ca_Kabuli_NC_021164.1_39417681	Ca_kabuli_chr05	39417681	[T/G]	1067298319
Ca_Kabuli_NC_021164.1_39477227	Ca_kabuli_chr05	39477227	[A/T]	1067298320

SNP IDs	Chromosomes	Physical positions (bp)	SNPs	NCBI SNPdb accession IDs (NCBI_ss#)
Ca_Kabuli_NC_021164.1_39477231	Ca_kabuli_chr05	39477231	[A/G]	1067298321
Ca_Kabuli_NC_021164.1_39477243	Ca_kabuli_chr05	39477243	[A/G]	1067298322
Ca_Kabuli_NC_021164.1_39477264	Ca_kabuli_chr05	39477264	[T/C]	1067298323
Ca_Kabuli_NC_021164.1_39547858	Ca_kabuli_chr05	39547858	[T/G]	1067298324
Ca_Kabuli_NC_021164.1_39585358	Ca_kabuli_chr05	39585358	[C/G]	1067298325
Ca_Kabuli_NC_021164.1_39597565	Ca_kabuli_chr05	39597565	[C/A]	1067298326
Ca_Kabuli_NC_021164.1_39597605	Ca_kabuli_chr05	39597605	[A/C]	1067298327
Ca_Kabuli_NC_021164.1_39608135	Ca_kabuli_chr05	39608135	[T/C]	1067298328
Ca_Kabuli_NC_021164.1_39616068	Ca_kabuli_chr05	39616068	[A/G]	1067298329
Ca_Kabuli_NC_021164.1_39616109	Ca_kabuli_chr05	39616109	[A/G]	1067298330
Ca_Kabuli_NC_021164.1_39661648	Ca_kabuli_chr05	39661648	[A/G]	1067298331
Ca_Kabuli_NC_021164.1_39661686	Ca_kabuli_chr05	39661686	[A/G]	1067298332
Ca_Kabuli_NC_021164.1_39661715	Ca_kabuli_chr05	39661715	[T/C]	1067298333
Ca_Kabuli_NC_021164.1_39794784	Ca_kabuli_chr05	39794784	[T/A]	1067298334
Ca_Kabuli_NC_021164.1_39794915	Ca_kabuli_chr05	39794915	[A/G]	1067298335
Ca_Kabuli_NC_021164.1_39831580	Ca_kabuli_chr05	39831580	[A/G]	1067298336
Ca_Kabuli_NC_021164.1_39869372	Ca_kabuli_chr05	39869372	[A/T]	1067298337
Ca_Kabuli_NC_021164.1_39869373	Ca_kabuli_chr05	39869373	[T/C]	1067298338
Ca_Kabuli_NC_021164.1_39869380	Ca_kabuli_chr05	39869380	[A/C]	1067298339
Ca_Kabuli_NC_021164.1_39869389	Ca_kabuli_chr05	39869389	[A/C]	1067298340
Ca_Kabuli_NC_021164.1_39869413	Ca_kabuli_chr05	39869413	[T/C]	1067298341
Ca_Kabuli_NC_021164.1_39906735	Ca_kabuli_chr05	39906735	[A/C]	1067298342
Ca_Kabuli_NC_021164.1_39943298	Ca_kabuli_chr05	39943298	[T/C]	1067298343
Ca_Kabuli_NC_021164.1_39964231	Ca_kabuli_chr05	39964231	[T/C]	1067298344
Ca_Kabuli_NC_021164.1_39984988	Ca_kabuli_chr05	39984988	[T/C]	1067298345
Ca_Kabuli_NC_021164.1_40018205	Ca_kabuli_chr05	40018205	[A/G]	1067298346
Ca_Kabuli_NC_021164.1_40018223	Ca_kabuli_chr05	40018223	[T/G]	1067298347
Ca_Kabuli_NC_021164.1_40018278	Ca_kabuli_chr05	40018278	[A/G]	1067298348
Ca_Kabuli_NC_021164.1_40018357	Ca_kabuli_chr05	40018357	[A/T]	1067298349
Ca_Kabuli_NC_021164.1_40018377	Ca_kabuli_chr05	40018377	[T/C]	1067298350
Ca_Kabuli_NC_021164.1_40025273	Ca_kabuli_chr05	40025273	[T/C]	1067298351
Ca_Kabuli_NC_021164.1_40025286	Ca_kabuli_chr05	40025286	[T/G]	1067298352
Ca_Kabuli_NC_021164.1_40025377	Ca_kabuli_chr05	40025377	[A/T]	1067298353
Ca_Kabuli_NC_021164.1_40076969	Ca_kabuli_chr05	40076969	[T/G]	1067298354

SNP IDs	Chromosomes	Physical positions (bp)	SNPs	NCBI SNPdb accession IDs (NCBI_ss#)
Ca_Kabuli_NC_021164.1_40077026	Ca_kabuli_chr05	40077026	[A/C]	1067298355
Ca_Kabuli_NC_021164.1_40177096	Ca_kabuli_chr05	40177096	[A/G]	1067298356
Ca_Kabuli_NC_021164.1_40194063	Ca_kabuli_chr05	40194063	[G/C]	1067298357
Ca_Kabuli_NC_021164.1_40194065	Ca_kabuli_chr05	40194065	[T/A]	1067298358
Ca_Kabuli_NC_021164.1_40194187	Ca_kabuli_chr05	40194187	[C/A]	1067298359
Ca_Kabuli_NC_021164.1_40225151	Ca_kabuli_chr05	40225151	[T/G]	1067298360
Ca_Kabuli_NC_021164.1_40225325	Ca_kabuli_chr05	40225325	[T/C]	1067298361
Ca_Kabuli_NC_021164.1_40225427	Ca_kabuli_chr05	40225427	[A/G]	1067298362
Ca_Kabuli_NC_021164.1_40231832	Ca_kabuli_chr05	40231832	[A/C]	1067298363
Ca_Kabuli_NC_021164.1_40239326	Ca_kabuli_chr05	40239326	[T/C]	1067298364
Ca_Kabuli_NC_021164.1_40359446	Ca_kabuli_chr05	40359446	[G/A]	1067298365
Ca_Kabuli_NC_021164.1_40359447	Ca_kabuli_chr05	40359447	[T/C]	1067298366
Ca_Kabuli_NC_021164.1_40359449	Ca_kabuli_chr05	40359449	[T/G]	1067298367
Ca_Kabuli_NC_021164.1_40359451	Ca_kabuli_chr05	40359451	[C/A]	1067298368
Ca_Kabuli_NC_021164.1_40465368	Ca_kabuli_chr05	40465368	[T/G]	1067298369
Ca_Kabuli_NC_021164.1_40507145	Ca_kabuli_chr05	40507145	[A/C]	1067298370
Ca_Kabuli_NC_021164.1_40507180	Ca_kabuli_chr05	40507180	[A/T]	1067298371
Ca_Kabuli_NC_021164.1_40507611	Ca_kabuli_chr05	40507611	[A/G]	1067298372
Ca_Kabuli_NC_021164.1_40507633	Ca_kabuli_chr05	40507633	[T/C]	1067298373
Ca_Kabuli_NC_021164.1_40507636	Ca_kabuli_chr05	40507636	[T/C]	1067298374
Ca_Kabuli_NC_021164.1_40507646	Ca_kabuli_chr05	40507646	[A/G]	1067298375
Ca_Kabuli_NC_021164.1_40507648	Ca_kabuli_chr05	40507648	[A/G]	1067298376
Ca_Kabuli_NC_021164.1_40507650	Ca_kabuli_chr05	40507650	[T/G]	1067298377
Ca_Kabuli_NC_021164.1_40507681	Ca_kabuli_chr05	40507681	[A/C]	1067298378
Ca_Kabuli_NC_021164.1_40507690	Ca_kabuli_chr05	40507690	[A/G]	1067298379
Ca_Kabuli_NC_021164.1_40522458	Ca_kabuli_chr05	40522458	[C/A]	1067298380
Ca_Kabuli_NC_021164.1_40522459	Ca_kabuli_chr05	40522459	[C/G]	1067298381
Ca_Kabuli_NC_021164.1_40522461	Ca_kabuli_chr05	40522461	[A/T]	1067298382
Ca_Kabuli_NC_021164.1_40532395	Ca_kabuli_chr05	40532395	[T/G]	1067298383
Ca_Kabuli_NC_021164.1_40555173	Ca_kabuli_chr05	40555173	[A/C]	1067298384
Ca_Kabuli_NC_021164.1_40581463	Ca_kabuli_chr05	40581463	[T/G]	1067298385
Ca_Kabuli_NC_021164.1_40632784	Ca_kabuli_chr05	40632784	[T/G]	1067298386
Ca_Kabuli_NC_021164.1_40632786	Ca_kabuli_chr05	40632786	[A/G]	1067298387
Ca_Kabuli_NC_021164.1_40632788	Ca_kabuli_chr05	40632788	[C/T]	1067298388

SNP IDs	Chromosomes	Physical positions (bp)	SNPs	NCBI SNPdb accession IDs (NCBI_ss#)
Ca_Kabuli_NC_021164.1_40645183	Ca_kabuli_chr05	40645183	[A/C]	1067298389
Ca_Kabuli_NC_021164.1_40645186	Ca_kabuli_chr05	40645186	[A/C]	1067298390
Ca_Kabuli_NC_021164.1_40700278	Ca_kabuli_chr05	40700278	[C/G]	1067298391
Ca_Kabuli_NC_021164.1_40700290	Ca_kabuli_chr05	40700290	[C/A]	1067298392
Ca_Kabuli_NC_021164.1_40700301	Ca_kabuli_chr05	40700301	[A/G]	1067298393
Ca_Kabuli_NC_021164.1_40700304	Ca_kabuli_chr05	40700304	[A/C]	1067298394
Ca_Kabuli_NC_021164.1_40700712	Ca_kabuli_chr05	40700712	[T/G]	1067298395
Ca_Kabuli_NC_021164.1_40700728	Ca_kabuli_chr05	40700728	[A/T]	1067298396
Ca_Kabuli_NC_021164.1_40710814	Ca_kabuli_chr05	40710814	[T/C]	1067298397
Ca_Kabuli_NC_021164.1_40710821	Ca_kabuli_chr05	40710821	[T/C]	1067298398
Ca_Kabuli_NC_021164.1_40736291	Ca_kabuli_chr05	40736291	[C/G]	1067298399
Ca_Kabuli_NC_021164.1_40812162	Ca_kabuli_chr05	40812162	[C/G]	1067298400
Ca_Kabuli_NC_021164.1_40828502	Ca_kabuli_chr05	40828502	[T/C]	1067298401
Ca_Kabuli_NC_021164.1_40828504	Ca_kabuli_chr05	40828504	[A/G]	1067298402
Ca_Kabuli_NC_021164.1_40828518	Ca_kabuli_chr05	40828518	[A/G]	1067298403
Ca_Kabuli_NC_021164.1_40828525	Ca_kabuli_chr05	40828525	[A/C]	1067298404
Ca_Kabuli_NC_021164.1_40828566	Ca_kabuli_chr05	40828566	[A/G]	1067298405
Ca_Kabuli_NC_021164.1_40874321	Ca_kabuli_chr05	40874321	[T/A]	1067298406
Ca_Kabuli_NC_021164.1_40883389	Ca_kabuli_chr05	40883389	[A/T]	1067298407
Ca_Kabuli_NC_021164.1_40883421	Ca_kabuli_chr05	40883421	[T/G]	1067298408
Ca_Kabuli_NC_021164.1_40883426	Ca_kabuli_chr05	40883426	[A/G]	1067298409
Ca_Kabuli_NC_021164.1_40883428	Ca_kabuli_chr05	40883428	[A/G]	1067298410
Ca_Kabuli_NC_021164.1_40883432	Ca_kabuli_chr05	40883432	[T/G]	1067298411
Ca_Kabuli_NC_021164.1_40953332	Ca_kabuli_chr05	40953332	[A/G]	1067298412
Ca_Kabuli_NC_021164.1_40953339	Ca_kabuli_chr05	40953339	[A/G]	1067298413
Ca_Kabuli_NC_021164.1_40966162	Ca_kabuli_chr05	40966162	[C/A]	1067298414
Ca_Kabuli_NC_021164.1_40966164	Ca_kabuli_chr05	40966164	[T/A]	1067298415
Ca_Kabuli_NC_021164.1_40966165	Ca_kabuli_chr05	40966165	[C/T]	1067298416
Ca_Kabuli_NC_021164.1_40981244	Ca_kabuli_chr05	40981244	[A/G]	1067298417
Ca_Kabuli_NC_021164.1_40981277	Ca_kabuli_chr05	40981277	[C/A]	1067298418
Ca_Kabuli_NC_021164.1_41024097	Ca_kabuli_chr05	41024097	[A/G]	1067298419
Ca_Kabuli_NC_021164.1_41024107	Ca_kabuli_chr05	41024107	[T/G]	1067298420
Ca_Kabuli_NC_021164.1_41054515	Ca_kabuli_chr05	41054515	[C/T]	1067298421
Ca_Kabuli_NC_021164.1_41054551	Ca_kabuli_chr05	41054551	[A/C]	1067298422

SNP IDs	Chromosomes	Physical positions (bp)	SNPs	NCBI SNPdb accession IDs (NCBI_ss#)
Ca_Kabuli_NC_021164.1_41054591	Ca_kabuli_chr05	41054591	[A/G]	1067298423
Ca_Kabuli_NC_021164.1_41073398	Ca_kabuli_chr05	41073398	[T/G]	1067298424
Ca_Kabuli_NC_021164.1_41073515	Ca_kabuli_chr05	41073515	[T/G]	1067298425
Ca_Kabuli_NC_021164.1_41073554	Ca_kabuli_chr05	41073554	[A/G]	1067298426
Ca_Kabuli_NC_021164.1_41074286	Ca_kabuli_chr05	41074286	[T/G]	1067298427
Ca_Kabuli_NC_021164.1_41211612	Ca_kabuli_chr05	41211612	[T/G]	1067298428
Ca_Kabuli_NC_021164.1_41282783	Ca_kabuli_chr05	41282783	[A/C]	1067298429
Ca_Kabuli_NC_021164.1_41282784	Ca_kabuli_chr05	41282784	[G/T]	1067298430
Ca_Kabuli_NC_021164.1_41282785	Ca_kabuli_chr05	41282785	[A/T]	1067298431
Ca_Kabuli_NC_021164.1_41282786	Ca_kabuli_chr05	41282786	[T/G]	1067298432
Ca_Kabuli_NC_021164.1_41283153	Ca_kabuli_chr05	41283153	[G/T]	1067298433
Ca_Kabuli_NC_021164.1_41283314	Ca_kabuli_chr05	41283314	[T/G]	1067298434
Ca_Kabuli_NC_021164.1_41283453	Ca_kabuli_chr05	41283453	[A/C]	1067298435
Ca_Kabuli_NC_021164.1_41285170	Ca_kabuli_chr05	41285170	[T/C]	1067298436
Ca_Kabuli_NC_021164.1_41285171	Ca_kabuli_chr05	41285171	[G/T]	1067298437
Ca_Kabuli_NC_021164.1_41301110	Ca_kabuli_chr05	41301110	[T/C]	1067298438
Ca_Kabuli_NC_021164.1_41301124	Ca_kabuli_chr05	41301124	[A/T]	1067298439
Ca_Kabuli_NC_021164.1_41301172	Ca_kabuli_chr05	41301172	[T/C]	1067298440
Ca_Kabuli_NC_021164.1_41301209	Ca_kabuli_chr05	41301209	[T/A]	1067298441
Ca_Kabuli_NC_021164.1_41301215	Ca_kabuli_chr05	41301215	[A/T]	1067298442
Ca_Kabuli_NC_021164.1_41301231	Ca_kabuli_chr05	41301231	[A/T]	1067298443
Ca_Kabuli_NC_021164.1_41464556	Ca_kabuli_chr05	41464556	[A/T]	1067298444
Ca_Kabuli_NC_021164.1_41464576	Ca_kabuli_chr05	41464576	[A/T]	1067298445
Ca_Kabuli_NC_021164.1_41524246	Ca_kabuli_chr05	41524246	[T/G]	1067298446
Ca_Kabuli_NC_021164.1_41525956	Ca_kabuli_chr05	41525956	[C/G]	1067298447
Ca_Kabuli_NC_021164.1_41525957	Ca_kabuli_chr05	41525957	[T/A]	1067298448
Ca_Kabuli_NC_021164.1_41525961	Ca_kabuli_chr05	41525961	[T/G]	1067298449
Ca_Kabuli_NC_021164.1_41635235	Ca_kabuli_chr05	41635235	[T/A]	1067298450
Ca_Kabuli_NC_021164.1_41635256	Ca_kabuli_chr05	41635256	[A/C]	1067298451
Ca_Kabuli_NC_021164.1_41635335	Ca_kabuli_chr05	41635335	[A/G]	1067298452
Ca_Kabuli_NC_021164.1_41635373	Ca_kabuli_chr05	41635373	[A/G]	1067298453
Ca_Kabuli_NC_021164.1_41635375	Ca_kabuli_chr05	41635375	[A/G]	1067298454
Ca_Kabuli_NC_021164.1_41678072	Ca_kabuli_chr05	41678072	[T/A]	1067298455
Ca_Kabuli_NC_021164.1_41696457	Ca_kabuli_chr05	41696457	[T/G]	1067298456

SNP IDs	Chromosomes	Physical positions (bp)	SNPs	NCBI SNPdb accession IDs (NCBI_ss#)
Ca_Kabuli_NC_021164.1_41710180	Ca_kabuli_chr05	41710180	[T/C]	1067298457
Ca_Kabuli_NC_021164.1_41711642	Ca_kabuli_chr05	41711642	[G/A]	1067298458
Ca_Kabuli_NC_021164.1_41711654	Ca_kabuli_chr05	41711654	[T/C]	1067298459
Ca_Kabuli_NC_021164.1_41834116	Ca_kabuli_chr05	41834116	[G/T]	1067298460
Ca_Kabuli_NC_021164.1_41837465	Ca_kabuli_chr05	41837465	[A/C]	1067298461
Ca_Kabuli_NC_021164.1_41837505	Ca_kabuli_chr05	41837505	[A/C]	1067298462
Ca_Kabuli_NC_021164.1_41849742	Ca_kabuli_chr05	41849742	[A/G]	1067298463
Ca_Kabuli_NC_021164.1_41863613	Ca_kabuli_chr05	41863613	[A/G]	1067298464
Ca_Kabuli_NC_021164.1_41863617	Ca_kabuli_chr05	41863617	[T/C]	1067298465
Ca_Kabuli_NC_021164.1_41863621	Ca_kabuli_chr05	41863621	[T/A]	1067298466
Ca_Kabuli_NC_021164.1_41863693	Ca_kabuli_chr05	41863693	[A/C]	1067298467
Ca_Kabuli_NC_021164.1_41863824	Ca_kabuli_chr05	41863824	[A/C]	1067298468
Ca_Kabuli_NC_021164.1_41996791	Ca_kabuli_chr05	41996791	[A/G]	1067298469
Ca_Kabuli_NC_021164.1_41996869	Ca_kabuli_chr05	41996869	[T/C]	1067298470
Ca_Kabuli_NC_021164.1_41996873	Ca_kabuli_chr05	41996873	[A/C]	1067298471
Ca_Kabuli_NC_021164.1_41996876	Ca_kabuli_chr05	41996876	[T/C]	1067298472
Ca_Kabuli_NC_021164.1_41996888	Ca_kabuli_chr05	41996888	[T/C]	1067298473
Ca_Kabuli_NC_021164.1_41996906	Ca_kabuli_chr05	41996906	[A/C]	1067298474
Ca_Kabuli_NC_021164.1_42004600	Ca_kabuli_chr05	42004600	[T/G]	1067298475
Ca_Kabuli_NC_021164.1_42004602	Ca_kabuli_chr05	42004602	[A/G]	1067298476
Ca_Kabuli_NC_021164.1_42004606	Ca_kabuli_chr05	42004606	[C/T]	1067298477
Ca_Kabuli_NC_021164.1_42011170	Ca_kabuli_chr05	42011170	[T/C]	1067298478
Ca_Kabuli_NC_021164.1_42011171	Ca_kabuli_chr05	42011171	[C/T]	1067298479
Ca_Kabuli_NC_021164.1_42011172	Ca_kabuli_chr05	42011172	[T/C]	1067298480
Ca_Kabuli_NC_021164.1_42013006	Ca_kabuli_chr05	42013006	[A/G]	1067298481
Ca_Kabuli_NC_021164.1_42013021	Ca_kabuli_chr05	42013021	[A/G]	1067298482
Ca_Kabuli_NC_021164.1_42013039	Ca_kabuli_chr05	42013039	[A/G]	1067298483
Ca_Kabuli_NC_021164.1_42028103	Ca_kabuli_chr05	42028103	[T/G]	1067298484
Ca_Kabuli_NC_021164.1_42052317	Ca_kabuli_chr05	42052317	[C/T]	1067298485
Ca_Kabuli_NC_021164.1_42052318	Ca_kabuli_chr05	42052318	[T/C]	1067298486
Ca_Kabuli_NC_021164.1_42052319	Ca_kabuli_chr05	42052319	[G/T]	1067298487
Ca_Kabuli_NC_021164.1_42120694	Ca_kabuli_chr05	42120694	[A/G]	1067298488
Ca_Kabuli_NC_021164.1_42120710	Ca_kabuli_chr05	42120710	[T/C]	1067298489
Ca_Kabuli_NC_021164.1_42120718	Ca_kabuli_chr05	42120718	[A/G]	1067298490

SNP IDs	Chromosomes	Physical positions (bp)	SNPs	NCBI SNPdb accession IDs (NCBI_ss#)
Ca_Kabuli_NC_021164.1_42120734	Ca_kabuli_chr05	42120734	[T/A]	1067298491
Ca_Kabuli_NC_021164.1_42225601	Ca_kabuli_chr05	42225601	[T/C]	1067298492
Ca_Kabuli_NC_021164.1_42275529	Ca_kabuli_chr05	42275529	[A/C]	1067298493
Ca_Kabuli_NC_021164.1_42351849	Ca_kabuli_chr05	42351849	[A/G]	1067298494
Ca_Kabuli_NC_021164.1_42351864	Ca_kabuli_chr05	42351864	[T/A]	1067298495
Ca_Kabuli_NC_021164.1_42351886	Ca_kabuli_chr05	42351886	[T/C]	1067298496
Ca_Kabuli_NC_021164.1_42376126	Ca_kabuli_chr05	42376126	[A/C]	1067298497
Ca_Kabuli_NC_021164.1_42376150	Ca_kabuli_chr05	42376150	[T/A]	1067298498
Ca_Kabuli_NC_021164.1_42397886	Ca_kabuli_chr05	42397886	[T/G]	1067298499
Ca_Kabuli_NC_021164.1_42397895	Ca_kabuli_chr05	42397895	[T/G]	1067298500
Ca_Kabuli_NC_021164.1_42397909	Ca_kabuli_chr05	42397909	[A/C]	1067298501
Ca_Kabuli_NC_021164.1_42397921	Ca_kabuli_chr05	42397921	[A/G]	1067298502
Ca_Kabuli_NC_021164.1_42397937	Ca_kabuli_chr05	42397937	[A/G]	1067298503
Ca_Kabuli_NC_021164.1_42412760	Ca_kabuli_chr05	42412760	[T/G]	1067298504
Ca_Kabuli_NC_021164.1_42422965	Ca_kabuli_chr05	42422965	[A/G]	1067298505
Ca_Kabuli_NC_021164.1_42435224	Ca_kabuli_chr05	42435224	[T/C]	1067298506
Ca_Kabuli_NC_021164.1_42443752	Ca_kabuli_chr05	42443752	[A/C]	1067298507
Ca_Kabuli_NC_021164.1_42465328	Ca_kabuli_chr05	42465328	[T/A]	1067298508
Ca_Kabuli_NC_021164.1_42465333	Ca_kabuli_chr05	42465333	[T/G]	1067298509
Ca_Kabuli_NC_021164.1_42465340	Ca_kabuli_chr05	42465340	[T/C]	1067298510
Ca_Kabuli_NC_021164.1_42499612	Ca_kabuli_chr05	42499612	[T/C]	1067298511
Ca_Kabuli_NC_021164.1_42499694	Ca_kabuli_chr05	42499694	[A/G]	1067298512
Ca_Kabuli_NC_021164.1_42499697	Ca_kabuli_chr05	42499697	[A/C]	1067298513
Ca_Kabuli_NC_021164.1_42528648	Ca_kabuli_chr05	42528648	[T/G]	1067298514
Ca_Kabuli_NC_021164.1_42528650	Ca_kabuli_chr05	42528650	[A/G]	1067298515
Ca_Kabuli_NC_021164.1_42528679	Ca_kabuli_chr05	42528679	[T/C]	1067298516
Ca_Kabuli_NC_021164.1_42528744	Ca_kabuli_chr05	42528744	[T/G]	1067298517
Ca_Kabuli_NC_021164.1_42528923	Ca_kabuli_chr05	42528923	[G/T]	1067298518
Ca_Kabuli_NC_021164.1_42528925	Ca_kabuli_chr05	42528925	[T/G]	1067298519
Ca_Kabuli_NC_021164.1_42528926	Ca_kabuli_chr05	42528926	[G/C]	1067298520
Ca_Kabuli_NC_021164.1_42528929	Ca_kabuli_chr05	42528929	[C/G]	1067298521
Ca_Kabuli_NC_021164.1_42528931	Ca_kabuli_chr05	42528931	[G/A]	1067298522
Ca_Kabuli_NC_021164.1_42528934	Ca_kabuli_chr05	42528934	[A/C]	1067298523
Ca_Kabuli_NC_021164.1_42528935	Ca_kabuli_chr05	42528935	[C/A]	1067298524

SNP IDs	Chromosomes	Physical positions (bp)	SNPs	NCBI SNPdb accession IDs (NCBI_ss#)
Ca_Kabuli_NC_021164.1_42528949	Ca_kabuli_chr05	42528949	[T/C]	1067298525
Ca_Kabuli_NC_021164.1_42528985	Ca_kabuli_chr05	42528985	[T/A]	1067298526
Ca_Kabuli_NC_021164.1_42528987	Ca_kabuli_chr05	42528987	[A/T]	1067298527
Ca_Kabuli_NC_021164.1_42615551	Ca_kabuli_chr05	42615551	[A/G]	1067298528
Ca_Kabuli_NC_021164.1_42655065	Ca_kabuli_chr05	42655065	[T/G]	1067298529
Ca_Kabuli_NC_021164.1_42655183	Ca_kabuli_chr05	42655183	[A/T]	1067298530
Ca_Kabuli_NC_021164.1_42655187	Ca_kabuli_chr05	42655187	[T/C]	1067298531
Ca_Kabuli_NC_021164.1_42655218	Ca_kabuli_chr05	42655218	[A/G]	1067298532
Ca_Kabuli_NC_021164.1_42791062	Ca_kabuli_chr05	42791062	[A/G]	1067298533
Ca_Kabuli_NC_021164.1_42791087	Ca_kabuli_chr05	42791087	[A/T]	1067298534
Ca_Kabuli_NC_021164.1_42791118	Ca_kabuli_chr05	42791118	[A/G]	1067298535
Ca_Kabuli_NC_021164.1_42792617	Ca_kabuli_chr05	42792617	[T/G]	1067298536
Ca_Kabuli_NC_021164.1_42811405	Ca_kabuli_chr05	42811405	[T/G]	1067298537
Ca_Kabuli_NC_021164.1_42856743	Ca_kabuli_chr05	42856743	[A/C]	1067298538
Ca_Kabuli_NC_021164.1_42863372	Ca_kabuli_chr05	42863372	[A/T]	1067298539
Ca_Kabuli_NC_021164.1_42882741	Ca_kabuli_chr05	42882741	[A/C]	1067298540
Ca_Kabuli_NC_021164.1_42882762	Ca_kabuli_chr05	42882762	[A/C]	1067298541
Ca_Kabuli_NC_021164.1_42882853	Ca_kabuli_chr05	42882853	[A/C]	1067298542
Ca_Kabuli_NC_021164.1_42882933	Ca_kabuli_chr05	42882933	[T/A]	1067298543
Ca_Kabuli_NC_021164.1_42882964	Ca_kabuli_chr05	42882964	[A/C]	1067298544
Ca_Kabuli_NC_021164.1_42882969	Ca_kabuli_chr05	42882969	[A/C]	1067298545
Ca_Kabuli_NC_021164.1_42910653	Ca_kabuli_chr05	42910653	[A/C]	1067298546
Ca_Kabuli_NC_021164.1_42928950	Ca_kabuli_chr05	42928950	[C/G]	1067298547
Ca_Kabuli_NC_021164.1_42928969	Ca_kabuli_chr05	42928969	[T/G]	1067298548
Ca_Kabuli_NC_021164.1_42928974	Ca_kabuli_chr05	42928974	[T/G]	1067298549
Ca_Kabuli_NC_021164.1_42929055	Ca_kabuli_chr05	42929055	[A/G]	1067298550
Ca_Kabuli_NC_021164.1_43012119	Ca_kabuli_chr05	43012119	[A/C]	1067298551
Ca_Kabuli_NC_021164.1_43022588	Ca_kabuli_chr05	43022588	[A/C]	1067298552
Ca_Kabuli_NC_021164.1_43035015	Ca_kabuli_chr05	43035015	[A/G]	1067298553
Ca_Kabuli_NC_021164.1_43035039	Ca_kabuli_chr05	43035039	[G/T]	1067298554
Ca_Kabuli_NC_021164.1_43035049	Ca_kabuli_chr05	43035049	[A/G]	1067298555
Ca_Kabuli_NC_021164.1_43037858	Ca_kabuli_chr05	43037858	[A/C]	1067298556
Ca_Kabuli_NC_021164.1_43059960	Ca_kabuli_chr05	43059960	[G/C]	1067298557
Ca_Kabuli_NC_021164.1_43060040	Ca_kabuli_chr05	43060040	[A/C]	1067298558

SNP IDs	Chromosomes	Physical positions (bp)	SNPs	NCBI SNPdb accession IDs (NCBI_ss#)
Ca_Kabuli_NC_021164.1_43060061	Ca_kabuli_chr05	43060061	[A/C]	1067298559
Ca_Kabuli_NC_021164.1_43060223	Ca_kabuli_chr05	43060223	[A/C]	1067298560
Ca_Kabuli_NC_021164.1_43080452	Ca_kabuli_chr05	43080452	[A/T]	1067298561
Ca_Kabuli_NC_021164.1_43080501	Ca_kabuli_chr05	43080501	[T/A]	1067298562
Ca_Kabuli_NC_021164.1_43081728	Ca_kabuli_chr05	43081728	[T/C]	1067298563
Ca_Kabuli_NC_021164.1_43081737	Ca_kabuli_chr05	43081737	[A/G]	1067298564
Ca_Kabuli_NC_021164.1_43081768	Ca_kabuli_chr05	43081768	[A/G]	1067298565
Ca_Kabuli_NC_021164.1_43101150	Ca_kabuli_chr05	43101150	[T/C]	1067298566
Ca_Kabuli_NC_021164.1_43167941	Ca_kabuli_chr05	43167941	[A/C]	1067298567
Ca_Kabuli_NC_021164.1_43175941	Ca_kabuli_chr05	43175941	[T/G]	1067298568
Ca_Kabuli_NC_021164.1_43188858	Ca_kabuli_chr05	43188858	[C/A]	1067298569
Ca_Kabuli_NC_021164.1_43188864	Ca_kabuli_chr05	43188864	[C/A]	1067298570
Ca_Kabuli_NC_021164.1_43188867	Ca_kabuli_chr05	43188867	[C/G]	1067298571
Ca_Kabuli_NC_021164.1_43188880	Ca_kabuli_chr05	43188880	[T/C]	1067298572
Ca_Kabuli_NC_021164.1_43188882	Ca_kabuli_chr05	43188882	[T/C]	1067298573
Ca_Kabuli_NC_021164.1_43188888	Ca_kabuli_chr05	43188888	[C/T]	1067298574
Ca_Kabuli_NC_021164.1_43206862	Ca_kabuli_chr05	43206862	[A/T]	1067298575
Ca_Kabuli_NC_021164.1_43212064	Ca_kabuli_chr05	43212064	[A/C]	1067298576
Ca_Kabuli_NC_021164.1_43212076	Ca_kabuli_chr05	43212076	[T/A]	1067298577
Ca_Kabuli_NC_021164.1_43212081	Ca_kabuli_chr05	43212081	[A/C]	1067298578
Ca_Kabuli_NC_021164.1_43221060	Ca_kabuli_chr05	43221060	[T/G]	1067298579
Ca_Kabuli_NC_021164.1_43253654	Ca_kabuli_chr05	43253654	[A/T]	1067298580
Ca_Kabuli_NC_021164.1_43262643	Ca_kabuli_chr05	43262643	[T/C]	1067298581
Ca_Kabuli_NC_021164.1_43307596	Ca_kabuli_chr05	43307596	[T/C]	1067298582
Ca_Kabuli_NC_021164.1_43307647	Ca_kabuli_chr05	43307647	[T/C]	1067298583
Ca_Kabuli_NC_021164.1_43307720	Ca_kabuli_chr05	43307720	[T/C]	1067298584
Ca_Kabuli_NC_021164.1_43307726	Ca_kabuli_chr05	43307726	[A/G]	1067298585
Ca_Kabuli_NC_021164.1_43326670	Ca_kabuli_chr05	43326670	[T/C]	1067298586
Ca_Kabuli_NC_021164.1_43388057	Ca_kabuli_chr05	43388057	[A/G]	1067298587
Ca_Kabuli_NC_021164.1_43407702	Ca_kabuli_chr05	43407702	[T/G]	1067298588
Ca_Kabuli_NC_021164.1_43410316	Ca_kabuli_chr05	43410316	[A/T]	1067298589
Ca_Kabuli_NC_021164.1_43423931	Ca_kabuli_chr05	43423931	[C/A]	1067298590
Ca_Kabuli_NC_021164.1_43512090	Ca_kabuli_chr05	43512090	[A/G]	1067298591
Ca_Kabuli_NC_021164.1_43512116	Ca_kabuli_chr05	43512116	[T/A]	1067298592

SNP IDs	Chromosomes	Physical positions (bp)	SNPs	NCBI SNPdb accession IDs (NCBI_ss#)
Ca_Kabuli_NC_021164.1_43512129	Ca_kabuli_chr05	43512129	[A/T]	1067298593
Ca_Kabuli_NC_021164.1_43521823	Ca_kabuli_chr05	43521823	[A/G]	1067298594
Ca_Kabuli_NC_021164.1_43523430	Ca_kabuli_chr05	43523430	[T/C]	1067298595
Ca_Kabuli_NC_021164.1_43523520	Ca_kabuli_chr05	43523520	[T/G]	1067298596
Ca_Kabuli_NC_021164.1_43530592	Ca_kabuli_chr05	43530592	[T/G]	1067298597
Ca_Kabuli_NC_021164.1_43632408	Ca_kabuli_chr05	43632408	[T/A]	1067298598
Ca_Kabuli_NC_021164.1_43698024	Ca_kabuli_chr05	43698024	[T/G]	1067298599
Ca_Kabuli_NC_021164.1_43698051	Ca_kabuli_chr05	43698051	[T/C]	1067298600
Ca_Kabuli_NC_021164.1_43731650	Ca_kabuli_chr05	43731650	[T/C]	1067298601
Ca_Kabuli_NC_021164.1_43754083	Ca_kabuli_chr05	43754083	[T/C]	1067298602
Ca_Kabuli_NC_021164.1_43754814	Ca_kabuli_chr05	43754814	[A/T]	1067298603
Ca_Kabuli_NC_021164.1_43754815	Ca_kabuli_chr05	43754815	[A/C]	1067298604
Ca_Kabuli_NC_021164.1_43754816	Ca_kabuli_chr05	43754816	[G/T]	1067298605
Ca_Kabuli_NC_021164.1_43754867	Ca_kabuli_chr05	43754867	[A/C]	1067298606
Ca_Kabuli_NC_021164.1_43755039	Ca_kabuli_chr05	43755039	[A/G]	1067298607
Ca_Kabuli_NC_021164.1_43755135	Ca_kabuli_chr05	43755135	[A/T]	1067298608
Ca_Kabuli_NC_021164.1_43758818	Ca_kabuli_chr05	43758818	[A/G]	1067298609
Ca_Kabuli_NC_021164.1_43758846	Ca_kabuli_chr05	43758846	[A/C]	1067298610
Ca_Kabuli_NC_021164.1_43759076	Ca_kabuli_chr05	43759076	[A/G]	1067298611
Ca_Kabuli_NC_021164.1_43759082	Ca_kabuli_chr05	43759082	[A/T]	1067298612
Ca_Kabuli_NC_021164.1_43759098	Ca_kabuli_chr05	43759098	[T/C]	1067298613
Ca_Kabuli_NC_021164.1_43779141	Ca_kabuli_chr05	43779141	[T/C]	1067298614
Ca_Kabuli_NC_021164.1_43779147	Ca_kabuli_chr05	43779147	[G/A]	1067298615
Ca_Kabuli_NC_021164.1_43779156	Ca_kabuli_chr05	43779156	[A/G]	1067298616
Ca_Kabuli_NC_021164.1_43779181	Ca_kabuli_chr05	43779181	[A/G]	1067298617
Ca_Kabuli_NC_021164.1_43789851	Ca_kabuli_chr05	43789851	[T/C]	1067298618
Ca_Kabuli_NC_021164.1_43789863	Ca_kabuli_chr05	43789863	[A/T]	1067298619
Ca_Kabuli_NC_021164.1_43789872	Ca_kabuli_chr05	43789872	[T/C]	1067298620
Ca_Kabuli_NC_021164.1_43790691	Ca_kabuli_chr05	43790691	[C/A]	1067298621
Ca_Kabuli_NC_021164.1_43790692	Ca_kabuli_chr05	43790692	[C/G]	1067298622
Ca_Kabuli_NC_021164.1_43790693	Ca_kabuli_chr05	43790693	[T/A]	1067298623
Ca_Kabuli_NC_021164.1_43790694	Ca_kabuli_chr05	43790694	[A/T]	1067298624
Ca_Kabuli_NC_021164.1_43834723	Ca_kabuli_chr05	43834723	[A/G]	1067298625
Ca_Kabuli_NC_021164.1_43834789	Ca_kabuli_chr05	43834789	[A/G]	1067298626

SNP IDs	Chromosomes	Physical positions (bp)	SNPs	NCBI SNPdb accession IDs (NCBI_ss#)
Ca_Kabuli_NC_021164.1_43855705	Ca_kabuli_chr05	43855705	[T/A]	1067298627
Ca_Kabuli_NC_021164.1_43892480	Ca_kabuli_chr05	43892480	[A/G]	1067298628
Ca_Kabuli_NC_021164.1_43892493	Ca_kabuli_chr05	43892493	[T/A]	1067298629
Ca_Kabuli_NC_021164.1_43904482	Ca_kabuli_chr05	43904482	[A/T]	1067298630
Ca_Kabuli_NC_021164.1_43904515	Ca_kabuli_chr05	43904515	[A/C]	1067298631
Ca_Kabuli_NC_021164.1_43919009	Ca_kabuli_chr05	43919009	[C/T]	1067298632
Ca_Kabuli_NC_021164.1_43919017	Ca_kabuli_chr05	43919017	[T/G]	1067298633
Ca_Kabuli_NC_021164.1_43919022	Ca_kabuli_chr05	43919022	[G/C]	1067298634
Ca_Kabuli_NC_021164.1_43919028	Ca_kabuli_chr05	43919028	[A/G]	1067298635
Ca_Kabuli_NC_021164.1_43919137	Ca_kabuli_chr05	43919137	[T/G]	1067298636
Ca_Kabuli_NC_021164.1_43982988	Ca_kabuli_chr05	43982988	[C/G]	1067298637
Ca_Kabuli_NC_021164.1_43982996	Ca_kabuli_chr05	43982996	[G/A]	1067298638
Ca_Kabuli_NC_021164.1_44019121	Ca_kabuli_chr05	44019121	[C/A]	1067298639
Ca_Kabuli_NC_021164.1_44019122	Ca_kabuli_chr05	44019122	[C/T]	1067298640
Ca_Kabuli_NC_021164.1_44019123	Ca_kabuli_chr05	44019123	[A/C]	1067298641
Ca_Kabuli_NC_021164.1_44019124	Ca_kabuli_chr05	44019124	[G/T]	1067298642
Ca_Kabuli_NC_021164.1_44060628	Ca_kabuli_chr05	44060628	[T/G]	1067298643
Ca_Kabuli_NC_021164.1_44060636	Ca_kabuli_chr05	44060636	[T/C]	1067298644
Ca_Kabuli_NC_021164.1_44060646	Ca_kabuli_chr05	44060646	[T/C]	1067298645
Ca_Kabuli_NC_021164.1_44060651	Ca_kabuli_chr05	44060651	[C/A]	1067298646
Ca_Kabuli_NC_021164.1_44060695	Ca_kabuli_chr05	44060695	[A/G]	1067298647
Ca_Kabuli_NC_021164.1_44080525	Ca_kabuli_chr05	44080525	[A/C]	1067298648
Ca_Kabuli_NC_021164.1_44101983	Ca_kabuli_chr05	44101983	[C/A]	1067298649
Ca_Kabuli_NC_021164.1_44101984	Ca_kabuli_chr05	44101984	[A/G]	1067298650
Ca_Kabuli_NC_021164.1_44101985	Ca_kabuli_chr05	44101985	[T/A]	1067298651
Ca_Kabuli_NC_021164.1_44101986	Ca_kabuli_chr05	44101986	[C/T]	1067298652
Ca_Kabuli_NC_021164.1_44101990	Ca_kabuli_chr05	44101990	[T/A]	1067298653
Ca_Kabuli_NC_021164.1_44109266	Ca_kabuli_chr05	44109266	[C/T]	1067298654
Ca_Kabuli_NC_021164.1_44109279	Ca_kabuli_chr05	44109279	[C/T]	1067298655
Ca_Kabuli_NC_021164.1_44159226	Ca_kabuli_chr05	44159226	[T/G]	1067298656
Ca_Kabuli_NC_021164.1_44159238	Ca_kabuli_chr05	44159238	[A/C]	1067298657
Ca_Kabuli_NC_021164.1_44215966	Ca_kabuli_chr05	44215966	[A/T]	1067298658
Ca_Kabuli_NC_021164.1_44244118	Ca_kabuli_chr05	44244118	[T/A]	1067298659
Ca_Kabuli_NC_021164.1_44275605	Ca_kabuli_chr05	44275605	[A/G]	1067298660

SNP IDs	Chromosomes	Physical positions (bp)	SNPs	NCBI SNPdb accession IDs (NCBI_ss#)
Ca_Kabuli_NC_021164.1_44300026	Ca_kabuli_chr05	44300026	[A/C]	1067298661
Ca_Kabuli_NC_021164.1_44308560	Ca_kabuli_chr05	44308560	[A/C]	1067298662
Ca_Kabuli_NC_021164.1_44308576	Ca_kabuli_chr05	44308576	[A/G]	1067298663
Ca_Kabuli_NC_021164.1_44334269	Ca_kabuli_chr05	44334269	[A/G]	1067298664
Ca_Kabuli_NC_021164.1_44334742	Ca_kabuli_chr05	44334742	[A/G]	1067298665
Ca_Kabuli_NC_021164.1_44334747	Ca_kabuli_chr05	44334747	[T/C]	1067298666
Ca_Kabuli_NC_021164.1_44334780	Ca_kabuli_chr05	44334780	[A/C]	1067298667
Ca_Kabuli_NC_021164.1_44334830	Ca_kabuli_chr05	44334830	[T/G]	1067298668
Ca_Kabuli_NC_021164.1_44334854	Ca_kabuli_chr05	44334854	[A/G]	1067298669
Ca_Kabuli_NC_021164.1_44422686	Ca_kabuli_chr05	44422686	[C/A]	1067298670
Ca_Kabuli_NC_021164.1_44422721	Ca_kabuli_chr05	44422721	[T/C]	1067298671
Ca_Kabuli_NC_021164.1_44428939	Ca_kabuli_chr05	44428939	[A/T]	1067298672
Ca_Kabuli_NC_021164.1_44428960	Ca_kabuli_chr05	44428960	[T/A]	1067298673
Ca_Kabuli_NC_021164.1_44428962	Ca_kabuli_chr05	44428962	[G/A]	1067298674
Ca_Kabuli_NC_021164.1_44555842	Ca_kabuli_chr05	44555842	[T/C]	1067298675
Ca_Kabuli_NC_021164.1_44555845	Ca_kabuli_chr05	44555845	[T/C]	1067298676
Ca_Kabuli_NC_021164.1_44555908	Ca_kabuli_chr05	44555908	[A/G]	1067298677
Ca_Kabuli_NC_021164.1_44561112	Ca_kabuli_chr05	44561112	[A/C]	1067298678
Ca_Kabuli_NC_021164.1_44561114	Ca_kabuli_chr05	44561114	[C/T]	1067298679
Ca_Kabuli_NC_021164.1_44656882	Ca_kabuli_chr05	44656882	[T/G]	1067298680
Ca_Kabuli_NC_021164.1_44760376	Ca_kabuli_chr05	44760376	[T/A]	1067298681
Ca_Kabuli_NC_021164.1_44760406	Ca_kabuli_chr05	44760406	[A/T]	1067298682
Ca_Kabuli_NC_021164.1_44768955	Ca_kabuli_chr05	44768955	[A/C]	1067298683
Ca_Kabuli_NC_021164.1_44769484	Ca_kabuli_chr05	44769484	[T/C]	1067298684
Ca_Kabuli_NC_021164.1_44769485	Ca_kabuli_chr05	44769485	[C/T]	1067298685
Ca_Kabuli_NC_021164.1_44772368	Ca_kabuli_chr05	44772368	[T/G]	1067298686
Ca_Kabuli_NC_021164.1_44772494	Ca_kabuli_chr05	44772494	[T/C]	1067298687
Ca_Kabuli_NC_021164.1_44772503	Ca_kabuli_chr05	44772503	[A/C]	1067298688
Ca_Kabuli_NC_021164.1_44822311	Ca_kabuli_chr05	44822311	[G/A]	1067298689
Ca_Kabuli_NC_021164.1_44822351	Ca_kabuli_chr05	44822351	[T/G]	1067298690
Ca_Kabuli_NC_021164.1_44824496	Ca_kabuli_chr05	44824496	[C/T]	1067298691
Ca_Kabuli_NC_021164.1_44824747	Ca_kabuli_chr05	44824747	[C/G]	1067298692
Ca_Kabuli_NC_021164.1_44832042	Ca_kabuli_chr05	44832042	[C/A]	1067298693
Ca_Kabuli_NC_021164.1_44832051	Ca_kabuli_chr05	44832051	[T/C]	1067298694

SNP IDs	Chromosomes	Physical positions (bp)	SNPs	NCBI SNPdb accession IDs (NCBI_ss#)
Ca_Kabuli_NC_021164.1_44875928	Ca_kabuli_chr05	44875928	[T/G]	1067298695
Ca_Kabuli_NC_021164.1_44887020	Ca_kabuli_chr05	44887020	[T/C]	1067298696
Ca_Kabuli_NC_021164.1_44890500	Ca_kabuli_chr05	44890500	[A/G]	1067298697
Ca_Kabuli_NC_021164.1_44895493	Ca_kabuli_chr05	44895493	[A/C]	1067298698
Ca_Kabuli_NC_021164.1_44895508	Ca_kabuli_chr05	44895508	[A/G]	1067298699
Ca_Kabuli_NC_021164.1_44895515	Ca_kabuli_chr05	44895515	[T/C]	1067298700
Ca_Kabuli_NC_021164.1_44895532	Ca_kabuli_chr05	44895532	[G/A]	1067298701
Ca_Kabuli_NC_021164.1_44911842	Ca_kabuli_chr05	44911842	[C/T]	1067298702
Ca_Kabuli_NC_021164.1_44918177	Ca_kabuli_chr05	44918177	[T/G]	1067298703
Ca_Kabuli_NC_021164.1_44918198	Ca_kabuli_chr05	44918198	[A/G]	1067298704
Ca_Kabuli_NC_021164.1_44938814	Ca_kabuli_chr05	44938814	[A/T]	1067298705
Ca_Kabuli_NC_021164.1_44972133	Ca_kabuli_chr05	44972133	[T/G]	1067298706
Ca_Kabuli_NC_021164.1_44975251	Ca_kabuli_chr05	44975251	[A/C]	1067298707
Ca_Kabuli_NC_021164.1_45219235	Ca_kabuli_chr05	45219235	[A/G]	1067298708
Ca_Kabuli_NC_021164.1_45219308	Ca_kabuli_chr05	45219308	[T/C]	1067298709
Ca_Kabuli_NC_021164.1_45219353	Ca_kabuli_chr05	45219353	[A/G]	1067298710
Ca_Kabuli_NC_021164.1_45254112	Ca_kabuli_chr05	45254112	[T/G]	1067298711
Ca_Kabuli_NC_021164.1_45301813	Ca_kabuli_chr05	45301813	[A/T]	1067298712
Ca_Kabuli_NC_021164.1_45312719	Ca_kabuli_chr05	45312719	[T/C]	1067298713
Ca_Kabuli_NC_021164.1_45337308	Ca_kabuli_chr05	45337308	[A/G]	1067298714
Ca_Kabuli_NC_021164.1_45337327	Ca_kabuli_chr05	45337327	[A/G]	1067298715
Ca_Kabuli_NC_021164.1_45337494	Ca_kabuli_chr05	45337494	[A/C]	1067298716
Ca_Kabuli_NC_021164.1_45337512	Ca_kabuli_chr05	45337512	[A/C]	1067298717
Ca_Kabuli_NC_021164.1_45407044	Ca_kabuli_chr05	45407044	[A/C]	1067298718
Ca_Kabuli_NC_021164.1_45413935	Ca_kabuli_chr05	45413935	[T/A]	1067298719
Ca_Kabuli_NC_021164.1_45418344	Ca_kabuli_chr05	45418344	[T/G]	1067298720
Ca_Kabuli_NC_021164.1_45418365	Ca_kabuli_chr05	45418365	[T/C]	1067298721
Ca_Kabuli_NC_021164.1_45478398	Ca_kabuli_chr05	45478398	[G/T]	1067298722
Ca_Kabuli_NC_021164.1_45480153	Ca_kabuli_chr05	45480153	[G/T]	1067298723
Ca_Kabuli_NC_021164.1_45480160	Ca_kabuli_chr05	45480160	[A/G]	1067298724
Ca_Kabuli_NC_021164.1_45480229	Ca_kabuli_chr05	45480229	[T/C]	1067298725
Ca_Kabuli_NC_021164.1_45480274	Ca_kabuli_chr05	45480274	[T/C]	1067298726
Ca_Kabuli_NC_021164.1_45506164	Ca_kabuli_chr05	45506164	[T/C]	1067298727
Ca_Kabuli_NC_021164.1_45607828	Ca_kabuli_chr05	45607828	[A/G]	1067298728

SNP IDs	Chromosomes	Physical positions (bp)	SNPs	NCBI SNPdb accession IDs (NCBI_ss#)
Ca_Kabuli_NC_021164.1_45642319	Ca_kabuli_chr05	45642319	[T/G]	1067298729
Ca_Kabuli_NC_021164.1_45642691	Ca_kabuli_chr05	45642691	[A/C]	1067298730
Ca_Kabuli_NC_021164.1_45646616	Ca_kabuli_chr05	45646616	[T/C]	1067298731
Ca_Kabuli_NC_021164.1_45646656	Ca_kabuli_chr05	45646656	[A/G]	1067298732
Ca_Kabuli_NC_021164.1_45646706	Ca_kabuli_chr05	45646706	[T/C]	1067298733
Ca_Kabuli_NC_021164.1_45647057	Ca_kabuli_chr05	45647057	[T/C]	1067298734
Ca_Kabuli_NC_021164.1_45649181	Ca_kabuli_chr05	45649181	[T/G]	1067298735
Ca_Kabuli_NC_021164.1_45649184	Ca_kabuli_chr05	45649184	[T/G]	1067298736
Ca_Kabuli_NC_021164.1_45694488	Ca_kabuli_chr05	45694488	[A/G]	1067298737
Ca_Kabuli_NC_021164.1_45694524	Ca_kabuli_chr05	45694524	[A/G]	1067298738
Ca_Kabuli_NC_021164.1_45704236	Ca_kabuli_chr05	45704236	[A/G]	1067298739
Ca_Kabuli_NC_021164.1_45704247	Ca_kabuli_chr05	45704247	[T/C]	1067298740
Ca_Kabuli_NC_021164.1_45704250	Ca_kabuli_chr05	45704250	[T/C]	1067298741
Ca_Kabuli_NC_021164.1_45704265	Ca_kabuli_chr05	45704265	[A/G]	1067298742
Ca_Kabuli_NC_021164.1_45704281	Ca_kabuli_chr05	45704281	[A/G]	1067298743
Ca_Kabuli_NC_021164.1_45710509	Ca_kabuli_chr05	45710509	[A/G]	1067298744
Ca_Kabuli_NC_021164.1_45710519	Ca_kabuli_chr05	45710519	[A/G]	1067298745
Ca_Kabuli_NC_021164.1_45731608	Ca_kabuli_chr05	45731608	[T/G]	1067298746
Ca_Kabuli_NC_021164.1_45731819	Ca_kabuli_chr05	45731819	[A/C]	1067298747
Ca_Kabuli_NC_021164.1_45732662	Ca_kabuli_chr05	45732662	[A/G]	1067298748
Ca_Kabuli_NC_021164.1_45841033	Ca_kabuli_chr05	45841033	[T/G]	1067298749
Ca_Kabuli_NC_021164.1_45841041	Ca_kabuli_chr05	45841041	[T/C]	1067298750
Ca_Kabuli_NC_021164.1_45841046	Ca_kabuli_chr05	45841046	[T/G]	1067298751
Ca_Kabuli_NC_021164.1_45841050	Ca_kabuli_chr05	45841050	[T/C]	1067298752
Ca_Kabuli_NC_021164.1_45848012	Ca_kabuli_chr05	45848012	[T/A]	1067298753
Ca_Kabuli_NC_021164.1_45924025	Ca_kabuli_chr05	45924025	[A/T]	1067298754
Ca_Kabuli_NC_021164.1_45924039	Ca_kabuli_chr05	45924039	[T/C]	1067298755
Ca_Kabuli_NC_021164.1_45924056	Ca_kabuli_chr05	45924056	[A/G]	1067298756
Ca_Kabuli_NC_021164.1_45952828	Ca_kabuli_chr05	45952828	[T/G]	1067298757
Ca_Kabuli_NC_021164.1_45952833	Ca_kabuli_chr05	45952833	[T/G]	1067298758
Ca_Kabuli_NC_021164.1_45994690	Ca_kabuli_chr05	45994690	[A/T]	1067298759
Ca_Kabuli_NC_021164.1_45994731	Ca_kabuli_chr05	45994731	[A/G]	1067298760
Ca_Kabuli_NC_021164.1_46019035	Ca_kabuli_chr05	46019035	[T/G]	1067298761
Ca_Kabuli_NC_021164.1_46019049	Ca_kabuli_chr05	46019049	[A/G]	1067298762

SNP IDs	Chromosomes	Physical positions (bp)	SNPs	NCBI SNPdb accession IDs (NCBI_ss#)
Ca_Kabuli_NC_021164.1_46077325	Ca_kabuli_chr05	46077325	[G/T]	1067298763
Ca_Kabuli_NC_021164.1_46119152	Ca_kabuli_chr05	46119152	[T/C]	1067298764
Ca_Kabuli_NC_021164.1_46130217	Ca_kabuli_chr05	46130217	[T/G]	1067298765
Ca_Kabuli_NC_021164.1_46156202	Ca_kabuli_chr05	46156202	[T/C]	1067298766
Ca_Kabuli_NC_021164.1_46156815	Ca_kabuli_chr05	46156815	[A/T]	1067298767
Ca_Kabuli_NC_021164.1_46156816	Ca_kabuli_chr05	46156816	[G/C]	1067298768
Ca_Kabuli_NC_021164.1_46156817	Ca_kabuli_chr05	46156817	[G/T]	1067298769
Ca_Kabuli_NC_021164.1_46196699	Ca_kabuli_chr05	46196699	[A/C]	1067298770
Ca_Kabuli_NC_021164.1_46225026	Ca_kabuli_chr05	46225026	[G/T]	1067298771
Ca_Kabuli_NC_021164.1_46260786	Ca_kabuli_chr05	46260786	[T/G]	1067298772
Ca_Kabuli_NC_021164.1_46260798	Ca_kabuli_chr05	46260798	[T/G]	1067298773
Ca_Kabuli_NC_021164.1_46260810	Ca_kabuli_chr05	46260810	[T/A]	1067298774
Ca_Kabuli_NC_021164.1_46260811	Ca_kabuli_chr05	46260811	[A/C]	1067298775
Ca_Kabuli_NC_021164.1_46260817	Ca_kabuli_chr05	46260817	[A/C]	1067298776
Ca_Kabuli_NC_021164.1_46260828	Ca_kabuli_chr05	46260828	[A/C]	1067298777
Ca_Kabuli_NC_021164.1_46260846	Ca_kabuli_chr05	46260846	[A/C]	1067298778
Ca_Kabuli_NC_021164.1_46263316	Ca_kabuli_chr05	46263316	[T/G]	1067298779
Ca_Kabuli_NC_021164.1_46263320	Ca_kabuli_chr05	46263320	[T/G]	1067298780
Ca_Kabuli_NC_021164.1_46272277	Ca_kabuli_chr05	46272277	[A/T]	1067298781
Ca_Kabuli_NC_021164.1_46272308	Ca_kabuli_chr05	46272308	[A/G]	1067298782
Ca_Kabuli_NC_021164.1_46301639	Ca_kabuli_chr05	46301639	[G/A]	1067298783
Ca_Kabuli_NC_021164.1_46310743	Ca_kabuli_chr05	46310743	[A/C]	1067298784
Ca_Kabuli_NC_021164.1_46328623	Ca_kabuli_chr05	46328623	[A/G]	1067298785
Ca_Kabuli_NC_021164.1_46328640	Ca_kabuli_chr05	46328640	[A/T]	1067298786
Ca_Kabuli_NC_021164.1_46344104	Ca_kabuli_chr05	46344104	[G/A]	1067298787
Ca_Kabuli_NC_021164.1_46491556	Ca_kabuli_chr05	46491556	[T/G]	1067298788
Ca_Kabuli_NC_021164.1_46513842	Ca_kabuli_chr05	46513842	[A/C]	1067298789
Ca_Kabuli_NC_021164.1_46535489	Ca_kabuli_chr05	46535489	[T/C]	1067298790
Ca_Kabuli_NC_021164.1_46535552	Ca_kabuli_chr05	46535552	[A/C]	1067298791
Ca_Kabuli_NC_021164.1_46535559	Ca_kabuli_chr05	46535559	[A/G]	1067298792
Ca_Kabuli_NC_021164.1_46592120	Ca_kabuli_chr05	46592120	[A/G]	1067298793
Ca_Kabuli_NC_021164.1_46592129	Ca_kabuli_chr05	46592129	[A/G]	1067298794
Ca_Kabuli_NC_021164.1_46607686	Ca_kabuli_chr05	46607686	[T/A]	1067298795
Ca_Kabuli_NC_021164.1_46611196	Ca_kabuli_chr05	46611196	[G/T]	1067298796

SNP IDs	Chromosomes	Physical positions (bp)	SNPs	NCBI SNPdb accession IDs (NCBI_ss#)
Ca_Kabuli_NC_021164.1_46611317	Ca_kabuli_chr05	46611317	[T/G]	1067298797
Ca_Kabuli_NC_021164.1_46611338	Ca_kabuli_chr05	46611338	[A/G]	1067298798
Ca_Kabuli_NC_021164.1_46621403	Ca_kabuli_chr05	46621403	[T/C]	1067298799
Ca_Kabuli_NC_021164.1_46703151	Ca_kabuli_chr05	46703151	[T/A]	1067298800
Ca_Kabuli_NC_021164.1_46703154	Ca_kabuli_chr05	46703154	[C/T]	1067298801
Ca_Kabuli_NC_021164.1_46703157	Ca_kabuli_chr05	46703157	[C/T]	1067298802
Ca_Kabuli_NC_021164.1_46723685	Ca_kabuli_chr05	46723685	[A/C]	1067298803
Ca_Kabuli_NC_021164.1_46771485	Ca_kabuli_chr05	46771485	[T/G]	1067298804
Ca_Kabuli_NC_021164.1_46792245	Ca_kabuli_chr05	46792245	[T/C]	1067298805
Ca_Kabuli_NC_021164.1_46799540	Ca_kabuli_chr05	46799540	[A/T]	1067298806
Ca_Kabuli_NC_021164.1_46799553	Ca_kabuli_chr05	46799553	[T/C]	1067298807
Ca_Kabuli_NC_021164.1_46799573	Ca_kabuli_chr05	46799573	[T/C]	1067298808
Ca_Kabuli_NC_021164.1_46799574	Ca_kabuli_chr05	46799574	[C/T]	1067298809
Ca_Kabuli_NC_021164.1_46799578	Ca_kabuli_chr05	46799578	[T/C]	1067298810
Ca_Kabuli_NC_021164.1_46799580	Ca_kabuli_chr05	46799580	[C/T]	1067298811
Ca_Kabuli_NC_021164.1_46945902	Ca_kabuli_chr05	46945902	[T/C]	1067298812
Ca_Kabuli_NC_021164.1_46962537	Ca_kabuli_chr05	46962537	[A/T]	1067298813
Ca_Kabuli_NC_021164.1_47060326	Ca_kabuli_chr05	47060326	[T/C]	1067298814
Ca_Kabuli_NC_021164.1_47060342	Ca_kabuli_chr05	47060342	[A/G]	1067298815
Ca_Kabuli_NC_021164.1_47060378	Ca_kabuli_chr05	47060378	[A/G]	1067298816
Ca_Kabuli_NC_021164.1_47061536	Ca_kabuli_chr05	47061536	[A/G]	1067298817
Ca_Kabuli_NC_021164.1_47068191	Ca_kabuli_chr05	47068191	[A/C]	1067298818
Ca_Kabuli_NC_021164.1_47068192	Ca_kabuli_chr05	47068192	[G/T]	1067298819
Ca_Kabuli_NC_021164.1_47068193	Ca_kabuli_chr05	47068193	[A/C]	1067298820
Ca_Kabuli_NC_021164.1_47068194	Ca_kabuli_chr05	47068194	[T/C]	1067298821
Ca_Kabuli_NC_021164.1_47073047	Ca_kabuli_chr05	47073047	[G/A]	1067298822
Ca_Kabuli_NC_021164.1_47073048	Ca_kabuli_chr05	47073048	[A/T]	1067298823
Ca_Kabuli_NC_021164.1_47073049	Ca_kabuli_chr05	47073049	[A/C]	1067298824
Ca_Kabuli_NC_021164.1_47073050	Ca_kabuli_chr05	47073050	[G/T]	1067298825
Ca_Kabuli_NC_021164.1_47091545	Ca_kabuli_chr05	47091545	[T/C]	1067298826
Ca_Kabuli_NC_021164.1_47165304	Ca_kabuli_chr05	47165304	[A/G]	1067298827
Ca_Kabuli_NC_021164.1_47165317	Ca_kabuli_chr05	47165317	[C/G]	1067298828
Ca_Kabuli_NC_021164.1_47165332	Ca_kabuli_chr05	47165332	[A/G]	1067298829
Ca_Kabuli_NC_021164.1_47165339	Ca_kabuli_chr05	47165339	[A/G]	1067298830

SNP IDs	Chromosomes	Physical positions (bp)	SNPs	NCBI SNPdb accession IDs (NCBI_ss#)
Ca_Kabuli_NC_021164.1_47165343	Ca_kabuli_chr05	47165343	[A/G]	1067298831
Ca_Kabuli_NC_021164.1_47207516	Ca_kabuli_chr05	47207516	[T/G]	1067298832
Ca_Kabuli_NC_021164.1_47211543	Ca_kabuli_chr05	47211543	[T/G]	1067298833
Ca_Kabuli_NC_021164.1_47211554	Ca_kabuli_chr05	47211554	[T/C]	1067298834
Ca_Kabuli_NC_021164.1_47223720	Ca_kabuli_chr05	47223720	[A/T]	1067298835
Ca_Kabuli_NC_021164.1_47286134	Ca_kabuli_chr05	47286134	[A/G]	1067298836
Ca_Kabuli_NC_021164.1_47286181	Ca_kabuli_chr05	47286181	[T/A]	1067298837
Ca_Kabuli_NC_021164.1_47329851	Ca_kabuli_chr05	47329851	[A/G]	1067298838
Ca_Kabuli_NC_021164.1_47329856	Ca_kabuli_chr05	47329856	[T/C]	1067298839
Ca_Kabuli_NC_021164.1_47357594	Ca_kabuli_chr05	47357594	[A/C]	1067298840
Ca_Kabuli_NC_021164.1_47364477	Ca_kabuli_chr05	47364477	[A/T]	1067298841
Ca_Kabuli_NC_021164.1_47364485	Ca_kabuli_chr05	47364485	[T/C]	1067298842
Ca_Kabuli_NC_021164.1_47364489	Ca_kabuli_chr05	47364489	[T/C]	1067298843
Ca_Kabuli_NC_021164.1_47422568	Ca_kabuli_chr05	47422568	[T/G]	1067298844
Ca_Kabuli_NC_021164.1_47534245	Ca_kabuli_chr05	47534245	[T/G]	1067298845
Ca_Kabuli_NC_021164.1_47534262	Ca_kabuli_chr05	47534262	[T/C]	1067298846
Ca_Kabuli_NC_021164.1_47641432	Ca_kabuli_chr05	47641432	[A/G]	1067298847
Ca_Kabuli_NC_021164.1_47641456	Ca_kabuli_chr05	47641456	[T/G]	1067298848
Ca_Kabuli_NC_021164.1_47669142	Ca_kabuli_chr05	47669142	[T/G]	1067298849
Ca_Kabuli_NC_021164.1_47669152	Ca_kabuli_chr05	47669152	[A/G]	1067298850
Ca_Kabuli_NC_021164.1_47669158	Ca_kabuli_chr05	47669158	[T/A]	1067298851
Ca_Kabuli_NC_021164.1_47669173	Ca_kabuli_chr05	47669173	[T/A]	1067298852
Ca_Kabuli_NC_021164.1_47669226	Ca_kabuli_chr05	47669226	[A/C]	1067298853
Ca_Kabuli_NC_021164.1_47669247	Ca_kabuli_chr05	47669247	[A/G]	1067298854
Ca_Kabuli_NC_021164.1_47702984	Ca_kabuli_chr05	47702984	[T/G]	1067298855
Ca_Kabuli_NC_021164.1_47811985	Ca_kabuli_chr05	47811985	[T/G]	1067298856
Ca_Kabuli_NC_021164.1_47829224	Ca_kabuli_chr05	47829224	[T/C]	1067298857
Ca_Kabuli_NC_021164.1_47877158	Ca_kabuli_chr05	47877158	[A/C]	1067298858
Ca_Kabuli_NC_021164.1_47881979	Ca_kabuli_chr05	47881979	[A/C]	1067298859
Ca_Kabuli_NC_021164.1_47901038	Ca_kabuli_chr05	47901038	[A/C]	1067298860
Ca_Kabuli_NC_021164.1_47931558	Ca_kabuli_chr05	47931558	[A/C]	1067298861
Ca_Kabuli_NC_021164.1_47931564	Ca_kabuli_chr05	47931564	[A/C]	1067298862
Ca_Kabuli_NC_021164.1_47931577	Ca_kabuli_chr05	47931577	[A/G]	1067298863
Ca_Kabuli_NC_021164.1_47941753	Ca_kabuli_chr05	47941753	[T/G]	1067298864

SNP IDs	Chromosomes	Physical positions (bp)	SNPs	NCBI SNPdb accession IDs (NCBI_ss#)
Ca_Kabuli_NC_021164.1_47943704	Ca_kabuli_chr05	47943704	[T/G]	1067298865
Ca_Kabuli_NC_021164.1_47951932	Ca_kabuli_chr05	47951932	[T/A]	1067298866
Ca_Kabuli_NC_021164.1_48005082	Ca_kabuli_chr05	48005082	[T/C]	1067298867
Ca_Kabuli_NC_021164.1_48005102	Ca_kabuli_chr05	48005102	[A/G]	1067298868
Ca_Kabuli_NC_021164.1_48005110	Ca_kabuli_chr05	48005110	[T/C]	1067298869
Ca_Kabuli_NC_021164.1_48019807	Ca_kabuli_chr05	48019807	[A/T]	1067298870
Ca_Kabuli_NC_021164.1_48034090	Ca_kabuli_chr05	48034090	[A/C]	1067298871
Ca_Kabuli_NC_021164.1_48042058	Ca_kabuli_chr05	48042058	[A/G]	1067298872
Ca_Kabuli_NC_021164.1_48042065	Ca_kabuli_chr05	48042065	[T/A]	1067298873
Ca_Kabuli_NC_021164.1_48042067	Ca_kabuli_chr05	48042067	[T/C]	1067298874
Ca_Kabuli_NC_021164.1_48042089	Ca_kabuli_chr05	48042089	[A/C]	1067298875
Ca_Kabuli_NC_021164.1_48046828	Ca_kabuli_chr05	48046828	[A/G]	1067298876
Ca_Kabuli_NC_021164.1_48046847	Ca_kabuli_chr05	48046847	[A/G]	1067298877
Ca_Kabuli_NC_021164.1_48046855	Ca_kabuli_chr05	48046855	[A/G]	1067298878
Ca_Kabuli_NC_021164.1_48058414	Ca_kabuli_chr05	48058414	[T/G]	1067298879
Ca_Kabuli_NC_021164.1_48058423	Ca_kabuli_chr05	48058423	[T/C]	1067298880
Ca_Kabuli_NC_021164.1_48069859	Ca_kabuli_chr05	48069859	[A/G]	1067298881
Ca_Kabuli_NC_021164.1_48111894	Ca_kabuli_chr05	48111894	[G/T]	1067298882
Ca_Kabuli_NC_021164.1_48111896	Ca_kabuli_chr05	48111896	[T/G]	1067298883
Ca_Kabuli_NC_021164.1_48111922	Ca_kabuli_chr05	48111922	[T/C]	1067298884
Ca_Kabuli_NC_021164.1_48111930	Ca_kabuli_chr05	48111930	[A/G]	1067298885
Ca_Kabuli_NC_021164.1_48112820	Ca_kabuli_chr05	48112820	[T/G]	1067298886
Ca_Kabuli_NC_021164.1_48113397	Ca_kabuli_chr05	48113397	[A/T]	1067298887
Ca_Kabuli_NC_021164.1_48113417	Ca_kabuli_chr05	48113417	[A/C]	1067298888
Ca_Kabuli_NC_021164.1_48115101	Ca_kabuli_chr05	48115101	[T/A]	1067298889
Ca_Kabuli_NC_021164.1_48115165	Ca_kabuli_chr05	48115165	[A/G]	1067298890
Ca_Kabuli_NC_021164.1_48128396	Ca_kabuli_chr05	48128396	[T/G]	1067298891
Ca_Kabuli_NC_021165.1_152519	Ca_kabuli_chr06	152519	[T/G]	1067298892
Ca_Kabuli_NC_021165.1_152520	Ca_kabuli_chr06	152520	[G/C]	1067298893
Ca_Kabuli_NC_021165.1_152529	Ca_kabuli_chr06	152529	[T/C]	1067298894
Ca_Kabuli_NC_021165.1_158938	Ca_kabuli_chr06	158938	[T/A]	1067298895
Ca_Kabuli_NC_021165.1_210513	Ca_kabuli_chr06	210513	[T/C]	1067298896
Ca_Kabuli_NC_021165.1_210527	Ca_kabuli_chr06	210527	[T/A]	1067298897
Ca_Kabuli_NC_021165.1_269296	Ca_kabuli_chr06	269296	[A/G]	1067298898

SNP IDs	Chromosomes	Physical positions (bp)	SNPs	NCBI SNPdb accession IDs (NCBI_ss#)
Ca_Kabuli_NC_021165.1_277061	Ca_kabuli_chr06	277061	[A/T]	1067298899
Ca_Kabuli_NC_021165.1_486952	Ca_kabuli_chr06	486952	[C/A]	1067298900
Ca_Kabuli_NC_021165.1_504936	Ca_kabuli_chr06	504936	[A/G]	1067298901
Ca_Kabuli_NC_021165.1_580515	Ca_kabuli_chr06	580515	[T/C]	1067298902
Ca_Kabuli_NC_021165.1_580521	Ca_kabuli_chr06	580521	[A/G]	1067298903
Ca_Kabuli_NC_021165.1_587169	Ca_kabuli_chr06	587169	[T/G]	1067298904
Ca_Kabuli_NC_021165.1_587221	Ca_kabuli_chr06	587221	[A/C]	1067298905
Ca_Kabuli_NC_021165.1_587235	Ca_kabuli_chr06	587235	[T/A]	1067298906
Ca_Kabuli_NC_021165.1_587236	Ca_kabuli_chr06	587236	[A/T]	1067298907
Ca_Kabuli_NC_021165.1_587251	Ca_kabuli_chr06	587251	[A/G]	1067298908
Ca_Kabuli_NC_021165.1_587256	Ca_kabuli_chr06	587256	[T/C]	1067298909
Ca_Kabuli_NC_021165.1_611246	Ca_kabuli_chr06	611246	[T/C]	1067298910
Ca_Kabuli_NC_021165.1_611280	Ca_kabuli_chr06	611280	[T/C]	1067298911
Ca_Kabuli_NC_021165.1_611293	Ca_kabuli_chr06	611293	[A/C]	1067298912
Ca_Kabuli_NC_021165.1_611300	Ca_kabuli_chr06	611300	[T/C]	1067298913
Ca_Kabuli_NC_021165.1_638961	Ca_kabuli_chr06	638961	[T/G]	1067298914
Ca_Kabuli_NC_021165.1_647270	Ca_kabuli_chr06	647270	[T/A]	1067298915
Ca_Kabuli_NC_021165.1_647322	Ca_kabuli_chr06	647322	[A/C]	1067298916
Ca_Kabuli_NC_021165.1_647329	Ca_kabuli_chr06	647329	[A/C]	1067298917
Ca_Kabuli_NC_021165.1_894893	Ca_kabuli_chr06	894893	[T/C]	1067298918
Ca_Kabuli_NC_021165.1_902445	Ca_kabuli_chr06	902445	[A/G]	1067298919
Ca_Kabuli_NC_021165.1_902495	Ca_kabuli_chr06	902495	[A/T]	1067298920
Ca_Kabuli_NC_021165.1_915496	Ca_kabuli_chr06	915496	[A/C]	1067298921
Ca_Kabuli_NC_021165.1_998581	Ca_kabuli_chr06	998581	[T/C]	1067298922
Ca_Kabuli_NC_021165.1_998582	Ca_kabuli_chr06	998582	[C/G]	1067298923
Ca_Kabuli_NC_021165.1_998583	Ca_kabuli_chr06	998583	[T/G]	1067298924
Ca_Kabuli_NC_021165.1_1005273	Ca_kabuli_chr06	1005273	[C/G]	1067298925
Ca_Kabuli_NC_021165.1_1116951	Ca_kabuli_chr06	1116951	[T/G]	1067298926
Ca_Kabuli_NC_021165.1_1117010	Ca_kabuli_chr06	1117010	[A/C]	1067298927
Ca_Kabuli_NC_021165.1_1130098	Ca_kabuli_chr06	1130098	[A/G]	1067298928
Ca_Kabuli_NC_021165.1_1181733	Ca_kabuli_chr06	1181733	[A/C]	1067298929
Ca_Kabuli_NC_021165.1_1181739	Ca_kabuli_chr06	1181739	[A/G]	1067298930
Ca_Kabuli_NC_021165.1_1193776	Ca_kabuli_chr06	1193776	[T/G]	1067298931
Ca_Kabuli_NC_021165.1_1212875	Ca_kabuli_chr06	1212875	[T/G]	1067298932

SNP IDs	Chromosomes	Physical positions (bp)	SNPs	NCBI SNPdb accession IDs (NCBI_ss#)
Ca_Kabuli_NC_021165.1_1214374	Ca_kabuli_chr06	1214374	[C/T]	1067298933
Ca_Kabuli_NC_021165.1_1220523	Ca_kabuli_chr06	1220523	[C/T]	1067298934
Ca_Kabuli_NC_021165.1_1236070	Ca_kabuli_chr06	1236070	[T/C]	1067298935
Ca_Kabuli_NC_021165.1_1236107	Ca_kabuli_chr06	1236107	[T/C]	1067298936
Ca_Kabuli_NC_021165.1_1236129	Ca_kabuli_chr06	1236129	[A/G]	1067298937
Ca_Kabuli_NC_021165.1_1236136	Ca_kabuli_chr06	1236136	[A/C]	1067298938
Ca_Kabuli_NC_021165.1_1274668	Ca_kabuli_chr06	1274668	[A/G]	1067298939
Ca_Kabuli_NC_021165.1_1276388	Ca_kabuli_chr06	1276388	[A/T]	1067298940
Ca_Kabuli_NC_021165.1_1362497	Ca_kabuli_chr06	1362497	[G/A]	1067298941
Ca_Kabuli_NC_021165.1_1362520	Ca_kabuli_chr06	1362520	[T/A]	1067298942
Ca_Kabuli_NC_021165.1_1362535	Ca_kabuli_chr06	1362535	[A/G]	1067298943
Ca_Kabuli_NC_021165.1_1643856	Ca_kabuli_chr06	1643856	[T/G]	1067298944
Ca_Kabuli_NC_021165.1_1643874	Ca_kabuli_chr06	1643874	[A/G]	1067298945
Ca_Kabuli_NC_021165.1_1811162	Ca_kabuli_chr06	1811162	[G/A]	1067298946
Ca_Kabuli_NC_021165.1_1811671	Ca_kabuli_chr06	1811671	[A/C]	1067298947
Ca_Kabuli_NC_021165.1_1815245	Ca_kabuli_chr06	1815245	[C/T]	1067298948
Ca_Kabuli_NC_021165.1_1815278	Ca_kabuli_chr06	1815278	[A/G]	1067298949
Ca_Kabuli_NC_021165.1_1817599	Ca_kabuli_chr06	1817599	[C/T]	1067298950
Ca_Kabuli_NC_021165.1_1817774	Ca_kabuli_chr06	1817774	[A/G]	1067298951
Ca_Kabuli_NC_021165.1_1817775	Ca_kabuli_chr06	1817775	[G/A]	1067298952
Ca_Kabuli_NC_021165.1_1817776	Ca_kabuli_chr06	1817776	[A/G]	1067298953
Ca_Kabuli_NC_021165.1_1817786	Ca_kabuli_chr06	1817786	[A/G]	1067298954
Ca_Kabuli_NC_021165.1_1818234	Ca_kabuli_chr06	1818234	[C/T]	1067298955
Ca_Kabuli_NC_021165.1_1843341	Ca_kabuli_chr06	1843341	[G/A]	1067298956
Ca_Kabuli_NC_021165.1_1843376	Ca_kabuli_chr06	1843376	[C/T]	1067298957
Ca_Kabuli_NC_021165.1_1867519	Ca_kabuli_chr06	1867519	[A/T]	1067298958
Ca_Kabuli_NC_021165.1_1867530	Ca_kabuli_chr06	1867530	[A/T]	1067298959
Ca_Kabuli_NC_021165.1_1979887	Ca_kabuli_chr06	1979887	[G/T]	1067298960
Ca_Kabuli_NC_021165.1_2001887	Ca_kabuli_chr06	2001887	[T/C]	1067298961
Ca_Kabuli_NC_021165.1_2001898	Ca_kabuli_chr06	2001898	[C/T]	1067298962
Ca_Kabuli_NC_021165.1_2001909	Ca_kabuli_chr06	2001909	[T/G]	1067298963
Ca_Kabuli_NC_021165.1_2001921	Ca_kabuli_chr06	2001921	[A/G]	1067298964
Ca_Kabuli_NC_021165.1_2001925	Ca_kabuli_chr06	2001925	[A/G]	1067298965
Ca_Kabuli_NC_021165.1_2001933	Ca_kabuli_chr06	2001933	[T/G]	1067298966

SNP IDs	Chromosomes	Physical positions (bp)	SNPs	NCBI SNPdb accession IDs (NCBI_ss#)
Ca_Kabuli_NC_021165.1_2001938	Ca_kabuli_chr06	2001938	[C/T]	1067298967
Ca_Kabuli_NC_021165.1_2037620	Ca_kabuli_chr06	2037620	[T/G]	1067298968
Ca_Kabuli_NC_021165.1_2037623	Ca_kabuli_chr06	2037623	[T/G]	1067298969
Ca_Kabuli_NC_021165.1_2079137	Ca_kabuli_chr06	2079137	[T/G]	1067298970
Ca_Kabuli_NC_021165.1_2140393	Ca_kabuli_chr06	2140393	[A/T]	1067298971
Ca_Kabuli_NC_021165.1_2140399	Ca_kabuli_chr06	2140399	[T/C]	1067298972
Ca_Kabuli_NC_021165.1_2140403	Ca_kabuli_chr06	2140403	[A/G]	1067298973
Ca_Kabuli_NC_021165.1_2156740	Ca_kabuli_chr06	2156740	[T/G]	1067298974
Ca_Kabuli_NC_021165.1_2171761	Ca_kabuli_chr06	2171761	[T/G]	1067298975
Ca_Kabuli_NC_021165.1_2172210	Ca_kabuli_chr06	2172210	[T/G]	1067298976
Ca_Kabuli_NC_021165.1_2172258	Ca_kabuli_chr06	2172258	[A/G]	1067298977
Ca_Kabuli_NC_021165.1_2175751	Ca_kabuli_chr06	2175751	[T/G]	1067298978
Ca_Kabuli_NC_021165.1_2175766	Ca_kabuli_chr06	2175766	[A/C]	1067298979
Ca_Kabuli_NC_021165.1_2175768	Ca_kabuli_chr06	2175768	[A/C]	1067298980
Ca_Kabuli_NC_021165.1_2244901	Ca_kabuli_chr06	2244901	[A/C]	1067298981
Ca_Kabuli_NC_021165.1_2286209	Ca_kabuli_chr06	2286209	[A/T]	1067298982
Ca_Kabuli_NC_021165.1_2286237	Ca_kabuli_chr06	2286237	[C/T]	1067298983
Ca_Kabuli_NC_021165.1_2299629	Ca_kabuli_chr06	2299629	[A/C]	1067298984
Ca_Kabuli_NC_021165.1_2326797	Ca_kabuli_chr06	2326797	[A/G]	1067298985
Ca_Kabuli_NC_021165.1_2326809	Ca_kabuli_chr06	2326809	[T/A]	1067298986
Ca_Kabuli_NC_021165.1_2327832	Ca_kabuli_chr06	2327832	[A/G]	1067298987
Ca_Kabuli_NC_021165.1_2408338	Ca_kabuli_chr06	2408338	[A/G]	1067298988
Ca_Kabuli_NC_021165.1_2414990	Ca_kabuli_chr06	2414990	[T/C]	1067298989
Ca_Kabuli_NC_021165.1_2448861	Ca_kabuli_chr06	2448861	[A/G]	1067298990
Ca_Kabuli_NC_021165.1_2467454	Ca_kabuli_chr06	2467454	[A/G]	1067298991
Ca_Kabuli_NC_021165.1_2484322	Ca_kabuli_chr06	2484322	[T/A]	1067298992
Ca_Kabuli_NC_021165.1_2484342	Ca_kabuli_chr06	2484342	[A/C]	1067298993
Ca_Kabuli_NC_021165.1_2527768	Ca_kabuli_chr06	2527768	[A/G]	1067298994
Ca_Kabuli_NC_021165.1_2540711	Ca_kabuli_chr06	2540711	[C/T]	1067298995
Ca_Kabuli_NC_021165.1_2540749	Ca_kabuli_chr06	2540749	[C/T]	1067298996
Ca_Kabuli_NC_021165.1_2541088	Ca_kabuli_chr06	2541088	[T/A]	1067298997
Ca_Kabuli_NC_021165.1_2542942	Ca_kabuli_chr06	2542942	[T/A]	1067298998
Ca_Kabuli_NC_021165.1_2542981	Ca_kabuli_chr06	2542981	[C/T]	1067298999
Ca_Kabuli_NC_021165.1_2543096	Ca_kabuli_chr06	2543096	[C/T]	1067299000

SNP IDs	Chromosomes	Physical positions (bp)	SNPs	NCBI SNPdb accession IDs (NCBI_ss#)
Ca_Kabuli_NC_021165.1_2550007	Ca_kabuli_chr06	2550007	[A/C]	1067299001
Ca_Kabuli_NC_021165.1_2550022	Ca_kabuli_chr06	2550022	[A/C]	1067299002
Ca_Kabuli_NC_021165.1_2550101	Ca_kabuli_chr06	2550101	[T/G]	1067299003
Ca_Kabuli_NC_021165.1_2592593	Ca_kabuli_chr06	2592593	[G/A]	1067299004
Ca_Kabuli_NC_021165.1_2592607	Ca_kabuli_chr06	2592607	[T/G]	1067299005
Ca_Kabuli_NC_021165.1_2592625	Ca_kabuli_chr06	2592625	[T/A]	1067299006
Ca_Kabuli_NC_021165.1_2604913	Ca_kabuli_chr06	2604913	[T/G]	1067299007
Ca_Kabuli_NC_021165.1_2604933	Ca_kabuli_chr06	2604933	[A/G]	1067299008
Ca_Kabuli_NC_021165.1_2623360	Ca_kabuli_chr06	2623360	[T/C]	1067299009
Ca_Kabuli_NC_021165.1_2636832	Ca_kabuli_chr06	2636832	[T/G]	1067299010
Ca_Kabuli_NC_021165.1_2636837	Ca_kabuli_chr06	2636837	[A/G]	1067299011
Ca_Kabuli_NC_021165.1_2676658	Ca_kabuli_chr06	2676658	[A/C]	1067299012
Ca_Kabuli_NC_021165.1_2715014	Ca_kabuli_chr06	2715014	[T/A]	1067299013
Ca_Kabuli_NC_021165.1_2719217	Ca_kabuli_chr06	2719217	[T/A]	1067299014
Ca_Kabuli_NC_021165.1_2740777	Ca_kabuli_chr06	2740777	[A/T]	1067299015
Ca_Kabuli_NC_021165.1_2740885	Ca_kabuli_chr06	2740885	[A/C]	1067299016
Ca_Kabuli_NC_021165.1_2802938	Ca_kabuli_chr06	2802938	[A/G]	1067299017
Ca_Kabuli_NC_021165.1_2802940	Ca_kabuli_chr06	2802940	[G/C]	1067299018
Ca_Kabuli_NC_021165.1_2802958	Ca_kabuli_chr06	2802958	[T/C]	1067299019
Ca_Kabuli_NC_021165.1_2802984	Ca_kabuli_chr06	2802984	[A/C]	1067299020
Ca_Kabuli_NC_021165.1_2802996	Ca_kabuli_chr06	2802996	[A/G]	1067299021
Ca_Kabuli_NC_021165.1_2812736	Ca_kabuli_chr06	2812736	[A/G]	1067299022
Ca_Kabuli_NC_021165.1_2812773	Ca_kabuli_chr06	2812773	[T/G]	1067299023
Ca_Kabuli_NC_021165.1_2812776	Ca_kabuli_chr06	2812776	[T/C]	1067299024
Ca_Kabuli_NC_021165.1_2812792	Ca_kabuli_chr06	2812792	[A/G]	1067299025
Ca_Kabuli_NC_021165.1_2823107	Ca_kabuli_chr06	2823107	[G/T]	1067299026
Ca_Kabuli_NC_021165.1_2823119	Ca_kabuli_chr06	2823119	[C/T]	1067299027
Ca_Kabuli_NC_021165.1_2927253	Ca_kabuli_chr06	2927253	[A/C]	1067299028
Ca_Kabuli_NC_021165.1_2927265	Ca_kabuli_chr06	2927265	[T/C]	1067299029
Ca_Kabuli_NC_021165.1_2927328	Ca_kabuli_chr06	2927328	[T/G]	1067299030
Ca_Kabuli_NC_021165.1_2927361	Ca_kabuli_chr06	2927361	[T/G]	1067299031
Ca_Kabuli_NC_021165.1_2927391	Ca_kabuli_chr06	2927391	[A/G]	1067299032
Ca_Kabuli_NC_021165.1_2929405	Ca_kabuli_chr06	2929405	[A/C]	1067299033
Ca_Kabuli_NC_021165.1_2930419	Ca_kabuli_chr06	2930419	[T/C]	1067299034

SNP IDs	Chromosomes	Physical positions (bp)	SNPs	NCBI SNPdb accession IDs (NCBI_ss#)
Ca_Kabuli_NC_021165.1_2930460	Ca_kabuli_chr06	2930460	[A/C]	1067299035
Ca_Kabuli_NC_021165.1_2930493	Ca_kabuli_chr06	2930493	[A/G]	1067299036
Ca_Kabuli_NC_021165.1_2930510	Ca_kabuli_chr06	2930510	[A/C]	1067299037
Ca_Kabuli_NC_021165.1_2930542	Ca_kabuli_chr06	2930542	[A/C]	1067299038
Ca_Kabuli_NC_021165.1_2931289	Ca_kabuli_chr06	2931289	[T/G]	1067299039
Ca_Kabuli_NC_021165.1_2931314	Ca_kabuli_chr06	2931314	[T/C]	1067299040
Ca_Kabuli_NC_021165.1_2931329	Ca_kabuli_chr06	2931329	[A/G]	1067299041
Ca_Kabuli_NC_021165.1_2973756	Ca_kabuli_chr06	2973756	[A/C]	1067299042
Ca_Kabuli_NC_021165.1_2973759	Ca_kabuli_chr06	2973759	[T/C]	1067299043
Ca_Kabuli_NC_021165.1_3213956	Ca_kabuli_chr06	3213956	[T/G]	1067299044
Ca_Kabuli_NC_021165.1_3213966	Ca_kabuli_chr06	3213966	[T/G]	1067299045
Ca_Kabuli_NC_021165.1_3213987	Ca_kabuli_chr06	3213987	[T/C]	1067299046
Ca_Kabuli_NC_021165.1_3273804	Ca_kabuli_chr06	3273804	[T/C]	1067299047
Ca_Kabuli_NC_021165.1_3273842	Ca_kabuli_chr06	3273842	[T/G]	1067299048
Ca_Kabuli_NC_021165.1_3276789	Ca_kabuli_chr06	3276789	[A/T]	1067299049
Ca_Kabuli_NC_021165.1_3280601	Ca_kabuli_chr06	3280601	[A/T]	1067299050
Ca_Kabuli_NC_021165.1_3288447	Ca_kabuli_chr06	3288447	[G/T]	1067299051
Ca_Kabuli_NC_021165.1_3288458	Ca_kabuli_chr06	3288458	[A/C]	1067299052
Ca_Kabuli_NC_021165.1_3288466	Ca_kabuli_chr06	3288466	[C/A]	1067299053
Ca_Kabuli_NC_021165.1_3288468	Ca_kabuli_chr06	3288468	[T/C]	1067299054
Ca_Kabuli_NC_021165.1_3288474	Ca_kabuli_chr06	3288474	[T/C]	1067299055
Ca_Kabuli_NC_021165.1_3288483	Ca_kabuli_chr06	3288483	[T/C]	1067299056
Ca_Kabuli_NC_021165.1_3288487	Ca_kabuli_chr06	3288487	[T/C]	1067299057
Ca_Kabuli_NC_021165.1_3288489	Ca_kabuli_chr06	3288489	[G/C]	1067299058
Ca_Kabuli_NC_021165.1_3288496	Ca_kabuli_chr06	3288496	[G/A]	1067299059
Ca_Kabuli_NC_021165.1_3321597	Ca_kabuli_chr06	3321597	[T/A]	1067299060
Ca_Kabuli_NC_021165.1_3321738	Ca_kabuli_chr06	3321738	[A/C]	1067299061
Ca_Kabuli_NC_021165.1_3394747	Ca_kabuli_chr06	3394747	[T/C]	1067299062
Ca_Kabuli_NC_021165.1_3394774	Ca_kabuli_chr06	3394774	[C/T]	1067299063
Ca_Kabuli_NC_021165.1_3394784	Ca_kabuli_chr06	3394784	[C/G]	1067299064
Ca_Kabuli_NC_021165.1_3394876	Ca_kabuli_chr06	3394876	[G/A]	1067299065
Ca_Kabuli_NC_021165.1_3394898	Ca_kabuli_chr06	3394898	[T/A]	1067299066
Ca_Kabuli_NC_021165.1_3394908	Ca_kabuli_chr06	3394908	[G/T]	1067299067
Ca_Kabuli_NC_021165.1_3394921	Ca_kabuli_chr06	3394921	[A/G]	1067299068

SNP IDs	Chromosomes	Physical positions (bp)	SNPs	NCBI SNPdb accession IDs (NCBI_ss#)
Ca_Kabuli_NC_021165.1_3396791	Ca_kabuli_chr06	3396791	[T/C]	1067299069
Ca_Kabuli_NC_021165.1_3396803	Ca_kabuli_chr06	3396803	[A/C]	1067299070
Ca_Kabuli_NC_021165.1_3396810	Ca_kabuli_chr06	3396810	[A/G]	1067299071
Ca_Kabuli_NC_021165.1_3396813	Ca_kabuli_chr06	3396813	[T/C]	1067299072
Ca_Kabuli_NC_021165.1_3507742	Ca_kabuli_chr06	3507742	[A/C]	1067299073
Ca_Kabuli_NC_021165.1_3654778	Ca_kabuli_chr06	3654778	[A/G]	1067299074
Ca_Kabuli_NC_021165.1_3654789	Ca_kabuli_chr06	3654789	[A/G]	1067299075
Ca_Kabuli_NC_021165.1_3720789	Ca_kabuli_chr06	3720789	[A/G]	1067299076
Ca_Kabuli_NC_021165.1_3722816	Ca_kabuli_chr06	3722816	[A/G]	1067299077
Ca_Kabuli_NC_021165.1_3722829	Ca_kabuli_chr06	3722829	[T/C]	1067299078
Ca_Kabuli_NC_021165.1_3722840	Ca_kabuli_chr06	3722840	[T/G]	1067299079
Ca_Kabuli_NC_021165.1_3733800	Ca_kabuli_chr06	3733800	[T/G]	1067299080
Ca_Kabuli_NC_021165.1_3733808	Ca_kabuli_chr06	3733808	[A/G]	1067299081
Ca_Kabuli_NC_021165.1_3733812	Ca_kabuli_chr06	3733812	[A/C]	1067299082
Ca_Kabuli_NC_021165.1_3745388	Ca_kabuli_chr06	3745388	[A/C]	1067299083
Ca_Kabuli_NC_021165.1_3761493	Ca_kabuli_chr06	3761493	[A/G]	1067299084
Ca_Kabuli_NC_021165.1_3765131	Ca_kabuli_chr06	3765131	[T/G]	1067299085
Ca_Kabuli_NC_021165.1_3786815	Ca_kabuli_chr06	3786815	[A/T]	1067299086
Ca_Kabuli_NC_021165.1_3787086	Ca_kabuli_chr06	3787086	[T/C]	1067299087
Ca_Kabuli_NC_021165.1_3803660	Ca_kabuli_chr06	3803660	[T/C]	1067299088
Ca_Kabuli_NC_021165.1_3803672	Ca_kabuli_chr06	3803672	[A/T]	1067299089
Ca_Kabuli_NC_021165.1_3803776	Ca_kabuli_chr06	3803776	[G/T]	1067299090
Ca_Kabuli_NC_021165.1_3803788	Ca_kabuli_chr06	3803788	[A/C]	1067299091
Ca_Kabuli_NC_021165.1_3803791	Ca_kabuli_chr06	3803791	[A/G]	1067299092
Ca_Kabuli_NC_021165.1_3803817	Ca_kabuli_chr06	3803817	[T/G]	1067299093
Ca_Kabuli_NC_021165.1_3829022	Ca_kabuli_chr06	3829022	[T/G]	1067299094
Ca_Kabuli_NC_021165.1_3829043	Ca_kabuli_chr06	3829043	[A/T]	1067299095
Ca_Kabuli_NC_021165.1_3960991	Ca_kabuli_chr06	3960991	[T/G]	1067299096
Ca_Kabuli_NC_021165.1_3962394	Ca_kabuli_chr06	3962394	[T/G]	1067299097
Ca_Kabuli_NC_021165.1_3962416	Ca_kabuli_chr06	3962416	[A/C]	1067299098
Ca_Kabuli_NC_021165.1_3962420	Ca_kabuli_chr06	3962420	[A/C]	1067299099
Ca_Kabuli_NC_021165.1_3962444	Ca_kabuli_chr06	3962444	[A/C]	1067299100
Ca_Kabuli_NC_021165.1_3962488	Ca_kabuli_chr06	3962488	[A/T]	1067299101
Ca_Kabuli_NC_021165.1_3965379	Ca_kabuli_chr06	3965379	[A/G]	1067299102

SNP IDs	Chromosomes	Physical positions (bp)	SNPs	NCBI SNPdb accession IDs (NCBI_ss#)
Ca_Kabuli_NC_021165.1_3965380	Ca_kabuli_chr06	3965380	[G/T]	1067299103
Ca_Kabuli_NC_021165.1_3965381	Ca_kabuli_chr06	3965381	[A/G]	1067299104
Ca_Kabuli_NC_021165.1_3965383	Ca_kabuli_chr06	3965383	[A/G]	1067299105
Ca_Kabuli_NC_021165.1_3965384	Ca_kabuli_chr06	3965384	[T/C]	1067299106
Ca_Kabuli_NC_021165.1_3965411	Ca_kabuli_chr06	3965411	[A/G]	1067299107
Ca_Kabuli_NC_021165.1_3965412	Ca_kabuli_chr06	3965412	[T/C]	1067299108
Ca_Kabuli_NC_021165.1_3965414	Ca_kabuli_chr06	3965414	[T/C]	1067299109
Ca_Kabuli_NC_021165.1_3965416	Ca_kabuli_chr06	3965416	[A/G]	1067299110
Ca_Kabuli_NC_021165.1_3985519	Ca_kabuli_chr06	3985519	[A/G]	1067299111
Ca_Kabuli_NC_021165.1_3990963	Ca_kabuli_chr06	3990963	[A/G]	1067299112
Ca_Kabuli_NC_021165.1_3992451	Ca_kabuli_chr06	3992451	[T/G]	1067299113
Ca_Kabuli_NC_021165.1_3992500	Ca_kabuli_chr06	3992500	[A/T]	1067299114
Ca_Kabuli_NC_021165.1_4093800	Ca_kabuli_chr06	4093800	[A/G]	1067299115
Ca_Kabuli_NC_021165.1_4120753	Ca_kabuli_chr06	4120753	[T/A]	1067299116
Ca_Kabuli_NC_021165.1_4120766	Ca_kabuli_chr06	4120766	[G/A]	1067299117
Ca_Kabuli_NC_021165.1_4120769	Ca_kabuli_chr06	4120769	[T/G]	1067299118
Ca_Kabuli_NC_021165.1_4216726	Ca_kabuli_chr06	4216726	[A/T]	1067299119
Ca_Kabuli_NC_021165.1_4348586	Ca_kabuli_chr06	4348586	[T/C]	1067299120
Ca_Kabuli_NC_021165.1_4348610	Ca_kabuli_chr06	4348610	[A/C]	1067299121
Ca_Kabuli_NC_021165.1_4350822	Ca_kabuli_chr06	4350822	[A/C]	1067299122
Ca_Kabuli_NC_021165.1_4362848	Ca_kabuli_chr06	4362848	[T/C]	1067299123
Ca_Kabuli_NC_021165.1_4362850	Ca_kabuli_chr06	4362850	[A/C]	1067299124
Ca_Kabuli_NC_021165.1_4362860	Ca_kabuli_chr06	4362860	[T/C]	1067299125
Ca_Kabuli_NC_021165.1_4487606	Ca_kabuli_chr06	4487606	[T/G]	1067299126
Ca_Kabuli_NC_021165.1_4496565	Ca_kabuli_chr06	4496565	[T/G]	1067299127
Ca_Kabuli_NC_021165.1_4496725	Ca_kabuli_chr06	4496725	[T/C]	1067299128
Ca_Kabuli_NC_021165.1_4508927	Ca_kabuli_chr06	4508927	[T/C]	1067299129
Ca_Kabuli_NC_021165.1_4508962	Ca_kabuli_chr06	4508962	[T/C]	1067299130
Ca_Kabuli_NC_021165.1_4510074	Ca_kabuli_chr06	4510074	[A/T]	1067299131
Ca_Kabuli_NC_021165.1_4520531	Ca_kabuli_chr06	4520531	[A/G]	1067299132
Ca_Kabuli_NC_021165.1_4525125	Ca_kabuli_chr06	4525125	[T/C]	1067299133
Ca_Kabuli_NC_021165.1_4562151	Ca_kabuli_chr06	4562151	[T/A]	1067299134
Ca_Kabuli_NC_021165.1_4562172	Ca_kabuli_chr06	4562172	[A/G]	1067299135
Ca_Kabuli_NC_021165.1_4562174	Ca_kabuli_chr06	4562174	[A/G]	1067299136

SNP IDs	Chromosomes	Physical positions (bp)	SNPs	NCBI SNPdb accession IDs (NCBI_ss#)
Ca_Kabuli_NC_021165.1_4562175	Ca_kabuli_chr06	4562175	[G/A]	1067299137
Ca_Kabuli_NC_021165.1_4562186	Ca_kabuli_chr06	4562186	[G/A]	1067299138
Ca_Kabuli_NC_021165.1_4576344	Ca_kabuli_chr06	4576344	[A/C]	1067299139
Ca_Kabuli_NC_021165.1_4576371	Ca_kabuli_chr06	4576371	[T/G]	1067299140
Ca_Kabuli_NC_021165.1_4593905	Ca_kabuli_chr06	4593905	[T/A]	1067299141
Ca_Kabuli_NC_021165.1_4593921	Ca_kabuli_chr06	4593921	[T/A]	1067299142
Ca_Kabuli_NC_021165.1_4593930	Ca_kabuli_chr06	4593930	[T/G]	1067299143
Ca_Kabuli_NC_021165.1_4658996	Ca_kabuli_chr06	4658996	[T/G]	1067299144
Ca_Kabuli_NC_021165.1_4659002	Ca_kabuli_chr06	4659002	[C/T]	1067299145
Ca_Kabuli_NC_021165.1_4659008	Ca_kabuli_chr06	4659008	[G/T]	1067299146
Ca_Kabuli_NC_021165.1_4678491	Ca_kabuli_chr06	4678491	[T/C]	1067299147
Ca_Kabuli_NC_021165.1_4678502	Ca_kabuli_chr06	4678502	[A/G]	1067299148
Ca_Kabuli_NC_021165.1_4678942	Ca_kabuli_chr06	4678942	[T/G]	1067299149
Ca_Kabuli_NC_021165.1_4678947	Ca_kabuli_chr06	4678947	[T/G]	1067299150
Ca_Kabuli_NC_021165.1_4726103	Ca_kabuli_chr06	4726103	[A/C]	1067299151
Ca_Kabuli_NC_021165.1_4804666	Ca_kabuli_chr06	4804666	[T/C]	1067299152
Ca_Kabuli_NC_021165.1_4807163	Ca_kabuli_chr06	4807163	[T/C]	1067299153
Ca_Kabuli_NC_021165.1_4807166	Ca_kabuli_chr06	4807166	[T/G]	1067299154
Ca_Kabuli_NC_021165.1_4807194	Ca_kabuli_chr06	4807194	[T/A]	1067299155
Ca_Kabuli_NC_021165.1_4807215	Ca_kabuli_chr06	4807215	[A/G]	1067299156
Ca_Kabuli_NC_021165.1_4898411	Ca_kabuli_chr06	4898411	[T/G]	1067299157
Ca_Kabuli_NC_021165.1_4979994	Ca_kabuli_chr06	4979994	[T/G]	1067299158
Ca_Kabuli_NC_021165.1_4980479	Ca_kabuli_chr06	4980479	[T/C]	1067299159
Ca_Kabuli_NC_021165.1_4980518	Ca_kabuli_chr06	4980518	[A/G]	1067299160
Ca_Kabuli_NC_021165.1_4994824	Ca_kabuli_chr06	4994824	[A/C]	1067299161
Ca_Kabuli_NC_021165.1_4994825	Ca_kabuli_chr06	4994825	[T/G]	1067299162
Ca_Kabuli_NC_021165.1_4994827	Ca_kabuli_chr06	4994827	[A/T]	1067299163
Ca_Kabuli_NC_021165.1_4994828	Ca_kabuli_chr06	4994828	[T/C]	1067299164
Ca_Kabuli_NC_021165.1_4994829	Ca_kabuli_chr06	4994829	[G/T]	1067299165
Ca_Kabuli_NC_021165.1_4994876	Ca_kabuli_chr06	4994876	[T/A]	1067299166
Ca_Kabuli_NC_021165.1_4996586	Ca_kabuli_chr06	4996586	[T/C]	1067299167
Ca_Kabuli_NC_021165.1_5024302	Ca_kabuli_chr06	5024302	[G/T]	1067299168
Ca_Kabuli_NC_021165.1_5185380	Ca_kabuli_chr06	5185380	[A/G]	1067299169
Ca_Kabuli_NC_021165.1_5189488	Ca_kabuli_chr06	5189488	[A/G]	1067299170

SNP IDs	Chromosomes	Physical positions (bp)	SNPs	NCBI SNPdb accession IDs (NCBI_ss#)
Ca_Kabuli_NC_021165.1_5189493	Ca_kabuli_chr06	5189493	[A/C]	1067299171
Ca_Kabuli_NC_021165.1_5338765	Ca_kabuli_chr06	5338765	[T/C]	1067299172
Ca_Kabuli_NC_021165.1_5341298	Ca_kabuli_chr06	5341298	[T/C]	1067299173
Ca_Kabuli_NC_021165.1_5341300	Ca_kabuli_chr06	5341300	[A/C]	1067299174
Ca_Kabuli_NC_021165.1_5341307	Ca_kabuli_chr06	5341307	[T/G]	1067299175
Ca_Kabuli_NC_021165.1_5366182	Ca_kabuli_chr06	5366182	[A/C]	1067299176
Ca_Kabuli_NC_021165.1_5392089	Ca_kabuli_chr06	5392089	[T/G]	1067299177
Ca_Kabuli_NC_021165.1_5392115	Ca_kabuli_chr06	5392115	[A/G]	1067299178
Ca_Kabuli_NC_021165.1_5392208	Ca_kabuli_chr06	5392208	[T/G]	1067299179
Ca_Kabuli_NC_021165.1_5396422	Ca_kabuli_chr06	5396422	[A/G]	1067299180
Ca_Kabuli_NC_021165.1_5396432	Ca_kabuli_chr06	5396432	[T/G]	1067299181
Ca_Kabuli_NC_021165.1_5396461	Ca_kabuli_chr06	5396461	[T/A]	1067299182
Ca_Kabuli_NC_021165.1_5396481	Ca_kabuli_chr06	5396481	[T/A]	1067299183
Ca_Kabuli_NC_021165.1_5396888	Ca_kabuli_chr06	5396888	[T/G]	1067299184
Ca_Kabuli_NC_021165.1_5396890	Ca_kabuli_chr06	5396890	[T/G]	1067299185
Ca_Kabuli_NC_021165.1_5396905	Ca_kabuli_chr06	5396905	[C/T]	1067299186
Ca_Kabuli_NC_021165.1_5401573	Ca_kabuli_chr06	5401573	[A/C]	1067299187
Ca_Kabuli_NC_021165.1_5401607	Ca_kabuli_chr06	5401607	[T/G]	1067299188
Ca_Kabuli_NC_021165.1_5401618	Ca_kabuli_chr06	5401618	[A/G]	1067299189
Ca_Kabuli_NC_021165.1_5492542	Ca_kabuli_chr06	5492542	[A/C]	1067299190
Ca_Kabuli_NC_021165.1_5492552	Ca_kabuli_chr06	5492552	[A/T]	1067299191
Ca_Kabuli_NC_021165.1_5497981	Ca_kabuli_chr06	5497981	[G/A]	1067299192
Ca_Kabuli_NC_021165.1_5497982	Ca_kabuli_chr06	5497982	[A/T]	1067299193
Ca_Kabuli_NC_021165.1_5497983	Ca_kabuli_chr06	5497983	[A/C]	1067299194
Ca_Kabuli_NC_021165.1_5497984	Ca_kabuli_chr06	5497984	[G/T]	1067299195
Ca_Kabuli_NC_021165.1_5498794	Ca_kabuli_chr06	5498794	[T/A]	1067299196
Ca_Kabuli_NC_021165.1_5498820	Ca_kabuli_chr06	5498820	[A/G]	1067299197
Ca_Kabuli_NC_021165.1_5498831	Ca_kabuli_chr06	5498831	[T/G]	1067299198
Ca_Kabuli_NC_021165.1_5498896	Ca_kabuli_chr06	5498896	[A/C]	1067299199
Ca_Kabuli_NC_021165.1_5508581	Ca_kabuli_chr06	5508581	[A/C]	1067299200
Ca_Kabuli_NC_021165.1_5508582	Ca_kabuli_chr06	5508582	[G/T]	1067299201
Ca_Kabuli_NC_021165.1_5508583	Ca_kabuli_chr06	5508583	[A/C]	1067299202
Ca_Kabuli_NC_021165.1_5508586	Ca_kabuli_chr06	5508586	[G/T]	1067299203
Ca_Kabuli_NC_021165.1_5533491	Ca_kabuli_chr06	5533491	[T/G]	1067299204

SNP IDs	Chromosomes	Physical positions (bp)	SNPs	NCBI SNPdb accession IDs (NCBI_ss#)
Ca_Kabuli_NC_021165.1_5534436	Ca_kabuli_chr06	5534436	[A/C]	1067299205
Ca_Kabuli_NC_021165.1_5566408	Ca_kabuli_chr06	5566408	[A/G]	1067299206
Ca_Kabuli_NC_021165.1_5566434	Ca_kabuli_chr06	5566434	[A/G]	1067299207
Ca_Kabuli_NC_021165.1_5566468	Ca_kabuli_chr06	5566468	[A/G]	1067299208
Ca_Kabuli_NC_021165.1_5566480	Ca_kabuli_chr06	5566480	[T/C]	1067299209
Ca_Kabuli_NC_021165.1_5567391	Ca_kabuli_chr06	5567391	[A/G]	1067299210
Ca_Kabuli_NC_021165.1_5567393	Ca_kabuli_chr06	5567393	[A/G]	1067299211
Ca_Kabuli_NC_021165.1_5567396	Ca_kabuli_chr06	5567396	[T/G]	1067299212
Ca_Kabuli_NC_021165.1_5567688	Ca_kabuli_chr06	5567688	[T/G]	1067299213
Ca_Kabuli_NC_021165.1_5590003	Ca_kabuli_chr06	5590003	[T/C]	1067299214
Ca_Kabuli_NC_021165.1_5590085	Ca_kabuli_chr06	5590085	[A/G]	1067299215
Ca_Kabuli_NC_021165.1_5628956	Ca_kabuli_chr06	5628956	[T/G]	1067299216
Ca_Kabuli_NC_021165.1_5694428	Ca_kabuli_chr06	5694428	[T/G]	1067299217
Ca_Kabuli_NC_021165.1_5702553	Ca_kabuli_chr06	5702553	[A/C]	1067299218
Ca_Kabuli_NC_021165.1_5702554	Ca_kabuli_chr06	5702554	[C/A]	1067299219
Ca_Kabuli_NC_021165.1_5725111	Ca_kabuli_chr06	5725111	[A/C]	1067299220
Ca_Kabuli_NC_021165.1_5745485	Ca_kabuli_chr06	5745485	[T/A]	1067299221
Ca_Kabuli_NC_021165.1_5745495	Ca_kabuli_chr06	5745495	[A/C]	1067299222
Ca_Kabuli_NC_021165.1_5745527	Ca_kabuli_chr06	5745527	[T/C]	1067299223
Ca_Kabuli_NC_021165.1_5871503	Ca_kabuli_chr06	5871503	[C/A]	1067299224
Ca_Kabuli_NC_021165.1_6028793	Ca_kabuli_chr06	6028793	[T/G]	1067299225
Ca_Kabuli_NC_021165.1_6028806	Ca_kabuli_chr06	6028806	[A/C]	1067299226
Ca_Kabuli_NC_021165.1_6028837	Ca_kabuli_chr06	6028837	[A/T]	1067299227
Ca_Kabuli_NC_021165.1_6092493	Ca_kabuli_chr06	6092493	[A/C]	1067299228
Ca_Kabuli_NC_021165.1_6119560	Ca_kabuli_chr06	6119560	[A/G]	1067299229
Ca_Kabuli_NC_021165.1_6169085	Ca_kabuli_chr06	6169085	[T/C]	1067299230
Ca_Kabuli_NC_021165.1_6227880	Ca_kabuli_chr06	6227880	[T/G]	1067299231
Ca_Kabuli_NC_021165.1_6240817	Ca_kabuli_chr06	6240817	[T/G]	1067299232
Ca_Kabuli_NC_021165.1_6248218	Ca_kabuli_chr06	6248218	[G/A]	1067299233
Ca_Kabuli_NC_021165.1_6248221	Ca_kabuli_chr06	6248221	[T/A]	1067299234
Ca_Kabuli_NC_021165.1_6259498	Ca_kabuli_chr06	6259498	[T/G]	1067299235
Ca_Kabuli_NC_021165.1_6260546	Ca_kabuli_chr06	6260546	[T/C]	1067299236
Ca_Kabuli_NC_021165.1_6444243	Ca_kabuli_chr06	6444243	[G/A]	1067299237
Ca_Kabuli_NC_021165.1_6444250	Ca_kabuli_chr06	6444250	[G/A]	1067299238

SNP IDs	Chromosomes	Physical positions (bp)	SNPs	NCBI SNPdb accession IDs (NCBI_ss#)
Ca_Kabuli_NC_021165.1_6517785	Ca_kabuli_chr06	6517785	[T/C]	1067299239
Ca_Kabuli_NC_021165.1_6538136	Ca_kabuli_chr06	6538136	[T/C]	1067299240
Ca_Kabuli_NC_021165.1_6538159	Ca_kabuli_chr06	6538159	[A/T]	1067299241
Ca_Kabuli_NC_021165.1_6604682	Ca_kabuli_chr06	6604682	[A/G]	1067299242
Ca_Kabuli_NC_021165.1_6604812	Ca_kabuli_chr06	6604812	[T/C]	1067299243
Ca_Kabuli_NC_021165.1_6604850	Ca_kabuli_chr06	6604850	[T/G]	1067299244
Ca_Kabuli_NC_021165.1_6607771	Ca_kabuli_chr06	6607771	[T/C]	1067299245
Ca_Kabuli_NC_021165.1_6607781	Ca_kabuli_chr06	6607781	[A/G]	1067299246
Ca_Kabuli_NC_021165.1_6607784	Ca_kabuli_chr06	6607784	[A/G]	1067299247
Ca_Kabuli_NC_021165.1_6607809	Ca_kabuli_chr06	6607809	[T/C]	1067299248
Ca_Kabuli_NC_021165.1_6607829	Ca_kabuli_chr06	6607829	[T/C]	1067299249
Ca_Kabuli_NC_021165.1_6607831	Ca_kabuli_chr06	6607831	[A/G]	1067299250
Ca_Kabuli_NC_021165.1_6617106	Ca_kabuli_chr06	6617106	[T/C]	1067299251
Ca_Kabuli_NC_021165.1_6631245	Ca_kabuli_chr06	6631245	[A/G]	1067299252
Ca_Kabuli_NC_021165.1_6654880	Ca_kabuli_chr06	6654880	[T/G]	1067299253
Ca_Kabuli_NC_021165.1_6654975	Ca_kabuli_chr06	6654975	[A/T]	1067299254
Ca_Kabuli_NC_021165.1_6654977	Ca_kabuli_chr06	6654977	[A/C]	1067299255
Ca_Kabuli_NC_021165.1_6684780	Ca_kabuli_chr06	6684780	[T/C]	1067299256
Ca_Kabuli_NC_021165.1_6684814	Ca_kabuli_chr06	6684814	[T/C]	1067299257
Ca_Kabuli_NC_021165.1_6778820	Ca_kabuli_chr06	6778820	[T/G]	1067299258
Ca_Kabuli_NC_021165.1_6779173	Ca_kabuli_chr06	6779173	[A/G]	1067299259
Ca_Kabuli_NC_021165.1_6802386	Ca_kabuli_chr06	6802386	[T/G]	1067299260
Ca_Kabuli_NC_021165.1_6803494	Ca_kabuli_chr06	6803494	[A/G]	1067299261
Ca_Kabuli_NC_021165.1_6814989	Ca_kabuli_chr06	6814989	[T/A]	1067299262
Ca_Kabuli_NC_021165.1_6815001	Ca_kabuli_chr06	6815001	[T/G]	1067299263
Ca_Kabuli_NC_021165.1_6815006	Ca_kabuli_chr06	6815006	[G/T]	1067299264
Ca_Kabuli_NC_021165.1_6817431	Ca_kabuli_chr06	6817431	[A/C]	1067299265
Ca_Kabuli_NC_021165.1_6817441	Ca_kabuli_chr06	6817441	[A/C]	1067299266
Ca_Kabuli_NC_021165.1_6876986	Ca_kabuli_chr06	6876986	[T/C]	1067299267
Ca_Kabuli_NC_021165.1_6877248	Ca_kabuli_chr06	6877248	[A/T]	1067299268
Ca_Kabuli_NC_021165.1_6877291	Ca_kabuli_chr06	6877291	[A/T]	1067299269
Ca_Kabuli_NC_021165.1_6877419	Ca_kabuli_chr06	6877419	[A/G]	1067299270
Ca_Kabuli_NC_021165.1_6905533	Ca_kabuli_chr06	6905533	[A/C]	1067299271
Ca_Kabuli_NC_021165.1_6905578	Ca_kabuli_chr06	6905578	[A/T]	1067299272

SNP IDs	Chromosomes	Physical positions (bp)	SNPs	NCBI SNPdb accession IDs (NCBI_ss#)
Ca_Kabuli_NC_021165.1_6905583	Ca_kabuli_chr06	6905583	[T/C]	1067299273
Ca_Kabuli_NC_021165.1_6913622	Ca_kabuli_chr06	6913622	[A/C]	1067299274
Ca_Kabuli_NC_021165.1_6933439	Ca_kabuli_chr06	6933439	[A/C]	1067299275
Ca_Kabuli_NC_021165.1_6933440	Ca_kabuli_chr06	6933440	[G/T]	1067299276
Ca_Kabuli_NC_021165.1_7034418	Ca_kabuli_chr06	7034418	[A/T]	1067299277
Ca_Kabuli_NC_021165.1_7040294	Ca_kabuli_chr06	7040294	[A/T]	1067299278
Ca_Kabuli_NC_021165.1_7054087	Ca_kabuli_chr06	7054087	[T/G]	1067299279
Ca_Kabuli_NC_021165.1_7054098	Ca_kabuli_chr06	7054098	[A/C]	1067299280
Ca_Kabuli_NC_021165.1_7054111	Ca_kabuli_chr06	7054111	[A/G]	1067299281
Ca_Kabuli_NC_021165.1_7126660	Ca_kabuli_chr06	7126660	[A/G]	1067299282
Ca_Kabuli_NC_021165.1_7138432	Ca_kabuli_chr06	7138432	[T/G]	1067299283
Ca_Kabuli_NC_021165.1_7185677	Ca_kabuli_chr06	7185677	[A/C]	1067299284
Ca_Kabuli_NC_021165.1_7187867	Ca_kabuli_chr06	7187867	[C/T]	1067299285
Ca_Kabuli_NC_021165.1_7262413	Ca_kabuli_chr06	7262413	[A/T]	1067299286
Ca_Kabuli_NC_021165.1_7262414	Ca_kabuli_chr06	7262414	[T/C]	1067299287
Ca_Kabuli_NC_021165.1_7349517	Ca_kabuli_chr06	7349517	[A/C]	1067299288
Ca_Kabuli_NC_021165.1_7414196	Ca_kabuli_chr06	7414196	[T/G]	1067299289
Ca_Kabuli_NC_021165.1_7414302	Ca_kabuli_chr06	7414302	[A/G]	1067299290
Ca_Kabuli_NC_021165.1_7532384	Ca_kabuli_chr06	7532384	[A/G]	1067299291
Ca_Kabuli_NC_021165.1_7559211	Ca_kabuli_chr06	7559211	[A/G]	1067299292
Ca_Kabuli_NC_021165.1_7575124	Ca_kabuli_chr06	7575124	[T/C]	1067299293
Ca_Kabuli_NC_021165.1_7576773	Ca_kabuli_chr06	7576773	[T/G]	1067299294
Ca_Kabuli_NC_021165.1_7581799	Ca_kabuli_chr06	7581799	[C/T]	1067299295
Ca_Kabuli_NC_021165.1_7581956	Ca_kabuli_chr06	7581956	[C/G]	1067299296
Ca_Kabuli_NC_021165.1_7582726	Ca_kabuli_chr06	7582726	[G/T]	1067299297
Ca_Kabuli_NC_021165.1_7609380	Ca_kabuli_chr06	7609380	[G/A]	1067299298
Ca_Kabuli_NC_021165.1_7609573	Ca_kabuli_chr06	7609573	[A/G]	1067299299
Ca_Kabuli_NC_021165.1_7618280	Ca_kabuli_chr06	7618280	[A/C]	1067299300
Ca_Kabuli_NC_021165.1_7630374	Ca_kabuli_chr06	7630374	[G/C]	1067299301
Ca_Kabuli_NC_021165.1_7647916	Ca_kabuli_chr06	7647916	[T/G]	1067299302
Ca_Kabuli_NC_021165.1_7661924	Ca_kabuli_chr06	7661924	[C/T]	1067299303
Ca_Kabuli_NC_021165.1_7661930	Ca_kabuli_chr06	7661930	[G/T]	1067299304
Ca_Kabuli_NC_021165.1_7667301	Ca_kabuli_chr06	7667301	[T/C]	1067299305
Ca_Kabuli_NC_021165.1_7667328	Ca_kabuli_chr06	7667328	[A/T]	1067299306

SNP IDs	Chromosomes	Physical positions (bp)	SNPs	NCBI SNPdb accession IDs (NCBI_ss#)
Ca_Kabuli_NC_021165.1_7694594	Ca_kabuli_chr06	7694594	[G/T]	1067299307
Ca_Kabuli_NC_021165.1_7694597	Ca_kabuli_chr06	7694597	[T/C]	1067299308
Ca_Kabuli_NC_021165.1_7694599	Ca_kabuli_chr06	7694599	[T/C]	1067299309
Ca_Kabuli_NC_021165.1_7719829	Ca_kabuli_chr06	7719829	[C/T]	1067299310
Ca_Kabuli_NC_021165.1_7719857	Ca_kabuli_chr06	7719857	[G/T]	1067299311
Ca_Kabuli_NC_021165.1_7719877	Ca_kabuli_chr06	7719877	[T/C]	1067299312
Ca_Kabuli_NC_021165.1_7744730	Ca_kabuli_chr06	7744730	[G/A]	1067299313
Ca_Kabuli_NC_021165.1_7745344	Ca_kabuli_chr06	7745344	[T/A]	1067299314
Ca_Kabuli_NC_021165.1_7745377	Ca_kabuli_chr06	7745377	[C/T]	1067299315
Ca_Kabuli_NC_021165.1_7745923	Ca_kabuli_chr06	7745923	[T/A]	1067299316
Ca_Kabuli_NC_021165.1_7745942	Ca_kabuli_chr06	7745942	[T/G]	1067299317
Ca_Kabuli_NC_021165.1_7747254	Ca_kabuli_chr06	7747254	[A/G]	1067299318
Ca_Kabuli_NC_021165.1_7753341	Ca_kabuli_chr06	7753341	[T/A]	1067299319
Ca_Kabuli_NC_021165.1_7765050	Ca_kabuli_chr06	7765050	[T/C]	1067299320
Ca_Kabuli_NC_021165.1_7765068	Ca_kabuli_chr06	7765068	[A/C]	1067299321
Ca_Kabuli_NC_021165.1_7765091	Ca_kabuli_chr06	7765091	[A/G]	1067299322
Ca_Kabuli_NC_021165.1_7770357	Ca_kabuli_chr06	7770357	[C/T]	1067299323
Ca_Kabuli_NC_021165.1_7810893	Ca_kabuli_chr06	7810893	[A/G]	1067299324
Ca_Kabuli_NC_021165.1_7810902	Ca_kabuli_chr06	7810902	[A/G]	1067299325
Ca_Kabuli_NC_021165.1_7810923	Ca_kabuli_chr06	7810923	[T/C]	1067299326
Ca_Kabuli_NC_021165.1_7819313	Ca_kabuli_chr06	7819313	[C/G]	1067299327
Ca_Kabuli_NC_021165.1_7819348	Ca_kabuli_chr06	7819348	[C/T]	1067299328
Ca_Kabuli_NC_021165.1_7820481	Ca_kabuli_chr06	7820481	[C/T]	1067299329
Ca_Kabuli_NC_021165.1_7820807	Ca_kabuli_chr06	7820807	[A/G]	1067299330
Ca_Kabuli_NC_021165.1_7829272	Ca_kabuli_chr06	7829272	[T/C]	1067299331
Ca_Kabuli_NC_021165.1_7829471	Ca_kabuli_chr06	7829471	[C/A]	1067299332
Ca_Kabuli_NC_021165.1_7829472	Ca_kabuli_chr06	7829472	[A/T]	1067299333
Ca_Kabuli_NC_021165.1_7829473	Ca_kabuli_chr06	7829473	[A/C]	1067299334
Ca_Kabuli_NC_021165.1_7829474	Ca_kabuli_chr06	7829474	[G/T]	1067299335
Ca_Kabuli_NC_021165.1_7829485	Ca_kabuli_chr06	7829485	[G/A]	1067299336
Ca_Kabuli_NC_021165.1_7829577	Ca_kabuli_chr06	7829577	[T/G]	1067299337
Ca_Kabuli_NC_021165.1_7829627	Ca_kabuli_chr06	7829627	[T/G]	1067299338
Ca_Kabuli_NC_021165.1_7831123	Ca_kabuli_chr06	7831123	[T/G]	1067299339
Ca_Kabuli_NC_021165.1_7831334	Ca_kabuli_chr06	7831334	[T/C]	1067299340

SNP IDs	Chromosomes	Physical positions (bp)	SNPs	NCBI SNPdb accession IDs (NCBI_ss#)
Ca_Kabuli_NC_021165.1_7831409	Ca_kabuli_chr06	7831409	[C/T]	1067299341
Ca_Kabuli_NC_021165.1_7831874	Ca_kabuli_chr06	7831874	[A/G]	1067299342
Ca_Kabuli_NC_021165.1_7831899	Ca_kabuli_chr06	7831899	[A/G]	1067299343
Ca_Kabuli_NC_021165.1_7832023	Ca_kabuli_chr06	7832023	[C/T]	1067299344
Ca_Kabuli_NC_021165.1_7832029	Ca_kabuli_chr06	7832029	[C/T]	1067299345
Ca_Kabuli_NC_021165.1_7834154	Ca_kabuli_chr06	7834154	[T/A]	1067299346
Ca_Kabuli_NC_021165.1_7834595	Ca_kabuli_chr06	7834595	[C/T]	1067299347
Ca_Kabuli_NC_021165.1_7834866	Ca_kabuli_chr06	7834866	[T/G]	1067299348
Ca_Kabuli_NC_021165.1_7834895	Ca_kabuli_chr06	7834895	[G/T]	1067299349
Ca_Kabuli_NC_021165.1_7834922	Ca_kabuli_chr06	7834922	[A/G]	1067299350
Ca_Kabuli_NC_021165.1_7835024	Ca_kabuli_chr06	7835024	[T/A]	1067299351
Ca_Kabuli_NC_021165.1_7835510	Ca_kabuli_chr06	7835510	[G/A]	1067299352
Ca_Kabuli_NC_021165.1_7835619	Ca_kabuli_chr06	7835619	[C/A]	1067299353
Ca_Kabuli_NC_021165.1_7835620	Ca_kabuli_chr06	7835620	[A/G]	1067299354
Ca_Kabuli_NC_021165.1_7835759	Ca_kabuli_chr06	7835759	[A/G]	1067299355
Ca_Kabuli_NC_021165.1_7835984	Ca_kabuli_chr06	7835984	[T/A]	1067299356
Ca_Kabuli_NC_021165.1_7836879	Ca_kabuli_chr06	7836879	[T/C]	1067299357
Ca_Kabuli_NC_021165.1_7846231	Ca_kabuli_chr06	7846231	[T/G]	1067299358
Ca_Kabuli_NC_021165.1_7846335	Ca_kabuli_chr06	7846335	[C/G]	1067299359
Ca_Kabuli_NC_021165.1_7849200	Ca_kabuli_chr06	7849200	[T/C]	1067299360
Ca_Kabuli_NC_021165.1_7851000	Ca_kabuli_chr06	7851000	[C/G]	1067299361
Ca_Kabuli_NC_021165.1_7851697	Ca_kabuli_chr06	7851697	[T/G]	1067299362
Ca_Kabuli_NC_021165.1_7852481	Ca_kabuli_chr06	7852481	[G/A]	1067299363
Ca_Kabuli_NC_021165.1_7876049	Ca_kabuli_chr06	7876049	[A/C]	1067299364
Ca_Kabuli_NC_021165.1_7878258	Ca_kabuli_chr06	7878258	[T/C]	1067299365
Ca_Kabuli_NC_021165.1_7879124	Ca_kabuli_chr06	7879124	[A/C]	1067299366
Ca_Kabuli_NC_021165.1_7879153	Ca_kabuli_chr06	7879153	[A/C]	1067299367
Ca_Kabuli_NC_021165.1_7879165	Ca_kabuli_chr06	7879165	[A/G]	1067299368
Ca_Kabuli_NC_021165.1_7879171	Ca_kabuli_chr06	7879171	[T/A]	1067299369
Ca_Kabuli_NC_021165.1_7884965	Ca_kabuli_chr06	7884965	[G/C]	1067299370
Ca_Kabuli_NC_021165.1_7926416	Ca_kabuli_chr06	7926416	[C/A]	1067299371
Ca_Kabuli_NC_021165.1_7926482	Ca_kabuli_chr06	7926482	[T/C]	1067299372
Ca_Kabuli_NC_021165.1_7926534	Ca_kabuli_chr06	7926534	[G/A]	1067299373
Ca_Kabuli_NC_021165.1_7928743	Ca_kabuli_chr06	7928743	[C/T]	1067299374

SNP IDs	Chromosomes	Physical positions (bp)	SNPs	NCBI SNPdb accession IDs (NCBI_ss#)
Ca_Kabuli_NC_021165.1_7929080	Ca_kabuli_chr06	7929080	[G/A]	1067299375
Ca_Kabuli_NC_021165.1_7929238	Ca_kabuli_chr06	7929238	[T/G]	1067299376
Ca_Kabuli_NC_021165.1_7929338	Ca_kabuli_chr06	7929338	[C/T]	1067299377
Ca_Kabuli_NC_021165.1_7929339	Ca_kabuli_chr06	7929339	[A/G]	1067299378
Ca_Kabuli_NC_021165.1_7929348	Ca_kabuli_chr06	7929348	[T/C]	1067299379
Ca_Kabuli_NC_021165.1_7929607	Ca_kabuli_chr06	7929607	[A/C]	1067299380
Ca_Kabuli_NC_021165.1_7929628	Ca_kabuli_chr06	7929628	[C/T]	1067299381
Ca_Kabuli_NC_021165.1_7929647	Ca_kabuli_chr06	7929647	[C/A]	1067299382
Ca_Kabuli_NC_021165.1_7939281	Ca_kabuli_chr06	7939281	[T/G]	1067299383
Ca_Kabuli_NC_021165.1_7943491	Ca_kabuli_chr06	7943491	[T/G]	1067299384
Ca_Kabuli_NC_021165.1_7961572	Ca_kabuli_chr06	7961572	[T/C]	1067299385
Ca_Kabuli_NC_021165.1_7961598	Ca_kabuli_chr06	7961598	[T/C]	1067299386
Ca_Kabuli_NC_021165.1_7994732	Ca_kabuli_chr06	7994732	[G/T]	1067299387
Ca_Kabuli_NC_021165.1_8006682	Ca_kabuli_chr06	8006682	[C/T]	1067299388
Ca_Kabuli_NC_021165.1_8006701	Ca_kabuli_chr06	8006701	[A/T]	1067299389
Ca_Kabuli_NC_021165.1_8006721	Ca_kabuli_chr06	8006721	[A/T]	1067299390
Ca_Kabuli_NC_021165.1_8011735	Ca_kabuli_chr06	8011735	[T/C]	1067299391
Ca_Kabuli_NC_021165.1_8028646	Ca_kabuli_chr06	8028646	[C/T]	1067299392
Ca_Kabuli_NC_021165.1_8029210	Ca_kabuli_chr06	8029210	[T/A]	1067299393
Ca_Kabuli_NC_021165.1_8029226	Ca_kabuli_chr06	8029226	[C/T]	1067299394
Ca_Kabuli_NC_021165.1_8029310	Ca_kabuli_chr06	8029310	[G/C]	1067299395
Ca_Kabuli_NC_021165.1_8052624	Ca_kabuli_chr06	8052624	[G/C]	1067299396
Ca_Kabuli_NC_021165.1_8070427	Ca_kabuli_chr06	8070427	[C/T]	1067299397
Ca_Kabuli_NC_021165.1_8076394	Ca_kabuli_chr06	8076394	[T/C]	1067299398
Ca_Kabuli_NC_021165.1_8076448	Ca_kabuli_chr06	8076448	[A/G]	1067299399
Ca_Kabuli_NC_021165.1_8084950	Ca_kabuli_chr06	8084950	[T/A]	1067299400
Ca_Kabuli_NC_021165.1_8090739	Ca_kabuli_chr06	8090739	[T/A]	1067299401
Ca_Kabuli_NC_021165.1_8091050	Ca_kabuli_chr06	8091050	[A/G]	1067299402
Ca_Kabuli_NC_021165.1_8091193	Ca_kabuli_chr06	8091193	[A/G]	1067299403
Ca_Kabuli_NC_021165.1_8091210	Ca_kabuli_chr06	8091210	[T/C]	1067299404
Ca_Kabuli_NC_021165.1_8091218	Ca_kabuli_chr06	8091218	[A/C]	1067299405
Ca_Kabuli_NC_021165.1_8091225	Ca_kabuli_chr06	8091225	[G/T]	1067299406
Ca_Kabuli_NC_021165.1_8091232	Ca_kabuli_chr06	8091232	[C/T]	1067299407
Ca_Kabuli_NC_021165.1_8134381	Ca_kabuli_chr06	8134381	[T/C]	1067299408

SNP IDs	Chromosomes	Physical positions (bp)	SNPs	NCBI SNPdb accession IDs (NCBI_ss#)
Ca_Kabuli_NC_021165.1_8139446	Ca_kabuli_chr06	8139446	[C/A]	1067299409
Ca_Kabuli_NC_021165.1_8161366	Ca_kabuli_chr06	8161366	[T/C]	1067299410
Ca_Kabuli_NC_021165.1_8161368	Ca_kabuli_chr06	8161368	[G/T]	1067299411
Ca_Kabuli_NC_021165.1_8166339	Ca_kabuli_chr06	8166339	[T/C]	1067299412
Ca_Kabuli_NC_021165.1_8166435	Ca_kabuli_chr06	8166435	[A/G]	1067299413
Ca_Kabuli_NC_021165.1_8168203	Ca_kabuli_chr06	8168203	[C/T]	1067299414
Ca_Kabuli_NC_021165.1_8170170	Ca_kabuli_chr06	8170170	[A/C]	1067299415
Ca_Kabuli_NC_021165.1_8170583	Ca_kabuli_chr06	8170583	[C/T]	1067299416
Ca_Kabuli_NC_021165.1_8170633	Ca_kabuli_chr06	8170633	[A/G]	1067299417
Ca_Kabuli_NC_021165.1_8222675	Ca_kabuli_chr06	8222675	[A/T]	1067299418
Ca_Kabuli_NC_021165.1_8246205	Ca_kabuli_chr06	8246205	[A/G]	1067299419
Ca_Kabuli_NC_021165.1_8246210	Ca_kabuli_chr06	8246210	[C/A]	1067299420
Ca_Kabuli_NC_021165.1_8246313	Ca_kabuli_chr06	8246313	[T/G]	1067299421
Ca_Kabuli_NC_021165.1_8589290	Ca_kabuli_chr06	8589290	[A/C]	1067299422
Ca_Kabuli_NC_021165.1_8592849	Ca_kabuli_chr06	8592849	[A/C]	1067299423
Ca_Kabuli_NC_021165.1_8592850	Ca_kabuli_chr06	8592850	[G/A]	1067299424
Ca_Kabuli_NC_021165.1_8592851	Ca_kabuli_chr06	8592851	[A/C]	1067299425
Ca_Kabuli_NC_021165.1_8633221	Ca_kabuli_chr06	8633221	[A/C]	1067299426
Ca_Kabuli_NC_021165.1_8634276	Ca_kabuli_chr06	8634276	[T/C]	1067299427
Ca_Kabuli_NC_021165.1_8634285	Ca_kabuli_chr06	8634285	[T/C]	1067299428
Ca_Kabuli_NC_021165.1_8635559	Ca_kabuli_chr06	8635559	[A/G]	1067299429
Ca_Kabuli_NC_021165.1_8655304	Ca_kabuli_chr06	8655304	[A/C]	1067299430
Ca_Kabuli_NC_021165.1_8655363	Ca_kabuli_chr06	8655363	[A/G]	1067299431
Ca_Kabuli_NC_021165.1_8655382	Ca_kabuli_chr06	8655382	[A/T]	1067299432
Ca_Kabuli_NC_021165.1_8655664	Ca_kabuli_chr06	8655664	[T/G]	1067299433
Ca_Kabuli_NC_021165.1_8655742	Ca_kabuli_chr06	8655742	[A/C]	1067299434
Ca_Kabuli_NC_021165.1_8657846	Ca_kabuli_chr06	8657846	[A/C]	1067299435
Ca_Kabuli_NC_021165.1_8657849	Ca_kabuli_chr06	8657849	[A/G]	1067299436
Ca_Kabuli_NC_021165.1_8706400	Ca_kabuli_chr06	8706400	[A/T]	1067299437
Ca_Kabuli_NC_021165.1_8706439	Ca_kabuli_chr06	8706439	[T/G]	1067299438
Ca_Kabuli_NC_021165.1_8711674	Ca_kabuli_chr06	8711674	[A/C]	1067299439
Ca_Kabuli_NC_021165.1_8711678	Ca_kabuli_chr06	8711678	[A/G]	1067299440
Ca_Kabuli_NC_021165.1_8711694	Ca_kabuli_chr06	8711694	[T/C]	1067299441
Ca_Kabuli_NC_021165.1_8738094	Ca_kabuli_chr06	8738094	[A/C]	1067299442

SNP IDs	Chromosomes	Physical positions (bp)	SNPs	NCBI SNPdb accession IDs (NCBI_ss#)
Ca_Kabuli_NC_021165.1_8749010	Ca_kabuli_chr06	8749010	[A/C]	1067299443
Ca_Kabuli_NC_021165.1_8787401	Ca_kabuli_chr06	8787401	[C/A]	1067299444
Ca_Kabuli_NC_021165.1_8787557	Ca_kabuli_chr06	8787557	[T/C]	1067299445
Ca_Kabuli_NC_021165.1_8787559	Ca_kabuli_chr06	8787559	[A/T]	1067299446
Ca_Kabuli_NC_021165.1_8958549	Ca_kabuli_chr06	8958549	[T/G]	1067299447
Ca_Kabuli_NC_021165.1_9088140	Ca_kabuli_chr06	9088140	[T/C]	1067299448
Ca_Kabuli_NC_021165.1_9088224	Ca_kabuli_chr06	9088224	[A/C]	1067299449
Ca_Kabuli_NC_021165.1_9109120	Ca_kabuli_chr06	9109120	[T/G]	1067299450
Ca_Kabuli_NC_021165.1_9133666	Ca_kabuli_chr06	9133666	[A/G]	1067299451
Ca_Kabuli_NC_021165.1_9133668	Ca_kabuli_chr06	9133668	[C/A]	1067299452
Ca_Kabuli_NC_021165.1_9133677	Ca_kabuli_chr06	9133677	[A/C]	1067299453
Ca_Kabuli_NC_021165.1_9133694	Ca_kabuli_chr06	9133694	[T/C]	1067299454
Ca_Kabuli_NC_021165.1_9133718	Ca_kabuli_chr06	9133718	[A/G]	1067299455
Ca_Kabuli_NC_021165.1_9150251	Ca_kabuli_chr06	9150251	[A/G]	1067299456
Ca_Kabuli_NC_021165.1_9150253	Ca_kabuli_chr06	9150253	[C/A]	1067299457
Ca_Kabuli_NC_021165.1_9191586	Ca_kabuli_chr06	9191586	[A/G]	1067299458
Ca_Kabuli_NC_021165.1_9191619	Ca_kabuli_chr06	9191619	[A/G]	1067299459
Ca_Kabuli_NC_021165.1_9196080	Ca_kabuli_chr06	9196080	[C/A]	1067299460
Ca_Kabuli_NC_021165.1_9196081	Ca_kabuli_chr06	9196081	[A/G]	1067299461
Ca_Kabuli_NC_021165.1_9196082	Ca_kabuli_chr06	9196082	[C/A]	1067299462
Ca_Kabuli_NC_021165.1_9205251	Ca_kabuli_chr06	9205251	[A/C]	1067299463
Ca_Kabuli_NC_021165.1_9205279	Ca_kabuli_chr06	9205279	[T/A]	1067299464
Ca_Kabuli_NC_021165.1_9213875	Ca_kabuli_chr06	9213875	[A/G]	1067299465
Ca_Kabuli_NC_021165.1_9223004	Ca_kabuli_chr06	9223004	[T/G]	1067299466
Ca_Kabuli_NC_021165.1_9227975	Ca_kabuli_chr06	9227975	[A/C]	1067299467
Ca_Kabuli_NC_021165.1_9227978	Ca_kabuli_chr06	9227978	[A/T]	1067299468
Ca_Kabuli_NC_021165.1_9227984	Ca_kabuli_chr06	9227984	[T/C]	1067299469
Ca_Kabuli_NC_021165.1_9227994	Ca_kabuli_chr06	9227994	[T/G]	1067299470
Ca_Kabuli_NC_021165.1_9292292	Ca_kabuli_chr06	9292292	[T/A]	1067299471
Ca_Kabuli_NC_021165.1_9338839	Ca_kabuli_chr06	9338839	[A/C]	1067299472
Ca_Kabuli_NC_021165.1_9338901	Ca_kabuli_chr06	9338901	[T/C]	1067299473
Ca_Kabuli_NC_021165.1_9340828	Ca_kabuli_chr06	9340828	[G/T]	1067299474
Ca_Kabuli_NC_021165.1_9341262	Ca_kabuli_chr06	9341262	[A/G]	1067299475
Ca_Kabuli_NC_021165.1_9341275	Ca_kabuli_chr06	9341275	[C/T]	1067299476

SNP IDs	Chromosomes	Physical positions (bp)	SNPs	NCBI SNPdb accession IDs (NCBI_ss#)
Ca_Kabuli_NC_021165.1_9341277	Ca_kabuli_chr06	9341277	[C/T]	1067299477
Ca_Kabuli_NC_021165.1_9372825	Ca_kabuli_chr06	9372825	[T/C]	1067299478
Ca_Kabuli_NC_021165.1_9382102	Ca_kabuli_chr06	9382102	[A/T]	1067299479
Ca_Kabuli_NC_021165.1_9409159	Ca_kabuli_chr06	9409159	[A/G]	1067299480
Ca_Kabuli_NC_021165.1_9409172	Ca_kabuli_chr06	9409172	[T/A]	1067299481
Ca_Kabuli_NC_021165.1_9409185	Ca_kabuli_chr06	9409185	[A/G]	1067299482
Ca_Kabuli_NC_021165.1_9423194	Ca_kabuli_chr06	9423194	[T/C]	1067299483
Ca_Kabuli_NC_021165.1_9423213	Ca_kabuli_chr06	9423213	[A/G]	1067299484
Ca_Kabuli_NC_021165.1_9423239	Ca_kabuli_chr06	9423239	[T/C]	1067299485
Ca_Kabuli_NC_021165.1_9428548	Ca_kabuli_chr06	9428548	[T/G]	1067299486
Ca_Kabuli_NC_021165.1_9428753	Ca_kabuli_chr06	9428753	[A/C]	1067299487
Ca_Kabuli_NC_021165.1_9430204	Ca_kabuli_chr06	9430204	[G/T]	1067299488
Ca_Kabuli_NC_021165.1_9430205	Ca_kabuli_chr06	9430205	[T/G]	1067299489
Ca_Kabuli_NC_021165.1_9520222	Ca_kabuli_chr06	9520222	[T/C]	1067299490
Ca_Kabuli_NC_021165.1_9544096	Ca_kabuli_chr06	9544096	[T/A]	1067299491
Ca_Kabuli_NC_021165.1_9562800	Ca_kabuli_chr06	9562800	[G/A]	1067299492
Ca_Kabuli_NC_021165.1_9562803	Ca_kabuli_chr06	9562803	[A/G]	1067299493
Ca_Kabuli_NC_021165.1_9565638	Ca_kabuli_chr06	9565638	[A/C]	1067299494
Ca_Kabuli_NC_021165.1_9565873	Ca_kabuli_chr06	9565873	[T/A]	1067299495
Ca_Kabuli_NC_021165.1_9568341	Ca_kabuli_chr06	9568341	[A/G]	1067299496
Ca_Kabuli_NC_021165.1_9574433	Ca_kabuli_chr06	9574433	[A/G]	1067299497
Ca_Kabuli_NC_021165.1_9574457	Ca_kabuli_chr06	9574457	[A/T]	1067299498
Ca_Kabuli_NC_021165.1_9689425	Ca_kabuli_chr06	9689425	[G/T]	1067299499
Ca_Kabuli_NC_021165.1_9831549	Ca_kabuli_chr06	9831549	[T/G]	1067299500
Ca_Kabuli_NC_021165.1_9831555	Ca_kabuli_chr06	9831555	[A/G]	1067299501
Ca_Kabuli_NC_021165.1_9831559	Ca_kabuli_chr06	9831559	[T/G]	1067299502
Ca_Kabuli_NC_021165.1_9834198	Ca_kabuli_chr06	9834198	[C/A]	1067299503
Ca_Kabuli_NC_021165.1_9834199	Ca_kabuli_chr06	9834199	[T/C]	1067299504
Ca_Kabuli_NC_021165.1_9834208	Ca_kabuli_chr06	9834208	[A/G]	1067299505
Ca_Kabuli_NC_021165.1_9845102	Ca_kabuli_chr06	9845102	[T/G]	1067299506
Ca_Kabuli_NC_021165.1_9959681	Ca_kabuli_chr06	9959681	[A/G]	1067299507
Ca_Kabuli_NC_021165.1_10027025	Ca_kabuli_chr06	10027025	[A/C]	1067299508
Ca_Kabuli_NC_021165.1_10029464	Ca_kabuli_chr06	10029464	[T/C]	1067299509
Ca_Kabuli_NC_021165.1_10089004	Ca_kabuli_chr06	10089004	[T/C]	1067299510

SNP IDs	Chromosomes	Physical positions (bp)	SNPs	NCBI SNPdb accession IDs (NCBI_ss#)
Ca_Kabuli_NC_021165.1_10089025	Ca_kabuli_chr06	10089025	[A/G]	1067299511
Ca_Kabuli_NC_021165.1_10111452	Ca_kabuli_chr06	10111452	[T/G]	1067299512
Ca_Kabuli_NC_021165.1_10136888	Ca_kabuli_chr06	10136888	[C/T]	1067299513
Ca_Kabuli_NC_021165.1_10136905	Ca_kabuli_chr06	10136905	[A/G]	1067299514
Ca_Kabuli_NC_021165.1_10177838	Ca_kabuli_chr06	10177838	[T/G]	1067299515
Ca_Kabuli_NC_021165.1_10177859	Ca_kabuli_chr06	10177859	[T/G]	1067299516
Ca_Kabuli_NC_021165.1_10231032	Ca_kabuli_chr06	10231032	[T/C]	1067299517
Ca_Kabuli_NC_021165.1_10231088	Ca_kabuli_chr06	10231088	[T/G]	1067299518
Ca_Kabuli_NC_021165.1_10231089	Ca_kabuli_chr06	10231089	[T/G]	1067299519
Ca_Kabuli_NC_021165.1_10231120	Ca_kabuli_chr06	10231120	[A/G]	1067299520
Ca_Kabuli_NC_021165.1_10231141	Ca_kabuli_chr06	10231141	[A/G]	1067299521
Ca_Kabuli_NC_021165.1_10234386	Ca_kabuli_chr06	10234386	[A/C]	1067299522
Ca_Kabuli_NC_021165.1_10234387	Ca_kabuli_chr06	10234387	[G/C]	1067299523
Ca_Kabuli_NC_021165.1_10234389	Ca_kabuli_chr06	10234389	[T/A]	1067299524
Ca_Kabuli_NC_021165.1_10234390	Ca_kabuli_chr06	10234390	[C/A]	1067299525
Ca_Kabuli_NC_021165.1_10259627	Ca_kabuli_chr06	10259627	[A/C]	1067299526
Ca_Kabuli_NC_021165.1_10259643	Ca_kabuli_chr06	10259643	[A/C]	1067299527
Ca_Kabuli_NC_021165.1_10259652	Ca_kabuli_chr06	10259652	[A/G]	1067299528
Ca_Kabuli_NC_021165.1_10259663	Ca_kabuli_chr06	10259663	[T/C]	1067299529
Ca_Kabuli_NC_021165.1_10260274	Ca_kabuli_chr06	10260274	[C/T]	1067299530
Ca_Kabuli_NC_021165.1_10315009	Ca_kabuli_chr06	10315009	[A/T]	1067299531
Ca_Kabuli_NC_021165.1_10395273	Ca_kabuli_chr06	10395273	[T/G]	1067299532
Ca_Kabuli_NC_021165.1_10395281	Ca_kabuli_chr06	10395281	[C/T]	1067299533
Ca_Kabuli_NC_021165.1_10441728	Ca_kabuli_chr06	10441728	[G/C]	1067299534
Ca_Kabuli_NC_021165.1_10441740	Ca_kabuli_chr06	10441740	[T/C]	1067299535
Ca_Kabuli_NC_021165.1_10441742	Ca_kabuli_chr06	10441742	[A/C]	1067299536
Ca_Kabuli_NC_021165.1_10441753	Ca_kabuli_chr06	10441753	[G/C]	1067299537
Ca_Kabuli_NC_021165.1_10448786	Ca_kabuli_chr06	10448786	[T/C]	1067299538
Ca_Kabuli_NC_021165.1_10457025	Ca_kabuli_chr06	10457025	[A/C]	1067299539
Ca_Kabuli_NC_021165.1_10502431	Ca_kabuli_chr06	10502431	[A/C]	1067299540
Ca_Kabuli_NC_021165.1_10627884	Ca_kabuli_chr06	10627884	[T/A]	1067299541
Ca_Kabuli_NC_021165.1_10627885	Ca_kabuli_chr06	10627885	[A/G]	1067299542
Ca_Kabuli_NC_021165.1_10670366	Ca_kabuli_chr06	10670366	[T/G]	1067299543
Ca_Kabuli_NC_021165.1_10670656	Ca_kabuli_chr06	10670656	[A/G]	1067299544

SNP IDs	Chromosomes	Physical positions (bp)	SNPs	NCBI SNPdb accession IDs (NCBI_ss#)
Ca_Kabuli_NC_021165.1_10670721	Ca_kabuli_chr06	10670721	[A/G]	1067299545
Ca_Kabuli_NC_021165.1_10670729	Ca_kabuli_chr06	10670729	[T/G]	1067299546
Ca_Kabuli_NC_021165.1_10670739	Ca_kabuli_chr06	10670739	[T/G]	1067299547
Ca_Kabuli_NC_021165.1_10670750	Ca_kabuli_chr06	10670750	[T/G]	1067299548
Ca_Kabuli_NC_021165.1_10670756	Ca_kabuli_chr06	10670756	[A/G]	1067299549
Ca_Kabuli_NC_021165.1_10671148	Ca_kabuli_chr06	10671148	[T/A]	1067299550
Ca_Kabuli_NC_021165.1_10672040	Ca_kabuli_chr06	10672040	[C/A]	1067299551
Ca_Kabuli_NC_021165.1_10672383	Ca_kabuli_chr06	10672383	[T/C]	1067299552
Ca_Kabuli_NC_021165.1_10677472	Ca_kabuli_chr06	10677472	[A/T]	1067299553
Ca_Kabuli_NC_021165.1_10677483	Ca_kabuli_chr06	10677483	[A/T]	1067299554
Ca_Kabuli_NC_021165.1_10682135	Ca_kabuli_chr06	10682135	[T/C]	1067299555
Ca_Kabuli_NC_021165.1_10684697	Ca_kabuli_chr06	10684697	[T/G]	1067299556
Ca_Kabuli_NC_021165.1_10684708	Ca_kabuli_chr06	10684708	[T/A]	1067299557
Ca_Kabuli_NC_021165.1_10684760	Ca_kabuli_chr06	10684760	[A/T]	1067299558
Ca_Kabuli_NC_021165.1_10698770	Ca_kabuli_chr06	10698770	[A/C]	1067299559
Ca_Kabuli_NC_021165.1_10698775	Ca_kabuli_chr06	10698775	[A/C]	1067299560
Ca_Kabuli_NC_021165.1_10698787	Ca_kabuli_chr06	10698787	[A/C]	1067299561
Ca_Kabuli_NC_021165.1_10698795	Ca_kabuli_chr06	10698795	[T/C]	1067299562
Ca_Kabuli_NC_021165.1_10698802	Ca_kabuli_chr06	10698802	[A/C]	1067299563
Ca_Kabuli_NC_021165.1_10716949	Ca_kabuli_chr06	10716949	[T/C]	1067299564
Ca_Kabuli_NC_021165.1_10716960	Ca_kabuli_chr06	10716960	[T/C]	1067299565
Ca_Kabuli_NC_021165.1_10717005	Ca_kabuli_chr06	10717005	[A/G]	1067299566
Ca_Kabuli_NC_021165.1_10743996	Ca_kabuli_chr06	10743996	[A/T]	1067299567
Ca_Kabuli_NC_021165.1_10744029	Ca_kabuli_chr06	10744029	[C/A]	1067299568
Ca_Kabuli_NC_021165.1_10748661	Ca_kabuli_chr06	10748661	[A/C]	1067299569
Ca_Kabuli_NC_021165.1_10748746	Ca_kabuli_chr06	10748746	[A/T]	1067299570
Ca_Kabuli_NC_021165.1_10748766	Ca_kabuli_chr06	10748766	[T/C]	1067299571
Ca_Kabuli_NC_021165.1_10778519	Ca_kabuli_chr06	10778519	[T/C]	1067299572
Ca_Kabuli_NC_021165.1_10779344	Ca_kabuli_chr06	10779344	[T/G]	1067299573
Ca_Kabuli_NC_021165.1_10779545	Ca_kabuli_chr06	10779545	[T/A]	1067299574
Ca_Kabuli_NC_021165.1_10779837	Ca_kabuli_chr06	10779837	[G/A]	1067299575
Ca_Kabuli_NC_021165.1_10780228	Ca_kabuli_chr06	10780228	[C/T]	1067299576
Ca_Kabuli_NC_021165.1_10780240	Ca_kabuli_chr06	10780240	[T/C]	1067299577
Ca_Kabuli_NC_021165.1_10781242	Ca_kabuli_chr06	10781242	[C/T]	1067299578

SNP IDs	Chromosomes	Physical positions (bp)	SNPs	NCBI SNPdb accession IDs (NCBI_ss#)
Ca_Kabuli_NC_021165.1_10781281	Ca_kabuli_chr06	10781281	[T/C]	1067299579
Ca_Kabuli_NC_021165.1_10781522	Ca_kabuli_chr06	10781522	[G/T]	1067299580
Ca_Kabuli_NC_021165.1_10785458	Ca_kabuli_chr06	10785458	[T/A]	1067299581
Ca_Kabuli_NC_021165.1_10785459	Ca_kabuli_chr06	10785459	[G/A]	1067299582
Ca_Kabuli_NC_021165.1_10785480	Ca_kabuli_chr06	10785480	[T/G]	1067299583
Ca_Kabuli_NC_021165.1_10790133	Ca_kabuli_chr06	10790133	[G/T]	1067299584
Ca_Kabuli_NC_021165.1_10806974	Ca_kabuli_chr06	10806974	[A/G]	1067299585
Ca_Kabuli_NC_021165.1_10806982	Ca_kabuli_chr06	10806982	[C/A]	1067299586
Ca_Kabuli_NC_021165.1_10870187	Ca_kabuli_chr06	10870187	[T/G]	1067299587
Ca_Kabuli_NC_021165.1_10888513	Ca_kabuli_chr06	10888513	[A/C]	1067299588
Ca_Kabuli_NC_021165.1_10888520	Ca_kabuli_chr06	10888520	[A/G]	1067299589
Ca_Kabuli_NC_021165.1_10991654	Ca_kabuli_chr06	10991654	[A/G]	1067299590
Ca_Kabuli_NC_021165.1_10991658	Ca_kabuli_chr06	10991658	[T/C]	1067299591
Ca_Kabuli_NC_021165.1_10991667	Ca_kabuli_chr06	10991667	[T/C]	1067299592
Ca_Kabuli_NC_021165.1_10991717	Ca_kabuli_chr06	10991717	[T/A]	1067299593
Ca_Kabuli_NC_021165.1_11011835	Ca_kabuli_chr06	11011835	[A/G]	1067299594
Ca_Kabuli_NC_021165.1_11011859	Ca_kabuli_chr06	11011859	[A/G]	1067299595
Ca_Kabuli_NC_021165.1_11038020	Ca_kabuli_chr06	11038020	[T/C]	1067299596
Ca_Kabuli_NC_021165.1_11064134	Ca_kabuli_chr06	11064134	[G/A]	1067299597
Ca_Kabuli_NC_021165.1_11069554	Ca_kabuli_chr06	11069554	[C/T]	1067299598
Ca_Kabuli_NC_021165.1_11098001	Ca_kabuli_chr06	11098001	[G/A]	1067299599
Ca_Kabuli_NC_021165.1_11105367	Ca_kabuli_chr06	11105367	[G/A]	1067299600
Ca_Kabuli_NC_021165.1_11106776	Ca_kabuli_chr06	11106776	[C/G]	1067299601
Ca_Kabuli_NC_021165.1_11128164	Ca_kabuli_chr06	11128164	[G/T]	1067299602
Ca_Kabuli_NC_021165.1_11131010	Ca_kabuli_chr06	11131010	[T/A]	1067299603
Ca_Kabuli_NC_021165.1_11131020	Ca_kabuli_chr06	11131020	[A/G]	1067299604
Ca_Kabuli_NC_021165.1_11163679	Ca_kabuli_chr06	11163679	[T/C]	1067299605
Ca_Kabuli_NC_021165.1_11163693	Ca_kabuli_chr06	11163693	[A/G]	1067299606
Ca_Kabuli_NC_021165.1_11163698	Ca_kabuli_chr06	11163698	[T/C]	1067299607
Ca_Kabuli_NC_021165.1_11163711	Ca_kabuli_chr06	11163711	[T/G]	1067299608
Ca_Kabuli_NC_021165.1_11163717	Ca_kabuli_chr06	11163717	[T/A]	1067299609
Ca_Kabuli_NC_021165.1_11163847	Ca_kabuli_chr06	11163847	[A/C]	1067299610
Ca_Kabuli_NC_021165.1_11180702	Ca_kabuli_chr06	11180702	[G/A]	1067299611
Ca_Kabuli_NC_021165.1_11185980	Ca_kabuli_chr06	11185980	[T/C]	1067299612

SNP IDs	Chromosomes	Physical positions (bp)	SNPs	NCBI SNPdb accession IDs (NCBI_ss#)
Ca_Kabuli_NC_021165.1_11188781	Ca_kabuli_chr06	11188781	[T/G]	1067299613
Ca_Kabuli_NC_021165.1_11188803	Ca_kabuli_chr06	11188803	[G/A]	1067299614
Ca_Kabuli_NC_021165.1_11189051	Ca_kabuli_chr06	11189051	[A/G]	1067299615
Ca_Kabuli_NC_021165.1_11191321	Ca_kabuli_chr06	11191321	[T/C]	1067299616
Ca_Kabuli_NC_021165.1_11191339	Ca_kabuli_chr06	11191339	[A/G]	1067299617
Ca_Kabuli_NC_021165.1_11285914	Ca_kabuli_chr06	11285914	[A/C]	1067299618
Ca_Kabuli_NC_021165.1_11302033	Ca_kabuli_chr06	11302033	[T/C]	1067299619
Ca_Kabuli_NC_021165.1_11302037	Ca_kabuli_chr06	11302037	[A/C]	1067299620
Ca_Kabuli_NC_021165.1_11302041	Ca_kabuli_chr06	11302041	[T/C]	1067299621
Ca_Kabuli_NC_021165.1_11302061	Ca_kabuli_chr06	11302061	[T/C]	1067299622
Ca_Kabuli_NC_021165.1_11354898	Ca_kabuli_chr06	11354898	[A/C]	1067299623
Ca_Kabuli_NC_021165.1_11394801	Ca_kabuli_chr06	11394801	[T/G]	1067299624
Ca_Kabuli_NC_021165.1_11399318	Ca_kabuli_chr06	11399318	[T/C]	1067299625
Ca_Kabuli_NC_021165.1_11508824	Ca_kabuli_chr06	11508824	[T/G]	1067299626
Ca_Kabuli_NC_021165.1_11508970	Ca_kabuli_chr06	11508970	[G/A]	1067299627
Ca_Kabuli_NC_021165.1_11508975	Ca_kabuli_chr06	11508975	[A/C]	1067299628
Ca_Kabuli_NC_021165.1_11543618	Ca_kabuli_chr06	11543618	[T/G]	1067299629
Ca_Kabuli_NC_021165.1_11659814	Ca_kabuli_chr06	11659814	[G/A]	1067299630
Ca_Kabuli_NC_021165.1_11659981	Ca_kabuli_chr06	11659981	[G/A]	1067299631
Ca_Kabuli_NC_021165.1_11665233	Ca_kabuli_chr06	11665233	[A/G]	1067299632
Ca_Kabuli_NC_021165.1_11665262	Ca_kabuli_chr06	11665262	[T/G]	1067299633
Ca_Kabuli_NC_021165.1_11665268	Ca_kabuli_chr06	11665268	[T/G]	1067299634
Ca_Kabuli_NC_021165.1_11665329	Ca_kabuli_chr06	11665329	[T/G]	1067299635
Ca_Kabuli_NC_021165.1_11665338	Ca_kabuli_chr06	11665338	[T/G]	1067299636
Ca_Kabuli_NC_021165.1_11668281	Ca_kabuli_chr06	11668281	[T/C]	1067299637
Ca_Kabuli_NC_021165.1_12035683	Ca_kabuli_chr06	12035683	[A/G]	1067299638
Ca_Kabuli_NC_021165.1_12035718	Ca_kabuli_chr06	12035718	[T/G]	1067299639
Ca_Kabuli_NC_021165.1_12435077	Ca_kabuli_chr06	12435077	[A/C]	1067299640
Ca_Kabuli_NC_021165.1_12436984	Ca_kabuli_chr06	12436984	[A/G]	1067299641
Ca_Kabuli_NC_021165.1_12437022	Ca_kabuli_chr06	12437022	[A/C]	1067299642
Ca_Kabuli_NC_021165.1_12437029	Ca_kabuli_chr06	12437029	[A/T]	1067299643
Ca_Kabuli_NC_021165.1_12437088	Ca_kabuli_chr06	12437088	[A/C]	1067299644
Ca_Kabuli_NC_021165.1_12437132	Ca_kabuli_chr06	12437132	[A/C]	1067299645
Ca_Kabuli_NC_021165.1_12448511	Ca_kabuli_chr06	12448511	[A/C]	1067299646

SNP IDs	Chromosomes	Physical positions (bp)	SNPs	NCBI SNPdb accession IDs (NCBI_ss#)
Ca_Kabuli_NC_021165.1_12448541	Ca_kabuli_chr06	12448541	[A/G]	1067299647
Ca_Kabuli_NC_021165.1_12490145	Ca_kabuli_chr06	12490145	[A/T]	1067299648
Ca_Kabuli_NC_021165.1_12535717	Ca_kabuli_chr06	12535717	[T/G]	1067299649
Ca_Kabuli_NC_021165.1_12535741	Ca_kabuli_chr06	12535741	[A/G]	1067299650
Ca_Kabuli_NC_021165.1_12775930	Ca_kabuli_chr06	12775930	[T/C]	1067299651
Ca_Kabuli_NC_021165.1_12775985	Ca_kabuli_chr06	12775985	[A/C]	1067299652
Ca_Kabuli_NC_021165.1_12799817	Ca_kabuli_chr06	12799817	[T/A]	1067299653
Ca_Kabuli_NC_021165.1_12799894	Ca_kabuli_chr06	12799894	[G/A]	1067299654
Ca_Kabuli_NC_021165.1_12799931	Ca_kabuli_chr06	12799931	[A/G]	1067299655
Ca_Kabuli_NC_021165.1_12804358	Ca_kabuli_chr06	12804358	[T/G]	1067299656
Ca_Kabuli_NC_021165.1_12810876	Ca_kabuli_chr06	12810876	[C/A]	1067299657
Ca_Kabuli_NC_021165.1_12810877	Ca_kabuli_chr06	12810877	[T/G]	1067299658
Ca_Kabuli_NC_021165.1_12817997	Ca_kabuli_chr06	12817997	[A/G]	1067299659
Ca_Kabuli_NC_021165.1_12818032	Ca_kabuli_chr06	12818032	[A/T]	1067299660
Ca_Kabuli_NC_021165.1_12844592	Ca_kabuli_chr06	12844592	[T/G]	1067299661
Ca_Kabuli_NC_021165.1_12882524	Ca_kabuli_chr06	12882524	[A/G]	1067299662
Ca_Kabuli_NC_021165.1_12939911	Ca_kabuli_chr06	12939911	[T/A]	1067299663
Ca_Kabuli_NC_021165.1_12939913	Ca_kabuli_chr06	12939913	[A/G]	1067299664
Ca_Kabuli_NC_021165.1_12939937	Ca_kabuli_chr06	12939937	[T/C]	1067299665
Ca_Kabuli_NC_021165.1_12939946	Ca_kabuli_chr06	12939946	[A/G]	1067299666
Ca_Kabuli_NC_021165.1_12939964	Ca_kabuli_chr06	12939964	[T/C]	1067299667
Ca_Kabuli_NC_021165.1_12941023	Ca_kabuli_chr06	12941023	[T/C]	1067299668
Ca_Kabuli_NC_021165.1_12976242	Ca_kabuli_chr06	12976242	[T/C]	1067299669
Ca_Kabuli_NC_021165.1_12985910	Ca_kabuli_chr06	12985910	[C/T]	1067299670
Ca_Kabuli_NC_021165.1_12986826	Ca_kabuli_chr06	12986826	[G/A]	1067299671
Ca_Kabuli_NC_021165.1_12986829	Ca_kabuli_chr06	12986829	[T/G]	1067299672
Ca_Kabuli_NC_021165.1_12986833	Ca_kabuli_chr06	12986833	[A/T]	1067299673
Ca_Kabuli_NC_021165.1_13073096	Ca_kabuli_chr06	13073096	[T/G]	1067299674
Ca_Kabuli_NC_021165.1_13107254	Ca_kabuli_chr06	13107254	[T/C]	1067299675
Ca_Kabuli_NC_021165.1_13107256	Ca_kabuli_chr06	13107256	[T/C]	1067299676
Ca_Kabuli_NC_021165.1_13107278	Ca_kabuli_chr06	13107278	[T/A]	1067299677
Ca_Kabuli_NC_021165.1_13107289	Ca_kabuli_chr06	13107289	[T/C]	1067299678
Ca_Kabuli_NC_021165.1_13107293	Ca_kabuli_chr06	13107293	[T/C]	1067299679
Ca_Kabuli_NC_021165.1_13107304	Ca_kabuli_chr06	13107304	[T/C]	1067299680

SNP IDs	Chromosomes	Physical positions (bp)	SNPs	NCBI SNPdb accession IDs (NCBI_ss#)
Ca_Kabuli_NC_021165.1_13123074	Ca_kabuli_chr06	13123074	[A/C]	1067299681
Ca_Kabuli_NC_021165.1_13131578	Ca_kabuli_chr06	13131578	[C/A]	1067299682
Ca_Kabuli_NC_021165.1_13136818	Ca_kabuli_chr06	13136818	[T/C]	1067299683
Ca_Kabuli_NC_021165.1_13136830	Ca_kabuli_chr06	13136830	[T/C]	1067299684
Ca_Kabuli_NC_021165.1_13192458	Ca_kabuli_chr06	13192458	[T/G]	1067299685
Ca_Kabuli_NC_021165.1_13201407	Ca_kabuli_chr06	13201407	[T/A]	1067299686
Ca_Kabuli_NC_021165.1_13221837	Ca_kabuli_chr06	13221837	[T/A]	1067299687
Ca_Kabuli_NC_021165.1_13221861	Ca_kabuli_chr06	13221861	[T/A]	1067299688
Ca_Kabuli_NC_021165.1_13221873	Ca_kabuli_chr06	13221873	[T/G]	1067299689
Ca_Kabuli_NC_021165.1_13222255	Ca_kabuli_chr06	13222255	[G/T]	1067299690
Ca_Kabuli_NC_021165.1_13222256	Ca_kabuli_chr06	13222256	[T/C]	1067299691
Ca_Kabuli_NC_021165.1_13222257	Ca_kabuli_chr06	13222257	[G/T]	1067299692
Ca_Kabuli_NC_021165.1_13244666	Ca_kabuli_chr06	13244666	[A/G]	1067299693
Ca_Kabuli_NC_021165.1_13244668	Ca_kabuli_chr06	13244668	[C/G]	1067299694
Ca_Kabuli_NC_021165.1_13244680	Ca_kabuli_chr06	13244680	[T/G]	1067299695
Ca_Kabuli_NC_021165.1_13296123	Ca_kabuli_chr06	13296123	[A/G]	1067299696
Ca_Kabuli_NC_021165.1_13300383	Ca_kabuli_chr06	13300383	[A/G]	1067299697
Ca_Kabuli_NC_021165.1_13340791	Ca_kabuli_chr06	13340791	[T/A]	1067299698
Ca_Kabuli_NC_021165.1_13483860	Ca_kabuli_chr06	13483860	[T/C]	1067299699
Ca_Kabuli_NC_021165.1_13483911	Ca_kabuli_chr06	13483911	[A/G]	1067299700
Ca_Kabuli_NC_021165.1_13569263	Ca_kabuli_chr06	13569263	[A/T]	1067299701
Ca_Kabuli_NC_021165.1_13569291	Ca_kabuli_chr06	13569291	[A/C]	1067299702
Ca_Kabuli_NC_021165.1_13572221	Ca_kabuli_chr06	13572221	[A/C]	1067299703
Ca_Kabuli_NC_021165.1_13575227	Ca_kabuli_chr06	13575227	[T/A]	1067299704
Ca_Kabuli_NC_021165.1_13628325	Ca_kabuli_chr06	13628325	[A/C]	1067299705
Ca_Kabuli_NC_021165.1_13628328	Ca_kabuli_chr06	13628328	[A/C]	1067299706
Ca_Kabuli_NC_021165.1_13628426	Ca_kabuli_chr06	13628426	[T/C]	1067299707
Ca_Kabuli_NC_021165.1_13628532	Ca_kabuli_chr06	13628532	[A/C]	1067299708
Ca_Kabuli_NC_021165.1_13637388	Ca_kabuli_chr06	13637388	[A/C]	1067299709
Ca_Kabuli_NC_021165.1_13637392	Ca_kabuli_chr06	13637392	[A/C]	1067299710
Ca_Kabuli_NC_021165.1_13639434	Ca_kabuli_chr06	13639434	[T/G]	1067299711
Ca_Kabuli_NC_021165.1_13685747	Ca_kabuli_chr06	13685747	[T/C]	1067299712
Ca_Kabuli_NC_021165.1_13714842	Ca_kabuli_chr06	13714842	[T/G]	1067299713
Ca_Kabuli_NC_021165.1_13714893	Ca_kabuli_chr06	13714893	[A/G]	1067299714

SNP IDs	Chromosomes	Physical positions (bp)	SNPs	NCBI SNPdb accession IDs (NCBI_ss#)
Ca_Kabuli_NC_021165.1_13826905	Ca_kabuli_chr06	13826905	[T/G]	1067299715
Ca_Kabuli_NC_021165.1_13913516	Ca_kabuli_chr06	13913516	[A/T]	1067299716
Ca_Kabuli_NC_021165.1_13917083	Ca_kabuli_chr06	13917083	[T/C]	1067299717
Ca_Kabuli_NC_021165.1_13921926	Ca_kabuli_chr06	13921926	[T/G]	1067299718
Ca_Kabuli_NC_021165.1_13921954	Ca_kabuli_chr06	13921954	[A/C]	1067299719
Ca_Kabuli_NC_021165.1_13936416	Ca_kabuli_chr06	13936416	[T/C]	1067299720
Ca_Kabuli_NC_021165.1_13936517	Ca_kabuli_chr06	13936517	[A/C]	1067299721
Ca_Kabuli_NC_021165.1_13948739	Ca_kabuli_chr06	13948739	[G/A]	1067299722
Ca_Kabuli_NC_021165.1_13948742	Ca_kabuli_chr06	13948742	[T/G]	1067299723
Ca_Kabuli_NC_021165.1_13986526	Ca_kabuli_chr06	13986526	[T/G]	1067299724
Ca_Kabuli_NC_021165.1_14094683	Ca_kabuli_chr06	14094683	[C/G]	1067299725
Ca_Kabuli_NC_021165.1_14107024	Ca_kabuli_chr06	14107024	[C/G]	1067299726
Ca_Kabuli_NC_021165.1_14107039	Ca_kabuli_chr06	14107039	[T/G]	1067299727
Ca_Kabuli_NC_021165.1_14107066	Ca_kabuli_chr06	14107066	[A/G]	1067299728
Ca_Kabuli_NC_021165.1_14107082	Ca_kabuli_chr06	14107082	[T/C]	1067299729
Ca_Kabuli_NC_021165.1_14107089	Ca_kabuli_chr06	14107089	[G/C]	1067299730
Ca_Kabuli_NC_021165.1_14141852	Ca_kabuli_chr06	14141852	[C/A]	1067299731
Ca_Kabuli_NC_021165.1_14141855	Ca_kabuli_chr06	14141855	[T/C]	1067299732
Ca_Kabuli_NC_021165.1_14141858	Ca_kabuli_chr06	14141858	[C/T]	1067299733
Ca_Kabuli_NC_021165.1_14148742	Ca_kabuli_chr06	14148742	[T/G]	1067299734
Ca_Kabuli_NC_021165.1_14160308	Ca_kabuli_chr06	14160308	[A/C]	1067299735
Ca_Kabuli_NC_021165.1_14160310	Ca_kabuli_chr06	14160310	[A/C]	1067299736
Ca_Kabuli_NC_021165.1_14200976	Ca_kabuli_chr06	14200976	[C/A]	1067299737
Ca_Kabuli_NC_021165.1_14200999	Ca_kabuli_chr06	14200999	[T/A]	1067299738
Ca_Kabuli_NC_021165.1_14255107	Ca_kabuli_chr06	14255107	[A/C]	1067299739
Ca_Kabuli_NC_021165.1_14309365	Ca_kabuli_chr06	14309365	[T/C]	1067299740
Ca_Kabuli_NC_021165.1_14309390	Ca_kabuli_chr06	14309390	[C/G]	1067299741
Ca_Kabuli_NC_021165.1_14309393	Ca_kabuli_chr06	14309393	[A/G]	1067299742
Ca_Kabuli_NC_021165.1_14332018	Ca_kabuli_chr06	14332018	[A/C]	1067299743
Ca_Kabuli_NC_021165.1_14353624	Ca_kabuli_chr06	14353624	[G/A]	1067299744
Ca_Kabuli_NC_021165.1_14371549	Ca_kabuli_chr06	14371549	[C/A]	1067299745
Ca_Kabuli_NC_021165.1_14371811	Ca_kabuli_chr06	14371811	[T/G]	1067299746
Ca_Kabuli_NC_021165.1_14371814	Ca_kabuli_chr06	14371814	[T/C]	1067299747
Ca_Kabuli_NC_021165.1_14371900	Ca_kabuli_chr06	14371900	[T/C]	1067299748

SNP IDs	Chromosomes	Physical positions (bp)	SNPs	NCBI SNPdb accession IDs (NCBI_ss#)
Ca_Kabuli_NC_021165.1_14416997	Ca_kabuli_chr06	14416997	[A/T]	1067299749
Ca_Kabuli_NC_021165.1_14419837	Ca_kabuli_chr06	14419837	[T/C]	1067299750
Ca_Kabuli_NC_021165.1_14419867	Ca_kabuli_chr06	14419867	[A/G]	1067299751
Ca_Kabuli_NC_021165.1_14419875	Ca_kabuli_chr06	14419875	[A/C]	1067299752
Ca_Kabuli_NC_021165.1_14419877	Ca_kabuli_chr06	14419877	[A/C]	1067299753
Ca_Kabuli_NC_021165.1_14421756	Ca_kabuli_chr06	14421756	[A/T]	1067299754
Ca_Kabuli_NC_021165.1_14421787	Ca_kabuli_chr06	14421787	[T/C]	1067299755
Ca_Kabuli_NC_021165.1_14421816	Ca_kabuli_chr06	14421816	[T/A]	1067299756
Ca_Kabuli_NC_021165.1_14526395	Ca_kabuli_chr06	14526395	[T/G]	1067299757
Ca_Kabuli_NC_021165.1_14606892	Ca_kabuli_chr06	14606892	[A/C]	1067299758
Ca_Kabuli_NC_021165.1_14606941	Ca_kabuli_chr06	14606941	[A/T]	1067299759
Ca_Kabuli_NC_021165.1_14606945	Ca_kabuli_chr06	14606945	[A/G]	1067299760
Ca_Kabuli_NC_021165.1_14630065	Ca_kabuli_chr06	14630065	[A/G]	1067299761
Ca_Kabuli_NC_021165.1_14630076	Ca_kabuli_chr06	14630076	[A/T]	1067299762
Ca_Kabuli_NC_021165.1_14630632	Ca_kabuli_chr06	14630632	[A/C]	1067299763
Ca_Kabuli_NC_021165.1_14691728	Ca_kabuli_chr06	14691728	[T/A]	1067299764
Ca_Kabuli_NC_021165.1_14719712	Ca_kabuli_chr06	14719712	[A/C]	1067299765
Ca_Kabuli_NC_021165.1_14719713	Ca_kabuli_chr06	14719713	[C/A]	1067299766
Ca_Kabuli_NC_021165.1_14719715	Ca_kabuli_chr06	14719715	[T/G]	1067299767
Ca_Kabuli_NC_021165.1_14719725	Ca_kabuli_chr06	14719725	[A/G]	1067299768
Ca_Kabuli_NC_021165.1_14719734	Ca_kabuli_chr06	14719734	[T/G]	1067299769
Ca_Kabuli_NC_021165.1_14719909	Ca_kabuli_chr06	14719909	[T/C]	1067299770
Ca_Kabuli_NC_021165.1_14719910	Ca_kabuli_chr06	14719910	[G/T]	1067299771
Ca_Kabuli_NC_021165.1_14769691	Ca_kabuli_chr06	14769691	[A/C]	1067299772
Ca_Kabuli_NC_021165.1_14769694	Ca_kabuli_chr06	14769694	[T/C]	1067299773
Ca_Kabuli_NC_021165.1_14769697	Ca_kabuli_chr06	14769697	[A/C]	1067299774
Ca_Kabuli_NC_021165.1_14769709	Ca_kabuli_chr06	14769709	[A/T]	1067299775
Ca_Kabuli_NC_021165.1_14771602	Ca_kabuli_chr06	14771602	[T/G]	1067299776
Ca_Kabuli_NC_021165.1_14857680	Ca_kabuli_chr06	14857680	[G/A]	1067299777
Ca_Kabuli_NC_021165.1_14971493	Ca_kabuli_chr06	14971493	[A/G]	1067299778
Ca_Kabuli_NC_021165.1_14971517	Ca_kabuli_chr06	14971517	[A/G]	1067299779
Ca_Kabuli_NC_021165.1_14991776	Ca_kabuli_chr06	14991776	[A/C]	1067299780
Ca_Kabuli_NC_021165.1_15019327	Ca_kabuli_chr06	15019327	[A/C]	1067299781
Ca_Kabuli_NC_021165.1_15024869	Ca_kabuli_chr06	15024869	[T/C]	1067299782

SNP IDs	Chromosomes	Physical positions (bp)	SNPs	NCBI SNPdb accession IDs (NCBI_ss#)
Ca_Kabuli_NC_021165.1_15024875	Ca_kabuli_chr06	15024875	[T/C]	1067299783
Ca_Kabuli_NC_021165.1_15024910	Ca_kabuli_chr06	15024910	[A/T]	1067299784
Ca_Kabuli_NC_021165.1_15024946	Ca_kabuli_chr06	15024946	[T/C]	1067299785
Ca_Kabuli_NC_021165.1_15056600	Ca_kabuli_chr06	15056600	[T/G]	1067299786
Ca_Kabuli_NC_021165.1_15056615	Ca_kabuli_chr06	15056615	[A/G]	1067299787
Ca_Kabuli_NC_021165.1_15061714	Ca_kabuli_chr06	15061714	[T/C]	1067299788
Ca_Kabuli_NC_021165.1_15061743	Ca_kabuli_chr06	15061743	[A/C]	1067299789
Ca_Kabuli_NC_021165.1_15061763	Ca_kabuli_chr06	15061763	[T/C]	1067299790
Ca_Kabuli_NC_021165.1_15093578	Ca_kabuli_chr06	15093578	[T/G]	1067299791
Ca_Kabuli_NC_021165.1_15123030	Ca_kabuli_chr06	15123030	[T/A]	1067299792
Ca_Kabuli_NC_021165.1_15123033	Ca_kabuli_chr06	15123033	[C/T]	1067299793
Ca_Kabuli_NC_021165.1_15123051	Ca_kabuli_chr06	15123051	[C/T]	1067299794
Ca_Kabuli_NC_021165.1_15123074	Ca_kabuli_chr06	15123074	[G/A]	1067299795
Ca_Kabuli_NC_021165.1_15123962	Ca_kabuli_chr06	15123962	[A/G]	1067299796
Ca_Kabuli_NC_021165.1_15133403	Ca_kabuli_chr06	15133403	[G/T]	1067299797
Ca_Kabuli_NC_021165.1_15133404	Ca_kabuli_chr06	15133404	[G/C]	1067299798
Ca_Kabuli_NC_021165.1_15133405	Ca_kabuli_chr06	15133405	[G/T]	1067299799
Ca_Kabuli_NC_021165.1_15168831	Ca_kabuli_chr06	15168831	[A/C]	1067299800
Ca_Kabuli_NC_021165.1_15204941	Ca_kabuli_chr06	15204941	[A/G]	1067299801
Ca_Kabuli_NC_021165.1_15235612	Ca_kabuli_chr06	15235612	[A/C]	1067299802
Ca_Kabuli_NC_021165.1_15301275	Ca_kabuli_chr06	15301275	[A/C]	1067299803
Ca_Kabuli_NC_021165.1_15306139	Ca_kabuli_chr06	15306139	[A/C]	1067299804
Ca_Kabuli_NC_021165.1_15306142	Ca_kabuli_chr06	15306142	[A/C]	1067299805
Ca_Kabuli_NC_021165.1_15313640	Ca_kabuli_chr06	15313640	[A/G]	1067299806
Ca_Kabuli_NC_021165.1_15386963	Ca_kabuli_chr06	15386963	[G/A]	1067299807
Ca_Kabuli_NC_021165.1_15451994	Ca_kabuli_chr06	15451994	[T/G]	1067299808
Ca_Kabuli_NC_021165.1_15463331	Ca_kabuli_chr06	15463331	[A/C]	1067299809
Ca_Kabuli_NC_021165.1_15463340	Ca_kabuli_chr06	15463340	[A/T]	1067299810
Ca_Kabuli_NC_021165.1_15463354	Ca_kabuli_chr06	15463354	[T/C]	1067299811
Ca_Kabuli_NC_021165.1_15537918	Ca_kabuli_chr06	15537918	[T/G]	1067299812
Ca_Kabuli_NC_021165.1_15537931	Ca_kabuli_chr06	15537931	[T/G]	1067299813
Ca_Kabuli_NC_021165.1_15543348	Ca_kabuli_chr06	15543348	[T/C]	1067299814
Ca_Kabuli_NC_021165.1_15544732	Ca_kabuli_chr06	15544732	[A/T]	1067299815
Ca_Kabuli_NC_021165.1_15589539	Ca_kabuli_chr06	15589539	[A/T]	1067299816

SNP IDs	Chromosomes	Physical positions (bp)	SNPs	NCBI SNPdb accession IDs (NCBI_ss#)
Ca_Kabuli_NC_021165.1_15749754	Ca_kabuli_chr06	15749754	[T/C]	1067299817
Ca_Kabuli_NC_021165.1_15749817	Ca_kabuli_chr06	15749817	[A/G]	1067299818
Ca_Kabuli_NC_021165.1_15845370	Ca_kabuli_chr06	15845370	[A/C]	1067299819
Ca_Kabuli_NC_021165.1_15846166	Ca_kabuli_chr06	15846166	[G/C]	1067299820
Ca_Kabuli_NC_021165.1_15857715	Ca_kabuli_chr06	15857715	[A/G]	1067299821
Ca_Kabuli_NC_021165.1_15861316	Ca_kabuli_chr06	15861316	[A/C]	1067299822
Ca_Kabuli_NC_021165.1_15876779	Ca_kabuli_chr06	15876779	[A/G]	1067299823
Ca_Kabuli_NC_021165.1_15879528	Ca_kabuli_chr06	15879528	[A/G]	1067299824
Ca_Kabuli_NC_021165.1_15879535	Ca_kabuli_chr06	15879535	[C/G]	1067299825
Ca_Kabuli_NC_021165.1_16174874	Ca_kabuli_chr06	16174874	[T/C]	1067299826
Ca_Kabuli_NC_021165.1_16175122	Ca_kabuli_chr06	16175122	[T/C]	1067299827
Ca_Kabuli_NC_021165.1_16175139	Ca_kabuli_chr06	16175139	[T/A]	1067299828
Ca_Kabuli_NC_021165.1_16175140	Ca_kabuli_chr06	16175140	[A/G]	1067299829
Ca_Kabuli_NC_021165.1_16175142	Ca_kabuli_chr06	16175142	[A/G]	1067299830
Ca_Kabuli_NC_021165.1_16189675	Ca_kabuli_chr06	16189675	[T/G]	1067299831
Ca_Kabuli_NC_021165.1_16189678	Ca_kabuli_chr06	16189678	[T/G]	1067299832
Ca_Kabuli_NC_021165.1_16196469	Ca_kabuli_chr06	16196469	[T/A]	1067299833
Ca_Kabuli_NC_021165.1_16196531	Ca_kabuli_chr06	16196531	[T/C]	1067299834
Ca_Kabuli_NC_021165.1_16196548	Ca_kabuli_chr06	16196548	[T/G]	1067299835
Ca_Kabuli_NC_021165.1_16196555	Ca_kabuli_chr06	16196555	[T/G]	1067299836
Ca_Kabuli_NC_021165.1_16203377	Ca_kabuli_chr06	16203377	[A/G]	1067299837
Ca_Kabuli_NC_021165.1_16321782	Ca_kabuli_chr06	16321782	[A/C]	1067299838
Ca_Kabuli_NC_021165.1_16460477	Ca_kabuli_chr06	16460477	[A/G]	1067299839
Ca_Kabuli_NC_021165.1_16460507	Ca_kabuli_chr06	16460507	[A/C]	1067299840
Ca_Kabuli_NC_021165.1_16460511	Ca_kabuli_chr06	16460511	[T/C]	1067299841
Ca_Kabuli_NC_021165.1_16463615	Ca_kabuli_chr06	16463615	[T/G]	1067299842
Ca_Kabuli_NC_021165.1_16463629	Ca_kabuli_chr06	16463629	[T/G]	1067299843
Ca_Kabuli_NC_021165.1_16549005	Ca_kabuli_chr06	16549005	[A/C]	1067299844
Ca_Kabuli_NC_021165.1_16549022	Ca_kabuli_chr06	16549022	[A/G]	1067299845
Ca_Kabuli_NC_021165.1_16549027	Ca_kabuli_chr06	16549027	[T/C]	1067299846
Ca_Kabuli_NC_021165.1_16549038	Ca_kabuli_chr06	16549038	[A/C]	1067299847
Ca_Kabuli_NC_021165.1_16560733	Ca_kabuli_chr06	16560733	[A/C]	1067299848
Ca_Kabuli_NC_021165.1_16560778	Ca_kabuli_chr06	16560778	[A/T]	1067299849
Ca_Kabuli_NC_021165.1_16560817	Ca_kabuli_chr06	16560817	[T/A]	1067299850

SNP IDs	Chromosomes	Physical positions (bp)	SNPs	NCBI SNPdb accession IDs (NCBI_ss#)
Ca_Kabuli_NC_021165.1_16560818	Ca_kabuli_chr06	16560818	[A/T]	1067299851
Ca_Kabuli_NC_021165.1_16633519	Ca_kabuli_chr06	16633519	[A/T]	1067299852
Ca_Kabuli_NC_021165.1_16633566	Ca_kabuli_chr06	16633566	[A/G]	1067299853
Ca_Kabuli_NC_021165.1_16637868	Ca_kabuli_chr06	16637868	[A/G]	1067299854
Ca_Kabuli_NC_021165.1_16646741	Ca_kabuli_chr06	16646741	[A/G]	1067299855
Ca_Kabuli_NC_021165.1_16646748	Ca_kabuli_chr06	16646748	[A/G]	1067299856
Ca_Kabuli_NC_021165.1_16646750	Ca_kabuli_chr06	16646750	[A/T]	1067299857
Ca_Kabuli_NC_021165.1_16646752	Ca_kabuli_chr06	16646752	[T/C]	1067299858
Ca_Kabuli_NC_021165.1_16646771	Ca_kabuli_chr06	16646771	[A/G]	1067299859
Ca_Kabuli_NC_021165.1_16646772	Ca_kabuli_chr06	16646772	[T/A]	1067299860
Ca_Kabuli_NC_021165.1_16646786	Ca_kabuli_chr06	16646786	[A/G]	1067299861
Ca_Kabuli_NC_021165.1_16670020	Ca_kabuli_chr06	16670020	[T/C]	1067299862
Ca_Kabuli_NC_021165.1_16685801	Ca_kabuli_chr06	16685801	[C/A]	1067299863
Ca_Kabuli_NC_021165.1_16685802	Ca_kabuli_chr06	16685802	[T/G]	1067299864
Ca_Kabuli_NC_021165.1_16685803	Ca_kabuli_chr06	16685803	[G/A]	1067299865
Ca_Kabuli_NC_021165.1_16685804	Ca_kabuli_chr06	16685804	[A/T]	1067299866
Ca_Kabuli_NC_021165.1_16707137	Ca_kabuli_chr06	16707137	[T/C]	1067299867
Ca_Kabuli_NC_021165.1_16715751	Ca_kabuli_chr06	16715751	[A/C]	1067299868
Ca_Kabuli_NC_021165.1_16715828	Ca_kabuli_chr06	16715828	[A/C]	1067299869
Ca_Kabuli_NC_021165.1_16715836	Ca_kabuli_chr06	16715836	[A/C]	1067299870
Ca_Kabuli_NC_021165.1_16719881	Ca_kabuli_chr06	16719881	[A/C]	1067299871
Ca_Kabuli_NC_021165.1_16719892	Ca_kabuli_chr06	16719892	[A/T]	1067299872
Ca_Kabuli_NC_021165.1_16748597	Ca_kabuli_chr06	16748597	[G/T]	1067299873
Ca_Kabuli_NC_021165.1_16748605	Ca_kabuli_chr06	16748605	[T/C]	1067299874
Ca_Kabuli_NC_021165.1_16823947	Ca_kabuli_chr06	16823947	[G/A]	1067299875
Ca_Kabuli_NC_021165.1_17007475	Ca_kabuli_chr06	17007475	[A/G]	1067299876
Ca_Kabuli_NC_021165.1_17007478	Ca_kabuli_chr06	17007478	[A/T]	1067299877
Ca_Kabuli_NC_021165.1_17007488	Ca_kabuli_chr06	17007488	[T/C]	1067299878
Ca_Kabuli_NC_021165.1_17007516	Ca_kabuli_chr06	17007516	[A/G]	1067299879
Ca_Kabuli_NC_021165.1_17007518	Ca_kabuli_chr06	17007518	[A/G]	1067299880
Ca_Kabuli_NC_021165.1_17026983	Ca_kabuli_chr06	17026983	[A/T]	1067299881
Ca_Kabuli_NC_021165.1_17027023	Ca_kabuli_chr06	17027023	[G/T]	1067299882
Ca_Kabuli_NC_021165.1_17141426	Ca_kabuli_chr06	17141426	[A/G]	1067299883
Ca_Kabuli_NC_021165.1_17141440	Ca_kabuli_chr06	17141440	[A/C]	1067299884

SNP IDs	Chromosomes	Physical positions (bp)	SNPs	NCBI SNPdb accession IDs (NCBI_ss#)
Ca_Kabuli_NC_021165.1_17263206	Ca_kabuli_chr06	17263206	[T/C]	1067299885
Ca_Kabuli_NC_021165.1_17263231	Ca_kabuli_chr06	17263231	[T/G]	1067299886
Ca_Kabuli_NC_021165.1_17307671	Ca_kabuli_chr06	17307671	[A/T]	1067299887
Ca_Kabuli_NC_021165.1_17307672	Ca_kabuli_chr06	17307672	[T/C]	1067299888
Ca_Kabuli_NC_021165.1_17307673	Ca_kabuli_chr06	17307673	[G/T]	1067299889
Ca_Kabuli_NC_021165.1_17353399	Ca_kabuli_chr06	17353399	[T/A]	1067299890
Ca_Kabuli_NC_021165.1_17353443	Ca_kabuli_chr06	17353443	[A/C]	1067299891
Ca_Kabuli_NC_021165.1_17369636	Ca_kabuli_chr06	17369636	[T/G]	1067299892
Ca_Kabuli_NC_021165.1_17369736	Ca_kabuli_chr06	17369736	[A/C]	1067299893
Ca_Kabuli_NC_021165.1_17477859	Ca_kabuli_chr06	17477859	[T/G]	1067299894
Ca_Kabuli_NC_021165.1_17481233	Ca_kabuli_chr06	17481233	[A/T]	1067299895
Ca_Kabuli_NC_021165.1_17541154	Ca_kabuli_chr06	17541154	[A/C]	1067299896
Ca_Kabuli_NC_021165.1_17541155	Ca_kabuli_chr06	17541155	[G/C]	1067299897
Ca_Kabuli_NC_021165.1_17561109	Ca_kabuli_chr06	17561109	[T/G]	1067299898
Ca_Kabuli_NC_021165.1_17573300	Ca_kabuli_chr06	17573300	[T/G]	1067299899
Ca_Kabuli_NC_021165.1_17588691	Ca_kabuli_chr06	17588691	[A/G]	1067299900
Ca_Kabuli_NC_021165.1_17588706	Ca_kabuli_chr06	17588706	[A/C]	1067299901
Ca_Kabuli_NC_021165.1_17658997	Ca_kabuli_chr06	17658997	[A/C]	1067299902
Ca_Kabuli_NC_021165.1_17659019	Ca_kabuli_chr06	17659019	[A/C]	1067299903
Ca_Kabuli_NC_021165.1_17725440	Ca_kabuli_chr06	17725440	[T/G]	1067299904
Ca_Kabuli_NC_021165.1_17725485	Ca_kabuli_chr06	17725485	[T/C]	1067299905
Ca_Kabuli_NC_021165.1_17726649	Ca_kabuli_chr06	17726649	[A/G]	1067299906
Ca_Kabuli_NC_021165.1_17727172	Ca_kabuli_chr06	17727172	[T/C]	1067299907
Ca_Kabuli_NC_021165.1_17730756	Ca_kabuli_chr06	17730756	[T/G]	1067299908
Ca_Kabuli_NC_021165.1_17730758	Ca_kabuli_chr06	17730758	[T/C]	1067299909
Ca_Kabuli_NC_021165.1_17730766	Ca_kabuli_chr06	17730766	[A/G]	1067299910
Ca_Kabuli_NC_021165.1_17730834	Ca_kabuli_chr06	17730834	[T/A]	1067299911
Ca_Kabuli_NC_021165.1_17730843	Ca_kabuli_chr06	17730843	[A/T]	1067299912
Ca_Kabuli_NC_021165.1_17735235	Ca_kabuli_chr06	17735235	[T/C]	1067299913
Ca_Kabuli_NC_021165.1_17740978	Ca_kabuli_chr06	17740978	[T/C]	1067299914
Ca_Kabuli_NC_021165.1_17741048	Ca_kabuli_chr06	17741048	[A/C]	1067299915
Ca_Kabuli_NC_021165.1_17741054	Ca_kabuli_chr06	17741054	[A/G]	1067299916
Ca_Kabuli_NC_021165.1_17741070	Ca_kabuli_chr06	17741070	[A/G]	1067299917
Ca_Kabuli_NC_021165.1_17751394	Ca_kabuli_chr06	17751394	[A/G]	1067299918

SNP IDs	Chromosomes	Physical positions (bp)	SNPs	NCBI SNPdb accession IDs (NCBI_ss#)
Ca_Kabuli_NC_021165.1_17751410	Ca_kabuli_chr06	17751410	[T/G]	1067299919
Ca_Kabuli_NC_021165.1_17787977	Ca_kabuli_chr06	17787977	[T/G]	1067299920
Ca_Kabuli_NC_021165.1_17788028	Ca_kabuli_chr06	17788028	[T/C]	1067299921
Ca_Kabuli_NC_021165.1_17788032	Ca_kabuli_chr06	17788032	[A/G]	1067299922
Ca_Kabuli_NC_021165.1_17788567	Ca_kabuli_chr06	17788567	[A/C]	1067299923
Ca_Kabuli_NC_021165.1_17788616	Ca_kabuli_chr06	17788616	[T/G]	1067299924
Ca_Kabuli_NC_021165.1_17788618	Ca_kabuli_chr06	17788618	[A/T]	1067299925
Ca_Kabuli_NC_021165.1_17788626	Ca_kabuli_chr06	17788626	[T/G]	1067299926
Ca_Kabuli_NC_021165.1_17788701	Ca_kabuli_chr06	17788701	[T/C]	1067299927
Ca_Kabuli_NC_021165.1_17863974	Ca_kabuli_chr06	17863974	[T/C]	1067299928
Ca_Kabuli_NC_021165.1_17897931	Ca_kabuli_chr06	17897931	[G/A]	1067299929
Ca_Kabuli_NC_021165.1_17897932	Ca_kabuli_chr06	17897932	[A/T]	1067299930
Ca_Kabuli_NC_021165.1_17897933	Ca_kabuli_chr06	17897933	[G/C]	1067299931
Ca_Kabuli_NC_021165.1_17897934	Ca_kabuli_chr06	17897934	[G/T]	1067299932
Ca_Kabuli_NC_021165.1_17985440	Ca_kabuli_chr06	17985440	[A/G]	1067299933
Ca_Kabuli_NC_021165.1_17985446	Ca_kabuli_chr06	17985446	[C/T]	1067299934
Ca_Kabuli_NC_021165.1_18026451	Ca_kabuli_chr06	18026451	[A/G]	1067299935
Ca_Kabuli_NC_021165.1_18026522	Ca_kabuli_chr06	18026522	[T/C]	1067299936
Ca_Kabuli_NC_021165.1_18026542	Ca_kabuli_chr06	18026542	[T/C]	1067299937
Ca_Kabuli_NC_021165.1_18094667	Ca_kabuli_chr06	18094667	[A/G]	1067299938
Ca_Kabuli_NC_021165.1_18125425	Ca_kabuli_chr06	18125425	[T/G]	1067299939
Ca_Kabuli_NC_021165.1_18125434	Ca_kabuli_chr06	18125434	[T/G]	1067299940
Ca_Kabuli_NC_021165.1_18125483	Ca_kabuli_chr06	18125483	[G/T]	1067299941
Ca_Kabuli_NC_021165.1_18233441	Ca_kabuli_chr06	18233441	[G/A]	1067299942
Ca_Kabuli_NC_021165.1_18233443	Ca_kabuli_chr06	18233443	[T/G]	1067299943
Ca_Kabuli_NC_021165.1_18264779	Ca_kabuli_chr06	18264779	[A/G]	1067299944
Ca_Kabuli_NC_021165.1_18389693	Ca_kabuli_chr06	18389693	[A/T]	1067299945
Ca_Kabuli_NC_021165.1_18389699	Ca_kabuli_chr06	18389699	[T/C]	1067299946
Ca_Kabuli_NC_021165.1_18389704	Ca_kabuli_chr06	18389704	[T/G]	1067299947
Ca_Kabuli_NC_021165.1_18629872	Ca_kabuli_chr06	18629872	[A/G]	1067299948
Ca_Kabuli_NC_021165.1_18747181	Ca_kabuli_chr06	18747181	[A/G]	1067299949
Ca_Kabuli_NC_021165.1_18747182	Ca_kabuli_chr06	18747182	[T/A]	1067299950
Ca_Kabuli_NC_021165.1_18747196	Ca_kabuli_chr06	18747196	[A/C]	1067299951
Ca_Kabuli_NC_021165.1_18747200	Ca_kabuli_chr06	18747200	[T/C]	1067299952

SNP IDs	Chromosomes	Physical positions (bp)	SNPs	NCBI SNPdb accession IDs (NCBI_ss#)
Ca_Kabuli_NC_021165.1_18768201	Ca_kabuli_chr06	18768201	[A/G]	1067299953
Ca_Kabuli_NC_021165.1_18771108	Ca_kabuli_chr06	18771108	[T/G]	1067299954
Ca_Kabuli_NC_021165.1_18772103	Ca_kabuli_chr06	18772103	[A/C]	1067299955
Ca_Kabuli_NC_021165.1_18852815	Ca_kabuli_chr06	18852815	[T/C]	1067299956
Ca_Kabuli_NC_021165.1_18852818	Ca_kabuli_chr06	18852818	[C/A]	1067299957
Ca_Kabuli_NC_021165.1_18852819	Ca_kabuli_chr06	18852819	[T/G]	1067299958
Ca_Kabuli_NC_021165.1_18852820	Ca_kabuli_chr06	18852820	[G/A]	1067299959
Ca_Kabuli_NC_021165.1_18852821	Ca_kabuli_chr06	18852821	[A/G]	1067299960
Ca_Kabuli_NC_021165.1_18852822	Ca_kabuli_chr06	18852822	[G/T]	1067299961
Ca_Kabuli_NC_021165.1_18852823	Ca_kabuli_chr06	18852823	[A/T]	1067299962
Ca_Kabuli_NC_021165.1_18852836	Ca_kabuli_chr06	18852836	[A/C]	1067299963
Ca_Kabuli_NC_021165.1_18852847	Ca_kabuli_chr06	18852847	[A/C]	1067299964
Ca_Kabuli_NC_021165.1_18916598	Ca_kabuli_chr06	18916598	[A/C]	1067299965
Ca_Kabuli_NC_021165.1_18916606	Ca_kabuli_chr06	18916606	[A/C]	1067299966
Ca_Kabuli_NC_021165.1_18922040	Ca_kabuli_chr06	18922040	[A/C]	1067299967
Ca_Kabuli_NC_021165.1_18924954	Ca_kabuli_chr06	18924954	[A/G]	1067299968
Ca_Kabuli_NC_021165.1_18924996	Ca_kabuli_chr06	18924996	[A/T]	1067299969
Ca_Kabuli_NC_021165.1_18938475	Ca_kabuli_chr06	18938475	[A/G]	1067299970
Ca_Kabuli_NC_021165.1_18938492	Ca_kabuli_chr06	18938492	[T/G]	1067299971
Ca_Kabuli_NC_021165.1_18953205	Ca_kabuli_chr06	18953205	[T/A]	1067299972
Ca_Kabuli_NC_021165.1_18953206	Ca_kabuli_chr06	18953206	[A/T]	1067299973
Ca_Kabuli_NC_021165.1_18953207	Ca_kabuli_chr06	18953207	[A/T]	1067299974
Ca_Kabuli_NC_021165.1_18953208	Ca_kabuli_chr06	18953208	[T/A]	1067299975
Ca_Kabuli_NC_021165.1_18953209	Ca_kabuli_chr06	18953209	[A/T]	1067299976
Ca_Kabuli_NC_021165.1_18987257	Ca_kabuli_chr06	18987257	[A/G]	1067299977
Ca_Kabuli_NC_021165.1_19000012	Ca_kabuli_chr06	19000012	[A/G]	1067299978
Ca_Kabuli_NC_021165.1_19114054	Ca_kabuli_chr06	19114054	[A/C]	1067299979
Ca_Kabuli_NC_021165.1_19143537	Ca_kabuli_chr06	19143537	[C/A]	1067299980
Ca_Kabuli_NC_021165.1_19178265	Ca_kabuli_chr06	19178265	[A/C]	1067299981
Ca_Kabuli_NC_021165.1_19247966	Ca_kabuli_chr06	19247966	[T/G]	1067299982
Ca_Kabuli_NC_021165.1_19307105	Ca_kabuli_chr06	19307105	[A/T]	1067299983
Ca_Kabuli_NC_021165.1_19394497	Ca_kabuli_chr06	19394497	[T/C]	1067299984
Ca_Kabuli_NC_021165.1_19394518	Ca_kabuli_chr06	19394518	[A/G]	1067299985
Ca_Kabuli_NC_021165.1_19394545	Ca_kabuli_chr06	19394545	[T/C]	1067299986

SNP IDs	Chromosomes	Physical positions (bp)	SNPs	NCBI SNPdb accession IDs (NCBI_ss#)
Ca_Kabuli_NC_021165.1_19433512	Ca_kabuli_chr06	19433512	[C/A]	1067299987
Ca_Kabuli_NC_021165.1_19433513	Ca_kabuli_chr06	19433513	[A/G]	1067299988
Ca_Kabuli_NC_021165.1_19433514	Ca_kabuli_chr06	19433514	[T/A]	1067299989
Ca_Kabuli_NC_021165.1_19433516	Ca_kabuli_chr06	19433516	[A/C]	1067299990
Ca_Kabuli_NC_021165.1_19433517	Ca_kabuli_chr06	19433517	[T/G]	1067299991
Ca_Kabuli_NC_021165.1_19433556	Ca_kabuli_chr06	19433556	[T/A]	1067299992
Ca_Kabuli_NC_021165.1_19437013	Ca_kabuli_chr06	19437013	[A/C]	1067299993
Ca_Kabuli_NC_021165.1_19474577	Ca_kabuli_chr06	19474577	[A/G]	1067299994
Ca_Kabuli_NC_021165.1_19474599	Ca_kabuli_chr06	19474599	[A/C]	1067299995
Ca_Kabuli_NC_021165.1_19474621	Ca_kabuli_chr06	19474621	[T/A]	1067299996
Ca_Kabuli_NC_021165.1_19474629	Ca_kabuli_chr06	19474629	[T/C]	1067299997
Ca_Kabuli_NC_021165.1_19478512	Ca_kabuli_chr06	19478512	[T/G]	1067299998
Ca_Kabuli_NC_021165.1_19478543	Ca_kabuli_chr06	19478543	[G/A]	1067299999
Ca_Kabuli_NC_021165.1_19478545	Ca_kabuli_chr06	19478545	[A/G]	1067300000
Ca_Kabuli_NC_021165.1_19478546	Ca_kabuli_chr06	19478546	[G/C]	1067300001
Ca_Kabuli_NC_021165.1_19502691	Ca_kabuli_chr06	19502691	[A/G]	1067300002
Ca_Kabuli_NC_021165.1_19502700	Ca_kabuli_chr06	19502700	[T/C]	1067300003
Ca_Kabuli_NC_021165.1_19653654	Ca_kabuli_chr06	19653654	[C/T]	1067300004
Ca_Kabuli_NC_021165.1_19674561	Ca_kabuli_chr06	19674561	[A/C]	1067300005
Ca_Kabuli_NC_021165.1_19709914	Ca_kabuli_chr06	19709914	[A/T]	1067300006
Ca_Kabuli_NC_021165.1_19713192	Ca_kabuli_chr06	19713192	[A/G]	1067300007
Ca_Kabuli_NC_021165.1_19713228	Ca_kabuli_chr06	19713228	[T/A]	1067300008
Ca_Kabuli_NC_021165.1_19726254	Ca_kabuli_chr06	19726254	[T/G]	1067300009
Ca_Kabuli_NC_021165.1_19735965	Ca_kabuli_chr06	19735965	[T/G]	1067300010
Ca_Kabuli_NC_021165.1_19735992	Ca_kabuli_chr06	19735992	[A/C]	1067300011
Ca_Kabuli_NC_021165.1_20505038	Ca_kabuli_chr06	20505038	[C/T]	1067300012
Ca_Kabuli_NC_021165.1_20660795	Ca_kabuli_chr06	20660795	[T/C]	1067300013
Ca_Kabuli_NC_021165.1_20660832	Ca_kabuli_chr06	20660832	[A/C]	1067300014
Ca_Kabuli_NC_021165.1_20667254	Ca_kabuli_chr06	20667254	[A/C]	1067300015
Ca_Kabuli_NC_021165.1_20671055	Ca_kabuli_chr06	20671055	[A/T]	1067300016
Ca_Kabuli_NC_021165.1_20679788	Ca_kabuli_chr06	20679788	[T/G]	1067300017
Ca_Kabuli_NC_021165.1_20679803	Ca_kabuli_chr06	20679803	[C/T]	1067300018
Ca_Kabuli_NC_021165.1_20679807	Ca_kabuli_chr06	20679807	[A/C]	1067300019
Ca_Kabuli_NC_021165.1_20679819	Ca_kabuli_chr06	20679819	[T/C]	1067300020

SNP IDs	Chromosomes	Physical positions (bp)	SNPs	NCBI SNPdb accession IDs (NCBI_ss#)
Ca_Kabuli_NC_021165.1_20679821	Ca_kabuli_chr06	20679821	[T/C]	1067300021
Ca_Kabuli_NC_021165.1_20679823	Ca_kabuli_chr06	20679823	[C/T]	1067300022
Ca_Kabuli_NC_021165.1_20679826	Ca_kabuli_chr06	20679826	[C/T]	1067300023
Ca_Kabuli_NC_021165.1_20693828	Ca_kabuli_chr06	20693828	[C/G]	1067300024
Ca_Kabuli_NC_021165.1_20738712	Ca_kabuli_chr06	20738712	[A/G]	1067300025
Ca_Kabuli_NC_021165.1_20738721	Ca_kabuli_chr06	20738721	[T/G]	1067300026
Ca_Kabuli_NC_021165.1_20738730	Ca_kabuli_chr06	20738730	[A/G]	1067300027
Ca_Kabuli_NC_021165.1_20766355	Ca_kabuli_chr06	20766355	[A/C]	1067300028
Ca_Kabuli_NC_021165.1_20766375	Ca_kabuli_chr06	20766375	[T/A]	1067300029
Ca_Kabuli_NC_021165.1_20766462	Ca_kabuli_chr06	20766462	[A/G]	1067300030
Ca_Kabuli_NC_021165.1_20780999	Ca_kabuli_chr06	20780999	[G/C]	1067300031
Ca_Kabuli_NC_021165.1_20781000	Ca_kabuli_chr06	20781000	[G/T]	1067300032
Ca_Kabuli_NC_021165.1_20786070	Ca_kabuli_chr06	20786070	[G/C]	1067300033
Ca_Kabuli_NC_021165.1_20786071	Ca_kabuli_chr06	20786071	[G/T]	1067300034
Ca_Kabuli_NC_021165.1_20802748	Ca_kabuli_chr06	20802748	[T/A]	1067300035
Ca_Kabuli_NC_021165.1_20802761	Ca_kabuli_chr06	20802761	[A/T]	1067300036
Ca_Kabuli_NC_021165.1_20889681	Ca_kabuli_chr06	20889681	[T/A]	1067300037
Ca_Kabuli_NC_021165.1_20890710	Ca_kabuli_chr06	20890710	[A/C]	1067300038
Ca_Kabuli_NC_021165.1_20917619	Ca_kabuli_chr06	20917619	[T/C]	1067300039
Ca_Kabuli_NC_021165.1_20917621	Ca_kabuli_chr06	20917621	[A/T]	1067300040
Ca_Kabuli_NC_021165.1_20917684	Ca_kabuli_chr06	20917684	[A/C]	1067300041
Ca_Kabuli_NC_021165.1_20917713	Ca_kabuli_chr06	20917713	[A/C]	1067300042
Ca_Kabuli_NC_021165.1_20935369	Ca_kabuli_chr06	20935369	[A/C]	1067300043
Ca_Kabuli_NC_021165.1_20935397	Ca_kabuli_chr06	20935397	[T/G]	1067300044
Ca_Kabuli_NC_021165.1_20935894	Ca_kabuli_chr06	20935894	[T/C]	1067300045
Ca_Kabuli_NC_021165.1_20966992	Ca_kabuli_chr06	20966992	[T/G]	1067300046
Ca_Kabuli_NC_021165.1_20998904	Ca_kabuli_chr06	20998904	[C/G]	1067300047
Ca_Kabuli_NC_021165.1_20998905	Ca_kabuli_chr06	20998905	[T/G]	1067300048
Ca_Kabuli_NC_021165.1_21045734	Ca_kabuli_chr06	21045734	[T/C]	1067300049
Ca_Kabuli_NC_021165.1_21045737	Ca_kabuli_chr06	21045737	[A/C]	1067300050
Ca_Kabuli_NC_021165.1_21058265	Ca_kabuli_chr06	21058265	[T/A]	1067300051
Ca_Kabuli_NC_021165.1_21061365	Ca_kabuli_chr06	21061365	[T/G]	1067300052
Ca_Kabuli_NC_021165.1_21170334	Ca_kabuli_chr06	21170334	[T/A]	1067300053
Ca_Kabuli_NC_021165.1_21176033	Ca_kabuli_chr06	21176033	[T/G]	1067300054

SNP IDs	Chromosomes	Physical positions (bp)	SNPs	NCBI SNPdb accession IDs (NCBI_ss#)
Ca_Kabuli_NC_021165.1_21237686	Ca_kabuli_chr06	21237686	[A/C]	1067300055
Ca_Kabuli_NC_021165.1_21238051	Ca_kabuli_chr06	21238051	[T/C]	1067300056
Ca_Kabuli_NC_021165.1_21238081	Ca_kabuli_chr06	21238081	[A/G]	1067300057
Ca_Kabuli_NC_021165.1_21238138	Ca_kabuli_chr06	21238138	[A/C]	1067300058
Ca_Kabuli_NC_021165.1_21238178	Ca_kabuli_chr06	21238178	[A/G]	1067300059
Ca_Kabuli_NC_021165.1_21238721	Ca_kabuli_chr06	21238721	[T/A]	1067300060
Ca_Kabuli_NC_021165.1_21238728	Ca_kabuli_chr06	21238728	[T/C]	1067300061
Ca_Kabuli_NC_021165.1_21238731	Ca_kabuli_chr06	21238731	[T/G]	1067300062
Ca_Kabuli_NC_021165.1_21238743	Ca_kabuli_chr06	21238743	[T/G]	1067300063
Ca_Kabuli_NC_021165.1_21238768	Ca_kabuli_chr06	21238768	[T/G]	1067300064
Ca_Kabuli_NC_021165.1_21322836	Ca_kabuli_chr06	21322836	[C/T]	1067300065
Ca_Kabuli_NC_021165.1_21322854	Ca_kabuli_chr06	21322854	[G/A]	1067300066
Ca_Kabuli_NC_021165.1_21322875	Ca_kabuli_chr06	21322875	[T/C]	1067300067
Ca_Kabuli_NC_021165.1_21322884	Ca_kabuli_chr06	21322884	[A/G]	1067300068
Ca_Kabuli_NC_021165.1_21324520	Ca_kabuli_chr06	21324520	[T/C]	1067300069
Ca_Kabuli_NC_021165.1_21324597	Ca_kabuli_chr06	21324597	[T/G]	1067300070
Ca_Kabuli_NC_021165.1_21324640	Ca_kabuli_chr06	21324640	[A/C]	1067300071
Ca_Kabuli_NC_021165.1_21326252	Ca_kabuli_chr06	21326252	[A/C]	1067300072
Ca_Kabuli_NC_021165.1_21326272	Ca_kabuli_chr06	21326272	[T/C]	1067300073
Ca_Kabuli_NC_021165.1_21326282	Ca_kabuli_chr06	21326282	[A/T]	1067300074
Ca_Kabuli_NC_021165.1_21477001	Ca_kabuli_chr06	21477001	[A/G]	1067300075
Ca_Kabuli_NC_021165.1_21478564	Ca_kabuli_chr06	21478564	[T/C]	1067300076
Ca_Kabuli_NC_021165.1_21478607	Ca_kabuli_chr06	21478607	[T/G]	1067300077
Ca_Kabuli_NC_021165.1_21479690	Ca_kabuli_chr06	21479690	[T/G]	1067300078
Ca_Kabuli_NC_021165.1_21489039	Ca_kabuli_chr06	21489039	[T/G]	1067300079
Ca_Kabuli_NC_021165.1_21489040	Ca_kabuli_chr06	21489040	[G/C]	1067300080
Ca_Kabuli_NC_021165.1_21489042	Ca_kabuli_chr06	21489042	[T/C]	1067300081
Ca_Kabuli_NC_021165.1_21489049	Ca_kabuli_chr06	21489049	[A/C]	1067300082
Ca_Kabuli_NC_021165.1_21489060	Ca_kabuli_chr06	21489060	[T/C]	1067300083
Ca_Kabuli_NC_021165.1_21497487	Ca_kabuli_chr06	21497487	[A/G]	1067300084
Ca_Kabuli_NC_021165.1_21506176	Ca_kabuli_chr06	21506176	[A/G]	1067300085
Ca_Kabuli_NC_021165.1_21506184	Ca_kabuli_chr06	21506184	[T/C]	1067300086
Ca_Kabuli_NC_021165.1_21506246	Ca_kabuli_chr06	21506246	[T/C]	1067300087
Ca_Kabuli_NC_021165.1_21506288	Ca_kabuli_chr06	21506288	[T/G]	1067300088

SNP IDs	Chromosomes	Physical positions (bp)	SNPs	NCBI SNPdb accession IDs (NCBI_ss#)
Ca_Kabuli_NC_021165.1_21506372	Ca_kabuli_chr06	21506372	[T/C]	1067300089
Ca_Kabuli_NC_021165.1_21506392	Ca_kabuli_chr06	21506392	[T/G]	1067300090
Ca_Kabuli_NC_021165.1_21548490	Ca_kabuli_chr06	21548490	[T/A]	1067300091
Ca_Kabuli_NC_021165.1_21576795	Ca_kabuli_chr06	21576795	[A/G]	1067300092
Ca_Kabuli_NC_021165.1_21601413	Ca_kabuli_chr06	21601413	[A/T]	1067300093
Ca_Kabuli_NC_021165.1_21601419	Ca_kabuli_chr06	21601419	[A/C]	1067300094
Ca_Kabuli_NC_021165.1_21601435	Ca_kabuli_chr06	21601435	[A/C]	1067300095
Ca_Kabuli_NC_021165.1_21603167	Ca_kabuli_chr06	21603167	[A/C]	1067300096
Ca_Kabuli_NC_021165.1_21675741	Ca_kabuli_chr06	21675741	[T/G]	1067300097
Ca_Kabuli_NC_021165.1_21675839	Ca_kabuli_chr06	21675839	[A/T]	1067300098
Ca_Kabuli_NC_021165.1_21683880	Ca_kabuli_chr06	21683880	[A/C]	1067300099
Ca_Kabuli_NC_021165.1_21718696	Ca_kabuli_chr06	21718696	[A/C]	1067300100
Ca_Kabuli_NC_021165.1_21718891	Ca_kabuli_chr06	21718891	[A/C]	1067300101
Ca_Kabuli_NC_021165.1_21809707	Ca_kabuli_chr06	21809707	[T/C]	1067300102
Ca_Kabuli_NC_021165.1_21809727	Ca_kabuli_chr06	21809727	[T/A]	1067300103
Ca_Kabuli_NC_021165.1_21809746	Ca_kabuli_chr06	21809746	[A/T]	1067300104
Ca_Kabuli_NC_021165.1_21865952	Ca_kabuli_chr06	21865952	[A/G]	1067300105
Ca_Kabuli_NC_021165.1_21914401	Ca_kabuli_chr06	21914401	[T/C]	1067300106
Ca_Kabuli_NC_021165.1_21914411	Ca_kabuli_chr06	21914411	[T/C]	1067300107
Ca_Kabuli_NC_021165.1_21914418	Ca_kabuli_chr06	21914418	[A/G]	1067300108
Ca_Kabuli_NC_021165.1_21917906	Ca_kabuli_chr06	21917906	[T/A]	1067300109
Ca_Kabuli_NC_021165.1_21917910	Ca_kabuli_chr06	21917910	[T/G]	1067300110
Ca_Kabuli_NC_021165.1_22016054	Ca_kabuli_chr06	22016054	[T/C]	1067300111
Ca_Kabuli_NC_021165.1_22017276	Ca_kabuli_chr06	22017276	[A/C]	1067300112
Ca_Kabuli_NC_021165.1_22017280	Ca_kabuli_chr06	22017280	[A/C]	1067300113
Ca_Kabuli_NC_021165.1_22017312	Ca_kabuli_chr06	22017312	[A/C]	1067300114
Ca_Kabuli_NC_021165.1_22019758	Ca_kabuli_chr06	22019758	[A/C]	1067300115
Ca_Kabuli_NC_021165.1_22019762	Ca_kabuli_chr06	22019762	[T/C]	1067300116
Ca_Kabuli_NC_021165.1_22019764	Ca_kabuli_chr06	22019764	[G/T]	1067300117
Ca_Kabuli_NC_021165.1_22071718	Ca_kabuli_chr06	22071718	[T/C]	1067300118
Ca_Kabuli_NC_021165.1_22071762	Ca_kabuli_chr06	22071762	[A/C]	1067300119
Ca_Kabuli_NC_021165.1_22074776	Ca_kabuli_chr06	22074776	[T/C]	1067300120
Ca_Kabuli_NC_021165.1_22074780	Ca_kabuli_chr06	22074780	[A/G]	1067300121
Ca_Kabuli_NC_021165.1_22074812	Ca_kabuli_chr06	22074812	[T/G]	1067300122

SNP IDs	Chromosomes	Physical positions (bp)	SNPs	NCBI SNPdb accession IDs (NCBI_ss#)
Ca_Kabuli_NC_021165.1_22074818	Ca_kabuli_chr06	22074818	[A/C]	1067300123
Ca_Kabuli_NC_021165.1_22074828	Ca_kabuli_chr06	22074828	[A/G]	1067300124
Ca_Kabuli_NC_021165.1_22074836	Ca_kabuli_chr06	22074836	[T/A]	1067300125
Ca_Kabuli_NC_021165.1_22079140	Ca_kabuli_chr06	22079140	[A/G]	1067300126
Ca_Kabuli_NC_021165.1_22120904	Ca_kabuli_chr06	22120904	[A/T]	1067300127
Ca_Kabuli_NC_021165.1_22137481	Ca_kabuli_chr06	22137481	[A/T]	1067300128
Ca_Kabuli_NC_021165.1_22192498	Ca_kabuli_chr06	22192498	[A/C]	1067300129
Ca_Kabuli_NC_021165.1_22194363	Ca_kabuli_chr06	22194363	[T/G]	1067300130
Ca_Kabuli_NC_021165.1_22201184	Ca_kabuli_chr06	22201184	[A/T]	1067300131
Ca_Kabuli_NC_021165.1_22201672	Ca_kabuli_chr06	22201672	[A/T]	1067300132
Ca_Kabuli_NC_021165.1_22201696	Ca_kabuli_chr06	22201696	[A/C]	1067300133
Ca_Kabuli_NC_021165.1_22201699	Ca_kabuli_chr06	22201699	[G/T]	1067300134
Ca_Kabuli_NC_021165.1_22208093	Ca_kabuli_chr06	22208093	[G/A]	1067300135
Ca_Kabuli_NC_021165.1_22208126	Ca_kabuli_chr06	22208126	[T/G]	1067300136
Ca_Kabuli_NC_021165.1_22208136	Ca_kabuli_chr06	22208136	[A/G]	1067300137
Ca_Kabuli_NC_021165.1_22208617	Ca_kabuli_chr06	22208617	[A/T]	1067300138
Ca_Kabuli_NC_021165.1_22208644	Ca_kabuli_chr06	22208644	[G/T]	1067300139
Ca_Kabuli_NC_021165.1_22208645	Ca_kabuli_chr06	22208645	[T/C]	1067300140
Ca_Kabuli_NC_021165.1_22213998	Ca_kabuli_chr06	22213998	[A/G]	1067300141
Ca_Kabuli_NC_021165.1_22214020	Ca_kabuli_chr06	22214020	[T/A]	1067300142
Ca_Kabuli_NC_021165.1_22230125	Ca_kabuli_chr06	22230125	[T/G]	1067300143
Ca_Kabuli_NC_021165.1_22267268	Ca_kabuli_chr06	22267268	[A/G]	1067300144
Ca_Kabuli_NC_021165.1_22267284	Ca_kabuli_chr06	22267284	[T/C]	1067300145
Ca_Kabuli_NC_021165.1_22278433	Ca_kabuli_chr06	22278433	[T/G]	1067300146
Ca_Kabuli_NC_021165.1_22278486	Ca_kabuli_chr06	22278486	[A/C]	1067300147
Ca_Kabuli_NC_021165.1_22278509	Ca_kabuli_chr06	22278509	[A/T]	1067300148
Ca_Kabuli_NC_021165.1_22295809	Ca_kabuli_chr06	22295809	[T/C]	1067300149
Ca_Kabuli_NC_021165.1_22334099	Ca_kabuli_chr06	22334099	[A/C]	1067300150
Ca_Kabuli_NC_021165.1_22334620	Ca_kabuli_chr06	22334620	[A/G]	1067300151
Ca_Kabuli_NC_021165.1_22334625	Ca_kabuli_chr06	22334625	[A/G]	1067300152
Ca_Kabuli_NC_021165.1_22334895	Ca_kabuli_chr06	22334895	[A/C]	1067300153
Ca_Kabuli_NC_021165.1_22334937	Ca_kabuli_chr06	22334937	[A/G]	1067300154
Ca_Kabuli_NC_021165.1_22354677	Ca_kabuli_chr06	22354677	[T/C]	1067300155
Ca_Kabuli_NC_021165.1_22372330	Ca_kabuli_chr06	22372330	[A/G]	1067300156

SNP IDs	Chromosomes	Physical positions (bp)	SNPs	NCBI SNPdb accession IDs (NCBI_ss#)
Ca_Kabuli_NC_021165.1_22951295	Ca_kabuli_chr06	22951295	[A/G]	1067300157
Ca_Kabuli_NC_021165.1_22951298	Ca_kabuli_chr06	22951298	[A/T]	1067300158
Ca_Kabuli_NC_021165.1_22951337	Ca_kabuli_chr06	22951337	[T/G]	1067300159
Ca_Kabuli_NC_021165.1_22951342	Ca_kabuli_chr06	22951342	[G/A]	1067300160
Ca_Kabuli_NC_021165.1_22965837	Ca_kabuli_chr06	22965837	[T/G]	1067300161
Ca_Kabuli_NC_021165.1_22965838	Ca_kabuli_chr06	22965838	[A/T]	1067300162
Ca_Kabuli_NC_021165.1_23010554	Ca_kabuli_chr06	23010554	[A/C]	1067300163
Ca_Kabuli_NC_021165.1_23010560	Ca_kabuli_chr06	23010560	[T/C]	1067300164
Ca_Kabuli_NC_021165.1_23014707	Ca_kabuli_chr06	23014707	[A/G]	1067300165
Ca_Kabuli_NC_021165.1_23014711	Ca_kabuli_chr06	23014711	[A/T]	1067300166
Ca_Kabuli_NC_021165.1_23014716	Ca_kabuli_chr06	23014716	[A/T]	1067300167
Ca_Kabuli_NC_021165.1_23014721	Ca_kabuli_chr06	23014721	[T/C]	1067300168
Ca_Kabuli_NC_021165.1_23023346	Ca_kabuli_chr06	23023346	[C/A]	1067300169
Ca_Kabuli_NC_021165.1_23039459	Ca_kabuli_chr06	23039459	[C/T]	1067300170
Ca_Kabuli_NC_021165.1_23128543	Ca_kabuli_chr06	23128543	[T/G]	1067300171
Ca_Kabuli_NC_021165.1_23139867	Ca_kabuli_chr06	23139867	[A/G]	1067300172
Ca_Kabuli_NC_021165.1_23139881	Ca_kabuli_chr06	23139881	[T/C]	1067300173
Ca_Kabuli_NC_021165.1_23145612	Ca_kabuli_chr06	23145612	[A/T]	1067300174
Ca_Kabuli_NC_021165.1_23145617	Ca_kabuli_chr06	23145617	[A/T]	1067300175
Ca_Kabuli_NC_021165.1_23145621	Ca_kabuli_chr06	23145621	[T/G]	1067300176
Ca_Kabuli_NC_021165.1_23145625	Ca_kabuli_chr06	23145625	[A/C]	1067300177
Ca_Kabuli_NC_021165.1_23166428	Ca_kabuli_chr06	23166428	[A/T]	1067300178
Ca_Kabuli_NC_021165.1_23199360	Ca_kabuli_chr06	23199360	[T/C]	1067300179
Ca_Kabuli_NC_021165.1_23254618	Ca_kabuli_chr06	23254618	[T/A]	1067300180
Ca_Kabuli_NC_021165.1_23284951	Ca_kabuli_chr06	23284951	[A/G]	1067300181
Ca_Kabuli_NC_021165.1_23284956	Ca_kabuli_chr06	23284956	[A/G]	1067300182
Ca_Kabuli_NC_021165.1_23284957	Ca_kabuli_chr06	23284957	[A/G]	1067300183
Ca_Kabuli_NC_021165.1_23284978	Ca_kabuli_chr06	23284978	[T/C]	1067300184
Ca_Kabuli_NC_021165.1_23298861	Ca_kabuli_chr06	23298861	[A/T]	1067300185
Ca_Kabuli_NC_021165.1_23298877	Ca_kabuli_chr06	23298877	[T/C]	1067300186
Ca_Kabuli_NC_021165.1_23380701	Ca_kabuli_chr06	23380701	[T/G]	1067300187
Ca_Kabuli_NC_021165.1_23380886	Ca_kabuli_chr06	23380886	[A/G]	1067300188
Ca_Kabuli_NC_021165.1_23405638	Ca_kabuli_chr06	23405638	[A/G]	1067300189
Ca_Kabuli_NC_021165.1_23423091	Ca_kabuli_chr06	23423091	[T/C]	1067300190

SNP IDs	Chromosomes	Physical positions (bp)	SNPs	NCBI SNPdb accession IDs (NCBI_ss#)
Ca_Kabuli_NC_021165.1_23423096	Ca_kabuli_chr06	23423096	[T/G]	1067300191
Ca_Kabuli_NC_021165.1_23426437	Ca_kabuli_chr06	23426437	[T/A]	1067300192
Ca_Kabuli_NC_021165.1_23426440	Ca_kabuli_chr06	23426440	[T/C]	1067300193
Ca_Kabuli_NC_021165.1_23435814	Ca_kabuli_chr06	23435814	[T/C]	1067300194
Ca_Kabuli_NC_021165.1_23461650	Ca_kabuli_chr06	23461650	[C/T]	1067300195
Ca_Kabuli_NC_021165.1_23478806	Ca_kabuli_chr06	23478806	[A/C]	1067300196
Ca_Kabuli_NC_021165.1_23478807	Ca_kabuli_chr06	23478807	[G/C]	1067300197
Ca_Kabuli_NC_021165.1_23478808	Ca_kabuli_chr06	23478808	[A/T]	1067300198
Ca_Kabuli_NC_021165.1_23478809	Ca_kabuli_chr06	23478809	[T/G]	1067300199
Ca_Kabuli_NC_021165.1_23483861	Ca_kabuli_chr06	23483861	[A/G]	1067300200
Ca_Kabuli_NC_021165.1_23483863	Ca_kabuli_chr06	23483863	[A/G]	1067300201
Ca_Kabuli_NC_021165.1_23483878	Ca_kabuli_chr06	23483878	[A/C]	1067300202
Ca_Kabuli_NC_021165.1_23483880	Ca_kabuli_chr06	23483880	[T/C]	1067300203
Ca_Kabuli_NC_021165.1_23507024	Ca_kabuli_chr06	23507024	[A/C]	1067300204
Ca_Kabuli_NC_021165.1_23549267	Ca_kabuli_chr06	23549267	[G/T]	1067300205
Ca_Kabuli_NC_021165.1_23611565	Ca_kabuli_chr06	23611565	[T/C]	1067300206
Ca_Kabuli_NC_021165.1_23611584	Ca_kabuli_chr06	23611584	[A/G]	1067300207
Ca_Kabuli_NC_021165.1_23629137	Ca_kabuli_chr06	23629137	[T/G]	1067300208
Ca_Kabuli_NC_021165.1_23629152	Ca_kabuli_chr06	23629152	[T/G]	1067300209
Ca_Kabuli_NC_021165.1_23629160	Ca_kabuli_chr06	23629160	[A/C]	1067300210
Ca_Kabuli_NC_021165.1_23638374	Ca_kabuli_chr06	23638374	[T/C]	1067300211
Ca_Kabuli_NC_021165.1_23638386	Ca_kabuli_chr06	23638386	[A/G]	1067300212
Ca_Kabuli_NC_021165.1_23638403	Ca_kabuli_chr06	23638403	[G/A]	1067300213
Ca_Kabuli_NC_021165.1_23638405	Ca_kabuli_chr06	23638405	[G/T]	1067300214
Ca_Kabuli_NC_021165.1_23638414	Ca_kabuli_chr06	23638414	[T/G]	1067300215
Ca_Kabuli_NC_021165.1_23638429	Ca_kabuli_chr06	23638429	[T/G]	1067300216
Ca_Kabuli_NC_021165.1_23638858	Ca_kabuli_chr06	23638858	[A/C]	1067300217
Ca_Kabuli_NC_021165.1_23638861	Ca_kabuli_chr06	23638861	[A/G]	1067300218
Ca_Kabuli_NC_021165.1_23638891	Ca_kabuli_chr06	23638891	[A/G]	1067300219
Ca_Kabuli_NC_021165.1_23694398	Ca_kabuli_chr06	23694398	[T/G]	1067300220
Ca_Kabuli_NC_021165.1_23696788	Ca_kabuli_chr06	23696788	[C/A]	1067300221
Ca_Kabuli_NC_021165.1_23696789	Ca_kabuli_chr06	23696789	[T/G]	1067300222
Ca_Kabuli_NC_021165.1_23725184	Ca_kabuli_chr06	23725184	[T/C]	1067300223
Ca_Kabuli_NC_021165.1_23725223	Ca_kabuli_chr06	23725223	[A/G]	1067300224

SNP IDs	Chromosomes	Physical positions (bp)	SNPs	NCBI SNPdb accession IDs (NCBI_ss#)
Ca_Kabuli_NC_021165.1_23767671	Ca_kabuli_chr06	23767671	[T/A]	1067300225
Ca_Kabuli_NC_021165.1_23776635	Ca_kabuli_chr06	23776635	[A/G]	1067300226
Ca_Kabuli_NC_021165.1_23776650	Ca_kabuli_chr06	23776650	[G/C]	1067300227
Ca_Kabuli_NC_021165.1_23776668	Ca_kabuli_chr06	23776668	[C/T]	1067300228
Ca_Kabuli_NC_021165.1_23803958	Ca_kabuli_chr06	23803958	[C/A]	1067300229
Ca_Kabuli_NC_021165.1_23803959	Ca_kabuli_chr06	23803959	[T/G]	1067300230
Ca_Kabuli_NC_021165.1_23803961	Ca_kabuli_chr06	23803961	[A/T]	1067300231
Ca_Kabuli_NC_021165.1_23809138	Ca_kabuli_chr06	23809138	[T/C]	1067300232
Ca_Kabuli_NC_021165.1_23809163	Ca_kabuli_chr06	23809163	[A/T]	1067300233
Ca_Kabuli_NC_021165.1_23811391	Ca_kabuli_chr06	23811391	[T/C]	1067300234
Ca_Kabuli_NC_021165.1_23845403	Ca_kabuli_chr06	23845403	[A/T]	1067300235
Ca_Kabuli_NC_021165.1_23853493	Ca_kabuli_chr06	23853493	[A/C]	1067300236
Ca_Kabuli_NC_021165.1_23884409	Ca_kabuli_chr06	23884409	[A/C]	1067300237
Ca_Kabuli_NC_021165.1_23884429	Ca_kabuli_chr06	23884429	[A/T]	1067300238
Ca_Kabuli_NC_021165.1_23884437	Ca_kabuli_chr06	23884437	[A/T]	1067300239
Ca_Kabuli_NC_021165.1_23890859	Ca_kabuli_chr06	23890859	[T/C]	1067300240
Ca_Kabuli_NC_021165.1_23890863	Ca_kabuli_chr06	23890863	[A/C]	1067300241
Ca_Kabuli_NC_021165.1_23891044	Ca_kabuli_chr06	23891044	[G/T]	1067300242
Ca_Kabuli_NC_021165.1_23953716	Ca_kabuli_chr06	23953716	[A/C]	1067300243
Ca_Kabuli_NC_021165.1_23953735	Ca_kabuli_chr06	23953735	[A/C]	1067300244
Ca_Kabuli_NC_021165.1_23978583	Ca_kabuli_chr06	23978583	[A/T]	1067300245
Ca_Kabuli_NC_021165.1_23989568	Ca_kabuli_chr06	23989568	[A/G]	1067300246
Ca_Kabuli_NC_021165.1_24085806	Ca_kabuli_chr06	24085806	[A/G]	1067300247
Ca_Kabuli_NC_021165.1_24174935	Ca_kabuli_chr06	24174935	[A/C]	1067300248
Ca_Kabuli_NC_021165.1_24174943	Ca_kabuli_chr06	24174943	[T/C]	1067300249
Ca_Kabuli_NC_021165.1_24176032	Ca_kabuli_chr06	24176032	[A/T]	1067300250
Ca_Kabuli_NC_021165.1_24235632	Ca_kabuli_chr06	24235632	[C/T]	1067300251
Ca_Kabuli_NC_021165.1_24235675	Ca_kabuli_chr06	24235675	[C/T]	1067300252
Ca_Kabuli_NC_021165.1_24255403	Ca_kabuli_chr06	24255403	[A/C]	1067300253
Ca_Kabuli_NC_021165.1_24260542	Ca_kabuli_chr06	24260542	[A/C]	1067300254
Ca_Kabuli_NC_021165.1_24302301	Ca_kabuli_chr06	24302301	[T/C]	1067300255
Ca_Kabuli_NC_021165.1_24302326	Ca_kabuli_chr06	24302326	[A/T]	1067300256
Ca_Kabuli_NC_021165.1_24315209	Ca_kabuli_chr06	24315209	[T/A]	1067300257
Ca_Kabuli_NC_021165.1_24315210	Ca_kabuli_chr06	24315210	[A/T]	1067300258

SNP IDs	Chromosomes	Physical positions (bp)	SNPs	NCBI SNPdb accession IDs (NCBI_ss#)
Ca_Kabuli_NC_021165.1_24413576	Ca_kabuli_chr06	24413576	[A/C]	1067300259
Ca_Kabuli_NC_021165.1_24413584	Ca_kabuli_chr06	24413584	[T/A]	1067300260
Ca_Kabuli_NC_021165.1_24413658	Ca_kabuli_chr06	24413658	[T/G]	1067300261
Ca_Kabuli_NC_021165.1_24413663	Ca_kabuli_chr06	24413663	[T/G]	1067300262
Ca_Kabuli_NC_021165.1_24413715	Ca_kabuli_chr06	24413715	[T/G]	1067300263
Ca_Kabuli_NC_021165.1_24414475	Ca_kabuli_chr06	24414475	[T/C]	1067300264
Ca_Kabuli_NC_021165.1_24414518	Ca_kabuli_chr06	24414518	[A/G]	1067300265
Ca_Kabuli_NC_021165.1_24479389	Ca_kabuli_chr06	24479389	[G/T]	1067300266
Ca_Kabuli_NC_021165.1_24480359	Ca_kabuli_chr06	24480359	[C/A]	1067300267
Ca_Kabuli_NC_021165.1_24482001	Ca_kabuli_chr06	24482001	[A/G]	1067300268
Ca_Kabuli_NC_021165.1_24482026	Ca_kabuli_chr06	24482026	[T/C]	1067300269
Ca_Kabuli_NC_021165.1_24482112	Ca_kabuli_chr06	24482112	[T/G]	1067300270
Ca_Kabuli_NC_021165.1_24482886	Ca_kabuli_chr06	24482886	[T/C]	1067300271
Ca_Kabuli_NC_021165.1_24482903	Ca_kabuli_chr06	24482903	[A/G]	1067300272
Ca_Kabuli_NC_021165.1_24482930	Ca_kabuli_chr06	24482930	[A/G]	1067300273
Ca_Kabuli_NC_021165.1_24498895	Ca_kabuli_chr06	24498895	[C/A]	1067300274
Ca_Kabuli_NC_021165.1_24498913	Ca_kabuli_chr06	24498913	[T/C]	1067300275
Ca_Kabuli_NC_021165.1_24498915	Ca_kabuli_chr06	24498915	[T/C]	1067300276
Ca_Kabuli_NC_021165.1_24498923	Ca_kabuli_chr06	24498923	[G/A]	1067300277
Ca_Kabuli_NC_021165.1_24516532	Ca_kabuli_chr06	24516532	[T/C]	1067300278
Ca_Kabuli_NC_021165.1_24547068	Ca_kabuli_chr06	24547068	[T/C]	1067300279
Ca_Kabuli_NC_021165.1_24547091	Ca_kabuli_chr06	24547091	[A/G]	1067300280
Ca_Kabuli_NC_021165.1_24586211	Ca_kabuli_chr06	24586211	[T/C]	1067300281
Ca_Kabuli_NC_021165.1_24587441	Ca_kabuli_chr06	24587441	[T/G]	1067300282
Ca_Kabuli_NC_021165.1_24587450	Ca_kabuli_chr06	24587450	[A/C]	1067300283
Ca_Kabuli_NC_021165.1_24591538	Ca_kabuli_chr06	24591538	[A/C]	1067300284
Ca_Kabuli_NC_021165.1_24626243	Ca_kabuli_chr06	24626243	[T/G]	1067300285
Ca_Kabuli_NC_021165.1_24641070	Ca_kabuli_chr06	24641070	[T/A]	1067300286
Ca_Kabuli_NC_021165.1_24644774	Ca_kabuli_chr06	24644774	[A/T]	1067300287
Ca_Kabuli_NC_021165.1_24644801	Ca_kabuli_chr06	24644801	[T/C]	1067300288
Ca_Kabuli_NC_021165.1_24662651	Ca_kabuli_chr06	24662651	[C/A]	1067300289
Ca_Kabuli_NC_021165.1_24662663	Ca_kabuli_chr06	24662663	[T/A]	1067300290
Ca_Kabuli_NC_021165.1_24665173	Ca_kabuli_chr06	24665173	[A/T]	1067300291
Ca_Kabuli_NC_021165.1_25105495	Ca_kabuli_chr06	25105495	[A/G]	1067300292

SNP IDs	Chromosomes	Physical positions (bp)	SNPs	NCBI SNPdb accession IDs (NCBI_ss#)
Ca_Kabuli_NC_021165.1_25351913	Ca_kabuli_chr06	25351913	[G/A]	1067300293
Ca_Kabuli_NC_021165.1_25351931	Ca_kabuli_chr06	25351931	[G/A]	1067300294
Ca_Kabuli_NC_021165.1_25351956	Ca_kabuli_chr06	25351956	[G/A]	1067300295
Ca_Kabuli_NC_021165.1_25574922	Ca_kabuli_chr06	25574922	[C/T]	1067300296
Ca_Kabuli_NC_021165.1_25574923	Ca_kabuli_chr06	25574923	[C/T]	1067300297
Ca_Kabuli_NC_021165.1_25575045	Ca_kabuli_chr06	25575045	[C/A]	1067300298
Ca_Kabuli_NC_021165.1_25626711	Ca_kabuli_chr06	25626711	[C/T]	1067300299
Ca_Kabuli_NC_021165.1_25853851	Ca_kabuli_chr06	25853851	[A/C]	1067300300
Ca_Kabuli_NC_021165.1_26151459	Ca_kabuli_chr06	26151459	[G/T]	1067300301
Ca_Kabuli_NC_021165.1_26151464	Ca_kabuli_chr06	26151464	[T/C]	1067300302
Ca_Kabuli_NC_021165.1_26151466	Ca_kabuli_chr06	26151466	[A/C]	1067300303
Ca_Kabuli_NC_021165.1_26345328	Ca_kabuli_chr06	26345328	[A/G]	1067300304
Ca_Kabuli_NC_021165.1_26345343	Ca_kabuli_chr06	26345343	[C/T]	1067300305
Ca_Kabuli_NC_021165.1_26353512	Ca_kabuli_chr06	26353512	[C/T]	1067300306
Ca_Kabuli_NC_021165.1_26353559	Ca_kabuli_chr06	26353559	[C/A]	1067300307
Ca_Kabuli_NC_021165.1_26353560	Ca_kabuli_chr06	26353560	[A/G]	1067300308
Ca_Kabuli_NC_021165.1_26353740	Ca_kabuli_chr06	26353740	[G/C]	1067300309
Ca_Kabuli_NC_021165.1_26357454	Ca_kabuli_chr06	26357454	[A/G]	1067300310
Ca_Kabuli_NC_021165.1_26360358	Ca_kabuli_chr06	26360358	[T/C]	1067300311
Ca_Kabuli_NC_021165.1_26383500	Ca_kabuli_chr06	26383500	[C/G]	1067300312
Ca_Kabuli_NC_021165.1_26427048	Ca_kabuli_chr06	26427048	[T/C]	1067300313
Ca_Kabuli_NC_021165.1_26551633	Ca_kabuli_chr06	26551633	[T/C]	1067300314
Ca_Kabuli_NC_021165.1_26655933	Ca_kabuli_chr06	26655933	[T/G]	1067300315
Ca_Kabuli_NC_021165.1_26655963	Ca_kabuli_chr06	26655963	[T/G]	1067300316
Ca_Kabuli_NC_021165.1_26701813	Ca_kabuli_chr06	26701813	[T/C]	1067300317
Ca_Kabuli_NC_021165.1_26769041	Ca_kabuli_chr06	26769041	[A/C]	1067300318
Ca_Kabuli_NC_021165.1_26769051	Ca_kabuli_chr06	26769051	[T/C]	1067300319
Ca_Kabuli_NC_021165.1_26769079	Ca_kabuli_chr06	26769079	[A/G]	1067300320
Ca_Kabuli_NC_021165.1_26769085	Ca_kabuli_chr06	26769085	[T/A]	1067300321
Ca_Kabuli_NC_021165.1_27053199	Ca_kabuli_chr06	27053199	[G/A]	1067300322
Ca_Kabuli_NC_021165.1_27053229	Ca_kabuli_chr06	27053229	[A/C]	1067300323
Ca_Kabuli_NC_021165.1_27053289	Ca_kabuli_chr06	27053289	[T/G]	1067300324
Ca_Kabuli_NC_021165.1_27053301	Ca_kabuli_chr06	27053301	[T/G]	1067300325
Ca_Kabuli_NC_021165.1_27053349	Ca_kabuli_chr06	27053349	[A/T]	1067300326

SNP IDs	Chromosomes	Physical positions (bp)	SNPs	NCBI SNPdb accession IDs (NCBI_ss#)
Ca_Kabuli_NC_021165.1_27091881	Ca_kabuli_chr06	27091881	[C/A]	1067300327
Ca_Kabuli_NC_021165.1_27091882	Ca_kabuli_chr06	27091882	[A/G]	1067300328
Ca_Kabuli_NC_021165.1_27091883	Ca_kabuli_chr06	27091883	[G/A]	1067300329
Ca_Kabuli_NC_021165.1_27091920	Ca_kabuli_chr06	27091920	[A/C]	1067300330
Ca_Kabuli_NC_021165.1_27091988	Ca_kabuli_chr06	27091988	[A/C]	1067300331
Ca_Kabuli_NC_021165.1_27122415	Ca_kabuli_chr06	27122415	[T/C]	1067300332
Ca_Kabuli_NC_021165.1_27122451	Ca_kabuli_chr06	27122451	[A/G]	1067300333
Ca_Kabuli_NC_021165.1_27192605	Ca_kabuli_chr06	27192605	[T/G]	1067300334
Ca_Kabuli_NC_021165.1_27223303	Ca_kabuli_chr06	27223303	[T/C]	1067300335
Ca_Kabuli_NC_021165.1_27223327	Ca_kabuli_chr06	27223327	[A/G]	1067300336
Ca_Kabuli_NC_021165.1_27223329	Ca_kabuli_chr06	27223329	[T/C]	1067300337
Ca_Kabuli_NC_021165.1_27223336	Ca_kabuli_chr06	27223336	[T/G]	1067300338
Ca_Kabuli_NC_021165.1_27229201	Ca_kabuli_chr06	27229201	[T/C]	1067300339
Ca_Kabuli_NC_021165.1_27229202	Ca_kabuli_chr06	27229202	[G/T]	1067300340
Ca_Kabuli_NC_021165.1_27310343	Ca_kabuli_chr06	27310343	[T/A]	1067300341
Ca_Kabuli_NC_021165.1_27310344	Ca_kabuli_chr06	27310344	[T/A]	1067300342
Ca_Kabuli_NC_021165.1_27326400	Ca_kabuli_chr06	27326400	[A/C]	1067300343
Ca_Kabuli_NC_021165.1_27330069	Ca_kabuli_chr06	27330069	[T/A]	1067300344
Ca_Kabuli_NC_021165.1_27331151	Ca_kabuli_chr06	27331151	[C/G]	1067300345
Ca_Kabuli_NC_021165.1_27331154	Ca_kabuli_chr06	27331154	[A/G]	1067300346
Ca_Kabuli_NC_021165.1_27331816	Ca_kabuli_chr06	27331816	[T/G]	1067300347
Ca_Kabuli_NC_021165.1_27331818	Ca_kabuli_chr06	27331818	[A/T]	1067300348
Ca_Kabuli_NC_021165.1_27331819	Ca_kabuli_chr06	27331819	[A/C]	1067300349
Ca_Kabuli_NC_021165.1_27331820	Ca_kabuli_chr06	27331820	[G/T]	1067300350
Ca_Kabuli_NC_021165.1_27332632	Ca_kabuli_chr06	27332632	[A/G]	1067300351
Ca_Kabuli_NC_021165.1_27332651	Ca_kabuli_chr06	27332651	[A/C]	1067300352
Ca_Kabuli_NC_021165.1_27332663	Ca_kabuli_chr06	27332663	[A/T]	1067300353
Ca_Kabuli_NC_021165.1_27383733	Ca_kabuli_chr06	27383733	[T/C]	1067300354
Ca_Kabuli_NC_021165.1_27383744	Ca_kabuli_chr06	27383744	[T/G]	1067300355
Ca_Kabuli_NC_021165.1_27383745	Ca_kabuli_chr06	27383745	[T/G]	1067300356
Ca_Kabuli_NC_021165.1_27383746	Ca_kabuli_chr06	27383746	[T/A]	1067300357
Ca_Kabuli_NC_021165.1_27383748	Ca_kabuli_chr06	27383748	[A/G]	1067300358
Ca_Kabuli_NC_021165.1_27385025	Ca_kabuli_chr06	27385025	[A/G]	1067300359
Ca_Kabuli_NC_021165.1_27515293	Ca_kabuli_chr06	27515293	[A/G]	1067300360

SNP IDs	Chromosomes	Physical positions (bp)	SNPs	NCBI SNPdb accession IDs (NCBI_ss#)
Ca_Kabuli_NC_021165.1_27591765	Ca_kabuli_chr06	27591765	[C/A]	1067300361
Ca_Kabuli_NC_021165.1_27607252	Ca_kabuli_chr06	27607252	[T/C]	1067300362
Ca_Kabuli_NC_021165.1_27621978	Ca_kabuli_chr06	27621978	[A/T]	1067300363
Ca_Kabuli_NC_021165.1_27621992	Ca_kabuli_chr06	27621992	[T/C]	1067300364
Ca_Kabuli_NC_021165.1_27622022	Ca_kabuli_chr06	27622022	[T/A]	1067300365
Ca_Kabuli_NC_021165.1_27622044	Ca_kabuli_chr06	27622044	[T/A]	1067300366
Ca_Kabuli_NC_021165.1_27710490	Ca_kabuli_chr06	27710490	[G/C]	1067300367
Ca_Kabuli_NC_021165.1_27710496	Ca_kabuli_chr06	27710496	[G/C]	1067300368
Ca_Kabuli_NC_021165.1_27710498	Ca_kabuli_chr06	27710498	[T/C]	1067300369
Ca_Kabuli_NC_021165.1_27710509	Ca_kabuli_chr06	27710509	[T/A]	1067300370
Ca_Kabuli_NC_021165.1_27787753	Ca_kabuli_chr06	27787753	[A/C]	1067300371
Ca_Kabuli_NC_021165.1_27787764	Ca_kabuli_chr06	27787764	[A/G]	1067300372
Ca_Kabuli_NC_021165.1_27932027	Ca_kabuli_chr06	27932027	[A/T]	1067300373
Ca_Kabuli_NC_021165.1_27986981	Ca_kabuli_chr06	27986981	[T/G]	1067300374
Ca_Kabuli_NC_021165.1_27987009	Ca_kabuli_chr06	27987009	[T/G]	1067300375
Ca_Kabuli_NC_021165.1_27987027	Ca_kabuli_chr06	27987027	[C/G]	1067300376
Ca_Kabuli_NC_021165.1_28044768	Ca_kabuli_chr06	28044768	[A/G]	1067300377
Ca_Kabuli_NC_021165.1_28044805	Ca_kabuli_chr06	28044805	[A/T]	1067300378
Ca_Kabuli_NC_021165.1_28044828	Ca_kabuli_chr06	28044828	[T/C]	1067300379
Ca_Kabuli_NC_021165.1_28045047	Ca_kabuli_chr06	28045047	[G/T]	1067300380
Ca_Kabuli_NC_021165.1_28052463	Ca_kabuli_chr06	28052463	[T/G]	1067300381
Ca_Kabuli_NC_021165.1_28074358	Ca_kabuli_chr06	28074358	[T/G]	1067300382
Ca_Kabuli_NC_021165.1_28076458	Ca_kabuli_chr06	28076458	[A/G]	1067300383
Ca_Kabuli_NC_021165.1_28077304	Ca_kabuli_chr06	28077304	[A/T]	1067300384
Ca_Kabuli_NC_021165.1_28077319	Ca_kabuli_chr06	28077319	[T/C]	1067300385
Ca_Kabuli_NC_021165.1_28077420	Ca_kabuli_chr06	28077420	[C/A]	1067300386
Ca_Kabuli_NC_021165.1_28077421	Ca_kabuli_chr06	28077421	[T/G]	1067300387
Ca_Kabuli_NC_021165.1_28077422	Ca_kabuli_chr06	28077422	[G/A]	1067300388
Ca_Kabuli_NC_021165.1_28177371	Ca_kabuli_chr06	28177371	[T/G]	1067300389
Ca_Kabuli_NC_021165.1_28177388	Ca_kabuli_chr06	28177388	[A/C]	1067300390
Ca_Kabuli_NC_021165.1_28177397	Ca_kabuli_chr06	28177397	[A/C]	1067300391
Ca_Kabuli_NC_021165.1_28177400	Ca_kabuli_chr06	28177400	[T/C]	1067300392
Ca_Kabuli_NC_021165.1_28177429	Ca_kabuli_chr06	28177429	[A/C]	1067300393
Ca_Kabuli_NC_021165.1_28177469	Ca_kabuli_chr06	28177469	[T/C]	1067300394

SNP IDs	Chromosomes	Physical positions (bp)	SNPs	NCBI SNPdb accession IDs (NCBI_ss#)
Ca_Kabuli_NC_021165.1_28177481	Ca_kabuli_chr06	28177481	[A/T]	1067300395
Ca_Kabuli_NC_021165.1_28284375	Ca_kabuli_chr06	28284375	[T/C]	1067300396
Ca_Kabuli_NC_021165.1_28325435	Ca_kabuli_chr06	28325435	[A/C]	1067300397
Ca_Kabuli_NC_021165.1_28325513	Ca_kabuli_chr06	28325513	[T/C]	1067300398
Ca_Kabuli_NC_021165.1_28325519	Ca_kabuli_chr06	28325519	[T/C]	1067300399
Ca_Kabuli_NC_021165.1_28325531	Ca_kabuli_chr06	28325531	[A/G]	1067300400
Ca_Kabuli_NC_021165.1_28325568	Ca_kabuli_chr06	28325568	[A/G]	1067300401
Ca_Kabuli_NC_021165.1_28325574	Ca_kabuli_chr06	28325574	[A/G]	1067300402
Ca_Kabuli_NC_021165.1_28325585	Ca_kabuli_chr06	28325585	[A/G]	1067300403
Ca_Kabuli_NC_021165.1_28325590	Ca_kabuli_chr06	28325590	[A/G]	1067300404
Ca_Kabuli_NC_021165.1_28453063	Ca_kabuli_chr06	28453063	[G/A]	1067300405
Ca_Kabuli_NC_021165.1_28575488	Ca_kabuli_chr06	28575488	[A/G]	1067300406
Ca_Kabuli_NC_021165.1_28575493	Ca_kabuli_chr06	28575493	[T/C]	1067300407
Ca_Kabuli_NC_021165.1_28575532	Ca_kabuli_chr06	28575532	[T/C]	1067300408
Ca_Kabuli_NC_021165.1_28612887	Ca_kabuli_chr06	28612887	[T/G]	1067300409
Ca_Kabuli_NC_021165.1_28612933	Ca_kabuli_chr06	28612933	[A/G]	1067300410
Ca_Kabuli_NC_021165.1_28727489	Ca_kabuli_chr06	28727489	[A/G]	1067300411
Ca_Kabuli_NC_021165.1_28727524	Ca_kabuli_chr06	28727524	[A/G]	1067300412
Ca_Kabuli_NC_021165.1_28893384	Ca_kabuli_chr06	28893384	[A/T]	1067300413
Ca_Kabuli_NC_021165.1_28893404	Ca_kabuli_chr06	28893404	[A/C]	1067300414
Ca_Kabuli_NC_021165.1_28893408	Ca_kabuli_chr06	28893408	[A/G]	1067300415
Ca_Kabuli_NC_021165.1_28893432	Ca_kabuli_chr06	28893432	[C/A]	1067300416
Ca_Kabuli_NC_021165.1_28893435	Ca_kabuli_chr06	28893435	[A/C]	1067300417
Ca_Kabuli_NC_021165.1_28893442	Ca_kabuli_chr06	28893442	[G/C]	1067300418
Ca_Kabuli_NC_021165.1_28893444	Ca_kabuli_chr06	28893444	[A/C]	1067300419
Ca_Kabuli_NC_021165.1_28893446	Ca_kabuli_chr06	28893446	[A/G]	1067300420
Ca_Kabuli_NC_021165.1_28916024	Ca_kabuli_chr06	28916024	[A/C]	1067300421
Ca_Kabuli_NC_021165.1_28916035	Ca_kabuli_chr06	28916035	[A/C]	1067300422
Ca_Kabuli_NC_021165.1_28917802	Ca_kabuli_chr06	28917802	[C/A]	1067300423
Ca_Kabuli_NC_021165.1_28917803	Ca_kabuli_chr06	28917803	[T/G]	1067300424
Ca_Kabuli_NC_021165.1_28917804	Ca_kabuli_chr06	28917804	[T/A]	1067300425
Ca_Kabuli_NC_021165.1_28917805	Ca_kabuli_chr06	28917805	[C/T]	1067300426
Ca_Kabuli_NC_021165.1_28990255	Ca_kabuli_chr06	28990255	[C/A]	1067300427
Ca_Kabuli_NC_021165.1_28990256	Ca_kabuli_chr06	28990256	[T/G]	1067300428

SNP IDs	Chromosomes	Physical positions (bp)	SNPs	NCBI SNPdb accession IDs (NCBI_ss#)
Ca_Kabuli_NC_021165.1_28990257	Ca_kabuli_chr06	28990257	[T/A]	1067300429
Ca_Kabuli_NC_021165.1_29163022	Ca_kabuli_chr06	29163022	[C/G]	1067300430
Ca_Kabuli_NC_021165.1_29163667	Ca_kabuli_chr06	29163667	[C/A]	1067300431
Ca_Kabuli_NC_021165.1_29203213	Ca_kabuli_chr06	29203213	[A/T]	1067300432
Ca_Kabuli_NC_021165.1_29203225	Ca_kabuli_chr06	29203225	[T/A]	1067300433
Ca_Kabuli_NC_021165.1_29203239	Ca_kabuli_chr06	29203239	[A/C]	1067300434
Ca_Kabuli_NC_021165.1_29203250	Ca_kabuli_chr06	29203250	[T/C]	1067300435
Ca_Kabuli_NC_021165.1_29205940	Ca_kabuli_chr06	29205940	[A/G]	1067300436
Ca_Kabuli_NC_021165.1_29277100	Ca_kabuli_chr06	29277100	[A/C]	1067300437
Ca_Kabuli_NC_021165.1_29277101	Ca_kabuli_chr06	29277101	[G/C]	1067300438
Ca_Kabuli_NC_021165.1_29338811	Ca_kabuli_chr06	29338811	[A/C]	1067300439
Ca_Kabuli_NC_021165.1_29498817	Ca_kabuli_chr06	29498817	[G/C]	1067300440
Ca_Kabuli_NC_021165.1_29689562	Ca_kabuli_chr06	29689562	[C/T]	1067300441
Ca_Kabuli_NC_021165.1_29870004	Ca_kabuli_chr06	29870004	[T/C]	1067300442
Ca_Kabuli_NC_021165.1_29870058	Ca_kabuli_chr06	29870058	[A/G]	1067300443
Ca_Kabuli_NC_021165.1_30087460	Ca_kabuli_chr06	30087460	[A/T]	1067300444
Ca_Kabuli_NC_021165.1_30087491	Ca_kabuli_chr06	30087491	[T/C]	1067300445
Ca_Kabuli_NC_021165.1_30090242	Ca_kabuli_chr06	30090242	[A/G]	1067300446
Ca_Kabuli_NC_021165.1_30127756	Ca_kabuli_chr06	30127756	[T/G]	1067300447
Ca_Kabuli_NC_021165.1_30127759	Ca_kabuli_chr06	30127759	[T/G]	1067300448
Ca_Kabuli_NC_021165.1_30417343	Ca_kabuli_chr06	30417343	[T/C]	1067300449
Ca_Kabuli_NC_021165.1_30417345	Ca_kabuli_chr06	30417345	[A/T]	1067300450
Ca_Kabuli_NC_021165.1_30417405	Ca_kabuli_chr06	30417405	[T/A]	1067300451
Ca_Kabuli_NC_021165.1_30417407	Ca_kabuli_chr06	30417407	[A/T]	1067300452
Ca_Kabuli_NC_021165.1_30674025	Ca_kabuli_chr06	30674025	[A/G]	1067300453
Ca_Kabuli_NC_021165.1_30674057	Ca_kabuli_chr06	30674057	[A/G]	1067300454
Ca_Kabuli_NC_021165.1_30674068	Ca_kabuli_chr06	30674068	[A/C]	1067300455
Ca_Kabuli_NC_021165.1_30676075	Ca_kabuli_chr06	30676075	[A/C]	1067300456
Ca_Kabuli_NC_021165.1_30730608	Ca_kabuli_chr06	30730608	[G/T]	1067300457
Ca_Kabuli_NC_021165.1_30927228	Ca_kabuli_chr06	30927228	[T/A]	1067300458
Ca_Kabuli_NC_021165.1_30927240	Ca_kabuli_chr06	30927240	[T/C]	1067300459
Ca_Kabuli_NC_021165.1_31011646	Ca_kabuli_chr06	31011646	[A/T]	1067300460
Ca_Kabuli_NC_021165.1_31042117	Ca_kabuli_chr06	31042117	[T/C]	1067300461
Ca_Kabuli_NC_021165.1_31049113	Ca_kabuli_chr06	31049113	[T/A]	1067300462

SNP IDs	Chromosomes	Physical positions (bp)	SNPs	NCBI SNPdb accession IDs (NCBI_ss#)
Ca_Kabuli_NC_021165.1_31057806	Ca_kabuli_chr06	31057806	[C/T]	1067300463
Ca_Kabuli_NC_021165.1_31250732	Ca_kabuli_chr06	31250732	[A/C]	1067300464
Ca_Kabuli_NC_021165.1_31250840	Ca_kabuli_chr06	31250840	[C/A]	1067300465
Ca_Kabuli_NC_021165.1_31250841	Ca_kabuli_chr06	31250841	[A/G]	1067300466
Ca_Kabuli_NC_021165.1_31250843	Ca_kabuli_chr06	31250843	[A/T]	1067300467
Ca_Kabuli_NC_021165.1_31250844	Ca_kabuli_chr06	31250844	[T/C]	1067300468
Ca_Kabuli_NC_021165.1_31294555	Ca_kabuli_chr06	31294555	[T/C]	1067300469
Ca_Kabuli_NC_021165.1_31324962	Ca_kabuli_chr06	31324962	[A/C]	1067300470
Ca_Kabuli_NC_021165.1_31324976	Ca_kabuli_chr06	31324976	[A/G]	1067300471
Ca_Kabuli_NC_021165.1_31898794	Ca_kabuli_chr06	31898794	[A/T]	1067300472
Ca_Kabuli_NC_021165.1_31900733	Ca_kabuli_chr06	31900733	[A/G]	1067300473
Ca_Kabuli_NC_021165.1_31900738	Ca_kabuli_chr06	31900738	[T/A]	1067300474
Ca_Kabuli_NC_021165.1_31900757	Ca_kabuli_chr06	31900757	[A/G]	1067300475
Ca_Kabuli_NC_021165.1_31900768	Ca_kabuli_chr06	31900768	[A/C]	1067300476
Ca_Kabuli_NC_021165.1_31902048	Ca_kabuli_chr06	31902048	[A/C]	1067300477
Ca_Kabuli_NC_021165.1_31902111	Ca_kabuli_chr06	31902111	[A/G]	1067300478
Ca_Kabuli_NC_021165.1_31902122	Ca_kabuli_chr06	31902122	[A/C]	1067300479
Ca_Kabuli_NC_021165.1_31902127	Ca_kabuli_chr06	31902127	[A/G]	1067300480
Ca_Kabuli_NC_021165.1_31902147	Ca_kabuli_chr06	31902147	[T/A]	1067300481
Ca_Kabuli_NC_021165.1_31902165	Ca_kabuli_chr06	31902165	[A/T]	1067300482
Ca_Kabuli_NC_021165.1_31910391	Ca_kabuli_chr06	31910391	[T/C]	1067300483
Ca_Kabuli_NC_021165.1_31990438	Ca_kabuli_chr06	31990438	[C/T]	1067300484
Ca_Kabuli_NC_021165.1_32012370	Ca_kabuli_chr06	32012370	[T/C]	1067300485
Ca_Kabuli_NC_021165.1_32035932	Ca_kabuli_chr06	32035932	[C/A]	1067300486
Ca_Kabuli_NC_021165.1_32035933	Ca_kabuli_chr06	32035933	[T/G]	1067300487
Ca_Kabuli_NC_021165.1_32035934	Ca_kabuli_chr06	32035934	[G/A]	1067300488
Ca_Kabuli_NC_021165.1_32149994	Ca_kabuli_chr06	32149994	[A/G]	1067300489
Ca_Kabuli_NC_021165.1_32149996	Ca_kabuli_chr06	32149996	[A/T]	1067300490
Ca_Kabuli_NC_021165.1_32201431	Ca_kabuli_chr06	32201431	[A/G]	1067300491
Ca_Kabuli_NC_021165.1_32201432	Ca_kabuli_chr06	32201432	[G/C]	1067300492
Ca_Kabuli_NC_021165.1_32262837	Ca_kabuli_chr06	32262837	[T/A]	1067300493
Ca_Kabuli_NC_021165.1_32717450	Ca_kabuli_chr06	32717450	[A/G]	1067300494
Ca_Kabuli_NC_021165.1_32718707	Ca_kabuli_chr06	32718707	[A/T]	1067300495
Ca_Kabuli_NC_021165.1_32718749	Ca_kabuli_chr06	32718749	[A/C]	1067300496

SNP IDs	Chromosomes	Physical positions (bp)	SNPs	NCBI SNPdb accession IDs (NCBI_ss#)
Ca_Kabuli_NC_021165.1_32798167	Ca_kabuli_chr06	32798167	[T/C]	1067300497
Ca_Kabuli_NC_021165.1_33168758	Ca_kabuli_chr06	33168758	[A/T]	1067300498
Ca_Kabuli_NC_021165.1_33495705	Ca_kabuli_chr06	33495705	[G/T]	1067300499
Ca_Kabuli_NC_021165.1_33495707	Ca_kabuli_chr06	33495707	[G/A]	1067300500
Ca_Kabuli_NC_021165.1_33495711	Ca_kabuli_chr06	33495711	[C/T]	1067300501
Ca_Kabuli_NC_021165.1_33495714	Ca_kabuli_chr06	33495714	[A/G]	1067300502
Ca_Kabuli_NC_021165.1_33495716	Ca_kabuli_chr06	33495716	[G/C]	1067300503
Ca_Kabuli_NC_021165.1_33733242	Ca_kabuli_chr06	33733242	[A/G]	1067300504
Ca_Kabuli_NC_021165.1_33734198	Ca_kabuli_chr06	33734198	[T/G]	1067300505
Ca_Kabuli_NC_021165.1_33759054	Ca_kabuli_chr06	33759054	[T/G]	1067300506
Ca_Kabuli_NC_021165.1_33759903	Ca_kabuli_chr06	33759903	[A/C]	1067300507
Ca_Kabuli_NC_021165.1_34222034	Ca_kabuli_chr06	34222034	[G/A]	1067300508
Ca_Kabuli_NC_021165.1_34231196	Ca_kabuli_chr06	34231196	[C/A]	1067300509
Ca_Kabuli_NC_021165.1_34235488	Ca_kabuli_chr06	34235488	[T/G]	1067300510
Ca_Kabuli_NC_021165.1_34235592	Ca_kabuli_chr06	34235592	[G/A]	1067300511
Ca_Kabuli_NC_021165.1_34235593	Ca_kabuli_chr06	34235593	[T/A]	1067300512
Ca_Kabuli_NC_021165.1_34367857	Ca_kabuli_chr06	34367857	[A/C]	1067300513
Ca_Kabuli_NC_021165.1_34481203	Ca_kabuli_chr06	34481203	[T/A]	1067300514
Ca_Kabuli_NC_021165.1_34580525	Ca_kabuli_chr06	34580525	[G/C]	1067300515
Ca_Kabuli_NC_021165.1_34633673	Ca_kabuli_chr06	34633673	[T/C]	1067300516
Ca_Kabuli_NC_021165.1_34633741	Ca_kabuli_chr06	34633741	[T/G]	1067300517
Ca_Kabuli_NC_021165.1_34651998	Ca_kabuli_chr06	34651998	[T/G]	1067300518
Ca_Kabuli_NC_021165.1_34652026	Ca_kabuli_chr06	34652026	[A/T]	1067300519
Ca_Kabuli_NC_021165.1_34771240	Ca_kabuli_chr06	34771240	[T/C]	1067300520
Ca_Kabuli_NC_021165.1_34771284	Ca_kabuli_chr06	34771284	[T/A]	1067300521
Ca_Kabuli_NC_021165.1_34771294	Ca_kabuli_chr06	34771294	[G/A]	1067300522
Ca_Kabuli_NC_021165.1_34771296	Ca_kabuli_chr06	34771296	[G/A]	1067300523
Ca_Kabuli_NC_021165.1_35053206	Ca_kabuli_chr06	35053206	[T/C]	1067300524
Ca_Kabuli_NC_021165.1_35126241	Ca_kabuli_chr06	35126241	[T/G]	1067300525
Ca_Kabuli_NC_021165.1_35126359	Ca_kabuli_chr06	35126359	[C/A]	1067300526
Ca_Kabuli_NC_021165.1_35337971	Ca_kabuli_chr06	35337971	[A/G]	1067300527
Ca_Kabuli_NC_021165.1_35337974	Ca_kabuli_chr06	35337974	[A/C]	1067300528
Ca_Kabuli_NC_021165.1_35337983	Ca_kabuli_chr06	35337983	[A/G]	1067300529
Ca_Kabuli_NC_021165.1_35337990	Ca_kabuli_chr06	35337990	[T/A]	1067300530

SNP IDs	Chromosomes	Physical positions (bp)	SNPs	NCBI SNPdb accession IDs (NCBI_ss#)
Ca_Kabuli_NC_021165.1_35338014	Ca_kabuli_chr06	35338014	[T/G]	1067300531
Ca_Kabuli_NC_021165.1_35596295	Ca_kabuli_chr06	35596295	[A/G]	1067300532
Ca_Kabuli_NC_021165.1_35630149	Ca_kabuli_chr06	35630149	[A/G]	1067300533
Ca_Kabuli_NC_021165.1_35719930	Ca_kabuli_chr06	35719930	[A/G]	1067300534
Ca_Kabuli_NC_021165.1_35719986	Ca_kabuli_chr06	35719986	[T/C]	1067300535
Ca_Kabuli_NC_021165.1_35719999	Ca_kabuli_chr06	35719999	[A/G]	1067300536
Ca_Kabuli_NC_021165.1_35750935	Ca_kabuli_chr06	35750935	[G/A]	1067300537
Ca_Kabuli_NC_021165.1_35750946	Ca_kabuli_chr06	35750946	[G/T]	1067300538
Ca_Kabuli_NC_021165.1_35750948	Ca_kabuli_chr06	35750948	[C/A]	1067300539
Ca_Kabuli_NC_021165.1_35750951	Ca_kabuli_chr06	35750951	[A/G]	1067300540
Ca_Kabuli_NC_021165.1_35750955	Ca_kabuli_chr06	35750955	[T/C]	1067300541
Ca_Kabuli_NC_021165.1_35796236	Ca_kabuli_chr06	35796236	[T/C]	1067300542
Ca_Kabuli_NC_021165.1_36082910	Ca_kabuli_chr06	36082910	[G/C]	1067300543
Ca_Kabuli_NC_021165.1_36082922	Ca_kabuli_chr06	36082922	[T/C]	1067300544
Ca_Kabuli_NC_021165.1_36082937	Ca_kabuli_chr06	36082937	[C/T]	1067300545
Ca_Kabuli_NC_021165.1_36082950	Ca_kabuli_chr06	36082950	[T/C]	1067300546
Ca_Kabuli_NC_021165.1_36227451	Ca_kabuli_chr06	36227451	[T/C]	1067300547
Ca_Kabuli_NC_021165.1_36227461	Ca_kabuli_chr06	36227461	[A/C]	1067300548
Ca_Kabuli_NC_021165.1_36227464	Ca_kabuli_chr06	36227464	[A/C]	1067300549
Ca_Kabuli_NC_021165.1_36246972	Ca_kabuli_chr06	36246972	[C/T]	1067300550
Ca_Kabuli_NC_021165.1_36247018	Ca_kabuli_chr06	36247018	[A/T]	1067300551
Ca_Kabuli_NC_021165.1_36309173	Ca_kabuli_chr06	36309173	[T/G]	1067300552
Ca_Kabuli_NC_021165.1_36309183	Ca_kabuli_chr06	36309183	[T/G]	1067300553
Ca_Kabuli_NC_021165.1_36309198	Ca_kabuli_chr06	36309198	[A/G]	1067300554
Ca_Kabuli_NC_021165.1_36580491	Ca_kabuli_chr06	36580491	[A/G]	1067300555
Ca_Kabuli_NC_021165.1_36689020	Ca_kabuli_chr06	36689020	[T/C]	1067300556
Ca_Kabuli_NC_021165.1_36689027	Ca_kabuli_chr06	36689027	[T/C]	1067300557
Ca_Kabuli_NC_021165.1_36689043	Ca_kabuli_chr06	36689043	[C/T]	1067300558
Ca_Kabuli_NC_021165.1_36689052	Ca_kabuli_chr06	36689052	[A/G]	1067300559
Ca_Kabuli_NC_021165.1_36689055	Ca_kabuli_chr06	36689055	[A/T]	1067300560
Ca_Kabuli_NC_021165.1_36699170	Ca_kabuli_chr06	36699170	[A/G]	1067300561
Ca_Kabuli_NC_021165.1_36861457	Ca_kabuli_chr06	36861457	[C/A]	1067300562
Ca_Kabuli_NC_021165.1_36861458	Ca_kabuli_chr06	36861458	[T/G]	1067300563
Ca_Kabuli_NC_021165.1_36861459	Ca_kabuli_chr06	36861459	[T/A]	1067300564

SNP IDs	Chromosomes	Physical positions (bp)	SNPs	NCBI SNPdb accession IDs (NCBI_ss#)
Ca_Kabuli_NC_021165.1_36861462	Ca_kabuli_chr06	36861462	[T/G]	1067300565
Ca_Kabuli_NC_021165.1_36895302	Ca_kabuli_chr06	36895302	[G/C]	1067300566
Ca_Kabuli_NC_021165.1_36901748	Ca_kabuli_chr06	36901748	[A/C]	1067300567
Ca_Kabuli_NC_021165.1_36985216	Ca_kabuli_chr06	36985216	[G/C]	1067300568
Ca_Kabuli_NC_021165.1_36985252	Ca_kabuli_chr06	36985252	[C/T]	1067300569
Ca_Kabuli_NC_021165.1_37139557	Ca_kabuli_chr06	37139557	[A/C]	1067300570
Ca_Kabuli_NC_021165.1_37280074	Ca_kabuli_chr06	37280074	[A/C]	1067300571
Ca_Kabuli_NC_021165.1_37280178	Ca_kabuli_chr06	37280178	[T/C]	1067300572
Ca_Kabuli_NC_021165.1_37490114	Ca_kabuli_chr06	37490114	[A/G]	1067300573
Ca_Kabuli_NC_021165.1_37491353	Ca_kabuli_chr06	37491353	[T/G]	1067300574
Ca_Kabuli_NC_021165.1_37491367	Ca_kabuli_chr06	37491367	[T/A]	1067300575
Ca_Kabuli_NC_021165.1_37491424	Ca_kabuli_chr06	37491424	[A/G]	1067300576
Ca_Kabuli_NC_021165.1_37491450	Ca_kabuli_chr06	37491450	[T/G]	1067300577
Ca_Kabuli_NC_021165.1_37545930	Ca_kabuli_chr06	37545930	[T/G]	1067300578
Ca_Kabuli_NC_021165.1_37545952	Ca_kabuli_chr06	37545952	[A/G]	1067300579
Ca_Kabuli_NC_021165.1_37943905	Ca_kabuli_chr06	37943905	[C/T]	1067300580
Ca_Kabuli_NC_021165.1_38106825	Ca_kabuli_chr06	38106825	[T/C]	1067300581
Ca_Kabuli_NC_021165.1_38313733	Ca_kabuli_chr06	38313733	[A/C]	1067300582
Ca_Kabuli_NC_021165.1_38333209	Ca_kabuli_chr06	38333209	[A/C]	1067300583
Ca_Kabuli_NC_021165.1_38363668	Ca_kabuli_chr06	38363668	[T/A]	1067300584
Ca_Kabuli_NC_021165.1_38499752	Ca_kabuli_chr06	38499752	[C/T]	1067300585
Ca_Kabuli_NC_021165.1_38499762	Ca_kabuli_chr06	38499762	[C/T]	1067300586
Ca_Kabuli_NC_021165.1_38514126	Ca_kabuli_chr06	38514126	[G/T]	1067300587
Ca_Kabuli_NC_021165.1_38514165	Ca_kabuli_chr06	38514165	[G/A]	1067300588
Ca_Kabuli_NC_021165.1_38514168	Ca_kabuli_chr06	38514168	[G/A]	1067300589
Ca_Kabuli_NC_021165.1_38563881	Ca_kabuli_chr06	38563881	[T/A]	1067300590
Ca_Kabuli_NC_021165.1_38563888	Ca_kabuli_chr06	38563888	[G/T]	1067300591
Ca_Kabuli_NC_021165.1_38563895	Ca_kabuli_chr06	38563895	[T/G]	1067300592
Ca_Kabuli_NC_021165.1_38563902	Ca_kabuli_chr06	38563902	[G/T]	1067300593
Ca_Kabuli_NC_021165.1_38810458	Ca_kabuli_chr06	38810458	[T/C]	1067300594
Ca_Kabuli_NC_021165.1_38814616	Ca_kabuli_chr06	38814616	[A/C]	1067300595
Ca_Kabuli_NC_021165.1_38814637	Ca_kabuli_chr06	38814637	[T/A]	1067300596
Ca_Kabuli_NC_021165.1_38816366	Ca_kabuli_chr06	38816366	[T/A]	1067300597
Ca_Kabuli_NC_021165.1_38816452	Ca_kabuli_chr06	38816452	[A/C]	1067300598

SNP IDs	Chromosomes	Physical positions (bp)	SNPs	NCBI SNPdb accession IDs (NCBI_ss#)
Ca_Kabuli_NC_021165.1_38838254	Ca_kabuli_chr06	38838254	[T/G]	1067300599
Ca_Kabuli_NC_021165.1_39028626	Ca_kabuli_chr06	39028626	[C/T]	1067300600
Ca_Kabuli_NC_021165.1_39049958	Ca_kabuli_chr06	39049958	[G/T]	1067300601
Ca_Kabuli_NC_021165.1_39049967	Ca_kabuli_chr06	39049967	[T/C]	1067300602
Ca_Kabuli_NC_021165.1_39184066	Ca_kabuli_chr06	39184066	[A/G]	1067300603
Ca_Kabuli_NC_021165.1_39382231	Ca_kabuli_chr06	39382231	[G/A]	1067300604
Ca_Kabuli_NC_021165.1_39382240	Ca_kabuli_chr06	39382240	[T/A]	1067300605
Ca_Kabuli_NC_021165.1_39382244	Ca_kabuli_chr06	39382244	[T/A]	1067300606
Ca_Kabuli_NC_021165.1_39382248	Ca_kabuli_chr06	39382248	[G/A]	1067300607
Ca_Kabuli_NC_021165.1_39724016	Ca_kabuli_chr06	39724016	[A/C]	1067300608
Ca_Kabuli_NC_021165.1_39724022	Ca_kabuli_chr06	39724022	[A/G]	1067300609
Ca_Kabuli_NC_021165.1_39908312	Ca_kabuli_chr06	39908312	[A/G]	1067300610
Ca_Kabuli_NC_021165.1_39908314	Ca_kabuli_chr06	39908314	[T/G]	1067300611
Ca_Kabuli_NC_021165.1_39998978	Ca_kabuli_chr06	39998978	[T/G]	1067300612
Ca_Kabuli_NC_021165.1_40128644	Ca_kabuli_chr06	40128644	[T/A]	1067300613
Ca_Kabuli_NC_021165.1_40128882	Ca_kabuli_chr06	40128882	[A/C]	1067300614
Ca_Kabuli_NC_021165.1_40128885	Ca_kabuli_chr06	40128885	[A/C]	1067300615
Ca_Kabuli_NC_021165.1_40468352	Ca_kabuli_chr06	40468352	[A/G]	1067300616
Ca_Kabuli_NC_021165.1_40549244	Ca_kabuli_chr06	40549244	[T/C]	1067300617
Ca_Kabuli_NC_021165.1_40549247	Ca_kabuli_chr06	40549247	[A/G]	1067300618
Ca_Kabuli_NC_021165.1_40549286	Ca_kabuli_chr06	40549286	[T/C]	1067300619
Ca_Kabuli_NC_021165.1_40657640	Ca_kabuli_chr06	40657640	[A/G]	1067300620
Ca_Kabuli_NC_021165.1_40657667	Ca_kabuli_chr06	40657667	[T/G]	1067300621
Ca_Kabuli_NC_021165.1_40658958	Ca_kabuli_chr06	40658958	[A/T]	1067300622
Ca_Kabuli_NC_021165.1_40663891	Ca_kabuli_chr06	40663891	[T/G]	1067300623
Ca_Kabuli_NC_021165.1_40735314	Ca_kabuli_chr06	40735314	[T/G]	1067300624
Ca_Kabuli_NC_021165.1_40741517	Ca_kabuli_chr06	40741517	[A/G]	1067300625
Ca_Kabuli_NC_021165.1_40741531	Ca_kabuli_chr06	40741531	[A/G]	1067300626
Ca_Kabuli_NC_021165.1_40772661	Ca_kabuli_chr06	40772661	[T/C]	1067300627
Ca_Kabuli_NC_021165.1_40772680	Ca_kabuli_chr06	40772680	[C/T]	1067300628
Ca_Kabuli_NC_021165.1_40772683	Ca_kabuli_chr06	40772683	[A/T]	1067300629
Ca_Kabuli_NC_021165.1_40772700	Ca_kabuli_chr06	40772700	[T/C]	1067300630
Ca_Kabuli_NC_021165.1_40822859	Ca_kabuli_chr06	40822859	[T/A]	1067300631
Ca_Kabuli_NC_021165.1_40823945	Ca_kabuli_chr06	40823945	[T/G]	1067300632

SNP IDs	Chromosomes	Physical positions (bp)	SNPs	NCBI SNPdb accession IDs (NCBI_ss#)
Ca_Kabuli_NC_021165.1_40851410	Ca_kabuli_chr06	40851410	[T/C]	1067300633
Ca_Kabuli_NC_021165.1_40851438	Ca_kabuli_chr06	40851438	[A/G]	1067300634
Ca_Kabuli_NC_021165.1_40935002	Ca_kabuli_chr06	40935002	[G/A]	1067300635
Ca_Kabuli_NC_021165.1_40942535	Ca_kabuli_chr06	40942535	[G/C]	1067300636
Ca_Kabuli_NC_021165.1_40942536	Ca_kabuli_chr06	40942536	[A/G]	1067300637
Ca_Kabuli_NC_021165.1_41128736	Ca_kabuli_chr06	41128736	[C/T]	1067300638
Ca_Kabuli_NC_021165.1_41363527	Ca_kabuli_chr06	41363527	[T/A]	1067300639
Ca_Kabuli_NC_021165.1_41363528	Ca_kabuli_chr06	41363528	[A/T]	1067300640
Ca_Kabuli_NC_021165.1_41420912	Ca_kabuli_chr06	41420912	[A/C]	1067300641
Ca_Kabuli_NC_021165.1_41516043	Ca_kabuli_chr06	41516043	[A/T]	1067300642
Ca_Kabuli_NC_021165.1_41682896	Ca_kabuli_chr06	41682896	[A/C]	1067300643
Ca_Kabuli_NC_021165.1_41682971	Ca_kabuli_chr06	41682971	[T/C]	1067300644
Ca_Kabuli_NC_021165.1_41682983	Ca_kabuli_chr06	41682983	[T/C]	1067300645
Ca_Kabuli_NC_021165.1_41682989	Ca_kabuli_chr06	41682989	[T/C]	1067300646
Ca_Kabuli_NC_021165.1_41682991	Ca_kabuli_chr06	41682991	[T/C]	1067300647
Ca_Kabuli_NC_021165.1_41699062	Ca_kabuli_chr06	41699062	[T/C]	1067300648
Ca_Kabuli_NC_021165.1_41699132	Ca_kabuli_chr06	41699132	[A/G]	1067300649
Ca_Kabuli_NC_021165.1_41699134	Ca_kabuli_chr06	41699134	[A/C]	1067300650
Ca_Kabuli_NC_021165.1_41699148	Ca_kabuli_chr06	41699148	[T/C]	1067300651
Ca_Kabuli_NC_021165.1_41699151	Ca_kabuli_chr06	41699151	[T/G]	1067300652
Ca_Kabuli_NC_021165.1_41699168	Ca_kabuli_chr06	41699168	[T/C]	1067300653
Ca_Kabuli_NC_021165.1_41708169	Ca_kabuli_chr06	41708169	[G/T]	1067300654
Ca_Kabuli_NC_021165.1_41708170	Ca_kabuli_chr06	41708170	[C/G]	1067300655
Ca_Kabuli_NC_021165.1_41708171	Ca_kabuli_chr06	41708171	[A/C]	1067300656
Ca_Kabuli_NC_021165.1_41709034	Ca_kabuli_chr06	41709034	[A/T]	1067300657
Ca_Kabuli_NC_021165.1_41709036	Ca_kabuli_chr06	41709036	[T/C]	1067300658
Ca_Kabuli_NC_021165.1_41709077	Ca_kabuli_chr06	41709077	[T/C]	1067300659
Ca_Kabuli_NC_021165.1_41711538	Ca_kabuli_chr06	41711538	[A/G]	1067300660
Ca_Kabuli_NC_021165.1_42004789	Ca_kabuli_chr06	42004789	[T/G]	1067300661
Ca_Kabuli_NC_021165.1_42161902	Ca_kabuli_chr06	42161902	[A/G]	1067300662
Ca_Kabuli_NC_021165.1_42507557	Ca_kabuli_chr06	42507557	[A/C]	1067300663
Ca_Kabuli_NC_021165.1_42521161	Ca_kabuli_chr06	42521161	[T/G]	1067300664
Ca_Kabuli_NC_021165.1_42521252	Ca_kabuli_chr06	42521252	[A/C]	1067300665
Ca_Kabuli_NC_021165.1_42923879	Ca_kabuli_chr06	42923879	[T/G]	1067300666

SNP IDs	Chromosomes	Physical positions (bp)	SNPs	NCBI SNPdb accession IDs (NCBI_ss#)
Ca_Kabuli_NC_021165.1_42923920	Ca_kabuli_chr06	42923920	[T/G]	1067300667
Ca_Kabuli_NC_021165.1_43682714	Ca_kabuli_chr06	43682714	[T/C]	1067300668
Ca_Kabuli_NC_021165.1_43682734	Ca_kabuli_chr06	43682734	[T/C]	1067300669
Ca_Kabuli_NC_021165.1_43878859	Ca_kabuli_chr06	43878859	[G/C]	1067300670
Ca_Kabuli_NC_021165.1_44022221	Ca_kabuli_chr06	44022221	[A/C]	1067300671
Ca_Kabuli_NC_021165.1_44385520	Ca_kabuli_chr06	44385520	[T/G]	1067300672
Ca_Kabuli_NC_021165.1_44385534	Ca_kabuli_chr06	44385534	[T/A]	1067300673
Ca_Kabuli_NC_021165.1_44385550	Ca_kabuli_chr06	44385550	[C/T]	1067300674
Ca_Kabuli_NC_021165.1_44395072	Ca_kabuli_chr06	44395072	[A/C]	1067300675
Ca_Kabuli_NC_021165.1_44530868	Ca_kabuli_chr06	44530868	[A/G]	1067300676
Ca_Kabuli_NC_021165.1_44711166	Ca_kabuli_chr06	44711166	[A/G]	1067300677
Ca_Kabuli_NC_021165.1_44944937	Ca_kabuli_chr06	44944937	[T/C]	1067300678
Ca_Kabuli_NC_021165.1_44944965	Ca_kabuli_chr06	44944965	[T/C]	1067300679
Ca_Kabuli_NC_021165.1_45096150	Ca_kabuli_chr06	45096150	[A/T]	1067300680
Ca_Kabuli_NC_021165.1_45131334	Ca_kabuli_chr06	45131334	[A/C]	1067300681
Ca_Kabuli_NC_021165.1_45132447	Ca_kabuli_chr06	45132447	[T/G]	1067300682
Ca_Kabuli_NC_021165.1_45239883	Ca_kabuli_chr06	45239883	[T/G]	1067300683
Ca_Kabuli_NC_021165.1_45293271	Ca_kabuli_chr06	45293271	[T/C]	1067300684
Ca_Kabuli_NC_021165.1_45345875	Ca_kabuli_chr06	45345875	[A/T]	1067300685
Ca_Kabuli_NC_021165.1_45419172	Ca_kabuli_chr06	45419172	[T/A]	1067300686
Ca_Kabuli_NC_021165.1_45500859	Ca_kabuli_chr06	45500859	[T/G]	1067300687
Ca_Kabuli_NC_021165.1_45500898	Ca_kabuli_chr06	45500898	[A/G]	1067300688
Ca_Kabuli_NC_021165.1_45519697	Ca_kabuli_chr06	45519697	[A/G]	1067300689
Ca_Kabuli_NC_021165.1_45549997	Ca_kabuli_chr06	45549997	[A/G]	1067300690
Ca_Kabuli_NC_021165.1_45553955	Ca_kabuli_chr06	45553955	[A/C]	1067300691
Ca_Kabuli_NC_021165.1_45553961	Ca_kabuli_chr06	45553961	[T/C]	1067300692
Ca_Kabuli_NC_021165.1_45553975	Ca_kabuli_chr06	45553975	[T/C]	1067300693
Ca_Kabuli_NC_021165.1_45554034	Ca_kabuli_chr06	45554034	[A/G]	1067300694
Ca_Kabuli_NC_021165.1_45554061	Ca_kabuli_chr06	45554061	[A/C]	1067300695
Ca_Kabuli_NC_021165.1_45554694	Ca_kabuli_chr06	45554694	[T/C]	1067300696
Ca_Kabuli_NC_021165.1_45614058	Ca_kabuli_chr06	45614058	[A/C]	1067300697
Ca_Kabuli_NC_021165.1_45614095	Ca_kabuli_chr06	45614095	[A/C]	1067300698
Ca_Kabuli_NC_021165.1_45706389	Ca_kabuli_chr06	45706389	[A/T]	1067300699
Ca_Kabuli_NC_021165.1_45706413	Ca_kabuli_chr06	45706413	[A/G]	1067300700

SNP IDs	Chromosomes	Physical positions (bp)	SNPs	NCBI SNPdb accession IDs (NCBI_ss#)
Ca_Kabuli_NC_021165.1_45773324	Ca_kabuli_chr06	45773324	[T/G]	1067300701
Ca_Kabuli_NC_021165.1_45773603	Ca_kabuli_chr06	45773603	[A/C]	1067300702
Ca_Kabuli_NC_021165.1_45841032	Ca_kabuli_chr06	45841032	[G/T]	1067300703
Ca_Kabuli_NC_021165.1_46158378	Ca_kabuli_chr06	46158378	[A/C]	1067300704
Ca_Kabuli_NC_021165.1_46160997	Ca_kabuli_chr06	46160997	[T/C]	1067300705
Ca_Kabuli_NC_021165.1_46537515	Ca_kabuli_chr06	46537515	[T/G]	1067300706
Ca_Kabuli_NC_021165.1_46734665	Ca_kabuli_chr06	46734665	[C/T]	1067300707
Ca_Kabuli_NC_021165.1_46734675	Ca_kabuli_chr06	46734675	[T/C]	1067300708
Ca_Kabuli_NC_021165.1_46734678	Ca_kabuli_chr06	46734678	[A/T]	1067300709
Ca_Kabuli_NC_021165.1_46734682	Ca_kabuli_chr06	46734682	[T/A]	1067300710
Ca_Kabuli_NC_021165.1_46734687	Ca_kabuli_chr06	46734687	[G/T]	1067300711
Ca_Kabuli_NC_021165.1_46734706	Ca_kabuli_chr06	46734706	[C/G]	1067300712
Ca_Kabuli_NC_021165.1_46734717	Ca_kabuli_chr06	46734717	[A/G]	1067300713
Ca_Kabuli_NC_021165.1_46765671	Ca_kabuli_chr06	46765671	[C/A]	1067300714
Ca_Kabuli_NC_021165.1_46896938	Ca_kabuli_chr06	46896938	[G/C]	1067300715
Ca_Kabuli_NC_021165.1_46896958	Ca_kabuli_chr06	46896958	[C/T]	1067300716
Ca_Kabuli_NC_021165.1_46898699	Ca_kabuli_chr06	46898699	[G/T]	1067300717
Ca_Kabuli_NC_021165.1_46898751	Ca_kabuli_chr06	46898751	[A/T]	1067300718
Ca_Kabuli_NC_021165.1_46898792	Ca_kabuli_chr06	46898792	[A/T]	1067300719
Ca_Kabuli_NC_021165.1_46898828	Ca_kabuli_chr06	46898828	[C/T]	1067300720
Ca_Kabuli_NC_021165.1_46899073	Ca_kabuli_chr06	46899073	[T/C]	1067300721
Ca_Kabuli_NC_021165.1_46899078	Ca_kabuli_chr06	46899078	[A/T]	1067300722
Ca_Kabuli_NC_021165.1_46899631	Ca_kabuli_chr06	46899631	[G/A]	1067300723
Ca_Kabuli_NC_021165.1_46906116	Ca_kabuli_chr06	46906116	[A/G]	1067300724
Ca_Kabuli_NC_021165.1_46943899	Ca_kabuli_chr06	46943899	[T/C]	1067300725
Ca_Kabuli_NC_021165.1_46943919	Ca_kabuli_chr06	46943919	[T/C]	1067300726
Ca_Kabuli_NC_021165.1_46943933	Ca_kabuli_chr06	46943933	[A/G]	1067300727
Ca_Kabuli_NC_021165.1_46943937	Ca_kabuli_chr06	46943937	[C/G]	1067300728
Ca_Kabuli_NC_021165.1_46943942	Ca_kabuli_chr06	46943942	[A/C]	1067300729
Ca_Kabuli_NC_021165.1_46952750	Ca_kabuli_chr06	46952750	[A/G]	1067300730
Ca_Kabuli_NC_021165.1_46972676	Ca_kabuli_chr06	46972676	[A/C]	1067300731
Ca_Kabuli_NC_021165.1_46979196	Ca_kabuli_chr06	46979196	[A/G]	1067300732
Ca_Kabuli_NC_021165.1_46979210	Ca_kabuli_chr06	46979210	[A/G]	1067300733
Ca_Kabuli_NC_021165.1_46979213	Ca_kabuli_chr06	46979213	[T/G]	1067300734

SNP IDs	Chromosomes	Physical positions (bp)	SNPs	NCBI SNPdb accession IDs (NCBI_ss#)
Ca_Kabuli_NC_021165.1_46979216	Ca_kabuli_chr06	46979216	[C/G]	1067300735
Ca_Kabuli_NC_021165.1_46979234	Ca_kabuli_chr06	46979234	[G/A]	1067300736
Ca_Kabuli_NC_021165.1_47116476	Ca_kabuli_chr06	47116476	[T/C]	1067300737
Ca_Kabuli_NC_021165.1_47116504	Ca_kabuli_chr06	47116504	[A/G]	1067300738
Ca_Kabuli_NC_021165.1_47367057	Ca_kabuli_chr06	47367057	[T/A]	1067300739
Ca_Kabuli_NC_021165.1_47810677	Ca_kabuli_chr06	47810677	[A/C]	1067300740
Ca_Kabuli_NC_021165.1_47859599	Ca_kabuli_chr06	47859599	[T/A]	1067300741
Ca_Kabuli_NC_021165.1_47859601	Ca_kabuli_chr06	47859601	[G/C]	1067300742
Ca_Kabuli_NC_021165.1_47859602	Ca_kabuli_chr06	47859602	[G/T]	1067300743
Ca_Kabuli_NC_021165.1_48255531	Ca_kabuli_chr06	48255531	[T/C]	1067300744
Ca_Kabuli_NC_021165.1_48255538	Ca_kabuli_chr06	48255538	[G/A]	1067300745
Ca_Kabuli_NC_021165.1_48255558	Ca_kabuli_chr06	48255558	[C/T]	1067300746
Ca_Kabuli_NC_021165.1_48255559	Ca_kabuli_chr06	48255559	[A/G]	1067300747
Ca_Kabuli_NC_021165.1_48255568	Ca_kabuli_chr06	48255568	[G/A]	1067300748
Ca_Kabuli_NC_021165.1_48314834	Ca_kabuli_chr06	48314834	[C/A]	1067300749
Ca_Kabuli_NC_021165.1_48314841	Ca_kabuli_chr06	48314841	[T/G]	1067300750
Ca_Kabuli_NC_021165.1_48315220	Ca_kabuli_chr06	48315220	[G/C]	1067300751
Ca_Kabuli_NC_021165.1_48315232	Ca_kabuli_chr06	48315232	[A/C]	1067300752
Ca_Kabuli_NC_021165.1_48315289	Ca_kabuli_chr06	48315289	[A/T]	1067300753
Ca_Kabuli_NC_021165.1_48315293	Ca_kabuli_chr06	48315293	[G/T]	1067300754
Ca_Kabuli_NC_021165.1_48315958	Ca_kabuli_chr06	48315958	[A/C]	1067300755
Ca_Kabuli_NC_021165.1_48316046	Ca_kabuli_chr06	48316046	[A/G]	1067300756
Ca_Kabuli_NC_021165.1_48694554	Ca_kabuli_chr06	48694554	[A/T]	1067300757
Ca_Kabuli_NC_021165.1_48694559	Ca_kabuli_chr06	48694559	[T/C]	1067300758
Ca_Kabuli_NC_021165.1_48869512	Ca_kabuli_chr06	48869512	[C/G]	1067300759
Ca_Kabuli_NC_021165.1_49044794	Ca_kabuli_chr06	49044794	[T/A]	1067300760
Ca_Kabuli_NC_021165.1_49044802	Ca_kabuli_chr06	49044802	[C/T]	1067300761
Ca_Kabuli_NC_021165.1_49044805	Ca_kabuli_chr06	49044805	[A/G]	1067300762
Ca_Kabuli_NC_021165.1_49044814	Ca_kabuli_chr06	49044814	[G/T]	1067300763
Ca_Kabuli_NC_021165.1_49100642	Ca_kabuli_chr06	49100642	[A/G]	1067300764
Ca_Kabuli_NC_021165.1_49100644	Ca_kabuli_chr06	49100644	[A/G]	1067300765
Ca_Kabuli_NC_021165.1_49100649	Ca_kabuli_chr06	49100649	[A/G]	1067300766
Ca_Kabuli_NC_021165.1_49100673	Ca_kabuli_chr06	49100673	[T/G]	1067300767
Ca_Kabuli_NC_021165.1_49101898	Ca_kabuli_chr06	49101898	[A/G]	1067300768

SNP IDs	Chromosomes	Physical positions (bp)	SNPs	NCBI SNPdb accession IDs (NCBI_ss#)
Ca_Kabuli_NC_021165.1_49167362	Ca_kabuli_chr06	49167362	[A/C]	1067300769
Ca_Kabuli_NC_021165.1_49167389	Ca_kabuli_chr06	49167389	[A/G]	1067300770
Ca_Kabuli_NC_021165.1_49222290	Ca_kabuli_chr06	49222290	[C/T]	1067300771
Ca_Kabuli_NC_021165.1_49222291	Ca_kabuli_chr06	49222291	[G/A]	1067300772
Ca_Kabuli_NC_021165.1_49377275	Ca_kabuli_chr06	49377275	[G/A]	1067300773
Ca_Kabuli_NC_021165.1_49377336	Ca_kabuli_chr06	49377336	[G/A]	1067300774
Ca_Kabuli_NC_021165.1_49377339	Ca_kabuli_chr06	49377339	[T/G]	1067300775
Ca_Kabuli_NC_021165.1_49379106	Ca_kabuli_chr06	49379106	[A/C]	1067300776
Ca_Kabuli_NC_021165.1_49555020	Ca_kabuli_chr06	49555020	[G/T]	1067300777
Ca_Kabuli_NC_021165.1_49557927	Ca_kabuli_chr06	49557927	[T/A]	1067300778
Ca_Kabuli_NC_021165.1_49588009	Ca_kabuli_chr06	49588009	[T/G]	1067300779
Ca_Kabuli_NC_021165.1_49629674	Ca_kabuli_chr06	49629674	[A/G]	1067300780
Ca_Kabuli_NC_021165.1_49830362	Ca_kabuli_chr06	49830362	[T/G]	1067300781
Ca_Kabuli_NC_021165.1_49830429	Ca_kabuli_chr06	49830429	[T/G]	1067300782
Ca_Kabuli_NC_021165.1_49833240	Ca_kabuli_chr06	49833240	[T/G]	1067300783
Ca_Kabuli_NC_021165.1_49833848	Ca_kabuli_chr06	49833848	[T/C]	1067300784
Ca_Kabuli_NC_021165.1_49833857	Ca_kabuli_chr06	49833857	[A/C]	1067300785
Ca_Kabuli_NC_021165.1_49833860	Ca_kabuli_chr06	49833860	[A/C]	1067300786
Ca_Kabuli_NC_021165.1_49833864	Ca_kabuli_chr06	49833864	[A/C]	1067300787
Ca_Kabuli_NC_021165.1_49843847	Ca_kabuli_chr06	49843847	[T/G]	1067300788
Ca_Kabuli_NC_021165.1_49843850	Ca_kabuli_chr06	49843850	[T/G]	1067300789
Ca_Kabuli_NC_021165.1_49843855	Ca_kabuli_chr06	49843855	[A/G]	1067300790
Ca_Kabuli_NC_021165.1_49846303	Ca_kabuli_chr06	49846303	[T/G]	1067300791
Ca_Kabuli_NC_021165.1_49847543	Ca_kabuli_chr06	49847543	[T/C]	1067300792
Ca_Kabuli_NC_021165.1_49847559	Ca_kabuli_chr06	49847559	[A/C]	1067300793
Ca_Kabuli_NC_021165.1_49847576	Ca_kabuli_chr06	49847576	[T/G]	1067300794
Ca_Kabuli_NC_021165.1_49931910	Ca_kabuli_chr06	49931910	[A/G]	1067300795
Ca_Kabuli_NC_021165.1_49932955	Ca_kabuli_chr06	49932955	[T/A]	1067300796
Ca_Kabuli_NC_021165.1_49940580	Ca_kabuli_chr06	49940580	[A/C]	1067300797
Ca_Kabuli_NC_021165.1_50071948	Ca_kabuli_chr06	50071948	[T/G]	1067300798
Ca_Kabuli_NC_021165.1_50378387	Ca_kabuli_chr06	50378387	[A/C]	1067300799
Ca_Kabuli_NC_021165.1_50378388	Ca_kabuli_chr06	50378388	[G/T]	1067300800
Ca_Kabuli_NC_021165.1_50378389	Ca_kabuli_chr06	50378389	[A/T]	1067300801
Ca_Kabuli_NC_021165.1_50384166	Ca_kabuli_chr06	50384166	[A/C]	1067300802

SNP IDs	Chromosomes	Physical positions (bp)	SNPs	NCBI SNPdb accession IDs (NCBI_ss#)
Ca_Kabuli_NC_021165.1_50476698	Ca_kabuli_chr06	50476698	[T/C]	1067300803
Ca_Kabuli_NC_021165.1_50476785	Ca_kabuli_chr06	50476785	[T/A]	1067300804
Ca_Kabuli_NC_021165.1_50476844	Ca_kabuli_chr06	50476844	[A/C]	1067300805
Ca_Kabuli_NC_021165.1_50477669	Ca_kabuli_chr06	50477669	[G/A]	1067300806
Ca_Kabuli_NC_021165.1_50477676	Ca_kabuli_chr06	50477676	[A/C]	1067300807
Ca_Kabuli_NC_021165.1_50857740	Ca_kabuli_chr06	50857740	[A/G]	1067300808
Ca_Kabuli_NC_021165.1_50857798	Ca_kabuli_chr06	50857798	[T/G]	1067300809
Ca_Kabuli_NC_021165.1_51062466	Ca_kabuli_chr06	51062466	[A/C]	1067300810
Ca_Kabuli_NC_021165.1_51089783	Ca_kabuli_chr06	51089783	[T/A]	1067300811
Ca_Kabuli_NC_021165.1_51240949	Ca_kabuli_chr06	51240949	[A/C]	1067300812
Ca_Kabuli_NC_021165.1_51240950	Ca_kabuli_chr06	51240950	[G/C]	1067300813
Ca_Kabuli_NC_021165.1_51240951	Ca_kabuli_chr06	51240951	[A/G]	1067300814
Ca_Kabuli_NC_021165.1_51240952	Ca_kabuli_chr06	51240952	[T/A]	1067300815
Ca_Kabuli_NC_021165.1_51281239	Ca_kabuli_chr06	51281239	[A/G]	1067300816
Ca_Kabuli_NC_021165.1_51345504	Ca_kabuli_chr06	51345504	[A/G]	1067300817
Ca_Kabuli_NC_021165.1_51444290	Ca_kabuli_chr06	51444290	[C/T]	1067300818
Ca_Kabuli_NC_021165.1_51444301	Ca_kabuli_chr06	51444301	[C/T]	1067300819
Ca_Kabuli_NC_021165.1_51444314	Ca_kabuli_chr06	51444314	[T/G]	1067300820
Ca_Kabuli_NC_021165.1_51470212	Ca_kabuli_chr06	51470212	[G/C]	1067300821
Ca_Kabuli_NC_021165.1_51470213	Ca_kabuli_chr06	51470213	[G/T]	1067300822
Ca_Kabuli_NC_021165.1_51471558	Ca_kabuli_chr06	51471558	[T/C]	1067300823
Ca_Kabuli_NC_021165.1_51476913	Ca_kabuli_chr06	51476913	[T/C]	1067300824
Ca_Kabuli_NC_021165.1_51477123	Ca_kabuli_chr06	51477123	[T/C]	1067300825
Ca_Kabuli_NC_021165.1_51477378	Ca_kabuli_chr06	51477378	[T/G]	1067300826
Ca_Kabuli_NC_021165.1_51477380	Ca_kabuli_chr06	51477380	[T/C]	1067300827
Ca_Kabuli_NC_021165.1_51509329	Ca_kabuli_chr06	51509329	[G/A]	1067300828
Ca_Kabuli_NC_021165.1_51509360	Ca_kabuli_chr06	51509360	[A/G]	1067300829
Ca_Kabuli_NC_021165.1_52164520	Ca_kabuli_chr06	52164520	[T/A]	1067300830
Ca_Kabuli_NC_021165.1_52164537	Ca_kabuli_chr06	52164537	[T/C]	1067300831
Ca_Kabuli_NC_021165.1_52220143	Ca_kabuli_chr06	52220143	[A/T]	1067300832
Ca_Kabuli_NC_021165.1_52220165	Ca_kabuli_chr06	52220165	[T/C]	1067300833
Ca_Kabuli_NC_021165.1_52285978	Ca_kabuli_chr06	52285978	[T/G]	1067300834
Ca_Kabuli_NC_021165.1_52379657	Ca_kabuli_chr06	52379657	[A/G]	1067300835
Ca_Kabuli_NC_021165.1_52417728	Ca_kabuli_chr06	52417728	[T/A]	1067300836

SNP IDs	Chromosomes	Physical positions (bp)	SNPs	NCBI SNPdb accession IDs (NCBI_ss#)
Ca_Kabuli_NC_021165.1_52436255	Ca_kabuli_chr06	52436255	[A/C]	1067300837
Ca_Kabuli_NC_021165.1_52618262	Ca_kabuli_chr06	52618262	[A/C]	1067300838
Ca_Kabuli_NC_021165.1_52618264	Ca_kabuli_chr06	52618264	[A/G]	1067300839
Ca_Kabuli_NC_021165.1_52618275	Ca_kabuli_chr06	52618275	[T/G]	1067300840
Ca_Kabuli_NC_021165.1_52630806	Ca_kabuli_chr06	52630806	[A/C]	1067300841
Ca_Kabuli_NC_021165.1_53084230	Ca_kabuli_chr06	53084230	[G/C]	1067300842
Ca_Kabuli_NC_021165.1_53084235	Ca_kabuli_chr06	53084235	[C/T]	1067300843
Ca_Kabuli_NC_021165.1_53084258	Ca_kabuli_chr06	53084258	[A/G]	1067300844
Ca_Kabuli_NC_021165.1_53097365	Ca_kabuli_chr06	53097365	[T/G]	1067300845
Ca_Kabuli_NC_021165.1_53124142	Ca_kabuli_chr06	53124142	[G/T]	1067300846
Ca_Kabuli_NC_021165.1_53168793	Ca_kabuli_chr06	53168793	[A/T]	1067300847
Ca_Kabuli_NC_021165.1_53222941	Ca_kabuli_chr06	53222941	[T/G]	1067300848
Ca_Kabuli_NC_021165.1_53349021	Ca_kabuli_chr06	53349021	[T/A]	1067300849
Ca_Kabuli_NC_021165.1_53349026	Ca_kabuli_chr06	53349026	[T/G]	1067300850
Ca_Kabuli_NC_021165.1_53349075	Ca_kabuli_chr06	53349075	[A/G]	1067300851
Ca_Kabuli_NC_021165.1_53350648	Ca_kabuli_chr06	53350648	[A/C]	1067300852
Ca_Kabuli_NC_021165.1_53350651	Ca_kabuli_chr06	53350651	[A/T]	1067300853
Ca_Kabuli_NC_021165.1_53350681	Ca_kabuli_chr06	53350681	[T/G]	1067300854
Ca_Kabuli_NC_021165.1_53382954	Ca_kabuli_chr06	53382954	[T/G]	1067300855
Ca_Kabuli_NC_021165.1_53390086	Ca_kabuli_chr06	53390086	[A/C]	1067300856
Ca_Kabuli_NC_021165.1_53499267	Ca_kabuli_chr06	53499267	[A/G]	1067300857
Ca_Kabuli_NC_021165.1_53499295	Ca_kabuli_chr06	53499295	[A/G]	1067300858
Ca_Kabuli_NC_021165.1_53558419	Ca_kabuli_chr06	53558419	[T/C]	1067300859
Ca_Kabuli_NC_021165.1_53558950	Ca_kabuli_chr06	53558950	[T/C]	1067300860
Ca_Kabuli_NC_021165.1_53558972	Ca_kabuli_chr06	53558972	[T/C]	1067300861
Ca_Kabuli_NC_021165.1_53558982	Ca_kabuli_chr06	53558982	[A/G]	1067300862
Ca_Kabuli_NC_021165.1_53566431	Ca_kabuli_chr06	53566431	[T/G]	1067300863
Ca_Kabuli_NC_021165.1_53625215	Ca_kabuli_chr06	53625215	[A/G]	1067300864
Ca_Kabuli_NC_021165.1_53625232	Ca_kabuli_chr06	53625232	[A/G]	1067300865
Ca_Kabuli_NC_021165.1_53654093	Ca_kabuli_chr06	53654093	[A/G]	1067300866
Ca_Kabuli_NC_021165.1_53654134	Ca_kabuli_chr06	53654134	[A/G]	1067300867
Ca_Kabuli_NC_021165.1_53654153	Ca_kabuli_chr06	53654153	[A/G]	1067300868
Ca_Kabuli_NC_021165.1_53654155	Ca_kabuli_chr06	53654155	[A/G]	1067300869
Ca_Kabuli_NC_021165.1_53683766	Ca_kabuli_chr06	53683766	[C/T]	1067300870

SNP IDs	Chromosomes	Physical positions (bp)	SNPs	NCBI SNPdb accession IDs (NCBI_ss#)
Ca_Kabuli_NC_021165.1_53783296	Ca_kabuli_chr06	53783296	[T/G]	1067300871
Ca_Kabuli_NC_021165.1_53783304	Ca_kabuli_chr06	53783304	[A/T]	1067300872
Ca_Kabuli_NC_021165.1_54208252	Ca_kabuli_chr06	54208252	[A/G]	1067300873
Ca_Kabuli_NC_021165.1_54237701	Ca_kabuli_chr06	54237701	[T/C]	1067300874
Ca_Kabuli_NC_021165.1_54237761	Ca_kabuli_chr06	54237761	[A/C]	1067300875
Ca_Kabuli_NC_021165.1_54237764	Ca_kabuli_chr06	54237764	[A/C]	1067300876
Ca_Kabuli_NC_021165.1_54237824	Ca_kabuli_chr06	54237824	[T/C]	1067300877
Ca_Kabuli_NC_021165.1_54237837	Ca_kabuli_chr06	54237837	[A/T]	1067300878
Ca_Kabuli_NC_021165.1_54250124	Ca_kabuli_chr06	54250124	[A/C]	1067300879
Ca_Kabuli_NC_021165.1_54333329	Ca_kabuli_chr06	54333329	[A/G]	1067300880
Ca_Kabuli_NC_021165.1_54335013	Ca_kabuli_chr06	54335013	[A/T]	1067300881
Ca_Kabuli_NC_021165.1_54335725	Ca_kabuli_chr06	54335725	[A/G]	1067300882
Ca_Kabuli_NC_021165.1_54335736	Ca_kabuli_chr06	54335736	[A/G]	1067300883
Ca_Kabuli_NC_021165.1_54335769	Ca_kabuli_chr06	54335769	[A/G]	1067300884
Ca_Kabuli_NC_021165.1_54337370	Ca_kabuli_chr06	54337370	[T/C]	1067300885
Ca_Kabuli_NC_021165.1_54337390	Ca_kabuli_chr06	54337390	[T/G]	1067300886
Ca_Kabuli_NC_021165.1_54337402	Ca_kabuli_chr06	54337402	[T/G]	1067300887
Ca_Kabuli_NC_021165.1_54337415	Ca_kabuli_chr06	54337415	[T/C]	1067300888
Ca_Kabuli_NC_021165.1_54337422	Ca_kabuli_chr06	54337422	[A/G]	1067300889
Ca_Kabuli_NC_021165.1_54358852	Ca_kabuli_chr06	54358852	[G/A]	1067300890
Ca_Kabuli_NC_021165.1_54997486	Ca_kabuli_chr06	54997486	[T/A]	1067300891
Ca_Kabuli_NC_021165.1_55024140	Ca_kabuli_chr06	55024140	[G/A]	1067300892
Ca_Kabuli_NC_021165.1_55024146	Ca_kabuli_chr06	55024146	[C/T]	1067300893
Ca_Kabuli_NC_021165.1_55119038	Ca_kabuli_chr06	55119038	[T/G]	1067300894
Ca_Kabuli_NC_021165.1_55119053	Ca_kabuli_chr06	55119053	[T/C]	1067300895
Ca_Kabuli_NC_021165.1_55119075	Ca_kabuli_chr06	55119075	[A/G]	1067300896
Ca_Kabuli_NC_021165.1_55332477	Ca_kabuli_chr06	55332477	[A/C]	1067300897
Ca_Kabuli_NC_021165.1_55575106	Ca_kabuli_chr06	55575106	[A/G]	1067300898
Ca_Kabuli_NC_021165.1_55575116	Ca_kabuli_chr06	55575116	[T/C]	1067300899
Ca_Kabuli_NC_021165.1_55618607	Ca_kabuli_chr06	55618607	[T/G]	1067300900
Ca_Kabuli_NC_021165.1_55633279	Ca_kabuli_chr06	55633279	[T/C]	1067300901
Ca_Kabuli_NC_021165.1_55711680	Ca_kabuli_chr06	55711680	[T/G]	1067300902
Ca_Kabuli_NC_021165.1_55711785	Ca_kabuli_chr06	55711785	[T/G]	1067300903
Ca_Kabuli_NC_021165.1_56025841	Ca_kabuli_chr06	56025841	[T/C]	1067300904

SNP IDs	Chromosomes	Physical positions (bp)	SNPs	NCBI SNPdb accession IDs (NCBI_ss#)
Ca_Kabuli_NC_021165.1_56025859	Ca_kabuli_chr06	56025859	[T/G]	1067300905
Ca_Kabuli_NC_021165.1_56303217	Ca_kabuli_chr06	56303217	[T/C]	1067300906
Ca_Kabuli_NC_021165.1_56553864	Ca_kabuli_chr06	56553864	[T/C]	1067300907
Ca_Kabuli_NC_021165.1_56553903	Ca_kabuli_chr06	56553903	[A/G]	1067300908
Ca_Kabuli_NC_021165.1_56593129	Ca_kabuli_chr06	56593129	[A/C]	1067300909
Ca_Kabuli_NC_021165.1_56706537	Ca_kabuli_chr06	56706537	[T/A]	1067300910
Ca_Kabuli_NC_021165.1_56706550	Ca_kabuli_chr06	56706550	[T/C]	1067300911
Ca_Kabuli_NC_021165.1_56765545	Ca_kabuli_chr06	56765545	[A/C]	1067300912
Ca_Kabuli_NC_021165.1_56793039	Ca_kabuli_chr06	56793039	[T/A]	1067300913
Ca_Kabuli_NC_021165.1_56793044	Ca_kabuli_chr06	56793044	[T/A]	1067300914
Ca_Kabuli_NC_021165.1_56793083	Ca_kabuli_chr06	56793083	[A/G]	1067300915
Ca_Kabuli_NC_021165.1_56839995	Ca_kabuli_chr06	56839995	[T/G]	1067300916
Ca_Kabuli_NC_021165.1_56854753	Ca_kabuli_chr06	56854753	[C/T]	1067300917
Ca_Kabuli_NC_021165.1_56854906	Ca_kabuli_chr06	56854906	[T/A]	1067300918
Ca_Kabuli_NC_021165.1_56854914	Ca_kabuli_chr06	56854914	[A/C]	1067300919
Ca_Kabuli_NC_021165.1_56860665	Ca_kabuli_chr06	56860665	[C/A]	1067300920
Ca_Kabuli_NC_021165.1_56860666	Ca_kabuli_chr06	56860666	[C/G]	1067300921
Ca_Kabuli_NC_021165.1_56860667	Ca_kabuli_chr06	56860667	[C/A]	1067300922
Ca_Kabuli_NC_021165.1_56860696	Ca_kabuli_chr06	56860696	[A/C]	1067300923
Ca_Kabuli_NC_021165.1_56860703	Ca_kabuli_chr06	56860703	[A/G]	1067300924
Ca_Kabuli_NC_021165.1_57044799	Ca_kabuli_chr06	57044799	[T/C]	1067300925
Ca_Kabuli_NC_021165.1_57173684	Ca_kabuli_chr06	57173684	[A/G]	1067300926
Ca_Kabuli_NC_021165.1_57173688	Ca_kabuli_chr06	57173688	[A/C]	1067300927
Ca_Kabuli_NC_021165.1_57173695	Ca_kabuli_chr06	57173695	[A/G]	1067300928
Ca_Kabuli_NC_021165.1_57344969	Ca_kabuli_chr06	57344969	[T/G]	1067300929
Ca_Kabuli_NC_021165.1_57382778	Ca_kabuli_chr06	57382778	[G/T]	1067300930
Ca_Kabuli_NC_021165.1_57425496	Ca_kabuli_chr06	57425496	[C/T]	1067300931
Ca_Kabuli_NC_021165.1_57434831	Ca_kabuli_chr06	57434831	[A/C]	1067300932
Ca_Kabuli_NC_021165.1_57436104	Ca_kabuli_chr06	57436104	[T/C]	1067300933
Ca_Kabuli_NC_021165.1_57437932	Ca_kabuli_chr06	57437932	[T/C]	1067300934
Ca_Kabuli_NC_021165.1_57437940	Ca_kabuli_chr06	57437940	[A/C]	1067300935
Ca_Kabuli_NC_021165.1_57437944	Ca_kabuli_chr06	57437944	[T/A]	1067300936
Ca_Kabuli_NC_021165.1_57533088	Ca_kabuli_chr06	57533088	[T/C]	1067300937
Ca_Kabuli_NC_021165.1_57533105	Ca_kabuli_chr06	57533105	[G/T]	1067300938

SNP IDs	Chromosomes	Physical positions (bp)	SNPs	NCBI SNPdb accession IDs (NCBI_ss#)
Ca_Kabuli_NC_021165.1_57533111	Ca_kabuli_chr06	57533111	[T/C]	1067300939
Ca_Kabuli_NC_021165.1_57533116	Ca_kabuli_chr06	57533116	[T/G]	1067300940
Ca_Kabuli_NC_021165.1_57533121	Ca_kabuli_chr06	57533121	[C/T]	1067300941
Ca_Kabuli_NC_021165.1_57579767	Ca_kabuli_chr06	57579767	[A/C]	1067300942
Ca_Kabuli_NC_021165.1_57579807	Ca_kabuli_chr06	57579807	[A/G]	1067300943
Ca_Kabuli_NC_021165.1_57625134	Ca_kabuli_chr06	57625134	[G/A]	1067300944
Ca_Kabuli_NC_021165.1_57625320	Ca_kabuli_chr06	57625320	[G/A]	1067300945
Ca_Kabuli_NC_021165.1_57631772	Ca_kabuli_chr06	57631772	[T/C]	1067300946
Ca_Kabuli_NC_021165.1_57631963	Ca_kabuli_chr06	57631963	[T/G]	1067300947
Ca_Kabuli_NC_021165.1_57632374	Ca_kabuli_chr06	57632374	[C/T]	1067300948
Ca_Kabuli_NC_021165.1_57632385	Ca_kabuli_chr06	57632385	[A/C]	1067300949
Ca_Kabuli_NC_021165.1_57634425	Ca_kabuli_chr06	57634425	[A/C]	1067300950
Ca_Kabuli_NC_021165.1_57640124	Ca_kabuli_chr06	57640124	[A/G]	1067300951
Ca_Kabuli_NC_021165.1_57640186	Ca_kabuli_chr06	57640186	[G/T]	1067300952
Ca_Kabuli_NC_021165.1_57640197	Ca_kabuli_chr06	57640197	[A/G]	1067300953
Ca_Kabuli_NC_021165.1_57640220	Ca_kabuli_chr06	57640220	[A/C]	1067300954
Ca_Kabuli_NC_021165.1_57644429	Ca_kabuli_chr06	57644429	[A/G]	1067300955
Ca_Kabuli_NC_021165.1_57703958	Ca_kabuli_chr06	57703958	[T/C]	1067300956
Ca_Kabuli_NC_021165.1_57703979	Ca_kabuli_chr06	57703979	[T/C]	1067300957
Ca_Kabuli_NC_021165.1_57703989	Ca_kabuli_chr06	57703989	[G/C]	1067300958
Ca_Kabuli_NC_021165.1_57703998	Ca_kabuli_chr06	57703998	[G/A]	1067300959
Ca_Kabuli_NC_021165.1_57704001	Ca_kabuli_chr06	57704001	[C/A]	1067300960
Ca_Kabuli_NC_021165.1_57704013	Ca_kabuli_chr06	57704013	[A/C]	1067300961
Ca_Kabuli_NC_021165.1_57704018	Ca_kabuli_chr06	57704018	[T/C]	1067300962
Ca_Kabuli_NC_021165.1_57704057	Ca_kabuli_chr06	57704057	[C/T]	1067300963
Ca_Kabuli_NC_021165.1_57704216	Ca_kabuli_chr06	57704216	[T/G]	1067300964
Ca_Kabuli_NC_021165.1_57704220	Ca_kabuli_chr06	57704220	[A/T]	1067300965
Ca_Kabuli_NC_021165.1_57704802	Ca_kabuli_chr06	57704802	[T/C]	1067300966
Ca_Kabuli_NC_021165.1_57705111	Ca_kabuli_chr06	57705111	[C/T]	1067300967
Ca_Kabuli_NC_021165.1_57705113	Ca_kabuli_chr06	57705113	[A/G]	1067300968
Ca_Kabuli_NC_021165.1_57707716	Ca_kabuli_chr06	57707716	[C/T]	1067300969
Ca_Kabuli_NC_021165.1_57707724	Ca_kabuli_chr06	57707724	[A/C]	1067300970
Ca_Kabuli_NC_021165.1_57707727	Ca_kabuli_chr06	57707727	[T/G]	1067300971
Ca_Kabuli_NC_021165.1_57707734	Ca_kabuli_chr06	57707734	[T/C]	1067300972

SNP IDs	Chromosomes	Physical positions (bp)	SNPs	NCBI SNPdb accession IDs (NCBI_ss#)
Ca_Kabuli_NC_021165.1_57707737	Ca_kabuli_chr06	57707737	[C/T]	1067300973
Ca_Kabuli_NC_021165.1_57707846	Ca_kabuli_chr06	57707846	[G/A]	1067300974
Ca_Kabuli_NC_021165.1_57707848	Ca_kabuli_chr06	57707848	[A/G]	1067300975
Ca_Kabuli_NC_021165.1_57707902	Ca_kabuli_chr06	57707902	[T/G]	1067300976
Ca_Kabuli_NC_021165.1_57707912	Ca_kabuli_chr06	57707912	[G/C]	1067300977
Ca_Kabuli_NC_021165.1_57707919	Ca_kabuli_chr06	57707919	[T/A]	1067300978
Ca_Kabuli_NC_021165.1_57707954	Ca_kabuli_chr06	57707954	[A/T]	1067300979
Ca_Kabuli_NC_021165.1_57707959	Ca_kabuli_chr06	57707959	[A/C]	1067300980
Ca_Kabuli_NC_021165.1_57708101	Ca_kabuli_chr06	57708101	[T/G]	1067300981
Ca_Kabuli_NC_021165.1_57708130	Ca_kabuli_chr06	57708130	[T/C]	1067300982
Ca_Kabuli_NC_021165.1_57710133	Ca_kabuli_chr06	57710133	[C/G]	1067300983
Ca_Kabuli_NC_021165.1_57720161	Ca_kabuli_chr06	57720161	[A/T]	1067300984
Ca_Kabuli_NC_021165.1_57720446	Ca_kabuli_chr06	57720446	[T/C]	1067300985
Ca_Kabuli_NC_021165.1_57720708	Ca_kabuli_chr06	57720708	[T/G]	1067300986
Ca_Kabuli_NC_021165.1_57720820	Ca_kabuli_chr06	57720820	[A/G]	1067300987
Ca_Kabuli_NC_021165.1_57720831	Ca_kabuli_chr06	57720831	[T/C]	1067300988
Ca_Kabuli_NC_021165.1_57720909	Ca_kabuli_chr06	57720909	[C/T]	1067300989
Ca_Kabuli_NC_021165.1_57747859	Ca_kabuli_chr06	57747859	[A/G]	1067300990
Ca_Kabuli_NC_021165.1_57747866	Ca_kabuli_chr06	57747866	[T/G]	1067300991
Ca_Kabuli_NC_021165.1_57748565	Ca_kabuli_chr06	57748565	[A/G]	1067300992
Ca_Kabuli_NC_021165.1_57750418	Ca_kabuli_chr06	57750418	[A/C]	1067300993
Ca_Kabuli_NC_021165.1_57750900	Ca_kabuli_chr06	57750900	[G/A]	1067300994
Ca_Kabuli_NC_021165.1_57753076	Ca_kabuli_chr06	57753076	[T/C]	1067300995
Ca_Kabuli_NC_021165.1_57753193	Ca_kabuli_chr06	57753193	[T/C]	1067300996
Ca_Kabuli_NC_021165.1_57756355	Ca_kabuli_chr06	57756355	[T/G]	1067300997
Ca_Kabuli_NC_021165.1_57756699	Ca_kabuli_chr06	57756699	[G/A]	1067300998
Ca_Kabuli_NC_021165.1_57759548	Ca_kabuli_chr06	57759548	[C/T]	1067300999
Ca_Kabuli_NC_021165.1_57760104	Ca_kabuli_chr06	57760104	[T/C]	1067301000
Ca_Kabuli_NC_021165.1_57761758	Ca_kabuli_chr06	57761758	[G/A]	1067301001
Ca_Kabuli_NC_021165.1_57765391	Ca_kabuli_chr06	57765391	[G/T]	1067301002
Ca_Kabuli_NC_021165.1_57765418	Ca_kabuli_chr06	57765418	[G/C]	1067301003
Ca_Kabuli_NC_021165.1_57765423	Ca_kabuli_chr06	57765423	[T/A]	1067301004
Ca_Kabuli_NC_021165.1_57769819	Ca_kabuli_chr06	57769819	[G/A]	1067301005
Ca_Kabuli_NC_021165.1_57831061	Ca_kabuli_chr06	57831061	[T/G]	1067301006

SNP IDs	Chromosomes	Physical positions (bp)	SNPs	NCBI SNPdb accession IDs (NCBI_ss#)
Ca_Kabuli_NC_021165.1_57835093	Ca_kabuli_chr06	57835093	[T/A]	1067301007
Ca_Kabuli_NC_021165.1_57835120	Ca_kabuli_chr06	57835120	[G/A]	1067301008
Ca_Kabuli_NC_021165.1_57835335	Ca_kabuli_chr06	57835335	[A/G]	1067301009
Ca_Kabuli_NC_021165.1_57835899	Ca_kabuli_chr06	57835899	[C/T]	1067301010
Ca_Kabuli_NC_021165.1_57836237	Ca_kabuli_chr06	57836237	[A/G]	1067301011
Ca_Kabuli_NC_021165.1_57836296	Ca_kabuli_chr06	57836296	[A/T]	1067301012
Ca_Kabuli_NC_021165.1_57853458	Ca_kabuli_chr06	57853458	[C/T]	1067301013
Ca_Kabuli_NC_021165.1_57854068	Ca_kabuli_chr06	57854068	[G/C]	1067301014
Ca_Kabuli_NC_021165.1_57854946	Ca_kabuli_chr06	57854946	[G/A]	1067301015
Ca_Kabuli_NC_021165.1_57856890	Ca_kabuli_chr06	57856890	[C/T]	1067301016
Ca_Kabuli_NC_021165.1_57870893	Ca_kabuli_chr06	57870893	[C/A]	1067301017
Ca_Kabuli_NC_021165.1_57882773	Ca_kabuli_chr06	57882773	[G/T]	1067301018
Ca_Kabuli_NC_021165.1_57891716	Ca_kabuli_chr06	57891716	[A/T]	1067301019
Ca_Kabuli_NC_021165.1_57894263	Ca_kabuli_chr06	57894263	[T/A]	1067301020
Ca_Kabuli_NC_021165.1_57894264	Ca_kabuli_chr06	57894264	[G/T]	1067301021
Ca_Kabuli_NC_021165.1_57894377	Ca_kabuli_chr06	57894377	[A/G]	1067301022
Ca_Kabuli_NC_021165.1_57894585	Ca_kabuli_chr06	57894585	[T/G]	1067301023
Ca_Kabuli_NC_021165.1_57894590	Ca_kabuli_chr06	57894590	[G/C]	1067301024
Ca_Kabuli_NC_021165.1_57898786	Ca_kabuli_chr06	57898786	[A/C]	1067301025
Ca_Kabuli_NC_021165.1_57899764	Ca_kabuli_chr06	57899764	[T/A]	1067301026
Ca_Kabuli_NC_021165.1_57900328	Ca_kabuli_chr06	57900328	[T/G]	1067301027
Ca_Kabuli_NC_021165.1_57931382	Ca_kabuli_chr06	57931382	[A/T]	1067301028
Ca_Kabuli_NC_021165.1_57931563	Ca_kabuli_chr06	57931563	[A/C]	1067301029
Ca_Kabuli_NC_021165.1_57937638	Ca_kabuli_chr06	57937638	[T/A]	1067301030
Ca_Kabuli_NC_021165.1_57937642	Ca_kabuli_chr06	57937642	[A/G]	1067301031
Ca_Kabuli_NC_021165.1_57937657	Ca_kabuli_chr06	57937657	[A/G]	1067301032
Ca_Kabuli_NC_021165.1_57942502	Ca_kabuli_chr06	57942502	[T/A]	1067301033
Ca_Kabuli_NC_021165.1_57948587	Ca_kabuli_chr06	57948587	[G/A]	1067301034
Ca_Kabuli_NC_021165.1_57960875	Ca_kabuli_chr06	57960875	[A/G]	1067301035
Ca_Kabuli_NC_021165.1_57960878	Ca_kabuli_chr06	57960878	[A/G]	1067301036
Ca_Kabuli_NC_021165.1_58028663	Ca_kabuli_chr06	58028663	[A/T]	1067301037
Ca_Kabuli_NC_021165.1_58028664	Ca_kabuli_chr06	58028664	[G/C]	1067301038
Ca_Kabuli_NC_021165.1_58028665	Ca_kabuli_chr06	58028665	[A/C]	1067301039
Ca_Kabuli_NC_021165.1_58053878	Ca_kabuli_chr06	58053878	[T/A]	1067301040

SNP IDs	Chromosomes	Physical positions (bp)	SNPs	NCBI SNPdb accession IDs (NCBI_ss#)
Ca_Kabuli_NC_021165.1_58110605	Ca_kabuli_chr06	58110605	[C/T]	1067301041
Ca_Kabuli_NC_021165.1_58161736	Ca_kabuli_chr06	58161736	[A/C]	1067301042
Ca_Kabuli_NC_021165.1_58161748	Ca_kabuli_chr06	58161748	[A/C]	1067301043
Ca_Kabuli_NC_021165.1_58165739	Ca_kabuli_chr06	58165739	[T/A]	1067301044
Ca_Kabuli_NC_021165.1_58175060	Ca_kabuli_chr06	58175060	[G/A]	1067301045
Ca_Kabuli_NC_021165.1_58187980	Ca_kabuli_chr06	58187980	[A/G]	1067301046
Ca_Kabuli_NC_021165.1_58190722	Ca_kabuli_chr06	58190722	[A/G]	1067301047
Ca_Kabuli_NC_021165.1_58190723	Ca_kabuli_chr06	58190723	[G/A]	1067301048
Ca_Kabuli_NC_021165.1_58190724	Ca_kabuli_chr06	58190724	[A/G]	1067301049
Ca_Kabuli_NC_021165.1_58191028	Ca_kabuli_chr06	58191028	[A/G]	1067301050
Ca_Kabuli_NC_021165.1_58191054	Ca_kabuli_chr06	58191054	[T/G]	1067301051
Ca_Kabuli_NC_021165.1_58191065	Ca_kabuli_chr06	58191065	[A/C]	1067301052
Ca_Kabuli_NC_021165.1_58191073	Ca_kabuli_chr06	58191073	[A/C]	1067301053
Ca_Kabuli_NC_021165.1_58201715	Ca_kabuli_chr06	58201715	[T/G]	1067301054
Ca_Kabuli_NC_021165.1_58223959	Ca_kabuli_chr06	58223959	[A/G]	1067301055
Ca_Kabuli_NC_021165.1_58224049	Ca_kabuli_chr06	58224049	[A/C]	1067301056
Ca_Kabuli_NC_021165.1_58224069	Ca_kabuli_chr06	58224069	[A/C]	1067301057
Ca_Kabuli_NC_021165.1_58233856	Ca_kabuli_chr06	58233856	[T/C]	1067301058
Ca_Kabuli_NC_021165.1_58233858	Ca_kabuli_chr06	58233858	[T/C]	1067301059
Ca_Kabuli_NC_021165.1_58233916	Ca_kabuli_chr06	58233916	[T/A]	1067301060
Ca_Kabuli_NC_021165.1_58244019	Ca_kabuli_chr06	58244019	[G/A]	1067301061
Ca_Kabuli_NC_021165.1_58244128	Ca_kabuli_chr06	58244128	[T/C]	1067301062
Ca_Kabuli_NC_021165.1_58244129	Ca_kabuli_chr06	58244129	[G/A]	1067301063
Ca_Kabuli_NC_021165.1_58326313	Ca_kabuli_chr06	58326313	[A/T]	1067301064
Ca_Kabuli_NC_021165.1_58326368	Ca_kabuli_chr06	58326368	[A/G]	1067301065
Ca_Kabuli_NC_021165.1_58401640	Ca_kabuli_chr06	58401640	[G/A]	1067301066
Ca_Kabuli_NC_021165.1_58434180	Ca_kabuli_chr06	58434180	[T/A]	1067301067
Ca_Kabuli_NC_021165.1_58434202	Ca_kabuli_chr06	58434202	[T/C]	1067301068
Ca_Kabuli_NC_021165.1_58434208	Ca_kabuli_chr06	58434208	[T/C]	1067301069
Ca_Kabuli_NC_021165.1_58452890	Ca_kabuli_chr06	58452890	[T/C]	1067301070
Ca_Kabuli_NC_021165.1_58569649	Ca_kabuli_chr06	58569649	[A/G]	1067301071
Ca_Kabuli_NC_021165.1_58571594	Ca_kabuli_chr06	58571594	[T/C]	1067301072
Ca_Kabuli_NC_021165.1_58571615	Ca_kabuli_chr06	58571615	[T/G]	1067301073
Ca_Kabuli_NC_021165.1_58571658	Ca_kabuli_chr06	58571658	[A/T]	1067301074

SNP IDs	Chromosomes	Physical positions (bp)	SNPs	NCBI SNPdb accession IDs (NCBI_ss#)
Ca_Kabuli_NC_021165.1_58571680	Ca_kabuli_chr06	58571680	[A/T]	1067301075
Ca_Kabuli_NC_021165.1_58618631	Ca_kabuli_chr06	58618631	[A/C]	1067301076
Ca_Kabuli_NC_021165.1_58650318	Ca_kabuli_chr06	58650318	[A/C]	1067301077
Ca_Kabuli_NC_021165.1_58650412	Ca_kabuli_chr06	58650412	[T/C]	1067301078
Ca_Kabuli_NC_021165.1_58650426	Ca_kabuli_chr06	58650426	[A/T]	1067301079
Ca_Kabuli_NC_021165.1_58652851	Ca_kabuli_chr06	58652851	[T/C]	1067301080
Ca_Kabuli_NC_021165.1_58676515	Ca_kabuli_chr06	58676515	[T/C]	1067301081
Ca_Kabuli_NC_021165.1_58676520	Ca_kabuli_chr06	58676520	[T/C]	1067301082
Ca_Kabuli_NC_021165.1_58682397	Ca_kabuli_chr06	58682397	[G/A]	1067301083
Ca_Kabuli_NC_021165.1_58682398	Ca_kabuli_chr06	58682398	[T/A]	1067301084
Ca_Kabuli_NC_021165.1_58746193	Ca_kabuli_chr06	58746193	[T/G]	1067301085
Ca_Kabuli_NC_021165.1_58746209	Ca_kabuli_chr06	58746209	[A/C]	1067301086
Ca_Kabuli_NC_021165.1_58753226	Ca_kabuli_chr06	58753226	[T/A]	1067301087
Ca_Kabuli_NC_021165.1_58820562	Ca_kabuli_chr06	58820562	[T/G]	1067301088
Ca_Kabuli_NC_021165.1_58832691	Ca_kabuli_chr06	58832691	[T/G]	1067301089
Ca_Kabuli_NC_021165.1_58832732	Ca_kabuli_chr06	58832732	[A/T]	1067301090
Ca_Kabuli_NC_021165.1_58864558	Ca_kabuli_chr06	58864558	[A/G]	1067301091
Ca_Kabuli_NC_021165.1_58864584	Ca_kabuli_chr06	58864584	[T/A]	1067301092
Ca_Kabuli_NC_021165.1_58879219	Ca_kabuli_chr06	58879219	[T/G]	1067301093
Ca_Kabuli_NC_021165.1_58879616	Ca_kabuli_chr06	58879616	[T/A]	1067301094
Ca_Kabuli_NC_021165.1_58897252	Ca_kabuli_chr06	58897252	[C/T]	1067301095
Ca_Kabuli_NC_021165.1_58908024	Ca_kabuli_chr06	58908024	[G/A]	1067301096
Ca_Kabuli_NC_021165.1_58911575	Ca_kabuli_chr06	58911575	[T/G]	1067301097
Ca_Kabuli_NC_021165.1_58911693	Ca_kabuli_chr06	58911693	[A/C]	1067301098
Ca_Kabuli_NC_021165.1_58951144	Ca_kabuli_chr06	58951144	[T/C]	1067301099
Ca_Kabuli_NC_021165.1_58984062	Ca_kabuli_chr06	58984062	[A/T]	1067301100
Ca_Kabuli_NC_021165.1_58984063	Ca_kabuli_chr06	58984063	[T/C]	1067301101
Ca_Kabuli_NC_021165.1_58984064	Ca_kabuli_chr06	58984064	[G/T]	1067301102
Ca_Kabuli_NC_021165.1_58993813	Ca_kabuli_chr06	58993813	[T/C]	1067301103
Ca_Kabuli_NC_021165.1_59000599	Ca_kabuli_chr06	59000599	[A/C]	1067301104
Ca_Kabuli_NC_021165.1_59010430	Ca_kabuli_chr06	59010430	[T/A]	1067301105
Ca_Kabuli_NC_021165.1_59010513	Ca_kabuli_chr06	59010513	[C/T]	1067301106
Ca_Kabuli_NC_021165.1_59010536	Ca_kabuli_chr06	59010536	[T/C]	1067301107
Ca_Kabuli_NC_021165.1_59064814	Ca_kabuli_chr06	59064814	[A/G]	1067301108

SNP IDs	Chromosomes	Physical positions (bp)	SNPs	NCBI SNPdb accession IDs (NCBI_ss#)
Ca_Kabuli_NC_021165.1_59081169	Ca_kabuli_chr06	59081169	[T/C]	1067301109
Ca_Kabuli_NC_021165.1_59095529	Ca_kabuli_chr06	59095529	[T/G]	1067301110
Ca_Kabuli_NC_021165.1_59102165	Ca_kabuli_chr06	59102165	[T/C]	1067301111
Ca_Kabuli_NC_021165.1_59135935	Ca_kabuli_chr06	59135935	[T/G]	1067301112
Ca_Kabuli_NC_021165.1_59219116	Ca_kabuli_chr06	59219116	[T/G]	1067301113
Ca_Kabuli_NC_021165.1_59304548	Ca_kabuli_chr06	59304548	[T/G]	1067301114
Ca_Kabuli_NC_021165.1_59367115	Ca_kabuli_chr06	59367115	[C/A]	1067301115
Ca_Kabuli_NC_021165.1_59367116	Ca_kabuli_chr06	59367116	[T/G]	1067301116
Ca_Kabuli_NC_021165.1_59367117	Ca_kabuli_chr06	59367117	[G/A]	1067301117
Ca_Kabuli_NC_021165.1_59367118	Ca_kabuli_chr06	59367118	[G/T]	1067301118
Ca_Kabuli_NC_021165.1_59417909	Ca_kabuli_chr06	59417909	[A/C]	1067301119
Ca_Kabuli_NC_021165.1_59417915	Ca_kabuli_chr06	59417915	[A/G]	1067301120
Ca_Kabuli_NC_021166.1_16589	Ca_kabuli_chr07	16589	[T/C]	1067301121
Ca_Kabuli_NC_021166.1_16881	Ca_kabuli_chr07	16881	[A/C]	1067301122
Ca_Kabuli_NC_021166.1_31121	Ca_kabuli_chr07	31121	[T/C]	1067301123
Ca_Kabuli_NC_021166.1_31129	Ca_kabuli_chr07	31129	[A/G]	1067301124
Ca_Kabuli_NC_021166.1_31137	Ca_kabuli_chr07	31137	[A/G]	1067301125
Ca_Kabuli_NC_021166.1_31139	Ca_kabuli_chr07	31139	[A/G]	1067301126
Ca_Kabuli_NC_021166.1_32278	Ca_kabuli_chr07	32278	[A/C]	1067301127
Ca_Kabuli_NC_021166.1_39091	Ca_kabuli_chr07	39091	[A/C]	1067301128
Ca_Kabuli_NC_021166.1_42032	Ca_kabuli_chr07	42032	[A/C]	1067301129
Ca_Kabuli_NC_021166.1_122600	Ca_kabuli_chr07	122600	[T/G]	1067301130
Ca_Kabuli_NC_021166.1_128956	Ca_kabuli_chr07	128956	[A/T]	1067301131
Ca_Kabuli_NC_021166.1_208851	Ca_kabuli_chr07	208851	[A/C]	1067301132
Ca_Kabuli_NC_021166.1_218330	Ca_kabuli_chr07	218330	[A/G]	1067301133
Ca_Kabuli_NC_021166.1_234618	Ca_kabuli_chr07	234618	[T/C]	1067301134
Ca_Kabuli_NC_021166.1_234624	Ca_kabuli_chr07	234624	[A/G]	1067301135
Ca_Kabuli_NC_021166.1_234648	Ca_kabuli_chr07	234648	[A/G]	1067301136
Ca_Kabuli_NC_021166.1_319218	Ca_kabuli_chr07	319218	[A/C]	1067301137
Ca_Kabuli_NC_021166.1_343628	Ca_kabuli_chr07	343628	[A/G]	1067301138
Ca_Kabuli_NC_021166.1_343919	Ca_kabuli_chr07	343919	[A/C]	1067301139
Ca_Kabuli_NC_021166.1_610723	Ca_kabuli_chr07	610723	[G/T]	1067301140
Ca_Kabuli_NC_021166.1_610737	Ca_kabuli_chr07	610737	[C/A]	1067301141
Ca_Kabuli_NC_021166.1_610741	Ca_kabuli_chr07	610741	[G/A]	1067301142

SNP IDs	Chromosomes	Physical positions (bp)	SNPs	NCBI SNPdb accession IDs (NCBI_ss#)
Ca_Kabuli_NC_021166.1_670596	Ca_kabuli_chr07	670596	[A/G]	1067301143
Ca_Kabuli_NC_021166.1_670605	Ca_kabuli_chr07	670605	[T/C]	1067301144
Ca_Kabuli_NC_021166.1_674764	Ca_kabuli_chr07	674764	[T/C]	1067301145
Ca_Kabuli_NC_021166.1_676748	Ca_kabuli_chr07	676748	[A/C]	1067301146
Ca_Kabuli_NC_021166.1_676759	Ca_kabuli_chr07	676759	[C/T]	1067301147
Ca_Kabuli_NC_021166.1_729733	Ca_kabuli_chr07	729733	[A/G]	1067301148
Ca_Kabuli_NC_021166.1_767739	Ca_kabuli_chr07	767739	[A/C]	1067301149
Ca_Kabuli_NC_021166.1_767896	Ca_kabuli_chr07	767896	[A/C]	1067301150
Ca_Kabuli_NC_021166.1_780997	Ca_kabuli_chr07	780997	[A/C]	1067301151
Ca_Kabuli_NC_021166.1_780998	Ca_kabuli_chr07	780998	[G/T]	1067301152
Ca_Kabuli_NC_021166.1_781341	Ca_kabuli_chr07	781341	[T/G]	1067301153
Ca_Kabuli_NC_021166.1_801799	Ca_kabuli_chr07	801799	[G/T]	1067301154
Ca_Kabuli_NC_021166.1_801802	Ca_kabuli_chr07	801802	[A/C]	1067301155
Ca_Kabuli_NC_021166.1_802085	Ca_kabuli_chr07	802085	[T/G]	1067301156
Ca_Kabuli_NC_021166.1_815447	Ca_kabuli_chr07	815447	[T/G]	1067301157
Ca_Kabuli_NC_021166.1_815452	Ca_kabuli_chr07	815452	[T/G]	1067301158
Ca_Kabuli_NC_021166.1_815466	Ca_kabuli_chr07	815466	[T/G]	1067301159
Ca_Kabuli_NC_021166.1_815471	Ca_kabuli_chr07	815471	[T/G]	1067301160
Ca_Kabuli_NC_021166.1_815504	Ca_kabuli_chr07	815504	[G/T]	1067301161
Ca_Kabuli_NC_021166.1_815505	Ca_kabuli_chr07	815505	[A/G]	1067301162
Ca_Kabuli_NC_021166.1_815506	Ca_kabuli_chr07	815506	[G/T]	1067301163
Ca_Kabuli_NC_021166.1_827475	Ca_kabuli_chr07	827475	[T/C]	1067301164
Ca_Kabuli_NC_021166.1_827495	Ca_kabuli_chr07	827495	[T/C]	1067301165
Ca_Kabuli_NC_021166.1_827514	Ca_kabuli_chr07	827514	[T/C]	1067301166
Ca_Kabuli_NC_021166.1_842109	Ca_kabuli_chr07	842109	[T/A]	1067301167
Ca_Kabuli_NC_021166.1_843870	Ca_kabuli_chr07	843870	[T/G]	1067301168
Ca_Kabuli_NC_021166.1_843871	Ca_kabuli_chr07	843871	[T/A]	1067301169
Ca_Kabuli_NC_021166.1_843873	Ca_kabuli_chr07	843873	[T/C]	1067301170
Ca_Kabuli_NC_021166.1_843874	Ca_kabuli_chr07	843874	[G/T]	1067301171
Ca_Kabuli_NC_021166.1_845430	Ca_kabuli_chr07	845430	[T/C]	1067301172
Ca_Kabuli_NC_021166.1_845475	Ca_kabuli_chr07	845475	[T/G]	1067301173
Ca_Kabuli_NC_021166.1_845482	Ca_kabuli_chr07	845482	[A/G]	1067301174
Ca_Kabuli_NC_021166.1_845492	Ca_kabuli_chr07	845492	[A/G]	1067301175
Ca_Kabuli_NC_021166.1_857952	Ca_kabuli_chr07	857952	[A/C]	1067301176

SNP IDs	Chromosomes	Physical positions (bp)	SNPs	NCBI SNPdb accession IDs (NCBI_ss#)
Ca_Kabuli_NC_021166.1_888948	Ca_kabuli_chr07	888948	[A/C]	1067301177
Ca_Kabuli_NC_021166.1_888954	Ca_kabuli_chr07	888954	[A/C]	1067301178
Ca_Kabuli_NC_021166.1_888957	Ca_kabuli_chr07	888957	[A/C]	1067301179
Ca_Kabuli_NC_021166.1_888979	Ca_kabuli_chr07	888979	[A/G]	1067301180
Ca_Kabuli_NC_021166.1_888982	Ca_kabuli_chr07	888982	[A/C]	1067301181
Ca_Kabuli_NC_021166.1_934378	Ca_kabuli_chr07	934378	[T/G]	1067301182
Ca_Kabuli_NC_021166.1_934384	Ca_kabuli_chr07	934384	[T/C]	1067301183
Ca_Kabuli_NC_021166.1_934418	Ca_kabuli_chr07	934418	[A/C]	1067301184
Ca_Kabuli_NC_021166.1_944687	Ca_kabuli_chr07	944687	[T/G]	1067301185
Ca_Kabuli_NC_021166.1_944696	Ca_kabuli_chr07	944696	[T/C]	1067301186
Ca_Kabuli_NC_021166.1_944702	Ca_kabuli_chr07	944702	[A/T]	1067301187
Ca_Kabuli_NC_021166.1_945251	Ca_kabuli_chr07	945251	[T/A]	1067301188
Ca_Kabuli_NC_021166.1_975248	Ca_kabuli_chr07	975248	[T/G]	1067301189
Ca_Kabuli_NC_021166.1_975255	Ca_kabuli_chr07	975255	[T/G]	1067301190
Ca_Kabuli_NC_021166.1_975262	Ca_kabuli_chr07	975262	[A/G]	1067301191
Ca_Kabuli_NC_021166.1_979745	Ca_kabuli_chr07	979745	[A/G]	1067301192
Ca_Kabuli_NC_021166.1_979748	Ca_kabuli_chr07	979748	[A/C]	1067301193
Ca_Kabuli_NC_021166.1_1023086	Ca_kabuli_chr07	1023086	[A/T]	1067301194
Ca_Kabuli_NC_021166.1_1023092	Ca_kabuli_chr07	1023092	[A/C]	1067301195
Ca_Kabuli_NC_021166.1_1044375	Ca_kabuli_chr07	1044375	[T/C]	1067301196
Ca_Kabuli_NC_021166.1_1086941	Ca_kabuli_chr07	1086941	[T/G]	1067301197
Ca_Kabuli_NC_021166.1_1108985	Ca_kabuli_chr07	1108985	[T/G]	1067301198
Ca_Kabuli_NC_021166.1_1109051	Ca_kabuli_chr07	1109051	[A/G]	1067301199
Ca_Kabuli_NC_021166.1_1120977	Ca_kabuli_chr07	1120977	[A/C]	1067301200
Ca_Kabuli_NC_021166.1_1127710	Ca_kabuli_chr07	1127710	[T/G]	1067301201
Ca_Kabuli_NC_021166.1_1153230	Ca_kabuli_chr07	1153230	[A/C]	1067301202
Ca_Kabuli_NC_021166.1_1153232	Ca_kabuli_chr07	1153232	[C/T]	1067301203
Ca_Kabuli_NC_021166.1_1178312	Ca_kabuli_chr07	1178312	[A/C]	1067301204
Ca_Kabuli_NC_021166.1_1178335	Ca_kabuli_chr07	1178335	[A/C]	1067301205
Ca_Kabuli_NC_021166.1_1210467	Ca_kabuli_chr07	1210467	[A/C]	1067301206
Ca_Kabuli_NC_021166.1_1261345	Ca_kabuli_chr07	1261345	[A/T]	1067301207
Ca_Kabuli_NC_021166.1_1261355	Ca_kabuli_chr07	1261355	[T/A]	1067301208
Ca_Kabuli_NC_021166.1_1261372	Ca_kabuli_chr07	1261372	[A/T]	1067301209
Ca_Kabuli_NC_021166.1_1261380	Ca_kabuli_chr07	1261380	[A/G]	1067301210

SNP IDs	Chromosomes	Physical positions (bp)	SNPs	NCBI SNPdb accession IDs (NCBI_ss#)
Ca_Kabuli_NC_021166.1_1261405	Ca_kabuli_chr07	1261405	[T/C]	1067301211
Ca_Kabuli_NC_021166.1_1278577	Ca_kabuli_chr07	1278577	[A/G]	1067301212
Ca_Kabuli_NC_021166.1_1278590	Ca_kabuli_chr07	1278590	[A/G]	1067301213
Ca_Kabuli_NC_021166.1_1278598	Ca_kabuli_chr07	1278598	[T/C]	1067301214
Ca_Kabuli_NC_021166.1_1293601	Ca_kabuli_chr07	1293601	[C/G]	1067301215
Ca_Kabuli_NC_021166.1_1317991	Ca_kabuli_chr07	1317991	[T/A]	1067301216
Ca_Kabuli_NC_021166.1_1350841	Ca_kabuli_chr07	1350841	[T/A]	1067301217
Ca_Kabuli_NC_021166.1_1350851	Ca_kabuli_chr07	1350851	[A/T]	1067301218
Ca_Kabuli_NC_021166.1_1350879	Ca_kabuli_chr07	1350879	[T/G]	1067301219
Ca_Kabuli_NC_021166.1_1392884	Ca_kabuli_chr07	1392884	[T/G]	1067301220
Ca_Kabuli_NC_021166.1_1392901	Ca_kabuli_chr07	1392901	[A/C]	1067301221
Ca_Kabuli_NC_021166.1_1436365	Ca_kabuli_chr07	1436365	[T/C]	1067301222
Ca_Kabuli_NC_021166.1_1461463	Ca_kabuli_chr07	1461463	[T/G]	1067301223
Ca_Kabuli_NC_021166.1_1461481	Ca_kabuli_chr07	1461481	[T/C]	1067301224
Ca_Kabuli_NC_021166.1_1461485	Ca_kabuli_chr07	1461485	[T/A]	1067301225
Ca_Kabuli_NC_021166.1_1473431	Ca_kabuli_chr07	1473431	[A/G]	1067301226
Ca_Kabuli_NC_021166.1_1505488	Ca_kabuli_chr07	1505488	[T/G]	1067301227
Ca_Kabuli_NC_021166.1_1510868	Ca_kabuli_chr07	1510868	[A/G]	1067301228
Ca_Kabuli_NC_021166.1_1542686	Ca_kabuli_chr07	1542686	[T/G]	1067301229
Ca_Kabuli_NC_021166.1_1542711	Ca_kabuli_chr07	1542711	[A/C]	1067301230
Ca_Kabuli_NC_021166.1_1655087	Ca_kabuli_chr07	1655087	[C/A]	1067301231
Ca_Kabuli_NC_021166.1_1668139	Ca_kabuli_chr07	1668139	[T/C]	1067301232
Ca_Kabuli_NC_021166.1_1668197	Ca_kabuli_chr07	1668197	[A/G]	1067301233
Ca_Kabuli_NC_021166.1_1690930	Ca_kabuli_chr07	1690930	[T/C]	1067301234
Ca_Kabuli_NC_021166.1_1704808	Ca_kabuli_chr07	1704808	[G/A]	1067301235
Ca_Kabuli_NC_021166.1_1704814	Ca_kabuli_chr07	1704814	[T/C]	1067301236
Ca_Kabuli_NC_021166.1_1787368	Ca_kabuli_chr07	1787368	[T/C]	1067301237
Ca_Kabuli_NC_021166.1_1813421	Ca_kabuli_chr07	1813421	[T/A]	1067301238
Ca_Kabuli_NC_021166.1_1818008	Ca_kabuli_chr07	1818008	[T/C]	1067301239
Ca_Kabuli_NC_021166.1_1818016	Ca_kabuli_chr07	1818016	[T/A]	1067301240
Ca_Kabuli_NC_021166.1_1962779	Ca_kabuli_chr07	1962779	[A/C]	1067301241
Ca_Kabuli_NC_021166.1_2012887	Ca_kabuli_chr07	2012887	[A/T]	1067301242
Ca_Kabuli_NC_021166.1_2075524	Ca_kabuli_chr07	2075524	[A/G]	1067301243
Ca_Kabuli_NC_021166.1_2076232	Ca_kabuli_chr07	2076232	[A/C]	1067301244

SNP IDs	Chromosomes	Physical positions (bp)	SNPs	NCBI SNPdb accession IDs (NCBI_ss#)
Ca_Kabuli_NC_021166.1_2078173	Ca_kabuli_chr07	2078173	[T/G]	1067301245
Ca_Kabuli_NC_021166.1_2078199	Ca_kabuli_chr07	2078199	[A/C]	1067301246
Ca_Kabuli_NC_021166.1_2078242	Ca_kabuli_chr07	2078242	[A/T]	1067301247
Ca_Kabuli_NC_021166.1_2082069	Ca_kabuli_chr07	2082069	[T/G]	1067301248
Ca_Kabuli_NC_021166.1_2086339	Ca_kabuli_chr07	2086339	[T/G]	1067301249
Ca_Kabuli_NC_021166.1_2086352	Ca_kabuli_chr07	2086352	[T/G]	1067301250
Ca_Kabuli_NC_021166.1_2106917	Ca_kabuli_chr07	2106917	[A/G]	1067301251
Ca_Kabuli_NC_021166.1_2167473	Ca_kabuli_chr07	2167473	[T/A]	1067301252
Ca_Kabuli_NC_021166.1_2167499	Ca_kabuli_chr07	2167499	[A/G]	1067301253
Ca_Kabuli_NC_021166.1_2167530	Ca_kabuli_chr07	2167530	[T/C]	1067301254
Ca_Kabuli_NC_021166.1_2211895	Ca_kabuli_chr07	2211895	[A/G]	1067301255
Ca_Kabuli_NC_021166.1_2211898	Ca_kabuli_chr07	2211898	[T/C]	1067301256
Ca_Kabuli_NC_021166.1_2211905	Ca_kabuli_chr07	2211905	[T/C]	1067301257
Ca_Kabuli_NC_021166.1_2213655	Ca_kabuli_chr07	2213655	[T/A]	1067301258
Ca_Kabuli_NC_021166.1_2230358	Ca_kabuli_chr07	2230358	[T/G]	1067301259
Ca_Kabuli_NC_021166.1_2259629	Ca_kabuli_chr07	2259629	[T/C]	1067301260
Ca_Kabuli_NC_021166.1_2259664	Ca_kabuli_chr07	2259664	[A/T]	1067301261
Ca_Kabuli_NC_021166.1_2262889	Ca_kabuli_chr07	2262889	[A/C]	1067301262
Ca_Kabuli_NC_021166.1_2262896	Ca_kabuli_chr07	2262896	[A/C]	1067301263
Ca_Kabuli_NC_021166.1_2391350	Ca_kabuli_chr07	2391350	[T/G]	1067301264
Ca_Kabuli_NC_021166.1_2453653	Ca_kabuli_chr07	2453653	[T/G]	1067301265
Ca_Kabuli_NC_021166.1_2487540	Ca_kabuli_chr07	2487540	[C/A]	1067301266
Ca_Kabuli_NC_021166.1_2487542	Ca_kabuli_chr07	2487542	[G/A]	1067301267
Ca_Kabuli_NC_021166.1_2522738	Ca_kabuli_chr07	2522738	[A/C]	1067301268
Ca_Kabuli_NC_021166.1_2522739	Ca_kabuli_chr07	2522739	[G/C]	1067301269
Ca_Kabuli_NC_021166.1_2549728	Ca_kabuli_chr07	2549728	[C/T]	1067301270
Ca_Kabuli_NC_021166.1_2549729	Ca_kabuli_chr07	2549729	[T/G]	1067301271
Ca_Kabuli_NC_021166.1_2554390	Ca_kabuli_chr07	2554390	[C/A]	1067301272
Ca_Kabuli_NC_021166.1_2554391	Ca_kabuli_chr07	2554391	[C/G]	1067301273
Ca_Kabuli_NC_021166.1_2554392	Ca_kabuli_chr07	2554392	[T/A]	1067301274
Ca_Kabuli_NC_021166.1_2554393	Ca_kabuli_chr07	2554393	[G/T]	1067301275
Ca_Kabuli_NC_021166.1_2609796	Ca_kabuli_chr07	2609796	[A/G]	1067301276
Ca_Kabuli_NC_021166.1_2639959	Ca_kabuli_chr07	2639959	[A/G]	1067301277
Ca_Kabuli_NC_021166.1_2639984	Ca_kabuli_chr07	2639984	[T/G]	1067301278

SNP IDs	Chromosomes	Physical positions (bp)	SNPs	NCBI SNPdb accession IDs (NCBI_ss#)
Ca_Kabuli_NC_021166.1_2654446	Ca_kabuli_chr07	2654446	[A/T]	1067301279
Ca_Kabuli_NC_021166.1_2740954	Ca_kabuli_chr07	2740954	[T/C]	1067301280
Ca_Kabuli_NC_021166.1_2740961	Ca_kabuli_chr07	2740961	[T/C]	1067301281
Ca_Kabuli_NC_021166.1_2745693	Ca_kabuli_chr07	2745693	[A/C]	1067301282
Ca_Kabuli_NC_021166.1_2745699	Ca_kabuli_chr07	2745699	[T/C]	1067301283
Ca_Kabuli_NC_021166.1_2793055	Ca_kabuli_chr07	2793055	[T/G]	1067301284
Ca_Kabuli_NC_021166.1_2881228	Ca_kabuli_chr07	2881228	[A/T]	1067301285
Ca_Kabuli_NC_021166.1_2881229	Ca_kabuli_chr07	2881229	[T/A]	1067301286
Ca_Kabuli_NC_021166.1_2881230	Ca_kabuli_chr07	2881230	[A/T]	1067301287
Ca_Kabuli_NC_021166.1_2881234	Ca_kabuli_chr07	2881234	[A/G]	1067301288
Ca_Kabuli_NC_021166.1_2881235	Ca_kabuli_chr07	2881235	[G/A]	1067301289
Ca_Kabuli_NC_021166.1_2881236	Ca_kabuli_chr07	2881236	[A/G]	1067301290
Ca_Kabuli_NC_021166.1_3012247	Ca_kabuli_chr07	3012247	[T/G]	1067301291
Ca_Kabuli_NC_021166.1_3012253	Ca_kabuli_chr07	3012253	[A/G]	1067301292
Ca_Kabuli_NC_021166.1_3022874	Ca_kabuli_chr07	3022874	[T/A]	1067301293
Ca_Kabuli_NC_021166.1_3022929	Ca_kabuli_chr07	3022929	[A/T]	1067301294
Ca_Kabuli_NC_021166.1_3024940	Ca_kabuli_chr07	3024940	[A/G]	1067301295
Ca_Kabuli_NC_021166.1_3029708	Ca_kabuli_chr07	3029708	[T/G]	1067301296
Ca_Kabuli_NC_021166.1_3029735	Ca_kabuli_chr07	3029735	[T/G]	1067301297
Ca_Kabuli_NC_021166.1_3070518	Ca_kabuli_chr07	3070518	[A/C]	1067301298
Ca_Kabuli_NC_021166.1_3070531	Ca_kabuli_chr07	3070531	[A/C]	1067301299
Ca_Kabuli_NC_021166.1_3089276	Ca_kabuli_chr07	3089276	[A/T]	1067301300
Ca_Kabuli_NC_021166.1_3093502	Ca_kabuli_chr07	3093502	[T/C]	1067301301
Ca_Kabuli_NC_021166.1_3186295	Ca_kabuli_chr07	3186295	[A/G]	1067301302
Ca_Kabuli_NC_021166.1_3198209	Ca_kabuli_chr07	3198209	[T/A]	1067301303
Ca_Kabuli_NC_021166.1_3198280	Ca_kabuli_chr07	3198280	[A/C]	1067301304
Ca_Kabuli_NC_021166.1_3265807	Ca_kabuli_chr07	3265807	[A/T]	1067301305
Ca_Kabuli_NC_021166.1_3265844	Ca_kabuli_chr07	3265844	[A/T]	1067301306
Ca_Kabuli_NC_021166.1_3336763	Ca_kabuli_chr07	3336763	[T/G]	1067301307
Ca_Kabuli_NC_021166.1_3336766	Ca_kabuli_chr07	3336766	[A/G]	1067301308
Ca_Kabuli_NC_021166.1_3350846	Ca_kabuli_chr07	3350846	[T/C]	1067301309
Ca_Kabuli_NC_021166.1_3353136	Ca_kabuli_chr07	3353136	[A/C]	1067301310
Ca_Kabuli_NC_021166.1_3354249	Ca_kabuli_chr07	3354249	[A/T]	1067301311
Ca_Kabuli_NC_021166.1_3355562	Ca_kabuli_chr07	3355562	[T/A]	1067301312

SNP IDs	Chromosomes	Physical positions (bp)	SNPs	NCBI SNPdb accession IDs (NCBI_ss#)
Ca_Kabuli_NC_021166.1_3355581	Ca_kabuli_chr07	3355581	[T/G]	1067301313
Ca_Kabuli_NC_021166.1_3355585	Ca_kabuli_chr07	3355585	[T/G]	1067301314
Ca_Kabuli_NC_021166.1_3355627	Ca_kabuli_chr07	3355627	[A/G]	1067301315
Ca_Kabuli_NC_021166.1_3356062	Ca_kabuli_chr07	3356062	[G/T]	1067301316
Ca_Kabuli_NC_021166.1_3356147	Ca_kabuli_chr07	3356147	[T/C]	1067301317
Ca_Kabuli_NC_021166.1_3356155	Ca_kabuli_chr07	3356155	[T/C]	1067301318
Ca_Kabuli_NC_021166.1_3356159	Ca_kabuli_chr07	3356159	[T/G]	1067301319
Ca_Kabuli_NC_021166.1_3469213	Ca_kabuli_chr07	3469213	[A/C]	1067301320
Ca_Kabuli_NC_021166.1_3511221	Ca_kabuli_chr07	3511221	[T/A]	1067301321
Ca_Kabuli_NC_021166.1_3511237	Ca_kabuli_chr07	3511237	[T/G]	1067301322
Ca_Kabuli_NC_021166.1_3519701	Ca_kabuli_chr07	3519701	[A/C]	1067301323
Ca_Kabuli_NC_021166.1_3521112	Ca_kabuli_chr07	3521112	[T/G]	1067301324
Ca_Kabuli_NC_021166.1_3521257	Ca_kabuli_chr07	3521257	[G/A]	1067301325
Ca_Kabuli_NC_021166.1_3521862	Ca_kabuli_chr07	3521862	[T/C]	1067301326
Ca_Kabuli_NC_021166.1_3540487	Ca_kabuli_chr07	3540487	[T/C]	1067301327
Ca_Kabuli_NC_021166.1_3540504	Ca_kabuli_chr07	3540504	[T/A]	1067301328
Ca_Kabuli_NC_021166.1_3555127	Ca_kabuli_chr07	3555127	[A/T]	1067301329
Ca_Kabuli_NC_021166.1_3555132	Ca_kabuli_chr07	3555132	[T/C]	1067301330
Ca_Kabuli_NC_021166.1_3555258	Ca_kabuli_chr07	3555258	[G/C]	1067301331
Ca_Kabuli_NC_021166.1_3560232	Ca_kabuli_chr07	3560232	[T/C]	1067301332
Ca_Kabuli_NC_021166.1_3582271	Ca_kabuli_chr07	3582271	[A/C]	1067301333
Ca_Kabuli_NC_021166.1_3596509	Ca_kabuli_chr07	3596509	[T/A]	1067301334
Ca_Kabuli_NC_021166.1_3600826	Ca_kabuli_chr07	3600826	[C/T]	1067301335
Ca_Kabuli_NC_021166.1_3600833	Ca_kabuli_chr07	3600833	[T/G]	1067301336
Ca_Kabuli_NC_021166.1_3600987	Ca_kabuli_chr07	3600987	[A/C]	1067301337
Ca_Kabuli_NC_021166.1_3606381	Ca_kabuli_chr07	3606381	[T/C]	1067301338
Ca_Kabuli_NC_021166.1_3606389	Ca_kabuli_chr07	3606389	[T/C]	1067301339
Ca_Kabuli_NC_021166.1_3606394	Ca_kabuli_chr07	3606394	[A/T]	1067301340
Ca_Kabuli_NC_021166.1_3665648	Ca_kabuli_chr07	3665648	[G/T]	1067301341
Ca_Kabuli_NC_021166.1_3665649	Ca_kabuli_chr07	3665649	[T/C]	1067301342
Ca_Kabuli_NC_021166.1_3665686	Ca_kabuli_chr07	3665686	[C/T]	1067301343
Ca_Kabuli_NC_021166.1_3672192	Ca_kabuli_chr07	3672192	[T/C]	1067301344
Ca_Kabuli_NC_021166.1_3672236	Ca_kabuli_chr07	3672236	[G/A]	1067301345
Ca_Kabuli_NC_021166.1_3734068	Ca_kabuli_chr07	3734068	[A/G]	1067301346

SNP IDs	Chromosomes	Physical positions (bp)	SNPs	NCBI SNPdb accession IDs (NCBI_ss#)
Ca_Kabuli_NC_021166.1_3746446	Ca_kabuli_chr07	3746446	[A/C]	1067301347
Ca_Kabuli_NC_021166.1_3792833	Ca_kabuli_chr07	3792833	[T/C]	1067301348
Ca_Kabuli_NC_021166.1_3803144	Ca_kabuli_chr07	3803144	[A/C]	1067301349
Ca_Kabuli_NC_021166.1_3828459	Ca_kabuli_chr07	3828459	[T/A]	1067301350
Ca_Kabuli_NC_021166.1_3828460	Ca_kabuli_chr07	3828460	[A/T]	1067301351
Ca_Kabuli_NC_021166.1_3828467	Ca_kabuli_chr07	3828467	[A/G]	1067301352
Ca_Kabuli_NC_021166.1_3829182	Ca_kabuli_chr07	3829182	[A/G]	1067301353
Ca_Kabuli_NC_021166.1_3843124	Ca_kabuli_chr07	3843124	[G/T]	1067301354
Ca_Kabuli_NC_021166.1_3867048	Ca_kabuli_chr07	3867048	[T/G]	1067301355
Ca_Kabuli_NC_021166.1_3884997	Ca_kabuli_chr07	3884997	[A/G]	1067301356
Ca_Kabuli_NC_021166.1_3922042	Ca_kabuli_chr07	3922042	[T/A]	1067301357
Ca_Kabuli_NC_021166.1_3922049	Ca_kabuli_chr07	3922049	[A/G]	1067301358
Ca_Kabuli_NC_021166.1_3924982	Ca_kabuli_chr07	3924982	[A/C]	1067301359
Ca_Kabuli_NC_021166.1_3925004	Ca_kabuli_chr07	3925004	[A/G]	1067301360
Ca_Kabuli_NC_021166.1_3971250	Ca_kabuli_chr07	3971250	[T/C]	1067301361
Ca_Kabuli_NC_021166.1_3982609	Ca_kabuli_chr07	3982609	[C/A]	1067301362
Ca_Kabuli_NC_021166.1_3982616	Ca_kabuli_chr07	3982616	[T/C]	1067301363
Ca_Kabuli_NC_021166.1_4017869	Ca_kabuli_chr07	4017869	[G/T]	1067301364
Ca_Kabuli_NC_021166.1_4017874	Ca_kabuli_chr07	4017874	[A/C]	1067301365
Ca_Kabuli_NC_021166.1_4073827	Ca_kabuli_chr07	4073827	[A/C]	1067301366
Ca_Kabuli_NC_021166.1_4073844	Ca_kabuli_chr07	4073844	[T/C]	1067301367
Ca_Kabuli_NC_021166.1_4079212	Ca_kabuli_chr07	4079212	[T/C]	1067301368
Ca_Kabuli_NC_021166.1_4080841	Ca_kabuli_chr07	4080841	[T/A]	1067301369
Ca_Kabuli_NC_021166.1_4080868	Ca_kabuli_chr07	4080868	[T/A]	1067301370
Ca_Kabuli_NC_021166.1_4106134	Ca_kabuli_chr07	4106134	[T/G]	1067301371
Ca_Kabuli_NC_021166.1_4183185	Ca_kabuli_chr07	4183185	[G/A]	1067301372
Ca_Kabuli_NC_021166.1_4194848	Ca_kabuli_chr07	4194848	[C/T]	1067301373
Ca_Kabuli_NC_021166.1_4194852	Ca_kabuli_chr07	4194852	[A/C]	1067301374
Ca_Kabuli_NC_021166.1_4194854	Ca_kabuli_chr07	4194854	[T/C]	1067301375
Ca_Kabuli_NC_021166.1_4198812	Ca_kabuli_chr07	4198812	[A/C]	1067301376
Ca_Kabuli_NC_021166.1_4198838	Ca_kabuli_chr07	4198838	[A/C]	1067301377
Ca_Kabuli_NC_021166.1_4200958	Ca_kabuli_chr07	4200958	[T/G]	1067301378
Ca_Kabuli_NC_021166.1_4209424	Ca_kabuli_chr07	4209424	[A/C]	1067301379
Ca_Kabuli_NC_021166.1_4225282	Ca_kabuli_chr07	4225282	[T/G]	1067301380

SNP IDs	Chromosomes	Physical positions (bp)	SNPs	NCBI SNPdb accession IDs (NCBI_ss#)
Ca_Kabuli_NC_021166.1_4225285	Ca_kabuli_chr07	4225285	[T/G]	1067301381
Ca_Kabuli_NC_021166.1_4225294	Ca_kabuli_chr07	4225294	[A/G]	1067301382
Ca_Kabuli_NC_021166.1_4225298	Ca_kabuli_chr07	4225298	[A/G]	1067301383
Ca_Kabuli_NC_021166.1_4225305	Ca_kabuli_chr07	4225305	[T/C]	1067301384
Ca_Kabuli_NC_021166.1_4240704	Ca_kabuli_chr07	4240704	[A/C]	1067301385
Ca_Kabuli_NC_021166.1_4240784	Ca_kabuli_chr07	4240784	[T/G]	1067301386
Ca_Kabuli_NC_021166.1_4240787	Ca_kabuli_chr07	4240787	[A/G]	1067301387
Ca_Kabuli_NC_021166.1_4240790	Ca_kabuli_chr07	4240790	[T/C]	1067301388
Ca_Kabuli_NC_021166.1_4240796	Ca_kabuli_chr07	4240796	[A/G]	1067301389
Ca_Kabuli_NC_021166.1_4240799	Ca_kabuli_chr07	4240799	[T/G]	1067301390
Ca_Kabuli_NC_021166.1_4284506	Ca_kabuli_chr07	4284506	[T/C]	1067301391
Ca_Kabuli_NC_021166.1_4284510	Ca_kabuli_chr07	4284510	[T/C]	1067301392
Ca_Kabuli_NC_021166.1_4345128	Ca_kabuli_chr07	4345128	[T/G]	1067301393
Ca_Kabuli_NC_021166.1_4345339	Ca_kabuli_chr07	4345339	[T/C]	1067301394
Ca_Kabuli_NC_021166.1_4358380	Ca_kabuli_chr07	4358380	[C/T]	1067301395
Ca_Kabuli_NC_021166.1_4470279	Ca_kabuli_chr07	4470279	[A/C]	1067301396
Ca_Kabuli_NC_021166.1_4495064	Ca_kabuli_chr07	4495064	[T/G]	1067301397
Ca_Kabuli_NC_021166.1_4495075	Ca_kabuli_chr07	4495075	[A/T]	1067301398
Ca_Kabuli_NC_021166.1_4552596	Ca_kabuli_chr07	4552596	[A/G]	1067301399
Ca_Kabuli_NC_021166.1_4552623	Ca_kabuli_chr07	4552623	[A/G]	1067301400
Ca_Kabuli_NC_021166.1_4552740	Ca_kabuli_chr07	4552740	[A/C]	1067301401
Ca_Kabuli_NC_021166.1_4552745	Ca_kabuli_chr07	4552745	[A/G]	1067301402
Ca_Kabuli_NC_021166.1_4552751	Ca_kabuli_chr07	4552751	[T/C]	1067301403
Ca_Kabuli_NC_021166.1_4553487	Ca_kabuli_chr07	4553487	[A/C]	1067301404
Ca_Kabuli_NC_021166.1_4573210	Ca_kabuli_chr07	4573210	[T/C]	1067301405
Ca_Kabuli_NC_021166.1_4625723	Ca_kabuli_chr07	4625723	[T/C]	1067301406
Ca_Kabuli_NC_021166.1_4625737	Ca_kabuli_chr07	4625737	[T/C]	1067301407
Ca_Kabuli_NC_021166.1_4625757	Ca_kabuli_chr07	4625757	[A/G]	1067301408
Ca_Kabuli_NC_021166.1_4653114	Ca_kabuli_chr07	4653114	[T/G]	1067301409
Ca_Kabuli_NC_021166.1_4653200	Ca_kabuli_chr07	4653200	[G/T]	1067301410
Ca_Kabuli_NC_021166.1_4653201	Ca_kabuli_chr07	4653201	[T/G]	1067301411
Ca_Kabuli_NC_021166.1_4653207	Ca_kabuli_chr07	4653207	[T/G]	1067301412
Ca_Kabuli_NC_021166.1_4705772	Ca_kabuli_chr07	4705772	[T/G]	1067301413
Ca_Kabuli_NC_021166.1_4705803	Ca_kabuli_chr07	4705803	[A/C]	1067301414

SNP IDs	Chromosomes	Physical positions (bp)	SNPs	NCBI SNPdb accession IDs (NCBI_ss#)
Ca_Kabuli_NC_021166.1_4726635	Ca_kabuli_chr07	4726635	[T/C]	1067301415
Ca_Kabuli_NC_021166.1_4726662	Ca_kabuli_chr07	4726662	[A/C]	1067301416
Ca_Kabuli_NC_021166.1_4726667	Ca_kabuli_chr07	4726667	[A/G]	1067301417
Ca_Kabuli_NC_021166.1_4726676	Ca_kabuli_chr07	4726676	[T/A]	1067301418
Ca_Kabuli_NC_021166.1_4749212	Ca_kabuli_chr07	4749212	[T/G]	1067301419
Ca_Kabuli_NC_021166.1_4749260	Ca_kabuli_chr07	4749260	[A/C]	1067301420
Ca_Kabuli_NC_021166.1_4749275	Ca_kabuli_chr07	4749275	[A/C]	1067301421
Ca_Kabuli_NC_021166.1_4752718	Ca_kabuli_chr07	4752718	[A/G]	1067301422
Ca_Kabuli_NC_021166.1_4752720	Ca_kabuli_chr07	4752720	[C/G]	1067301423
Ca_Kabuli_NC_021166.1_4752721	Ca_kabuli_chr07	4752721	[T/G]	1067301424
Ca_Kabuli_NC_021166.1_4756302	Ca_kabuli_chr07	4756302	[T/C]	1067301425
Ca_Kabuli_NC_021166.1_4772488	Ca_kabuli_chr07	4772488	[T/G]	1067301426
Ca_Kabuli_NC_021166.1_4772494	Ca_kabuli_chr07	4772494	[T/G]	1067301427
Ca_Kabuli_NC_021166.1_4772506	Ca_kabuli_chr07	4772506	[A/G]	1067301428
Ca_Kabuli_NC_021166.1_4772517	Ca_kabuli_chr07	4772517	[A/G]	1067301429
Ca_Kabuli_NC_021166.1_4772529	Ca_kabuli_chr07	4772529	[A/G]	1067301430
Ca_Kabuli_NC_021166.1_4772534	Ca_kabuli_chr07	4772534	[A/G]	1067301431
Ca_Kabuli_NC_021166.1_4872404	Ca_kabuli_chr07	4872404	[T/C]	1067301432
Ca_Kabuli_NC_021166.1_4884236	Ca_kabuli_chr07	4884236	[C/A]	1067301433
Ca_Kabuli_NC_021166.1_4946036	Ca_kabuli_chr07	4946036	[T/G]	1067301434
Ca_Kabuli_NC_021166.1_5167444	Ca_kabuli_chr07	5167444	[G/A]	1067301435
Ca_Kabuli_NC_021166.1_5258830	Ca_kabuli_chr07	5258830	[T/C]	1067301436
Ca_Kabuli_NC_021166.1_5310202	Ca_kabuli_chr07	5310202	[T/C]	1067301437
Ca_Kabuli_NC_021166.1_5310277	Ca_kabuli_chr07	5310277	[A/T]	1067301438
Ca_Kabuli_NC_021166.1_5314496	Ca_kabuli_chr07	5314496	[A/C]	1067301439
Ca_Kabuli_NC_021166.1_5335404	Ca_kabuli_chr07	5335404	[A/T]	1067301440
Ca_Kabuli_NC_021166.1_5335413	Ca_kabuli_chr07	5335413	[T/A]	1067301441
Ca_Kabuli_NC_021166.1_5335427	Ca_kabuli_chr07	5335427	[A/G]	1067301442
Ca_Kabuli_NC_021166.1_5338685	Ca_kabuli_chr07	5338685	[C/A]	1067301443
Ca_Kabuli_NC_021166.1_5361519	Ca_kabuli_chr07	5361519	[A/C]	1067301444
Ca_Kabuli_NC_021166.1_5361550	Ca_kabuli_chr07	5361550	[T/G]	1067301445
Ca_Kabuli_NC_021166.1_5379203	Ca_kabuli_chr07	5379203	[T/A]	1067301446
Ca_Kabuli_NC_021166.1_5383562	Ca_kabuli_chr07	5383562	[A/G]	1067301447
Ca_Kabuli_NC_021166.1_5383570	Ca_kabuli_chr07	5383570	[T/C]	1067301448

SNP IDs	Chromosomes	Physical positions (bp)	SNPs	NCBI SNPdb accession IDs (NCBI_ss#)
Ca_Kabuli_NC_021166.1_5383671	Ca_kabuli_chr07	5383671	[T/G]	1067301449
Ca_Kabuli_NC_021166.1_5383683	Ca_kabuli_chr07	5383683	[C/A]	1067301450
Ca_Kabuli_NC_021166.1_5383695	Ca_kabuli_chr07	5383695	[G/T]	1067301451
Ca_Kabuli_NC_021166.1_5383767	Ca_kabuli_chr07	5383767	[A/G]	1067301452
Ca_Kabuli_NC_021166.1_5384422	Ca_kabuli_chr07	5384422	[T/C]	1067301453
Ca_Kabuli_NC_021166.1_5406524	Ca_kabuli_chr07	5406524	[G/T]	1067301454
Ca_Kabuli_NC_021166.1_5421642	Ca_kabuli_chr07	5421642	[G/C]	1067301455
Ca_Kabuli_NC_021166.1_5421733	Ca_kabuli_chr07	5421733	[C/T]	1067301456
Ca_Kabuli_NC_021166.1_5447889	Ca_kabuli_chr07	5447889	[T/A]	1067301457
Ca_Kabuli_NC_021166.1_5464904	Ca_kabuli_chr07	5464904	[T/G]	1067301458
Ca_Kabuli_NC_021166.1_5466193	Ca_kabuli_chr07	5466193	[A/T]	1067301459
Ca_Kabuli_NC_021166.1_5467296	Ca_kabuli_chr07	5467296	[A/T]	1067301460
Ca_Kabuli_NC_021166.1_5467645	Ca_kabuli_chr07	5467645	[A/G]	1067301461
Ca_Kabuli_NC_021166.1_5467677	Ca_kabuli_chr07	5467677	[T/C]	1067301462
Ca_Kabuli_NC_021166.1_5467703	Ca_kabuli_chr07	5467703	[A/G]	1067301463
Ca_Kabuli_NC_021166.1_5467744	Ca_kabuli_chr07	5467744	[C/G]	1067301464
Ca_Kabuli_NC_021166.1_5474255	Ca_kabuli_chr07	5474255	[C/T]	1067301465
Ca_Kabuli_NC_021166.1_5477139	Ca_kabuli_chr07	5477139	[C/A]	1067301466
Ca_Kabuli_NC_021166.1_5504454	Ca_kabuli_chr07	5504454	[C/T]	1067301467
Ca_Kabuli_NC_021166.1_5508045	Ca_kabuli_chr07	5508045	[A/C]	1067301468
Ca_Kabuli_NC_021166.1_5508097	Ca_kabuli_chr07	5508097	[A/G]	1067301469
Ca_Kabuli_NC_021166.1_5508120	Ca_kabuli_chr07	5508120	[A/G]	1067301470
Ca_Kabuli_NC_021166.1_5508146	Ca_kabuli_chr07	5508146	[T/C]	1067301471
Ca_Kabuli_NC_021166.1_5508157	Ca_kabuli_chr07	5508157	[A/C]	1067301472
Ca_Kabuli_NC_021166.1_5508159	Ca_kabuli_chr07	5508159	[A/C]	1067301473
Ca_Kabuli_NC_021166.1_5508162	Ca_kabuli_chr07	5508162	[A/C]	1067301474
Ca_Kabuli_NC_021166.1_5510004	Ca_kabuli_chr07	5510004	[T/C]	1067301475
Ca_Kabuli_NC_021166.1_5510013	Ca_kabuli_chr07	5510013	[T/A]	1067301476
Ca_Kabuli_NC_021166.1_5510015	Ca_kabuli_chr07	5510015	[T/G]	1067301477
Ca_Kabuli_NC_021166.1_5513267	Ca_kabuli_chr07	5513267	[T/C]	1067301478
Ca_Kabuli_NC_021166.1_5520720	Ca_kabuli_chr07	5520720	[G/T]	1067301479
Ca_Kabuli_NC_021166.1_5521466	Ca_kabuli_chr07	5521466	[T/G]	1067301480
Ca_Kabuli_NC_021166.1_5521501	Ca_kabuli_chr07	5521501	[A/G]	1067301481
Ca_Kabuli_NC_021166.1_5521659	Ca_kabuli_chr07	5521659	[T/G]	1067301482

SNP IDs	Chromosomes	Physical positions (bp)	SNPs	NCBI SNPdb accession IDs (NCBI_ss#)
Ca_Kabuli_NC_021166.1_5529517	Ca_kabuli_chr07	5529517	[T/C]	1067301483
Ca_Kabuli_NC_021166.1_5529531	Ca_kabuli_chr07	5529531	[T/C]	1067301484
Ca_Kabuli_NC_021166.1_5529548	Ca_kabuli_chr07	5529548	[A/G]	1067301485
Ca_Kabuli_NC_021166.1_5529567	Ca_kabuli_chr07	5529567	[A/C]	1067301486
Ca_Kabuli_NC_021166.1_5529607	Ca_kabuli_chr07	5529607	[A/T]	1067301487
Ca_Kabuli_NC_021166.1_5529640	Ca_kabuli_chr07	5529640	[T/G]	1067301488
Ca_Kabuli_NC_021166.1_5529888	Ca_kabuli_chr07	5529888	[G/A]	1067301489
Ca_Kabuli_NC_021166.1_5537851	Ca_kabuli_chr07	5537851	[C/G]	1067301490
Ca_Kabuli_NC_021166.1_5537880	Ca_kabuli_chr07	5537880	[T/G]	1067301491
Ca_Kabuli_NC_021166.1_5542515	Ca_kabuli_chr07	5542515	[C/T]	1067301492
Ca_Kabuli_NC_021166.1_5542812	Ca_kabuli_chr07	5542812	[A/C]	1067301493
Ca_Kabuli_NC_021166.1_5549428	Ca_kabuli_chr07	5549428	[T/C]	1067301494
Ca_Kabuli_NC_021166.1_5549545	Ca_kabuli_chr07	5549545	[A/T]	1067301495
Ca_Kabuli_NC_021166.1_5554807	Ca_kabuli_chr07	5554807	[C/G]	1067301496
Ca_Kabuli_NC_021166.1_5554841	Ca_kabuli_chr07	5554841	[G/T]	1067301497
Ca_Kabuli_NC_021166.1_5560122	Ca_kabuli_chr07	5560122	[T/C]	1067301498
Ca_Kabuli_NC_021166.1_5577258	Ca_kabuli_chr07	5577258	[G/T]	1067301499
Ca_Kabuli_NC_021166.1_5577298	Ca_kabuli_chr07	5577298	[A/C]	1067301500
Ca_Kabuli_NC_021166.1_5577310	Ca_kabuli_chr07	5577310	[A/C]	1067301501
Ca_Kabuli_NC_021166.1_5577311	Ca_kabuli_chr07	5577311	[C/T]	1067301502
Ca_Kabuli_NC_021166.1_5577320	Ca_kabuli_chr07	5577320	[A/C]	1067301503
Ca_Kabuli_NC_021166.1_5577321	Ca_kabuli_chr07	5577321	[A/C]	1067301504
Ca_Kabuli_NC_021166.1_5577329	Ca_kabuli_chr07	5577329	[A/C]	1067301505
Ca_Kabuli_NC_021166.1_5593731	Ca_kabuli_chr07	5593731	[A/C]	1067301506
Ca_Kabuli_NC_021166.1_5631304	Ca_kabuli_chr07	5631304	[T/C]	1067301507
Ca_Kabuli_NC_021166.1_5662888	Ca_kabuli_chr07	5662888	[C/T]	1067301508
Ca_Kabuli_NC_021166.1_5662910	Ca_kabuli_chr07	5662910	[G/A]	1067301509
Ca_Kabuli_NC_021166.1_5668592	Ca_kabuli_chr07	5668592	[A/C]	1067301510
Ca_Kabuli_NC_021166.1_5671912	Ca_kabuli_chr07	5671912	[T/G]	1067301511
Ca_Kabuli_NC_021166.1_5671987	Ca_kabuli_chr07	5671987	[A/C]	1067301512
Ca_Kabuli_NC_021166.1_5672156	Ca_kabuli_chr07	5672156	[C/T]	1067301513
Ca_Kabuli_NC_021166.1_5672245	Ca_kabuli_chr07	5672245	[A/C]	1067301514
Ca_Kabuli_NC_021166.1_5672729	Ca_kabuli_chr07	5672729	[T/G]	1067301515
Ca_Kabuli_NC_021166.1_5678659	Ca_kabuli_chr07	5678659	[G/A]	1067301516

SNP IDs	Chromosomes	Physical positions (bp)	SNPs	NCBI SNPdb accession IDs (NCBI_ss#)
Ca_Kabuli_NC_021166.1_5678992	Ca_kabuli_chr07	5678992	[C/T]	1067301517
Ca_Kabuli_NC_021166.1_5694937	Ca_kabuli_chr07	5694937	[G/A]	1067301518
Ca_Kabuli_NC_021166.1_5694979	Ca_kabuli_chr07	5694979	[A/C]	1067301519
Ca_Kabuli_NC_021166.1_5694980	Ca_kabuli_chr07	5694980	[C/T]	1067301520
Ca_Kabuli_NC_021166.1_5713977	Ca_kabuli_chr07	5713977	[C/T]	1067301521
Ca_Kabuli_NC_021166.1_5714130	Ca_kabuli_chr07	5714130	[A/G]	1067301522
Ca_Kabuli_NC_021166.1_5728543	Ca_kabuli_chr07	5728543	[C/G]	1067301523
Ca_Kabuli_NC_021166.1_5730386	Ca_kabuli_chr07	5730386	[C/G]	1067301524
Ca_Kabuli_NC_021166.1_5731803	Ca_kabuli_chr07	5731803	[T/C]	1067301525
Ca_Kabuli_NC_021166.1_5731817	Ca_kabuli_chr07	5731817	[G/C]	1067301526
Ca_Kabuli_NC_021166.1_5735448	Ca_kabuli_chr07	5735448	[A/C]	1067301527
Ca_Kabuli_NC_021166.1_5735456	Ca_kabuli_chr07	5735456	[A/C]	1067301528
Ca_Kabuli_NC_021166.1_5735461	Ca_kabuli_chr07	5735461	[A/C]	1067301529
Ca_Kabuli_NC_021166.1_5735893	Ca_kabuli_chr07	5735893	[C/G]	1067301530
Ca_Kabuli_NC_021166.1_5735906	Ca_kabuli_chr07	5735906	[G/T]	1067301531
Ca_Kabuli_NC_021166.1_5737401	Ca_kabuli_chr07	5737401	[T/G]	1067301532
Ca_Kabuli_NC_021166.1_5737422	Ca_kabuli_chr07	5737422	[T/G]	1067301533
Ca_Kabuli_NC_021166.1_5756269	Ca_kabuli_chr07	5756269	[T/C]	1067301534
Ca_Kabuli_NC_021166.1_5769036	Ca_kabuli_chr07	5769036	[C/A]	1067301535
Ca_Kabuli_NC_021166.1_5772892	Ca_kabuli_chr07	5772892	[T/G]	1067301536
Ca_Kabuli_NC_021166.1_5774221	Ca_kabuli_chr07	5774221	[A/T]	1067301537
Ca_Kabuli_NC_021166.1_5774325	Ca_kabuli_chr07	5774325	[G/C]	1067301538
Ca_Kabuli_NC_021166.1_5774327	Ca_kabuli_chr07	5774327	[G/T]	1067301539
Ca_Kabuli_NC_021166.1_5774328	Ca_kabuli_chr07	5774328	[A/T]	1067301540
Ca_Kabuli_NC_021166.1_5774329	Ca_kabuli_chr07	5774329	[A/G]	1067301541
Ca_Kabuli_NC_021166.1_5802607	Ca_kabuli_chr07	5802607	[G/T]	1067301542
Ca_Kabuli_NC_021166.1_5834024	Ca_kabuli_chr07	5834024	[T/C]	1067301543
Ca_Kabuli_NC_021166.1_5836421	Ca_kabuli_chr07	5836421	[T/A]	1067301544
Ca_Kabuli_NC_021166.1_5846477	Ca_kabuli_chr07	5846477	[G/A]	1067301545
Ca_Kabuli_NC_021166.1_5849877	Ca_kabuli_chr07	5849877	[G/C]	1067301546
Ca_Kabuli_NC_021166.1_5849892	Ca_kabuli_chr07	5849892	[G/A]	1067301547
Ca_Kabuli_NC_021166.1_5849946	Ca_kabuli_chr07	5849946	[A/G]	1067301548
Ca_Kabuli_NC_021166.1_5849960	Ca_kabuli_chr07	5849960	[T/G]	1067301549
Ca_Kabuli_NC_021166.1_5852913	Ca_kabuli_chr07	5852913	[G/T]	1067301550

SNP IDs	Chromosomes	Physical positions (bp)	SNPs	NCBI SNPdb accession IDs (NCBI_ss#)
Ca_Kabuli_NC_021166.1_5854575	Ca_kabuli_chr07	5854575	[G/T]	1067301551
Ca_Kabuli_NC_021166.1_5854576	Ca_kabuli_chr07	5854576	[A/C]	1067301552
Ca_Kabuli_NC_021166.1_5854577	Ca_kabuli_chr07	5854577	[G/T]	1067301553
Ca_Kabuli_NC_021166.1_5854578	Ca_kabuli_chr07	5854578	[T/G]	1067301554
Ca_Kabuli_NC_021166.1_5854598	Ca_kabuli_chr07	5854598	[T/C]	1067301555
Ca_Kabuli_NC_021166.1_5916920	Ca_kabuli_chr07	5916920	[A/C]	1067301556
Ca_Kabuli_NC_021166.1_5916936	Ca_kabuli_chr07	5916936	[A/G]	1067301557
Ca_Kabuli_NC_021166.1_5916941	Ca_kabuli_chr07	5916941	[T/A]	1067301558
Ca_Kabuli_NC_021166.1_5925751	Ca_kabuli_chr07	5925751	[C/T]	1067301559
Ca_Kabuli_NC_021166.1_5933629	Ca_kabuli_chr07	5933629	[C/T]	1067301560
Ca_Kabuli_NC_021166.1_5946536	Ca_kabuli_chr07	5946536	[C/G]	1067301561
Ca_Kabuli_NC_021166.1_5950260	Ca_kabuli_chr07	5950260	[T/C]	1067301562
Ca_Kabuli_NC_021166.1_5950275	Ca_kabuli_chr07	5950275	[A/T]	1067301563
Ca_Kabuli_NC_021166.1_5950352	Ca_kabuli_chr07	5950352	[A/T]	1067301564
Ca_Kabuli_NC_021166.1_5951374	Ca_kabuli_chr07	5951374	[A/G]	1067301565
Ca_Kabuli_NC_021166.1_5957696	Ca_kabuli_chr07	5957696	[C/T]	1067301566
Ca_Kabuli_NC_021166.1_5957736	Ca_kabuli_chr07	5957736	[A/T]	1067301567
Ca_Kabuli_NC_021166.1_5963576	Ca_kabuli_chr07	5963576	[A/G]	1067301568
Ca_Kabuli_NC_021166.1_5963581	Ca_kabuli_chr07	5963581	[T/G]	1067301569
Ca_Kabuli_NC_021166.1_5963588	Ca_kabuli_chr07	5963588	[T/G]	1067301570
Ca_Kabuli_NC_021166.1_5963603	Ca_kabuli_chr07	5963603	[T/G]	1067301571
Ca_Kabuli_NC_021166.1_6012477	Ca_kabuli_chr07	6012477	[A/C]	1067301572
Ca_Kabuli_NC_021166.1_6012561	Ca_kabuli_chr07	6012561	[A/C]	1067301573
Ca_Kabuli_NC_021166.1_6029883	Ca_kabuli_chr07	6029883	[A/T]	1067301574
Ca_Kabuli_NC_021166.1_6029965	Ca_kabuli_chr07	6029965	[A/G]	1067301575
Ca_Kabuli_NC_021166.1_6029968	Ca_kabuli_chr07	6029968	[A/G]	1067301576
Ca_Kabuli_NC_021166.1_6029985	Ca_kabuli_chr07	6029985	[T/G]	1067301577
Ca_Kabuli_NC_021166.1_6040435	Ca_kabuli_chr07	6040435	[A/G]	1067301578
Ca_Kabuli_NC_021166.1_6066322	Ca_kabuli_chr07	6066322	[A/G]	1067301579
Ca_Kabuli_NC_021166.1_6069979	Ca_kabuli_chr07	6069979	[C/T]	1067301580
Ca_Kabuli_NC_021166.1_6070645	Ca_kabuli_chr07	6070645	[G/A]	1067301581
Ca_Kabuli_NC_021166.1_6096227	Ca_kabuli_chr07	6096227	[A/C]	1067301582
Ca_Kabuli_NC_021166.1_6096238	Ca_kabuli_chr07	6096238	[A/C]	1067301583
Ca_Kabuli_NC_021166.1_6108535	Ca_kabuli_chr07	6108535	[T/G]	1067301584

SNP IDs	Chromosomes	Physical positions (bp)	SNPs	NCBI SNPdb accession IDs (NCBI_ss#)
Ca_Kabuli_NC_021166.1_6108843	Ca_kabuli_chr07	6108843	[T/G]	1067301585
Ca_Kabuli_NC_021166.1_6108853	Ca_kabuli_chr07	6108853	[T/A]	1067301586
Ca_Kabuli_NC_021166.1_6109635	Ca_kabuli_chr07	6109635	[T/C]	1067301587
Ca_Kabuli_NC_021166.1_6109652	Ca_kabuli_chr07	6109652	[A/G]	1067301588
Ca_Kabuli_NC_021166.1_6110019	Ca_kabuli_chr07	6110019	[C/T]	1067301589
Ca_Kabuli_NC_021166.1_6110033	Ca_kabuli_chr07	6110033	[C/A]	1067301590
Ca_Kabuli_NC_021166.1_6110067	Ca_kabuli_chr07	6110067	[A/T]	1067301591
Ca_Kabuli_NC_021166.1_6111778	Ca_kabuli_chr07	6111778	[C/T]	1067301592
Ca_Kabuli_NC_021166.1_6136615	Ca_kabuli_chr07	6136615	[C/T]	1067301593
Ca_Kabuli_NC_021166.1_6195286	Ca_kabuli_chr07	6195286	[T/G]	1067301594
Ca_Kabuli_NC_021166.1_6213546	Ca_kabuli_chr07	6213546	[C/A]	1067301595
Ca_Kabuli_NC_021166.1_6213547	Ca_kabuli_chr07	6213547	[T/G]	1067301596
Ca_Kabuli_NC_021166.1_6325498	Ca_kabuli_chr07	6325498	[G/A]	1067301597
Ca_Kabuli_NC_021166.1_6372045	Ca_kabuli_chr07	6372045	[A/G]	1067301598
Ca_Kabuli_NC_021166.1_6372048	Ca_kabuli_chr07	6372048	[A/G]	1067301599
Ca_Kabuli_NC_021166.1_6372050	Ca_kabuli_chr07	6372050	[T/G]	1067301600
Ca_Kabuli_NC_021166.1_6393368	Ca_kabuli_chr07	6393368	[T/G]	1067301601
Ca_Kabuli_NC_021166.1_6410077	Ca_kabuli_chr07	6410077	[T/C]	1067301602
Ca_Kabuli_NC_021166.1_6492495	Ca_kabuli_chr07	6492495	[T/G]	1067301603
Ca_Kabuli_NC_021166.1_6539116	Ca_kabuli_chr07	6539116	[G/C]	1067301604
Ca_Kabuli_NC_021166.1_6539146	Ca_kabuli_chr07	6539146	[A/C]	1067301605
Ca_Kabuli_NC_021166.1_6540624	Ca_kabuli_chr07	6540624	[G/A]	1067301606
Ca_Kabuli_NC_021166.1_6559538	Ca_kabuli_chr07	6559538	[T/G]	1067301607
Ca_Kabuli_NC_021166.1_6559540	Ca_kabuli_chr07	6559540	[T/C]	1067301608
Ca_Kabuli_NC_021166.1_6559542	Ca_kabuli_chr07	6559542	[A/G]	1067301609
Ca_Kabuli_NC_021166.1_6559580	Ca_kabuli_chr07	6559580	[A/G]	1067301610
Ca_Kabuli_NC_021166.1_6559583	Ca_kabuli_chr07	6559583	[T/A]	1067301611
Ca_Kabuli_NC_021166.1_6559587	Ca_kabuli_chr07	6559587	[T/C]	1067301612
Ca_Kabuli_NC_021166.1_6559600	Ca_kabuli_chr07	6559600	[A/T]	1067301613
Ca_Kabuli_NC_021166.1_6559604	Ca_kabuli_chr07	6559604	[A/G]	1067301614
Ca_Kabuli_NC_021166.1_6565518	Ca_kabuli_chr07	6565518	[T/C]	1067301615
Ca_Kabuli_NC_021166.1_6566303	Ca_kabuli_chr07	6566303	[T/A]	1067301616
Ca_Kabuli_NC_021166.1_6581696	Ca_kabuli_chr07	6581696	[T/A]	1067301617
Ca_Kabuli_NC_021166.1_6581698	Ca_kabuli_chr07	6581698	[G/T]	1067301618

SNP IDs	Chromosomes	Physical positions (bp)	SNPs	NCBI SNPdb accession IDs (NCBI_ss#)
Ca_Kabuli_NC_021166.1_6648414	Ca_kabuli_chr07	6648414	[C/A]	1067301619
Ca_Kabuli_NC_021166.1_6654620	Ca_kabuli_chr07	6654620	[A/T]	1067301620
Ca_Kabuli_NC_021166.1_6654630	Ca_kabuli_chr07	6654630	[T/C]	1067301621
Ca_Kabuli_NC_021166.1_6654646	Ca_kabuli_chr07	6654646	[A/C]	1067301622
Ca_Kabuli_NC_021166.1_6681019	Ca_kabuli_chr07	6681019	[A/C]	1067301623
Ca_Kabuli_NC_021166.1_6681023	Ca_kabuli_chr07	6681023	[A/G]	1067301624
Ca_Kabuli_NC_021166.1_6703849	Ca_kabuli_chr07	6703849	[G/T]	1067301625
Ca_Kabuli_NC_021166.1_6721629	Ca_kabuli_chr07	6721629	[A/G]	1067301626
Ca_Kabuli_NC_021166.1_6721679	Ca_kabuli_chr07	6721679	[A/G]	1067301627
Ca_Kabuli_NC_021166.1_6721684	Ca_kabuli_chr07	6721684	[A/G]	1067301628
Ca_Kabuli_NC_021166.1_6721732	Ca_kabuli_chr07	6721732	[A/G]	1067301629
Ca_Kabuli_NC_021166.1_6721769	Ca_kabuli_chr07	6721769	[T/G]	1067301630
Ca_Kabuli_NC_021166.1_6726521	Ca_kabuli_chr07	6726521	[A/G]	1067301631
Ca_Kabuli_NC_021166.1_6742476	Ca_kabuli_chr07	6742476	[A/C]	1067301632
Ca_Kabuli_NC_021166.1_6769706	Ca_kabuli_chr07	6769706	[T/G]	1067301633
Ca_Kabuli_NC_021166.1_6769723	Ca_kabuli_chr07	6769723	[A/G]	1067301634
Ca_Kabuli_NC_021166.1_6780547	Ca_kabuli_chr07	6780547	[C/A]	1067301635
Ca_Kabuli_NC_021166.1_6780773	Ca_kabuli_chr07	6780773	[T/C]	1067301636
Ca_Kabuli_NC_021166.1_6788869	Ca_kabuli_chr07	6788869	[C/A]	1067301637
Ca_Kabuli_NC_021166.1_6792321	Ca_kabuli_chr07	6792321	[A/C]	1067301638
Ca_Kabuli_NC_021166.1_6792563	Ca_kabuli_chr07	6792563	[G/C]	1067301639
Ca_Kabuli_NC_021166.1_6792566	Ca_kabuli_chr07	6792566	[A/C]	1067301640
Ca_Kabuli_NC_021166.1_6792568	Ca_kabuli_chr07	6792568	[T/G]	1067301641
Ca_Kabuli_NC_021166.1_6807839	Ca_kabuli_chr07	6807839	[T/G]	1067301642
Ca_Kabuli_NC_021166.1_6807907	Ca_kabuli_chr07	6807907	[A/T]	1067301643
Ca_Kabuli_NC_021166.1_6826211	Ca_kabuli_chr07	6826211	[A/G]	1067301644
Ca_Kabuli_NC_021166.1_6826869	Ca_kabuli_chr07	6826869	[C/T]	1067301645
Ca_Kabuli_NC_021166.1_6826870	Ca_kabuli_chr07	6826870	[T/G]	1067301646
Ca_Kabuli_NC_021166.1_6865060	Ca_kabuli_chr07	6865060	[T/A]	1067301647
Ca_Kabuli_NC_021166.1_6865061	Ca_kabuli_chr07	6865061	[C/T]	1067301648
Ca_Kabuli_NC_021166.1_6865062	Ca_kabuli_chr07	6865062	[A/C]	1067301649
Ca_Kabuli_NC_021166.1_6865063	Ca_kabuli_chr07	6865063	[G/T]	1067301650
Ca_Kabuli_NC_021166.1_6923632	Ca_kabuli_chr07	6923632	[T/C]	1067301651
Ca_Kabuli_NC_021166.1_6923652	Ca_kabuli_chr07	6923652	[T/G]	1067301652

SNP IDs	Chromosomes	Physical positions (bp)	SNPs	NCBI SNPdb accession IDs (NCBI_ss#)
Ca_Kabuli_NC_021166.1_6970678	Ca_kabuli_chr07	6970678	[T/C]	1067301653
Ca_Kabuli_NC_021166.1_6970704	Ca_kabuli_chr07	6970704	[A/C]	1067301654
Ca_Kabuli_NC_021166.1_6979661	Ca_kabuli_chr07	6979661	[A/C]	1067301655
Ca_Kabuli_NC_021166.1_7008352	Ca_kabuli_chr07	7008352	[A/G]	1067301656
Ca_Kabuli_NC_021166.1_7043352	Ca_kabuli_chr07	7043352	[A/G]	1067301657
Ca_Kabuli_NC_021166.1_7043832	Ca_kabuli_chr07	7043832	[A/G]	1067301658
Ca_Kabuli_NC_021166.1_7059582	Ca_kabuli_chr07	7059582	[T/G]	1067301659
Ca_Kabuli_NC_021166.1_7089263	Ca_kabuli_chr07	7089263	[T/A]	1067301660
Ca_Kabuli_NC_021166.1_7089333	Ca_kabuli_chr07	7089333	[A/G]	1067301661
Ca_Kabuli_NC_021166.1_7089359	Ca_kabuli_chr07	7089359	[A/C]	1067301662
Ca_Kabuli_NC_021166.1_7127510	Ca_kabuli_chr07	7127510	[A/C]	1067301663
Ca_Kabuli_NC_021166.1_7127511	Ca_kabuli_chr07	7127511	[G/T]	1067301664
Ca_Kabuli_NC_021166.1_7139175	Ca_kabuli_chr07	7139175	[A/C]	1067301665
Ca_Kabuli_NC_021166.1_7139176	Ca_kabuli_chr07	7139176	[G/C]	1067301666
Ca_Kabuli_NC_021166.1_7139178	Ca_kabuli_chr07	7139178	[T/A]	1067301667
Ca_Kabuli_NC_021166.1_7146159	Ca_kabuli_chr07	7146159	[G/C]	1067301668
Ca_Kabuli_NC_021166.1_7234446	Ca_kabuli_chr07	7234446	[T/C]	1067301669
Ca_Kabuli_NC_021166.1_7234489	Ca_kabuli_chr07	7234489	[A/G]	1067301670
Ca_Kabuli_NC_021166.1_7261768	Ca_kabuli_chr07	7261768	[T/G]	1067301671
Ca_Kabuli_NC_021166.1_7279735	Ca_kabuli_chr07	7279735	[C/A]	1067301672
Ca_Kabuli_NC_021166.1_7279877	Ca_kabuli_chr07	7279877	[A/C]	1067301673
Ca_Kabuli_NC_021166.1_7304617	Ca_kabuli_chr07	7304617	[T/G]	1067301674
Ca_Kabuli_NC_021166.1_7304695	Ca_kabuli_chr07	7304695	[A/C]	1067301675
Ca_Kabuli_NC_021166.1_7327933	Ca_kabuli_chr07	7327933	[C/A]	1067301676
Ca_Kabuli_NC_021166.1_7327934	Ca_kabuli_chr07	7327934	[T/G]	1067301677
Ca_Kabuli_NC_021166.1_7391559	Ca_kabuli_chr07	7391559	[T/C]	1067301678
Ca_Kabuli_NC_021166.1_7391563	Ca_kabuli_chr07	7391563	[A/G]	1067301679
Ca_Kabuli_NC_021166.1_7391570	Ca_kabuli_chr07	7391570	[A/G]	1067301680
Ca_Kabuli_NC_021166.1_7423307	Ca_kabuli_chr07	7423307	[A/C]	1067301681
Ca_Kabuli_NC_021166.1_7423364	Ca_kabuli_chr07	7423364	[T/C]	1067301682
Ca_Kabuli_NC_021166.1_7423378	Ca_kabuli_chr07	7423378	[A/G]	1067301683
Ca_Kabuli_NC_021166.1_7423407	Ca_kabuli_chr07	7423407	[A/G]	1067301684
Ca_Kabuli_NC_021166.1_7429966	Ca_kabuli_chr07	7429966	[A/G]	1067301685
Ca_Kabuli_NC_021166.1_7433486	Ca_kabuli_chr07	7433486	[T/C]	1067301686

SNP IDs	Chromosomes	Physical positions (bp)	SNPs	NCBI SNPdb accession IDs (NCBI_ss#)
Ca_Kabuli_NC_021166.1_7433488	Ca_kabuli_chr07	7433488	[A/G]	1067301687
Ca_Kabuli_NC_021166.1_7433524	Ca_kabuli_chr07	7433524	[T/A]	1067301688
Ca_Kabuli_NC_021166.1_7433527	Ca_kabuli_chr07	7433527	[A/G]	1067301689
Ca_Kabuli_NC_021166.1_7433548	Ca_kabuli_chr07	7433548	[T/A]	1067301690
Ca_Kabuli_NC_021166.1_7433662	Ca_kabuli_chr07	7433662	[A/G]	1067301691
Ca_Kabuli_NC_021166.1_7433666	Ca_kabuli_chr07	7433666	[G/A]	1067301692
Ca_Kabuli_NC_021166.1_7433827	Ca_kabuli_chr07	7433827	[A/C]	1067301693
Ca_Kabuli_NC_021166.1_7435418	Ca_kabuli_chr07	7435418	[C/T]	1067301694
Ca_Kabuli_NC_021166.1_7435420	Ca_kabuli_chr07	7435420	[A/G]	1067301695
Ca_Kabuli_NC_021166.1_7435434	Ca_kabuli_chr07	7435434	[T/A]	1067301696
Ca_Kabuli_NC_021166.1_7435456	Ca_kabuli_chr07	7435456	[T/C]	1067301697
Ca_Kabuli_NC_021166.1_7435462	Ca_kabuli_chr07	7435462	[T/C]	1067301698
Ca_Kabuli_NC_021166.1_7441872	Ca_kabuli_chr07	7441872	[A/T]	1067301699
Ca_Kabuli_NC_021166.1_7462028	Ca_kabuli_chr07	7462028	[A/T]	1067301700
Ca_Kabuli_NC_021166.1_7462499	Ca_kabuli_chr07	7462499	[A/C]	1067301701
Ca_Kabuli_NC_021166.1_7528873	Ca_kabuli_chr07	7528873	[T/C]	1067301702
Ca_Kabuli_NC_021166.1_7528916	Ca_kabuli_chr07	7528916	[A/C]	1067301703
Ca_Kabuli_NC_021166.1_7579579	Ca_kabuli_chr07	7579579	[A/C]	1067301704
Ca_Kabuli_NC_021166.1_7580147	Ca_kabuli_chr07	7580147	[C/T]	1067301705
Ca_Kabuli_NC_021166.1_7580165	Ca_kabuli_chr07	7580165	[A/G]	1067301706
Ca_Kabuli_NC_021166.1_7580175	Ca_kabuli_chr07	7580175	[T/C]	1067301707
Ca_Kabuli_NC_021166.1_7585001	Ca_kabuli_chr07	7585001	[G/T]	1067301708
Ca_Kabuli_NC_021166.1_7586244	Ca_kabuli_chr07	7586244	[A/C]	1067301709
Ca_Kabuli_NC_021166.1_7586245	Ca_kabuli_chr07	7586245	[C/T]	1067301710
Ca_Kabuli_NC_021166.1_7586408	Ca_kabuli_chr07	7586408	[T/C]	1067301711
Ca_Kabuli_NC_021166.1_7586487	Ca_kabuli_chr07	7586487	[A/C]	1067301712
Ca_Kabuli_NC_021166.1_7591606	Ca_kabuli_chr07	7591606	[A/T]	1067301713
Ca_Kabuli_NC_021166.1_7591628	Ca_kabuli_chr07	7591628	[A/G]	1067301714
Ca_Kabuli_NC_021166.1_7602554	Ca_kabuli_chr07	7602554	[T/C]	1067301715
Ca_Kabuli_NC_021166.1_7602564	Ca_kabuli_chr07	7602564	[T/A]	1067301716
Ca_Kabuli_NC_021166.1_7625185	Ca_kabuli_chr07	7625185	[T/C]	1067301717
Ca_Kabuli_NC_021166.1_7625252	Ca_kabuli_chr07	7625252	[A/G]	1067301718
Ca_Kabuli_NC_021166.1_7625255	Ca_kabuli_chr07	7625255	[A/C]	1067301719
Ca_Kabuli_NC_021166.1_7625258	Ca_kabuli_chr07	7625258	[A/G]	1067301720

SNP IDs	Chromosomes	Physical positions (bp)	SNPs	NCBI SNPdb accession IDs (NCBI_ss#)
Ca_Kabuli_NC_021166.1_7647175	Ca_kabuli_chr07	7647175	[A/G]	1067301721
Ca_Kabuli_NC_021166.1_7665938	Ca_kabuli_chr07	7665938	[T/C]	1067301722
Ca_Kabuli_NC_021166.1_7697092	Ca_kabuli_chr07	7697092	[A/C]	1067301723
Ca_Kabuli_NC_021166.1_7737688	Ca_kabuli_chr07	7737688	[C/A]	1067301724
Ca_Kabuli_NC_021166.1_7737689	Ca_kabuli_chr07	7737689	[T/G]	1067301725
Ca_Kabuli_NC_021166.1_7737899	Ca_kabuli_chr07	7737899	[C/T]	1067301726
Ca_Kabuli_NC_021166.1_7756487	Ca_kabuli_chr07	7756487	[G/A]	1067301727
Ca_Kabuli_NC_021166.1_7756513	Ca_kabuli_chr07	7756513	[A/G]	1067301728
Ca_Kabuli_NC_021166.1_7756517	Ca_kabuli_chr07	7756517	[A/G]	1067301729
Ca_Kabuli_NC_021166.1_7756553	Ca_kabuli_chr07	7756553	[T/C]	1067301730
Ca_Kabuli_NC_021166.1_7792711	Ca_kabuli_chr07	7792711	[A/G]	1067301731
Ca_Kabuli_NC_021166.1_7792722	Ca_kabuli_chr07	7792722	[A/C]	1067301732
Ca_Kabuli_NC_021166.1_7792748	Ca_kabuli_chr07	7792748	[T/G]	1067301733
Ca_Kabuli_NC_021166.1_7809179	Ca_kabuli_chr07	7809179	[T/G]	1067301734
Ca_Kabuli_NC_021166.1_7982985	Ca_kabuli_chr07	7982985	[T/C]	1067301735
Ca_Kabuli_NC_021166.1_7983630	Ca_kabuli_chr07	7983630	[C/A]	1067301736
Ca_Kabuli_NC_021166.1_7983631	Ca_kabuli_chr07	7983631	[T/G]	1067301737
Ca_Kabuli_NC_021166.1_7983632	Ca_kabuli_chr07	7983632	[T/A]	1067301738
Ca_Kabuli_NC_021166.1_7983786	Ca_kabuli_chr07	7983786	[T/C]	1067301739
Ca_Kabuli_NC_021166.1_7983793	Ca_kabuli_chr07	7983793	[T/C]	1067301740
Ca_Kabuli_NC_021166.1_7983794	Ca_kabuli_chr07	7983794	[G/T]	1067301741
Ca_Kabuli_NC_021166.1_8007992	Ca_kabuli_chr07	8007992	[G/T]	1067301742
Ca_Kabuli_NC_021166.1_8008582	Ca_kabuli_chr07	8008582	[T/A]	1067301743
Ca_Kabuli_NC_021166.1_8008612	Ca_kabuli_chr07	8008612	[T/G]	1067301744
Ca_Kabuli_NC_021166.1_8041480	Ca_kabuli_chr07	8041480	[T/G]	1067301745
Ca_Kabuli_NC_021166.1_8111699	Ca_kabuli_chr07	8111699	[T/G]	1067301746
Ca_Kabuli_NC_021166.1_8111705	Ca_kabuli_chr07	8111705	[T/G]	1067301747
Ca_Kabuli_NC_021166.1_8126245	Ca_kabuli_chr07	8126245	[T/G]	1067301748
Ca_Kabuli_NC_021166.1_8144402	Ca_kabuli_chr07	8144402	[A/G]	1067301749
Ca_Kabuli_NC_021166.1_8179346	Ca_kabuli_chr07	8179346	[T/A]	1067301750
Ca_Kabuli_NC_021166.1_8209773	Ca_kabuli_chr07	8209773	[T/A]	1067301751
Ca_Kabuli_NC_021166.1_8249753	Ca_kabuli_chr07	8249753	[T/A]	1067301752
Ca_Kabuli_NC_021166.1_8287879	Ca_kabuli_chr07	8287879	[T/C]	1067301753
Ca_Kabuli_NC_021166.1_8312550	Ca_kabuli_chr07	8312550	[A/C]	1067301754

SNP IDs	Chromosomes	Physical positions (bp)	SNPs	NCBI SNPdb accession IDs (NCBI_ss#)
Ca_Kabuli_NC_021166.1_8312551	Ca_kabuli_chr07	8312551	[G/T]	1067301755
Ca_Kabuli_NC_021166.1_8312552	Ca_kabuli_chr07	8312552	[A/C]	1067301756
Ca_Kabuli_NC_021166.1_8415797	Ca_kabuli_chr07	8415797	[T/C]	1067301757
Ca_Kabuli_NC_021166.1_8415821	Ca_kabuli_chr07	8415821	[A/G]	1067301758
Ca_Kabuli_NC_021166.1_8415830	Ca_kabuli_chr07	8415830	[T/G]	1067301759
Ca_Kabuli_NC_021166.1_8415911	Ca_kabuli_chr07	8415911	[T/C]	1067301760
Ca_Kabuli_NC_021166.1_8448598	Ca_kabuli_chr07	8448598	[A/C]	1067301761
Ca_Kabuli_NC_021166.1_8448607	Ca_kabuli_chr07	8448607	[A/C]	1067301762
Ca_Kabuli_NC_021166.1_8448609	Ca_kabuli_chr07	8448609	[T/C]	1067301763
Ca_Kabuli_NC_021166.1_8514954	Ca_kabuli_chr07	8514954	[A/C]	1067301764
Ca_Kabuli_NC_021166.1_8514973	Ca_kabuli_chr07	8514973	[A/G]	1067301765
Ca_Kabuli_NC_021166.1_8626887	Ca_kabuli_chr07	8626887	[A/G]	1067301766
Ca_Kabuli_NC_021166.1_8742898	Ca_kabuli_chr07	8742898	[C/A]	1067301767
Ca_Kabuli_NC_021166.1_8742899	Ca_kabuli_chr07	8742899	[T/G]	1067301768
Ca_Kabuli_NC_021166.1_8770624	Ca_kabuli_chr07	8770624	[T/G]	1067301769
Ca_Kabuli_NC_021166.1_8770649	Ca_kabuli_chr07	8770649	[T/G]	1067301770
Ca_Kabuli_NC_021166.1_8786830	Ca_kabuli_chr07	8786830	[T/G]	1067301771
Ca_Kabuli_NC_021166.1_8795201	Ca_kabuli_chr07	8795201	[A/C]	1067301772
Ca_Kabuli_NC_021166.1_8795202	Ca_kabuli_chr07	8795202	[G/T]	1067301773
Ca_Kabuli_NC_021166.1_8795203	Ca_kabuli_chr07	8795203	[A/T]	1067301774
Ca_Kabuli_NC_021166.1_8795204	Ca_kabuli_chr07	8795204	[T/G]	1067301775
Ca_Kabuli_NC_021166.1_8802374	Ca_kabuli_chr07	8802374	[A/C]	1067301776
Ca_Kabuli_NC_021166.1_8802395	Ca_kabuli_chr07	8802395	[T/C]	1067301777
Ca_Kabuli_NC_021166.1_8802419	Ca_kabuli_chr07	8802419	[A/C]	1067301778
Ca_Kabuli_NC_021166.1_8815968	Ca_kabuli_chr07	8815968	[A/T]	1067301779
Ca_Kabuli_NC_021166.1_8829226	Ca_kabuli_chr07	8829226	[A/G]	1067301780
Ca_Kabuli_NC_021166.1_8829228	Ca_kabuli_chr07	8829228	[A/G]	1067301781
Ca_Kabuli_NC_021166.1_8829241	Ca_kabuli_chr07	8829241	[A/G]	1067301782
Ca_Kabuli_NC_021166.1_8830831	Ca_kabuli_chr07	8830831	[C/A]	1067301783
Ca_Kabuli_NC_021166.1_8831068	Ca_kabuli_chr07	8831068	[A/C]	1067301784
Ca_Kabuli_NC_021166.1_8835757	Ca_kabuli_chr07	8835757	[C/T]	1067301785
Ca_Kabuli_NC_021166.1_8848919	Ca_kabuli_chr07	8848919	[C/A]	1067301786
Ca_Kabuli_NC_021166.1_8848940	Ca_kabuli_chr07	8848940	[A/C]	1067301787
Ca_Kabuli_NC_021166.1_8894281	Ca_kabuli_chr07	8894281	[T/G]	1067301788

SNP IDs	Chromosomes	Physical positions (bp)	SNPs	NCBI SNPdb accession IDs (NCBI_ss#)
Ca_Kabuli_NC_021166.1_8895101	Ca_kabuli_chr07	8895101	[A/G]	1067301789
Ca_Kabuli_NC_021166.1_8895504	Ca_kabuli_chr07	8895504	[T/C]	1067301790
Ca_Kabuli_NC_021166.1_8908908	Ca_kabuli_chr07	8908908	[A/G]	1067301791
Ca_Kabuli_NC_021166.1_8908962	Ca_kabuli_chr07	8908962	[T/G]	1067301792
Ca_Kabuli_NC_021166.1_8909006	Ca_kabuli_chr07	8909006	[A/C]	1067301793
Ca_Kabuli_NC_021166.1_8909018	Ca_kabuli_chr07	8909018	[A/G]	1067301794
Ca_Kabuli_NC_021166.1_8937279	Ca_kabuli_chr07	8937279	[T/A]	1067301795
Ca_Kabuli_NC_021166.1_8937338	Ca_kabuli_chr07	8937338	[G/A]	1067301796
Ca_Kabuli_NC_021166.1_8937339	Ca_kabuli_chr07	8937339	[G/T]	1067301797
Ca_Kabuli_NC_021166.1_8937340	Ca_kabuli_chr07	8937340	[T/C]	1067301798
Ca_Kabuli_NC_021166.1_8937341	Ca_kabuli_chr07	8937341	[G/T]	1067301799
Ca_Kabuli_NC_021166.1_8937376	Ca_kabuli_chr07	8937376	[A/C]	1067301800
Ca_Kabuli_NC_021166.1_9099718	Ca_kabuli_chr07	9099718	[T/C]	1067301801
Ca_Kabuli_NC_021166.1_9230842	Ca_kabuli_chr07	9230842	[T/G]	1067301802
Ca_Kabuli_NC_021166.1_9281257	Ca_kabuli_chr07	9281257	[G/T]	1067301803
Ca_Kabuli_NC_021166.1_9301828	Ca_kabuli_chr07	9301828	[T/G]	1067301804
Ca_Kabuli_NC_021166.1_9348417	Ca_kabuli_chr07	9348417	[A/T]	1067301805
Ca_Kabuli_NC_021166.1_9416921	Ca_kabuli_chr07	9416921	[A/G]	1067301806
Ca_Kabuli_NC_021166.1_9497129	Ca_kabuli_chr07	9497129	[T/G]	1067301807
Ca_Kabuli_NC_021166.1_9535837	Ca_kabuli_chr07	9535837	[T/C]	1067301808
Ca_Kabuli_NC_021166.1_9535839	Ca_kabuli_chr07	9535839	[T/C]	1067301809
Ca_Kabuli_NC_021166.1_9535841	Ca_kabuli_chr07	9535841	[A/G]	1067301810
Ca_Kabuli_NC_021166.1_9535854	Ca_kabuli_chr07	9535854	[T/C]	1067301811
Ca_Kabuli_NC_021166.1_9535903	Ca_kabuli_chr07	9535903	[A/C]	1067301812
Ca_Kabuli_NC_021166.1_9535946	Ca_kabuli_chr07	9535946	[T/G]	1067301813
Ca_Kabuli_NC_021166.1_9549867	Ca_kabuli_chr07	9549867	[T/G]	1067301814
Ca_Kabuli_NC_021166.1_9550255	Ca_kabuli_chr07	9550255	[A/G]	1067301815
Ca_Kabuli_NC_021166.1_9555286	Ca_kabuli_chr07	9555286	[T/G]	1067301816
Ca_Kabuli_NC_021166.1_9555334	Ca_kabuli_chr07	9555334	[A/T]	1067301817
Ca_Kabuli_NC_021166.1_9593632	Ca_kabuli_chr07	9593632	[A/C]	1067301818
Ca_Kabuli_NC_021166.1_9593662	Ca_kabuli_chr07	9593662	[T/C]	1067301819
Ca_Kabuli_NC_021166.1_9593670	Ca_kabuli_chr07	9593670	[T/A]	1067301820
Ca_Kabuli_NC_021166.1_9762903	Ca_kabuli_chr07	9762903	[A/C]	1067301821
Ca_Kabuli_NC_021166.1_9762943	Ca_kabuli_chr07	9762943	[T/C]	1067301822

SNP IDs	Chromosomes	Physical positions (bp)	SNPs	NCBI SNPdb accession IDs (NCBI_ss#)
Ca_Kabuli_NC_021166.1_9888225	Ca_kabuli_chr07	9888225	[T/A]	1067301823
Ca_Kabuli_NC_021166.1_9888238	Ca_kabuli_chr07	9888238	[C/G]	1067301824
Ca_Kabuli_NC_021166.1_9888271	Ca_kabuli_chr07	9888271	[T/C]	1067301825
Ca_Kabuli_NC_021166.1_9888279	Ca_kabuli_chr07	9888279	[T/C]	1067301826
Ca_Kabuli_NC_021166.1_9909949	Ca_kabuli_chr07	9909949	[C/A]	1067301827
Ca_Kabuli_NC_021166.1_9909951	Ca_kabuli_chr07	9909951	[G/A]	1067301828
Ca_Kabuli_NC_021166.1_9909952	Ca_kabuli_chr07	9909952	[G/T]	1067301829
Ca_Kabuli_NC_021166.1_9936686	Ca_kabuli_chr07	9936686	[G/T]	1067301830
Ca_Kabuli_NC_021166.1_9939036	Ca_kabuli_chr07	9939036	[A/C]	1067301831
Ca_Kabuli_NC_021166.1_10000171	Ca_kabuli_chr07	10000171	[A/C]	1067301832
Ca_Kabuli_NC_021166.1_10001046	Ca_kabuli_chr07	10001046	[A/C]	1067301833
Ca_Kabuli_NC_021166.1_10001047	Ca_kabuli_chr07	10001047	[G/T]	1067301834
Ca_Kabuli_NC_021166.1_10049906	Ca_kabuli_chr07	10049906	[T/G]	1067301835
Ca_Kabuli_NC_021166.1_10137794	Ca_kabuli_chr07	10137794	[T/G]	1067301836
Ca_Kabuli_NC_021166.1_10137810	Ca_kabuli_chr07	10137810	[T/G]	1067301837
Ca_Kabuli_NC_021166.1_10137826	Ca_kabuli_chr07	10137826	[C/A]	1067301838
Ca_Kabuli_NC_021166.1_10180649	Ca_kabuli_chr07	10180649	[A/G]	1067301839
Ca_Kabuli_NC_021166.1_10208210	Ca_kabuli_chr07	10208210	[T/C]	1067301840
Ca_Kabuli_NC_021166.1_10208218	Ca_kabuli_chr07	10208218	[T/C]	1067301841
Ca_Kabuli_NC_021166.1_10216898	Ca_kabuli_chr07	10216898	[A/C]	1067301842
Ca_Kabuli_NC_021166.1_10223027	Ca_kabuli_chr07	10223027	[T/G]	1067301843
Ca_Kabuli_NC_021166.1_10223034	Ca_kabuli_chr07	10223034	[T/G]	1067301844
Ca_Kabuli_NC_021166.1_10223040	Ca_kabuli_chr07	10223040	[A/T]	1067301845
Ca_Kabuli_NC_021166.1_10223047	Ca_kabuli_chr07	10223047	[A/C]	1067301846
Ca_Kabuli_NC_021166.1_10231988	Ca_kabuli_chr07	10231988	[T/G]	1067301847
Ca_Kabuli_NC_021166.1_10232074	Ca_kabuli_chr07	10232074	[A/G]	1067301848
Ca_Kabuli_NC_021166.1_10251052	Ca_kabuli_chr07	10251052	[T/G]	1067301849
Ca_Kabuli_NC_021166.1_10328467	Ca_kabuli_chr07	10328467	[T/G]	1067301850
Ca_Kabuli_NC_021166.1_10328470	Ca_kabuli_chr07	10328470	[T/G]	1067301851
Ca_Kabuli_NC_021166.1_10328516	Ca_kabuli_chr07	10328516	[T/C]	1067301852
Ca_Kabuli_NC_021166.1_10328520	Ca_kabuli_chr07	10328520	[A/C]	1067301853
Ca_Kabuli_NC_021166.1_10328525	Ca_kabuli_chr07	10328525	[A/G]	1067301854
Ca_Kabuli_NC_021166.1_10333895	Ca_kabuli_chr07	10333895	[T/G]	1067301855
Ca_Kabuli_NC_021166.1_10429474	Ca_kabuli_chr07	10429474	[T/G]	1067301856

SNP IDs	Chromosomes	Physical positions (bp)	SNPs	NCBI SNPdb accession IDs (NCBI_ss#)
Ca_Kabuli_NC_021166.1_10465150	Ca_kabuli_chr07	10465150	[T/C]	1067301857
Ca_Kabuli_NC_021166.1_10473639	Ca_kabuli_chr07	10473639	[T/C]	1067301858
Ca_Kabuli_NC_021166.1_10483907	Ca_kabuli_chr07	10483907	[T/C]	1067301859
Ca_Kabuli_NC_021166.1_10485397	Ca_kabuli_chr07	10485397	[C/G]	1067301860
Ca_Kabuli_NC_021166.1_10521343	Ca_kabuli_chr07	10521343	[T/C]	1067301861
Ca_Kabuli_NC_021166.1_10521358	Ca_kabuli_chr07	10521358	[T/C]	1067301862
Ca_Kabuli_NC_021166.1_10521387	Ca_kabuli_chr07	10521387	[A/T]	1067301863
Ca_Kabuli_NC_021166.1_10521395	Ca_kabuli_chr07	10521395	[T/A]	1067301864
Ca_Kabuli_NC_021166.1_10521397	Ca_kabuli_chr07	10521397	[A/G]	1067301865
Ca_Kabuli_NC_021166.1_10521401	Ca_kabuli_chr07	10521401	[T/G]	1067301866
Ca_Kabuli_NC_021166.1_10521419	Ca_kabuli_chr07	10521419	[A/G]	1067301867
Ca_Kabuli_NC_021166.1_10521425	Ca_kabuli_chr07	10521425	[G/A]	1067301868
Ca_Kabuli_NC_021166.1_10521431	Ca_kabuli_chr07	10521431	[G/C]	1067301869
Ca_Kabuli_NC_021166.1_10521438	Ca_kabuli_chr07	10521438	[T/C]	1067301870
Ca_Kabuli_NC_021166.1_10521447	Ca_kabuli_chr07	10521447	[A/G]	1067301871
Ca_Kabuli_NC_021166.1_10521448	Ca_kabuli_chr07	10521448	[G/A]	1067301872
Ca_Kabuli_NC_021166.1_10568128	Ca_kabuli_chr07	10568128	[G/A]	1067301873
Ca_Kabuli_NC_021166.1_10568241	Ca_kabuli_chr07	10568241	[G/C]	1067301874
Ca_Kabuli_NC_021166.1_10614161	Ca_kabuli_chr07	10614161	[T/C]	1067301875
Ca_Kabuli_NC_021166.1_10614177	Ca_kabuli_chr07	10614177	[T/G]	1067301876
Ca_Kabuli_NC_021166.1_10641854	Ca_kabuli_chr07	10641854	[T/C]	1067301877
Ca_Kabuli_NC_021166.1_10649594	Ca_kabuli_chr07	10649594	[T/G]	1067301878
Ca_Kabuli_NC_021166.1_10649622	Ca_kabuli_chr07	10649622	[C/A]	1067301879
Ca_Kabuli_NC_021166.1_10649623	Ca_kabuli_chr07	10649623	[G/C]	1067301880
Ca_Kabuli_NC_021166.1_10720696	Ca_kabuli_chr07	10720696	[A/G]	1067301881
Ca_Kabuli_NC_021166.1_10795939	Ca_kabuli_chr07	10795939	[A/C]	1067301882
Ca_Kabuli_NC_021166.1_10812569	Ca_kabuli_chr07	10812569	[T/G]	1067301883
Ca_Kabuli_NC_021166.1_10816523	Ca_kabuli_chr07	10816523	[T/G]	1067301884
Ca_Kabuli_NC_021166.1_10900762	Ca_kabuli_chr07	10900762	[C/G]	1067301885
Ca_Kabuli_NC_021166.1_10900767	Ca_kabuli_chr07	10900767	[T/C]	1067301886
Ca_Kabuli_NC_021166.1_11033768	Ca_kabuli_chr07	11033768	[T/C]	1067301887
Ca_Kabuli_NC_021166.1_11033771	Ca_kabuli_chr07	11033771	[T/C]	1067301888
Ca_Kabuli_NC_021166.1_11192688	Ca_kabuli_chr07	11192688	[A/C]	1067301889
Ca_Kabuli_NC_021166.1_11192691	Ca_kabuli_chr07	11192691	[A/C]	1067301890

SNP IDs	Chromosomes	Physical positions (bp)	SNPs	NCBI SNPdb accession IDs (NCBI_ss#)
Ca_Kabuli_NC_021166.1_11192694	Ca_kabuli_chr07	11192694	[A/C]	1067301891
Ca_Kabuli_NC_021166.1_11192703	Ca_kabuli_chr07	11192703	[A/C]	1067301892
Ca_Kabuli_NC_021166.1_11192706	Ca_kabuli_chr07	11192706	[A/C]	1067301893
Ca_Kabuli_NC_021166.1_11383745	Ca_kabuli_chr07	11383745	[T/G]	1067301894
Ca_Kabuli_NC_021166.1_11383746	Ca_kabuli_chr07	11383746	[G/T]	1067301895
Ca_Kabuli_NC_021166.1_11383747	Ca_kabuli_chr07	11383747	[T/G]	1067301896
Ca_Kabuli_NC_021166.1_11383749	Ca_kabuli_chr07	11383749	[T/G]	1067301897
Ca_Kabuli_NC_021166.1_11383752	Ca_kabuli_chr07	11383752	[T/G]	1067301898
Ca_Kabuli_NC_021166.1_11417659	Ca_kabuli_chr07	11417659	[A/T]	1067301899
Ca_Kabuli_NC_021166.1_11468374	Ca_kabuli_chr07	11468374	[A/G]	1067301900
Ca_Kabuli_NC_021166.1_11521683	Ca_kabuli_chr07	11521683	[A/T]	1067301901
Ca_Kabuli_NC_021166.1_11521711	Ca_kabuli_chr07	11521711	[A/T]	1067301902
Ca_Kabuli_NC_021166.1_11545804	Ca_kabuli_chr07	11545804	[T/C]	1067301903
Ca_Kabuli_NC_021166.1_11545835	Ca_kabuli_chr07	11545835	[T/G]	1067301904
Ca_Kabuli_NC_021166.1_11545870	Ca_kabuli_chr07	11545870	[A/G]	1067301905
Ca_Kabuli_NC_021166.1_11545877	Ca_kabuli_chr07	11545877	[A/G]	1067301906
Ca_Kabuli_NC_021166.1_11630434	Ca_kabuli_chr07	11630434	[T/C]	1067301907
Ca_Kabuli_NC_021166.1_11630463	Ca_kabuli_chr07	11630463	[G/A]	1067301908
Ca_Kabuli_NC_021166.1_11630465	Ca_kabuli_chr07	11630465	[A/G]	1067301909
Ca_Kabuli_NC_021166.1_11638847	Ca_kabuli_chr07	11638847	[A/C]	1067301910
Ca_Kabuli_NC_021166.1_11685190	Ca_kabuli_chr07	11685190	[C/A]	1067301911
Ca_Kabuli_NC_021166.1_11685194	Ca_kabuli_chr07	11685194	[T/G]	1067301912
Ca_Kabuli_NC_021166.1_11686223	Ca_kabuli_chr07	11686223	[T/G]	1067301913
Ca_Kabuli_NC_021166.1_11725595	Ca_kabuli_chr07	11725595	[C/T]	1067301914
Ca_Kabuli_NC_021166.1_11734856	Ca_kabuli_chr07	11734856	[A/G]	1067301915
Ca_Kabuli_NC_021166.1_11734862	Ca_kabuli_chr07	11734862	[A/G]	1067301916
Ca_Kabuli_NC_021166.1_11734873	Ca_kabuli_chr07	11734873	[T/A]	1067301917
Ca_Kabuli_NC_021166.1_11734891	Ca_kabuli_chr07	11734891	[A/C]	1067301918
Ca_Kabuli_NC_021166.1_11734893	Ca_kabuli_chr07	11734893	[T/C]	1067301919
Ca_Kabuli_NC_021166.1_11734905	Ca_kabuli_chr07	11734905	[A/T]	1067301920
Ca_Kabuli_NC_021166.1_11734917	Ca_kabuli_chr07	11734917	[T/A]	1067301921
Ca_Kabuli_NC_021166.1_11736066	Ca_kabuli_chr07	11736066	[A/G]	1067301922
Ca_Kabuli_NC_021166.1_11791921	Ca_kabuli_chr07	11791921	[T/C]	1067301923
Ca_Kabuli_NC_021166.1_11812468	Ca_kabuli_chr07	11812468	[A/C]	1067301924

SNP IDs	Chromosomes	Physical positions (bp)	SNPs	NCBI SNPdb accession IDs (NCBI_ss#)
Ca_Kabuli_NC_021166.1_12067234	Ca_kabuli_chr07	12067234	[C/T]	1067301925
Ca_Kabuli_NC_021166.1_12071674	Ca_kabuli_chr07	12071674	[C/T]	1067301926
Ca_Kabuli_NC_021166.1_12071679	Ca_kabuli_chr07	12071679	[A/C]	1067301927
Ca_Kabuli_NC_021166.1_12073249	Ca_kabuli_chr07	12073249	[C/A]	1067301928
Ca_Kabuli_NC_021166.1_12073250	Ca_kabuli_chr07	12073250	[T/G]	1067301929
Ca_Kabuli_NC_021166.1_12073251	Ca_kabuli_chr07	12073251	[G/A]	1067301930
Ca_Kabuli_NC_021166.1_12073252	Ca_kabuli_chr07	12073252	[A/T]	1067301931
Ca_Kabuli_NC_021166.1_12124282	Ca_kabuli_chr07	12124282	[T/C]	1067301932
Ca_Kabuli_NC_021166.1_12158807	Ca_kabuli_chr07	12158807	[T/A]	1067301933
Ca_Kabuli_NC_021166.1_12161451	Ca_kabuli_chr07	12161451	[G/A]	1067301934
Ca_Kabuli_NC_021166.1_12333200	Ca_kabuli_chr07	12333200	[T/G]	1067301935
Ca_Kabuli_NC_021166.1_12366703	Ca_kabuli_chr07	12366703	[T/C]	1067301936
Ca_Kabuli_NC_021166.1_12366732	Ca_kabuli_chr07	12366732	[A/G]	1067301937
Ca_Kabuli_NC_021166.1_12384067	Ca_kabuli_chr07	12384067	[T/C]	1067301938
Ca_Kabuli_NC_021166.1_12390754	Ca_kabuli_chr07	12390754	[C/T]	1067301939
Ca_Kabuli_NC_021166.1_12390774	Ca_kabuli_chr07	12390774	[A/G]	1067301940
Ca_Kabuli_NC_021166.1_12429249	Ca_kabuli_chr07	12429249	[T/G]	1067301941
Ca_Kabuli_NC_021166.1_12474009	Ca_kabuli_chr07	12474009	[T/G]	1067301942
Ca_Kabuli_NC_021166.1_12482742	Ca_kabuli_chr07	12482742	[A/T]	1067301943
Ca_Kabuli_NC_021166.1_12483195	Ca_kabuli_chr07	12483195	[G/T]	1067301944
Ca_Kabuli_NC_021166.1_12492059	Ca_kabuli_chr07	12492059	[A/G]	1067301945
Ca_Kabuli_NC_021166.1_12492062	Ca_kabuli_chr07	12492062	[C/G]	1067301946
Ca_Kabuli_NC_021166.1_12492063	Ca_kabuli_chr07	12492063	[C/A]	1067301947
Ca_Kabuli_NC_021166.1_12492064	Ca_kabuli_chr07	12492064	[A/G]	1067301948
Ca_Kabuli_NC_021166.1_12492065	Ca_kabuli_chr07	12492065	[C/G]	1067301949
Ca_Kabuli_NC_021166.1_12492074	Ca_kabuli_chr07	12492074	[T/C]	1067301950
Ca_Kabuli_NC_021166.1_12492082	Ca_kabuli_chr07	12492082	[T/C]	1067301951
Ca_Kabuli_NC_021166.1_12590541	Ca_kabuli_chr07	12590541	[T/C]	1067301952
Ca_Kabuli_NC_021166.1_12614558	Ca_kabuli_chr07	12614558	[T/G]	1067301953
Ca_Kabuli_NC_021166.1_12628067	Ca_kabuli_chr07	12628067	[T/C]	1067301954
Ca_Kabuli_NC_021166.1_12628071	Ca_kabuli_chr07	12628071	[A/C]	1067301955
Ca_Kabuli_NC_021166.1_12628072	Ca_kabuli_chr07	12628072	[G/T]	1067301956
Ca_Kabuli_NC_021166.1_12629944	Ca_kabuli_chr07	12629944	[A/C]	1067301957
Ca_Kabuli_NC_021166.1_12635956	Ca_kabuli_chr07	12635956	[A/C]	1067301958

SNP IDs	Chromosomes	Physical positions (bp)	SNPs	NCBI SNPdb accession IDs (NCBI_ss#)
Ca_Kabuli_NC_021166.1_12681460	Ca_kabuli_chr07	12681460	[A/G]	1067301959
Ca_Kabuli_NC_021166.1_12681484	Ca_kabuli_chr07	12681484	[T/A]	1067301960
Ca_Kabuli_NC_021166.1_12681494	Ca_kabuli_chr07	12681494	[A/T]	1067301961
Ca_Kabuli_NC_021166.1_12681506	Ca_kabuli_chr07	12681506	[A/G]	1067301962
Ca_Kabuli_NC_021166.1_12692636	Ca_kabuli_chr07	12692636	[G/T]	1067301963
Ca_Kabuli_NC_021166.1_12730576	Ca_kabuli_chr07	12730576	[T/A]	1067301964
Ca_Kabuli_NC_021166.1_12764977	Ca_kabuli_chr07	12764977	[T/C]	1067301965
Ca_Kabuli_NC_021166.1_12765048	Ca_kabuli_chr07	12765048	[A/G]	1067301966
Ca_Kabuli_NC_021166.1_12847806	Ca_kabuli_chr07	12847806	[G/A]	1067301967
Ca_Kabuli_NC_021166.1_12892797	Ca_kabuli_chr07	12892797	[T/G]	1067301968
Ca_Kabuli_NC_021166.1_13021492	Ca_kabuli_chr07	13021492	[T/G]	1067301969
Ca_Kabuli_NC_021166.1_13021528	Ca_kabuli_chr07	13021528	[A/T]	1067301970
Ca_Kabuli_NC_021166.1_13046694	Ca_kabuli_chr07	13046694	[T/C]	1067301971
Ca_Kabuli_NC_021166.1_13046697	Ca_kabuli_chr07	13046697	[T/C]	1067301972
Ca_Kabuli_NC_021166.1_13059684	Ca_kabuli_chr07	13059684	[T/G]	1067301973
Ca_Kabuli_NC_021166.1_13059757	Ca_kabuli_chr07	13059757	[T/G]	1067301974
Ca_Kabuli_NC_021166.1_13059857	Ca_kabuli_chr07	13059857	[T/G]	1067301975
Ca_Kabuli_NC_021166.1_13069803	Ca_kabuli_chr07	13069803	[T/G]	1067301976
Ca_Kabuli_NC_021166.1_13180380	Ca_kabuli_chr07	13180380	[A/G]	1067301977
Ca_Kabuli_NC_021166.1_13215942	Ca_kabuli_chr07	13215942	[T/G]	1067301978
Ca_Kabuli_NC_021166.1_13220226	Ca_kabuli_chr07	13220226	[A/C]	1067301979
Ca_Kabuli_NC_021166.1_13220242	Ca_kabuli_chr07	13220242	[T/A]	1067301980
Ca_Kabuli_NC_021166.1_13220460	Ca_kabuli_chr07	13220460	[A/T]	1067301981
Ca_Kabuli_NC_021166.1_13288666	Ca_kabuli_chr07	13288666	[T/C]	1067301982
Ca_Kabuli_NC_021166.1_13288777	Ca_kabuli_chr07	13288777	[T/G]	1067301983
Ca_Kabuli_NC_021166.1_13319895	Ca_kabuli_chr07	13319895	[A/T]	1067301984
Ca_Kabuli_NC_021166.1_13481929	Ca_kabuli_chr07	13481929	[T/G]	1067301985
Ca_Kabuli_NC_021166.1_13514061	Ca_kabuli_chr07	13514061	[A/C]	1067301986
Ca_Kabuli_NC_021166.1_13514076	Ca_kabuli_chr07	13514076	[A/G]	1067301987
Ca_Kabuli_NC_021166.1_13514220	Ca_kabuli_chr07	13514220	[A/G]	1067301988
Ca_Kabuli_NC_021166.1_13514244	Ca_kabuli_chr07	13514244	[T/C]	1067301989
Ca_Kabuli_NC_021166.1_13580931	Ca_kabuli_chr07	13580931	[A/C]	1067301990
Ca_Kabuli_NC_021166.1_13580932	Ca_kabuli_chr07	13580932	[G/C]	1067301991
Ca_Kabuli_NC_021166.1_13580933	Ca_kabuli_chr07	13580933	[A/T]	1067301992

SNP IDs	Chromosomes	Physical positions (bp)	SNPs	NCBI SNPdb accession IDs (NCBI_ss#)
Ca_Kabuli_NC_021166.1_13580934	Ca_kabuli_chr07	13580934	[T/G]	1067301993
Ca_Kabuli_NC_021166.1_13581138	Ca_kabuli_chr07	13581138	[A/C]	1067301994
Ca_Kabuli_NC_021166.1_13581140	Ca_kabuli_chr07	13581140	[T/C]	1067301995
Ca_Kabuli_NC_021166.1_13581160	Ca_kabuli_chr07	13581160	[A/G]	1067301996
Ca_Kabuli_NC_021166.1_13581215	Ca_kabuli_chr07	13581215	[A/G]	1067301997
Ca_Kabuli_NC_021166.1_13581218	Ca_kabuli_chr07	13581218	[A/C]	1067301998
Ca_Kabuli_NC_021166.1_13581221	Ca_kabuli_chr07	13581221	[A/G]	1067301999
Ca_Kabuli_NC_021166.1_13581223	Ca_kabuli_chr07	13581223	[A/G]	1067302000
Ca_Kabuli_NC_021166.1_13581231	Ca_kabuli_chr07	13581231	[T/A]	1067302001
Ca_Kabuli_NC_021166.1_13581821	Ca_kabuli_chr07	13581821	[T/C]	1067302002
Ca_Kabuli_NC_021166.1_13581823	Ca_kabuli_chr07	13581823	[A/T]	1067302003
Ca_Kabuli_NC_021166.1_13605194	Ca_kabuli_chr07	13605194	[A/C]	1067302004
Ca_Kabuli_NC_021166.1_13605197	Ca_kabuli_chr07	13605197	[A/C]	1067302005
Ca_Kabuli_NC_021166.1_13605200	Ca_kabuli_chr07	13605200	[A/C]	1067302006
Ca_Kabuli_NC_021166.1_13605208	Ca_kabuli_chr07	13605208	[T/A]	1067302007
Ca_Kabuli_NC_021166.1_13605221	Ca_kabuli_chr07	13605221	[T/C]	1067302008
Ca_Kabuli_NC_021166.1_13667264	Ca_kabuli_chr07	13667264	[T/G]	1067302009
Ca_Kabuli_NC_021166.1_13760302	Ca_kabuli_chr07	13760302	[T/C]	1067302010
Ca_Kabuli_NC_021166.1_13760331	Ca_kabuli_chr07	13760331	[A/G]	1067302011
Ca_Kabuli_NC_021166.1_13776058	Ca_kabuli_chr07	13776058	[T/C]	1067302012
Ca_Kabuli_NC_021166.1_13776107	Ca_kabuli_chr07	13776107	[A/G]	1067302013
Ca_Kabuli_NC_021166.1_13776113	Ca_kabuli_chr07	13776113	[C/G]	1067302014
Ca_Kabuli_NC_021166.1_13781796	Ca_kabuli_chr07	13781796	[T/C]	1067302015
Ca_Kabuli_NC_021166.1_13781799	Ca_kabuli_chr07	13781799	[T/G]	1067302016
Ca_Kabuli_NC_021166.1_13781824	Ca_kabuli_chr07	13781824	[T/C]	1067302017
Ca_Kabuli_NC_021166.1_13819456	Ca_kabuli_chr07	13819456	[T/A]	1067302018
Ca_Kabuli_NC_021166.1_13860705	Ca_kabuli_chr07	13860705	[T/C]	1067302019
Ca_Kabuli_NC_021166.1_13860754	Ca_kabuli_chr07	13860754	[A/C]	1067302020
Ca_Kabuli_NC_021166.1_13904188	Ca_kabuli_chr07	13904188	[T/C]	1067302021
Ca_Kabuli_NC_021166.1_13949259	Ca_kabuli_chr07	13949259	[G/A]	1067302022
Ca_Kabuli_NC_021166.1_13951804	Ca_kabuli_chr07	13951804	[A/G]	1067302023
Ca_Kabuli_NC_021166.1_14133830	Ca_kabuli_chr07	14133830	[G/A]	1067302024
Ca_Kabuli_NC_021166.1_14171041	Ca_kabuli_chr07	14171041	[T/G]	1067302025
Ca_Kabuli_NC_021166.1_14198250	Ca_kabuli_chr07	14198250	[G/C]	1067302026

SNP IDs	Chromosomes	Physical positions (bp)	SNPs	NCBI SNPdb accession IDs (NCBI_ss#)
Ca_Kabuli_NC_021166.1_14198251	Ca_kabuli_chr07	14198251	[A/T]	1067302027
Ca_Kabuli_NC_021166.1_14199535	Ca_kabuli_chr07	14199535	[T/G]	1067302028
Ca_Kabuli_NC_021166.1_14199554	Ca_kabuli_chr07	14199554	[T/G]	1067302029
Ca_Kabuli_NC_021166.1_14282061	Ca_kabuli_chr07	14282061	[A/G]	1067302030
Ca_Kabuli_NC_021166.1_14282154	Ca_kabuli_chr07	14282154	[T/G]	1067302031
Ca_Kabuli_NC_021166.1_14325903	Ca_kabuli_chr07	14325903	[T/A]	1067302032
Ca_Kabuli_NC_021166.1_14325936	Ca_kabuli_chr07	14325936	[T/C]	1067302033
Ca_Kabuli_NC_021166.1_14325948	Ca_kabuli_chr07	14325948	[A/G]	1067302034
Ca_Kabuli_NC_021166.1_14470488	Ca_kabuli_chr07	14470488	[C/G]	1067302035
Ca_Kabuli_NC_021166.1_14470491	Ca_kabuli_chr07	14470491	[A/G]	1067302036
Ca_Kabuli_NC_021166.1_14492362	Ca_kabuli_chr07	14492362	[C/T]	1067302037
Ca_Kabuli_NC_021166.1_14552465	Ca_kabuli_chr07	14552465	[T/C]	1067302038
Ca_Kabuli_NC_021166.1_14556361	Ca_kabuli_chr07	14556361	[T/A]	1067302039
Ca_Kabuli_NC_021166.1_14558899	Ca_kabuli_chr07	14558899	[T/A]	1067302040
Ca_Kabuli_NC_021166.1_14558900	Ca_kabuli_chr07	14558900	[A/C]	1067302041
Ca_Kabuli_NC_021166.1_14685017	Ca_kabuli_chr07	14685017	[C/T]	1067302042
Ca_Kabuli_NC_021166.1_14685023	Ca_kabuli_chr07	14685023	[G/T]	1067302043
Ca_Kabuli_NC_021166.1_14685024	Ca_kabuli_chr07	14685024	[A/G]	1067302044
Ca_Kabuli_NC_021166.1_14776018	Ca_kabuli_chr07	14776018	[T/G]	1067302045
Ca_Kabuli_NC_021166.1_14776041	Ca_kabuli_chr07	14776041	[T/G]	1067302046
Ca_Kabuli_NC_021166.1_14782472	Ca_kabuli_chr07	14782472	[T/G]	1067302047
Ca_Kabuli_NC_021166.1_14782508	Ca_kabuli_chr07	14782508	[A/C]	1067302048
Ca_Kabuli_NC_021166.1_14782512	Ca_kabuli_chr07	14782512	[T/C]	1067302049
Ca_Kabuli_NC_021166.1_14782519	Ca_kabuli_chr07	14782519	[T/C]	1067302050
Ca_Kabuli_NC_021166.1_14782557	Ca_kabuli_chr07	14782557	[T/C]	1067302051
Ca_Kabuli_NC_021166.1_14782559	Ca_kabuli_chr07	14782559	[T/C]	1067302052
Ca_Kabuli_NC_021166.1_14782565	Ca_kabuli_chr07	14782565	[A/T]	1067302053
Ca_Kabuli_NC_021166.1_14782623	Ca_kabuli_chr07	14782623	[A/G]	1067302054
Ca_Kabuli_NC_021166.1_14866783	Ca_kabuli_chr07	14866783	[A/C]	1067302055
Ca_Kabuli_NC_021166.1_14866797	Ca_kabuli_chr07	14866797	[A/G]	1067302056
Ca_Kabuli_NC_021166.1_14899176	Ca_kabuli_chr07	14899176	[T/G]	1067302057
Ca_Kabuli_NC_021166.1_14902205	Ca_kabuli_chr07	14902205	[T/G]	1067302058
Ca_Kabuli_NC_021166.1_14933301	Ca_kabuli_chr07	14933301	[T/C]	1067302059
Ca_Kabuli_NC_021166.1_14933306	Ca_kabuli_chr07	14933306	[A/G]	1067302060

SNP IDs	Chromosomes	Physical positions (bp)	SNPs	NCBI SNPdb accession IDs (NCBI_ss#)
Ca_Kabuli_NC_021166.1_14949948	Ca_kabuli_chr07	14949948	[A/C]	1067302061
Ca_Kabuli_NC_021166.1_15013576	Ca_kabuli_chr07	15013576	[A/C]	1067302062
Ca_Kabuli_NC_021166.1_15013577	Ca_kabuli_chr07	15013577	[G/A]	1067302063
Ca_Kabuli_NC_021166.1_15021703	Ca_kabuli_chr07	15021703	[G/T]	1067302064
Ca_Kabuli_NC_021166.1_15039606	Ca_kabuli_chr07	15039606	[T/A]	1067302065
Ca_Kabuli_NC_021166.1_15097437	Ca_kabuli_chr07	15097437	[A/C]	1067302066
Ca_Kabuli_NC_021166.1_15100152	Ca_kabuli_chr07	15100152	[C/A]	1067302067
Ca_Kabuli_NC_021166.1_15125608	Ca_kabuli_chr07	15125608	[T/A]	1067302068
Ca_Kabuli_NC_021166.1_15125636	Ca_kabuli_chr07	15125636	[T/G]	1067302069
Ca_Kabuli_NC_021166.1_15129131	Ca_kabuli_chr07	15129131	[C/A]	1067302070
Ca_Kabuli_NC_021166.1_15201852	Ca_kabuli_chr07	15201852	[A/T]	1067302071
Ca_Kabuli_NC_021166.1_15233487	Ca_kabuli_chr07	15233487	[T/G]	1067302072
Ca_Kabuli_NC_021166.1_15283180	Ca_kabuli_chr07	15283180	[T/A]	1067302073
Ca_Kabuli_NC_021166.1_15283183	Ca_kabuli_chr07	15283183	[T/A]	1067302074
Ca_Kabuli_NC_021166.1_15309117	Ca_kabuli_chr07	15309117	[A/C]	1067302075
Ca_Kabuli_NC_021166.1_15309177	Ca_kabuli_chr07	15309177	[A/G]	1067302076
Ca_Kabuli_NC_021166.1_15309179	Ca_kabuli_chr07	15309179	[A/C]	1067302077
Ca_Kabuli_NC_021166.1_15621480	Ca_kabuli_chr07	15621480	[T/C]	1067302078
Ca_Kabuli_NC_021166.1_15621499	Ca_kabuli_chr07	15621499	[A/G]	1067302079
Ca_Kabuli_NC_021166.1_15621504	Ca_kabuli_chr07	15621504	[T/G]	1067302080
Ca_Kabuli_NC_021166.1_15621507	Ca_kabuli_chr07	15621507	[T/G]	1067302081
Ca_Kabuli_NC_021166.1_15624675	Ca_kabuli_chr07	15624675	[A/G]	1067302082
Ca_Kabuli_NC_021166.1_15701608	Ca_kabuli_chr07	15701608	[A/G]	1067302083
Ca_Kabuli_NC_021166.1_15740777	Ca_kabuli_chr07	15740777	[A/C]	1067302084
Ca_Kabuli_NC_021166.1_15779226	Ca_kabuli_chr07	15779226	[C/T]	1067302085
Ca_Kabuli_NC_021166.1_15814624	Ca_kabuli_chr07	15814624	[A/C]	1067302086
Ca_Kabuli_NC_021166.1_15887950	Ca_kabuli_chr07	15887950	[C/A]	1067302087
Ca_Kabuli_NC_021166.1_15887951	Ca_kabuli_chr07	15887951	[T/G]	1067302088
Ca_Kabuli_NC_021166.1_15887954	Ca_kabuli_chr07	15887954	[T/G]	1067302089
Ca_Kabuli_NC_021166.1_15918309	Ca_kabuli_chr07	15918309	[A/G]	1067302090
Ca_Kabuli_NC_021166.1_15918336	Ca_kabuli_chr07	15918336	[C/T]	1067302091
Ca_Kabuli_NC_021166.1_15918341	Ca_kabuli_chr07	15918341	[C/T]	1067302092
Ca_Kabuli_NC_021166.1_15918343	Ca_kabuli_chr07	15918343	[A/G]	1067302093
Ca_Kabuli_NC_021166.1_15966666	Ca_kabuli_chr07	15966666	[T/C]	1067302094

SNP IDs	Chromosomes	Physical positions (bp)	SNPs	NCBI SNPdb accession IDs (NCBI_ss#)
Ca_Kabuli_NC_021166.1_15966700	Ca_kabuli_chr07	15966700	[G/T]	1067302095
Ca_Kabuli_NC_021166.1_15966701	Ca_kabuli_chr07	15966701	[A/T]	1067302096
Ca_Kabuli_NC_021166.1_15966762	Ca_kabuli_chr07	15966762	[G/A]	1067302097
Ca_Kabuli_NC_021166.1_15966770	Ca_kabuli_chr07	15966770	[A/T]	1067302098
Ca_Kabuli_NC_021166.1_15966805	Ca_kabuli_chr07	15966805	[C/T]	1067302099
Ca_Kabuli_NC_021166.1_16178928	Ca_kabuli_chr07	16178928	[C/T]	1067302100
Ca_Kabuli_NC_021166.1_16178935	Ca_kabuli_chr07	16178935	[G/T]	1067302101
Ca_Kabuli_NC_021166.1_16268352	Ca_kabuli_chr07	16268352	[G/T]	1067302102
Ca_Kabuli_NC_021166.1_16282987	Ca_kabuli_chr07	16282987	[C/A]	1067302103
Ca_Kabuli_NC_021166.1_16282988	Ca_kabuli_chr07	16282988	[A/G]	1067302104
Ca_Kabuli_NC_021166.1_16282990	Ca_kabuli_chr07	16282990	[C/T]	1067302105
Ca_Kabuli_NC_021166.1_16282991	Ca_kabuli_chr07	16282991	[T/C]	1067302106
Ca_Kabuli_NC_021166.1_16289801	Ca_kabuli_chr07	16289801	[A/C]	1067302107
Ca_Kabuli_NC_021166.1_16381651	Ca_kabuli_chr07	16381651	[A/T]	1067302108
Ca_Kabuli_NC_021166.1_16381880	Ca_kabuli_chr07	16381880	[T/C]	1067302109
Ca_Kabuli_NC_021166.1_16381896	Ca_kabuli_chr07	16381896	[A/G]	1067302110
Ca_Kabuli_NC_021166.1_16466564	Ca_kabuli_chr07	16466564	[C/A]	1067302111
Ca_Kabuli_NC_021166.1_16624827	Ca_kabuli_chr07	16624827	[A/C]	1067302112
Ca_Kabuli_NC_021166.1_16626047	Ca_kabuli_chr07	16626047	[T/G]	1067302113
Ca_Kabuli_NC_021166.1_16626713	Ca_kabuli_chr07	16626713	[T/C]	1067302114
Ca_Kabuli_NC_021166.1_16651454	Ca_kabuli_chr07	16651454	[C/T]	1067302115
Ca_Kabuli_NC_021166.1_16725171	Ca_kabuli_chr07	16725171	[T/C]	1067302116
Ca_Kabuli_NC_021166.1_16725180	Ca_kabuli_chr07	16725180	[T/A]	1067302117
Ca_Kabuli_NC_021166.1_16735401	Ca_kabuli_chr07	16735401	[T/C]	1067302118
Ca_Kabuli_NC_021166.1_16735433	Ca_kabuli_chr07	16735433	[A/G]	1067302119
Ca_Kabuli_NC_021166.1_16735437	Ca_kabuli_chr07	16735437	[G/A]	1067302120
Ca_Kabuli_NC_021166.1_16816263	Ca_kabuli_chr07	16816263	[A/T]	1067302121
Ca_Kabuli_NC_021166.1_16816276	Ca_kabuli_chr07	16816276	[G/A]	1067302122
Ca_Kabuli_NC_021166.1_16816277	Ca_kabuli_chr07	16816277	[A/G]	1067302123
Ca_Kabuli_NC_021166.1_16816308	Ca_kabuli_chr07	16816308	[T/A]	1067302124
Ca_Kabuli_NC_021166.1_16821331	Ca_kabuli_chr07	16821331	[A/G]	1067302125
Ca_Kabuli_NC_021166.1_16821335	Ca_kabuli_chr07	16821335	[A/G]	1067302126
Ca_Kabuli_NC_021166.1_16919612	Ca_kabuli_chr07	16919612	[A/T]	1067302127
Ca_Kabuli_NC_021166.1_16936123	Ca_kabuli_chr07	16936123	[C/A]	1067302128

SNP IDs	Chromosomes	Physical positions (bp)	SNPs	NCBI SNPdb accession IDs (NCBI_ss#)
Ca_Kabuli_NC_021166.1_16936124	Ca_kabuli_chr07	16936124	[T/A]	1067302129
Ca_Kabuli_NC_021166.1_16936139	Ca_kabuli_chr07	16936139	[G/T]	1067302130
Ca_Kabuli_NC_021166.1_17063559	Ca_kabuli_chr07	17063559	[A/C]	1067302131
Ca_Kabuli_NC_021166.1_17063560	Ca_kabuli_chr07	17063560	[G/T]	1067302132
Ca_Kabuli_NC_021166.1_17063561	Ca_kabuli_chr07	17063561	[A/C]	1067302133
Ca_Kabuli_NC_021166.1_17063562	Ca_kabuli_chr07	17063562	[T/C]	1067302134
Ca_Kabuli_NC_021166.1_17064935	Ca_kabuli_chr07	17064935	[A/T]	1067302135
Ca_Kabuli_NC_021166.1_17064945	Ca_kabuli_chr07	17064945	[A/G]	1067302136
Ca_Kabuli_NC_021166.1_17064989	Ca_kabuli_chr07	17064989	[T/A]	1067302137
Ca_Kabuli_NC_021166.1_17133036	Ca_kabuli_chr07	17133036	[C/T]	1067302138
Ca_Kabuli_NC_021166.1_17133041	Ca_kabuli_chr07	17133041	[G/A]	1067302139
Ca_Kabuli_NC_021166.1_17133042	Ca_kabuli_chr07	17133042	[T/A]	1067302140
Ca_Kabuli_NC_021166.1_17133046	Ca_kabuli_chr07	17133046	[T/G]	1067302141
Ca_Kabuli_NC_021166.1_17140656	Ca_kabuli_chr07	17140656	[C/A]	1067302142
Ca_Kabuli_NC_021166.1_17140659	Ca_kabuli_chr07	17140659	[A/G]	1067302143
Ca_Kabuli_NC_021166.1_17140682	Ca_kabuli_chr07	17140682	[T/C]	1067302144
Ca_Kabuli_NC_021166.1_17140684	Ca_kabuli_chr07	17140684	[A/C]	1067302145
Ca_Kabuli_NC_021166.1_17222165	Ca_kabuli_chr07	17222165	[A/C]	1067302146
Ca_Kabuli_NC_021166.1_17222192	Ca_kabuli_chr07	17222192	[A/C]	1067302147
Ca_Kabuli_NC_021166.1_17304408	Ca_kabuli_chr07	17304408	[A/C]	1067302148
Ca_Kabuli_NC_021166.1_17304409	Ca_kabuli_chr07	17304409	[G/T]	1067302149
Ca_Kabuli_NC_021166.1_17304410	Ca_kabuli_chr07	17304410	[A/T]	1067302150
Ca_Kabuli_NC_021166.1_17304411	Ca_kabuli_chr07	17304411	[T/G]	1067302151
Ca_Kabuli_NC_021166.1_17559707	Ca_kabuli_chr07	17559707	[T/C]	1067302152
Ca_Kabuli_NC_021166.1_17559753	Ca_kabuli_chr07	17559753	[T/C]	1067302153
Ca_Kabuli_NC_021166.1_17588267	Ca_kabuli_chr07	17588267	[A/C]	1067302154
Ca_Kabuli_NC_021166.1_17595584	Ca_kabuli_chr07	17595584	[A/T]	1067302155
Ca_Kabuli_NC_021166.1_17595590	Ca_kabuli_chr07	17595590	[A/T]	1067302156
Ca_Kabuli_NC_021166.1_17597367	Ca_kabuli_chr07	17597367	[T/G]	1067302157
Ca_Kabuli_NC_021166.1_17620142	Ca_kabuli_chr07	17620142	[A/T]	1067302158
Ca_Kabuli_NC_021166.1_17620146	Ca_kabuli_chr07	17620146	[C/T]	1067302159
Ca_Kabuli_NC_021166.1_17662183	Ca_kabuli_chr07	17662183	[T/A]	1067302160
Ca_Kabuli_NC_021166.1_17662190	Ca_kabuli_chr07	17662190	[T/A]	1067302161
Ca_Kabuli_NC_021166.1_17662191	Ca_kabuli_chr07	17662191	[C/A]	1067302162

SNP IDs	Chromosomes	Physical positions (bp)	SNPs	NCBI SNPdb accession IDs (NCBI_ss#)
Ca_Kabuli_NC_021166.1_17757808	Ca_kabuli_chr07	17757808	[T/C]	1067302163
Ca_Kabuli_NC_021166.1_17757852	Ca_kabuli_chr07	17757852	[A/G]	1067302164
Ca_Kabuli_NC_021166.1_17757870	Ca_kabuli_chr07	17757870	[T/A]	1067302165
Ca_Kabuli_NC_021166.1_17757872	Ca_kabuli_chr07	17757872	[A/G]	1067302166
Ca_Kabuli_NC_021166.1_17775266	Ca_kabuli_chr07	17775266	[A/C]	1067302167
Ca_Kabuli_NC_021166.1_17779388	Ca_kabuli_chr07	17779388	[T/A]	1067302168
Ca_Kabuli_NC_021166.1_17779398	Ca_kabuli_chr07	17779398	[A/C]	1067302169
Ca_Kabuli_NC_021166.1_17779575	Ca_kabuli_chr07	17779575	[A/G]	1067302170
Ca_Kabuli_NC_021166.1_17779594	Ca_kabuli_chr07	17779594	[T/G]	1067302171
Ca_Kabuli_NC_021166.1_17779720	Ca_kabuli_chr07	17779720	[T/G]	1067302172
Ca_Kabuli_NC_021166.1_17779746	Ca_kabuli_chr07	17779746	[A/G]	1067302173
Ca_Kabuli_NC_021166.1_17789596	Ca_kabuli_chr07	17789596	[T/G]	1067302174
Ca_Kabuli_NC_021166.1_17789599	Ca_kabuli_chr07	17789599	[A/C]	1067302175
Ca_Kabuli_NC_021166.1_17789611	Ca_kabuli_chr07	17789611	[A/C]	1067302176
Ca_Kabuli_NC_021166.1_17789612	Ca_kabuli_chr07	17789612	[C/A]	1067302177
Ca_Kabuli_NC_021166.1_17789624	Ca_kabuli_chr07	17789624	[G/A]	1067302178
Ca_Kabuli_NC_021166.1_17789644	Ca_kabuli_chr07	17789644	[T/C]	1067302179
Ca_Kabuli_NC_021166.1_17862656	Ca_kabuli_chr07	17862656	[T/A]	1067302180
Ca_Kabuli_NC_021166.1_17862658	Ca_kabuli_chr07	17862658	[T/G]	1067302181
Ca_Kabuli_NC_021166.1_17862691	Ca_kabuli_chr07	17862691	[A/T]	1067302182
Ca_Kabuli_NC_021166.1_17862696	Ca_kabuli_chr07	17862696	[A/C]	1067302183
Ca_Kabuli_NC_021166.1_18319022	Ca_kabuli_chr07	18319022	[A/T]	1067302184
Ca_Kabuli_NC_021166.1_18319824	Ca_kabuli_chr07	18319824	[T/C]	1067302185
Ca_Kabuli_NC_021166.1_18462652	Ca_kabuli_chr07	18462652	[C/A]	1067302186
Ca_Kabuli_NC_021166.1_18462653	Ca_kabuli_chr07	18462653	[C/G]	1067302187
Ca_Kabuli_NC_021166.1_18462654	Ca_kabuli_chr07	18462654	[T/A]	1067302188
Ca_Kabuli_NC_021166.1_18527968	Ca_kabuli_chr07	18527968	[T/C]	1067302189
Ca_Kabuli_NC_021166.1_18642417	Ca_kabuli_chr07	18642417	[T/C]	1067302190
Ca_Kabuli_NC_021166.1_18642486	Ca_kabuli_chr07	18642486	[T/C]	1067302191
Ca_Kabuli_NC_021166.1_18656219	Ca_kabuli_chr07	18656219	[A/C]	1067302192
Ca_Kabuli_NC_021166.1_18658595	Ca_kabuli_chr07	18658595	[A/C]	1067302193
Ca_Kabuli_NC_021166.1_18697880	Ca_kabuli_chr07	18697880	[A/T]	1067302194
Ca_Kabuli_NC_021166.1_18697881	Ca_kabuli_chr07	18697881	[T/C]	1067302195
Ca_Kabuli_NC_021166.1_18697882	Ca_kabuli_chr07	18697882	[C/G]	1067302196

SNP IDs	Chromosomes	Physical positions (bp)	SNPs	NCBI SNPdb accession IDs (NCBI_ss#)
Ca_Kabuli_NC_021166.1_18697883	Ca_kabuli_chr07	18697883	[T/G]	1067302197
Ca_Kabuli_NC_021166.1_18719807	Ca_kabuli_chr07	18719807	[T/G]	1067302198
Ca_Kabuli_NC_021166.1_18719820	Ca_kabuli_chr07	18719820	[T/G]	1067302199
Ca_Kabuli_NC_021166.1_18946700	Ca_kabuli_chr07	18946700	[T/C]	1067302200
Ca_Kabuli_NC_021166.1_18946748	Ca_kabuli_chr07	18946748	[A/C]	1067302201
Ca_Kabuli_NC_021166.1_18946792	Ca_kabuli_chr07	18946792	[C/A]	1067302202
Ca_Kabuli_NC_021166.1_19002735	Ca_kabuli_chr07	19002735	[A/G]	1067302203
Ca_Kabuli_NC_021166.1_19042218	Ca_kabuli_chr07	19042218	[T/C]	1067302204
Ca_Kabuli_NC_021166.1_19081722	Ca_kabuli_chr07	19081722	[T/G]	1067302205
Ca_Kabuli_NC_021166.1_19081724	Ca_kabuli_chr07	19081724	[A/G]	1067302206
Ca_Kabuli_NC_021166.1_19081728	Ca_kabuli_chr07	19081728	[C/T]	1067302207
Ca_Kabuli_NC_021166.1_19081731	Ca_kabuli_chr07	19081731	[A/G]	1067302208
Ca_Kabuli_NC_021166.1_19217473	Ca_kabuli_chr07	19217473	[T/C]	1067302209
Ca_Kabuli_NC_021166.1_19217496	Ca_kabuli_chr07	19217496	[A/G]	1067302210
Ca_Kabuli_NC_021166.1_19233130	Ca_kabuli_chr07	19233130	[A/C]	1067302211
Ca_Kabuli_NC_021166.1_19233146	Ca_kabuli_chr07	19233146	[A/C]	1067302212
Ca_Kabuli_NC_021166.1_19233153	Ca_kabuli_chr07	19233153	[A/C]	1067302213
Ca_Kabuli_NC_021166.1_19233163	Ca_kabuli_chr07	19233163	[A/C]	1067302214
Ca_Kabuli_NC_021166.1_19523982	Ca_kabuli_chr07	19523982	[A/G]	1067302215
Ca_Kabuli_NC_021166.1_19523991	Ca_kabuli_chr07	19523991	[A/G]	1067302216
Ca_Kabuli_NC_021166.1_19523996	Ca_kabuli_chr07	19523996	[C/G]	1067302217
Ca_Kabuli_NC_021166.1_19523999	Ca_kabuli_chr07	19523999	[T/C]	1067302218
Ca_Kabuli_NC_021166.1_19524001	Ca_kabuli_chr07	19524001	[A/G]	1067302219
Ca_Kabuli_NC_021166.1_19549600	Ca_kabuli_chr07	19549600	[A/C]	1067302220
Ca_Kabuli_NC_021166.1_19549678	Ca_kabuli_chr07	19549678	[T/C]	1067302221
Ca_Kabuli_NC_021166.1_19549690	Ca_kabuli_chr07	19549690	[T/G]	1067302222
Ca_Kabuli_NC_021166.1_19549713	Ca_kabuli_chr07	19549713	[T/C]	1067302223
Ca_Kabuli_NC_021166.1_19620340	Ca_kabuli_chr07	19620340	[A/G]	1067302224
Ca_Kabuli_NC_021166.1_19707901	Ca_kabuli_chr07	19707901	[A/C]	1067302225
Ca_Kabuli_NC_021166.1_19988607	Ca_kabuli_chr07	19988607	[A/C]	1067302226
Ca_Kabuli_NC_021166.1_19988626	Ca_kabuli_chr07	19988626	[T/G]	1067302227
Ca_Kabuli_NC_021166.1_20082879	Ca_kabuli_chr07	20082879	[T/C]	1067302228
Ca_Kabuli_NC_021166.1_20083015	Ca_kabuli_chr07	20083015	[A/G]	1067302229
Ca_Kabuli_NC_021166.1_20099351	Ca_kabuli_chr07	20099351	[T/G]	1067302230

SNP IDs	Chromosomes	Physical positions (bp)	SNPs	NCBI SNPdb accession IDs (NCBI_ss#)
Ca_Kabuli_NC_021166.1_20099357	Ca_kabuli_chr07	20099357	[A/G]	1067302231
Ca_Kabuli_NC_021166.1_20111036	Ca_kabuli_chr07	20111036	[T/A]	1067302232
Ca_Kabuli_NC_021166.1_20111132	Ca_kabuli_chr07	20111132	[A/C]	1067302233
Ca_Kabuli_NC_021166.1_20111151	Ca_kabuli_chr07	20111151	[A/T]	1067302234
Ca_Kabuli_NC_021166.1_20111175	Ca_kabuli_chr07	20111175	[C/A]	1067302235
Ca_Kabuli_NC_021166.1_20111213	Ca_kabuli_chr07	20111213	[T/C]	1067302236
Ca_Kabuli_NC_021166.1_20143920	Ca_kabuli_chr07	20143920	[T/G]	1067302237
Ca_Kabuli_NC_021166.1_20175675	Ca_kabuli_chr07	20175675	[G/A]	1067302238
Ca_Kabuli_NC_021166.1_20175676	Ca_kabuli_chr07	20175676	[G/C]	1067302239
Ca_Kabuli_NC_021166.1_20175680	Ca_kabuli_chr07	20175680	[G/T]	1067302240
Ca_Kabuli_NC_021166.1_20218289	Ca_kabuli_chr07	20218289	[A/G]	1067302241
Ca_Kabuli_NC_021166.1_20218300	Ca_kabuli_chr07	20218300	[C/T]	1067302242
Ca_Kabuli_NC_021166.1_20234537	Ca_kabuli_chr07	20234537	[A/C]	1067302243
Ca_Kabuli_NC_021166.1_20253424	Ca_kabuli_chr07	20253424	[G/A]	1067302244
Ca_Kabuli_NC_021166.1_20293669	Ca_kabuli_chr07	20293669	[A/C]	1067302245
Ca_Kabuli_NC_021166.1_20293736	Ca_kabuli_chr07	20293736	[C/T]	1067302246
Ca_Kabuli_NC_021166.1_20373645	Ca_kabuli_chr07	20373645	[A/C]	1067302247
Ca_Kabuli_NC_021166.1_20373656	Ca_kabuli_chr07	20373656	[A/C]	1067302248
Ca_Kabuli_NC_021166.1_20373666	Ca_kabuli_chr07	20373666	[A/G]	1067302249
Ca_Kabuli_NC_021166.1_20373673	Ca_kabuli_chr07	20373673	[A/T]	1067302250
Ca_Kabuli_NC_021166.1_20412700	Ca_kabuli_chr07	20412700	[A/C]	1067302251
Ca_Kabuli_NC_021166.1_20412701	Ca_kabuli_chr07	20412701	[G/T]	1067302252
Ca_Kabuli_NC_021166.1_20412702	Ca_kabuli_chr07	20412702	[A/T]	1067302253
Ca_Kabuli_NC_021166.1_20412703	Ca_kabuli_chr07	20412703	[T/A]	1067302254
Ca_Kabuli_NC_021166.1_20419509	Ca_kabuli_chr07	20419509	[T/A]	1067302255
Ca_Kabuli_NC_021166.1_20419524	Ca_kabuli_chr07	20419524	[T/C]	1067302256
Ca_Kabuli_NC_021166.1_20452200	Ca_kabuli_chr07	20452200	[C/T]	1067302257
Ca_Kabuli_NC_021166.1_20452201	Ca_kabuli_chr07	20452201	[A/C]	1067302258
Ca_Kabuli_NC_021166.1_20452202	Ca_kabuli_chr07	20452202	[G/T]	1067302259
Ca_Kabuli_NC_021166.1_20452278	Ca_kabuli_chr07	20452278	[G/A]	1067302260
Ca_Kabuli_NC_021166.1_20452457	Ca_kabuli_chr07	20452457	[G/A]	1067302261
Ca_Kabuli_NC_021166.1_20496072	Ca_kabuli_chr07	20496072	[T/G]	1067302262
Ca_Kabuli_NC_021166.1_20496104	Ca_kabuli_chr07	20496104	[T/G]	1067302263
Ca_Kabuli_NC_021166.1_20496303	Ca_kabuli_chr07	20496303	[T/C]	1067302264

SNP IDs	Chromosomes	Physical positions (bp)	SNPs	NCBI SNPdb accession IDs (NCBI_ss#)
Ca_Kabuli_NC_021166.1_20496319	Ca_kabuli_chr07	20496319	[T/C]	1067302265
Ca_Kabuli_NC_021166.1_20496320	Ca_kabuli_chr07	20496320	[G/T]	1067302266
Ca_Kabuli_NC_021166.1_20496405	Ca_kabuli_chr07	20496405	[C/T]	1067302267
Ca_Kabuli_NC_021166.1_20496421	Ca_kabuli_chr07	20496421	[A/C]	1067302268
Ca_Kabuli_NC_021166.1_20498552	Ca_kabuli_chr07	20498552	[C/T]	1067302269
Ca_Kabuli_NC_021166.1_20498626	Ca_kabuli_chr07	20498626	[G/T]	1067302270
Ca_Kabuli_NC_021166.1_20538485	Ca_kabuli_chr07	20538485	[A/G]	1067302271
Ca_Kabuli_NC_021166.1_20543112	Ca_kabuli_chr07	20543112	[C/T]	1067302272
Ca_Kabuli_NC_021166.1_20543119	Ca_kabuli_chr07	20543119	[T/C]	1067302273
Ca_Kabuli_NC_021166.1_20543181	Ca_kabuli_chr07	20543181	[A/C]	1067302274
Ca_Kabuli_NC_021166.1_20556466	Ca_kabuli_chr07	20556466	[G/C]	1067302275
Ca_Kabuli_NC_021166.1_20564024	Ca_kabuli_chr07	20564024	[G/A]	1067302276
Ca_Kabuli_NC_021166.1_20564088	Ca_kabuli_chr07	20564088	[A/G]	1067302277
Ca_Kabuli_NC_021166.1_20564297	Ca_kabuli_chr07	20564297	[A/G]	1067302278
Ca_Kabuli_NC_021166.1_20586113	Ca_kabuli_chr07	20586113	[G/A]	1067302279
Ca_Kabuli_NC_021166.1_20603334	Ca_kabuli_chr07	20603334	[C/T]	1067302280
Ca_Kabuli_NC_021166.1_20606378	Ca_kabuli_chr07	20606378	[T/G]	1067302281
Ca_Kabuli_NC_021166.1_20606383	Ca_kabuli_chr07	20606383	[T/G]	1067302282
Ca_Kabuli_NC_021166.1_20607926	Ca_kabuli_chr07	20607926	[T/G]	1067302283
Ca_Kabuli_NC_021166.1_20610549	Ca_kabuli_chr07	20610549	[T/A]	1067302284
Ca_Kabuli_NC_021166.1_20613547	Ca_kabuli_chr07	20613547	[A/G]	1067302285
Ca_Kabuli_NC_021166.1_20613718	Ca_kabuli_chr07	20613718	[C/T]	1067302286
Ca_Kabuli_NC_021166.1_20637257	Ca_kabuli_chr07	20637257	[T/G]	1067302287
Ca_Kabuli_NC_021166.1_20637361	Ca_kabuli_chr07	20637361	[T/G]	1067302288
Ca_Kabuli_NC_021166.1_20637373	Ca_kabuli_chr07	20637373	[T/A]	1067302289
Ca_Kabuli_NC_021166.1_20637410	Ca_kabuli_chr07	20637410	[A/C]	1067302290
Ca_Kabuli_NC_021166.1_20637431	Ca_kabuli_chr07	20637431	[A/G]	1067302291
Ca_Kabuli_NC_021166.1_20921484	Ca_kabuli_chr07	20921484	[C/A]	1067302292
Ca_Kabuli_NC_021166.1_20921497	Ca_kabuli_chr07	20921497	[T/G]	1067302293
Ca_Kabuli_NC_021166.1_20921517	Ca_kabuli_chr07	20921517	[T/G]	1067302294
Ca_Kabuli_NC_021166.1_20921527	Ca_kabuli_chr07	20921527	[T/G]	1067302295
Ca_Kabuli_NC_021166.1_20921532	Ca_kabuli_chr07	20921532	[T/A]	1067302296
Ca_Kabuli_NC_021166.1_21073366	Ca_kabuli_chr07	21073366	[A/C]	1067302297
Ca_Kabuli_NC_021166.1_21073385	Ca_kabuli_chr07	21073385	[A/C]	1067302298

SNP IDs	Chromosomes	Physical positions (bp)	SNPs	NCBI SNPdb accession IDs (NCBI_ss#)
Ca_Kabuli_NC_021166.1_21404133	Ca_kabuli_chr07	21404133	[A/G]	1067302299
Ca_Kabuli_NC_021166.1_21511596	Ca_kabuli_chr07	21511596	[A/C]	1067302300
Ca_Kabuli_NC_021166.1_21580134	Ca_kabuli_chr07	21580134	[A/T]	1067302301
Ca_Kabuli_NC_021166.1_21592597	Ca_kabuli_chr07	21592597	[A/G]	1067302302
Ca_Kabuli_NC_021166.1_21595473	Ca_kabuli_chr07	21595473	[T/A]	1067302303
Ca_Kabuli_NC_021166.1_21595478	Ca_kabuli_chr07	21595478	[T/G]	1067302304
Ca_Kabuli_NC_021166.1_21595491	Ca_kabuli_chr07	21595491	[T/C]	1067302305
Ca_Kabuli_NC_021166.1_21595499	Ca_kabuli_chr07	21595499	[A/T]	1067302306
Ca_Kabuli_NC_021166.1_21616285	Ca_kabuli_chr07	21616285	[A/G]	1067302307
Ca_Kabuli_NC_021166.1_21616287	Ca_kabuli_chr07	21616287	[T/G]	1067302308
Ca_Kabuli_NC_021166.1_21616294	Ca_kabuli_chr07	21616294	[T/G]	1067302309
Ca_Kabuli_NC_021166.1_21616296	Ca_kabuli_chr07	21616296	[A/G]	1067302310
Ca_Kabuli_NC_021166.1_21633183	Ca_kabuli_chr07	21633183	[A/C]	1067302311
Ca_Kabuli_NC_021166.1_21639234	Ca_kabuli_chr07	21639234	[G/A]	1067302312
Ca_Kabuli_NC_021166.1_21639238	Ca_kabuli_chr07	21639238	[G/A]	1067302313
Ca_Kabuli_NC_021166.1_21639241	Ca_kabuli_chr07	21639241	[T/A]	1067302314
Ca_Kabuli_NC_021166.1_21639244	Ca_kabuli_chr07	21639244	[T/G]	1067302315
Ca_Kabuli_NC_021166.1_21639347	Ca_kabuli_chr07	21639347	[T/G]	1067302316
Ca_Kabuli_NC_021166.1_21644319	Ca_kabuli_chr07	21644319	[A/T]	1067302317
Ca_Kabuli_NC_021166.1_21644342	Ca_kabuli_chr07	21644342	[G/A]	1067302318
Ca_Kabuli_NC_021166.1_21736778	Ca_kabuli_chr07	21736778	[G/A]	1067302319
Ca_Kabuli_NC_021166.1_21744514	Ca_kabuli_chr07	21744514	[T/C]	1067302320
Ca_Kabuli_NC_021166.1_21809119	Ca_kabuli_chr07	21809119	[C/A]	1067302321
Ca_Kabuli_NC_021166.1_21809120	Ca_kabuli_chr07	21809120	[A/G]	1067302322
Ca_Kabuli_NC_021166.1_21814575	Ca_kabuli_chr07	21814575	[C/A]	1067302323
Ca_Kabuli_NC_021166.1_21814576	Ca_kabuli_chr07	21814576	[A/G]	1067302324
Ca_Kabuli_NC_021166.1_21814577	Ca_kabuli_chr07	21814577	[T/A]	1067302325
Ca_Kabuli_NC_021166.1_21814578	Ca_kabuli_chr07	21814578	[G/T]	1067302326
Ca_Kabuli_NC_021166.1_21814580	Ca_kabuli_chr07	21814580	[C/G]	1067302327
Ca_Kabuli_NC_021166.1_22236552	Ca_kabuli_chr07	22236552	[A/C]	1067302328
Ca_Kabuli_NC_021166.1_22496764	Ca_kabuli_chr07	22496764	[C/A]	1067302329
Ca_Kabuli_NC_021166.1_22707313	Ca_kabuli_chr07	22707313	[G/A]	1067302330
Ca_Kabuli_NC_021166.1_22707344	Ca_kabuli_chr07	22707344	[T/C]	1067302331
Ca_Kabuli_NC_021166.1_22721398	Ca_kabuli_chr07	22721398	[T/G]	1067302332

SNP IDs	Chromosomes	Physical positions (bp)	SNPs	NCBI SNPdb accession IDs (NCBI_ss#)
Ca_Kabuli_NC_021166.1_22723179	Ca_kabuli_chr07	22723179	[A/T]	1067302333
Ca_Kabuli_NC_021166.1_22731265	Ca_kabuli_chr07	22731265	[T/A]	1067302334
Ca_Kabuli_NC_021166.1_22731293	Ca_kabuli_chr07	22731293	[T/G]	1067302335
Ca_Kabuli_NC_021166.1_22731322	Ca_kabuli_chr07	22731322	[A/G]	1067302336
Ca_Kabuli_NC_021166.1_22731364	Ca_kabuli_chr07	22731364	[T/C]	1067302337
Ca_Kabuli_NC_021166.1_22731388	Ca_kabuli_chr07	22731388	[T/C]	1067302338
Ca_Kabuli_NC_021166.1_22731398	Ca_kabuli_chr07	22731398	[A/G]	1067302339
Ca_Kabuli_NC_021166.1_22731403	Ca_kabuli_chr07	22731403	[A/G]	1067302340
Ca_Kabuli_NC_021166.1_22731410	Ca_kabuli_chr07	22731410	[A/G]	1067302341
Ca_Kabuli_NC_021166.1_22731413	Ca_kabuli_chr07	22731413	[A/G]	1067302342
Ca_Kabuli_NC_021166.1_22750211	Ca_kabuli_chr07	22750211	[T/A]	1067302343
Ca_Kabuli_NC_021166.1_22750241	Ca_kabuli_chr07	22750241	[T/G]	1067302344
Ca_Kabuli_NC_021166.1_22750264	Ca_kabuli_chr07	22750264	[T/G]	1067302345
Ca_Kabuli_NC_021166.1_22750348	Ca_kabuli_chr07	22750348	[A/G]	1067302346
Ca_Kabuli_NC_021166.1_22803876	Ca_kabuli_chr07	22803876	[C/A]	1067302347
Ca_Kabuli_NC_021166.1_22803877	Ca_kabuli_chr07	22803877	[C/T]	1067302348
Ca_Kabuli_NC_021166.1_22803878	Ca_kabuli_chr07	22803878	[G/C]	1067302349
Ca_Kabuli_NC_021166.1_22803879	Ca_kabuli_chr07	22803879	[G/T]	1067302350
Ca_Kabuli_NC_021166.1_22839776	Ca_kabuli_chr07	22839776	[G/A]	1067302351
Ca_Kabuli_NC_021166.1_22893125	Ca_kabuli_chr07	22893125	[T/C]	1067302352
Ca_Kabuli_NC_021166.1_22893135	Ca_kabuli_chr07	22893135	[T/C]	1067302353
Ca_Kabuli_NC_021166.1_22893191	Ca_kabuli_chr07	22893191	[A/G]	1067302354
Ca_Kabuli_NC_021166.1_22958199	Ca_kabuli_chr07	22958199	[G/A]	1067302355
Ca_Kabuli_NC_021166.1_22987819	Ca_kabuli_chr07	22987819	[A/G]	1067302356
Ca_Kabuli_NC_021166.1_22987832	Ca_kabuli_chr07	22987832	[G/C]	1067302357
Ca_Kabuli_NC_021166.1_22987838	Ca_kabuli_chr07	22987838	[A/G]	1067302358
Ca_Kabuli_NC_021166.1_23103084	Ca_kabuli_chr07	23103084	[C/T]	1067302359
Ca_Kabuli_NC_021166.1_23115511	Ca_kabuli_chr07	23115511	[T/C]	1067302360
Ca_Kabuli_NC_021166.1_23115575	Ca_kabuli_chr07	23115575	[A/C]	1067302361
Ca_Kabuli_NC_021166.1_23439178	Ca_kabuli_chr07	23439178	[A/C]	1067302362
Ca_Kabuli_NC_021166.1_23439199	Ca_kabuli_chr07	23439199	[A/C]	1067302363
Ca_Kabuli_NC_021166.1_23574181	Ca_kabuli_chr07	23574181	[C/T]	1067302364
Ca_Kabuli_NC_021166.1_23574187	Ca_kabuli_chr07	23574187	[T/C]	1067302365
Ca_Kabuli_NC_021166.1_23574823	Ca_kabuli_chr07	23574823	[C/T]	1067302366

SNP IDs	Chromosomes	Physical positions (bp)	SNPs	NCBI SNPdb accession IDs (NCBI_ss#)
Ca_Kabuli_NC_021166.1_23575633	Ca_kabuli_chr07	23575633	[T/G]	1067302367
Ca_Kabuli_NC_021166.1_23615942	Ca_kabuli_chr07	23615942	[T/G]	1067302368
Ca_Kabuli_NC_021166.1_23615944	Ca_kabuli_chr07	23615944	[T/C]	1067302369
Ca_Kabuli_NC_021166.1_23615946	Ca_kabuli_chr07	23615946	[T/C]	1067302370
Ca_Kabuli_NC_021166.1_23615960	Ca_kabuli_chr07	23615960	[A/G]	1067302371
Ca_Kabuli_NC_021166.1_23615984	Ca_kabuli_chr07	23615984	[A/G]	1067302372
Ca_Kabuli_NC_021166.1_23616003	Ca_kabuli_chr07	23616003	[A/G]	1067302373
Ca_Kabuli_NC_021166.1_23616014	Ca_kabuli_chr07	23616014	[A/G]	1067302374
Ca_Kabuli_NC_021166.1_23616063	Ca_kabuli_chr07	23616063	[A/T]	1067302375
Ca_Kabuli_NC_021166.1_23664610	Ca_kabuli_chr07	23664610	[A/C]	1067302376
Ca_Kabuli_NC_021166.1_23664646	Ca_kabuli_chr07	23664646	[G/T]	1067302377
Ca_Kabuli_NC_021166.1_24021658	Ca_kabuli_chr07	24021658	[T/C]	1067302378
Ca_Kabuli_NC_021166.1_24021667	Ca_kabuli_chr07	24021667	[T/G]	1067302379
Ca_Kabuli_NC_021166.1_24021683	Ca_kabuli_chr07	24021683	[G/A]	1067302380
Ca_Kabuli_NC_021166.1_24021690	Ca_kabuli_chr07	24021690	[T/G]	1067302381
Ca_Kabuli_NC_021166.1_24021691	Ca_kabuli_chr07	24021691	[T/C]	1067302382
Ca_Kabuli_NC_021166.1_24181315	Ca_kabuli_chr07	24181315	[A/G]	1067302383
Ca_Kabuli_NC_021166.1_24181322	Ca_kabuli_chr07	24181322	[C/A]	1067302384
Ca_Kabuli_NC_021166.1_24181331	Ca_kabuli_chr07	24181331	[T/C]	1067302385
Ca_Kabuli_NC_021166.1_24181341	Ca_kabuli_chr07	24181341	[T/G]	1067302386
Ca_Kabuli_NC_021166.1_24415813	Ca_kabuli_chr07	24415813	[T/A]	1067302387
Ca_Kabuli_NC_021166.1_24415854	Ca_kabuli_chr07	24415854	[A/G]	1067302388
Ca_Kabuli_NC_021166.1_24415873	Ca_kabuli_chr07	24415873	[A/G]	1067302389
Ca_Kabuli_NC_021166.1_24415895	Ca_kabuli_chr07	24415895	[A/T]	1067302390
Ca_Kabuli_NC_021166.1_24543018	Ca_kabuli_chr07	24543018	[A/T]	1067302391
Ca_Kabuli_NC_021166.1_24543026	Ca_kabuli_chr07	24543026	[G/C]	1067302392
Ca_Kabuli_NC_021166.1_24543030	Ca_kabuli_chr07	24543030	[A/G]	1067302393
Ca_Kabuli_NC_021166.1_24544547	Ca_kabuli_chr07	24544547	[T/C]	1067302394
Ca_Kabuli_NC_021166.1_24544567	Ca_kabuli_chr07	24544567	[A/C]	1067302395
Ca_Kabuli_NC_021166.1_24882282	Ca_kabuli_chr07	24882282	[A/G]	1067302396
Ca_Kabuli_NC_021166.1_24882332	Ca_kabuli_chr07	24882332	[T/A]	1067302397
Ca_Kabuli_NC_021166.1_25194703	Ca_kabuli_chr07	25194703	[T/A]	1067302398
Ca_Kabuli_NC_021166.1_25218318	Ca_kabuli_chr07	25218318	[A/C]	1067302399
Ca_Kabuli_NC_021166.1_25218387	Ca_kabuli_chr07	25218387	[A/C]	1067302400

SNP IDs	Chromosomes	Physical positions (bp)	SNPs	NCBI SNPdb accession IDs (NCBI_ss#)
Ca_Kabuli_NC_021166.1_25304081	Ca_kabuli_chr07	25304081	[G/C]	1067302401
Ca_Kabuli_NC_021166.1_25428660	Ca_kabuli_chr07	25428660	[G/A]	1067302402
Ca_Kabuli_NC_021166.1_25581054	Ca_kabuli_chr07	25581054	[A/G]	1067302403
Ca_Kabuli_NC_021166.1_25581056	Ca_kabuli_chr07	25581056	[A/C]	1067302404
Ca_Kabuli_NC_021166.1_26144164	Ca_kabuli_chr07	26144164	[T/C]	1067302405
Ca_Kabuli_NC_021166.1_26144172	Ca_kabuli_chr07	26144172	[T/C]	1067302406
Ca_Kabuli_NC_021166.1_26149441	Ca_kabuli_chr07	26149441	[A/C]	1067302407
Ca_Kabuli_NC_021166.1_26149451	Ca_kabuli_chr07	26149451	[A/C]	1067302408
Ca_Kabuli_NC_021166.1_26161030	Ca_kabuli_chr07	26161030	[C/T]	1067302409
Ca_Kabuli_NC_021166.1_26226146	Ca_kabuli_chr07	26226146	[C/G]	1067302410
Ca_Kabuli_NC_021166.1_26226151	Ca_kabuli_chr07	26226151	[T/C]	1067302411
Ca_Kabuli_NC_021166.1_26226177	Ca_kabuli_chr07	26226177	[T/A]	1067302412
Ca_Kabuli_NC_021166.1_26580139	Ca_kabuli_chr07	26580139	[A/C]	1067302413
Ca_Kabuli_NC_021166.1_26692131	Ca_kabuli_chr07	26692131	[T/C]	1067302414
Ca_Kabuli_NC_021166.1_26692194	Ca_kabuli_chr07	26692194	[A/T]	1067302415
Ca_Kabuli_NC_021166.1_26692195	Ca_kabuli_chr07	26692195	[G/C]	1067302416
Ca_Kabuli_NC_021166.1_26694546	Ca_kabuli_chr07	26694546	[T/A]	1067302417
Ca_Kabuli_NC_021166.1_26700368	Ca_kabuli_chr07	26700368	[A/G]	1067302418
Ca_Kabuli_NC_021166.1_26700373	Ca_kabuli_chr07	26700373	[C/T]	1067302419
Ca_Kabuli_NC_021166.1_26701540	Ca_kabuli_chr07	26701540	[T/G]	1067302420
Ca_Kabuli_NC_021166.1_26701552	Ca_kabuli_chr07	26701552	[G/A]	1067302421
Ca_Kabuli_NC_021166.1_26701557	Ca_kabuli_chr07	26701557	[A/T]	1067302422
Ca_Kabuli_NC_021166.1_26701563	Ca_kabuli_chr07	26701563	[A/T]	1067302423
Ca_Kabuli_NC_021166.1_26701573	Ca_kabuli_chr07	26701573	[G/C]	1067302424
Ca_Kabuli_NC_021166.1_26701603	Ca_kabuli_chr07	26701603	[A/C]	1067302425
Ca_Kabuli_NC_021166.1_26701625	Ca_kabuli_chr07	26701625	[A/C]	1067302426
Ca_Kabuli_NC_021166.1_26701628	Ca_kabuli_chr07	26701628	[A/G]	1067302427
Ca_Kabuli_NC_021166.1_26701643	Ca_kabuli_chr07	26701643	[T/G]	1067302428
Ca_Kabuli_NC_021166.1_26761639	Ca_kabuli_chr07	26761639	[A/T]	1067302429
Ca_Kabuli_NC_021166.1_26882849	Ca_kabuli_chr07	26882849	[T/C]	1067302430
Ca_Kabuli_NC_021166.1_26940696	Ca_kabuli_chr07	26940696	[T/A]	1067302431
Ca_Kabuli_NC_021166.1_26940789	Ca_kabuli_chr07	26940789	[A/G]	1067302432
Ca_Kabuli_NC_021166.1_26940817	Ca_kabuli_chr07	26940817	[T/C]	1067302433
Ca_Kabuli_NC_021166.1_26940823	Ca_kabuli_chr07	26940823	[A/G]	1067302434

SNP IDs	Chromosomes	Physical positions (bp)	SNPs	NCBI SNPdb accession IDs (NCBI_ss#)
Ca_Kabuli_NC_021166.1_26940829	Ca_kabuli_chr07	26940829	[T/G]	1067302435
Ca_Kabuli_NC_021166.1_26940859	Ca_kabuli_chr07	26940859	[A/T]	1067302436
Ca_Kabuli_NC_021166.1_26940896	Ca_kabuli_chr07	26940896	[G/A]	1067302437
Ca_Kabuli_NC_021166.1_26940934	Ca_kabuli_chr07	26940934	[A/G]	1067302438
Ca_Kabuli_NC_021166.1_26940939	Ca_kabuli_chr07	26940939	[T/G]	1067302439
Ca_Kabuli_NC_021166.1_26940955	Ca_kabuli_chr07	26940955	[T/G]	1067302440
Ca_Kabuli_NC_021166.1_27051386	Ca_kabuli_chr07	27051386	[T/C]	1067302441
Ca_Kabuli_NC_021166.1_27051399	Ca_kabuli_chr07	27051399	[A/G]	1067302442
Ca_Kabuli_NC_021166.1_27051410	Ca_kabuli_chr07	27051410	[T/G]	1067302443
Ca_Kabuli_NC_021166.1_27051446	Ca_kabuli_chr07	27051446	[A/G]	1067302444
Ca_Kabuli_NC_021166.1_27051468	Ca_kabuli_chr07	27051468	[A/C]	1067302445
Ca_Kabuli_NC_021166.1_27051760	Ca_kabuli_chr07	27051760	[A/C]	1067302446
Ca_Kabuli_NC_021166.1_27070626	Ca_kabuli_chr07	27070626	[G/C]	1067302447
Ca_Kabuli_NC_021166.1_27070630	Ca_kabuli_chr07	27070630	[A/C]	1067302448
Ca_Kabuli_NC_021166.1_27070632	Ca_kabuli_chr07	27070632	[G/T]	1067302449
Ca_Kabuli_NC_021166.1_27070635	Ca_kabuli_chr07	27070635	[A/G]	1067302450
Ca_Kabuli_NC_021166.1_27157236	Ca_kabuli_chr07	27157236	[C/A]	1067302451
Ca_Kabuli_NC_021166.1_27176416	Ca_kabuli_chr07	27176416	[T/C]	1067302452
Ca_Kabuli_NC_021166.1_27210871	Ca_kabuli_chr07	27210871	[C/A]	1067302453
Ca_Kabuli_NC_021166.1_27210881	Ca_kabuli_chr07	27210881	[C/T]	1067302454
Ca_Kabuli_NC_021166.1_27210894	Ca_kabuli_chr07	27210894	[T/C]	1067302455
Ca_Kabuli_NC_021166.1_27210919	Ca_kabuli_chr07	27210919	[C/T]	1067302456
Ca_Kabuli_NC_021166.1_27350854	Ca_kabuli_chr07	27350854	[T/A]	1067302457
Ca_Kabuli_NC_021166.1_27419275	Ca_kabuli_chr07	27419275	[A/C]	1067302458
Ca_Kabuli_NC_021166.1_27419283	Ca_kabuli_chr07	27419283	[A/G]	1067302459
Ca_Kabuli_NC_021166.1_27420157	Ca_kabuli_chr07	27420157	[T/A]	1067302460
Ca_Kabuli_NC_021166.1_27633716	Ca_kabuli_chr07	27633716	[A/G]	1067302461
Ca_Kabuli_NC_021166.1_27633725	Ca_kabuli_chr07	27633725	[A/G]	1067302462
Ca_Kabuli_NC_021166.1_27633730	Ca_kabuli_chr07	27633730	[T/C]	1067302463
Ca_Kabuli_NC_021166.1_27633762	Ca_kabuli_chr07	27633762	[T/G]	1067302464
Ca_Kabuli_NC_021166.1_27633888	Ca_kabuli_chr07	27633888	[T/C]	1067302465
Ca_Kabuli_NC_021166.1_27869721	Ca_kabuli_chr07	27869721	[A/C]	1067302466
Ca_Kabuli_NC_021166.1_28343865	Ca_kabuli_chr07	28343865	[T/C]	1067302467
Ca_Kabuli_NC_021166.1_28442530	Ca_kabuli_chr07	28442530	[T/G]	1067302468

SNP IDs	Chromosomes	Physical positions (bp)	SNPs	NCBI SNPdb accession IDs (NCBI_ss#)
Ca_Kabuli_NC_021166.1_28442542	Ca_kabuli_chr07	28442542	[T/G]	1067302469
Ca_Kabuli_NC_021166.1_28442557	Ca_kabuli_chr07	28442557	[T/A]	1067302470
Ca_Kabuli_NC_021166.1_28442628	Ca_kabuli_chr07	28442628	[T/C]	1067302471
Ca_Kabuli_NC_021166.1_28443148	Ca_kabuli_chr07	28443148	[A/C]	1067302472
Ca_Kabuli_NC_021166.1_28443153	Ca_kabuli_chr07	28443153	[A/C]	1067302473
Ca_Kabuli_NC_021166.1_28467688	Ca_kabuli_chr07	28467688	[G/T]	1067302474
Ca_Kabuli_NC_021166.1_28520385	Ca_kabuli_chr07	28520385	[A/C]	1067302475
Ca_Kabuli_NC_021166.1_28549319	Ca_kabuli_chr07	28549319	[T/G]	1067302476
Ca_Kabuli_NC_021166.1_28577904	Ca_kabuli_chr07	28577904	[A/C]	1067302477
Ca_Kabuli_NC_021166.1_28598478	Ca_kabuli_chr07	28598478	[T/G]	1067302478
Ca_Kabuli_NC_021166.1_28613958	Ca_kabuli_chr07	28613958	[A/T]	1067302479
Ca_Kabuli_NC_021166.1_28620851	Ca_kabuli_chr07	28620851	[C/T]	1067302480
Ca_Kabuli_NC_021166.1_28655421	Ca_kabuli_chr07	28655421	[T/C]	1067302481
Ca_Kabuli_NC_021166.1_28675595	Ca_kabuli_chr07	28675595	[A/T]	1067302482
Ca_Kabuli_NC_021166.1_28931206	Ca_kabuli_chr07	28931206	[A/C]	1067302483
Ca_Kabuli_NC_021166.1_28931222	Ca_kabuli_chr07	28931222	[T/A]	1067302484
Ca_Kabuli_NC_021166.1_28931223	Ca_kabuli_chr07	28931223	[C/A]	1067302485
Ca_Kabuli_NC_021166.1_28931229	Ca_kabuli_chr07	28931229	[C/G]	1067302486
Ca_Kabuli_NC_021166.1_29109721	Ca_kabuli_chr07	29109721	[A/C]	1067302487
Ca_Kabuli_NC_021166.1_29139477	Ca_kabuli_chr07	29139477	[T/C]	1067302488
Ca_Kabuli_NC_021166.1_29139673	Ca_kabuli_chr07	29139673	[A/G]	1067302489
Ca_Kabuli_NC_021166.1_29139691	Ca_kabuli_chr07	29139691	[T/G]	1067302490
Ca_Kabuli_NC_021166.1_29148371	Ca_kabuli_chr07	29148371	[A/C]	1067302491
Ca_Kabuli_NC_021166.1_29148373	Ca_kabuli_chr07	29148373	[C/G]	1067302492
Ca_Kabuli_NC_021166.1_29150904	Ca_kabuli_chr07	29150904	[T/G]	1067302493
Ca_Kabuli_NC_021166.1_29153971	Ca_kabuli_chr07	29153971	[A/G]	1067302494
Ca_Kabuli_NC_021166.1_29154016	Ca_kabuli_chr07	29154016	[C/T]	1067302495
Ca_Kabuli_NC_021166.1_29154032	Ca_kabuli_chr07	29154032	[T/A]	1067302496
Ca_Kabuli_NC_021166.1_29190642	Ca_kabuli_chr07	29190642	[C/T]	1067302497
Ca_Kabuli_NC_021166.1_29190643	Ca_kabuli_chr07	29190643	[C/T]	1067302498
Ca_Kabuli_NC_021166.1_29190657	Ca_kabuli_chr07	29190657	[T/C]	1067302499
Ca_Kabuli_NC_021166.1_29190693	Ca_kabuli_chr07	29190693	[T/C]	1067302500
Ca_Kabuli_NC_021166.1_29190702	Ca_kabuli_chr07	29190702	[T/A]	1067302501
Ca_Kabuli_NC_021166.1_29221789	Ca_kabuli_chr07	29221789	[T/C]	1067302502

SNP IDs	Chromosomes	Physical positions (bp)	SNPs	NCBI SNPdb accession IDs (NCBI_ss#)
Ca_Kabuli_NC_021166.1_29223788	Ca_kabuli_chr07	29223788	[A/T]	1067302503
Ca_Kabuli_NC_021166.1_29225395	Ca_kabuli_chr07	29225395	[T/C]	1067302504
Ca_Kabuli_NC_021166.1_29313239	Ca_kabuli_chr07	29313239	[C/T]	1067302505
Ca_Kabuli_NC_021166.1_29313273	Ca_kabuli_chr07	29313273	[G/A]	1067302506
Ca_Kabuli_NC_021166.1_29313283	Ca_kabuli_chr07	29313283	[T/C]	1067302507
Ca_Kabuli_NC_021166.1_29473962	Ca_kabuli_chr07	29473962	[T/G]	1067302508
Ca_Kabuli_NC_021166.1_29796495	Ca_kabuli_chr07	29796495	[T/G]	1067302509
Ca_Kabuli_NC_021166.1_29798563	Ca_kabuli_chr07	29798563	[A/G]	1067302510
Ca_Kabuli_NC_021166.1_29837039	Ca_kabuli_chr07	29837039	[T/G]	1067302511
Ca_Kabuli_NC_021166.1_29841758	Ca_kabuli_chr07	29841758	[A/G]	1067302512
Ca_Kabuli_NC_021166.1_29848397	Ca_kabuli_chr07	29848397	[T/G]	1067302513
Ca_Kabuli_NC_021166.1_29849514	Ca_kabuli_chr07	29849514	[A/C]	1067302514
Ca_Kabuli_NC_021166.1_29884734	Ca_kabuli_chr07	29884734	[T/C]	1067302515
Ca_Kabuli_NC_021166.1_29884758	Ca_kabuli_chr07	29884758	[T/C]	1067302516
Ca_Kabuli_NC_021166.1_29884785	Ca_kabuli_chr07	29884785	[A/G]	1067302517
Ca_Kabuli_NC_021166.1_29884788	Ca_kabuli_chr07	29884788	[A/T]	1067302518
Ca_Kabuli_NC_021166.1_29902065	Ca_kabuli_chr07	29902065	[T/G]	1067302519
Ca_Kabuli_NC_021166.1_29924747	Ca_kabuli_chr07	29924747	[G/A]	1067302520
Ca_Kabuli_NC_021166.1_30060254	Ca_kabuli_chr07	30060254	[T/C]	1067302521
Ca_Kabuli_NC_021166.1_30060455	Ca_kabuli_chr07	30060455	[A/G]	1067302522
Ca_Kabuli_NC_021166.1_30060473	Ca_kabuli_chr07	30060473	[T/C]	1067302523
Ca_Kabuli_NC_021166.1_30114687	Ca_kabuli_chr07	30114687	[A/C]	1067302524
Ca_Kabuli_NC_021166.1_30114688	Ca_kabuli_chr07	30114688	[G/C]	1067302525
Ca_Kabuli_NC_021166.1_30118054	Ca_kabuli_chr07	30118054	[A/G]	1067302526
Ca_Kabuli_NC_021166.1_30283345	Ca_kabuli_chr07	30283345	[A/C]	1067302527
Ca_Kabuli_NC_021166.1_30340422	Ca_kabuli_chr07	30340422	[T/A]	1067302528
Ca_Kabuli_NC_021166.1_30398790	Ca_kabuli_chr07	30398790	[C/A]	1067302529
Ca_Kabuli_NC_021166.1_30691318	Ca_kabuli_chr07	30691318	[A/C]	1067302530
Ca_Kabuli_NC_021166.1_30691324	Ca_kabuli_chr07	30691324	[A/C]	1067302531
Ca_Kabuli_NC_021166.1_30810082	Ca_kabuli_chr07	30810082	[A/C]	1067302532
Ca_Kabuli_NC_021166.1_30837174	Ca_kabuli_chr07	30837174	[G/C]	1067302533
Ca_Kabuli_NC_021166.1_30837177	Ca_kabuli_chr07	30837177	[T/C]	1067302534
Ca_Kabuli_NC_021166.1_30837208	Ca_kabuli_chr07	30837208	[A/C]	1067302535
Ca_Kabuli_NC_021166.1_30850853	Ca_kabuli_chr07	30850853	[A/C]	1067302536

SNP IDs	Chromosomes	Physical positions (bp)	SNPs	NCBI SNPdb accession IDs (NCBI_ss#)
Ca_Kabuli_NC_021166.1_30850938	Ca_kabuli_chr07	30850938	[T/G]	1067302537
Ca_Kabuli_NC_021166.1_30850941	Ca_kabuli_chr07	30850941	[T/A]	1067302538
Ca_Kabuli_NC_021166.1_30850944	Ca_kabuli_chr07	30850944	[T/G]	1067302539
Ca_Kabuli_NC_021166.1_30850948	Ca_kabuli_chr07	30850948	[T/C]	1067302540
Ca_Kabuli_NC_021166.1_30850950	Ca_kabuli_chr07	30850950	[A/G]	1067302541
Ca_Kabuli_NC_021166.1_30865517	Ca_kabuli_chr07	30865517	[A/G]	1067302542
Ca_Kabuli_NC_021166.1_31125100	Ca_kabuli_chr07	31125100	[A/G]	1067302543
Ca_Kabuli_NC_021166.1_31189885	Ca_kabuli_chr07	31189885	[A/C]	1067302544
Ca_Kabuli_NC_021166.1_31190189	Ca_kabuli_chr07	31190189	[A/G]	1067302545
Ca_Kabuli_NC_021166.1_31193759	Ca_kabuli_chr07	31193759	[T/A]	1067302546
Ca_Kabuli_NC_021166.1_31193786	Ca_kabuli_chr07	31193786	[T/G]	1067302547
Ca_Kabuli_NC_021166.1_31223945	Ca_kabuli_chr07	31223945	[A/C]	1067302548
Ca_Kabuli_NC_021166.1_31223950	Ca_kabuli_chr07	31223950	[A/C]	1067302549
Ca_Kabuli_NC_021166.1_31223962	Ca_kabuli_chr07	31223962	[A/G]	1067302550
Ca_Kabuli_NC_021166.1_31223969	Ca_kabuli_chr07	31223969	[T/A]	1067302551
Ca_Kabuli_NC_021166.1_31223972	Ca_kabuli_chr07	31223972	[T/C]	1067302552
Ca_Kabuli_NC_021166.1_31254234	Ca_kabuli_chr07	31254234	[T/G]	1067302553
Ca_Kabuli_NC_021166.1_31314360	Ca_kabuli_chr07	31314360	[T/G]	1067302554
Ca_Kabuli_NC_021166.1_31314421	Ca_kabuli_chr07	31314421	[T/A]	1067302555
Ca_Kabuli_NC_021166.1_31318704	Ca_kabuli_chr07	31318704	[A/T]	1067302556
Ca_Kabuli_NC_021166.1_31318716	Ca_kabuli_chr07	31318716	[A/C]	1067302557
Ca_Kabuli_NC_021166.1_31364838	Ca_kabuli_chr07	31364838	[A/C]	1067302558
Ca_Kabuli_NC_021166.1_31364947	Ca_kabuli_chr07	31364947	[A/G]	1067302559
Ca_Kabuli_NC_021166.1_31455929	Ca_kabuli_chr07	31455929	[A/C]	1067302560
Ca_Kabuli_NC_021166.1_31457384	Ca_kabuli_chr07	31457384	[T/G]	1067302561
Ca_Kabuli_NC_021166.1_31612869	Ca_kabuli_chr07	31612869	[A/C]	1067302562
Ca_Kabuli_NC_021166.1_31635342	Ca_kabuli_chr07	31635342	[T/A]	1067302563
Ca_Kabuli_NC_021166.1_31639713	Ca_kabuli_chr07	31639713	[A/T]	1067302564
Ca_Kabuli_NC_021166.1_31759065	Ca_kabuli_chr07	31759065	[C/T]	1067302565
Ca_Kabuli_NC_021166.1_31759066	Ca_kabuli_chr07	31759066	[T/C]	1067302566
Ca_Kabuli_NC_021166.1_31804031	Ca_kabuli_chr07	31804031	[T/C]	1067302567
Ca_Kabuli_NC_021166.1_31806328	Ca_kabuli_chr07	31806328	[T/G]	1067302568
Ca_Kabuli_NC_021166.1_31825708	Ca_kabuli_chr07	31825708	[A/T]	1067302569
Ca_Kabuli_NC_021166.1_31825725	Ca_kabuli_chr07	31825725	[T/G]	1067302570

SNP IDs	Chromosomes	Physical positions (bp)	SNPs	NCBI SNPdb accession IDs (NCBI_ss#)
Ca_Kabuli_NC_021166.1_31825733	Ca_kabuli_chr07	31825733	[A/G]	1067302571
Ca_Kabuli_NC_021166.1_31825735	Ca_kabuli_chr07	31825735	[A/G]	1067302572
Ca_Kabuli_NC_021166.1_31851053	Ca_kabuli_chr07	31851053	[T/G]	1067302573
Ca_Kabuli_NC_021166.1_31851073	Ca_kabuli_chr07	31851073	[A/C]	1067302574
Ca_Kabuli_NC_021166.1_31867530	Ca_kabuli_chr07	31867530	[T/A]	1067302575
Ca_Kabuli_NC_021166.1_31867539	Ca_kabuli_chr07	31867539	[T/C]	1067302576
Ca_Kabuli_NC_021166.1_31867563	Ca_kabuli_chr07	31867563	[A/T]	1067302577
Ca_Kabuli_NC_021166.1_31873070	Ca_kabuli_chr07	31873070	[C/T]	1067302578
Ca_Kabuli_NC_021166.1_31873091	Ca_kabuli_chr07	31873091	[G/A]	1067302579
Ca_Kabuli_NC_021166.1_31881426	Ca_kabuli_chr07	31881426	[T/G]	1067302580
Ca_Kabuli_NC_021166.1_31934109	Ca_kabuli_chr07	31934109	[A/C]	1067302581
Ca_Kabuli_NC_021166.1_31960453	Ca_kabuli_chr07	31960453	[C/A]	1067302582
Ca_Kabuli_NC_021166.1_31994751	Ca_kabuli_chr07	31994751	[T/G]	1067302583
Ca_Kabuli_NC_021166.1_32242973	Ca_kabuli_chr07	32242973	[A/T]	1067302584
Ca_Kabuli_NC_021166.1_32242987	Ca_kabuli_chr07	32242987	[T/C]	1067302585
Ca_Kabuli_NC_021166.1_32271017	Ca_kabuli_chr07	32271017	[T/C]	1067302586
Ca_Kabuli_NC_021166.1_32271030	Ca_kabuli_chr07	32271030	[A/C]	1067302587
Ca_Kabuli_NC_021166.1_32274245	Ca_kabuli_chr07	32274245	[T/G]	1067302588
Ca_Kabuli_NC_021166.1_32288770	Ca_kabuli_chr07	32288770	[A/T]	1067302589
Ca_Kabuli_NC_021166.1_32309392	Ca_kabuli_chr07	32309392	[G/T]	1067302590
Ca_Kabuli_NC_021166.1_32309394	Ca_kabuli_chr07	32309394	[A/T]	1067302591
Ca_Kabuli_NC_021166.1_32309397	Ca_kabuli_chr07	32309397	[T/A]	1067302592
Ca_Kabuli_NC_021166.1_32373621	Ca_kabuli_chr07	32373621	[T/A]	1067302593
Ca_Kabuli_NC_021166.1_32373720	Ca_kabuli_chr07	32373720	[T/A]	1067302594
Ca_Kabuli_NC_021166.1_32374194	Ca_kabuli_chr07	32374194	[T/G]	1067302595
Ca_Kabuli_NC_021166.1_32382954	Ca_kabuli_chr07	32382954	[T/A]	1067302596
Ca_Kabuli_NC_021166.1_32406152	Ca_kabuli_chr07	32406152	[C/A]	1067302597
Ca_Kabuli_NC_021166.1_32406153	Ca_kabuli_chr07	32406153	[T/G]	1067302598
Ca_Kabuli_NC_021166.1_32406154	Ca_kabuli_chr07	32406154	[T/A]	1067302599
Ca_Kabuli_NC_021166.1_32406155	Ca_kabuli_chr07	32406155	[C/T]	1067302600
Ca_Kabuli_NC_021166.1_32448458	Ca_kabuli_chr07	32448458	[T/C]	1067302601
Ca_Kabuli_NC_021166.1_32448477	Ca_kabuli_chr07	32448477	[A/G]	1067302602
Ca_Kabuli_NC_021166.1_32522201	Ca_kabuli_chr07	32522201	[A/G]	1067302603
Ca_Kabuli_NC_021166.1_32522203	Ca_kabuli_chr07	32522203	[A/G]	1067302604

SNP IDs	Chromosomes	Physical positions (bp)	SNPs	NCBI SNPdb accession IDs (NCBI_ss#)
Ca_Kabuli_NC_021166.1_32522207	Ca_kabuli_chr07	32522207	[T/G]	1067302605
Ca_Kabuli_NC_021166.1_32522213	Ca_kabuli_chr07	32522213	[A/G]	1067302606
Ca_Kabuli_NC_021166.1_32522225	Ca_kabuli_chr07	32522225	[C/A]	1067302607
Ca_Kabuli_NC_021166.1_32522230	Ca_kabuli_chr07	32522230	[T/G]	1067302608
Ca_Kabuli_NC_021166.1_32522286	Ca_kabuli_chr07	32522286	[G/A]	1067302609
Ca_Kabuli_NC_021166.1_32522288	Ca_kabuli_chr07	32522288	[T/A]	1067302610
Ca_Kabuli_NC_021166.1_32753333	Ca_kabuli_chr07	32753333	[A/C]	1067302611
Ca_Kabuli_NC_021166.1_32754651	Ca_kabuli_chr07	32754651	[T/G]	1067302612
Ca_Kabuli_NC_021166.1_32755197	Ca_kabuli_chr07	32755197	[T/C]	1067302613
Ca_Kabuli_NC_021166.1_32755204	Ca_kabuli_chr07	32755204	[A/T]	1067302614
Ca_Kabuli_NC_021166.1_32755652	Ca_kabuli_chr07	32755652	[A/T]	1067302615
Ca_Kabuli_NC_021166.1_32758678	Ca_kabuli_chr07	32758678	[A/C]	1067302616
Ca_Kabuli_NC_021166.1_32782151	Ca_kabuli_chr07	32782151	[C/A]	1067302617
Ca_Kabuli_NC_021166.1_32782823	Ca_kabuli_chr07	32782823	[G/A]	1067302618
Ca_Kabuli_NC_021166.1_32784679	Ca_kabuli_chr07	32784679	[A/C]	1067302619
Ca_Kabuli_NC_021166.1_32784845	Ca_kabuli_chr07	32784845	[A/T]	1067302620
Ca_Kabuli_NC_021166.1_32784867	Ca_kabuli_chr07	32784867	[A/C]	1067302621
Ca_Kabuli_NC_021166.1_32784883	Ca_kabuli_chr07	32784883	[T/C]	1067302622
Ca_Kabuli_NC_021166.1_32784908	Ca_kabuli_chr07	32784908	[C/A]	1067302623
Ca_Kabuli_NC_021166.1_32784911	Ca_kabuli_chr07	32784911	[A/T]	1067302624
Ca_Kabuli_NC_021166.1_32784913	Ca_kabuli_chr07	32784913	[G/C]	1067302625
Ca_Kabuli_NC_021166.1_32784916	Ca_kabuli_chr07	32784916	[A/C]	1067302626
Ca_Kabuli_NC_021166.1_32784919	Ca_kabuli_chr07	32784919	[G/C]	1067302627
Ca_Kabuli_NC_021166.1_32784921	Ca_kabuli_chr07	32784921	[A/T]	1067302628
Ca_Kabuli_NC_021166.1_32784948	Ca_kabuli_chr07	32784948	[T/G]	1067302629
Ca_Kabuli_NC_021166.1_32785429	Ca_kabuli_chr07	32785429	[G/A]	1067302630
Ca_Kabuli_NC_021166.1_32785432	Ca_kabuli_chr07	32785432	[C/T]	1067302631
Ca_Kabuli_NC_021166.1_32840275	Ca_kabuli_chr07	32840275	[C/A]	1067302632
Ca_Kabuli_NC_021166.1_32875845	Ca_kabuli_chr07	32875845	[A/G]	1067302633
Ca_Kabuli_NC_021166.1_32875857	Ca_kabuli_chr07	32875857	[A/G]	1067302634
Ca_Kabuli_NC_021166.1_32875896	Ca_kabuli_chr07	32875896	[T/C]	1067302635
Ca_Kabuli_NC_021166.1_32875905	Ca_kabuli_chr07	32875905	[T/C]	1067302636
Ca_Kabuli_NC_021166.1_32942234	Ca_kabuli_chr07	32942234	[T/C]	1067302637
Ca_Kabuli_NC_021166.1_32942252	Ca_kabuli_chr07	32942252	[T/C]	1067302638

SNP IDs	Chromosomes	Physical positions (bp)	SNPs	NCBI SNPdb accession IDs (NCBI_ss#)
Ca_Kabuli_NC_021166.1_32942961	Ca_kabuli_chr07	32942961	[A/G]	1067302639
Ca_Kabuli_NC_021166.1_32942980	Ca_kabuli_chr07	32942980	[T/G]	1067302640
Ca_Kabuli_NC_021166.1_32943082	Ca_kabuli_chr07	32943082	[T/G]	1067302641
Ca_Kabuli_NC_021166.1_32943085	Ca_kabuli_chr07	32943085	[A/G]	1067302642
Ca_Kabuli_NC_021166.1_32943087	Ca_kabuli_chr07	32943087	[T/G]	1067302643
Ca_Kabuli_NC_021166.1_32943099	Ca_kabuli_chr07	32943099	[T/G]	1067302644
Ca_Kabuli_NC_021166.1_32989796	Ca_kabuli_chr07	32989796	[A/T]	1067302645
Ca_Kabuli_NC_021166.1_32989838	Ca_kabuli_chr07	32989838	[T/C]	1067302646
Ca_Kabuli_NC_021166.1_32989902	Ca_kabuli_chr07	32989902	[A/C]	1067302647
Ca_Kabuli_NC_021166.1_32989903	Ca_kabuli_chr07	32989903	[G/A]	1067302648
Ca_Kabuli_NC_021166.1_33006955	Ca_kabuli_chr07	33006955	[C/T]	1067302649
Ca_Kabuli_NC_021166.1_33026150	Ca_kabuli_chr07	33026150	[G/T]	1067302650
Ca_Kabuli_NC_021166.1_33065610	Ca_kabuli_chr07	33065610	[A/C]	1067302651
Ca_Kabuli_NC_021166.1_33099276	Ca_kabuli_chr07	33099276	[C/A]	1067302652
Ca_Kabuli_NC_021166.1_33099299	Ca_kabuli_chr07	33099299	[T/C]	1067302653
Ca_Kabuli_NC_021166.1_33099304	Ca_kabuli_chr07	33099304	[C/T]	1067302654
Ca_Kabuli_NC_021166.1_33099313	Ca_kabuli_chr07	33099313	[A/G]	1067302655
Ca_Kabuli_NC_021166.1_33099315	Ca_kabuli_chr07	33099315	[C/T]	1067302656
Ca_Kabuli_NC_021166.1_33121657	Ca_kabuli_chr07	33121657	[T/G]	1067302657
Ca_Kabuli_NC_021166.1_33121696	Ca_kabuli_chr07	33121696	[A/C]	1067302658
Ca_Kabuli_NC_021166.1_33121705	Ca_kabuli_chr07	33121705	[A/G]	1067302659
Ca_Kabuli_NC_021166.1_33122490	Ca_kabuli_chr07	33122490	[G/A]	1067302660
Ca_Kabuli_NC_021166.1_33122549	Ca_kabuli_chr07	33122549	[A/G]	1067302661
Ca_Kabuli_NC_021166.1_33140097	Ca_kabuli_chr07	33140097	[C/T]	1067302662
Ca_Kabuli_NC_021166.1_33182852	Ca_kabuli_chr07	33182852	[T/G]	1067302663
Ca_Kabuli_NC_021166.1_33182945	Ca_kabuli_chr07	33182945	[A/T]	1067302664
Ca_Kabuli_NC_021166.1_33184628	Ca_kabuli_chr07	33184628	[A/G]	1067302665
Ca_Kabuli_NC_021166.1_33184633	Ca_kabuli_chr07	33184633	[T/A]	1067302666
Ca_Kabuli_NC_021166.1_33184651	Ca_kabuli_chr07	33184651	[A/C]	1067302667
Ca_Kabuli_NC_021166.1_33184662	Ca_kabuli_chr07	33184662	[T/C]	1067302668
Ca_Kabuli_NC_021166.1_33225199	Ca_kabuli_chr07	33225199	[T/G]	1067302669
Ca_Kabuli_NC_021166.1_33262993	Ca_kabuli_chr07	33262993	[G/A]	1067302670
Ca_Kabuli_NC_021166.1_33267485	Ca_kabuli_chr07	33267485	[C/T]	1067302671
Ca_Kabuli_NC_021166.1_33267496	Ca_kabuli_chr07	33267496	[A/T]	1067302672

SNP IDs	Chromosomes	Physical positions (bp)	SNPs	NCBI SNPdb accession IDs (NCBI_ss#)
Ca_Kabuli_NC_021166.1_33336015	Ca_kabuli_chr07	33336015	[C/T]	1067302673
Ca_Kabuli_NC_021166.1_33349798	Ca_kabuli_chr07	33349798	[G/A]	1067302674
Ca_Kabuli_NC_021166.1_33358925	Ca_kabuli_chr07	33358925	[T/C]	1067302675
Ca_Kabuli_NC_021166.1_33359436	Ca_kabuli_chr07	33359436	[T/C]	1067302676
Ca_Kabuli_NC_021166.1_33359628	Ca_kabuli_chr07	33359628	[C/G]	1067302677
Ca_Kabuli_NC_021166.1_33359629	Ca_kabuli_chr07	33359629	[A/G]	1067302678
Ca_Kabuli_NC_021166.1_33364354	Ca_kabuli_chr07	33364354	[A/G]	1067302679
Ca_Kabuli_NC_021166.1_33376787	Ca_kabuli_chr07	33376787	[T/C]	1067302680
Ca_Kabuli_NC_021166.1_33376820	Ca_kabuli_chr07	33376820	[A/G]	1067302681
Ca_Kabuli_NC_021166.1_33377387	Ca_kabuli_chr07	33377387	[T/G]	1067302682
Ca_Kabuli_NC_021166.1_33377388	Ca_kabuli_chr07	33377388	[A/T]	1067302683
Ca_Kabuli_NC_021166.1_33377408	Ca_kabuli_chr07	33377408	[T/G]	1067302684
Ca_Kabuli_NC_021166.1_33378427	Ca_kabuli_chr07	33378427	[T/C]	1067302685
Ca_Kabuli_NC_021166.1_33379819	Ca_kabuli_chr07	33379819	[T/C]	1067302686
Ca_Kabuli_NC_021166.1_33379831	Ca_kabuli_chr07	33379831	[T/C]	1067302687
Ca_Kabuli_NC_021166.1_33380558	Ca_kabuli_chr07	33380558	[T/C]	1067302688
Ca_Kabuli_NC_021166.1_33380570	Ca_kabuli_chr07	33380570	[A/C]	1067302689
Ca_Kabuli_NC_021166.1_33380647	Ca_kabuli_chr07	33380647	[A/C]	1067302690
Ca_Kabuli_NC_021166.1_33380650	Ca_kabuli_chr07	33380650	[A/C]	1067302691
Ca_Kabuli_NC_021166.1_33411746	Ca_kabuli_chr07	33411746	[A/C]	1067302692
Ca_Kabuli_NC_021166.1_33460918	Ca_kabuli_chr07	33460918	[A/G]	1067302693
Ca_Kabuli_NC_021166.1_33461168	Ca_kabuli_chr07	33461168	[C/A]	1067302694
Ca_Kabuli_NC_021166.1_33463300	Ca_kabuli_chr07	33463300	[G/C]	1067302695
Ca_Kabuli_NC_021166.1_33464385	Ca_kabuli_chr07	33464385	[A/C]	1067302696
Ca_Kabuli_NC_021166.1_33465798	Ca_kabuli_chr07	33465798	[A/G]	1067302697
Ca_Kabuli_NC_021166.1_33465843	Ca_kabuli_chr07	33465843	[T/A]	1067302698
Ca_Kabuli_NC_021166.1_33468732	Ca_kabuli_chr07	33468732	[A/G]	1067302699
Ca_Kabuli_NC_021166.1_33471186	Ca_kabuli_chr07	33471186	[C/A]	1067302700
Ca_Kabuli_NC_021166.1_33480940	Ca_kabuli_chr07	33480940	[T/C]	1067302701
Ca_Kabuli_NC_021166.1_33481106	Ca_kabuli_chr07	33481106	[A/C]	1067302702
Ca_Kabuli_NC_021166.1_33481135	Ca_kabuli_chr07	33481135	[G/C]	1067302703
Ca_Kabuli_NC_021166.1_33518396	Ca_kabuli_chr07	33518396	[A/C]	1067302704
Ca_Kabuli_NC_021166.1_33573605	Ca_kabuli_chr07	33573605	[C/T]	1067302705
Ca_Kabuli_NC_021166.1_33573655	Ca_kabuli_chr07	33573655	[A/C]	1067302706

SNP IDs	Chromosomes	Physical positions (bp)	SNPs	NCBI SNPdb accession IDs (NCBI_ss#)
Ca_Kabuli_NC_021166.1_33586461	Ca_kabuli_chr07	33586461	[A/G]	1067302707
Ca_Kabuli_NC_021166.1_33586618	Ca_kabuli_chr07	33586618	[A/G]	1067302708
Ca_Kabuli_NC_021166.1_33641675	Ca_kabuli_chr07	33641675	[A/G]	1067302709
Ca_Kabuli_NC_021166.1_33641689	Ca_kabuli_chr07	33641689	[T/C]	1067302710
Ca_Kabuli_NC_021166.1_33641728	Ca_kabuli_chr07	33641728	[T/C]	1067302711
Ca_Kabuli_NC_021166.1_33641799	Ca_kabuli_chr07	33641799	[T/G]	1067302712
Ca_Kabuli_NC_021166.1_33641882	Ca_kabuli_chr07	33641882	[A/T]	1067302713
Ca_Kabuli_NC_021166.1_33674454	Ca_kabuli_chr07	33674454	[C/T]	1067302714
Ca_Kabuli_NC_021166.1_33674838	Ca_kabuli_chr07	33674838	[C/T]	1067302715
Ca_Kabuli_NC_021166.1_33740809	Ca_kabuli_chr07	33740809	[A/G]	1067302716
Ca_Kabuli_NC_021166.1_33750773	Ca_kabuli_chr07	33750773	[A/T]	1067302717
Ca_Kabuli_NC_021166.1_33751162	Ca_kabuli_chr07	33751162	[T/A]	1067302718
Ca_Kabuli_NC_021166.1_33753917	Ca_kabuli_chr07	33753917	[A/G]	1067302719
Ca_Kabuli_NC_021166.1_33761944	Ca_kabuli_chr07	33761944	[A/G]	1067302720
Ca_Kabuli_NC_021166.1_33762053	Ca_kabuli_chr07	33762053	[T/G]	1067302721
Ca_Kabuli_NC_021166.1_33809459	Ca_kabuli_chr07	33809459	[A/G]	1067302722
Ca_Kabuli_NC_021166.1_33835856	Ca_kabuli_chr07	33835856	[G/A]	1067302723
Ca_Kabuli_NC_021166.1_33865589	Ca_kabuli_chr07	33865589	[C/G]	1067302724
Ca_Kabuli_NC_021166.1_33867334	Ca_kabuli_chr07	33867334	[C/T]	1067302725
Ca_Kabuli_NC_021166.1_33867491	Ca_kabuli_chr07	33867491	[T/C]	1067302726
Ca_Kabuli_NC_021166.1_33867674	Ca_kabuli_chr07	33867674	[T/C]	1067302727
Ca_Kabuli_NC_021166.1_33868148	Ca_kabuli_chr07	33868148	[C/T]	1067302728
Ca_Kabuli_NC_021166.1_33869361	Ca_kabuli_chr07	33869361	[A/G]	1067302729
Ca_Kabuli_NC_021166.1_33869373	Ca_kabuli_chr07	33869373	[A/C]	1067302730
Ca_Kabuli_NC_021166.1_33869399	Ca_kabuli_chr07	33869399	[A/G]	1067302731
Ca_Kabuli_NC_021166.1_33869402	Ca_kabuli_chr07	33869402	[G/T]	1067302732
Ca_Kabuli_NC_021166.1_33869404	Ca_kabuli_chr07	33869404	[T/C]	1067302733
Ca_Kabuli_NC_021166.1_33869412	Ca_kabuli_chr07	33869412	[A/G]	1067302734
Ca_Kabuli_NC_021166.1_33869423	Ca_kabuli_chr07	33869423	[T/C]	1067302735
Ca_Kabuli_NC_021166.1_33879017	Ca_kabuli_chr07	33879017	[T/A]	1067302736
Ca_Kabuli_NC_021166.1_33890956	Ca_kabuli_chr07	33890956	[A/C]	1067302737
Ca_Kabuli_NC_021166.1_33910873	Ca_kabuli_chr07	33910873	[A/G]	1067302738
Ca_Kabuli_NC_021166.1_33910883	Ca_kabuli_chr07	33910883	[A/T]	1067302739
Ca_Kabuli_NC_021166.1_33910925	Ca_kabuli_chr07	33910925	[A/G]	1067302740

SNP IDs	Chromosomes	Physical positions (bp)	SNPs	NCBI SNPdb accession IDs (NCBI_ss#)
Ca_Kabuli_NC_021166.1_33912866	Ca_kabuli_chr07	33912866	[C/T]	1067302741
Ca_Kabuli_NC_021166.1_33912884	Ca_kabuli_chr07	33912884	[C/A]	1067302742
Ca_Kabuli_NC_021166.1_33912896	Ca_kabuli_chr07	33912896	[T/A]	1067302743
Ca_Kabuli_NC_021166.1_33912899	Ca_kabuli_chr07	33912899	[A/T]	1067302744
Ca_Kabuli_NC_021166.1_33912902	Ca_kabuli_chr07	33912902	[A/T]	1067302745
Ca_Kabuli_NC_021166.1_33912913	Ca_kabuli_chr07	33912913	[A/T]	1067302746
Ca_Kabuli_NC_021166.1_33912916	Ca_kabuli_chr07	33912916	[G/T]	1067302747
Ca_Kabuli_NC_021166.1_33912921	Ca_kabuli_chr07	33912921	[T/G]	1067302748
Ca_Kabuli_NC_021166.1_34139693	Ca_kabuli_chr07	34139693	[T/C]	1067302749
Ca_Kabuli_NC_021166.1_34145280	Ca_kabuli_chr07	34145280	[T/G]	1067302750
Ca_Kabuli_NC_021166.1_34146467	Ca_kabuli_chr07	34146467	[C/A]	1067302751
Ca_Kabuli_NC_021166.1_34146468	Ca_kabuli_chr07	34146468	[A/G]	1067302752
Ca_Kabuli_NC_021166.1_34146469	Ca_kabuli_chr07	34146469	[T/A]	1067302753
Ca_Kabuli_NC_021166.1_34146471	Ca_kabuli_chr07	34146471	[A/C]	1067302754
Ca_Kabuli_NC_021166.1_34146873	Ca_kabuli_chr07	34146873	[A/G]	1067302755
Ca_Kabuli_NC_021166.1_34146935	Ca_kabuli_chr07	34146935	[T/C]	1067302756
Ca_Kabuli_NC_021166.1_34147313	Ca_kabuli_chr07	34147313	[T/G]	1067302757
Ca_Kabuli_NC_021166.1_34152655	Ca_kabuli_chr07	34152655	[G/C]	1067302758
Ca_Kabuli_NC_021166.1_34152720	Ca_kabuli_chr07	34152720	[G/T]	1067302759
Ca_Kabuli_NC_021166.1_34193321	Ca_kabuli_chr07	34193321	[A/C]	1067302760
Ca_Kabuli_NC_021166.1_34196923	Ca_kabuli_chr07	34196923	[T/G]	1067302761
Ca_Kabuli_NC_021166.1_34196938	Ca_kabuli_chr07	34196938	[T/A]	1067302762
Ca_Kabuli_NC_021166.1_34208319	Ca_kabuli_chr07	34208319	[A/G]	1067302763
Ca_Kabuli_NC_021166.1_34235506	Ca_kabuli_chr07	34235506	[C/A]	1067302764
Ca_Kabuli_NC_021166.1_34235530	Ca_kabuli_chr07	34235530	[G/C]	1067302765
Ca_Kabuli_NC_021166.1_34277010	Ca_kabuli_chr07	34277010	[A/G]	1067302766
Ca_Kabuli_NC_021166.1_34277014	Ca_kabuli_chr07	34277014	[G/T]	1067302767
Ca_Kabuli_NC_021166.1_34279357	Ca_kabuli_chr07	34279357	[T/G]	1067302768
Ca_Kabuli_NC_021166.1_34279715	Ca_kabuli_chr07	34279715	[C/A]	1067302769
Ca_Kabuli_NC_021166.1_34279725	Ca_kabuli_chr07	34279725	[G/A]	1067302770
Ca_Kabuli_NC_021166.1_34318860	Ca_kabuli_chr07	34318860	[T/C]	1067302771
Ca_Kabuli_NC_021166.1_34318862	Ca_kabuli_chr07	34318862	[G/A]	1067302772
Ca_Kabuli_NC_021166.1_34376080	Ca_kabuli_chr07	34376080	[T/A]	1067302773
Ca_Kabuli_NC_021166.1_34474614	Ca_kabuli_chr07	34474614	[A/C]	1067302774

SNP IDs	Chromosomes	Physical positions (bp)	SNPs	NCBI SNPdb accession IDs (NCBI_ss#)
Ca_Kabuli_NC_021166.1_34474627	Ca_kabuli_chr07	34474627	[A/C]	1067302775
Ca_Kabuli_NC_021166.1_34808498	Ca_kabuli_chr07	34808498	[G/A]	1067302776
Ca_Kabuli_NC_021166.1_34857654	Ca_kabuli_chr07	34857654	[G/T]	1067302777
Ca_Kabuli_NC_021166.1_34875605	Ca_kabuli_chr07	34875605	[A/T]	1067302778
Ca_Kabuli_NC_021166.1_34875630	Ca_kabuli_chr07	34875630	[A/T]	1067302779
Ca_Kabuli_NC_021166.1_34894278	Ca_kabuli_chr07	34894278	[A/C]	1067302780
Ca_Kabuli_NC_021166.1_34947544	Ca_kabuli_chr07	34947544	[A/C]	1067302781
Ca_Kabuli_NC_021166.1_34947545	Ca_kabuli_chr07	34947545	[C/A]	1067302782
Ca_Kabuli_NC_021166.1_35037530	Ca_kabuli_chr07	35037530	[T/C]	1067302783
Ca_Kabuli_NC_021166.1_35037583	Ca_kabuli_chr07	35037583	[A/T]	1067302784
Ca_Kabuli_NC_021166.1_35037616	Ca_kabuli_chr07	35037616	[A/G]	1067302785
Ca_Kabuli_NC_021166.1_35037644	Ca_kabuli_chr07	35037644	[C/T]	1067302786
Ca_Kabuli_NC_021166.1_35038242	Ca_kabuli_chr07	35038242	[T/C]	1067302787
Ca_Kabuli_NC_021166.1_35038263	Ca_kabuli_chr07	35038263	[A/C]	1067302788
Ca_Kabuli_NC_021166.1_35057540	Ca_kabuli_chr07	35057540	[A/G]	1067302789
Ca_Kabuli_NC_021166.1_35112785	Ca_kabuli_chr07	35112785	[C/T]	1067302790
Ca_Kabuli_NC_021166.1_35112935	Ca_kabuli_chr07	35112935	[A/C]	1067302791
Ca_Kabuli_NC_021166.1_35114075	Ca_kabuli_chr07	35114075	[A/C]	1067302792
Ca_Kabuli_NC_021166.1_35134752	Ca_kabuli_chr07	35134752	[A/C]	1067302793
Ca_Kabuli_NC_021166.1_35134809	Ca_kabuli_chr07	35134809	[A/C]	1067302794
Ca_Kabuli_NC_021166.1_35134816	Ca_kabuli_chr07	35134816	[A/C]	1067302795
Ca_Kabuli_NC_021166.1_35134868	Ca_kabuli_chr07	35134868	[A/G]	1067302796
Ca_Kabuli_NC_021166.1_35134887	Ca_kabuli_chr07	35134887	[T/A]	1067302797
Ca_Kabuli_NC_021166.1_35134894	Ca_kabuli_chr07	35134894	[T/G]	1067302798
Ca_Kabuli_NC_021166.1_35181514	Ca_kabuli_chr07	35181514	[A/T]	1067302799
Ca_Kabuli_NC_021166.1_35181660	Ca_kabuli_chr07	35181660	[A/G]	1067302800
Ca_Kabuli_NC_021166.1_35181661	Ca_kabuli_chr07	35181661	[C/T]	1067302801
Ca_Kabuli_NC_021166.1_35181884	Ca_kabuli_chr07	35181884	[G/A]	1067302802
Ca_Kabuli_NC_021166.1_35220113	Ca_kabuli_chr07	35220113	[C/A]	1067302803
Ca_Kabuli_NC_021166.1_35227794	Ca_kabuli_chr07	35227794	[C/A]	1067302804
Ca_Kabuli_NC_021166.1_35239021	Ca_kabuli_chr07	35239021	[A/G]	1067302805
Ca_Kabuli_NC_021166.1_35239116	Ca_kabuli_chr07	35239116	[C/T]	1067302806
Ca_Kabuli_NC_021166.1_35239702	Ca_kabuli_chr07	35239702	[T/C]	1067302807
Ca_Kabuli_NC_021166.1_35241171	Ca_kabuli_chr07	35241171	[A/T]	1067302808

SNP IDs	Chromosomes	Physical positions (bp)	SNPs	NCBI SNPdb accession IDs (NCBI_ss#)
Ca_Kabuli_NC_021166.1_35258029	Ca_kabuli_chr07	35258029	[T/G]	1067302809
Ca_Kabuli_NC_021166.1_35263678	Ca_kabuli_chr07	35263678	[T/G]	1067302810
Ca_Kabuli_NC_021166.1_35263711	Ca_kabuli_chr07	35263711	[A/T]	1067302811
Ca_Kabuli_NC_021166.1_35263712	Ca_kabuli_chr07	35263712	[G/T]	1067302812
Ca_Kabuli_NC_021166.1_35263736	Ca_kabuli_chr07	35263736	[C/G]	1067302813
Ca_Kabuli_NC_021166.1_35263758	Ca_kabuli_chr07	35263758	[A/G]	1067302814
Ca_Kabuli_NC_021166.1_35274680	Ca_kabuli_chr07	35274680	[A/G]	1067302815
Ca_Kabuli_NC_021166.1_35274858	Ca_kabuli_chr07	35274858	[A/G]	1067302816
Ca_Kabuli_NC_021166.1_35274859	Ca_kabuli_chr07	35274859	[G/T]	1067302817
Ca_Kabuli_NC_021166.1_35278840	Ca_kabuli_chr07	35278840	[T/C]	1067302818
Ca_Kabuli_NC_021166.1_35280018	Ca_kabuli_chr07	35280018	[C/A]	1067302819
Ca_Kabuli_NC_021166.1_35280031	Ca_kabuli_chr07	35280031	[A/C]	1067302820
Ca_Kabuli_NC_021166.1_35280174	Ca_kabuli_chr07	35280174	[T/G]	1067302821
Ca_Kabuli_NC_021166.1_35337101	Ca_kabuli_chr07	35337101	[A/T]	1067302822
Ca_Kabuli_NC_021166.1_35339078	Ca_kabuli_chr07	35339078	[C/T]	1067302823
Ca_Kabuli_NC_021166.1_35340960	Ca_kabuli_chr07	35340960	[A/G]	1067302824
Ca_Kabuli_NC_021166.1_35342149	Ca_kabuli_chr07	35342149	[T/C]	1067302825
Ca_Kabuli_NC_021166.1_35342151	Ca_kabuli_chr07	35342151	[A/G]	1067302826
Ca_Kabuli_NC_021166.1_35342159	Ca_kabuli_chr07	35342159	[A/C]	1067302827
Ca_Kabuli_NC_021166.1_35342174	Ca_kabuli_chr07	35342174	[A/C]	1067302828
Ca_Kabuli_NC_021166.1_35342193	Ca_kabuli_chr07	35342193	[T/C]	1067302829
Ca_Kabuli_NC_021166.1_35342258	Ca_kabuli_chr07	35342258	[C/A]	1067302830
Ca_Kabuli_NC_021166.1_35518089	Ca_kabuli_chr07	35518089	[T/C]	1067302831
Ca_Kabuli_NC_021166.1_35518091	Ca_kabuli_chr07	35518091	[T/C]	1067302832
Ca_Kabuli_NC_021166.1_35518120	Ca_kabuli_chr07	35518120	[T/C]	1067302833
Ca_Kabuli_NC_021166.1_35611764	Ca_kabuli_chr07	35611764	[A/C]	1067302834
Ca_Kabuli_NC_021166.1_35679419	Ca_kabuli_chr07	35679419	[A/G]	1067302835
Ca_Kabuli_NC_021166.1_35679425	Ca_kabuli_chr07	35679425	[T/C]	1067302836
Ca_Kabuli_NC_021166.1_35680423	Ca_kabuli_chr07	35680423	[A/C]	1067302837
Ca_Kabuli_NC_021166.1_35680438	Ca_kabuli_chr07	35680438	[A/T]	1067302838
Ca_Kabuli_NC_021166.1_35796837	Ca_kabuli_chr07	35796837	[T/A]	1067302839
Ca_Kabuli_NC_021166.1_35812930	Ca_kabuli_chr07	35812930	[A/T]	1067302840
Ca_Kabuli_NC_021166.1_35812973	Ca_kabuli_chr07	35812973	[A/G]	1067302841
Ca_Kabuli_NC_021166.1_35812980	Ca_kabuli_chr07	35812980	[T/C]	1067302842

SNP IDs	Chromosomes	Physical positions (bp)	SNPs	NCBI SNPdb accession IDs (NCBI_ss#)
Ca_Kabuli_NC_021166.1_35871940	Ca_kabuli_chr07	35871940	[G/A]	1067302843
Ca_Kabuli_NC_021166.1_35871942	Ca_kabuli_chr07	35871942	[T/G]	1067302844
Ca_Kabuli_NC_021166.1_35871945	Ca_kabuli_chr07	35871945	[G/A]	1067302845
Ca_Kabuli_NC_021166.1_35871946	Ca_kabuli_chr07	35871946	[G/A]	1067302846
Ca_Kabuli_NC_021166.1_35882441	Ca_kabuli_chr07	35882441	[A/C]	1067302847
Ca_Kabuli_NC_021166.1_35906524	Ca_kabuli_chr07	35906524	[A/G]	1067302848
Ca_Kabuli_NC_021166.1_35911617	Ca_kabuli_chr07	35911617	[A/T]	1067302849
Ca_Kabuli_NC_021166.1_35911618	Ca_kabuli_chr07	35911618	[T/C]	1067302850
Ca_Kabuli_NC_021166.1_35911640	Ca_kabuli_chr07	35911640	[C/T]	1067302851
Ca_Kabuli_NC_021166.1_35936571	Ca_kabuli_chr07	35936571	[A/T]	1067302852
Ca_Kabuli_NC_021166.1_36003795	Ca_kabuli_chr07	36003795	[T/C]	1067302853
Ca_Kabuli_NC_021166.1_36100165	Ca_kabuli_chr07	36100165	[A/G]	1067302854
Ca_Kabuli_NC_021166.1_36217274	Ca_kabuli_chr07	36217274	[T/C]	1067302855
Ca_Kabuli_NC_021166.1_36410034	Ca_kabuli_chr07	36410034	[A/G]	1067302856
Ca_Kabuli_NC_021166.1_36410038	Ca_kabuli_chr07	36410038	[A/C]	1067302857
Ca_Kabuli_NC_021166.1_36550294	Ca_kabuli_chr07	36550294	[A/C]	1067302858
Ca_Kabuli_NC_021166.1_36709445	Ca_kabuli_chr07	36709445	[A/G]	1067302859
Ca_Kabuli_NC_021166.1_36709493	Ca_kabuli_chr07	36709493	[C/T]	1067302860
Ca_Kabuli_NC_021166.1_36709495	Ca_kabuli_chr07	36709495	[T/C]	1067302861
Ca_Kabuli_NC_021166.1_36709527	Ca_kabuli_chr07	36709527	[A/T]	1067302862
Ca_Kabuli_NC_021166.1_36709533	Ca_kabuli_chr07	36709533	[T/C]	1067302863
Ca_Kabuli_NC_021166.1_36710055	Ca_kabuli_chr07	36710055	[T/C]	1067302864
Ca_Kabuli_NC_021166.1_36710085	Ca_kabuli_chr07	36710085	[T/G]	1067302865
Ca_Kabuli_NC_021166.1_36711807	Ca_kabuli_chr07	36711807	[T/G]	1067302866
Ca_Kabuli_NC_021166.1_36711935	Ca_kabuli_chr07	36711935	[T/C]	1067302867
Ca_Kabuli_NC_021166.1_36713848	Ca_kabuli_chr07	36713848	[C/A]	1067302868
Ca_Kabuli_NC_021166.1_36713851	Ca_kabuli_chr07	36713851	[A/T]	1067302869
Ca_Kabuli_NC_021166.1_36713871	Ca_kabuli_chr07	36713871	[A/G]	1067302870
Ca_Kabuli_NC_021166.1_36713875	Ca_kabuli_chr07	36713875	[A/G]	1067302871
Ca_Kabuli_NC_021166.1_36713913	Ca_kabuli_chr07	36713913	[G/T]	1067302872
Ca_Kabuli_NC_021166.1_36788364	Ca_kabuli_chr07	36788364	[A/G]	1067302873
Ca_Kabuli_NC_021166.1_36810534	Ca_kabuli_chr07	36810534	[A/C]	1067302874
Ca_Kabuli_NC_021166.1_36855845	Ca_kabuli_chr07	36855845	[T/A]	1067302875
Ca_Kabuli_NC_021166.1_36855926	Ca_kabuli_chr07	36855926	[A/G]	1067302876

SNP IDs	Chromosomes	Physical positions (bp)	SNPs	NCBI SNPdb accession IDs (NCBI_ss#)
Ca_Kabuli_NC_021166.1_36915494	Ca_kabuli_chr07	36915494	[A/T]	1067302877
Ca_Kabuli_NC_021166.1_36915515	Ca_kabuli_chr07	36915515	[T/G]	1067302878
Ca_Kabuli_NC_021166.1_36922219	Ca_kabuli_chr07	36922219	[T/G]	1067302879
Ca_Kabuli_NC_021166.1_36935003	Ca_kabuli_chr07	36935003	[C/G]	1067302880
Ca_Kabuli_NC_021166.1_36998096	Ca_kabuli_chr07	36998096	[T/C]	1067302881
Ca_Kabuli_NC_021166.1_37025057	Ca_kabuli_chr07	37025057	[T/G]	1067302882
Ca_Kabuli_NC_021166.1_37058940	Ca_kabuli_chr07	37058940	[T/A]	1067302883
Ca_Kabuli_NC_021166.1_37059029	Ca_kabuli_chr07	37059029	[A/C]	1067302884
Ca_Kabuli_NC_021166.1_37626805	Ca_kabuli_chr07	37626805	[A/G]	1067302885
Ca_Kabuli_NC_021166.1_37626809	Ca_kabuli_chr07	37626809	[T/A]	1067302886
Ca_Kabuli_NC_021166.1_37672200	Ca_kabuli_chr07	37672200	[T/C]	1067302887
Ca_Kabuli_NC_021166.1_37672202	Ca_kabuli_chr07	37672202	[T/C]	1067302888
Ca_Kabuli_NC_021166.1_37672209	Ca_kabuli_chr07	37672209	[T/C]	1067302889
Ca_Kabuli_NC_021166.1_37672245	Ca_kabuli_chr07	37672245	[T/C]	1067302890
Ca_Kabuli_NC_021166.1_37684396	Ca_kabuli_chr07	37684396	[C/A]	1067302891
Ca_Kabuli_NC_021166.1_37687101	Ca_kabuli_chr07	37687101	[T/G]	1067302892
Ca_Kabuli_NC_021166.1_37886451	Ca_kabuli_chr07	37886451	[A/T]	1067302893
Ca_Kabuli_NC_021166.1_37965743	Ca_kabuli_chr07	37965743	[A/C]	1067302894
Ca_Kabuli_NC_021166.1_38001887	Ca_kabuli_chr07	38001887	[T/C]	1067302895
Ca_Kabuli_NC_021166.1_38001896	Ca_kabuli_chr07	38001896	[A/G]	1067302896
Ca_Kabuli_NC_021166.1_38001929	Ca_kabuli_chr07	38001929	[A/G]	1067302897
Ca_Kabuli_NC_021166.1_38135360	Ca_kabuli_chr07	38135360	[A/C]	1067302898
Ca_Kabuli_NC_021166.1_38143361	Ca_kabuli_chr07	38143361	[G/A]	1067302899
Ca_Kabuli_NC_021166.1_38143524	Ca_kabuli_chr07	38143524	[T/A]	1067302900
Ca_Kabuli_NC_021166.1_38150928	Ca_kabuli_chr07	38150928	[A/G]	1067302901
Ca_Kabuli_NC_021166.1_38238608	Ca_kabuli_chr07	38238608	[T/G]	1067302902
Ca_Kabuli_NC_021166.1_38283909	Ca_kabuli_chr07	38283909	[C/T]	1067302903
Ca_Kabuli_NC_021166.1_38409102	Ca_kabuli_chr07	38409102	[A/T]	1067302904
Ca_Kabuli_NC_021166.1_38431504	Ca_kabuli_chr07	38431504	[T/A]	1067302905
Ca_Kabuli_NC_021166.1_38592283	Ca_kabuli_chr07	38592283	[G/C]	1067302906
Ca_Kabuli_NC_021166.1_38592284	Ca_kabuli_chr07	38592284	[G/T]	1067302907
Ca_Kabuli_NC_021166.1_38772627	Ca_kabuli_chr07	38772627	[G/T]	1067302908
Ca_Kabuli_NC_021166.1_38821803	Ca_kabuli_chr07	38821803	[G/T]	1067302909
Ca_Kabuli_NC_021166.1_38821812	Ca_kabuli_chr07	38821812	[T/G]	1067302910

SNP IDs	Chromosomes	Physical positions (bp)	SNPs	NCBI SNPdb accession IDs (NCBI_ss#)
Ca_Kabuli_NC_021166.1_39095458	Ca_kabuli_chr07	39095458	[A/G]	1067302911
Ca_Kabuli_NC_021166.1_39095466	Ca_kabuli_chr07	39095466	[A/C]	1067302912
Ca_Kabuli_NC_021166.1_39182533	Ca_kabuli_chr07	39182533	[T/G]	1067302913
Ca_Kabuli_NC_021166.1_39185403	Ca_kabuli_chr07	39185403	[T/G]	1067302914
Ca_Kabuli_NC_021166.1_39214268	Ca_kabuli_chr07	39214268	[C/A]	1067302915
Ca_Kabuli_NC_021166.1_39214269	Ca_kabuli_chr07	39214269	[T/G]	1067302916
Ca_Kabuli_NC_021166.1_39214270	Ca_kabuli_chr07	39214270	[G/A]	1067302917
Ca_Kabuli_NC_021166.1_39214271	Ca_kabuli_chr07	39214271	[A/T]	1067302918
Ca_Kabuli_NC_021166.1_39214272	Ca_kabuli_chr07	39214272	[T/C]	1067302919
Ca_Kabuli_NC_021166.1_39215293	Ca_kabuli_chr07	39215293	[A/C]	1067302920
Ca_Kabuli_NC_021166.1_39218870	Ca_kabuli_chr07	39218870	[T/G]	1067302921
Ca_Kabuli_NC_021166.1_39218902	Ca_kabuli_chr07	39218902	[A/G]	1067302922
Ca_Kabuli_NC_021166.1_39235233	Ca_kabuli_chr07	39235233	[A/G]	1067302923
Ca_Kabuli_NC_021166.1_39235256	Ca_kabuli_chr07	39235256	[A/C]	1067302924
Ca_Kabuli_NC_021166.1_39235259	Ca_kabuli_chr07	39235259	[T/C]	1067302925
Ca_Kabuli_NC_021166.1_39235271	Ca_kabuli_chr07	39235271	[A/C]	1067302926
Ca_Kabuli_NC_021166.1_39235277	Ca_kabuli_chr07	39235277	[T/A]	1067302927
Ca_Kabuli_NC_021166.1_39235283	Ca_kabuli_chr07	39235283	[T/C]	1067302928
Ca_Kabuli_NC_021166.1_39339502	Ca_kabuli_chr07	39339502	[C/A]	1067302929
Ca_Kabuli_NC_021166.1_39339508	Ca_kabuli_chr07	39339508	[G/A]	1067302930
Ca_Kabuli_NC_021166.1_39467351	Ca_kabuli_chr07	39467351	[A/T]	1067302931
Ca_Kabuli_NC_021166.1_39467361	Ca_kabuli_chr07	39467361	[A/C]	1067302932
Ca_Kabuli_NC_021166.1_39467392	Ca_kabuli_chr07	39467392	[A/T]	1067302933
Ca_Kabuli_NC_021166.1_39470615	Ca_kabuli_chr07	39470615	[A/C]	1067302934
Ca_Kabuli_NC_021166.1_39470653	Ca_kabuli_chr07	39470653	[T/C]	1067302935
Ca_Kabuli_NC_021166.1_39534514	Ca_kabuli_chr07	39534514	[T/C]	1067302936
Ca_Kabuli_NC_021166.1_39543936	Ca_kabuli_chr07	39543936	[A/T]	1067302937
Ca_Kabuli_NC_021166.1_39680148	Ca_kabuli_chr07	39680148	[T/C]	1067302938
Ca_Kabuli_NC_021166.1_39680167	Ca_kabuli_chr07	39680167	[A/G]	1067302939
Ca_Kabuli_NC_021166.1_39768140	Ca_kabuli_chr07	39768140	[A/C]	1067302940
Ca_Kabuli_NC_021166.1_39768188	Ca_kabuli_chr07	39768188	[A/C]	1067302941
Ca_Kabuli_NC_021166.1_39944409	Ca_kabuli_chr07	39944409	[A/T]	1067302942
Ca_Kabuli_NC_021166.1_39944414	Ca_kabuli_chr07	39944414	[A/G]	1067302943
Ca_Kabuli_NC_021166.1_39944417	Ca_kabuli_chr07	39944417	[T/C]	1067302944

SNP IDs	Chromosomes	Physical positions (bp)	SNPs	NCBI SNPdb accession IDs (NCBI_ss#)
Ca_Kabuli_NC_021166.1_39944446	Ca_kabuli_chr07	39944446	[A/G]	1067302945
Ca_Kabuli_NC_021166.1_39944462	Ca_kabuli_chr07	39944462	[A/G]	1067302946
Ca_Kabuli_NC_021166.1_39944471	Ca_kabuli_chr07	39944471	[T/C]	1067302947
Ca_Kabuli_NC_021166.1_39944892	Ca_kabuli_chr07	39944892	[T/C]	1067302948
Ca_Kabuli_NC_021166.1_39944915	Ca_kabuli_chr07	39944915	[A/T]	1067302949
Ca_Kabuli_NC_021166.1_39944935	Ca_kabuli_chr07	39944935	[A/G]	1067302950
Ca_Kabuli_NC_021166.1_40170381	Ca_kabuli_chr07	40170381	[T/C]	1067302951
Ca_Kabuli_NC_021166.1_40183398	Ca_kabuli_chr07	40183398	[A/G]	1067302952
Ca_Kabuli_NC_021166.1_40241660	Ca_kabuli_chr07	40241660	[T/G]	1067302953
Ca_Kabuli_NC_021166.1_40243859	Ca_kabuli_chr07	40243859	[A/G]	1067302954
Ca_Kabuli_NC_021166.1_40287221	Ca_kabuli_chr07	40287221	[A/G]	1067302955
Ca_Kabuli_NC_021166.1_40288114	Ca_kabuli_chr07	40288114	[A/G]	1067302956
Ca_Kabuli_NC_021166.1_40305146	Ca_kabuli_chr07	40305146	[T/G]	1067302957
Ca_Kabuli_NC_021166.1_40340134	Ca_kabuli_chr07	40340134	[C/T]	1067302958
Ca_Kabuli_NC_021166.1_40482387	Ca_kabuli_chr07	40482387	[A/G]	1067302959
Ca_Kabuli_NC_021166.1_40482415	Ca_kabuli_chr07	40482415	[A/C]	1067302960
Ca_Kabuli_NC_021166.1_40544297	Ca_kabuli_chr07	40544297	[T/C]	1067302961
Ca_Kabuli_NC_021166.1_40594867	Ca_kabuli_chr07	40594867	[T/G]	1067302962
Ca_Kabuli_NC_021166.1_40692817	Ca_kabuli_chr07	40692817	[C/G]	1067302963
Ca_Kabuli_NC_021166.1_40692822	Ca_kabuli_chr07	40692822	[T/G]	1067302964
Ca_Kabuli_NC_021166.1_41219359	Ca_kabuli_chr07	41219359	[T/G]	1067302965
Ca_Kabuli_NC_021166.1_41325463	Ca_kabuli_chr07	41325463	[A/C]	1067302966
Ca_Kabuli_NC_021166.1_41339710	Ca_kabuli_chr07	41339710	[T/G]	1067302967
Ca_Kabuli_NC_021166.1_41387499	Ca_kabuli_chr07	41387499	[T/C]	1067302968
Ca_Kabuli_NC_021166.1_41387502	Ca_kabuli_chr07	41387502	[A/C]	1067302969
Ca_Kabuli_NC_021166.1_41425049	Ca_kabuli_chr07	41425049	[A/G]	1067302970
Ca_Kabuli_NC_021166.1_41657052	Ca_kabuli_chr07	41657052	[A/C]	1067302971
Ca_Kabuli_NC_021166.1_41658915	Ca_kabuli_chr07	41658915	[A/C]	1067302972
Ca_Kabuli_NC_021166.1_41673164	Ca_kabuli_chr07	41673164	[T/G]	1067302973
Ca_Kabuli_NC_021166.1_41673203	Ca_kabuli_chr07	41673203	[A/G]	1067302974
Ca_Kabuli_NC_021166.1_41673207	Ca_kabuli_chr07	41673207	[T/G]	1067302975
Ca_Kabuli_NC_021166.1_41890361	Ca_kabuli_chr07	41890361	[T/G]	1067302976
Ca_Kabuli_NC_021166.1_41890404	Ca_kabuli_chr07	41890404	[G/A]	1067302977
Ca_Kabuli_NC_021166.1_41936373	Ca_kabuli_chr07	41936373	[T/A]	1067302978

SNP IDs	Chromosomes	Physical positions (bp)	SNPs	NCBI SNPdb accession IDs (NCBI_ss#)
Ca_Kabuli_NC_021166.1_42094505	Ca_kabuli_chr07	42094505	[G/T]	1067302979
Ca_Kabuli_NC_021166.1_42106185	Ca_kabuli_chr07	42106185	[C/T]	1067302980
Ca_Kabuli_NC_021166.1_42106186	Ca_kabuli_chr07	42106186	[C/T]	1067302981
Ca_Kabuli_NC_021166.1_42106190	Ca_kabuli_chr07	42106190	[T/G]	1067302982
Ca_Kabuli_NC_021166.1_42272740	Ca_kabuli_chr07	42272740	[T/G]	1067302983
Ca_Kabuli_NC_021166.1_42272939	Ca_kabuli_chr07	42272939	[G/C]	1067302984
Ca_Kabuli_NC_021166.1_42915851	Ca_kabuli_chr07	42915851	[A/G]	1067302985
Ca_Kabuli_NC_021166.1_42915997	Ca_kabuli_chr07	42915997	[G/T]	1067302986
Ca_Kabuli_NC_021166.1_42916031	Ca_kabuli_chr07	42916031	[T/G]	1067302987
Ca_Kabuli_NC_021166.1_42949489	Ca_kabuli_chr07	42949489	[A/G]	1067302988
Ca_Kabuli_NC_021166.1_42949492	Ca_kabuli_chr07	42949492	[A/G]	1067302989
Ca_Kabuli_NC_021166.1_42949497	Ca_kabuli_chr07	42949497	[T/G]	1067302990
Ca_Kabuli_NC_021166.1_42949509	Ca_kabuli_chr07	42949509	[T/G]	1067302991
Ca_Kabuli_NC_021166.1_42964218	Ca_kabuli_chr07	42964218	[T/C]	1067302992
Ca_Kabuli_NC_021166.1_43119052	Ca_kabuli_chr07	43119052	[G/A]	1067302993
Ca_Kabuli_NC_021166.1_43119070	Ca_kabuli_chr07	43119070	[T/G]	1067302994
Ca_Kabuli_NC_021166.1_43119085	Ca_kabuli_chr07	43119085	[T/C]	1067302995
Ca_Kabuli_NC_021166.1_43210967	Ca_kabuli_chr07	43210967	[T/C]	1067302996
Ca_Kabuli_NC_021166.1_43646121	Ca_kabuli_chr07	43646121	[A/G]	1067302997
Ca_Kabuli_NC_021166.1_43743219	Ca_kabuli_chr07	43743219	[T/G]	1067302998
Ca_Kabuli_NC_021166.1_43999700	Ca_kabuli_chr07	43999700	[T/C]	1067302999
Ca_Kabuli_NC_021166.1_44148347	Ca_kabuli_chr07	44148347	[A/G]	1067303000
Ca_Kabuli_NC_021166.1_44148409	Ca_kabuli_chr07	44148409	[A/G]	1067303001
Ca_Kabuli_NC_021166.1_44149783	Ca_kabuli_chr07	44149783	[T/G]	1067303002
Ca_Kabuli_NC_021166.1_44149794	Ca_kabuli_chr07	44149794	[T/G]	1067303003
Ca_Kabuli_NC_021166.1_44149803	Ca_kabuli_chr07	44149803	[T/G]	1067303004
Ca_Kabuli_NC_021166.1_44232220	Ca_kabuli_chr07	44232220	[G/A]	1067303005
Ca_Kabuli_NC_021166.1_44232221	Ca_kabuli_chr07	44232221	[T/C]	1067303006
Ca_Kabuli_NC_021166.1_44232225	Ca_kabuli_chr07	44232225	[T/C]	1067303007
Ca_Kabuli_NC_021166.1_44232228	Ca_kabuli_chr07	44232228	[A/T]	1067303008
Ca_Kabuli_NC_021166.1_44232231	Ca_kabuli_chr07	44232231	[A/C]	1067303009
Ca_Kabuli_NC_021166.1_44758351	Ca_kabuli_chr07	44758351	[A/C]	1067303010
Ca_Kabuli_NC_021166.1_44877300	Ca_kabuli_chr07	44877300	[T/G]	1067303011
Ca_Kabuli_NC_021166.1_44889401	Ca_kabuli_chr07	44889401	[C/A]	1067303012

SNP IDs	Chromosomes	Physical positions (bp)	SNPs	NCBI SNPdb accession IDs (NCBI_ss#)
Ca_Kabuli_NC_021166.1_44889402	Ca_kabuli_chr07	44889402	[C/G]	1067303013
Ca_Kabuli_NC_021166.1_44889403	Ca_kabuli_chr07	44889403	[T/A]	1067303014
Ca_Kabuli_NC_021166.1_44889404	Ca_kabuli_chr07	44889404	[C/T]	1067303015
Ca_Kabuli_NC_021166.1_44889532	Ca_kabuli_chr07	44889532	[T/G]	1067303016
Ca_Kabuli_NC_021166.1_44899440	Ca_kabuli_chr07	44899440	[A/C]	1067303017
Ca_Kabuli_NC_021166.1_44958134	Ca_kabuli_chr07	44958134	[T/C]	1067303018
Ca_Kabuli_NC_021166.1_44958148	Ca_kabuli_chr07	44958148	[T/C]	1067303019
Ca_Kabuli_NC_021166.1_45034281	Ca_kabuli_chr07	45034281	[T/C]	1067303020
Ca_Kabuli_NC_021166.1_45057710	Ca_kabuli_chr07	45057710	[A/C]	1067303021
Ca_Kabuli_NC_021166.1_45077803	Ca_kabuli_chr07	45077803	[T/G]	1067303022
Ca_Kabuli_NC_021166.1_45077815	Ca_kabuli_chr07	45077815	[A/C]	1067303023
Ca_Kabuli_NC_021166.1_45197476	Ca_kabuli_chr07	45197476	[T/G]	1067303024
Ca_Kabuli_NC_021166.1_45308355	Ca_kabuli_chr07	45308355	[G/T]	1067303025
Ca_Kabuli_NC_021166.1_45383082	Ca_kabuli_chr07	45383082	[A/T]	1067303026
Ca_Kabuli_NC_021166.1_45383165	Ca_kabuli_chr07	45383165	[T/A]	1067303027
Ca_Kabuli_NC_021166.1_45936523	Ca_kabuli_chr07	45936523	[T/C]	1067303028
Ca_Kabuli_NC_021166.1_45936538	Ca_kabuli_chr07	45936538	[T/G]	1067303029
Ca_Kabuli_NC_021166.1_45936571	Ca_kabuli_chr07	45936571	[A/G]	1067303030
Ca_Kabuli_NC_021166.1_45940417	Ca_kabuli_chr07	45940417	[A/C]	1067303031
Ca_Kabuli_NC_021166.1_46485934	Ca_kabuli_chr07	46485934	[G/A]	1067303032
Ca_Kabuli_NC_021166.1_46823544	Ca_kabuli_chr07	46823544	[A/C]	1067303033
Ca_Kabuli_NC_021166.1_46823641	Ca_kabuli_chr07	46823641	[C/A]	1067303034
Ca_Kabuli_NC_021166.1_46823642	Ca_kabuli_chr07	46823642	[A/G]	1067303035
Ca_Kabuli_NC_021166.1_46823644	Ca_kabuli_chr07	46823644	[A/T]	1067303036
Ca_Kabuli_NC_021166.1_46823929	Ca_kabuli_chr07	46823929	[T/C]	1067303037
Ca_Kabuli_NC_021166.1_46875624	Ca_kabuli_chr07	46875624	[T/A]	1067303038
Ca_Kabuli_NC_021166.1_46875629	Ca_kabuli_chr07	46875629	[A/T]	1067303039
Ca_Kabuli_NC_021166.1_46875657	Ca_kabuli_chr07	46875657	[T/A]	1067303040
Ca_Kabuli_NC_021166.1_46875675	Ca_kabuli_chr07	46875675	[A/G]	1067303041
Ca_Kabuli_NC_021166.1_47130817	Ca_kabuli_chr07	47130817	[T/G]	1067303042
Ca_Kabuli_NC_021166.1_47205637	Ca_kabuli_chr07	47205637	[T/A]	1067303043
Ca_Kabuli_NC_021166.1_47335940	Ca_kabuli_chr07	47335940	[G/A]	1067303044
Ca_Kabuli_NC_021166.1_47395540	Ca_kabuli_chr07	47395540	[C/T]	1067303045
Ca_Kabuli_NC_021166.1_47694704	Ca_kabuli_chr07	47694704	[T/C]	1067303046

SNP IDs	Chromosomes	Physical positions (bp)	SNPs	NCBI SNPdb accession IDs (NCBI_ss#)
Ca_Kabuli_NC_021166.1_47840107	Ca_kabuli_chr07	47840107	[A/C]	1067303047
Ca_Kabuli_NC_021166.1_47840111	Ca_kabuli_chr07	47840111	[A/C]	1067303048
Ca_Kabuli_NC_021166.1_47840119	Ca_kabuli_chr07	47840119	[A/C]	1067303049
Ca_Kabuli_NC_021166.1_47840123	Ca_kabuli_chr07	47840123	[T/C]	1067303050
Ca_Kabuli_NC_021166.1_48251532	Ca_kabuli_chr07	48251532	[C/T]	1067303051
Ca_Kabuli_NC_021167.1_41355	Ca_kabuli_chr08	41355	[T/G]	1067303052
Ca_Kabuli_NC_021167.1_54128	Ca_kabuli_chr08	54128	[T/G]	1067303053
Ca_Kabuli_NC_021167.1_54147	Ca_kabuli_chr08	54147	[T/C]	1067303054
Ca_Kabuli_NC_021167.1_54166	Ca_kabuli_chr08	54166	[A/G]	1067303055
Ca_Kabuli_NC_021167.1_90353	Ca_kabuli_chr08	90353	[T/G]	1067303056
Ca_Kabuli_NC_021167.1_120838	Ca_kabuli_chr08	120838	[T/G]	1067303057
Ca_Kabuli_NC_021167.1_132517	Ca_kabuli_chr08	132517	[A/G]	1067303058
Ca_Kabuli_NC_021167.1_177453	Ca_kabuli_chr08	177453	[T/C]	1067303059
Ca_Kabuli_NC_021167.1_177456	Ca_kabuli_chr08	177456	[A/T]	1067303060
Ca_Kabuli_NC_021167.1_178228	Ca_kabuli_chr08	178228	[G/T]	1067303061
Ca_Kabuli_NC_021167.1_184409	Ca_kabuli_chr08	184409	[C/T]	1067303062
Ca_Kabuli_NC_021167.1_218917	Ca_kabuli_chr08	218917	[A/G]	1067303063
Ca_Kabuli_NC_021167.1_218950	Ca_kabuli_chr08	218950	[A/G]	1067303064
Ca_Kabuli_NC_021167.1_253604	Ca_kabuli_chr08	253604	[T/C]	1067303065
Ca_Kabuli_NC_021167.1_253622	Ca_kabuli_chr08	253622	[T/A]	1067303066
Ca_Kabuli_NC_021167.1_253650	Ca_kabuli_chr08	253650	[A/C]	1067303067
Ca_Kabuli_NC_021167.1_253661	Ca_kabuli_chr08	253661	[T/C]	1067303068
Ca_Kabuli_NC_021167.1_253675	Ca_kabuli_chr08	253675	[A/G]	1067303069
Ca_Kabuli_NC_021167.1_253685	Ca_kabuli_chr08	253685	[A/G]	1067303070
Ca_Kabuli_NC_021167.1_258729	Ca_kabuli_chr08	258729	[T/C]	1067303071
Ca_Kabuli_NC_021167.1_258735	Ca_kabuli_chr08	258735	[T/C]	1067303072
Ca_Kabuli_NC_021167.1_281566	Ca_kabuli_chr08	281566	[T/C]	1067303073
Ca_Kabuli_NC_021167.1_281579	Ca_kabuli_chr08	281579	[A/G]	1067303074
Ca_Kabuli_NC_021167.1_281622	Ca_kabuli_chr08	281622	[G/A]	1067303075
Ca_Kabuli_NC_021167.1_283444	Ca_kabuli_chr08	283444	[T/C]	1067303076
Ca_Kabuli_NC_021167.1_306458	Ca_kabuli_chr08	306458	[A/C]	1067303077
Ca_Kabuli_NC_021167.1_306477	Ca_kabuli_chr08	306477	[A/C]	1067303078
Ca_Kabuli_NC_021167.1_310107	Ca_kabuli_chr08	310107	[T/C]	1067303079
Ca_Kabuli_NC_021167.1_310131	Ca_kabuli_chr08	310131	[A/C]	1067303080

SNP IDs	Chromosomes	Physical positions (bp)	SNPs	NCBI SNPdb accession IDs (NCBI_ss#)
Ca_Kabuli_NC_021167.1_353470	Ca_kabuli_chr08	353470	[A/C]	1067303081
Ca_Kabuli_NC_021167.1_353535	Ca_kabuli_chr08	353535	[A/C]	1067303082
Ca_Kabuli_NC_021167.1_447550	Ca_kabuli_chr08	447550	[T/C]	1067303083
Ca_Kabuli_NC_021167.1_499873	Ca_kabuli_chr08	499873	[A/C]	1067303084
Ca_Kabuli_NC_021167.1_499876	Ca_kabuli_chr08	499876	[A/C]	1067303085
Ca_Kabuli_NC_021167.1_499879	Ca_kabuli_chr08	499879	[A/C]	1067303086
Ca_Kabuli_NC_021167.1_510851	Ca_kabuli_chr08	510851	[A/G]	1067303087
Ca_Kabuli_NC_021167.1_535892	Ca_kabuli_chr08	535892	[A/G]	1067303088
Ca_Kabuli_NC_021167.1_535903	Ca_kabuli_chr08	535903	[T/C]	1067303089
Ca_Kabuli_NC_021167.1_560472	Ca_kabuli_chr08	560472	[A/G]	1067303090
Ca_Kabuli_NC_021167.1_560484	Ca_kabuli_chr08	560484	[T/G]	1067303091
Ca_Kabuli_NC_021167.1_560740	Ca_kabuli_chr08	560740	[T/G]	1067303092
Ca_Kabuli_NC_021167.1_634819	Ca_kabuli_chr08	634819	[T/G]	1067303093
Ca_Kabuli_NC_021167.1_634853	Ca_kabuli_chr08	634853	[T/G]	1067303094
Ca_Kabuli_NC_021167.1_634913	Ca_kabuli_chr08	634913	[G/C]	1067303095
Ca_Kabuli_NC_021167.1_634932	Ca_kabuli_chr08	634932	[A/G]	1067303096
Ca_Kabuli_NC_021167.1_634936	Ca_kabuli_chr08	634936	[A/G]	1067303097
Ca_Kabuli_NC_021167.1_634945	Ca_kabuli_chr08	634945	[T/C]	1067303098
Ca_Kabuli_NC_021167.1_637223	Ca_kabuli_chr08	637223	[T/G]	1067303099
Ca_Kabuli_NC_021167.1_637253	Ca_kabuli_chr08	637253	[T/G]	1067303100
Ca_Kabuli_NC_021167.1_637257	Ca_kabuli_chr08	637257	[T/G]	1067303101
Ca_Kabuli_NC_021167.1_637261	Ca_kabuli_chr08	637261	[T/C]	1067303102
Ca_Kabuli_NC_021167.1_637308	Ca_kabuli_chr08	637308	[T/C]	1067303103
Ca_Kabuli_NC_021167.1_637309	Ca_kabuli_chr08	637309	[C/T]	1067303104
Ca_Kabuli_NC_021167.1_637317	Ca_kabuli_chr08	637317	[G/C]	1067303105
Ca_Kabuli_NC_021167.1_637322	Ca_kabuli_chr08	637322	[T/C]	1067303106
Ca_Kabuli_NC_021167.1_637335	Ca_kabuli_chr08	637335	[A/T]	1067303107
Ca_Kabuli_NC_021167.1_649502	Ca_kabuli_chr08	649502	[T/G]	1067303108
Ca_Kabuli_NC_021167.1_659335	Ca_kabuli_chr08	659335	[A/C]	1067303109
Ca_Kabuli_NC_021167.1_690131	Ca_kabuli_chr08	690131	[T/G]	1067303110
Ca_Kabuli_NC_021167.1_709105	Ca_kabuli_chr08	709105	[T/G]	1067303111
Ca_Kabuli_NC_021167.1_777765	Ca_kabuli_chr08	777765	[T/G]	1067303112
Ca_Kabuli_NC_021167.1_779659	Ca_kabuli_chr08	779659	[A/G]	1067303113
Ca_Kabuli_NC_021167.1_822850	Ca_kabuli_chr08	822850	[A/C]	1067303114

SNP IDs	Chromosomes	Physical positions (bp)	SNPs	NCBI SNPdb accession IDs (NCBI_ss#)
Ca_Kabuli_NC_021167.1_822853	Ca_kabuli_chr08	822853	[C/A]	1067303115
Ca_Kabuli_NC_021167.1_840012	Ca_kabuli_chr08	840012	[T/G]	1067303116
Ca_Kabuli_NC_021167.1_840015	Ca_kabuli_chr08	840015	[T/G]	1067303117
Ca_Kabuli_NC_021167.1_840041	Ca_kabuli_chr08	840041	[T/G]	1067303118
Ca_Kabuli_NC_021167.1_840075	Ca_kabuli_chr08	840075	[A/G]	1067303119
Ca_Kabuli_NC_021167.1_875236	Ca_kabuli_chr08	875236	[T/G]	1067303120
Ca_Kabuli_NC_021167.1_883520	Ca_kabuli_chr08	883520	[T/G]	1067303121
Ca_Kabuli_NC_021167.1_884757	Ca_kabuli_chr08	884757	[T/C]	1067303122
Ca_Kabuli_NC_021167.1_897400	Ca_kabuli_chr08	897400	[T/G]	1067303123
Ca_Kabuli_NC_021167.1_897465	Ca_kabuli_chr08	897465	[A/G]	1067303124
Ca_Kabuli_NC_021167.1_910687	Ca_kabuli_chr08	910687	[A/C]	1067303125
Ca_Kabuli_NC_021167.1_910716	Ca_kabuli_chr08	910716	[A/G]	1067303126
Ca_Kabuli_NC_021167.1_910719	Ca_kabuli_chr08	910719	[T/G]	1067303127
Ca_Kabuli_NC_021167.1_924661	Ca_kabuli_chr08	924661	[T/C]	1067303128
Ca_Kabuli_NC_021167.1_924715	Ca_kabuli_chr08	924715	[A/G]	1067303129
Ca_Kabuli_NC_021167.1_995091	Ca_kabuli_chr08	995091	[T/A]	1067303130
Ca_Kabuli_NC_021167.1_995092	Ca_kabuli_chr08	995092	[A/G]	1067303131
Ca_Kabuli_NC_021167.1_995138	Ca_kabuli_chr08	995138	[A/T]	1067303132
Ca_Kabuli_NC_021167.1_1011362	Ca_kabuli_chr08	1011362	[T/C]	1067303133
Ca_Kabuli_NC_021167.1_1108533	Ca_kabuli_chr08	1108533	[T/C]	1067303134
Ca_Kabuli_NC_021167.1_1131183	Ca_kabuli_chr08	1131183	[T/G]	1067303135
Ca_Kabuli_NC_021167.1_1131202	Ca_kabuli_chr08	1131202	[T/C]	1067303136
Ca_Kabuli_NC_021167.1_1194886	Ca_kabuli_chr08	1194886	[G/A]	1067303137
Ca_Kabuli_NC_021167.1_1262555	Ca_kabuli_chr08	1262555	[T/C]	1067303138
Ca_Kabuli_NC_021167.1_1262583	Ca_kabuli_chr08	1262583	[T/A]	1067303139
Ca_Kabuli_NC_021167.1_1265506	Ca_kabuli_chr08	1265506	[T/A]	1067303140
Ca_Kabuli_NC_021167.1_1265508	Ca_kabuli_chr08	1265508	[T/G]	1067303141
Ca_Kabuli_NC_021167.1_1265509	Ca_kabuli_chr08	1265509	[T/A]	1067303142
Ca_Kabuli_NC_021167.1_1265603	Ca_kabuli_chr08	1265603	[A/G]	1067303143
Ca_Kabuli_NC_021167.1_1276472	Ca_kabuli_chr08	1276472	[A/G]	1067303144
Ca_Kabuli_NC_021167.1_1290371	Ca_kabuli_chr08	1290371	[A/C]	1067303145
Ca_Kabuli_NC_021167.1_1290386	Ca_kabuli_chr08	1290386	[T/C]	1067303146
Ca_Kabuli_NC_021167.1_1325152	Ca_kabuli_chr08	1325152	[A/C]	1067303147
Ca_Kabuli_NC_021167.1_1362659	Ca_kabuli_chr08	1362659	[A/G]	1067303148

SNP IDs	Chromosomes	Physical positions (bp)	SNPs	NCBI SNPdb accession IDs (NCBI_ss#)
Ca_Kabuli_NC_021167.1_1362673	Ca_kabuli_chr08	1362673	[A/C]	1067303149
Ca_Kabuli_NC_021167.1_1363721	Ca_kabuli_chr08	1363721	[T/G]	1067303150
Ca_Kabuli_NC_021167.1_1363755	Ca_kabuli_chr08	1363755	[A/C]	1067303151
Ca_Kabuli_NC_021167.1_1370295	Ca_kabuli_chr08	1370295	[A/C]	1067303152
Ca_Kabuli_NC_021167.1_1465284	Ca_kabuli_chr08	1465284	[A/T]	1067303153
Ca_Kabuli_NC_021167.1_1475872	Ca_kabuli_chr08	1475872	[G/T]	1067303154
Ca_Kabuli_NC_021167.1_1475873	Ca_kabuli_chr08	1475873	[A/C]	1067303155
Ca_Kabuli_NC_021167.1_1475874	Ca_kabuli_chr08	1475874	[G/T]	1067303156
Ca_Kabuli_NC_021167.1_1507766	Ca_kabuli_chr08	1507766	[G/A]	1067303157
Ca_Kabuli_NC_021167.1_1554893	Ca_kabuli_chr08	1554893	[T/A]	1067303158
Ca_Kabuli_NC_021167.1_1555159	Ca_kabuli_chr08	1555159	[A/G]	1067303159
Ca_Kabuli_NC_021167.1_1612186	Ca_kabuli_chr08	1612186	[T/A]	1067303160
Ca_Kabuli_NC_021167.1_1675894	Ca_kabuli_chr08	1675894	[A/T]	1067303161
Ca_Kabuli_NC_021167.1_1675917	Ca_kabuli_chr08	1675917	[T/C]	1067303162
Ca_Kabuli_NC_021167.1_1708730	Ca_kabuli_chr08	1708730	[T/G]	1067303163
Ca_Kabuli_NC_021167.1_1708895	Ca_kabuli_chr08	1708895	[A/G]	1067303164
Ca_Kabuli_NC_021167.1_1711439	Ca_kabuli_chr08	1711439	[A/G]	1067303165
Ca_Kabuli_NC_021167.1_1718014	Ca_kabuli_chr08	1718014	[T/C]	1067303166
Ca_Kabuli_NC_021167.1_1718018	Ca_kabuli_chr08	1718018	[A/G]	1067303167
Ca_Kabuli_NC_021167.1_1718031	Ca_kabuli_chr08	1718031	[A/C]	1067303168
Ca_Kabuli_NC_021167.1_1723577	Ca_kabuli_chr08	1723577	[T/A]	1067303169
Ca_Kabuli_NC_021167.1_1742958	Ca_kabuli_chr08	1742958	[G/T]	1067303170
Ca_Kabuli_NC_021167.1_1742959	Ca_kabuli_chr08	1742959	[G/C]	1067303171
Ca_Kabuli_NC_021167.1_1742960	Ca_kabuli_chr08	1742960	[G/T]	1067303172
Ca_Kabuli_NC_021167.1_1747528	Ca_kabuli_chr08	1747528	[A/T]	1067303173
Ca_Kabuli_NC_021167.1_1782034	Ca_kabuli_chr08	1782034	[C/G]	1067303174
Ca_Kabuli_NC_021167.1_1794696	Ca_kabuli_chr08	1794696	[A/G]	1067303175
Ca_Kabuli_NC_021167.1_1794714	Ca_kabuli_chr08	1794714	[T/G]	1067303176
Ca_Kabuli_NC_021167.1_1794717	Ca_kabuli_chr08	1794717	[T/A]	1067303177
Ca_Kabuli_NC_021167.1_1794745	Ca_kabuli_chr08	1794745	[A/G]	1067303178
Ca_Kabuli_NC_021167.1_1794804	Ca_kabuli_chr08	1794804	[G/T]	1067303179
Ca_Kabuli_NC_021167.1_1794812	Ca_kabuli_chr08	1794812	[G/C]	1067303180
Ca_Kabuli_NC_021167.1_1794813	Ca_kabuli_chr08	1794813	[T/G]	1067303181
Ca_Kabuli_NC_021167.1_1794814	Ca_kabuli_chr08	1794814	[G/T]	1067303182

SNP IDs	Chromosomes	Physical positions (bp)	SNPs	NCBI SNPdb accession IDs (NCBI_ss#)
Ca_Kabuli_NC_021167.1_1794815	Ca_kabuli_chr08	1794815	[A/C]	1067303183
Ca_Kabuli_NC_021167.1_1794915	Ca_kabuli_chr08	1794915	[A/G]	1067303184
Ca_Kabuli_NC_021167.1_1829515	Ca_kabuli_chr08	1829515	[A/G]	1067303185
Ca_Kabuli_NC_021167.1_1880101	Ca_kabuli_chr08	1880101	[T/C]	1067303186
Ca_Kabuli_NC_021167.1_1880113	Ca_kabuli_chr08	1880113	[A/G]	1067303187
Ca_Kabuli_NC_021167.1_1880158	Ca_kabuli_chr08	1880158	[T/C]	1067303188
Ca_Kabuli_NC_021167.1_1883131	Ca_kabuli_chr08	1883131	[T/G]	1067303189
Ca_Kabuli_NC_021167.1_1958409	Ca_kabuli_chr08	1958409	[T/G]	1067303190
Ca_Kabuli_NC_021167.1_1963543	Ca_kabuli_chr08	1963543	[T/G]	1067303191
Ca_Kabuli_NC_021167.1_1970309	Ca_kabuli_chr08	1970309	[A/C]	1067303192
Ca_Kabuli_NC_021167.1_1989467	Ca_kabuli_chr08	1989467	[T/G]	1067303193
Ca_Kabuli_NC_021167.1_2029411	Ca_kabuli_chr08	2029411	[A/G]	1067303194
Ca_Kabuli_NC_021167.1_2032659	Ca_kabuli_chr08	2032659	[T/C]	1067303195
Ca_Kabuli_NC_021167.1_2073741	Ca_kabuli_chr08	2073741	[T/C]	1067303196
Ca_Kabuli_NC_021167.1_2073782	Ca_kabuli_chr08	2073782	[T/C]	1067303197
Ca_Kabuli_NC_021167.1_2073806	Ca_kabuli_chr08	2073806	[A/T]	1067303198
Ca_Kabuli_NC_021167.1_2074562	Ca_kabuli_chr08	2074562	[A/C]	1067303199
Ca_Kabuli_NC_021167.1_2074685	Ca_kabuli_chr08	2074685	[A/C]	1067303200
Ca_Kabuli_NC_021167.1_2074706	Ca_kabuli_chr08	2074706	[A/C]	1067303201
Ca_Kabuli_NC_021167.1_2075260	Ca_kabuli_chr08	2075260	[A/T]	1067303202
Ca_Kabuli_NC_021167.1_2075276	Ca_kabuli_chr08	2075276	[A/T]	1067303203
Ca_Kabuli_NC_021167.1_2079734	Ca_kabuli_chr08	2079734	[A/G]	1067303204
Ca_Kabuli_NC_021167.1_2079765	Ca_kabuli_chr08	2079765	[T/C]	1067303205
Ca_Kabuli_NC_021167.1_2079787	Ca_kabuli_chr08	2079787	[A/G]	1067303206
Ca_Kabuli_NC_021167.1_2080545	Ca_kabuli_chr08	2080545	[A/C]	1067303207
Ca_Kabuli_NC_021167.1_2080621	Ca_kabuli_chr08	2080621	[A/G]	1067303208
Ca_Kabuli_NC_021167.1_2088903	Ca_kabuli_chr08	2088903	[T/G]	1067303209
Ca_Kabuli_NC_021167.1_2088919	Ca_kabuli_chr08	2088919	[A/G]	1067303210
Ca_Kabuli_NC_021167.1_2088944	Ca_kabuli_chr08	2088944	[T/C]	1067303211
Ca_Kabuli_NC_021167.1_2088965	Ca_kabuli_chr08	2088965	[A/T]	1067303212
Ca_Kabuli_NC_021167.1_2089009	Ca_kabuli_chr08	2089009	[A/T]	1067303213
Ca_Kabuli_NC_021167.1_2093176	Ca_kabuli_chr08	2093176	[A/G]	1067303214
Ca_Kabuli_NC_021167.1_2093212	Ca_kabuli_chr08	2093212	[A/C]	1067303215
Ca_Kabuli_NC_021167.1_2093214	Ca_kabuli_chr08	2093214	[T/G]	1067303216

SNP IDs	Chromosomes	Physical positions (bp)	SNPs	NCBI SNPdb accession IDs (NCBI_ss#)
Ca_Kabuli_NC_021167.1_2093224	Ca_kabuli_chr08	2093224	[A/G]	1067303217
Ca_Kabuli_NC_021167.1_2093226	Ca_kabuli_chr08	2093226	[T/G]	1067303218
Ca_Kabuli_NC_021167.1_2109980	Ca_kabuli_chr08	2109980	[A/C]	1067303219
Ca_Kabuli_NC_021167.1_2136222	Ca_kabuli_chr08	2136222	[T/C]	1067303220
Ca_Kabuli_NC_021167.1_2136229	Ca_kabuli_chr08	2136229	[T/G]	1067303221
Ca_Kabuli_NC_021167.1_2136243	Ca_kabuli_chr08	2136243	[T/G]	1067303222
Ca_Kabuli_NC_021167.1_2136282	Ca_kabuli_chr08	2136282	[T/G]	1067303223
Ca_Kabuli_NC_021167.1_2186379	Ca_kabuli_chr08	2186379	[A/T]	1067303224
Ca_Kabuli_NC_021167.1_2186380	Ca_kabuli_chr08	2186380	[T/A]	1067303225
Ca_Kabuli_NC_021167.1_2190581	Ca_kabuli_chr08	2190581	[C/T]	1067303226
Ca_Kabuli_NC_021167.1_2194871	Ca_kabuli_chr08	2194871	[T/C]	1067303227
Ca_Kabuli_NC_021167.1_2264188	Ca_kabuli_chr08	2264188	[T/C]	1067303228
Ca_Kabuli_NC_021167.1_2281792	Ca_kabuli_chr08	2281792	[T/C]	1067303229
Ca_Kabuli_NC_021167.1_2281799	Ca_kabuli_chr08	2281799	[T/C]	1067303230
Ca_Kabuli_NC_021167.1_2281814	Ca_kabuli_chr08	2281814	[A/G]	1067303231
Ca_Kabuli_NC_021167.1_2417216	Ca_kabuli_chr08	2417216	[T/G]	1067303232
Ca_Kabuli_NC_021167.1_2417233	Ca_kabuli_chr08	2417233	[T/G]	1067303233
Ca_Kabuli_NC_021167.1_2452031	Ca_kabuli_chr08	2452031	[A/G]	1067303234
Ca_Kabuli_NC_021167.1_2459578	Ca_kabuli_chr08	2459578	[T/A]	1067303235
Ca_Kabuli_NC_021167.1_2463102	Ca_kabuli_chr08	2463102	[A/C]	1067303236
Ca_Kabuli_NC_021167.1_2463549	Ca_kabuli_chr08	2463549	[A/G]	1067303237
Ca_Kabuli_NC_021167.1_2464356	Ca_kabuli_chr08	2464356	[T/C]	1067303238
Ca_Kabuli_NC_021167.1_2482765	Ca_kabuli_chr08	2482765	[T/C]	1067303239
Ca_Kabuli_NC_021167.1_2482798	Ca_kabuli_chr08	2482798	[A/G]	1067303240
Ca_Kabuli_NC_021167.1_2483120	Ca_kabuli_chr08	2483120	[A/T]	1067303241
Ca_Kabuli_NC_021167.1_2524150	Ca_kabuli_chr08	2524150	[T/G]	1067303242
Ca_Kabuli_NC_021167.1_2524153	Ca_kabuli_chr08	2524153	[T/G]	1067303243
Ca_Kabuli_NC_021167.1_2524166	Ca_kabuli_chr08	2524166	[A/G]	1067303244
Ca_Kabuli_NC_021167.1_2529535	Ca_kabuli_chr08	2529535	[T/C]	1067303245
Ca_Kabuli_NC_021167.1_2529563	Ca_kabuli_chr08	2529563	[T/A]	1067303246
Ca_Kabuli_NC_021167.1_2540613	Ca_kabuli_chr08	2540613	[T/G]	1067303247
Ca_Kabuli_NC_021167.1_2543145	Ca_kabuli_chr08	2543145	[T/A]	1067303248
Ca_Kabuli_NC_021167.1_2543151	Ca_kabuli_chr08	2543151	[A/C]	1067303249
Ca_Kabuli_NC_021167.1_2545208	Ca_kabuli_chr08	2545208	[A/T]	1067303250

SNP IDs	Chromosomes	Physical positions (bp)	SNPs	NCBI SNPdb accession IDs (NCBI_ss#)
Ca_Kabuli_NC_021167.1_2545211	Ca_kabuli_chr08	2545211	[T/C]	1067303251
Ca_Kabuli_NC_021167.1_2580092	Ca_kabuli_chr08	2580092	[T/G]	1067303252
Ca_Kabuli_NC_021167.1_2580170	Ca_kabuli_chr08	2580170	[A/G]	1067303253
Ca_Kabuli_NC_021167.1_2580194	Ca_kabuli_chr08	2580194	[T/G]	1067303254
Ca_Kabuli_NC_021167.1_2580273	Ca_kabuli_chr08	2580273	[A/G]	1067303255
Ca_Kabuli_NC_021167.1_2617595	Ca_kabuli_chr08	2617595	[T/A]	1067303256
Ca_Kabuli_NC_021167.1_2622008	Ca_kabuli_chr08	2622008	[T/G]	1067303257
Ca_Kabuli_NC_021167.1_2623534	Ca_kabuli_chr08	2623534	[T/C]	1067303258
Ca_Kabuli_NC_021167.1_2623544	Ca_kabuli_chr08	2623544	[T/A]	1067303259
Ca_Kabuli_NC_021167.1_2623545	Ca_kabuli_chr08	2623545	[A/T]	1067303260
Ca_Kabuli_NC_021167.1_2651888	Ca_kabuli_chr08	2651888	[T/A]	1067303261
Ca_Kabuli_NC_021167.1_2651889	Ca_kabuli_chr08	2651889	[A/C]	1067303262
Ca_Kabuli_NC_021167.1_2651895	Ca_kabuli_chr08	2651895	[A/T]	1067303263
Ca_Kabuli_NC_021167.1_2651902	Ca_kabuli_chr08	2651902	[T/G]	1067303264
Ca_Kabuli_NC_021167.1_2651925	Ca_kabuli_chr08	2651925	[C/G]	1067303265
Ca_Kabuli_NC_021167.1_2686597	Ca_kabuli_chr08	2686597	[A/C]	1067303266
Ca_Kabuli_NC_021167.1_2686610	Ca_kabuli_chr08	2686610	[A/C]	1067303267
Ca_Kabuli_NC_021167.1_2686615	Ca_kabuli_chr08	2686615	[A/T]	1067303268
Ca_Kabuli_NC_021167.1_2722647	Ca_kabuli_chr08	2722647	[T/C]	1067303269
Ca_Kabuli_NC_021167.1_2722660	Ca_kabuli_chr08	2722660	[T/G]	1067303270
Ca_Kabuli_NC_021167.1_2722664	Ca_kabuli_chr08	2722664	[A/G]	1067303271
Ca_Kabuli_NC_021167.1_2729806	Ca_kabuli_chr08	2729806	[A/T]	1067303272
Ca_Kabuli_NC_021167.1_2729827	Ca_kabuli_chr08	2729827	[A/G]	1067303273
Ca_Kabuli_NC_021167.1_2827779	Ca_kabuli_chr08	2827779	[T/C]	1067303274
Ca_Kabuli_NC_021167.1_2827781	Ca_kabuli_chr08	2827781	[G/A]	1067303275
Ca_Kabuli_NC_021167.1_2827820	Ca_kabuli_chr08	2827820	[A/G]	1067303276
Ca_Kabuli_NC_021167.1_2827832	Ca_kabuli_chr08	2827832	[T/A]	1067303277
Ca_Kabuli_NC_021167.1_2859885	Ca_kabuli_chr08	2859885	[T/C]	1067303278
Ca_Kabuli_NC_021167.1_2889430	Ca_kabuli_chr08	2889430	[T/A]	1067303279
Ca_Kabuli_NC_021167.1_2889717	Ca_kabuli_chr08	2889717	[T/C]	1067303280
Ca_Kabuli_NC_021167.1_2889720	Ca_kabuli_chr08	2889720	[T/C]	1067303281
Ca_Kabuli_NC_021167.1_2889747	Ca_kabuli_chr08	2889747	[T/C]	1067303282
Ca_Kabuli_NC_021167.1_2943660	Ca_kabuli_chr08	2943660	[A/G]	1067303283
Ca_Kabuli_NC_021167.1_2947511	Ca_kabuli_chr08	2947511	[A/G]	1067303284

SNP IDs	Chromosomes	Physical positions (bp)	SNPs	NCBI SNPdb accession IDs (NCBI_ss#)
Ca_Kabuli_NC_021167.1_2955532	Ca_kabuli_chr08	2955532	[A/C]	1067303285
Ca_Kabuli_NC_021167.1_2974555	Ca_kabuli_chr08	2974555	[A/C]	1067303286
Ca_Kabuli_NC_021167.1_3013920	Ca_kabuli_chr08	3013920	[T/C]	1067303287
Ca_Kabuli_NC_021167.1_3017548	Ca_kabuli_chr08	3017548	[G/T]	1067303288
Ca_Kabuli_NC_021167.1_3017550	Ca_kabuli_chr08	3017550	[G/T]	1067303289
Ca_Kabuli_NC_021167.1_3020987	Ca_kabuli_chr08	3020987	[A/T]	1067303290
Ca_Kabuli_NC_021167.1_3021000	Ca_kabuli_chr08	3021000	[T/G]	1067303291
Ca_Kabuli_NC_021167.1_3021006	Ca_kabuli_chr08	3021006	[T/G]	1067303292
Ca_Kabuli_NC_021167.1_3021007	Ca_kabuli_chr08	3021007	[A/C]	1067303293
Ca_Kabuli_NC_021167.1_3041872	Ca_kabuli_chr08	3041872	[A/C]	1067303294
Ca_Kabuli_NC_021167.1_3171296	Ca_kabuli_chr08	3171296	[A/G]	1067303295
Ca_Kabuli_NC_021167.1_3171321	Ca_kabuli_chr08	3171321	[A/G]	1067303296
Ca_Kabuli_NC_021167.1_3179618	Ca_kabuli_chr08	3179618	[C/T]	1067303297
Ca_Kabuli_NC_021167.1_3269063	Ca_kabuli_chr08	3269063	[T/G]	1067303298
Ca_Kabuli_NC_021167.1_3269085	Ca_kabuli_chr08	3269085	[A/C]	1067303299
Ca_Kabuli_NC_021167.1_3269088	Ca_kabuli_chr08	3269088	[A/C]	1067303300
Ca_Kabuli_NC_021167.1_3394738	Ca_kabuli_chr08	3394738	[T/G]	1067303301
Ca_Kabuli_NC_021167.1_3396787	Ca_kabuli_chr08	3396787	[T/C]	1067303302
Ca_Kabuli_NC_021167.1_3396797	Ca_kabuli_chr08	3396797	[T/A]	1067303303
Ca_Kabuli_NC_021167.1_3396849	Ca_kabuli_chr08	3396849	[T/C]	1067303304
Ca_Kabuli_NC_021167.1_3498977	Ca_kabuli_chr08	3498977	[T/G]	1067303305
Ca_Kabuli_NC_021167.1_3499001	Ca_kabuli_chr08	3499001	[A/G]	1067303306
Ca_Kabuli_NC_021167.1_3499013	Ca_kabuli_chr08	3499013	[T/G]	1067303307
Ca_Kabuli_NC_021167.1_3499016	Ca_kabuli_chr08	3499016	[A/C]	1067303308
Ca_Kabuli_NC_021167.1_3530213	Ca_kabuli_chr08	3530213	[A/G]	1067303309
Ca_Kabuli_NC_021167.1_3533284	Ca_kabuli_chr08	3533284	[T/G]	1067303310
Ca_Kabuli_NC_021167.1_3533306	Ca_kabuli_chr08	3533306	[A/T]	1067303311
Ca_Kabuli_NC_021167.1_3541771	Ca_kabuli_chr08	3541771	[C/A]	1067303312
Ca_Kabuli_NC_021167.1_3541773	Ca_kabuli_chr08	3541773	[C/A]	1067303313
Ca_Kabuli_NC_021167.1_3541774	Ca_kabuli_chr08	3541774	[C/T]	1067303314
Ca_Kabuli_NC_021167.1_3571098	Ca_kabuli_chr08	3571098	[A/C]	1067303315
Ca_Kabuli_NC_021167.1_3601540	Ca_kabuli_chr08	3601540	[T/G]	1067303316
Ca_Kabuli_NC_021167.1_3665577	Ca_kabuli_chr08	3665577	[T/G]	1067303317
Ca_Kabuli_NC_021167.1_3709184	Ca_kabuli_chr08	3709184	[C/G]	1067303318

SNP IDs	Chromosomes	Physical positions (bp)	SNPs	NCBI SNPdb accession IDs (NCBI_ss#)
Ca_Kabuli_NC_021167.1_3709185	Ca_kabuli_chr08	3709185	[G/A]	1067303319
Ca_Kabuli_NC_021167.1_3709187	Ca_kabuli_chr08	3709187	[T/C]	1067303320
Ca_Kabuli_NC_021167.1_3709188	Ca_kabuli_chr08	3709188	[G/T]	1067303321
Ca_Kabuli_NC_021167.1_3730339	Ca_kabuli_chr08	3730339	[T/G]	1067303322
Ca_Kabuli_NC_021167.1_3775861	Ca_kabuli_chr08	3775861	[A/C]	1067303323
Ca_Kabuli_NC_021167.1_3813951	Ca_kabuli_chr08	3813951	[A/C]	1067303324
Ca_Kabuli_NC_021167.1_3829581	Ca_kabuli_chr08	3829581	[T/G]	1067303325
Ca_Kabuli_NC_021167.1_3829646	Ca_kabuli_chr08	3829646	[A/G]	1067303326
Ca_Kabuli_NC_021167.1_4006216	Ca_kabuli_chr08	4006216	[A/G]	1067303327
Ca_Kabuli_NC_021167.1_4023040	Ca_kabuli_chr08	4023040	[T/C]	1067303328
Ca_Kabuli_NC_021167.1_4023049	Ca_kabuli_chr08	4023049	[T/G]	1067303329
Ca_Kabuli_NC_021167.1_4023103	Ca_kabuli_chr08	4023103	[A/C]	1067303330
Ca_Kabuli_NC_021167.1_4023148	Ca_kabuli_chr08	4023148	[A/C]	1067303331
Ca_Kabuli_NC_021167.1_4073076	Ca_kabuli_chr08	4073076	[A/T]	1067303332
Ca_Kabuli_NC_021167.1_4073077	Ca_kabuli_chr08	4073077	[A/C]	1067303333
Ca_Kabuli_NC_021167.1_4091923	Ca_kabuli_chr08	4091923	[A/C]	1067303334
Ca_Kabuli_NC_021167.1_4091927	Ca_kabuli_chr08	4091927	[A/C]	1067303335
Ca_Kabuli_NC_021167.1_4091944	Ca_kabuli_chr08	4091944	[T/C]	1067303336
Ca_Kabuli_NC_021167.1_4102954	Ca_kabuli_chr08	4102954	[T/G]	1067303337
Ca_Kabuli_NC_021167.1_4102960	Ca_kabuli_chr08	4102960	[A/C]	1067303338
Ca_Kabuli_NC_021167.1_4172244	Ca_kabuli_chr08	4172244	[T/A]	1067303339
Ca_Kabuli_NC_021167.1_4172245	Ca_kabuli_chr08	4172245	[A/T]	1067303340
Ca_Kabuli_NC_021167.1_4172257	Ca_kabuli_chr08	4172257	[T/G]	1067303341
Ca_Kabuli_NC_021167.1_4172269	Ca_kabuli_chr08	4172269	[T/G]	1067303342
Ca_Kabuli_NC_021167.1_4172306	Ca_kabuli_chr08	4172306	[T/C]	1067303343
Ca_Kabuli_NC_021167.1_4172313	Ca_kabuli_chr08	4172313	[T/G]	1067303344
Ca_Kabuli_NC_021167.1_4172357	Ca_kabuli_chr08	4172357	[C/A]	1067303345
Ca_Kabuli_NC_021167.1_4172368	Ca_kabuli_chr08	4172368	[T/G]	1067303346
Ca_Kabuli_NC_021167.1_4198240	Ca_kabuli_chr08	4198240	[A/G]	1067303347
Ca_Kabuli_NC_021167.1_4198242	Ca_kabuli_chr08	4198242	[C/T]	1067303348
Ca_Kabuli_NC_021167.1_4198246	Ca_kabuli_chr08	4198246	[T/C]	1067303349
Ca_Kabuli_NC_021167.1_4203698	Ca_kabuli_chr08	4203698	[A/T]	1067303350
Ca_Kabuli_NC_021167.1_4203727	Ca_kabuli_chr08	4203727	[A/C]	1067303351
Ca_Kabuli_NC_021167.1_4203752	Ca_kabuli_chr08	4203752	[T/A]	1067303352

SNP IDs	Chromosomes	Physical positions (bp)	SNPs	NCBI SNPdb accession IDs (NCBI_ss#)
Ca_Kabuli_NC_021167.1_4271533	Ca_kabuli_chr08	4271533	[T/C]	1067303353
Ca_Kabuli_NC_021167.1_4271563	Ca_kabuli_chr08	4271563	[T/C]	1067303354
Ca_Kabuli_NC_021167.1_4271597	Ca_kabuli_chr08	4271597	[A/C]	1067303355
Ca_Kabuli_NC_021167.1_4280008	Ca_kabuli_chr08	4280008	[T/C]	1067303356
Ca_Kabuli_NC_021167.1_4280009	Ca_kabuli_chr08	4280009	[C/G]	1067303357
Ca_Kabuli_NC_021167.1_4290671	Ca_kabuli_chr08	4290671	[A/T]	1067303358
Ca_Kabuli_NC_021167.1_4290706	Ca_kabuli_chr08	4290706	[T/C]	1067303359
Ca_Kabuli_NC_021167.1_4290707	Ca_kabuli_chr08	4290707	[C/G]	1067303360
Ca_Kabuli_NC_021167.1_4346497	Ca_kabuli_chr08	4346497	[G/T]	1067303361
Ca_Kabuli_NC_021167.1_4346706	Ca_kabuli_chr08	4346706	[T/A]	1067303362
Ca_Kabuli_NC_021167.1_4346724	Ca_kabuli_chr08	4346724	[A/G]	1067303363
Ca_Kabuli_NC_021167.1_4346765	Ca_kabuli_chr08	4346765	[A/G]	1067303364
Ca_Kabuli_NC_021167.1_4346803	Ca_kabuli_chr08	4346803	[A/C]	1067303365
Ca_Kabuli_NC_021167.1_4346860	Ca_kabuli_chr08	4346860	[A/C]	1067303366
Ca_Kabuli_NC_021167.1_4346890	Ca_kabuli_chr08	4346890	[A/G]	1067303367
Ca_Kabuli_NC_021167.1_4347532	Ca_kabuli_chr08	4347532	[A/C]	1067303368
Ca_Kabuli_NC_021167.1_4347535	Ca_kabuli_chr08	4347535	[A/C]	1067303369
Ca_Kabuli_NC_021167.1_4356492	Ca_kabuli_chr08	4356492	[T/G]	1067303370
Ca_Kabuli_NC_021167.1_4363714	Ca_kabuli_chr08	4363714	[A/G]	1067303371
Ca_Kabuli_NC_021167.1_4363715	Ca_kabuli_chr08	4363715	[A/G]	1067303372
Ca_Kabuli_NC_021167.1_4363716	Ca_kabuli_chr08	4363716	[A/G]	1067303373
Ca_Kabuli_NC_021167.1_4395606	Ca_kabuli_chr08	4395606	[C/A]	1067303374
Ca_Kabuli_NC_021167.1_4406956	Ca_kabuli_chr08	4406956	[C/G]	1067303375
Ca_Kabuli_NC_021167.1_4421422	Ca_kabuli_chr08	4421422	[T/C]	1067303376
Ca_Kabuli_NC_021167.1_4421563	Ca_kabuli_chr08	4421563	[T/G]	1067303377
Ca_Kabuli_NC_021167.1_4421593	Ca_kabuli_chr08	4421593	[T/C]	1067303378
Ca_Kabuli_NC_021167.1_4421719	Ca_kabuli_chr08	4421719	[A/C]	1067303379
Ca_Kabuli_NC_021167.1_4430537	Ca_kabuli_chr08	4430537	[A/T]	1067303380
Ca_Kabuli_NC_021167.1_4476229	Ca_kabuli_chr08	4476229	[A/C]	1067303381
Ca_Kabuli_NC_021167.1_4483402	Ca_kabuli_chr08	4483402	[T/G]	1067303382
Ca_Kabuli_NC_021167.1_4484606	Ca_kabuli_chr08	4484606	[T/G]	1067303383
Ca_Kabuli_NC_021167.1_4512293	Ca_kabuli_chr08	4512293	[A/T]	1067303384
Ca_Kabuli_NC_021167.1_4512490	Ca_kabuli_chr08	4512490	[A/C]	1067303385
Ca_Kabuli_NC_021167.1_4542365	Ca_kabuli_chr08	4542365	[A/C]	1067303386

SNP IDs	Chromosomes	Physical positions (bp)	SNPs	NCBI SNPdb accession IDs (NCBI_ss#)
Ca_Kabuli_NC_021167.1_4555825	Ca_kabuli_chr08	4555825	[T/C]	1067303387
Ca_Kabuli_NC_021167.1_4556684	Ca_kabuli_chr08	4556684	[G/T]	1067303388
Ca_Kabuli_NC_021167.1_4565731	Ca_kabuli_chr08	4565731	[T/G]	1067303389
Ca_Kabuli_NC_021167.1_4580453	Ca_kabuli_chr08	4580453	[A/C]	1067303390
Ca_Kabuli_NC_021167.1_4611043	Ca_kabuli_chr08	4611043	[A/G]	1067303391
Ca_Kabuli_NC_021167.1_4611125	Ca_kabuli_chr08	4611125	[T/C]	1067303392
Ca_Kabuli_NC_021167.1_4611893	Ca_kabuli_chr08	4611893	[A/T]	1067303393
Ca_Kabuli_NC_021167.1_4614681	Ca_kabuli_chr08	4614681	[G/T]	1067303394
Ca_Kabuli_NC_021167.1_4714590	Ca_kabuli_chr08	4714590	[T/G]	1067303395
Ca_Kabuli_NC_021167.1_4764559	Ca_kabuli_chr08	4764559	[A/C]	1067303396
Ca_Kabuli_NC_021167.1_4773207	Ca_kabuli_chr08	4773207	[A/G]	1067303397
Ca_Kabuli_NC_021167.1_4807080	Ca_kabuli_chr08	4807080	[G/T]	1067303398
Ca_Kabuli_NC_021167.1_4807088	Ca_kabuli_chr08	4807088	[T/G]	1067303399
Ca_Kabuli_NC_021167.1_4807090	Ca_kabuli_chr08	4807090	[C/T]	1067303400
Ca_Kabuli_NC_021167.1_4807094	Ca_kabuli_chr08	4807094	[A/G]	1067303401
Ca_Kabuli_NC_021167.1_4807099	Ca_kabuli_chr08	4807099	[A/C]	1067303402
Ca_Kabuli_NC_021167.1_4807106	Ca_kabuli_chr08	4807106	[G/A]	1067303403
Ca_Kabuli_NC_021167.1_4810601	Ca_kabuli_chr08	4810601	[A/T]	1067303404
Ca_Kabuli_NC_021167.1_4895122	Ca_kabuli_chr08	4895122	[A/C]	1067303405
Ca_Kabuli_NC_021167.1_4895123	Ca_kabuli_chr08	4895123	[G/T]	1067303406
Ca_Kabuli_NC_021167.1_5196989	Ca_kabuli_chr08	5196989	[T/C]	1067303407
Ca_Kabuli_NC_021167.1_5196992	Ca_kabuli_chr08	5196992	[T/A]	1067303408
Ca_Kabuli_NC_021167.1_5197033	Ca_kabuli_chr08	5197033	[A/G]	1067303409
Ca_Kabuli_NC_021167.1_5197035	Ca_kabuli_chr08	5197035	[A/C]	1067303410
Ca_Kabuli_NC_021167.1_5197287	Ca_kabuli_chr08	5197287	[A/G]	1067303411
Ca_Kabuli_NC_021167.1_5233627	Ca_kabuli_chr08	5233627	[T/C]	1067303412
Ca_Kabuli_NC_021167.1_5233635	Ca_kabuli_chr08	5233635	[T/C]	1067303413
Ca_Kabuli_NC_021167.1_5233668	Ca_kabuli_chr08	5233668	[A/T]	1067303414
Ca_Kabuli_NC_021167.1_5238280	Ca_kabuli_chr08	5238280	[A/C]	1067303415
Ca_Kabuli_NC_021167.1_5238282	Ca_kabuli_chr08	5238282	[T/C]	1067303416
Ca_Kabuli_NC_021167.1_5238284	Ca_kabuli_chr08	5238284	[T/A]	1067303417
Ca_Kabuli_NC_021167.1_5238288	Ca_kabuli_chr08	5238288	[A/C]	1067303418
Ca_Kabuli_NC_021167.1_5238308	Ca_kabuli_chr08	5238308	[A/G]	1067303419
Ca_Kabuli_NC_021167.1_5352588	Ca_kabuli_chr08	5352588	[A/G]	1067303420

SNP IDs	Chromosomes	Physical positions (bp)	SNPs	NCBI SNPdb accession IDs (NCBI_ss#)
Ca_Kabuli_NC_021167.1_5395004	Ca_kabuli_chr08	5395004	[A/C]	1067303421
Ca_Kabuli_NC_021167.1_5399553	Ca_kabuli_chr08	5399553	[A/C]	1067303422
Ca_Kabuli_NC_021167.1_5399562	Ca_kabuli_chr08	5399562	[T/G]	1067303423
Ca_Kabuli_NC_021167.1_5487879	Ca_kabuli_chr08	5487879	[A/G]	1067303424
Ca_Kabuli_NC_021167.1_5487890	Ca_kabuli_chr08	5487890	[A/G]	1067303425
Ca_Kabuli_NC_021167.1_5536327	Ca_kabuli_chr08	5536327	[T/G]	1067303426
Ca_Kabuli_NC_021167.1_5539039	Ca_kabuli_chr08	5539039	[A/C]	1067303427
Ca_Kabuli_NC_021167.1_5539042	Ca_kabuli_chr08	5539042	[A/C]	1067303428
Ca_Kabuli_NC_021167.1_5568446	Ca_kabuli_chr08	5568446	[A/C]	1067303429
Ca_Kabuli_NC_021167.1_5586096	Ca_kabuli_chr08	5586096	[A/T]	1067303430
Ca_Kabuli_NC_021167.1_5795614	Ca_kabuli_chr08	5795614	[C/A]	1067303431
Ca_Kabuli_NC_021167.1_5798565	Ca_kabuli_chr08	5798565	[A/C]	1067303432
Ca_Kabuli_NC_021167.1_5804741	Ca_kabuli_chr08	5804741	[A/C]	1067303433
Ca_Kabuli_NC_021167.1_5811121	Ca_kabuli_chr08	5811121	[T/G]	1067303434
Ca_Kabuli_NC_021167.1_5816919	Ca_kabuli_chr08	5816919	[T/C]	1067303435
Ca_Kabuli_NC_021167.1_5816940	Ca_kabuli_chr08	5816940	[T/A]	1067303436
Ca_Kabuli_NC_021167.1_5819130	Ca_kabuli_chr08	5819130	[T/C]	1067303437
Ca_Kabuli_NC_021167.1_5819186	Ca_kabuli_chr08	5819186	[A/G]	1067303438
Ca_Kabuli_NC_021167.1_5870503	Ca_kabuli_chr08	5870503	[A/C]	1067303439
Ca_Kabuli_NC_021167.1_5870589	Ca_kabuli_chr08	5870589	[A/G]	1067303440
Ca_Kabuli_NC_021167.1_5870616	Ca_kabuli_chr08	5870616	[T/C]	1067303441
Ca_Kabuli_NC_021167.1_5870619	Ca_kabuli_chr08	5870619	[T/A]	1067303442
Ca_Kabuli_NC_021167.1_5870626	Ca_kabuli_chr08	5870626	[T/C]	1067303443
Ca_Kabuli_NC_021167.1_5870634	Ca_kabuli_chr08	5870634	[A/C]	1067303444
Ca_Kabuli_NC_021167.1_5870646	Ca_kabuli_chr08	5870646	[A/G]	1067303445
Ca_Kabuli_NC_021167.1_5870664	Ca_kabuli_chr08	5870664	[T/C]	1067303446
Ca_Kabuli_NC_021167.1_5870747	Ca_kabuli_chr08	5870747	[T/C]	1067303447
Ca_Kabuli_NC_021167.1_5871361	Ca_kabuli_chr08	5871361	[T/G]	1067303448
Ca_Kabuli_NC_021167.1_5871364	Ca_kabuli_chr08	5871364	[T/G]	1067303449
Ca_Kabuli_NC_021167.1_5871367	Ca_kabuli_chr08	5871367	[T/G]	1067303450
Ca_Kabuli_NC_021167.1_5928093	Ca_kabuli_chr08	5928093	[C/A]	1067303451
Ca_Kabuli_NC_021167.1_5928094	Ca_kabuli_chr08	5928094	[A/G]	1067303452
Ca_Kabuli_NC_021167.1_5928096	Ca_kabuli_chr08	5928096	[A/T]	1067303453
Ca_Kabuli_NC_021167.1_5928097	Ca_kabuli_chr08	5928097	[G/C]	1067303454

SNP IDs	Chromosomes	Physical positions (bp)	SNPs	NCBI SNPdb accession IDs (NCBI_ss#)
Ca_Kabuli_NC_021167.1_5928610	Ca_kabuli_chr08	5928610	[T/C]	1067303455
Ca_Kabuli_NC_021167.1_5935392	Ca_kabuli_chr08	5935392	[A/G]	1067303456
Ca_Kabuli_NC_021167.1_5935430	Ca_kabuli_chr08	5935430	[T/G]	1067303457
Ca_Kabuli_NC_021167.1_5940320	Ca_kabuli_chr08	5940320	[T/G]	1067303458
Ca_Kabuli_NC_021167.1_5940336	Ca_kabuli_chr08	5940336	[A/C]	1067303459
Ca_Kabuli_NC_021167.1_6019988	Ca_kabuli_chr08	6019988	[T/A]	1067303460
Ca_Kabuli_NC_021167.1_6026487	Ca_kabuli_chr08	6026487	[T/G]	1067303461
Ca_Kabuli_NC_021167.1_6030777	Ca_kabuli_chr08	6030777	[A/C]	1067303462
Ca_Kabuli_NC_021167.1_6192937	Ca_kabuli_chr08	6192937	[A/G]	1067303463
Ca_Kabuli_NC_021167.1_6192944	Ca_kabuli_chr08	6192944	[T/G]	1067303464
Ca_Kabuli_NC_021167.1_6192949	Ca_kabuli_chr08	6192949	[T/A]	1067303465
Ca_Kabuli_NC_021167.1_6193075	Ca_kabuli_chr08	6193075	[G/A]	1067303466
Ca_Kabuli_NC_021167.1_6193078	Ca_kabuli_chr08	6193078	[G/A]	1067303467
Ca_Kabuli_NC_021167.1_6261325	Ca_kabuli_chr08	6261325	[A/C]	1067303468
Ca_Kabuli_NC_021167.1_6266307	Ca_kabuli_chr08	6266307	[C/T]	1067303469
Ca_Kabuli_NC_021167.1_6266308	Ca_kabuli_chr08	6266308	[T/C]	1067303470
Ca_Kabuli_NC_021167.1_6278623	Ca_kabuli_chr08	6278623	[A/G]	1067303471
Ca_Kabuli_NC_021167.1_6302023	Ca_kabuli_chr08	6302023	[T/C]	1067303472
Ca_Kabuli_NC_021167.1_6477772	Ca_kabuli_chr08	6477772	[G/T]	1067303473
Ca_Kabuli_NC_021167.1_6477775	Ca_kabuli_chr08	6477775	[T/G]	1067303474
Ca_Kabuli_NC_021167.1_6477777	Ca_kabuli_chr08	6477777	[C/T]	1067303475
Ca_Kabuli_NC_021167.1_6544246	Ca_kabuli_chr08	6544246	[G/T]	1067303476
Ca_Kabuli_NC_021167.1_6549053	Ca_kabuli_chr08	6549053	[T/G]	1067303477
Ca_Kabuli_NC_021167.1_6600793	Ca_kabuli_chr08	6600793	[A/G]	1067303478
Ca_Kabuli_NC_021167.1_6600810	Ca_kabuli_chr08	6600810	[T/G]	1067303479
Ca_Kabuli_NC_021167.1_6600820	Ca_kabuli_chr08	6600820	[T/G]	1067303480
Ca_Kabuli_NC_021167.1_6600838	Ca_kabuli_chr08	6600838	[T/G]	1067303481
Ca_Kabuli_NC_021167.1_6630623	Ca_kabuli_chr08	6630623	[A/T]	1067303482
Ca_Kabuli_NC_021167.1_6630627	Ca_kabuli_chr08	6630627	[A/G]	1067303483
Ca_Kabuli_NC_021167.1_6630666	Ca_kabuli_chr08	6630666	[T/C]	1067303484
Ca_Kabuli_NC_021167.1_6630675	Ca_kabuli_chr08	6630675	[T/A]	1067303485
Ca_Kabuli_NC_021167.1_6677430	Ca_kabuli_chr08	6677430	[T/C]	1067303486
Ca_Kabuli_NC_021167.1_6677471	Ca_kabuli_chr08	6677471	[A/G]	1067303487
Ca_Kabuli_NC_021167.1_6708909	Ca_kabuli_chr08	6708909	[T/G]	1067303488

SNP IDs	Chromosomes	Physical positions (bp)	SNPs	NCBI SNPdb accession IDs (NCBI_ss#)
Ca_Kabuli_NC_021167.1_6737822	Ca_kabuli_chr08	6737822	[T/C]	1067303489
Ca_Kabuli_NC_021167.1_6737842	Ca_kabuli_chr08	6737842	[G/C]	1067303490
Ca_Kabuli_NC_021167.1_6737848	Ca_kabuli_chr08	6737848	[T/C]	1067303491
Ca_Kabuli_NC_021167.1_6737867	Ca_kabuli_chr08	6737867	[A/G]	1067303492
Ca_Kabuli_NC_021167.1_6747753	Ca_kabuli_chr08	6747753	[T/A]	1067303493
Ca_Kabuli_NC_021167.1_6747755	Ca_kabuli_chr08	6747755	[A/C]	1067303494
Ca_Kabuli_NC_021167.1_6747756	Ca_kabuli_chr08	6747756	[G/T]	1067303495
Ca_Kabuli_NC_021167.1_6748252	Ca_kabuli_chr08	6748252	[T/A]	1067303496
Ca_Kabuli_NC_021167.1_6748253	Ca_kabuli_chr08	6748253	[A/T]	1067303497
Ca_Kabuli_NC_021167.1_6794586	Ca_kabuli_chr08	6794586	[T/G]	1067303498
Ca_Kabuli_NC_021167.1_6941567	Ca_kabuli_chr08	6941567	[A/C]	1067303499
Ca_Kabuli_NC_021167.1_6968084	Ca_kabuli_chr08	6968084	[T/C]	1067303500
Ca_Kabuli_NC_021167.1_6986280	Ca_kabuli_chr08	6986280	[A/C]	1067303501
Ca_Kabuli_NC_021167.1_6987551	Ca_kabuli_chr08	6987551	[T/C]	1067303502
Ca_Kabuli_NC_021167.1_6989447	Ca_kabuli_chr08	6989447	[A/C]	1067303503
Ca_Kabuli_NC_021167.1_7037845	Ca_kabuli_chr08	7037845	[T/C]	1067303504
Ca_Kabuli_NC_021167.1_7037855	Ca_kabuli_chr08	7037855	[C/G]	1067303505
Ca_Kabuli_NC_021167.1_7041792	Ca_kabuli_chr08	7041792	[T/C]	1067303506
Ca_Kabuli_NC_021167.1_7041823	Ca_kabuli_chr08	7041823	[A/G]	1067303507
Ca_Kabuli_NC_021167.1_7099572	Ca_kabuli_chr08	7099572	[A/C]	1067303508
Ca_Kabuli_NC_021167.1_7165021	Ca_kabuli_chr08	7165021	[A/T]	1067303509
Ca_Kabuli_NC_021167.1_7165063	Ca_kabuli_chr08	7165063	[A/T]	1067303510
Ca_Kabuli_NC_021167.1_7165070	Ca_kabuli_chr08	7165070	[A/G]	1067303511
Ca_Kabuli_NC_021167.1_7174094	Ca_kabuli_chr08	7174094	[T/A]	1067303512
Ca_Kabuli_NC_021167.1_7191898	Ca_kabuli_chr08	7191898	[G/A]	1067303513
Ca_Kabuli_NC_021167.1_7191904	Ca_kabuli_chr08	7191904	[C/A]	1067303514
Ca_Kabuli_NC_021167.1_7191930	Ca_kabuli_chr08	7191930	[T/C]	1067303515
Ca_Kabuli_NC_021167.1_7419447	Ca_kabuli_chr08	7419447	[T/C]	1067303516
Ca_Kabuli_NC_021167.1_7475051	Ca_kabuli_chr08	7475051	[A/C]	1067303517
Ca_Kabuli_NC_021167.1_7475187	Ca_kabuli_chr08	7475187	[A/C]	1067303518
Ca_Kabuli_NC_021167.1_7475192	Ca_kabuli_chr08	7475192	[A/G]	1067303519
Ca_Kabuli_NC_021167.1_7475249	Ca_kabuli_chr08	7475249	[T/A]	1067303520
Ca_Kabuli_NC_021167.1_7506032	Ca_kabuli_chr08	7506032	[T/C]	1067303521
Ca_Kabuli_NC_021167.1_7506042	Ca_kabuli_chr08	7506042	[A/C]	1067303522

SNP IDs	Chromosomes	Physical positions (bp)	SNPs	NCBI SNPdb accession IDs (NCBI_ss#)
Ca_Kabuli_NC_021167.1_7563373	Ca_kabuli_chr08	7563373	[T/A]	1067303523
Ca_Kabuli_NC_021167.1_7563378	Ca_kabuli_chr08	7563378	[T/A]	1067303524
Ca_Kabuli_NC_021167.1_7563382	Ca_kabuli_chr08	7563382	[C/G]	1067303525
Ca_Kabuli_NC_021167.1_7563387	Ca_kabuli_chr08	7563387	[T/A]	1067303526
Ca_Kabuli_NC_021167.1_7563650	Ca_kabuli_chr08	7563650	[A/C]	1067303527
Ca_Kabuli_NC_021167.1_7566603	Ca_kabuli_chr08	7566603	[A/T]	1067303528
Ca_Kabuli_NC_021167.1_7613558	Ca_kabuli_chr08	7613558	[A/G]	1067303529
Ca_Kabuli_NC_021167.1_7614314	Ca_kabuli_chr08	7614314	[A/C]	1067303530
Ca_Kabuli_NC_021167.1_7614319	Ca_kabuli_chr08	7614319	[T/C]	1067303531
Ca_Kabuli_NC_021167.1_7628694	Ca_kabuli_chr08	7628694	[A/G]	1067303532
Ca_Kabuli_NC_021167.1_7694692	Ca_kabuli_chr08	7694692	[A/C]	1067303533
Ca_Kabuli_NC_021167.1_7694771	Ca_kabuli_chr08	7694771	[T/G]	1067303534
Ca_Kabuli_NC_021167.1_7696107	Ca_kabuli_chr08	7696107	[T/A]	1067303535
Ca_Kabuli_NC_021167.1_7696112	Ca_kabuli_chr08	7696112	[T/G]	1067303536
Ca_Kabuli_NC_021167.1_7696220	Ca_kabuli_chr08	7696220	[A/T]	1067303537
Ca_Kabuli_NC_021167.1_7699161	Ca_kabuli_chr08	7699161	[T/G]	1067303538
Ca_Kabuli_NC_021167.1_7699179	Ca_kabuli_chr08	7699179	[A/T]	1067303539
Ca_Kabuli_NC_021167.1_7699194	Ca_kabuli_chr08	7699194	[T/C]	1067303540
Ca_Kabuli_NC_021167.1_7733604	Ca_kabuli_chr08	7733604	[T/A]	1067303541
Ca_Kabuli_NC_021167.1_7733620	Ca_kabuli_chr08	7733620	[A/T]	1067303542
Ca_Kabuli_NC_021167.1_7733671	Ca_kabuli_chr08	7733671	[T/C]	1067303543
Ca_Kabuli_NC_021167.1_7734545	Ca_kabuli_chr08	7734545	[T/G]	1067303544
Ca_Kabuli_NC_021167.1_7771232	Ca_kabuli_chr08	7771232	[A/T]	1067303545
Ca_Kabuli_NC_021167.1_7775908	Ca_kabuli_chr08	7775908	[G/T]	1067303546
Ca_Kabuli_NC_021167.1_7776897	Ca_kabuli_chr08	7776897	[A/T]	1067303547
Ca_Kabuli_NC_021167.1_7977246	Ca_kabuli_chr08	7977246	[C/G]	1067303548
Ca_Kabuli_NC_021167.1_7977260	Ca_kabuli_chr08	7977260	[A/C]	1067303549
Ca_Kabuli_NC_021167.1_7977306	Ca_kabuli_chr08	7977306	[T/C]	1067303550
Ca_Kabuli_NC_021167.1_7977314	Ca_kabuli_chr08	7977314	[T/G]	1067303551
Ca_Kabuli_NC_021167.1_7978219	Ca_kabuli_chr08	7978219	[A/G]	1067303552
Ca_Kabuli_NC_021167.1_8029278	Ca_kabuli_chr08	8029278	[A/C]	1067303553
Ca_Kabuli_NC_021167.1_8029289	Ca_kabuli_chr08	8029289	[T/C]	1067303554
Ca_Kabuli_NC_021167.1_8059901	Ca_kabuli_chr08	8059901	[A/T]	1067303555
Ca_Kabuli_NC_021167.1_8066006	Ca_kabuli_chr08	8066006	[A/C]	1067303556

SNP IDs	Chromosomes	Physical positions (bp)	SNPs	NCBI SNPdb accession IDs (NCBI_ss#)
Ca_Kabuli_NC_021167.1_8096681	Ca_kabuli_chr08	8096681	[T/G]	1067303557
Ca_Kabuli_NC_021167.1_8194268	Ca_kabuli_chr08	8194268	[T/G]	1067303558
Ca_Kabuli_NC_021167.1_8194279	Ca_kabuli_chr08	8194279	[C/T]	1067303559
Ca_Kabuli_NC_021167.1_8202723	Ca_kabuli_chr08	8202723	[T/C]	1067303560
Ca_Kabuli_NC_021167.1_8232428	Ca_kabuli_chr08	8232428	[T/C]	1067303561
Ca_Kabuli_NC_021167.1_8232469	Ca_kabuli_chr08	8232469	[A/C]	1067303562
Ca_Kabuli_NC_021167.1_8232488	Ca_kabuli_chr08	8232488	[A/T]	1067303563
Ca_Kabuli_NC_021167.1_8270652	Ca_kabuli_chr08	8270652	[A/C]	1067303564
Ca_Kabuli_NC_021167.1_8389108	Ca_kabuli_chr08	8389108	[A/C]	1067303565
Ca_Kabuli_NC_021167.1_8452457	Ca_kabuli_chr08	8452457	[T/C]	1067303566
Ca_Kabuli_NC_021167.1_8509462	Ca_kabuli_chr08	8509462	[C/T]	1067303567
Ca_Kabuli_NC_021167.1_8509469	Ca_kabuli_chr08	8509469	[C/T]	1067303568
Ca_Kabuli_NC_021167.1_8509493	Ca_kabuli_chr08	8509493	[A/G]	1067303569
Ca_Kabuli_NC_021167.1_8509497	Ca_kabuli_chr08	8509497	[A/G]	1067303570
Ca_Kabuli_NC_021167.1_8533479	Ca_kabuli_chr08	8533479	[G/T]	1067303571
Ca_Kabuli_NC_021167.1_8533481	Ca_kabuli_chr08	8533481	[C/G]	1067303572
Ca_Kabuli_NC_021167.1_8560271	Ca_kabuli_chr08	8560271	[A/C]	1067303573
Ca_Kabuli_NC_021167.1_8560283	Ca_kabuli_chr08	8560283	[A/C]	1067303574
Ca_Kabuli_NC_021167.1_8560313	Ca_kabuli_chr08	8560313	[A/T]	1067303575
Ca_Kabuli_NC_021167.1_8560315	Ca_kabuli_chr08	8560315	[C/T]	1067303576
Ca_Kabuli_NC_021167.1_8560328	Ca_kabuli_chr08	8560328	[T/C]	1067303577
Ca_Kabuli_NC_021167.1_8560334	Ca_kabuli_chr08	8560334	[C/T]	1067303578
Ca_Kabuli_NC_021167.1_8560337	Ca_kabuli_chr08	8560337	[T/C]	1067303579
Ca_Kabuli_NC_021167.1_8560341	Ca_kabuli_chr08	8560341	[C/T]	1067303580
Ca_Kabuli_NC_021167.1_8560360	Ca_kabuli_chr08	8560360	[T/C]	1067303581
Ca_Kabuli_NC_021167.1_8560362	Ca_kabuli_chr08	8560362	[T/C]	1067303582
Ca_Kabuli_NC_021167.1_8560378	Ca_kabuli_chr08	8560378	[T/C]	1067303583
Ca_Kabuli_NC_021167.1_8661236	Ca_kabuli_chr08	8661236	[T/C]	1067303584
Ca_Kabuli_NC_021167.1_8661255	Ca_kabuli_chr08	8661255	[T/C]	1067303585
Ca_Kabuli_NC_021167.1_8661315	Ca_kabuli_chr08	8661315	[T/A]	1067303586
Ca_Kabuli_NC_021167.1_8842341	Ca_kabuli_chr08	8842341	[T/G]	1067303587
Ca_Kabuli_NC_021167.1_8842365	Ca_kabuli_chr08	8842365	[T/G]	1067303588
Ca_Kabuli_NC_021167.1_8842409	Ca_kabuli_chr08	8842409	[C/T]	1067303589
Ca_Kabuli_NC_021167.1_8842418	Ca_kabuli_chr08	8842418	[A/G]	1067303590

SNP IDs	Chromosomes	Physical positions (bp)	SNPs	NCBI SNPdb accession IDs (NCBI_ss#)
Ca_Kabuli_NC_021167.1_8887172	Ca_kabuli_chr08	8887172	[A/G]	1067303591
Ca_Kabuli_NC_021167.1_8888718	Ca_kabuli_chr08	8888718	[A/G]	1067303592
Ca_Kabuli_NC_021167.1_8888748	Ca_kabuli_chr08	8888748	[T/C]	1067303593
Ca_Kabuli_NC_021167.1_8902939	Ca_kabuli_chr08	8902939	[C/T]	1067303594
Ca_Kabuli_NC_021167.1_8902962	Ca_kabuli_chr08	8902962	[A/G]	1067303595
Ca_Kabuli_NC_021167.1_8904365	Ca_kabuli_chr08	8904365	[T/A]	1067303596
Ca_Kabuli_NC_021167.1_8904366	Ca_kabuli_chr08	8904366	[A/G]	1067303597
Ca_Kabuli_NC_021167.1_8911466	Ca_kabuli_chr08	8911466	[G/A]	1067303598
Ca_Kabuli_NC_021167.1_9064683	Ca_kabuli_chr08	9064683	[A/T]	1067303599
Ca_Kabuli_NC_021167.1_9064805	Ca_kabuli_chr08	9064805	[A/G]	1067303600
Ca_Kabuli_NC_021167.1_9064924	Ca_kabuli_chr08	9064924	[T/A]	1067303601
Ca_Kabuli_NC_021167.1_9097271	Ca_kabuli_chr08	9097271	[A/T]	1067303602
Ca_Kabuli_NC_021167.1_9097297	Ca_kabuli_chr08	9097297	[C/A]	1067303603
Ca_Kabuli_NC_021167.1_9097300	Ca_kabuli_chr08	9097300	[G/C]	1067303604
Ca_Kabuli_NC_021167.1_9097585	Ca_kabuli_chr08	9097585	[A/C]	1067303605
Ca_Kabuli_NC_021167.1_9099798	Ca_kabuli_chr08	9099798	[G/T]	1067303606
Ca_Kabuli_NC_021167.1_9099900	Ca_kabuli_chr08	9099900	[T/G]	1067303607
Ca_Kabuli_NC_021167.1_9099901	Ca_kabuli_chr08	9099901	[G/C]	1067303608
Ca_Kabuli_NC_021167.1_9100111	Ca_kabuli_chr08	9100111	[C/T]	1067303609
Ca_Kabuli_NC_021167.1_9100160	Ca_kabuli_chr08	9100160	[T/C]	1067303610
Ca_Kabuli_NC_021167.1_9110059	Ca_kabuli_chr08	9110059	[A/G]	1067303611
Ca_Kabuli_NC_021167.1_9112109	Ca_kabuli_chr08	9112109	[A/C]	1067303612
Ca_Kabuli_NC_021167.1_9112112	Ca_kabuli_chr08	9112112	[A/C]	1067303613
Ca_Kabuli_NC_021167.1_9112117	Ca_kabuli_chr08	9112117	[A/C]	1067303614
Ca_Kabuli_NC_021167.1_9112169	Ca_kabuli_chr08	9112169	[A/C]	1067303615
Ca_Kabuli_NC_021167.1_9112172	Ca_kabuli_chr08	9112172	[A/C]	1067303616
Ca_Kabuli_NC_021167.1_9112184	Ca_kabuli_chr08	9112184	[A/C]	1067303617
Ca_Kabuli_NC_021167.1_9112190	Ca_kabuli_chr08	9112190	[C/T]	1067303618
Ca_Kabuli_NC_021167.1_9112199	Ca_kabuli_chr08	9112199	[A/C]	1067303619
Ca_Kabuli_NC_021167.1_9118255	Ca_kabuli_chr08	9118255	[A/C]	1067303620
Ca_Kabuli_NC_021167.1_9118582	Ca_kabuli_chr08	9118582	[C/T]	1067303621
Ca_Kabuli_NC_021167.1_9129997	Ca_kabuli_chr08	9129997	[A/G]	1067303622
Ca_Kabuli_NC_021167.1_9130000	Ca_kabuli_chr08	9130000	[T/G]	1067303623
Ca_Kabuli_NC_021167.1_9352700	Ca_kabuli_chr08	9352700	[G/A]	1067303624

SNP IDs	Chromosomes	Physical positions (bp)	SNPs	NCBI SNPdb accession IDs (NCBI_ss#)
Ca_Kabuli_NC_021167.1_9366845	Ca_kabuli_chr08	9366845	[T/A]	1067303625
Ca_Kabuli_NC_021167.1_9371383	Ca_kabuli_chr08	9371383	[A/G]	1067303626
Ca_Kabuli_NC_021167.1_9371396	Ca_kabuli_chr08	9371396	[A/G]	1067303627
Ca_Kabuli_NC_021167.1_9371402	Ca_kabuli_chr08	9371402	[T/C]	1067303628
Ca_Kabuli_NC_021167.1_9383023	Ca_kabuli_chr08	9383023	[C/A]	1067303629
Ca_Kabuli_NC_021167.1_9386614	Ca_kabuli_chr08	9386614	[T/G]	1067303630
Ca_Kabuli_NC_021167.1_9386626	Ca_kabuli_chr08	9386626	[A/T]	1067303631
Ca_Kabuli_NC_021167.1_9386678	Ca_kabuli_chr08	9386678	[C/T]	1067303632
Ca_Kabuli_NC_021167.1_9386682	Ca_kabuli_chr08	9386682	[C/T]	1067303633
Ca_Kabuli_NC_021167.1_9386685	Ca_kabuli_chr08	9386685	[C/A]	1067303634
Ca_Kabuli_NC_021167.1_9386686	Ca_kabuli_chr08	9386686	[A/C]	1067303635
Ca_Kabuli_NC_021167.1_9386701	Ca_kabuli_chr08	9386701	[A/C]	1067303636
Ca_Kabuli_NC_021167.1_9386722	Ca_kabuli_chr08	9386722	[A/G]	1067303637
Ca_Kabuli_NC_021167.1_9387656	Ca_kabuli_chr08	9387656	[G/T]	1067303638
Ca_Kabuli_NC_021167.1_9387668	Ca_kabuli_chr08	9387668	[T/C]	1067303639
Ca_Kabuli_NC_021167.1_9387680	Ca_kabuli_chr08	9387680	[A/C]	1067303640
Ca_Kabuli_NC_021167.1_9388734	Ca_kabuli_chr08	9388734	[A/T]	1067303641
Ca_Kabuli_NC_021167.1_10004054	Ca_kabuli_chr08	10004054	[T/G]	1067303642
Ca_Kabuli_NC_021167.1_10004490	Ca_kabuli_chr08	10004490	[A/T]	1067303643
Ca_Kabuli_NC_021167.1_10080270	Ca_kabuli_chr08	10080270	[G/T]	1067303644
Ca_Kabuli_NC_021167.1_10080287	Ca_kabuli_chr08	10080287	[A/G]	1067303645
Ca_Kabuli_NC_021167.1_10080319	Ca_kabuli_chr08	10080319	[A/G]	1067303646
Ca_Kabuli_NC_021167.1_10080348	Ca_kabuli_chr08	10080348	[T/G]	1067303647
Ca_Kabuli_NC_021167.1_10080361	Ca_kabuli_chr08	10080361	[T/G]	1067303648
Ca_Kabuli_NC_021167.1_10147041	Ca_kabuli_chr08	10147041	[A/G]	1067303649
Ca_Kabuli_NC_021167.1_10155981	Ca_kabuli_chr08	10155981	[A/G]	1067303650
Ca_Kabuli_NC_021167.1_10257576	Ca_kabuli_chr08	10257576	[T/G]	1067303651
Ca_Kabuli_NC_021167.1_10257577	Ca_kabuli_chr08	10257577	[A/T]	1067303652
Ca_Kabuli_NC_021167.1_10257601	Ca_kabuli_chr08	10257601	[A/G]	1067303653
Ca_Kabuli_NC_021167.1_10257640	Ca_kabuli_chr08	10257640	[A/G]	1067303654
Ca_Kabuli_NC_021167.1_10257647	Ca_kabuli_chr08	10257647	[C/T]	1067303655
Ca_Kabuli_NC_021167.1_10258920	Ca_kabuli_chr08	10258920	[T/G]	1067303656
Ca_Kabuli_NC_021167.1_10258924	Ca_kabuli_chr08	10258924	[A/G]	1067303657
Ca_Kabuli_NC_021167.1_10261903	Ca_kabuli_chr08	10261903	[T/G]	1067303658

SNP IDs	Chromosomes	Physical positions (bp)	SNPs	NCBI SNPdb accession IDs (NCBI_ss#)
Ca_Kabuli_NC_021167.1_10261981	Ca_kabuli_chr08	10261981	[T/C]	1067303659
Ca_Kabuli_NC_021167.1_10354329	Ca_kabuli_chr08	10354329	[A/G]	1067303660
Ca_Kabuli_NC_021167.1_10354334	Ca_kabuli_chr08	10354334	[T/G]	1067303661
Ca_Kabuli_NC_021167.1_10354348	Ca_kabuli_chr08	10354348	[A/G]	1067303662
Ca_Kabuli_NC_021167.1_10434008	Ca_kabuli_chr08	10434008	[A/C]	1067303663
Ca_Kabuli_NC_021167.1_10436667	Ca_kabuli_chr08	10436667	[A/C]	1067303664
Ca_Kabuli_NC_021167.1_10452098	Ca_kabuli_chr08	10452098	[A/T]	1067303665
Ca_Kabuli_NC_021167.1_10452101	Ca_kabuli_chr08	10452101	[A/G]	1067303666
Ca_Kabuli_NC_021167.1_10452982	Ca_kabuli_chr08	10452982	[A/C]	1067303667
Ca_Kabuli_NC_021167.1_10556112	Ca_kabuli_chr08	10556112	[A/C]	1067303668
Ca_Kabuli_NC_021167.1_10786843	Ca_kabuli_chr08	10786843	[C/A]	1067303669
Ca_Kabuli_NC_021167.1_10815888	Ca_kabuli_chr08	10815888	[A/G]	1067303670
Ca_Kabuli_NC_021167.1_10815904	Ca_kabuli_chr08	10815904	[T/A]	1067303671
Ca_Kabuli_NC_021167.1_10822123	Ca_kabuli_chr08	10822123	[A/G]	1067303672
Ca_Kabuli_NC_021167.1_10887267	Ca_kabuli_chr08	10887267	[T/A]	1067303673
Ca_Kabuli_NC_021167.1_10887272	Ca_kabuli_chr08	10887272	[T/A]	1067303674
Ca_Kabuli_NC_021167.1_10887292	Ca_kabuli_chr08	10887292	[C/T]	1067303675
Ca_Kabuli_NC_021167.1_10887296	Ca_kabuli_chr08	10887296	[C/T]	1067303676
Ca_Kabuli_NC_021167.1_10887558	Ca_kabuli_chr08	10887558	[T/G]	1067303677
Ca_Kabuli_NC_021167.1_10887580	Ca_kabuli_chr08	10887580	[C/T]	1067303678
Ca_Kabuli_NC_021167.1_10887602	Ca_kabuli_chr08	10887602	[G/T]	1067303679
Ca_Kabuli_NC_021167.1_10915747	Ca_kabuli_chr08	10915747	[C/T]	1067303680
Ca_Kabuli_NC_021167.1_11066644	Ca_kabuli_chr08	11066644	[C/T]	1067303681
Ca_Kabuli_NC_021167.1_11066728	Ca_kabuli_chr08	11066728	[G/A]	1067303682
Ca_Kabuli_NC_021167.1_11066975	Ca_kabuli_chr08	11066975	[T/C]	1067303683
Ca_Kabuli_NC_021167.1_11067014	Ca_kabuli_chr08	11067014	[G/A]	1067303684
Ca_Kabuli_NC_021167.1_11067090	Ca_kabuli_chr08	11067090	[C/T]	1067303685
Ca_Kabuli_NC_021167.1_11072880	Ca_kabuli_chr08	11072880	[G/A]	1067303686
Ca_Kabuli_NC_021167.1_11072896	Ca_kabuli_chr08	11072896	[C/T]	1067303687
Ca_Kabuli_NC_021167.1_11072914	Ca_kabuli_chr08	11072914	[T/C]	1067303688
Ca_Kabuli_NC_021167.1_11072923	Ca_kabuli_chr08	11072923	[G/A]	1067303689
Ca_Kabuli_NC_021167.1_11087123	Ca_kabuli_chr08	11087123	[C/T]	1067303690
Ca_Kabuli_NC_021167.1_11087143	Ca_kabuli_chr08	11087143	[A/G]	1067303691
Ca_Kabuli_NC_021167.1_11087302	Ca_kabuli_chr08	11087302	[A/T]	1067303692

SNP IDs	Chromosomes	Physical positions (bp)	SNPs	NCBI SNPdb accession IDs (NCBI_ss#)
Ca_Kabuli_NC_021167.1_11102884	Ca_kabuli_chr08	11102884	[T/G]	1067303693
Ca_Kabuli_NC_021167.1_11102892	Ca_kabuli_chr08	11102892	[T/A]	1067303694
Ca_Kabuli_NC_021167.1_11121469	Ca_kabuli_chr08	11121469	[A/C]	1067303695
Ca_Kabuli_NC_021167.1_11121777	Ca_kabuli_chr08	11121777	[G/A]	1067303696
Ca_Kabuli_NC_021167.1_11128030	Ca_kabuli_chr08	11128030	[T/A]	1067303697
Ca_Kabuli_NC_021167.1_11176583	Ca_kabuli_chr08	11176583	[G/T]	1067303698
Ca_Kabuli_NC_021167.1_11231820	Ca_kabuli_chr08	11231820	[A/G]	1067303699
Ca_Kabuli_NC_021167.1_11231834	Ca_kabuli_chr08	11231834	[A/G]	1067303700
Ca_Kabuli_NC_021167.1_11231837	Ca_kabuli_chr08	11231837	[A/G]	1067303701
Ca_Kabuli_NC_021167.1_11240205	Ca_kabuli_chr08	11240205	[T/G]	1067303702
Ca_Kabuli_NC_021167.1_11240211	Ca_kabuli_chr08	11240211	[T/G]	1067303703
Ca_Kabuli_NC_021167.1_11240212	Ca_kabuli_chr08	11240212	[A/T]	1067303704
Ca_Kabuli_NC_021167.1_11240234	Ca_kabuli_chr08	11240234	[A/G]	1067303705
Ca_Kabuli_NC_021167.1_11240258	Ca_kabuli_chr08	11240258	[A/G]	1067303706
Ca_Kabuli_NC_021167.1_11240408	Ca_kabuli_chr08	11240408	[T/G]	1067303707
Ca_Kabuli_NC_021167.1_11243243	Ca_kabuli_chr08	11243243	[T/C]	1067303708
Ca_Kabuli_NC_021167.1_11243256	Ca_kabuli_chr08	11243256	[A/C]	1067303709
Ca_Kabuli_NC_021167.1_11243263	Ca_kabuli_chr08	11243263	[A/C]	1067303710
Ca_Kabuli_NC_021167.1_11332970	Ca_kabuli_chr08	11332970	[T/A]	1067303711
Ca_Kabuli_NC_021167.1_11377770	Ca_kabuli_chr08	11377770	[T/G]	1067303712
Ca_Kabuli_NC_021167.1_11403805	Ca_kabuli_chr08	11403805	[A/G]	1067303713
Ca_Kabuli_NC_021167.1_11548440	Ca_kabuli_chr08	11548440	[T/G]	1067303714
Ca_Kabuli_NC_021167.1_11629709	Ca_kabuli_chr08	11629709	[T/C]	1067303715
Ca_Kabuli_NC_021167.1_11630126	Ca_kabuli_chr08	11630126	[A/C]	1067303716
Ca_Kabuli_NC_021167.1_11631807	Ca_kabuli_chr08	11631807	[T/G]	1067303717
Ca_Kabuli_NC_021167.1_11632814	Ca_kabuli_chr08	11632814	[A/G]	1067303718
Ca_Kabuli_NC_021167.1_11696117	Ca_kabuli_chr08	11696117	[A/G]	1067303719
Ca_Kabuli_NC_021167.1_11696123	Ca_kabuli_chr08	11696123	[A/C]	1067303720
Ca_Kabuli_NC_021167.1_11722520	Ca_kabuli_chr08	11722520	[T/A]	1067303721
Ca_Kabuli_NC_021167.1_11722522	Ca_kabuli_chr08	11722522	[A/T]	1067303722
Ca_Kabuli_NC_021167.1_11751059	Ca_kabuli_chr08	11751059	[A/T]	1067303723
Ca_Kabuli_NC_021167.1_11751065	Ca_kabuli_chr08	11751065	[A/G]	1067303724
Ca_Kabuli_NC_021167.1_11751093	Ca_kabuli_chr08	11751093	[T/G]	1067303725
Ca_Kabuli_NC_021167.1_11843704	Ca_kabuli_chr08	11843704	[G/T]	1067303726

SNP IDs	Chromosomes	Physical positions (bp)	SNPs	NCBI SNPdb accession IDs (NCBI_ss#)
Ca_Kabuli_NC_021167.1_11906090	Ca_kabuli_chr08	11906090	[T/G]	1067303727
Ca_Kabuli_NC_021167.1_11906095	Ca_kabuli_chr08	11906095	[T/G]	1067303728
Ca_Kabuli_NC_021167.1_12857978	Ca_kabuli_chr08	12857978	[A/C]	1067303729
Ca_Kabuli_NC_021167.1_12865268	Ca_kabuli_chr08	12865268	[T/A]	1067303730
Ca_Kabuli_NC_021167.1_13057906	Ca_kabuli_chr08	13057906	[A/T]	1067303731
Ca_Kabuli_NC_021167.1_13057957	Ca_kabuli_chr08	13057957	[A/G]	1067303732
Ca_Kabuli_NC_021167.1_13058012	Ca_kabuli_chr08	13058012	[A/T]	1067303733
Ca_Kabuli_NC_021167.1_13058018	Ca_kabuli_chr08	13058018	[A/T]	1067303734
Ca_Kabuli_NC_021167.1_13134787	Ca_kabuli_chr08	13134787	[T/G]	1067303735
Ca_Kabuli_NC_021167.1_13134791	Ca_kabuli_chr08	13134791	[T/G]	1067303736
Ca_Kabuli_NC_021167.1_13248268	Ca_kabuli_chr08	13248268	[T/C]	1067303737
Ca_Kabuli_NC_021167.1_13830326	Ca_kabuli_chr08	13830326	[T/G]	1067303738
Ca_Kabuli_NC_021167.1_13830332	Ca_kabuli_chr08	13830332	[A/G]	1067303739
Ca_Kabuli_NC_021167.1_13830341	Ca_kabuli_chr08	13830341	[A/G]	1067303740
Ca_Kabuli_NC_021167.1_13840715	Ca_kabuli_chr08	13840715	[G/A]	1067303741
Ca_Kabuli_NC_021167.1_13840721	Ca_kabuli_chr08	13840721	[T/C]	1067303742
Ca_Kabuli_NC_021167.1_13840741	Ca_kabuli_chr08	13840741	[G/C]	1067303743
Ca_Kabuli_NC_021167.1_13968426	Ca_kabuli_chr08	13968426	[A/G]	1067303744
Ca_Kabuli_NC_021167.1_13982906	Ca_kabuli_chr08	13982906	[T/A]	1067303745
Ca_Kabuli_NC_021167.1_13982907	Ca_kabuli_chr08	13982907	[C/A]	1067303746
Ca_Kabuli_NC_021167.1_13982908	Ca_kabuli_chr08	13982908	[T/G]	1067303747
Ca_Kabuli_NC_021167.1_14007360	Ca_kabuli_chr08	14007360	[A/T]	1067303748
Ca_Kabuli_NC_021167.1_14124588	Ca_kabuli_chr08	14124588	[C/A]	1067303749
Ca_Kabuli_NC_021167.1_14253920	Ca_kabuli_chr08	14253920	[G/A]	1067303750
Ca_Kabuli_NC_021167.1_14260970	Ca_kabuli_chr08	14260970	[G/T]	1067303751
Ca_Kabuli_NC_021167.1_14320494	Ca_kabuli_chr08	14320494	[T/A]	1067303752
Ca_Kabuli_NC_021167.1_14320512	Ca_kabuli_chr08	14320512	[A/C]	1067303753
Ca_Kabuli_NC_021167.1_14320523	Ca_kabuli_chr08	14320523	[A/G]	1067303754
Ca_Kabuli_NC_021167.1_14324155	Ca_kabuli_chr08	14324155	[A/C]	1067303755
Ca_Kabuli_NC_021167.1_14324185	Ca_kabuli_chr08	14324185	[T/A]	1067303756
Ca_Kabuli_NC_021167.1_14325980	Ca_kabuli_chr08	14325980	[C/A]	1067303757
Ca_Kabuli_NC_021167.1_14347844	Ca_kabuli_chr08	14347844	[A/G]	1067303758
Ca_Kabuli_NC_021167.1_14444375	Ca_kabuli_chr08	14444375	[T/G]	1067303759
Ca_Kabuli_NC_021167.1_14468477	Ca_kabuli_chr08	14468477	[T/C]	1067303760

SNP IDs	Chromosomes	Physical positions (bp)	SNPs	NCBI SNPdb accession IDs (NCBI_ss#)
Ca_Kabuli_NC_021167.1_14528882	Ca_kabuli_chr08	14528882	[T/G]	1067303761
Ca_Kabuli_NC_021167.1_14640805	Ca_kabuli_chr08	14640805	[T/G]	1067303762
Ca_Kabuli_NC_021167.1_14640878	Ca_kabuli_chr08	14640878	[T/G]	1067303763
Ca_Kabuli_NC_021167.1_14640943	Ca_kabuli_chr08	14640943	[T/C]	1067303764
Ca_Kabuli_NC_021167.1_14640967	Ca_kabuli_chr08	14640967	[A/C]	1067303765
Ca_Kabuli_NC_021167.1_14715490	Ca_kabuli_chr08	14715490	[T/G]	1067303766
Ca_Kabuli_NC_021167.1_14715495	Ca_kabuli_chr08	14715495	[T/G]	1067303767
Ca_Kabuli_NC_021167.1_14718763	Ca_kabuli_chr08	14718763	[A/T]	1067303768
Ca_Kabuli_NC_021167.1_14749076	Ca_kabuli_chr08	14749076	[T/G]	1067303769
Ca_Kabuli_NC_021167.1_14753542	Ca_kabuli_chr08	14753542	[T/G]	1067303770
Ca_Kabuli_NC_021167.1_14760860	Ca_kabuli_chr08	14760860	[T/C]	1067303771
Ca_Kabuli_NC_021167.1_14803020	Ca_kabuli_chr08	14803020	[A/C]	1067303772
Ca_Kabuli_NC_021167.1_14803048	Ca_kabuli_chr08	14803048	[T/G]	1067303773
Ca_Kabuli_NC_021167.1_14821416	Ca_kabuli_chr08	14821416	[G/A]	1067303774
Ca_Kabuli_NC_021167.1_14821979	Ca_kabuli_chr08	14821979	[G/A]	1067303775
Ca_Kabuli_NC_021167.1_14822011	Ca_kabuli_chr08	14822011	[C/T]	1067303776
Ca_Kabuli_NC_021167.1_14862045	Ca_kabuli_chr08	14862045	[T/C]	1067303777
Ca_Kabuli_NC_021167.1_14862048	Ca_kabuli_chr08	14862048	[T/C]	1067303778
Ca_Kabuli_NC_021167.1_14862189	Ca_kabuli_chr08	14862189	[T/G]	1067303779
Ca_Kabuli_NC_021167.1_14970630	Ca_kabuli_chr08	14970630	[T/A]	1067303780
Ca_Kabuli_NC_021167.1_14986504	Ca_kabuli_chr08	14986504	[A/G]	1067303781
Ca_Kabuli_NC_021167.1_15131125	Ca_kabuli_chr08	15131125	[T/A]	1067303782
Ca_Kabuli_NC_021167.1_15131129	Ca_kabuli_chr08	15131129	[T/G]	1067303783
Ca_Kabuli_NC_021167.1_15131151	Ca_kabuli_chr08	15131151	[A/C]	1067303784
Ca_Kabuli_NC_021167.1_15131175	Ca_kabuli_chr08	15131175	[G/A]	1067303785
Ca_Kabuli_NC_021167.1_15219937	Ca_kabuli_chr08	15219937	[A/T]	1067303786
Ca_Kabuli_NC_021167.1_15321476	Ca_kabuli_chr08	15321476	[G/A]	1067303787
Ca_Kabuli_NC_021167.1_15375254	Ca_kabuli_chr08	15375254	[A/C]	1067303788
Ca_Kabuli_NC_021167.1_15380024	Ca_kabuli_chr08	15380024	[A/T]	1067303789
Ca_Kabuli_NC_021167.1_15479341	Ca_kabuli_chr08	15479341	[T/G]	1067303790
Ca_Kabuli_NC_021167.1_15482079	Ca_kabuli_chr08	15482079	[G/T]	1067303791
Ca_Kabuli_NC_021167.1_15482534	Ca_kabuli_chr08	15482534	[A/C]	1067303792
Ca_Kabuli_NC_021167.1_15484031	Ca_kabuli_chr08	15484031	[A/T]	1067303793
Ca_Kabuli_NC_021167.1_15484046	Ca_kabuli_chr08	15484046	[T/G]	1067303794

SNP IDs	Chromosomes	Physical positions (bp)	SNPs	NCBI SNPdb accession IDs (NCBI_ss#)
Ca_Kabuli_NC_021167.1_15532056	Ca_kabuli_chr08	15532056	[T/C]	1067303795
Ca_Kabuli_NC_021167.1_15555742	Ca_kabuli_chr08	15555742	[A/G]	1067303796
Ca_Kabuli_NC_021167.1_15590280	Ca_kabuli_chr08	15590280	[A/C]	1067303797
Ca_Kabuli_NC_021167.1_15638443	Ca_kabuli_chr08	15638443	[A/C]	1067303798
Ca_Kabuli_NC_021167.1_15692070	Ca_kabuli_chr08	15692070	[G/A]	1067303799
Ca_Kabuli_NC_021167.1_15695815	Ca_kabuli_chr08	15695815	[A/G]	1067303800
Ca_Kabuli_NC_021167.1_15764837	Ca_kabuli_chr08	15764837	[A/G]	1067303801
Ca_Kabuli_NC_021167.1_15764865	Ca_kabuli_chr08	15764865	[A/G]	1067303802
Ca_Kabuli_NC_021167.1_15764899	Ca_kabuli_chr08	15764899	[T/C]	1067303803
Ca_Kabuli_NC_021167.1_15771041	Ca_kabuli_chr08	15771041	[A/G]	1067303804
Ca_Kabuli_NC_021167.1_15812581	Ca_kabuli_chr08	15812581	[A/G]	1067303805
Ca_Kabuli_NC_021167.1_15812587	Ca_kabuli_chr08	15812587	[T/C]	1067303806
Ca_Kabuli_NC_021167.1_15826107	Ca_kabuli_chr08	15826107	[T/G]	1067303807
Ca_Kabuli_NC_021167.1_15828286	Ca_kabuli_chr08	15828286	[A/T]	1067303808
Ca_Kabuli_NC_021167.1_15828325	Ca_kabuli_chr08	15828325	[T/A]	1067303809
Ca_Kabuli_NC_021167.1_15863470	Ca_kabuli_chr08	15863470	[A/G]	1067303810
Ca_Kabuli_NC_021167.1_15868952	Ca_kabuli_chr08	15868952	[T/C]	1067303811
Ca_Kabuli_NC_021167.1_15873044	Ca_kabuli_chr08	15873044	[A/C]	1067303812
Ca_Kabuli_NC_021167.1_15935288	Ca_kabuli_chr08	15935288	[A/G]	1067303813
Ca_Kabuli_NC_021167.1_15935314	Ca_kabuli_chr08	15935314	[A/C]	1067303814
Ca_Kabuli_NC_021167.1_16022432	Ca_kabuli_chr08	16022432	[C/T]	1067303815
Ca_Kabuli_NC_021167.1_16022511	Ca_kabuli_chr08	16022511	[T/C]	1067303816
Ca_Kabuli_NC_021167.1_16076156	Ca_kabuli_chr08	16076156	[A/C]	1067303817
Ca_Kabuli_NC_021167.1_16076220	Ca_kabuli_chr08	16076220	[T/C]	1067303818
Ca_Kabuli_NC_021167.1_16076223	Ca_kabuli_chr08	16076223	[A/T]	1067303819
Ca_Kabuli_NC_021167.1_16076255	Ca_kabuli_chr08	16076255	[A/C]	1067303820
Ca_Kabuli_NC_021167.1_16102356	Ca_kabuli_chr08	16102356	[G/T]	1067303821
Ca_Kabuli_NC_021167.1_16129739	Ca_kabuli_chr08	16129739	[T/G]	1067303822
Ca_Kabuli_NC_021167.1_16129741	Ca_kabuli_chr08	16129741	[T/A]	1067303823
Ca_Kabuli_NC_021167.1_16129767	Ca_kabuli_chr08	16129767	[A/G]	1067303824
Ca_Kabuli_NC_021167.1_16151176	Ca_kabuli_chr08	16151176	[C/A]	1067303825
Ca_Kabuli_NC_021167.1_16151177	Ca_kabuli_chr08	16151177	[A/T]	1067303826
Ca_Kabuli_NC_021167.1_16151178	Ca_kabuli_chr08	16151178	[A/C]	1067303827
Ca_Kabuli_NC_021167.1_16151179	Ca_kabuli_chr08	16151179	[G/T]	1067303828

SNP IDs	Chromosomes	Physical positions (bp)	SNPs	NCBI SNPdb accession IDs (NCBI_ss#)
Ca_Kabuli_NC_021167.1_16222056	Ca_kabuli_chr08	16222056	[T/A]	1067303829
Ca_Kabuli_NC_021167.1_16264262	Ca_kabuli_chr08	16264262	[T/G]	1067303830
Ca_Kabuli_NC_021167.1_16284238	Ca_kabuli_chr08	16284238	[C/T]	1067303831
Ca_Kabuli_NC_021167.1_16326539	Ca_kabuli_chr08	16326539	[A/C]	1067303832
Ca_Kabuli_NC_021167.1_16333842	Ca_kabuli_chr08	16333842	[T/C]	1067303833
Ca_Kabuli_NC_021167.1_16411504	Ca_kabuli_chr08	16411504	[T/C]	1067303834
Ca_Kabuli_NC_021167.1_16412539	Ca_kabuli_chr08	16412539	[T/C]	1067303835
Ca_Kabuli_NC_021167.1_16413199	Ca_kabuli_chr08	16413199	[T/G]	1067303836
Ca_Kabuli_NC_021167.1_16421377	Ca_kabuli_chr08	16421377	[A/G]	1067303837
Ca_Kabuli_NC_021167.1_16421410	Ca_kabuli_chr08	16421410	[A/T]	1067303838
Ca_Kabuli_NC_021167.1_16421487	Ca_kabuli_chr08	16421487	[T/C]	1067303839
Ca_Kabuli_NC_021167.1_16421534	Ca_kabuli_chr08	16421534	[T/G]	1067303840
Ca_Kabuli_NC_021167.1_16421549	Ca_kabuli_chr08	16421549	[T/C]	1067303841
Ca_Kabuli_NC_021167.1_16421551	Ca_kabuli_chr08	16421551	[A/G]	1067303842
Ca_Kabuli_NC_021167.1_16421613	Ca_kabuli_chr08	16421613	[A/G]	1067303843
Ca_Kabuli_NC_021167.1_16421629	Ca_kabuli_chr08	16421629	[T/C]	1067303844
Ca_Kabuli_NC_021167.1_16432863	Ca_kabuli_chr08	16432863	[A/G]	1067303845
Ca_Kabuli_NC_021167.1_16432866	Ca_kabuli_chr08	16432866	[T/A]	1067303846
Ca_Kabuli_NW_004515636.1_4014	Ca_kabuli_scaffold	4014	[T/C]	1067303847
Ca_Kabuli_NW_004515636.1_4065	Ca_kabuli_scaffold	4065	[T/C]	1067303848
Ca_Kabuli_NW_004515636.1_5063	Ca_kabuli_scaffold	5063	[A/G]	1067303849
Ca_Kabuli_NW_004515636.1_5064	Ca_kabuli_scaffold	5064	[G/A]	1067303850
Ca_Kabuli_NW_004515636.1_5065	Ca_kabuli_scaffold	5065	[C/T]	1067303851
Ca_Kabuli_NW_004515636.1_5143	Ca_kabuli_scaffold	5143	[C/A]	1067303852
Ca_Kabuli_NW_004515636.1_5145	Ca_kabuli_scaffold	5145	[T/C]	1067303853
Ca_Kabuli_NW_004515636.1_5149	Ca_kabuli_scaffold	5149	[C/A]	1067303854
Ca_Kabuli_NW_004515636.1_5550	Ca_kabuli_scaffold	5550	[T/C]	1067303855
Ca_Kabuli_NW_004515636.1_5811	Ca_kabuli_scaffold	5811	[T/C]	1067303856
Ca_Kabuli_NW_004515636.1_6264	Ca_kabuli_scaffold	6264	[G/A]	1067303857
Ca_Kabuli_NW_004515636.1_6265	Ca_kabuli_scaffold	6265	[C/T]	1067303858
Ca_Kabuli_NW_004515636.1_6268	Ca_kabuli_scaffold	6268	[T/C]	1067303859
Ca_Kabuli_NW_004515636.1_6322	Ca_kabuli_scaffold	6322	[A/G]	1067303860
Ca_Kabuli_NW_004515636.1_6325	Ca_kabuli_scaffold	6325	[A/T]	1067303861
Ca_Kabuli_NW_004515636.1_6326	Ca_kabuli_scaffold	6326	[T/G]	1067303862

SNP IDs	Chromosomes	Physical positions (bp)	SNPs	NCBI SNPdb accession IDs (NCBI_ss#)
Ca_Kabuli_NW_004515636.1_19605	Ca_kabuli_scaffold	19605	[C/T]	1067303863
Ca_Kabuli_NW_004515636.1_19615	Ca_kabuli_scaffold	19615	[C/T]	1067303864
Ca_Kabuli_NW_004515636.1_19624	Ca_kabuli_scaffold	19624	[G/A]	1067303865
Ca_Kabuli_NW_004515636.1_19659	Ca_kabuli_scaffold	19659	[G/A]	1067303866
Ca_Kabuli_NW_004515636.1_19662	Ca_kabuli_scaffold	19662	[G/A]	1067303867
Ca_Kabuli_NW_004515636.1_19669	Ca_kabuli_scaffold	19669	[G/A]	1067303868
Ca_Kabuli_NW_004515636.1_19686	Ca_kabuli_scaffold	19686	[A/T]	1067303869
Ca_Kabuli_NW_004515636.1_20193	Ca_kabuli_scaffold	20193	[A/G]	1067303870
Ca_Kabuli_NW_004515636.1_20198	Ca_kabuli_scaffold	20198	[A/G]	1067303871
Ca_Kabuli_NW_004515636.1_20306	Ca_kabuli_scaffold	20306	[T/A]	1067303872
Ca_Kabuli_NW_004515636.1_23439	Ca_kabuli_scaffold	23439	[A/C]	1067303873
Ca_Kabuli_NW_004515636.1_25828	Ca_kabuli_scaffold	25828	[T/C]	1067303874
Ca_Kabuli_NW_004515636.1_25829	Ca_kabuli_scaffold	25829	[G/A]	1067303875
Ca_Kabuli_NW_004515636.1_25830	Ca_kabuli_scaffold	25830	[C/T]	1067303876
Ca_Kabuli_NW_004515636.1_27212	Ca_kabuli_scaffold	27212	[A/G]	1067303877
Ca_Kabuli_NW_004515636.1_27588	Ca_kabuli_scaffold	27588	[A/C]	1067303878
Ca_Kabuli_NW_004515636.1_27603	Ca_kabuli_scaffold	27603	[T/G]	1067303879
Ca_Kabuli_NW_004515636.1_27605	Ca_kabuli_scaffold	27605	[C/A]	1067303880
Ca_Kabuli_NW_004515636.1_27622	Ca_kabuli_scaffold	27622	[A/G]	1067303881
Ca_Kabuli_NW_004515636.1_27768	Ca_kabuli_scaffold	27768	[A/T]	1067303882
Ca_Kabuli_NW_004515636.1_46448	Ca_kabuli_scaffold	46448	[G/A]	1067303883
Ca_Kabuli_NW_004515636.1_46449	Ca_kabuli_scaffold	46449	[C/T]	1067303884
Ca_Kabuli_NW_004515636.1_46450	Ca_kabuli_scaffold	46450	[C/T]	1067303885
Ca_Kabuli_NW_004515636.1_47700	Ca_kabuli_scaffold	47700	[G/A]	1067303886
Ca_Kabuli_NW_004515636.1_47701	Ca_kabuli_scaffold	47701	[C/T]	1067303887
Ca_Kabuli_NW_004515636.1_47706	Ca_kabuli_scaffold	47706	[T/G]	1067303888
Ca_Kabuli_NW_004515636.1_47744	Ca_kabuli_scaffold	47744	[T/G]	1067303889
Ca_Kabuli_NW_004515636.1_47756	Ca_kabuli_scaffold	47756	[T/A]	1067303890
Ca_Kabuli_NW_004515636.1_47760	Ca_kabuli_scaffold	47760	[G/C]	1067303891
Ca_Kabuli_NW_004515636.1_47761	Ca_kabuli_scaffold	47761	[G/T]	1067303892
Ca_Kabuli_NW_004515636.1_47762	Ca_kabuli_scaffold	47762	[A/G]	1067303893
Ca_Kabuli_NW_004515636.1_47783	Ca_kabuli_scaffold	47783	[A/G]	1067303894
Ca_Kabuli_NW_004515636.1_47789	Ca_kabuli_scaffold	47789	[A/G]	1067303895
Ca_Kabuli_NW_004515636.1_50492	Ca_kabuli_scaffold	50492	[T/C]	1067303896

SNP IDs	Chromosomes	Physical positions (bp)	SNPs	NCBI SNPdb accession IDs (NCBI_ss#)
Ca_Kabuli_NW_004515636.1_83152	Ca_kabuli_scaffold	83152	[T/G]	1067303897
Ca_Kabuli_NW_004515636.1_113771	Ca_kabuli_scaffold	113771	[A/G]	1067303898
Ca_Kabuli_NW_004515636.1_113829	Ca_kabuli_scaffold	113829	[G/A]	1067303899
Ca_Kabuli_NW_004515636.1_113830	Ca_kabuli_scaffold	113830	[C/G]	1067303900
Ca_Kabuli_NW_004515636.1_113831	Ca_kabuli_scaffold	113831	[T/G]	1067303901
Ca_Kabuli_NW_004515636.1_117587	Ca_kabuli_scaffold	117587	[A/T]	1067303902
Ca_Kabuli_NW_004515636.1_117594	Ca_kabuli_scaffold	117594	[T/C]	1067303903
Ca_Kabuli_NW_004515636.1_123633	Ca_kabuli_scaffold	123633	[A/T]	1067303904
Ca_Kabuli_NW_004515636.1_123649	Ca_kabuli_scaffold	123649	[T/C]	1067303905
Ca_Kabuli_NW_004515636.1_123700	Ca_kabuli_scaffold	123700	[T/C]	1067303906
Ca_Kabuli_NW_004515636.1_123726	Ca_kabuli_scaffold	123726	[T/C]	1067303907
Ca_Kabuli_NW_004515638.1_137309	Ca_kabuli_scaffold	137309	[T/G]	1067303908
Ca_Kabuli_NW_004515638.1_137415	Ca_kabuli_scaffold	137415	[G/T]	1067303909
Ca_Kabuli_NW_004515638.1_137430	Ca_kabuli_scaffold	137430	[T/G]	1067303910
Ca_Kabuli_NW_004515638.1_345193	Ca_kabuli_scaffold	345193	[A/G]	1067303911
Ca_Kabuli_NW_004515642.1_48870	Ca_kabuli_scaffold	48870	[C/G]	1067303912
Ca_Kabuli_NW_004515642.1_48871	Ca_kabuli_scaffold	48871	[C/A]	1067303913
Ca_Kabuli_NW_004515642.1_48874	Ca_kabuli_scaffold	48874	[G/T]	1067303914
Ca_Kabuli_NW_004515644.1_95	Ca_kabuli_scaffold	95	[G/A]	1067303915
Ca_Kabuli_NW_004515644.1_134	Ca_kabuli_scaffold	134	[T/C]	1067303916
Ca_Kabuli_NW_004515644.1_136	Ca_kabuli_scaffold	136	[A/G]	1067303917
Ca_Kabuli_NW_004515644.1_140	Ca_kabuli_scaffold	140	[C/A]	1067303918
Ca_Kabuli_NW_004515644.1_230	Ca_kabuli_scaffold	230	[C/T]	1067303919
Ca_Kabuli_NW_004515644.1_249	Ca_kabuli_scaffold	249	[T/C]	1067303920
Ca_Kabuli_NW_004515644.1_200563	Ca_kabuli_scaffold	200563	[A/G]	1067303921
Ca_Kabuli_NW_004515644.1_332963	Ca_kabuli_scaffold	332963	[C/T]	1067303922
Ca_Kabuli_NW_004515645.1_94025	Ca_kabuli_scaffold	94025	[T/C]	1067303923
Ca_Kabuli_NW_004515645.1_94054	Ca_kabuli_scaffold	94054	[A/G]	1067303924
Ca_Kabuli_NW_004515645.1_94076	Ca_kabuli_scaffold	94076	[A/T]	1067303925
Ca_Kabuli_NW_004515645.1_94087	Ca_kabuli_scaffold	94087	[T/C]	1067303926
Ca_Kabuli_NW_004515645.1_94089	Ca_kabuli_scaffold	94089	[A/T]	1067303927
Ca_Kabuli_NW_004515646.1_113532	Ca_kabuli_scaffold	113532	[A/G]	1067303928
Ca_Kabuli_NW_004515646.1_113582	Ca_kabuli_scaffold	113582	[G/C]	1067303929
Ca_Kabuli_NW_004515646.1_113584	Ca_kabuli_scaffold	113584	[G/A]	1067303930

SNP IDs	Chromosomes	Physical positions (bp)	SNPs	NCBI SNPdb accession IDs (NCBI_ss#)
Ca_Kabuli_NW_004515651.1_449103	Ca_kabuli_scaffold	449103	[A/G]	1067303931
Ca_Kabuli_NW_004515651.1_495572	Ca_kabuli_scaffold	495572	[G/A]	1067303932
Ca_Kabuli_NW_004515651.1_495579	Ca_kabuli_scaffold	495579	[A/G]	1067303933
Ca_Kabuli_NW_004515651.1_495584	Ca_kabuli_scaffold	495584	[G/A]	1067303934
Ca_Kabuli_NW_004515651.1_495604	Ca_kabuli_scaffold	495604	[A/G]	1067303935
Ca_Kabuli_NW_004515651.1_501445	Ca_kabuli_scaffold	501445	[G/A]	1067303936
Ca_Kabuli_NW_004515653.1_43725	Ca_kabuli_scaffold	43725	[G/A]	1067303937
Ca_Kabuli_NW_004515653.1_43769	Ca_kabuli_scaffold	43769	[T/A]	1067303938
Ca_Kabuli_NW_004515656.1_413691	Ca_kabuli_scaffold	413691	[G/A]	1067303939
Ca_Kabuli_NW_004515656.1_413713	Ca_kabuli_scaffold	413713	[A/C]	1067303940
Ca_Kabuli_NW_004515656.1_413719	Ca_kabuli_scaffold	413719	[G/A]	1067303941
Ca_Kabuli_NW_004515656.1_457416	Ca_kabuli_scaffold	457416	[A/G]	1067303942
Ca_Kabuli_NW_004515656.1_457455	Ca_kabuli_scaffold	457455	[A/G]	1067303943
Ca_Kabuli_NW_004515656.1_480520	Ca_kabuli_scaffold	480520	[A/C]	1067303944
Ca_Kabuli_NW_004515656.1_520311	Ca_kabuli_scaffold	520311	[A/G]	1067303945
Ca_Kabuli_NW_004515656.1_541373	Ca_kabuli_scaffold	541373	[G/T]	1067303946
Ca_Kabuli_NW_004515658.1_378128	Ca_kabuli_scaffold	378128	[A/C]	1067303947
Ca_Kabuli_NW_004515658.1_548610	Ca_kabuli_scaffold	548610	[T/G]	1067303948
Ca_Kabuli_NW_004515658.1_563568	Ca_kabuli_scaffold	563568	[A/C]	1067303949
Ca_Kabuli_NW_004515658.1_871280	Ca_kabuli_scaffold	871280	[A/G]	1067303950
Ca_Kabuli_NW_004515658.1_871346	Ca_kabuli_scaffold	871346	[A/G]	1067303951
Ca_Kabuli_NW_004515658.1_911602	Ca_kabuli_scaffold	911602	[G/T]	1067303952
Ca_Kabuli_NW_004515658.1_961089	Ca_kabuli_scaffold	961089	[A/G]	1067303953
Ca_Kabuli_NW_004515658.1_962807	Ca_kabuli_scaffold	962807	[T/A]	1067303954
Ca_Kabuli_NW_004515658.1_992528	Ca_kabuli_scaffold	992528	[T/C]	1067303955
Ca_Kabuli_NW_004515658.1_992544	Ca_kabuli_scaffold	992544	[A/G]	1067303956
Ca_Kabuli_NW_004515658.1_992549	Ca_kabuli_scaffold	992549	[T/G]	1067303957
Ca_Kabuli_NW_004515658.1_992635	Ca_kabuli_scaffold	992635	[T/G]	1067303958
Ca_Kabuli_NW_004515658.1_997950	Ca_kabuli_scaffold	997950	[A/C]	1067303959
Ca_Kabuli_NW_004515658.1_1002666	Ca_kabuli_scaffold	1002666	[T/G]	1067303960
Ca_Kabuli_NW_004515658.1_1002673	Ca_kabuli_scaffold	1002673	[T/G]	1067303961
Ca_Kabuli_NW_004515658.1_1002679	Ca_kabuli_scaffold	1002679	[G/T]	1067303962
Ca_Kabuli_NW_004515658.1_1002680	Ca_kabuli_scaffold	1002680	[T/G]	1067303963
Ca_Kabuli_NW_004515658.1_1002765	Ca_kabuli_scaffold	1002765	[C/G]	1067303964

SNP IDs	Chromosomes	Physical positions (bp)	SNPs	NCBI SNPdb accession IDs (NCBI_ss#)
Ca_Kabuli_NW_004515658.1_1020708	Ca_kabuli_scaffold	1020708	[A/T]	1067303965
Ca_Kabuli_NW_004515658.1_1039242	Ca_kabuli_scaffold	1039242	[A/C]	1067303966
Ca_Kabuli_NW_004515658.1_1049122	Ca_kabuli_scaffold	1049122	[A/G]	1067303967
Ca_Kabuli_NW_004515658.1_1049135	Ca_kabuli_scaffold	1049135	[C/A]	1067303968
Ca_Kabuli_NW_004515658.1_1049702	Ca_kabuli_scaffold	1049702	[T/C]	1067303969
Ca_Kabuli_NW_004515658.1_1050164	Ca_kabuli_scaffold	1050164	[T/C]	1067303970
Ca_Kabuli_NW_004515658.1_1052918	Ca_kabuli_scaffold	1052918	[T/G]	1067303971
Ca_Kabuli_NW_004515658.1_1052919	Ca_kabuli_scaffold	1052919	[C/G]	1067303972
Ca_Kabuli_NW_004515658.1_1052920	Ca_kabuli_scaffold	1052920	[T/C]	1067303973
Ca_Kabuli_NW_004515658.1_1052921	Ca_kabuli_scaffold	1052921	[T/C]	1067303974
Ca_Kabuli_NW_004515658.1_1052922	Ca_kabuli_scaffold	1052922	[T/A]	1067303975
Ca_Kabuli_NW_004515658.1_1052923	Ca_kabuli_scaffold	1052923	[A/T]	1067303976
Ca_Kabuli_NW_004515658.1_1052983	Ca_kabuli_scaffold	1052983	[A/G]	1067303977
Ca_Kabuli_NW_004515658.1_1053185	Ca_kabuli_scaffold	1053185	[T/G]	1067303978
Ca_Kabuli_NW_004515658.1_1053215	Ca_kabuli_scaffold	1053215	[A/G]	1067303979
Ca_Kabuli_NW_004515658.1_1053247	Ca_kabuli_scaffold	1053247	[A/G]	1067303980
Ca_Kabuli_NW_004515658.1_1053261	Ca_kabuli_scaffold	1053261	[C/T]	1067303981
Ca_Kabuli_NW_004515659.1_137462	Ca_kabuli_scaffold	137462	[G/A]	1067303982
Ca_Kabuli_NW_004515662.1_39438	Ca_kabuli_scaffold	39438	[C/A]	1067303983
Ca_Kabuli_NW_004515662.1_39470	Ca_kabuli_scaffold	39470	[C/T]	1067303984
Ca_Kabuli_NW_004515663.1_74704	Ca_kabuli_scaffold	74704	[T/C]	1067303985
Ca_Kabuli_NW_004515663.1_74707	Ca_kabuli_scaffold	74707	[T/C]	1067303986
Ca_Kabuli_NW_004515663.1_74710	Ca_kabuli_scaffold	74710	[T/A]	1067303987
Ca_Kabuli_NW_004515663.1_74714	Ca_kabuli_scaffold	74714	[C/A]	1067303988
Ca_Kabuli_NW_004515663.1_74722	Ca_kabuli_scaffold	74722	[T/C]	1067303989
Ca_Kabuli_NW_004515663.1_74743	Ca_kabuli_scaffold	74743	[G/A]	1067303990
Ca_Kabuli_NW_004515663.1_74749	Ca_kabuli_scaffold	74749	[G/A]	1067303991
Ca_Kabuli_NW_004515663.1_224738	Ca_kabuli_scaffold	224738	[T/C]	1067303992
Ca_Kabuli_NW_004515665.1_78661	Ca_kabuli_scaffold	78661	[T/C]	1067303993
Ca_Kabuli_NW_004515665.1_151898	Ca_kabuli_scaffold	151898	[A/C]	1067303994
Ca_Kabuli_NW_004515665.1_191558	Ca_kabuli_scaffold	191558	[T/G]	1067303995
Ca_Kabuli_NW_004515665.1_192020	Ca_kabuli_scaffold	192020	[T/G]	1067303996
Ca_Kabuli_NW_004515665.1_192023	Ca_kabuli_scaffold	192023	[A/G]	1067303997
Ca_Kabuli_NW_004515665.1_192061	Ca_kabuli_scaffold	192061	[A/T]	1067303998

SNP IDs	Chromosomes	Physical positions (bp)	SNPs	NCBI SNPdb accession IDs (NCBI_ss#)
Ca_Kabuli_NW_004515665.1_192163	Ca_kabuli_scaffold	192163	[T/G]	1067303999
Ca_Kabuli_NW_004515665.1_195831	Ca_kabuli_scaffold	195831	[T/C]	1067304000
Ca_Kabuli_NW_004515665.1_195897	Ca_kabuli_scaffold	195897	[A/C]	1067304001
Ca_Kabuli_NW_004515665.1_196116	Ca_kabuli_scaffold	196116	[T/C]	1067304002
Ca_Kabuli_NW_004515665.1_448978	Ca_kabuli_scaffold	448978	[C/A]	1067304003
Ca_Kabuli_NW_004515665.1_448980	Ca_kabuli_scaffold	448980	[T/C]	1067304004
Ca_Kabuli_NW_004515665.1_449023	Ca_kabuli_scaffold	449023	[A/C]	1067304005
Ca_Kabuli_NW_004515665.1_449041	Ca_kabuli_scaffold	449041	[A/G]	1067304006
Ca_Kabuli_NW_004515666.1_35900	Ca_kabuli_scaffold	35900	[T/C]	1067304007
Ca_Kabuli_NW_004515666.1_47641	Ca_kabuli_scaffold	47641	[A/T]	1067304008
Ca_Kabuli_NW_004515666.1_47663	Ca_kabuli_scaffold	47663	[A/T]	1067304009
Ca_Kabuli_NW_004515666.1_47687	Ca_kabuli_scaffold	47687	[T/C]	1067304010
Ca_Kabuli_NW_004515666.1_47696	Ca_kabuli_scaffold	47696	[T/A]	1067304011
Ca_Kabuli_NW_004515666.1_49282	Ca_kabuli_scaffold	49282	[T/G]	1067304012
Ca_Kabuli_NW_004515666.1_49429	Ca_kabuli_scaffold	49429	[T/A]	1067304013
Ca_Kabuli_NW_004515666.1_125185	Ca_kabuli_scaffold	125185	[T/G]	1067304014
Ca_Kabuli_NW_004515673.1_34108	Ca_kabuli_scaffold	34108	[C/T]	1067304015
Ca_Kabuli_NW_004515673.1_34111	Ca_kabuli_scaffold	34111	[C/A]	1067304016
Ca_Kabuli_NW_004515677.1_376643	Ca_kabuli_scaffold	376643	[C/A]	1067304017
Ca_Kabuli_NW_004515677.1_420436	Ca_kabuli_scaffold	420436	[T/C]	1067304018
Ca_Kabuli_NW_004515677.1_420439	Ca_kabuli_scaffold	420439	[T/G]	1067304019
Ca_Kabuli_NW_004515677.1_420443	Ca_kabuli_scaffold	420443	[T/G]	1067304020
Ca_Kabuli_NW_004515677.1_420447	Ca_kabuli_scaffold	420447	[A/G]	1067304021
Ca_Kabuli_NW_004515677.1_420451	Ca_kabuli_scaffold	420451	[A/G]	1067304022
Ca_Kabuli_NW_004515677.1_420473	Ca_kabuli_scaffold	420473	[T/G]	1067304023
Ca_Kabuli_NW_004515677.1_420480	Ca_kabuli_scaffold	420480	[T/C]	1067304024
Ca_Kabuli_NW_004515677.1_420499	Ca_kabuli_scaffold	420499	[A/C]	1067304025
Ca_Kabuli_NW_004515677.1_433404	Ca_kabuli_scaffold	433404	[T/C]	1067304026
Ca_Kabuli_NW_004515677.1_433419	Ca_kabuli_scaffold	433419	[T/C]	1067304027
Ca_Kabuli_NW_004515677.1_433441	Ca_kabuli_scaffold	433441	[T/C]	1067304028
Ca_Kabuli_NW_004515677.1_433467	Ca_kabuli_scaffold	433467	[A/G]	1067304029
Ca_Kabuli_NW_004515677.1_437526	Ca_kabuli_scaffold	437526	[A/G]	1067304030
Ca_Kabuli_NW_004515677.1_437554	Ca_kabuli_scaffold	437554	[A/G]	1067304031
Ca_Kabuli_NW_004515677.1_437970	Ca_kabuli_scaffold	437970	[A/T]	1067304032

SNP IDs	Chromosomes	Physical positions (bp)	SNPs	NCBI SNPdb accession IDs (NCBI_ss#)
Ca_Kabuli_NW_004515677.1_437974	Ca_kabuli_scaffold	437974	[T/C]	1067304033
Ca_Kabuli_NW_004515677.1_437979	Ca_kabuli_scaffold	437979	[A/C]	1067304034
Ca_Kabuli_NW_004515677.1_438016	Ca_kabuli_scaffold	438016	[T/C]	1067304035
Ca_Kabuli_NW_004515677.1_438130	Ca_kabuli_scaffold	438130	[A/G]	1067304036
Ca_Kabuli_NW_004515678.1_73626	Ca_kabuli_scaffold	73626	[A/G]	1067304037
Ca_Kabuli_NW_004515678.1_77955	Ca_kabuli_scaffold	77955	[A/G]	1067304038
Ca_Kabuli_NW_004515683.1_271596	Ca_kabuli_scaffold	271596	[A/T]	1067304039
Ca_Kabuli_NW_004515683.1_271597	Ca_kabuli_scaffold	271597	[G/C]	1067304040
Ca_Kabuli_NW_004515683.1_271598	Ca_kabuli_scaffold	271598	[G/T]	1067304041
Ca_Kabuli_NW_004515684.1_4256	Ca_kabuli_scaffold	4256	[C/G]	1067304042
Ca_Kabuli_NW_004515684.1_4268	Ca_kabuli_scaffold	4268	[T/C]	1067304043
Ca_Kabuli_NW_004515684.1_4273	Ca_kabuli_scaffold	4273	[C/G]	1067304044
Ca_Kabuli_NW_004515684.1_4299	Ca_kabuli_scaffold	4299	[A/G]	1067304045
Ca_Kabuli_NW_004515684.1_16344	Ca_kabuli_scaffold	16344	[A/G]	1067304046
Ca_Kabuli_NW_004515684.1_16354	Ca_kabuli_scaffold	16354	[T/C]	1067304047
Ca_Kabuli_NW_004515684.1_16380	Ca_kabuli_scaffold	16380	[T/G]	1067304048
Ca_Kabuli_NW_004515684.1_16401	Ca_kabuli_scaffold	16401	[T/G]	1067304049
Ca_Kabuli_NW_004515684.1_16414	Ca_kabuli_scaffold	16414	[A/G]	1067304050
Ca_Kabuli_NW_004515684.1_29934	Ca_kabuli_scaffold	29934	[A/T]	1067304051
Ca_Kabuli_NW_004515684.1_110948	Ca_kabuli_scaffold	110948	[T/C]	1067304052
Ca_Kabuli_NW_004515684.1_110960	Ca_kabuli_scaffold	110960	[A/G]	1067304053
Ca_Kabuli_NW_004515684.1_110972	Ca_kabuli_scaffold	110972	[A/G]	1067304054
Ca_Kabuli_NW_004515684.1_174790	Ca_kabuli_scaffold	174790	[C/A]	1067304055
Ca_Kabuli_NW_004515684.1_174791	Ca_kabuli_scaffold	174791	[T/G]	1067304056
Ca_Kabuli_NW_004515684.1_174792	Ca_kabuli_scaffold	174792	[C/A]	1067304057
Ca_Kabuli_NW_004515684.1_253574	Ca_kabuli_scaffold	253574	[T/C]	1067304058
Ca_Kabuli_NW_004515684.1_253596	Ca_kabuli_scaffold	253596	[G/A]	1067304059
Ca_Kabuli_NW_004515684.1_257023	Ca_kabuli_scaffold	257023	[A/G]	1067304060
Ca_Kabuli_NW_004515684.1_257344	Ca_kabuli_scaffold	257344	[C/T]	1067304061
Ca_Kabuli_NW_004515684.1_257376	Ca_kabuli_scaffold	257376	[T/A]	1067304062
Ca_Kabuli_NW_004515684.1_258573	Ca_kabuli_scaffold	258573	[A/G]	1067304063
Ca_Kabuli_NW_004515685.1_128324	Ca_kabuli_scaffold	128324	[C/A]	1067304064
Ca_Kabuli_NW_004515685.1_128328	Ca_kabuli_scaffold	128328	[A/C]	1067304065
Ca_Kabuli_NW_004515687.1_578164	Ca_kabuli_scaffold	578164	[T/G]	1067304066

SNP IDs	Chromosomes	Physical positions (bp)	SNPs	NCBI SNPdb accession IDs (NCBI_ss#)
Ca_Kabuli_NW_004515687.1_647366	Ca_kabuli_scaffold	647366	[T/C]	1067304067
Ca_Kabuli_NW_004515688.1_27925	Ca_kabuli_scaffold	27925	[T/G]	1067304068
Ca_Kabuli_NW_004515688.1_27928	Ca_kabuli_scaffold	27928	[T/G]	1067304069
Ca_Kabuli_NW_004515688.1_28967	Ca_kabuli_scaffold	28967	[A/T]	1067304070
Ca_Kabuli_NW_004515688.1_28979	Ca_kabuli_scaffold	28979	[C/T]	1067304071
Ca_Kabuli_NW_004515688.1_294455	Ca_kabuli_scaffold	294455	[T/G]	1067304072
Ca_Kabuli_NW_004515688.1_294487	Ca_kabuli_scaffold	294487	[T/G]	1067304073
Ca_Kabuli_NW_004515688.1_294493	Ca_kabuli_scaffold	294493	[A/T]	1067304074
Ca_Kabuli_NW_004515688.1_294505	Ca_kabuli_scaffold	294505	[A/G]	1067304075
Ca_Kabuli_NW_004515689.1_287933	Ca_kabuli_scaffold	287933	[T/C]	1067304076
Ca_Kabuli_NW_004515689.1_399800	Ca_kabuli_scaffold	399800	[T/G]	1067304077
Ca_Kabuli_NW_004515689.1_406183	Ca_kabuli_scaffold	406183	[A/T]	1067304078
Ca_Kabuli_NW_004515689.1_562990	Ca_kabuli_scaffold	562990	[T/G]	1067304079
Ca_Kabuli_NW_004515689.1_563004	Ca_kabuli_scaffold	563004	[T/A]	1067304080
Ca_Kabuli_NW_004515689.1_568164	Ca_kabuli_scaffold	568164	[T/C]	1067304081
Ca_Kabuli_NW_004515689.1_568237	Ca_kabuli_scaffold	568237	[T/C]	1067304082
Ca_Kabuli_NW_004515689.1_606105	Ca_kabuli_scaffold	606105	[A/C]	1067304083
Ca_Kabuli_NW_004515695.1_226164	Ca_kabuli_scaffold	226164	[A/C]	1067304084
Ca_Kabuli_NW_004515695.1_226165	Ca_kabuli_scaffold	226165	[G/T]	1067304085
Ca_Kabuli_NW_004515695.1_226166	Ca_kabuli_scaffold	226166	[A/T]	1067304086
Ca_Kabuli_NW_004515695.1_226167	Ca_kabuli_scaffold	226167	[T/A]	1067304087
Ca_Kabuli_NW_004515695.1_303176	Ca_kabuli_scaffold	303176	[T/G]	1067304088
Ca_Kabuli_NW_004515695.1_303187	Ca_kabuli_scaffold	303187	[T/G]	1067304089
Ca_Kabuli_NW_004515695.1_305323	Ca_kabuli_scaffold	305323	[A/C]	1067304090
Ca_Kabuli_NW_004515695.1_337216	Ca_kabuli_scaffold	337216	[T/G]	1067304091
Ca_Kabuli_NW_004515695.1_441589	Ca_kabuli_scaffold	441589	[T/G]	1067304092
Ca_Kabuli_NW_004515695.1_450197	Ca_kabuli_scaffold	450197	[T/G]	1067304093
Ca_Kabuli_NW_004515695.1_586229	Ca_kabuli_scaffold	586229	[T/G]	1067304094
Ca_Kabuli_NW_004515695.1_586256	Ca_kabuli_scaffold	586256	[T/C]	1067304095
Ca_Kabuli_NW_004515695.1_586257	Ca_kabuli_scaffold	586257	[G/T]	1067304096
Ca_Kabuli_NW_004515695.1_588208	Ca_kabuli_scaffold	588208	[A/C]	1067304097
Ca_Kabuli_NW_004515695.1_588211	Ca_kabuli_scaffold	588211	[T/C]	1067304098
Ca_Kabuli_NW_004515695.1_696869	Ca_kabuli_scaffold	696869	[A/C]	1067304099
Ca_Kabuli_NW_004515695.1_705958	Ca_kabuli_scaffold	705958	[A/G]	1067304100

SNP IDs	Chromosomes	Physical positions (bp)	SNPs	NCBI SNPdb accession IDs (NCBI_ss#)
Ca_Kabuli_NW_004515695.1_707956	Ca_kabuli_scaffold	707956	[A/C]	1067304101
Ca_Kabuli_NW_004515695.1_779324	Ca_kabuli_scaffold	779324	[G/A]	1067304102
Ca_Kabuli_NW_004515701.1_113829	Ca_kabuli_scaffold	113829	[C/T]	1067304103
Ca_Kabuli_NW_004515701.1_299931	Ca_kabuli_scaffold	299931	[T/G]	1067304104
Ca_Kabuli_NW_004515701.1_299939	Ca_kabuli_scaffold	299939	[A/G]	1067304105
Ca_Kabuli_NW_004515701.1_303730	Ca_kabuli_scaffold	303730	[A/G]	1067304106
Ca_Kabuli_NW_004515701.1_303743	Ca_kabuli_scaffold	303743	[T/C]	1067304107
Ca_Kabuli_NW_004515701.1_303772	Ca_kabuli_scaffold	303772	[A/G]	1067304108
Ca_Kabuli_NW_004515701.1_303776	Ca_kabuli_scaffold	303776	[T/C]	1067304109
Ca_Kabuli_NW_004515701.1_303782	Ca_kabuli_scaffold	303782	[T/C]	1067304110
Ca_Kabuli_NW_004515701.1_303787	Ca_kabuli_scaffold	303787	[T/A]	1067304111
Ca_Kabuli_NW_004515701.1_307253	Ca_kabuli_scaffold	307253	[A/T]	1067304112
Ca_Kabuli_NW_004515701.1_332667	Ca_kabuli_scaffold	332667	[T/G]	1067304113
Ca_Kabuli_NW_004515701.1_332673	Ca_kabuli_scaffold	332673	[C/A]	1067304114
Ca_Kabuli_NW_004515701.1_349187	Ca_kabuli_scaffold	349187	[A/C]	1067304115
Ca_Kabuli_NW_004515701.1_349196	Ca_kabuli_scaffold	349196	[A/T]	1067304116
Ca_Kabuli_NW_004515704.1_314312	Ca_kabuli_scaffold	314312	[A/G]	1067304117
Ca_Kabuli_NW_004515704.1_314314	Ca_kabuli_scaffold	314314	[A/C]	1067304118
Ca_Kabuli_NW_004515708.1_266134	Ca_kabuli_scaffold	266134	[T/C]	1067304119
Ca_Kabuli_NW_004515708.1_317302	Ca_kabuli_scaffold	317302	[T/C]	1067304120
Ca_Kabuli_NW_004515708.1_317339	Ca_kabuli_scaffold	317339	[T/G]	1067304121
Ca_Kabuli_NW_004515708.1_320914	Ca_kabuli_scaffold	320914	[A/C]	1067304122
Ca_Kabuli_NW_004515708.1_461668	Ca_kabuli_scaffold	461668	[A/T]	1067304123
Ca_Kabuli_NW_004515708.1_860788	Ca_kabuli_scaffold	860788	[G/C]	1067304124
Ca_Kabuli_NW_004515712.1_218244	Ca_kabuli_scaffold	218244	[T/G]	1067304125
Ca_Kabuli_NW_004515712.1_218321	Ca_kabuli_scaffold	218321	[T/G]	1067304126
Ca_Kabuli_NW_004515712.1_218336	Ca_kabuli_scaffold	218336	[T/G]	1067304127
Ca_Kabuli_NW_004515712.1_218352	Ca_kabuli_scaffold	218352	[T/G]	1067304128
Ca_Kabuli_NW_004515712.1_218359	Ca_kabuli_scaffold	218359	[T/G]	1067304129
Ca_Kabuli_NW_004515712.1_223513	Ca_kabuli_scaffold	223513	[A/C]	1067304130
Ca_Kabuli_NW_004515712.1_233039	Ca_kabuli_scaffold	233039	[A/C]	1067304131
Ca_Kabuli_NW_004515712.1_369512	Ca_kabuli_scaffold	369512	[T/C]	1067304132
Ca_Kabuli_NW_004515712.1_409583	Ca_kabuli_scaffold	409583	[A/T]	1067304133
Ca_Kabuli_NW_004515717.1_14442	Ca_kabuli_scaffold	14442	[T/C]	1067304134

SNP IDs	Chromosomes	Physical positions (bp)	SNPs	NCBI SNPdb accession IDs (NCBI_ss#)
Ca_Kabuli_NW_004515717.1_14555	Ca_kabuli_scaffold	14555	[G/A]	1067304135
Ca_Kabuli_NW_004515717.1_14618	Ca_kabuli_scaffold	14618	[G/A]	1067304136
Ca_Kabuli_NW_004515719.1_33482	Ca_kabuli_scaffold	33482	[T/G]	1067304137
Ca_Kabuli_NW_004515719.1_33504	Ca_kabuli_scaffold	33504	[T/G]	1067304138
Ca_Kabuli_NW_004515723.1_193172	Ca_kabuli_scaffold	193172	[G/C]	1067304139
Ca_Kabuli_NW_004515723.1_193174	Ca_kabuli_scaffold	193174	[G/A]	1067304140
Ca_Kabuli_NW_004515723.1_193175	Ca_kabuli_scaffold	193175	[T/G]	1067304141
Ca_Kabuli_NW_004515723.1_229653	Ca_kabuli_scaffold	229653	[A/C]	1067304142
Ca_Kabuli_NW_004515723.1_245866	Ca_kabuli_scaffold	245866	[C/A]	1067304143
Ca_Kabuli_NW_004515723.1_267060	Ca_kabuli_scaffold	267060	[T/G]	1067304144
Ca_Kabuli_NW_004515723.1_267064	Ca_kabuli_scaffold	267064	[T/G]	1067304145
Ca_Kabuli_NW_004515723.1_267092	Ca_kabuli_scaffold	267092	[T/G]	1067304146
Ca_Kabuli_NW_004515723.1_275838	Ca_kabuli_scaffold	275838	[A/T]	1067304147
Ca_Kabuli_NW_004515726.1_100149	Ca_kabuli_scaffold	100149	[C/T]	1067304148
Ca_Kabuli_NW_004515726.1_100228	Ca_kabuli_scaffold	100228	[A/G]	1067304149
Ca_Kabuli_NW_004515726.1_100276	Ca_kabuli_scaffold	100276	[A/C]	1067304150
Ca_Kabuli_NW_004515726.1_100529	Ca_kabuli_scaffold	100529	[T/C]	1067304151
Ca_Kabuli_NW_004515726.1_103027	Ca_kabuli_scaffold	103027	[C/T]	1067304152
Ca_Kabuli_NW_004515726.1_103741	Ca_kabuli_scaffold	103741	[G/C]	1067304153
Ca_Kabuli_NW_004515726.1_106331	Ca_kabuli_scaffold	106331	[G/A]	1067304154
Ca_Kabuli_NW_004515726.1_106349	Ca_kabuli_scaffold	106349	[C/T]	1067304155
Ca_Kabuli_NW_004515726.1_121260	Ca_kabuli_scaffold	121260	[G/T]	1067304156
Ca_Kabuli_NW_004515726.1_121293	Ca_kabuli_scaffold	121293	[T/C]	1067304157
Ca_Kabuli_NW_004515726.1_133596	Ca_kabuli_scaffold	133596	[A/C]	1067304158
Ca_Kabuli_NW_004515726.1_142843	Ca_kabuli_scaffold	142843	[C/T]	1067304159
Ca_Kabuli_NW_004515726.1_151081	Ca_kabuli_scaffold	151081	[G/A]	1067304160
Ca_Kabuli_NW_004515726.1_151197	Ca_kabuli_scaffold	151197	[T/G]	1067304161
Ca_Kabuli_NW_004515726.1_151747	Ca_kabuli_scaffold	151747	[A/G]	1067304162
Ca_Kabuli_NW_004515726.1_151777	Ca_kabuli_scaffold	151777	[T/C]	1067304163
Ca_Kabuli_NW_004515726.1_213404	Ca_kabuli_scaffold	213404	[G/T]	1067304164
Ca_Kabuli_NW_004515726.1_213518	Ca_kabuli_scaffold	213518	[C/T]	1067304165
Ca_Kabuli_NW_004515726.1_214188	Ca_kabuli_scaffold	214188	[A/T]	1067304166
Ca_Kabuli_NW_004515726.1_237825	Ca_kabuli_scaffold	237825	[G/A]	1067304167
Ca_Kabuli_NW_004515726.1_352747	Ca_kabuli_scaffold	352747	[T/C]	1067304168

SNP IDs	Chromosomes	Physical positions (bp)	SNPs	NCBI SNPdb accession IDs (NCBI_ss#)
Ca_Kabuli_NW_004515726.1_355222	Ca_kabuli_scaffold	355222	[G/A]	1067304169
Ca_Kabuli_NW_004515726.1_469137	Ca_kabuli_scaffold	469137	[A/G]	1067304170
Ca_Kabuli_NW_004515726.1_469173	Ca_kabuli_scaffold	469173	[T/G]	1067304171
Ca_Kabuli_NW_004515726.1_469176	Ca_kabuli_scaffold	469176	[T/G]	1067304172
Ca_Kabuli_NW_004515730.1_239649	Ca_kabuli_scaffold	239649	[T/A]	1067304173
Ca_Kabuli_NW_004515732.1_115111	Ca_kabuli_scaffold	115111	[A/C]	1067304174
Ca_Kabuli_NW_004515732.1_115114	Ca_kabuli_scaffold	115114	[A/C]	1067304175
Ca_Kabuli_NW_004515732.1_115133	Ca_kabuli_scaffold	115133	[A/G]	1067304176
Ca_Kabuli_NW_004515732.1_115151	Ca_kabuli_scaffold	115151	[A/G]	1067304177
Ca_Kabuli_NW_004515732.1_155666	Ca_kabuli_scaffold	155666	[A/G]	1067304178
Ca_Kabuli_NW_004515732.1_160974	Ca_kabuli_scaffold	160974	[T/C]	1067304179
Ca_Kabuli_NW_004515732.1_160980	Ca_kabuli_scaffold	160980	[T/C]	1067304180
Ca_Kabuli_NW_004515732.1_186119	Ca_kabuli_scaffold	186119	[T/C]	1067304181
Ca_Kabuli_NW_004515732.1_186149	Ca_kabuli_scaffold	186149	[A/C]	1067304182
Ca_Kabuli_NW_004515732.1_186155	Ca_kabuli_scaffold	186155	[A/G]	1067304183
Ca_Kabuli_NW_004515732.1_186158	Ca_kabuli_scaffold	186158	[A/G]	1067304184
Ca_Kabuli_NW_004515732.1_207939	Ca_kabuli_scaffold	207939	[T/A]	1067304185
Ca_Kabuli_NW_004515734.1_17756	Ca_kabuli_scaffold	17756	[T/A]	1067304186
Ca_Kabuli_NW_004515734.1_17761	Ca_kabuli_scaffold	17761	[A/C]	1067304187
Ca_Kabuli_NW_004515734.1_243844	Ca_kabuli_scaffold	243844	[T/C]	1067304188
Ca_Kabuli_NW_004515734.1_263899	Ca_kabuli_scaffold	263899	[G/A]	1067304189
Ca_Kabuli_NW_004515734.1_457634	Ca_kabuli_scaffold	457634	[T/A]	1067304190
Ca_Kabuli_NW_004515734.1_457813	Ca_kabuli_scaffold	457813	[T/G]	1067304191
Ca_Kabuli_NW_004515734.1_458099	Ca_kabuli_scaffold	458099	[A/C]	1067304192
Ca_Kabuli_NW_004515739.1_11965	Ca_kabuli_scaffold	11965	[A/C]	1067304193
Ca_Kabuli_NW_004515739.1_168489	Ca_kabuli_scaffold	168489	[T/A]	1067304194
Ca_Kabuli_NW_004515739.1_168517	Ca_kabuli_scaffold	168517	[T/C]	1067304195
Ca_Kabuli_NW_004515739.1_173329	Ca_kabuli_scaffold	173329	[T/C]	1067304196
Ca_Kabuli_NW_004515739.1_173375	Ca_kabuli_scaffold	173375	[T/A]	1067304197
Ca_Kabuli_NW_004515739.1_280023	Ca_kabuli_scaffold	280023	[T/C]	1067304198
Ca_Kabuli_NW_004515739.1_280035	Ca_kabuli_scaffold	280035	[A/C]	1067304199
Ca_Kabuli_NW_004515739.1_280047	Ca_kabuli_scaffold	280047	[G/A]	1067304200
Ca_Kabuli_NW_004515739.1_280062	Ca_kabuli_scaffold	280062	[C/T]	1067304201
Ca_Kabuli_NW_004515741.1_48869	Ca_kabuli_scaffold	48869	[T/G]	1067304202

SNP IDs	Chromosomes	Physical positions (bp)	SNPs	NCBI SNPdb accession IDs (NCBI_ss#)
Ca_Kabuli_NW_004515741.1_132016	Ca_kabuli_scaffold	132016	[A/G]	1067304203
Ca_Kabuli_NW_004515741.1_244722	Ca_kabuli_scaffold	244722	[T/G]	1067304204
Ca_Kabuli_NW_004515741.1_244732	Ca_kabuli_scaffold	244732	[G/A]	1067304205
Ca_Kabuli_NW_004515741.1_244735	Ca_kabuli_scaffold	244735	[T/G]	1067304206
Ca_Kabuli_NW_004515745.1_145511	Ca_kabuli_scaffold	145511	[T/C]	1067304207
Ca_Kabuli_NW_004515745.1_145544	Ca_kabuli_scaffold	145544	[A/G]	1067304208
Ca_Kabuli_NW_004515745.1_145577	Ca_kabuli_scaffold	145577	[A/G]	1067304209
Ca_Kabuli_NW_004515745.1_145591	Ca_kabuli_scaffold	145591	[T/C]	1067304210
Ca_Kabuli_NW_004515745.1_145596	Ca_kabuli_scaffold	145596	[T/C]	1067304211
Ca_Kabuli_NW_004515745.1_166626	Ca_kabuli_scaffold	166626	[T/C]	1067304212
Ca_Kabuli_NW_004515745.1_166630	Ca_kabuli_scaffold	166630	[T/A]	1067304213
Ca_Kabuli_NW_004515745.1_166678	Ca_kabuli_scaffold	166678	[A/G]	1067304214
Ca_Kabuli_NW_004515745.1_424522	Ca_kabuli_scaffold	424522	[T/C]	1067304215
Ca_Kabuli_NW_004515745.1_546706	Ca_kabuli_scaffold	546706	[T/C]	1067304216
Ca_Kabuli_NW_004515745.1_546722	Ca_kabuli_scaffold	546722	[T/C]	1067304217
Ca_Kabuli_NW_004515745.1_586052	Ca_kabuli_scaffold	586052	[A/G]	1067304218
Ca_Kabuli_NW_004515747.1_108985	Ca_kabuli_scaffold	108985	[G/T]	1067304219
Ca_Kabuli_NW_004515748.1_96363	Ca_kabuli_scaffold	96363	[T/G]	1067304220
Ca_Kabuli_NW_004515748.1_96394	Ca_kabuli_scaffold	96394	[T/A]	1067304221
Ca_Kabuli_NW_004515748.1_96397	Ca_kabuli_scaffold	96397	[G/A]	1067304222
Ca_Kabuli_NW_004515748.1_96420	Ca_kabuli_scaffold	96420	[C/A]	1067304223
Ca_Kabuli_NW_004515748.1_96435	Ca_kabuli_scaffold	96435	[A/G]	1067304224
Ca_Kabuli_NW_004515748.1_96444	Ca_kabuli_scaffold	96444	[A/G]	1067304225
Ca_Kabuli_NW_004515748.1_96456	Ca_kabuli_scaffold	96456	[C/T]	1067304226
Ca_Kabuli_NW_004515748.1_96464	Ca_kabuli_scaffold	96464	[T/G]	1067304227
Ca_Kabuli_NW_004515748.1_96474	Ca_kabuli_scaffold	96474	[T/G]	1067304228
Ca_Kabuli_NW_004515748.1_96532	Ca_kabuli_scaffold	96532	[G/T]	1067304229
Ca_Kabuli_NW_004515748.1_96580	Ca_kabuli_scaffold	96580	[G/T]	1067304230
Ca_Kabuli_NW_004515748.1_96589	Ca_kabuli_scaffold	96589	[A/T]	1067304231
Ca_Kabuli_NW_004515748.1_98426	Ca_kabuli_scaffold	98426	[C/G]	1067304232
Ca_Kabuli_NW_004515748.1_99086	Ca_kabuli_scaffold	99086	[A/C]	1067304233
Ca_Kabuli_NW_004515748.1_99116	Ca_kabuli_scaffold	99116	[A/C]	1067304234
Ca_Kabuli_NW_004515748.1_99121	Ca_kabuli_scaffold	99121	[T/C]	1067304235
Ca_Kabuli_NW_004515748.1_99157	Ca_kabuli_scaffold	99157	[T/C]	1067304236

SNP IDs	Chromosomes	Physical positions (bp)	SNPs	NCBI SNPdb accession IDs (NCBI_ss#)
Ca_Kabuli_NW_004515748.1_99161	Ca_kabuli_scaffold	99161	[T/C]	1067304237
Ca_Kabuli_NW_004515750.1_143426	Ca_kabuli_scaffold	143426	[C/T]	1067304238
Ca_Kabuli_NW_004515750.1_143434	Ca_kabuli_scaffold	143434	[A/T]	1067304239
Ca_Kabuli_NW_004515753.1_65276	Ca_kabuli_scaffold	65276	[A/C]	1067304240
Ca_Kabuli_NW_004515755.1_550	Ca_kabuli_scaffold	550	[C/T]	1067304241
Ca_Kabuli_NW_004515755.1_553	Ca_kabuli_scaffold	553	[T/A]	1067304242
Ca_Kabuli_NW_004515755.1_578	Ca_kabuli_scaffold	578	[C/A]	1067304243
Ca_Kabuli_NW_004515755.1_355232	Ca_kabuli_scaffold	355232	[A/G]	1067304244
Ca_Kabuli_NW_004515755.1_355282	Ca_kabuli_scaffold	355282	[T/C]	1067304245
Ca_Kabuli_NW_004515755.1_355750	Ca_kabuli_scaffold	355750	[C/G]	1067304246
Ca_Kabuli_NW_004515755.1_355771	Ca_kabuli_scaffold	355771	[A/T]	1067304247
Ca_Kabuli_NW_004515755.1_355791	Ca_kabuli_scaffold	355791	[A/T]	1067304248
Ca_Kabuli_NW_004515755.1_517803	Ca_kabuli_scaffold	517803	[A/C]	1067304249
Ca_Kabuli_NW_004515755.1_517853	Ca_kabuli_scaffold	517853	[A/T]	1067304250
Ca_Kabuli_NW_004515762.1_52213	Ca_kabuli_scaffold	52213	[A/C]	1067304251
Ca_Kabuli_NW_004515762.1_53621	Ca_kabuli_scaffold	53621	[T/G]	1067304252
Ca_Kabuli_NW_004515762.1_53633	Ca_kabuli_scaffold	53633	[A/C]	1067304253
Ca_Kabuli_NW_004515762.1_131228	Ca_kabuli_scaffold	131228	[T/C]	1067304254
Ca_Kabuli_NW_004515770.1_101376	Ca_kabuli_scaffold	101376	[C/T]	1067304255
Ca_Kabuli_NW_004515770.1_101382	Ca_kabuli_scaffold	101382	[T/C]	1067304256
Ca_Kabuli_NW_004515770.1_101411	Ca_kabuli_scaffold	101411	[A/G]	1067304257
Ca_Kabuli_NW_004515774.1_30857	Ca_kabuli_scaffold	30857	[A/G]	1067304258
Ca_Kabuli_NW_004515776.1_58689	Ca_kabuli_scaffold	58689	[T/A]	1067304259
Ca_Kabuli_NW_004515776.1_432685	Ca_kabuli_scaffold	432685	[T/G]	1067304260
Ca_Kabuli_NW_004515781.1_38302	Ca_kabuli_scaffold	38302	[A/C]	1067304261
Ca_Kabuli_NW_004515785.1_25861	Ca_kabuli_scaffold	25861	[A/G]	1067304262
Ca_Kabuli_NW_004515785.1_25865	Ca_kabuli_scaffold	25865	[A/G]	1067304263
Ca_Kabuli_NW_004515785.1_25888	Ca_kabuli_scaffold	25888	[T/C]	1067304264
Ca_Kabuli_NW_004515785.1_25896	Ca_kabuli_scaffold	25896	[T/C]	1067304265
Ca_Kabuli_NW_004515787.1_226793	Ca_kabuli_scaffold	226793	[T/C]	1067304266
Ca_Kabuli_NW_004515792.1_3151	Ca_kabuli_scaffold	3151	[G/T]	1067304267
Ca_Kabuli_NW_004515792.1_3158	Ca_kabuli_scaffold	3158	[C/G]	1067304268
Ca_Kabuli_NW_004515794.1_192327	Ca_kabuli_scaffold	192327	[G/A]	1067304269
Ca_Kabuli_NW_004515794.1_204040	Ca_kabuli_scaffold	204040	[A/G]	1067304270

SNP IDs	Chromosomes	Physical positions (bp)	SNPs	NCBI SNPdb accession IDs (NCBI_ss#)
Ca_Kabuli_NW_004515794.1_204041	Ca_kabuli_scaffold	204041	[T/A]	1067304271
Ca_Kabuli_NW_004515794.1_205536	Ca_kabuli_scaffold	205536	[T/G]	1067304272
Ca_Kabuli_NW_004515794.1_205637	Ca_kabuli_scaffold	205637	[T/A]	1067304273
Ca_Kabuli_NW_004515795.1_208768	Ca_kabuli_scaffold	208768	[G/A]	1067304274
Ca_Kabuli_NW_004515795.1_208780	Ca_kabuli_scaffold	208780	[A/C]	1067304275
Ca_Kabuli_NW_004515795.1_208785	Ca_kabuli_scaffold	208785	[T/A]	1067304276
Ca_Kabuli_NW_004515795.1_208786	Ca_kabuli_scaffold	208786	[G/C]	1067304277
Ca_Kabuli_NW_004515795.1_208788	Ca_kabuli_scaffold	208788	[C/T]	1067304278
Ca_Kabuli_NW_004515795.1_208789	Ca_kabuli_scaffold	208789	[A/C]	1067304279
Ca_Kabuli_NW_004515795.1_208793	Ca_kabuli_scaffold	208793	[G/C]	1067304280
Ca_Kabuli_NW_004515795.1_208932	Ca_kabuli_scaffold	208932	[G/T]	1067304281
Ca_Kabuli_NW_004515795.1_208939	Ca_kabuli_scaffold	208939	[G/A]	1067304282
Ca_Kabuli_NW_004515795.1_208957	Ca_kabuli_scaffold	208957	[C/T]	1067304283
Ca_Kabuli_NW_004515795.1_208958	Ca_kabuli_scaffold	208958	[C/T]	1067304284
Ca_Kabuli_NW_004515795.1_208981	Ca_kabuli_scaffold	208981	[G/T]	1067304285
Ca_Kabuli_NW_004515795.1_404479	Ca_kabuli_scaffold	404479	[C/A]	1067304286
Ca_Kabuli_NW_004515797.1_72469	Ca_kabuli_scaffold	72469	[A/G]	1067304287
Ca_Kabuli_NW_004515797.1_72491	Ca_kabuli_scaffold	72491	[T/C]	1067304288
Ca_Kabuli_NW_004515797.1_72495	Ca_kabuli_scaffold	72495	[G/A]	1067304289
Ca_Kabuli_NW_004515798.1_108082	Ca_kabuli_scaffold	108082	[T/G]	1067304290
Ca_Kabuli_NW_004515798.1_108110	Ca_kabuli_scaffold	108110	[T/A]	1067304291
Ca_Kabuli_NW_004515798.1_112349	Ca_kabuli_scaffold	112349	[T/C]	1067304292
Ca_Kabuli_NW_004515798.1_118812	Ca_kabuli_scaffold	118812	[A/C]	1067304293
Ca_Kabuli_NW_004515798.1_118821	Ca_kabuli_scaffold	118821	[G/C]	1067304294
Ca_Kabuli_NW_004515798.1_118838	Ca_kabuli_scaffold	118838	[A/T]	1067304295
Ca_Kabuli_NW_004515798.1_118839	Ca_kabuli_scaffold	118839	[A/G]	1067304296
Ca_Kabuli_NW_004515798.1_118840	Ca_kabuli_scaffold	118840	[G/A]	1067304297
Ca_Kabuli_NW_004515799.1_26658	Ca_kabuli_scaffold	26658	[G/A]	1067304298
Ca_Kabuli_NW_004515802.1_640348	Ca_kabuli_scaffold	640348	[C/T]	1067304299
Ca_Kabuli_NW_004515802.1_640349	Ca_kabuli_scaffold	640349	[G/C]	1067304300
Ca_Kabuli_NW_004515802.1_640350	Ca_kabuli_scaffold	640350	[G/T]	1067304301
Ca_Kabuli_NW_004515802.1_640351	Ca_kabuli_scaffold	640351	[T/G]	1067304302
Ca_Kabuli_NW_004515802.1_640364	Ca_kabuli_scaffold	640364	[T/G]	1067304303
Ca_Kabuli_NW_004515802.1_654534	Ca_kabuli_scaffold	654534	[T/G]	1067304304

SNP IDs	Chromosomes	Physical positions (bp)	SNPs	NCBI SNPdb accession IDs (NCBI_ss#)
Ca_Kabuli_NW_004515802.1_654573	Ca_kabuli_scaffold	654573	[A/C]	1067304305
Ca_Kabuli_NW_004515802.1_654611	Ca_kabuli_scaffold	654611	[A/G]	1067304306
Ca_Kabuli_NW_004515802.1_728756	Ca_kabuli_scaffold	728756	[C/T]	1067304307
Ca_Kabuli_NW_004515802.1_728793	Ca_kabuli_scaffold	728793	[C/T]	1067304308
Ca_Kabuli_NW_004515802.1_728809	Ca_kabuli_scaffold	728809	[A/T]	1067304309
Ca_Kabuli_NW_004515802.1_728812	Ca_kabuli_scaffold	728812	[A/G]	1067304310
Ca_Kabuli_NW_004515804.1_18913	Ca_kabuli_scaffold	18913	[T/C]	1067304311
Ca_Kabuli_NW_004515804.1_18915	Ca_kabuli_scaffold	18915	[G/T]	1067304312
Ca_Kabuli_NW_004515804.1_18916	Ca_kabuli_scaffold	18916	[C/T]	1067304313
Ca_Kabuli_NW_004515804.1_163581	Ca_kabuli_scaffold	163581	[T/C]	1067304314
Ca_Kabuli_NW_004515806.1_84042	Ca_kabuli_scaffold	84042	[T/C]	1067304315
Ca_Kabuli_NW_004515806.1_123585	Ca_kabuli_scaffold	123585	[C/G]	1067304316
Ca_Kabuli_NW_004515806.1_242468	Ca_kabuli_scaffold	242468	[T/C]	1067304317
Ca_Kabuli_NW_004515806.1_251371	Ca_kabuli_scaffold	251371	[A/G]	1067304318
Ca_Kabuli_NW_004515806.1_251412	Ca_kabuli_scaffold	251412	[G/A]	1067304319
Ca_Kabuli_NW_004515806.1_251415	Ca_kabuli_scaffold	251415	[G/A]	1067304320
Ca_Kabuli_NW_004515806.1_438803	Ca_kabuli_scaffold	438803	[A/C]	1067304321
Ca_Kabuli_NW_004515806.1_438866	Ca_kabuli_scaffold	438866	[T/C]	1067304322
Ca_Kabuli_NW_004515806.1_449803	Ca_kabuli_scaffold	449803	[T/G]	1067304323
Ca_Kabuli_NW_004515806.1_449806	Ca_kabuli_scaffold	449806	[T/G]	1067304324
Ca_Kabuli_NW_004515806.1_626620	Ca_kabuli_scaffold	626620	[C/A]	1067304325
Ca_Kabuli_NW_004515806.1_631375	Ca_kabuli_scaffold	631375	[G/A]	1067304326
Ca_Kabuli_NW_004515806.1_636641	Ca_kabuli_scaffold	636641	[T/C]	1067304327
Ca_Kabuli_NW_004515806.1_636672	Ca_kabuli_scaffold	636672	[A/G]	1067304328
Ca_Kabuli_NW_004515806.1_711220	Ca_kabuli_scaffold	711220	[G/A]	1067304329
Ca_Kabuli_NW_004515806.1_800084	Ca_kabuli_scaffold	800084	[A/G]	1067304330
Ca_Kabuli_NW_004515806.1_853235	Ca_kabuli_scaffold	853235	[C/A]	1067304331
Ca_Kabuli_NW_004515810.1_56331	Ca_kabuli_scaffold	56331	[A/G]	1067304332
Ca_Kabuli_NW_004515810.1_59807	Ca_kabuli_scaffold	59807	[A/C]	1067304333
Ca_Kabuli_NW_004515810.1_361586	Ca_kabuli_scaffold	361586	[T/G]	1067304334
Ca_Kabuli_NW_004515810.1_361598	Ca_kabuli_scaffold	361598	[T/G]	1067304335
Ca_Kabuli_NW_004515810.1_474335	Ca_kabuli_scaffold	474335	[T/G]	1067304336
Ca_Kabuli_NW_004515810.1_474371	Ca_kabuli_scaffold	474371	[A/G]	1067304337
Ca_Kabuli_NW_004515810.1_477263	Ca_kabuli_scaffold	477263	[A/G]	1067304338

SNP IDs	Chromosomes	Physical positions (bp)	SNPs	NCBI SNPdb accession IDs (NCBI_ss#)
Ca_Kabuli_NW_004515810.1_477274	Ca_kabuli_scaffold	477274	[A/C]	1067304339
Ca_Kabuli_NW_004515810.1_477276	Ca_kabuli_scaffold	477276	[A/G]	1067304340
Ca_Kabuli_NW_004515810.1_478337	Ca_kabuli_scaffold	478337	[A/G]	1067304341
Ca_Kabuli_NW_004515811.1_150147	Ca_kabuli_scaffold	150147	[C/G]	1067304342
Ca_Kabuli_NW_004515811.1_150172	Ca_kabuli_scaffold	150172	[T/G]	1067304343
Ca_Kabuli_NW_004515811.1_150181	Ca_kabuli_scaffold	150181	[A/G]	1067304344
Ca_Kabuli_NW_004515813.1_111933	Ca_kabuli_scaffold	111933	[C/T]	1067304345
Ca_Kabuli_NW_004515813.1_111936	Ca_kabuli_scaffold	111936	[C/A]	1067304346
Ca_Kabuli_NW_004515817.1_49253	Ca_kabuli_scaffold	49253	[G/T]	1067304347
Ca_Kabuli_NW_004515817.1_49263	Ca_kabuli_scaffold	49263	[G/A]	1067304348
Ca_Kabuli_NW_004515817.1_49265	Ca_kabuli_scaffold	49265	[G/A]	1067304349
Ca_Kabuli_NW_004515817.1_49266	Ca_kabuli_scaffold	49266	[T/G]	1067304350
Ca_Kabuli_NW_004515817.1_49268	Ca_kabuli_scaffold	49268	[C/G]	1067304351
Ca_Kabuli_NW_004515817.1_49272	Ca_kabuli_scaffold	49272	[A/C]	1067304352
Ca_Kabuli_NW_004515817.1_117071	Ca_kabuli_scaffold	117071	[A/G]	1067304353
Ca_Kabuli_NW_004515817.1_138929	Ca_kabuli_scaffold	138929	[G/A]	1067304354
Ca_Kabuli_NW_004515817.1_194998	Ca_kabuli_scaffold	194998	[A/C]	1067304355
Ca_Kabuli_NW_004515817.1_195000	Ca_kabuli_scaffold	195000	[C/T]	1067304356
Ca_Kabuli_NW_004515818.1_15529	Ca_kabuli_scaffold	15529	[A/T]	1067304357
Ca_Kabuli_NW_004515818.1_15548	Ca_kabuli_scaffold	15548	[A/C]	1067304358
Ca_Kabuli_NW_004515818.1_19215	Ca_kabuli_scaffold	19215	[C/A]	1067304359
Ca_Kabuli_NW_004515818.1_28542	Ca_kabuli_scaffold	28542	[T/G]	1067304360
Ca_Kabuli_NW_004515818.1_28559	Ca_kabuli_scaffold	28559	[T/G]	1067304361
Ca_Kabuli_NW_004515818.1_33267	Ca_kabuli_scaffold	33267	[A/C]	1067304362
Ca_Kabuli_NW_004515818.1_221374	Ca_kabuli_scaffold	221374	[G/A]	1067304363
Ca_Kabuli_NW_004515818.1_221375	Ca_kabuli_scaffold	221375	[G/C]	1067304364
Ca_Kabuli_NW_004515818.1_221376	Ca_kabuli_scaffold	221376	[A/C]	1067304365
Ca_Kabuli_NW_004515818.1_410571	Ca_kabuli_scaffold	410571	[C/A]	1067304366
Ca_Kabuli_NW_004515819.1_143867	Ca_kabuli_scaffold	143867	[C/T]	1067304367
Ca_Kabuli_NW_004515819.1_143910	Ca_kabuli_scaffold	143910	[T/C]	1067304368
Ca_Kabuli_NW_004515819.1_144276	Ca_kabuli_scaffold	144276	[T/G]	1067304369
Ca_Kabuli_NW_004515819.1_144316	Ca_kabuli_scaffold	144316	[A/G]	1067304370
Ca_Kabuli_NW_004515819.1_144320	Ca_kabuli_scaffold	144320	[T/C]	1067304371
Ca_Kabuli_NW_004515819.1_144365	Ca_kabuli_scaffold	144365	[T/C]	1067304372

SNP IDs	Chromosomes	Physical positions (bp)	SNPs	NCBI SNPdb accession IDs (NCBI_ss#)
Ca_Kabuli_NW_004515819.1_144385	Ca_kabuli_scaffold	144385	[T/C]	1067304373
Ca_Kabuli_NW_004515819.1_144402	Ca_kabuli_scaffold	144402	[T/G]	1067304374
Ca_Kabuli_NW_004515819.1_144405	Ca_kabuli_scaffold	144405	[T/G]	1067304375
Ca_Kabuli_NW_004515819.1_144465	Ca_kabuli_scaffold	144465	[T/G]	1067304376
Ca_Kabuli_NW_004515819.1_152512	Ca_kabuli_scaffold	152512	[T/G]	1067304377
Ca_Kabuli_NW_004515819.1_283227	Ca_kabuli_scaffold	283227	[T/G]	1067304378
Ca_Kabuli_NW_004515821.1_3546	Ca_kabuli_scaffold	3546	[A/C]	1067304379
Ca_Kabuli_NW_004515824.1_432431	Ca_kabuli_scaffold	432431	[A/C]	1067304380
Ca_Kabuli_NW_004515824.1_494593	Ca_kabuli_scaffold	494593	[A/C]	1067304381
Ca_Kabuli_NW_004515824.1_494604	Ca_kabuli_scaffold	494604	[A/G]	1067304382
Ca_Kabuli_NW_004515824.1_494607	Ca_kabuli_scaffold	494607	[T/A]	1067304383
Ca_Kabuli_NW_004515824.1_494636	Ca_kabuli_scaffold	494636	[T/A]	1067304384
Ca_Kabuli_NW_004515824.1_535289	Ca_kabuli_scaffold	535289	[A/C]	1067304385
Ca_Kabuli_NW_004515824.1_620942	Ca_kabuli_scaffold	620942	[T/C]	1067304386
Ca_Kabuli_NW_004515824.1_620957	Ca_kabuli_scaffold	620957	[T/C]	1067304387
Ca_Kabuli_NW_004515824.1_620965	Ca_kabuli_scaffold	620965	[T/C]	1067304388
Ca_Kabuli_NW_004515826.1_34409	Ca_kabuli_scaffold	34409	[T/A]	1067304389
Ca_Kabuli_NW_004515826.1_34468	Ca_kabuli_scaffold	34468	[A/T]	1067304390
Ca_Kabuli_NW_004515828.1_72799	Ca_kabuli_scaffold	72799	[A/T]	1067304391
Ca_Kabuli_NW_004515831.1_15590	Ca_kabuli_scaffold	15590	[G/T]	1067304392
Ca_Kabuli_NW_004515838.1_215096	Ca_kabuli_scaffold	215096	[A/G]	1067304393
Ca_Kabuli_NW_004515838.1_215099	Ca_kabuli_scaffold	215099	[G/T]	1067304394
Ca_Kabuli_NW_004515838.1_218577	Ca_kabuli_scaffold	218577	[A/G]	1067304395
Ca_Kabuli_NW_004515838.1_218580	Ca_kabuli_scaffold	218580	[G/T]	1067304396
Ca_Kabuli_NW_004515841.1_60842	Ca_kabuli_scaffold	60842	[A/G]	1067304397
Ca_Kabuli_NW_004515841.1_60856	Ca_kabuli_scaffold	60856	[T/C]	1067304398
Ca_Kabuli_NW_004515841.1_60859	Ca_kabuli_scaffold	60859	[A/G]	1067304399
Ca_Kabuli_NW_004515841.1_60923	Ca_kabuli_scaffold	60923	[T/G]	1067304400
Ca_Kabuli_NW_004515841.1_60932	Ca_kabuli_scaffold	60932	[T/G]	1067304401
Ca_Kabuli_NW_004515841.1_60934	Ca_kabuli_scaffold	60934	[A/G]	1067304402
Ca_Kabuli_NW_004515841.1_60949	Ca_kabuli_scaffold	60949	[T/C]	1067304403
Ca_Kabuli_NW_004515841.1_60950	Ca_kabuli_scaffold	60950	[T/C]	1067304404
Ca_Kabuli_NW_004515841.1_136077	Ca_kabuli_scaffold	136077	[C/G]	1067304405
Ca_Kabuli_NW_004515841.1_136096	Ca_kabuli_scaffold	136096	[T/C]	1067304406

SNP IDs	Chromosomes	Physical positions (bp)	SNPs	NCBI SNPdb accession IDs (NCBI_ss#)
Ca_Kabuli_NW_004515841.1_136106	Ca_kabuli_scaffold	136106	[G/A]	1067304407
Ca_Kabuli_NW_004515842.1_172132	Ca_kabuli_scaffold	172132	[T/G]	1067304408
Ca_Kabuli_NW_004515842.1_172190	Ca_kabuli_scaffold	172190	[A/G]	1067304409
Ca_Kabuli_NW_004515842.1_636950	Ca_kabuli_scaffold	636950	[A/C]	1067304410
Ca_Kabuli_NW_004515843.1_8242	Ca_kabuli_scaffold	8242	[G/T]	1067304411
Ca_Kabuli_NW_004515843.1_14642	Ca_kabuli_scaffold	14642	[T/G]	1067304412
Ca_Kabuli_NW_004515843.1_14654	Ca_kabuli_scaffold	14654	[T/G]	1067304413
Ca_Kabuli_NW_004515844.1_172829	Ca_kabuli_scaffold	172829	[A/C]	1067304414
Ca_Kabuli_NW_004515846.1_359400	Ca_kabuli_scaffold	359400	[T/G]	1067304415
Ca_Kabuli_NW_004515850.1_62780	Ca_kabuli_scaffold	62780	[C/A]	1067304416
Ca_Kabuli_NW_004515850.1_71326	Ca_kabuli_scaffold	71326	[A/G]	1067304417
Ca_Kabuli_NW_004515853.1_30224	Ca_kabuli_scaffold	30224	[G/A]	1067304418
Ca_Kabuli_NW_004515853.1_30252	Ca_kabuli_scaffold	30252	[C/T]	1067304419
Ca_Kabuli_NW_004515853.1_107892	Ca_kabuli_scaffold	107892	[A/G]	1067304420
Ca_Kabuli_NW_004515853.1_107896	Ca_kabuli_scaffold	107896	[G/A]	1067304421
Ca_Kabuli_NW_004515853.1_107897	Ca_kabuli_scaffold	107897	[T/C]	1067304422
Ca_Kabuli_NW_004515853.1_107908	Ca_kabuli_scaffold	107908	[C/T]	1067304423
Ca_Kabuli_NW_004515853.1_188959	Ca_kabuli_scaffold	188959	[T/G]	1067304424
Ca_Kabuli_NW_004515859.1_165014	Ca_kabuli_scaffold	165014	[A/G]	1067304425
Ca_Kabuli_NW_004515859.1_165818	Ca_kabuli_scaffold	165818	[G/A]	1067304426
Ca_Kabuli_NW_004515865.1_182405	Ca_kabuli_scaffold	182405	[C/T]	1067304427
Ca_Kabuli_NW_004515866.1_7549	Ca_kabuli_scaffold	7549	[G/C]	1067304428
Ca_Kabuli_NW_004515867.1_54627	Ca_kabuli_scaffold	54627	[C/T]	1067304429
Ca_Kabuli_NW_004515867.1_132873	Ca_kabuli_scaffold	132873	[A/G]	1067304430
Ca_Kabuli_NW_004515867.1_132896	Ca_kabuli_scaffold	132896	[T/C]	1067304431
Ca_Kabuli_NW_004515867.1_132907	Ca_kabuli_scaffold	132907	[A/C]	1067304432
Ca_Kabuli_NW_004515870.1_65676	Ca_kabuli_scaffold	65676	[T/G]	1067304433
Ca_Kabuli_NW_004515870.1_177270	Ca_kabuli_scaffold	177270	[T/C]	1067304434
Ca_Kabuli_NW_004515870.1_177314	Ca_kabuli_scaffold	177314	[T/A]	1067304435
Ca_Kabuli_NW_004515870.1_177323	Ca_kabuli_scaffold	177323	[G/A]	1067304436
Ca_Kabuli_NW_004515870.1_227601	Ca_kabuli_scaffold	227601	[C/T]	1067304437
Ca_Kabuli_NW_004515870.1_227604	Ca_kabuli_scaffold	227604	[C/A]	1067304438
Ca_Kabuli_NW_004515870.1_227606	Ca_kabuli_scaffold	227606	[T/A]	1067304439
Ca_Kabuli_NW_004515870.1_227608	Ca_kabuli_scaffold	227608	[A/T]	1067304440

SNP IDs	Chromosomes	Physical positions (bp)	SNPs	NCBI SNPdb accession IDs (NCBI_ss#)
Ca_Kabuli_NW_004515870.1_227646	Ca_kabuli_scaffold	227646	[C/T]	1067304441
Ca_Kabuli_NW_004515874.1_3175	Ca_kabuli_scaffold	3175	[A/G]	1067304442
Ca_Kabuli_NW_004515874.1_3221	Ca_kabuli_scaffold	3221	[A/C]	1067304443
Ca_Kabuli_NW_004515874.1_3263	Ca_kabuli_scaffold	3263	[A/G]	1067304444
Ca_Kabuli_NW_004515881.1_121690	Ca_kabuli_scaffold	121690	[T/G]	1067304445
Ca_Kabuli_NW_004515881.1_297345	Ca_kabuli_scaffold	297345	[A/C]	1067304446
Ca_Kabuli_NW_004515882.1_385384	Ca_kabuli_scaffold	385384	[G/C]	1067304447
Ca_Kabuli_NW_004515883.1_46734	Ca_kabuli_scaffold	46734	[A/G]	1067304448
Ca_Kabuli_NW_004515883.1_271846	Ca_kabuli_scaffold	271846	[A/C]	1067304449
Ca_Kabuli_NW_004515883.1_272994	Ca_kabuli_scaffold	272994	[A/G]	1067304450
Ca_Kabuli_NW_004515883.1_273007	Ca_kabuli_scaffold	273007	[T/C]	1067304451
Ca_Kabuli_NW_004515883.1_273016	Ca_kabuli_scaffold	273016	[A/G]	1067304452
Ca_Kabuli_NW_004515883.1_273054	Ca_kabuli_scaffold	273054	[T/C]	1067304453
Ca_Kabuli_NW_004515883.1_299009	Ca_kabuli_scaffold	299009	[A/C]	1067304454
Ca_Kabuli_NW_004515883.1_299010	Ca_kabuli_scaffold	299010	[G/T]	1067304455
Ca_Kabuli_NW_004515883.1_441561	Ca_kabuli_scaffold	441561	[A/C]	1067304456
Ca_Kabuli_NW_004515883.1_441566	Ca_kabuli_scaffold	441566	[T/C]	1067304457
Ca_Kabuli_NW_004515883.1_441589	Ca_kabuli_scaffold	441589	[T/C]	1067304458
Ca_Kabuli_NW_004515883.1_441592	Ca_kabuli_scaffold	441592	[T/C]	1067304459
Ca_Kabuli_NW_004515883.1_441609	Ca_kabuli_scaffold	441609	[A/T]	1067304460
Ca_Kabuli_NW_004515887.1_340219	Ca_kabuli_scaffold	340219	[A/G]	1067304461
Ca_Kabuli_NW_004515887.1_340240	Ca_kabuli_scaffold	340240	[A/C]	1067304462
Ca_Kabuli_NW_004515894.1_100139	Ca_kabuli_scaffold	100139	[A/G]	1067304463
Ca_Kabuli_NW_004515895.1_16423	Ca_kabuli_scaffold	16423	[C/T]	1067304464
Ca_Kabuli_NW_004515895.1_158827	Ca_kabuli_scaffold	158827	[T/C]	1067304465
Ca_Kabuli_NW_004515895.1_158877	Ca_kabuli_scaffold	158877	[A/C]	1067304466
Ca_Kabuli_NW_004515895.1_158880	Ca_kabuli_scaffold	158880	[A/G]	1067304467
Ca_Kabuli_NW_004515895.1_160613	Ca_kabuli_scaffold	160613	[T/C]	1067304468
Ca_Kabuli_NW_004515895.1_160626	Ca_kabuli_scaffold	160626	[A/C]	1067304469
Ca_Kabuli_NW_004515895.1_160630	Ca_kabuli_scaffold	160630	[A/G]	1067304470
Ca_Kabuli_NW_004515895.1_201875	Ca_kabuli_scaffold	201875	[T/G]	1067304471
Ca_Kabuli_NW_004515896.1_34058	Ca_kabuli_scaffold	34058	[T/A]	1067304472
Ca_Kabuli_NW_004515896.1_34060	Ca_kabuli_scaffold	34060	[A/G]	1067304473
Ca_Kabuli_NW_004515896.1_34105	Ca_kabuli_scaffold	34105	[A/T]	1067304474

SNP IDs	Chromosomes	Physical positions (bp)	SNPs	NCBI SNPdb accession IDs (NCBI_ss#)
Ca_Kabuli_NW_004515896.1_34118	Ca_kabuli_scaffold	34118	[T/A]	1067304475
Ca_Kabuli_NW_004515896.1_34175	Ca_kabuli_scaffold	34175	[T/G]	1067304476
Ca_Kabuli_NW_004515896.1_34178	Ca_kabuli_scaffold	34178	[T/G]	1067304477
Ca_Kabuli_NW_004515896.1_34202	Ca_kabuli_scaffold	34202	[A/C]	1067304478
Ca_Kabuli_NW_004515896.1_42506	Ca_kabuli_scaffold	42506	[A/C]	1067304479
Ca_Kabuli_NW_004515896.1_42563	Ca_kabuli_scaffold	42563	[T/C]	1067304480
Ca_Kabuli_NW_004515896.1_43153	Ca_kabuli_scaffold	43153	[A/T]	1067304481
Ca_Kabuli_NW_004515896.1_43158	Ca_kabuli_scaffold	43158	[C/A]	1067304482
Ca_Kabuli_NW_004515896.1_43170	Ca_kabuli_scaffold	43170	[A/T]	1067304483
Ca_Kabuli_NW_004515896.1_43179	Ca_kabuli_scaffold	43179	[A/C]	1067304484
Ca_Kabuli_NW_004515896.1_43265	Ca_kabuli_scaffold	43265	[T/C]	1067304485
Ca_Kabuli_NW_004515896.1_43299	Ca_kabuli_scaffold	43299	[C/A]	1067304486
Ca_Kabuli_NW_004515896.1_43300	Ca_kabuli_scaffold	43300	[C/G]	1067304487
Ca_Kabuli_NW_004515896.1_43301	Ca_kabuli_scaffold	43301	[T/A]	1067304488
Ca_Kabuli_NW_004515896.1_43302	Ca_kabuli_scaffold	43302	[G/T]	1067304489
Ca_Kabuli_NW_004515896.1_43360	Ca_kabuli_scaffold	43360	[A/G]	1067304490
Ca_Kabuli_NW_004515896.1_44391	Ca_kabuli_scaffold	44391	[C/T]	1067304491
Ca_Kabuli_NW_004515896.1_44392	Ca_kabuli_scaffold	44392	[A/C]	1067304492
Ca_Kabuli_NW_004515896.1_44393	Ca_kabuli_scaffold	44393	[G/T]	1067304493
Ca_Kabuli_NW_004515896.1_254902	Ca_kabuli_scaffold	254902	[A/G]	1067304494
Ca_Kabuli_NW_004515897.1_33465	Ca_kabuli_scaffold	33465	[T/G]	1067304495
Ca_Kabuli_NW_004515897.1_33475	Ca_kabuli_scaffold	33475	[T/G]	1067304496
Ca_Kabuli_NW_004515897.1_33479	Ca_kabuli_scaffold	33479	[A/C]	1067304497
Ca_Kabuli_NW_004515897.1_33494	Ca_kabuli_scaffold	33494	[T/G]	1067304498
Ca_Kabuli_NW_004515897.1_35788	Ca_kabuli_scaffold	35788	[A/G]	1067304499
Ca_Kabuli_NW_004515897.1_340974	Ca_kabuli_scaffold	340974	[T/G]	1067304500
Ca_Kabuli_NW_004515897.1_341102	Ca_kabuli_scaffold	341102	[A/G]	1067304501
Ca_Kabuli_NW_004515897.1_341124	Ca_kabuli_scaffold	341124	[T/G]	1067304502
Ca_Kabuli_NW_004515898.1_25528	Ca_kabuli_scaffold	25528	[A/C]	1067304503
Ca_Kabuli_NW_004515898.1_81750	Ca_kabuli_scaffold	81750	[G/A]	1067304504
Ca_Kabuli_NW_004515898.1_81751	Ca_kabuli_scaffold	81751	[G/T]	1067304505
Ca_Kabuli_NW_004515898.1_81752	Ca_kabuli_scaffold	81752	[A/C]	1067304506
Ca_Kabuli_NW_004515898.1_81753	Ca_kabuli_scaffold	81753	[G/T]	1067304507
Ca_Kabuli_NW_004515909.1_122032	Ca_kabuli_scaffold	122032	[T/C]	1067304508

SNP IDs	Chromosomes	Physical positions (bp)	SNPs	NCBI SNPdb accession IDs (NCBI_ss#)
Ca_Kabuli_NW_004515912.1_1297	Ca_kabuli_scaffold	1297	[T/C]	1067304509
Ca_Kabuli_NW_004515918.1_41902	Ca_kabuli_scaffold	41902	[A/T]	1067304510
Ca_Kabuli_NW_004515918.1_197253	Ca_kabuli_scaffold	197253	[A/T]	1067304511
Ca_Kabuli_NW_004515918.1_197258	Ca_kabuli_scaffold	197258	[T/G]	1067304512
Ca_Kabuli_NW_004515918.1_250140	Ca_kabuli_scaffold	250140	[A/G]	1067304513
Ca_Kabuli_NW_004515918.1_275479	Ca_kabuli_scaffold	275479	[A/G]	1067304514
Ca_Kabuli_NW_004515918.1_275488	Ca_kabuli_scaffold	275488	[A/C]	1067304515
Ca_Kabuli_NW_004515918.1_275497	Ca_kabuli_scaffold	275497	[T/C]	1067304516
Ca_Kabuli_NW_004515918.1_275511	Ca_kabuli_scaffold	275511	[A/T]	1067304517
Ca_Kabuli_NW_004515918.1_275517	Ca_kabuli_scaffold	275517	[T/A]	1067304518
Ca_Kabuli_NW_004515918.1_275519	Ca_kabuli_scaffold	275519	[T/C]	1067304519
Ca_Kabuli_NW_004515918.1_275532	Ca_kabuli_scaffold	275532	[T/C]	1067304520
Ca_Kabuli_NW_004515918.1_369299	Ca_kabuli_scaffold	369299	[C/T]	1067304521
Ca_Kabuli_NW_004515918.1_430673	Ca_kabuli_scaffold	430673	[A/C]	1067304522
Ca_Kabuli_NW_004515919.1_103596	Ca_kabuli_scaffold	103596	[A/G]	1067304523
Ca_Kabuli_NW_004515919.1_128379	Ca_kabuli_scaffold	128379	[A/C]	1067304524
Ca_Kabuli_NW_004515919.1_128380	Ca_kabuli_scaffold	128380	[G/T]	1067304525
Ca_Kabuli_NW_004515919.1_226673	Ca_kabuli_scaffold	226673	[C/A]	1067304526
Ca_Kabuli_NW_004515919.1_428338	Ca_kabuli_scaffold	428338	[A/G]	1067304527
Ca_Kabuli_NW_004515919.1_428393	Ca_kabuli_scaffold	428393	[T/C]	1067304528
Ca_Kabuli_NW_004515919.1_493576	Ca_kabuli_scaffold	493576	[T/G]	1067304529
Ca_Kabuli_NW_004515919.1_493596	Ca_kabuli_scaffold	493596	[G/A]	1067304530
Ca_Kabuli_NW_004515921.1_43504	Ca_kabuli_scaffold	43504	[A/C]	1067304531
Ca_Kabuli_NW_004515921.1_45345	Ca_kabuli_scaffold	45345	[A/G]	1067304532
Ca_Kabuli_NW_004515921.1_154415	Ca_kabuli_scaffold	154415	[T/C]	1067304533
Ca_Kabuli_NW_004515921.1_154475	Ca_kabuli_scaffold	154475	[A/G]	1067304534
Ca_Kabuli_NW_004515921.1_478760	Ca_kabuli_scaffold	478760	[G/T]	1067304535
Ca_Kabuli_NW_004515921.1_478761	Ca_kabuli_scaffold	478761	[C/G]	1067304536
Ca_Kabuli_NW_004515921.1_478807	Ca_kabuli_scaffold	478807	[A/C]	1067304537
Ca_Kabuli_NW_004515921.1_478826	Ca_kabuli_scaffold	478826	[A/C]	1067304538
Ca_Kabuli_NW_004515931.1_32026	Ca_kabuli_scaffold	32026	[C/T]	1067304539
Ca_Kabuli_NW_004515934.1_129869	Ca_kabuli_scaffold	129869	[A/C]	1067304540
Ca_Kabuli_NW_004515934.1_215946	Ca_kabuli_scaffold	215946	[T/G]	1067304541
Ca_Kabuli_NW_004515934.1_215949	Ca_kabuli_scaffold	215949	[A/C]	1067304542

SNP IDs	Chromosomes	Physical positions (bp)	SNPs	NCBI SNPdb accession IDs (NCBI_ss#)
Ca_Kabuli_NW_004515934.1_215950	Ca_kabuli_scaffold	215950	[G/C]	1067304543
Ca_Kabuli_NW_004515934.1_215952	Ca_kabuli_scaffold	215952	[C/A]	1067304544
Ca_Kabuli_NW_004515937.1_136806	Ca_kabuli_scaffold	136806	[G/T]	1067304545
Ca_Kabuli_NW_004515937.1_136822	Ca_kabuli_scaffold	136822	[A/G]	1067304546
Ca_Kabuli_NW_004515939.1_8270	Ca_kabuli_scaffold	8270	[T/C]	1067304547
Ca_Kabuli_NW_004515939.1_8303	Ca_kabuli_scaffold	8303	[A/G]	1067304548
Ca_Kabuli_NW_004515942.1_64422	Ca_kabuli_scaffold	64422	[A/T]	1067304549
Ca_Kabuli_NW_004515942.1_64446	Ca_kabuli_scaffold	64446	[A/C]	1067304550
Ca_Kabuli_NW_004515942.1_64448	Ca_kabuli_scaffold	64448	[A/C]	1067304551
Ca_Kabuli_NW_004515942.1_64460	Ca_kabuli_scaffold	64460	[A/G]	1067304552
Ca_Kabuli_NW_004515945.1_203830	Ca_kabuli_scaffold	203830	[T/A]	1067304553
Ca_Kabuli_NW_004515945.1_203879	Ca_kabuli_scaffold	203879	[T/G]	1067304554
Ca_Kabuli_NW_004515945.1_203973	Ca_kabuli_scaffold	203973	[T/A]	1067304555
Ca_Kabuli_NW_004515945.1_209730	Ca_kabuli_scaffold	209730	[A/G]	1067304556
Ca_Kabuli_NW_004515945.1_209746	Ca_kabuli_scaffold	209746	[A/G]	1067304557
Ca_Kabuli_NW_004515945.1_209762	Ca_kabuli_scaffold	209762	[A/C]	1067304558
Ca_Kabuli_NW_004515945.1_432229	Ca_kabuli_scaffold	432229	[A/C]	1067304559
Ca_Kabuli_NW_004515945.1_660749	Ca_kabuli_scaffold	660749	[G/C]	1067304560
Ca_Kabuli_NW_004515945.1_660796	Ca_kabuli_scaffold	660796	[A/C]	1067304561
Ca_Kabuli_NW_004515945.1_660816	Ca_kabuli_scaffold	660816	[A/G]	1067304562
Ca_Kabuli_NW_004515945.1_660826	Ca_kabuli_scaffold	660826	[A/C]	1067304563
Ca_Kabuli_NW_004515953.1_77778	Ca_kabuli_scaffold	77778	[A/T]	1067304564
Ca_Kabuli_NW_004515954.1_111894	Ca_kabuli_scaffold	111894	[A/T]	1067304565
Ca_Kabuli_NW_004515954.1_131751	Ca_kabuli_scaffold	131751	[G/C]	1067304566
Ca_Kabuli_NW_004515954.1_133397	Ca_kabuli_scaffold	133397	[G/T]	1067304567
Ca_Kabuli_NW_004515957.1_148622	Ca_kabuli_scaffold	148622	[A/C]	1067304568
Ca_Kabuli_NW_004515963.1_78149	Ca_kabuli_scaffold	78149	[C/T]	1067304569
Ca_Kabuli_NW_004515963.1_78156	Ca_kabuli_scaffold	78156	[T/C]	1067304570
Ca_Kabuli_NW_004515965.1_172269	Ca_kabuli_scaffold	172269	[A/G]	1067304571
Ca_Kabuli_NW_004515965.1_172296	Ca_kabuli_scaffold	172296	[A/G]	1067304572
Ca_Kabuli_NW_004515965.1_172302	Ca_kabuli_scaffold	172302	[T/C]	1067304573
Ca_Kabuli_NW_004515965.1_172304	Ca_kabuli_scaffold	172304	[A/G]	1067304574
Ca_Kabuli_NW_004515965.1_173404	Ca_kabuli_scaffold	173404	[A/C]	1067304575
Ca_Kabuli_NW_004515967.1_7241	Ca_kabuli_scaffold	7241	[T/C]	1067304576

SNP IDs	Chromosomes	Physical positions (bp)	SNPs	NCBI SNPdb accession IDs (NCBI_ss#)
Ca_Kabuli_NW_004515967.1_7260	Ca_kabuli_scaffold	7260	[A/C]	1067304577
Ca_Kabuli_NW_004515973.1_690	Ca_kabuli_scaffold	690	[G/A]	1067304578
Ca_Kabuli_NW_004515973.1_721	Ca_kabuli_scaffold	721	[C/T]	1067304579
Ca_Kabuli_NW_004515974.1_191705	Ca_kabuli_scaffold	191705	[A/T]	1067304580
Ca_Kabuli_NW_004515974.1_191723	Ca_kabuli_scaffold	191723	[A/T]	1067304581
Ca_Kabuli_NW_004515974.1_191929	Ca_kabuli_scaffold	191929	[C/T]	1067304582
Ca_Kabuli_NW_004515974.1_191973	Ca_kabuli_scaffold	191973	[A/T]	1067304583
Ca_Kabuli_NW_004515974.1_247948	Ca_kabuli_scaffold	247948	[T/A]	1067304584
Ca_Kabuli_NW_004515974.1_247957	Ca_kabuli_scaffold	247957	[T/G]	1067304585
Ca_Kabuli_NW_004515974.1_265942	Ca_kabuli_scaffold	265942	[T/G]	1067304586
Ca_Kabuli_NW_004515976.1_83032	Ca_kabuli_scaffold	83032	[A/G]	1067304587
Ca_Kabuli_NW_004515976.1_83033	Ca_kabuli_scaffold	83033	[G/A]	1067304588
Ca_Kabuli_NW_004515976.1_83034	Ca_kabuli_scaffold	83034	[C/T]	1067304589
Ca_Kabuli_NW_004515976.1_83036	Ca_kabuli_scaffold	83036	[G/T]	1067304590
Ca_Kabuli_NW_004515976.1_83094	Ca_kabuli_scaffold	83094	[A/T]	1067304591
Ca_Kabuli_NW_004515976.1_112095	Ca_kabuli_scaffold	112095	[T/C]	1067304592
Ca_Kabuli_NW_004515976.1_112121	Ca_kabuli_scaffold	112121	[T/A]	1067304593
Ca_Kabuli_NW_004515976.1_112130	Ca_kabuli_scaffold	112130	[A/C]	1067304594
Ca_Kabuli_NW_004515976.1_118571	Ca_kabuli_scaffold	118571	[T/A]	1067304595
Ca_Kabuli_NW_004515976.1_118613	Ca_kabuli_scaffold	118613	[A/G]	1067304596
Ca_Kabuli_NW_004515976.1_184646	Ca_kabuli_scaffold	184646	[A/G]	1067304597
Ca_Kabuli_NW_004515979.1_139569	Ca_kabuli_scaffold	139569	[A/C]	1067304598
Ca_Kabuli_NW_004515981.1_207045	Ca_kabuli_scaffold	207045	[A/T]	1067304599
Ca_Kabuli_NW_004515981.1_207048	Ca_kabuli_scaffold	207048	[T/C]	1067304600
Ca_Kabuli_NW_004515981.1_207094	Ca_kabuli_scaffold	207094	[G/T]	1067304601
Ca_Kabuli_NW_004515981.1_207105	Ca_kabuli_scaffold	207105	[T/C]	1067304602
Ca_Kabuli_NW_004515981.1_207107	Ca_kabuli_scaffold	207107	[A/G]	1067304603
Ca_Kabuli_NW_004515981.1_220119	Ca_kabuli_scaffold	220119	[T/C]	1067304604
Ca_Kabuli_NW_004515981.1_220160	Ca_kabuli_scaffold	220160	[T/A]	1067304605
Ca_Kabuli_NW_004515981.1_220166	Ca_kabuli_scaffold	220166	[T/A]	1067304606
Ca_Kabuli_NW_004515981.1_222163	Ca_kabuli_scaffold	222163	[A/G]	1067304607
Ca_Kabuli_NW_004515981.1_222173	Ca_kabuli_scaffold	222173	[T/A]	1067304608
Ca_Kabuli_NW_004515981.1_222175	Ca_kabuli_scaffold	222175	[T/C]	1067304609
Ca_Kabuli_NW_004515981.1_222176	Ca_kabuli_scaffold	222176	[T/G]	1067304610

SNP IDs	Chromosomes	Physical positions (bp)	SNPs	NCBI SNPdb accession IDs (NCBI_ss#)
Ca_Kabuli_NW_004515981.1_223137	Ca_kabuli_scaffold	223137	[A/T]	1067304611
Ca_Kabuli_NW_004515981.1_238192	Ca_kabuli_scaffold	238192	[A/C]	1067304612
Ca_Kabuli_NW_004515981.1_238193	Ca_kabuli_scaffold	238193	[G/T]	1067304613
Ca_Kabuli_NW_004515984.1_3401	Ca_kabuli_scaffold	3401	[T/A]	1067304614
Ca_Kabuli_NW_004515984.1_20980	Ca_kabuli_scaffold	20980	[T/G]	1067304615
Ca_Kabuli_NW_004515984.1_74806	Ca_kabuli_scaffold	74806	[T/G]	1067304616
Ca_Kabuli_NW_004515984.1_74828	Ca_kabuli_scaffold	74828	[A/G]	1067304617
Ca_Kabuli_NW_004515984.1_74833	Ca_kabuli_scaffold	74833	[C/G]	1067304618
Ca_Kabuli_NW_004515984.1_266729	Ca_kabuli_scaffold	266729	[A/C]	1067304619
Ca_Kabuli_NW_004515984.1_266741	Ca_kabuli_scaffold	266741	[A/C]	1067304620
Ca_Kabuli_NW_004515984.1_266761	Ca_kabuli_scaffold	266761	[A/G]	1067304621
Ca_Kabuli_NW_004515984.1_266820	Ca_kabuli_scaffold	266820	[C/G]	1067304622
Ca_Kabuli_NW_004515984.1_266822	Ca_kabuli_scaffold	266822	[T/C]	1067304623
Ca_Kabuli_NW_004515984.1_266847	Ca_kabuli_scaffold	266847	[A/G]	1067304624
Ca_Kabuli_NW_004515984.1_266848	Ca_kabuli_scaffold	266848	[A/C]	1067304625
Ca_Kabuli_NW_004515984.1_266852	Ca_kabuli_scaffold	266852	[T/C]	1067304626
Ca_Kabuli_NW_004515984.1_266866	Ca_kabuli_scaffold	266866	[A/C]	1067304627
Ca_Kabuli_NW_004515984.1_266871	Ca_kabuli_scaffold	266871	[A/C]	1067304628
Ca_Kabuli_NW_004515985.1_53632	Ca_kabuli_scaffold	53632	[G/T]	1067304629
Ca_Kabuli_NW_004515986.1_11292	Ca_kabuli_scaffold	11292	[C/T]	1067304630
Ca_Kabuli_NW_004515986.1_83402	Ca_kabuli_scaffold	83402	[A/C]	1067304631
Ca_Kabuli_NW_004515986.1_83406	Ca_kabuli_scaffold	83406	[A/C]	1067304632
Ca_Kabuli_NW_004515986.1_83449	Ca_kabuli_scaffold	83449	[A/G]	1067304633
Ca_Kabuli_NW_004515986.1_83503	Ca_kabuli_scaffold	83503	[A/G]	1067304634
Ca_Kabuli_NW_004515986.1_138914	Ca_kabuli_scaffold	138914	[T/G]	1067304635
Ca_Kabuli_NW_004515986.1_147568	Ca_kabuli_scaffold	147568	[T/G]	1067304636
Ca_Kabuli_NW_004515986.1_218699	Ca_kabuli_scaffold	218699	[A/C]	1067304637
Ca_Kabuli_NW_004515986.1_227149	Ca_kabuli_scaffold	227149	[T/C]	1067304638
Ca_Kabuli_NW_004515986.1_227166	Ca_kabuli_scaffold	227166	[T/C]	1067304639
Ca_Kabuli_NW_004515986.1_227169	Ca_kabuli_scaffold	227169	[A/G]	1067304640
Ca_Kabuli_NW_004515986.1_227192	Ca_kabuli_scaffold	227192	[G/A]	1067304641
Ca_Kabuli_NW_004515986.1_227203	Ca_kabuli_scaffold	227203	[A/G]	1067304642
Ca_Kabuli_NW_004515986.1_227205	Ca_kabuli_scaffold	227205	[A/G]	1067304643
Ca_Kabuli_NW_004515986.1_227213	Ca_kabuli_scaffold	227213	[T/C]	1067304644

SNP IDs	Chromosomes	Physical positions (bp)	SNPs	NCBI SNPdb accession IDs (NCBI_ss#)
Ca_Kabuli_NW_004515989.1_17488	Ca_kabuli_scaffold	17488	[A/C]	1067304645
Ca_Kabuli_NW_004515989.1_648290	Ca_kabuli_scaffold	648290	[A/G]	1067304646
Ca_Kabuli_NW_004515989.1_648351	Ca_kabuli_scaffold	648351	[T/G]	1067304647
Ca_Kabuli_NW_004515992.1_235181	Ca_kabuli_scaffold	235181	[T/A]	1067304648
Ca_Kabuli_NW_004515992.1_235191	Ca_kabuli_scaffold	235191	[T/G]	1067304649
Ca_Kabuli_NW_004515992.1_235227	Ca_kabuli_scaffold	235227	[A/T]	1067304650
Ca_Kabuli_NW_004515992.1_235362	Ca_kabuli_scaffold	235362	[T/A]	1067304651
Ca_Kabuli_NW_004515992.1_291235	Ca_kabuli_scaffold	291235	[A/C]	1067304652
Ca_Kabuli_NW_004515992.1_401898	Ca_kabuli_scaffold	401898	[T/A]	1067304653
Ca_Kabuli_NW_004515992.1_401904	Ca_kabuli_scaffold	401904	[T/C]	1067304654
Ca_Kabuli_NW_004515992.1_401905	Ca_kabuli_scaffold	401905	[A/T]	1067304655
Ca_Kabuli_NW_004515992.1_401923	Ca_kabuli_scaffold	401923	[T/G]	1067304656
Ca_Kabuli_NW_004515992.1_401947	Ca_kabuli_scaffold	401947	[T/C]	1067304657
Ca_Kabuli_NW_004515992.1_402658	Ca_kabuli_scaffold	402658	[A/T]	1067304658
Ca_Kabuli_NW_004515992.1_402663	Ca_kabuli_scaffold	402663	[A/C]	1067304659
Ca_Kabuli_NW_004515992.1_442094	Ca_kabuli_scaffold	442094	[T/C]	1067304660
Ca_Kabuli_NW_004515992.1_442114	Ca_kabuli_scaffold	442114	[A/G]	1067304661
Ca_Kabuli_NW_004515992.1_443299	Ca_kabuli_scaffold	443299	[A/C]	1067304662
Ca_Kabuli_NW_004515992.1_607428	Ca_kabuli_scaffold	607428	[T/A]	1067304663
Ca_Kabuli_NW_004515992.1_607596	Ca_kabuli_scaffold	607596	[T/A]	1067304664
Ca_Kabuli_NW_004515992.1_607657	Ca_kabuli_scaffold	607657	[A/G]	1067304665
Ca_Kabuli_NW_004515992.1_608668	Ca_kabuli_scaffold	608668	[A/G]	1067304666
Ca_Kabuli_NW_004515992.1_715934	Ca_kabuli_scaffold	715934	[A/C]	1067304667
Ca_Kabuli_NW_004515992.1_793782	Ca_kabuli_scaffold	793782	[A/G]	1067304668
Ca_Kabuli_NW_004515992.1_793807	Ca_kabuli_scaffold	793807	[A/G]	1067304669
Ca_Kabuli_NW_004515992.1_794403	Ca_kabuli_scaffold	794403	[T/A]	1067304670
Ca_Kabuli_NW_004515992.1_795669	Ca_kabuli_scaffold	795669	[C/A]	1067304671
Ca_Kabuli_NW_004515998.1_49143	Ca_kabuli_scaffold	49143	[T/C]	1067304672
Ca_Kabuli_NW_004515998.1_49173	Ca_kabuli_scaffold	49173	[A/G]	1067304673
Ca_Kabuli_NW_004515998.1_87361	Ca_kabuli_scaffold	87361	[G/T]	1067304674
Ca_Kabuli_NW_004515998.1_116793	Ca_kabuli_scaffold	116793	[G/T]	1067304675
Ca_Kabuli_NW_004515999.1_221770	Ca_kabuli_scaffold	221770	[C/A]	1067304676
Ca_Kabuli_NW_004515999.1_221778	Ca_kabuli_scaffold	221778	[G/A]	1067304677
Ca_Kabuli_NW_004515999.1_221789	Ca_kabuli_scaffold	221789	[T/C]	1067304678

SNP IDs	Chromosomes	Physical positions (bp)	SNPs	NCBI SNPdb accession IDs (NCBI_ss#)
Ca_Kabuli_NW_004515999.1_221792	Ca_kabuli_scaffold	221792	[C/A]	1067304679
Ca_Kabuli_NW_004516009.1_85858	Ca_kabuli_scaffold	85858	[C/A]	1067304680
Ca_Kabuli_NW_004516009.1_85866	Ca_kabuli_scaffold	85866	[C/T]	1067304681
Ca_Kabuli_NW_004516009.1_96389	Ca_kabuli_scaffold	96389	[T/G]	1067304682
Ca_Kabuli_NW_004516009.1_99047	Ca_kabuli_scaffold	99047	[G/T]	1067304683
Ca_Kabuli_NW_004516009.1_99050	Ca_kabuli_scaffold	99050	[C/G]	1067304684
Ca_Kabuli_NW_004516009.1_99059	Ca_kabuli_scaffold	99059	[G/T]	1067304685
Ca_Kabuli_NW_004516009.1_99065	Ca_kabuli_scaffold	99065	[A/G]	1067304686
Ca_Kabuli_NW_004516009.1_99068	Ca_kabuli_scaffold	99068	[G/T]	1067304687
Ca_Kabuli_NW_004516009.1_102713	Ca_kabuli_scaffold	102713	[C/T]	1067304688
Ca_Kabuli_NW_004516009.1_102725	Ca_kabuli_scaffold	102725	[T/A]	1067304689
Ca_Kabuli_NW_004516009.1_102731	Ca_kabuli_scaffold	102731	[C/T]	1067304690
Ca_Kabuli_NW_004516009.1_110278	Ca_kabuli_scaffold	110278	[T/C]	1067304691
Ca_Kabuli_NW_004516009.1_110299	Ca_kabuli_scaffold	110299	[A/G]	1067304692
Ca_Kabuli_NW_004516009.1_110301	Ca_kabuli_scaffold	110301	[T/C]	1067304693
Ca_Kabuli_NW_004516009.1_110753	Ca_kabuli_scaffold	110753	[G/A]	1067304694
Ca_Kabuli_NW_004516009.1_110757	Ca_kabuli_scaffold	110757	[T/C]	1067304695
Ca_Kabuli_NW_004516009.1_110759	Ca_kabuli_scaffold	110759	[C/T]	1067304696
Ca_Kabuli_NW_004516009.1_110765	Ca_kabuli_scaffold	110765	[C/T]	1067304697
Ca_Kabuli_NW_004516009.1_110772	Ca_kabuli_scaffold	110772	[G/T]	1067304698
Ca_Kabuli_NW_004516009.1_110785	Ca_kabuli_scaffold	110785	[C/T]	1067304699
Ca_Kabuli_NW_004516009.1_112053	Ca_kabuli_scaffold	112053	[A/C]	1067304700
Ca_Kabuli_NW_004516009.1_112071	Ca_kabuli_scaffold	112071	[A/T]	1067304701
Ca_Kabuli_NW_004516009.1_112079	Ca_kabuli_scaffold	112079	[A/G]	1067304702
Ca_Kabuli_NW_004516009.1_112373	Ca_kabuli_scaffold	112373	[A/G]	1067304703
Ca_Kabuli_NW_004516009.1_112384	Ca_kabuli_scaffold	112384	[A/C]	1067304704
Ca_Kabuli_NW_004516009.1_112432	Ca_kabuli_scaffold	112432	[G/A]	1067304705
Ca_Kabuli_NW_004516009.1_112433	Ca_kabuli_scaffold	112433	[G/A]	1067304706
Ca_Kabuli_NW_004516009.1_112434	Ca_kabuli_scaffold	112434	[C/T]	1067304707
Ca_Kabuli_NW_004516009.1_112479	Ca_kabuli_scaffold	112479	[A/G]	1067304708
Ca_Kabuli_NW_004516009.1_112488	Ca_kabuli_scaffold	112488	[A/G]	1067304709
Ca_Kabuli_NW_004516009.1_112495	Ca_kabuli_scaffold	112495	[T/C]	1067304710
Ca_Kabuli_NW_004516009.1_112497	Ca_kabuli_scaffold	112497	[T/C]	1067304711
Ca_Kabuli_NW_004516009.1_112585	Ca_kabuli_scaffold	112585	[A/C]	1067304712

SNP IDs	Chromosomes	Physical positions (bp)	SNPs	NCBI SNPdb accession IDs (NCBI_ss#)
Ca_Kabuli_NW_004516009.1_112607	Ca_kabuli_scaffold	112607	[A/G]	1067304713
Ca_Kabuli_NW_004516009.1_113780	Ca_kabuli_scaffold	113780	[T/A]	1067304714
Ca_Kabuli_NW_004516009.1_113823	Ca_kabuli_scaffold	113823	[A/C]	1067304715
Ca_Kabuli_NW_004516014.1_88419	Ca_kabuli_scaffold	88419	[A/G]	1067304716
Ca_Kabuli_NW_004516015.1_150202	Ca_kabuli_scaffold	150202	[G/A]	1067304717
Ca_Kabuli_NW_004516015.1_150215	Ca_kabuli_scaffold	150215	[G/A]	1067304718
Ca_Kabuli_NW_004516015.1_150232	Ca_kabuli_scaffold	150232	[G/A]	1067304719
Ca_Kabuli_NW_004516015.1_150237	Ca_kabuli_scaffold	150237	[C/T]	1067304720
Ca_Kabuli_NW_004516029.1_102993	Ca_kabuli_scaffold	102993	[A/G]	1067304721
Ca_Kabuli_NW_004516029.1_102998	Ca_kabuli_scaffold	102998	[T/G]	1067304722
Ca_Kabuli_NW_004516029.1_137196	Ca_kabuli_scaffold	137196	[G/T]	1067304723
Ca_Kabuli_NW_004516029.1_161754	Ca_kabuli_scaffold	161754	[T/C]	1067304724
Ca_Kabuli_NW_004516029.1_300921	Ca_kabuli_scaffold	300921	[A/C]	1067304725
Ca_Kabuli_NW_004516029.1_300944	Ca_kabuli_scaffold	300944	[T/C]	1067304726
Ca_Kabuli_NW_004516029.1_300957	Ca_kabuli_scaffold	300957	[T/G]	1067304727
Ca_Kabuli_NW_004516029.1_300960	Ca_kabuli_scaffold	300960	[C/T]	1067304728
Ca_Kabuli_NW_004516029.1_300966	Ca_kabuli_scaffold	300966	[A/T]	1067304729
Ca_Kabuli_NW_004516029.1_300967	Ca_kabuli_scaffold	300967	[G/T]	1067304730
Ca_Kabuli_NW_004516029.1_300972	Ca_kabuli_scaffold	300972	[G/T]	1067304731
Ca_Kabuli_NW_004516029.1_390787	Ca_kabuli_scaffold	390787	[A/C]	1067304732
Ca_Kabuli_NW_004516029.1_404045	Ca_kabuli_scaffold	404045	[T/A]	1067304733
Ca_Kabuli_NW_004516029.1_404364	Ca_kabuli_scaffold	404364	[T/C]	1067304734
Ca_Kabuli_NW_004516029.1_410447	Ca_kabuli_scaffold	410447	[A/T]	1067304735
Ca_Kabuli_NW_004516030.1_110316	Ca_kabuli_scaffold	110316	[T/C]	1067304736
Ca_Kabuli_NW_004516030.1_240474	Ca_kabuli_scaffold	240474	[A/T]	1067304737
Ca_Kabuli_NW_004516030.1_294438	Ca_kabuli_scaffold	294438	[G/A]	1067304738
Ca_Kabuli_NW_004516031.1_71998	Ca_kabuli_scaffold	71998	[A/T]	1067304739
Ca_Kabuli_NW_004516031.1_72041	Ca_kabuli_scaffold	72041	[A/C]	1067304740
Ca_Kabuli_NW_004516031.1_110896	Ca_kabuli_scaffold	110896	[C/A]	1067304741
Ca_Kabuli_NW_004516031.1_110898	Ca_kabuli_scaffold	110898	[A/T]	1067304742
Ca_Kabuli_NW_004516031.1_110909	Ca_kabuli_scaffold	110909	[A/T]	1067304743
Ca_Kabuli_NW_004516043.1_62540	Ca_kabuli_scaffold	62540	[A/G]	1067304744
Ca_Kabuli_NW_004516043.1_93984	Ca_kabuli_scaffold	93984	[G/A]	1067304745
Ca_Kabuli_NW_004516048.1_87982	Ca_kabuli_scaffold	87982	[T/G]	1067304746

SNP IDs	Chromosomes	Physical positions (bp)	SNPs	NCBI SNPdb accession IDs (NCBI_ss#)
Ca_Kabuli_NW_004516050.1_343389	Ca_kabuli_scaffold	343389	[A/G]	1067304747
Ca_Kabuli_NW_004516050.1_343407	Ca_kabuli_scaffold	343407	[A/T]	1067304748
Ca_Kabuli_NW_004516050.1_343411	Ca_kabuli_scaffold	343411	[A/G]	1067304749
Ca_Kabuli_NW_004516058.1_31412	Ca_kabuli_scaffold	31412	[A/G]	1067304750
Ca_Kabuli_NW_004516058.1_31470	Ca_kabuli_scaffold	31470	[T/C]	1067304751
Ca_Kabuli_NW_004516058.1_31480	Ca_kabuli_scaffold	31480	[A/C]	1067304752
Ca_Kabuli_NW_004516058.1_107549	Ca_kabuli_scaffold	107549	[A/G]	1067304753
Ca_Kabuli_NW_004516058.1_107605	Ca_kabuli_scaffold	107605	[T/C]	1067304754
Ca_Kabuli_NW_004516058.1_107612	Ca_kabuli_scaffold	107612	[T/A]	1067304755
Ca_Kabuli_NW_004516058.1_107618	Ca_kabuli_scaffold	107618	[T/C]	1067304756
Ca_Kabuli_NW_004516058.1_107623	Ca_kabuli_scaffold	107623	[G/A]	1067304757
Ca_Kabuli_NW_004516058.1_107625	Ca_kabuli_scaffold	107625	[T/C]	1067304758
Ca_Kabuli_NW_004516059.1_190652	Ca_kabuli_scaffold	190652	[T/C]	1067304759
Ca_Kabuli_NW_004516061.1_7306	Ca_kabuli_scaffold	7306	[T/G]	1067304760
Ca_Kabuli_NW_004516061.1_44096	Ca_kabuli_scaffold	44096	[T/C]	1067304761
Ca_Kabuli_NW_004516061.1_48008	Ca_kabuli_scaffold	48008	[A/G]	1067304762
Ca_Kabuli_NW_004516061.1_145557	Ca_kabuli_scaffold	145557	[A/C]	1067304763
Ca_Kabuli_NW_004516061.1_145559	Ca_kabuli_scaffold	145559	[C/G]	1067304764
Ca_Kabuli_NW_004516061.1_145560	Ca_kabuli_scaffold	145560	[T/G]	1067304765
Ca_Kabuli_NW_004516061.1_572432	Ca_kabuli_scaffold	572432	[A/G]	1067304766
Ca_Kabuli_NW_004516061.1_572434	Ca_kabuli_scaffold	572434	[A/G]	1067304767
Ca_Kabuli_NW_004516061.1_572444	Ca_kabuli_scaffold	572444	[A/G]	1067304768
Ca_Kabuli_NW_004516061.1_572459	Ca_kabuli_scaffold	572459	[T/C]	1067304769
Ca_Kabuli_NW_004516061.1_614966	Ca_kabuli_scaffold	614966	[T/C]	1067304770
Ca_Kabuli_NW_004516061.1_614968	Ca_kabuli_scaffold	614968	[T/C]	1067304771
Ca_Kabuli_NW_004516061.1_614970	Ca_kabuli_scaffold	614970	[G/T]	1067304772
Ca_Kabuli_NW_004516062.1_18115	Ca_kabuli_scaffold	18115	[A/G]	1067304773
Ca_Kabuli_NW_004516063.1_176958	Ca_kabuli_scaffold	176958	[A/T]	1067304774
Ca_Kabuli_NW_004516063.1_214380	Ca_kabuli_scaffold	214380	[A/C]	1067304775
Ca_Kabuli_NW_004516063.1_214409	Ca_kabuli_scaffold	214409	[T/A]	1067304776
Ca_Kabuli_NW_004516063.1_239854	Ca_kabuli_scaffold	239854	[A/G]	1067304777
Ca_Kabuli_NW_004516063.1_245029	Ca_kabuli_scaffold	245029	[T/A]	1067304778
Ca_Kabuli_NW_004516063.1_245034	Ca_kabuli_scaffold	245034	[C/T]	1067304779
Ca_Kabuli_NW_004516063.1_245036	Ca_kabuli_scaffold	245036	[T/A]	1067304780

SNP IDs	Chromosomes	Physical positions (bp)	SNPs	NCBI SNPdb accession IDs (NCBI_ss#)
Ca_Kabuli_NW_004516070.1_53912	Ca_kabuli_scaffold	53912	[T/A]	1067304781
Ca_Kabuli_NW_004516070.1_53913	Ca_kabuli_scaffold	53913	[C/A]	1067304782
Ca_Kabuli_NW_004516070.1_53914	Ca_kabuli_scaffold	53914	[T/G]	1067304783
Ca_Kabuli_NW_004516070.1_142907	Ca_kabuli_scaffold	142907	[A/C]	1067304784
Ca_Kabuli_NW_004516070.1_176821	Ca_kabuli_scaffold	176821	[T/A]	1067304785
Ca_Kabuli_NW_004516070.1_183054	Ca_kabuli_scaffold	183054	[T/C]	1067304786
Ca_Kabuli_NW_004516070.1_183063	Ca_kabuli_scaffold	183063	[G/A]	1067304787
Ca_Kabuli_NW_004516070.1_191740	Ca_kabuli_scaffold	191740	[T/C]	1067304788
Ca_Kabuli_NW_004516070.1_191748	Ca_kabuli_scaffold	191748	[T/A]	1067304789
Ca_Kabuli_NW_004516076.1_159222	Ca_kabuli_scaffold	159222	[C/A]	1067304790
Ca_Kabuli_NW_004516076.1_159223	Ca_kabuli_scaffold	159223	[T/G]	1067304791
Ca_Kabuli_NW_004516076.1_311139	Ca_kabuli_scaffold	311139	[G/T]	1067304792
Ca_Kabuli_NW_004516076.1_350644	Ca_kabuli_scaffold	350644	[T/G]	1067304793
Ca_Kabuli_NW_004516076.1_350659	Ca_kabuli_scaffold	350659	[A/C]	1067304794
Ca_Kabuli_NW_004516076.1_472607	Ca_kabuli_scaffold	472607	[A/T]	1067304795
Ca_Kabuli_NW_004516076.1_472614	Ca_kabuli_scaffold	472614	[T/A]	1067304796
Ca_Kabuli_NW_004516078.1_156608	Ca_kabuli_scaffold	156608	[T/G]	1067304797
Ca_Kabuli_NW_004516078.1_156624	Ca_kabuli_scaffold	156624	[T/G]	1067304798
Ca_Kabuli_NW_004516078.1_156630	Ca_kabuli_scaffold	156630	[T/G]	1067304799
Ca_Kabuli_NW_004516088.1_136995	Ca_kabuli_scaffold	136995	[C/A]	1067304800
Ca_Kabuli_NW_004516088.1_265998	Ca_kabuli_scaffold	265998	[A/C]	1067304801
Ca_Kabuli_NW_004516088.1_309725	Ca_kabuli_scaffold	309725	[C/T]	1067304802
Ca_Kabuli_NW_004516088.1_582040	Ca_kabuli_scaffold	582040	[T/G]	1067304803
Ca_Kabuli_NW_004516088.1_582047	Ca_kabuli_scaffold	582047	[A/C]	1067304804
Ca_Kabuli_NW_004516088.1_608617	Ca_kabuli_scaffold	608617	[T/G]	1067304805
Ca_Kabuli_NW_004516088.1_608619	Ca_kabuli_scaffold	608619	[G/T]	1067304806
Ca_Kabuli_NW_004516088.1_608620	Ca_kabuli_scaffold	608620	[C/G]	1067304807
Ca_Kabuli_NW_004516088.1_608623	Ca_kabuli_scaffold	608623	[T/G]	1067304808
Ca_Kabuli_NW_004516088.1_608627	Ca_kabuli_scaffold	608627	[A/T]	1067304809
Ca_Kabuli_NW_004516088.1_608630	Ca_kabuli_scaffold	608630	[G/A]	1067304810
Ca_Kabuli_NW_004516088.1_608632	Ca_kabuli_scaffold	608632	[A/C]	1067304811
Ca_Kabuli_NW_004516088.1_608633	Ca_kabuli_scaffold	608633	[C/A]	1067304812
Ca_Kabuli_NW_004516088.1_635370	Ca_kabuli_scaffold	635370	[G/T]	1067304813
Ca_Kabuli_NW_004516088.1_637666	Ca_kabuli_scaffold	637666	[A/T]	1067304814

SNP IDs	Chromosomes	Physical positions (bp)	SNPs	NCBI SNPdb accession IDs (NCBI_ss#)
Ca_Kabuli_NW_004516088.1_652539	Ca_kabuli_scaffold	652539	[A/C]	1067304815
Ca_Kabuli_NW_004516088.1_662223	Ca_kabuli_scaffold	662223	[A/T]	1067304816
Ca_Kabuli_NW_004516088.1_770252	Ca_kabuli_scaffold	770252	[A/G]	1067304817
Ca_Kabuli_NW_004516095.1_149789	Ca_kabuli_scaffold	149789	[G/T]	1067304818
Ca_Kabuli_NW_004516095.1_156744	Ca_kabuli_scaffold	156744	[A/C]	1067304819
Ca_Kabuli_NW_004516095.1_156786	Ca_kabuli_scaffold	156786	[T/A]	1067304820
Ca_Kabuli_NW_004516095.1_238439	Ca_kabuli_scaffold	238439	[T/G]	1067304821
Ca_Kabuli_NW_004516095.1_541994	Ca_kabuli_scaffold	541994	[A/T]	1067304822
Ca_Kabuli_NW_004516095.1_542034	Ca_kabuli_scaffold	542034	[A/G]	1067304823
Ca_Kabuli_NW_004516095.1_542039	Ca_kabuli_scaffold	542039	[A/G]	1067304824
Ca_Kabuli_NW_004516095.1_542055	Ca_kabuli_scaffold	542055	[T/C]	1067304825
Ca_Kabuli_NW_004516097.1_78842	Ca_kabuli_scaffold	78842	[G/A]	1067304826
Ca_Kabuli_NW_004516097.1_78864	Ca_kabuli_scaffold	78864	[C/T]	1067304827
Ca_Kabuli_NW_004516097.1_78871	Ca_kabuli_scaffold	78871	[C/G]	1067304828
Ca_Kabuli_NW_004516097.1_78883	Ca_kabuli_scaffold	78883	[T/G]	1067304829
Ca_Kabuli_NW_004516099.1_220971	Ca_kabuli_scaffold	220971	[G/A]	1067304830
Ca_Kabuli_NW_004516109.1_22949	Ca_kabuli_scaffold	22949	[A/C]	1067304831
Ca_Kabuli_NW_004516109.1_247526	Ca_kabuli_scaffold	247526	[A/C]	1067304832
Ca_Kabuli_NW_004516114.1_137132	Ca_kabuli_scaffold	137132	[C/A]	1067304833
Ca_Kabuli_NW_004516114.1_137133	Ca_kabuli_scaffold	137133	[A/G]	1067304834
Ca_Kabuli_NW_004516114.1_137134	Ca_kabuli_scaffold	137134	[T/A]	1067304835
Ca_Kabuli_NW_004516114.1_138239	Ca_kabuli_scaffold	138239	[A/C]	1067304836
Ca_Kabuli_NW_004516117.1_183276	Ca_kabuli_scaffold	183276	[C/T]	1067304837
Ca_Kabuli_NW_004516119.1_52611	Ca_kabuli_scaffold	52611	[T/G]	1067304838
Ca_Kabuli_NW_004516120.1_130103	Ca_kabuli_scaffold	130103	[T/C]	1067304839
Ca_Kabuli_NW_004516120.1_196956	Ca_kabuli_scaffold	196956	[T/G]	1067304840
Ca_Kabuli_NW_004516120.1_370374	Ca_kabuli_scaffold	370374	[C/T]	1067304841
Ca_Kabuli_NW_004516120.1_370559	Ca_kabuli_scaffold	370559	[C/G]	1067304842
Ca_Kabuli_NW_004516120.1_370635	Ca_kabuli_scaffold	370635	[A/G]	1067304843
Ca_Kabuli_NW_004516120.1_370637	Ca_kabuli_scaffold	370637	[T/C]	1067304844
Ca_Kabuli_NW_004516120.1_370639	Ca_kabuli_scaffold	370639	[T/A]	1067304845
Ca_Kabuli_NW_004516120.1_370650	Ca_kabuli_scaffold	370650	[A/C]	1067304846
Ca_Kabuli_NW_004516123.1_25019	Ca_kabuli_scaffold	25019	[A/G]	1067304847
Ca_Kabuli_NW_004516126.1_106230	Ca_kabuli_scaffold	106230	[A/G]	1067304848

SNP IDs	Chromosomes	Physical positions (bp)	SNPs	NCBI SNPdb accession IDs (NCBI_ss#)
Ca_Kabuli_NW_004516126.1_362149	Ca_kabuli_scaffold	362149	[G/A]	1067304849
Ca_Kabuli_NW_004516126.1_362158	Ca_kabuli_scaffold	362158	[A/G]	1067304850
Ca_Kabuli_NW_004516126.1_362178	Ca_kabuli_scaffold	362178	[A/C]	1067304851
Ca_Kabuli_NW_004516126.1_372294	Ca_kabuli_scaffold	372294	[T/C]	1067304852
Ca_Kabuli_NW_004516126.1_372313	Ca_kabuli_scaffold	372313	[T/G]	1067304853
Ca_Kabuli_NW_004516126.1_372318	Ca_kabuli_scaffold	372318	[A/G]	1067304854
Ca_Kabuli_NW_004516126.1_372982	Ca_kabuli_scaffold	372982	[A/C]	1067304855
Ca_Kabuli_NW_004516141.1_2830	Ca_kabuli_scaffold	2830	[G/A]	1067304856
Ca_Kabuli_NW_004516141.1_382255	Ca_kabuli_scaffold	382255	[T/A]	1067304857
Ca_Kabuli_NW_004516141.1_382388	Ca_kabuli_scaffold	382388	[G/A]	1067304858
Ca_Kabuli_NW_004516142.1_7429	Ca_kabuli_scaffold	7429	[A/T]	1067304859
Ca_Kabuli_NW_004516145.1_72913	Ca_kabuli_scaffold	72913	[G/A]	1067304860
Ca_Kabuli_NW_004516145.1_72918	Ca_kabuli_scaffold	72918	[C/T]	1067304861
Ca_Kabuli_NW_004516145.1_72928	Ca_kabuli_scaffold	72928	[G/C]	1067304862
Ca_Kabuli_NW_004516145.1_72939	Ca_kabuli_scaffold	72939	[A/C]	1067304863
Ca_Kabuli_NW_004516145.1_72953	Ca_kabuli_scaffold	72953	[A/T]	1067304864
Ca_Kabuli_NW_004516152.1_16087	Ca_kabuli_scaffold	16087	[G/A]	1067304865
Ca_Kabuli_NW_004516152.1_16095	Ca_kabuli_scaffold	16095	[T/G]	1067304866
Ca_Kabuli_NW_004516155.1_14191	Ca_kabuli_scaffold	14191	[A/G]	1067304867
Ca_Kabuli_NW_004516155.1_14228	Ca_kabuli_scaffold	14228	[C/T]	1067304868
Ca_Kabuli_NW_004516156.1_108823	Ca_kabuli_scaffold	108823	[G/A]	1067304869
Ca_Kabuli_NW_004516156.1_108825	Ca_kabuli_scaffold	108825	[C/T]	1067304870
Ca_Kabuli_NW_004516157.1_31290	Ca_kabuli_scaffold	31290	[G/A]	1067304871
Ca_Kabuli_NW_004516157.1_69442	Ca_kabuli_scaffold	69442	[A/G]	1067304872
Ca_Kabuli_NW_004516157.1_69445	Ca_kabuli_scaffold	69445	[A/G]	1067304873
Ca_Kabuli_NW_004516157.1_231759	Ca_kabuli_scaffold	231759	[A/C]	1067304874
Ca_Kabuli_NW_004516157.1_231827	Ca_kabuli_scaffold	231827	[C/T]	1067304875
Ca_Kabuli_NW_004516159.1_223453	Ca_kabuli_scaffold	223453	[A/G]	1067304876
Ca_Kabuli_NW_004516159.1_279987	Ca_kabuli_scaffold	279987	[A/G]	1067304877
Ca_Kabuli_NW_004516159.1_356052	Ca_kabuli_scaffold	356052	[G/T]	1067304878
Ca_Kabuli_NW_004516159.1_356092	Ca_kabuli_scaffold	356092	[A/G]	1067304879
Ca_Kabuli_NW_004516159.1_356109	Ca_kabuli_scaffold	356109	[T/C]	1067304880
Ca_Kabuli_NW_004516160.1_41384	Ca_kabuli_scaffold	41384	[G/T]	1067304881
Ca_Kabuli_NW_004516162.1_3037	Ca_kabuli_scaffold	3037	[G/T]	1067304882

SNP IDs	Chromosomes	Physical positions (bp)	SNPs	NCBI SNPdb accession IDs (NCBI_ss#)
Ca_Kabuli_NW_004516162.1_3055	Ca_kabuli_scaffold	3055	[A/G]	1067304883
Ca_Kabuli_NW_004516165.1_102949	Ca_kabuli_scaffold	102949	[C/A]	1067304884
Ca_Kabuli_NW_004516165.1_259304	Ca_kabuli_scaffold	259304	[T/G]	1067304885
Ca_Kabuli_NW_004516165.1_259396	Ca_kabuli_scaffold	259396	[G/T]	1067304886
Ca_Kabuli_NW_004516169.1_360756	Ca_kabuli_scaffold	360756	[T/C]	1067304887
Ca_Kabuli_NW_004516171.1_52221	Ca_kabuli_scaffold	52221	[A/C]	1067304888
Ca_Kabuli_NW_004516179.1_110755	Ca_kabuli_scaffold	110755	[T/A]	1067304889
Ca_Kabuli_NW_004516179.1_110758	Ca_kabuli_scaffold	110758	[A/G]	1067304890
Ca_Kabuli_NW_004516179.1_110781	Ca_kabuli_scaffold	110781	[A/G]	1067304891
Ca_Kabuli_NW_004516179.1_153720	Ca_kabuli_scaffold	153720	[A/T]	1067304892
Ca_Kabuli_NW_004516182.1_129263	Ca_kabuli_scaffold	129263	[A/G]	1067304893
Ca_Kabuli_NW_004516182.1_129265	Ca_kabuli_scaffold	129265	[G/T]	1067304894
Ca_Kabuli_NW_004516185.1_19878	Ca_kabuli_scaffold	19878	[A/C]	1067304895
Ca_Kabuli_NW_004516185.1_19904	Ca_kabuli_scaffold	19904	[A/G]	1067304896
Ca_Kabuli_NW_004516185.1_29415	Ca_kabuli_scaffold	29415	[T/G]	1067304897
Ca_Kabuli_NW_004516185.1_29432	Ca_kabuli_scaffold	29432	[A/C]	1067304898
Ca_Kabuli_NW_004516185.1_81392	Ca_kabuli_scaffold	81392	[T/G]	1067304899
Ca_Kabuli_NW_004516185.1_81402	Ca_kabuli_scaffold	81402	[T/G]	1067304900
Ca_Kabuli_NW_004516185.1_81445	Ca_kabuli_scaffold	81445	[A/G]	1067304901
Ca_Kabuli_NW_004516186.1_34684	Ca_kabuli_scaffold	34684	[G/A]	1067304902
Ca_Kabuli_NW_004516186.1_34700	Ca_kabuli_scaffold	34700	[A/C]	1067304903
Ca_Kabuli_NW_004516186.1_34708	Ca_kabuli_scaffold	34708	[G/T]	1067304904
Ca_Kabuli_NW_004516186.1_197437	Ca_kabuli_scaffold	197437	[G/T]	1067304905
Ca_Kabuli_NW_004516186.1_197499	Ca_kabuli_scaffold	197499	[A/T]	1067304906
Ca_Kabuli_NW_004516186.1_197525	Ca_kabuli_scaffold	197525	[G/A]	1067304907
Ca_Kabuli_NW_004516186.1_197537	Ca_kabuli_scaffold	197537	[T/A]	1067304908
Ca_Kabuli_NW_004516186.1_246201	Ca_kabuli_scaffold	246201	[A/C]	1067304909
Ca_Kabuli_NW_004516186.1_396019	Ca_kabuli_scaffold	396019	[T/G]	1067304910
Ca_Kabuli_NW_004516186.1_465704	Ca_kabuli_scaffold	465704	[T/C]	1067304911
Ca_Kabuli_NW_004516186.1_465714	Ca_kabuli_scaffold	465714	[G/T]	1067304912
Ca_Kabuli_NW_004516186.1_465722	Ca_kabuli_scaffold	465722	[G/T]	1067304913
Ca_Kabuli_NW_004516211.1_28172	Ca_kabuli_scaffold	28172	[T/G]	1067304914
Ca_Kabuli_NW_004516211.1_67951	Ca_kabuli_scaffold	67951	[A/T]	1067304915
Ca_Kabuli_NW_004516211.1_67961	Ca_kabuli_scaffold	67961	[C/T]	1067304916

SNP IDs	Chromosomes	Physical positions (bp)	SNPs	NCBI SNPdb accession IDs (NCBI_ss#)
Ca_Kabuli_NW_004516211.1_68001	Ca_kabuli_scaffold	68001	[A/T]	1067304917
Ca_Kabuli_NW_004516211.1_68328	Ca_kabuli_scaffold	68328	[G/A]	1067304918
Ca_Kabuli_NW_004516211.1_78956	Ca_kabuli_scaffold	78956	[G/A]	1067304919
Ca_Kabuli_NW_004516211.1_129326	Ca_kabuli_scaffold	129326	[G/C]	1067304920
Ca_Kabuli_NW_004516211.1_138081	Ca_kabuli_scaffold	138081	[A/C]	1067304921
Ca_Kabuli_NW_004516211.1_138401	Ca_kabuli_scaffold	138401	[A/C]	1067304922
Ca_Kabuli_NW_004516211.1_144585	Ca_kabuli_scaffold	144585	[A/C]	1067304923
Ca_Kabuli_NW_004516211.1_174105	Ca_kabuli_scaffold	174105	[G/T]	1067304924
Ca_Kabuli_NW_004516211.1_185332	Ca_kabuli_scaffold	185332	[T/G]	1067304925
Ca_Kabuli_NW_004516211.1_185425	Ca_kabuli_scaffold	185425	[T/A]	1067304926
Ca_Kabuli_NW_004516211.1_205964	Ca_kabuli_scaffold	205964	[A/C]	1067304927
Ca_Kabuli_NW_004516211.1_206579	Ca_kabuli_scaffold	206579	[A/C]	1067304928
Ca_Kabuli_NW_004516211.1_206672	Ca_kabuli_scaffold	206672	[A/G]	1067304929
Ca_Kabuli_NW_004516212.1_100282	Ca_kabuli_scaffold	100282	[T/G]	1067304930
Ca_Kabuli_NW_004516212.1_100329	Ca_kabuli_scaffold	100329	[A/T]	1067304931
Ca_Kabuli_NW_004516212.1_100375	Ca_kabuli_scaffold	100375	[T/C]	1067304932
Ca_Kabuli_NW_004516212.1_100457	Ca_kabuli_scaffold	100457	[A/G]	1067304933
Ca_Kabuli_NW_004516212.1_107969	Ca_kabuli_scaffold	107969	[T/G]	1067304934
Ca_Kabuli_NW_004516212.1_108825	Ca_kabuli_scaffold	108825	[T/A]	1067304935
Ca_Kabuli_NW_004516212.1_108908	Ca_kabuli_scaffold	108908	[G/T]	1067304936
Ca_Kabuli_NW_004516212.1_111061	Ca_kabuli_scaffold	111061	[T/C]	1067304937
Ca_Kabuli_NW_004516212.1_111084	Ca_kabuli_scaffold	111084	[A/C]	1067304938
Ca_Kabuli_NW_004516213.1_39870	Ca_kabuli_scaffold	39870	[A/C]	1067304939
Ca_Kabuli_NW_004516213.1_39934	Ca_kabuli_scaffold	39934	[T/G]	1067304940
Ca_Kabuli_NW_004516213.1_39943	Ca_kabuli_scaffold	39943	[C/T]	1067304941
Ca_Kabuli_NW_004516213.1_86055	Ca_kabuli_scaffold	86055	[A/C]	1067304942
Ca_Kabuli_NW_004516213.1_86085	Ca_kabuli_scaffold	86085	[T/C]	1067304943
Ca_Kabuli_NW_004516213.1_90401	Ca_kabuli_scaffold	90401	[C/A]	1067304944
Ca_Kabuli_NW_004516213.1_90407	Ca_kabuli_scaffold	90407	[A/T]	1067304945
Ca_Kabuli_NW_004516213.1_90416	Ca_kabuli_scaffold	90416	[C/A]	1067304946
Ca_Kabuli_NW_004516213.1_155289	Ca_kabuli_scaffold	155289	[G/A]	1067304947
Ca_Kabuli_NW_004516227.1_295823	Ca_kabuli_scaffold	295823	[C/T]	1067304948
Ca_Kabuli_NW_004516230.1_71697	Ca_kabuli_scaffold	71697	[T/G]	1067304949
Ca_Kabuli_NW_004516231.1_131705	Ca_kabuli_scaffold	131705	[A/G]	1067304950

SNP IDs	Chromosomes	Physical positions (bp)	SNPs	NCBI SNPdb accession IDs (NCBI_ss#)
Ca_Kabuli_NW_004516234.1_188769	Ca_kabuli_scaffold	188769	[C/T]	1067304951
Ca_Kabuli_NW_004516234.1_265247	Ca_kabuli_scaffold	265247	[A/C]	1067304952
Ca_Kabuli_NW_004516235.1_51703	Ca_kabuli_scaffold	51703	[C/G]	1067304953
Ca_Kabuli_NW_004516235.1_52687	Ca_kabuli_scaffold	52687	[C/A]	1067304954
Ca_Kabuli_NW_004516235.1_52688	Ca_kabuli_scaffold	52688	[T/G]	1067304955
Ca_Kabuli_NW_004516235.1_52689	Ca_kabuli_scaffold	52689	[T/A]	1067304956
Ca_Kabuli_NW_004516235.1_52707	Ca_kabuli_scaffold	52707	[A/G]	1067304957
Ca_Kabuli_NW_004516235.1_62660	Ca_kabuli_scaffold	62660	[C/T]	1067304958
Ca_Kabuli_NW_004516240.1_23282	Ca_kabuli_scaffold	23282	[A/C]	1067304959
Ca_Kabuli_NW_004516240.1_358416	Ca_kabuli_scaffold	358416	[T/C]	1067304960
Ca_Kabuli_NW_004516240.1_358430	Ca_kabuli_scaffold	358430	[T/C]	1067304961
Ca_Kabuli_NW_004516243.1_62868	Ca_kabuli_scaffold	62868	[A/G]	1067304962
Ca_Kabuli_NW_004516243.1_69495	Ca_kabuli_scaffold	69495	[G/T]	1067304963
Ca_Kabuli_NW_004516243.1_114062	Ca_kabuli_scaffold	114062	[A/G]	1067304964
Ca_Kabuli_NW_004516244.1_26108	Ca_kabuli_scaffold	26108	[G/T]	1067304965
Ca_Kabuli_NW_004516244.1_26113	Ca_kabuli_scaffold	26113	[C/T]	1067304966
Ca_Kabuli_NW_004516244.1_26121	Ca_kabuli_scaffold	26121	[A/C]	1067304967
Ca_Kabuli_NW_004516244.1_26134	Ca_kabuli_scaffold	26134	[C/A]	1067304968
Ca_Kabuli_NW_004516244.1_52694	Ca_kabuli_scaffold	52694	[A/G]	1067304969
Ca_Kabuli_NW_004516244.1_52725	Ca_kabuli_scaffold	52725	[T/C]	1067304970
Ca_Kabuli_NW_004516244.1_52740	Ca_kabuli_scaffold	52740	[T/C]	1067304971
Ca_Kabuli_NW_004516244.1_138204	Ca_kabuli_scaffold	138204	[T/G]	1067304972
Ca_Kabuli_NW_004516244.1_144885	Ca_kabuli_scaffold	144885	[C/T]	1067304973
Ca_Kabuli_NW_004516244.1_161061	Ca_kabuli_scaffold	161061	[T/C]	1067304974
Ca_Kabuli_NW_004516251.1_18559	Ca_kabuli_scaffold	18559	[T/A]	1067304975
Ca_Kabuli_NW_004516251.1_40849	Ca_kabuli_scaffold	40849	[A/C]	1067304976
Ca_Kabuli_NW_004516257.1_15425	Ca_kabuli_scaffold	15425	[A/G]	1067304977
Ca_Kabuli_NW_004516257.1_15429	Ca_kabuli_scaffold	15429	[C/T]	1067304978
Ca_Kabuli_NW_004516264.1_11776	Ca_kabuli_scaffold	11776	[C/T]	1067304979
Ca_Kabuli_NW_004516264.1_11787	Ca_kabuli_scaffold	11787	[G/A]	1067304980
Ca_Kabuli_NW_004516264.1_11819	Ca_kabuli_scaffold	11819	[C/T]	1067304981
Ca_Kabuli_NW_004516265.1_165122	Ca_kabuli_scaffold	165122	[T/C]	1067304982
Ca_Kabuli_NW_004516266.1_81377	Ca_kabuli_scaffold	81377	[A/G]	1067304983
Ca_Kabuli_NW_004516267.1_192902	Ca_kabuli_scaffold	192902	[T/G]	1067304984

SNP IDs	Chromosomes	Physical positions (bp)	SNPs	NCBI SNPdb accession IDs (NCBI_ss#)
Ca_Kabuli_NW_004516267.1_205028	Ca_kabuli_scaffold	205028	[T/C]	1067304985
Ca_Kabuli_NW_004516267.1_205033	Ca_kabuli_scaffold	205033	[T/G]	1067304986
Ca_Kabuli_NW_004516267.1_205958	Ca_kabuli_scaffold	205958	[T/A]	1067304987
Ca_Kabuli_NW_004516267.1_206008	Ca_kabuli_scaffold	206008	[A/T]	1067304988
Ca_Kabuli_NW_004516267.1_206040	Ca_kabuli_scaffold	206040	[A/G]	1067304989
Ca_Kabuli_NW_004516267.1_206058	Ca_kabuli_scaffold	206058	[A/G]	1067304990
Ca_Kabuli_NW_004516267.1_206060	Ca_kabuli_scaffold	206060	[T/G]	1067304991
Ca_Kabuli_NW_004516267.1_275489	Ca_kabuli_scaffold	275489	[G/T]	1067304992
Ca_Kabuli_NW_004516267.1_275493	Ca_kabuli_scaffold	275493	[A/T]	1067304993
Ca_Kabuli_NW_004516267.1_295864	Ca_kabuli_scaffold	295864	[G/T]	1067304994
Ca_Kabuli_NW_004516267.1_453871	Ca_kabuli_scaffold	453871	[T/C]	1067304995
Ca_Kabuli_NW_004516267.1_466233	Ca_kabuli_scaffold	466233	[A/G]	1067304996
Ca_Kabuli_NW_004516267.1_466262	Ca_kabuli_scaffold	466262	[A/T]	1067304997
Ca_Kabuli_NW_004516267.1_466354	Ca_kabuli_scaffold	466354	[A/G]	1067304998
Ca_Kabuli_NW_004516267.1_471762	Ca_kabuli_scaffold	471762	[A/C]	1067304999
Ca_Kabuli_NW_004516267.1_471801	Ca_kabuli_scaffold	471801	[C/G]	1067305000
Ca_Kabuli_NW_004516267.1_471808	Ca_kabuli_scaffold	471808	[A/T]	1067305001
Ca_Kabuli_NW_004516270.1_317391	Ca_kabuli_scaffold	317391	[G/A]	1067305002
Ca_Kabuli_NW_004516270.1_456454	Ca_kabuli_scaffold	456454	[G/A]	1067305003
Ca_Kabuli_NW_004516277.1_235785	Ca_kabuli_scaffold	235785	[A/G]	1067305004
Ca_Kabuli_NW_004516280.1_129166	Ca_kabuli_scaffold	129166	[T/C]	1067305005
Ca_Kabuli_NW_004516280.1_134737	Ca_kabuli_scaffold	134737	[A/C]	1067305006
Ca_Kabuli_NW_004516284.1_212420	Ca_kabuli_scaffold	212420	[A/C]	1067305007
Ca_Kabuli_NW_004516284.1_212423	Ca_kabuli_scaffold	212423	[A/C]	1067305008
Ca_Kabuli_NW_004516288.1_21076	Ca_kabuli_scaffold	21076	[T/C]	1067305009
Ca_Kabuli_NW_004516288.1_21087	Ca_kabuli_scaffold	21087	[T/G]	1067305010
Ca_Kabuli_NW_004516293.1_42329	Ca_kabuli_scaffold	42329	[T/C]	1067305011
Ca_Kabuli_NW_004516296.1_20765	Ca_kabuli_scaffold	20765	[G/T]	1067305012
Ca_Kabuli_NW_004516296.1_21718	Ca_kabuli_scaffold	21718	[T/C]	1067305013
Ca_Kabuli_NW_004516296.1_21742	Ca_kabuli_scaffold	21742	[A/T]	1067305014
Ca_Kabuli_NW_004516296.1_21951	Ca_kabuli_scaffold	21951	[T/G]	1067305015
Ca_Kabuli_NW_004516296.1_22585	Ca_kabuli_scaffold	22585	[T/G]	1067305016
Ca_Kabuli_NW_004516296.1_22590	Ca_kabuli_scaffold	22590	[T/G]	1067305017
Ca_Kabuli_NW_004516296.1_22732	Ca_kabuli_scaffold	22732	[G/T]	1067305018

SNP IDs	Chromosomes	Physical positions (bp)	SNPs	NCBI SNPdb accession IDs (NCBI_ss#)
Ca_Kabuli_NW_004516296.1_23613	Ca_kabuli_scaffold	23613	[A/T]	1067305019
Ca_Kabuli_NW_004516296.1_23646	Ca_kabuli_scaffold	23646	[A/G]	1067305020
Ca_Kabuli_NW_004516296.1_28461	Ca_kabuli_scaffold	28461	[C/T]	1067305021
Ca_Kabuli_NW_004516300.1_97230	Ca_kabuli_scaffold	97230	[A/C]	1067305022
Ca_Kabuli_NW_004516300.1_97243	Ca_kabuli_scaffold	97243	[A/T]	1067305023
Ca_Kabuli_NW_004516300.1_97290	Ca_kabuli_scaffold	97290	[T/G]	1067305024
Ca_Kabuli_NW_004516300.1_97310	Ca_kabuli_scaffold	97310	[A/C]	1067305025
Ca_Kabuli_NW_004516300.1_97323	Ca_kabuli_scaffold	97323	[A/G]	1067305026
Ca_Kabuli_NW_004516300.1_98645	Ca_kabuli_scaffold	98645	[C/T]	1067305027
Ca_Kabuli_NW_004516300.1_106864	Ca_kabuli_scaffold	106864	[T/G]	1067305028
Ca_Kabuli_NW_004516300.1_106865	Ca_kabuli_scaffold	106865	[G/T]	1067305029
Ca_Kabuli_NW_004516300.1_106866	Ca_kabuli_scaffold	106866	[G/C]	1067305030
Ca_Kabuli_NW_004516300.1_106867	Ca_kabuli_scaffold	106867	[C/T]	1067305031
Ca_Kabuli_NW_004516300.1_106868	Ca_kabuli_scaffold	106868	[C/T]	1067305032
Ca_Kabuli_NW_004516302.1_77084	Ca_kabuli_scaffold	77084	[T/G]	1067305033
Ca_Kabuli_NW_004516309.1_18523	Ca_kabuli_scaffold	18523	[C/G]	1067305034
Ca_Kabuli_NW_004516312.1_3364	Ca_kabuli_scaffold	3364	[G/T]	1067305035
Ca_Kabuli_NW_004516312.1_3397	Ca_kabuli_scaffold	3397	[G/A]	1067305036
Ca_Kabuli_NW_004516316.1_165968	Ca_kabuli_scaffold	165968	[T/C]	1067305037
Ca_Kabuli_NW_004516316.1_165974	Ca_kabuli_scaffold	165974	[T/C]	1067305038
Ca_Kabuli_NW_004516316.1_224151	Ca_kabuli_scaffold	224151	[A/C]	1067305039
Ca_Kabuli_NW_004516330.1_169260	Ca_kabuli_scaffold	169260	[T/C]	1067305040
Ca_Kabuli_NW_004516330.1_235258	Ca_kabuli_scaffold	235258	[T/G]	1067305041
Ca_Kabuli_NW_004516330.1_436930	Ca_kabuli_scaffold	436930	[A/C]	1067305042
Ca_Kabuli_NW_004516330.1_491166	Ca_kabuli_scaffold	491166	[T/C]	1067305043
Ca_Kabuli_NW_004516330.1_491172	Ca_kabuli_scaffold	491172	[T/G]	1067305044
Ca_Kabuli_NW_004516330.1_491190	Ca_kabuli_scaffold	491190	[A/C]	1067305045
Ca_Kabuli_NW_004516330.1_491204	Ca_kabuli_scaffold	491204	[C/G]	1067305046
Ca_Kabuli_NW_004516330.1_492368	Ca_kabuli_scaffold	492368	[T/C]	1067305047
Ca_Kabuli_NW_004516330.1_492384	Ca_kabuli_scaffold	492384	[A/G]	1067305048
Ca_Kabuli_NW_004516330.1_492398	Ca_kabuli_scaffold	492398	[A/C]	1067305049
Ca_Kabuli_NW_004516330.1_497212	Ca_kabuli_scaffold	497212	[T/C]	1067305050
Ca_Kabuli_NW_004516330.1_497269	Ca_kabuli_scaffold	497269	[A/C]	1067305051
Ca_Kabuli_NW_004516330.1_642807	Ca_kabuli_scaffold	642807	[T/C]	1067305052

SNP IDs	Chromosomes	Physical positions (bp)	SNPs	NCBI SNPdb accession IDs (NCBI_ss#)
Ca_Kabuli_NW_004516330.1_642850	Ca_kabuli_scaffold	642850	[A/C]	1067305053
Ca_Kabuli_NW_004516330.1_642859	Ca_kabuli_scaffold	642859	[A/C]	1067305054
Ca_Kabuli_NW_004516330.1_644893	Ca_kabuli_scaffold	644893	[A/C]	1067305055
Ca_Kabuli_NW_004516344.1_251996	Ca_kabuli_scaffold	251996	[A/G]	1067305056
Ca_Kabuli_NW_004516344.1_251998	Ca_kabuli_scaffold	251998	[A/G]	1067305057
Ca_Kabuli_NW_004516344.1_252044	Ca_kabuli_scaffold	252044	[A/C]	1067305058
Ca_Kabuli_NW_004516344.1_252629	Ca_kabuli_scaffold	252629	[T/G]	1067305059
Ca_Kabuli_NW_004516344.1_252652	Ca_kabuli_scaffold	252652	[A/G]	1067305060
Ca_Kabuli_NW_004516344.1_252659	Ca_kabuli_scaffold	252659	[T/G]	1067305061
Ca_Kabuli_NW_004516344.1_254068	Ca_kabuli_scaffold	254068	[A/G]	1067305062
Ca_Kabuli_NW_004516344.1_254074	Ca_kabuli_scaffold	254074	[A/C]	1067305063
Ca_Kabuli_NW_004516344.1_254096	Ca_kabuli_scaffold	254096	[A/G]	1067305064
Ca_Kabuli_NW_004516344.1_256575	Ca_kabuli_scaffold	256575	[C/A]	1067305065
Ca_Kabuli_NW_004516344.1_256577	Ca_kabuli_scaffold	256577	[G/A]	1067305066
Ca_Kabuli_NW_004516344.1_256589	Ca_kabuli_scaffold	256589	[C/T]	1067305067
Ca_Kabuli_NW_004516354.1_16355	Ca_kabuli_scaffold	16355	[T/A]	1067305068
Ca_Kabuli_NW_004516354.1_90013	Ca_kabuli_scaffold	90013	[G/T]	1067305069
Ca_Kabuli_NW_004516358.1_51557	Ca_kabuli_scaffold	51557	[T/A]	1067305070
Ca_Kabuli_NW_004516358.1_51573	Ca_kabuli_scaffold	51573	[T/A]	1067305071
Ca_Kabuli_NW_004516358.1_89948	Ca_kabuli_scaffold	89948	[T/G]	1067305072
Ca_Kabuli_NW_004516358.1_92991	Ca_kabuli_scaffold	92991	[A/C]	1067305073
Ca_Kabuli_NW_004516358.1_113321	Ca_kabuli_scaffold	113321	[T/G]	1067305074
Ca_Kabuli_NW_004516358.1_303408	Ca_kabuli_scaffold	303408	[A/G]	1067305075
Ca_Kabuli_NW_004516358.1_303409	Ca_kabuli_scaffold	303409	[C/T]	1067305076
Ca_Kabuli_NW_004516358.1_303410	Ca_kabuli_scaffold	303410	[C/A]	1067305077
Ca_Kabuli_NW_004516358.1_342717	Ca_kabuli_scaffold	342717	[C/G]	1067305078
Ca_Kabuli_NW_004516358.1_342718	Ca_kabuli_scaffold	342718	[T/A]	1067305079
Ca_Kabuli_NW_004516358.1_406705	Ca_kabuli_scaffold	406705	[C/A]	1067305080
Ca_Kabuli_NW_004516358.1_406706	Ca_kabuli_scaffold	406706	[T/G]	1067305081
Ca_Kabuli_NW_004516358.1_430362	Ca_kabuli_scaffold	430362	[A/C]	1067305082
Ca_Kabuli_NW_004516358.1_430408	Ca_kabuli_scaffold	430408	[A/G]	1067305083
Ca_Kabuli_NW_004516358.1_430426	Ca_kabuli_scaffold	430426	[A/G]	1067305084
Ca_Kabuli_NW_004516358.1_430431	Ca_kabuli_scaffold	430431	[T/G]	1067305085
Ca_Kabuli_NW_004516358.1_439402	Ca_kabuli_scaffold	439402	[T/A]	1067305086

SNP IDs	Chromosomes	Physical positions (bp)	SNPs	NCBI SNPdb accession IDs (NCBI_ss#)
Ca_Kabuli_NW_004516358.1_457913	Ca_kabuli_scaffold	457913	[A/C]	1067305087
Ca_Kabuli_NW_004516358.1_577902	Ca_kabuli_scaffold	577902	[T/G]	1067305088
Ca_Kabuli_NW_004516360.1_70241	Ca_kabuli_scaffold	70241	[T/A]	1067305089
Ca_Kabuli_NW_004516361.1_128669	Ca_kabuli_scaffold	128669	[T/G]	1067305090
Ca_Kabuli_NW_004516363.1_131866	Ca_kabuli_scaffold	131866	[C/T]	1067305091
Ca_Kabuli_NW_004516365.1_318328	Ca_kabuli_scaffold	318328	[C/A]	1067305092
Ca_Kabuli_NW_004516366.1_173366	Ca_kabuli_scaffold	173366	[A/T]	1067305093
Ca_Kabuli_NW_004516366.1_174360	Ca_kabuli_scaffold	174360	[A/G]	1067305094
Ca_Kabuli_NW_004516370.1_141233	Ca_kabuli_scaffold	141233	[C/T]	1067305095
Ca_Kabuli_NW_004516370.1_141254	Ca_kabuli_scaffold	141254	[A/G]	1067305096
Ca_Kabuli_NW_004516371.1_63620	Ca_kabuli_scaffold	63620	[C/T]	1067305097
Ca_Kabuli_NW_004516371.1_63662	Ca_kabuli_scaffold	63662	[T/G]	1067305098
Ca_Kabuli_NW_004516371.1_63669	Ca_kabuli_scaffold	63669	[C/T]	1067305099
Ca_Kabuli_NW_004516371.1_63671	Ca_kabuli_scaffold	63671	[C/T]	1067305100
Ca_Kabuli_NW_004516371.1_63703	Ca_kabuli_scaffold	63703	[T/C]	1067305101
Ca_Kabuli_NW_004516373.1_142671	Ca_kabuli_scaffold	142671	[T/G]	1067305102
Ca_Kabuli_NW_004516373.1_144885	Ca_kabuli_scaffold	144885	[C/A]	1067305103
Ca_Kabuli_NW_004516373.1_147299	Ca_kabuli_scaffold	147299	[T/G]	1067305104
Ca_Kabuli_NW_004516373.1_147319	Ca_kabuli_scaffold	147319	[T/G]	1067305105
Ca_Kabuli_NW_004516373.1_147324	Ca_kabuli_scaffold	147324	[A/C]	1067305106
Ca_Kabuli_NW_004516373.1_148636	Ca_kabuli_scaffold	148636	[A/C]	1067305107
Ca_Kabuli_NW_004516373.1_148947	Ca_kabuli_scaffold	148947	[G/T]	1067305108
Ca_Kabuli_NW_004516373.1_152195	Ca_kabuli_scaffold	152195	[T/C]	1067305109
Ca_Kabuli_NW_004516373.1_152233	Ca_kabuli_scaffold	152233	[A/G]	1067305110
Ca_Kabuli_NW_004516373.1_152265	Ca_kabuli_scaffold	152265	[T/C]	1067305111
Ca_Kabuli_NW_004516373.1_152307	Ca_kabuli_scaffold	152307	[T/A]	1067305112
Ca_Kabuli_NW_004516375.1_70680	Ca_kabuli_scaffold	70680	[T/C]	1067305113
Ca_Kabuli_NW_004516375.1_70714	Ca_kabuli_scaffold	70714	[A/G]	1067305114
Ca_Kabuli_NW_004516375.1_70731	Ca_kabuli_scaffold	70731	[T/C]	1067305115
Ca_Kabuli_NW_004516375.1_214087	Ca_kabuli_scaffold	214087	[T/G]	1067305116
Ca_Kabuli_NW_004516375.1_214191	Ca_kabuli_scaffold	214191	[T/G]	1067305117
Ca_Kabuli_NW_004516375.1_214208	Ca_kabuli_scaffold	214208	[A/G]	1067305118
Ca_Kabuli_NW_004516375.1_214283	Ca_kabuli_scaffold	214283	[A/G]	1067305119
Ca_Kabuli_NW_004516375.1_221952	Ca_kabuli_scaffold	221952	[C/A]	1067305120

SNP IDs	Chromosomes	Physical positions (bp)	SNPs	NCBI SNPdb accession IDs (NCBI_ss#)
Ca_Kabuli_NW_004516375.1_221984	Ca_kabuli_scaffold	221984	[A/C]	1067305121
Ca_Kabuli_NW_004516375.1_222535	Ca_kabuli_scaffold	222535	[A/C]	1067305122
Ca_Kabuli_NW_004516375.1_240787	Ca_kabuli_scaffold	240787	[A/G]	1067305123
Ca_Kabuli_NW_004516375.1_240798	Ca_kabuli_scaffold	240798	[T/C]	1067305124
Ca_Kabuli_NW_004516375.1_270720	Ca_kabuli_scaffold	270720	[T/A]	1067305125
Ca_Kabuli_NW_004516375.1_270749	Ca_kabuli_scaffold	270749	[T/C]	1067305126
Ca_Kabuli_NW_004516391.1_11099	Ca_kabuli_scaffold	11099	[C/A]	1067305127
Ca_Kabuli_NW_004516391.1_11108	Ca_kabuli_scaffold	11108	[C/A]	1067305128
Ca_Kabuli_NW_004516391.1_11115	Ca_kabuli_scaffold	11115	[G/T]	1067305129
Ca_Kabuli_NW_004516391.1_11144	Ca_kabuli_scaffold	11144	[T/A]	1067305130
Ca_Kabuli_NW_004516391.1_33746	Ca_kabuli_scaffold	33746	[A/T]	1067305131
Ca_Kabuli_NW_004516398.1_1650	Ca_kabuli_scaffold	1650	[A/G]	1067305132
Ca_Kabuli_NW_004516399.1_6852	Ca_kabuli_scaffold	6852	[C/T]	1067305133
Ca_Kabuli_NW_004516402.1_58074	Ca_kabuli_scaffold	58074	[T/C]	1067305134
Ca_Kabuli_NW_004516409.1_6197	Ca_kabuli_scaffold	6197	[T/C]	1067305135
Ca_Kabuli_NW_004516409.1_6199	Ca_kabuli_scaffold	6199	[A/G]	1067305136
Ca_Kabuli_NW_004516409.1_6208	Ca_kabuli_scaffold	6208	[T/G]	1067305137
Ca_Kabuli_NW_004516409.1_6211	Ca_kabuli_scaffold	6211	[T/G]	1067305138
Ca_Kabuli_NW_004516409.1_6229	Ca_kabuli_scaffold	6229	[A/C]	1067305139
Ca_Kabuli_NW_004516409.1_6271	Ca_kabuli_scaffold	6271	[A/G]	1067305140
Ca_Kabuli_NW_004516409.1_87275	Ca_kabuli_scaffold	87275	[G/A]	1067305141
Ca_Kabuli_NW_004516409.1_99910	Ca_kabuli_scaffold	99910	[A/C]	1067305142
Ca_Kabuli_NW_004516409.1_100684	Ca_kabuli_scaffold	100684	[T/C]	1067305143
Ca_Kabuli_NW_004516411.1_7624	Ca_kabuli_scaffold	7624	[T/C]	1067305144
Ca_Kabuli_NW_004516417.1_341416	Ca_kabuli_scaffold	341416	[T/A]	1067305145
Ca_Kabuli_NW_004516417.1_360480	Ca_kabuli_scaffold	360480	[T/A]	1067305146
Ca_Kabuli_NW_004516417.1_360483	Ca_kabuli_scaffold	360483	[T/C]	1067305147
Ca_Kabuli_NW_004516417.1_360521	Ca_kabuli_scaffold	360521	[T/C]	1067305148
Ca_Kabuli_NW_004516417.1_360532	Ca_kabuli_scaffold	360532	[A/T]	1067305149
Ca_Kabuli_NW_004516417.1_360585	Ca_kabuli_scaffold	360585	[A/G]	1067305150
Ca_Kabuli_NW_004516417.1_374016	Ca_kabuli_scaffold	374016	[A/T]	1067305151
Ca_Kabuli_NW_004516417.1_374058	Ca_kabuli_scaffold	374058	[A/C]	1067305152
Ca_Kabuli_NW_004516417.1_374099	Ca_kabuli_scaffold	374099	[T/C]	1067305153
Ca_Kabuli_NW_004516417.1_374105	Ca_kabuli_scaffold	374105	[A/G]	1067305154

SNP IDs	Chromosomes	Physical positions (bp)	SNPs	NCBI SNPdb accession IDs (NCBI_ss#)
Ca_Kabuli_NW_004516422.1_4502	Ca_kabuli_scaffold	4502	[A/T]	1067305155
Ca_Kabuli_NW_004516423.1_55085	Ca_kabuli_scaffold	55085	[A/G]	1067305156
Ca_Kabuli_NW_004516435.1_128537	Ca_kabuli_scaffold	128537	[T/A]	1067305157
Ca_Kabuli_NW_004516455.1_52161	Ca_kabuli_scaffold	52161	[C/A]	1067305158
Ca_Kabuli_NW_004516455.1_96321	Ca_kabuli_scaffold	96321	[T/A]	1067305159
Ca_Kabuli_NW_004516455.1_96322	Ca_kabuli_scaffold	96322	[C/T]	1067305160
Ca_Kabuli_NW_004516455.1_96323	Ca_kabuli_scaffold	96323	[G/C]	1067305161
Ca_Kabuli_NW_004516455.1_96324	Ca_kabuli_scaffold	96324	[G/T]	1067305162
Ca_Kabuli_NW_004516455.1_178179	Ca_kabuli_scaffold	178179	[T/A]	1067305163
Ca_Kabuli_NW_004516455.1_178248	Ca_kabuli_scaffold	178248	[A/G]	1067305164
Ca_Kabuli_NW_004516455.1_324304	Ca_kabuli_scaffold	324304	[G/A]	1067305165
Ca_Kabuli_NW_004516455.1_330789	Ca_kabuli_scaffold	330789	[A/T]	1067305166
Ca_Kabuli_NW_004516455.1_330827	Ca_kabuli_scaffold	330827	[A/T]	1067305167
Ca_Kabuli_NW_004516459.1_32523	Ca_kabuli_scaffold	32523	[T/A]	1067305168
Ca_Kabuli_NW_004516459.1_32535	Ca_kabuli_scaffold	32535	[T/A]	1067305169
Ca_Kabuli_NW_004516459.1_32769	Ca_kabuli_scaffold	32769	[T/C]	1067305170
Ca_Kabuli_NW_004516459.1_33582	Ca_kabuli_scaffold	33582	[T/A]	1067305171
Ca_Kabuli_NW_004516461.1_5322	Ca_kabuli_scaffold	5322	[C/T]	1067305172
Ca_Kabuli_NW_004516463.1_1014	Ca_kabuli_scaffold	1014	[A/C]	1067305173
Ca_Kabuli_NW_004516471.1_70503	Ca_kabuli_scaffold	70503	[C/A]	1067305174
Ca_Kabuli_NW_004516471.1_70506	Ca_kabuli_scaffold	70506	[G/A]	1067305175
Ca_Kabuli_NW_004516471.1_70526	Ca_kabuli_scaffold	70526	[T/C]	1067305176
Ca_Kabuli_NW_004516471.1_70535	Ca_kabuli_scaffold	70535	[G/A]	1067305177
Ca_Kabuli_NW_004516471.1_70544	Ca_kabuli_scaffold	70544	[T/A]	1067305178
Ca_Kabuli_NW_004516471.1_70549	Ca_kabuli_scaffold	70549	[G/A]	1067305179
Ca_Kabuli_NW_004516472.1_453069	Ca_kabuli_scaffold	453069	[A/C]	1067305180
Ca_Kabuli_NW_004516474.1_46904	Ca_kabuli_scaffold	46904	[T/C]	1067305181
Ca_Kabuli_NW_004516474.1_46923	Ca_kabuli_scaffold	46923	[A/C]	1067305182
Ca_Kabuli_NW_004516474.1_86949	Ca_kabuli_scaffold	86949	[T/C]	1067305183
Ca_Kabuli_NW_004516474.1_86981	Ca_kabuli_scaffold	86981	[G/A]	1067305184
Ca_Kabuli_NW_004516474.1_87137	Ca_kabuli_scaffold	87137	[G/T]	1067305185
Ca_Kabuli_NW_004516483.1_85047	Ca_kabuli_scaffold	85047	[T/C]	1067305186
Ca_Kabuli_NW_004516483.1_89990	Ca_kabuli_scaffold	89990	[T/C]	1067305187
Ca_Kabuli_NW_004516483.1_90027	Ca_kabuli_scaffold	90027	[A/G]	1067305188

SNP IDs	Chromosomes	Physical positions (bp)	SNPs	NCBI SNPdb accession IDs (NCBI_ss#)
Ca_Kabuli_NW_004516485.1_7846	Ca_kabuli_scaffold	7846	[A/C]	1067305189
Ca_Kabuli_NW_004516514.1_71597	Ca_kabuli_scaffold	71597	[A/T]	1067305190
Ca_Kabuli_NW_004516514.1_71620	Ca_kabuli_scaffold	71620	[T/A]	1067305191
Ca_Kabuli_NW_004516514.1_75766	Ca_kabuli_scaffold	75766	[T/A]	1067305192
Ca_Kabuli_NW_004516514.1_75785	Ca_kabuli_scaffold	75785	[A/G]	1067305193
Ca_Kabuli_NW_004516521.1_62751	Ca_kabuli_scaffold	62751	[C/T]	1067305194
Ca_Kabuli_NW_004516521.1_62775	Ca_kabuli_scaffold	62775	[T/C]	1067305195
Ca_Kabuli_NW_004516521.1_62778	Ca_kabuli_scaffold	62778	[T/A]	1067305196
Ca_Kabuli_NW_004516521.1_133056	Ca_kabuli_scaffold	133056	[A/G]	1067305197
Ca_Kabuli_NW_004516521.1_133107	Ca_kabuli_scaffold	133107	[C/T]	1067305198
Ca_Kabuli_NW_004516525.1_615	Ca_kabuli_scaffold	615	[A/T]	1067305199
Ca_Kabuli_NW_004516525.1_618	Ca_kabuli_scaffold	618	[G/A]	1067305200
Ca_Kabuli_NW_004516525.1_623	Ca_kabuli_scaffold	623	[T/C]	1067305201
Ca_Kabuli_NW_004516526.1_63185	Ca_kabuli_scaffold	63185	[G/A]	1067305202
Ca_Kabuli_NW_004516526.1_63237	Ca_kabuli_scaffold	63237	[T/C]	1067305203
Ca_Kabuli_NW_004516528.1_55699	Ca_kabuli_scaffold	55699	[A/C]	1067305204
Ca_Kabuli_NW_004516528.1_211511	Ca_kabuli_scaffold	211511	[T/C]	1067305205
Ca_Kabuli_NW_004516528.1_211512	Ca_kabuli_scaffold	211512	[G/T]	1067305206
Ca_Kabuli_NW_004516528.1_211548	Ca_kabuli_scaffold	211548	[A/C]	1067305207
Ca_Kabuli_NW_004516528.1_244118	Ca_kabuli_scaffold	244118	[T/C]	1067305208
Ca_Kabuli_NW_004516528.1_315994	Ca_kabuli_scaffold	315994	[A/C]	1067305209
Ca_Kabuli_NW_004516528.1_316037	Ca_kabuli_scaffold	316037	[G/A]	1067305210
Ca_Kabuli_NW_004516528.1_398892	Ca_kabuli_scaffold	398892	[T/C]	1067305211
Ca_Kabuli_NW_004516528.1_398950	Ca_kabuli_scaffold	398950	[A/G]	1067305212
Ca_Kabuli_NW_004516528.1_398960	Ca_kabuli_scaffold	398960	[A/G]	1067305213
Ca_Kabuli_NW_004516528.1_404196	Ca_kabuli_scaffold	404196	[G/A]	1067305214
Ca_Kabuli_NW_004516528.1_408211	Ca_kabuli_scaffold	408211	[A/C]	1067305215
Ca_Kabuli_NW_004516528.1_409760	Ca_kabuli_scaffold	409760	[C/A]	1067305216
Ca_Kabuli_NW_004516528.1_409761	Ca_kabuli_scaffold	409761	[A/G]	1067305217
Ca_Kabuli_NW_004516528.1_409762	Ca_kabuli_scaffold	409762	[T/A]	1067305218
Ca_Kabuli_NW_004516528.1_409763	Ca_kabuli_scaffold	409763	[C/T]	1067305219
Ca_Kabuli_NW_004516528.1_463286	Ca_kabuli_scaffold	463286	[C/T]	1067305220
Ca_Kabuli_NW_004516530.1_68225	Ca_kabuli_scaffold	68225	[T/A]	1067305221
Ca_Kabuli_NW_004516530.1_192108	Ca_kabuli_scaffold	192108	[A/C]	1067305222

SNP IDs	Chromosomes	Physical positions (bp)	SNPs	NCBI SNPdb accession IDs (NCBI_ss#)
Ca_Kabuli_NW_004516530.1_294949	Ca_kabuli_scaffold	294949	[T/A]	1067305223
Ca_Kabuli_NW_004516539.1_45813	Ca_kabuli_scaffold	45813	[G/C]	1067305224
Ca_Kabuli_NW_004516539.1_45814	Ca_kabuli_scaffold	45814	[G/T]	1067305225
Ca_Kabuli_NW_004516539.1_86925	Ca_kabuli_scaffold	86925	[T/G]	1067305226
Ca_Kabuli_NW_004516539.1_86939	Ca_kabuli_scaffold	86939	[A/C]	1067305227
Ca_Kabuli_NW_004516539.1_152304	Ca_kabuli_scaffold	152304	[T/G]	1067305228
Ca_Kabuli_NW_004516539.1_157317	Ca_kabuli_scaffold	157317	[T/G]	1067305229
Ca_Kabuli_NW_004516539.1_158551	Ca_kabuli_scaffold	158551	[A/G]	1067305230
Ca_Kabuli_NW_004516539.1_188401	Ca_kabuli_scaffold	188401	[A/C]	1067305231
Ca_Kabuli_NW_004516539.1_188407	Ca_kabuli_scaffold	188407	[A/C]	1067305232
Ca_Kabuli_NW_004516539.1_253742	Ca_kabuli_scaffold	253742	[A/C]	1067305233
Ca_Kabuli_NW_004516551.1_115808	Ca_kabuli_scaffold	115808	[C/T]	1067305234
Ca_Kabuli_NW_004516559.1_7296	Ca_kabuli_scaffold	7296	[A/T]	1067305235
Ca_Kabuli_NW_004516559.1_7306	Ca_kabuli_scaffold	7306	[G/A]	1067305236
Ca_Kabuli_NW_004516559.1_138230	Ca_kabuli_scaffold	138230	[A/G]	1067305237
Ca_Kabuli_NW_004516559.1_138304	Ca_kabuli_scaffold	138304	[C/A]	1067305238
Ca_Kabuli_NW_004516559.1_138407	Ca_kabuli_scaffold	138407	[T/C]	1067305239
Ca_Kabuli_NW_004516559.1_138411	Ca_kabuli_scaffold	138411	[G/T]	1067305240
Ca_Kabuli_NW_004516559.1_228328	Ca_kabuli_scaffold	228328	[G/T]	1067305241
Ca_Kabuli_NW_004516559.1_228459	Ca_kabuli_scaffold	228459	[T/A]	1067305242
Ca_Kabuli_NW_004516559.1_228679	Ca_kabuli_scaffold	228679	[G/A]	1067305243
Ca_Kabuli_NW_004516559.1_228753	Ca_kabuli_scaffold	228753	[A/C]	1067305244
Ca_Kabuli_NW_004516559.1_241569	Ca_kabuli_scaffold	241569	[C/T]	1067305245
Ca_Kabuli_NW_004516559.1_648030	Ca_kabuli_scaffold	648030	[A/C]	1067305246
Ca_Kabuli_NW_004516559.1_648031	Ca_kabuli_scaffold	648031	[G/T]	1067305247
Ca_Kabuli_NW_004516564.1_32927	Ca_kabuli_scaffold	32927	[A/C]	1067305248
Ca_Kabuli_NW_004516564.1_32941	Ca_kabuli_scaffold	32941	[A/T]	1067305249
Ca_Kabuli_NW_004516564.1_32967	Ca_kabuli_scaffold	32967	[T/G]	1067305250
Ca_Kabuli_NW_004516575.1_151274	Ca_kabuli_scaffold	151274	[A/C]	1067305251
Ca_Kabuli_NW_004516575.1_212085	Ca_kabuli_scaffold	212085	[T/G]	1067305252
Ca_Kabuli_NW_004516575.1_216064	Ca_kabuli_scaffold	216064	[A/C]	1067305253
Ca_Kabuli_NW_004516575.1_261512	Ca_kabuli_scaffold	261512	[A/C]	1067305254
Ca_Kabuli_NW_004516575.1_290943	Ca_kabuli_scaffold	290943	[A/G]	1067305255
Ca_Kabuli_NW_004516575.1_300406	Ca_kabuli_scaffold	300406	[A/T]	1067305256

SNP IDs	Chromosomes	Physical positions (bp)	SNPs	NCBI SNPdb accession IDs (NCBI_ss#)
Ca_Kabuli_NW_004516575.1_300449	Ca_kabuli_scaffold	300449	[A/G]	1067305257
Ca_Kabuli_NW_004516575.1_300459	Ca_kabuli_scaffold	300459	[T/A]	1067305258
Ca_Kabuli_NW_004516575.1_322151	Ca_kabuli_scaffold	322151	[A/T]	1067305259
Ca_Kabuli_NW_004516575.1_373027	Ca_kabuli_scaffold	373027	[A/G]	1067305260
Ca_Kabuli_NW_004516575.1_373032	Ca_kabuli_scaffold	373032	[A/T]	1067305261
Ca_Kabuli_NW_004516575.1_373091	Ca_kabuli_scaffold	373091	[A/G]	1067305262
Ca_Kabuli_NW_004516575.1_451962	Ca_kabuli_scaffold	451962	[T/G]	1067305263
Ca_Kabuli_NW_004516575.1_451964	Ca_kabuli_scaffold	451964	[A/C]	1067305264
Ca_Kabuli_NW_004516575.1_451968	Ca_kabuli_scaffold	451968	[A/C]	1067305265
Ca_Kabuli_NW_004516575.1_451971	Ca_kabuli_scaffold	451971	[A/G]	1067305266
Ca_Kabuli_NW_004516575.1_451981	Ca_kabuli_scaffold	451981	[A/C]	1067305267
Ca_Kabuli_NW_004516575.1_451989	Ca_kabuli_scaffold	451989	[A/G]	1067305268
Ca_Kabuli_NW_004516575.1_471152	Ca_kabuli_scaffold	471152	[A/C]	1067305269
Ca_Kabuli_NW_004516575.1_499216	Ca_kabuli_scaffold	499216	[T/C]	1067305270
Ca_Kabuli_NW_004516575.1_499225	Ca_kabuli_scaffold	499225	[A/T]	1067305271
Ca_Kabuli_NW_004516575.1_499843	Ca_kabuli_scaffold	499843	[A/G]	1067305272
Ca_Kabuli_NW_004516575.1_500601	Ca_kabuli_scaffold	500601	[T/C]	1067305273
Ca_Kabuli_NW_004516575.1_500640	Ca_kabuli_scaffold	500640	[T/A]	1067305274
Ca_Kabuli_NW_004516575.1_506478	Ca_kabuli_scaffold	506478	[T/G]	1067305275
Ca_Kabuli_NW_004516575.1_533198	Ca_kabuli_scaffold	533198	[A/G]	1067305276
Ca_Kabuli_NW_004516575.1_679013	Ca_kabuli_scaffold	679013	[A/G]	1067305277
Ca_Kabuli_NW_004516575.1_679022	Ca_kabuli_scaffold	679022	[T/C]	1067305278
Ca_Kabuli_NW_004516575.1_679037	Ca_kabuli_scaffold	679037	[T/C]	1067305279
Ca_Kabuli_NW_004516575.1_709177	Ca_kabuli_scaffold	709177	[T/C]	1067305280
Ca_Kabuli_NW_004516575.1_709191	Ca_kabuli_scaffold	709191	[T/A]	1067305281
Ca_Kabuli_NW_004516575.1_730109	Ca_kabuli_scaffold	730109	[T/G]	1067305282
Ca_Kabuli_NW_004516575.1_730126	Ca_kabuli_scaffold	730126	[A/C]	1067305283
Ca_Kabuli_NW_004516575.1_730137	Ca_kabuli_scaffold	730137	[A/T]	1067305284
Ca_Kabuli_NW_004516575.1_730142	Ca_kabuli_scaffold	730142	[A/G]	1067305285
Ca_Kabuli_NW_004516575.1_732450	Ca_kabuli_scaffold	732450	[A/C]	1067305286
Ca_Kabuli_NW_004516575.1_732454	Ca_kabuli_scaffold	732454	[T/G]	1067305287
Ca_Kabuli_NW_004516575.1_732463	Ca_kabuli_scaffold	732463	[G/T]	1067305288
Ca_Kabuli_NW_004516575.1_735834	Ca_kabuli_scaffold	735834	[A/T]	1067305289
Ca_Kabuli_NW_004516575.1_745915	Ca_kabuli_scaffold	745915	[T/C]	1067305290

SNP IDs	Chromosomes	Physical positions (bp)	SNPs	NCBI SNPdb accession IDs (NCBI_ss#)
Ca_Kabuli_NW_004516575.1_750149	Ca_kabuli_scaffold	750149	[T/C]	1067305291
Ca_Kabuli_NW_004516575.1_750153	Ca_kabuli_scaffold	750153	[A/T]	1067305292
Ca_Kabuli_NW_004516575.1_750157	Ca_kabuli_scaffold	750157	[A/C]	1067305293
Ca_Kabuli_NW_004516575.1_750167	Ca_kabuli_scaffold	750167	[A/C]	1067305294
Ca_Kabuli_NW_004516575.1_750215	Ca_kabuli_scaffold	750215	[A/G]	1067305295
Ca_Kabuli_NW_004516575.1_771024	Ca_kabuli_scaffold	771024	[A/G]	1067305296
Ca_Kabuli_NW_004516575.1_771036	Ca_kabuli_scaffold	771036	[A/C]	1067305297
Ca_Kabuli_NW_004516575.1_800500	Ca_kabuli_scaffold	800500	[C/T]	1067305298
Ca_Kabuli_NW_004516575.1_871632	Ca_kabuli_scaffold	871632	[G/A]	1067305299
Ca_Kabuli_NW_004516575.1_871634	Ca_kabuli_scaffold	871634	[A/G]	1067305300
Ca_Kabuli_NW_004516575.1_871635	Ca_kabuli_scaffold	871635	[A/C]	1067305301
Ca_Kabuli_NW_004516575.1_909909	Ca_kabuli_scaffold	909909	[A/C]	1067305302
Ca_Kabuli_NW_004516575.1_923881	Ca_kabuli_scaffold	923881	[T/A]	1067305303
Ca_Kabuli_NW_004516575.1_923907	Ca_kabuli_scaffold	923907	[A/C]	1067305304
Ca_Kabuli_NW_004516575.1_923936	Ca_kabuli_scaffold	923936	[A/C]	1067305305
Ca_Kabuli_NW_004516575.1_923939	Ca_kabuli_scaffold	923939	[A/C]	1067305306
Ca_Kabuli_NW_004516575.1_924154	Ca_kabuli_scaffold	924154	[T/G]	1067305307
Ca_Kabuli_NW_004516575.1_971191	Ca_kabuli_scaffold	971191	[A/C]	1067305308
Ca_Kabuli_NW_004516575.1_971230	Ca_kabuli_scaffold	971230	[A/G]	1067305309
Ca_Kabuli_NW_004516575.1_971232	Ca_kabuli_scaffold	971232	[C/A]	1067305310
Ca_Kabuli_NW_004516575.1_971237	Ca_kabuli_scaffold	971237	[A/G]	1067305311
Ca_Kabuli_NW_004516575.1_971261	Ca_kabuli_scaffold	971261	[T/C]	1067305312
Ca_Kabuli_NW_004516575.1_971281	Ca_kabuli_scaffold	971281	[T/G]	1067305313
Ca_Kabuli_NW_004516575.1_971284	Ca_kabuli_scaffold	971284	[T/C]	1067305314
Ca_Kabuli_NW_004516575.1_971292	Ca_kabuli_scaffold	971292	[T/C]	1067305315
Ca_Kabuli_NW_004516575.1_1066731	Ca_kabuli_scaffold	1066731	[A/C]	1067305316
Ca_Kabuli_NW_004516575.1_1072750	Ca_kabuli_scaffold	1072750	[T/C]	1067305317
Ca_Kabuli_NW_004516575.1_1072970	Ca_kabuli_scaffold	1072970	[T/G]	1067305318
Ca_Kabuli_NW_004516575.1_1073115	Ca_kabuli_scaffold	1073115	[T/G]	1067305319
Ca_Kabuli_NW_004516575.1_1172634	Ca_kabuli_scaffold	1172634	[T/C]	1067305320
Ca_Kabuli_NW_004516575.1_1207416	Ca_kabuli_scaffold	1207416	[T/G]	1067305321
Ca_Kabuli_NW_004516575.1_1234641	Ca_kabuli_scaffold	1234641	[T/G]	1067305322
Ca_Kabuli_NW_004516575.1_1234662	Ca_kabuli_scaffold	1234662	[A/G]	1067305323
Ca_Kabuli_NW_004516575.1_1263414	Ca_kabuli_scaffold	1263414	[T/C]	1067305324

SNP IDs	Chromosomes	Physical positions (bp)	SNPs	NCBI SNPdb accession IDs (NCBI_ss#)
Ca_Kabuli_NW_004516575.1_1263442	Ca_kabuli_scaffold	1263442	[T/C]	1067305325
Ca_Kabuli_NW_004516575.1_1263455	Ca_kabuli_scaffold	1263455	[C/A]	1067305326
Ca_Kabuli_NW_004516575.1_1263457	Ca_kabuli_scaffold	1263457	[C/A]	1067305327
Ca_Kabuli_NW_004516575.1_1263469	Ca_kabuli_scaffold	1263469	[A/G]	1067305328
Ca_Kabuli_NW_004516575.1_1263503	Ca_kabuli_scaffold	1263503	[A/G]	1067305329
Ca_Kabuli_NW_004516575.1_1263537	Ca_kabuli_scaffold	1263537	[T/C]	1067305330
Ca_Kabuli_NW_004516575.1_1265627	Ca_kabuli_scaffold	1265627	[C/T]	1067305331
Ca_Kabuli_NW_004516575.1_1265638	Ca_kabuli_scaffold	1265638	[T/G]	1067305332
Ca_Kabuli_NW_004516575.1_1285272	Ca_kabuli_scaffold	1285272	[T/G]	1067305333
Ca_Kabuli_NW_004516575.1_1285275	Ca_kabuli_scaffold	1285275	[T/G]	1067305334
Ca_Kabuli_NW_004516575.1_1287875	Ca_kabuli_scaffold	1287875	[T/G]	1067305335
Ca_Kabuli_NW_004516575.1_1287887	Ca_kabuli_scaffold	1287887	[T/G]	1067305336
Ca_Kabuli_NW_004516575.1_1288404	Ca_kabuli_scaffold	1288404	[T/C]	1067305337
Ca_Kabuli_NW_004516575.1_1288428	Ca_kabuli_scaffold	1288428	[A/T]	1067305338
Ca_Kabuli_NW_004516575.1_1288440	Ca_kabuli_scaffold	1288440	[T/G]	1067305339
Ca_Kabuli_NW_004516577.1_137295	Ca_kabuli_scaffold	137295	[A/C]	1067305340
Ca_Kabuli_NW_004516577.1_137296	Ca_kabuli_scaffold	137296	[G/T]	1067305341
Ca_Kabuli_NW_004516577.1_137297	Ca_kabuli_scaffold	137297	[A/C]	1067305342
Ca_Kabuli_NW_004516577.1_204629	Ca_kabuli_scaffold	204629	[C/A]	1067305343
Ca_Kabuli_NW_004516577.1_204633	Ca_kabuli_scaffold	204633	[T/A]	1067305344
Ca_Kabuli_NW_004516577.1_204642	Ca_kabuli_scaffold	204642	[T/A]	1067305345
Ca_Kabuli_NW_004516577.1_272771	Ca_kabuli_scaffold	272771	[A/G]	1067305346
Ca_Kabuli_NW_004516593.1_8010	Ca_kabuli_scaffold	8010	[A/T]	1067305347
Ca_Kabuli_NW_004516593.1_8058	Ca_kabuli_scaffold	8058	[A/G]	1067305348
Ca_Kabuli_NW_004516593.1_8086	Ca_kabuli_scaffold	8086	[T/G]	1067305349
Ca_Kabuli_NW_004516593.1_8115	Ca_kabuli_scaffold	8115	[T/G]	1067305350
Ca_Kabuli_NW_004516593.1_8211	Ca_kabuli_scaffold	8211	[A/G]	1067305351
Ca_Kabuli_NW_004516593.1_8315	Ca_kabuli_scaffold	8315	[T/G]	1067305352
Ca_Kabuli_NW_004516593.1_8336	Ca_kabuli_scaffold	8336	[A/G]	1067305353
Ca_Kabuli_NW_004516593.1_20569	Ca_kabuli_scaffold	20569	[T/G]	1067305354
Ca_Kabuli_NW_004516593.1_20683	Ca_kabuli_scaffold	20683	[T/G]	1067305355
Ca_Kabuli_NW_004516593.1_31000	Ca_kabuli_scaffold	31000	[A/G]	1067305356
Ca_Kabuli_NW_004516593.1_31044	Ca_kabuli_scaffold	31044	[T/G]	1067305357
Ca_Kabuli_NW_004516593.1_33034	Ca_kabuli_scaffold	33034	[T/C]	1067305358

SNP IDs	Chromosomes	Physical positions (bp)	SNPs	NCBI SNPdb accession IDs (NCBI_ss#)
Ca_Kabuli_NW_004516593.1_104453	Ca_kabuli_scaffold	104453	[T/C]	1067305359
Ca_Kabuli_NW_004516595.1_85314	Ca_kabuli_scaffold	85314	[A/G]	1067305360
Ca_Kabuli_NW_004516595.1_85315	Ca_kabuli_scaffold	85315	[A/G]	1067305361
Ca_Kabuli_NW_004516602.1_186361	Ca_kabuli_scaffold	186361	[A/C]	1067305362
Ca_Kabuli_NW_004516623.1_47161	Ca_kabuli_scaffold	47161	[C/T]	1067305363
Ca_Kabuli_NW_004516628.1_76597	Ca_kabuli_scaffold	76597	[A/C]	1067305364
Ca_Kabuli_NW_004516628.1_76623	Ca_kabuli_scaffold	76623	[C/T]	1067305365
Ca_Kabuli_NW_004516628.1_76627	Ca_kabuli_scaffold	76627	[A/C]	1067305366
Ca_Kabuli_NW_004516628.1_131551	Ca_kabuli_scaffold	131551	[A/C]	1067305367
Ca_Kabuli_NW_004516628.1_257812	Ca_kabuli_scaffold	257812	[A/C]	1067305368
Ca_Kabuli_NW_004516628.1_257858	Ca_kabuli_scaffold	257858	[A/C]	1067305369
Ca_Kabuli_NW_004516629.1_96216	Ca_kabuli_scaffold	96216	[T/C]	1067305370
Ca_Kabuli_NW_004516630.1_25681	Ca_kabuli_scaffold	25681	[C/G]	1067305371
Ca_Kabuli_NW_004516641.1_1723	Ca_kabuli_scaffold	1723	[C/T]	1067305372
Ca_Kabuli_NW_004516643.1_242575	Ca_kabuli_scaffold	242575	[T/G]	1067305373
Ca_Kabuli_NW_004516650.1_48447	Ca_kabuli_scaffold	48447	[T/C]	1067305374
Ca_Kabuli_NW_004516650.1_70424	Ca_kabuli_scaffold	70424	[G/A]	1067305375
Ca_Kabuli_NW_004516650.1_74434	Ca_kabuli_scaffold	74434	[C/T]	1067305376
Ca_Kabuli_NW_004516650.1_74436	Ca_kabuli_scaffold	74436	[C/T]	1067305377
Ca_Kabuli_NW_004516650.1_74521	Ca_kabuli_scaffold	74521	[A/C]	1067305378
Ca_Kabuli_NW_004516652.1_11017	Ca_kabuli_scaffold	11017	[A/G]	1067305379
Ca_Kabuli_NW_004516654.1_3592	Ca_kabuli_scaffold	3592	[A/T]	1067305380
Ca_Kabuli_NW_004516654.1_3606	Ca_kabuli_scaffold	3606	[T/C]	1067305381
Ca_Kabuli_NW_004516654.1_3758	Ca_kabuli_scaffold	3758	[T/G]	1067305382
Ca_Kabuli_NW_004516654.1_3764	Ca_kabuli_scaffold	3764	[G/T]	1067305383
Ca_Kabuli_NW_004516654.1_3793	Ca_kabuli_scaffold	3793	[G/A]	1067305384
Ca_Kabuli_NW_004516654.1_3800	Ca_kabuli_scaffold	3800	[T/G]	1067305385
Ca_Kabuli_NW_004516666.1_86018	Ca_kabuli_scaffold	86018	[C/T]	1067305386
Ca_Kabuli_NW_004516666.1_86049	Ca_kabuli_scaffold	86049	[G/A]	1067305387
Ca_Kabuli_NW_004516666.1_322287	Ca_kabuli_scaffold	322287	[G/A]	1067305388
Ca_Kabuli_NW_004516666.1_322315	Ca_kabuli_scaffold	322315	[G/T]	1067305389
Ca_Kabuli_NW_004516666.1_365902	Ca_kabuli_scaffold	365902	[A/C]	1067305390
Ca_Kabuli_NW_004516666.1_365910	Ca_kabuli_scaffold	365910	[A/C]	1067305391
Ca_Kabuli_NW_004516677.1_4751	Ca_kabuli_scaffold	4751	[A/T]	1067305392

SNP IDs	Chromosomes	Physical positions (bp)	SNPs	NCBI SNPdb accession IDs (NCBI_ss#)
Ca_Kabuli_NW_004516687.1_81765	Ca_kabuli_scaffold	81765	[C/A]	1067305393
Ca_Kabuli_NW_004516687.1_81779	Ca_kabuli_scaffold	81779	[A/C]	1067305394
Ca_Kabuli_NW_004516690.1_113837	Ca_kabuli_scaffold	113837	[A/G]	1067305395
Ca_Kabuli_NW_004516690.1_113840	Ca_kabuli_scaffold	113840	[A/G]	1067305396
Ca_Kabuli_NW_004516690.1_113857	Ca_kabuli_scaffold	113857	[T/G]	1067305397
Ca_Kabuli_NW_004516690.1_113871	Ca_kabuli_scaffold	113871	[T/C]	1067305398
Ca_Kabuli_NW_004516690.1_113877	Ca_kabuli_scaffold	113877	[A/G]	1067305399
Ca_Kabuli_NW_004516690.1_113887	Ca_kabuli_scaffold	113887	[T/A]	1067305400
Ca_Kabuli_NW_004516690.1_289997	Ca_kabuli_scaffold	289997	[G/T]	1067305401
Ca_Kabuli_NW_004516695.1_160749	Ca_kabuli_scaffold	160749	[T/C]	1067305402
Ca_Kabuli_NW_004516695.1_189700	Ca_kabuli_scaffold	189700	[A/G]	1067305403
Ca_Kabuli_NW_004516695.1_197789	Ca_kabuli_scaffold	197789	[G/A]	1067305404
Ca_Kabuli_NW_004516695.1_197790	Ca_kabuli_scaffold	197790	[G/A]	1067305405
Ca_Kabuli_NW_004516695.1_197819	Ca_kabuli_scaffold	197819	[T/G]	1067305406
Ca_Kabuli_NW_004516696.1_33121	Ca_kabuli_scaffold	33121	[A/C]	1067305407
Ca_Kabuli_NW_004516696.1_33165	Ca_kabuli_scaffold	33165	[A/C]	1067305408
Ca_Kabuli_NW_004516696.1_33188	Ca_kabuli_scaffold	33188	[A/C]	1067305409
Ca_Kabuli_NW_004516696.1_33955	Ca_kabuli_scaffold	33955	[T/G]	1067305410
Ca_Kabuli_NW_004516701.1_92106	Ca_kabuli_scaffold	92106	[C/G]	1067305411
Ca_Kabuli_NW_004516701.1_98231	Ca_kabuli_scaffold	98231	[A/G]	1067305412
Ca_Kabuli_NW_004516709.1_20734	Ca_kabuli_scaffold	20734	[A/C]	1067305413
Ca_Kabuli_NW_004516717.1_10495	Ca_kabuli_scaffold	10495	[C/G]	1067305414
Ca_Kabuli_NW_004516718.1_65715	Ca_kabuli_scaffold	65715	[G/A]	1067305415
Ca_Kabuli_NW_004516724.1_81468	Ca_kabuli_scaffold	81468	[G/C]	1067305416
Ca_Kabuli_NW_004516724.1_81521	Ca_kabuli_scaffold	81521	[G/A]	1067305417
Ca_Kabuli_NW_004516724.1_111497	Ca_kabuli_scaffold	111497	[T/A]	1067305418
Ca_Kabuli_NW_004516724.1_113100	Ca_kabuli_scaffold	113100	[T/G]	1067305419
Ca_Kabuli_NW_004516724.1_113104	Ca_kabuli_scaffold	113104	[T/A]	1067305420
Ca_Kabuli_NW_004516724.1_113109	Ca_kabuli_scaffold	113109	[A/C]	1067305421
Ca_Kabuli_NW_004516724.1_113125	Ca_kabuli_scaffold	113125	[A/T]	1067305422
Ca_Kabuli_NW_004516724.1_113137	Ca_kabuli_scaffold	113137	[A/C]	1067305423
Ca_Kabuli_NW_004516724.1_137569	Ca_kabuli_scaffold	137569	[T/C]	1067305424
Ca_Kabuli_NW_004516724.1_233503	Ca_kabuli_scaffold	233503	[A/G]	1067305425
Ca_Kabuli_NW_004516724.1_233606	Ca_kabuli_scaffold	233606	[A/T]	1067305426

SNP IDs	Chromosomes	Physical positions (bp)	SNPs	NCBI SNPdb accession IDs (NCBI_ss#)
Ca_Kabuli_NW_004516724.1_233613	Ca_kabuli_scaffold	233613	[A/C]	1067305427
Ca_Kabuli_NW_004516724.1_253847	Ca_kabuli_scaffold	253847	[A/G]	1067305428
Ca_Kabuli_NW_004516724.1_310664	Ca_kabuli_scaffold	310664	[A/T]	1067305429
Ca_Kabuli_NW_004516724.1_312266	Ca_kabuli_scaffold	312266	[T/G]	1067305430
Ca_Kabuli_NW_004516724.1_312284	Ca_kabuli_scaffold	312284	[A/T]	1067305431
Ca_Kabuli_NW_004516754.1_23431	Ca_kabuli_scaffold	23431	[G/T]	1067305432
Ca_Kabuli_NW_004516754.1_23433	Ca_kabuli_scaffold	23433	[T/C]	1067305433
Ca_Kabuli_NW_004516754.1_23440	Ca_kabuli_scaffold	23440	[A/T]	1067305434
Ca_Kabuli_NW_004516754.1_24156	Ca_kabuli_scaffold	24156	[A/C]	1067305435
Ca_Kabuli_NW_004516754.1_119677	Ca_kabuli_scaffold	119677	[C/T]	1067305436
Ca_Kabuli_NW_004516754.1_119694	Ca_kabuli_scaffold	119694	[T/G]	1067305437
Ca_Kabuli_NW_004516754.1_119720	Ca_kabuli_scaffold	119720	[C/A]	1067305438
Ca_Kabuli_NW_004516754.1_119721	Ca_kabuli_scaffold	119721	[C/G]	1067305439
Ca_Kabuli_NW_004516754.1_152072	Ca_kabuli_scaffold	152072	[A/C]	1067305440
Ca_Kabuli_NW_004516754.1_188914	Ca_kabuli_scaffold	188914	[A/C]	1067305441
Ca_Kabuli_NW_004516780.1_23445	Ca_kabuli_scaffold	23445	[A/C]	1067305442
Ca_Kabuli_NW_004516783.1_31589	Ca_kabuli_scaffold	31589	[T/C]	1067305443
Ca_Kabuli_NW_004516783.1_202430	Ca_kabuli_scaffold	202430	[A/C]	1067305444
Ca_Kabuli_NW_004516800.1_26433	Ca_kabuli_scaffold	26433	[T/A]	1067305445
Ca_Kabuli_NW_004516804.1_332198	Ca_kabuli_scaffold	332198	[A/C]	1067305446
Ca_Kabuli_NW_004516804.1_332369	Ca_kabuli_scaffold	332369	[C/T]	1067305447
Ca_Kabuli_NW_004516806.1_70487	Ca_kabuli_scaffold	70487	[A/C]	1067305448
Ca_Kabuli_NW_004516808.1_146315	Ca_kabuli_scaffold	146315	[A/C]	1067305449
Ca_Kabuli_NW_004516808.1_150281	Ca_kabuli_scaffold	150281	[G/T]	1067305450
Ca_Kabuli_NW_004516823.1_82859	Ca_kabuli_scaffold	82859	[A/T]	1067305451
Ca_Kabuli_NW_004516823.1_82935	Ca_kabuli_scaffold	82935	[A/C]	1067305452
Ca_Kabuli_NW_004516823.1_82971	Ca_kabuli_scaffold	82971	[T/A]	1067305453
Ca_Kabuli_NW_004516823.1_82974	Ca_kabuli_scaffold	82974	[T/A]	1067305454
Ca_Kabuli_NW_004516834.1_29477	Ca_kabuli_scaffold	29477	[G/A]	1067305455
Ca_Kabuli_NW_004516834.1_29481	Ca_kabuli_scaffold	29481	[T/A]	1067305456
Ca_Kabuli_NW_004516834.1_29483	Ca_kabuli_scaffold	29483	[G/A]	1067305457
Ca_Kabuli_NW_004516834.1_29511	Ca_kabuli_scaffold	29511	[G/A]	1067305458
Ca_Kabuli_NW_004516834.1_29514	Ca_kabuli_scaffold	29514	[C/T]	1067305459
Ca_Kabuli_NW_004516834.1_73620	Ca_kabuli_scaffold	73620	[A/C]	1067305460

SNP IDs	Chromosomes	Physical positions (bp)	SNPs	NCBI SNPdb accession IDs (NCBI_ss#)
Ca_Kabuli_NW_004516836.1_146622	Ca_kabuli_scaffold	146622	[T/G]	1067305461
Ca_Kabuli_NW_004516836.1_146630	Ca_kabuli_scaffold	146630	[T/G]	1067305462
Ca_Kabuli_NW_004516838.1_87052	Ca_kabuli_scaffold	87052	[T/G]	1067305463
Ca_Kabuli_NW_004516838.1_87053	Ca_kabuli_scaffold	87053	[C/A]	1067305464
Ca_Kabuli_NW_004516838.1_87054	Ca_kabuli_scaffold	87054	[T/G]	1067305465
Ca_Kabuli_NW_004516842.1_70605	Ca_kabuli_scaffold	70605	[T/G]	1067305466
Ca_Kabuli_NW_004516847.1_207	Ca_kabuli_scaffold	207	[G/A]	1067305467
Ca_Kabuli_NW_004516850.1_133488	Ca_kabuli_scaffold	133488	[T/C]	1067305468
Ca_Kabuli_NW_004516850.1_133489	Ca_kabuli_scaffold	133489	[G/A]	1067305469
Ca_Kabuli_NW_004516852.1_22370	Ca_kabuli_scaffold	22370	[T/A]	1067305470
Ca_Kabuli_NW_004516863.1_9003	Ca_kabuli_scaffold	9003	[G/A]	1067305471
Ca_Kabuli_NW_004516863.1_9015	Ca_kabuli_scaffold	9015	[C/T]	1067305472
Ca_Kabuli_NW_004516872.1_86066	Ca_kabuli_scaffold	86066	[C/A]	1067305473
Ca_Kabuli_NW_004516872.1_86077	Ca_kabuli_scaffold	86077	[G/A]	1067305474
Ca_Kabuli_NW_004516872.1_86078	Ca_kabuli_scaffold	86078	[G/A]	1067305475
Ca_Kabuli_NW_004516872.1_86084	Ca_kabuli_scaffold	86084	[C/T]	1067305476
Ca_Kabuli_NW_004516872.1_192399	Ca_kabuli_scaffold	192399	[A/C]	1067305477
Ca_Kabuli_NW_004516872.1_192417	Ca_kabuli_scaffold	192417	[C/A]	1067305478
Ca_Kabuli_NW_004516872.1_192713	Ca_kabuli_scaffold	192713	[G/T]	1067305479
Ca_Kabuli_NW_004516872.1_193519	Ca_kabuli_scaffold	193519	[A/T]	1067305480
Ca_Kabuli_NW_004516872.1_194345	Ca_kabuli_scaffold	194345	[C/G]	1067305481
Ca_Kabuli_NW_004516872.1_266559	Ca_kabuli_scaffold	266559	[C/T]	1067305482
Ca_Kabuli_NW_004516872.1_401085	Ca_kabuli_scaffold	401085	[A/T]	1067305483
Ca_Kabuli_NW_004516872.1_502015	Ca_kabuli_scaffold	502015	[A/T]	1067305484
Ca_Kabuli_NW_004516872.1_528896	Ca_kabuli_scaffold	528896	[A/C]	1067305485
Ca_Kabuli_NW_004516872.1_625332	Ca_kabuli_scaffold	625332	[A/C]	1067305486
Ca_Kabuli_NW_004516872.1_625336	Ca_kabuli_scaffold	625336	[A/G]	1067305487
Ca_Kabuli_NW_004516872.1_625365	Ca_kabuli_scaffold	625365	[T/C]	1067305488
Ca_Kabuli_NW_004516872.1_625388	Ca_kabuli_scaffold	625388	[C/T]	1067305489
Ca_Kabuli_NW_004516890.1_120037	Ca_kabuli_scaffold	120037	[A/G]	1067305490
Ca_Kabuli_NW_004516890.1_120040	Ca_kabuli_scaffold	120040	[T/C]	1067305491
Ca_Kabuli_NW_004516890.1_120084	Ca_kabuli_scaffold	120084	[A/G]	1067305492
Ca_Kabuli_NW_004516890.1_120090	Ca_kabuli_scaffold	120090	[T/C]	1067305493
Ca_Kabuli_NW_004516890.1_121557	Ca_kabuli_scaffold	121557	[A/G]	1067305494

SNP IDs	Chromosomes	Physical positions (bp)	SNPs	NCBI SNPdb accession IDs (NCBI_ss#)
Ca_Kabuli_NW_004516890.1_121565	Ca_kabuli_scaffold	121565	[T/G]	1067305495
Ca_Kabuli_NW_004516890.1_164730	Ca_kabuli_scaffold	164730	[T/G]	1067305496
Ca_Kabuli_NW_004516890.1_164769	Ca_kabuli_scaffold	164769	[C/T]	1067305497
Ca_Kabuli_NW_004516890.1_164771	Ca_kabuli_scaffold	164771	[T/C]	1067305498
Ca_Kabuli_NW_004516890.1_281901	Ca_kabuli_scaffold	281901	[T/C]	1067305499
Ca_Kabuli_NW_004516890.1_281915	Ca_kabuli_scaffold	281915	[A/G]	1067305500
Ca_Kabuli_NW_004516914.1_84756	Ca_kabuli_scaffold	84756	[T/G]	1067305501
Ca_Kabuli_NW_004516932.1_7165	Ca_kabuli_scaffold	7165	[G/A]	1067305502
Ca_Kabuli_NW_004516932.1_9398	Ca_kabuli_scaffold	9398	[C/A]	1067305503
Ca_Kabuli_NW_004516932.1_10541	Ca_kabuli_scaffold	10541	[C/G]	1067305504
Ca_Kabuli_NW_004516932.1_10563	Ca_kabuli_scaffold	10563	[T/C]	1067305505
Ca_Kabuli_NW_004516932.1_10925	Ca_kabuli_scaffold	10925	[A/C]	1067305506
Ca_Kabuli_NW_004516932.1_10926	Ca_kabuli_scaffold	10926	[G/T]	1067305507
Ca_Kabuli_NW_004516932.1_10945	Ca_kabuli_scaffold	10945	[T/C]	1067305508
Ca_Kabuli_NW_004516932.1_10950	Ca_kabuli_scaffold	10950	[T/C]	1067305509
Ca_Kabuli_NW_004516932.1_10978	Ca_kabuli_scaffold	10978	[T/C]	1067305510
Ca_Kabuli_NW_004516932.1_10980	Ca_kabuli_scaffold	10980	[A/G]	1067305511
Ca_Kabuli_NW_004516938.1_69848	Ca_kabuli_scaffold	69848	[A/T]	1067305512
Ca_Kabuli_NW_004516939.1_96488	Ca_kabuli_scaffold	96488	[A/G]	1067305513
Ca_Kabuli_NW_004516939.1_101253	Ca_kabuli_scaffold	101253	[A/G]	1067305514
Ca_Kabuli_NW_004516956.1_106332	Ca_kabuli_scaffold	106332	[T/C]	1067305515
Ca_Kabuli_NW_004516957.1_339103	Ca_kabuli_scaffold	339103	[C/A]	1067305516
Ca_Kabuli_NW_004516968.1_40085	Ca_kabuli_scaffold	40085	[C/G]	1067305517
Ca_Kabuli_NW_004516968.1_40089	Ca_kabuli_scaffold	40089	[C/T]	1067305518
Ca_Kabuli_NW_004516995.1_237807	Ca_kabuli_scaffold	237807	[C/T]	1067305519
Ca_Kabuli_NW_004517006.1_50192	Ca_kabuli_scaffold	50192	[C/A]	1067305520
Ca_Kabuli_NW_004517017.1_343519	Ca_kabuli_scaffold	343519	[G/A]	1067305521
Ca_Kabuli_NW_004517017.1_343553	Ca_kabuli_scaffold	343553	[A/G]	1067305522
Ca_Kabuli_NW_004517017.1_349282	Ca_kabuli_scaffold	349282	[A/G]	1067305523
Ca_Kabuli_NW_004517017.1_349284	Ca_kabuli_scaffold	349284	[T/C]	1067305524
Ca_Kabuli_NW_004517030.1_83810	Ca_kabuli_scaffold	83810	[G/A]	1067305525
Ca_Kabuli_NW_004517035.1_127148	Ca_kabuli_scaffold	127148	[A/C]	1067305526
Ca_Kabuli_NW_004517046.1_517	Ca_kabuli_scaffold	517	[G/A]	1067305527
Ca_Kabuli_NW_004517046.1_535	Ca_kabuli_scaffold	535	[T/C]	1067305528

SNP IDs	Chromosomes	Physical positions (bp)	SNPs	NCBI SNPdb accession IDs (NCBI_ss#)
Ca_Kabuli_NW_004517046.1_537	Ca_kabuli_scaffold	537	[T/G]	1067305529
Ca_Kabuli_NW_004517046.1_545	Ca_kabuli_scaffold	545	[C/T]	1067305530
Ca_Kabuli_NW_004517047.1_139066	Ca_kabuli_scaffold	139066	[T/C]	1067305531
Ca_Kabuli_NW_004517047.1_192469	Ca_kabuli_scaffold	192469	[A/G]	1067305532
Ca_Kabuli_NW_004517047.1_241618	Ca_kabuli_scaffold	241618	[T/C]	1067305533
Ca_Kabuli_NW_004517047.1_241697	Ca_kabuli_scaffold	241697	[T/C]	1067305534
Ca_Kabuli_NW_004517047.1_241701	Ca_kabuli_scaffold	241701	[T/C]	1067305535
Ca_Kabuli_NW_004517047.1_241708	Ca_kabuli_scaffold	241708	[T/C]	1067305536
Ca_Kabuli_NW_004517047.1_241754	Ca_kabuli_scaffold	241754	[A/T]	1067305537
Ca_Kabuli_NW_004517047.1_269891	Ca_kabuli_scaffold	269891	[G/T]	1067305538
Ca_Kabuli_NW_004517047.1_328575	Ca_kabuli_scaffold	328575	[A/C]	1067305539
Ca_Kabuli_NW_004517047.1_328816	Ca_kabuli_scaffold	328816	[T/A]	1067305540
Ca_Kabuli_NW_004517047.1_328817	Ca_kabuli_scaffold	328817	[T/G]	1067305541
Ca_Kabuli_NW_004517047.1_328823	Ca_kabuli_scaffold	328823	[T/A]	1067305542
Ca_Kabuli_NW_004517047.1_328828	Ca_kabuli_scaffold	328828	[T/A]	1067305543
Ca_Kabuli_NW_004517047.1_380574	Ca_kabuli_scaffold	380574	[C/A]	1067305544
Ca_Kabuli_NW_004517047.1_380575	Ca_kabuli_scaffold	380575	[A/G]	1067305545
Ca_Kabuli_NW_004517047.1_380576	Ca_kabuli_scaffold	380576	[G/A]	1067305546
Ca_Kabuli_NW_004517047.1_382520	Ca_kabuli_scaffold	382520	[A/G]	1067305547
Ca_Kabuli_NW_004517047.1_384780	Ca_kabuli_scaffold	384780	[T/C]	1067305548
Ca_Kabuli_NW_004517047.1_384837	Ca_kabuli_scaffold	384837	[T/A]	1067305549
Ca_Kabuli_NW_004517047.1_390797	Ca_kabuli_scaffold	390797	[A/C]	1067305550
Ca_Kabuli_NW_004517047.1_390807	Ca_kabuli_scaffold	390807	[C/T]	1067305551
Ca_Kabuli_NW_004517050.1_106	Ca_kabuli_scaffold	106	[T/C]	1067305552
Ca_Kabuli_NW_004517050.1_5648	Ca_kabuli_scaffold	5648	[G/A]	1067305553
Ca_Kabuli_NW_004517050.1_212015	Ca_kabuli_scaffold	212015	[G/A]	1067305554
Ca_Kabuli_NW_004517050.1_215894	Ca_kabuli_scaffold	215894	[T/C]	1067305555
Ca_Kabuli_NW_004517050.1_215903	Ca_kabuli_scaffold	215903	[T/C]	1067305556
Ca_Kabuli_NW_004517050.1_215929	Ca_kabuli_scaffold	215929	[A/G]	1067305557
Ca_Kabuli_NW_004517050.1_215931	Ca_kabuli_scaffold	215931	[A/G]	1067305558
Ca_Kabuli_NW_004517050.1_215942	Ca_kabuli_scaffold	215942	[A/G]	1067305559
Ca_Kabuli_NW_004517088.1_70567	Ca_kabuli_scaffold	70567	[T/C]	1067305560
Ca_Kabuli_NW_004517088.1_71580	Ca_kabuli_scaffold	71580	[C/A]	1067305561
Ca_Kabuli_NW_004517088.1_71581	Ca_kabuli_scaffold	71581	[T/G]	1067305562

SNP IDs	Chromosomes	Physical positions (bp)	SNPs	NCBI SNPdb accession IDs (NCBI_ss#)
Ca_Kabuli_NW_004517088.1_71582	Ca_kabuli_scaffold	71582	[T/A]	1067305563
Ca_Kabuli_NW_004517088.1_111576	Ca_kabuli_scaffold	111576	[A/C]	1067305564
Ca_Kabuli_NW_004517088.1_111596	Ca_kabuli_scaffold	111596	[T/C]	1067305565
Ca_Kabuli_NW_004517088.1_111612	Ca_kabuli_scaffold	111612	[A/G]	1067305566
Ca_Kabuli_NW_004517088.1_111614	Ca_kabuli_scaffold	111614	[A/G]	1067305567
Ca_Kabuli_NW_004517088.1_113856	Ca_kabuli_scaffold	113856	[G/T]	1067305568
Ca_Kabuli_NW_004517093.1_50241	Ca_kabuli_scaffold	50241	[A/G]	1067305569
Ca_Kabuli_NW_004517093.1_122512	Ca_kabuli_scaffold	122512	[T/G]	1067305570
Ca_Kabuli_NW_004517093.1_122526	Ca_kabuli_scaffold	122526	[A/G]	1067305571
Ca_Kabuli_NW_004517093.1_132140	Ca_kabuli_scaffold	132140	[T/C]	1067305572
Ca_Kabuli_NW_004517093.1_132153	Ca_kabuli_scaffold	132153	[T/A]	1067305573
Ca_Kabuli_NW_004517093.1_132165	Ca_kabuli_scaffold	132165	[T/G]	1067305574
Ca_Kabuli_NW_004517093.1_134659	Ca_kabuli_scaffold	134659	[A/T]	1067305575
Ca_Kabuli_NW_004517093.1_134667	Ca_kabuli_scaffold	134667	[T/G]	1067305576
Ca_Kabuli_NW_004517093.1_134670	Ca_kabuli_scaffold	134670	[T/G]	1067305577
Ca_Kabuli_NW_004517096.1_93240	Ca_kabuli_scaffold	93240	[A/G]	1067305578
Ca_Kabuli_NW_004517096.1_226138	Ca_kabuli_scaffold	226138	[T/C]	1067305579
Ca_Kabuli_NW_004517099.1_72632	Ca_kabuli_scaffold	72632	[C/T]	1067305580
Ca_Kabuli_NW_004517099.1_179754	Ca_kabuli_scaffold	179754	[A/G]	1067305581
Ca_Kabuli_NW_004517099.1_179759	Ca_kabuli_scaffold	179759	[T/G]	1067305582
Ca_Kabuli_NW_004517106.1_163331	Ca_kabuli_scaffold	163331	[A/G]	1067305583
Ca_Kabuli_NW_004517141.1_26870	Ca_kabuli_scaffold	26870	[G/A]	1067305584
Ca_Kabuli_NW_004517141.1_26880	Ca_kabuli_scaffold	26880	[C/T]	1067305585
Ca_Kabuli_NW_004517141.1_154212	Ca_kabuli_scaffold	154212	[T/G]	1067305586
Ca_Kabuli_NW_004517142.1_61653	Ca_kabuli_scaffold	61653	[A/C]	1067305587
Ca_Kabuli_NW_004517156.1_14932	Ca_kabuli_scaffold	14932	[A/C]	1067305588
Ca_Kabuli_NW_004517176.1_249375	Ca_kabuli_scaffold	249375	[G/A]	1067305589
Ca_Kabuli_NW_004517182.1_226179	Ca_kabuli_scaffold	226179	[C/A]	1067305590
Ca_Kabuli_NW_004517185.1_68292	Ca_kabuli_scaffold	68292	[G/A]	1067305591
Ca_Kabuli_NW_004517185.1_209695	Ca_kabuli_scaffold	209695	[A/G]	1067305592
Ca_Kabuli_NW_004517185.1_209719	Ca_kabuli_scaffold	209719	[A/C]	1067305593
Ca_Kabuli_NW_004517185.1_209730	Ca_kabuli_scaffold	209730	[A/C]	1067305594
Ca_Kabuli_NW_004517185.1_209757	Ca_kabuli_scaffold	209757	[A/G]	1067305595
Ca_Kabuli_NW_004517185.1_229209	Ca_kabuli_scaffold	229209	[T/C]	1067305596

SNP IDs	Chromosomes	Physical positions (bp)	SNPs	NCBI SNPdb accession IDs (NCBI_ss#)
Ca_Kabuli_NW_004517185.1_229228	Ca_kabuli_scaffold	229228	[T/G]	1067305597
Ca_Kabuli_NW_004517185.1_229235	Ca_kabuli_scaffold	229235	[A/G]	1067305598
Ca_Kabuli_NW_004517188.1_4821	Ca_kabuli_scaffold	4821	[G/A]	1067305599
Ca_Kabuli_NW_004517206.1_35180	Ca_kabuli_scaffold	35180	[T/A]	1067305600
Ca_Kabuli_NW_004517206.1_199116	Ca_kabuli_scaffold	199116	[G/T]	1067305601
Ca_Kabuli_NW_004517206.1_199117	Ca_kabuli_scaffold	199117	[C/G]	1067305602
Ca_Kabuli_NW_004517206.1_199118	Ca_kabuli_scaffold	199118	[T/C]	1067305603
Ca_Kabuli_NW_004517206.1_199119	Ca_kabuli_scaffold	199119	[T/G]	1067305604
Ca_Kabuli_NW_004517206.1_199120	Ca_kabuli_scaffold	199120	[C/A]	1067305605
Ca_Kabuli_NW_004517206.1_199129	Ca_kabuli_scaffold	199129	[T/C]	1067305606
Ca_Kabuli_NW_004517206.1_199169	Ca_kabuli_scaffold	199169	[G/T]	1067305607
Ca_Kabuli_NW_004517206.1_212519	Ca_kabuli_scaffold	212519	[T/A]	1067305608
Ca_Kabuli_NW_004517249.1_5538	Ca_kabuli_scaffold	5538	[A/T]	1067305609
Ca_Kabuli_NW_004517249.1_5562	Ca_kabuli_scaffold	5562	[C/T]	1067305610
Ca_Kabuli_NW_004517249.1_5570	Ca_kabuli_scaffold	5570	[C/T]	1067305611
Ca_Kabuli_NW_004517250.1_4502	Ca_kabuli_scaffold	4502	[A/C]	1067305612
Ca_Kabuli_NW_004517250.1_4510	Ca_kabuli_scaffold	4510	[A/C]	1067305613
Ca_Kabuli_NW_004517264.1_125981	Ca_kabuli_scaffold	125981	[T/G]	1067305614
Ca_Kabuli_NW_004517264.1_125991	Ca_kabuli_scaffold	125991	[A/C]	1067305615
Ca_Kabuli_NW_004517264.1_125997	Ca_kabuli_scaffold	125997	[A/G]	1067305616
Ca_Kabuli_NW_004517264.1_126006	Ca_kabuli_scaffold	126006	[T/C]	1067305617
Ca_Kabuli_NW_004517264.1_126011	Ca_kabuli_scaffold	126011	[T/A]	1067305618
Ca_Kabuli_NW_004517303.1_185	Ca_kabuli_scaffold	185	[C/A]	1067305619
Ca_Kabuli_NW_004517303.1_50671	Ca_kabuli_scaffold	50671	[A/G]	1067305620
Ca_Kabuli_NW_004517303.1_50680	Ca_kabuli_scaffold	50680	[A/C]	1067305621
Ca_Kabuli_NW_004517303.1_50701	Ca_kabuli_scaffold	50701	[T/A]	1067305622
Ca_Kabuli_NW_004517303.1_50704	Ca_kabuli_scaffold	50704	[A/T]	1067305623
Ca_Kabuli_NW_004517303.1_50707	Ca_kabuli_scaffold	50707	[G/A]	1067305624
Ca_Kabuli_NW_004517303.1_50722	Ca_kabuli_scaffold	50722	[A/G]	1067305625
Ca_Kabuli_NW_004517306.1_55438	Ca_kabuli_scaffold	55438	[G/T]	1067305626
Ca_Kabuli_NW_004517306.1_55455	Ca_kabuli_scaffold	55455	[A/C]	1067305627
Ca_Kabuli_NW_004517306.1_65551	Ca_kabuli_scaffold	65551	[T/C]	1067305628
Ca_Kabuli_NW_004517306.1_83254	Ca_kabuli_scaffold	83254	[T/G]	1067305629
Ca_Kabuli_NW_004517320.1_132563	Ca_kabuli_scaffold	132563	[A/C]	1067305630

SNP IDs	Chromosomes	Physical positions (bp)	SNPs	NCBI SNPdb accession IDs (NCBI_ss#)
Ca_Kabuli_NW_004517320.1_132564	Ca_kabuli_scaffold	132564	[G/T]	1067305631
Ca_Kabuli_NW_004517320.1_132568	Ca_kabuli_scaffold	132568	[G/C]	1067305632
Ca_Kabuli_NW_004517320.1_132569	Ca_kabuli_scaffold	132569	[G/A]	1067305633
Ca_Kabuli_NW_004517327.1_124871	Ca_kabuli_scaffold	124871	[A/C]	1067305634
Ca_Kabuli_NW_004517327.1_222808	Ca_kabuli_scaffold	222808	[T/A]	1067305635
Ca_Kabuli_NW_004517327.1_222846	Ca_kabuli_scaffold	222846	[G/A]	1067305636
Ca_Kabuli_NW_004517334.1_30844	Ca_kabuli_scaffold	30844	[G/C]	1067305637
Ca_Kabuli_NW_004517339.1_63215	Ca_kabuli_scaffold	63215	[A/C]	1067305638
Ca_Kabuli_NW_004517339.1_271529	Ca_kabuli_scaffold	271529	[A/C]	1067305639
Ca_Kabuli_NW_004517339.1_321631	Ca_kabuli_scaffold	321631	[A/C]	1067305640
Ca_Kabuli_NW_004517339.1_321637	Ca_kabuli_scaffold	321637	[T/C]	1067305641
Ca_Kabuli_NW_004517341.1_3923	Ca_kabuli_scaffold	3923	[T/C]	1067305642
Ca_Kabuli_NW_004517341.1_3936	Ca_kabuli_scaffold	3936	[A/G]	1067305643
Ca_Kabuli_NW_004517352.1_63722	Ca_kabuli_scaffold	63722	[A/C]	1067305644
Ca_Kabuli_NW_004517356.1_30492	Ca_kabuli_scaffold	30492	[T/A]	1067305645
Ca_Kabuli_NW_004517356.1_30494	Ca_kabuli_scaffold	30494	[A/T]	1067305646
Ca_Kabuli_NW_004517356.1_30529	Ca_kabuli_scaffold	30529	[T/C]	1067305647
Ca_Kabuli_NW_004517356.1_30540	Ca_kabuli_scaffold	30540	[A/G]	1067305648
Ca_Kabuli_NW_004517356.1_33370	Ca_kabuli_scaffold	33370	[A/T]	1067305649
Ca_Kabuli_NW_004517356.1_33413	Ca_kabuli_scaffold	33413	[T/A]	1067305650
Ca_Kabuli_NW_004517356.1_340785	Ca_kabuli_scaffold	340785	[A/G]	1067305651
Ca_Kabuli_NW_004517356.1_340797	Ca_kabuli_scaffold	340797	[T/C]	1067305652
Ca_Kabuli_NW_004517356.1_340801	Ca_kabuli_scaffold	340801	[A/G]	1067305653
Ca_Kabuli_NW_004517356.1_340803	Ca_kabuli_scaffold	340803	[T/G]	1067305654
Ca_Kabuli_NW_004517356.1_340815	Ca_kabuli_scaffold	340815	[T/A]	1067305655
Ca_Kabuli_NW_004517402.1_28507	Ca_kabuli_scaffold	28507	[A/G]	1067305656
Ca_Kabuli_NW_004517402.1_28529	Ca_kabuli_scaffold	28529	[A/C]	1067305657
Ca_Kabuli_NW_004517402.1_28547	Ca_kabuli_scaffold	28547	[G/A]	1067305658
Ca_Kabuli_NW_004517443.1_41511	Ca_kabuli_scaffold	41511	[T/C]	1067305659
Ca_Kabuli_NW_004517447.1_67840	Ca_kabuli_scaffold	67840	[T/G]	1067305660
Ca_Kabuli_NW_004517457.1_37063	Ca_kabuli_scaffold	37063	[T/A]	1067305661
Ca_Kabuli_NW_004517481.1_58079	Ca_kabuli_scaffold	58079	[T/G]	1067305662
Ca_Kabuli_NW_004517481.1_58106	Ca_kabuli_scaffold	58106	[A/C]	1067305663
Ca_Kabuli_NW_004517481.1_88514	Ca_kabuli_scaffold	88514	[A/G]	1067305664

SNP IDs	Chromosomes	Physical positions (bp)	SNPs	NCBI SNPdb accession IDs (NCBI_ss#)
Ca_Kabuli_NW_004517481.1_88521	Ca_kabuli_scaffold	88521	[A/G]	1067305665
Ca_Kabuli_NW_004517481.1_88522	Ca_kabuli_scaffold	88522	[G/A]	1067305666
Ca_Kabuli_NW_004517487.1_58701	Ca_kabuli_scaffold	58701	[A/G]	1067305667
Ca_Kabuli_NW_004517487.1_58707	Ca_kabuli_scaffold	58707	[T/G]	1067305668
Ca_Kabuli_NW_004517487.1_58714	Ca_kabuli_scaffold	58714	[T/G]	1067305669
Ca_Kabuli_NW_004517487.1_58750	Ca_kabuli_scaffold	58750	[T/G]	1067305670
Ca_Kabuli_NW_004517487.1_60138	Ca_kabuli_scaffold	60138	[T/G]	1067305671
Ca_Kabuli_NW_004517487.1_60164	Ca_kabuli_scaffold	60164	[T/G]	1067305672
Ca_Kabuli_NW_004517487.1_101197	Ca_kabuli_scaffold	101197	[A/C]	1067305673
Ca_Kabuli_NW_004517487.1_129934	Ca_kabuli_scaffold	129934	[C/A]	1067305674
Ca_Kabuli_NW_004517487.1_129937	Ca_kabuli_scaffold	129937	[G/C]	1067305675
Ca_Kabuli_NW_004517487.1_129944	Ca_kabuli_scaffold	129944	[T/C]	1067305676
Ca_Kabuli_NW_004517487.1_129950	Ca_kabuli_scaffold	129950	[T/C]	1067305677
Ca_Kabuli_NW_004517487.1_129952	Ca_kabuli_scaffold	129952	[T/G]	1067305678
Ca_Kabuli_NW_004517487.1_129956	Ca_kabuli_scaffold	129956	[T/A]	1067305679
Ca_Kabuli_NW_004517487.1_129963	Ca_kabuli_scaffold	129963	[C/G]	1067305680
Ca_Kabuli_NW_004517520.1_175212	Ca_kabuli_scaffold	175212	[A/G]	1067305681
Ca_Kabuli_NW_004517520.1_463271	Ca_kabuli_scaffold	463271	[T/C]	1067305682
Ca_Kabuli_NW_004517520.1_482352	Ca_kabuli_scaffold	482352	[T/A]	1067305683
Ca_Kabuli_NW_004517520.1_482401	Ca_kabuli_scaffold	482401	[A/C]	1067305684
Ca_Kabuli_NW_004517591.1_180056	Ca_kabuli_scaffold	180056	[A/C]	1067305685
Ca_Kabuli_NW_004517591.1_180060	Ca_kabuli_scaffold	180060	[A/T]	1067305686
Ca_Kabuli_NW_004517592.1_75477	Ca_kabuli_scaffold	75477	[T/G]	1067305687
Ca_Kabuli_NW_004517592.1_75546	Ca_kabuli_scaffold	75546	[T/G]	1067305688
Ca_Kabuli_NW_004517592.1_75593	Ca_kabuli_scaffold	75593	[T/G]	1067305689
Ca_Kabuli_NW_004517592.1_75598	Ca_kabuli_scaffold	75598	[T/G]	1067305690
Ca_Kabuli_NW_004517592.1_75616	Ca_kabuli_scaffold	75616	[T/G]	1067305691
Ca_Kabuli_NW_004517597.1_966	Ca_kabuli_scaffold	966	[A/C]	1067305692
Ca_Kabuli_NW_004517597.1_1172	Ca_kabuli_scaffold	1172	[T/C]	1067305693
Ca_Kabuli_NW_004517624.1_21347	Ca_kabuli_scaffold	21347	[A/C]	1067305694
Ca_Kabuli_NW_004517624.1_24466	Ca_kabuli_scaffold	24466	[T/G]	1067305695
Ca_Kabuli_NW_004517624.1_30420	Ca_kabuli_scaffold	30420	[A/G]	1067305696
Ca_Kabuli_NW_004517624.1_42786	Ca_kabuli_scaffold	42786	[A/G]	1067305697
Ca_Kabuli_NW_004517624.1_43325	Ca_kabuli_scaffold	43325	[A/G]	1067305698

SNP IDs	Chromosomes	Physical positions (bp)	SNPs	NCBI SNPdb accession IDs (NCBI_ss#)
Ca_Kabuli_NW_004517624.1_49533	Ca_kabuli_scaffold	49533	[T/A]	1067305699
Ca_Kabuli_NW_004517624.1_49622	Ca_kabuli_scaffold	49622	[T/G]	1067305700
Ca_Kabuli_NW_004517624.1_49700	Ca_kabuli_scaffold	49700	[G/A]	1067305701
Ca_Kabuli_NW_004517624.1_82199	Ca_kabuli_scaffold	82199	[T/C]	1067305702
Ca_Kabuli_NW_004517624.1_82208	Ca_kabuli_scaffold	82208	[T/C]	1067305703
Ca_Kabuli_NW_004517624.1_82266	Ca_kabuli_scaffold	82266	[A/C]	1067305704
Ca_Kabuli_NW_004517624.1_82284	Ca_kabuli_scaffold	82284	[A/C]	1067305705
Ca_Kabuli_NW_004517624.1_82319	Ca_kabuli_scaffold	82319	[T/C]	1067305706
Ca_Kabuli_NW_004517624.1_82328	Ca_kabuli_scaffold	82328	[A/G]	1067305707
Ca_Kabuli_NW_004517624.1_82342	Ca_kabuli_scaffold	82342	[C/T]	1067305708
Ca_Kabuli_NW_004517624.1_82369	Ca_kabuli_scaffold	82369	[G/A]	1067305709
Ca_Kabuli_NW_004517624.1_82397	Ca_kabuli_scaffold	82397	[T/C]	1067305710
Ca_Kabuli_NW_004517624.1_82417	Ca_kabuli_scaffold	82417	[C/T]	1067305711
Ca_Kabuli_NW_004517624.1_93769	Ca_kabuli_scaffold	93769	[T/C]	1067305712
Ca_Kabuli_NW_004517624.1_94085	Ca_kabuli_scaffold	94085	[C/A]	1067305713
Ca_Kabuli_NW_004517624.1_94245	Ca_kabuli_scaffold	94245	[C/T]	1067305714
Ca_Kabuli_NW_004517624.1_94277	Ca_kabuli_scaffold	94277	[T/C]	1067305715
Ca_Kabuli_NW_004517624.1_96526	Ca_kabuli_scaffold	96526	[T/C]	1067305716
Ca_Kabuli_NW_004517624.1_99364	Ca_kabuli_scaffold	99364	[A/G]	1067305717
Ca_Kabuli_NW_004517624.1_99477	Ca_kabuli_scaffold	99477	[T/A]	1067305718
Ca_Kabuli_NW_004517624.1_99533	Ca_kabuli_scaffold	99533	[T/G]	1067305719
Ca_Kabuli_NW_004517624.1_99544	Ca_kabuli_scaffold	99544	[A/T]	1067305720
Ca_Kabuli_NW_004517624.1_101084	Ca_kabuli_scaffold	101084	[A/C]	1067305721
Ca_Kabuli_NW_004517624.1_101448	Ca_kabuli_scaffold	101448	[A/T]	1067305722
Ca_Kabuli_NW_004517624.1_101490	Ca_kabuli_scaffold	101490	[C/T]	1067305723
Ca_Kabuli_NW_004517624.1_104391	Ca_kabuli_scaffold	104391	[T/G]	1067305724
Ca_Kabuli_NW_004517624.1_104549	Ca_kabuli_scaffold	104549	[C/G]	1067305725
Ca_Kabuli_NW_004517624.1_105126	Ca_kabuli_scaffold	105126	[T/C]	1067305726
Ca_Kabuli_NW_004517624.1_105170	Ca_kabuli_scaffold	105170	[T/C]	1067305727
Ca_Kabuli_NW_004517624.1_106920	Ca_kabuli_scaffold	106920	[T/C]	1067305728
Ca_Kabuli_NW_004517624.1_106936	Ca_kabuli_scaffold	106936	[G/A]	1067305729
Ca_Kabuli_NW_004517624.1_106974	Ca_kabuli_scaffold	106974	[T/C]	1067305730
Ca_Kabuli_NW_004517624.1_107087	Ca_kabuli_scaffold	107087	[G/A]	1067305731
Ca_Kabuli_NW_004517624.1_107311	Ca_kabuli_scaffold	107311	[A/T]	1067305732

SNP IDs	Chromosomes	Physical positions (bp)	SNPs	NCBI SNPdb accession IDs (NCBI_ss#)
Ca_Kabuli_NW_004517624.1_107314	Ca_kabuli_scaffold	107314	[A/C]	1067305733
Ca_Kabuli_NW_004517624.1_107325	Ca_kabuli_scaffold	107325	[A/G]	1067305734
Ca_Kabuli_NW_004517624.1_107345	Ca_kabuli_scaffold	107345	[T/G]	1067305735
Ca_Kabuli_NW_004517624.1_107347	Ca_kabuli_scaffold	107347	[G/T]	1067305736
Ca_Kabuli_NW_004517624.1_107355	Ca_kabuli_scaffold	107355	[G/A]	1067305737
Ca_Kabuli_NW_004517624.1_107376	Ca_kabuli_scaffold	107376	[C/T]	1067305738
Ca_Kabuli_NW_004517624.1_107413	Ca_kabuli_scaffold	107413	[A/G]	1067305739
Ca_Kabuli_NW_004517624.1_107437	Ca_kabuli_scaffold	107437	[T/C]	1067305740
Ca_Kabuli_NW_004517624.1_107486	Ca_kabuli_scaffold	107486	[T/C]	1067305741
Ca_Kabuli_NW_004517624.1_107488	Ca_kabuli_scaffold	107488	[T/C]	1067305742
Ca_Kabuli_NW_004517624.1_108560	Ca_kabuli_scaffold	108560	[T/G]	1067305743
Ca_Kabuli_NW_004517624.1_108599	Ca_kabuli_scaffold	108599	[T/A]	1067305744
Ca_Kabuli_NW_004517624.1_108763	Ca_kabuli_scaffold	108763	[T/G]	1067305745
Ca_Kabuli_NW_004517624.1_112346	Ca_kabuli_scaffold	112346	[T/C]	1067305746
Ca_Kabuli_NW_004517624.1_112359	Ca_kabuli_scaffold	112359	[T/G]	1067305747
Ca_Kabuli_NW_004517624.1_112382	Ca_kabuli_scaffold	112382	[A/C]	1067305748
Ca_Kabuli_NW_004517624.1_112836	Ca_kabuli_scaffold	112836	[C/T]	1067305749
Ca_Kabuli_NW_004517624.1_125716	Ca_kabuli_scaffold	125716	[T/C]	1067305750
Ca_Kabuli_NW_004517624.1_128027	Ca_kabuli_scaffold	128027	[G/A]	1067305751
Ca_Kabuli_NW_004517624.1_133462	Ca_kabuli_scaffold	133462	[G/A]	1067305752
Ca_Kabuli_NW_004517718.1_56358	Ca_kabuli_scaffold	56358	[T/G]	1067305753
Ca_Kabuli_NW_004517746.1_248	Ca_kabuli_scaffold	248	[A/G]	1067305754
Ca_Kabuli_NW_004517746.1_253	Ca_kabuli_scaffold	253	[C/T]	1067305755
Ca_Kabuli_NW_004517746.1_272	Ca_kabuli_scaffold	272	[C/T]	1067305756
Ca_Kabuli_NW_004517766.1_33631	Ca_kabuli_scaffold	33631	[A/G]	1067305757
Ca_Kabuli_NW_004517766.1_33632	Ca_kabuli_scaffold	33632	[G/T]	1067305758
Ca_Kabuli_NW_004517766.1_33655	Ca_kabuli_scaffold	33655	[A/G]	1067305759
Ca_Kabuli_NW_004517767.1_77	Ca_kabuli_scaffold	77	[G/C]	1067305760
Ca_Kabuli_NW_004517767.1_88	Ca_kabuli_scaffold	88	[C/T]	1067305761
Ca_Kabuli_NW_004517767.1_119	Ca_kabuli_scaffold	119	[A/T]	1067305762
Ca_Kabuli_NW_004517767.1_129	Ca_kabuli_scaffold	129	[C/G]	1067305763
Ca_Kabuli_NW_004517773.1_7348	Ca_kabuli_scaffold	7348	[G/A]	1067305764
Ca_Kabuli_NW_004517773.1_7364	Ca_kabuli_scaffold	7364	[C/A]	1067305765
Ca_Kabuli_NW_004517773.1_7377	Ca_kabuli_scaffold	7377	[A/G]	1067305766

SNP IDs	Chromosomes	Physical positions (bp)	SNPs	NCBI SNPdb accession IDs (NCBI_ss#)
Ca_Kabuli_NW_004517815.1_21636	Ca_kabuli_scaffold	21636	[A/C]	1067305767
Ca_Kabuli_NW_004517815.1_33530	Ca_kabuli_scaffold	33530	[G/T]	1067305768
Ca_Kabuli_NW_004517815.1_33532	Ca_kabuli_scaffold	33532	[C/A]	1067305769
Ca_Kabuli_NW_004517815.1_64310	Ca_kabuli_scaffold	64310	[T/G]	1067305770
Ca_Kabuli_NW_004517815.1_64316	Ca_kabuli_scaffold	64316	[T/A]	1067305771
Ca_Kabuli_NW_004517903.1_75955	Ca_kabuli_scaffold	75955	[C/T]	1067305772
Ca_Kabuli_NW_004517917.1_888	Ca_kabuli_scaffold	888	[C/G]	1067305773
Ca_Kabuli_NW_004517917.1_896	Ca_kabuli_scaffold	896	[G/T]	1067305774
Ca_Kabuli_NW_004517917.1_904	Ca_kabuli_scaffold	904	[A/T]	1067305775
Ca_Kabuli_NW_004517929.1_77463	Ca_kabuli_scaffold	77463	[T/C]	1067305776
Ca_Kabuli_NW_004517929.1_146047	Ca_kabuli_scaffold	146047	[T/C]	1067305777
Ca_Kabuli_NW_004517976.1_947	Ca_kabuli_scaffold	947	[C/T]	1067305778
Ca_Kabuli_NW_004517976.1_961	Ca_kabuli_scaffold	961	[C/T]	1067305779
Ca_Kabuli_NW_004517976.1_977	Ca_kabuli_scaffold	977	[C/A]	1067305780
Ca_Kabuli_NW_004517998.1_28221	Ca_kabuli_scaffold	28221	[A/G]	1067305781
Ca_Kabuli_NW_004517998.1_28231	Ca_kabuli_scaffold	28231	[A/G]	1067305782
Ca_Kabuli_NW_004517998.1_28240	Ca_kabuli_scaffold	28240	[A/C]	1067305783
Ca_Kabuli_NW_004517998.1_46413	Ca_kabuli_scaffold	46413	[T/G]	1067305784
Ca_Kabuli_NW_004517998.1_186175	Ca_kabuli_scaffold	186175	[T/A]	1067305785
Ca_Kabuli_NW_004517998.1_186185	Ca_kabuli_scaffold	186185	[A/G]	1067305786
Ca_Kabuli_NW_004517998.1_186189	Ca_kabuli_scaffold	186189	[T/G]	1067305787
Ca_Kabuli_NW_004517998.1_249521	Ca_kabuli_scaffold	249521	[A/T]	1067305788
Ca_Kabuli_NW_004518044.1_17930	Ca_kabuli_scaffold	17930	[T/G]	1067305789
Ca_Kabuli_NW_004518104.1_38566	Ca_kabuli_scaffold	38566	[T/G]	1067305790
Ca_Kabuli_NW_004518104.1_60525	Ca_kabuli_scaffold	60525	[T/C]	1067305791
Ca_Kabuli_NW_004518104.1_60545	Ca_kabuli_scaffold	60545	[T/C]	1067305792
Ca_Kabuli_NW_004518104.1_62768	Ca_kabuli_scaffold	62768	[G/A]	1067305793
Ca_Kabuli_NW_004518104.1_106137	Ca_kabuli_scaffold	106137	[G/A]	1067305794
Ca_Kabuli_NW_004518104.1_106142	Ca_kabuli_scaffold	106142	[C/T]	1067305795
Ca_Kabuli_NW_004518104.1_106174	Ca_kabuli_scaffold	106174	[A/T]	1067305796
Ca_Kabuli_NW_004518104.1_106187	Ca_kabuli_scaffold	106187	[A/T]	1067305797
Ca_Kabuli_NW_004518120.1_93003	Ca_kabuli_scaffold	93003	[A/G]	1067305798
Ca_Kabuli_NW_004518183.1_2902	Ca_kabuli_scaffold	2902	[T/G]	1067305799
Ca_Kabuli_NW_004518201.1_1431	Ca_kabuli_scaffold	1431	[G/A]	1067305800

SNP IDs	Chromosomes	Physical positions (bp)	SNPs	NCBI SNPdb accession IDs (NCBI_ss#)
Ca_Kabuli_NW_004518201.1_1550	Ca_kabuli_scaffold	1550	[T/G]	1067305801
Ca_Kabuli_NW_004518237.1_1651	Ca_kabuli_scaffold	1651	[G/A]	1067305802
Ca_Kabuli_NW_004518354.1_66073	Ca_kabuli_scaffold	66073	[T/C]	1067305803
Ca_Kabuli_NW_004518416.1_5372	Ca_kabuli_scaffold	5372	[G/T]	1067305804
Ca_Kabuli_NW_004518416.1_5385	Ca_kabuli_scaffold	5385	[G/C]	1067305805
Ca_Kabuli_NW_004518445.1_12209	Ca_kabuli_scaffold	12209	[C/G]	1067305806
Ca_Kabuli_NW_004518445.1_12210	Ca_kabuli_scaffold	12210	[C/A]	1067305807
Ca_Kabuli_NW_004518445.1_12212	Ca_kabuli_scaffold	12212	[G/C]	1067305808
Ca_Kabuli_NW_004518445.1_12213	Ca_kabuli_scaffold	12213	[G/T]	1067305809
Ca_Kabuli_NW_004518445.1_65004	Ca_kabuli_scaffold	65004	[G/C]	1067305810
Ca_Kabuli_NW_004518452.1_25070	Ca_kabuli_scaffold	25070	[C/T]	1067305811
Ca_Kabuli_NW_004518483.1_7314	Ca_kabuli_scaffold	7314	[T/C]	1067305812
Ca_Kabuli_NW_004518485.1_2222	Ca_kabuli_scaffold	2222	[A/G]	1067305813
Ca_Kabuli_NW_004518498.1_17198	Ca_kabuli_scaffold	17198	[C/T]	1067305814
Ca_Kabuli_NW_004518498.1_17199	Ca_kabuli_scaffold	17199	[A/G]	1067305815
Ca_Kabuli_NW_004518508.1_1192	Ca_kabuli_scaffold	1192	[G/C]	1067305816
Ca_Kabuli_NW_004518508.1_1194	Ca_kabuli_scaffold	1194	[A/T]	1067305817
Ca_Kabuli_NW_004518508.1_1196	Ca_kabuli_scaffold	1196	[C/T]	1067305818
Ca_Kabuli_NW_004518508.1_1198	Ca_kabuli_scaffold	1198	[C/G]	1067305819
Ca_Kabuli_NW_004518508.1_1201	Ca_kabuli_scaffold	1201	[G/T]	1067305820
Ca_Kabuli_NW_004518508.1_1206	Ca_kabuli_scaffold	1206	[A/C]	1067305821
Ca_Kabuli_NW_004518508.1_1208	Ca_kabuli_scaffold	1208	[A/G]	1067305822
Ca_Kabuli_NW_004518508.1_1214	Ca_kabuli_scaffold	1214	[G/C]	1067305823
Ca_Kabuli_NW_004518508.1_3349	Ca_kabuli_scaffold	3349	[T/G]	1067305824
Ca_Kabuli_NW_004518508.1_3694	Ca_kabuli_scaffold	3694	[A/T]	1067305825
Ca_Kabuli_NW_004518508.1_3716	Ca_kabuli_scaffold	3716	[A/G]	1067305826
Ca_Kabuli_NW_004518508.1_3751	Ca_kabuli_scaffold	3751	[T/A]	1067305827
Ca_Kabuli_NW_004518508.1_3752	Ca_kabuli_scaffold	3752	[G/T]	1067305828
Ca_Kabuli_NW_004518508.1_3753	Ca_kabuli_scaffold	3753	[A/C]	1067305829
Ca_Kabuli_NW_004518508.1_3754	Ca_kabuli_scaffold	3754	[G/T]	1067305830
Ca_Kabuli_NW_004518508.1_3806	Ca_kabuli_scaffold	3806	[C/G]	1067305831
Ca_Kabuli_NW_004518508.1_3807	Ca_kabuli_scaffold	3807	[T/A]	1067305832
Ca_Kabuli_NW_004518508.1_3808	Ca_kabuli_scaffold	3808	[G/T]	1067305833
Ca_Kabuli_NW_004518508.1_3809	Ca_kabuli_scaffold	3809	[T/C]	1067305834

SNP IDs	Chromosomes	Physical positions (bp)	SNPs	NCBI SNPdb accession IDs (NCBI_ss#)
Ca_Kabuli_NW_004518508.1_3810	Ca_kabuli_scaffold	3810	[G/T]	1067305835
Ca_Kabuli_NW_004518508.1_4630	Ca_kabuli_scaffold	4630	[T/G]	1067305836
Ca_Kabuli_NW_004518508.1_6749	Ca_kabuli_scaffold	6749	[A/G]	1067305837
Ca_Kabuli_NW_004518508.1_6776	Ca_kabuli_scaffold	6776	[A/G]	1067305838
Ca_Kabuli_NW_004518508.1_6791	Ca_kabuli_scaffold	6791	[T/A]	1067305839
Ca_Kabuli_NW_004518508.1_6802	Ca_kabuli_scaffold	6802	[G/A]	1067305840
Ca_Kabuli_NW_004518508.1_7058	Ca_kabuli_scaffold	7058	[A/G]	1067305841
Ca_Kabuli_NW_004518508.1_7062	Ca_kabuli_scaffold	7062	[C/T]	1067305842
Ca_Kabuli_NW_004518508.1_7068	Ca_kabuli_scaffold	7068	[T/C]	1067305843
Ca_Kabuli_NW_004518508.1_7088	Ca_kabuli_scaffold	7088	[A/G]	1067305844
Ca_Kabuli_NW_004518508.1_7310	Ca_kabuli_scaffold	7310	[T/G]	1067305845
Ca_Kabuli_NW_004518508.1_7345	Ca_kabuli_scaffold	7345	[G/A]	1067305846
Ca_Kabuli_NW_004518508.1_7346	Ca_kabuli_scaffold	7346	[C/T]	1067305847
Ca_Kabuli_NW_004518508.1_7347	Ca_kabuli_scaffold	7347	[G/A]	1067305848
Ca_Kabuli_NW_004518508.1_7367	Ca_kabuli_scaffold	7367	[T/C]	1067305849
Ca_Kabuli_NW_004518508.1_7375	Ca_kabuli_scaffold	7375	[A/C]	1067305850
Ca_Kabuli_NW_004518508.1_7378	Ca_kabuli_scaffold	7378	[T/C]	1067305851
Ca_Kabuli_NW_004518508.1_11968	Ca_kabuli_scaffold	11968	[C/A]	1067305852
Ca_Kabuli_NW_004518508.1_12034	Ca_kabuli_scaffold	12034	[T/C]	1067305853
Ca_Kabuli_NW_004518508.1_12063	Ca_kabuli_scaffold	12063	[T/G]	1067305854
Ca_Kabuli_NW_004518508.1_12218	Ca_kabuli_scaffold	12218	[A/C]	1067305855
Ca_Kabuli_NW_004518508.1_12231	Ca_kabuli_scaffold	12231	[A/T]	1067305856
Ca_Kabuli_NW_004518508.1_13773	Ca_kabuli_scaffold	13773	[C/T]	1067305857
Ca_Kabuli_NW_004518508.1_13810	Ca_kabuli_scaffold	13810	[G/T]	1067305858
Ca_Kabuli_NW_004518508.1_13811	Ca_kabuli_scaffold	13811	[C/T]	1067305859
Ca_Kabuli_NW_004518508.1_15064	Ca_kabuli_scaffold	15064	[T/G]	1067305860
Ca_Kabuli_NW_004518508.1_15067	Ca_kabuli_scaffold	15067	[T/G]	1067305861
Ca_Kabuli_NW_004518508.1_16936	Ca_kabuli_scaffold	16936	[G/A]	1067305862
Ca_Kabuli_NW_004518508.1_16937	Ca_kabuli_scaffold	16937	[C/A]	1067305863
Ca_Kabuli_NW_004518508.1_16938	Ca_kabuli_scaffold	16938	[C/T]	1067305864
Ca_Kabuli_NW_004518558.1_96007	Ca_kabuli_scaffold	96007	[A/G]	1067305865
Ca_Kabuli_NW_004518558.1_96223	Ca_kabuli_scaffold	96223	[C/G]	1067305866
Ca_Kabuli_NW_004518558.1_148921	Ca_kabuli_scaffold	148921	[A/C]	1067305867
Ca_Kabuli_NW_004518579.1_33391	Ca_kabuli_scaffold	33391	[T/A]	1067305868

SNP IDs	Chromosomes	Physical positions (bp)	SNPs	NCBI SNPdb accession IDs (NCBI_ss#)
Ca_Kabuli_NW_004518589.1_16508	Ca_kabuli_scaffold	16508	[T/G]	1067305869
Ca_Kabuli_NW_004518682.1_13135	Ca_kabuli_scaffold	13135	[C/T]	1067305870
Ca_Kabuli_NW_004518682.1_13153	Ca_kabuli_scaffold	13153	[T/A]	1067305871
Ca_Kabuli_NW_004518682.1_13169	Ca_kabuli_scaffold	13169	[A/C]	1067305872
Ca_Kabuli_NW_004518682.1_13180	Ca_kabuli_scaffold	13180	[T/C]	1067305873
Ca_Kabuli_NW_004518704.1_53441	Ca_kabuli_scaffold	53441	[C/A]	1067305874
Ca_Kabuli_NW_004518704.1_53448	Ca_kabuli_scaffold	53448	[T/C]	1067305875
Ca_Kabuli_NW_004518732.1_7222	Ca_kabuli_scaffold	7222	[A/C]	1067305876
Ca_Kabuli_NW_004518732.1_7237	Ca_kabuli_scaffold	7237	[C/A]	1067305877
Ca_Kabuli_NW_004518732.1_7251	Ca_kabuli_scaffold	7251	[A/G]	1067305878
Ca_Kabuli_NW_004518732.1_7255	Ca_kabuli_scaffold	7255	[T/A]	1067305879
Ca_Kabuli_NW_004518732.1_10304	Ca_kabuli_scaffold	10304	[A/T]	1067305880
Ca_Kabuli_NW_004518830.1_276	Ca_kabuli_scaffold	276	[C/T]	1067305881
Ca_Kabuli_NW_004518856.1_36723	Ca_kabuli_scaffold	36723	[T/A]	1067305882
Ca_Kabuli_NW_004518856.1_36727	Ca_kabuli_scaffold	36727	[A/G]	1067305883
Ca_Kabuli_NW_004518897.1_23636	Ca_kabuli_scaffold	23636	[A/G]	1067305884
Ca_Kabuli_NW_004519037.1_1026	Ca_kabuli_scaffold	1026	[C/A]	1067305885
Ca_Kabuli_NW_004519077.1_16211	Ca_kabuli_scaffold	16211	[T/G]	1067305886
Ca_Kabuli_NW_004519077.1_55292	Ca_kabuli_scaffold	55292	[T/A]	1067305887
Ca_Kabuli_NW_004519077.1_55347	Ca_kabuli_scaffold	55347	[T/G]	1067305888
Ca_Kabuli_NW_004519077.1_64377	Ca_kabuli_scaffold	64377	[A/C]	1067305889
Ca_Kabuli_NW_004519077.1_71640	Ca_kabuli_scaffold	71640	[T/A]	1067305890
Ca_Kabuli_NW_004519077.1_72655	Ca_kabuli_scaffold	72655	[A/C]	1067305891
Ca_Kabuli_NW_004519077.1_106016	Ca_kabuli_scaffold	106016	[A/G]	1067305892
Ca_Kabuli_NW_004519077.1_111989	Ca_kabuli_scaffold	111989	[T/A]	1067305893
Ca_Kabuli_NW_004519081.1_1404	Ca_kabuli_scaffold	1404	[C/A]	1067305894
Ca_Kabuli_NW_004519117.1_6666	Ca_kabuli_scaffold	6666	[C/G]	1067305895
Ca_Kabuli_NW_004519117.1_6693	Ca_kabuli_scaffold	6693	[T/C]	1067305896
Ca_Kabuli_NW_004519117.1_6722	Ca_kabuli_scaffold	6722	[A/C]	1067305897
Ca_Kabuli_NW_004519117.1_6736	Ca_kabuli_scaffold	6736	[G/A]	1067305898
Ca_Kabuli_NW_004519365.1_5908	Ca_kabuli_scaffold	5908	[T/C]	1067305899
Ca_Kabuli_NW_004519449.1_1115	Ca_kabuli_scaffold	1115	[A/T]	1067305900
Ca_Kabuli_NW_004519694.1_15766	Ca_kabuli_scaffold	15766	[T/G]	1067305901
Ca_Kabuli_NW_004519694.1_36309	Ca_kabuli_scaffold	36309	[T/G]	1067305902

SNP IDs	Chromosomes	Physical positions (bp)	SNPs	NCBI SNPdb accession IDs (NCBI_ss#)
Ca_Kabuli_NW_004519694.1_36336	Ca_kabuli_scaffold	36336	[T/G]	1067305903
Ca_Kabuli_NW_004519694.1_36338	Ca_kabuli_scaffold	36338	[T/G]	1067305904
Ca_Kabuli_NW_004519694.1_36368	Ca_kabuli_scaffold	36368	[A/G]	1067305905
Ca_Kabuli_NW_004519745.1_65184	Ca_kabuli_scaffold	65184	[A/G]	1067305906
Ca_Kabuli_NW_004519745.1_65186	Ca_kabuli_scaffold	65186	[T/G]	1067305907
Ca_Kabuli_NW_004519745.1_65198	Ca_kabuli_scaffold	65198	[C/T]	1067305908
Ca_Kabuli_NW_004519745.1_86405	Ca_kabuli_scaffold	86405	[T/G]	1067305909
Ca_Kabuli_NW_004519745.1_86597	Ca_kabuli_scaffold	86597	[A/T]	1067305910
Ca_Kabuli_NW_004519863.1_1146	Ca_kabuli_scaffold	1146	[T/G]	1067305911
Ca_Kabuli_NW_004519863.1_1165	Ca_kabuli_scaffold	1165	[T/C]	1067305912
Ca_Kabuli_NW_004519863.1_1205	Ca_kabuli_scaffold	1205	[T/C]	1067305913
Ca_Kabuli_NW_004519863.1_1214	Ca_kabuli_scaffold	1214	[A/G]	1067305914
Ca_Kabuli_NW_004519863.1_1218	Ca_kabuli_scaffold	1218	[T/C]	1067305915
Ca_Kabuli_NW_004519863.1_1270	Ca_kabuli_scaffold	1270	[C/T]	1067305916
Ca_Kabuli_NW_004519863.1_1284	Ca_kabuli_scaffold	1284	[T/C]	1067305917
Ca_Kabuli_NW_004519863.1_1294	Ca_kabuli_scaffold	1294	[T/C]	1067305918
Ca_Kabuli_NW_004519890.1_697	Ca_kabuli_scaffold	697	[T/C]	1067305919
Ca_Kabuli_NW_004519890.1_725	Ca_kabuli_scaffold	725	[A/C]	1067305920
Ca_Kabuli_NW_004519913.1_8190	Ca_kabuli_scaffold	8190	[T/G]	1067305921
Ca_Kabuli_NW_004519944.1_1007	Ca_kabuli_scaffold	1007	[G/T]	1067305922
Ca_Kabuli_NW_004519944.1_1010	Ca_kabuli_scaffold	1010	[C/T]	1067305923
Ca_Kabuli_NW_004519944.1_1034	Ca_kabuli_scaffold	1034	[C/T]	1067305924
Ca_Kabuli_NW_004519944.1_1038	Ca_kabuli_scaffold	1038	[G/T]	1067305925
Ca_Kabuli_NW_004519971.1_2114	Ca_kabuli_scaffold	2114	[A/T]	1067305926
Ca_Kabuli_NW_004520065.1_542	Ca_kabuli_scaffold	542	[G/A]	1067305927
Ca_Kabuli_NW_004520065.1_567	Ca_kabuli_scaffold	567	[A/T]	1067305928
Ca_Kabuli_NW_004520065.1_576	Ca_kabuli_scaffold	576	[T/C]	1067305929
Ca_Kabuli_NW_004520127.1_6819	Ca_kabuli_scaffold	6819	[A/G]	1067305930
Ca_Kabuli_NW_004520127.1_25710	Ca_kabuli_scaffold	25710	[T/G]	1067305931
Ca_Kabuli_NW_004520127.1_25740	Ca_kabuli_scaffold	25740	[A/C]	1067305932
Ca_Kabuli_NW_004520466.1_195	Ca_kabuli_scaffold	195	[C/T]	1067305933
Ca_Kabuli_NW_004520497.1_23645	Ca_kabuli_scaffold	23645	[T/A]	1067305934
Ca_Kabuli_NW_004520497.1_23661	Ca_kabuli_scaffold	23661	[T/G]	1067305935
Ca_Kabuli_NW_004520497.1_23694	Ca_kabuli_scaffold	23694	[A/G]	1067305936

SNP IDs	Chromosomes	Physical positions (bp)	SNPs	NCBI SNPdb accession IDs (NCBI_ss#)
Ca_Kabuli_NW_004520497.1_43433	Ca_kabuli_scaffold	43433	[A/C]	1067305937
Ca_Kabuli_NW_004520747.1_1686	Ca_kabuli_scaffold	1686	[G/C]	1067305938
Ca_Kabuli_NW_004520754.1_9558	Ca_kabuli_scaffold	9558	[A/G]	1067305939
Ca_Kabuli_NW_004520754.1_9563	Ca_kabuli_scaffold	9563	[A/G]	1067305940
Ca_Kabuli_NW_004520942.1_503	Ca_kabuli_scaffold	503	[C/T]	1067305941
Ca_Kabuli_NW_004520942.1_549	Ca_kabuli_scaffold	549	[G/A]	1067305942
Ca_Kabuli_NW_004520987.1_2577	Ca_kabuli_scaffold	2577	[T/A]	1067305943
Ca_Kabuli_NW_004520987.1_2579	Ca_kabuli_scaffold	2579	[C/T]	1067305944
Ca_Kabuli_NW_004520987.1_2583	Ca_kabuli_scaffold	2583	[C/G]	1067305945
Ca_Kabuli_NW_004520987.1_2625	Ca_kabuli_scaffold	2625	[G/A]	1067305946
Ca_Kabuli_NW_004520988.1_695	Ca_kabuli_scaffold	695	[C/A]	1067305947
Ca_Kabuli_NW_004520988.1_707	Ca_kabuli_scaffold	707	[G/A]	1067305948
Ca_Kabuli_NW_004521072.1_3305	Ca_kabuli_scaffold	3305	[C/T]	1067305949
Ca_Kabuli_NW_004521105.1_287	Ca_kabuli_scaffold	287	[A/G]	1067305950
Ca_Kabuli_NW_004521105.1_323	Ca_kabuli_scaffold	323	[G/T]	1067305951
Ca_Kabuli_NW_004521184.1_6564	Ca_kabuli_scaffold	6564	[G/A]	1067305952
Ca_Kabuli_NW_004521184.1_21590	Ca_kabuli_scaffold	21590	[T/C]	1067305953
Ca_Kabuli_NW_004521184.1_21592	Ca_kabuli_scaffold	21592	[G/T]	1067305954
Ca_Kabuli_NW_004521184.1_21624	Ca_kabuli_scaffold	21624	[G/A]	1067305955
Ca_Kabuli_NW_004521184.1_21649	Ca_kabuli_scaffold	21649	[T/C]	1067305956
Ca_Kabuli_NW_004521204.1_938	Ca_kabuli_scaffold	938	[G/C]	1067305957
Ca_Kabuli_NW_004521340.1_1592	Ca_kabuli_scaffold	1592	[G/A]	1067305958
Ca_Kabuli_NW_004521363.1_4415	Ca_kabuli_scaffold	4415	[A/G]	1067305959
Ca_Kabuli_NW_004521393.1_2056	Ca_kabuli_scaffold	2056	[A/T]	1067305960
Ca_Kabuli_NW_004521407.1_761	Ca_kabuli_scaffold	761	[G/T]	1067305961
Ca_Kabuli_NW_004521407.1_763	Ca_kabuli_scaffold	763	[G/A]	1067305962
Ca_Kabuli_NW_004521679.1_1167	Ca_kabuli_scaffold	1167	[C/T]	1067305963
Ca_Kabuli_NW_004521679.1_1205	Ca_kabuli_scaffold	1205	[A/G]	1067305964
Ca_Kabuli_NW_004521839.1_402	Ca_kabuli_scaffold	402	[G/A]	1067305965
Ca_Kabuli_NW_004521839.1_575	Ca_kabuli_scaffold	575	[C/T]	1067305966
Ca_Kabuli_NW_004521839.1_595	Ca_kabuli_scaffold	595	[T/A]	1067305967
Ca_Kabuli_NW_004521903.1_110	Ca_kabuli_scaffold	110	[T/G]	1067305968
Ca_Kabuli_NW_004521998.1_373	Ca_kabuli_scaffold	373	[T/C]	1067305969
Ca_Kabuli_NW_004521998.1_375	Ca_kabuli_scaffold	375	[G/C]	1067305970

SNP IDs	Chromosomes	Physical positions (bp)	SNPs	NCBI SNPdb accession IDs (NCBI_ss#)
Ca_Kabuli_NW_004521998.1_386	Ca_kabuli_scaffold	386	[G/T]	1067305971
Ca_Kabuli_NW_004521998.1_392	Ca_kabuli_scaffold	392	[T/G]	1067305972
Ca_Kabuli_NW_004521998.1_397	Ca_kabuli_scaffold	397	[G/T]	1067305973
Ca_Kabuli_NW_004521998.1_400	Ca_kabuli_scaffold	400	[C/T]	1067305974
Ca_Kabuli_NW_004521998.1_410	Ca_kabuli_scaffold	410	[A/C]	1067305975
Ca_Kabuli_NW_004521998.1_575	Ca_kabuli_scaffold	575	[A/G]	1067305976
Ca_Kabuli_NW_004522063.1_5406	Ca_kabuli_scaffold	5406	[A/G]	1067305977
Ca_Kabuli_NW_004522064.1_23738	Ca_kabuli_scaffold	23738	[T/G]	1067305978
Ca_Kabuli_NW_004522064.1_23912	Ca_kabuli_scaffold	23912	[T/C]	1067305979
Ca_Kabuli_NW_004522064.1_24906	Ca_kabuli_scaffold	24906	[A/T]	1067305980
Ca_Kabuli_NW_004522064.1_31506	Ca_kabuli_scaffold	31506	[T/A]	1067305981
Ca_Kabuli_NW_004522064.1_31520	Ca_kabuli_scaffold	31520	[T/G]	1067305982
Ca_Kabuli_NW_004522064.1_31785	Ca_kabuli_scaffold	31785	[T/C]	1067305983
Ca_Kabuli_NW_004522064.1_31820	Ca_kabuli_scaffold	31820	[T/C]	1067305984
Ca_Kabuli_NW_004522064.1_31823	Ca_kabuli_scaffold	31823	[A/G]	1067305985
Ca_Kabuli_NW_004522102.1_858	Ca_kabuli_scaffold	858	[A/G]	1067305986
Ca_Kabuli_NW_004522102.1_859	Ca_kabuli_scaffold	859	[C/A]	1067305987
Ca_Kabuli_NW_004522102.1_860	Ca_kabuli_scaffold	860	[G/T]	1067305988
Ca_Kabuli_NW_004522102.1_862	Ca_kabuli_scaffold	862	[G/T]	1067305989
Ca_Kabuli_NW_004522170.1_106	Ca_kabuli_scaffold	106	[T/C]	1067305990
Ca_Kabuli_NW_004522170.1_117	Ca_kabuli_scaffold	117	[T/C]	1067305991
Ca_Kabuli_NW_004522170.1_137	Ca_kabuli_scaffold	137	[A/G]	1067305992
Ca_Kabuli_NW_004522170.1_274	Ca_kabuli_scaffold	274	[T/C]	1067305993
Ca_Kabuli_NW_004522206.1_372	Ca_kabuli_scaffold	372	[T/G]	1067305994
Ca_Kabuli_NW_004522236.1_79	Ca_kabuli_scaffold	79	[T/A]	1067305995
Ca_Kabuli_NW_004522241.1_870	Ca_kabuli_scaffold	870	[A/G]	1067305996
Ca_Kabuli_NW_004522315.1_41	Ca_kabuli_scaffold	41	[A/C]	1067305997
Ca_Kabuli_NW_004522315.1_67	Ca_kabuli_scaffold	67	[A/T]	1067305998
Ca_Kabuli_NW_004522315.1_269	Ca_kabuli_scaffold	269	[T/G]	1067305999
Ca_Kabuli_NW_004522315.1_874	Ca_kabuli_scaffold	874	[T/A]	1067306000
Ca_Kabuli_NW_004522315.1_881	Ca_kabuli_scaffold	881	[T/C]	1067306001
Ca_Kabuli_NW_004522315.1_896	Ca_kabuli_scaffold	896	[C/A]	1067306002
Ca_Kabuli_NW_004522315.1_897	Ca_kabuli_scaffold	897	[C/G]	1067306003
Ca_Kabuli_NW_004522315.1_898	Ca_kabuli_scaffold	898	[T/A]	1067306004

SNP IDs	Chromosomes	Physical positions (bp)	SNPs	NCBI SNPdb accession IDs (NCBI_ss#)
Ca_Kabuli_NW_004522315.1_899	Ca_kabuli_scaffold	899	[A/T]	1067306005
Ca_Kabuli_NW_004522315.1_1242	Ca_kabuli_scaffold	1242	[A/G]	1067306006
Ca_Kabuli_NW_004522315.1_1257	Ca_kabuli_scaffold	1257	[A/C]	1067306007
Ca_Kabuli_NW_004522315.1_1261	Ca_kabuli_scaffold	1261	[T/C]	1067306008
Ca_Kabuli_NW_004522315.1_1267	Ca_kabuli_scaffold	1267	[A/C]	1067306009
Ca_Kabuli_NW_004522325.1_616	Ca_kabuli_scaffold	616	[G/T]	1067306010
Ca_Kabuli_NW_004522325.1_618	Ca_kabuli_scaffold	618	[A/G]	1067306011
Ca_Kabuli_NW_004522394.1_1080	Ca_kabuli_scaffold	1080	[A/T]	1067306012
Ca_Kabuli_NW_004522394.1_1122	Ca_kabuli_scaffold	1122	[A/G]	1067306013
Ca_Kabuli_NW_004522394.1_1137	Ca_kabuli_scaffold	1137	[T/C]	1067306014
Ca_Kabuli_NW_004522394.1_1174	Ca_kabuli_scaffold	1174	[T/C]	1067306015
Ca_Kabuli_NW_004522394.1_1178	Ca_kabuli_scaffold	1178	[T/C]	1067306016
Ca_Kabuli_NW_004522394.1_1184	Ca_kabuli_scaffold	1184	[A/G]	1067306017
Ca_Kabuli_NW_004522394.1_1325	Ca_kabuli_scaffold	1325	[T/C]	1067306018
Ca_Kabuli_NW_004522394.1_1332	Ca_kabuli_scaffold	1332	[T/C]	1067306019
Ca_Kabuli_NW_004522394.1_1405	Ca_kabuli_scaffold	1405	[T/G]	1067306020
Ca_Kabuli_NW_004522396.1_789	Ca_kabuli_scaffold	789	[T/C]	1067306021
Ca_Kabuli_NW_004522396.1_856	Ca_kabuli_scaffold	856	[T/C]	1067306022
Ca_Kabuli_NW_004522401.1_1550	Ca_kabuli_scaffold	1550	[T/G]	1067306023
Ca_Kabuli_NW_004522401.1_1561	Ca_kabuli_scaffold	1561	[G/A]	1067306024
Ca_Kabuli_NW_004522401.1_1562	Ca_kabuli_scaffold	1562	[C/T]	1067306025
Ca_Kabuli_NW_004522401.1_1563	Ca_kabuli_scaffold	1563	[C/T]	1067306026
Ca_Kabuli_NW_004522502.1_1008	Ca_kabuli_scaffold	1008	[A/C]	1067306027
Ca_Kabuli_NW_004522695.1_737	Ca_kabuli_scaffold	737	[T/A]	1067306028
Ca_Kabuli_NW_004522695.1_798	Ca_kabuli_scaffold	798	[A/T]	1067306029
Ca_Kabuli_NW_004522695.1_818	Ca_kabuli_scaffold	818	[A/T]	1067306030
Ca_Kabuli_NW_004522695.1_831	Ca_kabuli_scaffold	831	[A/G]	1067306031
Ca_Kabuli_NW_004522695.1_2088	Ca_kabuli_scaffold	2088	[G/C]	1067306032
Ca_Kabuli_NW_004522695.1_2152	Ca_kabuli_scaffold	2152	[G/C]	1067306033
Ca_Kabuli_NW_004522695.1_2169	Ca_kabuli_scaffold	2169	[T/A]	1067306034
Ca_Kabuli_NW_004522717.1_3414	Ca_kabuli_scaffold	3414	[A/C]	1067306035
Ca_Kabuli_NW_004522717.1_3417	Ca_kabuli_scaffold	3417	[T/G]	1067306036
Ca_Kabuli_NW_004522739.1_1677	Ca_kabuli_scaffold	1677	[A/T]	1067306037
Ca_Kabuli_NW_004522739.1_1681	Ca_kabuli_scaffold	1681	[C/G]	1067306038

SNP IDs	Chromosomes	Physical positions (bp)	SNPs	NCBI SNPdb accession IDs (NCBI_ss#)
Ca_Kabuli_NW_004522739.1_1683	Ca_kabuli_scaffold	1683	[G/A]	1067306039
Ca_Kabuli_NW_004522739.1_1684	Ca_kabuli_scaffold	1684	[G/C]	1067306040
Ca_Kabuli_NW_004522739.1_1685	Ca_kabuli_scaffold	1685	[T/C]	1067306041
Ca_Kabuli_NW_004522739.1_1709	Ca_kabuli_scaffold	1709	[A/G]	1067306042
Ca_Kabuli_NW_004522739.1_1712	Ca_kabuli_scaffold	1712	[A/G]	1067306043
Ca_Kabuli_NW_004522739.1_1718	Ca_kabuli_scaffold	1718	[A/G]	1067306044
Ca_Kabuli_NW_004522743.1_964	Ca_kabuli_scaffold	964	[C/A]	1067306045
Ca_Kabuli_NW_004522743.1_965	Ca_kabuli_scaffold	965	[T/G]	1067306046
Ca_Kabuli_NW_004522743.1_966	Ca_kabuli_scaffold	966	[C/A]	1067306047
Ca_Kabuli_NW_004522749.1_6261	Ca_kabuli_scaffold	6261	[T/A]	1067306048
Ca_Kabuli_NW_004522749.1_6266	Ca_kabuli_scaffold	6266	[T/C]	1067306049
Ca_Kabuli_NW_004522749.1_7714	Ca_kabuli_scaffold	7714	[T/A]	1067306050
Ca_Kabuli_NW_004522749.1_7716	Ca_kabuli_scaffold	7716	[A/C]	1067306051
Ca_Kabuli_NW_004522749.1_7748	Ca_kabuli_scaffold	7748	[A/T]	1067306052
Ca_Kabuli_NW_004522751.1_8303	Ca_kabuli_scaffold	8303	[T/G]	1067306053
Ca_Kabuli_NW_004522751.1_8326	Ca_kabuli_scaffold	8326	[T/G]	1067306054
Ca_Kabuli_NW_004522751.1_8348	Ca_kabuli_scaffold	8348	[A/T]	1067306055
Ca_Kabuli_NW_004522751.1_8351	Ca_kabuli_scaffold	8351	[A/C]	1067306056
Ca_Desi_Chr_1_27204	Ca_desi_chr01	27204	[C/T]	NA
Ca_Desi_Chr_1_152666	Ca_desi_chr01	152666	[A/G]	NA
Ca_Desi_Chr_1_179175	Ca_desi_chr01	179175	[A/C]	NA
Ca_Desi_Chr_1_179181	Ca_desi_chr01	179181	[T/C]	NA
Ca_Desi_Chr_1_179192	Ca_desi_chr01	179192	[T/C]	NA
Ca_Desi_Chr_1_179214	Ca_desi_chr01	179214	[A/C]	NA
Ca_Desi_Chr_1_179225	Ca_desi_chr01	179225	[A/G]	NA
Ca_Desi_Chr_1_404101	Ca_desi_chr01	404101	[A/C]	NA
Ca_Desi_Chr_1_436795	Ca_desi_chr01	436795	[T/C]	NA
Ca_Desi_Chr_1_436816	Ca_desi_chr01	436816	[T/G]	NA
Ca_Desi_Chr_1_436819	Ca_desi_chr01	436819	[A/G]	NA
Ca_Desi_Chr_1_436822	Ca_desi_chr01	436822	[T/G]	NA
Ca_Desi_Chr_1_436847	Ca_desi_chr01	436847	[T/C]	NA
Ca_Desi_Chr_1_436854	Ca_desi_chr01	436854	[A/C]	NA
Ca_Desi_Chr_1_436856	Ca_desi_chr01	436856	[T/G]	NA
Ca_Desi_Chr_1_436875	Ca_desi_chr01	436875	[C/A]	NA

SNP IDs	Chromosomes	Physical positions (bp)	SNPs	NCBI SNPdb accession IDs (NCBI_ss#)
Ca_Desi_Chr_1_436895	Ca_desi_chr01	436895	[A/C]	NA
Ca_Desi_Chr_1_453497	Ca_desi_chr01	453497	[A/G]	NA
Ca_Desi_Chr_1_498877	Ca_desi_chr01	498877	[T/A]	NA
Ca_Desi_Chr_1_498901	Ca_desi_chr01	498901	[A/C]	NA
Ca_Desi_Chr_1_503863	Ca_desi_chr01	503863	[A/C]	NA
Ca_Desi_Chr_1_503871	Ca_desi_chr01	503871	[A/C]	NA
Ca_Desi_Chr_1_527113	Ca_desi_chr01	527113	[T/G]	NA
Ca_Desi_Chr_1_527114	Ca_desi_chr01	527114	[G/T]	NA
Ca_Desi_Chr_1_527115	Ca_desi_chr01	527115	[T/G]	NA
Ca_Desi_Chr_1_531575	Ca_desi_chr01	531575	[C/A]	NA
Ca_Desi_Chr_1_537971	Ca_desi_chr01	537971	[A/C]	NA
Ca_Desi_Chr_1_537978	Ca_desi_chr01	537978	[A/C]	NA
Ca_Desi_Chr_1_548146	Ca_desi_chr01	548146	[A/G]	NA
Ca_Desi_Chr_1_548150	Ca_desi_chr01	548150	[A/G]	NA
Ca_Desi_Chr_1_548152	Ca_desi_chr01	548152	[T/G]	NA
Ca_Desi_Chr_1_548179	Ca_desi_chr01	548179	[A/G]	NA
Ca_Desi_Chr_1_548182	Ca_desi_chr01	548182	[T/C]	NA
Ca_Desi_Chr_1_548186	Ca_desi_chr01	548186	[A/G]	NA
Ca_Desi_Chr_1_548188	Ca_desi_chr01	548188	[A/G]	NA
Ca_Desi_Chr_1_548200	Ca_desi_chr01	548200	[T/C]	NA
Ca_Desi_Chr_1_548203	Ca_desi_chr01	548203	[C/T]	NA
Ca_Desi_Chr_1_551543	Ca_desi_chr01	551543	[A/G]	NA
Ca_Desi_Chr_1_551545	Ca_desi_chr01	551545	[A/G]	NA
Ca_Desi_Chr_1_551557	Ca_desi_chr01	551557	[T/C]	NA
Ca_Desi_Chr_1_558467	Ca_desi_chr01	558467	[A/G]	NA
Ca_Desi_Chr_1_592872	Ca_desi_chr01	592872	[A/C]	NA
Ca_Desi_Chr_1_608899	Ca_desi_chr01	608899	[A/C]	NA
Ca_Desi_Chr_1_643785	Ca_desi_chr01	643785	[A/C]	NA
Ca_Desi_Chr_1_664764	Ca_desi_chr01	664764	[G/A]	NA
Ca_Desi_Chr_1_664769	Ca_desi_chr01	664769	[A/G]	NA
Ca_Desi_Chr_1_674805	Ca_desi_chr01	674805	[A/C]	NA
Ca_Desi_Chr_1_674920	Ca_desi_chr01	674920	[T/G]	NA
Ca_Desi_Chr_1_674965	Ca_desi_chr01	674965	[A/G]	NA
Ca_Desi_Chr_1_675131	Ca_desi_chr01	675131	[A/G]	NA

SNP IDs	Chromosomes	Physical positions (bp)	SNPs	NCBI SNPdb accession IDs (NCBI_ss#)
Ca_Desi_Chr_1_685529	Ca_desi_chr01	685529	[T/A]	NA
Ca_Desi_Chr_1_740370	Ca_desi_chr01	740370	[T/C]	NA
Ca_Desi_Chr_1_757585	Ca_desi_chr01	757585	[A/G]	NA
Ca_Desi_Chr_1_757618	Ca_desi_chr01	757618	[T/G]	NA
Ca_Desi_Chr_1_757623	Ca_desi_chr01	757623	[T/G]	NA
Ca_Desi_Chr_1_766156	Ca_desi_chr01	766156	[T/G]	NA
Ca_Desi_Chr_1_780860	Ca_desi_chr01	780860	[A/G]	NA
Ca_Desi_Chr_1_780869	Ca_desi_chr01	780869	[A/G]	NA
Ca_Desi_Chr_1_780879	Ca_desi_chr01	780879	[T/C]	NA
Ca_Desi_Chr_1_780894	Ca_desi_chr01	780894	[A/G]	NA
Ca_Desi_Chr_1_780898	Ca_desi_chr01	780898	[A/G]	NA
Ca_Desi_Chr_1_780903	Ca_desi_chr01	780903	[T/G]	NA
Ca_Desi_Chr_1_780905	Ca_desi_chr01	780905	[T/C]	NA
Ca_Desi_Chr_1_792132	Ca_desi_chr01	792132	[T/G]	NA
Ca_Desi_Chr_1_799321	Ca_desi_chr01	799321	[T/C]	NA
Ca_Desi_Chr_1_801385	Ca_desi_chr01	801385	[T/G]	NA
Ca_Desi_Chr_1_801409	Ca_desi_chr01	801409	[T/A]	NA
Ca_Desi_Chr_1_815397	Ca_desi_chr01	815397	[G/T]	NA
Ca_Desi_Chr_1_836330	Ca_desi_chr01	836330	[T/G]	NA
Ca_Desi_Chr_1_836351	Ca_desi_chr01	836351	[C/T]	NA
Ca_Desi_Chr_1_836842	Ca_desi_chr01	836842	[T/G]	NA
Ca_Desi_Chr_1_836849	Ca_desi_chr01	836849	[T/G]	NA
Ca_Desi_Chr_1_836859	Ca_desi_chr01	836859	[G/A]	NA
Ca_Desi_Chr_1_840912	Ca_desi_chr01	840912	[T/C]	NA
Ca_Desi_Chr_1_845508	Ca_desi_chr01	845508	[C/T]	NA
Ca_Desi_Chr_1_872247	Ca_desi_chr01	872247	[G/A]	NA
Ca_Desi_Chr_1_872280	Ca_desi_chr01	872280	[C/T]	NA
Ca_Desi_Chr_1_872563	Ca_desi_chr01	872563	[C/G]	NA
Ca_Desi_Chr_1_872564	Ca_desi_chr01	872564	[T/G]	NA
Ca_Desi_Chr_1_886339	Ca_desi_chr01	886339	[G/T]	NA
Ca_Desi_Chr_1_968338	Ca_desi_chr01	968338	[T/G]	NA
Ca_Desi_Chr_1_968392	Ca_desi_chr01	968392	[A/C]	NA
Ca_Desi_Chr_1_973829	Ca_desi_chr01	973829	[T/G]	NA
Ca_Desi_Chr_1_973840	Ca_desi_chr01	973840	[T/G]	NA

SNP IDs	Chromosomes	Physical positions (bp)	SNPs	NCBI SNPdb accession IDs (NCBI_ss#)
Ca_Desi_Chr_1_973854	Ca_desi_chr01	973854	[T/G]	NA
Ca_Desi_Chr_1_973874	Ca_desi_chr01	973874	[A/T]	NA
Ca_Desi_Chr_1_973881	Ca_desi_chr01	973881	[A/G]	NA
Ca_Desi_Chr_1_982879	Ca_desi_chr01	982879	[T/G]	NA
Ca_Desi_Chr_1_982984	Ca_desi_chr01	982984	[T/G]	NA
Ca_Desi_Chr_1_982991	Ca_desi_chr01	982991	[T/G]	NA
Ca_Desi_Chr_1_983014	Ca_desi_chr01	983014	[T/G]	NA
Ca_Desi_Chr_1_983067	Ca_desi_chr01	983067	[T/A]	NA
Ca_Desi_Chr_1_983103	Ca_desi_chr01	983103	[C/A]	NA
Ca_Desi_Chr_1_983241	Ca_desi_chr01	983241	[A/G]	NA
Ca_Desi_Chr_1_999785	Ca_desi_chr01	999785	[T/A]	NA
Ca_Desi_Chr_1_999823	Ca_desi_chr01	999823	[A/T]	NA
Ca_Desi_Chr_1_1025489	Ca_desi_chr01	1025489	[A/C]	NA
Ca_Desi_Chr_1_1025542	Ca_desi_chr01	1025542	[A/G]	NA
Ca_Desi_Chr_1_1025585	Ca_desi_chr01	1025585	[T/C]	NA
Ca_Desi_Chr_1_1025590	Ca_desi_chr01	1025590	[A/G]	NA
Ca_Desi_Chr_1_1025593	Ca_desi_chr01	1025593	[C/A]	NA
Ca_Desi_Chr_1_1069245	Ca_desi_chr01	1069245	[C/T]	NA
Ca_Desi_Chr_1_1069295	Ca_desi_chr01	1069295	[A/C]	NA
Ca_Desi_Chr_1_1069425	Ca_desi_chr01	1069425	[A/C]	NA
Ca_Desi_Chr_1_1069436	Ca_desi_chr01	1069436	[G/A]	NA
Ca_Desi_Chr_1_1072415	Ca_desi_chr01	1072415	[T/C]	NA
Ca_Desi_Chr_1_1072623	Ca_desi_chr01	1072623	[G/A]	NA
Ca_Desi_Chr_1_1072906	Ca_desi_chr01	1072906	[C/T]	NA
Ca_Desi_Chr_1_1072930	Ca_desi_chr01	1072930	[A/G]	NA
Ca_Desi_Chr_1_1072955	Ca_desi_chr01	1072955	[A/G]	NA
Ca_Desi_Chr_1_1072971	Ca_desi_chr01	1072971	[T/G]	NA
Ca_Desi_Chr_1_1072973	Ca_desi_chr01	1072973	[T/C]	NA
Ca_Desi_Chr_1_1072976	Ca_desi_chr01	1072976	[A/G]	NA
Ca_Desi_Chr_1_1105109	Ca_desi_chr01	1105109	[C/T]	NA
Ca_Desi_Chr_1_1146211	Ca_desi_chr01	1146211	[C/T]	NA
Ca_Desi_Chr_1_1146659	Ca_desi_chr01	1146659	[C/T]	NA
Ca_Desi_Chr_1_1158279	Ca_desi_chr01	1158279	[T/G]	NA
Ca_Desi_Chr_1_1158316	Ca_desi_chr01	1158316	[A/G]	NA

SNP IDs	Chromosomes	Physical positions (bp)	SNPs	NCBI SNPdb accession IDs (NCBI_ss#)
Ca_Desi_Chr_1_1276048	Ca_desi_chr01	1276048	[A/G]	NA
Ca_Desi_Chr_1_1276059	Ca_desi_chr01	1276059	[A/G]	NA
Ca_Desi_Chr_1_1276070	Ca_desi_chr01	1276070	[A/G]	NA
Ca_Desi_Chr_1_1374791	Ca_desi_chr01	1374791	[A/G]	NA
Ca_Desi_Chr_1_1375912	Ca_desi_chr01	1375912	[T/G]	NA
Ca_Desi_Chr_1_1375969	Ca_desi_chr01	1375969	[A/C]	NA
Ca_Desi_Chr_1_1375985	Ca_desi_chr01	1375985	[A/C]	NA
Ca_Desi_Chr_1_1375997	Ca_desi_chr01	1375997	[A/C]	NA
Ca_Desi_Chr_1_1389951	Ca_desi_chr01	1389951	[T/C]	NA
Ca_Desi_Chr_1_1390763	Ca_desi_chr01	1390763	[T/C]	NA
Ca_Desi_Chr_1_1390853	Ca_desi_chr01	1390853	[A/G]	NA
Ca_Desi_Chr_1_1451028	Ca_desi_chr01	1451028	[A/C]	NA
Ca_Desi_Chr_1_1463196	Ca_desi_chr01	1463196	[A/G]	NA
Ca_Desi_Chr_1_1463211	Ca_desi_chr01	1463211	[T/G]	NA
Ca_Desi_Chr_1_1463229	Ca_desi_chr01	1463229	[A/C]	NA
Ca_Desi_Chr_1_1463371	Ca_desi_chr01	1463371	[A/C]	NA
Ca_Desi_Chr_1_1479584	Ca_desi_chr01	1479584	[A/G]	NA
Ca_Desi_Chr_1_1479595	Ca_desi_chr01	1479595	[A/C]	NA
Ca_Desi_Chr_1_1484126	Ca_desi_chr01	1484126	[T/C]	NA
Ca_Desi_Chr_1_1486022	Ca_desi_chr01	1486022	[T/C]	NA
Ca_Desi_Chr_1_1486040	Ca_desi_chr01	1486040	[T/C]	NA
Ca_Desi_Chr_1_1486042	Ca_desi_chr01	1486042	[A/G]	NA
Ca_Desi_Chr_1_1486052	Ca_desi_chr01	1486052	[A/G]	NA
Ca_Desi_Chr_1_1486054	Ca_desi_chr01	1486054	[T/G]	NA
Ca_Desi_Chr_1_1486055	Ca_desi_chr01	1486055	[G/A]	NA
Ca_Desi_Chr_1_1486073	Ca_desi_chr01	1486073	[C/A]	NA
Ca_Desi_Chr_1_1486074	Ca_desi_chr01	1486074	[T/G]	NA
Ca_Desi_Chr_1_1553309	Ca_desi_chr01	1553309	[A/G]	NA
Ca_Desi_Chr_1_1553325	Ca_desi_chr01	1553325	[T/C]	NA
Ca_Desi_Chr_1_1600018	Ca_desi_chr01	1600018	[T/G]	NA
Ca_Desi_Chr_1_1600031	Ca_desi_chr01	1600031	[A/T]	NA
Ca_Desi_Chr_1_1600034	Ca_desi_chr01	1600034	[A/G]	NA
Ca_Desi_Chr_1_1600071	Ca_desi_chr01	1600071	[T/C]	NA
Ca_Desi_Chr_1_1600086	Ca_desi_chr01	1600086	[T/A]	NA

SNP IDs	Chromosomes	Physical positions (bp)	SNPs	NCBI SNPdb accession IDs (NCBI_ss#)
Ca_Desi_Chr_1_1601983	Ca_desi_chr01	1601983	[A/G]	NA
Ca_Desi_Chr_1_1601991	Ca_desi_chr01	1601991	[T/C]	NA
Ca_Desi_Chr_1_1602055	Ca_desi_chr01	1602055	[A/C]	NA
Ca_Desi_Chr_1_1609624	Ca_desi_chr01	1609624	[A/G]	NA
Ca_Desi_Chr_1_1609647	Ca_desi_chr01	1609647	[C/A]	NA
Ca_Desi_Chr_1_1632918	Ca_desi_chr01	1632918	[T/G]	NA
Ca_Desi_Chr_1_1632937	Ca_desi_chr01	1632937	[A/G]	NA
Ca_Desi_Chr_1_1632985	Ca_desi_chr01	1632985	[A/C]	NA
Ca_Desi_Chr_1_1712381	Ca_desi_chr01	1712381	[T/G]	NA
Ca_Desi_Chr_1_1727816	Ca_desi_chr01	1727816	[T/C]	NA
Ca_Desi_Chr_1_1727983	Ca_desi_chr01	1727983	[A/C]	NA
Ca_Desi_Chr_1_1746825	Ca_desi_chr01	1746825	[A/G]	NA
Ca_Desi_Chr_1_1746848	Ca_desi_chr01	1746848	[A/C]	NA
Ca_Desi_Chr_1_1746855	Ca_desi_chr01	1746855	[T/C]	NA
Ca_Desi_Chr_1_1746868	Ca_desi_chr01	1746868	[T/G]	NA
Ca_Desi_Chr_1_1746884	Ca_desi_chr01	1746884	[T/C]	NA
Ca_Desi_Chr_1_1746933	Ca_desi_chr01	1746933	[T/G]	NA
Ca_Desi_Chr_1_1747393	Ca_desi_chr01	1747393	[A/C]	NA
Ca_Desi_Chr_1_1815576	Ca_desi_chr01	1815576	[G/T]	NA
Ca_Desi_Chr_1_1826964	Ca_desi_chr01	1826964	[A/C]	NA
Ca_Desi_Chr_1_1838101	Ca_desi_chr01	1838101	[A/G]	NA
Ca_Desi_Chr_1_1838107	Ca_desi_chr01	1838107	[T/C]	NA
Ca_Desi_Chr_1_1838117	Ca_desi_chr01	1838117	[A/G]	NA
Ca_Desi_Chr_1_1838148	Ca_desi_chr01	1838148	[A/G]	NA
Ca_Desi_Chr_1_1838154	Ca_desi_chr01	1838154	[T/A]	NA
Ca_Desi_Chr_1_1842530	Ca_desi_chr01	1842530	[T/G]	NA
Ca_Desi_Chr_1_1842546	Ca_desi_chr01	1842546	[A/G]	NA
Ca_Desi_Chr_1_1845185	Ca_desi_chr01	1845185	[A/G]	NA
Ca_Desi_Chr_1_1906324	Ca_desi_chr01	1906324	[A/C]	NA
Ca_Desi_Chr_1_1906333	Ca_desi_chr01	1906333	[A/C]	NA
Ca_Desi_Chr_1_1906392	Ca_desi_chr01	1906392	[A/T]	NA
Ca_Desi_Chr_1_1913803	Ca_desi_chr01	1913803	[A/T]	NA
Ca_Desi_Chr_1_1913810	Ca_desi_chr01	1913810	[T/C]	NA
Ca_Desi_Chr_1_1913811	Ca_desi_chr01	1913811	[C/T]	NA

SNP IDs	Chromosomes	Physical positions (bp)	SNPs	NCBI SNPdb accession IDs (NCBI_ss#)
Ca_Desi_Chr_1_1913882	Ca_desi_chr01	1913882	[A/C]	NA
Ca_Desi_Chr_1_1931718	Ca_desi_chr01	1931718	[T/C]	NA
Ca_Desi_Chr_1_1931745	Ca_desi_chr01	1931745	[T/G]	NA
Ca_Desi_Chr_1_1931750	Ca_desi_chr01	1931750	[A/G]	NA
Ca_Desi_Chr_1_1936668	Ca_desi_chr01	1936668	[A/C]	NA
Ca_Desi_Chr_1_1936733	Ca_desi_chr01	1936733	[A/G]	NA
Ca_Desi_Chr_1_1937320	Ca_desi_chr01	1937320	[A/C]	NA
Ca_Desi_Chr_1_1937326	Ca_desi_chr01	1937326	[T/C]	NA
Ca_Desi_Chr_1_1937359	Ca_desi_chr01	1937359	[A/G]	NA
Ca_Desi_Chr_1_1938752	Ca_desi_chr01	1938752	[G/T]	NA
Ca_Desi_Chr_1_1938758	Ca_desi_chr01	1938758	[G/T]	NA
Ca_Desi_Chr_1_1938761	Ca_desi_chr01	1938761	[T/G]	NA
Ca_Desi_Chr_1_1938763	Ca_desi_chr01	1938763	[G/A]	NA
Ca_Desi_Chr_1_1938766	Ca_desi_chr01	1938766	[A/G]	NA
Ca_Desi_Chr_1_1938769	Ca_desi_chr01	1938769	[C/G]	NA
Ca_Desi_Chr_1_1938940	Ca_desi_chr01	1938940	[T/G]	NA
Ca_Desi_Chr_1_1960791	Ca_desi_chr01	1960791	[T/C]	NA
Ca_Desi_Chr_1_1960845	Ca_desi_chr01	1960845	[A/G]	NA
Ca_Desi_Chr_1_1960854	Ca_desi_chr01	1960854	[A/G]	NA
Ca_Desi_Chr_1_1960888	Ca_desi_chr01	1960888	[T/A]	NA
Ca_Desi_Chr_1_1960896	Ca_desi_chr01	1960896	[A/C]	NA
Ca_Desi_Chr_1_1961533	Ca_desi_chr01	1961533	[A/C]	NA
Ca_Desi_Chr_1_1971574	Ca_desi_chr01	1971574	[A/C]	NA
Ca_Desi_Chr_1_1971598	Ca_desi_chr01	1971598	[A/C]	NA
Ca_Desi_Chr_1_1982256	Ca_desi_chr01	1982256	[T/C]	NA
Ca_Desi_Chr_1_1982277	Ca_desi_chr01	1982277	[A/C]	NA
Ca_Desi_Chr_1_1982349	Ca_desi_chr01	1982349	[T/C]	NA
Ca_Desi_Chr_1_1982380	Ca_desi_chr01	1982380	[A/G]	NA
Ca_Desi_Chr_1_1982390	Ca_desi_chr01	1982390	[A/G]	NA
Ca_Desi_Chr_1_2021757	Ca_desi_chr01	2021757	[T/C]	NA
Ca_Desi_Chr_1_2021761	Ca_desi_chr01	2021761	[A/G]	NA
Ca_Desi_Chr_1_2021767	Ca_desi_chr01	2021767	[T/C]	NA
Ca_Desi_Chr_1_2021772	Ca_desi_chr01	2021772	[T/G]	NA
Ca_Desi_Chr_1_2021774	Ca_desi_chr01	2021774	[T/C]	NA

SNP IDs	Chromosomes	Physical positions (bp)	SNPs	NCBI SNPdb accession IDs (NCBI_ss#)
Ca_Desi_Chr_1_2021780	Ca_desi_chr01	2021780	[A/G]	NA
Ca_Desi_Chr_1_2021792	Ca_desi_chr01	2021792	[A/G]	NA
Ca_Desi_Chr_1_2021793	Ca_desi_chr01	2021793	[T/C]	NA
Ca_Desi_Chr_1_2021813	Ca_desi_chr01	2021813	[A/G]	NA
Ca_Desi_Chr_1_2021818	Ca_desi_chr01	2021818	[T/C]	NA
Ca_Desi_Chr_1_2021819	Ca_desi_chr01	2021819	[G/A]	NA
Ca_Desi_Chr_1_2067261	Ca_desi_chr01	2067261	[A/C]	NA
Ca_Desi_Chr_1_2084407	Ca_desi_chr01	2084407	[T/C]	NA
Ca_Desi_Chr_1_2084421	Ca_desi_chr01	2084421	[T/G]	NA
Ca_Desi_Chr_1_2084438	Ca_desi_chr01	2084438	[A/C]	NA
Ca_Desi_Chr_1_2084454	Ca_desi_chr01	2084454	[A/G]	NA
Ca_Desi_Chr_1_2084465	Ca_desi_chr01	2084465	[A/G]	NA
Ca_Desi_Chr_1_2084498	Ca_desi_chr01	2084498	[T/G]	NA
Ca_Desi_Chr_1_2084578	Ca_desi_chr01	2084578	[T/C]	NA
Ca_Desi_Chr_1_2084631	Ca_desi_chr01	2084631	[T/A]	NA
Ca_Desi_Chr_1_2084656	Ca_desi_chr01	2084656	[T/C]	NA
Ca_Desi_Chr_1_2084658	Ca_desi_chr01	2084658	[T/C]	NA
Ca_Desi_Chr_1_2184928	Ca_desi_chr01	2184928	[A/T]	NA
Ca_Desi_Chr_1_2184950	Ca_desi_chr01	2184950	[A/T]	NA
Ca_Desi_Chr_1_2184992	Ca_desi_chr01	2184992	[A/C]	NA
Ca_Desi_Chr_1_2184997	Ca_desi_chr01	2184997	[C/T]	NA
Ca_Desi_Chr_1_2184999	Ca_desi_chr01	2184999	[A/T]	NA
Ca_Desi_Chr_1_2185002	Ca_desi_chr01	2185002	[G/T]	NA
Ca_Desi_Chr_1_2185003	Ca_desi_chr01	2185003	[C/T]	NA
Ca_Desi_Chr_1_2185007	Ca_desi_chr01	2185007	[C/A]	NA
Ca_Desi_Chr_1_2185016	Ca_desi_chr01	2185016	[A/G]	NA
Ca_Desi_Chr_1_2188015	Ca_desi_chr01	2188015	[A/C]	NA
Ca_Desi_Chr_1_2188625	Ca_desi_chr01	2188625	[A/G]	NA
Ca_Desi_Chr_1_2213631	Ca_desi_chr01	2213631	[T/C]	NA
Ca_Desi_Chr_1_2213652	Ca_desi_chr01	2213652	[A/G]	NA
Ca_Desi_Chr_1_2213674	Ca_desi_chr01	2213674	[T/C]	NA
Ca_Desi_Chr_1_2216699	Ca_desi_chr01	2216699	[T/G]	NA
Ca_Desi_Chr_1_2216718	Ca_desi_chr01	2216718	[A/T]	NA
Ca_Desi_Chr_1_2241298	Ca_desi_chr01	2241298	[T/A]	NA

SNP IDs	Chromosomes	Physical positions (bp)	SNPs	NCBI SNPdb accession IDs (NCBI_ss#)
Ca_Desi_Chr_1_2249232	Ca_desi_chr01	2249232	[A/G]	NA
Ca_Desi_Chr_1_2263530	Ca_desi_chr01	2263530	[A/G]	NA
Ca_Desi_Chr_1_2263557	Ca_desi_chr01	2263557	[T/G]	NA
Ca_Desi_Chr_1_2269248	Ca_desi_chr01	2269248	[C/G]	NA
Ca_Desi_Chr_1_2281497	Ca_desi_chr01	2281497	[T/G]	NA
Ca_Desi_Chr_1_2281498	Ca_desi_chr01	2281498	[T/G]	NA
Ca_Desi_Chr_1_2281499	Ca_desi_chr01	2281499	[G/T]	NA
Ca_Desi_Chr_1_2281552	Ca_desi_chr01	2281552	[T/C]	NA
Ca_Desi_Chr_1_2281554	Ca_desi_chr01	2281554	[C/T]	NA
Ca_Desi_Chr_1_2320790	Ca_desi_chr01	2320790	[C/T]	NA
Ca_Desi_Chr_1_2322967	Ca_desi_chr01	2322967	[G/T]	NA
Ca_Desi_Chr_1_2327449	Ca_desi_chr01	2327449	[T/A]	NA
Ca_Desi_Chr_1_2335377	Ca_desi_chr01	2335377	[T/G]	NA
Ca_Desi_Chr_1_2335392	Ca_desi_chr01	2335392	[T/G]	NA
Ca_Desi_Chr_1_2335395	Ca_desi_chr01	2335395	[A/G]	NA
Ca_Desi_Chr_1_2349688	Ca_desi_chr01	2349688	[A/C]	NA
Ca_Desi_Chr_1_2356930	Ca_desi_chr01	2356930	[T/C]	NA
Ca_Desi_Chr_1_2356959	Ca_desi_chr01	2356959	[T/G]	NA
Ca_Desi_Chr_1_2410385	Ca_desi_chr01	2410385	[T/C]	NA
Ca_Desi_Chr_1_2417658	Ca_desi_chr01	2417658	[A/G]	NA
Ca_Desi_Chr_1_2417675	Ca_desi_chr01	2417675	[G/A]	NA
Ca_Desi_Chr_1_2417996	Ca_desi_chr01	2417996	[C/T]	NA
Ca_Desi_Chr_1_2418161	Ca_desi_chr01	2418161	[C/A]	NA
Ca_Desi_Chr_1_2418212	Ca_desi_chr01	2418212	[T/A]	NA
Ca_Desi_Chr_1_2418521	Ca_desi_chr01	2418521	[G/A]	NA
Ca_Desi_Chr_1_2418752	Ca_desi_chr01	2418752	[A/T]	NA
Ca_Desi_Chr_1_2418782	Ca_desi_chr01	2418782	[A/C]	NA
Ca_Desi_Chr_1_2436035	Ca_desi_chr01	2436035	[T/C]	NA
Ca_Desi_Chr_1_2437920	Ca_desi_chr01	2437920	[A/C]	NA
Ca_Desi_Chr_1_2437941	Ca_desi_chr01	2437941	[T/G]	NA
Ca_Desi_Chr_1_2437946	Ca_desi_chr01	2437946	[A/G]	NA
Ca_Desi_Chr_1_2437948	Ca_desi_chr01	2437948	[T/G]	NA
Ca_Desi_Chr_1_2438688	Ca_desi_chr01	2438688	[A/T]	NA
Ca_Desi_Chr_1_2438692	Ca_desi_chr01	2438692	[A/G]	NA

SNP IDs	Chromosomes	Physical positions (bp)	SNPs	NCBI SNPdb accession IDs (NCBI_ss#)
Ca_Desi_Chr_1_2438694	Ca_desi_chr01	2438694	[T/C]	NA
Ca_Desi_Chr_1_2438700	Ca_desi_chr01	2438700	[G/T]	NA
Ca_Desi_Chr_1_2438706	Ca_desi_chr01	2438706	[A/G]	NA
Ca_Desi_Chr_1_2438711	Ca_desi_chr01	2438711	[A/G]	NA
Ca_Desi_Chr_1_2438716	Ca_desi_chr01	2438716	[A/C]	NA
Ca_Desi_Chr_1_2438719	Ca_desi_chr01	2438719	[T/A]	NA
Ca_Desi_Chr_1_2438722	Ca_desi_chr01	2438722	[T/C]	NA
Ca_Desi_Chr_1_2438726	Ca_desi_chr01	2438726	[A/G]	NA
Ca_Desi_Chr_1_2438730	Ca_desi_chr01	2438730	[A/G]	NA
Ca_Desi_Chr_1_2438732	Ca_desi_chr01	2438732	[A/G]	NA
Ca_Desi_Chr_1_2438733	Ca_desi_chr01	2438733	[T/A]	NA
Ca_Desi_Chr_1_2438736	Ca_desi_chr01	2438736	[A/T]	NA
Ca_Desi_Chr_1_2449527	Ca_desi_chr01	2449527	[G/A]	NA
Ca_Desi_Chr_1_2471793	Ca_desi_chr01	2471793	[C/T]	NA
Ca_Desi_Chr_1_2471996	Ca_desi_chr01	2471996	[G/A]	NA
Ca_Desi_Chr_1_2491480	Ca_desi_chr01	2491480	[A/T]	NA
Ca_Desi_Chr_1_2491481	Ca_desi_chr01	2491481	[A/C]	NA
Ca_Desi_Chr_1_2491483	Ca_desi_chr01	2491483	[T/A]	NA
Ca_Desi_Chr_1_2491484	Ca_desi_chr01	2491484	[T/A]	NA
Ca_Desi_Chr_1_2504132	Ca_desi_chr01	2504132	[G/A]	NA
Ca_Desi_Chr_1_2536767	Ca_desi_chr01	2536767	[T/C]	NA
Ca_Desi_Chr_1_2536826	Ca_desi_chr01	2536826	[A/C]	NA
Ca_Desi_Chr_1_2546433	Ca_desi_chr01	2546433	[A/C]	NA
Ca_Desi_Chr_1_2546434	Ca_desi_chr01	2546434	[G/T]	NA
Ca_Desi_Chr_1_2619204	Ca_desi_chr01	2619204	[C/T]	NA
Ca_Desi_Chr_1_2637647	Ca_desi_chr01	2637647	[C/G]	NA
Ca_Desi_Chr_1_2637849	Ca_desi_chr01	2637849	[C/T]	NA
Ca_Desi_Chr_1_2637890	Ca_desi_chr01	2637890	[G/C]	NA
Ca_Desi_Chr_1_2824561	Ca_desi_chr01	2824561	[C/A]	NA
Ca_Desi_Chr_1_2824588	Ca_desi_chr01	2824588	[T/C]	NA
Ca_Desi_Chr_1_2824628	Ca_desi_chr01	2824628	[A/C]	NA
Ca_Desi_Chr_1_2888339	Ca_desi_chr01	2888339	[A/G]	NA
Ca_Desi_Chr_1_2888351	Ca_desi_chr01	2888351	[T/C]	NA
Ca_Desi_Chr_1_2958061	Ca_desi_chr01	2958061	[G/A]	NA

SNP IDs	Chromosomes	Physical positions (bp)	SNPs	NCBI SNPdb accession IDs (NCBI_ss#)
Ca_Desi_Chr_1_2961157	Ca_desi_chr01	2961157	[T/G]	NA
Ca_Desi_Chr_1_2961169	Ca_desi_chr01	2961169	[A/G]	NA
Ca_Desi_Chr_1_2965413	Ca_desi_chr01	2965413	[T/A]	NA
Ca_Desi_Chr_1_3038901	Ca_desi_chr01	3038901	[C/A]	NA
Ca_Desi_Chr_1_3083142	Ca_desi_chr01	3083142	[A/G]	NA
Ca_Desi_Chr_1_3098844	Ca_desi_chr01	3098844	[A/G]	NA
Ca_Desi_Chr_1_3099404	Ca_desi_chr01	3099404	[C/G]	NA
Ca_Desi_Chr_1_3151844	Ca_desi_chr01	3151844	[A/T]	NA
Ca_Desi_Chr_1_3151866	Ca_desi_chr01	3151866	[A/C]	NA
Ca_Desi_Chr_1_3151955	Ca_desi_chr01	3151955	[T/G]	NA
Ca_Desi_Chr_1_3152052	Ca_desi_chr01	3152052	[A/T]	NA
Ca_Desi_Chr_1_3191486	Ca_desi_chr01	3191486	[T/C]	NA
Ca_Desi_Chr_1_3191487	Ca_desi_chr01	3191487	[T/C]	NA
Ca_Desi_Chr_1_3191496	Ca_desi_chr01	3191496	[G/T]	NA
Ca_Desi_Chr_1_3191499	Ca_desi_chr01	3191499	[C/T]	NA
Ca_Desi_Chr_1_3216071	Ca_desi_chr01	3216071	[A/T]	NA
Ca_Desi_Chr_1_3216095	Ca_desi_chr01	3216095	[T/G]	NA
Ca_Desi_Chr_1_3220988	Ca_desi_chr01	3220988	[T/A]	NA
Ca_Desi_Chr_1_3307455	Ca_desi_chr01	3307455	[T/A]	NA
Ca_Desi_Chr_1_3328457	Ca_desi_chr01	3328457	[C/A]	NA
Ca_Desi_Chr_1_3328510	Ca_desi_chr01	3328510	[C/A]	NA
Ca_Desi_Chr_1_3328583	Ca_desi_chr01	3328583	[A/C]	NA
Ca_Desi_Chr_1_3328660	Ca_desi_chr01	3328660	[T/C]	NA
Ca_Desi_Chr_1_3328682	Ca_desi_chr01	3328682	[T/G]	NA
Ca_Desi_Chr_1_3328695	Ca_desi_chr01	3328695	[A/C]	NA
Ca_Desi_Chr_1_3328700	Ca_desi_chr01	3328700	[A/T]	NA
Ca_Desi_Chr_1_3370457	Ca_desi_chr01	3370457	[T/C]	NA
Ca_Desi_Chr_1_3370481	Ca_desi_chr01	3370481	[T/A]	NA
Ca_Desi_Chr_1_3370815	Ca_desi_chr01	3370815	[A/C]	NA
Ca_Desi_Chr_1_3370833	Ca_desi_chr01	3370833	[T/C]	NA
Ca_Desi_Chr_1_3370852	Ca_desi_chr01	3370852	[A/G]	NA
Ca_Desi_Chr_1_3372364	Ca_desi_chr01	3372364	[C/T]	NA
Ca_Desi_Chr_1_3372365	Ca_desi_chr01	3372365	[G/C]	NA
Ca_Desi_Chr_1_3372366	Ca_desi_chr01	3372366	[G/T]	NA

SNP IDs	Chromosomes	Physical positions (bp)	SNPs	NCBI SNPdb accession IDs (NCBI_ss#)
Ca_Desi_Chr_1_3425903	Ca_desi_chr01	3425903	[T/G]	NA
Ca_Desi_Chr_1_3436819	Ca_desi_chr01	3436819	[A/G]	NA
Ca_Desi_Chr_1_3440874	Ca_desi_chr01	3440874	[A/G]	NA
Ca_Desi_Chr_1_3545305	Ca_desi_chr01	3545305	[T/G]	NA
Ca_Desi_Chr_1_3545307	Ca_desi_chr01	3545307	[A/G]	NA
Ca_Desi_Chr_1_3545317	Ca_desi_chr01	3545317	[T/A]	NA
Ca_Desi_Chr_1_3545323	Ca_desi_chr01	3545323	[T/G]	NA
Ca_Desi_Chr_1_3545332	Ca_desi_chr01	3545332	[T/G]	NA
Ca_Desi_Chr_1_3545358	Ca_desi_chr01	3545358	[A/C]	NA
Ca_Desi_Chr_1_3570664	Ca_desi_chr01	3570664	[G/A]	NA
Ca_Desi_Chr_1_3570667	Ca_desi_chr01	3570667	[G/A]	NA
Ca_Desi_Chr_1_3570670	Ca_desi_chr01	3570670	[T/A]	NA
Ca_Desi_Chr_1_3570671	Ca_desi_chr01	3570671	[T/G]	NA
Ca_Desi_Chr_1_3674643	Ca_desi_chr01	3674643	[A/G]	NA
Ca_Desi_Chr_1_3674649	Ca_desi_chr01	3674649	[T/C]	NA
Ca_Desi_Chr_1_3674669	Ca_desi_chr01	3674669	[T/C]	NA
Ca_Desi_Chr_1_3675565	Ca_desi_chr01	3675565	[A/C]	NA
Ca_Desi_Chr_1_3675568	Ca_desi_chr01	3675568	[A/G]	NA
Ca_Desi_Chr_1_3675770	Ca_desi_chr01	3675770	[T/C]	NA
Ca_Desi_Chr_1_3675775	Ca_desi_chr01	3675775	[T/G]	NA
Ca_Desi_Chr_1_3675840	Ca_desi_chr01	3675840	[A/G]	NA
Ca_Desi_Chr_1_3675925	Ca_desi_chr01	3675925	[T/C]	NA
Ca_Desi_Chr_1_3740980	Ca_desi_chr01	3740980	[A/G]	NA
Ca_Desi_Chr_1_3742716	Ca_desi_chr01	3742716	[T/G]	NA
Ca_Desi_Chr_1_3742739	Ca_desi_chr01	3742739	[A/G]	NA
Ca_Desi_Chr_1_3742966	Ca_desi_chr01	3742966	[T/C]	NA
Ca_Desi_Chr_1_3742969	Ca_desi_chr01	3742969	[T/G]	NA
Ca_Desi_Chr_1_3742987	Ca_desi_chr01	3742987	[T/C]	NA
Ca_Desi_Chr_1_3743002	Ca_desi_chr01	3743002	[T/C]	NA
Ca_Desi_Chr_1_3743013	Ca_desi_chr01	3743013	[A/G]	NA
Ca_Desi_Chr_1_3743017	Ca_desi_chr01	3743017	[A/G]	NA
Ca_Desi_Chr_1_3758449	Ca_desi_chr01	3758449	[A/G]	NA
Ca_Desi_Chr_1_3771480	Ca_desi_chr01	3771480	[T/G]	NA
Ca_Desi_Chr_1_3771639	Ca_desi_chr01	3771639	[T/A]	NA

SNP IDs	Chromosomes	Physical positions (bp)	SNPs	NCBI SNPdb accession IDs (NCBI_ss#)
Ca_Desi_Chr_1_3771641	Ca_desi_chr01	3771641	[T/C]	NA
Ca_Desi_Chr_1_3771642	Ca_desi_chr01	3771642	[G/T]	NA
Ca_Desi_Chr_1_3783294	Ca_desi_chr01	3783294	[T/G]	NA
Ca_Desi_Chr_1_3783321	Ca_desi_chr01	3783321	[T/G]	NA
Ca_Desi_Chr_1_3804309	Ca_desi_chr01	3804309	[A/C]	NA
Ca_Desi_Chr_1_3844870	Ca_desi_chr01	3844870	[A/T]	NA
Ca_Desi_Chr_1_3844883	Ca_desi_chr01	3844883	[T/G]	NA
Ca_Desi_Chr_1_3859118	Ca_desi_chr01	3859118	[A/C]	NA
Ca_Desi_Chr_1_3887816	Ca_desi_chr01	3887816	[C/A]	NA
Ca_Desi_Chr_1_3887817	Ca_desi_chr01	3887817	[C/G]	NA
Ca_Desi_Chr_1_3996817	Ca_desi_chr01	3996817	[T/G]	NA
Ca_Desi_Chr_1_3996909	Ca_desi_chr01	3996909	[T/G]	NA
Ca_Desi_Chr_1_3996922	Ca_desi_chr01	3996922	[T/G]	NA
Ca_Desi_Chr_1_4001436	Ca_desi_chr01	4001436	[A/T]	NA
Ca_Desi_Chr_1_4002049	Ca_desi_chr01	4002049	[T/A]	NA
Ca_Desi_Chr_1_5004855	Ca_desi_chr01	5004855	[C/A]	NA
Ca_Desi_Chr_1_5004857	Ca_desi_chr01	5004857	[C/G]	NA
Ca_Desi_Chr_1_5014318	Ca_desi_chr01	5014318	[G/T]	NA
Ca_Desi_Chr_1_5022262	Ca_desi_chr01	5022262	[T/G]	NA
Ca_Desi_Chr_1_5050016	Ca_desi_chr01	5050016	[C/T]	NA
Ca_Desi_Chr_1_5050028	Ca_desi_chr01	5050028	[T/G]	NA
Ca_Desi_Chr_1_5050172	Ca_desi_chr01	5050172	[T/C]	NA
Ca_Desi_Chr_1_5056212	Ca_desi_chr01	5056212	[C/T]	NA
Ca_Desi_Chr_1_5059318	Ca_desi_chr01	5059318	[A/T]	NA
Ca_Desi_Chr_1_5059365	Ca_desi_chr01	5059365	[C/A]	NA
Ca_Desi_Chr_1_5059752	Ca_desi_chr01	5059752	[G/A]	NA
Ca_Desi_Chr_1_5061066	Ca_desi_chr01	5061066	[A/T]	NA
Ca_Desi_Chr_1_5061125	Ca_desi_chr01	5061125	[C/G]	NA
Ca_Desi_Chr_1_5061196	Ca_desi_chr01	5061196	[A/G]	NA
Ca_Desi_Chr_1_5094752	Ca_desi_chr01	5094752	[A/G]	NA
Ca_Desi_Chr_1_5094757	Ca_desi_chr01	5094757	[C/G]	NA
Ca_Desi_Chr_1_5138356	Ca_desi_chr01	5138356	[A/C]	NA
Ca_Desi_Chr_1_5138369	Ca_desi_chr01	5138369	[A/C]	NA
Ca_Desi_Chr_1_5161176	Ca_desi_chr01	5161176	[A/C]	NA

SNP IDs	Chromosomes	Physical positions (bp)	SNPs	NCBI SNPdb accession IDs (NCBI_ss#)
Ca_Desi_Chr_1_5161177	Ca_desi_chr01	5161177	[G/C]	NA
Ca_Desi_Chr_1_5161178	Ca_desi_chr01	5161178	[A/G]	NA
Ca_Desi_Chr_1_5161179	Ca_desi_chr01	5161179	[T/A]	NA
Ca_Desi_Chr_1_5161805	Ca_desi_chr01	5161805	[T/G]	NA
Ca_Desi_Chr_1_5197089	Ca_desi_chr01	5197089	[T/C]	NA
Ca_Desi_Chr_1_5197101	Ca_desi_chr01	5197101	[T/C]	NA
Ca_Desi_Chr_1_5197119	Ca_desi_chr01	5197119	[A/G]	NA
Ca_Desi_Chr_1_5197138	Ca_desi_chr01	5197138	[A/G]	NA
Ca_Desi_Chr_1_5197206	Ca_desi_chr01	5197206	[C/A]	NA
Ca_Desi_Chr_1_5197352	Ca_desi_chr01	5197352	[A/T]	NA
Ca_Desi_Chr_1_5197392	Ca_desi_chr01	5197392	[A/T]	NA
Ca_Desi_Chr_1_5197399	Ca_desi_chr01	5197399	[A/G]	NA
Ca_Desi_Chr_1_5197414	Ca_desi_chr01	5197414	[A/C]	NA
Ca_Desi_Chr_1_5197417	Ca_desi_chr01	5197417	[A/C]	NA
Ca_Desi_Chr_1_5197426	Ca_desi_chr01	5197426	[A/G]	NA
Ca_Desi_Chr_1_5197431	Ca_desi_chr01	5197431	[A/G]	NA
Ca_Desi_Chr_1_5197439	Ca_desi_chr01	5197439	[A/G]	NA
Ca_Desi_Chr_1_5207859	Ca_desi_chr01	5207859	[A/C]	NA
Ca_Desi_Chr_1_5207882	Ca_desi_chr01	5207882	[A/T]	NA
Ca_Desi_Chr_1_5207897	Ca_desi_chr01	5207897	[A/T]	NA
Ca_Desi_Chr_1_5238574	Ca_desi_chr01	5238574	[A/G]	NA
Ca_Desi_Chr_1_5270448	Ca_desi_chr01	5270448	[A/G]	NA
Ca_Desi_Chr_1_5270491	Ca_desi_chr01	5270491	[T/A]	NA
Ca_Desi_Chr_1_5270502	Ca_desi_chr01	5270502	[A/G]	NA
Ca_Desi_Chr_1_5292727	Ca_desi_chr01	5292727	[T/G]	NA
Ca_Desi_Chr_1_5325657	Ca_desi_chr01	5325657	[A/T]	NA
Ca_Desi_Chr_1_5325658	Ca_desi_chr01	5325658	[T/C]	NA
Ca_Desi_Chr_1_5325659	Ca_desi_chr01	5325659	[G/T]	NA
Ca_Desi_Chr_1_5418081	Ca_desi_chr01	5418081	[T/C]	NA
Ca_Desi_Chr_1_5428987	Ca_desi_chr01	5428987	[A/C]	NA
Ca_Desi_Chr_1_5428991	Ca_desi_chr01	5428991	[T/G]	NA
Ca_Desi_Chr_1_5428996	Ca_desi_chr01	5428996	[T/C]	NA
Ca_Desi_Chr_1_5429008	Ca_desi_chr01	5429008	[T/G]	NA
Ca_Desi_Chr_1_5429017	Ca_desi_chr01	5429017	[A/G]	NA

SNP IDs	Chromosomes	Physical positions (bp)	SNPs	NCBI SNPdb accession IDs (NCBI_ss#)
Ca_Desi_Chr_1_5446600	Ca_desi_chr01	5446600	[T/A]	NA
Ca_Desi_Chr_1_5498897	Ca_desi_chr01	5498897	[G/A]	NA
Ca_Desi_Chr_1_5498926	Ca_desi_chr01	5498926	[G/A]	NA
Ca_Desi_Chr_1_5536536	Ca_desi_chr01	5536536	[T/C]	NA
Ca_Desi_Chr_1_5537397	Ca_desi_chr01	5537397	[T/A]	NA
Ca_Desi_Chr_1_5605853	Ca_desi_chr01	5605853	[A/G]	NA
Ca_Desi_Chr_1_5605857	Ca_desi_chr01	5605857	[T/G]	NA
Ca_Desi_Chr_1_5605878	Ca_desi_chr01	5605878	[A/G]	NA
Ca_Desi_Chr_1_5605898	Ca_desi_chr01	5605898	[A/T]	NA
Ca_Desi_Chr_1_5605932	Ca_desi_chr01	5605932	[T/G]	NA
Ca_Desi_Chr_1_5631421	Ca_desi_chr01	5631421	[T/C]	NA
Ca_Desi_Chr_1_5631425	Ca_desi_chr01	5631425	[T/G]	NA
Ca_Desi_Chr_1_5631427	Ca_desi_chr01	5631427	[T/G]	NA
Ca_Desi_Chr_1_5631434	Ca_desi_chr01	5631434	[G/A]	NA
Ca_Desi_Chr_1_5645008	Ca_desi_chr01	5645008	[A/C]	NA
Ca_Desi_Chr_1_5645440	Ca_desi_chr01	5645440	[A/G]	NA
Ca_Desi_Chr_1_5645458	Ca_desi_chr01	5645458	[A/G]	NA
Ca_Desi_Chr_1_5676625	Ca_desi_chr01	5676625	[T/G]	NA
Ca_Desi_Chr_1_5704266	Ca_desi_chr01	5704266	[A/C]	NA
Ca_Desi_Chr_1_5745978	Ca_desi_chr01	5745978	[A/G]	NA
Ca_Desi_Chr_1_5775874	Ca_desi_chr01	5775874	[T/C]	NA
Ca_Desi_Chr_1_5776295	Ca_desi_chr01	5776295	[A/G]	NA
Ca_Desi_Chr_1_5776303	Ca_desi_chr01	5776303	[T/A]	NA
Ca_Desi_Chr_1_5776357	Ca_desi_chr01	5776357	[T/C]	NA
Ca_Desi_Chr_1_5776375	Ca_desi_chr01	5776375	[T/G]	NA
Ca_Desi_Chr_1_5778049	Ca_desi_chr01	5778049	[T/G]	NA
Ca_Desi_Chr_1_5778100	Ca_desi_chr01	5778100	[A/G]	NA
Ca_Desi_Chr_1_5807563	Ca_desi_chr01	5807563	[A/G]	NA
Ca_Desi_Chr_1_5807567	Ca_desi_chr01	5807567	[C/G]	NA
Ca_Desi_Chr_1_5807568	Ca_desi_chr01	5807568	[A/G]	NA
Ca_Desi_Chr_1_5808524	Ca_desi_chr01	5808524	[T/G]	NA
Ca_Desi_Chr_1_5854367	Ca_desi_chr01	5854367	[A/G]	NA
Ca_Desi_Chr_1_5854372	Ca_desi_chr01	5854372	[T/C]	NA
Ca_Desi_Chr_1_5854373	Ca_desi_chr01	5854373	[C/T]	NA

SNP IDs	Chromosomes	Physical positions (bp)	SNPs	NCBI SNPdb accession IDs (NCBI_ss#)
Ca_Desi_Chr_1_5897095	Ca_desi_chr01	5897095	[G/T]	NA
Ca_Desi_Chr_1_5923406	Ca_desi_chr01	5923406	[A/G]	NA
Ca_Desi_Chr_1_5923417	Ca_desi_chr01	5923417	[T/C]	NA
Ca_Desi_Chr_1_6037738	Ca_desi_chr01	6037738	[T/G]	NA
Ca_Desi_Chr_1_6037741	Ca_desi_chr01	6037741	[T/G]	NA
Ca_Desi_Chr_1_6037760	Ca_desi_chr01	6037760	[A/G]	NA
Ca_Desi_Chr_1_6085137	Ca_desi_chr01	6085137	[T/C]	NA
Ca_Desi_Chr_1_6107956	Ca_desi_chr01	6107956	[T/C]	NA
Ca_Desi_Chr_1_6107959	Ca_desi_chr01	6107959	[T/A]	NA
Ca_Desi_Chr_1_6107975	Ca_desi_chr01	6107975	[T/C]	NA
Ca_Desi_Chr_1_6107977	Ca_desi_chr01	6107977	[T/C]	NA
Ca_Desi_Chr_1_6107986	Ca_desi_chr01	6107986	[A/G]	NA
Ca_Desi_Chr_1_6107993	Ca_desi_chr01	6107993	[A/C]	NA
Ca_Desi_Chr_1_6108002	Ca_desi_chr01	6108002	[A/T]	NA
Ca_Desi_Chr_1_6108125	Ca_desi_chr01	6108125	[T/C]	NA
Ca_Desi_Chr_1_6108144	Ca_desi_chr01	6108144	[A/C]	NA
Ca_Desi_Chr_1_6108149	Ca_desi_chr01	6108149	[A/C]	NA
Ca_Desi_Chr_1_6108153	Ca_desi_chr01	6108153	[T/C]	NA
Ca_Desi_Chr_1_6108171	Ca_desi_chr01	6108171	[A/G]	NA
Ca_Desi_Chr_1_6112183	Ca_desi_chr01	6112183	[T/C]	NA
Ca_Desi_Chr_1_6112215	Ca_desi_chr01	6112215	[T/C]	NA
Ca_Desi_Chr_1_6112231	Ca_desi_chr01	6112231	[C/A]	NA
Ca_Desi_Chr_1_6141126	Ca_desi_chr01	6141126	[A/C]	NA
Ca_Desi_Chr_1_6141133	Ca_desi_chr01	6141133	[A/C]	NA
Ca_Desi_Chr_1_6149342	Ca_desi_chr01	6149342	[T/G]	NA
Ca_Desi_Chr_1_6149351	Ca_desi_chr01	6149351	[T/G]	NA
Ca_Desi_Chr_1_6151735	Ca_desi_chr01	6151735	[T/C]	NA
Ca_Desi_Chr_1_6152480	Ca_desi_chr01	6152480	[C/T]	NA
Ca_Desi_Chr_1_6255031	Ca_desi_chr01	6255031	[A/C]	NA
Ca_Desi_Chr_1_6255122	Ca_desi_chr01	6255122	[G/C]	NA
Ca_Desi_Chr_1_6265874	Ca_desi_chr01	6265874	[T/C]	NA
Ca_Desi_Chr_1_6265884	Ca_desi_chr01	6265884	[T/C]	NA
Ca_Desi_Chr_1_6265895	Ca_desi_chr01	6265895	[T/G]	NA
Ca_Desi_Chr_1_6265914	Ca_desi_chr01	6265914	[T/G]	NA

SNP IDs	Chromosomes	Physical positions (bp)	SNPs	NCBI SNPdb accession IDs (NCBI_ss#)
Ca_Desi_Chr_1_6309686	Ca_desi_chr01	6309686	[A/G]	NA
Ca_Desi_Chr_1_6309699	Ca_desi_chr01	6309699	[T/A]	NA
Ca_Desi_Chr_1_6314614	Ca_desi_chr01	6314614	[A/T]	NA
Ca_Desi_Chr_1_6314619	Ca_desi_chr01	6314619	[A/G]	NA
Ca_Desi_Chr_1_6314632	Ca_desi_chr01	6314632	[T/C]	NA
Ca_Desi_Chr_1_6314655	Ca_desi_chr01	6314655	[T/G]	NA
Ca_Desi_Chr_1_6314660	Ca_desi_chr01	6314660	[T/G]	NA
Ca_Desi_Chr_1_6318909	Ca_desi_chr01	6318909	[A/G]	NA
Ca_Desi_Chr_1_6319020	Ca_desi_chr01	6319020	[C/T]	NA
Ca_Desi_Chr_1_6319021	Ca_desi_chr01	6319021	[G/C]	NA
Ca_Desi_Chr_1_6319022	Ca_desi_chr01	6319022	[G/T]	NA
Ca_Desi_Chr_1_6319039	Ca_desi_chr01	6319039	[A/C]	NA
Ca_Desi_Chr_1_6319042	Ca_desi_chr01	6319042	[A/C]	NA
Ca_Desi_Chr_1_6333298	Ca_desi_chr01	6333298	[A/C]	NA
Ca_Desi_Chr_1_6333313	Ca_desi_chr01	6333313	[T/C]	NA
Ca_Desi_Chr_1_6333321	Ca_desi_chr01	6333321	[T/C]	NA
Ca_Desi_Chr_1_6333325	Ca_desi_chr01	6333325	[A/G]	NA
Ca_Desi_Chr_1_6333411	Ca_desi_chr01	6333411	[A/C]	NA
Ca_Desi_Chr_1_6333416	Ca_desi_chr01	6333416	[T/G]	NA
Ca_Desi_Chr_1_6333439	Ca_desi_chr01	6333439	[A/C]	NA
Ca_Desi_Chr_1_6379006	Ca_desi_chr01	6379006	[A/G]	NA
Ca_Desi_Chr_1_6379018	Ca_desi_chr01	6379018	[T/C]	NA
Ca_Desi_Chr_1_6379037	Ca_desi_chr01	6379037	[A/G]	NA
Ca_Desi_Chr_1_6379981	Ca_desi_chr01	6379981	[T/G]	NA
Ca_Desi_Chr_1_6733205	Ca_desi_chr01	6733205	[A/C]	NA
Ca_Desi_Chr_1_6747889	Ca_desi_chr01	6747889	[T/C]	NA
Ca_Desi_Chr_1_6747891	Ca_desi_chr01	6747891	[T/C]	NA
Ca_Desi_Chr_1_6750106	Ca_desi_chr01	6750106	[T/C]	NA
Ca_Desi_Chr_1_6752118	Ca_desi_chr01	6752118	[T/C]	NA
Ca_Desi_Chr_1_6798203	Ca_desi_chr01	6798203	[T/C]	NA
Ca_Desi_Chr_1_6798225	Ca_desi_chr01	6798225	[A/G]	NA
Ca_Desi_Chr_1_6835265	Ca_desi_chr01	6835265	[G/T]	NA
Ca_Desi_Chr_1_6835266	Ca_desi_chr01	6835266	[T/C]	NA
Ca_Desi_Chr_1_6835267	Ca_desi_chr01	6835267	[G/T]	NA

SNP IDs	Chromosomes	Physical positions (bp)	SNPs	NCBI SNPdb accession IDs (NCBI_ss#)
Ca_Desi_Chr_1_6836135	Ca_desi_chr01	6836135	[A/T]	NA
Ca_Desi_Chr_1_6859622	Ca_desi_chr01	6859622	[A/G]	NA
Ca_Desi_Chr_1_6859628	Ca_desi_chr01	6859628	[G/A]	NA
Ca_Desi_Chr_1_6859634	Ca_desi_chr01	6859634	[T/C]	NA
Ca_Desi_Chr_1_6859661	Ca_desi_chr01	6859661	[C/A]	NA
Ca_Desi_Chr_1_6862924	Ca_desi_chr01	6862924	[A/C]	NA
Ca_Desi_Chr_1_6918766	Ca_desi_chr01	6918766	[T/A]	NA
Ca_Desi_Chr_1_6918782	Ca_desi_chr01	6918782	[G/T]	NA
Ca_Desi_Chr_1_6918819	Ca_desi_chr01	6918819	[T/A]	NA
Ca_Desi_Chr_1_6945781	Ca_desi_chr01	6945781	[T/C]	NA
Ca_Desi_Chr_1_6945783	Ca_desi_chr01	6945783	[T/C]	NA
Ca_Desi_Chr_1_6945792	Ca_desi_chr01	6945792	[T/C]	NA
Ca_Desi_Chr_1_6945801	Ca_desi_chr01	6945801	[A/C]	NA
Ca_Desi_Chr_1_6945816	Ca_desi_chr01	6945816	[A/G]	NA
Ca_Desi_Chr_1_6945818	Ca_desi_chr01	6945818	[T/C]	NA
Ca_Desi_Chr_1_6945819	Ca_desi_chr01	6945819	[G/T]	NA
Ca_Desi_Chr_1_6945822	Ca_desi_chr01	6945822	[C/T]	NA
Ca_Desi_Chr_1_6946116	Ca_desi_chr01	6946116	[A/T]	NA
Ca_Desi_Chr_1_6956513	Ca_desi_chr01	6956513	[G/A]	NA
Ca_Desi_Chr_1_7013165	Ca_desi_chr01	7013165	[A/G]	NA
Ca_Desi_Chr_1_7013172	Ca_desi_chr01	7013172	[A/G]	NA
Ca_Desi_Chr_1_7013184	Ca_desi_chr01	7013184	[A/G]	NA
Ca_Desi_Chr_1_7013204	Ca_desi_chr01	7013204	[T/G]	NA
Ca_Desi_Chr_1_7013222	Ca_desi_chr01	7013222	[A/G]	NA
Ca_Desi_Chr_1_7013225	Ca_desi_chr01	7013225	[A/T]	NA
Ca_Desi_Chr_1_7143434	Ca_desi_chr01	7143434	[T/C]	NA
Ca_Desi_Chr_1_7194317	Ca_desi_chr01	7194317	[T/C]	NA
Ca_Desi_Chr_1_7194320	Ca_desi_chr01	7194320	[A/G]	NA
Ca_Desi_Chr_1_7194328	Ca_desi_chr01	7194328	[T/G]	NA
Ca_Desi_Chr_1_7194346	Ca_desi_chr01	7194346	[A/T]	NA
Ca_Desi_Chr_1_7253375	Ca_desi_chr01	7253375	[T/G]	NA
Ca_Desi_Chr_1_7253386	Ca_desi_chr01	7253386	[T/G]	NA
Ca_Desi_Chr_1_7255522	Ca_desi_chr01	7255522	[A/C]	NA
Ca_Desi_Chr_1_7282698	Ca_desi_chr01	7282698	[A/G]	NA

SNP IDs	Chromosomes	Physical positions (bp)	SNPs	NCBI SNPdb accession IDs (NCBI_ss#)
Ca_Desi_Chr_1_7282703	Ca_desi_chr01	7282703	[T/G]	NA
Ca_Desi_Chr_1_7383248	Ca_desi_chr01	7383248	[T/G]	NA
Ca_Desi_Chr_1_7383770	Ca_desi_chr01	7383770	[T/G]	NA
Ca_Desi_Chr_1_7383784	Ca_desi_chr01	7383784	[A/G]	NA
Ca_Desi_Chr_1_7452534	Ca_desi_chr01	7452534	[A/T]	NA
Ca_Desi_Chr_1_7452535	Ca_desi_chr01	7452535	[T/A]	NA
Ca_Desi_Chr_1_7452536	Ca_desi_chr01	7452536	[C/A]	NA
Ca_Desi_Chr_1_7452537	Ca_desi_chr01	7452537	[T/G]	NA
Ca_Desi_Chr_1_7489471	Ca_desi_chr01	7489471	[T/A]	NA
Ca_Desi_Chr_1_7489492	Ca_desi_chr01	7489492	[T/A]	NA
Ca_Desi_Chr_1_7489519	Ca_desi_chr01	7489519	[T/A]	NA
Ca_Desi_Chr_1_7606885	Ca_desi_chr01	7606885	[C/G]	NA
Ca_Desi_Chr_1_7606933	Ca_desi_chr01	7606933	[C/T]	NA
Ca_Desi_Chr_1_7632274	Ca_desi_chr01	7632274	[G/T]	NA
Ca_Desi_Chr_1_7632275	Ca_desi_chr01	7632275	[T/A]	NA
Ca_Desi_Chr_1_7632307	Ca_desi_chr01	7632307	[A/G]	NA
Ca_Desi_Chr_1_7632356	Ca_desi_chr01	7632356	[C/T]	NA
Ca_Desi_Chr_1_7665478	Ca_desi_chr01	7665478	[T/A]	NA
Ca_Desi_Chr_1_7669587	Ca_desi_chr01	7669587	[A/G]	NA
Ca_Desi_Chr_1_7674913	Ca_desi_chr01	7674913	[T/C]	NA
Ca_Desi_Chr_1_7674926	Ca_desi_chr01	7674926	[T/G]	NA
Ca_Desi_Chr_1_7712731	Ca_desi_chr01	7712731	[T/C]	NA
Ca_Desi_Chr_1_7712744	Ca_desi_chr01	7712744	[A/T]	NA
Ca_Desi_Chr_1_7917190	Ca_desi_chr01	7917190	[A/T]	NA
Ca_Desi_Chr_1_7940077	Ca_desi_chr01	7940077	[T/A]	NA
Ca_Desi_Chr_1_7940088	Ca_desi_chr01	7940088	[A/C]	NA
Ca_Desi_Chr_1_7940111	Ca_desi_chr01	7940111	[T/G]	NA
Ca_Desi_Chr_1_8219724	Ca_desi_chr01	8219724	[C/T]	NA
Ca_Desi_Chr_1_8221010	Ca_desi_chr01	8221010	[A/C]	NA
Ca_Desi_Chr_1_8232979	Ca_desi_chr01	8232979	[A/G]	NA
Ca_Desi_Chr_1_8232988	Ca_desi_chr01	8232988	[A/G]	NA
Ca_Desi_Chr_1_8232993	Ca_desi_chr01	8232993	[A/G]	NA
Ca_Desi_Chr_1_8232998	Ca_desi_chr01	8232998	[T/G]	NA
Ca_Desi_Chr_1_8233002	Ca_desi_chr01	8233002	[T/A]	NA

SNP IDs	Chromosomes	Physical positions (bp)	SNPs	NCBI SNPdb accession IDs (NCBI_ss#)
Ca_Desi_Chr_1_8233006	Ca_desi_chr01	8233006	[T/C]	NA
Ca_Desi_Chr_1_8276418	Ca_desi_chr01	8276418	[T/C]	NA
Ca_Desi_Chr_1_8276422	Ca_desi_chr01	8276422	[T/C]	NA
Ca_Desi_Chr_1_8276424	Ca_desi_chr01	8276424	[A/G]	NA
Ca_Desi_Chr_1_8276426	Ca_desi_chr01	8276426	[T/G]	NA
Ca_Desi_Chr_1_8276440	Ca_desi_chr01	8276440	[A/G]	NA
Ca_Desi_Chr_1_8276478	Ca_desi_chr01	8276478	[A/G]	NA
Ca_Desi_Chr_1_8318581	Ca_desi_chr01	8318581	[T/G]	NA
Ca_Desi_Chr_1_8351754	Ca_desi_chr01	8351754	[A/G]	NA
Ca_Desi_Chr_1_8351766	Ca_desi_chr01	8351766	[A/C]	NA
Ca_Desi_Chr_1_8351802	Ca_desi_chr01	8351802	[A/G]	NA
Ca_Desi_Chr_1_8351809	Ca_desi_chr01	8351809	[A/G]	NA
Ca_Desi_Chr_1_8365693	Ca_desi_chr01	8365693	[T/A]	NA
Ca_Desi_Chr_1_8372866	Ca_desi_chr01	8372866	[A/C]	NA
Ca_Desi_Chr_1_8375608	Ca_desi_chr01	8375608	[A/G]	NA
Ca_Desi_Chr_1_8375630	Ca_desi_chr01	8375630	[T/G]	NA
Ca_Desi_Chr_1_8390584	Ca_desi_chr01	8390584	[A/C]	NA
Ca_Desi_Chr_1_8398205	Ca_desi_chr01	8398205	[A/C]	NA
Ca_Desi_Chr_1_8416742	Ca_desi_chr01	8416742	[T/C]	NA
Ca_Desi_Chr_1_8416760	Ca_desi_chr01	8416760	[A/C]	NA
Ca_Desi_Chr_1_8738253	Ca_desi_chr01	8738253	[T/G]	NA
Ca_Desi_Chr_1_8785536	Ca_desi_chr01	8785536	[A/G]	NA
Ca_Desi_Chr_1_8786915	Ca_desi_chr01	8786915	[T/G]	NA
Ca_Desi_Chr_1_8871880	Ca_desi_chr01	8871880	[T/C]	NA
Ca_Desi_Chr_1_8871912	Ca_desi_chr01	8871912	[T/G]	NA
Ca_Desi_Chr_1_8902146	Ca_desi_chr01	8902146	[C/A]	NA
Ca_Desi_Chr_1_8902154	Ca_desi_chr01	8902154	[C/T]	NA
Ca_Desi_Chr_1_8934250	Ca_desi_chr01	8934250	[T/C]	NA
Ca_Desi_Chr_1_8973166	Ca_desi_chr01	8973166	[T/C]	NA
Ca_Desi_Chr_1_8988073	Ca_desi_chr01	8988073	[G/A]	NA
Ca_Desi_Chr_1_8988083	Ca_desi_chr01	8988083	[C/T]	NA
Ca_Desi_Chr_1_8988088	Ca_desi_chr01	8988088	[A/C]	NA
Ca_Desi_Chr_1_8988091	Ca_desi_chr01	8988091	[G/C]	NA
Ca_Desi_Chr_1_8988092	Ca_desi_chr01	8988092	[T/C]	NA

SNP IDs	Chromosomes	Physical positions (bp)	SNPs	NCBI SNPdb accession IDs (NCBI_ss#)
Ca_Desi_Chr_1_9022980	Ca_desi_chr01	9022980	[T/C]	NA
Ca_Desi_Chr_1_9022983	Ca_desi_chr01	9022983	[A/G]	NA
Ca_Desi_Chr_1_9022987	Ca_desi_chr01	9022987	[T/C]	NA
Ca_Desi_Chr_1_9023008	Ca_desi_chr01	9023008	[T/C]	NA
Ca_Desi_Chr_1_9023018	Ca_desi_chr01	9023018	[A/G]	NA
Ca_Desi_Chr_1_9023283	Ca_desi_chr01	9023283	[A/C]	NA
Ca_Desi_Chr_1_9028906	Ca_desi_chr01	9028906	[A/T]	NA
Ca_Desi_Chr_1_9029900	Ca_desi_chr01	9029900	[A/G]	NA
Ca_Desi_Chr_1_9030074	Ca_desi_chr01	9030074	[A/C]	NA
Ca_Desi_Chr_1_9193853	Ca_desi_chr01	9193853	[A/G]	NA
Ca_Desi_Chr_1_9193872	Ca_desi_chr01	9193872	[T/G]	NA
Ca_Desi_Chr_1_9193878	Ca_desi_chr01	9193878	[T/G]	NA
Ca_Desi_Chr_1_9216572	Ca_desi_chr01	9216572	[T/C]	NA
Ca_Desi_Chr_1_9216596	Ca_desi_chr01	9216596	[A/C]	NA
Ca_Desi_Chr_1_9216611	Ca_desi_chr01	9216611	[A/C]	NA
Ca_Desi_Chr_1_9217172	Ca_desi_chr01	9217172	[A/G]	NA
Ca_Desi_Chr_1_9217272	Ca_desi_chr01	9217272	[A/C]	NA
Ca_Desi_Chr_1_9217363	Ca_desi_chr01	9217363	[C/A]	NA
Ca_Desi_Chr_1_9217425	Ca_desi_chr01	9217425	[A/C]	NA
Ca_Desi_Chr_1_9217558	Ca_desi_chr01	9217558	[A/C]	NA
Ca_Desi_Chr_1_9217567	Ca_desi_chr01	9217567	[A/C]	NA
Ca_Desi_Chr_1_9217931	Ca_desi_chr01	9217931	[A/C]	NA
Ca_Desi_Chr_1_9217933	Ca_desi_chr01	9217933	[A/G]	NA
Ca_Desi_Chr_1_9218868	Ca_desi_chr01	9218868	[A/G]	NA
Ca_Desi_Chr_1_9242309	Ca_desi_chr01	9242309	[T/A]	NA
Ca_Desi_Chr_1_9265798	Ca_desi_chr01	9265798	[G/T]	NA
Ca_Desi_Chr_1_9297444	Ca_desi_chr01	9297444	[T/G]	NA
Ca_Desi_Chr_1_9297467	Ca_desi_chr01	9297467	[G/T]	NA
Ca_Desi_Chr_1_9350681	Ca_desi_chr01	9350681	[A/C]	NA
Ca_Desi_Chr_1_9356038	Ca_desi_chr01	9356038	[A/C]	NA
Ca_Desi_Chr_1_9356060	Ca_desi_chr01	9356060	[T/A]	NA
Ca_Desi_Chr_1_9361622	Ca_desi_chr01	9361622	[T/C]	NA
Ca_Desi_Chr_1_9362439	Ca_desi_chr01	9362439	[A/C]	NA
Ca_Desi_Chr_1_9362450	Ca_desi_chr01	9362450	[A/C]	NA

SNP IDs	Chromosomes	Physical positions (bp)	SNPs	NCBI SNPdb accession IDs (NCBI_ss#)
Ca_Desi_Chr_1_9362470	Ca_desi_chr01	9362470	[A/C]	NA
Ca_Desi_Chr_1_9743611	Ca_desi_chr01	9743611	[G/T]	NA
Ca_Desi_Chr_1_9808324	Ca_desi_chr01	9808324	[C/A]	NA
Ca_Desi_Chr_1_9808325	Ca_desi_chr01	9808325	[C/G]	NA
Ca_Desi_Chr_1_9808326	Ca_desi_chr01	9808326	[T/A]	NA
Ca_Desi_Chr_1_10012838	Ca_desi_chr01	10012838	[A/C]	NA
Ca_Desi_Chr_1_10039233	Ca_desi_chr01	10039233	[T/C]	NA
Ca_Desi_Chr_1_10039245	Ca_desi_chr01	10039245	[A/C]	NA
Ca_Desi_Chr_1_10076230	Ca_desi_chr01	10076230	[C/A]	NA
Ca_Desi_Chr_1_10076231	Ca_desi_chr01	10076231	[C/T]	NA
Ca_Desi_Chr_1_10076232	Ca_desi_chr01	10076232	[G/C]	NA
Ca_Desi_Chr_1_10076233	Ca_desi_chr01	10076233	[G/T]	NA
Ca_Desi_Chr_1_10102257	Ca_desi_chr01	10102257	[A/C]	NA
Ca_Desi_Chr_1_10150162	Ca_desi_chr01	10150162	[C/T]	NA
Ca_Desi_Chr_1_10197211	Ca_desi_chr01	10197211	[C/A]	NA
Ca_Desi_Chr_1_10197212	Ca_desi_chr01	10197212	[A/G]	NA
Ca_Desi_Chr_1_10209832	Ca_desi_chr01	10209832	[A/C]	NA
Ca_Desi_Chr_1_10288683	Ca_desi_chr01	10288683	[T/G]	NA
Ca_Desi_Chr_1_10292762	Ca_desi_chr01	10292762	[T/A]	NA
Ca_Desi_Chr_1_10326430	Ca_desi_chr01	10326430	[T/G]	NA
Ca_Desi_Chr_1_10329721	Ca_desi_chr01	10329721	[A/G]	NA
Ca_Desi_Chr_1_10329749	Ca_desi_chr01	10329749	[A/C]	NA
Ca_Desi_Chr_1_10329754	Ca_desi_chr01	10329754	[G/A]	NA
Ca_Desi_Chr_1_10329756	Ca_desi_chr01	10329756	[T/C]	NA
Ca_Desi_Chr_1_10331523	Ca_desi_chr01	10331523	[A/G]	NA
Ca_Desi_Chr_1_10331526	Ca_desi_chr01	10331526	[A/G]	NA
Ca_Desi_Chr_1_10331535	Ca_desi_chr01	10331535	[A/G]	NA
Ca_Desi_Chr_1_10331538	Ca_desi_chr01	10331538	[T/C]	NA
Ca_Desi_Chr_1_10331541	Ca_desi_chr01	10331541	[T/C]	NA
Ca_Desi_Chr_1_10331548	Ca_desi_chr01	10331548	[A/G]	NA
Ca_Desi_Chr_1_10331553	Ca_desi_chr01	10331553	[A/G]	NA
Ca_Desi_Chr_1_10331562	Ca_desi_chr01	10331562	[A/G]	NA
Ca_Desi_Chr_1_10339620	Ca_desi_chr01	10339620	[T/G]	NA
Ca_Desi_Chr_1_10366520	Ca_desi_chr01	10366520	[T/C]	NA

SNP IDs	Chromosomes	Physical positions (bp)	SNPs	NCBI SNPdb accession IDs (NCBI_ss#)
Ca_Desi_Chr_1_10371008	Ca_desi_chr01	10371008	[A/T]	NA
Ca_Desi_Chr_1_10371022	Ca_desi_chr01	10371022	[T/A]	NA
Ca_Desi_Chr_1_10371032	Ca_desi_chr01	10371032	[A/G]	NA
Ca_Desi_Chr_1_10441613	Ca_desi_chr01	10441613	[G/A]	NA
Ca_Desi_Chr_1_10441631	Ca_desi_chr01	10441631	[A/T]	NA
Ca_Desi_Chr_1_10441640	Ca_desi_chr01	10441640	[A/G]	NA
Ca_Desi_Chr_1_10441697	Ca_desi_chr01	10441697	[C/A]	NA
Ca_Desi_Chr_1_10441699	Ca_desi_chr01	10441699	[A/G]	NA
Ca_Desi_Chr_1_10441719	Ca_desi_chr01	10441719	[G/A]	NA
Ca_Desi_Chr_1_10441750	Ca_desi_chr01	10441750	[T/C]	NA
Ca_Desi_Chr_1_10441806	Ca_desi_chr01	10441806	[A/C]	NA
Ca_Desi_Chr_1_10441837	Ca_desi_chr01	10441837	[A/G]	NA
Ca_Desi_Chr_1_10525376	Ca_desi_chr01	10525376	[A/G]	NA
Ca_Desi_Chr_1_10525418	Ca_desi_chr01	10525418	[T/G]	NA
Ca_Desi_Chr_1_10525426	Ca_desi_chr01	10525426	[T/A]	NA
Ca_Desi_Chr_1_10525507	Ca_desi_chr01	10525507	[T/A]	NA
Ca_Desi_Chr_1_10525509	Ca_desi_chr01	10525509	[G/T]	NA
Ca_Desi_Chr_1_10525544	Ca_desi_chr01	10525544	[A/C]	NA
Ca_Desi_Chr_1_10530998	Ca_desi_chr01	10530998	[C/T]	NA
Ca_Desi_Chr_1_10530999	Ca_desi_chr01	10530999	[T/C]	NA
Ca_Desi_Chr_1_10531007	Ca_desi_chr01	10531007	[T/G]	NA
Ca_Desi_Chr_1_10539706	Ca_desi_chr01	10539706	[T/C]	NA
Ca_Desi_Chr_1_10539735	Ca_desi_chr01	10539735	[A/C]	NA
Ca_Desi_Chr_1_10723310	Ca_desi_chr01	10723310	[A/G]	NA
Ca_Desi_Chr_1_10723320	Ca_desi_chr01	10723320	[T/G]	NA
Ca_Desi_Chr_1_10726182	Ca_desi_chr01	10726182	[T/C]	NA
Ca_Desi_Chr_1_10741072	Ca_desi_chr01	10741072	[A/C]	NA
Ca_Desi_Chr_1_10763967	Ca_desi_chr01	10763967	[A/G]	NA
Ca_Desi_Chr_1_10763991	Ca_desi_chr01	10763991	[A/G]	NA
Ca_Desi_Chr_1_10763995	Ca_desi_chr01	10763995	[A/G]	NA
Ca_Desi_Chr_1_10764000	Ca_desi_chr01	10764000	[A/G]	NA
Ca_Desi_Chr_1_10778822	Ca_desi_chr01	10778822	[A/C]	NA
Ca_Desi_Chr_1_10778823	Ca_desi_chr01	10778823	[G/T]	NA
Ca_Desi_Chr_1_10778824	Ca_desi_chr01	10778824	[A/C]	NA

SNP IDs	Chromosomes	Physical positions (bp)	SNPs	NCBI SNPdb accession IDs (NCBI_ss#)
Ca_Desi_Chr_1_10778825	Ca_desi_chr01	10778825	[T/A]	NA
Ca_Desi_Chr_1_10873943	Ca_desi_chr01	10873943	[C/A]	NA
Ca_Desi_Chr_1_10873944	Ca_desi_chr01	10873944	[T/G]	NA
Ca_Desi_Chr_1_10873945	Ca_desi_chr01	10873945	[T/A]	NA
Ca_Desi_Chr_1_10873946	Ca_desi_chr01	10873946	[A/T]	NA
Ca_Desi_Chr_1_10888834	Ca_desi_chr01	10888834	[C/A]	NA
Ca_Desi_Chr_1_10891980	Ca_desi_chr01	10891980	[A/C]	NA
Ca_Desi_Chr_1_10892013	Ca_desi_chr01	10892013	[A/C]	NA
Ca_Desi_Chr_1_10892555	Ca_desi_chr01	10892555	[A/C]	NA
Ca_Desi_Chr_1_10893257	Ca_desi_chr01	10893257	[T/C]	NA
Ca_Desi_Chr_1_10893270	Ca_desi_chr01	10893270	[T/C]	NA
Ca_Desi_Chr_1_10893917	Ca_desi_chr01	10893917	[T/G]	NA
Ca_Desi_Chr_1_10893921	Ca_desi_chr01	10893921	[T/G]	NA
Ca_Desi_Chr_1_10893923	Ca_desi_chr01	10893923	[A/T]	NA
Ca_Desi_Chr_1_10893939	Ca_desi_chr01	10893939	[T/A]	NA
Ca_Desi_Chr_1_10970320	Ca_desi_chr01	10970320	[C/A]	NA
Ca_Desi_Chr_1_10970322	Ca_desi_chr01	10970322	[T/A]	NA
Ca_Desi_Chr_1_10970324	Ca_desi_chr01	10970324	[A/C]	NA
Ca_Desi_Chr_1_10970326	Ca_desi_chr01	10970326	[T/G]	NA
Ca_Desi_Chr_1_11071982	Ca_desi_chr01	11071982	[A/C]	NA
Ca_Desi_Chr_1_11109217	Ca_desi_chr01	11109217	[A/C]	NA
Ca_Desi_Chr_1_11109229	Ca_desi_chr01	11109229	[A/G]	NA
Ca_Desi_Chr_1_11203460	Ca_desi_chr01	11203460	[T/G]	NA
Ca_Desi_Chr_1_11208230	Ca_desi_chr01	11208230	[T/G]	NA
Ca_Desi_Chr_1_11208245	Ca_desi_chr01	11208245	[A/C]	NA
Ca_Desi_Chr_1_11255584	Ca_desi_chr01	11255584	[T/G]	NA
Ca_Desi_Chr_1_11305633	Ca_desi_chr01	11305633	[T/G]	NA
Ca_Desi_Chr_1_11403246	Ca_desi_chr01	11403246	[T/C]	NA
Ca_Desi_Chr_1_11403267	Ca_desi_chr01	11403267	[A/G]	NA
Ca_Desi_Chr_1_11442093	Ca_desi_chr01	11442093	[A/G]	NA
Ca_Desi_Chr_1_11442116	Ca_desi_chr01	11442116	[T/A]	NA
Ca_Desi_Chr_1_11457196	Ca_desi_chr01	11457196	[T/C]	NA
Ca_Desi_Chr_1_11470507	Ca_desi_chr01	11470507	[A/C]	NA
Ca_Desi_Chr_1_11521599	Ca_desi_chr01	11521599	[A/C]	NA

SNP IDs	Chromosomes	Physical positions (bp)	SNPs	NCBI SNPdb accession IDs (NCBI_ss#)
Ca_Desi_Chr_1_11521627	Ca_desi_chr01	11521627	[T/C]	NA
Ca_Desi_Chr_1_11521638	Ca_desi_chr01	11521638	[T/C]	NA
Ca_Desi_Chr_1_11521732	Ca_desi_chr01	11521732	[G/T]	NA
Ca_Desi_Chr_1_11522463	Ca_desi_chr01	11522463	[A/C]	NA
Ca_Desi_Chr_1_11523137	Ca_desi_chr01	11523137	[T/A]	NA
Ca_Desi_Chr_1_11523158	Ca_desi_chr01	11523158	[A/C]	NA
Ca_Desi_Chr_1_11569811	Ca_desi_chr01	11569811	[A/C]	NA
Ca_Desi_Chr_1_11586936	Ca_desi_chr01	11586936	[T/C]	NA
Ca_Desi_Chr_1_11587001	Ca_desi_chr01	11587001	[A/G]	NA
Ca_Desi_Chr_1_11909488	Ca_desi_chr01	11909488	[A/G]	NA
Ca_Desi_Chr_1_11909509	Ca_desi_chr01	11909509	[T/C]	NA
Ca_Desi_Chr_1_11909533	Ca_desi_chr01	11909533	[T/G]	NA
Ca_Desi_Chr_1_11929981	Ca_desi_chr01	11929981	[C/T]	NA
Ca_Desi_Chr_1_11940120	Ca_desi_chr01	11940120	[T/G]	NA
Ca_Desi_Chr_1_11968306	Ca_desi_chr01	11968306	[A/G]	NA
Ca_Desi_Chr_1_12086680	Ca_desi_chr01	12086680	[T/A]	NA
Ca_Desi_Chr_1_12137403	Ca_desi_chr01	12137403	[T/G]	NA
Ca_Desi_Chr_1_12137406	Ca_desi_chr01	12137406	[T/G]	NA
Ca_Desi_Chr_1_12141258	Ca_desi_chr01	12141258	[A/G]	NA
Ca_Desi_Chr_1_12155910	Ca_desi_chr01	12155910	[T/G]	NA
Ca_Desi_Chr_1_12155983	Ca_desi_chr01	12155983	[G/T]	NA
Ca_Desi_Chr_1_12162733	Ca_desi_chr01	12162733	[A/T]	NA
Ca_Desi_Chr_1_12162779	Ca_desi_chr01	12162779	[T/C]	NA
Ca_Desi_Chr_1_12162855	Ca_desi_chr01	12162855	[A/C]	NA
Ca_Desi_Chr_1_12189288	Ca_desi_chr01	12189288	[A/C]	NA
Ca_Desi_Chr_1_12192033	Ca_desi_chr01	12192033	[T/G]	NA
Ca_Desi_Chr_1_12192083	Ca_desi_chr01	12192083	[T/C]	NA
Ca_Desi_Chr_1_12192100	Ca_desi_chr01	12192100	[T/C]	NA
Ca_Desi_Chr_1_12192102	Ca_desi_chr01	12192102	[A/G]	NA
Ca_Desi_Chr_1_12192120	Ca_desi_chr01	12192120	[A/G]	NA
Ca_Desi_Chr_1_13584259	Ca_desi_chr01	13584259	[A/G]	NA
Ca_Desi_Chr_1_13590315	Ca_desi_chr01	13590315	[A/G]	NA
Ca_Desi_Chr_1_13590326	Ca_desi_chr01	13590326	[T/C]	NA
Ca_Desi_Chr_1_13590364	Ca_desi_chr01	13590364	[A/G]	NA

SNP IDs	Chromosomes	Physical positions (bp)	SNPs	NCBI SNPdb accession IDs (NCBI_ss#)
Ca_Desi_Chr_1_13590369	Ca_desi_chr01	13590369	[A/G]	NA
Ca_Desi_Chr_1_13592155	Ca_desi_chr01	13592155	[T/G]	NA
Ca_Desi_Chr_1_13592851	Ca_desi_chr01	13592851	[A/C]	NA
Ca_Desi_Chr_1_13598225	Ca_desi_chr01	13598225	[A/C]	NA
Ca_Desi_Chr_1_13602414	Ca_desi_chr01	13602414	[A/G]	NA
Ca_Desi_Chr_1_13602415	Ca_desi_chr01	13602415	[C/T]	NA
Ca_Desi_Chr_1_13602470	Ca_desi_chr01	13602470	[A/C]	NA
Ca_Desi_Chr_1_13620061	Ca_desi_chr01	13620061	[A/T]	NA
Ca_Desi_Chr_1_13641365	Ca_desi_chr01	13641365	[T/G]	NA
Ca_Desi_Chr_1_13641423	Ca_desi_chr01	13641423	[A/T]	NA
Ca_Desi_Chr_1_13645462	Ca_desi_chr01	13645462	[A/C]	NA
Ca_Desi_Chr_1_13653278	Ca_desi_chr01	13653278	[T/C]	NA
Ca_Desi_Chr_1_13653279	Ca_desi_chr01	13653279	[G/T]	NA
Ca_Desi_Chr_1_13692588	Ca_desi_chr01	13692588	[T/C]	NA
Ca_Desi_Chr_1_13692615	Ca_desi_chr01	13692615	[T/C]	NA
Ca_Desi_Chr_1_13692633	Ca_desi_chr01	13692633	[T/C]	NA
Ca_Desi_Chr_1_13692635	Ca_desi_chr01	13692635	[A/C]	NA
Ca_Desi_Chr_1_13692646	Ca_desi_chr01	13692646	[A/G]	NA
Ca_Desi_Chr_1_13692649	Ca_desi_chr01	13692649	[A/C]	NA
Ca_Desi_Chr_1_13692664	Ca_desi_chr01	13692664	[A/G]	NA
Ca_Desi_Chr_1_13692681	Ca_desi_chr01	13692681	[T/G]	NA
Ca_Desi_Chr_1_13692705	Ca_desi_chr01	13692705	[G/A]	NA
Ca_Desi_Chr_1_13692716	Ca_desi_chr01	13692716	[T/C]	NA
Ca_Desi_Chr_1_13692721	Ca_desi_chr01	13692721	[A/C]	NA
Ca_Desi_Chr_1_13692724	Ca_desi_chr01	13692724	[A/C]	NA
Ca_Desi_Chr_1_13692727	Ca_desi_chr01	13692727	[A/C]	NA
Ca_Desi_Chr_1_13798747	Ca_desi_chr01	13798747	[A/G]	NA
Ca_Desi_Chr_1_13798784	Ca_desi_chr01	13798784	[A/T]	NA
Ca_Desi_Chr_1_13805553	Ca_desi_chr01	13805553	[A/C]	NA
Ca_Desi_Chr_1_13805554	Ca_desi_chr01	13805554	[G/T]	NA
Ca_Desi_Chr_1_13831773	Ca_desi_chr01	13831773	[T/G]	NA
Ca_Desi_Chr_1_13831799	Ca_desi_chr01	13831799	[T/C]	NA
Ca_Desi_Chr_1_13864931	Ca_desi_chr01	13864931	[T/C]	NA
Ca_Desi_Chr_1_13865960	Ca_desi_chr01	13865960	[T/G]	NA

SNP IDs	Chromosomes	Physical positions (bp)	SNPs	NCBI SNPdb accession IDs (NCBI_ss#)
Ca_Desi_Chr_1_13903458	Ca_desi_chr01	13903458	[T/G]	NA
Ca_Desi_Chr_1_13903477	Ca_desi_chr01	13903477	[A/G]	NA
Ca_Desi_Chr_1_13906207	Ca_desi_chr01	13906207	[A/C]	NA
Ca_Desi_Chr_1_13906208	Ca_desi_chr01	13906208	[G/T]	NA
Ca_Desi_Chr_1_13906209	Ca_desi_chr01	13906209	[A/T]	NA
Ca_Desi_Chr_1_13919264	Ca_desi_chr01	13919264	[A/G]	NA
Ca_Desi_Chr_1_13935560	Ca_desi_chr01	13935560	[G/A]	NA
Ca_Desi_Chr_1_13935569	Ca_desi_chr01	13935569	[T/C]	NA
Ca_Desi_Chr_1_13969540	Ca_desi_chr01	13969540	[C/T]	NA
Ca_Desi_Chr_1_14019371	Ca_desi_chr01	14019371	[T/A]	NA
Ca_Desi_Chr_1_14019442	Ca_desi_chr01	14019442	[A/G]	NA
Ca_Desi_Chr_1_14093167	Ca_desi_chr01	14093167	[A/G]	NA
Ca_Desi_Chr_1_14113496	Ca_desi_chr01	14113496	[T/C]	NA
Ca_Desi_Chr_1_14113554	Ca_desi_chr01	14113554	[T/C]	NA
Ca_Desi_Chr_1_14113558	Ca_desi_chr01	14113558	[A/G]	NA
Ca_Desi_Chr_1_14113571	Ca_desi_chr01	14113571	[T/A]	NA
Ca_Desi_Chr_1_14113588	Ca_desi_chr01	14113588	[A/C]	NA
Ca_Desi_Chr_1_14127376	Ca_desi_chr01	14127376	[C/T]	NA
Ca_Desi_Chr_1_14127778	Ca_desi_chr01	14127778	[T/G]	NA
Ca_Desi_Chr_1_14127804	Ca_desi_chr01	14127804	[A/T]	NA
Ca_Desi_Chr_1_14127815	Ca_desi_chr01	14127815	[A/C]	NA
Ca_Desi_Chr_1_14142347	Ca_desi_chr01	14142347	[A/G]	NA
Ca_Desi_Chr_1_14169398	Ca_desi_chr01	14169398	[T/C]	NA
Ca_Desi_Chr_1_14169409	Ca_desi_chr01	14169409	[C/T]	NA
Ca_Desi_Chr_1_14169421	Ca_desi_chr01	14169421	[A/G]	NA
Ca_Desi_Chr_1_14170600	Ca_desi_chr01	14170600	[T/G]	NA
Ca_Desi_Chr_1_14190369	Ca_desi_chr01	14190369	[C/T]	NA
Ca_Desi_Chr_2_16455	Ca_desi_chr02	16455	[T/C]	NA
Ca_Desi_Chr_2_16457	Ca_desi_chr02	16457	[T/C]	NA
Ca_Desi_Chr_2_16482	Ca_desi_chr02	16482	[T/G]	NA
Ca_Desi_Chr_2_16485	Ca_desi_chr02	16485	[A/G]	NA
Ca_Desi_Chr_2_16492	Ca_desi_chr02	16492	[A/G]	NA
Ca_Desi_Chr_2_82639	Ca_desi_chr02	82639	[T/C]	NA
Ca_Desi_Chr_2_83864	Ca_desi_chr02	83864	[A/C]	NA

SNP IDs	Chromosomes	Physical positions (bp)	SNPs	NCBI SNPdb accession IDs (NCBI_ss#)
Ca_Desi_Chr_2_83884	Ca_desi_chr02	83884	[A/C]	NA
Ca_Desi_Chr_2_83888	Ca_desi_chr02	83888	[T/C]	NA
Ca_Desi_Chr_2_83893	Ca_desi_chr02	83893	[T/G]	NA
Ca_Desi_Chr_2_83895	Ca_desi_chr02	83895	[T/C]	NA
Ca_Desi_Chr_2_201886	Ca_desi_chr02	201886	[T/G]	NA
Ca_Desi_Chr_2_278031	Ca_desi_chr02	278031	[C/T]	NA
Ca_Desi_Chr_2_278283	Ca_desi_chr02	278283	[T/A]	NA
Ca_Desi_Chr_2_279234	Ca_desi_chr02	279234	[T/C]	NA
Ca_Desi_Chr_2_279239	Ca_desi_chr02	279239	[A/G]	NA
Ca_Desi_Chr_2_279241	Ca_desi_chr02	279241	[T/G]	NA
Ca_Desi_Chr_2_279256	Ca_desi_chr02	279256	[T/G]	NA
Ca_Desi_Chr_2_281905	Ca_desi_chr02	281905	[T/C]	NA
Ca_Desi_Chr_2_308828	Ca_desi_chr02	308828	[C/T]	NA
Ca_Desi_Chr_2_314070	Ca_desi_chr02	314070	[A/G]	NA
Ca_Desi_Chr_2_318397	Ca_desi_chr02	318397	[A/C]	NA
Ca_Desi_Chr_2_318541	Ca_desi_chr02	318541	[T/G]	NA
Ca_Desi_Chr_2_326926	Ca_desi_chr02	326926	[G/T]	NA
Ca_Desi_Chr_2_327450	Ca_desi_chr02	327450	[G/C]	NA
Ca_Desi_Chr_2_327691	Ca_desi_chr02	327691	[C/G]	NA
Ca_Desi_Chr_2_327712	Ca_desi_chr02	327712	[T/C]	NA
Ca_Desi_Chr_2_412581	Ca_desi_chr02	412581	[C/G]	NA
Ca_Desi_Chr_2_412583	Ca_desi_chr02	412583	[G/T]	NA
Ca_Desi_Chr_2_412587	Ca_desi_chr02	412587	[A/G]	NA
Ca_Desi_Chr_2_412594	Ca_desi_chr02	412594	[T/G]	NA
Ca_Desi_Chr_2_412597	Ca_desi_chr02	412597	[T/G]	NA
Ca_Desi_Chr_2_412608	Ca_desi_chr02	412608	[T/C]	NA
Ca_Desi_Chr_2_429417	Ca_desi_chr02	429417	[T/C]	NA
Ca_Desi_Chr_2_429459	Ca_desi_chr02	429459	[A/T]	NA
Ca_Desi_Chr_2_430152	Ca_desi_chr02	430152	[C/G]	NA
Ca_Desi_Chr_2_437401	Ca_desi_chr02	437401	[C/T]	NA
Ca_Desi_Chr_2_437438	Ca_desi_chr02	437438	[T/C]	NA
Ca_Desi_Chr_2_437445	Ca_desi_chr02	437445	[T/C]	NA
Ca_Desi_Chr_2_437646	Ca_desi_chr02	437646	[T/G]	NA
Ca_Desi_Chr_2_437745	Ca_desi_chr02	437745	[T/C]	NA

SNP IDs	Chromosomes	Physical positions (bp)	SNPs	NCBI SNPdb accession IDs (NCBI_ss#)
Ca_Desi_Chr_2_437750	Ca_desi_chr02	437750	[A/C]	NA
Ca_Desi_Chr_2_440424	Ca_desi_chr02	440424	[G/T]	NA
Ca_Desi_Chr_2_440724	Ca_desi_chr02	440724	[T/C]	NA
Ca_Desi_Chr_2_440754	Ca_desi_chr02	440754	[A/T]	NA
Ca_Desi_Chr_2_527061	Ca_desi_chr02	527061	[G/A]	NA
Ca_Desi_Chr_2_527074	Ca_desi_chr02	527074	[C/T]	NA
Ca_Desi_Chr_2_527083	Ca_desi_chr02	527083	[C/T]	NA
Ca_Desi_Chr_2_527084	Ca_desi_chr02	527084	[G/A]	NA
Ca_Desi_Chr_2_527631	Ca_desi_chr02	527631	[T/C]	NA
Ca_Desi_Chr_2_527633	Ca_desi_chr02	527633	[A/T]	NA
Ca_Desi_Chr_2_527634	Ca_desi_chr02	527634	[A/C]	NA
Ca_Desi_Chr_2_527635	Ca_desi_chr02	527635	[A/C]	NA
Ca_Desi_Chr_2_527646	Ca_desi_chr02	527646	[A/G]	NA
Ca_Desi_Chr_2_527670	Ca_desi_chr02	527670	[A/G]	NA
Ca_Desi_Chr_2_527684	Ca_desi_chr02	527684	[A/G]	NA
Ca_Desi_Chr_2_574020	Ca_desi_chr02	574020	[T/G]	NA
Ca_Desi_Chr_2_574032	Ca_desi_chr02	574032	[T/G]	NA
Ca_Desi_Chr_2_574042	Ca_desi_chr02	574042	[T/G]	NA
Ca_Desi_Chr_2_574051	Ca_desi_chr02	574051	[T/C]	NA
Ca_Desi_Chr_2_574061	Ca_desi_chr02	574061	[A/G]	NA
Ca_Desi_Chr_2_574863	Ca_desi_chr02	574863	[C/A]	NA
Ca_Desi_Chr_2_574864	Ca_desi_chr02	574864	[T/G]	NA
Ca_Desi_Chr_2_574865	Ca_desi_chr02	574865	[T/A]	NA
Ca_Desi_Chr_2_574867	Ca_desi_chr02	574867	[A/C]	NA
Ca_Desi_Chr_2_575529	Ca_desi_chr02	575529	[T/C]	NA
Ca_Desi_Chr_2_575532	Ca_desi_chr02	575532	[G/C]	NA
Ca_Desi_Chr_2_576614	Ca_desi_chr02	576614	[A/T]	NA
Ca_Desi_Chr_2_580283	Ca_desi_chr02	580283	[T/G]	NA
Ca_Desi_Chr_2_595835	Ca_desi_chr02	595835	[A/T]	NA
Ca_Desi_Chr_2_595836	Ca_desi_chr02	595836	[A/T]	NA
Ca_Desi_Chr_2_694565	Ca_desi_chr02	694565	[T/A]	NA
Ca_Desi_Chr_2_694574	Ca_desi_chr02	694574	[T/G]	NA
Ca_Desi_Chr_2_709390	Ca_desi_chr02	709390	[G/T]	NA
Ca_Desi_Chr_2_760163	Ca_desi_chr02	760163	[T/C]	NA

SNP IDs	Chromosomes	Physical positions (bp)	SNPs	NCBI SNPdb accession IDs (NCBI_ss#)
Ca_Desi_Chr_2_760175	Ca_desi_chr02	760175	[T/C]	NA
Ca_Desi_Chr_2_760179	Ca_desi_chr02	760179	[T/C]	NA
Ca_Desi_Chr_2_760183	Ca_desi_chr02	760183	[A/G]	NA
Ca_Desi_Chr_2_760194	Ca_desi_chr02	760194	[A/G]	NA
Ca_Desi_Chr_2_760214	Ca_desi_chr02	760214	[A/G]	NA
Ca_Desi_Chr_2_941677	Ca_desi_chr02	941677	[T/A]	NA
Ca_Desi_Chr_2_941690	Ca_desi_chr02	941690	[T/C]	NA
Ca_Desi_Chr_2_992313	Ca_desi_chr02	992313	[A/C]	NA
Ca_Desi_Chr_2_1019710	Ca_desi_chr02	1019710	[T/A]	NA
Ca_Desi_Chr_2_1019712	Ca_desi_chr02	1019712	[A/G]	NA
Ca_Desi_Chr_2_1019715	Ca_desi_chr02	1019715	[T/A]	NA
Ca_Desi_Chr_2_1019724	Ca_desi_chr02	1019724	[A/G]	NA
Ca_Desi_Chr_2_1019754	Ca_desi_chr02	1019754	[A/G]	NA
Ca_Desi_Chr_2_1066604	Ca_desi_chr02	1066604	[T/G]	NA
Ca_Desi_Chr_2_1081581	Ca_desi_chr02	1081581	[C/T]	NA
Ca_Desi_Chr_2_1081734	Ca_desi_chr02	1081734	[T/A]	NA
Ca_Desi_Chr_2_1081742	Ca_desi_chr02	1081742	[A/C]	NA
Ca_Desi_Chr_2_1089512	Ca_desi_chr02	1089512	[C/A]	NA
Ca_Desi_Chr_2_1089513	Ca_desi_chr02	1089513	[C/G]	NA
Ca_Desi_Chr_2_1089514	Ca_desi_chr02	1089514	[C/A]	NA
Ca_Desi_Chr_2_1089543	Ca_desi_chr02	1089543	[A/C]	NA
Ca_Desi_Chr_2_1089550	Ca_desi_chr02	1089550	[A/G]	NA
Ca_Desi_Chr_2_1210268	Ca_desi_chr02	1210268	[T/C]	NA
Ca_Desi_Chr_2_1210294	Ca_desi_chr02	1210294	[T/G]	NA
Ca_Desi_Chr_2_1210298	Ca_desi_chr02	1210298	[A/C]	NA
Ca_Desi_Chr_2_1210310	Ca_desi_chr02	1210310	[A/T]	NA
Ca_Desi_Chr_2_1216751	Ca_desi_chr02	1216751	[T/G]	NA
Ca_Desi_Chr_2_1216760	Ca_desi_chr02	1216760	[A/T]	NA
Ca_Desi_Chr_2_1216786	Ca_desi_chr02	1216786	[A/C]	NA
Ca_Desi_Chr_2_1245288	Ca_desi_chr02	1245288	[T/C]	NA
Ca_Desi_Chr_2_1245292	Ca_desi_chr02	1245292	[A/G]	NA
Ca_Desi_Chr_2_1245302	Ca_desi_chr02	1245302	[C/G]	NA
Ca_Desi_Chr_2_1245305	Ca_desi_chr02	1245305	[T/A]	NA
Ca_Desi_Chr_2_1245363	Ca_desi_chr02	1245363	[C/A]	NA

SNP IDs	Chromosomes	Physical positions (bp)	SNPs	NCBI SNPdb accession IDs (NCBI_ss#)
Ca_Desi_Chr_2_1245365	Ca_desi_chr02	1245365	[G/A]	NA
Ca_Desi_Chr_2_1245366	Ca_desi_chr02	1245366	[C/T]	NA
Ca_Desi_Chr_2_1245367	Ca_desi_chr02	1245367	[T/C]	NA
Ca_Desi_Chr_2_1312276	Ca_desi_chr02	1312276	[A/G]	NA
Ca_Desi_Chr_2_1312332	Ca_desi_chr02	1312332	[A/G]	NA
Ca_Desi_Chr_2_1341643	Ca_desi_chr02	1341643	[C/A]	NA
Ca_Desi_Chr_2_1521874	Ca_desi_chr02	1521874	[A/T]	NA
Ca_Desi_Chr_2_1553220	Ca_desi_chr02	1553220	[A/G]	NA
Ca_Desi_Chr_2_1603397	Ca_desi_chr02	1603397	[G/A]	NA
Ca_Desi_Chr_2_1603398	Ca_desi_chr02	1603398	[G/A]	NA
Ca_Desi_Chr_2_1603415	Ca_desi_chr02	1603415	[T/A]	NA
Ca_Desi_Chr_2_1621777	Ca_desi_chr02	1621777	[T/G]	NA
Ca_Desi_Chr_2_1621788	Ca_desi_chr02	1621788	[G/A]	NA
Ca_Desi_Chr_2_1710080	Ca_desi_chr02	1710080	[G/A]	NA
Ca_Desi_Chr_2_1727785	Ca_desi_chr02	1727785	[A/C]	NA
Ca_Desi_Chr_2_1749427	Ca_desi_chr02	1749427	[T/C]	NA
Ca_Desi_Chr_2_1823564	Ca_desi_chr02	1823564	[A/G]	NA
Ca_Desi_Chr_2_1857292	Ca_desi_chr02	1857292	[A/C]	NA
Ca_Desi_Chr_2_1861404	Ca_desi_chr02	1861404	[G/C]	NA
Ca_Desi_Chr_2_1861410	Ca_desi_chr02	1861410	[A/C]	NA
Ca_Desi_Chr_2_1867952	Ca_desi_chr02	1867952	[T/C]	NA
Ca_Desi_Chr_2_1867957	Ca_desi_chr02	1867957	[A/C]	NA
Ca_Desi_Chr_2_1867962	Ca_desi_chr02	1867962	[T/C]	NA
Ca_Desi_Chr_2_1867963	Ca_desi_chr02	1867963	[A/G]	NA
Ca_Desi_Chr_2_1867968	Ca_desi_chr02	1867968	[T/C]	NA
Ca_Desi_Chr_2_1867975	Ca_desi_chr02	1867975	[A/G]	NA
Ca_Desi_Chr_2_1868831	Ca_desi_chr02	1868831	[T/C]	NA
Ca_Desi_Chr_2_1868845	Ca_desi_chr02	1868845	[A/C]	NA
Ca_Desi_Chr_2_1868860	Ca_desi_chr02	1868860	[A/C]	NA
Ca_Desi_Chr_2_1883086	Ca_desi_chr02	1883086	[T/C]	NA
Ca_Desi_Chr_2_1883106	Ca_desi_chr02	1883106	[T/A]	NA
Ca_Desi_Chr_2_1883127	Ca_desi_chr02	1883127	[A/G]	NA
Ca_Desi_Chr_2_1894071	Ca_desi_chr02	1894071	[A/T]	NA
Ca_Desi_Chr_2_1894072	Ca_desi_chr02	1894072	[G/A]	NA

SNP IDs	Chromosomes	Physical positions (bp)	SNPs	NCBI SNPdb accession IDs (NCBI_ss#)
Ca_Desi_Chr_2_1894073	Ca_desi_chr02	1894073	[T/G]	NA
Ca_Desi_Chr_2_2056877	Ca_desi_chr02	2056877	[A/G]	NA
Ca_Desi_Chr_2_2056883	Ca_desi_chr02	2056883	[T/G]	NA
Ca_Desi_Chr_2_2056890	Ca_desi_chr02	2056890	[T/G]	NA
Ca_Desi_Chr_2_2056926	Ca_desi_chr02	2056926	[T/G]	NA
Ca_Desi_Chr_2_2058314	Ca_desi_chr02	2058314	[T/G]	NA
Ca_Desi_Chr_2_2058340	Ca_desi_chr02	2058340	[T/G]	NA
Ca_Desi_Chr_2_2100204	Ca_desi_chr02	2100204	[A/C]	NA
Ca_Desi_Chr_2_2100219	Ca_desi_chr02	2100219	[A/G]	NA
Ca_Desi_Chr_2_2100223	Ca_desi_chr02	2100223	[T/C]	NA
Ca_Desi_Chr_2_2124915	Ca_desi_chr02	2124915	[C/A]	NA
Ca_Desi_Chr_2_2124918	Ca_desi_chr02	2124918	[G/C]	NA
Ca_Desi_Chr_2_2124925	Ca_desi_chr02	2124925	[T/C]	NA
Ca_Desi_Chr_2_2124928	Ca_desi_chr02	2124928	[C/A]	NA
Ca_Desi_Chr_2_2124931	Ca_desi_chr02	2124931	[T/C]	NA
Ca_Desi_Chr_2_2124933	Ca_desi_chr02	2124933	[T/G]	NA
Ca_Desi_Chr_2_2124937	Ca_desi_chr02	2124937	[T/A]	NA
Ca_Desi_Chr_2_2124940	Ca_desi_chr02	2124940	[A/G]	NA
Ca_Desi_Chr_2_2124944	Ca_desi_chr02	2124944	[C/G]	NA
Ca_Desi_Chr_2_2257840	Ca_desi_chr02	2257840	[A/G]	NA
Ca_Desi_Chr_2_2257849	Ca_desi_chr02	2257849	[T/C]	NA
Ca_Desi_Chr_2_2257855	Ca_desi_chr02	2257855	[T/C]	NA
Ca_Desi_Chr_2_2402200	Ca_desi_chr02	2402200	[T/G]	NA
Ca_Desi_Chr_2_2449580	Ca_desi_chr02	2449580	[T/G]	NA
Ca_Desi_Chr_2_2527594	Ca_desi_chr02	2527594	[T/G]	NA
Ca_Desi_Chr_2_2563476	Ca_desi_chr02	2563476	[T/G]	NA
Ca_Desi_Chr_2_2574523	Ca_desi_chr02	2574523	[T/G]	NA
Ca_Desi_Chr_2_2589893	Ca_desi_chr02	2589893	[G/T]	NA
Ca_Desi_Chr_2_2607993	Ca_desi_chr02	2607993	[A/G]	NA
Ca_Desi_Chr_2_2608996	Ca_desi_chr02	2608996	[C/A]	NA
Ca_Desi_Chr_2_2608997	Ca_desi_chr02	2608997	[T/G]	NA
Ca_Desi_Chr_2_2664631	Ca_desi_chr02	2664631	[T/A]	NA
Ca_Desi_Chr_2_2664651	Ca_desi_chr02	2664651	[A/C]	NA
Ca_Desi_Chr_2_2664685	Ca_desi_chr02	2664685	[A/G]	NA

SNP IDs	Chromosomes	Physical positions (bp)	SNPs	NCBI SNPdb accession IDs (NCBI_ss#)
Ca_Desi_Chr_2_2664707	Ca_desi_chr02	2664707	[T/G]	NA
Ca_Desi_Chr_2_2764678	Ca_desi_chr02	2764678	[A/T]	NA
Ca_Desi_Chr_2_2798801	Ca_desi_chr02	2798801	[A/G]	NA
Ca_Desi_Chr_2_2889652	Ca_desi_chr02	2889652	[A/T]	NA
Ca_Desi_Chr_2_2938053	Ca_desi_chr02	2938053	[T/C]	NA
Ca_Desi_Chr_2_2938058	Ca_desi_chr02	2938058	[T/C]	NA
Ca_Desi_Chr_2_2955638	Ca_desi_chr02	2955638	[T/C]	NA
Ca_Desi_Chr_2_3153816	Ca_desi_chr02	3153816	[A/G]	NA
Ca_Desi_Chr_2_3153827	Ca_desi_chr02	3153827	[A/G]	NA
Ca_Desi_Chr_2_3154549	Ca_desi_chr02	3154549	[A/G]	NA
Ca_Desi_Chr_2_3154581	Ca_desi_chr02	3154581	[A/G]	NA
Ca_Desi_Chr_2_3154589	Ca_desi_chr02	3154589	[T/G]	NA
Ca_Desi_Chr_2_3155845	Ca_desi_chr02	3155845	[A/C]	NA
Ca_Desi_Chr_2_3155866	Ca_desi_chr02	3155866	[T/C]	NA
Ca_Desi_Chr_2_3157430	Ca_desi_chr02	3157430	[A/C]	NA
Ca_Desi_Chr_2_3567723	Ca_desi_chr02	3567723	[T/G]	NA
Ca_Desi_Chr_2_3567744	Ca_desi_chr02	3567744	[A/C]	NA
Ca_Desi_Chr_2_3567747	Ca_desi_chr02	3567747	[A/C]	NA
Ca_Desi_Chr_2_3567834	Ca_desi_chr02	3567834	[T/G]	NA
Ca_Desi_Chr_2_3567843	Ca_desi_chr02	3567843	[A/G]	NA
Ca_Desi_Chr_2_3567848	Ca_desi_chr02	3567848	[T/C]	NA
Ca_Desi_Chr_2_3567863	Ca_desi_chr02	3567863	[A/G]	NA
Ca_Desi_Chr_2_3571890	Ca_desi_chr02	3571890	[G/T]	NA
Ca_Desi_Chr_2_3571894	Ca_desi_chr02	3571894	[C/A]	NA
Ca_Desi_Chr_2_3604350	Ca_desi_chr02	3604350	[A/C]	NA
Ca_Desi_Chr_2_3722082	Ca_desi_chr02	3722082	[T/G]	NA
Ca_Desi_Chr_2_3722112	Ca_desi_chr02	3722112	[T/C]	NA
Ca_Desi_Chr_2_3722146	Ca_desi_chr02	3722146	[A/G]	NA
Ca_Desi_Chr_2_3722164	Ca_desi_chr02	3722164	[T/A]	NA
Ca_Desi_Chr_2_3775127	Ca_desi_chr02	3775127	[A/C]	NA
Ca_Desi_Chr_2_3794000	Ca_desi_chr02	3794000	[A/T]	NA
Ca_Desi_Chr_2_3794024	Ca_desi_chr02	3794024	[A/G]	NA
Ca_Desi_Chr_2_3808702	Ca_desi_chr02	3808702	[T/G]	NA
Ca_Desi_Chr_2_3808720	Ca_desi_chr02	3808720	[A/G]	NA

SNP IDs	Chromosomes	Physical positions (bp)	SNPs	NCBI SNPdb accession IDs (NCBI_ss#)
Ca_Desi_Chr_2_3808724	Ca_desi_chr02	3808724	[A/C]	NA
Ca_Desi_Chr_2_3808952	Ca_desi_chr02	3808952	[A/G]	NA
Ca_Desi_Chr_2_3808962	Ca_desi_chr02	3808962	[A/G]	NA
Ca_Desi_Chr_2_3808969	Ca_desi_chr02	3808969	[A/C]	NA
Ca_Desi_Chr_2_3808983	Ca_desi_chr02	3808983	[T/C]	NA
Ca_Desi_Chr_2_3808988	Ca_desi_chr02	3808988	[T/C]	NA
Ca_Desi_Chr_2_4041343	Ca_desi_chr02	4041343	[A/C]	NA
Ca_Desi_Chr_2_4041362	Ca_desi_chr02	4041362	[A/C]	NA
Ca_Desi_Chr_2_4058389	Ca_desi_chr02	4058389	[G/A]	NA
Ca_Desi_Chr_2_4238236	Ca_desi_chr02	4238236	[A/C]	NA
Ca_Desi_Chr_2_4238244	Ca_desi_chr02	4238244	[A/G]	NA
Ca_Desi_Chr_2_4238256	Ca_desi_chr02	4238256	[T/C]	NA
Ca_Desi_Chr_2_4238258	Ca_desi_chr02	4238258	[T/C]	NA
Ca_Desi_Chr_2_4238264	Ca_desi_chr02	4238264	[A/C]	NA
Ca_Desi_Chr_2_4238266	Ca_desi_chr02	4238266	[A/C]	NA
Ca_Desi_Chr_2_4238269	Ca_desi_chr02	4238269	[T/C]	NA
Ca_Desi_Chr_2_4238275	Ca_desi_chr02	4238275	[T/C]	NA
Ca_Desi_Chr_2_4259547	Ca_desi_chr02	4259547	[A/C]	NA
Ca_Desi_Chr_2_4259580	Ca_desi_chr02	4259580	[A/C]	NA
Ca_Desi_Chr_2_4318111	Ca_desi_chr02	4318111	[A/T]	NA
Ca_Desi_Chr_2_4539973	Ca_desi_chr02	4539973	[T/A]	NA
Ca_Desi_Chr_2_4539997	Ca_desi_chr02	4539997	[T/C]	NA
Ca_Desi_Chr_2_4540012	Ca_desi_chr02	4540012	[T/C]	NA
Ca_Desi_Chr_2_4540029	Ca_desi_chr02	4540029	[A/C]	NA
Ca_Desi_Chr_2_4541895	Ca_desi_chr02	4541895	[G/T]	NA
Ca_Desi_Chr_2_4552083	Ca_desi_chr02	4552083	[A/G]	NA
Ca_Desi_Chr_2_4552112	Ca_desi_chr02	4552112	[C/T]	NA
Ca_Desi_Chr_2_4552121	Ca_desi_chr02	4552121	[T/C]	NA
Ca_Desi_Chr_2_4800835	Ca_desi_chr02	4800835	[A/C]	NA
Ca_Desi_Chr_2_4801057	Ca_desi_chr02	4801057	[T/G]	NA
Ca_Desi_Chr_2_4816390	Ca_desi_chr02	4816390	[T/C]	NA
Ca_Desi_Chr_2_4816421	Ca_desi_chr02	4816421	[A/G]	NA
Ca_Desi_Chr_2_4816424	Ca_desi_chr02	4816424	[A/G]	NA
Ca_Desi_Chr_2_5124304	Ca_desi_chr02	5124304	[T/G]	NA

SNP IDs	Chromosomes	Physical positions (bp)	SNPs	NCBI SNPdb accession IDs (NCBI_ss#)
Ca_Desi_Chr_2_5168624	Ca_desi_chr02	5168624	[A/C]	NA
Ca_Desi_Chr_2_5168625	Ca_desi_chr02	5168625	[C/A]	NA
Ca_Desi_Chr_2_5176013	Ca_desi_chr02	5176013	[C/A]	NA
Ca_Desi_Chr_2_5176014	Ca_desi_chr02	5176014	[T/G]	NA
Ca_Desi_Chr_2_5176015	Ca_desi_chr02	5176015	[G/A]	NA
Ca_Desi_Chr_2_5176016	Ca_desi_chr02	5176016	[G/T]	NA
Ca_Desi_Chr_2_5223368	Ca_desi_chr02	5223368	[A/C]	NA
Ca_Desi_Chr_2_5223374	Ca_desi_chr02	5223374	[A/G]	NA
Ca_Desi_Chr_2_5273243	Ca_desi_chr02	5273243	[T/C]	NA
Ca_Desi_Chr_2_5402731	Ca_desi_chr02	5402731	[A/C]	NA
Ca_Desi_Chr_2_5402734	Ca_desi_chr02	5402734	[T/G]	NA
Ca_Desi_Chr_2_5500858	Ca_desi_chr02	5500858	[A/T]	NA
Ca_Desi_Chr_2_5503405	Ca_desi_chr02	5503405	[T/A]	NA
Ca_Desi_Chr_2_5503406	Ca_desi_chr02	5503406	[G/T]	NA
Ca_Desi_Chr_2_5503507	Ca_desi_chr02	5503507	[C/A]	NA
Ca_Desi_Chr_2_5503509	Ca_desi_chr02	5503509	[A/G]	NA
Ca_Desi_Chr_2_5503519	Ca_desi_chr02	5503519	[G/A]	NA
Ca_Desi_Chr_2_5503727	Ca_desi_chr02	5503727	[T/G]	NA
Ca_Desi_Chr_2_5503732	Ca_desi_chr02	5503732	[G/C]	NA
Ca_Desi_Chr_2_5507928	Ca_desi_chr02	5507928	[A/C]	NA
Ca_Desi_Chr_2_5508906	Ca_desi_chr02	5508906	[T/A]	NA
Ca_Desi_Chr_2_5509470	Ca_desi_chr02	5509470	[T/G]	NA
Ca_Desi_Chr_2_5537769	Ca_desi_chr02	5537769	[A/T]	NA
Ca_Desi_Chr_2_5537950	Ca_desi_chr02	5537950	[A/C]	NA
Ca_Desi_Chr_2_5544027	Ca_desi_chr02	5544027	[A/G]	NA
Ca_Desi_Chr_2_5544042	Ca_desi_chr02	5544042	[A/G]	NA
Ca_Desi_Chr_2_5548807	Ca_desi_chr02	5548807	[C/G]	NA
Ca_Desi_Chr_2_5548819	Ca_desi_chr02	5548819	[G/A]	NA
Ca_Desi_Chr_2_5548885	Ca_desi_chr02	5548885	[T/A]	NA
Ca_Desi_Chr_2_5553723	Ca_desi_chr02	5553723	[G/A]	NA
Ca_Desi_Chr_2_5565953	Ca_desi_chr02	5565953	[A/G]	NA
Ca_Desi_Chr_2_5565956	Ca_desi_chr02	5565956	[A/G]	NA
Ca_Desi_Chr_2_5577178	Ca_desi_chr02	5577178	[T/A]	NA
Ca_Desi_Chr_2_5632020	Ca_desi_chr02	5632020	[T/G]	NA

SNP IDs	Chromosomes	Physical positions (bp)	SNPs	NCBI SNPdb accession IDs (NCBI_ss#)
Ca_Desi_Chr_2_5632028	Ca_desi_chr02	5632028	[T/G]	NA
Ca_Desi_Chr_2_5632039	Ca_desi_chr02	5632039	[A/C]	NA
Ca_Desi_Chr_2_5632049	Ca_desi_chr02	5632049	[T/G]	NA
Ca_Desi_Chr_2_5632065	Ca_desi_chr02	5632065	[T/C]	NA
Ca_Desi_Chr_2_5632369	Ca_desi_chr02	5632369	[T/C]	NA
Ca_Desi_Chr_2_5632370	Ca_desi_chr02	5632370	[C/T]	NA
Ca_Desi_Chr_2_5632371	Ca_desi_chr02	5632371	[T/C]	NA
Ca_Desi_Chr_2_5635057	Ca_desi_chr02	5635057	[T/G]	NA
Ca_Desi_Chr_2_5648192	Ca_desi_chr02	5648192	[C/T]	NA
Ca_Desi_Chr_2_5657507	Ca_desi_chr02	5657507	[A/T]	NA
Ca_Desi_Chr_2_5661445	Ca_desi_chr02	5661445	[T/G]	NA
Ca_Desi_Chr_2_5661457	Ca_desi_chr02	5661457	[T/G]	NA
Ca_Desi_Chr_2_5694139	Ca_desi_chr02	5694139	[T/G]	NA
Ca_Desi_Chr_2_5698170	Ca_desi_chr02	5698170	[T/A]	NA
Ca_Desi_Chr_2_5698197	Ca_desi_chr02	5698197	[G/A]	NA
Ca_Desi_Chr_2_5698412	Ca_desi_chr02	5698412	[A/G]	NA
Ca_Desi_Chr_2_5698976	Ca_desi_chr02	5698976	[C/T]	NA
Ca_Desi_Chr_2_5699314	Ca_desi_chr02	5699314	[A/G]	NA
Ca_Desi_Chr_2_5699404	Ca_desi_chr02	5699404	[T/C]	NA
Ca_Desi_Chr_2_5699406	Ca_desi_chr02	5699406	[A/C]	NA
Ca_Desi_Chr_2_5716475	Ca_desi_chr02	5716475	[C/T]	NA
Ca_Desi_Chr_2_5717021	Ca_desi_chr02	5717021	[G/C]	NA
Ca_Desi_Chr_2_5717899	Ca_desi_chr02	5717899	[G/A]	NA
Ca_Desi_Chr_2_5719843	Ca_desi_chr02	5719843	[C/T]	NA
Ca_Desi_Chr_2_5733756	Ca_desi_chr02	5733756	[C/A]	NA
Ca_Desi_Chr_2_5740309	Ca_desi_chr02	5740309	[C/A]	NA
Ca_Desi_Chr_2_5745551	Ca_desi_chr02	5745551	[G/T]	NA
Ca_Desi_Chr_2_5917903	Ca_desi_chr02	5917903	[T/C]	NA
Ca_Desi_Chr_2_5917920	Ca_desi_chr02	5917920	[G/T]	NA
Ca_Desi_Chr_2_5917931	Ca_desi_chr02	5917931	[T/G]	NA
Ca_Desi_Chr_2_5917936	Ca_desi_chr02	5917936	[C/T]	NA
Ca_Desi_Chr_2_5917946	Ca_desi_chr02	5917946	[T/A]	NA
Ca_Desi_Chr_2_5965390	Ca_desi_chr02	5965390	[A/C]	NA
Ca_Desi_Chr_2_5965398	Ca_desi_chr02	5965398	[T/G]	NA

SNP IDs	Chromosomes	Physical positions (bp)	SNPs	NCBI SNPdb accession IDs (NCBI_ss#)
Ca_Desi_Chr_2_5965430	Ca_desi_chr02	5965430	[A/G]	NA
Ca_Desi_Chr_2_6010840	Ca_desi_chr02	6010840	[G/A]	NA
Ca_Desi_Chr_2_6011026	Ca_desi_chr02	6011026	[G/A]	NA
Ca_Desi_Chr_2_6017319	Ca_desi_chr02	6017319	[T/C]	NA
Ca_Desi_Chr_2_6017510	Ca_desi_chr02	6017510	[T/G]	NA
Ca_Desi_Chr_2_6017921	Ca_desi_chr02	6017921	[C/T]	NA
Ca_Desi_Chr_2_6017932	Ca_desi_chr02	6017932	[A/C]	NA
Ca_Desi_Chr_2_6018033	Ca_desi_chr02	6018033	[C/T]	NA
Ca_Desi_Chr_2_6019972	Ca_desi_chr02	6019972	[A/G]	NA
Ca_Desi_Chr_2_6025668	Ca_desi_chr02	6025668	[A/G]	NA
Ca_Desi_Chr_2_6025730	Ca_desi_chr02	6025730	[G/T]	NA
Ca_Desi_Chr_2_6025741	Ca_desi_chr02	6025741	[A/G]	NA
Ca_Desi_Chr_2_6025764	Ca_desi_chr02	6025764	[A/C]	NA
Ca_Desi_Chr_2_6029971	Ca_desi_chr02	6029971	[A/G]	NA
Ca_Desi_Chr_2_6088205	Ca_desi_chr02	6088205	[T/C]	NA
Ca_Desi_Chr_2_6088226	Ca_desi_chr02	6088226	[T/C]	NA
Ca_Desi_Chr_2_6088236	Ca_desi_chr02	6088236	[G/C]	NA
Ca_Desi_Chr_2_6088245	Ca_desi_chr02	6088245	[G/A]	NA
Ca_Desi_Chr_2_6088248	Ca_desi_chr02	6088248	[C/A]	NA
Ca_Desi_Chr_2_6088252	Ca_desi_chr02	6088252	[A/G]	NA
Ca_Desi_Chr_2_6088260	Ca_desi_chr02	6088260	[A/C]	NA
Ca_Desi_Chr_2_6088265	Ca_desi_chr02	6088265	[T/C]	NA
Ca_Desi_Chr_2_6088304	Ca_desi_chr02	6088304	[C/T]	NA
Ca_Desi_Chr_2_6088443	Ca_desi_chr02	6088443	[A/G]	NA
Ca_Desi_Chr_2_6088463	Ca_desi_chr02	6088463	[T/G]	NA
Ca_Desi_Chr_2_6088467	Ca_desi_chr02	6088467	[A/T]	NA
Ca_Desi_Chr_2_6089048	Ca_desi_chr02	6089048	[T/C]	NA
Ca_Desi_Chr_2_6089357	Ca_desi_chr02	6089357	[C/T]	NA
Ca_Desi_Chr_2_6089359	Ca_desi_chr02	6089359	[A/G]	NA
Ca_Desi_Chr_2_6091962	Ca_desi_chr02	6091962	[C/T]	NA
Ca_Desi_Chr_2_6091970	Ca_desi_chr02	6091970	[A/C]	NA
Ca_Desi_Chr_2_6091973	Ca_desi_chr02	6091973	[T/G]	NA
Ca_Desi_Chr_2_6091980	Ca_desi_chr02	6091980	[T/C]	NA
Ca_Desi_Chr_2_6091983	Ca_desi_chr02	6091983	[C/T]	NA

SNP IDs	Chromosomes	Physical positions (bp)	SNPs	NCBI SNPdb accession IDs (NCBI_ss#)
Ca_Desi_Chr_2_6092092	Ca_desi_chr02	6092092	[G/A]	NA
Ca_Desi_Chr_2_6092094	Ca_desi_chr02	6092094	[A/G]	NA
Ca_Desi_Chr_2_6092148	Ca_desi_chr02	6092148	[T/G]	NA
Ca_Desi_Chr_2_6092158	Ca_desi_chr02	6092158	[G/C]	NA
Ca_Desi_Chr_2_6092165	Ca_desi_chr02	6092165	[T/G]	NA
Ca_Desi_Chr_2_6092200	Ca_desi_chr02	6092200	[A/C]	NA
Ca_Desi_Chr_2_6092205	Ca_desi_chr02	6092205	[A/C]	NA
Ca_Desi_Chr_2_6092347	Ca_desi_chr02	6092347	[T/G]	NA
Ca_Desi_Chr_2_6092376	Ca_desi_chr02	6092376	[T/C]	NA
Ca_Desi_Chr_2_6094379	Ca_desi_chr02	6094379	[C/G]	NA
Ca_Desi_Chr_2_6103617	Ca_desi_chr02	6103617	[A/T]	NA
Ca_Desi_Chr_2_6103902	Ca_desi_chr02	6103902	[T/C]	NA
Ca_Desi_Chr_2_6104164	Ca_desi_chr02	6104164	[T/G]	NA
Ca_Desi_Chr_2_6104305	Ca_desi_chr02	6104305	[A/G]	NA
Ca_Desi_Chr_2_6104365	Ca_desi_chr02	6104365	[C/T]	NA
Ca_Desi_Chr_2_6107404	Ca_desi_chr02	6107404	[T/A]	NA
Ca_Desi_Chr_2_6107474	Ca_desi_chr02	6107474	[T/G]	NA
Ca_Desi_Chr_2_6107483	Ca_desi_chr02	6107483	[T/G]	NA
Ca_Desi_Chr_2_6107492	Ca_desi_chr02	6107492	[T/G]	NA
Ca_Desi_Chr_2_6107519	Ca_desi_chr02	6107519	[A/C]	NA
Ca_Desi_Chr_2_6107526	Ca_desi_chr02	6107526	[A/G]	NA
Ca_Desi_Chr_2_6107530	Ca_desi_chr02	6107530	[T/C]	NA
Ca_Desi_Chr_2_6131587	Ca_desi_chr02	6131587	[T/C]	NA
Ca_Desi_Chr_2_6131704	Ca_desi_chr02	6131704	[T/C]	NA
Ca_Desi_Chr_2_6134866	Ca_desi_chr02	6134866	[T/G]	NA
Ca_Desi_Chr_2_6135210	Ca_desi_chr02	6135210	[G/A]	NA
Ca_Desi_Chr_2_6138059	Ca_desi_chr02	6138059	[C/T]	NA
Ca_Desi_Chr_2_6138789	Ca_desi_chr02	6138789	[T/C]	NA
Ca_Desi_Chr_2_6140423	Ca_desi_chr02	6140423	[G/A]	NA
Ca_Desi_Chr_2_6142753	Ca_desi_chr02	6142753	[T/G]	NA
Ca_Desi_Chr_2_6144056	Ca_desi_chr02	6144056	[G/T]	NA
Ca_Desi_Chr_2_6144083	Ca_desi_chr02	6144083	[G/C]	NA
Ca_Desi_Chr_2_6144088	Ca_desi_chr02	6144088	[T/A]	NA
Ca_Desi_Chr_2_6148501	Ca_desi_chr02	6148501	[G/A]	NA

SNP IDs	Chromosomes	Physical positions (bp)	SNPs	NCBI SNPdb accession IDs (NCBI_ss#)
Ca_Desi_Chr_2_6267621	Ca_desi_chr02	6267621	[T/C]	NA
Ca_Desi_Chr_2_6270242	Ca_desi_chr02	6270242	[T/G]	NA
Ca_Desi_Chr_2_6270265	Ca_desi_chr02	6270265	[T/G]	NA
Ca_Desi_Chr_2_6436332	Ca_desi_chr02	6436332	[A/T]	NA
Ca_Desi_Chr_2_6437145	Ca_desi_chr02	6437145	[C/T]	NA
Ca_Desi_Chr_2_6440301	Ca_desi_chr02	6440301	[C/T]	NA
Ca_Desi_Chr_2_6440323	Ca_desi_chr02	6440323	[T/C]	NA
Ca_Desi_Chr_2_6440324	Ca_desi_chr02	6440324	[G/A]	NA
Ca_Desi_Chr_2_6532976	Ca_desi_chr02	6532976	[T/G]	NA
Ca_Desi_Chr_2_6533732	Ca_desi_chr02	6533732	[C/T]	NA
Ca_Desi_Chr_2_6557277	Ca_desi_chr02	6557277	[T/G]	NA
Ca_Desi_Chr_2_6684226	Ca_desi_chr02	6684226	[T/C]	NA
Ca_Desi_Chr_2_6695692	Ca_desi_chr02	6695692	[A/C]	NA
Ca_Desi_Chr_2_6697269	Ca_desi_chr02	6697269	[T/A]	NA
Ca_Desi_Chr_2_6711737	Ca_desi_chr02	6711737	[C/A]	NA
Ca_Desi_Chr_2_6711738	Ca_desi_chr02	6711738	[T/G]	NA
Ca_Desi_Chr_2_6711829	Ca_desi_chr02	6711829	[T/G]	NA
Ca_Desi_Chr_2_6796974	Ca_desi_chr02	6796974	[T/C]	NA
Ca_Desi_Chr_2_6841451	Ca_desi_chr02	6841451	[T/G]	NA
Ca_Desi_Chr_2_6869453	Ca_desi_chr02	6869453	[A/G]	NA
Ca_Desi_Chr_2_6869512	Ca_desi_chr02	6869512	[A/G]	NA
Ca_Desi_Chr_2_6908937	Ca_desi_chr02	6908937	[A/G]	NA
Ca_Desi_Chr_2_6909024	Ca_desi_chr02	6909024	[A/C]	NA
Ca_Desi_Chr_2_6968271	Ca_desi_chr02	6968271	[C/G]	NA
Ca_Desi_Chr_2_6968273	Ca_desi_chr02	6968273	[T/G]	NA
Ca_Desi_Chr_2_6968275	Ca_desi_chr02	6968275	[C/T]	NA
Ca_Desi_Chr_2_6968279	Ca_desi_chr02	6968279	[G/C]	NA
Ca_Desi_Chr_2_7029728	Ca_desi_chr02	7029728	[A/G]	NA
Ca_Desi_Chr_2_7059768	Ca_desi_chr02	7059768	[A/C]	NA
Ca_Desi_Chr_2_7062008	Ca_desi_chr02	7062008	[T/G]	NA
Ca_Desi_Chr_2_7062016	Ca_desi_chr02	7062016	[A/C]	NA
Ca_Desi_Chr_2_7062076	Ca_desi_chr02	7062076	[T/C]	NA
Ca_Desi_Chr_2_7062082	Ca_desi_chr02	7062082	[T/A]	NA
Ca_Desi_Chr_2_7062151	Ca_desi_chr02	7062151	[A/C]	NA

SNP IDs	Chromosomes	Physical positions (bp)	SNPs	NCBI SNPdb accession IDs (NCBI_ss#)
Ca_Desi_Chr_2_7082410	Ca_desi_chr02	7082410	[T/C]	NA
Ca_Desi_Chr_2_7082448	Ca_desi_chr02	7082448	[T/A]	NA
Ca_Desi_Chr_2_7241488	Ca_desi_chr02	7241488	[A/G]	NA
Ca_Desi_Chr_2_7241492	Ca_desi_chr02	7241492	[T/A]	NA
Ca_Desi_Chr_2_7241496	Ca_desi_chr02	7241496	[A/G]	NA
Ca_Desi_Chr_2_7241508	Ca_desi_chr02	7241508	[T/C]	NA
Ca_Desi_Chr_2_7241512	Ca_desi_chr02	7241512	[T/C]	NA
Ca_Desi_Chr_2_7241513	Ca_desi_chr02	7241513	[T/C]	NA
Ca_Desi_Chr_2_7241529	Ca_desi_chr02	7241529	[T/C]	NA
Ca_Desi_Chr_2_7241538	Ca_desi_chr02	7241538	[T/A]	NA
Ca_Desi_Chr_2_7241736	Ca_desi_chr02	7241736	[T/C]	NA
Ca_Desi_Chr_2_7241742	Ca_desi_chr02	7241742	[A/G]	NA
Ca_Desi_Chr_2_7241760	Ca_desi_chr02	7241760	[A/G]	NA
Ca_Desi_Chr_2_7241761	Ca_desi_chr02	7241761	[A/C]	NA
Ca_Desi_Chr_2_7241762	Ca_desi_chr02	7241762	[C/A]	NA
Ca_Desi_Chr_2_7241764	Ca_desi_chr02	7241764	[T/C]	NA
Ca_Desi_Chr_2_7241765	Ca_desi_chr02	7241765	[G/T]	NA
Ca_Desi_Chr_2_7241767	Ca_desi_chr02	7241767	[T/C]	NA
Ca_Desi_Chr_2_7241770	Ca_desi_chr02	7241770	[G/T]	NA
Ca_Desi_Chr_2_7241771	Ca_desi_chr02	7241771	[C/G]	NA
Ca_Desi_Chr_2_7241776	Ca_desi_chr02	7241776	[G/T]	NA
Ca_Desi_Chr_2_7241782	Ca_desi_chr02	7241782	[G/T]	NA
Ca_Desi_Chr_2_7241783	Ca_desi_chr02	7241783	[C/G]	NA
Ca_Desi_Chr_2_7241793	Ca_desi_chr02	7241793	[A/G]	NA
Ca_Desi_Chr_2_7242012	Ca_desi_chr02	7242012	[T/G]	NA
Ca_Desi_Chr_2_7242138	Ca_desi_chr02	7242138	[T/G]	NA
Ca_Desi_Chr_2_7242197	Ca_desi_chr02	7242197	[T/G]	NA
Ca_Desi_Chr_2_7242231	Ca_desi_chr02	7242231	[T/G]	NA
Ca_Desi_Chr_2_7242933	Ca_desi_chr02	7242933	[A/C]	NA
Ca_Desi_Chr_2_7314336	Ca_desi_chr02	7314336	[A/C]	NA
Ca_Desi_Chr_2_7394649	Ca_desi_chr02	7394649	[T/C]	NA
Ca_Desi_Chr_2_7430532	Ca_desi_chr02	7430532	[G/C]	NA
Ca_Desi_Chr_2_7458498	Ca_desi_chr02	7458498	[A/C]	NA
Ca_Desi_Chr_2_7459213	Ca_desi_chr02	7459213	[A/G]	NA

SNP IDs	Chromosomes	Physical positions (bp)	SNPs	NCBI SNPdb accession IDs (NCBI_ss#)
Ca_Desi_Chr_2_7459421	Ca_desi_chr02	7459421	[A/C]	NA
Ca_Desi_Chr_2_7462045	Ca_desi_chr02	7462045	[A/G]	NA
Ca_Desi_Chr_2_7462049	Ca_desi_chr02	7462049	[A/C]	NA
Ca_Desi_Chr_2_7462051	Ca_desi_chr02	7462051	[A/C]	NA
Ca_Desi_Chr_2_7462054	Ca_desi_chr02	7462054	[A/G]	NA
Ca_Desi_Chr_2_7462059	Ca_desi_chr02	7462059	[T/C]	NA
Ca_Desi_Chr_2_7462073	Ca_desi_chr02	7462073	[A/G]	NA
Ca_Desi_Chr_2_7462092	Ca_desi_chr02	7462092	[T/C]	NA
Ca_Desi_Chr_2_7462100	Ca_desi_chr02	7462100	[T/A]	NA
Ca_Desi_Chr_2_7610218	Ca_desi_chr02	7610218	[T/G]	NA
Ca_Desi_Chr_2_7630495	Ca_desi_chr02	7630495	[T/G]	NA
Ca_Desi_Chr_2_7630506	Ca_desi_chr02	7630506	[A/G]	NA
Ca_Desi_Chr_2_7630508	Ca_desi_chr02	7630508	[T/G]	NA
Ca_Desi_Chr_2_7699780	Ca_desi_chr02	7699780	[C/A]	NA
Ca_Desi_Chr_2_7699781	Ca_desi_chr02	7699781	[T/G]	NA
Ca_Desi_Chr_2_7699782	Ca_desi_chr02	7699782	[T/A]	NA
Ca_Desi_Chr_2_7699783	Ca_desi_chr02	7699783	[C/T]	NA
Ca_Desi_Chr_2_7708750	Ca_desi_chr02	7708750	[T/C]	NA
Ca_Desi_Chr_2_7708754	Ca_desi_chr02	7708754	[T/C]	NA
Ca_Desi_Chr_2_7708756	Ca_desi_chr02	7708756	[A/G]	NA
Ca_Desi_Chr_2_7708765	Ca_desi_chr02	7708765	[A/C]	NA
Ca_Desi_Chr_2_7708773	Ca_desi_chr02	7708773	[T/C]	NA
Ca_Desi_Chr_2_7708776	Ca_desi_chr02	7708776	[T/C]	NA
Ca_Desi_Chr_2_7708788	Ca_desi_chr02	7708788	[A/G]	NA
Ca_Desi_Chr_2_7708797	Ca_desi_chr02	7708797	[A/G]	NA
Ca_Desi_Chr_2_7708799	Ca_desi_chr02	7708799	[T/C]	NA
Ca_Desi_Chr_2_7709713	Ca_desi_chr02	7709713	[A/T]	NA
Ca_Desi_Chr_2_7726210	Ca_desi_chr02	7726210	[A/C]	NA
Ca_Desi_Chr_2_7732284	Ca_desi_chr02	7732284	[T/G]	NA
Ca_Desi_Chr_2_7732319	Ca_desi_chr02	7732319	[A/C]	NA
Ca_Desi_Chr_2_7810363	Ca_desi_chr02	7810363	[T/C]	NA
Ca_Desi_Chr_2_7810373	Ca_desi_chr02	7810373	[T/G]	NA
Ca_Desi_Chr_2_7813832	Ca_desi_chr02	7813832	[T/G]	NA
Ca_Desi_Chr_2_7814065	Ca_desi_chr02	7814065	[T/G]	NA

SNP IDs	Chromosomes	Physical positions (bp)	SNPs	NCBI SNPdb accession IDs (NCBI_ss#)
Ca_Desi_Chr_2_7814069	Ca_desi_chr02	7814069	[G/T]	NA
Ca_Desi_Chr_2_7814090	Ca_desi_chr02	7814090	[A/G]	NA
Ca_Desi_Chr_2_7814097	Ca_desi_chr02	7814097	[A/G]	NA
Ca_Desi_Chr_2_7814101	Ca_desi_chr02	7814101	[T/C]	NA
Ca_Desi_Chr_2_7814103	Ca_desi_chr02	7814103	[T/G]	NA
Ca_Desi_Chr_2_7814115	Ca_desi_chr02	7814115	[T/C]	NA
Ca_Desi_Chr_2_7886723	Ca_desi_chr02	7886723	[C/T]	NA
Ca_Desi_Chr_2_7894131	Ca_desi_chr02	7894131	[A/C]	NA
Ca_Desi_Chr_2_7894150	Ca_desi_chr02	7894150	[A/C]	NA
Ca_Desi_Chr_2_7897881	Ca_desi_chr02	7897881	[C/A]	NA
Ca_Desi_Chr_2_7907189	Ca_desi_chr02	7907189	[T/G]	NA
Ca_Desi_Chr_2_7907191	Ca_desi_chr02	7907191	[T/C]	NA
Ca_Desi_Chr_2_7907198	Ca_desi_chr02	7907198	[T/G]	NA
Ca_Desi_Chr_2_7907215	Ca_desi_chr02	7907215	[T/G]	NA
Ca_Desi_Chr_2_7908862	Ca_desi_chr02	7908862	[G/T]	NA
Ca_Desi_Chr_2_7911889	Ca_desi_chr02	7911889	[A/C]	NA
Ca_Desi_Chr_2_7956698	Ca_desi_chr02	7956698	[A/T]	NA
Ca_Desi_Chr_2_7956702	Ca_desi_chr02	7956702	[T/G]	NA
Ca_Desi_Chr_2_7956710	Ca_desi_chr02	7956710	[A/G]	NA
Ca_Desi_Chr_2_7958537	Ca_desi_chr02	7958537	[A/G]	NA
Ca_Desi_Chr_2_7959810	Ca_desi_chr02	7959810	[T/G]	NA
Ca_Desi_Chr_2_7980007	Ca_desi_chr02	7980007	[T/G]	NA
Ca_Desi_Chr_2_7980010	Ca_desi_chr02	7980010	[T/G]	NA
Ca_Desi_Chr_2_8012689	Ca_desi_chr02	8012689	[C/A]	NA
Ca_Desi_Chr_2_8013247	Ca_desi_chr02	8013247	[T/C]	NA
Ca_Desi_Chr_2_8037205	Ca_desi_chr02	8037205	[A/C]	NA
Ca_Desi_Chr_2_8065947	Ca_desi_chr02	8065947	[A/T]	NA
Ca_Desi_Chr_2_8065976	Ca_desi_chr02	8065976	[T/C]	NA
Ca_Desi_Chr_2_8113708	Ca_desi_chr02	8113708	[T/G]	NA
Ca_Desi_Chr_2_8113723	Ca_desi_chr02	8113723	[C/T]	NA
Ca_Desi_Chr_2_8113738	Ca_desi_chr02	8113738	[C/T]	NA
Ca_Desi_Chr_2_8149819	Ca_desi_chr02	8149819	[T/C]	NA
Ca_Desi_Chr_2_8160750	Ca_desi_chr02	8160750	[A/G]	NA
Ca_Desi_Chr_2_8167139	Ca_desi_chr02	8167139	[T/C]	NA

SNP IDs	Chromosomes	Physical positions (bp)	SNPs	NCBI SNPdb accession IDs (NCBI_ss#)
Ca_Desi_Chr_2_8167166	Ca_desi_chr02	8167166	[T/C]	NA
Ca_Desi_Chr_2_8167697	Ca_desi_chr02	8167697	[T/C]	NA
Ca_Desi_Chr_2_8167719	Ca_desi_chr02	8167719	[T/C]	NA
Ca_Desi_Chr_2_8167729	Ca_desi_chr02	8167729	[A/G]	NA
Ca_Desi_Chr_2_8167781	Ca_desi_chr02	8167781	[T/G]	NA
Ca_Desi_Chr_2_8174383	Ca_desi_chr02	8174383	[T/G]	NA
Ca_Desi_Chr_2_8234036	Ca_desi_chr02	8234036	[T/C]	NA
Ca_Desi_Chr_2_8234042	Ca_desi_chr02	8234042	[A/G]	NA
Ca_Desi_Chr_2_8234047	Ca_desi_chr02	8234047	[A/G]	NA
Ca_Desi_Chr_2_8234048	Ca_desi_chr02	8234048	[A/G]	NA
Ca_Desi_Chr_2_8234050	Ca_desi_chr02	8234050	[A/G]	NA
Ca_Desi_Chr_2_8234059	Ca_desi_chr02	8234059	[T/C]	NA
Ca_Desi_Chr_2_8234067	Ca_desi_chr02	8234067	[A/G]	NA
Ca_Desi_Chr_2_8234071	Ca_desi_chr02	8234071	[A/G]	NA
Ca_Desi_Chr_2_8234076	Ca_desi_chr02	8234076	[T/C]	NA
Ca_Desi_Chr_2_8234078	Ca_desi_chr02	8234078	[A/G]	NA
Ca_Desi_Chr_2_8234083	Ca_desi_chr02	8234083	[A/G]	NA
Ca_Desi_Chr_2_8264499	Ca_desi_chr02	8264499	[A/G]	NA
Ca_Desi_Chr_2_8264509	Ca_desi_chr02	8264509	[T/C]	NA
Ca_Desi_Chr_2_8264540	Ca_desi_chr02	8264540	[A/G]	NA
Ca_Desi_Chr_2_8294596	Ca_desi_chr02	8294596	[C/T]	NA
Ca_Desi_Chr_2_8322715	Ca_desi_chr02	8322715	[A/G]	NA
Ca_Desi_Chr_2_8322717	Ca_desi_chr02	8322717	[A/G]	NA
Ca_Desi_Chr_2_8322723	Ca_desi_chr02	8322723	[A/G]	NA
Ca_Desi_Chr_2_8322735	Ca_desi_chr02	8322735	[A/G]	NA
Ca_Desi_Chr_2_8322810	Ca_desi_chr02	8322810	[T/G]	NA
Ca_Desi_Chr_2_8397866	Ca_desi_chr02	8397866	[G/A]	NA
Ca_Desi_Chr_2_8397883	Ca_desi_chr02	8397883	[A/G]	NA
Ca_Desi_Chr_2_8397896	Ca_desi_chr02	8397896	[A/C]	NA
Ca_Desi_Chr_2_8397956	Ca_desi_chr02	8397956	[T/G]	NA
Ca_Desi_Chr_2_8397968	Ca_desi_chr02	8397968	[T/G]	NA
Ca_Desi_Chr_2_8398016	Ca_desi_chr02	8398016	[A/T]	NA
Ca_Desi_Chr_2_8436239	Ca_desi_chr02	8436239	[C/A]	NA
Ca_Desi_Chr_2_8436240	Ca_desi_chr02	8436240	[A/G]	NA

SNP IDs	Chromosomes	Physical positions (bp)	SNPs	NCBI SNPdb accession IDs (NCBI_ss#)
Ca_Desi_Chr_2_8436241	Ca_desi_chr02	8436241	[G/A]	NA
Ca_Desi_Chr_2_8436278	Ca_desi_chr02	8436278	[A/C]	NA
Ca_Desi_Chr_2_8436331	Ca_desi_chr02	8436331	[T/C]	NA
Ca_Desi_Chr_2_8436346	Ca_desi_chr02	8436346	[A/C]	NA
Ca_Desi_Chr_2_8436354	Ca_desi_chr02	8436354	[A/C]	NA
Ca_Desi_Chr_2_8465571	Ca_desi_chr02	8465571	[T/C]	NA
Ca_Desi_Chr_2_8465607	Ca_desi_chr02	8465607	[A/G]	NA
Ca_Desi_Chr_2_8495240	Ca_desi_chr02	8495240	[T/G]	NA
Ca_Desi_Chr_2_8510313	Ca_desi_chr02	8510313	[C/T]	NA
Ca_Desi_Chr_2_8530697	Ca_desi_chr02	8530697	[T/G]	NA
Ca_Desi_Chr_2_8562610	Ca_desi_chr02	8562610	[T/C]	NA
Ca_Desi_Chr_2_8562634	Ca_desi_chr02	8562634	[A/G]	NA
Ca_Desi_Chr_2_8562636	Ca_desi_chr02	8562636	[T/C]	NA
Ca_Desi_Chr_2_8562643	Ca_desi_chr02	8562643	[T/G]	NA
Ca_Desi_Chr_2_8568490	Ca_desi_chr02	8568490	[C/T]	NA
Ca_Desi_Chr_2_8568507	Ca_desi_chr02	8568507	[T/C]	NA
Ca_Desi_Chr_2_8568508	Ca_desi_chr02	8568508	[G/T]	NA
Ca_Desi_Chr_2_9844991	Ca_desi_chr02	9844991	[A/C]	NA
Ca_Desi_Chr_2_9873040	Ca_desi_chr02	9873040	[T/C]	NA
Ca_Desi_Chr_2_9873100	Ca_desi_chr02	9873100	[A/C]	NA
Ca_Desi_Chr_2_9873103	Ca_desi_chr02	9873103	[A/C]	NA
Ca_Desi_Chr_2_9873163	Ca_desi_chr02	9873163	[T/C]	NA
Ca_Desi_Chr_2_9873176	Ca_desi_chr02	9873176	[A/T]	NA
Ca_Desi_Chr_2_9885462	Ca_desi_chr02	9885462	[A/C]	NA
Ca_Desi_Chr_2_10370225	Ca_desi_chr02	10370225	[T/C]	NA
Ca_Desi_Chr_2_10436496	Ca_desi_chr02	10436496	[T/G]	NA
Ca_Desi_Chr_2_10436512	Ca_desi_chr02	10436512	[T/C]	NA
Ca_Desi_Chr_2_10436614	Ca_desi_chr02	10436614	[C/A]	NA
Ca_Desi_Chr_2_10776571	Ca_desi_chr02	10776571	[A/C]	NA
Ca_Desi_Chr_2_10864698	Ca_desi_chr02	10864698	[A/G]	NA
Ca_Desi_Chr_2_10864699	Ca_desi_chr02	10864699	[G/A]	NA
Ca_Desi_Chr_2_10864722	Ca_desi_chr02	10864722	[T/C]	NA
Ca_Desi_Chr_2_10864728	Ca_desi_chr02	10864728	[C/A]	NA
Ca_Desi_Chr_2_11084623	Ca_desi_chr02	11084623	[C/T]	NA

SNP IDs	Chromosomes	Physical positions (bp)	SNPs	NCBI SNPdb accession IDs (NCBI_ss#)
Ca_Desi_Chr_2_11110656	Ca_desi_chr02	11110656	[T/G]	NA
Ca_Desi_Chr_2_11170287	Ca_desi_chr02	11170287	[T/A]	NA
Ca_Desi_Chr_2_11170288	Ca_desi_chr02	11170288	[A/T]	NA
Ca_Desi_Chr_2_11276117	Ca_desi_chr02	11276117	[T/C]	NA
Ca_Desi_Chr_2_11366639	Ca_desi_chr02	11366639	[C/G]	NA
Ca_Desi_Chr_2_11366670	Ca_desi_chr02	11366670	[A/T]	NA
Ca_Desi_Chr_2_11393824	Ca_desi_chr02	11393824	[C/T]	NA
Ca_Desi_Chr_2_11393862	Ca_desi_chr02	11393862	[C/G]	NA
Ca_Desi_Chr_2_11394066	Ca_desi_chr02	11394066	[A/G]	NA
Ca_Desi_Chr_2_11394075	Ca_desi_chr02	11394075	[G/A]	NA
Ca_Desi_Chr_2_11394153	Ca_desi_chr02	11394153	[C/T]	NA
Ca_Desi_Chr_2_11403656	Ca_desi_chr02	11403656	[C/T]	NA
Ca_Desi_Chr_2_11403678	Ca_desi_chr02	11403678	[C/T]	NA
Ca_Desi_Chr_2_11403821	Ca_desi_chr02	11403821	[T/A]	NA
Ca_Desi_Chr_2_11403828	Ca_desi_chr02	11403828	[C/T]	NA
Ca_Desi_Chr_2_11403900	Ca_desi_chr02	11403900	[C/A]	NA
Ca_Desi_Chr_2_11422719	Ca_desi_chr02	11422719	[G/T]	NA
Ca_Desi_Chr_2_11422759	Ca_desi_chr02	11422759	[T/A]	NA
Ca_Desi_Chr_2_11423000	Ca_desi_chr02	11423000	[C/A]	NA
Ca_Desi_Chr_2_11423022	Ca_desi_chr02	11423022	[C/T]	NA
Ca_Desi_Chr_2_11423145	Ca_desi_chr02	11423145	[G/A]	NA
Ca_Desi_Chr_2_11423165	Ca_desi_chr02	11423165	[T/A]	NA
Ca_Desi_Chr_2_11634542	Ca_desi_chr02	11634542	[A/C]	NA
Ca_Desi_Chr_2_11680277	Ca_desi_chr02	11680277	[T/C]	NA
Ca_Desi_Chr_2_11853713	Ca_desi_chr02	11853713	[A/G]	NA
Ca_Desi_Chr_2_11979951	Ca_desi_chr02	11979951	[A/C]	NA
Ca_Desi_Chr_2_11979952	Ca_desi_chr02	11979952	[G/T]	NA
Ca_Desi_Chr_2_11979953	Ca_desi_chr02	11979953	[A/T]	NA
Ca_Desi_Chr_2_11985730	Ca_desi_chr02	11985730	[A/C]	NA
Ca_Desi_Chr_2_12071136	Ca_desi_chr02	12071136	[T/C]	NA
Ca_Desi_Chr_2_12071223	Ca_desi_chr02	12071223	[T/A]	NA
Ca_Desi_Chr_2_12071282	Ca_desi_chr02	12071282	[A/C]	NA
Ca_Desi_Chr_2_12072107	Ca_desi_chr02	12072107	[G/A]	NA
Ca_Desi_Chr_2_12072114	Ca_desi_chr02	12072114	[A/C]	NA

SNP IDs	Chromosomes	Physical positions (bp)	SNPs	NCBI SNPdb accession IDs (NCBI_ss#)
Ca_Desi_Chr_2_12271439	Ca_desi_chr02	12271439	[T/G]	NA
Ca_Desi_Chr_2_12280233	Ca_desi_chr02	12280233	[G/A]	NA
Ca_Desi_Chr_2_12280249	Ca_desi_chr02	12280249	[A/T]	NA
Ca_Desi_Chr_2_12280263	Ca_desi_chr02	12280263	[A/C]	NA
Ca_Desi_Chr_2_12311555	Ca_desi_chr02	12311555	[A/C]	NA
Ca_Desi_Chr_2_12417990	Ca_desi_chr02	12417990	[A/G]	NA
Ca_Desi_Chr_2_12418007	Ca_desi_chr02	12418007	[A/C]	NA
Ca_Desi_Chr_2_12478434	Ca_desi_chr02	12478434	[A/G]	NA
Ca_Desi_Chr_2_12478439	Ca_desi_chr02	12478439	[G/C]	NA
Ca_Desi_Chr_2_12478444	Ca_desi_chr02	12478444	[C/T]	NA
Ca_Desi_Chr_2_12478467	Ca_desi_chr02	12478467	[A/G]	NA
Ca_Desi_Chr_2_12489642	Ca_desi_chr02	12489642	[T/G]	NA
Ca_Desi_Chr_2_12518481	Ca_desi_chr02	12518481	[G/T]	NA
Ca_Desi_Chr_2_12560867	Ca_desi_chr02	12560867	[A/T]	NA
Ca_Desi_Chr_2_12609758	Ca_desi_chr02	12609758	[T/G]	NA
Ca_Desi_Chr_2_12729592	Ca_desi_chr02	12729592	[A/C]	NA
Ca_Desi_Chr_2_12729595	Ca_desi_chr02	12729595	[A/C]	NA
Ca_Desi_Chr_2_12729864	Ca_desi_chr02	12729864	[A/G]	NA
Ca_Desi_Chr_2_12729870	Ca_desi_chr02	12729870	[T/A]	NA
Ca_Desi_Chr_2_12729884	Ca_desi_chr02	12729884	[G/A]	NA
Ca_Desi_Chr_2_12729885	Ca_desi_chr02	12729885	[T/A]	NA
Ca_Desi_Chr_2_12796796	Ca_desi_chr02	12796796	[T/A]	NA
Ca_Desi_Chr_2_12796811	Ca_desi_chr02	12796811	[A/T]	NA
Ca_Desi_Chr_2_12796832	Ca_desi_chr02	12796832	[T/C]	NA
Ca_Desi_Chr_2_12796839	Ca_desi_chr02	12796839	[A/G]	NA
Ca_Desi_Chr_2_12796843	Ca_desi_chr02	12796843	[T/C]	NA
Ca_Desi_Chr_2_12796858	Ca_desi_chr02	12796858	[T/C]	NA
Ca_Desi_Chr_2_12796865	Ca_desi_chr02	12796865	[T/G]	NA
Ca_Desi_Chr_2_12796868	Ca_desi_chr02	12796868	[A/G]	NA
Ca_Desi_Chr_2_12796870	Ca_desi_chr02	12796870	[A/G]	NA
Ca_Desi_Chr_2_12796876	Ca_desi_chr02	12796876	[T/C]	NA
Ca_Desi_Chr_2_12796878	Ca_desi_chr02	12796878	[G/T]	NA
Ca_Desi_Chr_2_12796882	Ca_desi_chr02	12796882	[T/G]	NA
Ca_Desi_Chr_2_12796886	Ca_desi_chr02	12796886	[A/T]	NA

SNP IDs	Chromosomes	Physical positions (bp)	SNPs	NCBI SNPdb accession IDs (NCBI_ss#)
Ca_Desi_Chr_2_12797598	Ca_desi_chr02	12797598	[G/A]	NA
Ca_Desi_Chr_2_12797640	Ca_desi_chr02	12797640	[G/A]	NA
Ca_Desi_Chr_2_12833383	Ca_desi_chr02	12833383	[T/G]	NA
Ca_Desi_Chr_2_12835773	Ca_desi_chr02	12835773	[C/A]	NA
Ca_Desi_Chr_2_12835774	Ca_desi_chr02	12835774	[T/G]	NA
Ca_Desi_Chr_2_12863996	Ca_desi_chr02	12863996	[T/A]	NA
Ca_Desi_Chr_2_12864035	Ca_desi_chr02	12864035	[A/G]	NA
Ca_Desi_Chr_2_12985136	Ca_desi_chr02	12985136	[G/A]	NA
Ca_Desi_Chr_2_13002878	Ca_desi_chr02	13002878	[A/G]	NA
Ca_Desi_Chr_2_13002894	Ca_desi_chr02	13002894	[T/A]	NA
Ca_Desi_Chr_2_13002923	Ca_desi_chr02	13002923	[T/G]	NA
Ca_Desi_Chr_2_13016783	Ca_desi_chr02	13016783	[A/G]	NA
Ca_Desi_Chr_2_13016804	Ca_desi_chr02	13016804	[T/C]	NA
Ca_Desi_Chr_2_13016805	Ca_desi_chr02	13016805	[T/C]	NA
Ca_Desi_Chr_2_13016810	Ca_desi_chr02	13016810	[T/C]	NA
Ca_Desi_Chr_2_13016833	Ca_desi_chr02	13016833	[A/G]	NA
Ca_Desi_Chr_2_13038360	Ca_desi_chr02	13038360	[A/T]	NA
Ca_Desi_Chr_2_13039224	Ca_desi_chr02	13039224	[A/T]	NA
Ca_Desi_Chr_2_13122853	Ca_desi_chr02	13122853	[T/G]	NA
Ca_Desi_Chr_2_13127040	Ca_desi_chr02	13127040	[T/A]	NA
Ca_Desi_Chr_2_13147111	Ca_desi_chr02	13147111	[T/G]	NA
Ca_Desi_Chr_2_13147119	Ca_desi_chr02	13147119	[A/T]	NA
Ca_Desi_Chr_2_13152854	Ca_desi_chr02	13152854	[A/G]	NA
Ca_Desi_Chr_2_13152868	Ca_desi_chr02	13152868	[T/C]	NA
Ca_Desi_Chr_2_13163809	Ca_desi_chr02	13163809	[A/C]	NA
Ca_Desi_Chr_2_13260498	Ca_desi_chr02	13260498	[G/A]	NA
Ca_Desi_Chr_2_13260559	Ca_desi_chr02	13260559	[G/A]	NA
Ca_Desi_Chr_2_13260562	Ca_desi_chr02	13260562	[T/G]	NA
Ca_Desi_Chr_2_13262329	Ca_desi_chr02	13262329	[A/C]	NA
Ca_Desi_Chr_2_13410457	Ca_desi_chr02	13410457	[G/T]	NA
Ca_Desi_Chr_2_13413397	Ca_desi_chr02	13413397	[T/C]	NA
Ca_Desi_Chr_2_13443269	Ca_desi_chr02	13443269	[T/G]	NA
Ca_Desi_Chr_2_13536762	Ca_desi_chr02	13536762	[A/C]	NA
Ca_Desi_Chr_2_13900388	Ca_desi_chr02	13900388	[A/C]	NA

SNP IDs	Chromosomes	Physical positions (bp)	SNPs	NCBI SNPdb accession IDs (NCBI_ss#)
Ca_Desi_Chr_2_13924504	Ca_desi_chr02	13924504	[T/C]	NA
Ca_Desi_Chr_2_13924506	Ca_desi_chr02	13924506	[A/T]	NA
Ca_Desi_Chr_2_13924569	Ca_desi_chr02	13924569	[A/C]	NA
Ca_Desi_Chr_2_13924598	Ca_desi_chr02	13924598	[A/C]	NA
Ca_Desi_Chr_2_13941805	Ca_desi_chr02	13941805	[A/C]	NA
Ca_Desi_Chr_2_13941833	Ca_desi_chr02	13941833	[T/G]	NA
Ca_Desi_Chr_2_13941844	Ca_desi_chr02	13941844	[C/G]	NA
Ca_Desi_Chr_2_13942330	Ca_desi_chr02	13942330	[T/C]	NA
Ca_Desi_Chr_2_13980480	Ca_desi_chr02	13980480	[T/G]	NA
Ca_Desi_Chr_2_14007286	Ca_desi_chr02	14007286	[C/G]	NA
Ca_Desi_Chr_2_14007287	Ca_desi_chr02	14007287	[T/G]	NA
Ca_Desi_Chr_2_14051746	Ca_desi_chr02	14051746	[T/C]	NA
Ca_Desi_Chr_2_14051749	Ca_desi_chr02	14051749	[A/C]	NA
Ca_Desi_Chr_2_14064974	Ca_desi_chr02	14064974	[T/A]	NA
Ca_Desi_Chr_2_14068074	Ca_desi_chr02	14068074	[T/G]	NA
Ca_Desi_Chr_2_14068088	Ca_desi_chr02	14068088	[T/G]	NA
Ca_Desi_Chr_2_14124391	Ca_desi_chr02	14124391	[A/C]	NA
Ca_Desi_Chr_2_14124411	Ca_desi_chr02	14124411	[T/C]	NA
Ca_Desi_Chr_2_14124416	Ca_desi_chr02	14124416	[A/G]	NA
Ca_Desi_Chr_2_14124419	Ca_desi_chr02	14124419	[A/G]	NA
Ca_Desi_Chr_2_14124421	Ca_desi_chr02	14124421	[A/C]	NA
Ca_Desi_Chr_2_14160600	Ca_desi_chr02	14160600	[A/G]	NA
Ca_Desi_Chr_2_14163542	Ca_desi_chr02	14163542	[A/C]	NA
Ca_Desi_Chr_2_14163551	Ca_desi_chr02	14163551	[A/C]	NA
Ca_Desi_Chr_2_14163612	Ca_desi_chr02	14163612	[A/C]	NA
Ca_Desi_Chr_2_14163618	Ca_desi_chr02	14163618	[A/C]	NA
Ca_Desi_Chr_2_14163647	Ca_desi_chr02	14163647	[C/T]	NA
Ca_Desi_Chr_2_14169133	Ca_desi_chr02	14169133	[C/T]	NA
Ca_Desi_Chr_2_14169300	Ca_desi_chr02	14169300	[C/T]	NA
Ca_Desi_Chr_2_14209948	Ca_desi_chr02	14209948	[A/C]	NA
Ca_Desi_Chr_2_14209949	Ca_desi_chr02	14209949	[A/G]	NA
Ca_Desi_Chr_2_14209951	Ca_desi_chr02	14209951	[A/T]	NA
Ca_Desi_Chr_2_14209961	Ca_desi_chr02	14209961	[T/C]	NA
Ca_Desi_Chr_2_14211958	Ca_desi_chr02	14211958	[A/T]	NA

SNP IDs	Chromosomes	Physical positions (bp)	SNPs	NCBI SNPdb accession IDs (NCBI_ss#)
Ca_Desi_Chr_2_14212005	Ca_desi_chr02	14212005	[A/G]	NA
Ca_Desi_Chr_2_14225002	Ca_desi_chr02	14225002	[A/G]	NA
Ca_Desi_Chr_2_14225008	Ca_desi_chr02	14225008	[A/G]	NA
Ca_Desi_Chr_2_14225009	Ca_desi_chr02	14225009	[G/T]	NA
Ca_Desi_Chr_2_14225010	Ca_desi_chr02	14225010	[T/C]	NA
Ca_Desi_Chr_2_14225012	Ca_desi_chr02	14225012	[T/C]	NA
Ca_Desi_Chr_2_14225013	Ca_desi_chr02	14225013	[C/A]	NA
Ca_Desi_Chr_2_14225016	Ca_desi_chr02	14225016	[A/G]	NA
Ca_Desi_Chr_2_14225017	Ca_desi_chr02	14225017	[G/C]	NA
Ca_Desi_Chr_2_14225019	Ca_desi_chr02	14225019	[C/A]	NA
Ca_Desi_Chr_2_14225020	Ca_desi_chr02	14225020	[A/G]	NA
Ca_Desi_Chr_2_14225033	Ca_desi_chr02	14225033	[A/G]	NA
Ca_Desi_Chr_2_14225038	Ca_desi_chr02	14225038	[C/A]	NA
Ca_Desi_Chr_2_14225039	Ca_desi_chr02	14225039	[T/C]	NA
Ca_Desi_Chr_2_14225056	Ca_desi_chr02	14225056	[T/A]	NA
Ca_Desi_Chr_2_14225059	Ca_desi_chr02	14225059	[A/G]	NA
Ca_Desi_Chr_2_14225062	Ca_desi_chr02	14225062	[T/A]	NA
Ca_Desi_Chr_2_14406592	Ca_desi_chr02	14406592	[T/C]	NA
Ca_Desi_Chr_2_14406613	Ca_desi_chr02	14406613	[A/G]	NA
Ca_Desi_Chr_2_14406640	Ca_desi_chr02	14406640	[T/C]	NA
Ca_Desi_Chr_2_14445991	Ca_desi_chr02	14445991	[C/A]	NA
Ca_Desi_Chr_2_14445992	Ca_desi_chr02	14445992	[A/G]	NA
Ca_Desi_Chr_2_14445993	Ca_desi_chr02	14445993	[T/A]	NA
Ca_Desi_Chr_2_14445995	Ca_desi_chr02	14445995	[A/C]	NA
Ca_Desi_Chr_2_14445996	Ca_desi_chr02	14445996	[T/G]	NA
Ca_Desi_Chr_2_14446035	Ca_desi_chr02	14446035	[T/A]	NA
Ca_Desi_Chr_2_14449492	Ca_desi_chr02	14449492	[A/C]	NA
Ca_Desi_Chr_2_14449506	Ca_desi_chr02	14449506	[A/C]	NA
Ca_Desi_Chr_2_14489890	Ca_desi_chr02	14489890	[A/C]	NA
Ca_Desi_Chr_2_14489912	Ca_desi_chr02	14489912	[T/A]	NA
Ca_Desi_Chr_2_14489920	Ca_desi_chr02	14489920	[T/C]	NA
Ca_Desi_Chr_2_14493800	Ca_desi_chr02	14493800	[T/G]	NA
Ca_Desi_Chr_2_14493803	Ca_desi_chr02	14493803	[T/G]	NA
Ca_Desi_Chr_2_14493808	Ca_desi_chr02	14493808	[T/G]	NA

SNP IDs	Chromosomes	Physical positions (bp)	SNPs	NCBI SNPdb accession IDs (NCBI_ss#)
Ca_Desi_Chr_2_14493834	Ca_desi_chr02	14493834	[G/A]	NA
Ca_Desi_Chr_2_14493836	Ca_desi_chr02	14493836	[A/G]	NA
Ca_Desi_Chr_2_14493837	Ca_desi_chr02	14493837	[G/A]	NA
Ca_Desi_Chr_2_14493838	Ca_desi_chr02	14493838	[C/A]	NA
Ca_Desi_Chr_2_14493840	Ca_desi_chr02	14493840	[A/G]	NA
Ca_Desi_Chr_2_14518598	Ca_desi_chr02	14518598	[A/G]	NA
Ca_Desi_Chr_2_14518607	Ca_desi_chr02	14518607	[T/C]	NA
Ca_Desi_Chr_2_14660979	Ca_desi_chr02	14660979	[C/T]	NA
Ca_Desi_Chr_2_14682646	Ca_desi_chr02	14682646	[A/C]	NA
Ca_Desi_Chr_2_14713714	Ca_desi_chr02	14713714	[A/T]	NA
Ca_Desi_Chr_2_14716976	Ca_desi_chr02	14716976	[T/C]	NA
Ca_Desi_Chr_2_14716991	Ca_desi_chr02	14716991	[A/G]	NA
Ca_Desi_Chr_2_14717018	Ca_desi_chr02	14717018	[A/G]	NA
Ca_Desi_Chr_2_14730254	Ca_desi_chr02	14730254	[T/G]	NA
Ca_Desi_Chr_2_14739960	Ca_desi_chr02	14739960	[T/G]	NA
Ca_Desi_Chr_2_14739987	Ca_desi_chr02	14739987	[A/C]	NA
Ca_Desi_Chr_2_14756579	Ca_desi_chr02	14756579	[G/T]	NA
Ca_Desi_Chr_2_14756596	Ca_desi_chr02	14756596	[A/C]	NA
Ca_Desi_Chr_2_14766621	Ca_desi_chr02	14766621	[T/C]	NA
Ca_Desi_Chr_2_14766627	Ca_desi_chr02	14766627	[T/C]	NA
Ca_Desi_Chr_2_14766632	Ca_desi_chr02	14766632	[A/G]	NA
Ca_Desi_Chr_2_14766636	Ca_desi_chr02	14766636	[T/G]	NA
Ca_Desi_Chr_2_14766648	Ca_desi_chr02	14766648	[A/G]	NA
Ca_Desi_Chr_2_14766672	Ca_desi_chr02	14766672	[T/C]	NA
Ca_Desi_Chr_2_14766675	Ca_desi_chr02	14766675	[T/C]	NA
Ca_Desi_Chr_2_14766681	Ca_desi_chr02	14766681	[T/C]	NA
Ca_Desi_Chr_2_14766683	Ca_desi_chr02	14766683	[T/C]	NA
Ca_Desi_Chr_2_14784385	Ca_desi_chr02	14784385	[T/G]	NA
Ca_Desi_Chr_2_14861116	Ca_desi_chr02	14861116	[A/C]	NA
Ca_Desi_Chr_2_14861121	Ca_desi_chr02	14861121	[A/G]	NA
Ca_Desi_Chr_2_14861127	Ca_desi_chr02	14861127	[T/A]	NA
Ca_Desi_Chr_2_14982834	Ca_desi_chr02	14982834	[T/C]	NA
Ca_Desi_Chr_2_15006769	Ca_desi_chr02	15006769	[A/G]	NA
Ca_Desi_Chr_2_15006778	Ca_desi_chr02	15006778	[A/C]	NA

SNP IDs	Chromosomes	Physical positions (bp)	SNPs	NCBI SNPdb accession IDs (NCBI_ss#)
Ca_Desi_Chr_2_15006797	Ca_desi_chr02	15006797	[T/C]	NA
Ca_Desi_Chr_2_15006809	Ca_desi_chr02	15006809	[T/G]	NA
Ca_Desi_Chr_2_15006815	Ca_desi_chr02	15006815	[A/G]	NA
Ca_Desi_Chr_2_15014175	Ca_desi_chr02	15014175	[A/C]	NA
Ca_Desi_Chr_2_15014177	Ca_desi_chr02	15014177	[C/T]	NA
Ca_Desi_Chr_2_15121017	Ca_desi_chr02	15121017	[C/A]	NA
Ca_Desi_Chr_2_15121066	Ca_desi_chr02	15121066	[A/C]	NA
Ca_Desi_Chr_2_15121075	Ca_desi_chr02	15121075	[A/C]	NA
Ca_Desi_Chr_2_15152269	Ca_desi_chr02	15152269	[T/C]	NA
Ca_Desi_Chr_2_15223689	Ca_desi_chr02	15223689	[T/C]	NA
Ca_Desi_Chr_2_15223697	Ca_desi_chr02	15223697	[T/C]	NA
Ca_Desi_Chr_2_15223701	Ca_desi_chr02	15223701	[A/G]	NA
Ca_Desi_Chr_2_15223705	Ca_desi_chr02	15223705	[A/G]	NA
Ca_Desi_Chr_2_15223707	Ca_desi_chr02	15223707	[A/G]	NA
Ca_Desi_Chr_2_15223713	Ca_desi_chr02	15223713	[A/G]	NA
Ca_Desi_Chr_2_15223716	Ca_desi_chr02	15223716	[A/G]	NA
Ca_Desi_Chr_2_15223718	Ca_desi_chr02	15223718	[A/G]	NA
Ca_Desi_Chr_2_15223721	Ca_desi_chr02	15223721	[A/G]	NA
Ca_Desi_Chr_2_15223724	Ca_desi_chr02	15223724	[A/G]	NA
Ca_Desi_Chr_2_15223725	Ca_desi_chr02	15223725	[A/G]	NA
Ca_Desi_Chr_2_15223726	Ca_desi_chr02	15223726	[A/C]	NA
Ca_Desi_Chr_2_15223728	Ca_desi_chr02	15223728	[T/C]	NA
Ca_Desi_Chr_2_15223796	Ca_desi_chr02	15223796	[T/C]	NA
Ca_Desi_Chr_2_15270725	Ca_desi_chr02	15270725	[T/G]	NA
Ca_Desi_Chr_2_15299666	Ca_desi_chr02	15299666	[G/T]	NA
Ca_Desi_Chr_2_15304940	Ca_desi_chr02	15304940	[T/G]	NA
Ca_Desi_Chr_2_15329581	Ca_desi_chr02	15329581	[T/G]	NA
Ca_Desi_Chr_2_15427040	Ca_desi_chr02	15427040	[T/C]	NA
Ca_Desi_Chr_2_15438696	Ca_desi_chr02	15438696	[T/C]	NA
Ca_Desi_Chr_2_15438711	Ca_desi_chr02	15438711	[T/C]	NA
Ca_Desi_Chr_2_15473629	Ca_desi_chr02	15473629	[T/A]	NA
Ca_Desi_Chr_2_15473630	Ca_desi_chr02	15473630	[A/T]	NA
Ca_Desi_Chr_2_15473631	Ca_desi_chr02	15473631	[T/A]	NA
Ca_Desi_Chr_2_15473632	Ca_desi_chr02	15473632	[T/A]	NA

SNP IDs	Chromosomes	Physical positions (bp)	SNPs	NCBI SNPdb accession IDs (NCBI_ss#)
Ca_Desi_Chr_2_15473633	Ca_desi_chr02	15473633	[A/T]	NA
Ca_Desi_Chr_2_15488573	Ca_desi_chr02	15488573	[A/C]	NA
Ca_Desi_Chr_2_15488590	Ca_desi_chr02	15488590	[T/C]	NA
Ca_Desi_Chr_2_15502299	Ca_desi_chr02	15502299	[T/A]	NA
Ca_Desi_Chr_2_15502341	Ca_desi_chr02	15502341	[T/C]	NA
Ca_Desi_Chr_2_15505255	Ca_desi_chr02	15505255	[T/G]	NA
Ca_Desi_Chr_2_15505286	Ca_desi_chr02	15505286	[A/G]	NA
Ca_Desi_Chr_2_15510688	Ca_desi_chr02	15510688	[T/G]	NA
Ca_Desi_Chr_2_15510696	Ca_desi_chr02	15510696	[T/G]	NA
Ca_Desi_Chr_2_15575100	Ca_desi_chr02	15575100	[T/G]	NA
Ca_Desi_Chr_2_15575111	Ca_desi_chr02	15575111	[T/G]	NA
Ca_Desi_Chr_2_15575124	Ca_desi_chr02	15575124	[T/A]	NA
Ca_Desi_Chr_2_15575125	Ca_desi_chr02	15575125	[C/A]	NA
Ca_Desi_Chr_2_15575126	Ca_desi_chr02	15575126	[T/C]	NA
Ca_Desi_Chr_2_15575127	Ca_desi_chr02	15575127	[C/T]	NA
Ca_Desi_Chr_2_15575128	Ca_desi_chr02	15575128	[A/C]	NA
Ca_Desi_Chr_2_15575129	Ca_desi_chr02	15575129	[G/T]	NA
Ca_Desi_Chr_2_15575132	Ca_desi_chr02	15575132	[A/G]	NA
Ca_Desi_Chr_2_15652498	Ca_desi_chr02	15652498	[T/G]	NA
Ca_Desi_Chr_2_15653200	Ca_desi_chr02	15653200	[A/C]	NA
Ca_Desi_Chr_2_15656107	Ca_desi_chr02	15656107	[T/C]	NA
Ca_Desi_Chr_2_15677613	Ca_desi_chr02	15677613	[T/C]	NA
Ca_Desi_Chr_2_15677616	Ca_desi_chr02	15677616	[T/C]	NA
Ca_Desi_Chr_2_15677632	Ca_desi_chr02	15677632	[A/G]	NA
Ca_Desi_Chr_2_15677646	Ca_desi_chr02	15677646	[A/G]	NA
Ca_Desi_Chr_2_15677650	Ca_desi_chr02	15677650	[T/G]	NA
Ca_Desi_Chr_2_15822308	Ca_desi_chr02	15822308	[A/G]	NA
Ca_Desi_Chr_2_15822318	Ca_desi_chr02	15822318	[A/G]	NA
Ca_Desi_Chr_2_15822346	Ca_desi_chr02	15822346	[T/G]	NA
Ca_Desi_Chr_2_15876643	Ca_desi_chr02	15876643	[A/T]	NA
Ca_Desi_Chr_2_15878389	Ca_desi_chr02	15878389	[T/C]	NA
Ca_Desi_Chr_2_15878476	Ca_desi_chr02	15878476	[A/T]	NA
Ca_Desi_Chr_2_15878496	Ca_desi_chr02	15878496	[T/G]	NA
Ca_Desi_Chr_2_15905877	Ca_desi_chr02	15905877	[A/C]	NA

SNP IDs	Chromosomes	Physical positions (bp)	SNPs	NCBI SNPdb accession IDs (NCBI_ss#)
Ca_Desi_Chr_2_15905883	Ca_desi_chr02	15905883	[T/C]	NA
Ca_Desi_Chr_2_15905898	Ca_desi_chr02	15905898	[T/C]	NA
Ca_Desi_Chr_2_15905916	Ca_desi_chr02	15905916	[T/C]	NA
Ca_Desi_Chr_2_15905919	Ca_desi_chr02	15905919	[T/G]	NA
Ca_Desi_Chr_2_15905925	Ca_desi_chr02	15905925	[A/C]	NA
Ca_Desi_Chr_2_15905931	Ca_desi_chr02	15905931	[T/C]	NA
Ca_Desi_Chr_2_15905934	Ca_desi_chr02	15905934	[T/C]	NA
Ca_Desi_Chr_2_15926923	Ca_desi_chr02	15926923	[C/T]	NA
Ca_Desi_Chr_2_15937203	Ca_desi_chr02	15937203	[T/C]	NA
Ca_Desi_Chr_2_15937209	Ca_desi_chr02	15937209	[T/C]	NA
Ca_Desi_Chr_2_15937214	Ca_desi_chr02	15937214	[G/C]	NA
Ca_Desi_Chr_2_15937217	Ca_desi_chr02	15937217	[T/C]	NA
Ca_Desi_Chr_2_15937221	Ca_desi_chr02	15937221	[T/C]	NA
Ca_Desi_Chr_2_15937224	Ca_desi_chr02	15937224	[T/C]	NA
Ca_Desi_Chr_2_15937226	Ca_desi_chr02	15937226	[G/C]	NA
Ca_Desi_Chr_2_15937229	Ca_desi_chr02	15937229	[A/C]	NA
Ca_Desi_Chr_2_15937231	Ca_desi_chr02	15937231	[A/G]	NA
Ca_Desi_Chr_2_15937232	Ca_desi_chr02	15937232	[G/A]	NA
Ca_Desi_Chr_2_15937233	Ca_desi_chr02	15937233	[A/G]	NA
Ca_Desi_Chr_2_15937238	Ca_desi_chr02	15937238	[G/C]	NA
Ca_Desi_Chr_2_15937243	Ca_desi_chr02	15937243	[T/G]	NA
Ca_Desi_Chr_2_15937247	Ca_desi_chr02	15937247	[T/C]	NA
Ca_Desi_Chr_2_15937249	Ca_desi_chr02	15937249	[A/C]	NA
Ca_Desi_Chr_2_15937258	Ca_desi_chr02	15937258	[C/G]	NA
Ca_Desi_Chr_2_15951042	Ca_desi_chr02	15951042	[T/G]	NA
Ca_Desi_Chr_2_15951050	Ca_desi_chr02	15951050	[T/G]	NA
Ca_Desi_Chr_2_15951058	Ca_desi_chr02	15951058	[A/G]	NA
Ca_Desi_Chr_2_15951060	Ca_desi_chr02	15951060	[A/G]	NA
Ca_Desi_Chr_2_15951065	Ca_desi_chr02	15951065	[G/A]	NA
Ca_Desi_Chr_2_15951067	Ca_desi_chr02	15951067	[G/A]	NA
Ca_Desi_Chr_2_15951069	Ca_desi_chr02	15951069	[A/G]	NA
Ca_Desi_Chr_2_15951070	Ca_desi_chr02	15951070	[G/A]	NA
Ca_Desi_Chr_2_15951072	Ca_desi_chr02	15951072	[A/G]	NA
Ca_Desi_Chr_2_15951073	Ca_desi_chr02	15951073	[G/A]	NA

SNP IDs	Chromosomes	Physical positions (bp)	SNPs	NCBI SNPdb accession IDs (NCBI_ss#)
Ca_Desi_Chr_2_15951077	Ca_desi_chr02	15951077	[T/G]	NA
Ca_Desi_Chr_2_15951086	Ca_desi_chr02	15951086	[T/G]	NA
Ca_Desi_Chr_2_15951090	Ca_desi_chr02	15951090	[G/A]	NA
Ca_Desi_Chr_2_15951105	Ca_desi_chr02	15951105	[A/C]	NA
Ca_Desi_Chr_2_15959845	Ca_desi_chr02	15959845	[T/A]	NA
Ca_Desi_Chr_2_15963642	Ca_desi_chr02	15963642	[T/G]	NA
Ca_Desi_Chr_2_15970064	Ca_desi_chr02	15970064	[T/G]	NA
Ca_Desi_Chr_2_15970101	Ca_desi_chr02	15970101	[A/G]	NA
Ca_Desi_Chr_2_16114811	Ca_desi_chr02	16114811	[A/T]	NA
Ca_Desi_Chr_2_16136668	Ca_desi_chr02	16136668	[C/T]	NA
Ca_Desi_Chr_2_16138961	Ca_desi_chr02	16138961	[A/G]	NA
Ca_Desi_Chr_2_16138966	Ca_desi_chr02	16138966	[C/A]	NA
Ca_Desi_Chr_2_16139069	Ca_desi_chr02	16139069	[T/G]	NA
Ca_Desi_Chr_2_16216850	Ca_desi_chr02	16216850	[A/C]	NA
Ca_Desi_Chr_2_16216857	Ca_desi_chr02	16216857	[C/T]	NA
Ca_Desi_Chr_2_16216861	Ca_desi_chr02	16216861	[A/C]	NA
Ca_Desi_Chr_2_16676201	Ca_desi_chr02	16676201	[C/A]	NA
Ca_Desi_Chr_2_16683365	Ca_desi_chr02	16683365	[G/C]	NA
Ca_Desi_Chr_2_16706522	Ca_desi_chr02	16706522	[T/C]	NA
Ca_Desi_Chr_2_16714762	Ca_desi_chr02	16714762	[A/G]	NA
Ca_Desi_Chr_2_16716541	Ca_desi_chr02	16716541	[T/G]	NA
Ca_Desi_Chr_2_16741187	Ca_desi_chr02	16741187	[C/T]	NA
Ca_Desi_Chr_2_16741261	Ca_desi_chr02	16741261	[T/C]	NA
Ca_Desi_Chr_2_16741272	Ca_desi_chr02	16741272	[A/C]	NA
Ca_Desi_Chr_2_16741302	Ca_desi_chr02	16741302	[C/T]	NA
Ca_Desi_Chr_2_16748554	Ca_desi_chr02	16748554	[G/T]	NA
Ca_Desi_Chr_2_16748556	Ca_desi_chr02	16748556	[T/G]	NA
Ca_Desi_Chr_2_16748679	Ca_desi_chr02	16748679	[T/A]	NA
Ca_Desi_Chr_2_16748700	Ca_desi_chr02	16748700	[G/A]	NA
Ca_Desi_Chr_2_16748726	Ca_desi_chr02	16748726	[T/A]	NA
Ca_Desi_Chr_2_16748779	Ca_desi_chr02	16748779	[T/A]	NA
Ca_Desi_Chr_2_16753289	Ca_desi_chr02	16753289	[T/C]	NA
Ca_Desi_Chr_2_16757057	Ca_desi_chr02	16757057	[T/G]	NA
Ca_Desi_Chr_2_16757086	Ca_desi_chr02	16757086	[T/C]	NA

SNP IDs	Chromosomes	Physical positions (bp)	SNPs	NCBI SNPdb accession IDs (NCBI_ss#)
Ca_Desi_Chr_2_16757108	Ca_desi_chr02	16757108	[A/G]	NA
Ca_Desi_Chr_2_16838408	Ca_desi_chr02	16838408	[T/G]	NA
Ca_Desi_Chr_2_16838430	Ca_desi_chr02	16838430	[T/C]	NA
Ca_Desi_Chr_2_16838436	Ca_desi_chr02	16838436	[T/C]	NA
Ca_Desi_Chr_2_16856375	Ca_desi_chr02	16856375	[C/T]	NA
Ca_Desi_Chr_2_16856387	Ca_desi_chr02	16856387	[T/A]	NA
Ca_Desi_Chr_2_16856404	Ca_desi_chr02	16856404	[T/C]	NA
Ca_Desi_Chr_2_16856406	Ca_desi_chr02	16856406	[G/C]	NA
Ca_Desi_Chr_2_16856409	Ca_desi_chr02	16856409	[T/G]	NA
Ca_Desi_Chr_2_16954879	Ca_desi_chr02	16954879	[A/G]	NA
Ca_Desi_Chr_2_16992629	Ca_desi_chr02	16992629	[A/G]	NA
Ca_Desi_Chr_2_16992640	Ca_desi_chr02	16992640	[T/G]	NA
Ca_Desi_Chr_2_16992666	Ca_desi_chr02	16992666	[T/C]	NA
Ca_Desi_Chr_2_16992676	Ca_desi_chr02	16992676	[T/C]	NA
Ca_Desi_Chr_2_16992678	Ca_desi_chr02	16992678	[T/C]	NA
Ca_Desi_Chr_2_17048232	Ca_desi_chr02	17048232	[G/T]	NA
Ca_Desi_Chr_2_17048276	Ca_desi_chr02	17048276	[T/A]	NA
Ca_Desi_Chr_2_17048306	Ca_desi_chr02	17048306	[A/G]	NA
Ca_Desi_Chr_2_17048324	Ca_desi_chr02	17048324	[A/G]	NA
Ca_Desi_Chr_2_17187204	Ca_desi_chr02	17187204	[A/G]	NA
Ca_Desi_Chr_2_17187220	Ca_desi_chr02	17187220	[T/C]	NA
Ca_Desi_Chr_2_17187228	Ca_desi_chr02	17187228	[A/G]	NA
Ca_Desi_Chr_2_17187244	Ca_desi_chr02	17187244	[T/A]	NA
Ca_Desi_Chr_2_17230663	Ca_desi_chr02	17230663	[A/G]	NA
Ca_Desi_Chr_3_126478	Ca_desi_chr03	126478	[A/G]	NA
Ca_Desi_Chr_3_126485	Ca_desi_chr03	126485	[T/G]	NA
Ca_Desi_Chr_3_126490	Ca_desi_chr03	126490	[T/A]	NA
Ca_Desi_Chr_3_126616	Ca_desi_chr03	126616	[G/A]	NA
Ca_Desi_Chr_3_126619	Ca_desi_chr03	126619	[G/A]	NA
Ca_Desi_Chr_3_234208	Ca_desi_chr03	234208	[A/T]	NA
Ca_Desi_Chr_3_234219	Ca_desi_chr03	234219	[A/T]	NA
Ca_Desi_Chr_3_347881	Ca_desi_chr03	347881	[T/G]	NA
Ca_Desi_Chr_3_348960	Ca_desi_chr03	348960	[G/T]	NA
Ca_Desi_Chr_3_644787	Ca_desi_chr03	644787	[G/A]	NA

SNP IDs	Chromosomes	Physical positions (bp)	SNPs	NCBI SNPdb accession IDs (NCBI_ss#)
Ca_Desi_Chr_3_644790	Ca_desi_chr03	644790	[C/A]	NA
Ca_Desi_Chr_3_644806	Ca_desi_chr03	644806	[C/A]	NA
Ca_Desi_Chr_3_644808	Ca_desi_chr03	644808	[C/T]	NA
Ca_Desi_Chr_3_644812	Ca_desi_chr03	644812	[G/T]	NA
Ca_Desi_Chr_3_645024	Ca_desi_chr03	645024	[A/C]	NA
Ca_Desi_Chr_3_668396	Ca_desi_chr03	668396	[A/G]	NA
Ca_Desi_Chr_3_669328	Ca_desi_chr03	669328	[A/T]	NA
Ca_Desi_Chr_3_684390	Ca_desi_chr03	684390	[A/G]	NA
Ca_Desi_Chr_3_691462	Ca_desi_chr03	691462	[A/G]	NA
Ca_Desi_Chr_3_691479	Ca_desi_chr03	691479	[T/A]	NA
Ca_Desi_Chr_3_691509	Ca_desi_chr03	691509	[A/C]	NA
Ca_Desi_Chr_3_691519	Ca_desi_chr03	691519	[T/G]	NA
Ca_Desi_Chr_3_697924	Ca_desi_chr03	697924	[T/G]	NA
Ca_Desi_Chr_3_698163	Ca_desi_chr03	698163	[T/A]	NA
Ca_Desi_Chr_3_698170	Ca_desi_chr03	698170	[T/A]	NA
Ca_Desi_Chr_3_762890	Ca_desi_chr03	762890	[A/C]	NA
Ca_Desi_Chr_3_764477	Ca_desi_chr03	764477	[A/C]	NA
Ca_Desi_Chr_3_764593	Ca_desi_chr03	764593	[A/C]	NA
Ca_Desi_Chr_3_782913	Ca_desi_chr03	782913	[T/G]	NA
Ca_Desi_Chr_3_783054	Ca_desi_chr03	783054	[T/G]	NA
Ca_Desi_Chr_3_783093	Ca_desi_chr03	783093	[A/C]	NA
Ca_Desi_Chr_3_783107	Ca_desi_chr03	783107	[A/G]	NA
Ca_Desi_Chr_3_839877	Ca_desi_chr03	839877	[T/G]	NA
Ca_Desi_Chr_3_841817	Ca_desi_chr03	841817	[C/A]	NA
Ca_Desi_Chr_3_841818	Ca_desi_chr03	841818	[A/G]	NA
Ca_Desi_Chr_3_841819	Ca_desi_chr03	841819	[T/A]	NA
Ca_Desi_Chr_3_854698	Ca_desi_chr03	854698	[C/T]	NA
Ca_Desi_Chr_3_867821	Ca_desi_chr03	867821	[A/G]	NA
Ca_Desi_Chr_3_869249	Ca_desi_chr03	869249	[C/T]	NA
Ca_Desi_Chr_3_870904	Ca_desi_chr03	870904	[T/C]	NA
Ca_Desi_Chr_3_930269	Ca_desi_chr03	930269	[T/G]	NA
Ca_Desi_Chr_3_987408	Ca_desi_chr03	987408	[A/G]	NA
Ca_Desi_Chr_3_987412	Ca_desi_chr03	987412	[T/G]	NA
Ca_Desi_Chr_3_987450	Ca_desi_chr03	987450	[A/G]	NA

SNP IDs	Chromosomes	Physical positions (bp)	SNPs	NCBI SNPdb accession IDs (NCBI_ss#)
Ca_Desi_Chr_3_987453	Ca_desi_chr03	987453	[T/G]	NA
Ca_Desi_Chr_3_993124	Ca_desi_chr03	993124	[A/C]	NA
Ca_Desi_Chr_3_993125	Ca_desi_chr03	993125	[C/A]	NA
Ca_Desi_Chr_3_993126	Ca_desi_chr03	993126	[A/G]	NA
Ca_Desi_Chr_3_993127	Ca_desi_chr03	993127	[G/C]	NA
Ca_Desi_Chr_3_995109	Ca_desi_chr03	995109	[A/C]	NA
Ca_Desi_Chr_3_995118	Ca_desi_chr03	995118	[T/C]	NA
Ca_Desi_Chr_3_1000504	Ca_desi_chr03	1000504	[T/C]	NA
Ca_Desi_Chr_3_1006248	Ca_desi_chr03	1006248	[T/C]	NA
Ca_Desi_Chr_3_1006273	Ca_desi_chr03	1006273	[A/G]	NA
Ca_Desi_Chr_3_1006284	Ca_desi_chr03	1006284	[A/T]	NA
Ca_Desi_Chr_3_1011960	Ca_desi_chr03	1011960	[T/C]	NA
Ca_Desi_Chr_3_1039492	Ca_desi_chr03	1039492	[G/T]	NA
Ca_Desi_Chr_3_1040662	Ca_desi_chr03	1040662	[A/C]	NA
Ca_Desi_Chr_3_1040685	Ca_desi_chr03	1040685	[G/T]	NA
Ca_Desi_Chr_3_1040786	Ca_desi_chr03	1040786	[T/A]	NA
Ca_Desi_Chr_3_1125469	Ca_desi_chr03	1125469	[A/G]	NA
Ca_Desi_Chr_3_1125503	Ca_desi_chr03	1125503	[T/A]	NA
Ca_Desi_Chr_3_1142171	Ca_desi_chr03	1142171	[T/C]	NA
Ca_Desi_Chr_3_1142311	Ca_desi_chr03	1142311	[T/C]	NA
Ca_Desi_Chr_3_1142320	Ca_desi_chr03	1142320	[T/G]	NA
Ca_Desi_Chr_3_1147159	Ca_desi_chr03	1147159	[A/T]	NA
Ca_Desi_Chr_3_1147168	Ca_desi_chr03	1147168	[A/G]	NA
Ca_Desi_Chr_3_1147179	Ca_desi_chr03	1147179	[A/G]	NA
Ca_Desi_Chr_3_1147185	Ca_desi_chr03	1147185	[T/C]	NA
Ca_Desi_Chr_3_1147188	Ca_desi_chr03	1147188	[A/G]	NA
Ca_Desi_Chr_3_1147191	Ca_desi_chr03	1147191	[A/G]	NA
Ca_Desi_Chr_3_1147194	Ca_desi_chr03	1147194	[T/C]	NA
Ca_Desi_Chr_3_1147210	Ca_desi_chr03	1147210	[T/C]	NA
Ca_Desi_Chr_3_1147213	Ca_desi_chr03	1147213	[A/C]	NA
Ca_Desi_Chr_3_1147216	Ca_desi_chr03	1147216	[T/C]	NA
Ca_Desi_Chr_3_1147219	Ca_desi_chr03	1147219	[T/C]	NA
Ca_Desi_Chr_3_1298323	Ca_desi_chr03	1298323	[A/C]	NA
Ca_Desi_Chr_3_1298325	Ca_desi_chr03	1298325	[G/A]	NA

SNP IDs	Chromosomes	Physical positions (bp)	SNPs	NCBI SNPdb accession IDs (NCBI_ss#)
Ca_Desi_Chr_3_1298346	Ca_desi_chr03	1298346	[A/G]	NA
Ca_Desi_Chr_3_1298497	Ca_desi_chr03	1298497	[C/T]	NA
Ca_Desi_Chr_3_1298524	Ca_desi_chr03	1298524	[G/A]	NA
Ca_Desi_Chr_3_1322765	Ca_desi_chr03	1322765	[T/G]	NA
Ca_Desi_Chr_3_1351568	Ca_desi_chr03	1351568	[C/A]	NA
Ca_Desi_Chr_3_1351569	Ca_desi_chr03	1351569	[C/G]	NA
Ca_Desi_Chr_3_1351570	Ca_desi_chr03	1351570	[T/A]	NA
Ca_Desi_Chr_3_1351571	Ca_desi_chr03	1351571	[A/T]	NA
Ca_Desi_Chr_3_1404591	Ca_desi_chr03	1404591	[T/G]	NA
Ca_Desi_Chr_3_1404643	Ca_desi_chr03	1404643	[A/G]	NA
Ca_Desi_Chr_3_1415294	Ca_desi_chr03	1415294	[T/G]	NA
Ca_Desi_Chr_3_1415304	Ca_desi_chr03	1415304	[T/G]	NA
Ca_Desi_Chr_3_1416895	Ca_desi_chr03	1416895	[A/T]	NA
Ca_Desi_Chr_3_1416904	Ca_desi_chr03	1416904	[A/T]	NA
Ca_Desi_Chr_3_1416907	Ca_desi_chr03	1416907	[T/C]	NA
Ca_Desi_Chr_3_1416913	Ca_desi_chr03	1416913	[A/G]	NA
Ca_Desi_Chr_3_1416922	Ca_desi_chr03	1416922	[A/G]	NA
Ca_Desi_Chr_3_1416925	Ca_desi_chr03	1416925	[C/T]	NA
Ca_Desi_Chr_3_1416929	Ca_desi_chr03	1416929	[A/G]	NA
Ca_Desi_Chr_3_1416934	Ca_desi_chr03	1416934	[T/C]	NA
Ca_Desi_Chr_3_1416939	Ca_desi_chr03	1416939	[T/C]	NA
Ca_Desi_Chr_3_1416940	Ca_desi_chr03	1416940	[G/A]	NA
Ca_Desi_Chr_3_1416942	Ca_desi_chr03	1416942	[T/A]	NA
Ca_Desi_Chr_3_1416943	Ca_desi_chr03	1416943	[A/G]	NA
Ca_Desi_Chr_3_1416947	Ca_desi_chr03	1416947	[T/C]	NA
Ca_Desi_Chr_3_1416949	Ca_desi_chr03	1416949	[A/G]	NA
Ca_Desi_Chr_3_1416951	Ca_desi_chr03	1416951	[T/C]	NA
Ca_Desi_Chr_3_1416960	Ca_desi_chr03	1416960	[T/C]	NA
Ca_Desi_Chr_3_1417037	Ca_desi_chr03	1417037	[A/T]	NA
Ca_Desi_Chr_3_1427049	Ca_desi_chr03	1427049	[T/G]	NA
Ca_Desi_Chr_3_1463002	Ca_desi_chr03	1463002	[A/G]	NA
Ca_Desi_Chr_3_1471389	Ca_desi_chr03	1471389	[T/G]	NA
Ca_Desi_Chr_3_1471416	Ca_desi_chr03	1471416	[A/G]	NA
Ca_Desi_Chr_3_1471436	Ca_desi_chr03	1471436	[A/G]	NA

SNP IDs	Chromosomes	Physical positions (bp)	SNPs	NCBI SNPdb accession IDs (NCBI_ss#)
Ca_Desi_Chr_3_1532210	Ca_desi_chr03	1532210	[T/G]	NA
Ca_Desi_Chr_3_1532213	Ca_desi_chr03	1532213	[T/G]	NA
Ca_Desi_Chr_3_1532309	Ca_desi_chr03	1532309	[A/C]	NA
Ca_Desi_Chr_3_1533752	Ca_desi_chr03	1533752	[T/C]	NA
Ca_Desi_Chr_3_1533802	Ca_desi_chr03	1533802	[T/C]	NA
Ca_Desi_Chr_3_1533815	Ca_desi_chr03	1533815	[A/C]	NA
Ca_Desi_Chr_3_1533817	Ca_desi_chr03	1533817	[T/C]	NA
Ca_Desi_Chr_3_1533828	Ca_desi_chr03	1533828	[A/G]	NA
Ca_Desi_Chr_3_1533889	Ca_desi_chr03	1533889	[T/A]	NA
Ca_Desi_Chr_3_1533903	Ca_desi_chr03	1533903	[T/G]	NA
Ca_Desi_Chr_3_1536599	Ca_desi_chr03	1536599	[T/C]	NA
Ca_Desi_Chr_3_1536637	Ca_desi_chr03	1536637	[A/G]	NA
Ca_Desi_Chr_3_1536728	Ca_desi_chr03	1536728	[T/C]	NA
Ca_Desi_Chr_3_1536741	Ca_desi_chr03	1536741	[T/G]	NA
Ca_Desi_Chr_3_1554451	Ca_desi_chr03	1554451	[T/C]	NA
Ca_Desi_Chr_3_1619028	Ca_desi_chr03	1619028	[T/A]	NA
Ca_Desi_Chr_3_1633019	Ca_desi_chr03	1633019	[T/C]	NA
Ca_Desi_Chr_3_1633071	Ca_desi_chr03	1633071	[T/C]	NA
Ca_Desi_Chr_3_1642870	Ca_desi_chr03	1642870	[A/C]	NA
Ca_Desi_Chr_3_1666335	Ca_desi_chr03	1666335	[T/C]	NA
Ca_Desi_Chr_3_1666355	Ca_desi_chr03	1666355	[T/C]	NA
Ca_Desi_Chr_3_1667608	Ca_desi_chr03	1667608	[A/C]	NA
Ca_Desi_Chr_3_1686916	Ca_desi_chr03	1686916	[T/G]	NA
Ca_Desi_Chr_3_1687065	Ca_desi_chr03	1687065	[T/G]	NA
Ca_Desi_Chr_3_1690793	Ca_desi_chr03	1690793	[A/C]	NA
Ca_Desi_Chr_3_1690825	Ca_desi_chr03	1690825	[T/C]	NA
Ca_Desi_Chr_3_1701505	Ca_desi_chr03	1701505	[T/G]	NA
Ca_Desi_Chr_3_1701592	Ca_desi_chr03	1701592	[A/G]	NA
Ca_Desi_Chr_3_1705565	Ca_desi_chr03	1705565	[T/G]	NA
Ca_Desi_Chr_3_1922630	Ca_desi_chr03	1922630	[A/C]	NA
Ca_Desi_Chr_3_1923782	Ca_desi_chr03	1923782	[A/G]	NA
Ca_Desi_Chr_3_1923788	Ca_desi_chr03	1923788	[T/C]	NA
Ca_Desi_Chr_3_1923792	Ca_desi_chr03	1923792	[A/G]	NA
Ca_Desi_Chr_3_1923795	Ca_desi_chr03	1923795	[T/C]	NA

SNP IDs	Chromosomes	Physical positions (bp)	SNPs	NCBI SNPdb accession IDs (NCBI_ss#)
Ca_Desi_Chr_3_1923802	Ca_desi_chr03	1923802	[A/C]	NA
Ca_Desi_Chr_3_1923804	Ca_desi_chr03	1923804	[A/G]	NA
Ca_Desi_Chr_3_1923832	Ca_desi_chr03	1923832	[T/C]	NA
Ca_Desi_Chr_3_1923842	Ca_desi_chr03	1923842	[T/C]	NA
Ca_Desi_Chr_3_2672487	Ca_desi_chr03	2672487	[A/C]	NA
Ca_Desi_Chr_3_2672507	Ca_desi_chr03	2672507	[T/C]	NA
Ca_Desi_Chr_3_2672511	Ca_desi_chr03	2672511	[T/C]	NA
Ca_Desi_Chr_3_2674392	Ca_desi_chr03	2674392	[A/G]	NA
Ca_Desi_Chr_3_2674393	Ca_desi_chr03	2674393	[G/A]	NA
Ca_Desi_Chr_3_2679441	Ca_desi_chr03	2679441	[A/C]	NA
Ca_Desi_Chr_3_2679446	Ca_desi_chr03	2679446	[T/C]	NA
Ca_Desi_Chr_3_2679455	Ca_desi_chr03	2679455	[A/G]	NA
Ca_Desi_Chr_3_2679464	Ca_desi_chr03	2679464	[T/C]	NA
Ca_Desi_Chr_3_2679467	Ca_desi_chr03	2679467	[T/C]	NA
Ca_Desi_Chr_3_2679475	Ca_desi_chr03	2679475	[T/G]	NA
Ca_Desi_Chr_3_2693543	Ca_desi_chr03	2693543	[T/G]	NA
Ca_Desi_Chr_3_2698420	Ca_desi_chr03	2698420	[T/C]	NA
Ca_Desi_Chr_3_2698433	Ca_desi_chr03	2698433	[A/G]	NA
Ca_Desi_Chr_3_2700887	Ca_desi_chr03	2700887	[T/C]	NA
Ca_Desi_Chr_3_2716534	Ca_desi_chr03	2716534	[T/G]	NA
Ca_Desi_Chr_3_2716539	Ca_desi_chr03	2716539	[T/G]	NA
Ca_Desi_Chr_3_2725582	Ca_desi_chr03	2725582	[T/G]	NA
Ca_Desi_Chr_3_2725700	Ca_desi_chr03	2725700	[A/C]	NA
Ca_Desi_Chr_3_2725715	Ca_desi_chr03	2725715	[T/G]	NA
Ca_Desi_Chr_3_2729419	Ca_desi_chr03	2729419	[T/C]	NA
Ca_Desi_Chr_3_2729454	Ca_desi_chr03	2729454	[A/G]	NA
Ca_Desi_Chr_3_2729462	Ca_desi_chr03	2729462	[T/A]	NA
Ca_Desi_Chr_3_2729464	Ca_desi_chr03	2729464	[T/A]	NA
Ca_Desi_Chr_3_2758003	Ca_desi_chr03	2758003	[C/A]	NA
Ca_Desi_Chr_3_2758011	Ca_desi_chr03	2758011	[T/G]	NA
Ca_Desi_Chr_3_2787341	Ca_desi_chr03	2787341	[A/G]	NA
Ca_Desi_Chr_3_2787348	Ca_desi_chr03	2787348	[T/C]	NA
Ca_Desi_Chr_3_2787364	Ca_desi_chr03	2787364	[A/G]	NA
Ca_Desi_Chr_3_2787382	Ca_desi_chr03	2787382	[A/G]	NA

SNP IDs	Chromosomes	Physical positions (bp)	SNPs	NCBI SNPdb accession IDs (NCBI_ss#)
Ca_Desi_Chr_3_2787384	Ca_desi_chr03	2787384	[A/G]	NA
Ca_Desi_Chr_3_2787386	Ca_desi_chr03	2787386	[A/G]	NA
Ca_Desi_Chr_3_2787402	Ca_desi_chr03	2787402	[T/C]	NA
Ca_Desi_Chr_3_2787477	Ca_desi_chr03	2787477	[T/G]	NA
Ca_Desi_Chr_3_2791740	Ca_desi_chr03	2791740	[T/C]	NA
Ca_Desi_Chr_3_2796304	Ca_desi_chr03	2796304	[A/G]	NA
Ca_Desi_Chr_3_2803734	Ca_desi_chr03	2803734	[T/C]	NA
Ca_Desi_Chr_3_2803750	Ca_desi_chr03	2803750	[A/G]	NA
Ca_Desi_Chr_3_2803764	Ca_desi_chr03	2803764	[A/C]	NA
Ca_Desi_Chr_3_2803768	Ca_desi_chr03	2803768	[T/G]	NA
Ca_Desi_Chr_3_2812004	Ca_desi_chr03	2812004	[A/G]	NA
Ca_Desi_Chr_3_2842049	Ca_desi_chr03	2842049	[T/A]	NA
Ca_Desi_Chr_3_2915510	Ca_desi_chr03	2915510	[C/T]	NA
Ca_Desi_Chr_3_2920357	Ca_desi_chr03	2920357	[A/T]	NA
Ca_Desi_Chr_3_2920360	Ca_desi_chr03	2920360	[C/A]	NA
Ca_Desi_Chr_3_2920363	Ca_desi_chr03	2920363	[A/C]	NA
Ca_Desi_Chr_3_2920376	Ca_desi_chr03	2920376	[G/A]	NA
Ca_Desi_Chr_3_2940815	Ca_desi_chr03	2940815	[A/C]	NA
Ca_Desi_Chr_3_2940824	Ca_desi_chr03	2940824	[A/G]	NA
Ca_Desi_Chr_3_2952449	Ca_desi_chr03	2952449	[T/A]	NA
Ca_Desi_Chr_3_2952484	Ca_desi_chr03	2952484	[G/A]	NA
Ca_Desi_Chr_3_2952498	Ca_desi_chr03	2952498	[A/T]	NA
Ca_Desi_Chr_3_2953042	Ca_desi_chr03	2953042	[T/C]	NA
Ca_Desi_Chr_3_2953045	Ca_desi_chr03	2953045	[A/C]	NA
Ca_Desi_Chr_3_2972597	Ca_desi_chr03	2972597	[A/G]	NA
Ca_Desi_Chr_3_3002455	Ca_desi_chr03	3002455	[A/C]	NA
Ca_Desi_Chr_3_3112966	Ca_desi_chr03	3112966	[A/G]	NA
Ca_Desi_Chr_3_3130747	Ca_desi_chr03	3130747	[A/T]	NA
Ca_Desi_Chr_3_3130769	Ca_desi_chr03	3130769	[A/C]	NA
Ca_Desi_Chr_3_3130786	Ca_desi_chr03	3130786	[T/G]	NA
Ca_Desi_Chr_3_3187922	Ca_desi_chr03	3187922	[T/C]	NA
Ca_Desi_Chr_3_3209764	Ca_desi_chr03	3209764	[T/C]	NA
Ca_Desi_Chr_3_3209766	Ca_desi_chr03	3209766	[A/G]	NA
Ca_Desi_Chr_3_3212024	Ca_desi_chr03	3212024	[T/G]	NA

SNP IDs	Chromosomes	Physical positions (bp)	SNPs	NCBI SNPdb accession IDs (NCBI_ss#)
Ca_Desi_Chr_3_3212040	Ca_desi_chr03	3212040	[A/G]	NA
Ca_Desi_Chr_3_3224426	Ca_desi_chr03	3224426	[A/G]	NA
Ca_Desi_Chr_3_3224434	Ca_desi_chr03	3224434	[A/T]	NA
Ca_Desi_Chr_3_3224437	Ca_desi_chr03	3224437	[A/C]	NA
Ca_Desi_Chr_3_3224442	Ca_desi_chr03	3224442	[T/G]	NA
Ca_Desi_Chr_3_3224451	Ca_desi_chr03	3224451	[T/C]	NA
Ca_Desi_Chr_3_3224452	Ca_desi_chr03	3224452	[C/T]	NA
Ca_Desi_Chr_3_3224453	Ca_desi_chr03	3224453	[T/C]	NA
Ca_Desi_Chr_3_3224457	Ca_desi_chr03	3224457	[C/T]	NA
Ca_Desi_Chr_3_3224459	Ca_desi_chr03	3224459	[T/C]	NA
Ca_Desi_Chr_3_3224466	Ca_desi_chr03	3224466	[T/G]	NA
Ca_Desi_Chr_3_3224480	Ca_desi_chr03	3224480	[T/C]	NA
Ca_Desi_Chr_3_3255048	Ca_desi_chr03	3255048	[T/G]	NA
Ca_Desi_Chr_3_3259228	Ca_desi_chr03	3259228	[A/G]	NA
Ca_Desi_Chr_3_3259256	Ca_desi_chr03	3259256	[C/T]	NA
Ca_Desi_Chr_3_3259263	Ca_desi_chr03	3259263	[A/G]	NA
Ca_Desi_Chr_3_3259274	Ca_desi_chr03	3259274	[A/G]	NA
Ca_Desi_Chr_3_3259276	Ca_desi_chr03	3259276	[T/C]	NA
Ca_Desi_Chr_3_3267977	Ca_desi_chr03	3267977	[T/G]	NA
Ca_Desi_Chr_3_3270139	Ca_desi_chr03	3270139	[T/C]	NA
Ca_Desi_Chr_3_3296028	Ca_desi_chr03	3296028	[A/G]	NA
Ca_Desi_Chr_3_3296055	Ca_desi_chr03	3296055	[T/G]	NA
Ca_Desi_Chr_3_3296064	Ca_desi_chr03	3296064	[A/G]	NA
Ca_Desi_Chr_3_3318519	Ca_desi_chr03	3318519	[T/C]	NA
Ca_Desi_Chr_3_3339758	Ca_desi_chr03	3339758	[T/G]	NA
Ca_Desi_Chr_3_3404161	Ca_desi_chr03	3404161	[T/G]	NA
Ca_Desi_Chr_3_3427232	Ca_desi_chr03	3427232	[T/C]	NA
Ca_Desi_Chr_3_3427257	Ca_desi_chr03	3427257	[T/C]	NA
Ca_Desi_Chr_3_3427275	Ca_desi_chr03	3427275	[T/C]	NA
Ca_Desi_Chr_3_3478951	Ca_desi_chr03	3478951	[T/C]	NA
Ca_Desi_Chr_3_3493826	Ca_desi_chr03	3493826	[C/T]	NA
Ca_Desi_Chr_3_3493840	Ca_desi_chr03	3493840	[G/C]	NA
Ca_Desi_Chr_3_3495485	Ca_desi_chr03	3495485	[A/C]	NA
Ca_Desi_Chr_3_3495509	Ca_desi_chr03	3495509	[T/C]	NA

SNP IDs	Chromosomes	Physical positions (bp)	SNPs	NCBI SNPdb accession IDs (NCBI_ss#)
Ca_Desi_Chr_3_3495521	Ca_desi_chr03	3495521	[A/C]	NA
Ca_Desi_Chr_3_3495585	Ca_desi_chr03	3495585	[A/C]	NA
Ca_Desi_Chr_3_3495610	Ca_desi_chr03	3495610	[A/C]	NA
Ca_Desi_Chr_3_3500165	Ca_desi_chr03	3500165	[T/A]	NA
Ca_Desi_Chr_3_3504436	Ca_desi_chr03	3504436	[A/G]	NA
Ca_Desi_Chr_3_3509590	Ca_desi_chr03	3509590	[C/G]	NA
Ca_Desi_Chr_3_3509602	Ca_desi_chr03	3509602	[G/T]	NA
Ca_Desi_Chr_3_3509612	Ca_desi_chr03	3509612	[T/A]	NA
Ca_Desi_Chr_3_3509623	Ca_desi_chr03	3509623	[T/C]	NA
Ca_Desi_Chr_3_3512869	Ca_desi_chr03	3512869	[A/G]	NA
Ca_Desi_Chr_3_3514344	Ca_desi_chr03	3514344	[A/C]	NA
Ca_Desi_Chr_3_3518795	Ca_desi_chr03	3518795	[G/C]	NA
Ca_Desi_Chr_3_3519589	Ca_desi_chr03	3519589	[T/C]	NA
Ca_Desi_Chr_3_3522928	Ca_desi_chr03	3522928	[C/A]	NA
Ca_Desi_Chr_3_3523403	Ca_desi_chr03	3523403	[T/C]	NA
Ca_Desi_Chr_3_3524914	Ca_desi_chr03	3524914	[A/G]	NA
Ca_Desi_Chr_3_3538547	Ca_desi_chr03	3538547	[C/A]	NA
Ca_Desi_Chr_3_3540716	Ca_desi_chr03	3540716	[T/C]	NA
Ca_Desi_Chr_3_3540720	Ca_desi_chr03	3540720	[A/G]	NA
Ca_Desi_Chr_3_3540727	Ca_desi_chr03	3540727	[A/G]	NA
Ca_Desi_Chr_3_3540732	Ca_desi_chr03	3540732	[T/G]	NA
Ca_Desi_Chr_3_3540734	Ca_desi_chr03	3540734	[T/G]	NA
Ca_Desi_Chr_3_3540737	Ca_desi_chr03	3540737	[T/C]	NA
Ca_Desi_Chr_3_3540746	Ca_desi_chr03	3540746	[A/G]	NA
Ca_Desi_Chr_3_3552607	Ca_desi_chr03	3552607	[A/C]	NA
Ca_Desi_Chr_3_3567286	Ca_desi_chr03	3567286	[T/G]	NA
Ca_Desi_Chr_3_3567287	Ca_desi_chr03	3567287	[T/G]	NA
Ca_Desi_Chr_3_3567288	Ca_desi_chr03	3567288	[T/A]	NA
Ca_Desi_Chr_3_3567289	Ca_desi_chr03	3567289	[T/G]	NA
Ca_Desi_Chr_3_3567295	Ca_desi_chr03	3567295	[T/C]	NA
Ca_Desi_Chr_3_3567444	Ca_desi_chr03	3567444	[C/T]	NA
Ca_Desi_Chr_3_3576516	Ca_desi_chr03	3576516	[C/T]	NA
Ca_Desi_Chr_3_3576517	Ca_desi_chr03	3576517	[T/C]	NA
Ca_Desi_Chr_3_3576686	Ca_desi_chr03	3576686	[A/G]	NA

SNP IDs	Chromosomes	Physical positions (bp)	SNPs	NCBI SNPdb accession IDs (NCBI_ss#)
Ca_Desi_Chr_3_3579279	Ca_desi_chr03	3579279	[A/T]	NA
Ca_Desi_Chr_3_3580883	Ca_desi_chr03	3580883	[C/T]	NA
Ca_Desi_Chr_3_3581298	Ca_desi_chr03	3581298	[G/A]	NA
Ca_Desi_Chr_3_3581299	Ca_desi_chr03	3581299	[A/C]	NA
Ca_Desi_Chr_3_3582913	Ca_desi_chr03	3582913	[T/C]	NA
Ca_Desi_Chr_3_3582918	Ca_desi_chr03	3582918	[T/G]	NA
Ca_Desi_Chr_3_3582950	Ca_desi_chr03	3582950	[T/G]	NA
Ca_Desi_Chr_3_3590707	Ca_desi_chr03	3590707	[C/T]	NA
Ca_Desi_Chr_3_3593776	Ca_desi_chr03	3593776	[A/G]	NA
Ca_Desi_Chr_3_3609578	Ca_desi_chr03	3609578	[T/C]	NA
Ca_Desi_Chr_3_3612217	Ca_desi_chr03	3612217	[T/C]	NA
Ca_Desi_Chr_3_3631276	Ca_desi_chr03	3631276	[C/T]	NA
Ca_Desi_Chr_3_3631358	Ca_desi_chr03	3631358	[A/C]	NA
Ca_Desi_Chr_3_3631402	Ca_desi_chr03	3631402	[A/G]	NA
Ca_Desi_Chr_3_3632461	Ca_desi_chr03	3632461	[T/A]	NA
Ca_Desi_Chr_3_3632483	Ca_desi_chr03	3632483	[T/C]	NA
Ca_Desi_Chr_3_3633582	Ca_desi_chr03	3633582	[A/G]	NA
Ca_Desi_Chr_3_3633583	Ca_desi_chr03	3633583	[T/G]	NA
Ca_Desi_Chr_3_3635915	Ca_desi_chr03	3635915	[A/G]	NA
Ca_Desi_Chr_3_3635965	Ca_desi_chr03	3635965	[T/C]	NA
Ca_Desi_Chr_3_3635966	Ca_desi_chr03	3635966	[A/C]	NA
Ca_Desi_Chr_3_3635967	Ca_desi_chr03	3635967	[G/A]	NA
Ca_Desi_Chr_3_3636468	Ca_desi_chr03	3636468	[C/A]	NA
Ca_Desi_Chr_3_3680723	Ca_desi_chr03	3680723	[T/G]	NA
Ca_Desi_Chr_3_3680780	Ca_desi_chr03	3680780	[A/C]	NA
Ca_Desi_Chr_3_3680792	Ca_desi_chr03	3680792	[A/C]	NA
Ca_Desi_Chr_3_3773958	Ca_desi_chr03	3773958	[A/C]	NA
Ca_Desi_Chr_3_3773990	Ca_desi_chr03	3773990	[A/C]	NA
Ca_Desi_Chr_3_3790820	Ca_desi_chr03	3790820	[A/G]	NA
Ca_Desi_Chr_3_3794394	Ca_desi_chr03	3794394	[T/C]	NA
Ca_Desi_Chr_3_3794401	Ca_desi_chr03	3794401	[G/C]	NA
Ca_Desi_Chr_3_3794410	Ca_desi_chr03	3794410	[T/C]	NA
Ca_Desi_Chr_3_3794414	Ca_desi_chr03	3794414	[T/C]	NA
Ca_Desi_Chr_3_3794446	Ca_desi_chr03	3794446	[T/C]	NA

SNP IDs	Chromosomes	Physical positions (bp)	SNPs	NCBI SNPdb accession IDs (NCBI_ss#)
Ca_Desi_Chr_3_3818254	Ca_desi_chr03	3818254	[A/C]	NA
Ca_Desi_Chr_3_3818304	Ca_desi_chr03	3818304	[T/A]	NA
Ca_Desi_Chr_3_3818414	Ca_desi_chr03	3818414	[T/C]	NA
Ca_Desi_Chr_3_3818437	Ca_desi_chr03	3818437	[A/G]	NA
Ca_Desi_Chr_3_3818447	Ca_desi_chr03	3818447	[T/G]	NA
Ca_Desi_Chr_3_3860402	Ca_desi_chr03	3860402	[T/C]	NA
Ca_Desi_Chr_3_3860697	Ca_desi_chr03	3860697	[A/C]	NA
Ca_Desi_Chr_3_3860703	Ca_desi_chr03	3860703	[A/C]	NA
Ca_Desi_Chr_3_3884552	Ca_desi_chr03	3884552	[T/C]	NA
Ca_Desi_Chr_3_3884585	Ca_desi_chr03	3884585	[T/G]	NA
Ca_Desi_Chr_3_3891391	Ca_desi_chr03	3891391	[T/C]	NA
Ca_Desi_Chr_3_3891433	Ca_desi_chr03	3891433	[A/G]	NA
Ca_Desi_Chr_3_3891482	Ca_desi_chr03	3891482	[T/G]	NA
Ca_Desi_Chr_3_3946457	Ca_desi_chr03	3946457	[G/T]	NA
Ca_Desi_Chr_3_3946535	Ca_desi_chr03	3946535	[T/C]	NA
Ca_Desi_Chr_3_3967388	Ca_desi_chr03	3967388	[A/G]	NA
Ca_Desi_Chr_3_3973381	Ca_desi_chr03	3973381	[G/A]	NA
Ca_Desi_Chr_3_3978527	Ca_desi_chr03	3978527	[G/C]	NA
Ca_Desi_Chr_3_4003568	Ca_desi_chr03	4003568	[C/A]	NA
Ca_Desi_Chr_3_4004966	Ca_desi_chr03	4004966	[G/C]	NA
Ca_Desi_Chr_3_4004974	Ca_desi_chr03	4004974	[T/A]	NA
Ca_Desi_Chr_3_4004986	Ca_desi_chr03	4004986	[G/A]	NA
Ca_Desi_Chr_3_4007763	Ca_desi_chr03	4007763	[T/C]	NA
Ca_Desi_Chr_3_4016065	Ca_desi_chr03	4016065	[A/C]	NA
Ca_Desi_Chr_3_4016154	Ca_desi_chr03	4016154	[T/C]	NA
Ca_Desi_Chr_3_4016157	Ca_desi_chr03	4016157	[A/C]	NA
Ca_Desi_Chr_3_4016158	Ca_desi_chr03	4016158	[C/G]	NA
Ca_Desi_Chr_3_4016163	Ca_desi_chr03	4016163	[A/C]	NA
Ca_Desi_Chr_3_4016170	Ca_desi_chr03	4016170	[T/C]	NA
Ca_Desi_Chr_3_4016171	Ca_desi_chr03	4016171	[C/T]	NA
Ca_Desi_Chr_3_4016176	Ca_desi_chr03	4016176	[A/C]	NA
Ca_Desi_Chr_3_4016179	Ca_desi_chr03	4016179	[T/C]	NA
Ca_Desi_Chr_3_4016181	Ca_desi_chr03	4016181	[A/C]	NA
Ca_Desi_Chr_3_4016186	Ca_desi_chr03	4016186	[C/T]	NA

SNP IDs	Chromosomes	Physical positions (bp)	SNPs	NCBI SNPdb accession IDs (NCBI_ss#)
Ca_Desi_Chr_3_4019445	Ca_desi_chr03	4019445	[G/T]	NA
Ca_Desi_Chr_3_4019505	Ca_desi_chr03	4019505	[C/T]	NA
Ca_Desi_Chr_3_4029005	Ca_desi_chr03	4029005	[T/G]	NA
Ca_Desi_Chr_3_4029036	Ca_desi_chr03	4029036	[A/C]	NA
Ca_Desi_Chr_3_4034617	Ca_desi_chr03	4034617	[T/G]	NA
Ca_Desi_Chr_3_4034689	Ca_desi_chr03	4034689	[C/T]	NA
Ca_Desi_Chr_3_4038954	Ca_desi_chr03	4038954	[C/G]	NA
Ca_Desi_Chr_3_4039042	Ca_desi_chr03	4039042	[G/A]	NA
Ca_Desi_Chr_3_4040430	Ca_desi_chr03	4040430	[A/G]	NA
Ca_Desi_Chr_3_4053024	Ca_desi_chr03	4053024	[A/G]	NA
Ca_Desi_Chr_3_4053071	Ca_desi_chr03	4053071	[A/C]	NA
Ca_Desi_Chr_3_4053086	Ca_desi_chr03	4053086	[G/T]	NA
Ca_Desi_Chr_3_4086443	Ca_desi_chr03	4086443	[C/T]	NA
Ca_Desi_Chr_3_4086479	Ca_desi_chr03	4086479	[T/C]	NA
Ca_Desi_Chr_3_4100562	Ca_desi_chr03	4100562	[A/G]	NA
Ca_Desi_Chr_3_4100572	Ca_desi_chr03	4100572	[T/C]	NA
Ca_Desi_Chr_3_4100597	Ca_desi_chr03	4100597	[T/C]	NA
Ca_Desi_Chr_3_4100603	Ca_desi_chr03	4100603	[A/G]	NA
Ca_Desi_Chr_3_4100616	Ca_desi_chr03	4100616	[T/G]	NA
Ca_Desi_Chr_3_4100725	Ca_desi_chr03	4100725	[T/A]	NA
Ca_Desi_Chr_3_4100741	Ca_desi_chr03	4100741	[A/G]	NA
Ca_Desi_Chr_3_4100757	Ca_desi_chr03	4100757	[G/T]	NA
Ca_Desi_Chr_3_4118888	Ca_desi_chr03	4118888	[A/G]	NA
Ca_Desi_Chr_3_4146675	Ca_desi_chr03	4146675	[A/G]	NA
Ca_Desi_Chr_3_4162921	Ca_desi_chr03	4162921	[G/C]	NA
Ca_Desi_Chr_3_4174824	Ca_desi_chr03	4174824	[G/T]	NA
Ca_Desi_Chr_3_4174837	Ca_desi_chr03	4174837	[G/A]	NA
Ca_Desi_Chr_3_4174863	Ca_desi_chr03	4174863	[G/A]	NA
Ca_Desi_Chr_3_4175057	Ca_desi_chr03	4175057	[A/G]	NA
Ca_Desi_Chr_3_4175066	Ca_desi_chr03	4175066	[T/C]	NA
Ca_Desi_Chr_3_4175622	Ca_desi_chr03	4175622	[C/A]	NA
Ca_Desi_Chr_3_4182356	Ca_desi_chr03	4182356	[T/A]	NA
Ca_Desi_Chr_3_4197023	Ca_desi_chr03	4197023	[T/A]	NA
Ca_Desi_Chr_3_4197024	Ca_desi_chr03	4197024	[T/A]	NA

SNP IDs	Chromosomes	Physical positions (bp)	SNPs	NCBI SNPdb accession IDs (NCBI_ss#)
Ca_Desi_Chr_3_4197306	Ca_desi_chr03	4197306	[A/G]	NA
Ca_Desi_Chr_3_4197393	Ca_desi_chr03	4197393	[G/T]	NA
Ca_Desi_Chr_3_4197395	Ca_desi_chr03	4197395	[T/G]	NA
Ca_Desi_Chr_3_4197482	Ca_desi_chr03	4197482	[T/A]	NA
Ca_Desi_Chr_3_4197503	Ca_desi_chr03	4197503	[A/G]	NA
Ca_Desi_Chr_3_4211443	Ca_desi_chr03	4211443	[A/T]	NA
Ca_Desi_Chr_3_4211452	Ca_desi_chr03	4211452	[A/G]	NA
Ca_Desi_Chr_3_4211455	Ca_desi_chr03	4211455	[C/T]	NA
Ca_Desi_Chr_3_4211456	Ca_desi_chr03	4211456	[T/C]	NA
Ca_Desi_Chr_3_4211470	Ca_desi_chr03	4211470	[T/C]	NA
Ca_Desi_Chr_3_4211474	Ca_desi_chr03	4211474	[T/C]	NA
Ca_Desi_Chr_3_4211476	Ca_desi_chr03	4211476	[T/C]	NA
Ca_Desi_Chr_3_4211480	Ca_desi_chr03	4211480	[A/G]	NA
Ca_Desi_Chr_3_4211495	Ca_desi_chr03	4211495	[T/A]	NA
Ca_Desi_Chr_3_4216738	Ca_desi_chr03	4216738	[T/C]	NA
Ca_Desi_Chr_3_4229793	Ca_desi_chr03	4229793	[A/G]	NA
Ca_Desi_Chr_3_4250039	Ca_desi_chr03	4250039	[T/A]	NA
Ca_Desi_Chr_3_4250042	Ca_desi_chr03	4250042	[T/G]	NA
Ca_Desi_Chr_3_4250045	Ca_desi_chr03	4250045	[T/G]	NA
Ca_Desi_Chr_3_4250048	Ca_desi_chr03	4250048	[T/G]	NA
Ca_Desi_Chr_3_4250059	Ca_desi_chr03	4250059	[A/T]	NA
Ca_Desi_Chr_3_4250077	Ca_desi_chr03	4250077	[A/G]	NA
Ca_Desi_Chr_3_4261374	Ca_desi_chr03	4261374	[A/G]	NA
Ca_Desi_Chr_3_4261482	Ca_desi_chr03	4261482	[T/C]	NA
Ca_Desi_Chr_3_4300111	Ca_desi_chr03	4300111	[T/C]	NA
Ca_Desi_Chr_3_4303915	Ca_desi_chr03	4303915	[A/G]	NA
Ca_Desi_Chr_3_4303961	Ca_desi_chr03	4303961	[T/G]	NA
Ca_Desi_Chr_3_4330756	Ca_desi_chr03	4330756	[C/T]	NA
Ca_Desi_Chr_3_4347515	Ca_desi_chr03	4347515	[G/T]	NA
Ca_Desi_Chr_3_4349644	Ca_desi_chr03	4349644	[G/A]	NA
Ca_Desi_Chr_3_4356981	Ca_desi_chr03	4356981	[T/C]	NA
Ca_Desi_Chr_3_4376815	Ca_desi_chr03	4376815	[G/A]	NA
Ca_Desi_Chr_3_4387022	Ca_desi_chr03	4387022	[G/A]	NA
Ca_Desi_Chr_3_4387126	Ca_desi_chr03	4387126	[A/C]	NA

SNP IDs	Chromosomes	Physical positions (bp)	SNPs	NCBI SNPdb accession IDs (NCBI_ss#)
Ca_Desi_Chr_3_4401074	Ca_desi_chr03	4401074	[G/A]	NA
Ca_Desi_Chr_3_4409758	Ca_desi_chr03	4409758	[A/G]	NA
Ca_Desi_Chr_3_4409776	Ca_desi_chr03	4409776	[T/A]	NA
Ca_Desi_Chr_3_4409777	Ca_desi_chr03	4409777	[G/A]	NA
Ca_Desi_Chr_3_4409778	Ca_desi_chr03	4409778	[T/C]	NA
Ca_Desi_Chr_3_4415709	Ca_desi_chr03	4415709	[C/T]	NA
Ca_Desi_Chr_3_4431197	Ca_desi_chr03	4431197	[A/T]	NA
Ca_Desi_Chr_3_4518693	Ca_desi_chr03	4518693	[A/C]	NA
Ca_Desi_Chr_3_4518697	Ca_desi_chr03	4518697	[A/C]	NA
Ca_Desi_Chr_3_4518740	Ca_desi_chr03	4518740	[A/G]	NA
Ca_Desi_Chr_3_4518794	Ca_desi_chr03	4518794	[A/G]	NA
Ca_Desi_Chr_3_4573017	Ca_desi_chr03	4573017	[T/G]	NA
Ca_Desi_Chr_3_4581079	Ca_desi_chr03	4581079	[T/G]	NA
Ca_Desi_Chr_3_4656390	Ca_desi_chr03	4656390	[A/C]	NA
Ca_Desi_Chr_3_4664841	Ca_desi_chr03	4664841	[T/C]	NA
Ca_Desi_Chr_3_4664858	Ca_desi_chr03	4664858	[T/G]	NA
Ca_Desi_Chr_3_4664861	Ca_desi_chr03	4664861	[A/G]	NA
Ca_Desi_Chr_3_4664863	Ca_desi_chr03	4664863	[T/G]	NA
Ca_Desi_Chr_3_4664877	Ca_desi_chr03	4664877	[T/C]	NA
Ca_Desi_Chr_3_4664884	Ca_desi_chr03	4664884	[G/A]	NA
Ca_Desi_Chr_3_4664895	Ca_desi_chr03	4664895	[A/G]	NA
Ca_Desi_Chr_3_4664897	Ca_desi_chr03	4664897	[A/G]	NA
Ca_Desi_Chr_3_4664905	Ca_desi_chr03	4664905	[T/C]	NA
Ca_Desi_Chr_3_4787268	Ca_desi_chr03	4787268	[T/G]	NA
Ca_Desi_Chr_3_4787305	Ca_desi_chr03	4787305	[T/C]	NA
Ca_Desi_Chr_3_4787347	Ca_desi_chr03	4787347	[T/C]	NA
Ca_Desi_Chr_3_4787351	Ca_desi_chr03	4787351	[A/C]	NA
Ca_Desi_Chr_3_4788785	Ca_desi_chr03	4788785	[T/C]	NA
Ca_Desi_Chr_3_4790674	Ca_desi_chr03	4790674	[A/G]	NA
Ca_Desi_Chr_3_4790712	Ca_desi_chr03	4790712	[T/G]	NA
Ca_Desi_Chr_3_4801073	Ca_desi_chr03	4801073	[A/C]	NA
Ca_Desi_Chr_3_4801676	Ca_desi_chr03	4801676	[T/G]	NA
Ca_Desi_Chr_3_4801690	Ca_desi_chr03	4801690	[T/C]	NA
Ca_Desi_Chr_3_4850722	Ca_desi_chr03	4850722	[T/C]	NA

SNP IDs	Chromosomes	Physical positions (bp)	SNPs	NCBI SNPdb accession IDs (NCBI_ss#)
Ca_Desi_Chr_3_4850735	Ca_desi_chr03	4850735	[T/G]	NA
Ca_Desi_Chr_3_4850763	Ca_desi_chr03	4850763	[T/A]	NA
Ca_Desi_Chr_3_4850764	Ca_desi_chr03	4850764	[A/G]	NA
Ca_Desi_Chr_3_4850767	Ca_desi_chr03	4850767	[A/G]	NA
Ca_Desi_Chr_3_4850770	Ca_desi_chr03	4850770	[T/C]	NA
Ca_Desi_Chr_3_5804656	Ca_desi_chr03	5804656	[A/G]	NA
Ca_Desi_Chr_3_5804673	Ca_desi_chr03	5804673	[T/C]	NA
Ca_Desi_Chr_3_5804690	Ca_desi_chr03	5804690	[A/G]	NA
Ca_Desi_Chr_3_5804696	Ca_desi_chr03	5804696	[A/C]	NA
Ca_Desi_Chr_3_5804704	Ca_desi_chr03	5804704	[T/C]	NA
Ca_Desi_Chr_3_5804708	Ca_desi_chr03	5804708	[A/C]	NA
Ca_Desi_Chr_3_5804745	Ca_desi_chr03	5804745	[C/G]	NA
Ca_Desi_Chr_3_5819987	Ca_desi_chr03	5819987	[T/G]	NA
Ca_Desi_Chr_3_5820012	Ca_desi_chr03	5820012	[A/C]	NA
Ca_Desi_Chr_3_5820035	Ca_desi_chr03	5820035	[A/C]	NA
Ca_Desi_Chr_3_5955064	Ca_desi_chr03	5955064	[A/G]	NA
Ca_Desi_Chr_3_6011843	Ca_desi_chr03	6011843	[A/G]	NA
Ca_Desi_Chr_3_6011856	Ca_desi_chr03	6011856	[A/G]	NA
Ca_Desi_Chr_3_6011861	Ca_desi_chr03	6011861	[T/C]	NA
Ca_Desi_Chr_3_6012086	Ca_desi_chr03	6012086	[T/G]	NA
Ca_Desi_Chr_3_6055661	Ca_desi_chr03	6055661	[T/A]	NA
Ca_Desi_Chr_3_6078517	Ca_desi_chr03	6078517	[T/C]	NA
Ca_Desi_Chr_3_6078534	Ca_desi_chr03	6078534	[T/C]	NA
Ca_Desi_Chr_3_6078559	Ca_desi_chr03	6078559	[A/G]	NA
Ca_Desi_Chr_3_6108989	Ca_desi_chr03	6108989	[A/C]	NA
Ca_Desi_Chr_3_6120642	Ca_desi_chr03	6120642	[A/G]	NA
Ca_Desi_Chr_3_6120754	Ca_desi_chr03	6120754	[T/C]	NA
Ca_Desi_Chr_3_6120883	Ca_desi_chr03	6120883	[T/C]	NA
Ca_Desi_Chr_3_6120918	Ca_desi_chr03	6120918	[A/G]	NA
Ca_Desi_Chr_3_6149264	Ca_desi_chr03	6149264	[A/C]	NA
Ca_Desi_Chr_3_6149274	Ca_desi_chr03	6149274	[A/C]	NA
Ca_Desi_Chr_3_6149280	Ca_desi_chr03	6149280	[T/C]	NA
Ca_Desi_Chr_3_6149794	Ca_desi_chr03	6149794	[A/T]	NA
Ca_Desi_Chr_3_6149808	Ca_desi_chr03	6149808	[C/T]	NA

SNP IDs	Chromosomes	Physical positions (bp)	SNPs	NCBI SNPdb accession IDs (NCBI_ss#)
Ca_Desi_Chr_3_6149814	Ca_desi_chr03	6149814	[T/C]	NA
Ca_Desi_Chr_3_6149822	Ca_desi_chr03	6149822	[A/C]	NA
Ca_Desi_Chr_3_6149827	Ca_desi_chr03	6149827	[A/G]	NA
Ca_Desi_Chr_3_6149832	Ca_desi_chr03	6149832	[A/C]	NA
Ca_Desi_Chr_3_6149838	Ca_desi_chr03	6149838	[T/C]	NA
Ca_Desi_Chr_3_6149847	Ca_desi_chr03	6149847	[T/A]	NA
Ca_Desi_Chr_3_6172170	Ca_desi_chr03	6172170	[G/A]	NA
Ca_Desi_Chr_3_6172185	Ca_desi_chr03	6172185	[A/G]	NA
Ca_Desi_Chr_3_6172225	Ca_desi_chr03	6172225	[T/A]	NA
Ca_Desi_Chr_3_6173806	Ca_desi_chr03	6173806	[T/C]	NA
Ca_Desi_Chr_3_6173819	Ca_desi_chr03	6173819	[T/G]	NA
Ca_Desi_Chr_3_6173833	Ca_desi_chr03	6173833	[A/C]	NA
Ca_Desi_Chr_3_6234271	Ca_desi_chr03	6234271	[T/G]	NA
Ca_Desi_Chr_3_6234275	Ca_desi_chr03	6234275	[T/G]	NA
Ca_Desi_Chr_3_6258573	Ca_desi_chr03	6258573	[A/G]	NA
Ca_Desi_Chr_3_6258596	Ca_desi_chr03	6258596	[T/C]	NA
Ca_Desi_Chr_3_6295067	Ca_desi_chr03	6295067	[A/C]	NA
Ca_Desi_Chr_3_6295076	Ca_desi_chr03	6295076	[A/C]	NA
Ca_Desi_Chr_3_6324004	Ca_desi_chr03	6324004	[T/C]	NA
Ca_Desi_Chr_3_6324015	Ca_desi_chr03	6324015	[T/G]	NA
Ca_Desi_Chr_3_6352904	Ca_desi_chr03	6352904	[A/T]	NA
Ca_Desi_Chr_3_6357520	Ca_desi_chr03	6357520	[A/C]	NA
Ca_Desi_Chr_3_6357533	Ca_desi_chr03	6357533	[T/C]	NA
Ca_Desi_Chr_3_6357547	Ca_desi_chr03	6357547	[T/G]	NA
Ca_Desi_Chr_3_6357559	Ca_desi_chr03	6357559	[T/G]	NA
Ca_Desi_Chr_3_6390861	Ca_desi_chr03	6390861	[G/A]	NA
Ca_Desi_Chr_3_6423021	Ca_desi_chr03	6423021	[T/G]	NA
Ca_Desi_Chr_3_6423031	Ca_desi_chr03	6423031	[G/T]	NA
Ca_Desi_Chr_3_6423039	Ca_desi_chr03	6423039	[T/C]	NA
Ca_Desi_Chr_3_6426438	Ca_desi_chr03	6426438	[A/G]	NA
Ca_Desi_Chr_3_6426450	Ca_desi_chr03	6426450	[T/G]	NA
Ca_Desi_Chr_3_6426464	Ca_desi_chr03	6426464	[A/C]	NA
Ca_Desi_Chr_3_6430332	Ca_desi_chr03	6430332	[T/C]	NA
Ca_Desi_Chr_3_6430353	Ca_desi_chr03	6430353	[T/C]	NA

SNP IDs	Chromosomes	Physical positions (bp)	SNPs	NCBI SNPdb accession IDs (NCBI_ss#)
Ca_Desi_Chr_3_6434304	Ca_desi_chr03	6434304	[G/T]	NA
Ca_Desi_Chr_3_6434644	Ca_desi_chr03	6434644	[C/T]	NA
Ca_Desi_Chr_3_6434961	Ca_desi_chr03	6434961	[T/A]	NA
Ca_Desi_Chr_3_6435004	Ca_desi_chr03	6435004	[T/G]	NA
Ca_Desi_Chr_3_6435914	Ca_desi_chr03	6435914	[C/A]	NA
Ca_Desi_Chr_3_6435997	Ca_desi_chr03	6435997	[G/A]	NA
Ca_Desi_Chr_3_6436024	Ca_desi_chr03	6436024	[A/G]	NA
Ca_Desi_Chr_3_6436047	Ca_desi_chr03	6436047	[A/G]	NA
Ca_Desi_Chr_3_6436085	Ca_desi_chr03	6436085	[A/G]	NA
Ca_Desi_Chr_3_6436108	Ca_desi_chr03	6436108	[C/A]	NA
Ca_Desi_Chr_3_6449885	Ca_desi_chr03	6449885	[G/T]	NA
Ca_Desi_Chr_3_6449886	Ca_desi_chr03	6449886	[T/G]	NA
Ca_Desi_Chr_3_6490965	Ca_desi_chr03	6490965	[T/C]	NA
Ca_Desi_Chr_3_6499735	Ca_desi_chr03	6499735	[C/A]	NA
Ca_Desi_Chr_3_6499738	Ca_desi_chr03	6499738	[A/C]	NA
Ca_Desi_Chr_3_6499775	Ca_desi_chr03	6499775	[A/G]	NA
Ca_Desi_Chr_3_6531280	Ca_desi_chr03	6531280	[A/C]	NA
Ca_Desi_Chr_3_6531284	Ca_desi_chr03	6531284	[A/C]	NA
Ca_Desi_Chr_3_6531306	Ca_desi_chr03	6531306	[A/G]	NA
Ca_Desi_Chr_3_6539810	Ca_desi_chr03	6539810	[C/T]	NA
Ca_Desi_Chr_3_6543089	Ca_desi_chr03	6543089	[T/A]	NA
Ca_Desi_Chr_3_6543102	Ca_desi_chr03	6543102	[G/A]	NA
Ca_Desi_Chr_3_6543338	Ca_desi_chr03	6543338	[C/A]	NA
Ca_Desi_Chr_3_6543339	Ca_desi_chr03	6543339	[T/A]	NA
Ca_Desi_Chr_3_6543340	Ca_desi_chr03	6543340	[A/T]	NA
Ca_Desi_Chr_3_6543406	Ca_desi_chr03	6543406	[T/C]	NA
Ca_Desi_Chr_3_6544021	Ca_desi_chr03	6544021	[A/T]	NA
Ca_Desi_Chr_3_6544048	Ca_desi_chr03	6544048	[T/C]	NA
Ca_Desi_Chr_3_6544126	Ca_desi_chr03	6544126	[A/G]	NA
Ca_Desi_Chr_3_6544365	Ca_desi_chr03	6544365	[A/G]	NA
Ca_Desi_Chr_3_6547078	Ca_desi_chr03	6547078	[C/T]	NA
Ca_Desi_Chr_3_6547513	Ca_desi_chr03	6547513	[G/A]	NA
Ca_Desi_Chr_3_6547617	Ca_desi_chr03	6547617	[A/C]	NA
Ca_Desi_Chr_3_6585721	Ca_desi_chr03	6585721	[C/A]	NA

SNP IDs	Chromosomes	Physical positions (bp)	SNPs	NCBI SNPdb accession IDs (NCBI_ss#)
Ca_Desi_Chr_3_6593404	Ca_desi_chr03	6593404	[A/G]	NA
Ca_Desi_Chr_3_6593488	Ca_desi_chr03	6593488	[C/T]	NA
Ca_Desi_Chr_3_6593914	Ca_desi_chr03	6593914	[A/G]	NA
Ca_Desi_Chr_3_6616912	Ca_desi_chr03	6616912	[A/G]	NA
Ca_Desi_Chr_3_6635977	Ca_desi_chr03	6635977	[T/C]	NA
Ca_Desi_Chr_3_6636009	Ca_desi_chr03	6636009	[T/G]	NA
Ca_Desi_Chr_3_6636054	Ca_desi_chr03	6636054	[A/C]	NA
Ca_Desi_Chr_3_6641323	Ca_desi_chr03	6641323	[A/G]	NA
Ca_Desi_Chr_3_6659003	Ca_desi_chr03	6659003	[A/C]	NA
Ca_Desi_Chr_3_6660661	Ca_desi_chr03	6660661	[A/C]	NA
Ca_Desi_Chr_3_6660873	Ca_desi_chr03	6660873	[T/C]	NA
Ca_Desi_Chr_3_6662460	Ca_desi_chr03	6662460	[A/C]	NA
Ca_Desi_Chr_3_6717275	Ca_desi_chr03	6717275	[T/G]	NA
Ca_Desi_Chr_3_6720703	Ca_desi_chr03	6720703	[A/C]	NA
Ca_Desi_Chr_3_6721108	Ca_desi_chr03	6721108	[C/G]	NA
Ca_Desi_Chr_3_6721110	Ca_desi_chr03	6721110	[C/A]	NA
Ca_Desi_Chr_3_6721112	Ca_desi_chr03	6721112	[A/C]	NA
Ca_Desi_Chr_3_6723814	Ca_desi_chr03	6723814	[A/G]	NA
Ca_Desi_Chr_3_6723839	Ca_desi_chr03	6723839	[G/A]	NA
Ca_Desi_Chr_3_6723852	Ca_desi_chr03	6723852	[C/G]	NA
Ca_Desi_Chr_3_6738310	Ca_desi_chr03	6738310	[T/C]	NA
Ca_Desi_Chr_3_6738325	Ca_desi_chr03	6738325	[T/G]	NA
Ca_Desi_Chr_3_6738954	Ca_desi_chr03	6738954	[A/G]	NA
Ca_Desi_Chr_3_6738958	Ca_desi_chr03	6738958	[A/G]	NA
Ca_Desi_Chr_3_6738968	Ca_desi_chr03	6738968	[T/G]	NA
Ca_Desi_Chr_3_6738992	Ca_desi_chr03	6738992	[A/G]	NA
Ca_Desi_Chr_3_6738997	Ca_desi_chr03	6738997	[T/A]	NA
Ca_Desi_Chr_3_6738999	Ca_desi_chr03	6738999	[T/C]	NA
Ca_Desi_Chr_3_6748500	Ca_desi_chr03	6748500	[T/C]	NA
Ca_Desi_Chr_3_6757836	Ca_desi_chr03	6757836	[G/A]	NA
Ca_Desi_Chr_3_6757904	Ca_desi_chr03	6757904	[A/G]	NA
Ca_Desi_Chr_3_6758225	Ca_desi_chr03	6758225	[G/A]	NA
Ca_Desi_Chr_3_6758227	Ca_desi_chr03	6758227	[T/C]	NA
Ca_Desi_Chr_3_6758332	Ca_desi_chr03	6758332	[G/A]	NA

SNP IDs	Chromosomes	Physical positions (bp)	SNPs	NCBI SNPdb accession IDs (NCBI_ss#)
Ca_Desi_Chr_3_6758362	Ca_desi_chr03	6758362	[T/C]	NA
Ca_Desi_Chr_3_6758389	Ca_desi_chr03	6758389	[C/T]	NA
Ca_Desi_Chr_3_6758514	Ca_desi_chr03	6758514	[C/T]	NA
Ca_Desi_Chr_3_6776131	Ca_desi_chr03	6776131	[T/C]	NA
Ca_Desi_Chr_3_6776192	Ca_desi_chr03	6776192	[G/A]	NA
Ca_Desi_Chr_3_6776547	Ca_desi_chr03	6776547	[C/T]	NA
Ca_Desi_Chr_3_6790176	Ca_desi_chr03	6790176	[A/C]	NA
Ca_Desi_Chr_3_6790184	Ca_desi_chr03	6790184	[A/G]	NA
Ca_Desi_Chr_3_6790193	Ca_desi_chr03	6790193	[A/G]	NA
Ca_Desi_Chr_3_6799092	Ca_desi_chr03	6799092	[T/A]	NA
Ca_Desi_Chr_3_6799102	Ca_desi_chr03	6799102	[T/C]	NA
Ca_Desi_Chr_3_6799103	Ca_desi_chr03	6799103	[T/C]	NA
Ca_Desi_Chr_3_6812090	Ca_desi_chr03	6812090	[C/T]	NA
Ca_Desi_Chr_3_6812095	Ca_desi_chr03	6812095	[T/C]	NA
Ca_Desi_Chr_3_6812106	Ca_desi_chr03	6812106	[A/G]	NA
Ca_Desi_Chr_3_6812111	Ca_desi_chr03	6812111	[A/G]	NA
Ca_Desi_Chr_3_6812169	Ca_desi_chr03	6812169	[A/G]	NA
Ca_Desi_Chr_3_6812183	Ca_desi_chr03	6812183	[G/C]	NA
Ca_Desi_Chr_3_6812195	Ca_desi_chr03	6812195	[C/T]	NA
Ca_Desi_Chr_3_6833926	Ca_desi_chr03	6833926	[A/C]	NA
Ca_Desi_Chr_3_6833932	Ca_desi_chr03	6833932	[C/G]	NA
Ca_Desi_Chr_3_6833975	Ca_desi_chr03	6833975	[T/C]	NA
Ca_Desi_Chr_3_6877283	Ca_desi_chr03	6877283	[A/T]	NA
Ca_Desi_Chr_3_6877311	Ca_desi_chr03	6877311	[T/C]	NA
Ca_Desi_Chr_3_6877359	Ca_desi_chr03	6877359	[A/G]	NA
Ca_Desi_Chr_3_6963249	Ca_desi_chr03	6963249	[T/G]	NA
Ca_Desi_Chr_3_6963252	Ca_desi_chr03	6963252	[A/G]	NA
Ca_Desi_Chr_3_7059071	Ca_desi_chr03	7059071	[A/G]	NA
Ca_Desi_Chr_3_7059191	Ca_desi_chr03	7059191	[T/C]	NA
Ca_Desi_Chr_3_7059195	Ca_desi_chr03	7059195	[T/C]	NA
Ca_Desi_Chr_3_7059225	Ca_desi_chr03	7059225	[T/C]	NA
Ca_Desi_Chr_3_7059324	Ca_desi_chr03	7059324	[C/T]	NA
Ca_Desi_Chr_3_7059357	Ca_desi_chr03	7059357	[A/C]	NA
Ca_Desi_Chr_3_7104060	Ca_desi_chr03	7104060	[G/A]	NA

SNP IDs	Chromosomes	Physical positions (bp)	SNPs	NCBI SNPdb accession IDs (NCBI_ss#)
Ca_Desi_Chr_3_7105481	Ca_desi_chr03	7105481	[A/C]	NA
Ca_Desi_Chr_3_7105495	Ca_desi_chr03	7105495	[A/C]	NA
Ca_Desi_Chr_3_7105497	Ca_desi_chr03	7105497	[T/C]	NA
Ca_Desi_Chr_3_7139294	Ca_desi_chr03	7139294	[T/G]	NA
Ca_Desi_Chr_3_7218323	Ca_desi_chr03	7218323	[T/G]	NA
Ca_Desi_Chr_3_7221607	Ca_desi_chr03	7221607	[T/C]	NA
Ca_Desi_Chr_3_7221632	Ca_desi_chr03	7221632	[A/C]	NA
Ca_Desi_Chr_3_7221635	Ca_desi_chr03	7221635	[G/C]	NA
Ca_Desi_Chr_3_7221640	Ca_desi_chr03	7221640	[A/C]	NA
Ca_Desi_Chr_3_7221650	Ca_desi_chr03	7221650	[A/T]	NA
Ca_Desi_Chr_3_7226963	Ca_desi_chr03	7226963	[A/G]	NA
Ca_Desi_Chr_3_7226964	Ca_desi_chr03	7226964	[G/T]	NA
Ca_Desi_Chr_3_7226966	Ca_desi_chr03	7226966	[T/A]	NA
Ca_Desi_Chr_3_8448964	Ca_desi_chr03	8448964	[T/G]	NA
Ca_Desi_Chr_3_8454758	Ca_desi_chr03	8454758	[A/C]	NA
Ca_Desi_Chr_3_8454789	Ca_desi_chr03	8454789	[C/T]	NA
Ca_Desi_Chr_3_8454791	Ca_desi_chr03	8454791	[C/T]	NA
Ca_Desi_Chr_3_8456436	Ca_desi_chr03	8456436	[A/C]	NA
Ca_Desi_Chr_3_8498709	Ca_desi_chr03	8498709	[T/C]	NA
Ca_Desi_Chr_3_8498710	Ca_desi_chr03	8498710	[C/G]	NA
Ca_Desi_Chr_3_8498746	Ca_desi_chr03	8498746	[A/G]	NA
Ca_Desi_Chr_3_8498748	Ca_desi_chr03	8498748	[T/C]	NA
Ca_Desi_Chr_3_8499543	Ca_desi_chr03	8499543	[T/G]	NA
Ca_Desi_Chr_3_8499725	Ca_desi_chr03	8499725	[A/G]	NA
Ca_Desi_Chr_3_8499765	Ca_desi_chr03	8499765	[T/G]	NA
Ca_Desi_Chr_3_8540832	Ca_desi_chr03	8540832	[T/A]	NA
Ca_Desi_Chr_3_8556843	Ca_desi_chr03	8556843	[A/T]	NA
Ca_Desi_Chr_3_8556851	Ca_desi_chr03	8556851	[A/G]	NA
Ca_Desi_Chr_3_8556866	Ca_desi_chr03	8556866	[C/T]	NA
Ca_Desi_Chr_3_8556888	Ca_desi_chr03	8556888	[A/T]	NA
Ca_Desi_Chr_3_8556914	Ca_desi_chr03	8556914	[A/C]	NA
Ca_Desi_Chr_3_8556941	Ca_desi_chr03	8556941	[A/G]	NA
Ca_Desi_Chr_3_8576619	Ca_desi_chr03	8576619	[T/G]	NA
Ca_Desi_Chr_3_8583785	Ca_desi_chr03	8583785	[T/G]	NA

SNP IDs	Chromosomes	Physical positions (bp)	SNPs	NCBI SNPdb accession IDs (NCBI_ss#)
Ca_Desi_Chr_3_8814644	Ca_desi_chr03	8814644	[A/G]	NA
Ca_Desi_Chr_3_8824939	Ca_desi_chr03	8824939	[T/C]	NA
Ca_Desi_Chr_3_8844825	Ca_desi_chr03	8844825	[A/T]	NA
Ca_Desi_Chr_3_8844832	Ca_desi_chr03	8844832	[G/C]	NA
Ca_Desi_Chr_3_8844834	Ca_desi_chr03	8844834	[T/G]	NA
Ca_Desi_Chr_3_8844836	Ca_desi_chr03	8844836	[A/C]	NA
Ca_Desi_Chr_3_8894544	Ca_desi_chr03	8894544	[G/A]	NA
Ca_Desi_Chr_3_8894554	Ca_desi_chr03	8894554	[T/C]	NA
Ca_Desi_Chr_3_8894991	Ca_desi_chr03	8894991	[A/C]	NA
Ca_Desi_Chr_3_8907485	Ca_desi_chr03	8907485	[A/T]	NA
Ca_Desi_Chr_3_8974956	Ca_desi_chr03	8974956	[T/G]	NA
Ca_Desi_Chr_3_9852567	Ca_desi_chr03	9852567	[T/C]	NA
Ca_Desi_Chr_3_9852588	Ca_desi_chr03	9852588	[G/A]	NA
Ca_Desi_Chr_3_9852607	Ca_desi_chr03	9852607	[T/G]	NA
Ca_Desi_Chr_3_9929365	Ca_desi_chr03	9929365	[T/C]	NA
Ca_Desi_Chr_3_9929366	Ca_desi_chr03	9929366	[T/C]	NA
Ca_Desi_Chr_3_9932975	Ca_desi_chr03	9932975	[T/G]	NA
Ca_Desi_Chr_3_9932979	Ca_desi_chr03	9932979	[T/G]	NA
Ca_Desi_Chr_3_9933051	Ca_desi_chr03	9933051	[A/T]	NA
Ca_Desi_Chr_3_9933054	Ca_desi_chr03	9933054	[A/G]	NA
Ca_Desi_Chr_3_9938057	Ca_desi_chr03	9938057	[A/C]	NA
Ca_Desi_Chr_3_9951202	Ca_desi_chr03	9951202	[A/T]	NA
Ca_Desi_Chr_3_9951203	Ca_desi_chr03	9951203	[C/T]	NA
Ca_Desi_Chr_3_9951222	Ca_desi_chr03	9951222	[A/C]	NA
Ca_Desi_Chr_3_10078354	Ca_desi_chr03	10078354	[A/C]	NA
Ca_Desi_Chr_3_10078357	Ca_desi_chr03	10078357	[A/C]	NA
Ca_Desi_Chr_3_10078375	Ca_desi_chr03	10078375	[A/G]	NA
Ca_Desi_Chr_3_10078392	Ca_desi_chr03	10078392	[A/G]	NA
Ca_Desi_Chr_3_10157456	Ca_desi_chr03	10157456	[T/A]	NA
Ca_Desi_Chr_3_10175589	Ca_desi_chr03	10175589	[T/C]	NA
Ca_Desi_Chr_3_10175600	Ca_desi_chr03	10175600	[A/G]	NA
Ca_Desi_Chr_3_10175607	Ca_desi_chr03	10175607	[T/G]	NA
Ca_Desi_Chr_3_10175622	Ca_desi_chr03	10175622	[T/C]	NA
Ca_Desi_Chr_3_10233609	Ca_desi_chr03	10233609	[T/C]	NA

SNP IDs	Chromosomes	Physical positions (bp)	SNPs	NCBI SNPdb accession IDs (NCBI_ss#)
Ca_Desi_Chr_3_10233610	Ca_desi_chr03	10233610	[T/G]	NA
Ca_Desi_Chr_3_10233615	Ca_desi_chr03	10233615	[C/A]	NA
Ca_Desi_Chr_3_10233646	Ca_desi_chr03	10233646	[A/C]	NA
Ca_Desi_Chr_3_10290850	Ca_desi_chr03	10290850	[A/C]	NA
Ca_Desi_Chr_3_10308956	Ca_desi_chr03	10308956	[A/C]	NA
Ca_Desi_Chr_3_10350068	Ca_desi_chr03	10350068	[T/G]	NA
Ca_Desi_Chr_3_10350155	Ca_desi_chr03	10350155	[A/G]	NA
Ca_Desi_Chr_3_10387947	Ca_desi_chr03	10387947	[A/C]	NA
Ca_Desi_Chr_3_10388055	Ca_desi_chr03	10388055	[A/C]	NA
Ca_Desi_Chr_3_10446502	Ca_desi_chr03	10446502	[T/C]	NA
Ca_Desi_Chr_3_10446531	Ca_desi_chr03	10446531	[A/G]	NA
Ca_Desi_Chr_3_10468060	Ca_desi_chr03	10468060	[A/T]	NA
Ca_Desi_Chr_3_10509349	Ca_desi_chr03	10509349	[A/C]	NA
Ca_Desi_Chr_3_10509908	Ca_desi_chr03	10509908	[T/G]	NA
Ca_Desi_Chr_3_10509917	Ca_desi_chr03	10509917	[T/G]	NA
Ca_Desi_Chr_3_10535686	Ca_desi_chr03	10535686	[T/A]	NA
Ca_Desi_Chr_3_10535735	Ca_desi_chr03	10535735	[A/C]	NA
Ca_Desi_Chr_3_10535765	Ca_desi_chr03	10535765	[A/C]	NA
Ca_Desi_Chr_3_10535777	Ca_desi_chr03	10535777	[A/C]	NA
Ca_Desi_Chr_3_10598646	Ca_desi_chr03	10598646	[A/T]	NA
Ca_Desi_Chr_3_10598647	Ca_desi_chr03	10598647	[T/C]	NA
Ca_Desi_Chr_3_10598648	Ca_desi_chr03	10598648	[C/G]	NA
Ca_Desi_Chr_3_10598649	Ca_desi_chr03	10598649	[T/G]	NA
Ca_Desi_Chr_3_10656870	Ca_desi_chr03	10656870	[T/C]	NA
Ca_Desi_Chr_3_10656878	Ca_desi_chr03	10656878	[T/C]	NA
Ca_Desi_Chr_3_10676528	Ca_desi_chr03	10676528	[T/A]	NA
Ca_Desi_Chr_3_10676573	Ca_desi_chr03	10676573	[A/G]	NA
Ca_Desi_Chr_3_10676579	Ca_desi_chr03	10676579	[A/G]	NA
Ca_Desi_Chr_3_10811646	Ca_desi_chr03	10811646	[T/G]	NA
Ca_Desi_Chr_3_10812132	Ca_desi_chr03	10812132	[T/C]	NA
Ca_Desi_Chr_3_10881091	Ca_desi_chr03	10881091	[A/G]	NA
Ca_Desi_Chr_3_11018969	Ca_desi_chr03	11018969	[A/G]	NA
Ca_Desi_Chr_3_11018974	Ca_desi_chr03	11018974	[T/C]	NA
Ca_Desi_Chr_3_11018981	Ca_desi_chr03	11018981	[T/C]	NA

SNP IDs	Chromosomes	Physical positions (bp)	SNPs	NCBI SNPdb accession IDs (NCBI_ss#)
Ca_Desi_Chr_3_11018982	Ca_desi_chr03	11018982	[C/T]	NA
Ca_Desi_Chr_3_11018990	Ca_desi_chr03	11018990	[T/G]	NA
Ca_Desi_Chr_3_11018998	Ca_desi_chr03	11018998	[A/C]	NA
Ca_Desi_Chr_3_11019001	Ca_desi_chr03	11019001	[T/C]	NA
Ca_Desi_Chr_3_11019823	Ca_desi_chr03	11019823	[T/G]	NA
Ca_Desi_Chr_3_11019824	Ca_desi_chr03	11019824	[A/G]	NA
Ca_Desi_Chr_3_11051027	Ca_desi_chr03	11051027	[G/A]	NA
Ca_Desi_Chr_3_11051028	Ca_desi_chr03	11051028	[A/T]	NA
Ca_Desi_Chr_3_11051029	Ca_desi_chr03	11051029	[G/C]	NA
Ca_Desi_Chr_3_11051030	Ca_desi_chr03	11051030	[G/T]	NA
Ca_Desi_Chr_3_11064390	Ca_desi_chr03	11064390	[A/G]	NA
Ca_Desi_Chr_3_11071524	Ca_desi_chr03	11071524	[C/T]	NA
Ca_Desi_Chr_3_11071527	Ca_desi_chr03	11071527	[C/T]	NA
Ca_Desi_Chr_3_11071530	Ca_desi_chr03	11071530	[A/T]	NA
Ca_Desi_Chr_3_11071533	Ca_desi_chr03	11071533	[C/T]	NA
Ca_Desi_Chr_3_11071541	Ca_desi_chr03	11071541	[A/C]	NA
Ca_Desi_Chr_3_11071549	Ca_desi_chr03	11071549	[A/G]	NA
Ca_Desi_Chr_3_11072965	Ca_desi_chr03	11072965	[A/G]	NA
Ca_Desi_Chr_3_11115658	Ca_desi_chr03	11115658	[A/G]	NA
Ca_Desi_Chr_3_11146467	Ca_desi_chr03	11146467	[A/C]	NA
Ca_Desi_Chr_3_11146473	Ca_desi_chr03	11146473	[A/C]	NA
Ca_Desi_Chr_3_11146477	Ca_desi_chr03	11146477	[T/C]	NA
Ca_Desi_Chr_3_11146483	Ca_desi_chr03	11146483	[T/C]	NA
Ca_Desi_Chr_3_11146484	Ca_desi_chr03	11146484	[A/C]	NA
Ca_Desi_Chr_3_11146590	Ca_desi_chr03	11146590	[C/A]	NA
Ca_Desi_Chr_3_11146591	Ca_desi_chr03	11146591	[T/G]	NA
Ca_Desi_Chr_3_11147755	Ca_desi_chr03	11147755	[A/G]	NA
Ca_Desi_Chr_3_11148303	Ca_desi_chr03	11148303	[T/C]	NA
Ca_Desi_Chr_3_11191033	Ca_desi_chr03	11191033	[A/G]	NA
Ca_Desi_Chr_3_11209903	Ca_desi_chr03	11209903	[A/T]	NA
Ca_Desi_Chr_3_11209929	Ca_desi_chr03	11209929	[T/G]	NA
Ca_Desi_Chr_3_11209934	Ca_desi_chr03	11209934	[T/C]	NA
Ca_Desi_Chr_3_11295878	Ca_desi_chr03	11295878	[T/C]	NA
Ca_Desi_Chr_3_11329775	Ca_desi_chr03	11329775	[A/T]	NA

SNP IDs	Chromosomes	Physical positions (bp)	SNPs	NCBI SNPdb accession IDs (NCBI_ss#)
Ca_Desi_Chr_3_11329776	Ca_desi_chr03	11329776	[T/G]	NA
Ca_Desi_Chr_3_11329777	Ca_desi_chr03	11329777	[C/T]	NA
Ca_Desi_Chr_3_11329778	Ca_desi_chr03	11329778	[T/G]	NA
Ca_Desi_Chr_3_11390981	Ca_desi_chr03	11390981	[T/C]	NA
Ca_Desi_Chr_3_11390985	Ca_desi_chr03	11390985	[A/G]	NA
Ca_Desi_Chr_3_11391277	Ca_desi_chr03	11391277	[T/G]	NA
Ca_Desi_Chr_3_11391310	Ca_desi_chr03	11391310	[A/G]	NA
Ca_Desi_Chr_3_11391311	Ca_desi_chr03	11391311	[A/G]	NA
Ca_Desi_Chr_3_11391312	Ca_desi_chr03	11391312	[A/G]	NA
Ca_Desi_Chr_3_11391313	Ca_desi_chr03	11391313	[A/G]	NA
Ca_Desi_Chr_3_11391314	Ca_desi_chr03	11391314	[A/G]	NA
Ca_Desi_Chr_3_11391315	Ca_desi_chr03	11391315	[A/G]	NA
Ca_Desi_Chr_3_11392020	Ca_desi_chr03	11392020	[T/C]	NA
Ca_Desi_Chr_3_11426559	Ca_desi_chr03	11426559	[T/C]	NA
Ca_Desi_Chr_3_11427980	Ca_desi_chr03	11427980	[T/G]	NA
Ca_Desi_Chr_3_11430507	Ca_desi_chr03	11430507	[T/C]	NA
Ca_Desi_Chr_3_11432250	Ca_desi_chr03	11432250	[C/A]	NA
Ca_Desi_Chr_3_11432510	Ca_desi_chr03	11432510	[T/C]	NA
Ca_Desi_Chr_3_11432514	Ca_desi_chr03	11432514	[T/G]	NA
Ca_Desi_Chr_3_11432519	Ca_desi_chr03	11432519	[T/C]	NA
Ca_Desi_Chr_3_11432534	Ca_desi_chr03	11432534	[T/C]	NA
Ca_Desi_Chr_3_11432572	Ca_desi_chr03	11432572	[G/T]	NA
Ca_Desi_Chr_3_11432577	Ca_desi_chr03	11432577	[A/G]	NA
Ca_Desi_Chr_3_11434575	Ca_desi_chr03	11434575	[A/C]	NA
Ca_Desi_Chr_3_11436131	Ca_desi_chr03	11436131	[A/C]	NA
Ca_Desi_Chr_3_11436133	Ca_desi_chr03	11436133	[A/C]	NA
Ca_Desi_Chr_3_11436135	Ca_desi_chr03	11436135	[A/G]	NA
Ca_Desi_Chr_3_11436165	Ca_desi_chr03	11436165	[T/C]	NA
Ca_Desi_Chr_3_11444578	Ca_desi_chr03	11444578	[T/C]	NA
Ca_Desi_Chr_3_11444582	Ca_desi_chr03	11444582	[A/C]	NA
Ca_Desi_Chr_3_11444586	Ca_desi_chr03	11444586	[T/A]	NA
Ca_Desi_Chr_3_11444596	Ca_desi_chr03	11444596	[A/C]	NA
Ca_Desi_Chr_3_11444599	Ca_desi_chr03	11444599	[G/C]	NA
Ca_Desi_Chr_3_11457601	Ca_desi_chr03	11457601	[T/C]	NA

SNP IDs	Chromosomes	Physical positions (bp)	SNPs	NCBI SNPdb accession IDs (NCBI_ss#)
Ca_Desi_Chr_3_11457608	Ca_desi_chr03	11457608	[T/G]	NA
Ca_Desi_Chr_3_11457620	Ca_desi_chr03	11457620	[T/C]	NA
Ca_Desi_Chr_3_11457628	Ca_desi_chr03	11457628	[T/C]	NA
Ca_Desi_Chr_3_11457633	Ca_desi_chr03	11457633	[C/G]	NA
Ca_Desi_Chr_3_11457635	Ca_desi_chr03	11457635	[A/G]	NA
Ca_Desi_Chr_3_11490712	Ca_desi_chr03	11490712	[C/G]	NA
Ca_Desi_Chr_3_11533440	Ca_desi_chr03	11533440	[A/T]	NA
Ca_Desi_Chr_3_11543200	Ca_desi_chr03	11543200	[T/G]	NA
Ca_Desi_Chr_3_11543223	Ca_desi_chr03	11543223	[T/G]	NA
Ca_Desi_Chr_3_11544159	Ca_desi_chr03	11544159	[A/G]	NA
Ca_Desi_Chr_3_11588299	Ca_desi_chr03	11588299	[A/C]	NA
Ca_Desi_Chr_3_11588324	Ca_desi_chr03	11588324	[A/C]	NA
Ca_Desi_Chr_3_11624201	Ca_desi_chr03	11624201	[A/C]	NA
Ca_Desi_Chr_3_11631332	Ca_desi_chr03	11631332	[A/C]	NA
Ca_Desi_Chr_3_11633970	Ca_desi_chr03	11633970	[A/T]	NA
Ca_Desi_Chr_3_11656929	Ca_desi_chr03	11656929	[T/C]	NA
Ca_Desi_Chr_3_11656937	Ca_desi_chr03	11656937	[A/G]	NA
Ca_Desi_Chr_3_11656942	Ca_desi_chr03	11656942	[G/T]	NA
Ca_Desi_Chr_3_11656944	Ca_desi_chr03	11656944	[T/C]	NA
Ca_Desi_Chr_3_11656947	Ca_desi_chr03	11656947	[A/G]	NA
Ca_Desi_Chr_3_11656952	Ca_desi_chr03	11656952	[T/G]	NA
Ca_Desi_Chr_3_11656958	Ca_desi_chr03	11656958	[A/G]	NA
Ca_Desi_Chr_3_11656968	Ca_desi_chr03	11656968	[A/G]	NA
Ca_Desi_Chr_3_11656970	Ca_desi_chr03	11656970	[T/C]	NA
Ca_Desi_Chr_3_11656981	Ca_desi_chr03	11656981	[A/G]	NA
Ca_Desi_Chr_3_11656989	Ca_desi_chr03	11656989	[T/C]	NA
Ca_Desi_Chr_3_11656997	Ca_desi_chr03	11656997	[A/G]	NA
Ca_Desi_Chr_3_11667188	Ca_desi_chr03	11667188	[T/C]	NA
Ca_Desi_Chr_3_11667230	Ca_desi_chr03	11667230	[T/C]	NA
Ca_Desi_Chr_3_11668974	Ca_desi_chr03	11668974	[T/C]	NA
Ca_Desi_Chr_3_11668989	Ca_desi_chr03	11668989	[T/C]	NA
Ca_Desi_Chr_3_11669023	Ca_desi_chr03	11669023	[A/G]	NA
Ca_Desi_Chr_3_11669956	Ca_desi_chr03	11669956	[T/C]	NA
Ca_Desi_Chr_3_11669959	Ca_desi_chr03	11669959	[T/C]	NA

SNP IDs	Chromosomes	Physical positions (bp)	SNPs	NCBI SNPdb accession IDs (NCBI_ss#)
Ca_Desi_Chr_3_11669967	Ca_desi_chr03	11669967	[T/C]	NA
Ca_Desi_Chr_3_11669983	Ca_desi_chr03	11669983	[A/C]	NA
Ca_Desi_Chr_3_11722008	Ca_desi_chr03	11722008	[T/G]	NA
Ca_Desi_Chr_3_11722095	Ca_desi_chr03	11722095	[T/G]	NA
Ca_Desi_Chr_3_11722125	Ca_desi_chr03	11722125	[T/G]	NA
Ca_Desi_Chr_3_11740564	Ca_desi_chr03	11740564	[T/C]	NA
Ca_Desi_Chr_3_11740588	Ca_desi_chr03	11740588	[A/G]	NA
Ca_Desi_Chr_3_11740660	Ca_desi_chr03	11740660	[T/C]	NA
Ca_Desi_Chr_3_11740671	Ca_desi_chr03	11740671	[A/G]	NA
Ca_Desi_Chr_3_11750910	Ca_desi_chr03	11750910	[C/A]	NA
Ca_Desi_Chr_3_11750911	Ca_desi_chr03	11750911	[A/G]	NA
Ca_Desi_Chr_3_11781905	Ca_desi_chr03	11781905	[T/G]	NA
Ca_Desi_Chr_3_11782791	Ca_desi_chr03	11782791	[A/T]	NA
Ca_Desi_Chr_3_11782818	Ca_desi_chr03	11782818	[T/A]	NA
Ca_Desi_Chr_3_11783339	Ca_desi_chr03	11783339	[A/T]	NA
Ca_Desi_Chr_3_11783341	Ca_desi_chr03	11783341	[C/A]	NA
Ca_Desi_Chr_3_11783344	Ca_desi_chr03	11783344	[G/A]	NA
Ca_Desi_Chr_3_11783353	Ca_desi_chr03	11783353	[A/G]	NA
Ca_Desi_Chr_3_11783359	Ca_desi_chr03	11783359	[T/C]	NA
Ca_Desi_Chr_3_11795727	Ca_desi_chr03	11795727	[A/G]	NA
Ca_Desi_Chr_3_11795733	Ca_desi_chr03	11795733	[T/C]	NA
Ca_Desi_Chr_3_11795758	Ca_desi_chr03	11795758	[A/G]	NA
Ca_Desi_Chr_3_11795771	Ca_desi_chr03	11795771	[T/G]	NA
Ca_Desi_Chr_3_11951018	Ca_desi_chr03	11951018	[T/G]	NA
Ca_Desi_Chr_3_11952326	Ca_desi_chr03	11952326	[T/G]	NA
Ca_Desi_Chr_3_11952338	Ca_desi_chr03	11952338	[T/G]	NA
Ca_Desi_Chr_3_11952353	Ca_desi_chr03	11952353	[T/G]	NA
Ca_Desi_Chr_3_11952371	Ca_desi_chr03	11952371	[A/G]	NA
Ca_Desi_Chr_3_11952374	Ca_desi_chr03	11952374	[A/G]	NA
Ca_Desi_Chr_3_11952416	Ca_desi_chr03	11952416	[A/T]	NA
Ca_Desi_Chr_3_11952431	Ca_desi_chr03	11952431	[A/G]	NA
Ca_Desi_Chr_3_11952440	Ca_desi_chr03	11952440	[T/G]	NA
Ca_Desi_Chr_3_11952443	Ca_desi_chr03	11952443	[T/G]	NA
Ca_Desi_Chr_3_11952463	Ca_desi_chr03	11952463	[A/G]	NA

SNP IDs	Chromosomes	Physical positions (bp)	SNPs	NCBI SNPdb accession IDs (NCBI_ss#)
Ca_Desi_Chr_3_11952492	Ca_desi_chr03	11952492	[T/G]	NA
Ca_Desi_Chr_3_11952517	Ca_desi_chr03	11952517	[A/C]	NA
Ca_Desi_Chr_3_11965650	Ca_desi_chr03	11965650	[T/G]	NA
Ca_Desi_Chr_3_11965669	Ca_desi_chr03	11965669	[T/A]	NA
Ca_Desi_Chr_3_12096021	Ca_desi_chr03	12096021	[A/T]	NA
Ca_Desi_Chr_3_12096051	Ca_desi_chr03	12096051	[A/G]	NA
Ca_Desi_Chr_3_12163289	Ca_desi_chr03	12163289	[A/C]	NA
Ca_Desi_Chr_3_12341044	Ca_desi_chr03	12341044	[A/G]	NA
Ca_Desi_Chr_3_12341064	Ca_desi_chr03	12341064	[T/C]	NA
Ca_Desi_Chr_3_12342074	Ca_desi_chr03	12342074	[T/G]	NA
Ca_Desi_Chr_3_12385705	Ca_desi_chr03	12385705	[T/C]	NA
Ca_Desi_Chr_3_12385707	Ca_desi_chr03	12385707	[A/C]	NA
Ca_Desi_Chr_3_12385723	Ca_desi_chr03	12385723	[T/C]	NA
Ca_Desi_Chr_3_12387388	Ca_desi_chr03	12387388	[A/C]	NA
Ca_Desi_Chr_3_12506621	Ca_desi_chr03	12506621	[T/C]	NA
Ca_Desi_Chr_3_12513130	Ca_desi_chr03	12513130	[A/T]	NA
Ca_Desi_Chr_3_12534470	Ca_desi_chr03	12534470	[T/C]	NA
Ca_Desi_Chr_3_12534491	Ca_desi_chr03	12534491	[T/G]	NA
Ca_Desi_Chr_3_12536000	Ca_desi_chr03	12536000	[C/A]	NA
Ca_Desi_Chr_3_12536003	Ca_desi_chr03	12536003	[A/C]	NA
Ca_Desi_Chr_3_12536005	Ca_desi_chr03	12536005	[T/G]	NA
Ca_Desi_Chr_3_12543780	Ca_desi_chr03	12543780	[T/C]	NA
Ca_Desi_Chr_3_12543781	Ca_desi_chr03	12543781	[T/C]	NA
Ca_Desi_Chr_3_12543812	Ca_desi_chr03	12543812	[A/G]	NA
Ca_Desi_Chr_3_12543826	Ca_desi_chr03	12543826	[T/C]	NA
Ca_Desi_Chr_3_12547098	Ca_desi_chr03	12547098	[T/G]	NA
Ca_Desi_Chr_3_12846630	Ca_desi_chr03	12846630	[C/T]	NA
Ca_Desi_Chr_3_12846631	Ca_desi_chr03	12846631	[T/C]	NA
Ca_Desi_Chr_3_12846632	Ca_desi_chr03	12846632	[T/C]	NA
Ca_Desi_Chr_3_12863760	Ca_desi_chr03	12863760	[G/A]	NA
Ca_Desi_Chr_3_12863764	Ca_desi_chr03	12863764	[C/T]	NA
Ca_Desi_Chr_3_12863779	Ca_desi_chr03	12863779	[T/C]	NA
Ca_Desi_Chr_3_12863789	Ca_desi_chr03	12863789	[C/A]	NA
Ca_Desi_Chr_3_12863810	Ca_desi_chr03	12863810	[C/T]	NA

SNP IDs	Chromosomes	Physical positions (bp)	SNPs	NCBI SNPdb accession IDs (NCBI_ss#)
Ca_Desi_Chr_3_12863812	Ca_desi_chr03	12863812	[C/T]	NA
Ca_Desi_Chr_3_12896089	Ca_desi_chr03	12896089	[T/C]	NA
Ca_Desi_Chr_3_12896100	Ca_desi_chr03	12896100	[A/G]	NA
Ca_Desi_Chr_3_12897384	Ca_desi_chr03	12897384	[G/T]	NA
Ca_Desi_Chr_3_12897498	Ca_desi_chr03	12897498	[T/C]	NA
Ca_Desi_Chr_3_12899996	Ca_desi_chr03	12899996	[T/A]	NA
Ca_Desi_Chr_3_12900039	Ca_desi_chr03	12900039	[A/C]	NA
Ca_Desi_Chr_3_13015032	Ca_desi_chr03	13015032	[A/G]	NA
Ca_Desi_Chr_3_13015036	Ca_desi_chr03	13015036	[T/C]	NA
Ca_Desi_Chr_3_13015045	Ca_desi_chr03	13015045	[T/C]	NA
Ca_Desi_Chr_3_13015076	Ca_desi_chr03	13015076	[A/T]	NA
Ca_Desi_Chr_3_13207072	Ca_desi_chr03	13207072	[T/G]	NA
Ca_Desi_Chr_3_13353108	Ca_desi_chr03	13353108	[T/C]	NA
Ca_Desi_Chr_3_13353122	Ca_desi_chr03	13353122	[A/G]	NA
Ca_Desi_Chr_3_13423139	Ca_desi_chr03	13423139	[A/T]	NA
Ca_Desi_Chr_3_13423147	Ca_desi_chr03	13423147	[T/C]	NA
Ca_Desi_Chr_3_13423161	Ca_desi_chr03	13423161	[T/A]	NA
Ca_Desi_Chr_3_13423162	Ca_desi_chr03	13423162	[A/T]	NA
Ca_Desi_Chr_3_13423164	Ca_desi_chr03	13423164	[T/C]	NA
Ca_Desi_Chr_3_13423175	Ca_desi_chr03	13423175	[T/C]	NA
Ca_Desi_Chr_3_13518038	Ca_desi_chr03	13518038	[A/C]	NA
Ca_Desi_Chr_3_13533180	Ca_desi_chr03	13533180	[T/C]	NA
Ca_Desi_Chr_3_13533191	Ca_desi_chr03	13533191	[T/C]	NA
Ca_Desi_Chr_3_13533219	Ca_desi_chr03	13533219	[T/G]	NA
Ca_Desi_Chr_3_13533221	Ca_desi_chr03	13533221	[T/G]	NA
Ca_Desi_Chr_3_13533224	Ca_desi_chr03	13533224	[T/G]	NA
Ca_Desi_Chr_3_13533234	Ca_desi_chr03	13533234	[T/C]	NA
Ca_Desi_Chr_3_13535547	Ca_desi_chr03	13535547	[A/T]	NA
Ca_Desi_Chr_3_13535548	Ca_desi_chr03	13535548	[T/G]	NA
Ca_Desi_Chr_3_13535549	Ca_desi_chr03	13535549	[C/T]	NA
Ca_Desi_Chr_3_13535550	Ca_desi_chr03	13535550	[T/G]	NA
Ca_Desi_Chr_3_13535696	Ca_desi_chr03	13535696	[A/G]	NA
Ca_Desi_Chr_3_13535715	Ca_desi_chr03	13535715	[A/C]	NA
Ca_Desi_Chr_3_13538576	Ca_desi_chr03	13538576	[A/C]	NA

SNP IDs	Chromosomes	Physical positions (bp)	SNPs	NCBI SNPdb accession IDs (NCBI_ss#)
Ca_Desi_Chr_3_13578505	Ca_desi_chr03	13578505	[T/G]	NA
Ca_Desi_Chr_3_13664230	Ca_desi_chr03	13664230	[T/C]	NA
Ca_Desi_Chr_3_13761986	Ca_desi_chr03	13761986	[A/G]	NA
Ca_Desi_Chr_3_13762005	Ca_desi_chr03	13762005	[A/G]	NA
Ca_Desi_Chr_3_13762011	Ca_desi_chr03	13762011	[A/G]	NA
Ca_Desi_Chr_3_13762031	Ca_desi_chr03	13762031	[A/G]	NA
Ca_Desi_Chr_3_13762033	Ca_desi_chr03	13762033	[A/G]	NA
Ca_Desi_Chr_3_13762041	Ca_desi_chr03	13762041	[T/C]	NA
Ca_Desi_Chr_3_13762042	Ca_desi_chr03	13762042	[A/G]	NA
Ca_Desi_Chr_3_13823885	Ca_desi_chr03	13823885	[T/C]	NA
Ca_Desi_Chr_3_13825432	Ca_desi_chr03	13825432	[T/G]	NA
Ca_Desi_Chr_3_13829504	Ca_desi_chr03	13829504	[T/C]	NA
Ca_Desi_Chr_3_13829505	Ca_desi_chr03	13829505	[G/T]	NA
Ca_Desi_Chr_3_13841844	Ca_desi_chr03	13841844	[T/C]	NA
Ca_Desi_Chr_3_13841848	Ca_desi_chr03	13841848	[T/C]	NA
Ca_Desi_Chr_3_13841860	Ca_desi_chr03	13841860	[T/C]	NA
Ca_Desi_Chr_3_13841862	Ca_desi_chr03	13841862	[A/C]	NA
Ca_Desi_Chr_3_13841866	Ca_desi_chr03	13841866	[T/C]	NA
Ca_Desi_Chr_3_13841868	Ca_desi_chr03	13841868	[A/C]	NA
Ca_Desi_Chr_3_13892233	Ca_desi_chr03	13892233	[A/C]	NA
Ca_Desi_Chr_3_13892255	Ca_desi_chr03	13892255	[T/C]	NA
Ca_Desi_Chr_3_13892260	Ca_desi_chr03	13892260	[T/C]	NA
Ca_Desi_Chr_3_13894609	Ca_desi_chr03	13894609	[T/G]	NA
Ca_Desi_Chr_3_13993896	Ca_desi_chr03	13993896	[T/C]	NA
Ca_Desi_Chr_3_14005543	Ca_desi_chr03	14005543	[G/C]	NA
Ca_Desi_Chr_3_14006868	Ca_desi_chr03	14006868	[G/A]	NA
Ca_Desi_Chr_3_14137865	Ca_desi_chr03	14137865	[C/A]	NA
Ca_Desi_Chr_3_14172683	Ca_desi_chr03	14172683	[T/G]	NA
Ca_Desi_Chr_3_14172702	Ca_desi_chr03	14172702	[A/G]	NA
Ca_Desi_Chr_3_14619693	Ca_desi_chr03	14619693	[A/G]	NA
Ca_Desi_Chr_3_14732868	Ca_desi_chr03	14732868	[A/T]	NA
Ca_Desi_Chr_3_14733058	Ca_desi_chr03	14733058	[T/C]	NA
Ca_Desi_Chr_3_14817890	Ca_desi_chr03	14817890	[A/C]	NA
Ca_Desi_Chr_3_14860658	Ca_desi_chr03	14860658	[A/C]	NA

SNP IDs	Chromosomes	Physical positions (bp)	SNPs	NCBI SNPdb accession IDs (NCBI_ss#)
Ca_Desi_Chr_3_14927117	Ca_desi_chr03	14927117	[A/G]	NA
Ca_Desi_Chr_3_14927170	Ca_desi_chr03	14927170	[C/G]	NA
Ca_Desi_Chr_3_14933824	Ca_desi_chr03	14933824	[T/C]	NA
Ca_Desi_Chr_3_14933842	Ca_desi_chr03	14933842	[A/G]	NA
Ca_Desi_Chr_3_14933844	Ca_desi_chr03	14933844	[T/G]	NA
Ca_Desi_Chr_3_14936353	Ca_desi_chr03	14936353	[A/G]	NA
Ca_Desi_Chr_3_15070440	Ca_desi_chr03	15070440	[T/G]	NA
Ca_Desi_Chr_3_15070457	Ca_desi_chr03	15070457	[T/G]	NA
Ca_Desi_Chr_3_15103597	Ca_desi_chr03	15103597	[A/G]	NA
Ca_Desi_Chr_3_15111143	Ca_desi_chr03	15111143	[T/A]	NA
Ca_Desi_Chr_3_15114667	Ca_desi_chr03	15114667	[A/C]	NA
Ca_Desi_Chr_3_15115114	Ca_desi_chr03	15115114	[A/G]	NA
Ca_Desi_Chr_3_15115921	Ca_desi_chr03	15115921	[T/G]	NA
Ca_Desi_Chr_3_15134443	Ca_desi_chr03	15134443	[T/C]	NA
Ca_Desi_Chr_3_15134476	Ca_desi_chr03	15134476	[A/C]	NA
Ca_Desi_Chr_3_15134798	Ca_desi_chr03	15134798	[A/T]	NA
Ca_Desi_Chr_3_15280200	Ca_desi_chr03	15280200	[T/G]	NA
Ca_Desi_Chr_3_15280210	Ca_desi_chr03	15280210	[A/T]	NA
Ca_Desi_Chr_3_15281781	Ca_desi_chr03	15281781	[T/C]	NA
Ca_Desi_Chr_3_15332029	Ca_desi_chr03	15332029	[T/A]	NA
Ca_Desi_Chr_3_15332140	Ca_desi_chr03	15332140	[G/A]	NA
Ca_Desi_Chr_3_15332490	Ca_desi_chr03	15332490	[C/G]	NA
Ca_Desi_Chr_3_15332493	Ca_desi_chr03	15332493	[C/A]	NA
Ca_Desi_Chr_3_15332497	Ca_desi_chr03	15332497	[C/T]	NA
Ca_Desi_Chr_3_15332593	Ca_desi_chr03	15332593	[A/G]	NA
Ca_Desi_Chr_3_15376333	Ca_desi_chr03	15376333	[A/C]	NA
Ca_Desi_Chr_3_15386028	Ca_desi_chr03	15386028	[C/A]	NA
Ca_Desi_Chr_3_15386029	Ca_desi_chr03	15386029	[A/G]	NA
Ca_Desi_Chr_3_15386093	Ca_desi_chr03	15386093	[A/C]	NA
Ca_Desi_Chr_3_15399290	Ca_desi_chr03	15399290	[A/G]	NA
Ca_Desi_Chr_3_15446493	Ca_desi_chr03	15446493	[T/A]	NA
Ca_Desi_Chr_3_15449371	Ca_desi_chr03	15449371	[A/G]	NA
Ca_Desi_Chr_3_15449374	Ca_desi_chr03	15449374	[A/T]	NA
Ca_Desi_Chr_3_15459723	Ca_desi_chr03	15459723	[A/C]	NA

SNP IDs	Chromosomes	Physical positions (bp)	SNPs	NCBI SNPdb accession IDs (NCBI_ss#)
Ca_Desi_Chr_3_15459742	Ca_desi_chr03	15459742	[A/T]	NA
Ca_Desi_Chr_3_15459759	Ca_desi_chr03	15459759	[A/C]	NA
Ca_Desi_Chr_3_15497381	Ca_desi_chr03	15497381	[A/C]	NA
Ca_Desi_Chr_3_15497425	Ca_desi_chr03	15497425	[A/G]	NA
Ca_Desi_Chr_3_15497431	Ca_desi_chr03	15497431	[A/C]	NA
Ca_Desi_Chr_3_15502436	Ca_desi_chr03	15502436	[T/C]	NA
Ca_Desi_Chr_3_15502457	Ca_desi_chr03	15502457	[T/C]	NA
Ca_Desi_Chr_3_15502468	Ca_desi_chr03	15502468	[T/C]	NA
Ca_Desi_Chr_3_15502478	Ca_desi_chr03	15502478	[A/C]	NA
Ca_Desi_Chr_3_15502480	Ca_desi_chr03	15502480	[T/C]	NA
Ca_Desi_Chr_3_15502487	Ca_desi_chr03	15502487	[T/C]	NA
Ca_Desi_Chr_3_15875169	Ca_desi_chr03	15875169	[A/G]	NA
Ca_Desi_Chr_3_15875193	Ca_desi_chr03	15875193	[T/C]	NA
Ca_Desi_Chr_3_15875221	Ca_desi_chr03	15875221	[A/C]	NA
Ca_Desi_Chr_3_15875231	Ca_desi_chr03	15875231	[A/G]	NA
Ca_Desi_Chr_3_15877280	Ca_desi_chr03	15877280	[A/C]	NA
Ca_Desi_Chr_3_15991384	Ca_desi_chr03	15991384	[T/C]	NA
Ca_Desi_Chr_3_15991386	Ca_desi_chr03	15991386	[G/A]	NA
Ca_Desi_Chr_3_15991400	Ca_desi_chr03	15991400	[A/G]	NA
Ca_Desi_Chr_3_15991425	Ca_desi_chr03	15991425	[A/G]	NA
Ca_Desi_Chr_3_15991437	Ca_desi_chr03	15991437	[T/A]	NA
Ca_Desi_Chr_3_16022419	Ca_desi_chr03	16022419	[T/C]	NA
Ca_Desi_Chr_3_16050997	Ca_desi_chr03	16050997	[T/C]	NA
Ca_Desi_Chr_3_16051042	Ca_desi_chr03	16051042	[T/C]	NA
Ca_Desi_Chr_3_16051284	Ca_desi_chr03	16051284	[T/C]	NA
Ca_Desi_Chr_3_16051287	Ca_desi_chr03	16051287	[T/C]	NA
Ca_Desi_Chr_3_16051314	Ca_desi_chr03	16051314	[T/C]	NA
Ca_Desi_Chr_3_16051338	Ca_desi_chr03	16051338	[T/C]	NA
Ca_Desi_Chr_3_16104847	Ca_desi_chr03	16104847	[T/A]	NA
Ca_Desi_Chr_3_16104854	Ca_desi_chr03	16104854	[A/G]	NA
Ca_Desi_Chr_3_16104860	Ca_desi_chr03	16104860	[T/C]	NA
Ca_Desi_Chr_3_16108704	Ca_desi_chr03	16108704	[A/G]	NA
Ca_Desi_Chr_3_16116725	Ca_desi_chr03	16116725	[A/C]	NA
Ca_Desi_Chr_3_16136964	Ca_desi_chr03	16136964	[A/C]	NA

SNP IDs	Chromosomes	Physical positions (bp)	SNPs	NCBI SNPdb accession IDs (NCBI_ss#)
Ca_Desi_Chr_3_16181581	Ca_desi_chr03	16181581	[T/C]	NA
Ca_Desi_Chr_3_16185209	Ca_desi_chr03	16185209	[G/T]	NA
Ca_Desi_Chr_3_16185211	Ca_desi_chr03	16185211	[G/T]	NA
Ca_Desi_Chr_3_16188628	Ca_desi_chr03	16188628	[A/G]	NA
Ca_Desi_Chr_3_16188642	Ca_desi_chr03	16188642	[T/C]	NA
Ca_Desi_Chr_3_16188647	Ca_desi_chr03	16188647	[A/T]	NA
Ca_Desi_Chr_3_16188660	Ca_desi_chr03	16188660	[T/G]	NA
Ca_Desi_Chr_3_16188666	Ca_desi_chr03	16188666	[T/G]	NA
Ca_Desi_Chr_3_16188667	Ca_desi_chr03	16188667	[A/C]	NA
Ca_Desi_Chr_3_16209367	Ca_desi_chr03	16209367	[A/T]	NA
Ca_Desi_Chr_3_16209405	Ca_desi_chr03	16209405	[A/T]	NA
Ca_Desi_Chr_3_16209416	Ca_desi_chr03	16209416	[A/G]	NA
Ca_Desi_Chr_3_16209423	Ca_desi_chr03	16209423	[A/C]	NA
Ca_Desi_Chr_3_16209429	Ca_desi_chr03	16209429	[T/C]	NA
Ca_Desi_Chr_3_16209436	Ca_desi_chr03	16209436	[T/C]	NA
Ca_Desi_Chr_3_16258540	Ca_desi_chr03	16258540	[T/C]	NA
Ca_Desi_Chr_3_16337223	Ca_desi_chr03	16337223	[A/G]	NA
Ca_Desi_Chr_3_16337248	Ca_desi_chr03	16337248	[A/G]	NA
Ca_Desi_Chr_3_16346395	Ca_desi_chr03	16346395	[C/T]	NA
Ca_Desi_Chr_3_16429235	Ca_desi_chr03	16429235	[T/G]	NA
Ca_Desi_Chr_3_16429257	Ca_desi_chr03	16429257	[A/C]	NA
Ca_Desi_Chr_3_16429260	Ca_desi_chr03	16429260	[A/C]	NA
Ca_Desi_Chr_3_16546869	Ca_desi_chr03	16546869	[T/G]	NA
Ca_Desi_Chr_3_16546893	Ca_desi_chr03	16546893	[A/G]	NA
Ca_Desi_Chr_3_16546905	Ca_desi_chr03	16546905	[T/G]	NA
Ca_Desi_Chr_3_16546908	Ca_desi_chr03	16546908	[A/C]	NA
Ca_Desi_Chr_3_16579001	Ca_desi_chr03	16579001	[A/T]	NA
Ca_Desi_Chr_3_16579024	Ca_desi_chr03	16579024	[A/G]	NA
Ca_Desi_Chr_3_16582095	Ca_desi_chr03	16582095	[T/G]	NA
Ca_Desi_Chr_3_16582117	Ca_desi_chr03	16582117	[A/T]	NA
Ca_Desi_Chr_3_16590579	Ca_desi_chr03	16590579	[C/A]	NA
Ca_Desi_Chr_3_16590581	Ca_desi_chr03	16590581	[C/A]	NA
Ca_Desi_Chr_3_16590582	Ca_desi_chr03	16590582	[C/T]	NA
Ca_Desi_Chr_3_16620455	Ca_desi_chr03	16620455	[A/C]	NA

SNP IDs	Chromosomes	Physical positions (bp)	SNPs	NCBI SNPdb accession IDs (NCBI_ss#)
Ca_Desi_Chr_3_16650411	Ca_desi_chr03	16650411	[T/G]	NA
Ca_Desi_Chr_3_16712047	Ca_desi_chr03	16712047	[T/G]	NA
Ca_Desi_Chr_3_16765694	Ca_desi_chr03	16765694	[C/G]	NA
Ca_Desi_Chr_3_16765695	Ca_desi_chr03	16765695	[G/A]	NA
Ca_Desi_Chr_3_16765697	Ca_desi_chr03	16765697	[T/C]	NA
Ca_Desi_Chr_3_16765698	Ca_desi_chr03	16765698	[G/T]	NA
Ca_Desi_Chr_3_16786497	Ca_desi_chr03	16786497	[T/G]	NA
Ca_Desi_Chr_3_16829075	Ca_desi_chr03	16829075	[G/C]	NA
Ca_Desi_Chr_3_16829079	Ca_desi_chr03	16829079	[T/A]	NA
Ca_Desi_Chr_3_16829122	Ca_desi_chr03	16829122	[C/G]	NA
Ca_Desi_Chr_3_16829185	Ca_desi_chr03	16829185	[A/C]	NA
Ca_Desi_Chr_3_16868994	Ca_desi_chr03	16868994	[A/C]	NA
Ca_Desi_Chr_3_16885690	Ca_desi_chr03	16885690	[T/G]	NA
Ca_Desi_Chr_3_16885755	Ca_desi_chr03	16885755	[A/G]	NA
Ca_Desi_Chr_3_16925658	Ca_desi_chr03	16925658	[A/G]	NA
Ca_Desi_Chr_3_16925687	Ca_desi_chr03	16925687	[A/C]	NA
Ca_Desi_Chr_3_16925701	Ca_desi_chr03	16925701	[T/C]	NA
Ca_Desi_Chr_3_17011085	Ca_desi_chr03	17011085	[T/C]	NA
Ca_Desi_Chr_3_17065579	Ca_desi_chr03	17065579	[A/G]	NA
Ca_Desi_Chr_3_17065582	Ca_desi_chr03	17065582	[T/G]	NA
Ca_Desi_Chr_3_17065613	Ca_desi_chr03	17065613	[A/G]	NA
Ca_Desi_Chr_3_17065616	Ca_desi_chr03	17065616	[T/G]	NA
Ca_Desi_Chr_3_17065622	Ca_desi_chr03	17065622	[A/G]	NA
Ca_Desi_Chr_3_17088304	Ca_desi_chr03	17088304	[T/A]	NA
Ca_Desi_Chr_3_17088308	Ca_desi_chr03	17088308	[T/C]	NA
Ca_Desi_Chr_3_17088317	Ca_desi_chr03	17088317	[A/G]	NA
Ca_Desi_Chr_3_17088323	Ca_desi_chr03	17088323	[A/G]	NA
Ca_Desi_Chr_3_17088325	Ca_desi_chr03	17088325	[A/G]	NA
Ca_Desi_Chr_3_17088328	Ca_desi_chr03	17088328	[A/G]	NA
Ca_Desi_Chr_3_17088349	Ca_desi_chr03	17088349	[T/A]	NA
Ca_Desi_Chr_3_17096871	Ca_desi_chr03	17096871	[A/G]	NA
Ca_Desi_Chr_3_17135033	Ca_desi_chr03	17135033	[A/C]	NA
Ca_Desi_Chr_3_17135048	Ca_desi_chr03	17135048	[A/G]	NA
Ca_Desi_Chr_3_17138109	Ca_desi_chr03	17138109	[C/A]	NA

SNP IDs	Chromosomes	Physical positions (bp)	SNPs	NCBI SNPdb accession IDs (NCBI_ss#)
Ca_Desi_Chr_3_17138110	Ca_desi_chr03	17138110	[T/G]	NA
Ca_Desi_Chr_3_17138111	Ca_desi_chr03	17138111	[T/A]	NA
Ca_Desi_Chr_3_17138112	Ca_desi_chr03	17138112	[G/T]	NA
Ca_Desi_Chr_3_17227479	Ca_desi_chr03	17227479	[A/G]	NA
Ca_Desi_Chr_3_17227518	Ca_desi_chr03	17227518	[A/C]	NA
Ca_Desi_Chr_3_17227520	Ca_desi_chr03	17227520	[A/C]	NA
Ca_Desi_Chr_3_17227527	Ca_desi_chr03	17227527	[A/G]	NA
Ca_Desi_Chr_3_17296688	Ca_desi_chr03	17296688	[T/C]	NA
Ca_Desi_Chr_3_17296711	Ca_desi_chr03	17296711	[G/A]	NA
Ca_Desi_Chr_3_17646522	Ca_desi_chr03	17646522	[T/C]	NA
Ca_Desi_Chr_3_17646527	Ca_desi_chr03	17646527	[T/C]	NA
Ca_Desi_Chr_3_17646542	Ca_desi_chr03	17646542	[A/G]	NA
Ca_Desi_Chr_3_17646549	Ca_desi_chr03	17646549	[A/G]	NA
Ca_Desi_Chr_3_17664144	Ca_desi_chr03	17664144	[A/G]	NA
Ca_Desi_Chr_3_17732927	Ca_desi_chr03	17732927	[A/G]	NA
Ca_Desi_Chr_3_17737216	Ca_desi_chr03	17737216	[G/A]	NA
Ca_Desi_Chr_3_17741417	Ca_desi_chr03	17741417	[A/T]	NA
Ca_Desi_Chr_3_17741418	Ca_desi_chr03	17741418	[T/A]	NA
Ca_Desi_Chr_3_17792610	Ca_desi_chr03	17792610	[A/C]	NA
Ca_Desi_Chr_3_17792649	Ca_desi_chr03	17792649	[A/C]	NA
Ca_Desi_Chr_3_17792663	Ca_desi_chr03	17792663	[A/C]	NA
Ca_Desi_Chr_3_17818902	Ca_desi_chr03	17818902	[T/G]	NA
Ca_Desi_Chr_3_17818917	Ca_desi_chr03	17818917	[T/G]	NA
Ca_Desi_Chr_3_17835717	Ca_desi_chr03	17835717	[A/C]	NA
Ca_Desi_Chr_3_17835719	Ca_desi_chr03	17835719	[T/C]	NA
Ca_Desi_Chr_3_17835724	Ca_desi_chr03	17835724	[T/C]	NA
Ca_Desi_Chr_3_17835729	Ca_desi_chr03	17835729	[A/C]	NA
Ca_Desi_Chr_3_17835731	Ca_desi_chr03	17835731	[T/G]	NA
Ca_Desi_Chr_3_17835767	Ca_desi_chr03	17835767	[T/C]	NA
Ca_Desi_Chr_3_17839934	Ca_desi_chr03	17839934	[T/A]	NA
Ca_Desi_Chr_3_17839978	Ca_desi_chr03	17839978	[T/A]	NA
Ca_Desi_Chr_3_17839999	Ca_desi_chr03	17839999	[A/G]	NA
Ca_Desi_Chr_3_17840024	Ca_desi_chr03	17840024	[T/C]	NA
Ca_Desi_Chr_3_17840040	Ca_desi_chr03	17840040	[A/C]	NA

SNP IDs	Chromosomes	Physical positions (bp)	SNPs	NCBI SNPdb accession IDs (NCBI_ss#)
Ca_Desi_Chr_3_17840042	Ca_desi_chr03	17840042	[T/C]	NA
Ca_Desi_Chr_3_17840054	Ca_desi_chr03	17840054	[T/C]	NA
Ca_Desi_Chr_3_17840063	Ca_desi_chr03	17840063	[A/G]	NA
Ca_Desi_Chr_3_17840066	Ca_desi_chr03	17840066	[T/C]	NA
Ca_Desi_Chr_3_17848319	Ca_desi_chr03	17848319	[T/C]	NA
Ca_Desi_Chr_3_17848395	Ca_desi_chr03	17848395	[T/G]	NA
Ca_Desi_Chr_3_17848919	Ca_desi_chr03	17848919	[A/G]	NA
Ca_Desi_Chr_3_17848950	Ca_desi_chr03	17848950	[T/C]	NA
Ca_Desi_Chr_3_17853432	Ca_desi_chr03	17853432	[T/A]	NA
Ca_Desi_Chr_3_17853448	Ca_desi_chr03	17853448	[T/A]	NA
Ca_Desi_Chr_3_17854002	Ca_desi_chr03	17854002	[T/G]	NA
Ca_Desi_Chr_3_17854023	Ca_desi_chr03	17854023	[T/G]	NA
Ca_Desi_Chr_3_17854146	Ca_desi_chr03	17854146	[T/G]	NA
Ca_Desi_Chr_3_17855204	Ca_desi_chr03	17855204	[A/G]	NA
Ca_Desi_Chr_3_17855245	Ca_desi_chr03	17855245	[A/G]	NA
Ca_Desi_Chr_3_17895424	Ca_desi_chr03	17895424	[T/C]	NA
Ca_Desi_Chr_3_17952334	Ca_desi_chr03	17952334	[T/G]	NA
Ca_Desi_Chr_3_17952564	Ca_desi_chr03	17952564	[T/C]	NA
Ca_Desi_Chr_3_17966694	Ca_desi_chr03	17966694	[A/C]	NA
Ca_Desi_Chr_3_17975273	Ca_desi_chr03	17975273	[C/A]	NA
Ca_Desi_Chr_3_17976107	Ca_desi_chr03	17976107	[T/G]	NA
Ca_Desi_Chr_3_17976111	Ca_desi_chr03	17976111	[T/C]	NA
Ca_Desi_Chr_3_17976132	Ca_desi_chr03	17976132	[A/G]	NA
Ca_Desi_Chr_3_17989589	Ca_desi_chr03	17989589	[T/G]	NA
Ca_Desi_Chr_3_18017147	Ca_desi_chr03	18017147	[T/G]	NA
Ca_Desi_Chr_3_18017151	Ca_desi_chr03	18017151	[A/C]	NA
Ca_Desi_Chr_3_18017344	Ca_desi_chr03	18017344	[T/A]	NA
Ca_Desi_Chr_3_18045434	Ca_desi_chr03	18045434	[A/C]	NA
Ca_Desi_Chr_3_18046638	Ca_desi_chr03	18046638	[A/C]	NA
Ca_Desi_Chr_3_18053804	Ca_desi_chr03	18053804	[T/G]	NA
Ca_Desi_Chr_3_18101790	Ca_desi_chr03	18101790	[T/G]	NA
Ca_Desi_Chr_3_18101916	Ca_desi_chr03	18101916	[A/G]	NA
Ca_Desi_Chr_3_18101946	Ca_desi_chr03	18101946	[A/C]	NA
Ca_Desi_Chr_3_18102043	Ca_desi_chr03	18102043	[T/C]	NA

SNP IDs	Chromosomes	Physical positions (bp)	SNPs	NCBI SNPdb accession IDs (NCBI_ss#)
Ca_Desi_Chr_3_18102072	Ca_desi_chr03	18102072	[T/G]	NA
Ca_Desi_Chr_3_18102085	Ca_desi_chr03	18102085	[T/C]	NA
Ca_Desi_Chr_3_18102087	Ca_desi_chr03	18102087	[A/G]	NA
Ca_Desi_Chr_3_18121201	Ca_desi_chr03	18121201	[T/C]	NA
Ca_Desi_Chr_3_18121210	Ca_desi_chr03	18121210	[T/G]	NA
Ca_Desi_Chr_3_18121247	Ca_desi_chr03	18121247	[T/A]	NA
Ca_Desi_Chr_3_18121264	Ca_desi_chr03	18121264	[A/C]	NA
Ca_Desi_Chr_3_18121309	Ca_desi_chr03	18121309	[A/C]	NA
Ca_Desi_Chr_3_18171145	Ca_desi_chr03	18171145	[A/T]	NA
Ca_Desi_Chr_3_18171146	Ca_desi_chr03	18171146	[A/C]	NA
Ca_Desi_Chr_3_18192692	Ca_desi_chr03	18192692	[A/C]	NA
Ca_Desi_Chr_3_18192696	Ca_desi_chr03	18192696	[A/C]	NA
Ca_Desi_Chr_3_18192713	Ca_desi_chr03	18192713	[T/C]	NA
Ca_Desi_Chr_3_18203721	Ca_desi_chr03	18203721	[T/G]	NA
Ca_Desi_Chr_3_18203727	Ca_desi_chr03	18203727	[A/C]	NA
Ca_Desi_Chr_3_18272990	Ca_desi_chr03	18272990	[T/A]	NA
Ca_Desi_Chr_3_18272991	Ca_desi_chr03	18272991	[A/T]	NA
Ca_Desi_Chr_3_18273003	Ca_desi_chr03	18273003	[T/G]	NA
Ca_Desi_Chr_3_18273006	Ca_desi_chr03	18273006	[A/G]	NA
Ca_Desi_Chr_3_18273015	Ca_desi_chr03	18273015	[T/G]	NA
Ca_Desi_Chr_3_18273023	Ca_desi_chr03	18273023	[T/G]	NA
Ca_Desi_Chr_3_18273059	Ca_desi_chr03	18273059	[T/G]	NA
Ca_Desi_Chr_3_18273103	Ca_desi_chr03	18273103	[C/A]	NA
Ca_Desi_Chr_3_18273114	Ca_desi_chr03	18273114	[T/G]	NA
Ca_Desi_Chr_3_18299751	Ca_desi_chr03	18299751	[A/G]	NA
Ca_Desi_Chr_3_18299753	Ca_desi_chr03	18299753	[C/T]	NA
Ca_Desi_Chr_3_18299757	Ca_desi_chr03	18299757	[T/C]	NA
Ca_Desi_Chr_3_18305209	Ca_desi_chr03	18305209	[A/T]	NA
Ca_Desi_Chr_3_18305215	Ca_desi_chr03	18305215	[T/C]	NA
Ca_Desi_Chr_3_18305219	Ca_desi_chr03	18305219	[T/C]	NA
Ca_Desi_Chr_3_18305230	Ca_desi_chr03	18305230	[A/G]	NA
Ca_Desi_Chr_3_18305257	Ca_desi_chr03	18305257	[A/G]	NA
Ca_Desi_Chr_3_18305259	Ca_desi_chr03	18305259	[T/C]	NA
Ca_Desi_Chr_3_18305263	Ca_desi_chr03	18305263	[T/A]	NA

SNP IDs	Chromosomes	Physical positions (bp)	SNPs	NCBI SNPdb accession IDs (NCBI_ss#)
Ca_Desi_Chr_3_18370545	Ca_desi_chr03	18370545	[T/C]	NA
Ca_Desi_Chr_3_18370575	Ca_desi_chr03	18370575	[T/C]	NA
Ca_Desi_Chr_3_18370609	Ca_desi_chr03	18370609	[A/C]	NA
Ca_Desi_Chr_3_18378861	Ca_desi_chr03	18378861	[T/C]	NA
Ca_Desi_Chr_3_18378862	Ca_desi_chr03	18378862	[C/G]	NA
Ca_Desi_Chr_3_18439917	Ca_desi_chr03	18439917	[G/T]	NA
Ca_Desi_Chr_3_18440126	Ca_desi_chr03	18440126	[T/A]	NA
Ca_Desi_Chr_3_18440144	Ca_desi_chr03	18440144	[A/G]	NA
Ca_Desi_Chr_3_18440185	Ca_desi_chr03	18440185	[A/G]	NA
Ca_Desi_Chr_3_18440223	Ca_desi_chr03	18440223	[A/C]	NA
Ca_Desi_Chr_3_18440280	Ca_desi_chr03	18440280	[A/C]	NA
Ca_Desi_Chr_3_18440310	Ca_desi_chr03	18440310	[A/G]	NA
Ca_Desi_Chr_3_18440952	Ca_desi_chr03	18440952	[A/C]	NA
Ca_Desi_Chr_3_18440955	Ca_desi_chr03	18440955	[A/C]	NA
Ca_Desi_Chr_3_18448780	Ca_desi_chr03	18448780	[A/C]	NA
Ca_Desi_Chr_3_18449839	Ca_desi_chr03	18449839	[T/G]	NA
Ca_Desi_Chr_3_18457058	Ca_desi_chr03	18457058	[A/G]	NA
Ca_Desi_Chr_3_18457059	Ca_desi_chr03	18457059	[A/G]	NA
Ca_Desi_Chr_3_18457060	Ca_desi_chr03	18457060	[A/G]	NA
Ca_Desi_Chr_3_18489032	Ca_desi_chr03	18489032	[C/A]	NA
Ca_Desi_Chr_3_18500277	Ca_desi_chr03	18500277	[C/G]	NA
Ca_Desi_Chr_3_18530331	Ca_desi_chr03	18530331	[A/T]	NA
Ca_Desi_Chr_3_18530349	Ca_desi_chr03	18530349	[A/C]	NA
Ca_Desi_Chr_3_18530555	Ca_desi_chr03	18530555	[C/T]	NA
Ca_Desi_Chr_3_18530599	Ca_desi_chr03	18530599	[A/T]	NA
Ca_Desi_Chr_3_18543482	Ca_desi_chr03	18543482	[C/T]	NA
Ca_Desi_Chr_3_18543500	Ca_desi_chr03	18543500	[G/T]	NA
Ca_Desi_Chr_3_18594788	Ca_desi_chr03	18594788	[T/A]	NA
Ca_Desi_Chr_3_18594797	Ca_desi_chr03	18594797	[T/G]	NA
Ca_Desi_Chr_3_18612095	Ca_desi_chr03	18612095	[T/G]	NA
Ca_Desi_Chr_3_18668485	Ca_desi_chr03	18668485	[T/C]	NA
Ca_Desi_Chr_3_18702849	Ca_desi_chr03	18702849	[T/C]	NA
Ca_Desi_Chr_3_18702949	Ca_desi_chr03	18702949	[T/G]	NA
Ca_Desi_Chr_3_18702950	Ca_desi_chr03	18702950	[C/A]	NA

SNP IDs	Chromosomes	Physical positions (bp)	SNPs	NCBI SNPdb accession IDs (NCBI_ss#)
Ca_Desi_Chr_3_18702951	Ca_desi_chr03	18702951	[A/C]	NA
Ca_Desi_Chr_3_18702952	Ca_desi_chr03	18702952	[C/G]	NA
Ca_Desi_Chr_3_18702960	Ca_desi_chr03	18702960	[C/A]	NA
Ca_Desi_Chr_3_18703047	Ca_desi_chr03	18703047	[A/T]	NA
Ca_Desi_Chr_3_18703050	Ca_desi_chr03	18703050	[A/C]	NA
Ca_Desi_Chr_3_18703068	Ca_desi_chr03	18703068	[T/C]	NA
Ca_Desi_Chr_3_18715729	Ca_desi_chr03	18715729	[G/C]	NA
Ca_Desi_Chr_3_18823826	Ca_desi_chr03	18823826	[C/A]	NA
Ca_Desi_Chr_3_18823827	Ca_desi_chr03	18823827	[T/G]	NA
Ca_Desi_Chr_3_18935406	Ca_desi_chr03	18935406	[T/C]	NA
Ca_Desi_Chr_3_18935413	Ca_desi_chr03	18935413	[A/G]	NA
Ca_Desi_Chr_3_18935426	Ca_desi_chr03	18935426	[T/G]	NA
Ca_Desi_Chr_3_18935432	Ca_desi_chr03	18935432	[A/G]	NA
Ca_Desi_Chr_3_18935434	Ca_desi_chr03	18935434	[T/G]	NA
Ca_Desi_Chr_3_18940044	Ca_desi_chr03	18940044	[T/A]	NA
Ca_Desi_Chr_3_18940077	Ca_desi_chr03	18940077	[A/G]	NA
Ca_Desi_Chr_3_18940085	Ca_desi_chr03	18940085	[A/G]	NA
Ca_Desi_Chr_3_18976026	Ca_desi_chr03	18976026	[T/C]	NA
Ca_Desi_Chr_3_18976029	Ca_desi_chr03	18976029	[A/C]	NA
Ca_Desi_Chr_3_18976267	Ca_desi_chr03	18976267	[A/G]	NA
Ca_Desi_Chr_3_18976278	Ca_desi_chr03	18976278	[T/G]	NA
Ca_Desi_Chr_3_18976280	Ca_desi_chr03	18976280	[T/C]	NA
Ca_Desi_Chr_3_18976287	Ca_desi_chr03	18976287	[T/C]	NA
Ca_Desi_Chr_3_18976321	Ca_desi_chr03	18976321	[A/T]	NA
Ca_Desi_Chr_3_18976324	Ca_desi_chr03	18976324	[A/G]	NA
Ca_Desi_Chr_3_19038579	Ca_desi_chr03	19038579	[C/A]	NA
Ca_Desi_Chr_3_19041530	Ca_desi_chr03	19041530	[A/C]	NA
Ca_Desi_Chr_3_19041854	Ca_desi_chr03	19041854	[T/C]	NA
Ca_Desi_Chr_3_19041856	Ca_desi_chr03	19041856	[T/C]	NA
Ca_Desi_Chr_3_19041868	Ca_desi_chr03	19041868	[T/G]	NA
Ca_Desi_Chr_3_19047706	Ca_desi_chr03	19047706	[A/C]	NA
Ca_Desi_Chr_3_19054086	Ca_desi_chr03	19054086	[T/G]	NA
Ca_Desi_Chr_3_19059884	Ca_desi_chr03	19059884	[T/C]	NA
Ca_Desi_Chr_3_19059895	Ca_desi_chr03	19059895	[T/C]	NA

SNP IDs	Chromosomes	Physical positions (bp)	SNPs	NCBI SNPdb accession IDs (NCBI_ss#)
Ca_Desi_Chr_3_19059902	Ca_desi_chr03	19059902	[A/G]	NA
Ca_Desi_Chr_3_19059905	Ca_desi_chr03	19059905	[T/G]	NA
Ca_Desi_Chr_3_19059915	Ca_desi_chr03	19059915	[A/C]	NA
Ca_Desi_Chr_3_19062095	Ca_desi_chr03	19062095	[T/C]	NA
Ca_Desi_Chr_3_19062134	Ca_desi_chr03	19062134	[T/C]	NA
Ca_Desi_Chr_3_19062151	Ca_desi_chr03	19062151	[A/G]	NA
Ca_Desi_Chr_3_19113242	Ca_desi_chr03	19113242	[A/C]	NA
Ca_Desi_Chr_3_19113328	Ca_desi_chr03	19113328	[A/G]	NA
Ca_Desi_Chr_3_19113355	Ca_desi_chr03	19113355	[T/C]	NA
Ca_Desi_Chr_3_19113358	Ca_desi_chr03	19113358	[T/A]	NA
Ca_Desi_Chr_3_19113365	Ca_desi_chr03	19113365	[T/C]	NA
Ca_Desi_Chr_3_19113373	Ca_desi_chr03	19113373	[A/C]	NA
Ca_Desi_Chr_3_19113385	Ca_desi_chr03	19113385	[A/G]	NA
Ca_Desi_Chr_3_19113403	Ca_desi_chr03	19113403	[T/C]	NA
Ca_Desi_Chr_3_19113486	Ca_desi_chr03	19113486	[T/C]	NA
Ca_Desi_Chr_3_19114100	Ca_desi_chr03	19114100	[T/G]	NA
Ca_Desi_Chr_3_19114103	Ca_desi_chr03	19114103	[T/G]	NA
Ca_Desi_Chr_3_19114106	Ca_desi_chr03	19114106	[T/G]	NA
Ca_Desi_Chr_3_19166628	Ca_desi_chr03	19166628	[C/A]	NA
Ca_Desi_Chr_3_19166629	Ca_desi_chr03	19166629	[A/G]	NA
Ca_Desi_Chr_3_19166631	Ca_desi_chr03	19166631	[A/T]	NA
Ca_Desi_Chr_3_19166632	Ca_desi_chr03	19166632	[G/C]	NA
Ca_Desi_Chr_3_19167145	Ca_desi_chr03	19167145	[T/G]	NA
Ca_Desi_Chr_3_19172948	Ca_desi_chr03	19172948	[A/G]	NA
Ca_Desi_Chr_3_19172975	Ca_desi_chr03	19172975	[T/A]	NA
Ca_Desi_Chr_3_19172986	Ca_desi_chr03	19172986	[T/G]	NA
Ca_Desi_Chr_3_19177878	Ca_desi_chr03	19177878	[T/G]	NA
Ca_Desi_Chr_3_19177894	Ca_desi_chr03	19177894	[A/C]	NA
Ca_Desi_Chr_3_19472702	Ca_desi_chr03	19472702	[T/G]	NA
Ca_Desi_Chr_3_19476992	Ca_desi_chr03	19476992	[A/C]	NA
Ca_Desi_Chr_3_19476996	Ca_desi_chr03	19476996	[A/C]	NA
Ca_Desi_Chr_3_19483576	Ca_desi_chr03	19483576	[T/A]	NA
Ca_Desi_Chr_3_19483577	Ca_desi_chr03	19483577	[A/C]	NA
Ca_Desi_Chr_3_19538135	Ca_desi_chr03	19538135	[A/G]	NA

SNP IDs	Chromosomes	Physical positions (bp)	SNPs	NCBI SNPdb accession IDs (NCBI_ss#)
Ca_Desi_Chr_3_19538150	Ca_desi_chr03	19538150	[T/C]	NA
Ca_Desi_Chr_3_19538163	Ca_desi_chr03	19538163	[A/G]	NA
Ca_Desi_Chr_3_19538167	Ca_desi_chr03	19538167	[A/G]	NA
Ca_Desi_Chr_3_19538176	Ca_desi_chr03	19538176	[A/C]	NA
Ca_Desi_Chr_3_19538180	Ca_desi_chr03	19538180	[T/C]	NA
Ca_Desi_Chr_3_19538186	Ca_desi_chr03	19538186	[T/C]	NA
Ca_Desi_Chr_3_19562100	Ca_desi_chr03	19562100	[A/G]	NA
Ca_Desi_Chr_3_19562112	Ca_desi_chr03	19562112	[T/G]	NA
Ca_Desi_Chr_3_19562368	Ca_desi_chr03	19562368	[T/G]	NA
Ca_Desi_Chr_3_19634516	Ca_desi_chr03	19634516	[T/G]	NA
Ca_Desi_Chr_3_19634546	Ca_desi_chr03	19634546	[T/G]	NA
Ca_Desi_Chr_3_19634550	Ca_desi_chr03	19634550	[T/G]	NA
Ca_Desi_Chr_3_19634610	Ca_desi_chr03	19634610	[G/C]	NA
Ca_Desi_Chr_3_19634629	Ca_desi_chr03	19634629	[A/G]	NA
Ca_Desi_Chr_3_19634633	Ca_desi_chr03	19634633	[A/G]	NA
Ca_Desi_Chr_3_19634642	Ca_desi_chr03	19634642	[T/C]	NA
Ca_Desi_Chr_3_19636920	Ca_desi_chr03	19636920	[T/G]	NA
Ca_Desi_Chr_3_19636940	Ca_desi_chr03	19636940	[T/G]	NA
Ca_Desi_Chr_3_19636950	Ca_desi_chr03	19636950	[T/G]	NA
Ca_Desi_Chr_3_19636954	Ca_desi_chr03	19636954	[T/G]	NA
Ca_Desi_Chr_3_19636958	Ca_desi_chr03	19636958	[T/C]	NA
Ca_Desi_Chr_3_19636997	Ca_desi_chr03	19636997	[T/C]	NA
Ca_Desi_Chr_3_19637005	Ca_desi_chr03	19637005	[T/C]	NA
Ca_Desi_Chr_3_19637006	Ca_desi_chr03	19637006	[C/T]	NA
Ca_Desi_Chr_3_19637012	Ca_desi_chr03	19637012	[G/C]	NA
Ca_Desi_Chr_3_19637014	Ca_desi_chr03	19637014	[G/C]	NA
Ca_Desi_Chr_3_19637019	Ca_desi_chr03	19637019	[T/C]	NA
Ca_Desi_Chr_3_19637032	Ca_desi_chr03	19637032	[A/G]	NA
Ca_Desi_Chr_3_19637036	Ca_desi_chr03	19637036	[A/C]	NA
Ca_Desi_Chr_3_19649188	Ca_desi_chr03	19649188	[T/G]	NA
Ca_Desi_Chr_3_19659019	Ca_desi_chr03	19659019	[A/C]	NA
Ca_Desi_Chr_3_19690042	Ca_desi_chr03	19690042	[T/C]	NA
Ca_Desi_Chr_3_19690052	Ca_desi_chr03	19690052	[T/C]	NA
Ca_Desi_Chr_3_19690074	Ca_desi_chr03	19690074	[A/G]	NA

SNP IDs	Chromosomes	Physical positions (bp)	SNPs	NCBI SNPdb accession IDs (NCBI_ss#)
Ca_Desi_Chr_3_19690078	Ca_desi_chr03	19690078	[T/G]	NA
Ca_Desi_Chr_3_19710047	Ca_desi_chr03	19710047	[T/G]	NA
Ca_Desi_Chr_3_19779281	Ca_desi_chr03	19779281	[T/G]	NA
Ca_Desi_Chr_3_19781175	Ca_desi_chr03	19781175	[A/G]	NA
Ca_Desi_Chr_3_19827447	Ca_desi_chr03	19827447	[A/C]	NA
Ca_Desi_Chr_3_19827450	Ca_desi_chr03	19827450	[C/A]	NA
Ca_Desi_Chr_3_19844603	Ca_desi_chr03	19844603	[T/G]	NA
Ca_Desi_Chr_3_19844606	Ca_desi_chr03	19844606	[T/G]	NA
Ca_Desi_Chr_3_19844632	Ca_desi_chr03	19844632	[T/G]	NA
Ca_Desi_Chr_3_19844666	Ca_desi_chr03	19844666	[A/C]	NA
Ca_Desi_Chr_3_19877477	Ca_desi_chr03	19877477	[T/G]	NA
Ca_Desi_Chr_3_19884972	Ca_desi_chr03	19884972	[T/G]	NA
Ca_Desi_Chr_3_19886207	Ca_desi_chr03	19886207	[T/C]	NA
Ca_Desi_Chr_3_19886210	Ca_desi_chr03	19886210	[A/G]	NA
Ca_Desi_Chr_3_19898847	Ca_desi_chr03	19898847	[T/G]	NA
Ca_Desi_Chr_3_19898912	Ca_desi_chr03	19898912	[A/C]	NA
Ca_Desi_Chr_3_19912158	Ca_desi_chr03	19912158	[A/G]	NA
Ca_Desi_Chr_3_19912161	Ca_desi_chr03	19912161	[T/G]	NA
Ca_Desi_Chr_3_19912164	Ca_desi_chr03	19912164	[A/C]	NA
Ca_Desi_Chr_3_19925874	Ca_desi_chr03	19925874	[T/C]	NA
Ca_Desi_Chr_3_19925928	Ca_desi_chr03	19925928	[A/G]	NA
Ca_Desi_Chr_3_19962321	Ca_desi_chr03	19962321	[A/G]	NA
Ca_Desi_Chr_3_19962333	Ca_desi_chr03	19962333	[A/G]	NA
Ca_Desi_Chr_3_19986924	Ca_desi_chr03	19986924	[T/A]	NA
Ca_Desi_Chr_3_19986925	Ca_desi_chr03	19986925	[A/G]	NA
Ca_Desi_Chr_3_20002675	Ca_desi_chr03	20002675	[T/C]	NA
Ca_Desi_Chr_3_20002678	Ca_desi_chr03	20002678	[A/C]	NA
Ca_Desi_Chr_3_20002698	Ca_desi_chr03	20002698	[T/C]	NA
Ca_Desi_Chr_3_20002708	Ca_desi_chr03	20002708	[T/C]	NA
Ca_Desi_Chr_3_20040729	Ca_desi_chr03	20040729	[A/T]	NA
Ca_Desi_Chr_3_20040743	Ca_desi_chr03	20040743	[T/C]	NA
Ca_Desi_Chr_3_20040765	Ca_desi_chr03	20040765	[T/C]	NA
Ca_Desi_Chr_3_20074748	Ca_desi_chr03	20074748	[T/C]	NA
Ca_Desi_Chr_3_20096073	Ca_desi_chr03	20096073	[T/G]	NA

SNP IDs	Chromosomes	Physical positions (bp)	SNPs	NCBI SNPdb accession IDs (NCBI_ss#)
Ca_Desi_Chr_3_20096092	Ca_desi_chr03	20096092	[T/C]	NA
Ca_Desi_Chr_3_20161445	Ca_desi_chr03	20161445	[G/A]	NA
Ca_Desi_Chr_3_20229567	Ca_desi_chr03	20229567	[T/C]	NA
Ca_Desi_Chr_3_20229573	Ca_desi_chr03	20229573	[A/G]	NA
Ca_Desi_Chr_3_20229596	Ca_desi_chr03	20229596	[A/G]	NA
Ca_Desi_Chr_3_20240165	Ca_desi_chr03	20240165	[G/T]	NA
Ca_Desi_Chr_3_20240166	Ca_desi_chr03	20240166	[A/C]	NA
Ca_Desi_Chr_3_20240167	Ca_desi_chr03	20240167	[G/T]	NA
Ca_Desi_Chr_3_20267301	Ca_desi_chr03	20267301	[G/A]	NA
Ca_Desi_Chr_3_20316673	Ca_desi_chr03	20316673	[T/A]	NA
Ca_Desi_Chr_3_20316939	Ca_desi_chr03	20316939	[A/G]	NA
Ca_Desi_Chr_3_20372696	Ca_desi_chr03	20372696	[T/A]	NA
Ca_Desi_Chr_3_20603580	Ca_desi_chr03	20603580	[T/C]	NA
Ca_Desi_Chr_3_20614553	Ca_desi_chr03	20614553	[T/G]	NA
Ca_Desi_Chr_3_20614556	Ca_desi_chr03	20614556	[T/G]	NA
Ca_Desi_Chr_3_20614559	Ca_desi_chr03	20614559	[T/G]	NA
Ca_Desi_Chr_3_21054191	Ca_desi_chr03	21054191	[A/T]	NA
Ca_Desi_Chr_3_21054198	Ca_desi_chr03	21054198	[C/G]	NA
Ca_Desi_Chr_3_21054199	Ca_desi_chr03	21054199	[T/C]	NA
Ca_Desi_Chr_3_21054201	Ca_desi_chr03	21054201	[T/C]	NA
Ca_Desi_Chr_3_21058099	Ca_desi_chr03	21058099	[T/C]	NA
Ca_Desi_Chr_3_21058103	Ca_desi_chr03	21058103	[T/C]	NA
Ca_Desi_Chr_3_21058105	Ca_desi_chr03	21058105	[T/C]	NA
Ca_Desi_Chr_3_21058111	Ca_desi_chr03	21058111	[A/G]	NA
Ca_Desi_Chr_3_21058117	Ca_desi_chr03	21058117	[T/C]	NA
Ca_Desi_Chr_3_21058130	Ca_desi_chr03	21058130	[T/C]	NA
Ca_Desi_Chr_3_21058131	Ca_desi_chr03	21058131	[T/C]	NA
Ca_Desi_Chr_3_21058134	Ca_desi_chr03	21058134	[T/C]	NA
Ca_Desi_Chr_3_21058136	Ca_desi_chr03	21058136	[T/C]	NA
Ca_Desi_Chr_3_21058138	Ca_desi_chr03	21058138	[A/C]	NA
Ca_Desi_Chr_3_21058142	Ca_desi_chr03	21058142	[T/C]	NA
Ca_Desi_Chr_3_21058143	Ca_desi_chr03	21058143	[T/C]	NA
Ca_Desi_Chr_3_21058147	Ca_desi_chr03	21058147	[A/G]	NA
Ca_Desi_Chr_3_21058165	Ca_desi_chr03	21058165	[A/G]	NA

SNP IDs	Chromosomes	Physical positions (bp)	SNPs	NCBI SNPdb accession IDs (NCBI_ss#)
Ca_Desi_Chr_3_21114954	Ca_desi_chr03	21114954	[A/C]	NA
Ca_Desi_Chr_3_21182936	Ca_desi_chr03	21182936	[A/T]	NA
Ca_Desi_Chr_3_21182978	Ca_desi_chr03	21182978	[A/T]	NA
Ca_Desi_Chr_3_21182981	Ca_desi_chr03	21182981	[T/C]	NA
Ca_Desi_Chr_3_21182985	Ca_desi_chr03	21182985	[A/G]	NA
Ca_Desi_Chr_3_21182989	Ca_desi_chr03	21182989	[A/G]	NA
Ca_Desi_Chr_3_21182995	Ca_desi_chr03	21182995	[T/G]	NA
Ca_Desi_Chr_3_21183003	Ca_desi_chr03	21183003	[T/C]	NA
Ca_Desi_Chr_3_21183011	Ca_desi_chr03	21183011	[T/C]	NA
Ca_Desi_Chr_3_21183015	Ca_desi_chr03	21183015	[T/C]	NA
Ca_Desi_Chr_3_21192011	Ca_desi_chr03	21192011	[T/A]	NA
Ca_Desi_Chr_3_21209794	Ca_desi_chr03	21209794	[G/A]	NA
Ca_Desi_Chr_3_21209800	Ca_desi_chr03	21209800	[C/A]	NA
Ca_Desi_Chr_3_21209826	Ca_desi_chr03	21209826	[T/C]	NA
Ca_Desi_Chr_3_21437812	Ca_desi_chr03	21437812	[T/C]	NA
Ca_Desi_Chr_3_21550846	Ca_desi_chr03	21550846	[G/A]	NA
Ca_Desi_Chr_3_21550970	Ca_desi_chr03	21550970	[T/A]	NA
Ca_Desi_Chr_3_21552409	Ca_desi_chr03	21552409	[A/G]	NA
Ca_Desi_Chr_3_21552416	Ca_desi_chr03	21552416	[G/A]	NA
Ca_Desi_Chr_3_21552417	Ca_desi_chr03	21552417	[A/G]	NA
Ca_Desi_Chr_3_21552418	Ca_desi_chr03	21552418	[T/G]	NA
Ca_Desi_Chr_3_21552427	Ca_desi_chr03	21552427	[A/G]	NA
Ca_Desi_Chr_3_21552431	Ca_desi_chr03	21552431	[A/G]	NA
Ca_Desi_Chr_3_21552432	Ca_desi_chr03	21552432	[A/G]	NA
Ca_Desi_Chr_3_21552438	Ca_desi_chr03	21552438	[A/G]	NA
Ca_Desi_Chr_3_21552439	Ca_desi_chr03	21552439	[A/G]	NA
Ca_Desi_Chr_3_21552441	Ca_desi_chr03	21552441	[A/G]	NA
Ca_Desi_Chr_3_21552453	Ca_desi_chr03	21552453	[T/C]	NA
Ca_Desi_Chr_3_21552455	Ca_desi_chr03	21552455	[T/C]	NA
Ca_Desi_Chr_3_21552462	Ca_desi_chr03	21552462	[T/C]	NA
Ca_Desi_Chr_3_21587264	Ca_desi_chr03	21587264	[A/C]	NA
Ca_Desi_Chr_3_21588107	Ca_desi_chr03	21588107	[A/G]	NA
Ca_Desi_Chr_3_21588117	Ca_desi_chr03	21588117	[A/G]	NA
Ca_Desi_Chr_3_21588138	Ca_desi_chr03	21588138	[A/G]	NA

SNP IDs	Chromosomes	Physical positions (bp)	SNPs	NCBI SNPdb accession IDs (NCBI_ss#)
Ca_Desi_Chr_3_21588189	Ca_desi_chr03	21588189	[T/A]	NA
Ca_Desi_Chr_3_21588205	Ca_desi_chr03	21588205	[A/C]	NA
Ca_Desi_Chr_3_21588215	Ca_desi_chr03	21588215	[A/C]	NA
Ca_Desi_Chr_3_21592386	Ca_desi_chr03	21592386	[T/C]	NA
Ca_Desi_Chr_3_21592405	Ca_desi_chr03	21592405	[T/C]	NA
Ca_Desi_Chr_3_21592409	Ca_desi_chr03	21592409	[A/G]	NA
Ca_Desi_Chr_3_21592410	Ca_desi_chr03	21592410	[T/G]	NA
Ca_Desi_Chr_3_21592439	Ca_desi_chr03	21592439	[A/G]	NA
Ca_Desi_Chr_3_21621191	Ca_desi_chr03	21621191	[A/G]	NA
Ca_Desi_Chr_3_21621224	Ca_desi_chr03	21621224	[A/C]	NA
Ca_Desi_Chr_3_21624166	Ca_desi_chr03	21624166	[T/C]	NA
Ca_Desi_Chr_3_21624170	Ca_desi_chr03	21624170	[A/T]	NA
Ca_Desi_Chr_3_21624274	Ca_desi_chr03	21624274	[A/C]	NA
Ca_Desi_Chr_3_21624279	Ca_desi_chr03	21624279	[A/T]	NA
Ca_Desi_Chr_3_21625614	Ca_desi_chr03	21625614	[A/C]	NA
Ca_Desi_Chr_3_21625693	Ca_desi_chr03	21625693	[T/G]	NA
Ca_Desi_Chr_3_21675730	Ca_desi_chr03	21675730	[T/C]	NA
Ca_Desi_Chr_3_21747046	Ca_desi_chr03	21747046	[T/G]	NA
Ca_Desi_Chr_3_21747048	Ca_desi_chr03	21747048	[T/A]	NA
Ca_Desi_Chr_3_21747074	Ca_desi_chr03	21747074	[A/G]	NA
Ca_Desi_Chr_3_21768472	Ca_desi_chr03	21768472	[C/A]	NA
Ca_Desi_Chr_3_21768473	Ca_desi_chr03	21768473	[A/T]	NA
Ca_Desi_Chr_3_21768474	Ca_desi_chr03	21768474	[A/C]	NA
Ca_Desi_Chr_3_21768475	Ca_desi_chr03	21768475	[G/T]	NA
Ca_Desi_Chr_3_21774427	Ca_desi_chr03	21774427	[A/C]	NA
Ca_Desi_Chr_3_21835284	Ca_desi_chr03	21835284	[T/A]	NA
Ca_Desi_Chr_3_21867107	Ca_desi_chr03	21867107	[T/G]	NA
Ca_Desi_Chr_3_21920964	Ca_desi_chr03	21920964	[T/G]	NA
Ca_Desi_Chr_3_21935578	Ca_desi_chr03	21935578	[A/C]	NA
Ca_Desi_Chr_3_21942881	Ca_desi_chr03	21942881	[T/C]	NA
Ca_Desi_Chr_3_22003665	Ca_desi_chr03	22003665	[T/C]	NA
Ca_Desi_Chr_3_22004700	Ca_desi_chr03	22004700	[T/C]	NA
Ca_Desi_Chr_3_22005360	Ca_desi_chr03	22005360	[T/G]	NA
Ca_Desi_Chr_3_22023130	Ca_desi_chr03	22023130	[A/G]	NA

SNP IDs	Chromosomes	Physical positions (bp)	SNPs	NCBI SNPdb accession IDs (NCBI_ss#)
Ca_Desi_Chr_3_22023133	Ca_desi_chr03	22023133	[T/A]	NA
Ca_Desi_Chr_3_22571755	Ca_desi_chr03	22571755	[T/G]	NA
Ca_Desi_Chr_3_22581329	Ca_desi_chr03	22581329	[T/C]	NA
Ca_Desi_Chr_3_22618251	Ca_desi_chr03	22618251	[A/G]	NA
Ca_Desi_Chr_3_22618265	Ca_desi_chr03	22618265	[T/C]	NA
Ca_Desi_Chr_3_22618284	Ca_desi_chr03	22618284	[A/G]	NA
Ca_Desi_Chr_3_22618310	Ca_desi_chr03	22618310	[A/C]	NA
Ca_Desi_Chr_3_22687823	Ca_desi_chr03	22687823	[C/T]	NA
Ca_Desi_Chr_3_22687902	Ca_desi_chr03	22687902	[T/C]	NA
Ca_Desi_Chr_3_22737849	Ca_desi_chr03	22737849	[T/C]	NA
Ca_Desi_Chr_3_22737873	Ca_desi_chr03	22737873	[A/T]	NA
Ca_Desi_Chr_3_22737951	Ca_desi_chr03	22737951	[A/C]	NA
Ca_Desi_Chr_3_22737981	Ca_desi_chr03	22737981	[A/G]	NA
Ca_Desi_Chr_3_22830261	Ca_desi_chr03	22830261	[G/A]	NA
Ca_Desi_Chr_3_22834176	Ca_desi_chr03	22834176	[T/C]	NA
Ca_Desi_Chr_3_22834208	Ca_desi_chr03	22834208	[A/G]	NA
Ca_Desi_Chr_3_22903655	Ca_desi_chr03	22903655	[A/G]	NA
Ca_Desi_Chr_3_22903659	Ca_desi_chr03	22903659	[A/G]	NA
Ca_Desi_Chr_3_22903680	Ca_desi_chr03	22903680	[T/G]	NA
Ca_Desi_Chr_3_22903689	Ca_desi_chr03	22903689	[T/C]	NA
Ca_Desi_Chr_3_22909826	Ca_desi_chr03	22909826	[A/G]	NA
Ca_Desi_Chr_3_22954634	Ca_desi_chr03	22954634	[A/G]	NA
Ca_Desi_Chr_3_22954640	Ca_desi_chr03	22954640	[T/C]	NA
Ca_Desi_Chr_3_22967778	Ca_desi_chr03	22967778	[T/G]	NA
Ca_Desi_Chr_3_22969949	Ca_desi_chr03	22969949	[A/G]	NA
Ca_Desi_Chr_3_22969957	Ca_desi_chr03	22969957	[A/T]	NA
Ca_Desi_Chr_3_22969964	Ca_desi_chr03	22969964	[T/C]	NA
Ca_Desi_Chr_3_22969980	Ca_desi_chr03	22969980	[A/C]	NA
Ca_Desi_Chr_3_22969991	Ca_desi_chr03	22969991	[A/G]	NA
Ca_Desi_Chr_3_22969996	Ca_desi_chr03	22969996	[T/A]	NA
Ca_Desi_Chr_3_22969998	Ca_desi_chr03	22969998	[A/G]	NA
Ca_Desi_Chr_3_22973066	Ca_desi_chr03	22973066	[C/T]	NA
Ca_Desi_Chr_3_22973073	Ca_desi_chr03	22973073	[T/C]	NA
Ca_Desi_Chr_3_22973135	Ca_desi_chr03	22973135	[A/C]	NA

SNP IDs	Chromosomes	Physical positions (bp)	SNPs	NCBI SNPdb accession IDs (NCBI_ss#)
Ca_Desi_Chr_3_22980522	Ca_desi_chr03	22980522	[A/G]	NA
Ca_Desi_Chr_3_22980534	Ca_desi_chr03	22980534	[G/C]	NA
Ca_Desi_Chr_3_22984947	Ca_desi_chr03	22984947	[G/C]	NA
Ca_Desi_Chr_3_22992410	Ca_desi_chr03	22992410	[G/A]	NA
Ca_Desi_Chr_3_22992503	Ca_desi_chr03	22992503	[G/A]	NA
Ca_Desi_Chr_3_22992567	Ca_desi_chr03	22992567	[A/G]	NA
Ca_Desi_Chr_3_22992776	Ca_desi_chr03	22992776	[A/G]	NA
Ca_Desi_Chr_3_23022149	Ca_desi_chr03	23022149	[C/T]	NA
Ca_Desi_Chr_3_23024193	Ca_desi_chr03	23024193	[C/A]	NA
Ca_Desi_Chr_3_23024242	Ca_desi_chr03	23024242	[G/T]	NA
Ca_Desi_Chr_3_23025194	Ca_desi_chr03	23025194	[T/G]	NA
Ca_Desi_Chr_3_23025199	Ca_desi_chr03	23025199	[T/G]	NA
Ca_Desi_Chr_3_23026742	Ca_desi_chr03	23026742	[T/G]	NA
Ca_Desi_Chr_3_23029365	Ca_desi_chr03	23029365	[T/A]	NA
Ca_Desi_Chr_3_23032363	Ca_desi_chr03	23032363	[A/G]	NA
Ca_Desi_Chr_3_23032534	Ca_desi_chr03	23032534	[C/T]	NA
Ca_Desi_Chr_3_23056479	Ca_desi_chr03	23056479	[T/G]	NA
Ca_Desi_Chr_3_23056583	Ca_desi_chr03	23056583	[T/G]	NA
Ca_Desi_Chr_3_23056595	Ca_desi_chr03	23056595	[T/A]	NA
Ca_Desi_Chr_3_23056632	Ca_desi_chr03	23056632	[A/C]	NA
Ca_Desi_Chr_3_23056653	Ca_desi_chr03	23056653	[A/G]	NA
Ca_Desi_Chr_3_23096441	Ca_desi_chr03	23096441	[T/G]	NA
Ca_Desi_Chr_3_23096465	Ca_desi_chr03	23096465	[T/G]	NA
Ca_Desi_Chr_3_23096509	Ca_desi_chr03	23096509	[C/T]	NA
Ca_Desi_Chr_3_23096518	Ca_desi_chr03	23096518	[A/G]	NA
Ca_Desi_Chr_3_23142943	Ca_desi_chr03	23142943	[A/G]	NA
Ca_Desi_Chr_3_23144500	Ca_desi_chr03	23144500	[T/C]	NA
Ca_Desi_Chr_3_23144512	Ca_desi_chr03	23144512	[A/T]	NA
Ca_Desi_Chr_3_23144519	Ca_desi_chr03	23144519	[T/C]	NA
Ca_Desi_Chr_3_23156234	Ca_desi_chr03	23156234	[C/T]	NA
Ca_Desi_Chr_3_23156257	Ca_desi_chr03	23156257	[A/G]	NA
Ca_Desi_Chr_3_23157660	Ca_desi_chr03	23157660	[T/A]	NA
Ca_Desi_Chr_3_23157661	Ca_desi_chr03	23157661	[A/G]	NA
Ca_Desi_Chr_3_23164356	Ca_desi_chr03	23164356	[T/C]	NA

SNP IDs	Chromosomes	Physical positions (bp)	SNPs	NCBI SNPdb accession IDs (NCBI_ss#)
Ca_Desi_Chr_3_23164542	Ca_desi_chr03	23164542	[T/C]	NA
Ca_Desi_Chr_3_23164549	Ca_desi_chr03	23164549	[C/A]	NA
Ca_Desi_Chr_3_23164625	Ca_desi_chr03	23164625	[G/A]	NA
Ca_Desi_Chr_3_23164937	Ca_desi_chr03	23164937	[A/G]	NA
Ca_Desi_Chr_3_23164953	Ca_desi_chr03	23164953	[T/C]	NA
Ca_Desi_Chr_3_23164983	Ca_desi_chr03	23164983	[C/T]	NA
Ca_Desi_Chr_3_23235399	Ca_desi_chr03	23235399	[A/G]	NA
Ca_Desi_Chr_3_23325891	Ca_desi_chr03	23325891	[C/A]	NA
Ca_Desi_Chr_4_7882	Ca_desi_chr04	7882	[T/G]	NA
Ca_Desi_Chr_4_40641	Ca_desi_chr04	40641	[A/G]	NA
Ca_Desi_Chr_4_112110	Ca_desi_chr04	112110	[C/A]	NA
Ca_Desi_Chr_4_122800	Ca_desi_chr04	122800	[T/C]	NA
Ca_Desi_Chr_4_122834	Ca_desi_chr04	122834	[T/C]	NA
Ca_Desi_Chr_4_123046	Ca_desi_chr04	123046	[G/A]	NA
Ca_Desi_Chr_4_130836	Ca_desi_chr04	130836	[C/T]	NA
Ca_Desi_Chr_4_130850	Ca_desi_chr04	130850	[G/A]	NA
Ca_Desi_Chr_4_130883	Ca_desi_chr04	130883	[T/G]	NA
Ca_Desi_Chr_4_131227	Ca_desi_chr04	131227	[A/G]	NA
Ca_Desi_Chr_4_133375	Ca_desi_chr04	133375	[C/A]	NA
Ca_Desi_Chr_4_145927	Ca_desi_chr04	145927	[A/G]	NA
Ca_Desi_Chr_4_149986	Ca_desi_chr04	149986	[T/C]	NA
Ca_Desi_Chr_4_150577	Ca_desi_chr04	150577	[T/C]	NA
Ca_Desi_Chr_4_150640	Ca_desi_chr04	150640	[A/C]	NA
Ca_Desi_Chr_4_150643	Ca_desi_chr04	150643	[A/C]	NA
Ca_Desi_Chr_4_151462	Ca_desi_chr04	151462	[G/C]	NA
Ca_Desi_Chr_4_181954	Ca_desi_chr04	181954	[C/G]	NA
Ca_Desi_Chr_4_198665	Ca_desi_chr04	198665	[T/C]	NA
Ca_Desi_Chr_4_223008	Ca_desi_chr04	223008	[G/A]	NA
Ca_Desi_Chr_4_223012	Ca_desi_chr04	223012	[T/G]	NA
Ca_Desi_Chr_4_232316	Ca_desi_chr04	232316	[T/A]	NA
Ca_Desi_Chr_4_232426	Ca_desi_chr04	232426	[T/G]	NA
Ca_Desi_Chr_4_234977	Ca_desi_chr04	234977	[T/G]	NA
Ca_Desi_Chr_4_237082	Ca_desi_chr04	237082	[C/G]	NA
Ca_Desi_Chr_4_238567	Ca_desi_chr04	238567	[G/T]	NA

SNP IDs	Chromosomes	Physical positions (bp)	SNPs	NCBI SNPdb accession IDs (NCBI_ss#)
Ca_Desi_Chr_4_240061	Ca_desi_chr04	240061	[T/C]	NA
Ca_Desi_Chr_4_240109	Ca_desi_chr04	240109	[A/C]	NA
Ca_Desi_Chr_4_241125	Ca_desi_chr04	241125	[A/G]	NA
Ca_Desi_Chr_4_243174	Ca_desi_chr04	243174	[G/A]	NA
Ca_Desi_Chr_4_256283	Ca_desi_chr04	256283	[C/T]	NA
Ca_Desi_Chr_4_256346	Ca_desi_chr04	256346	[C/G]	NA
Ca_Desi_Chr_4_257906	Ca_desi_chr04	257906	[T/C]	NA
Ca_Desi_Chr_4_257924	Ca_desi_chr04	257924	[A/G]	NA
Ca_Desi_Chr_4_257926	Ca_desi_chr04	257926	[T/A]	NA
Ca_Desi_Chr_4_257927	Ca_desi_chr04	257927	[A/C]	NA
Ca_Desi_Chr_4_325326	Ca_desi_chr04	325326	[A/G]	NA
Ca_Desi_Chr_4_325342	Ca_desi_chr04	325342	[T/A]	NA
Ca_Desi_Chr_4_325345	Ca_desi_chr04	325345	[A/G]	NA
Ca_Desi_Chr_4_325358	Ca_desi_chr04	325358	[A/G]	NA
Ca_Desi_Chr_4_325410	Ca_desi_chr04	325410	[A/T]	NA
Ca_Desi_Chr_4_325682	Ca_desi_chr04	325682	[T/C]	NA
Ca_Desi_Chr_4_325982	Ca_desi_chr04	325982	[T/G]	NA
Ca_Desi_Chr_4_326013	Ca_desi_chr04	326013	[T/G]	NA
Ca_Desi_Chr_4_326048	Ca_desi_chr04	326048	[A/T]	NA
Ca_Desi_Chr_4_328232	Ca_desi_chr04	328232	[C/T]	NA
Ca_Desi_Chr_4_334803	Ca_desi_chr04	334803	[T/G]	NA
Ca_Desi_Chr_4_335462	Ca_desi_chr04	335462	[A/G]	NA
Ca_Desi_Chr_4_340829	Ca_desi_chr04	340829	[G/A]	NA
Ca_Desi_Chr_4_345799	Ca_desi_chr04	345799	[C/T]	NA
Ca_Desi_Chr_4_345813	Ca_desi_chr04	345813	[G/A]	NA
Ca_Desi_Chr_4_348202	Ca_desi_chr04	348202	[T/A]	NA
Ca_Desi_Chr_4_348344	Ca_desi_chr04	348344	[A/G]	NA
Ca_Desi_Chr_4_348384	Ca_desi_chr04	348384	[T/C]	NA
Ca_Desi_Chr_4_348770	Ca_desi_chr04	348770	[A/C]	NA
Ca_Desi_Chr_4_354152	Ca_desi_chr04	354152	[A/G]	NA
Ca_Desi_Chr_4_366996	Ca_desi_chr04	366996	[A/G]	NA
Ca_Desi_Chr_4_367014	Ca_desi_chr04	367014	[A/G]	NA
Ca_Desi_Chr_4_367025	Ca_desi_chr04	367025	[A/G]	NA
Ca_Desi_Chr_4_367026	Ca_desi_chr04	367026	[G/A]	NA

SNP IDs	Chromosomes	Physical positions (bp)	SNPs	NCBI SNPdb accession IDs (NCBI_ss#)
Ca_Desi_Chr_4_367027	Ca_desi_chr04	367027	[A/G]	NA
Ca_Desi_Chr_4_367801	Ca_desi_chr04	367801	[A/T]	NA
Ca_Desi_Chr_4_367804	Ca_desi_chr04	367804	[A/G]	NA
Ca_Desi_Chr_4_367805	Ca_desi_chr04	367805	[A/C]	NA
Ca_Desi_Chr_4_367808	Ca_desi_chr04	367808	[A/C]	NA
Ca_Desi_Chr_4_367811	Ca_desi_chr04	367811	[A/G]	NA
Ca_Desi_Chr_4_367814	Ca_desi_chr04	367814	[T/G]	NA
Ca_Desi_Chr_4_367816	Ca_desi_chr04	367816	[T/G]	NA
Ca_Desi_Chr_4_367818	Ca_desi_chr04	367818	[A/G]	NA
Ca_Desi_Chr_4_367821	Ca_desi_chr04	367821	[G/T]	NA
Ca_Desi_Chr_4_367822	Ca_desi_chr04	367822	[T/G]	NA
Ca_Desi_Chr_4_367825	Ca_desi_chr04	367825	[G/T]	NA
Ca_Desi_Chr_4_367826	Ca_desi_chr04	367826	[T/C]	NA
Ca_Desi_Chr_4_367827	Ca_desi_chr04	367827	[C/A]	NA
Ca_Desi_Chr_4_367829	Ca_desi_chr04	367829	[A/C]	NA
Ca_Desi_Chr_4_367832	Ca_desi_chr04	367832	[T/C]	NA
Ca_Desi_Chr_4_367833	Ca_desi_chr04	367833	[G/C]	NA
Ca_Desi_Chr_4_367835	Ca_desi_chr04	367835	[A/C]	NA
Ca_Desi_Chr_4_367838	Ca_desi_chr04	367838	[T/C]	NA
Ca_Desi_Chr_4_367844	Ca_desi_chr04	367844	[T/C]	NA
Ca_Desi_Chr_4_367943	Ca_desi_chr04	367943	[A/G]	NA
Ca_Desi_Chr_4_368030	Ca_desi_chr04	368030	[A/T]	NA
Ca_Desi_Chr_4_368270	Ca_desi_chr04	368270	[A/G]	NA
Ca_Desi_Chr_4_368285	Ca_desi_chr04	368285	[G/C]	NA
Ca_Desi_Chr_4_368438	Ca_desi_chr04	368438	[G/T]	NA
Ca_Desi_Chr_4_368472	Ca_desi_chr04	368472	[C/T]	NA
Ca_Desi_Chr_4_371113	Ca_desi_chr04	371113	[A/T]	NA
Ca_Desi_Chr_4_371576	Ca_desi_chr04	371576	[G/T]	NA
Ca_Desi_Chr_4_371754	Ca_desi_chr04	371754	[A/T]	NA
Ca_Desi_Chr_4_372779	Ca_desi_chr04	372779	[A/G]	NA
Ca_Desi_Chr_4_372806	Ca_desi_chr04	372806	[C/T]	NA
Ca_Desi_Chr_4_372810	Ca_desi_chr04	372810	[C/G]	NA
Ca_Desi_Chr_4_454624	Ca_desi_chr04	454624	[T/G]	NA
Ca_Desi_Chr_4_454634	Ca_desi_chr04	454634	[T/G]	NA

SNP IDs	Chromosomes	Physical positions (bp)	SNPs	NCBI SNPdb accession IDs (NCBI_ss#)
Ca_Desi_Chr_4_454671	Ca_desi_chr04	454671	[A/C]	NA
Ca_Desi_Chr_4_600659	Ca_desi_chr04	600659	[T/C]	NA
Ca_Desi_Chr_4_600687	Ca_desi_chr04	600687	[T/G]	NA
Ca_Desi_Chr_4_600743	Ca_desi_chr04	600743	[T/G]	NA
Ca_Desi_Chr_4_600805	Ca_desi_chr04	600805	[T/G]	NA
Ca_Desi_Chr_4_677279	Ca_desi_chr04	677279	[A/C]	NA
Ca_Desi_Chr_4_677289	Ca_desi_chr04	677289	[A/C]	NA
Ca_Desi_Chr_4_677300	Ca_desi_chr04	677300	[T/C]	NA
Ca_Desi_Chr_4_686239	Ca_desi_chr04	686239	[T/G]	NA
Ca_Desi_Chr_4_686310	Ca_desi_chr04	686310	[A/C]	NA
Ca_Desi_Chr_4_686313	Ca_desi_chr04	686313	[T/C]	NA
Ca_Desi_Chr_4_686341	Ca_desi_chr04	686341	[A/G]	NA
Ca_Desi_Chr_4_686343	Ca_desi_chr04	686343	[T/C]	NA
Ca_Desi_Chr_4_686346	Ca_desi_chr04	686346	[T/C]	NA
Ca_Desi_Chr_4_686358	Ca_desi_chr04	686358	[T/A]	NA
Ca_Desi_Chr_4_705227	Ca_desi_chr04	705227	[A/G]	NA
Ca_Desi_Chr_4_748189	Ca_desi_chr04	748189	[A/C]	NA
Ca_Desi_Chr_4_748485	Ca_desi_chr04	748485	[A/C]	NA
Ca_Desi_Chr_4_748638	Ca_desi_chr04	748638	[T/C]	NA
Ca_Desi_Chr_4_748644	Ca_desi_chr04	748644	[A/G]	NA
Ca_Desi_Chr_4_748653	Ca_desi_chr04	748653	[T/C]	NA
Ca_Desi_Chr_4_748660	Ca_desi_chr04	748660	[T/C]	NA
Ca_Desi_Chr_4_748666	Ca_desi_chr04	748666	[A/G]	NA
Ca_Desi_Chr_4_748679	Ca_desi_chr04	748679	[C/A]	NA
Ca_Desi_Chr_4_771278	Ca_desi_chr04	771278	[A/G]	NA
Ca_Desi_Chr_4_771281	Ca_desi_chr04	771281	[T/C]	NA
Ca_Desi_Chr_4_771305	Ca_desi_chr04	771305	[T/G]	NA
Ca_Desi_Chr_4_878326	Ca_desi_chr04	878326	[T/C]	NA
Ca_Desi_Chr_4_889511	Ca_desi_chr04	889511	[T/G]	NA
Ca_Desi_Chr_4_889512	Ca_desi_chr04	889512	[A/C]	NA
Ca_Desi_Chr_4_889522	Ca_desi_chr04	889522	[T/G]	NA
Ca_Desi_Chr_4_889535	Ca_desi_chr04	889535	[C/G]	NA
Ca_Desi_Chr_4_889538	Ca_desi_chr04	889538	[T/C]	NA
Ca_Desi_Chr_4_908522	Ca_desi_chr04	908522	[A/G]	NA

SNP IDs	Chromosomes	Physical positions (bp)	SNPs	NCBI SNPdb accession IDs (NCBI_ss#)
Ca_Desi_Chr_4_937980	Ca_desi_chr04	937980	[A/T]	NA
Ca_Desi_Chr_4_988768	Ca_desi_chr04	988768	[T/C]	NA
Ca_Desi_Chr_4_988774	Ca_desi_chr04	988774	[A/C]	NA
Ca_Desi_Chr_4_1018475	Ca_desi_chr04	1018475	[A/G]	NA
Ca_Desi_Chr_4_1022130	Ca_desi_chr04	1022130	[T/C]	NA
Ca_Desi_Chr_4_1022131	Ca_desi_chr04	1022131	[G/T]	NA
Ca_Desi_Chr_4_1104662	Ca_desi_chr04	1104662	[A/G]	NA
Ca_Desi_Chr_4_1104670	Ca_desi_chr04	1104670	[T/C]	NA
Ca_Desi_Chr_4_1104677	Ca_desi_chr04	1104677	[T/G]	NA
Ca_Desi_Chr_4_1104683	Ca_desi_chr04	1104683	[A/C]	NA
Ca_Desi_Chr_4_1104692	Ca_desi_chr04	1104692	[T/C]	NA
Ca_Desi_Chr_4_1104703	Ca_desi_chr04	1104703	[T/C]	NA
Ca_Desi_Chr_4_1104705	Ca_desi_chr04	1104705	[A/G]	NA
Ca_Desi_Chr_4_1104721	Ca_desi_chr04	1104721	[T/C]	NA
Ca_Desi_Chr_4_1104818	Ca_desi_chr04	1104818	[A/T]	NA
Ca_Desi_Chr_4_1213556	Ca_desi_chr04	1213556	[C/T]	NA
Ca_Desi_Chr_4_1224856	Ca_desi_chr04	1224856	[C/T]	NA
Ca_Desi_Chr_4_1225164	Ca_desi_chr04	1225164	[G/A]	NA
Ca_Desi_Chr_4_1225194	Ca_desi_chr04	1225194	[T/C]	NA
Ca_Desi_Chr_4_1225198	Ca_desi_chr04	1225198	[A/T]	NA
Ca_Desi_Chr_4_1225209	Ca_desi_chr04	1225209	[A/C]	NA
Ca_Desi_Chr_4_1225214	Ca_desi_chr04	1225214	[T/G]	NA
Ca_Desi_Chr_4_1226703	Ca_desi_chr04	1226703	[A/C]	NA
Ca_Desi_Chr_4_1227379	Ca_desi_chr04	1227379	[A/C]	NA
Ca_Desi_Chr_4_1228415	Ca_desi_chr04	1228415	[A/C]	NA
Ca_Desi_Chr_4_1228422	Ca_desi_chr04	1228422	[C/A]	NA
Ca_Desi_Chr_4_1228423	Ca_desi_chr04	1228423	[T/G]	NA
Ca_Desi_Chr_4_1228424	Ca_desi_chr04	1228424	[T/A]	NA
Ca_Desi_Chr_4_1228477	Ca_desi_chr04	1228477	[A/C]	NA
Ca_Desi_Chr_4_1228490	Ca_desi_chr04	1228490	[T/C]	NA
Ca_Desi_Chr_4_1228507	Ca_desi_chr04	1228507	[A/G]	NA
Ca_Desi_Chr_4_1291858	Ca_desi_chr04	1291858	[T/G]	NA
Ca_Desi_Chr_4_1323272	Ca_desi_chr04	1323272	[A/G]	NA
Ca_Desi_Chr_4_1323273	Ca_desi_chr04	1323273	[A/G]	NA

SNP IDs	Chromosomes	Physical positions (bp)	SNPs	NCBI SNPdb accession IDs (NCBI_ss#)
Ca_Desi_Chr_4_1323281	Ca_desi_chr04	1323281	[T/C]	NA
Ca_Desi_Chr_4_1323295	Ca_desi_chr04	1323295	[T/A]	NA
Ca_Desi_Chr_4_1323302	Ca_desi_chr04	1323302	[C/A]	NA
Ca_Desi_Chr_4_1323358	Ca_desi_chr04	1323358	[T/G]	NA
Ca_Desi_Chr_4_1324130	Ca_desi_chr04	1324130	[T/G]	NA
Ca_Desi_Chr_4_1324142	Ca_desi_chr04	1324142	[T/G]	NA
Ca_Desi_Chr_4_1324156	Ca_desi_chr04	1324156	[G/A]	NA
Ca_Desi_Chr_4_1400955	Ca_desi_chr04	1400955	[C/A]	NA
Ca_Desi_Chr_4_1400956	Ca_desi_chr04	1400956	[A/G]	NA
Ca_Desi_Chr_4_1414209	Ca_desi_chr04	1414209	[T/G]	NA
Ca_Desi_Chr_4_1414218	Ca_desi_chr04	1414218	[T/G]	NA
Ca_Desi_Chr_4_1414223	Ca_desi_chr04	1414223	[T/G]	NA
Ca_Desi_Chr_4_1414231	Ca_desi_chr04	1414231	[A/C]	NA
Ca_Desi_Chr_4_1770546	Ca_desi_chr04	1770546	[A/C]	NA
Ca_Desi_Chr_4_1798668	Ca_desi_chr04	1798668	[T/G]	NA
Ca_Desi_Chr_4_1826373	Ca_desi_chr04	1826373	[G/T]	NA
Ca_Desi_Chr_4_1929722	Ca_desi_chr04	1929722	[A/C]	NA
Ca_Desi_Chr_4_2040754	Ca_desi_chr04	2040754	[T/G]	NA
Ca_Desi_Chr_4_2051124	Ca_desi_chr04	2051124	[T/C]	NA
Ca_Desi_Chr_4_2057214	Ca_desi_chr04	2057214	[A/G]	NA
Ca_Desi_Chr_4_2057278	Ca_desi_chr04	2057278	[T/C]	NA
Ca_Desi_Chr_4_2057299	Ca_desi_chr04	2057299	[T/C]	NA
Ca_Desi_Chr_4_2057309	Ca_desi_chr04	2057309	[A/G]	NA
Ca_Desi_Chr_4_2057324	Ca_desi_chr04	2057324	[T/C]	NA
Ca_Desi_Chr_4_2057337	Ca_desi_chr04	2057337	[A/G]	NA
Ca_Desi_Chr_4_2172640	Ca_desi_chr04	2172640	[C/T]	NA
Ca_Desi_Chr_4_2172655	Ca_desi_chr04	2172655	[G/A]	NA
Ca_Desi_Chr_4_2174884	Ca_desi_chr04	2174884	[A/G]	NA
Ca_Desi_Chr_4_2174886	Ca_desi_chr04	2174886	[G/T]	NA
Ca_Desi_Chr_4_2181555	Ca_desi_chr04	2181555	[C/T]	NA
Ca_Desi_Chr_4_2181568	Ca_desi_chr04	2181568	[G/A]	NA
Ca_Desi_Chr_4_2181583	Ca_desi_chr04	2181583	[T/C]	NA
Ca_Desi_Chr_4_2181587	Ca_desi_chr04	2181587	[G/A]	NA
Ca_Desi_Chr_4_2363586	Ca_desi_chr04	2363586	[T/G]	NA

SNP IDs	Chromosomes	Physical positions (bp)	SNPs	NCBI SNPdb accession IDs (NCBI_ss#)
Ca_Desi_Chr_4_2391174	Ca_desi_chr04	2391174	[A/G]	NA
Ca_Desi_Chr_4_2391280	Ca_desi_chr04	2391280	[C/T]	NA
Ca_Desi_Chr_4_2391300	Ca_desi_chr04	2391300	[A/C]	NA
Ca_Desi_Chr_4_2396211	Ca_desi_chr04	2396211	[A/G]	NA
Ca_Desi_Chr_4_2396248	Ca_desi_chr04	2396248	[A/G]	NA
Ca_Desi_Chr_4_2396431	Ca_desi_chr04	2396431	[T/G]	NA
Ca_Desi_Chr_4_2396436	Ca_desi_chr04	2396436	[T/C]	NA
Ca_Desi_Chr_4_2396520	Ca_desi_chr04	2396520	[T/G]	NA
Ca_Desi_Chr_4_2396528	Ca_desi_chr04	2396528	[A/C]	NA
Ca_Desi_Chr_4_2396547	Ca_desi_chr04	2396547	[T/G]	NA
Ca_Desi_Chr_4_2396629	Ca_desi_chr04	2396629	[T/G]	NA
Ca_Desi_Chr_4_2396679	Ca_desi_chr04	2396679	[C/T]	NA
Ca_Desi_Chr_4_2396689	Ca_desi_chr04	2396689	[A/C]	NA
Ca_Desi_Chr_4_2396695	Ca_desi_chr04	2396695	[A/C]	NA
Ca_Desi_Chr_4_2396705	Ca_desi_chr04	2396705	[G/A]	NA
Ca_Desi_Chr_4_2397043	Ca_desi_chr04	2397043	[C/A]	NA
Ca_Desi_Chr_4_2410917	Ca_desi_chr04	2410917	[G/C]	NA
Ca_Desi_Chr_4_2410949	Ca_desi_chr04	2410949	[A/T]	NA
Ca_Desi_Chr_4_2410950	Ca_desi_chr04	2410950	[A/C]	NA
Ca_Desi_Chr_4_2410951	Ca_desi_chr04	2410951	[G/T]	NA
Ca_Desi_Chr_4_2410996	Ca_desi_chr04	2410996	[A/C]	NA
Ca_Desi_Chr_4_2412911	Ca_desi_chr04	2412911	[T/C]	NA
Ca_Desi_Chr_4_2413091	Ca_desi_chr04	2413091	[C/T]	NA
Ca_Desi_Chr_4_2416463	Ca_desi_chr04	2416463	[C/A]	NA
Ca_Desi_Chr_4_2416464	Ca_desi_chr04	2416464	[A/G]	NA
Ca_Desi_Chr_4_2449602	Ca_desi_chr04	2449602	[T/A]	NA
Ca_Desi_Chr_4_2450642	Ca_desi_chr04	2450642	[G/A]	NA
Ca_Desi_Chr_4_2451122	Ca_desi_chr04	2451122	[A/G]	NA
Ca_Desi_Chr_4_2451258	Ca_desi_chr04	2451258	[A/G]	NA
Ca_Desi_Chr_4_2456945	Ca_desi_chr04	2456945	[C/T]	NA
Ca_Desi_Chr_4_2457952	Ca_desi_chr04	2457952	[A/G]	NA
Ca_Desi_Chr_4_2457958	Ca_desi_chr04	2457958	[A/C]	NA
Ca_Desi_Chr_4_2457985	Ca_desi_chr04	2457985	[A/C]	NA
Ca_Desi_Chr_4_2463339	Ca_desi_chr04	2463339	[G/A]	NA

SNP IDs	Chromosomes	Physical positions (bp)	SNPs	NCBI SNPdb accession IDs (NCBI_ss#)
Ca_Desi_Chr_4_2464105	Ca_desi_chr04	2464105	[G/T]	NA
Ca_Desi_Chr_4_2464106	Ca_desi_chr04	2464106	[A/C]	NA
Ca_Desi_Chr_4_2464107	Ca_desi_chr04	2464107	[G/T]	NA
Ca_Desi_Chr_4_2464122	Ca_desi_chr04	2464122	[C/A]	NA
Ca_Desi_Chr_4_2479967	Ca_desi_chr04	2479967	[A/G]	NA
Ca_Desi_Chr_4_2481151	Ca_desi_chr04	2481151	[A/T]	NA
Ca_Desi_Chr_4_2481165	Ca_desi_chr04	2481165	[T/A]	NA
Ca_Desi_Chr_4_2489929	Ca_desi_chr04	2489929	[T/C]	NA
Ca_Desi_Chr_4_2490877	Ca_desi_chr04	2490877	[A/T]	NA
Ca_Desi_Chr_4_2507278	Ca_desi_chr04	2507278	[A/T]	NA
Ca_Desi_Chr_4_2507304	Ca_desi_chr04	2507304	[T/C]	NA
Ca_Desi_Chr_4_2511841	Ca_desi_chr04	2511841	[G/T]	NA
Ca_Desi_Chr_4_2511853	Ca_desi_chr04	2511853	[C/T]	NA
Ca_Desi_Chr_4_2511894	Ca_desi_chr04	2511894	[T/C]	NA
Ca_Desi_Chr_4_2511901	Ca_desi_chr04	2511901	[A/G]	NA
Ca_Desi_Chr_4_2511906	Ca_desi_chr04	2511906	[A/G]	NA
Ca_Desi_Chr_4_2511911	Ca_desi_chr04	2511911	[A/G]	NA
Ca_Desi_Chr_4_2511913	Ca_desi_chr04	2511913	[A/G]	NA
Ca_Desi_Chr_4_2511924	Ca_desi_chr04	2511924	[T/G]	NA
Ca_Desi_Chr_4_2511932	Ca_desi_chr04	2511932	[A/G]	NA
Ca_Desi_Chr_4_2511937	Ca_desi_chr04	2511937	[T/C]	NA
Ca_Desi_Chr_4_2527581	Ca_desi_chr04	2527581	[G/A]	NA
Ca_Desi_Chr_4_2547432	Ca_desi_chr04	2547432	[T/C]	NA
Ca_Desi_Chr_4_2548472	Ca_desi_chr04	2548472	[T/A]	NA
Ca_Desi_Chr_4_2609258	Ca_desi_chr04	2609258	[T/G]	NA
Ca_Desi_Chr_4_2620933	Ca_desi_chr04	2620933	[T/A]	NA
Ca_Desi_Chr_4_2620965	Ca_desi_chr04	2620965	[T/G]	NA
Ca_Desi_Chr_4_2620974	Ca_desi_chr04	2620974	[A/G]	NA
Ca_Desi_Chr_4_2692233	Ca_desi_chr04	2692233	[C/A]	NA
Ca_Desi_Chr_4_2692234	Ca_desi_chr04	2692234	[T/G]	NA
Ca_Desi_Chr_4_2692243	Ca_desi_chr04	2692243	[T/A]	NA
Ca_Desi_Chr_4_2694573	Ca_desi_chr04	2694573	[A/C]	NA
Ca_Desi_Chr_4_2750818	Ca_desi_chr04	2750818	[C/T]	NA
Ca_Desi_Chr_4_2750844	Ca_desi_chr04	2750844	[G/A]	NA

SNP IDs	Chromosomes	Physical positions (bp)	SNPs	NCBI SNPdb accession IDs (NCBI_ss#)
Ca_Desi_Chr_4_2750863	Ca_desi_chr04	2750863	[A/C]	NA
Ca_Desi_Chr_4_2751571	Ca_desi_chr04	2751571	[G/T]	NA
Ca_Desi_Chr_4_2761588	Ca_desi_chr04	2761588	[C/T]	NA
Ca_Desi_Chr_4_2761592	Ca_desi_chr04	2761592	[G/A]	NA
Ca_Desi_Chr_4_2761601	Ca_desi_chr04	2761601	[A/G]	NA
Ca_Desi_Chr_4_2793481	Ca_desi_chr04	2793481	[G/A]	NA
Ca_Desi_Chr_4_2812659	Ca_desi_chr04	2812659	[T/G]	NA
Ca_Desi_Chr_4_2812775	Ca_desi_chr04	2812775	[C/T]	NA
Ca_Desi_Chr_4_2873185	Ca_desi_chr04	2873185	[C/T]	NA
Ca_Desi_Chr_4_2882614	Ca_desi_chr04	2882614	[T/A]	NA
Ca_Desi_Chr_4_2882615	Ca_desi_chr04	2882615	[A/C]	NA
Ca_Desi_Chr_4_2906536	Ca_desi_chr04	2906536	[A/T]	NA
Ca_Desi_Chr_4_2977378	Ca_desi_chr04	2977378	[C/T]	NA
Ca_Desi_Chr_4_2978413	Ca_desi_chr04	2978413	[T/C]	NA
Ca_Desi_Chr_4_2978459	Ca_desi_chr04	2978459	[A/T]	NA
Ca_Desi_Chr_4_2987115	Ca_desi_chr04	2987115	[A/T]	NA
Ca_Desi_Chr_4_2990741	Ca_desi_chr04	2990741	[T/A]	NA
Ca_Desi_Chr_4_2991777	Ca_desi_chr04	2991777	[A/C]	NA
Ca_Desi_Chr_4_2991804	Ca_desi_chr04	2991804	[C/A]	NA
Ca_Desi_Chr_4_2997005	Ca_desi_chr04	2997005	[C/T]	NA
Ca_Desi_Chr_4_2997017	Ca_desi_chr04	2997017	[C/T]	NA
Ca_Desi_Chr_4_2997039	Ca_desi_chr04	2997039	[G/C]	NA
Ca_Desi_Chr_4_2997431	Ca_desi_chr04	2997431	[C/T]	NA
Ca_Desi_Chr_4_3009470	Ca_desi_chr04	3009470	[A/G]	NA
Ca_Desi_Chr_4_3009471	Ca_desi_chr04	3009471	[C/T]	NA
Ca_Desi_Chr_4_3010593	Ca_desi_chr04	3010593	[A/G]	NA
Ca_Desi_Chr_4_3012464	Ca_desi_chr04	3012464	[G/C]	NA
Ca_Desi_Chr_4_3035000	Ca_desi_chr04	3035000	[A/T]	NA
Ca_Desi_Chr_4_3035085	Ca_desi_chr04	3035085	[C/T]	NA
Ca_Desi_Chr_4_3035131	Ca_desi_chr04	3035131	[G/C]	NA
Ca_Desi_Chr_4_3055686	Ca_desi_chr04	3055686	[A/C]	NA
Ca_Desi_Chr_4_3055803	Ca_desi_chr04	3055803	[T/C]	NA
Ca_Desi_Chr_4_3055868	Ca_desi_chr04	3055868	[T/C]	NA
Ca_Desi_Chr_4_3057462	Ca_desi_chr04	3057462	[A/G]	NA

SNP IDs	Chromosomes	Physical positions (bp)	SNPs	NCBI SNPdb accession IDs (NCBI_ss#)
Ca_Desi_Chr_4_3063851	Ca_desi_chr04	3063851	[G/T]	NA
Ca_Desi_Chr_4_3063929	Ca_desi_chr04	3063929	[C/T]	NA
Ca_Desi_Chr_4_3070931	Ca_desi_chr04	3070931	[G/A]	NA
Ca_Desi_Chr_4_3076119	Ca_desi_chr04	3076119	[G/A]	NA
Ca_Desi_Chr_4_3077529	Ca_desi_chr04	3077529	[C/A]	NA
Ca_Desi_Chr_4_3077536	Ca_desi_chr04	3077536	[A/G]	NA
Ca_Desi_Chr_4_3077546	Ca_desi_chr04	3077546	[A/G]	NA
Ca_Desi_Chr_4_3077547	Ca_desi_chr04	3077547	[A/G]	NA
Ca_Desi_Chr_4_3077570	Ca_desi_chr04	3077570	[A/G]	NA
Ca_Desi_Chr_4_3081906	Ca_desi_chr04	3081906	[T/C]	NA
Ca_Desi_Chr_4_3123418	Ca_desi_chr04	3123418	[C/T]	NA
Ca_Desi_Chr_4_3147919	Ca_desi_chr04	3147919	[A/G]	NA
Ca_Desi_Chr_4_3147921	Ca_desi_chr04	3147921	[A/C]	NA
Ca_Desi_Chr_4_3147922	Ca_desi_chr04	3147922	[C/A]	NA
Ca_Desi_Chr_4_3147925	Ca_desi_chr04	3147925	[T/C]	NA
Ca_Desi_Chr_4_3147933	Ca_desi_chr04	3147933	[A/C]	NA
Ca_Desi_Chr_4_3147939	Ca_desi_chr04	3147939	[A/G]	NA
Ca_Desi_Chr_4_3147959	Ca_desi_chr04	3147959	[T/C]	NA
Ca_Desi_Chr_4_3152370	Ca_desi_chr04	3152370	[A/C]	NA
Ca_Desi_Chr_4_3152371	Ca_desi_chr04	3152371	[G/C]	NA
Ca_Desi_Chr_4_3163412	Ca_desi_chr04	3163412	[A/G]	NA
Ca_Desi_Chr_4_3163422	Ca_desi_chr04	3163422	[A/G]	NA
Ca_Desi_Chr_4_3163432	Ca_desi_chr04	3163432	[A/C]	NA
Ca_Desi_Chr_4_3163449	Ca_desi_chr04	3163449	[T/G]	NA
Ca_Desi_Chr_4_3163451	Ca_desi_chr04	3163451	[T/C]	NA
Ca_Desi_Chr_4_3248622	Ca_desi_chr04	3248622	[A/G]	NA
Ca_Desi_Chr_4_3248640	Ca_desi_chr04	3248640	[T/A]	NA
Ca_Desi_Chr_4_3248802	Ca_desi_chr04	3248802	[T/G]	NA
Ca_Desi_Chr_4_3248832	Ca_desi_chr04	3248832	[T/G]	NA
Ca_Desi_Chr_4_3326279	Ca_desi_chr04	3326279	[G/A]	NA
Ca_Desi_Chr_4_3326281	Ca_desi_chr04	3326281	[T/G]	NA
Ca_Desi_Chr_4_3326283	Ca_desi_chr04	3326283	[A/G]	NA
Ca_Desi_Chr_4_3326298	Ca_desi_chr04	3326298	[T/G]	NA
Ca_Desi_Chr_4_3343775	Ca_desi_chr04	3343775	[T/C]	NA

SNP IDs	Chromosomes	Physical positions (bp)	SNPs	NCBI SNPdb accession IDs (NCBI_ss#)
Ca_Desi_Chr_4_3367557	Ca_desi_chr04	3367557	[A/G]	NA
Ca_Desi_Chr_4_3369431	Ca_desi_chr04	3369431	[T/G]	NA
Ca_Desi_Chr_4_3379061	Ca_desi_chr04	3379061	[G/A]	NA
Ca_Desi_Chr_4_3379084	Ca_desi_chr04	3379084	[T/C]	NA
Ca_Desi_Chr_4_3412898	Ca_desi_chr04	3412898	[A/C]	NA
Ca_Desi_Chr_4_3412921	Ca_desi_chr04	3412921	[A/G]	NA
Ca_Desi_Chr_4_3414893	Ca_desi_chr04	3414893	[T/G]	NA
Ca_Desi_Chr_4_3505903	Ca_desi_chr04	3505903	[T/G]	NA
Ca_Desi_Chr_4_3521277	Ca_desi_chr04	3521277	[A/G]	NA
Ca_Desi_Chr_4_3530482	Ca_desi_chr04	3530482	[A/G]	NA
Ca_Desi_Chr_4_3530510	Ca_desi_chr04	3530510	[A/C]	NA
Ca_Desi_Chr_4_3530522	Ca_desi_chr04	3530522	[A/C]	NA
Ca_Desi_Chr_4_3530532	Ca_desi_chr04	3530532	[A/G]	NA
Ca_Desi_Chr_4_3530535	Ca_desi_chr04	3530535	[T/C]	NA
Ca_Desi_Chr_4_3530537	Ca_desi_chr04	3530537	[T/A]	NA
Ca_Desi_Chr_4_3530546	Ca_desi_chr04	3530546	[T/C]	NA
Ca_Desi_Chr_4_3530549	Ca_desi_chr04	3530549	[T/A]	NA
Ca_Desi_Chr_4_3859413	Ca_desi_chr04	3859413	[T/C]	NA
Ca_Desi_Chr_4_3871014	Ca_desi_chr04	3871014	[A/C]	NA
Ca_Desi_Chr_4_3871024	Ca_desi_chr04	3871024	[A/G]	NA
Ca_Desi_Chr_4_3875097	Ca_desi_chr04	3875097	[T/C]	NA
Ca_Desi_Chr_4_3875100	Ca_desi_chr04	3875100	[C/A]	NA
Ca_Desi_Chr_4_3875312	Ca_desi_chr04	3875312	[A/C]	NA
Ca_Desi_Chr_4_3875332	Ca_desi_chr04	3875332	[G/A]	NA
Ca_Desi_Chr_4_3882524	Ca_desi_chr04	3882524	[T/C]	NA
Ca_Desi_Chr_4_3904046	Ca_desi_chr04	3904046	[T/G]	NA
Ca_Desi_Chr_4_3954836	Ca_desi_chr04	3954836	[G/A]	NA
Ca_Desi_Chr_4_3955413	Ca_desi_chr04	3955413	[T/G]	NA
Ca_Desi_Chr_4_3977839	Ca_desi_chr04	3977839	[A/C]	NA
Ca_Desi_Chr_4_3977996	Ca_desi_chr04	3977996	[C/T]	NA
Ca_Desi_Chr_4_3978024	Ca_desi_chr04	3978024	[T/C]	NA
Ca_Desi_Chr_4_3978121	Ca_desi_chr04	3978121	[C/G]	NA
Ca_Desi_Chr_4_4013763	Ca_desi_chr04	4013763	[A/T]	NA
Ca_Desi_Chr_4_4018921	Ca_desi_chr04	4018921	[G/A]	NA

SNP IDs	Chromosomes	Physical positions (bp)	SNPs	NCBI SNPdb accession IDs (NCBI_ss#)
Ca_Desi_Chr_4_4018931	Ca_desi_chr04	4018931	[A/G]	NA
Ca_Desi_Chr_4_4018936	Ca_desi_chr04	4018936	[A/G]	NA
Ca_Desi_Chr_4_4028415	Ca_desi_chr04	4028415	[A/G]	NA
Ca_Desi_Chr_4_4028417	Ca_desi_chr04	4028417	[A/G]	NA
Ca_Desi_Chr_4_4053275	Ca_desi_chr04	4053275	[T/C]	NA
Ca_Desi_Chr_4_4053375	Ca_desi_chr04	4053375	[C/A]	NA
Ca_Desi_Chr_4_4053376	Ca_desi_chr04	4053376	[T/G]	NA
Ca_Desi_Chr_4_4087180	Ca_desi_chr04	4087180	[A/G]	NA
Ca_Desi_Chr_4_4087191	Ca_desi_chr04	4087191	[A/G]	NA
Ca_Desi_Chr_4_4087209	Ca_desi_chr04	4087209	[C/T]	NA
Ca_Desi_Chr_4_4087248	Ca_desi_chr04	4087248	[T/G]	NA
Ca_Desi_Chr_4_4087269	Ca_desi_chr04	4087269	[C/T]	NA
Ca_Desi_Chr_4_4087272	Ca_desi_chr04	4087272	[C/T]	NA
Ca_Desi_Chr_4_4102802	Ca_desi_chr04	4102802	[C/T]	NA
Ca_Desi_Chr_4_4116022	Ca_desi_chr04	4116022	[T/C]	NA
Ca_Desi_Chr_4_4116300	Ca_desi_chr04	4116300	[A/G]	NA
Ca_Desi_Chr_4_4219005	Ca_desi_chr04	4219005	[A/C]	NA
Ca_Desi_Chr_4_4219027	Ca_desi_chr04	4219027	[A/C]	NA
Ca_Desi_Chr_4_4219042	Ca_desi_chr04	4219042	[A/C]	NA
Ca_Desi_Chr_4_4273941	Ca_desi_chr04	4273941	[A/C]	NA
Ca_Desi_Chr_4_4492198	Ca_desi_chr04	4492198	[A/C]	NA
Ca_Desi_Chr_4_4531349	Ca_desi_chr04	4531349	[T/G]	NA
Ca_Desi_Chr_4_4531357	Ca_desi_chr04	4531357	[A/G]	NA
Ca_Desi_Chr_4_4592740	Ca_desi_chr04	4592740	[T/A]	NA
Ca_Desi_Chr_4_4592803	Ca_desi_chr04	4592803	[A/T]	NA
Ca_Desi_Chr_4_4592814	Ca_desi_chr04	4592814	[T/G]	NA
Ca_Desi_Chr_4_4592822	Ca_desi_chr04	4592822	[T/C]	NA
Ca_Desi_Chr_4_4601772	Ca_desi_chr04	4601772	[A/T]	NA
Ca_Desi_Chr_4_4606607	Ca_desi_chr04	4606607	[A/G]	NA
Ca_Desi_Chr_4_4606612	Ca_desi_chr04	4606612	[A/G]	NA
Ca_Desi_Chr_4_4606617	Ca_desi_chr04	4606617	[A/G]	NA
Ca_Desi_Chr_4_4606623	Ca_desi_chr04	4606623	[T/G]	NA
Ca_Desi_Chr_4_4606631	Ca_desi_chr04	4606631	[A/C]	NA
Ca_Desi_Chr_4_4606650	Ca_desi_chr04	4606650	[A/C]	NA

SNP IDs	Chromosomes	Physical positions (bp)	SNPs	NCBI SNPdb accession IDs (NCBI_ss#)
Ca_Desi_Chr_4_4606668	Ca_desi_chr04	4606668	[T/C]	NA
Ca_Desi_Chr_4_4613305	Ca_desi_chr04	4613305	[A/G]	NA
Ca_Desi_Chr_4_4613309	Ca_desi_chr04	4613309	[T/C]	NA
Ca_Desi_Chr_4_4613333	Ca_desi_chr04	4613333	[T/C]	NA
Ca_Desi_Chr_4_4613619	Ca_desi_chr04	4613619	[A/C]	NA
Ca_Desi_Chr_4_4613639	Ca_desi_chr04	4613639	[A/C]	NA
Ca_Desi_Chr_4_4613648	Ca_desi_chr04	4613648	[A/C]	NA
Ca_Desi_Chr_4_4652989	Ca_desi_chr04	4652989	[A/G]	NA
Ca_Desi_Chr_4_4653036	Ca_desi_chr04	4653036	[A/C]	NA
Ca_Desi_Chr_4_4656183	Ca_desi_chr04	4656183	[C/A]	NA
Ca_Desi_Chr_4_4687385	Ca_desi_chr04	4687385	[T/C]	NA
Ca_Desi_Chr_4_4687388	Ca_desi_chr04	4687388	[C/G]	NA
Ca_Desi_Chr_4_4690429	Ca_desi_chr04	4690429	[T/G]	NA
Ca_Desi_Chr_4_4701443	Ca_desi_chr04	4701443	[T/A]	NA
Ca_Desi_Chr_4_4701545	Ca_desi_chr04	4701545	[C/G]	NA
Ca_Desi_Chr_4_4714619	Ca_desi_chr04	4714619	[A/C]	NA
Ca_Desi_Chr_4_4765059	Ca_desi_chr04	4765059	[A/T]	NA
Ca_Desi_Chr_4_4765096	Ca_desi_chr04	4765096	[C/G]	NA
Ca_Desi_Chr_4_4768275	Ca_desi_chr04	4768275	[T/G]	NA
Ca_Desi_Chr_4_4768373	Ca_desi_chr04	4768373	[T/C]	NA
Ca_Desi_Chr_4_4768397	Ca_desi_chr04	4768397	[C/A]	NA
Ca_Desi_Chr_4_4768614	Ca_desi_chr04	4768614	[G/A]	NA
Ca_Desi_Chr_4_4769567	Ca_desi_chr04	4769567	[G/A]	NA
Ca_Desi_Chr_4_4769570	Ca_desi_chr04	4769570	[G/A]	NA
Ca_Desi_Chr_4_4771448	Ca_desi_chr04	4771448	[T/C]	NA
Ca_Desi_Chr_4_4771473	Ca_desi_chr04	4771473	[T/A]	NA
Ca_Desi_Chr_4_4771490	Ca_desi_chr04	4771490	[C/T]	NA
Ca_Desi_Chr_4_4771528	Ca_desi_chr04	4771528	[G/T]	NA
Ca_Desi_Chr_4_4771544	Ca_desi_chr04	4771544	[A/C]	NA
Ca_Desi_Chr_4_4771545	Ca_desi_chr04	4771545	[C/A]	NA
Ca_Desi_Chr_4_4771546	Ca_desi_chr04	4771546	[A/C]	NA
Ca_Desi_Chr_4_4771548	Ca_desi_chr04	4771548	[A/T]	NA
Ca_Desi_Chr_4_4771549	Ca_desi_chr04	4771549	[T/A]	NA
Ca_Desi_Chr_4_4771738	Ca_desi_chr04	4771738	[T/C]	NA

SNP IDs	Chromosomes	Physical positions (bp)	SNPs	NCBI SNPdb accession IDs (NCBI_ss#)
Ca_Desi_Chr_4_4817421	Ca_desi_chr04	4817421	[T/C]	NA
Ca_Desi_Chr_4_4817433	Ca_desi_chr04	4817433	[C/G]	NA
Ca_Desi_Chr_4_4817578	Ca_desi_chr04	4817578	[T/G]	NA
Ca_Desi_Chr_4_4817638	Ca_desi_chr04	4817638	[T/C]	NA
Ca_Desi_Chr_4_4837373	Ca_desi_chr04	4837373	[T/G]	NA
Ca_Desi_Chr_4_4837602	Ca_desi_chr04	4837602	[A/G]	NA
Ca_Desi_Chr_4_4837616	Ca_desi_chr04	4837616	[A/G]	NA
Ca_Desi_Chr_4_4848482	Ca_desi_chr04	4848482	[A/T]	NA
Ca_Desi_Chr_4_4848730	Ca_desi_chr04	4848730	[G/A]	NA
Ca_Desi_Chr_4_4848762	Ca_desi_chr04	4848762	[G/A]	NA
Ca_Desi_Chr_4_4878419	Ca_desi_chr04	4878419	[C/T]	NA
Ca_Desi_Chr_4_4878479	Ca_desi_chr04	4878479	[G/A]	NA
Ca_Desi_Chr_4_4884064	Ca_desi_chr04	4884064	[T/C]	NA
Ca_Desi_Chr_4_4905849	Ca_desi_chr04	4905849	[G/A]	NA
Ca_Desi_Chr_4_4909312	Ca_desi_chr04	4909312	[C/A]	NA
Ca_Desi_Chr_4_4913686	Ca_desi_chr04	4913686	[C/G]	NA
Ca_Desi_Chr_4_4936836	Ca_desi_chr04	4936836	[G/A]	NA
Ca_Desi_Chr_4_4939952	Ca_desi_chr04	4939952	[C/T]	NA
Ca_Desi_Chr_4_4955046	Ca_desi_chr04	4955046	[C/G]	NA
Ca_Desi_Chr_4_4955066	Ca_desi_chr04	4955066	[A/G]	NA
Ca_Desi_Chr_4_4955282	Ca_desi_chr04	4955282	[T/A]	NA
Ca_Desi_Chr_4_5027928	Ca_desi_chr04	5027928	[C/T]	NA
Ca_Desi_Chr_4_5028184	Ca_desi_chr04	5028184	[C/T]	NA
Ca_Desi_Chr_4_5096410	Ca_desi_chr04	5096410	[A/G]	NA
Ca_Desi_Chr_4_5096422	Ca_desi_chr04	5096422	[T/A]	NA
Ca_Desi_Chr_4_5097092	Ca_desi_chr04	5097092	[G/A]	NA
Ca_Desi_Chr_4_5097206	Ca_desi_chr04	5097206	[C/A]	NA
Ca_Desi_Chr_4_5183254	Ca_desi_chr04	5183254	[A/G]	NA
Ca_Desi_Chr_4_5407138	Ca_desi_chr04	5407138	[A/G]	NA
Ca_Desi_Chr_4_5407150	Ca_desi_chr04	5407150	[T/A]	NA
Ca_Desi_Chr_4_5408655	Ca_desi_chr04	5408655	[C/T]	NA
Ca_Desi_Chr_4_5408962	Ca_desi_chr04	5408962	[C/T]	NA
Ca_Desi_Chr_4_5410884	Ca_desi_chr04	5410884	[C/T]	NA
Ca_Desi_Chr_4_5533565	Ca_desi_chr04	5533565	[A/T]	NA

SNP IDs	Chromosomes	Physical positions (bp)	SNPs	NCBI SNPdb accession IDs (NCBI_ss#)
Ca_Desi_Chr_4_5533572	Ca_desi_chr04	5533572	[A/C]	NA
Ca_Desi_Chr_4_5533601	Ca_desi_chr04	5533601	[A/T]	NA
Ca_Desi_Chr_4_5571122	Ca_desi_chr04	5571122	[T/C]	NA
Ca_Desi_Chr_4_5571123	Ca_desi_chr04	5571123	[T/C]	NA
Ca_Desi_Chr_4_5571135	Ca_desi_chr04	5571135	[A/G]	NA
Ca_Desi_Chr_4_5571162	Ca_desi_chr04	5571162	[T/G]	NA
Ca_Desi_Chr_4_5571170	Ca_desi_chr04	5571170	[A/G]	NA
Ca_Desi_Chr_4_5610460	Ca_desi_chr04	5610460	[A/C]	NA
Ca_Desi_Chr_4_5610468	Ca_desi_chr04	5610468	[G/T]	NA
Ca_Desi_Chr_4_5610487	Ca_desi_chr04	5610487	[C/T]	NA
Ca_Desi_Chr_4_5610496	Ca_desi_chr04	5610496	[A/G]	NA
Ca_Desi_Chr_4_5678555	Ca_desi_chr04	5678555	[T/A]	NA
Ca_Desi_Chr_4_5678597	Ca_desi_chr04	5678597	[A/G]	NA
Ca_Desi_Chr_4_5678668	Ca_desi_chr04	5678668	[A/G]	NA
Ca_Desi_Chr_4_5678675	Ca_desi_chr04	5678675	[T/C]	NA
Ca_Desi_Chr_4_5678686	Ca_desi_chr04	5678686	[G/C]	NA
Ca_Desi_Chr_4_5678688	Ca_desi_chr04	5678688	[A/G]	NA
Ca_Desi_Chr_4_5678690	Ca_desi_chr04	5678690	[C/G]	NA
Ca_Desi_Chr_4_5678693	Ca_desi_chr04	5678693	[T/G]	NA
Ca_Desi_Chr_4_5678699	Ca_desi_chr04	5678699	[T/G]	NA
Ca_Desi_Chr_4_5678700	Ca_desi_chr04	5678700	[G/T]	NA
Ca_Desi_Chr_4_5678708	Ca_desi_chr04	5678708	[T/A]	NA
Ca_Desi_Chr_4_5755799	Ca_desi_chr04	5755799	[T/C]	NA
Ca_Desi_Chr_4_5755812	Ca_desi_chr04	5755812	[C/T]	NA
Ca_Desi_Chr_4_5755819	Ca_desi_chr04	5755819	[A/G]	NA
Ca_Desi_Chr_4_5755829	Ca_desi_chr04	5755829	[A/C]	NA
Ca_Desi_Chr_4_5782799	Ca_desi_chr04	5782799	[G/C]	NA
Ca_Desi_Chr_4_5798390	Ca_desi_chr04	5798390	[T/G]	NA
Ca_Desi_Chr_4_5800667	Ca_desi_chr04	5800667	[A/G]	NA
Ca_Desi_Chr_4_5800696	Ca_desi_chr04	5800696	[A/G]	NA
Ca_Desi_Chr_4_5854578	Ca_desi_chr04	5854578	[T/G]	NA
Ca_Desi_Chr_4_5854587	Ca_desi_chr04	5854587	[A/G]	NA
Ca_Desi_Chr_4_5854618	Ca_desi_chr04	5854618	[A/C]	NA
Ca_Desi_Chr_4_6008650	Ca_desi_chr04	6008650	[T/G]	NA

SNP IDs	Chromosomes	Physical positions (bp)	SNPs	NCBI SNPdb accession IDs (NCBI_ss#)
Ca_Desi_Chr_4_6017239	Ca_desi_chr04	6017239	[A/G]	NA
Ca_Desi_Chr_4_6017285	Ca_desi_chr04	6017285	[A/G]	NA
Ca_Desi_Chr_4_6020659	Ca_desi_chr04	6020659	[A/G]	NA
Ca_Desi_Chr_4_6110371	Ca_desi_chr04	6110371	[A/G]	NA
Ca_Desi_Chr_4_6145876	Ca_desi_chr04	6145876	[A/G]	NA
Ca_Desi_Chr_4_6278532	Ca_desi_chr04	6278532	[T/C]	NA
Ca_Desi_Chr_4_6278534	Ca_desi_chr04	6278534	[A/G]	NA
Ca_Desi_Chr_4_6278538	Ca_desi_chr04	6278538	[A/T]	NA
Ca_Desi_Chr_4_6278542	Ca_desi_chr04	6278542	[T/C]	NA
Ca_Desi_Chr_4_6278576	Ca_desi_chr04	6278576	[T/C]	NA
Ca_Desi_Chr_4_6278587	Ca_desi_chr04	6278587	[C/A]	NA
Ca_Desi_Chr_4_6318869	Ca_desi_chr04	6318869	[T/G]	NA
Ca_Desi_Chr_4_6325212	Ca_desi_chr04	6325212	[T/C]	NA
Ca_Desi_Chr_4_6325213	Ca_desi_chr04	6325213	[C/T]	NA
Ca_Desi_Chr_4_6325277	Ca_desi_chr04	6325277	[G/T]	NA
Ca_Desi_Chr_4_6507283	Ca_desi_chr04	6507283	[T/G]	NA
Ca_Desi_Chr_4_6511894	Ca_desi_chr04	6511894	[A/G]	NA
Ca_Desi_Chr_4_6511920	Ca_desi_chr04	6511920	[T/A]	NA
Ca_Desi_Chr_4_6511927	Ca_desi_chr04	6511927	[A/G]	NA
Ca_Desi_Chr_4_6512146	Ca_desi_chr04	6512146	[A/C]	NA
Ca_Desi_Chr_4_6512153	Ca_desi_chr04	6512153	[A/G]	NA
Ca_Desi_Chr_4_6512163	Ca_desi_chr04	6512163	[A/C]	NA
Ca_Desi_Chr_4_6512183	Ca_desi_chr04	6512183	[C/G]	NA
Ca_Desi_Chr_4_6512185	Ca_desi_chr04	6512185	[A/G]	NA
Ca_Desi_Chr_4_6512187	Ca_desi_chr04	6512187	[T/C]	NA
Ca_Desi_Chr_4_6521261	Ca_desi_chr04	6521261	[T/G]	NA
Ca_Desi_Chr_4_6521349	Ca_desi_chr04	6521349	[T/C]	NA
Ca_Desi_Chr_4_6521359	Ca_desi_chr04	6521359	[T/A]	NA
Ca_Desi_Chr_4_6798716	Ca_desi_chr04	6798716	[T/G]	NA
Ca_Desi_Chr_4_6935117	Ca_desi_chr04	6935117	[G/A]	NA
Ca_Desi_Chr_4_6935151	Ca_desi_chr04	6935151	[A/G]	NA
Ca_Desi_Chr_4_6935214	Ca_desi_chr04	6935214	[T/C]	NA
Ca_Desi_Chr_4_6940879	Ca_desi_chr04	6940879	[A/G]	NA
Ca_Desi_Chr_4_6940881	Ca_desi_chr04	6940881	[T/C]	NA

SNP IDs	Chromosomes	Physical positions (bp)	SNPs	NCBI SNPdb accession IDs (NCBI_ss#)
Ca_Desi_Chr_4_7035560	Ca_desi_chr04	7035560	[A/C]	NA
Ca_Desi_Chr_4_7035565	Ca_desi_chr04	7035565	[A/G]	NA
Ca_Desi_Chr_4_7035566	Ca_desi_chr04	7035566	[C/T]	NA
Ca_Desi_Chr_4_7153280	Ca_desi_chr04	7153280	[A/G]	NA
Ca_Desi_Chr_4_7153308	Ca_desi_chr04	7153308	[G/T]	NA
Ca_Desi_Chr_4_7153309	Ca_desi_chr04	7153309	[T/G]	NA
Ca_Desi_Chr_4_7156704	Ca_desi_chr04	7156704	[A/G]	NA
Ca_Desi_Chr_4_7218308	Ca_desi_chr04	7218308	[T/C]	NA
Ca_Desi_Chr_4_7218328	Ca_desi_chr04	7218328	[A/G]	NA
Ca_Desi_Chr_4_7218347	Ca_desi_chr04	7218347	[A/G]	NA
Ca_Desi_Chr_4_7218357	Ca_desi_chr04	7218357	[A/G]	NA
Ca_Desi_Chr_4_7225087	Ca_desi_chr04	7225087	[T/A]	NA
Ca_Desi_Chr_4_7225109	Ca_desi_chr04	7225109	[A/C]	NA
Ca_Desi_Chr_4_7225116	Ca_desi_chr04	7225116	[T/G]	NA
Ca_Desi_Chr_4_7262111	Ca_desi_chr04	7262111	[T/A]	NA
Ca_Desi_Chr_4_7267078	Ca_desi_chr04	7267078	[T/G]	NA
Ca_Desi_Chr_4_7267110	Ca_desi_chr04	7267110	[T/G]	NA
Ca_Desi_Chr_4_7347313	Ca_desi_chr04	7347313	[A/G]	NA
Ca_Desi_Chr_4_7353061	Ca_desi_chr04	7353061	[A/T]	NA
Ca_Desi_Chr_4_7353066	Ca_desi_chr04	7353066	[G/T]	NA
Ca_Desi_Chr_4_7356849	Ca_desi_chr04	7356849	[A/C]	NA
Ca_Desi_Chr_4_7395723	Ca_desi_chr04	7395723	[T/C]	NA
Ca_Desi_Chr_4_7406408	Ca_desi_chr04	7406408	[G/C]	NA
Ca_Desi_Chr_4_7411622	Ca_desi_chr04	7411622	[A/C]	NA
Ca_Desi_Chr_4_7411626	Ca_desi_chr04	7411626	[T/A]	NA
Ca_Desi_Chr_4_7411629	Ca_desi_chr04	7411629	[A/G]	NA
Ca_Desi_Chr_4_7411730	Ca_desi_chr04	7411730	[A/C]	NA
Ca_Desi_Chr_4_7411732	Ca_desi_chr04	7411732	[T/C]	NA
Ca_Desi_Chr_4_7411736	Ca_desi_chr04	7411736	[T/C]	NA
Ca_Desi_Chr_4_7411738	Ca_desi_chr04	7411738	[A/G]	NA
Ca_Desi_Chr_4_7411741	Ca_desi_chr04	7411741	[A/G]	NA
Ca_Desi_Chr_4_7411743	Ca_desi_chr04	7411743	[G/A]	NA
Ca_Desi_Chr_4_7411744	Ca_desi_chr04	7411744	[A/G]	NA
Ca_Desi_Chr_4_7411750	Ca_desi_chr04	7411750	[T/C]	NA

SNP IDs	Chromosomes	Physical positions (bp)	SNPs	NCBI SNPdb accession IDs (NCBI_ss#)
Ca_Desi_Chr_4_7411755	Ca_desi_chr04	7411755	[A/G]	NA
Ca_Desi_Chr_4_7411761	Ca_desi_chr04	7411761	[A/G]	NA
Ca_Desi_Chr_4_7491358	Ca_desi_chr04	7491358	[C/A]	NA
Ca_Desi_Chr_4_7509507	Ca_desi_chr04	7509507	[A/G]	NA
Ca_Desi_Chr_4_7509510	Ca_desi_chr04	7509510	[A/G]	NA
Ca_Desi_Chr_4_7519141	Ca_desi_chr04	7519141	[A/C]	NA
Ca_Desi_Chr_4_7575953	Ca_desi_chr04	7575953	[T/G]	NA
Ca_Desi_Chr_4_7580290	Ca_desi_chr04	7580290	[C/A]	NA
Ca_Desi_Chr_4_7580291	Ca_desi_chr04	7580291	[C/G]	NA
Ca_Desi_Chr_4_7580292	Ca_desi_chr04	7580292	[G/A]	NA
Ca_Desi_Chr_4_7580293	Ca_desi_chr04	7580293	[C/T]	NA
Ca_Desi_Chr_4_7580307	Ca_desi_chr04	7580307	[A/T]	NA
Ca_Desi_Chr_4_7580319	Ca_desi_chr04	7580319	[T/G]	NA
Ca_Desi_Chr_4_7601426	Ca_desi_chr04	7601426	[G/T]	NA
Ca_Desi_Chr_4_7601513	Ca_desi_chr04	7601513	[T/C]	NA
Ca_Desi_Chr_4_7601528	Ca_desi_chr04	7601528	[T/G]	NA
Ca_Desi_Chr_4_7601616	Ca_desi_chr04	7601616	[G/A]	NA
Ca_Desi_Chr_4_7601652	Ca_desi_chr04	7601652	[C/G]	NA
Ca_Desi_Chr_4_7601659	Ca_desi_chr04	7601659	[A/G]	NA
Ca_Desi_Chr_4_7601661	Ca_desi_chr04	7601661	[A/G]	NA
Ca_Desi_Chr_4_7601671	Ca_desi_chr04	7601671	[A/G]	NA
Ca_Desi_Chr_4_7601672	Ca_desi_chr04	7601672	[G/A]	NA
Ca_Desi_Chr_4_7601673	Ca_desi_chr04	7601673	[A/G]	NA
Ca_Desi_Chr_4_7601679	Ca_desi_chr04	7601679	[T/G]	NA
Ca_Desi_Chr_4_7623385	Ca_desi_chr04	7623385	[A/C]	NA
Ca_Desi_Chr_4_7625428	Ca_desi_chr04	7625428	[A/G]	NA
Ca_Desi_Chr_4_7625450	Ca_desi_chr04	7625450	[T/A]	NA
Ca_Desi_Chr_4_7630664	Ca_desi_chr04	7630664	[A/C]	NA
Ca_Desi_Chr_4_7647658	Ca_desi_chr04	7647658	[G/T]	NA
Ca_Desi_Chr_4_7649928	Ca_desi_chr04	7649928	[A/C]	NA
Ca_Desi_Chr_4_7649941	Ca_desi_chr04	7649941	[A/C]	NA
Ca_Desi_Chr_4_7649960	Ca_desi_chr04	7649960	[A/G]	NA
Ca_Desi_Chr_4_7666026	Ca_desi_chr04	7666026	[T/C]	NA
Ca_Desi_Chr_4_7674762	Ca_desi_chr04	7674762	[A/C]	NA

SNP IDs	Chromosomes	Physical positions (bp)	SNPs	NCBI SNPdb accession IDs (NCBI_ss#)
Ca_Desi_Chr_4_7680251	Ca_desi_chr04	7680251	[A/C]	NA
Ca_Desi_Chr_4_7730453	Ca_desi_chr04	7730453	[A/T]	NA
Ca_Desi_Chr_4_7778309	Ca_desi_chr04	7778309	[G/A]	NA
Ca_Desi_Chr_4_7778310	Ca_desi_chr04	7778310	[T/G]	NA
Ca_Desi_Chr_4_7780206	Ca_desi_chr04	7780206	[T/A]	NA
Ca_Desi_Chr_4_7780208	Ca_desi_chr04	7780208	[C/A]	NA
Ca_Desi_Chr_4_7781961	Ca_desi_chr04	7781961	[T/C]	NA
Ca_Desi_Chr_4_7781965	Ca_desi_chr04	7781965	[T/C]	NA
Ca_Desi_Chr_4_7781974	Ca_desi_chr04	7781974	[T/C]	NA
Ca_Desi_Chr_4_7781980	Ca_desi_chr04	7781980	[A/G]	NA
Ca_Desi_Chr_4_7782109	Ca_desi_chr04	7782109	[A/C]	NA
Ca_Desi_Chr_4_7783322	Ca_desi_chr04	7783322	[T/G]	NA
Ca_Desi_Chr_4_7809791	Ca_desi_chr04	7809791	[A/G]	NA
Ca_Desi_Chr_4_7809793	Ca_desi_chr04	7809793	[G/A]	NA
Ca_Desi_Chr_4_7812668	Ca_desi_chr04	7812668	[A/G]	NA
Ca_Desi_Chr_4_7813621	Ca_desi_chr04	7813621	[T/C]	NA
Ca_Desi_Chr_4_7813648	Ca_desi_chr04	7813648	[T/C]	NA
Ca_Desi_Chr_4_7813664	Ca_desi_chr04	7813664	[A/G]	NA
Ca_Desi_Chr_4_7823729	Ca_desi_chr04	7823729	[G/T]	NA
Ca_Desi_Chr_4_7860994	Ca_desi_chr04	7860994	[T/A]	NA
Ca_Desi_Chr_4_7861017	Ca_desi_chr04	7861017	[A/G]	NA
Ca_Desi_Chr_4_7885258	Ca_desi_chr04	7885258	[A/G]	NA
Ca_Desi_Chr_4_7885263	Ca_desi_chr04	7885263	[T/G]	NA
Ca_Desi_Chr_4_7885266	Ca_desi_chr04	7885266	[T/G]	NA
Ca_Desi_Chr_4_7886657	Ca_desi_chr04	7886657	[T/C]	NA
Ca_Desi_Chr_4_7886664	Ca_desi_chr04	7886664	[T/A]	NA
Ca_Desi_Chr_4_7886670	Ca_desi_chr04	7886670	[A/G]	NA
Ca_Desi_Chr_4_7886677	Ca_desi_chr04	7886677	[T/C]	NA
Ca_Desi_Chr_4_7886695	Ca_desi_chr04	7886695	[T/C]	NA
Ca_Desi_Chr_4_7886707	Ca_desi_chr04	7886707	[A/G]	NA
Ca_Desi_Chr_4_7886711	Ca_desi_chr04	7886711	[A/T]	NA
Ca_Desi_Chr_4_7934811	Ca_desi_chr04	7934811	[C/A]	NA
Ca_Desi_Chr_4_7934812	Ca_desi_chr04	7934812	[C/G]	NA
Ca_Desi_Chr_4_7934814	Ca_desi_chr04	7934814	[G/T]	NA

SNP IDs	Chromosomes	Physical positions (bp)	SNPs	NCBI SNPdb accession IDs (NCBI_ss#)
Ca_Desi_Chr_4_7934815	Ca_desi_chr04	7934815	[G/C]	NA
Ca_Desi_Chr_4_7983047	Ca_desi_chr04	7983047	[T/A]	NA
Ca_Desi_Chr_4_8009915	Ca_desi_chr04	8009915	[T/C]	NA
Ca_Desi_Chr_4_8038003	Ca_desi_chr04	8038003	[A/G]	NA
Ca_Desi_Chr_4_8038010	Ca_desi_chr04	8038010	[A/C]	NA
Ca_Desi_Chr_4_8063854	Ca_desi_chr04	8063854	[T/G]	NA
Ca_Desi_Chr_4_8066412	Ca_desi_chr04	8066412	[A/G]	NA
Ca_Desi_Chr_4_8066455	Ca_desi_chr04	8066455	[A/G]	NA
Ca_Desi_Chr_4_8066464	Ca_desi_chr04	8066464	[T/C]	NA
Ca_Desi_Chr_4_8066468	Ca_desi_chr04	8066468	[T/C]	NA
Ca_Desi_Chr_4_8108885	Ca_desi_chr04	8108885	[T/C]	NA
Ca_Desi_Chr_4_8130436	Ca_desi_chr04	8130436	[A/G]	NA
Ca_Desi_Chr_4_8131032	Ca_desi_chr04	8131032	[T/G]	NA
Ca_Desi_Chr_4_8131044	Ca_desi_chr04	8131044	[T/G]	NA
Ca_Desi_Chr_4_8131052	Ca_desi_chr04	8131052	[T/G]	NA
Ca_Desi_Chr_4_8131067	Ca_desi_chr04	8131067	[A/C]	NA
Ca_Desi_Chr_4_8131353	Ca_desi_chr04	8131353	[T/G]	NA
Ca_Desi_Chr_4_8131359	Ca_desi_chr04	8131359	[T/C]	NA
Ca_Desi_Chr_4_8131371	Ca_desi_chr04	8131371	[T/C]	NA
Ca_Desi_Chr_4_8131375	Ca_desi_chr04	8131375	[T/C]	NA
Ca_Desi_Chr_4_8131384	Ca_desi_chr04	8131384	[T/G]	NA
Ca_Desi_Chr_4_8131395	Ca_desi_chr04	8131395	[T/C]	NA
Ca_Desi_Chr_4_8131398	Ca_desi_chr04	8131398	[C/G]	NA
Ca_Desi_Chr_4_8131401	Ca_desi_chr04	8131401	[G/C]	NA
Ca_Desi_Chr_4_8141384	Ca_desi_chr04	8141384	[A/G]	NA
Ca_Desi_Chr_4_8146783	Ca_desi_chr04	8146783	[A/G]	NA
Ca_Desi_Chr_4_8146813	Ca_desi_chr04	8146813	[T/G]	NA
Ca_Desi_Chr_4_8149684	Ca_desi_chr04	8149684	[T/G]	NA
Ca_Desi_Chr_4_8149814	Ca_desi_chr04	8149814	[C/A]	NA
Ca_Desi_Chr_4_8150574	Ca_desi_chr04	8150574	[A/T]	NA
Ca_Desi_Chr_4_8150597	Ca_desi_chr04	8150597	[A/G]	NA
Ca_Desi_Chr_4_8192799	Ca_desi_chr04	8192799	[A/G]	NA
Ca_Desi_Chr_4_8198525	Ca_desi_chr04	8198525	[T/G]	NA
Ca_Desi_Chr_4_8198540	Ca_desi_chr04	8198540	[A/C]	NA

SNP IDs	Chromosomes	Physical positions (bp)	SNPs	NCBI SNPdb accession IDs (NCBI_ss#)
Ca_Desi_Chr_4_8198544	Ca_desi_chr04	8198544	[T/C]	NA
Ca_Desi_Chr_4_8199122	Ca_desi_chr04	8199122	[T/A]	NA
Ca_Desi_Chr_4_8311192	Ca_desi_chr04	8311192	[T/G]	NA
Ca_Desi_Chr_4_8311201	Ca_desi_chr04	8311201	[A/C]	NA
Ca_Desi_Chr_4_8314144	Ca_desi_chr04	8314144	[T/C]	NA
Ca_Desi_Chr_4_8323074	Ca_desi_chr04	8323074	[A/C]	NA
Ca_Desi_Chr_4_8363099	Ca_desi_chr04	8363099	[A/C]	NA
Ca_Desi_Chr_4_8393192	Ca_desi_chr04	8393192	[T/G]	NA
Ca_Desi_Chr_4_8413938	Ca_desi_chr04	8413938	[T/G]	NA
Ca_Desi_Chr_4_8413940	Ca_desi_chr04	8413940	[T/A]	NA
Ca_Desi_Chr_4_8536781	Ca_desi_chr04	8536781	[G/A]	NA
Ca_Desi_Chr_4_8536783	Ca_desi_chr04	8536783	[T/A]	NA
Ca_Desi_Chr_4_8848071	Ca_desi_chr04	8848071	[T/C]	NA
Ca_Desi_Chr_4_8848274	Ca_desi_chr04	8848274	[C/T]	NA
Ca_Desi_Chr_4_8848318	Ca_desi_chr04	8848318	[T/G]	NA
Ca_Desi_Chr_4_8866108	Ca_desi_chr04	8866108	[G/A]	NA
Ca_Desi_Chr_4_8876149	Ca_desi_chr04	8876149	[G/A]	NA
Ca_Desi_Chr_4_8884356	Ca_desi_chr04	8884356	[G/A]	NA
Ca_Desi_Chr_4_8884501	Ca_desi_chr04	8884501	[T/A]	NA
Ca_Desi_Chr_4_8884795	Ca_desi_chr04	8884795	[G/T]	NA
Ca_Desi_Chr_4_8891953	Ca_desi_chr04	8891953	[A/G]	NA
Ca_Desi_Chr_4_8892359	Ca_desi_chr04	8892359	[A/G]	NA
Ca_Desi_Chr_4_8892369	Ca_desi_chr04	8892369	[T/C]	NA
Ca_Desi_Chr_4_8892411	Ca_desi_chr04	8892411	[A/C]	NA
Ca_Desi_Chr_4_8908491	Ca_desi_chr04	8908491	[A/T]	NA
Ca_Desi_Chr_4_8913646	Ca_desi_chr04	8913646	[A/G]	NA
Ca_Desi_Chr_4_8914102	Ca_desi_chr04	8914102	[G/A]	NA
Ca_Desi_Chr_4_9004327	Ca_desi_chr04	9004327	[A/G]	NA
Ca_Desi_Chr_4_9009754	Ca_desi_chr04	9009754	[T/G]	NA
Ca_Desi_Chr_4_9009757	Ca_desi_chr04	9009757	[T/G]	NA
Ca_Desi_Chr_4_9098900	Ca_desi_chr04	9098900	[T/G]	NA
Ca_Desi_Chr_4_9109103	Ca_desi_chr04	9109103	[T/C]	NA
Ca_Desi_Chr_4_9179824	Ca_desi_chr04	9179824	[T/G]	NA
Ca_Desi_Chr_4_9179853	Ca_desi_chr04	9179853	[A/C]	NA

SNP IDs	Chromosomes	Physical positions (bp)	SNPs	NCBI SNPdb accession IDs (NCBI_ss#)
Ca_Desi_Chr_4_9181097	Ca_desi_chr04	9181097	[T/A]	NA
Ca_Desi_Chr_4_9181115	Ca_desi_chr04	9181115	[A/G]	NA
Ca_Desi_Chr_4_9219351	Ca_desi_chr04	9219351	[T/A]	NA
Ca_Desi_Chr_4_9222551	Ca_desi_chr04	9222551	[T/A]	NA
Ca_Desi_Chr_4_9224483	Ca_desi_chr04	9224483	[T/G]	NA
Ca_Desi_Chr_4_9224606	Ca_desi_chr04	9224606	[A/G]	NA
Ca_Desi_Chr_4_9224613	Ca_desi_chr04	9224613	[A/C]	NA
Ca_Desi_Chr_4_9225020	Ca_desi_chr04	9225020	[T/G]	NA
Ca_Desi_Chr_4_9225407	Ca_desi_chr04	9225407	[T/C]	NA
Ca_Desi_Chr_4_9225412	Ca_desi_chr04	9225412	[T/G]	NA
Ca_Desi_Chr_4_9225421	Ca_desi_chr04	9225421	[T/G]	NA
Ca_Desi_Chr_4_9225429	Ca_desi_chr04	9225429	[T/G]	NA
Ca_Desi_Chr_4_9225679	Ca_desi_chr04	9225679	[T/C]	NA
Ca_Desi_Chr_4_9225700	Ca_desi_chr04	9225700	[T/G]	NA
Ca_Desi_Chr_4_9225706	Ca_desi_chr04	9225706	[T/G]	NA
Ca_Desi_Chr_4_9225745	Ca_desi_chr04	9225745	[A/G]	NA
Ca_Desi_Chr_4_9270773	Ca_desi_chr04	9270773	[G/T]	NA
Ca_Desi_Chr_4_9338194	Ca_desi_chr04	9338194	[A/C]	NA
Ca_Desi_Chr_4_9338211	Ca_desi_chr04	9338211	[A/C]	NA
Ca_Desi_Chr_4_9345303	Ca_desi_chr04	9345303	[A/G]	NA
Ca_Desi_Chr_4_9345307	Ca_desi_chr04	9345307	[A/G]	NA
Ca_Desi_Chr_4_9345338	Ca_desi_chr04	9345338	[T/G]	NA
Ca_Desi_Chr_4_9345340	Ca_desi_chr04	9345340	[G/T]	NA
Ca_Desi_Chr_4_9345834	Ca_desi_chr04	9345834	[A/T]	NA
Ca_Desi_Chr_4_9358122	Ca_desi_chr04	9358122	[T/G]	NA
Ca_Desi_Chr_4_9358135	Ca_desi_chr04	9358135	[T/G]	NA
Ca_Desi_Chr_4_9385944	Ca_desi_chr04	9385944	[T/C]	NA
Ca_Desi_Chr_4_9387412	Ca_desi_chr04	9387412	[C/A]	NA
Ca_Desi_Chr_4_9387413	Ca_desi_chr04	9387413	[C/G]	NA
Ca_Desi_Chr_4_9387414	Ca_desi_chr04	9387414	[C/A]	NA
Ca_Desi_Chr_4_9484411	Ca_desi_chr04	9484411	[T/G]	NA
Ca_Desi_Chr_4_9484426	Ca_desi_chr04	9484426	[G/T]	NA
Ca_Desi_Chr_4_9484435	Ca_desi_chr04	9484435	[G/T]	NA
Ca_Desi_Chr_4_9494437	Ca_desi_chr04	9494437	[T/G]	NA

SNP IDs	Chromosomes	Physical positions (bp)	SNPs	NCBI SNPdb accession IDs (NCBI_ss#)
Ca_Desi_Chr_4_9513616	Ca_desi_chr04	9513616	[T/G]	NA
Ca_Desi_Chr_4_9561753	Ca_desi_chr04	9561753	[G/T]	NA
Ca_Desi_Chr_4_9561761	Ca_desi_chr04	9561761	[A/T]	NA
Ca_Desi_Chr_4_9594025	Ca_desi_chr04	9594025	[A/C]	NA
Ca_Desi_Chr_4_9594080	Ca_desi_chr04	9594080	[T/C]	NA
Ca_Desi_Chr_4_9594151	Ca_desi_chr04	9594151	[A/T]	NA
Ca_Desi_Chr_4_9637313	Ca_desi_chr04	9637313	[A/C]	NA
Ca_Desi_Chr_4_9637318	Ca_desi_chr04	9637318	[T/G]	NA
Ca_Desi_Chr_4_9726645	Ca_desi_chr04	9726645	[A/G]	NA
Ca_Desi_Chr_4_9726657	Ca_desi_chr04	9726657	[T/G]	NA
Ca_Desi_Chr_4_9726673	Ca_desi_chr04	9726673	[T/C]	NA
Ca_Desi_Chr_4_9726697	Ca_desi_chr04	9726697	[A/T]	NA
Ca_Desi_Chr_4_9735919	Ca_desi_chr04	9735919	[T/C]	NA
Ca_Desi_Chr_4_9747994	Ca_desi_chr04	9747994	[G/A]	NA
Ca_Desi_Chr_4_9756394	Ca_desi_chr04	9756394	[A/G]	NA
Ca_Desi_Chr_4_9756398	Ca_desi_chr04	9756398	[A/G]	NA
Ca_Desi_Chr_4_9756400	Ca_desi_chr04	9756400	[T/A]	NA
Ca_Desi_Chr_4_9756414	Ca_desi_chr04	9756414	[G/T]	NA
Ca_Desi_Chr_4_9756420	Ca_desi_chr04	9756420	[T/C]	NA
Ca_Desi_Chr_4_9756428	Ca_desi_chr04	9756428	[A/G]	NA
Ca_Desi_Chr_4_9756437	Ca_desi_chr04	9756437	[A/G]	NA
Ca_Desi_Chr_4_9756443	Ca_desi_chr04	9756443	[A/G]	NA
Ca_Desi_Chr_4_9809512	Ca_desi_chr04	9809512	[A/C]	NA
Ca_Desi_Chr_4_9820705	Ca_desi_chr04	9820705	[T/C]	NA
Ca_Desi_Chr_4_9820711	Ca_desi_chr04	9820711	[T/C]	NA
Ca_Desi_Chr_4_9834884	Ca_desi_chr04	9834884	[C/T]	NA
Ca_Desi_Chr_4_9851330	Ca_desi_chr04	9851330	[T/A]	NA
Ca_Desi_Chr_4_9851366	Ca_desi_chr04	9851366	[G/T]	NA
Ca_Desi_Chr_4_9851385	Ca_desi_chr04	9851385	[G/T]	NA
Ca_Desi_Chr_4_9851424	Ca_desi_chr04	9851424	[A/T]	NA
Ca_Desi_Chr_4_9855664	Ca_desi_chr04	9855664	[A/C]	NA
Ca_Desi_Chr_4_9855725	Ca_desi_chr04	9855725	[T/G]	NA
Ca_Desi_Chr_4_9856520	Ca_desi_chr04	9856520	[G/A]	NA
Ca_Desi_Chr_4_9856716	Ca_desi_chr04	9856716	[T/C]	NA

SNP IDs	Chromosomes	Physical positions (bp)	SNPs	NCBI SNPdb accession IDs (NCBI_ss#)
Ca_Desi_Chr_4_9856753	Ca_desi_chr04	9856753	[C/T]	NA
Ca_Desi_Chr_4_9856760	Ca_desi_chr04	9856760	[T/C]	NA
Ca_Desi_Chr_4_9856771	Ca_desi_chr04	9856771	[T/C]	NA
Ca_Desi_Chr_4_9856774	Ca_desi_chr04	9856774	[A/C]	NA
Ca_Desi_Chr_4_9856777	Ca_desi_chr04	9856777	[T/G]	NA
Ca_Desi_Chr_4_9856813	Ca_desi_chr04	9856813	[T/A]	NA
Ca_Desi_Chr_4_9857066	Ca_desi_chr04	9857066	[A/T]	NA
Ca_Desi_Chr_4_9857069	Ca_desi_chr04	9857069	[A/C]	NA
Ca_Desi_Chr_4_9857164	Ca_desi_chr04	9857164	[T/G]	NA
Ca_Desi_Chr_4_9857179	Ca_desi_chr04	9857179	[T/G]	NA
Ca_Desi_Chr_4_9857200	Ca_desi_chr04	9857200	[A/C]	NA
Ca_Desi_Chr_4_9857710	Ca_desi_chr04	9857710	[C/T]	NA
Ca_Desi_Chr_4_9857716	Ca_desi_chr04	9857716	[T/C]	NA
Ca_Desi_Chr_4_9858208	Ca_desi_chr04	9858208	[C/T]	NA
Ca_Desi_Chr_4_9858220	Ca_desi_chr04	9858220	[A/C]	NA
Ca_Desi_Chr_4_9858348	Ca_desi_chr04	9858348	[T/G]	NA
Ca_Desi_Chr_4_9858379	Ca_desi_chr04	9858379	[A/G]	NA
Ca_Desi_Chr_4_9858726	Ca_desi_chr04	9858726	[A/C]	NA
Ca_Desi_Chr_4_9858764	Ca_desi_chr04	9858764	[A/C]	NA
Ca_Desi_Chr_4_9858782	Ca_desi_chr04	9858782	[T/C]	NA
Ca_Desi_Chr_4_9858822	Ca_desi_chr04	9858822	[A/G]	NA
Ca_Desi_Chr_4_9858832	Ca_desi_chr04	9858832	[T/G]	NA
Ca_Desi_Chr_4_9858878	Ca_desi_chr04	9858878	[A/C]	NA
Ca_Desi_Chr_4_9858889	Ca_desi_chr04	9858889	[A/G]	NA
Ca_Desi_Chr_4_9909450	Ca_desi_chr04	9909450	[G/A]	NA
Ca_Desi_Chr_4_9923241	Ca_desi_chr04	9923241	[A/G]	NA
Ca_Desi_Chr_4_9923245	Ca_desi_chr04	9923245	[T/C]	NA
Ca_Desi_Chr_4_9938517	Ca_desi_chr04	9938517	[A/T]	NA
Ca_Desi_Chr_4_9989761	Ca_desi_chr04	9989761	[A/C]	NA
Ca_Desi_Chr_4_9989766	Ca_desi_chr04	9989766	[A/C]	NA
Ca_Desi_Chr_4_9998058	Ca_desi_chr04	9998058	[T/C]	NA
Ca_Desi_Chr_4_9998061	Ca_desi_chr04	9998061	[T/A]	NA
Ca_Desi_Chr_4_10003273	Ca_desi_chr04	10003273	[T/C]	NA
Ca_Desi_Chr_4_10003279	Ca_desi_chr04	10003279	[T/C]	NA

SNP IDs	Chromosomes	Physical positions (bp)	SNPs	NCBI SNPdb accession IDs (NCBI_ss#)
Ca_Desi_Chr_4_10003290	Ca_desi_chr04	10003290	[A/G]	NA
Ca_Desi_Chr_4_10003304	Ca_desi_chr04	10003304	[A/G]	NA
Ca_Desi_Chr_4_10003316	Ca_desi_chr04	10003316	[A/G]	NA
Ca_Desi_Chr_4_10036008	Ca_desi_chr04	10036008	[G/A]	NA
Ca_Desi_Chr_4_10036010	Ca_desi_chr04	10036010	[A/G]	NA
Ca_Desi_Chr_4_10081995	Ca_desi_chr04	10081995	[A/C]	NA
Ca_Desi_Chr_4_10162745	Ca_desi_chr04	10162745	[A/C]	NA
Ca_Desi_Chr_4_10162751	Ca_desi_chr04	10162751	[C/T]	NA
Ca_Desi_Chr_4_10162917	Ca_desi_chr04	10162917	[A/C]	NA
Ca_Desi_Chr_4_10162918	Ca_desi_chr04	10162918	[G/C]	NA
Ca_Desi_Chr_4_10162919	Ca_desi_chr04	10162919	[A/C]	NA
Ca_Desi_Chr_4_10229405	Ca_desi_chr04	10229405	[G/C]	NA
Ca_Desi_Chr_4_10229406	Ca_desi_chr04	10229406	[G/T]	NA
Ca_Desi_Chr_4_10248282	Ca_desi_chr04	10248282	[G/T]	NA
Ca_Desi_Chr_4_10249171	Ca_desi_chr04	10249171	[T/G]	NA
Ca_Desi_Chr_4_10249225	Ca_desi_chr04	10249225	[C/T]	NA
Ca_Desi_Chr_4_10249247	Ca_desi_chr04	10249247	[A/T]	NA
Ca_Desi_Chr_4_10249248	Ca_desi_chr04	10249248	[T/C]	NA
Ca_Desi_Chr_4_10249249	Ca_desi_chr04	10249249	[G/T]	NA
Ca_Desi_Chr_4_10249253	Ca_desi_chr04	10249253	[T/C]	NA
Ca_Desi_Chr_4_10249319	Ca_desi_chr04	10249319	[A/G]	NA
Ca_Desi_Chr_4_10249331	Ca_desi_chr04	10249331	[T/C]	NA
Ca_Desi_Chr_4_10249367	Ca_desi_chr04	10249367	[T/C]	NA
Ca_Desi_Chr_4_10291941	Ca_desi_chr04	10291941	[A/C]	NA
Ca_Desi_Chr_4_10294037	Ca_desi_chr04	10294037	[A/G]	NA
Ca_Desi_Chr_4_10297381	Ca_desi_chr04	10297381	[A/C]	NA
Ca_Desi_Chr_4_10297440	Ca_desi_chr04	10297440	[T/G]	NA
Ca_Desi_Chr_4_10297454	Ca_desi_chr04	10297454	[A/G]	NA
Ca_Desi_Chr_4_10297457	Ca_desi_chr04	10297457	[A/C]	NA
Ca_Desi_Chr_4_10297463	Ca_desi_chr04	10297463	[A/G]	NA
Ca_Desi_Chr_4_10297464	Ca_desi_chr04	10297464	[T/C]	NA
Ca_Desi_Chr_4_10297474	Ca_desi_chr04	10297474	[A/G]	NA
Ca_Desi_Chr_4_10311544	Ca_desi_chr04	10311544	[A/G]	NA
Ca_Desi_Chr_4_10339397	Ca_desi_chr04	10339397	[A/G]	NA

SNP IDs	Chromosomes	Physical positions (bp)	SNPs	NCBI SNPdb accession IDs (NCBI_ss#)
Ca_Desi_Chr_4_10339669	Ca_desi_chr04	10339669	[T/C]	NA
Ca_Desi_Chr_4_10349823	Ca_desi_chr04	10349823	[A/T]	NA
Ca_Desi_Chr_4_10349826	Ca_desi_chr04	10349826	[G/A]	NA
Ca_Desi_Chr_4_10349867	Ca_desi_chr04	10349867	[T/A]	NA
Ca_Desi_Chr_4_10351636	Ca_desi_chr04	10351636	[A/G]	NA
Ca_Desi_Chr_4_10354408	Ca_desi_chr04	10354408	[G/A]	NA
Ca_Desi_Chr_4_10354585	Ca_desi_chr04	10354585	[A/G]	NA
Ca_Desi_Chr_4_10356296	Ca_desi_chr04	10356296	[A/G]	NA
Ca_Desi_Chr_4_10365321	Ca_desi_chr04	10365321	[G/T]	NA
Ca_Desi_Chr_4_10369201	Ca_desi_chr04	10369201	[G/A]	NA
Ca_Desi_Chr_4_10376604	Ca_desi_chr04	10376604	[C/G]	NA
Ca_Desi_Chr_4_10378656	Ca_desi_chr04	10378656	[T/G]	NA
Ca_Desi_Chr_4_10577049	Ca_desi_chr04	10577049	[T/G]	NA
Ca_Desi_Chr_4_10613217	Ca_desi_chr04	10613217	[T/G]	NA
Ca_Desi_Chr_4_10618306	Ca_desi_chr04	10618306	[T/C]	NA
Ca_Desi_Chr_4_10618316	Ca_desi_chr04	10618316	[T/G]	NA
Ca_Desi_Chr_4_10618335	Ca_desi_chr04	10618335	[T/G]	NA
Ca_Desi_Chr_4_10618342	Ca_desi_chr04	10618342	[A/G]	NA
Ca_Desi_Chr_4_10685259	Ca_desi_chr04	10685259	[A/C]	NA
Ca_Desi_Chr_4_10685279	Ca_desi_chr04	10685279	[T/A]	NA
Ca_Desi_Chr_4_10721599	Ca_desi_chr04	10721599	[T/G]	NA
Ca_Desi_Chr_4_10721635	Ca_desi_chr04	10721635	[T/C]	NA
Ca_Desi_Chr_4_10735683	Ca_desi_chr04	10735683	[A/T]	NA
Ca_Desi_Chr_4_10746478	Ca_desi_chr04	10746478	[T/G]	NA
Ca_Desi_Chr_4_10746501	Ca_desi_chr04	10746501	[T/G]	NA
Ca_Desi_Chr_4_10746537	Ca_desi_chr04	10746537	[A/C]	NA
Ca_Desi_Chr_4_10817293	Ca_desi_chr04	10817293	[A/G]	NA
Ca_Desi_Chr_4_10840283	Ca_desi_chr04	10840283	[A/T]	NA
Ca_Desi_Chr_4_10840291	Ca_desi_chr04	10840291	[A/G]	NA
Ca_Desi_Chr_4_10840302	Ca_desi_chr04	10840302	[A/G]	NA
Ca_Desi_Chr_4_10840305	Ca_desi_chr04	10840305	[T/G]	NA
Ca_Desi_Chr_4_10840308	Ca_desi_chr04	10840308	[A/G]	NA
Ca_Desi_Chr_4_10840320	Ca_desi_chr04	10840320	[A/G]	NA
Ca_Desi_Chr_4_10840333	Ca_desi_chr04	10840333	[T/G]	NA

SNP IDs	Chromosomes	Physical positions (bp)	SNPs	NCBI SNPdb accession IDs (NCBI_ss#)
Ca_Desi_Chr_4_10840336	Ca_desi_chr04	10840336	[T/C]	NA
Ca_Desi_Chr_4_10840340	Ca_desi_chr04	10840340	[T/C]	NA
Ca_Desi_Chr_4_10911544	Ca_desi_chr04	10911544	[T/G]	NA
Ca_Desi_Chr_4_11013040	Ca_desi_chr04	11013040	[G/C]	NA
Ca_Desi_Chr_4_11038188	Ca_desi_chr04	11038188	[T/G]	NA
Ca_Desi_Chr_4_11038463	Ca_desi_chr04	11038463	[T/C]	NA
Ca_Desi_Chr_4_11038479	Ca_desi_chr04	11038479	[A/C]	NA
Ca_Desi_Chr_4_11072007	Ca_desi_chr04	11072007	[A/C]	NA
Ca_Desi_Chr_4_11072222	Ca_desi_chr04	11072222	[T/C]	NA
Ca_Desi_Chr_4_11072259	Ca_desi_chr04	11072259	[T/C]	NA
Ca_Desi_Chr_4_11072266	Ca_desi_chr04	11072266	[A/G]	NA
Ca_Desi_Chr_4_11089936	Ca_desi_chr04	11089936	[A/C]	NA
Ca_Desi_Chr_4_11089940	Ca_desi_chr04	11089940	[C/T]	NA
Ca_Desi_Chr_4_11182579	Ca_desi_chr04	11182579	[A/T]	NA
Ca_Desi_Chr_4_11229015	Ca_desi_chr04	11229015	[T/C]	NA
Ca_Desi_Chr_4_11229016	Ca_desi_chr04	11229016	[C/T]	NA
Ca_Desi_Chr_4_11229018	Ca_desi_chr04	11229018	[C/T]	NA
Ca_Desi_Chr_4_11387821	Ca_desi_chr04	11387821	[T/C]	NA
Ca_Desi_Chr_4_11387845	Ca_desi_chr04	11387845	[T/G]	NA
Ca_Desi_Chr_4_11389484	Ca_desi_chr04	11389484	[A/G]	NA
Ca_Desi_Chr_4_11389492	Ca_desi_chr04	11389492	[T/C]	NA
Ca_Desi_Chr_4_11389523	Ca_desi_chr04	11389523	[T/C]	NA
Ca_Desi_Chr_4_11399082	Ca_desi_chr04	11399082	[A/C]	NA
Ca_Desi_Chr_4_11399086	Ca_desi_chr04	11399086	[T/C]	NA
Ca_Desi_Chr_4_11399098	Ca_desi_chr04	11399098	[A/G]	NA
Ca_Desi_Chr_4_11399105	Ca_desi_chr04	11399105	[A/C]	NA
Ca_Desi_Chr_4_11399117	Ca_desi_chr04	11399117	[A/C]	NA
Ca_Desi_Chr_4_11407191	Ca_desi_chr04	11407191	[T/G]	NA
Ca_Desi_Chr_4_11457370	Ca_desi_chr04	11457370	[T/G]	NA
Ca_Desi_Chr_4_11457402	Ca_desi_chr04	11457402	[T/G]	NA
Ca_Desi_Chr_4_11458944	Ca_desi_chr04	11458944	[T/G]	NA
Ca_Desi_Chr_4_11458965	Ca_desi_chr04	11458965	[G/T]	NA
Ca_Desi_Chr_4_11459978	Ca_desi_chr04	11459978	[T/C]	NA
Ca_Desi_Chr_4_11491065	Ca_desi_chr04	11491065	[A/C]	NA

SNP IDs	Chromosomes	Physical positions (bp)	SNPs	NCBI SNPdb accession IDs (NCBI_ss#)
Ca_Desi_Chr_4_11538965	Ca_desi_chr04	11538965	[G/C]	NA
Ca_Desi_Chr_4_11539026	Ca_desi_chr04	11539026	[A/G]	NA
Ca_Desi_Chr_4_11539401	Ca_desi_chr04	11539401	[C/T]	NA
Ca_Desi_Chr_4_11540055	Ca_desi_chr04	11540055	[G/A]	NA
Ca_Desi_Chr_4_11540126	Ca_desi_chr04	11540126	[G/C]	NA
Ca_Desi_Chr_4_11540314	Ca_desi_chr04	11540314	[A/G]	NA
Ca_Desi_Chr_4_11540353	Ca_desi_chr04	11540353	[A/G]	NA
Ca_Desi_Chr_4_11540373	Ca_desi_chr04	11540373	[T/C]	NA
Ca_Desi_Chr_4_11540399	Ca_desi_chr04	11540399	[T/C]	NA
Ca_Desi_Chr_4_11542258	Ca_desi_chr04	11542258	[G/A]	NA
Ca_Desi_Chr_4_11543134	Ca_desi_chr04	11543134	[A/T]	NA
Ca_Desi_Chr_4_11543211	Ca_desi_chr04	11543211	[A/G]	NA
Ca_Desi_Chr_4_11543635	Ca_desi_chr04	11543635	[G/T]	NA
Ca_Desi_Chr_4_11543651	Ca_desi_chr04	11543651	[G/A]	NA
Ca_Desi_Chr_4_11558941	Ca_desi_chr04	11558941	[G/A]	NA
Ca_Desi_Chr_4_11567921	Ca_desi_chr04	11567921	[T/A]	NA
Ca_Desi_Chr_4_11570353	Ca_desi_chr04	11570353	[A/G]	NA
Ca_Desi_Chr_4_11570362	Ca_desi_chr04	11570362	[T/C]	NA
Ca_Desi_Chr_4_11570433	Ca_desi_chr04	11570433	[T/C]	NA
Ca_Desi_Chr_4_11572131	Ca_desi_chr04	11572131	[A/C]	NA
Ca_Desi_Chr_4_11572192	Ca_desi_chr04	11572192	[C/A]	NA
Ca_Desi_Chr_4_11572638	Ca_desi_chr04	11572638	[C/T]	NA
Ca_Desi_Chr_4_11572654	Ca_desi_chr04	11572654	[A/G]	NA
Ca_Desi_Chr_4_11585262	Ca_desi_chr04	11585262	[A/G]	NA
Ca_Desi_Chr_4_11585285	Ca_desi_chr04	11585285	[T/C]	NA
Ca_Desi_Chr_4_11585305	Ca_desi_chr04	11585305	[C/T]	NA
Ca_Desi_Chr_4_11585310	Ca_desi_chr04	11585310	[C/T]	NA
Ca_Desi_Chr_4_11585312	Ca_desi_chr04	11585312	[A/G]	NA
Ca_Desi_Chr_4_11585316	Ca_desi_chr04	11585316	[T/C]	NA
Ca_Desi_Chr_4_11585318	Ca_desi_chr04	11585318	[A/G]	NA
Ca_Desi_Chr_4_11585360	Ca_desi_chr04	11585360	[A/G]	NA
Ca_Desi_Chr_4_11585368	Ca_desi_chr04	11585368	[T/C]	NA
Ca_Desi_Chr_4_11585391	Ca_desi_chr04	11585391	[A/G]	NA
Ca_Desi_Chr_4_11586003	Ca_desi_chr04	11586003	[T/C]	NA

SNP IDs	Chromosomes	Physical positions (bp)	SNPs	NCBI SNPdb accession IDs (NCBI_ss#)
Ca_Desi_Chr_4_11586015	Ca_desi_chr04	11586015	[T/G]	NA
Ca_Desi_Chr_4_11586059	Ca_desi_chr04	11586059	[T/G]	NA
Ca_Desi_Chr_4_11586074	Ca_desi_chr04	11586074	[T/G]	NA
Ca_Desi_Chr_4_11603349	Ca_desi_chr04	11603349	[G/A]	NA
Ca_Desi_Chr_4_11603546	Ca_desi_chr04	11603546	[A/G]	NA
Ca_Desi_Chr_4_11618946	Ca_desi_chr04	11618946	[T/G]	NA
Ca_Desi_Chr_4_11663437	Ca_desi_chr04	11663437	[T/C]	NA
Ca_Desi_Chr_4_11664283	Ca_desi_chr04	11664283	[A/G]	NA
Ca_Desi_Chr_4_11664297	Ca_desi_chr04	11664297	[T/C]	NA
Ca_Desi_Chr_4_11664301	Ca_desi_chr04	11664301	[A/G]	NA
Ca_Desi_Chr_4_11666412	Ca_desi_chr04	11666412	[T/C]	NA
Ca_Desi_Chr_4_11666417	Ca_desi_chr04	11666417	[T/C]	NA
Ca_Desi_Chr_4_11674004	Ca_desi_chr04	11674004	[G/C]	NA
Ca_Desi_Chr_4_11674038	Ca_desi_chr04	11674038	[A/G]	NA
Ca_Desi_Chr_4_11674058	Ca_desi_chr04	11674058	[A/G]	NA
Ca_Desi_Chr_4_11674107	Ca_desi_chr04	11674107	[A/G]	NA
Ca_Desi_Chr_4_11674195	Ca_desi_chr04	11674195	[A/G]	NA
Ca_Desi_Chr_4_11674605	Ca_desi_chr04	11674605	[G/A]	NA
Ca_Desi_Chr_4_11674615	Ca_desi_chr04	11674615	[C/A]	NA
Ca_Desi_Chr_4_11674619	Ca_desi_chr04	11674619	[A/C]	NA
Ca_Desi_Chr_4_11674624	Ca_desi_chr04	11674624	[A/C]	NA
Ca_Desi_Chr_4_11679016	Ca_desi_chr04	11679016	[T/A]	NA
Ca_Desi_Chr_4_11714449	Ca_desi_chr04	11714449	[A/T]	NA
Ca_Desi_Chr_4_11714450	Ca_desi_chr04	11714450	[A/C]	NA
Ca_Desi_Chr_4_11714451	Ca_desi_chr04	11714451	[G/T]	NA
Ca_Desi_Chr_4_11784964	Ca_desi_chr04	11784964	[A/T]	NA
Ca_Desi_Chr_4_11784979	Ca_desi_chr04	11784979	[T/C]	NA
Ca_Desi_Chr_4_11784986	Ca_desi_chr04	11784986	[A/T]	NA
Ca_Desi_Chr_4_11784996	Ca_desi_chr04	11784996	[T/G]	NA
Ca_Desi_Chr_4_11832110	Ca_desi_chr04	11832110	[A/C]	NA
Ca_Desi_Chr_4_11832118	Ca_desi_chr04	11832118	[A/C]	NA
Ca_Desi_Chr_4_11837366	Ca_desi_chr04	11837366	[A/C]	NA
Ca_Desi_Chr_4_11933382	Ca_desi_chr04	11933382	[T/G]	NA
Ca_Desi_Chr_4_11933384	Ca_desi_chr04	11933384	[T/A]	NA

SNP IDs	Chromosomes	Physical positions (bp)	SNPs	NCBI SNPdb accession IDs (NCBI_ss#)
Ca_Desi_Chr_4_11933404	Ca_desi_chr04	11933404	[A/T]	NA
Ca_Desi_Chr_4_11933417	Ca_desi_chr04	11933417	[T/A]	NA
Ca_Desi_Chr_4_11933440	Ca_desi_chr04	11933440	[A/G]	NA
Ca_Desi_Chr_4_11984997	Ca_desi_chr04	11984997	[T/G]	NA
Ca_Desi_Chr_4_11985017	Ca_desi_chr04	11985017	[A/T]	NA
Ca_Desi_Chr_4_12098589	Ca_desi_chr04	12098589	[C/T]	NA
Ca_Desi_Chr_4_12098592	Ca_desi_chr04	12098592	[C/T]	NA
Ca_Desi_Chr_4_12118440	Ca_desi_chr04	12118440	[T/C]	NA
Ca_Desi_Chr_4_12119463	Ca_desi_chr04	12119463	[A/G]	NA
Ca_Desi_Chr_4_12119500	Ca_desi_chr04	12119500	[T/C]	NA
Ca_Desi_Chr_4_12119513	Ca_desi_chr04	12119513	[T/A]	NA
Ca_Desi_Chr_4_12135273	Ca_desi_chr04	12135273	[T/C]	NA
Ca_Desi_Chr_4_12204368	Ca_desi_chr04	12204368	[A/T]	NA
Ca_Desi_Chr_4_12204413	Ca_desi_chr04	12204413	[A/T]	NA
Ca_Desi_Chr_4_12204460	Ca_desi_chr04	12204460	[T/G]	NA
Ca_Desi_Chr_4_12204644	Ca_desi_chr04	12204644	[T/C]	NA
Ca_Desi_Chr_4_12204653	Ca_desi_chr04	12204653	[T/C]	NA
Ca_Desi_Chr_4_12204669	Ca_desi_chr04	12204669	[A/G]	NA
Ca_Desi_Chr_4_12204700	Ca_desi_chr04	12204700	[A/G]	NA
Ca_Desi_Chr_4_12204710	Ca_desi_chr04	12204710	[A/G]	NA
Ca_Desi_Chr_4_12271895	Ca_desi_chr04	12271895	[A/G]	NA
Ca_Desi_Chr_4_12276816	Ca_desi_chr04	12276816	[A/G]	NA
Ca_Desi_Chr_4_12276826	Ca_desi_chr04	12276826	[A/G]	NA
Ca_Desi_Chr_4_12276828	Ca_desi_chr04	12276828	[A/C]	NA
Ca_Desi_Chr_4_12276839	Ca_desi_chr04	12276839	[T/A]	NA
Ca_Desi_Chr_4_12276848	Ca_desi_chr04	12276848	[A/G]	NA
Ca_Desi_Chr_4_12298981	Ca_desi_chr04	12298981	[T/C]	NA
Ca_Desi_Chr_4_12298984	Ca_desi_chr04	12298984	[A/G]	NA
Ca_Desi_Chr_4_12299002	Ca_desi_chr04	12299002	[A/C]	NA
Ca_Desi_Chr_4_12299004	Ca_desi_chr04	12299004	[A/C]	NA
Ca_Desi_Chr_4_12299032	Ca_desi_chr04	12299032	[T/A]	NA
Ca_Desi_Chr_4_12315915	Ca_desi_chr04	12315915	[T/A]	NA
Ca_Desi_Chr_4_12315954	Ca_desi_chr04	12315954	[A/G]	NA
Ca_Desi_Chr_4_12318263	Ca_desi_chr04	12318263	[A/C]	NA

SNP IDs	Chromosomes	Physical positions (bp)	SNPs	NCBI SNPdb accession IDs (NCBI_ss#)
Ca_Desi_Chr_4_12318282	Ca_desi_chr04	12318282	[A/G]	NA
Ca_Desi_Chr_4_12335457	Ca_desi_chr04	12335457	[T/C]	NA
Ca_Desi_Chr_4_12335461	Ca_desi_chr04	12335461	[A/C]	NA
Ca_Desi_Chr_4_12335473	Ca_desi_chr04	12335473	[A/C]	NA
Ca_Desi_Chr_4_12335490	Ca_desi_chr04	12335490	[A/G]	NA
Ca_Desi_Chr_4_12339437	Ca_desi_chr04	12339437	[A/T]	NA
Ca_Desi_Chr_4_12339438	Ca_desi_chr04	12339438	[G/C]	NA
Ca_Desi_Chr_4_12339439	Ca_desi_chr04	12339439	[G/T]	NA
Ca_Desi_Chr_4_12366200	Ca_desi_chr04	12366200	[T/C]	NA
Ca_Desi_Chr_4_12415487	Ca_desi_chr04	12415487	[A/G]	NA
Ca_Desi_Chr_4_12415495	Ca_desi_chr04	12415495	[A/G]	NA
Ca_Desi_Chr_4_12415500	Ca_desi_chr04	12415500	[T/C]	NA
Ca_Desi_Chr_4_12415509	Ca_desi_chr04	12415509	[A/G]	NA
Ca_Desi_Chr_4_12415511	Ca_desi_chr04	12415511	[T/C]	NA
Ca_Desi_Chr_4_12415516	Ca_desi_chr04	12415516	[G/A]	NA
Ca_Desi_Chr_4_12415530	Ca_desi_chr04	12415530	[T/C]	NA
Ca_Desi_Chr_4_12415532	Ca_desi_chr04	12415532	[A/G]	NA
Ca_Desi_Chr_4_12415539	Ca_desi_chr04	12415539	[C/A]	NA
Ca_Desi_Chr_4_12415656	Ca_desi_chr04	12415656	[A/G]	NA
Ca_Desi_Chr_4_12483075	Ca_desi_chr04	12483075	[T/C]	NA
Ca_Desi_Chr_4_12483136	Ca_desi_chr04	12483136	[A/G]	NA
Ca_Desi_Chr_4_12483371	Ca_desi_chr04	12483371	[A/G]	NA
Ca_Desi_Chr_4_12499443	Ca_desi_chr04	12499443	[A/C]	NA
Ca_Desi_Chr_4_12501779	Ca_desi_chr04	12501779	[T/G]	NA
Ca_Desi_Chr_4_12574293	Ca_desi_chr04	12574293	[A/T]	NA
Ca_Desi_Chr_4_12662712	Ca_desi_chr04	12662712	[T/C]	NA
Ca_Desi_Chr_4_12662782	Ca_desi_chr04	12662782	[T/A]	NA
Ca_Desi_Chr_4_12662784	Ca_desi_chr04	12662784	[A/G]	NA
Ca_Desi_Chr_4_12662818	Ca_desi_chr04	12662818	[A/G]	NA
Ca_Desi_Chr_4_12705809	Ca_desi_chr04	12705809	[A/C]	NA
Ca_Desi_Chr_4_12705814	Ca_desi_chr04	12705814	[A/C]	NA
Ca_Desi_Chr_4_12705829	Ca_desi_chr04	12705829	[T/G]	NA
Ca_Desi_Chr_4_12748482	Ca_desi_chr04	12748482	[G/A]	NA
Ca_Desi_Chr_4_12748483	Ca_desi_chr04	12748483	[T/G]	NA

SNP IDs	Chromosomes	Physical positions (bp)	SNPs	NCBI SNPdb accession IDs (NCBI_ss#)
Ca_Desi_Chr_4_12750219	Ca_desi_chr04	12750219	[A/C]	NA
Ca_Desi_Chr_4_12750227	Ca_desi_chr04	12750227	[A/G]	NA
Ca_Desi_Chr_4_12750232	Ca_desi_chr04	12750232	[A/C]	NA
Ca_Desi_Chr_4_12791207	Ca_desi_chr04	12791207	[A/C]	NA
Ca_Desi_Chr_4_12791217	Ca_desi_chr04	12791217	[A/T]	NA
Ca_Desi_Chr_4_12837386	Ca_desi_chr04	12837386	[T/G]	NA
Ca_Desi_Chr_4_12915143	Ca_desi_chr04	12915143	[A/T]	NA
Ca_Desi_Chr_4_12915144	Ca_desi_chr04	12915144	[T/A]	NA
Ca_Desi_Chr_4_12921723	Ca_desi_chr04	12921723	[A/G]	NA
Ca_Desi_Chr_4_12921734	Ca_desi_chr04	12921734	[T/G]	NA
Ca_Desi_Chr_4_12921757	Ca_desi_chr04	12921757	[A/T]	NA
Ca_Desi_Chr_4_12998000	Ca_desi_chr04	12998000	[T/G]	NA
Ca_Desi_Chr_4_12998008	Ca_desi_chr04	12998008	[T/C]	NA
Ca_Desi_Chr_4_13005176	Ca_desi_chr04	13005176	[T/G]	NA
Ca_Desi_Chr_4_13005197	Ca_desi_chr04	13005197	[T/G]	NA
Ca_Desi_Chr_4_15813359	Ca_desi_chr04	15813359	[T/G]	NA
Ca_Desi_Chr_4_15874744	Ca_desi_chr04	15874744	[T/C]	NA
Ca_Desi_Chr_4_15874913	Ca_desi_chr04	15874913	[G/T]	NA
Ca_Desi_Chr_4_15874914	Ca_desi_chr04	15874914	[T/G]	NA
Ca_Desi_Chr_4_15874915	Ca_desi_chr04	15874915	[T/A]	NA
Ca_Desi_Chr_4_15907269	Ca_desi_chr04	15907269	[T/G]	NA
Ca_Desi_Chr_4_15949386	Ca_desi_chr04	15949386	[A/C]	NA
Ca_Desi_Chr_4_15949396	Ca_desi_chr04	15949396	[A/T]	NA
Ca_Desi_Chr_4_15957911	Ca_desi_chr04	15957911	[T/A]	NA
Ca_Desi_Chr_4_15957917	Ca_desi_chr04	15957917	[A/G]	NA
Ca_Desi_Chr_4_15957923	Ca_desi_chr04	15957923	[C/A]	NA
Ca_Desi_Chr_4_15957925	Ca_desi_chr04	15957925	[G/A]	NA
Ca_Desi_Chr_4_15957928	Ca_desi_chr04	15957928	[G/A]	NA
Ca_Desi_Chr_4_15957942	Ca_desi_chr04	15957942	[G/A]	NA
Ca_Desi_Chr_4_16009843	Ca_desi_chr04	16009843	[T/C]	NA
Ca_Desi_Chr_4_16009846	Ca_desi_chr04	16009846	[A/C]	NA
Ca_Desi_Chr_4_16009850	Ca_desi_chr04	16009850	[A/G]	NA
Ca_Desi_Chr_4_16009855	Ca_desi_chr04	16009855	[T/C]	NA
Ca_Desi_Chr_4_16009858	Ca_desi_chr04	16009858	[A/C]	NA

SNP IDs	Chromosomes	Physical positions (bp)	SNPs	NCBI SNPdb accession IDs (NCBI_ss#)
Ca_Desi_Chr_4_16009861	Ca_desi_chr04	16009861	[A/C]	NA
Ca_Desi_Chr_4_16009868	Ca_desi_chr04	16009868	[T/C]	NA
Ca_Desi_Chr_4_16043495	Ca_desi_chr04	16043495	[T/C]	NA
Ca_Desi_Chr_4_16061775	Ca_desi_chr04	16061775	[T/C]	NA
Ca_Desi_Chr_4_16061787	Ca_desi_chr04	16061787	[T/G]	NA
Ca_Desi_Chr_4_16079978	Ca_desi_chr04	16079978	[T/A]	NA
Ca_Desi_Chr_4_16080709	Ca_desi_chr04	16080709	[T/A]	NA
Ca_Desi_Chr_4_16140061	Ca_desi_chr04	16140061	[T/C]	NA
Ca_Desi_Chr_4_16150551	Ca_desi_chr04	16150551	[A/G]	NA
Ca_Desi_Chr_4_16150553	Ca_desi_chr04	16150553	[T/A]	NA
Ca_Desi_Chr_4_16150576	Ca_desi_chr04	16150576	[A/G]	NA
Ca_Desi_Chr_4_16150578	Ca_desi_chr04	16150578	[A/G]	NA
Ca_Desi_Chr_4_16150587	Ca_desi_chr04	16150587	[T/C]	NA
Ca_Desi_Chr_4_16150599	Ca_desi_chr04	16150599	[T/C]	NA
Ca_Desi_Chr_4_16174574	Ca_desi_chr04	16174574	[T/A]	NA
Ca_Desi_Chr_4_16176556	Ca_desi_chr04	16176556	[A/C]	NA
Ca_Desi_Chr_4_16176557	Ca_desi_chr04	16176557	[G/T]	NA
Ca_Desi_Chr_4_16206300	Ca_desi_chr04	16206300	[A/T]	NA
Ca_Desi_Chr_4_16206301	Ca_desi_chr04	16206301	[A/C]	NA
Ca_Desi_Chr_4_16206302	Ca_desi_chr04	16206302	[G/T]	NA
Ca_Desi_Chr_4_16206544	Ca_desi_chr04	16206544	[T/G]	NA
Ca_Desi_Chr_4_16206693	Ca_desi_chr04	16206693	[T/G]	NA
Ca_Desi_Chr_4_16219656	Ca_desi_chr04	16219656	[A/G]	NA
Ca_Desi_Chr_4_16219684	Ca_desi_chr04	16219684	[G/A]	NA
Ca_Desi_Chr_4_16219688	Ca_desi_chr04	16219688	[T/C]	NA
Ca_Desi_Chr_4_16219795	Ca_desi_chr04	16219795	[A/C]	NA
Ca_Desi_Chr_4_16230591	Ca_desi_chr04	16230591	[T/G]	NA
Ca_Desi_Chr_4_16230610	Ca_desi_chr04	16230610	[T/G]	NA
Ca_Desi_Chr_4_16235291	Ca_desi_chr04	16235291	[T/G]	NA
Ca_Desi_Chr_4_16235463	Ca_desi_chr04	16235463	[A/C]	NA
Ca_Desi_Chr_4_16237454	Ca_desi_chr04	16237454	[A/C]	NA
Ca_Desi_Chr_4_16289101	Ca_desi_chr04	16289101	[A/G]	NA
Ca_Desi_Chr_4_16318794	Ca_desi_chr04	16318794	[C/T]	NA
Ca_Desi_Chr_4_16318820	Ca_desi_chr04	16318820	[G/A]	NA

SNP IDs	Chromosomes	Physical positions (bp)	SNPs	NCBI SNPdb accession IDs (NCBI_ss#)
Ca_Desi_Chr_4_16362421	Ca_desi_chr04	16362421	[T/G]	NA
Ca_Desi_Chr_4_16362424	Ca_desi_chr04	16362424	[T/G]	NA
Ca_Desi_Chr_4_16362429	Ca_desi_chr04	16362429	[A/G]	NA
Ca_Desi_Chr_4_16551106	Ca_desi_chr04	16551106	[A/G]	NA
Ca_Desi_Chr_4_16551129	Ca_desi_chr04	16551129	[T/C]	NA
Ca_Desi_Chr_4_16557296	Ca_desi_chr04	16557296	[A/G]	NA
Ca_Desi_Chr_4_16557304	Ca_desi_chr04	16557304	[A/T]	NA
Ca_Desi_Chr_4_16557332	Ca_desi_chr04	16557332	[A/G]	NA
Ca_Desi_Chr_4_16591976	Ca_desi_chr04	16591976	[A/C]	NA
Ca_Desi_Chr_4_16674009	Ca_desi_chr04	16674009	[T/C]	NA
Ca_Desi_Chr_4_16674012	Ca_desi_chr04	16674012	[T/C]	NA
Ca_Desi_Chr_4_16674017	Ca_desi_chr04	16674017	[T/C]	NA
Ca_Desi_Chr_4_16674023	Ca_desi_chr04	16674023	[T/C]	NA
Ca_Desi_Chr_4_16674028	Ca_desi_chr04	16674028	[T/C]	NA
Ca_Desi_Chr_4_16674030	Ca_desi_chr04	16674030	[T/C]	NA
Ca_Desi_Chr_4_16674032	Ca_desi_chr04	16674032	[A/G]	NA
Ca_Desi_Chr_4_16674037	Ca_desi_chr04	16674037	[A/G]	NA
Ca_Desi_Chr_4_16674040	Ca_desi_chr04	16674040	[T/C]	NA
Ca_Desi_Chr_4_16674041	Ca_desi_chr04	16674041	[C/T]	NA
Ca_Desi_Chr_4_16674042	Ca_desi_chr04	16674042	[T/C]	NA
Ca_Desi_Chr_4_16674047	Ca_desi_chr04	16674047	[T/C]	NA
Ca_Desi_Chr_4_16674050	Ca_desi_chr04	16674050	[T/C]	NA
Ca_Desi_Chr_4_16674052	Ca_desi_chr04	16674052	[T/C]	NA
Ca_Desi_Chr_4_16676187	Ca_desi_chr04	16676187	[A/C]	NA
Ca_Desi_Chr_4_16676201	Ca_desi_chr04	16676201	[A/G]	NA
Ca_Desi_Chr_4_16706739	Ca_desi_chr04	16706739	[A/C]	NA
Ca_Desi_Chr_4_16724916	Ca_desi_chr04	16724916	[A/C]	NA
Ca_Desi_Chr_4_16730046	Ca_desi_chr04	16730046	[A/T]	NA
Ca_Desi_Chr_4_16741314	Ca_desi_chr04	16741314	[A/G]	NA
Ca_Desi_Chr_4_16741317	Ca_desi_chr04	16741317	[A/G]	NA
Ca_Desi_Chr_4_16741327	Ca_desi_chr04	16741327	[A/T]	NA
Ca_Desi_Chr_4_16741344	Ca_desi_chr04	16741344	[A/G]	NA
Ca_Desi_Chr_4_16741350	Ca_desi_chr04	16741350	[A/G]	NA
Ca_Desi_Chr_4_16741368	Ca_desi_chr04	16741368	[T/A]	NA

SNP IDs	Chromosomes	Physical positions (bp)	SNPs	NCBI SNPdb accession IDs (NCBI_ss#)
Ca_Desi_Chr_4_16741428	Ca_desi_chr04	16741428	[A/T]	NA
Ca_Desi_Chr_4_16741429	Ca_desi_chr04	16741429	[T/A]	NA
Ca_Desi_Chr_4_16741430	Ca_desi_chr04	16741430	[T/C]	NA
Ca_Desi_Chr_4_16744614	Ca_desi_chr04	16744614	[T/C]	NA
Ca_Desi_Chr_4_16744617	Ca_desi_chr04	16744617	[A/G]	NA
Ca_Desi_Chr_4_16744629	Ca_desi_chr04	16744629	[A/G]	NA
Ca_Desi_Chr_4_16744634	Ca_desi_chr04	16744634	[A/G]	NA
Ca_Desi_Chr_4_16744648	Ca_desi_chr04	16744648	[T/C]	NA
Ca_Desi_Chr_4_16744665	Ca_desi_chr04	16744665	[T/C]	NA
Ca_Desi_Chr_4_16744669	Ca_desi_chr04	16744669	[A/G]	NA
Ca_Desi_Chr_4_16744676	Ca_desi_chr04	16744676	[T/C]	NA
Ca_Desi_Chr_4_16744678	Ca_desi_chr04	16744678	[T/C]	NA
Ca_Desi_Chr_4_16744683	Ca_desi_chr04	16744683	[A/G]	NA
Ca_Desi_Chr_4_16770730	Ca_desi_chr04	16770730	[A/G]	NA
Ca_Desi_Chr_4_16791446	Ca_desi_chr04	16791446	[A/C]	NA
Ca_Desi_Chr_4_16791504	Ca_desi_chr04	16791504	[T/A]	NA
Ca_Desi_Chr_4_16791513	Ca_desi_chr04	16791513	[A/G]	NA
Ca_Desi_Chr_4_16791541	Ca_desi_chr04	16791541	[A/C]	NA
Ca_Desi_Chr_4_16791666	Ca_desi_chr04	16791666	[A/C]	NA
Ca_Desi_Chr_4_16795332	Ca_desi_chr04	16795332	[A/C]	NA
Ca_Desi_Chr_4_16795366	Ca_desi_chr04	16795366	[A/C]	NA
Ca_Desi_Chr_4_16795500	Ca_desi_chr04	16795500	[G/T]	NA
Ca_Desi_Chr_4_16795501	Ca_desi_chr04	16795501	[A/C]	NA
Ca_Desi_Chr_4_16795502	Ca_desi_chr04	16795502	[G/T]	NA
Ca_Desi_Chr_4_16802460	Ca_desi_chr04	16802460	[C/G]	NA
Ca_Desi_Chr_4_16818312	Ca_desi_chr04	16818312	[T/A]	NA
Ca_Desi_Chr_4_16827801	Ca_desi_chr04	16827801	[A/C]	NA
Ca_Desi_Chr_4_16827824	Ca_desi_chr04	16827824	[T/G]	NA
Ca_Desi_Chr_4_16827840	Ca_desi_chr04	16827840	[A/T]	NA
Ca_Desi_Chr_4_16894642	Ca_desi_chr04	16894642	[A/C]	NA
Ca_Desi_Chr_4_16900160	Ca_desi_chr04	16900160	[T/C]	NA
Ca_Desi_Chr_4_16900349	Ca_desi_chr04	16900349	[A/G]	NA
Ca_Desi_Chr_4_16907164	Ca_desi_chr04	16907164	[A/C]	NA
Ca_Desi_Chr_4_16949996	Ca_desi_chr04	16949996	[A/C]	NA

SNP IDs	Chromosomes	Physical positions (bp)	SNPs	NCBI SNPdb accession IDs (NCBI_ss#)
Ca_Desi_Chr_4_16949999	Ca_desi_chr04	16949999	[A/G]	NA
Ca_Desi_Chr_4_16950003	Ca_desi_chr04	16950003	[T/C]	NA
Ca_Desi_Chr_4_16950004	Ca_desi_chr04	16950004	[C/T]	NA
Ca_Desi_Chr_4_16950056	Ca_desi_chr04	16950056	[C/T]	NA
Ca_Desi_Chr_4_17016503	Ca_desi_chr04	17016503	[T/C]	NA
Ca_Desi_Chr_4_17016536	Ca_desi_chr04	17016536	[T/C]	NA
Ca_Desi_Chr_4_17026018	Ca_desi_chr04	17026018	[T/G]	NA
Ca_Desi_Chr_4_17026051	Ca_desi_chr04	17026051	[A/T]	NA
Ca_Desi_Chr_4_17026071	Ca_desi_chr04	17026071	[A/T]	NA
Ca_Desi_Chr_4_18043880	Ca_desi_chr04	18043880	[A/C]	NA
Ca_Desi_Chr_4_18043884	Ca_desi_chr04	18043884	[A/C]	NA
Ca_Desi_Chr_4_18043974	Ca_desi_chr04	18043974	[A/G]	NA
Ca_Desi_Chr_4_18043980	Ca_desi_chr04	18043980	[A/G]	NA
Ca_Desi_Chr_4_18043989	Ca_desi_chr04	18043989	[A/C]	NA
Ca_Desi_Chr_4_18044043	Ca_desi_chr04	18044043	[A/G]	NA
Ca_Desi_Chr_4_18044067	Ca_desi_chr04	18044067	[A/G]	NA
Ca_Desi_Chr_4_18063839	Ca_desi_chr04	18063839	[A/G]	NA
Ca_Desi_Chr_4_18064756	Ca_desi_chr04	18064756	[A/G]	NA
Ca_Desi_Chr_4_18064758	Ca_desi_chr04	18064758	[G/T]	NA
Ca_Desi_Chr_4_18064759	Ca_desi_chr04	18064759	[A/C]	NA
Ca_Desi_Chr_4_18064760	Ca_desi_chr04	18064760	[G/T]	NA
Ca_Desi_Chr_4_18064793	Ca_desi_chr04	18064793	[A/G]	NA
Ca_Desi_Chr_4_18064806	Ca_desi_chr04	18064806	[A/G]	NA
Ca_Desi_Chr_4_18117934	Ca_desi_chr04	18117934	[A/G]	NA
Ca_Desi_Chr_4_18148828	Ca_desi_chr04	18148828	[T/A]	NA
Ca_Desi_Chr_4_18198384	Ca_desi_chr04	18198384	[A/G]	NA
Ca_Desi_Chr_4_18198406	Ca_desi_chr04	18198406	[A/G]	NA
Ca_Desi_Chr_4_18198419	Ca_desi_chr04	18198419	[G/A]	NA
Ca_Desi_Chr_4_18198420	Ca_desi_chr04	18198420	[A/G]	NA
Ca_Desi_Chr_4_18198434	Ca_desi_chr04	18198434	[T/C]	NA
Ca_Desi_Chr_4_18198440	Ca_desi_chr04	18198440	[T/C]	NA
Ca_Desi_Chr_4_18240126	Ca_desi_chr04	18240126	[A/T]	NA
Ca_Desi_Chr_4_18280158	Ca_desi_chr04	18280158	[A/C]	NA
Ca_Desi_Chr_4_18291886	Ca_desi_chr04	18291886	[A/C]	NA

SNP IDs	Chromosomes	Physical positions (bp)	SNPs	NCBI SNPdb accession IDs (NCBI_ss#)
Ca_Desi_Chr_4_18319776	Ca_desi_chr04	18319776	[A/C]	NA
Ca_Desi_Chr_4_18319783	Ca_desi_chr04	18319783	[A/G]	NA
Ca_Desi_Chr_4_18319792	Ca_desi_chr04	18319792	[T/C]	NA
Ca_Desi_Chr_4_18319797	Ca_desi_chr04	18319797	[T/C]	NA
Ca_Desi_Chr_4_18319809	Ca_desi_chr04	18319809	[T/A]	NA
Ca_Desi_Chr_4_18321956	Ca_desi_chr04	18321956	[T/A]	NA
Ca_Desi_Chr_4_18322603	Ca_desi_chr04	18322603	[A/G]	NA
Ca_Desi_Chr_4_18351480	Ca_desi_chr04	18351480	[T/G]	NA
Ca_Desi_Chr_4_18487120	Ca_desi_chr04	18487120	[T/C]	NA
Ca_Desi_Chr_4_18487130	Ca_desi_chr04	18487130	[A/T]	NA
Ca_Desi_Chr_4_18487141	Ca_desi_chr04	18487141	[T/G]	NA
Ca_Desi_Chr_4_18522023	Ca_desi_chr04	18522023	[T/G]	NA
Ca_Desi_Chr_4_18632703	Ca_desi_chr04	18632703	[T/G]	NA
Ca_Desi_Chr_4_18643701	Ca_desi_chr04	18643701	[A/G]	NA
Ca_Desi_Chr_4_18643704	Ca_desi_chr04	18643704	[T/C]	NA
Ca_Desi_Chr_4_18643718	Ca_desi_chr04	18643718	[T/C]	NA
Ca_Desi_Chr_4_18645215	Ca_desi_chr04	18645215	[A/G]	NA
Ca_Desi_Chr_4_18661657	Ca_desi_chr04	18661657	[A/G]	NA
Ca_Desi_Chr_4_18663062	Ca_desi_chr04	18663062	[T/C]	NA
Ca_Desi_Chr_4_18663080	Ca_desi_chr04	18663080	[T/C]	NA
Ca_Desi_Chr_4_18687613	Ca_desi_chr04	18687613	[T/G]	NA
Ca_Desi_Chr_4_18778202	Ca_desi_chr04	18778202	[A/C]	NA
Ca_Desi_Chr_4_18778215	Ca_desi_chr04	18778215	[T/A]	NA
Ca_Desi_Chr_4_18790760	Ca_desi_chr04	18790760	[G/A]	NA
Ca_Desi_Chr_4_18798562	Ca_desi_chr04	18798562	[C/A]	NA
Ca_Desi_Chr_4_18798674	Ca_desi_chr04	18798674	[A/C]	NA
Ca_Desi_Chr_4_18821576	Ca_desi_chr04	18821576	[T/G]	NA
Ca_Desi_Chr_4_18846803	Ca_desi_chr04	18846803	[A/C]	NA
Ca_Desi_Chr_4_18847969	Ca_desi_chr04	18847969	[A/G]	NA
Ca_Desi_Chr_4_18847971	Ca_desi_chr04	18847971	[T/C]	NA
Ca_Desi_Chr_4_18847981	Ca_desi_chr04	18847981	[A/T]	NA
Ca_Desi_Chr_4_18848005	Ca_desi_chr04	18848005	[A/T]	NA
Ca_Desi_Chr_4_18855469	Ca_desi_chr04	18855469	[T/G]	NA
Ca_Desi_Chr_4_18876605	Ca_desi_chr04	18876605	[A/C]	NA

SNP IDs	Chromosomes	Physical positions (bp)	SNPs	NCBI SNPdb accession IDs (NCBI_ss#)
Ca_Desi_Chr_4_18921404	Ca_desi_chr04	18921404	[A/C]	NA
Ca_Desi_Chr_4_18925847	Ca_desi_chr04	18925847	[T/C]	NA
Ca_Desi_Chr_4_18925851	Ca_desi_chr04	18925851	[T/C]	NA
Ca_Desi_Chr_4_18925861	Ca_desi_chr04	18925861	[T/C]	NA
Ca_Desi_Chr_4_18925866	Ca_desi_chr04	18925866	[A/G]	NA
Ca_Desi_Chr_4_18925873	Ca_desi_chr04	18925873	[A/G]	NA
Ca_Desi_Chr_4_18925879	Ca_desi_chr04	18925879	[T/C]	NA
Ca_Desi_Chr_4_18925881	Ca_desi_chr04	18925881	[T/C]	NA
Ca_Desi_Chr_4_18925905	Ca_desi_chr04	18925905	[T/C]	NA
Ca_Desi_Chr_4_18929073	Ca_desi_chr04	18929073	[T/G]	NA
Ca_Desi_Chr_4_18929079	Ca_desi_chr04	18929079	[T/G]	NA
Ca_Desi_Chr_4_18929106	Ca_desi_chr04	18929106	[T/C]	NA
Ca_Desi_Chr_4_18929115	Ca_desi_chr04	18929115	[T/G]	NA
Ca_Desi_Chr_4_18929121	Ca_desi_chr04	18929121	[A/G]	NA
Ca_Desi_Chr_4_19026642	Ca_desi_chr04	19026642	[C/A]	NA
Ca_Desi_Chr_4_19026644	Ca_desi_chr04	19026644	[C/A]	NA
Ca_Desi_Chr_4_19026645	Ca_desi_chr04	19026645	[C/T]	NA
Ca_Desi_Chr_4_20306540	Ca_desi_chr04	20306540	[A/T]	NA
Ca_Desi_Chr_4_20306561	Ca_desi_chr04	20306561	[T/G]	NA
Ca_Desi_Chr_4_20306564	Ca_desi_chr04	20306564	[A/C]	NA
Ca_Desi_Chr_4_20317283	Ca_desi_chr04	20317283	[T/G]	NA
Ca_Desi_Chr_4_20331123	Ca_desi_chr04	20331123	[T/C]	NA
Ca_Desi_Chr_4_20331138	Ca_desi_chr04	20331138	[A/C]	NA
Ca_Desi_Chr_4_20331175	Ca_desi_chr04	20331175	[G/C]	NA
Ca_Desi_Chr_4_20331211	Ca_desi_chr04	20331211	[A/C]	NA
Ca_Desi_Chr_4_20331255	Ca_desi_chr04	20331255	[A/G]	NA
Ca_Desi_Chr_4_20360811	Ca_desi_chr04	20360811	[T/G]	NA
Ca_Desi_Chr_4_20360852	Ca_desi_chr04	20360852	[T/C]	NA
Ca_Desi_Chr_4_20386426	Ca_desi_chr04	20386426	[T/G]	NA
Ca_Desi_Chr_4_20386458	Ca_desi_chr04	20386458	[C/A]	NA
Ca_Desi_Chr_4_20386459	Ca_desi_chr04	20386459	[A/C]	NA
Ca_Desi_Chr_4_20386460	Ca_desi_chr04	20386460	[A/G]	NA
Ca_Desi_Chr_4_20386465	Ca_desi_chr04	20386465	[G/A]	NA
Ca_Desi_Chr_4_20386548	Ca_desi_chr04	20386548	[A/G]	NA

SNP IDs	Chromosomes	Physical positions (bp)	SNPs	NCBI SNPdb accession IDs (NCBI_ss#)
Ca_Desi_Chr_4_20386550	Ca_desi_chr04	20386550	[T/G]	NA
Ca_Desi_Chr_4_20388102	Ca_desi_chr04	20388102	[T/G]	NA
Ca_Desi_Chr_4_20397473	Ca_desi_chr04	20397473	[A/C]	NA
Ca_Desi_Chr_4_20397475	Ca_desi_chr04	20397475	[C/T]	NA
Ca_Desi_Chr_4_20412433	Ca_desi_chr04	20412433	[A/T]	NA
Ca_Desi_Chr_4_20412441	Ca_desi_chr04	20412441	[T/G]	NA
Ca_Desi_Chr_4_20430508	Ca_desi_chr04	20430508	[C/T]	NA
Ca_Desi_Chr_4_20507409	Ca_desi_chr04	20507409	[A/G]	NA
Ca_Desi_Chr_4_20507419	Ca_desi_chr04	20507419	[T/C]	NA
Ca_Desi_Chr_4_20507431	Ca_desi_chr04	20507431	[T/C]	NA
Ca_Desi_Chr_4_20507436	Ca_desi_chr04	20507436	[A/G]	NA
Ca_Desi_Chr_4_20507439	Ca_desi_chr04	20507439	[T/C]	NA
Ca_Desi_Chr_4_20507457	Ca_desi_chr04	20507457	[T/C]	NA
Ca_Desi_Chr_4_20507459	Ca_desi_chr04	20507459	[C/T]	NA
Ca_Desi_Chr_4_20507462	Ca_desi_chr04	20507462	[T/C]	NA
Ca_Desi_Chr_4_20507477	Ca_desi_chr04	20507477	[A/G]	NA
Ca_Desi_Chr_4_20557113	Ca_desi_chr04	20557113	[A/G]	NA
Ca_Desi_Chr_4_20557160	Ca_desi_chr04	20557160	[A/C]	NA
Ca_Desi_Chr_4_20557215	Ca_desi_chr04	20557215	[A/G]	NA
Ca_Desi_Chr_4_20557218	Ca_desi_chr04	20557218	[T/G]	NA
Ca_Desi_Chr_4_20557281	Ca_desi_chr04	20557281	[A/T]	NA
Ca_Desi_Chr_4_20574069	Ca_desi_chr04	20574069	[T/C]	NA
Ca_Desi_Chr_4_20574072	Ca_desi_chr04	20574072	[A/C]	NA
Ca_Desi_Chr_4_20585908	Ca_desi_chr04	20585908	[T/C]	NA
Ca_Desi_Chr_4_20585933	Ca_desi_chr04	20585933	[T/C]	NA
Ca_Desi_Chr_4_20585942	Ca_desi_chr04	20585942	[T/C]	NA
Ca_Desi_Chr_4_20587971	Ca_desi_chr04	20587971	[T/G]	NA
Ca_Desi_Chr_4_20588491	Ca_desi_chr04	20588491	[T/G]	NA
Ca_Desi_Chr_4_20588528	Ca_desi_chr04	20588528	[A/C]	NA
Ca_Desi_Chr_4_20633483	Ca_desi_chr04	20633483	[A/C]	NA
Ca_Desi_Chr_4_20643346	Ca_desi_chr04	20643346	[T/C]	NA
Ca_Desi_Chr_4_20643353	Ca_desi_chr04	20643353	[T/C]	NA
Ca_Desi_Chr_4_20643378	Ca_desi_chr04	20643378	[A/C]	NA
Ca_Desi_Chr_4_20757296	Ca_desi_chr04	20757296	[A/C]	NA

SNP IDs	Chromosomes	Physical positions (bp)	SNPs	NCBI SNPdb accession IDs (NCBI_ss#)
Ca_Desi_Chr_4_20776317	Ca_desi_chr04	20776317	[T/C]	NA
Ca_Desi_Chr_4_20833510	Ca_desi_chr04	20833510	[T/C]	NA
Ca_Desi_Chr_4_20839259	Ca_desi_chr04	20839259	[A/G]	NA
Ca_Desi_Chr_4_20841182	Ca_desi_chr04	20841182	[A/C]	NA
Ca_Desi_Chr_4_20855255	Ca_desi_chr04	20855255	[T/C]	NA
Ca_Desi_Chr_4_20855302	Ca_desi_chr04	20855302	[T/C]	NA
Ca_Desi_Chr_4_20855332	Ca_desi_chr04	20855332	[A/C]	NA
Ca_Desi_Chr_4_20855381	Ca_desi_chr04	20855381	[A/G]	NA
Ca_Desi_Chr_4_20870761	Ca_desi_chr04	20870761	[T/C]	NA
Ca_Desi_Chr_4_20870767	Ca_desi_chr04	20870767	[A/T]	NA
Ca_Desi_Chr_4_20878436	Ca_desi_chr04	20878436	[A/G]	NA
Ca_Desi_Chr_4_20878440	Ca_desi_chr04	20878440	[T/G]	NA
Ca_Desi_Chr_4_20878441	Ca_desi_chr04	20878441	[A/C]	NA
Ca_Desi_Chr_4_20878459	Ca_desi_chr04	20878459	[A/T]	NA
Ca_Desi_Chr_4_20878471	Ca_desi_chr04	20878471	[T/C]	NA
Ca_Desi_Chr_4_20879043	Ca_desi_chr04	20879043	[T/C]	NA
Ca_Desi_Chr_4_20879049	Ca_desi_chr04	20879049	[A/T]	NA
Ca_Desi_Chr_4_20905526	Ca_desi_chr04	20905526	[A/G]	NA
Ca_Desi_Chr_4_20905539	Ca_desi_chr04	20905539	[G/C]	NA
Ca_Desi_Chr_4_20905544	Ca_desi_chr04	20905544	[G/C]	NA
Ca_Desi_Chr_4_20905555	Ca_desi_chr04	20905555	[A/C]	NA
Ca_Desi_Chr_4_20907952	Ca_desi_chr04	20907952	[T/G]	NA
Ca_Desi_Chr_4_20907973	Ca_desi_chr04	20907973	[T/G]	NA
Ca_Desi_Chr_4_20908040	Ca_desi_chr04	20908040	[A/G]	NA
Ca_Desi_Chr_4_20908047	Ca_desi_chr04	20908047	[A/C]	NA
Ca_Desi_Chr_4_20975702	Ca_desi_chr04	20975702	[A/C]	NA
Ca_Desi_Chr_4_20975750	Ca_desi_chr04	20975750	[T/C]	NA
Ca_Desi_Chr_4_20975760	Ca_desi_chr04	20975760	[A/G]	NA
Ca_Desi_Chr_4_20975772	Ca_desi_chr04	20975772	[A/G]	NA
Ca_Desi_Chr_4_20975774	Ca_desi_chr04	20975774	[G/C]	NA
Ca_Desi_Chr_4_20975800	Ca_desi_chr04	20975800	[T/C]	NA
Ca_Desi_Chr_4_20975802	Ca_desi_chr04	20975802	[A/G]	NA
Ca_Desi_Chr_4_20975803	Ca_desi_chr04	20975803	[A/G]	NA
Ca_Desi_Chr_4_20975804	Ca_desi_chr04	20975804	[A/C]	NA

SNP IDs	Chromosomes	Physical positions (bp)	SNPs	NCBI SNPdb accession IDs (NCBI_ss#)
Ca_Desi_Chr_4_20975815	Ca_desi_chr04	20975815	[T/C]	NA
Ca_Desi_Chr_4_21045582	Ca_desi_chr04	21045582	[T/C]	NA
Ca_Desi_Chr_4_21045586	Ca_desi_chr04	21045586	[A/C]	NA
Ca_Desi_Chr_4_21045613	Ca_desi_chr04	21045613	[A/G]	NA
Ca_Desi_Chr_4_21045617	Ca_desi_chr04	21045617	[A/G]	NA
Ca_Desi_Chr_4_21045624	Ca_desi_chr04	21045624	[T/C]	NA
Ca_Desi_Chr_4_21051249	Ca_desi_chr04	21051249	[C/A]	NA
Ca_Desi_Chr_4_21060603	Ca_desi_chr04	21060603	[A/G]	NA
Ca_Desi_Chr_4_21113561	Ca_desi_chr04	21113561	[A/C]	NA
Ca_Desi_Chr_4_21113571	Ca_desi_chr04	21113571	[A/G]	NA
Ca_Desi_Chr_4_21113576	Ca_desi_chr04	21113576	[T/C]	NA
Ca_Desi_Chr_4_21144274	Ca_desi_chr04	21144274	[T/C]	NA
Ca_Desi_Chr_4_21144276	Ca_desi_chr04	21144276	[A/G]	NA
Ca_Desi_Chr_4_21144278	Ca_desi_chr04	21144278	[T/C]	NA
Ca_Desi_Chr_4_21144287	Ca_desi_chr04	21144287	[A/G]	NA
Ca_Desi_Chr_4_21166975	Ca_desi_chr04	21166975	[T/G]	NA
Ca_Desi_Chr_4_21166978	Ca_desi_chr04	21166978	[A/G]	NA
Ca_Desi_Chr_4_21166981	Ca_desi_chr04	21166981	[T/G]	NA
Ca_Desi_Chr_4_21166991	Ca_desi_chr04	21166991	[A/G]	NA
Ca_Desi_Chr_4_21166994	Ca_desi_chr04	21166994	[A/G]	NA
Ca_Desi_Chr_4_21166998	Ca_desi_chr04	21166998	[A/G]	NA
Ca_Desi_Chr_4_21167003	Ca_desi_chr04	21167003	[T/C]	NA
Ca_Desi_Chr_4_21182020	Ca_desi_chr04	21182020	[A/T]	NA
Ca_Desi_Chr_4_21191220	Ca_desi_chr04	21191220	[T/A]	NA
Ca_Desi_Chr_4_21191250	Ca_desi_chr04	21191250	[C/A]	NA
Ca_Desi_Chr_4_21191251	Ca_desi_chr04	21191251	[G/C]	NA
Ca_Desi_Chr_4_21191258	Ca_desi_chr04	21191258	[A/C]	NA
Ca_Desi_Chr_4_21191260	Ca_desi_chr04	21191260	[T/C]	NA
Ca_Desi_Chr_4_21192467	Ca_desi_chr04	21192467	[A/C]	NA
Ca_Desi_Chr_4_21192468	Ca_desi_chr04	21192468	[C/A]	NA
Ca_Desi_Chr_4_21192494	Ca_desi_chr04	21192494	[A/T]	NA
Ca_Desi_Chr_4_21192547	Ca_desi_chr04	21192547	[A/C]	NA
Ca_Desi_Chr_4_21192578	Ca_desi_chr04	21192578	[T/C]	NA
Ca_Desi_Chr_4_21229885	Ca_desi_chr04	21229885	[A/C]	NA

SNP IDs	Chromosomes	Physical positions (bp)	SNPs	NCBI SNPdb accession IDs (NCBI_ss#)
Ca_Desi_Chr_4_21231303	Ca_desi_chr04	21231303	[T/C]	NA
Ca_Desi_Chr_4_21238813	Ca_desi_chr04	21238813	[A/C]	NA
Ca_Desi_Chr_4_21254265	Ca_desi_chr04	21254265	[C/G]	NA
Ca_Desi_Chr_4_21254266	Ca_desi_chr04	21254266	[T/G]	NA
Ca_Desi_Chr_4_21380333	Ca_desi_chr04	21380333	[A/G]	NA
Ca_Desi_Chr_4_21380344	Ca_desi_chr04	21380344	[T/G]	NA
Ca_Desi_Chr_4_21380380	Ca_desi_chr04	21380380	[A/G]	NA
Ca_Desi_Chr_4_21382192	Ca_desi_chr04	21382192	[T/C]	NA
Ca_Desi_Chr_4_21385594	Ca_desi_chr04	21385594	[T/G]	NA
Ca_Desi_Chr_4_21385596	Ca_desi_chr04	21385596	[A/G]	NA
Ca_Desi_Chr_4_21385604	Ca_desi_chr04	21385604	[T/C]	NA
Ca_Desi_Chr_4_21385607	Ca_desi_chr04	21385607	[T/C]	NA
Ca_Desi_Chr_4_21385613	Ca_desi_chr04	21385613	[A/G]	NA
Ca_Desi_Chr_4_21385616	Ca_desi_chr04	21385616	[A/G]	NA
Ca_Desi_Chr_4_21385628	Ca_desi_chr04	21385628	[T/C]	NA
Ca_Desi_Chr_4_21385631	Ca_desi_chr04	21385631	[T/C]	NA
Ca_Desi_Chr_4_21385654	Ca_desi_chr04	21385654	[A/G]	NA
Ca_Desi_Chr_4_21385688	Ca_desi_chr04	21385688	[T/C]	NA
Ca_Desi_Chr_4_21387958	Ca_desi_chr04	21387958	[T/C]	NA
Ca_Desi_Chr_4_21650367	Ca_desi_chr04	21650367	[T/C]	NA
Ca_Desi_Chr_4_21654440	Ca_desi_chr04	21654440	[A/C]	NA
Ca_Desi_Chr_4_21678248	Ca_desi_chr04	21678248	[T/A]	NA
Ca_Desi_Chr_4_21746585	Ca_desi_chr04	21746585	[G/A]	NA
Ca_Desi_Chr_4_21746588	Ca_desi_chr04	21746588	[T/G]	NA
Ca_Desi_Chr_4_21747380	Ca_desi_chr04	21747380	[A/C]	NA
Ca_Desi_Chr_4_21752105	Ca_desi_chr04	21752105	[T/C]	NA
Ca_Desi_Chr_4_21752109	Ca_desi_chr04	21752109	[A/G]	NA
Ca_Desi_Chr_4_21752165	Ca_desi_chr04	21752165	[A/G]	NA
Ca_Desi_Chr_4_21752213	Ca_desi_chr04	21752213	[A/G]	NA
Ca_Desi_Chr_4_21752228	Ca_desi_chr04	21752228	[A/G]	NA
Ca_Desi_Chr_4_21752234	Ca_desi_chr04	21752234	[A/G]	NA
Ca_Desi_Chr_4_21752237	Ca_desi_chr04	21752237	[A/G]	NA
Ca_Desi_Chr_4_21752246	Ca_desi_chr04	21752246	[T/G]	NA
Ca_Desi_Chr_4_21752255	Ca_desi_chr04	21752255	[T/G]	NA

SNP IDs	Chromosomes	Physical positions (bp)	SNPs	NCBI SNPdb accession IDs (NCBI_ss#)
Ca_Desi_Chr_4_21752258	Ca_desi_chr04	21752258	[T/C]	NA
Ca_Desi_Chr_4_21752270	Ca_desi_chr04	21752270	[T/C]	NA
Ca_Desi_Chr_4_21788204	Ca_desi_chr04	21788204	[A/T]	NA
Ca_Desi_Chr_4_21806082	Ca_desi_chr04	21806082	[A/C]	NA
Ca_Desi_Chr_4_21811973	Ca_desi_chr04	21811973	[A/C]	NA
Ca_Desi_Chr_4_21829118	Ca_desi_chr04	21829118	[A/G]	NA
Ca_Desi_Chr_4_21829146	Ca_desi_chr04	21829146	[T/C]	NA
Ca_Desi_Chr_4_21829160	Ca_desi_chr04	21829160	[T/G]	NA
Ca_Desi_Chr_4_21829165	Ca_desi_chr04	21829165	[T/G]	NA
Ca_Desi_Chr_4_21830583	Ca_desi_chr04	21830583	[T/A]	NA
Ca_Desi_Chr_4_21846456	Ca_desi_chr04	21846456	[A/G]	NA
Ca_Desi_Chr_4_21846482	Ca_desi_chr04	21846482	[T/A]	NA
Ca_Desi_Chr_4_21846507	Ca_desi_chr04	21846507	[A/C]	NA
Ca_Desi_Chr_4_21887275	Ca_desi_chr04	21887275	[C/A]	NA
Ca_Desi_Chr_4_21887293	Ca_desi_chr04	21887293	[A/C]	NA
Ca_Desi_Chr_4_21887299	Ca_desi_chr04	21887299	[A/C]	NA
Ca_Desi_Chr_4_21887305	Ca_desi_chr04	21887305	[A/C]	NA
Ca_Desi_Chr_4_21895581	Ca_desi_chr04	21895581	[T/G]	NA
Ca_Desi_Chr_4_21896088	Ca_desi_chr04	21896088	[G/T]	NA
Ca_Desi_Chr_4_21906328	Ca_desi_chr04	21906328	[T/G]	NA
Ca_Desi_Chr_4_21924957	Ca_desi_chr04	21924957	[G/T]	NA
Ca_Desi_Chr_4_21928532	Ca_desi_chr04	21928532	[C/T]	NA
Ca_Desi_Chr_4_21928536	Ca_desi_chr04	21928536	[C/T]	NA
Ca_Desi_Chr_4_21967908	Ca_desi_chr04	21967908	[T/C]	NA
Ca_Desi_Chr_4_21981995	Ca_desi_chr04	21981995	[T/G]	NA
Ca_Desi_Chr_4_21987497	Ca_desi_chr04	21987497	[T/C]	NA
Ca_Desi_Chr_4_21987510	Ca_desi_chr04	21987510	[A/C]	NA
Ca_Desi_Chr_4_21987524	Ca_desi_chr04	21987524	[T/C]	NA
Ca_Desi_Chr_4_21987527	Ca_desi_chr04	21987527	[A/G]	NA
Ca_Desi_Chr_4_21987529	Ca_desi_chr04	21987529	[T/C]	NA
Ca_Desi_Chr_4_22000319	Ca_desi_chr04	22000319	[T/G]	NA
Ca_Desi_Chr_4_22000353	Ca_desi_chr04	22000353	[T/C]	NA
Ca_Desi_Chr_4_22000417	Ca_desi_chr04	22000417	[T/C]	NA
Ca_Desi_Chr_4_22000430	Ca_desi_chr04	22000430	[A/C]	NA

SNP IDs	Chromosomes	Physical positions (bp)	SNPs	NCBI SNPdb accession IDs (NCBI_ss#)
Ca_Desi_Chr_4_22000431	Ca_desi_chr04	22000431	[G/T]	NA
Ca_Desi_Chr_4_22000435	Ca_desi_chr04	22000435	[T/C]	NA
Ca_Desi_Chr_4_22000449	Ca_desi_chr04	22000449	[T/C]	NA
Ca_Desi_Chr_4_22035738	Ca_desi_chr04	22035738	[C/G]	NA
Ca_Desi_Chr_4_22035742	Ca_desi_chr04	22035742	[G/A]	NA
Ca_Desi_Chr_4_22035744	Ca_desi_chr04	22035744	[A/G]	NA
Ca_Desi_Chr_4_22035746	Ca_desi_chr04	22035746	[C/A]	NA
Ca_Desi_Chr_4_22037164	Ca_desi_chr04	22037164	[A/G]	NA
Ca_Desi_Chr_4_22037167	Ca_desi_chr04	22037167	[A/G]	NA
Ca_Desi_Chr_4_22037171	Ca_desi_chr04	22037171	[G/A]	NA
Ca_Desi_Chr_4_22037173	Ca_desi_chr04	22037173	[G/A]	NA
Ca_Desi_Chr_4_22037195	Ca_desi_chr04	22037195	[T/A]	NA
Ca_Desi_Chr_4_22037304	Ca_desi_chr04	22037304	[A/G]	NA
Ca_Desi_Chr_4_22037306	Ca_desi_chr04	22037306	[C/G]	NA
Ca_Desi_Chr_5_36933	Ca_desi_chr05	36933	[T/G]	NA
Ca_Desi_Chr_5_36972	Ca_desi_chr05	36972	[A/G]	NA
Ca_Desi_Chr_5_64605	Ca_desi_chr05	64605	[A/G]	NA
Ca_Desi_Chr_5_96270	Ca_desi_chr05	96270	[A/G]	NA
Ca_Desi_Chr_5_96281	Ca_desi_chr05	96281	[A/G]	NA
Ca_Desi_Chr_5_100208	Ca_desi_chr05	100208	[T/C]	NA
Ca_Desi_Chr_5_100228	Ca_desi_chr05	100228	[A/C]	NA
Ca_Desi_Chr_5_100234	Ca_desi_chr05	100234	[T/C]	NA
Ca_Desi_Chr_5_100239	Ca_desi_chr05	100239	[A/G]	NA
Ca_Desi_Chr_5_100248	Ca_desi_chr05	100248	[T/C]	NA
Ca_Desi_Chr_5_100249	Ca_desi_chr05	100249	[A/G]	NA
Ca_Desi_Chr_5_100307	Ca_desi_chr05	100307	[A/G]	NA
Ca_Desi_Chr_5_100330	Ca_desi_chr05	100330	[A/G]	NA
Ca_Desi_Chr_5_100334	Ca_desi_chr05	100334	[A/C]	NA
Ca_Desi_Chr_5_100967	Ca_desi_chr05	100967	[T/C]	NA
Ca_Desi_Chr_5_154275	Ca_desi_chr05	154275	[A/C]	NA
Ca_Desi_Chr_5_154312	Ca_desi_chr05	154312	[A/C]	NA
Ca_Desi_Chr_5_264254	Ca_desi_chr05	264254	[C/T]	NA
Ca_Desi_Chr_5_452391	Ca_desi_chr05	452391	[A/G]	NA
Ca_Desi_Chr_5_452406	Ca_desi_chr05	452406	[A/G]	NA

SNP IDs	Chromosomes	Physical positions (bp)	SNPs	NCBI SNPdb accession IDs (NCBI_ss#)
Ca_Desi_Chr_5_452416	Ca_desi_chr05	452416	[A/G]	NA
Ca_Desi_Chr_5_452425	Ca_desi_chr05	452425	[T/G]	NA
Ca_Desi_Chr_5_546601	Ca_desi_chr05	546601	[A/C]	NA
Ca_Desi_Chr_5_558115	Ca_desi_chr05	558115	[T/G]	NA
Ca_Desi_Chr_5_558133	Ca_desi_chr05	558133	[T/G]	NA
Ca_Desi_Chr_5_600687	Ca_desi_chr05	600687	[A/T]	NA
Ca_Desi_Chr_5_601816	Ca_desi_chr05	601816	[T/C]	NA
Ca_Desi_Chr_5_601827	Ca_desi_chr05	601827	[T/G]	NA
Ca_Desi_Chr_5_601838	Ca_desi_chr05	601838	[T/G]	NA
Ca_Desi_Chr_5_671968	Ca_desi_chr05	671968	[A/G]	NA
Ca_Desi_Chr_5_671985	Ca_desi_chr05	671985	[T/A]	NA
Ca_Desi_Chr_5_689741	Ca_desi_chr05	689741	[T/A]	NA
Ca_Desi_Chr_5_1291893	Ca_desi_chr05	1291893	[T/G]	NA
Ca_Desi_Chr_5_1291903	Ca_desi_chr05	1291903	[C/A]	NA
Ca_Desi_Chr_5_1291921	Ca_desi_chr05	1291921	[T/C]	NA
Ca_Desi_Chr_5_1291939	Ca_desi_chr05	1291939	[A/G]	NA
Ca_Desi_Chr_5_1291944	Ca_desi_chr05	1291944	[A/G]	NA
Ca_Desi_Chr_5_1291947	Ca_desi_chr05	1291947	[A/T]	NA
Ca_Desi_Chr_5_1333612	Ca_desi_chr05	1333612	[T/C]	NA
Ca_Desi_Chr_5_1333641	Ca_desi_chr05	1333641	[C/T]	NA
Ca_Desi_Chr_5_1333643	Ca_desi_chr05	1333643	[A/G]	NA
Ca_Desi_Chr_5_1333644	Ca_desi_chr05	1333644	[A/G]	NA
Ca_Desi_Chr_5_1333659	Ca_desi_chr05	1333659	[T/C]	NA
Ca_Desi_Chr_5_1333662	Ca_desi_chr05	1333662	[T/A]	NA
Ca_Desi_Chr_5_1361879	Ca_desi_chr05	1361879	[T/G]	NA
Ca_Desi_Chr_5_1385612	Ca_desi_chr05	1385612	[T/A]	NA
Ca_Desi_Chr_5_1385613	Ca_desi_chr05	1385613	[C/T]	NA
Ca_Desi_Chr_5_1385614	Ca_desi_chr05	1385614	[G/C]	NA
Ca_Desi_Chr_5_1385615	Ca_desi_chr05	1385615	[G/T]	NA
Ca_Desi_Chr_5_1398738	Ca_desi_chr05	1398738	[C/T]	NA
Ca_Desi_Chr_5_1434852	Ca_desi_chr05	1434852	[T/C]	NA
Ca_Desi_Chr_5_1435472	Ca_desi_chr05	1435472	[T/A]	NA
Ca_Desi_Chr_5_1440852	Ca_desi_chr05	1440852	[A/G]	NA
Ca_Desi_Chr_5_1440857	Ca_desi_chr05	1440857	[T/G]	NA

SNP IDs	Chromosomes	Physical positions (bp)	SNPs	NCBI SNPdb accession IDs (NCBI_ss#)
Ca_Desi_Chr_5_1440868	Ca_desi_chr05	1440868	[T/G]	NA
Ca_Desi_Chr_5_1440870	Ca_desi_chr05	1440870	[T/C]	NA
Ca_Desi_Chr_5_1440886	Ca_desi_chr05	1440886	[T/C]	NA
Ca_Desi_Chr_5_1440891	Ca_desi_chr05	1440891	[T/C]	NA
Ca_Desi_Chr_5_1440893	Ca_desi_chr05	1440893	[A/G]	NA
Ca_Desi_Chr_5_1440898	Ca_desi_chr05	1440898	[C/G]	NA
Ca_Desi_Chr_5_1443892	Ca_desi_chr05	1443892	[A/C]	NA
Ca_Desi_Chr_5_1443923	Ca_desi_chr05	1443923	[A/C]	NA
Ca_Desi_Chr_5_1471621	Ca_desi_chr05	1471621	[A/C]	NA
Ca_Desi_Chr_5_1478118	Ca_desi_chr05	1478118	[T/C]	NA
Ca_Desi_Chr_5_1478122	Ca_desi_chr05	1478122	[A/C]	NA
Ca_Desi_Chr_5_1483283	Ca_desi_chr05	1483283	[T/C]	NA
Ca_Desi_Chr_5_1483285	Ca_desi_chr05	1483285	[T/C]	NA
Ca_Desi_Chr_5_1483303	Ca_desi_chr05	1483303	[A/G]	NA
Ca_Desi_Chr_5_1483315	Ca_desi_chr05	1483315	[T/C]	NA
Ca_Desi_Chr_5_1483319	Ca_desi_chr05	1483319	[A/C]	NA
Ca_Desi_Chr_5_1483338	Ca_desi_chr05	1483338	[T/C]	NA
Ca_Desi_Chr_5_1483405	Ca_desi_chr05	1483405	[T/A]	NA
Ca_Desi_Chr_5_1487902	Ca_desi_chr05	1487902	[T/G]	NA
Ca_Desi_Chr_5_1498889	Ca_desi_chr05	1498889	[A/T]	NA
Ca_Desi_Chr_5_1498901	Ca_desi_chr05	1498901	[T/C]	NA
Ca_Desi_Chr_5_1498905	Ca_desi_chr05	1498905	[A/G]	NA
Ca_Desi_Chr_5_1498912	Ca_desi_chr05	1498912	[A/G]	NA
Ca_Desi_Chr_5_1498927	Ca_desi_chr05	1498927	[T/C]	NA
Ca_Desi_Chr_5_1498930	Ca_desi_chr05	1498930	[T/C]	NA
Ca_Desi_Chr_5_1498936	Ca_desi_chr05	1498936	[A/C]	NA
Ca_Desi_Chr_5_1520349	Ca_desi_chr05	1520349	[A/C]	NA
Ca_Desi_Chr_5_1605735	Ca_desi_chr05	1605735	[T/G]	NA
Ca_Desi_Chr_5_1605755	Ca_desi_chr05	1605755	[T/G]	NA
Ca_Desi_Chr_5_1608133	Ca_desi_chr05	1608133	[C/A]	NA
Ca_Desi_Chr_5_1608134	Ca_desi_chr05	1608134	[A/G]	NA
Ca_Desi_Chr_5_1615818	Ca_desi_chr05	1615818	[T/G]	NA
Ca_Desi_Chr_5_1615972	Ca_desi_chr05	1615972	[A/C]	NA
Ca_Desi_Chr_5_1631169	Ca_desi_chr05	1631169	[A/G]	NA

SNP IDs	Chromosomes	Physical positions (bp)	SNPs	NCBI SNPdb accession IDs (NCBI_ss#)
Ca_Desi_Chr_5_1631216	Ca_desi_chr05	1631216	[A/G]	NA
Ca_Desi_Chr_5_1671297	Ca_desi_chr05	1671297	[C/A]	NA
Ca_Desi_Chr_5_1671298	Ca_desi_chr05	1671298	[T/G]	NA
Ca_Desi_Chr_5_1702543	Ca_desi_chr05	1702543	[A/G]	NA
Ca_Desi_Chr_5_1702549	Ca_desi_chr05	1702549	[G/A]	NA
Ca_Desi_Chr_5_1702550	Ca_desi_chr05	1702550	[A/G]	NA
Ca_Desi_Chr_5_1702551	Ca_desi_chr05	1702551	[G/A]	NA
Ca_Desi_Chr_5_1702553	Ca_desi_chr05	1702553	[G/A]	NA
Ca_Desi_Chr_5_1702571	Ca_desi_chr05	1702571	[T/G]	NA
Ca_Desi_Chr_5_1815460	Ca_desi_chr05	1815460	[T/A]	NA
Ca_Desi_Chr_5_1914996	Ca_desi_chr05	1914996	[T/C]	NA
Ca_Desi_Chr_5_1915013	Ca_desi_chr05	1915013	[A/G]	NA
Ca_Desi_Chr_5_1976226	Ca_desi_chr05	1976226	[A/C]	NA
Ca_Desi_Chr_5_1980703	Ca_desi_chr05	1980703	[T/C]	NA
Ca_Desi_Chr_5_1991320	Ca_desi_chr05	1991320	[T/C]	NA
Ca_Desi_Chr_5_1991364	Ca_desi_chr05	1991364	[A/G]	NA
Ca_Desi_Chr_5_1993371	Ca_desi_chr05	1993371	[T/G]	NA
Ca_Desi_Chr_5_2036697	Ca_desi_chr05	2036697	[T/G]	NA
Ca_Desi_Chr_5_2303670	Ca_desi_chr05	2303670	[T/A]	NA
Ca_Desi_Chr_5_2319345	Ca_desi_chr05	2319345	[A/G]	NA
Ca_Desi_Chr_5_2319372	Ca_desi_chr05	2319372	[A/T]	NA
Ca_Desi_Chr_5_2325476	Ca_desi_chr05	2325476	[T/G]	NA
Ca_Desi_Chr_5_2340832	Ca_desi_chr05	2340832	[T/G]	NA
Ca_Desi_Chr_5_2340833	Ca_desi_chr05	2340833	[T/C]	NA
Ca_Desi_Chr_5_2340835	Ca_desi_chr05	2340835	[T/C]	NA
Ca_Desi_Chr_5_2357765	Ca_desi_chr05	2357765	[A/T]	NA
Ca_Desi_Chr_5_2418371	Ca_desi_chr05	2418371	[A/T]	NA
Ca_Desi_Chr_5_2439381	Ca_desi_chr05	2439381	[T/C]	NA
Ca_Desi_Chr_5_2524127	Ca_desi_chr05	2524127	[T/G]	NA
Ca_Desi_Chr_5_2562484	Ca_desi_chr05	2562484	[T/C]	NA
Ca_Desi_Chr_5_2562488	Ca_desi_chr05	2562488	[A/C]	NA
Ca_Desi_Chr_5_2562493	Ca_desi_chr05	2562493	[A/G]	NA
Ca_Desi_Chr_5_2562500	Ca_desi_chr05	2562500	[T/C]	NA
Ca_Desi_Chr_5_2562505	Ca_desi_chr05	2562505	[A/C]	NA

SNP IDs	Chromosomes	Physical positions (bp)	SNPs	NCBI SNPdb accession IDs (NCBI_ss#)
Ca_Desi_Chr_5_2562518	Ca_desi_chr05	2562518	[A/T]	NA
Ca_Desi_Chr_5_2562538	Ca_desi_chr05	2562538	[A/G]	NA
Ca_Desi_Chr_5_2562573	Ca_desi_chr05	2562573	[A/G]	NA
Ca_Desi_Chr_5_2562575	Ca_desi_chr05	2562575	[A/G]	NA
Ca_Desi_Chr_5_2562578	Ca_desi_chr05	2562578	[A/G]	NA
Ca_Desi_Chr_5_2701081	Ca_desi_chr05	2701081	[A/C]	NA
Ca_Desi_Chr_5_2701090	Ca_desi_chr05	2701090	[T/C]	NA
Ca_Desi_Chr_5_2743851	Ca_desi_chr05	2743851	[A/C]	NA
Ca_Desi_Chr_5_2743878	Ca_desi_chr05	2743878	[A/G]	NA
Ca_Desi_Chr_5_2745071	Ca_desi_chr05	2745071	[T/G]	NA
Ca_Desi_Chr_5_2875172	Ca_desi_chr05	2875172	[A/C]	NA
Ca_Desi_Chr_5_2885352	Ca_desi_chr05	2885352	[A/C]	NA
Ca_Desi_Chr_5_2904483	Ca_desi_chr05	2904483	[G/A]	NA
Ca_Desi_Chr_5_2904497	Ca_desi_chr05	2904497	[A/C]	NA
Ca_Desi_Chr_5_2904503	Ca_desi_chr05	2904503	[A/G]	NA
Ca_Desi_Chr_5_2983562	Ca_desi_chr05	2983562	[T/C]	NA
Ca_Desi_Chr_5_2983574	Ca_desi_chr05	2983574	[T/C]	NA
Ca_Desi_Chr_5_3046643	Ca_desi_chr05	3046643	[A/G]	NA
Ca_Desi_Chr_5_3046656	Ca_desi_chr05	3046656	[A/G]	NA
Ca_Desi_Chr_5_3047008	Ca_desi_chr05	3047008	[T/A]	NA
Ca_Desi_Chr_5_3047095	Ca_desi_chr05	3047095	[T/G]	NA
Ca_Desi_Chr_5_3047175	Ca_desi_chr05	3047175	[A/C]	NA
Ca_Desi_Chr_5_3047247	Ca_desi_chr05	3047247	[A/G]	NA
Ca_Desi_Chr_5_3047255	Ca_desi_chr05	3047255	[A/G]	NA
Ca_Desi_Chr_5_3047265	Ca_desi_chr05	3047265	[A/G]	NA
Ca_Desi_Chr_5_3047269	Ca_desi_chr05	3047269	[A/G]	NA
Ca_Desi_Chr_5_3047272	Ca_desi_chr05	3047272	[A/G]	NA
Ca_Desi_Chr_5_3047276	Ca_desi_chr05	3047276	[T/C]	NA
Ca_Desi_Chr_5_3047283	Ca_desi_chr05	3047283	[C/T]	NA
Ca_Desi_Chr_5_3047284	Ca_desi_chr05	3047284	[A/G]	NA
Ca_Desi_Chr_5_3068414	Ca_desi_chr05	3068414	[T/A]	NA
Ca_Desi_Chr_5_3068419	Ca_desi_chr05	3068419	[T/C]	NA
Ca_Desi_Chr_5_3068421	Ca_desi_chr05	3068421	[A/C]	NA
Ca_Desi_Chr_5_3068436	Ca_desi_chr05	3068436	[A/C]	NA

SNP IDs	Chromosomes	Physical positions (bp)	SNPs	NCBI SNPdb accession IDs (NCBI_ss#)
Ca_Desi_Chr_5_3069867	Ca_desi_chr05	3069867	[T/A]	NA
Ca_Desi_Chr_5_3069869	Ca_desi_chr05	3069869	[A/C]	NA
Ca_Desi_Chr_5_3164617	Ca_desi_chr05	3164617	[A/C]	NA
Ca_Desi_Chr_5_3164623	Ca_desi_chr05	3164623	[A/G]	NA
Ca_Desi_Chr_5_3177238	Ca_desi_chr05	3177238	[A/G]	NA
Ca_Desi_Chr_5_3177243	Ca_desi_chr05	3177243	[T/G]	NA
Ca_Desi_Chr_5_3177246	Ca_desi_chr05	3177246	[T/G]	NA
Ca_Desi_Chr_5_3177252	Ca_desi_chr05	3177252	[A/G]	NA
Ca_Desi_Chr_5_3177253	Ca_desi_chr05	3177253	[G/A]	NA
Ca_Desi_Chr_5_3177257	Ca_desi_chr05	3177257	[A/G]	NA
Ca_Desi_Chr_5_3177259	Ca_desi_chr05	3177259	[T/C]	NA
Ca_Desi_Chr_5_3177279	Ca_desi_chr05	3177279	[A/G]	NA
Ca_Desi_Chr_5_3177299	Ca_desi_chr05	3177299	[T/C]	NA
Ca_Desi_Chr_5_3214910	Ca_desi_chr05	3214910	[A/C]	NA
Ca_Desi_Chr_5_3215641	Ca_desi_chr05	3215641	[A/C]	NA
Ca_Desi_Chr_5_3218116	Ca_desi_chr05	3218116	[A/C]	NA
Ca_Desi_Chr_5_3218123	Ca_desi_chr05	3218123	[A/G]	NA
Ca_Desi_Chr_5_3218134	Ca_desi_chr05	3218134	[T/G]	NA
Ca_Desi_Chr_5_3218138	Ca_desi_chr05	3218138	[T/G]	NA
Ca_Desi_Chr_5_3218142	Ca_desi_chr05	3218142	[A/G]	NA
Ca_Desi_Chr_5_3218181	Ca_desi_chr05	3218181	[T/G]	NA
Ca_Desi_Chr_5_3219073	Ca_desi_chr05	3219073	[T/C]	NA
Ca_Desi_Chr_5_3219074	Ca_desi_chr05	3219074	[G/T]	NA
Ca_Desi_Chr_5_3220318	Ca_desi_chr05	3220318	[T/A]	NA
Ca_Desi_Chr_5_3220319	Ca_desi_chr05	3220319	[C/A]	NA
Ca_Desi_Chr_5_3220320	Ca_desi_chr05	3220320	[T/G]	NA
Ca_Desi_Chr_5_3253211	Ca_desi_chr05	3253211	[A/G]	NA
Ca_Desi_Chr_5_3262387	Ca_desi_chr05	3262387	[T/G]	NA
Ca_Desi_Chr_5_3264562	Ca_desi_chr05	3264562	[A/G]	NA
Ca_Desi_Chr_5_3264605	Ca_desi_chr05	3264605	[T/G]	NA
Ca_Desi_Chr_5_3373975	Ca_desi_chr05	3373975	[A/C]	NA
Ca_Desi_Chr_5_3373980	Ca_desi_chr05	3373980	[A/G]	NA
Ca_Desi_Chr_5_3373993	Ca_desi_chr05	3373993	[A/C]	NA
Ca_Desi_Chr_5_3405272	Ca_desi_chr05	3405272	[A/C]	NA

SNP IDs	Chromosomes	Physical positions (bp)	SNPs	NCBI SNPdb accession IDs (NCBI_ss#)
Ca_Desi_Chr_5_3405577	Ca_desi_chr05	3405577	[T/G]	NA
Ca_Desi_Chr_5_3405646	Ca_desi_chr05	3405646	[T/A]	NA
Ca_Desi_Chr_5_3405699	Ca_desi_chr05	3405699	[A/G]	NA
Ca_Desi_Chr_5_3405702	Ca_desi_chr05	3405702	[A/G]	NA
Ca_Desi_Chr_5_3409699	Ca_desi_chr05	3409699	[T/G]	NA
Ca_Desi_Chr_5_3409704	Ca_desi_chr05	3409704	[A/C]	NA
Ca_Desi_Chr_5_3416021	Ca_desi_chr05	3416021	[A/G]	NA
Ca_Desi_Chr_5_3416039	Ca_desi_chr05	3416039	[A/G]	NA
Ca_Desi_Chr_5_3416043	Ca_desi_chr05	3416043	[T/G]	NA
Ca_Desi_Chr_5_3416049	Ca_desi_chr05	3416049	[T/G]	NA
Ca_Desi_Chr_5_3416055	Ca_desi_chr05	3416055	[T/C]	NA
Ca_Desi_Chr_5_3416058	Ca_desi_chr05	3416058	[T/C]	NA
Ca_Desi_Chr_5_3416068	Ca_desi_chr05	3416068	[G/A]	NA
Ca_Desi_Chr_5_3416072	Ca_desi_chr05	3416072	[A/G]	NA
Ca_Desi_Chr_5_3416076	Ca_desi_chr05	3416076	[T/C]	NA
Ca_Desi_Chr_5_3416079	Ca_desi_chr05	3416079	[T/C]	NA
Ca_Desi_Chr_5_3441989	Ca_desi_chr05	3441989	[T/G]	NA
Ca_Desi_Chr_5_3459994	Ca_desi_chr05	3459994	[A/C]	NA
Ca_Desi_Chr_5_3460114	Ca_desi_chr05	3460114	[A/C]	NA
Ca_Desi_Chr_5_3490281	Ca_desi_chr05	3490281	[A/C]	NA
Ca_Desi_Chr_5_3524310	Ca_desi_chr05	3524310	[T/C]	NA
Ca_Desi_Chr_5_3541986	Ca_desi_chr05	3541986	[A/C]	NA
Ca_Desi_Chr_5_3541992	Ca_desi_chr05	3541992	[A/C]	NA
Ca_Desi_Chr_5_4413238	Ca_desi_chr05	4413238	[G/A]	NA
Ca_Desi_Chr_5_4413259	Ca_desi_chr05	4413259	[T/A]	NA
Ca_Desi_Chr_5_4413292	Ca_desi_chr05	4413292	[C/A]	NA
Ca_Desi_Chr_5_4413294	Ca_desi_chr05	4413294	[A/G]	NA
Ca_Desi_Chr_5_4413316	Ca_desi_chr05	4413316	[T/G]	NA
Ca_Desi_Chr_5_4413334	Ca_desi_chr05	4413334	[G/A]	NA
Ca_Desi_Chr_5_4413370	Ca_desi_chr05	4413370	[T/C]	NA
Ca_Desi_Chr_5_4413859	Ca_desi_chr05	4413859	[T/C]	NA
Ca_Desi_Chr_5_4414372	Ca_desi_chr05	4414372	[A/T]	NA
Ca_Desi_Chr_5_4414378	Ca_desi_chr05	4414378	[C/A]	NA
Ca_Desi_Chr_5_4414399	Ca_desi_chr05	4414399	[T/A]	NA

SNP IDs	Chromosomes	Physical positions (bp)	SNPs	NCBI SNPdb accession IDs (NCBI_ss#)
Ca_Desi_Chr_5_4431412	Ca_desi_chr05	4431412	[T/A]	NA
Ca_Desi_Chr_5_4431415	Ca_desi_chr05	4431415	[T/G]	NA
Ca_Desi_Chr_5_4447507	Ca_desi_chr05	4447507	[A/C]	NA
Ca_Desi_Chr_5_4447512	Ca_desi_chr05	4447512	[A/C]	NA
Ca_Desi_Chr_5_4462169	Ca_desi_chr05	4462169	[A/C]	NA
Ca_Desi_Chr_5_4519775	Ca_desi_chr05	4519775	[T/C]	NA
Ca_Desi_Chr_5_4540829	Ca_desi_chr05	4540829	[A/C]	NA
Ca_Desi_Chr_5_4563108	Ca_desi_chr05	4563108	[A/G]	NA
Ca_Desi_Chr_5_4563120	Ca_desi_chr05	4563120	[A/G]	NA
Ca_Desi_Chr_5_4703801	Ca_desi_chr05	4703801	[T/C]	NA
Ca_Desi_Chr_5_4703804	Ca_desi_chr05	4703804	[A/G]	NA
Ca_Desi_Chr_5_4703824	Ca_desi_chr05	4703824	[A/G]	NA
Ca_Desi_Chr_5_4753041	Ca_desi_chr05	4753041	[T/G]	NA
Ca_Desi_Chr_5_4779900	Ca_desi_chr05	4779900	[A/G]	NA
Ca_Desi_Chr_5_4779911	Ca_desi_chr05	4779911	[A/C]	NA
Ca_Desi_Chr_5_4837584	Ca_desi_chr05	4837584	[G/T]	NA
Ca_Desi_Chr_5_4837586	Ca_desi_chr05	4837586	[T/G]	NA
Ca_Desi_Chr_5_4837588	Ca_desi_chr05	4837588	[A/T]	NA
Ca_Desi_Chr_5_4837595	Ca_desi_chr05	4837595	[G/C]	NA
Ca_Desi_Chr_5_4837598	Ca_desi_chr05	4837598	[A/G]	NA
Ca_Desi_Chr_5_4837600	Ca_desi_chr05	4837600	[A/G]	NA
Ca_Desi_Chr_5_4837608	Ca_desi_chr05	4837608	[A/T]	NA
Ca_Desi_Chr_5_4853671	Ca_desi_chr05	4853671	[T/C]	NA
Ca_Desi_Chr_5_4909331	Ca_desi_chr05	4909331	[A/G]	NA
Ca_Desi_Chr_5_4909336	Ca_desi_chr05	4909336	[G/T]	NA
Ca_Desi_Chr_5_4909338	Ca_desi_chr05	4909338	[C/T]	NA
Ca_Desi_Chr_5_4910606	Ca_desi_chr05	4910606	[A/C]	NA
Ca_Desi_Chr_5_4931928	Ca_desi_chr05	4931928	[A/C]	NA
Ca_Desi_Chr_5_4931951	Ca_desi_chr05	4931951	[A/G]	NA
Ca_Desi_Chr_5_4939697	Ca_desi_chr05	4939697	[A/G]	NA
Ca_Desi_Chr_5_4939708	Ca_desi_chr05	4939708	[T/G]	NA
Ca_Desi_Chr_5_4939710	Ca_desi_chr05	4939710	[T/G]	NA
Ca_Desi_Chr_5_4939716	Ca_desi_chr05	4939716	[A/G]	NA
Ca_Desi_Chr_5_4939722	Ca_desi_chr05	4939722	[A/G]	NA

SNP IDs	Chromosomes	Physical positions (bp)	SNPs	NCBI SNPdb accession IDs (NCBI_ss#)
Ca_Desi_Chr_5_4939728	Ca_desi_chr05	4939728	[T/G]	NA
Ca_Desi_Chr_5_4939731	Ca_desi_chr05	4939731	[A/G]	NA
Ca_Desi_Chr_5_4939735	Ca_desi_chr05	4939735	[A/G]	NA
Ca_Desi_Chr_5_4939737	Ca_desi_chr05	4939737	[A/G]	NA
Ca_Desi_Chr_5_4939742	Ca_desi_chr05	4939742	[T/G]	NA
Ca_Desi_Chr_5_4939750	Ca_desi_chr05	4939750	[A/G]	NA
Ca_Desi_Chr_5_4939751	Ca_desi_chr05	4939751	[A/G]	NA
Ca_Desi_Chr_5_4939754	Ca_desi_chr05	4939754	[T/C]	NA
Ca_Desi_Chr_5_4951632	Ca_desi_chr05	4951632	[T/C]	NA
Ca_Desi_Chr_5_5045259	Ca_desi_chr05	5045259	[A/T]	NA
Ca_Desi_Chr_5_5048631	Ca_desi_chr05	5048631	[A/C]	NA
Ca_Desi_Chr_5_5115215	Ca_desi_chr05	5115215	[A/G]	NA
Ca_Desi_Chr_5_5115250	Ca_desi_chr05	5115250	[A/G]	NA
Ca_Desi_Chr_5_5123280	Ca_desi_chr05	5123280	[C/G]	NA
Ca_Desi_Chr_5_5123286	Ca_desi_chr05	5123286	[A/G]	NA
Ca_Desi_Chr_5_5123295	Ca_desi_chr05	5123295	[T/G]	NA
Ca_Desi_Chr_5_5123297	Ca_desi_chr05	5123297	[T/G]	NA
Ca_Desi_Chr_5_5209349	Ca_desi_chr05	5209349	[T/C]	NA
Ca_Desi_Chr_5_5209361	Ca_desi_chr05	5209361	[T/C]	NA
Ca_Desi_Chr_5_5328665	Ca_desi_chr05	5328665	[A/C]	NA
Ca_Desi_Chr_5_5361327	Ca_desi_chr05	5361327	[A/G]	NA
Ca_Desi_Chr_5_5361612	Ca_desi_chr05	5361612	[C/T]	NA
Ca_Desi_Chr_5_5385395	Ca_desi_chr05	5385395	[A/G]	NA
Ca_Desi_Chr_5_5495882	Ca_desi_chr05	5495882	[T/A]	NA
Ca_Desi_Chr_5_5495910	Ca_desi_chr05	5495910	[T/G]	NA
Ca_Desi_Chr_5_5495913	Ca_desi_chr05	5495913	[C/G]	NA
Ca_Desi_Chr_5_5495917	Ca_desi_chr05	5495917	[A/G]	NA
Ca_Desi_Chr_5_5495919	Ca_desi_chr05	5495919	[A/C]	NA
Ca_Desi_Chr_5_5495922	Ca_desi_chr05	5495922	[C/A]	NA
Ca_Desi_Chr_5_5495924	Ca_desi_chr05	5495924	[G/C]	NA
Ca_Desi_Chr_5_5495933	Ca_desi_chr05	5495933	[A/G]	NA
Ca_Desi_Chr_5_5495935	Ca_desi_chr05	5495935	[A/C]	NA
Ca_Desi_Chr_5_5506488	Ca_desi_chr05	5506488	[T/G]	NA
Ca_Desi_Chr_5_5506511	Ca_desi_chr05	5506511	[T/C]	NA

SNP IDs	Chromosomes	Physical positions (bp)	SNPs	NCBI SNPdb accession IDs (NCBI_ss#)
Ca_Desi_Chr_5_5527369	Ca_desi_chr05	5527369	[T/C]	NA
Ca_Desi_Chr_5_5527416	Ca_desi_chr05	5527416	[A/C]	NA
Ca_Desi_Chr_5_5528798	Ca_desi_chr05	5528798	[A/G]	NA
Ca_Desi_Chr_5_5531395	Ca_desi_chr05	5531395	[A/G]	NA
Ca_Desi_Chr_5_5531396	Ca_desi_chr05	5531396	[A/C]	NA
Ca_Desi_Chr_5_5531400	Ca_desi_chr05	5531400	[A/T]	NA
Ca_Desi_Chr_5_5536484	Ca_desi_chr05	5536484	[T/G]	NA
Ca_Desi_Chr_5_5556030	Ca_desi_chr05	5556030	[A/C]	NA
Ca_Desi_Chr_5_5556096	Ca_desi_chr05	5556096	[A/C]	NA
Ca_Desi_Chr_5_5556111	Ca_desi_chr05	5556111	[T/C]	NA
Ca_Desi_Chr_5_5556241	Ca_desi_chr05	5556241	[T/G]	NA
Ca_Desi_Chr_5_5556280	Ca_desi_chr05	5556280	[G/T]	NA
Ca_Desi_Chr_5_5556288	Ca_desi_chr05	5556288	[A/C]	NA
Ca_Desi_Chr_5_5556354	Ca_desi_chr05	5556354	[T/G]	NA
Ca_Desi_Chr_5_5556427	Ca_desi_chr05	5556427	[T/G]	NA
Ca_Desi_Chr_5_5556477	Ca_desi_chr05	5556477	[A/G]	NA
Ca_Desi_Chr_5_5556480	Ca_desi_chr05	5556480	[A/C]	NA
Ca_Desi_Chr_5_5617812	Ca_desi_chr05	5617812	[T/G]	NA
Ca_Desi_Chr_5_5650591	Ca_desi_chr05	5650591	[A/T]	NA
Ca_Desi_Chr_5_5656111	Ca_desi_chr05	5656111	[T/C]	NA
Ca_Desi_Chr_5_5656112	Ca_desi_chr05	5656112	[T/C]	NA
Ca_Desi_Chr_5_5656126	Ca_desi_chr05	5656126	[T/C]	NA
Ca_Desi_Chr_5_5656153	Ca_desi_chr05	5656153	[T/C]	NA
Ca_Desi_Chr_5_5656155	Ca_desi_chr05	5656155	[T/C]	NA
Ca_Desi_Chr_5_5656162	Ca_desi_chr05	5656162	[T/C]	NA
Ca_Desi_Chr_5_5656171	Ca_desi_chr05	5656171	[A/G]	NA
Ca_Desi_Chr_5_5656173	Ca_desi_chr05	5656173	[T/C]	NA
Ca_Desi_Chr_5_5694190	Ca_desi_chr05	5694190	[T/C]	NA
Ca_Desi_Chr_5_5694203	Ca_desi_chr05	5694203	[A/T]	NA
Ca_Desi_Chr_5_5700017	Ca_desi_chr05	5700017	[A/C]	NA
Ca_Desi_Chr_5_5700019	Ca_desi_chr05	5700019	[C/T]	NA
Ca_Desi_Chr_5_5700025	Ca_desi_chr05	5700025	[A/T]	NA
Ca_Desi_Chr_5_5700063	Ca_desi_chr05	5700063	[A/C]	NA
Ca_Desi_Chr_5_5700318	Ca_desi_chr05	5700318	[T/C]	NA

SNP IDs	Chromosomes	Physical positions (bp)	SNPs	NCBI SNPdb accession IDs (NCBI_ss#)
Ca_Desi_Chr_5_5700319	Ca_desi_chr05	5700319	[A/C]	NA
Ca_Desi_Chr_5_5702668	Ca_desi_chr05	5702668	[T/G]	NA
Ca_Desi_Chr_5_5702689	Ca_desi_chr05	5702689	[A/G]	NA
Ca_Desi_Chr_5_5797279	Ca_desi_chr05	5797279	[T/C]	NA
Ca_Desi_Chr_5_5821472	Ca_desi_chr05	5821472	[T/G]	NA
Ca_Desi_Chr_5_5902984	Ca_desi_chr05	5902984	[T/G]	NA
Ca_Desi_Chr_5_5902987	Ca_desi_chr05	5902987	[T/G]	NA
Ca_Desi_Chr_5_5903030	Ca_desi_chr05	5903030	[A/T]	NA
Ca_Desi_Chr_5_5963186	Ca_desi_chr05	5963186	[T/G]	NA
Ca_Desi_Chr_5_5973516	Ca_desi_chr05	5973516	[A/C]	NA
Ca_Desi_Chr_5_5973528	Ca_desi_chr05	5973528	[T/C]	NA
Ca_Desi_Chr_5_5973590	Ca_desi_chr05	5973590	[T/C]	NA
Ca_Desi_Chr_5_5973596	Ca_desi_chr05	5973596	[A/G]	NA
Ca_Desi_Chr_5_5973605	Ca_desi_chr05	5973605	[A/C]	NA
Ca_Desi_Chr_5_5973639	Ca_desi_chr05	5973639	[T/C]	NA
Ca_Desi_Chr_5_5973649	Ca_desi_chr05	5973649	[A/G]	NA
Ca_Desi_Chr_5_5973687	Ca_desi_chr05	5973687	[T/G]	NA
Ca_Desi_Chr_5_5973689	Ca_desi_chr05	5973689	[G/A]	NA
Ca_Desi_Chr_5_5999500	Ca_desi_chr05	5999500	[T/G]	NA
Ca_Desi_Chr_5_6051569	Ca_desi_chr05	6051569	[T/C]	NA
Ca_Desi_Chr_5_6088875	Ca_desi_chr05	6088875	[T/G]	NA
Ca_Desi_Chr_5_6088877	Ca_desi_chr05	6088877	[T/C]	NA
Ca_Desi_Chr_5_6088881	Ca_desi_chr05	6088881	[T/G]	NA
Ca_Desi_Chr_5_6088883	Ca_desi_chr05	6088883	[A/G]	NA
Ca_Desi_Chr_5_6088884	Ca_desi_chr05	6088884	[T/A]	NA
Ca_Desi_Chr_5_6088894	Ca_desi_chr05	6088894	[G/C]	NA
Ca_Desi_Chr_5_6088904	Ca_desi_chr05	6088904	[A/G]	NA
Ca_Desi_Chr_5_6088910	Ca_desi_chr05	6088910	[T/G]	NA
Ca_Desi_Chr_5_6203237	Ca_desi_chr05	6203237	[A/T]	NA
Ca_Desi_Chr_5_6204610	Ca_desi_chr05	6204610	[T/G]	NA
Ca_Desi_Chr_5_6204613	Ca_desi_chr05	6204613	[A/G]	NA
Ca_Desi_Chr_5_6204615	Ca_desi_chr05	6204615	[A/G]	NA
Ca_Desi_Chr_5_6204622	Ca_desi_chr05	6204622	[T/G]	NA
Ca_Desi_Chr_5_6204628	Ca_desi_chr05	6204628	[T/G]	NA

SNP IDs	Chromosomes	Physical positions (bp)	SNPs	NCBI SNPdb accession IDs (NCBI_ss#)
Ca_Desi_Chr_5_6204641	Ca_desi_chr05	6204641	[T/G]	NA
Ca_Desi_Chr_5_6204661	Ca_desi_chr05	6204661	[A/G]	NA
Ca_Desi_Chr_5_6204666	Ca_desi_chr05	6204666	[A/C]	NA
Ca_Desi_Chr_5_6264933	Ca_desi_chr05	6264933	[A/T]	NA
Ca_Desi_Chr_5_6268419	Ca_desi_chr05	6268419	[T/A]	NA
Ca_Desi_Chr_5_6321306	Ca_desi_chr05	6321306	[T/G]	NA
Ca_Desi_Chr_5_6321363	Ca_desi_chr05	6321363	[A/G]	NA
Ca_Desi_Chr_5_6371514	Ca_desi_chr05	6371514	[A/G]	NA
Ca_Desi_Chr_5_6376546	Ca_desi_chr05	6376546	[T/A]	NA
Ca_Desi_Chr_5_6376637	Ca_desi_chr05	6376637	[A/C]	NA
Ca_Desi_Chr_5_6376650	Ca_desi_chr05	6376650	[A/G]	NA
Ca_Desi_Chr_5_6383542	Ca_desi_chr05	6383542	[A/G]	NA
Ca_Desi_Chr_5_6383562	Ca_desi_chr05	6383562	[T/A]	NA
Ca_Desi_Chr_5_6383641	Ca_desi_chr05	6383641	[T/C]	NA
Ca_Desi_Chr_5_6383696	Ca_desi_chr05	6383696	[A/C]	NA
Ca_Desi_Chr_5_6383714	Ca_desi_chr05	6383714	[T/C]	NA
Ca_Desi_Chr_5_6416789	Ca_desi_chr05	6416789	[A/G]	NA
Ca_Desi_Chr_5_6437385	Ca_desi_chr05	6437385	[A/G]	NA
Ca_Desi_Chr_5_6458466	Ca_desi_chr05	6458466	[A/G]	NA
Ca_Desi_Chr_5_6509675	Ca_desi_chr05	6509675	[T/C]	NA
Ca_Desi_Chr_5_6509718	Ca_desi_chr05	6509718	[T/G]	NA
Ca_Desi_Chr_5_6547174	Ca_desi_chr05	6547174	[A/C]	NA
Ca_Desi_Chr_5_6547198	Ca_desi_chr05	6547198	[T/G]	NA
Ca_Desi_Chr_5_6547205	Ca_desi_chr05	6547205	[G/T]	NA
Ca_Desi_Chr_5_6547207	Ca_desi_chr05	6547207	[T/G]	NA
Ca_Desi_Chr_5_6547210	Ca_desi_chr05	6547210	[A/G]	NA
Ca_Desi_Chr_5_6585073	Ca_desi_chr05	6585073	[T/C]	NA
Ca_Desi_Chr_5_6621480	Ca_desi_chr05	6621480	[T/C]	NA
Ca_Desi_Chr_5_6621511	Ca_desi_chr05	6621511	[A/G]	NA
Ca_Desi_Chr_5_6621611	Ca_desi_chr05	6621611	[A/T]	NA
Ca_Desi_Chr_5_6748718	Ca_desi_chr05	6748718	[A/G]	NA
Ca_Desi_Chr_5_6748747	Ca_desi_chr05	6748747	[T/C]	NA
Ca_Desi_Chr_5_6748769	Ca_desi_chr05	6748769	[A/G]	NA
Ca_Desi_Chr_5_6748785	Ca_desi_chr05	6748785	[T/C]	NA

SNP IDs	Chromosomes	Physical positions (bp)	SNPs	NCBI SNPdb accession IDs (NCBI_ss#)
Ca_Desi_Chr_5_6789739	Ca_desi_chr05	6789739	[T/C]	NA
Ca_Desi_Chr_5_6789780	Ca_desi_chr05	6789780	[T/C]	NA
Ca_Desi_Chr_5_6810969	Ca_desi_chr05	6810969	[T/G]	NA
Ca_Desi_Chr_5_6836746	Ca_desi_chr05	6836746	[T/G]	NA
Ca_Desi_Chr_5_6885427	Ca_desi_chr05	6885427	[A/T]	NA
Ca_Desi_Chr_5_6902602	Ca_desi_chr05	6902602	[A/C]	NA
Ca_Desi_Chr_5_7007017	Ca_desi_chr05	7007017	[A/G]	NA
Ca_Desi_Chr_5_7036529	Ca_desi_chr05	7036529	[T/C]	NA
Ca_Desi_Chr_5_7036550	Ca_desi_chr05	7036550	[T/G]	NA
Ca_Desi_Chr_5_7036603	Ca_desi_chr05	7036603	[A/T]	NA
Ca_Desi_Chr_5_7036618	Ca_desi_chr05	7036618	[A/T]	NA
Ca_Desi_Chr_5_7036634	Ca_desi_chr05	7036634	[A/C]	NA
Ca_Desi_Chr_5_7064545	Ca_desi_chr05	7064545	[A/C]	NA
Ca_Desi_Chr_5_7064569	Ca_desi_chr05	7064569	[T/C]	NA
Ca_Desi_Chr_5_7175827	Ca_desi_chr05	7175827	[T/G]	NA
Ca_Desi_Chr_5_7175834	Ca_desi_chr05	7175834	[A/G]	NA
Ca_Desi_Chr_5_7175836	Ca_desi_chr05	7175836	[A/G]	NA
Ca_Desi_Chr_5_7175838	Ca_desi_chr05	7175838	[A/G]	NA
Ca_Desi_Chr_5_7175855	Ca_desi_chr05	7175855	[A/C]	NA
Ca_Desi_Chr_5_7266466	Ca_desi_chr05	7266466	[A/G]	NA
Ca_Desi_Chr_5_7266471	Ca_desi_chr05	7266471	[T/C]	NA
Ca_Desi_Chr_5_7293797	Ca_desi_chr05	7293797	[A/C]	NA
Ca_Desi_Chr_5_7300680	Ca_desi_chr05	7300680	[A/T]	NA
Ca_Desi_Chr_5_7300688	Ca_desi_chr05	7300688	[T/C]	NA
Ca_Desi_Chr_5_7300692	Ca_desi_chr05	7300692	[T/C]	NA
Ca_Desi_Chr_5_7357370	Ca_desi_chr05	7357370	[T/G]	NA
Ca_Desi_Chr_5_7567944	Ca_desi_chr05	7567944	[A/G]	NA
Ca_Desi_Chr_5_7567969	Ca_desi_chr05	7567969	[A/T]	NA
Ca_Desi_Chr_5_7569499	Ca_desi_chr05	7569499	[T/G]	NA
Ca_Desi_Chr_5_7582801	Ca_desi_chr05	7582801	[T/G]	NA
Ca_Desi_Chr_5_7622349	Ca_desi_chr05	7622349	[A/C]	NA
Ca_Desi_Chr_5_7628971	Ca_desi_chr05	7628971	[A/T]	NA
Ca_Desi_Chr_5_7648219	Ca_desi_chr05	7648219	[A/C]	NA
Ca_Desi_Chr_5_7648240	Ca_desi_chr05	7648240	[A/C]	NA

SNP IDs	Chromosomes	Physical positions (bp)	SNPs	NCBI SNPdb accession IDs (NCBI_ss#)
Ca_Desi_Chr_5_7648331	Ca_desi_chr05	7648331	[A/C]	NA
Ca_Desi_Chr_5_7648411	Ca_desi_chr05	7648411	[T/A]	NA
Ca_Desi_Chr_5_7648442	Ca_desi_chr05	7648442	[A/C]	NA
Ca_Desi_Chr_5_7648447	Ca_desi_chr05	7648447	[A/C]	NA
Ca_Desi_Chr_5_7681731	Ca_desi_chr05	7681731	[A/T]	NA
Ca_Desi_Chr_5_7699220	Ca_desi_chr05	7699220	[C/G]	NA
Ca_Desi_Chr_5_7699223	Ca_desi_chr05	7699223	[T/C]	NA
Ca_Desi_Chr_5_7699239	Ca_desi_chr05	7699239	[T/G]	NA
Ca_Desi_Chr_5_7699244	Ca_desi_chr05	7699244	[T/G]	NA
Ca_Desi_Chr_5_7699325	Ca_desi_chr05	7699325	[A/G]	NA
Ca_Desi_Chr_5_7781390	Ca_desi_chr05	7781390	[A/C]	NA
Ca_Desi_Chr_5_7791001	Ca_desi_chr05	7791001	[A/C]	NA
Ca_Desi_Chr_5_7803996	Ca_desi_chr05	7803996	[A/G]	NA
Ca_Desi_Chr_5_7804020	Ca_desi_chr05	7804020	[G/T]	NA
Ca_Desi_Chr_5_7804030	Ca_desi_chr05	7804030	[A/G]	NA
Ca_Desi_Chr_5_7806838	Ca_desi_chr05	7806838	[A/C]	NA
Ca_Desi_Chr_5_7827564	Ca_desi_chr05	7827564	[G/A]	NA
Ca_Desi_Chr_5_7827569	Ca_desi_chr05	7827569	[T/G]	NA
Ca_Desi_Chr_5_7827576	Ca_desi_chr05	7827576	[A/C]	NA
Ca_Desi_Chr_5_7827644	Ca_desi_chr05	7827644	[A/C]	NA
Ca_Desi_Chr_5_7827665	Ca_desi_chr05	7827665	[A/C]	NA
Ca_Desi_Chr_5_7827827	Ca_desi_chr05	7827827	[A/C]	NA
Ca_Desi_Chr_5_7835454	Ca_desi_chr05	7835454	[T/A]	NA
Ca_Desi_Chr_5_7835466	Ca_desi_chr05	7835466	[T/G]	NA
Ca_Desi_Chr_5_7863792	Ca_desi_chr05	7863792	[T/C]	NA
Ca_Desi_Chr_5_7863803	Ca_desi_chr05	7863803	[A/C]	NA
Ca_Desi_Chr_5_7863817	Ca_desi_chr05	7863817	[A/G]	NA
Ca_Desi_Chr_5_7910722	Ca_desi_chr05	7910722	[A/C]	NA
Ca_Desi_Chr_5_7918717	Ca_desi_chr05	7918717	[T/G]	NA
Ca_Desi_Chr_5_7932501	Ca_desi_chr05	7932501	[C/A]	NA
Ca_Desi_Chr_5_7932506	Ca_desi_chr05	7932506	[G/C]	NA
Ca_Desi_Chr_5_7932507	Ca_desi_chr05	7932507	[C/A]	NA
Ca_Desi_Chr_5_7932509	Ca_desi_chr05	7932509	[A/C]	NA
Ca_Desi_Chr_5_7932510	Ca_desi_chr05	7932510	[C/G]	NA

SNP IDs	Chromosomes	Physical positions (bp)	SNPs	NCBI SNPdb accession IDs (NCBI_ss#)
Ca_Desi_Chr_5_7932523	Ca_desi_chr05	7932523	[T/C]	NA
Ca_Desi_Chr_5_7932525	Ca_desi_chr05	7932525	[T/C]	NA
Ca_Desi_Chr_5_7932531	Ca_desi_chr05	7932531	[C/T]	NA
Ca_Desi_Chr_5_7932532	Ca_desi_chr05	7932532	[T/C]	NA
Ca_Desi_Chr_5_7948554	Ca_desi_chr05	7948554	[A/C]	NA
Ca_Desi_Chr_5_7953753	Ca_desi_chr05	7953753	[A/C]	NA
Ca_Desi_Chr_5_7953765	Ca_desi_chr05	7953765	[T/A]	NA
Ca_Desi_Chr_5_7953770	Ca_desi_chr05	7953770	[A/C]	NA
Ca_Desi_Chr_5_7962748	Ca_desi_chr05	7962748	[T/G]	NA
Ca_Desi_Chr_5_7995495	Ca_desi_chr05	7995495	[A/T]	NA
Ca_Desi_Chr_5_8004788	Ca_desi_chr05	8004788	[T/C]	NA
Ca_Desi_Chr_5_8050041	Ca_desi_chr05	8050041	[T/C]	NA
Ca_Desi_Chr_5_8050092	Ca_desi_chr05	8050092	[T/C]	NA
Ca_Desi_Chr_5_8050165	Ca_desi_chr05	8050165	[T/C]	NA
Ca_Desi_Chr_5_8050171	Ca_desi_chr05	8050171	[A/G]	NA
Ca_Desi_Chr_5_8069445	Ca_desi_chr05	8069445	[T/C]	NA
Ca_Desi_Chr_5_8131674	Ca_desi_chr05	8131674	[A/G]	NA
Ca_Desi_Chr_5_8151149	Ca_desi_chr05	8151149	[T/G]	NA
Ca_Desi_Chr_5_8153763	Ca_desi_chr05	8153763	[A/T]	NA
Ca_Desi_Chr_5_8168421	Ca_desi_chr05	8168421	[C/A]	NA
Ca_Desi_Chr_5_8256887	Ca_desi_chr05	8256887	[A/G]	NA
Ca_Desi_Chr_5_8256913	Ca_desi_chr05	8256913	[T/A]	NA
Ca_Desi_Chr_5_8256925	Ca_desi_chr05	8256925	[T/C]	NA
Ca_Desi_Chr_5_8256926	Ca_desi_chr05	8256926	[A/G]	NA
Ca_Desi_Chr_5_8266612	Ca_desi_chr05	8266612	[A/G]	NA
Ca_Desi_Chr_5_8268219	Ca_desi_chr05	8268219	[T/C]	NA
Ca_Desi_Chr_5_8268309	Ca_desi_chr05	8268309	[T/G]	NA
Ca_Desi_Chr_5_8275380	Ca_desi_chr05	8275380	[T/G]	NA
Ca_Desi_Chr_5_8389017	Ca_desi_chr05	8389017	[T/C]	NA
Ca_Desi_Chr_5_8389029	Ca_desi_chr05	8389029	[T/G]	NA
Ca_Desi_Chr_5_8454486	Ca_desi_chr05	8454486	[T/C]	NA
Ca_Desi_Chr_5_8454497	Ca_desi_chr05	8454497	[A/G]	NA
Ca_Desi_Chr_5_8454501	Ca_desi_chr05	8454501	[T/G]	NA
Ca_Desi_Chr_5_8454507	Ca_desi_chr05	8454507	[T/G]	NA

SNP IDs	Chromosomes	Physical positions (bp)	SNPs	NCBI SNPdb accession IDs (NCBI_ss#)
Ca_Desi_Chr_5_8454509	Ca_desi_chr05	8454509	[T/G]	NA
Ca_Desi_Chr_5_8454514	Ca_desi_chr05	8454514	[T/G]	NA
Ca_Desi_Chr_5_8454517	Ca_desi_chr05	8454517	[C/G]	NA
Ca_Desi_Chr_5_8454541	Ca_desi_chr05	8454541	[T/C]	NA
Ca_Desi_Chr_5_8488000	Ca_desi_chr05	8488000	[T/C]	NA
Ca_Desi_Chr_5_8510520	Ca_desi_chr05	8510520	[T/C]	NA
Ca_Desi_Chr_5_8511251	Ca_desi_chr05	8511251	[A/T]	NA
Ca_Desi_Chr_5_8511252	Ca_desi_chr05	8511252	[A/C]	NA
Ca_Desi_Chr_5_8511253	Ca_desi_chr05	8511253	[G/T]	NA
Ca_Desi_Chr_5_8511304	Ca_desi_chr05	8511304	[A/C]	NA
Ca_Desi_Chr_5_8511476	Ca_desi_chr05	8511476	[A/G]	NA
Ca_Desi_Chr_5_8511572	Ca_desi_chr05	8511572	[A/T]	NA
Ca_Desi_Chr_5_8515255	Ca_desi_chr05	8515255	[A/G]	NA
Ca_Desi_Chr_5_8515283	Ca_desi_chr05	8515283	[A/C]	NA
Ca_Desi_Chr_5_8515513	Ca_desi_chr05	8515513	[A/G]	NA
Ca_Desi_Chr_5_8515519	Ca_desi_chr05	8515519	[A/T]	NA
Ca_Desi_Chr_5_8534414	Ca_desi_chr05	8534414	[T/C]	NA
Ca_Desi_Chr_5_8534420	Ca_desi_chr05	8534420	[G/A]	NA
Ca_Desi_Chr_5_8534429	Ca_desi_chr05	8534429	[A/G]	NA
Ca_Desi_Chr_5_8534454	Ca_desi_chr05	8534454	[A/G]	NA
Ca_Desi_Chr_5_8545122	Ca_desi_chr05	8545122	[T/C]	NA
Ca_Desi_Chr_5_8545134	Ca_desi_chr05	8545134	[A/C]	NA
Ca_Desi_Chr_5_8545143	Ca_desi_chr05	8545143	[T/C]	NA
Ca_Desi_Chr_5_8545962	Ca_desi_chr05	8545962	[C/A]	NA
Ca_Desi_Chr_5_8545963	Ca_desi_chr05	8545963	[C/G]	NA
Ca_Desi_Chr_5_8545964	Ca_desi_chr05	8545964	[T/A]	NA
Ca_Desi_Chr_5_8545965	Ca_desi_chr05	8545965	[A/T]	NA
Ca_Desi_Chr_5_8564546	Ca_desi_chr05	8564546	[G/T]	NA
Ca_Desi_Chr_5_8600365	Ca_desi_chr05	8600365	[A/T]	NA
Ca_Desi_Chr_5_8600386	Ca_desi_chr05	8600386	[A/G]	NA
Ca_Desi_Chr_5_8600406	Ca_desi_chr05	8600406	[A/G]	NA
Ca_Desi_Chr_5_8600425	Ca_desi_chr05	8600425	[T/C]	NA
Ca_Desi_Chr_5_8627437	Ca_desi_chr05	8627437	[A/T]	NA
Ca_Desi_Chr_5_8681049	Ca_desi_chr05	8681049	[G/T]	NA

SNP IDs	Chromosomes	Physical positions (bp)	SNPs	NCBI SNPdb accession IDs (NCBI_ss#)
Ca_Desi_Chr_5_8728734	Ca_desi_chr05	8728734	[T/G]	NA
Ca_Desi_Chr_5_8738931	Ca_desi_chr05	8738931	[T/G]	NA
Ca_Desi_Chr_5_8764866	Ca_desi_chr05	8764866	[T/C]	NA
Ca_Desi_Chr_5_8765479	Ca_desi_chr05	8765479	[A/T]	NA
Ca_Desi_Chr_5_8765480	Ca_desi_chr05	8765480	[G/C]	NA
Ca_Desi_Chr_5_8765481	Ca_desi_chr05	8765481	[G/T]	NA
Ca_Desi_Chr_5_8804721	Ca_desi_chr05	8804721	[A/C]	NA
Ca_Desi_Chr_5_8813565	Ca_desi_chr05	8813565	[A/T]	NA
Ca_Desi_Chr_5_8813569	Ca_desi_chr05	8813569	[A/C]	NA
Ca_Desi_Chr_5_8813596	Ca_desi_chr05	8813596	[A/G]	NA
Ca_Desi_Chr_5_8813608	Ca_desi_chr05	8813608	[A/G]	NA
Ca_Desi_Chr_5_8839849	Ca_desi_chr05	8839849	[T/G]	NA
Ca_Desi_Chr_5_8839851	Ca_desi_chr05	8839851	[T/C]	NA
Ca_Desi_Chr_5_8839857	Ca_desi_chr05	8839857	[T/C]	NA
Ca_Desi_Chr_5_8842551	Ca_desi_chr05	8842551	[G/A]	NA
Ca_Desi_Chr_5_8851732	Ca_desi_chr05	8851732	[A/C]	NA
Ca_Desi_Chr_5_8868697	Ca_desi_chr05	8868697	[A/G]	NA
Ca_Desi_Chr_5_8868714	Ca_desi_chr05	8868714	[A/T]	NA
Ca_Desi_Chr_5_9033610	Ca_desi_chr05	9033610	[T/G]	NA
Ca_Desi_Chr_5_9051729	Ca_desi_chr05	9051729	[A/C]	NA
Ca_Desi_Chr_5_9065183	Ca_desi_chr05	9065183	[T/C]	NA
Ca_Desi_Chr_5_9073799	Ca_desi_chr05	9073799	[T/C]	NA
Ca_Desi_Chr_5_9073862	Ca_desi_chr05	9073862	[A/C]	NA
Ca_Desi_Chr_5_9073869	Ca_desi_chr05	9073869	[A/G]	NA
Ca_Desi_Chr_5_9130735	Ca_desi_chr05	9130735	[T/A]	NA
Ca_Desi_Chr_5_9130736	Ca_desi_chr05	9130736	[A/G]	NA
Ca_Desi_Chr_5_9130745	Ca_desi_chr05	9130745	[A/G]	NA
Ca_Desi_Chr_5_9146295	Ca_desi_chr05	9146295	[T/A]	NA
Ca_Desi_Chr_5_9149804	Ca_desi_chr05	9149804	[G/T]	NA
Ca_Desi_Chr_5_9149908	Ca_desi_chr05	9149908	[T/C]	NA
Ca_Desi_Chr_5_9149925	Ca_desi_chr05	9149925	[T/G]	NA
Ca_Desi_Chr_5_9149946	Ca_desi_chr05	9149946	[A/G]	NA
Ca_Desi_Chr_5_9159865	Ca_desi_chr05	9159865	[T/C]	NA
Ca_Desi_Chr_5_9242586	Ca_desi_chr05	9242586	[T/A]	NA

SNP IDs	Chromosomes	Physical positions (bp)	SNPs	NCBI SNPdb accession IDs (NCBI_ss#)
Ca_Desi_Chr_5_9242589	Ca_desi_chr05	9242589	[C/T]	NA
Ca_Desi_Chr_5_9242592	Ca_desi_chr05	9242592	[C/T]	NA
Ca_Desi_Chr_5_9252432	Ca_desi_chr05	9252432	[T/C]	NA
Ca_Desi_Chr_5_9252447	Ca_desi_chr05	9252447	[T/C]	NA
Ca_Desi_Chr_5_9252450	Ca_desi_chr05	9252450	[A/C]	NA
Ca_Desi_Chr_5_9252468	Ca_desi_chr05	9252468	[A/G]	NA
Ca_Desi_Chr_5_9252471	Ca_desi_chr05	9252471	[T/C]	NA
Ca_Desi_Chr_5_9262321	Ca_desi_chr05	9262321	[A/C]	NA
Ca_Desi_Chr_5_9276300	Ca_desi_chr05	9276300	[T/C]	NA
Ca_Desi_Chr_5_9306310	Ca_desi_chr05	9306310	[T/G]	NA
Ca_Desi_Chr_5_9327067	Ca_desi_chr05	9327067	[T/C]	NA
Ca_Desi_Chr_5_9334354	Ca_desi_chr05	9334354	[A/T]	NA
Ca_Desi_Chr_5_9334367	Ca_desi_chr05	9334367	[T/C]	NA
Ca_Desi_Chr_5_9334376	Ca_desi_chr05	9334376	[T/G]	NA
Ca_Desi_Chr_5_9334379	Ca_desi_chr05	9334379	[T/C]	NA
Ca_Desi_Chr_5_9334384	Ca_desi_chr05	9334384	[T/C]	NA
Ca_Desi_Chr_5_9334387	Ca_desi_chr05	9334387	[T/C]	NA
Ca_Desi_Chr_5_9334388	Ca_desi_chr05	9334388	[C/T]	NA
Ca_Desi_Chr_5_9334392	Ca_desi_chr05	9334392	[T/C]	NA
Ca_Desi_Chr_5_9334394	Ca_desi_chr05	9334394	[C/T]	NA
Ca_Desi_Chr_5_9334396	Ca_desi_chr05	9334396	[T/C]	NA
Ca_Desi_Chr_5_9334402	Ca_desi_chr05	9334402	[T/A]	NA
Ca_Desi_Chr_5_9404637	Ca_desi_chr05	9404637	[A/G]	NA
Ca_Desi_Chr_5_9404673	Ca_desi_chr05	9404673	[A/G]	NA
Ca_Desi_Chr_5_9414382	Ca_desi_chr05	9414382	[A/G]	NA
Ca_Desi_Chr_5_9414393	Ca_desi_chr05	9414393	[T/C]	NA
Ca_Desi_Chr_5_9414396	Ca_desi_chr05	9414396	[T/C]	NA
Ca_Desi_Chr_5_9414411	Ca_desi_chr05	9414411	[A/G]	NA
Ca_Desi_Chr_5_9414427	Ca_desi_chr05	9414427	[A/G]	NA
Ca_Desi_Chr_5_9443377	Ca_desi_chr05	9443377	[T/G]	NA
Ca_Desi_Chr_5_9443588	Ca_desi_chr05	9443588	[A/C]	NA
Ca_Desi_Chr_5_9444431	Ca_desi_chr05	9444431	[A/G]	NA
Ca_Desi_Chr_5_9558483	Ca_desi_chr05	9558483	[T/G]	NA
Ca_Desi_Chr_5_9558491	Ca_desi_chr05	9558491	[T/C]	NA

SNP IDs	Chromosomes	Physical positions (bp)	SNPs	NCBI SNPdb accession IDs (NCBI_ss#)
Ca_Desi_Chr_5_9558496	Ca_desi_chr05	9558496	[T/G]	NA
Ca_Desi_Chr_5_9558500	Ca_desi_chr05	9558500	[T/C]	NA
Ca_Desi_Chr_5_9564890	Ca_desi_chr05	9564890	[T/A]	NA
Ca_Desi_Chr_5_9637797	Ca_desi_chr05	9637797	[A/T]	NA
Ca_Desi_Chr_5_9637811	Ca_desi_chr05	9637811	[T/C]	NA
Ca_Desi_Chr_5_9637828	Ca_desi_chr05	9637828	[A/G]	NA
Ca_Desi_Chr_5_9666137	Ca_desi_chr05	9666137	[T/G]	NA
Ca_Desi_Chr_5_9666142	Ca_desi_chr05	9666142	[T/G]	NA
Ca_Desi_Chr_5_9724592	Ca_desi_chr05	9724592	[T/G]	NA
Ca_Desi_Chr_5_9772984	Ca_desi_chr05	9772984	[A/T]	NA
Ca_Desi_Chr_5_9783887	Ca_desi_chr05	9783887	[T/C]	NA
Ca_Desi_Chr_5_9783919	Ca_desi_chr05	9783919	[T/G]	NA
Ca_Desi_Chr_5_9806434	Ca_desi_chr05	9806434	[A/G]	NA
Ca_Desi_Chr_5_9806620	Ca_desi_chr05	9806620	[A/C]	NA
Ca_Desi_Chr_5_9806638	Ca_desi_chr05	9806638	[A/C]	NA
Ca_Desi_Chr_5_9872975	Ca_desi_chr05	9872975	[A/C]	NA
Ca_Desi_Chr_5_9881537	Ca_desi_chr05	9881537	[T/G]	NA
Ca_Desi_Chr_5_9885946	Ca_desi_chr05	9885946	[T/G]	NA
Ca_Desi_Chr_5_9885955	Ca_desi_chr05	9885955	[A/G]	NA
Ca_Desi_Chr_5_9885967	Ca_desi_chr05	9885967	[T/C]	NA
Ca_Desi_Chr_5_9885970	Ca_desi_chr05	9885970	[T/C]	NA
Ca_Desi_Chr_5_9895834	Ca_desi_chr05	9895834	[T/G]	NA
Ca_Desi_Chr_5_9945176	Ca_desi_chr05	9945176	[G/T]	NA
Ca_Desi_Chr_5_9946931	Ca_desi_chr05	9946931	[G/T]	NA
Ca_Desi_Chr_5_9946938	Ca_desi_chr05	9946938	[A/G]	NA
Ca_Desi_Chr_5_9947007	Ca_desi_chr05	9947007	[T/C]	NA
Ca_Desi_Chr_5_9947052	Ca_desi_chr05	9947052	[T/C]	NA
Ca_Desi_Chr_5_9962810	Ca_desi_chr05	9962810	[A/C]	NA
Ca_Desi_Chr_5_9972166	Ca_desi_chr05	9972166	[T/A]	NA
Ca_Desi_Chr_5_10077108	Ca_desi_chr05	10077108	[T/G]	NA
Ca_Desi_Chr_5_10077114	Ca_desi_chr05	10077114	[A/G]	NA
Ca_Desi_Chr_5_10105094	Ca_desi_chr05	10105094	[T/G]	NA
Ca_Desi_Chr_5_10105466	Ca_desi_chr05	10105466	[A/C]	NA
Ca_Desi_Chr_5_10109391	Ca_desi_chr05	10109391	[T/C]	NA

SNP IDs	Chromosomes	Physical positions (bp)	SNPs	NCBI SNPdb accession IDs (NCBI_ss#)
Ca_Desi_Chr_5_10109431	Ca_desi_chr05	10109431	[A/G]	NA
Ca_Desi_Chr_5_10109481	Ca_desi_chr05	10109481	[T/C]	NA
Ca_Desi_Chr_5_10109832	Ca_desi_chr05	10109832	[T/C]	NA
Ca_Desi_Chr_5_10109834	Ca_desi_chr05	10109834	[A/G]	NA
Ca_Desi_Chr_5_10109867	Ca_desi_chr05	10109867	[T/C]	NA
Ca_Desi_Chr_5_10109872	Ca_desi_chr05	10109872	[T/C]	NA
Ca_Desi_Chr_5_10111956	Ca_desi_chr05	10111956	[T/G]	NA
Ca_Desi_Chr_5_10111959	Ca_desi_chr05	10111959	[T/G]	NA
Ca_Desi_Chr_5_10595054	Ca_desi_chr05	10595054	[G/T]	NA
Ca_Desi_Chr_5_10598483	Ca_desi_chr05	10598483	[A/T]	NA
Ca_Desi_Chr_5_10598523	Ca_desi_chr05	10598523	[A/C]	NA
Ca_Desi_Chr_5_10610760	Ca_desi_chr05	10610760	[A/G]	NA
Ca_Desi_Chr_5_10625145	Ca_desi_chr05	10625145	[A/G]	NA
Ca_Desi_Chr_5_10625149	Ca_desi_chr05	10625149	[T/C]	NA
Ca_Desi_Chr_5_10625153	Ca_desi_chr05	10625153	[T/A]	NA
Ca_Desi_Chr_5_10625168	Ca_desi_chr05	10625168	[T/G]	NA
Ca_Desi_Chr_5_10625181	Ca_desi_chr05	10625181	[T/G]	NA
Ca_Desi_Chr_5_10625225	Ca_desi_chr05	10625225	[A/C]	NA
Ca_Desi_Chr_5_10625356	Ca_desi_chr05	10625356	[A/C]	NA
Ca_Desi_Chr_5_10743453	Ca_desi_chr05	10743453	[A/C]	NA
Ca_Desi_Chr_5_10744185	Ca_desi_chr05	10744185	[T/C]	NA
Ca_Desi_Chr_5_10744224	Ca_desi_chr05	10744224	[A/C]	NA
Ca_Desi_Chr_5_10744341	Ca_desi_chr05	10744341	[A/C]	NA
Ca_Desi_Chr_5_10763827	Ca_desi_chr05	10763827	[T/C]	NA
Ca_Desi_Chr_5_10763867	Ca_desi_chr05	10763867	[T/G]	NA
Ca_Desi_Chr_5_10763903	Ca_desi_chr05	10763903	[G/A]	NA
Ca_Desi_Chr_5_10793641	Ca_desi_chr05	10793641	[A/C]	NA
Ca_Desi_Chr_5_10793651	Ca_desi_chr05	10793651	[T/C]	NA
Ca_Desi_Chr_5_10836604	Ca_desi_chr05	10836604	[G/T]	NA
Ca_Desi_Chr_5_10836623	Ca_desi_chr05	10836623	[A/C]	NA
Ca_Desi_Chr_5_10836637	Ca_desi_chr05	10836637	[T/C]	NA
Ca_Desi_Chr_5_10852928	Ca_desi_chr05	10852928	[G/A]	NA
Ca_Desi_Chr_5_10852929	Ca_desi_chr05	10852929	[A/T]	NA
Ca_Desi_Chr_5_10852931	Ca_desi_chr05	10852931	[G/T]	NA

SNP IDs	Chromosomes	Physical positions (bp)	SNPs	NCBI SNPdb accession IDs (NCBI_ss#)
Ca_Desi_Chr_5_10865753	Ca_desi_chr05	10865753	[T/C]	NA
Ca_Desi_Chr_5_10865760	Ca_desi_chr05	10865760	[T/C]	NA
Ca_Desi_Chr_5_10888364	Ca_desi_chr05	10888364	[A/C]	NA
Ca_Desi_Chr_5_10914643	Ca_desi_chr05	10914643	[T/G]	NA
Ca_Desi_Chr_5_10960809	Ca_desi_chr05	10960809	[T/G]	NA
Ca_Desi_Chr_5_10960811	Ca_desi_chr05	10960811	[A/G]	NA
Ca_Desi_Chr_5_10960813	Ca_desi_chr05	10960813	[C/T]	NA
Ca_Desi_Chr_5_10973116	Ca_desi_chr05	10973116	[A/C]	NA
Ca_Desi_Chr_5_10973119	Ca_desi_chr05	10973119	[A/C]	NA
Ca_Desi_Chr_5_11028658	Ca_desi_chr05	11028658	[C/G]	NA
Ca_Desi_Chr_5_11028670	Ca_desi_chr05	11028670	[C/A]	NA
Ca_Desi_Chr_5_11028684	Ca_desi_chr05	11028684	[A/C]	NA
Ca_Desi_Chr_5_11029092	Ca_desi_chr05	11029092	[T/G]	NA
Ca_Desi_Chr_5_11029108	Ca_desi_chr05	11029108	[A/T]	NA
Ca_Desi_Chr_5_11038446	Ca_desi_chr05	11038446	[T/C]	NA
Ca_Desi_Chr_5_11038453	Ca_desi_chr05	11038453	[T/C]	NA
Ca_Desi_Chr_5_11063910	Ca_desi_chr05	11063910	[C/G]	NA
Ca_Desi_Chr_5_11143975	Ca_desi_chr05	11143975	[C/T]	NA
Ca_Desi_Chr_5_11160609	Ca_desi_chr05	11160609	[A/G]	NA
Ca_Desi_Chr_5_11160614	Ca_desi_chr05	11160614	[T/C]	NA
Ca_Desi_Chr_5_11160620	Ca_desi_chr05	11160620	[C/G]	NA
Ca_Desi_Chr_5_11160623	Ca_desi_chr05	11160623	[A/G]	NA
Ca_Desi_Chr_5_11160626	Ca_desi_chr05	11160626	[T/C]	NA
Ca_Desi_Chr_5_11160633	Ca_desi_chr05	11160633	[T/C]	NA
Ca_Desi_Chr_5_11160638	Ca_desi_chr05	11160638	[T/C]	NA
Ca_Desi_Chr_5_11160641	Ca_desi_chr05	11160641	[A/G]	NA
Ca_Desi_Chr_5_11160652	Ca_desi_chr05	11160652	[T/C]	NA
Ca_Desi_Chr_5_11160656	Ca_desi_chr05	11160656	[T/C]	NA
Ca_Desi_Chr_5_11160660	Ca_desi_chr05	11160660	[T/C]	NA
Ca_Desi_Chr_5_11160669	Ca_desi_chr05	11160669	[T/C]	NA
Ca_Desi_Chr_5_11205333	Ca_desi_chr05	11205333	[T/A]	NA
Ca_Desi_Chr_5_11214425	Ca_desi_chr05	11214425	[A/G]	NA
Ca_Desi_Chr_5_11214446	Ca_desi_chr05	11214446	[T/G]	NA
Ca_Desi_Chr_5_11214451	Ca_desi_chr05	11214451	[A/G]	NA

SNP IDs	Chromosomes	Physical positions (bp)	SNPs	NCBI SNPdb accession IDs (NCBI_ss#)
Ca_Desi_Chr_5_11214453	Ca_desi_chr05	11214453	[A/G]	NA
Ca_Desi_Chr_5_11214457	Ca_desi_chr05	11214457	[T/G]	NA
Ca_Desi_Chr_5_11392894	Ca_desi_chr05	11392894	[A/G]	NA
Ca_Desi_Chr_5_11400385	Ca_desi_chr05	11400385	[T/G]	NA
Ca_Desi_Chr_5_11406738	Ca_desi_chr05	11406738	[T/C]	NA
Ca_Desi_Chr_5_11406771	Ca_desi_chr05	11406771	[T/C]	NA
Ca_Desi_Chr_5_11406840	Ca_desi_chr05	11406840	[A/G]	NA
Ca_Desi_Chr_5_11407014	Ca_desi_chr05	11407014	[A/C]	NA
Ca_Desi_Chr_5_11437653	Ca_desi_chr05	11437653	[G/T]	NA
Ca_Desi_Chr_5_11437775	Ca_desi_chr05	11437775	[A/T]	NA
Ca_Desi_Chr_5_11437777	Ca_desi_chr05	11437777	[C/G]	NA
Ca_Desi_Chr_5_11514037	Ca_desi_chr05	11514037	[A/T]	NA
Ca_Desi_Chr_5_11514041	Ca_desi_chr05	11514041	[A/C]	NA
Ca_Desi_Chr_5_11514043	Ca_desi_chr05	11514043	[A/C]	NA
Ca_Desi_Chr_5_11514053	Ca_desi_chr05	11514053	[A/G]	NA
Ca_Desi_Chr_5_11514057	Ca_desi_chr05	11514057	[T/C]	NA
Ca_Desi_Chr_5_11514074	Ca_desi_chr05	11514074	[T/C]	NA
Ca_Desi_Chr_5_11514078	Ca_desi_chr05	11514078	[T/C]	NA
Ca_Desi_Chr_5_11589918	Ca_desi_chr05	11589918	[T/G]	NA
Ca_Desi_Chr_5_11604545	Ca_desi_chr05	11604545	[T/G]	NA
Ca_Desi_Chr_5_11604585	Ca_desi_chr05	11604585	[G/T]	NA
Ca_Desi_Chr_5_11604659	Ca_desi_chr05	11604659	[T/C]	NA
Ca_Desi_Chr_5_11604679	Ca_desi_chr05	11604679	[T/G]	NA
Ca_Desi_Chr_5_11604681	Ca_desi_chr05	11604681	[T/G]	NA
Ca_Desi_Chr_5_11604691	Ca_desi_chr05	11604691	[A/G]	NA
Ca_Desi_Chr_5_11604698	Ca_desi_chr05	11604698	[T/C]	NA
Ca_Desi_Chr_5_11609169	Ca_desi_chr05	11609169	[G/T]	NA
Ca_Desi_Chr_5_11609170	Ca_desi_chr05	11609170	[A/T]	NA
Ca_Desi_Chr_5_11616547	Ca_desi_chr05	11616547	[G/C]	NA
Ca_Desi_Chr_5_11656990	Ca_desi_chr05	11656990	[T/G]	NA
Ca_Desi_Chr_5_11657030	Ca_desi_chr05	11657030	[T/C]	NA
Ca_Desi_Chr_5_11657035	Ca_desi_chr05	11657035	[T/C]	NA
Ca_Desi_Chr_5_11685392	Ca_desi_chr05	11685392	[T/C]	NA
Ca_Desi_Chr_5_11685412	Ca_desi_chr05	11685412	[T/A]	NA

SNP IDs	Chromosomes	Physical positions (bp)	SNPs	NCBI SNPdb accession IDs (NCBI_ss#)
Ca_Desi_Chr_5_11685416	Ca_desi_chr05	11685416	[T/C]	NA
Ca_Desi_Chr_5_11685421	Ca_desi_chr05	11685421	[A/G]	NA
Ca_Desi_Chr_5_11733909	Ca_desi_chr05	11733909	[A/C]	NA
Ca_Desi_Chr_5_11752359	Ca_desi_chr05	11752359	[T/G]	NA
Ca_Desi_Chr_5_11753756	Ca_desi_chr05	11753756	[T/A]	NA
Ca_Desi_Chr_5_11793251	Ca_desi_chr05	11793251	[C/A]	NA
Ca_Desi_Chr_5_11793252	Ca_desi_chr05	11793252	[T/G]	NA
Ca_Desi_Chr_5_11793291	Ca_desi_chr05	11793291	[A/C]	NA
Ca_Desi_Chr_5_11793302	Ca_desi_chr05	11793302	[T/C]	NA
Ca_Desi_Chr_5_11795551	Ca_desi_chr05	11795551	[A/C]	NA
Ca_Desi_Chr_5_11868634	Ca_desi_chr05	11868634	[A/C]	NA
Ca_Desi_Chr_5_11868638	Ca_desi_chr05	11868638	[A/T]	NA
Ca_Desi_Chr_5_11868639	Ca_desi_chr05	11868639	[G/C]	NA
Ca_Desi_Chr_5_11870348	Ca_desi_chr05	11870348	[A/C]	NA
Ca_Desi_Chr_5_11973730	Ca_desi_chr05	11973730	[T/G]	NA
Ca_Desi_Chr_5_11973786	Ca_desi_chr05	11973786	[T/C]	NA
Ca_Desi_Chr_5_11987499	Ca_desi_chr05	11987499	[A/C]	NA
Ca_Desi_Chr_5_11989946	Ca_desi_chr05	11989946	[T/C]	NA
Ca_Desi_Chr_5_11990014	Ca_desi_chr05	11990014	[A/G]	NA
Ca_Desi_Chr_5_12010099	Ca_desi_chr05	12010099	[A/G]	NA
Ca_Desi_Chr_5_12010101	Ca_desi_chr05	12010101	[T/G]	NA
Ca_Desi_Chr_5_12029705	Ca_desi_chr05	12029705	[T/G]	NA
Ca_Desi_Chr_5_12036244	Ca_desi_chr05	12036244	[T/G]	NA
Ca_Desi_Chr_5_12036278	Ca_desi_chr05	12036278	[A/C]	NA
Ca_Desi_Chr_5_12037326	Ca_desi_chr05	12037326	[T/G]	NA
Ca_Desi_Chr_5_12037340	Ca_desi_chr05	12037340	[T/C]	NA
Ca_Desi_Chr_5_12079134	Ca_desi_chr05	12079134	[T/G]	NA
Ca_Desi_Chr_5_12107002	Ca_desi_chr05	12107002	[A/G]	NA
Ca_Desi_Chr_5_12112980	Ca_desi_chr05	12112980	[C/T]	NA
Ca_Desi_Chr_5_12148739	Ca_desi_chr05	12148739	[A/C]	NA
Ca_Desi_Chr_5_12148774	Ca_desi_chr05	12148774	[T/A]	NA
Ca_Desi_Chr_5_12225476	Ca_desi_chr05	12225476	[A/C]	NA
Ca_Desi_Chr_5_12361818	Ca_desi_chr05	12361818	[A/G]	NA
Ca_Desi_Chr_5_12361972	Ca_desi_chr05	12361972	[G/T]	NA

SNP IDs	Chromosomes	Physical positions (bp)	SNPs	NCBI SNPdb accession IDs (NCBI_ss#)
Ca_Desi_Chr_5_12393527	Ca_desi_chr05	12393527	[A/G]	NA
Ca_Desi_Chr_5_12393547	Ca_desi_chr05	12393547	[T/C]	NA
Ca_Desi_Chr_5_12393549	Ca_desi_chr05	12393549	[A/G]	NA
Ca_Desi_Chr_5_12393553	Ca_desi_chr05	12393553	[A/G]	NA
Ca_Desi_Chr_5_12393557	Ca_desi_chr05	12393557	[A/G]	NA
Ca_Desi_Chr_5_12393562	Ca_desi_chr05	12393562	[T/G]	NA
Ca_Desi_Chr_5_12393571	Ca_desi_chr05	12393571	[T/C]	NA
Ca_Desi_Chr_5_12393573	Ca_desi_chr05	12393573	[C/G]	NA
Ca_Desi_Chr_5_12393584	Ca_desi_chr05	12393584	[T/C]	NA
Ca_Desi_Chr_5_12393609	Ca_desi_chr05	12393609	[T/G]	NA
Ca_Desi_Chr_5_12393792	Ca_desi_chr05	12393792	[T/C]	NA
Ca_Desi_Chr_5_12393793	Ca_desi_chr05	12393793	[T/G]	NA
Ca_Desi_Chr_5_12393808	Ca_desi_chr05	12393808	[A/G]	NA
Ca_Desi_Chr_5_12396987	Ca_desi_chr05	12396987	[G/A]	NA
Ca_Desi_Chr_5_12404859	Ca_desi_chr05	12404859	[A/T]	NA
Ca_Desi_Chr_5_12404871	Ca_desi_chr05	12404871	[A/G]	NA
Ca_Desi_Chr_5_12404876	Ca_desi_chr05	12404876	[C/A]	NA
Ca_Desi_Chr_5_12404880	Ca_desi_chr05	12404880	[A/G]	NA
Ca_Desi_Chr_5_12404890	Ca_desi_chr05	12404890	[T/C]	NA
Ca_Desi_Chr_5_12404898	Ca_desi_chr05	12404898	[A/C]	NA
Ca_Desi_Chr_5_12404902	Ca_desi_chr05	12404902	[A/G]	NA
Ca_Desi_Chr_5_12404904	Ca_desi_chr05	12404904	[A/G]	NA
Ca_Desi_Chr_5_12406448	Ca_desi_chr05	12406448	[A/C]	NA
Ca_Desi_Chr_5_12434400	Ca_desi_chr05	12434400	[T/G]	NA
Ca_Desi_Chr_5_12461772	Ca_desi_chr05	12461772	[C/A]	NA
Ca_Desi_Chr_5_12461782	Ca_desi_chr05	12461782	[A/C]	NA
Ca_Desi_Chr_5_12461786	Ca_desi_chr05	12461786	[C/A]	NA
Ca_Desi_Chr_5_12467135	Ca_desi_chr05	12467135	[T/C]	NA
Ca_Desi_Chr_5_12467236	Ca_desi_chr05	12467236	[G/T]	NA
Ca_Desi_Chr_5_12467266	Ca_desi_chr05	12467266	[A/G]	NA
Ca_Desi_Chr_5_12467268	Ca_desi_chr05	12467268	[C/T]	NA
Ca_Desi_Chr_5_12467275	Ca_desi_chr05	12467275	[T/G]	NA
Ca_Desi_Chr_5_12467279	Ca_desi_chr05	12467279	[T/C]	NA
Ca_Desi_Chr_5_12467282	Ca_desi_chr05	12467282	[C/A]	NA

SNP IDs	Chromosomes	Physical positions (bp)	SNPs	NCBI SNPdb accession IDs (NCBI_ss#)
Ca_Desi_Chr_5_12467283	Ca_desi_chr05	12467283	[A/G]	NA
Ca_Desi_Chr_5_12467284	Ca_desi_chr05	12467284	[A/G]	NA
Ca_Desi_Chr_5_12467285	Ca_desi_chr05	12467285	[A/G]	NA
Ca_Desi_Chr_5_12485019	Ca_desi_chr05	12485019	[A/C]	NA
Ca_Desi_Chr_5_12485024	Ca_desi_chr05	12485024	[A/G]	NA
Ca_Desi_Chr_5_12485026	Ca_desi_chr05	12485026	[T/A]	NA
Ca_Desi_Chr_5_12485034	Ca_desi_chr05	12485034	[T/C]	NA
Ca_Desi_Chr_5_12485036	Ca_desi_chr05	12485036	[A/G]	NA
Ca_Desi_Chr_5_12485049	Ca_desi_chr05	12485049	[T/C]	NA
Ca_Desi_Chr_5_12487417	Ca_desi_chr05	12487417	[A/T]	NA
Ca_Desi_Chr_5_12490013	Ca_desi_chr05	12490013	[C/A]	NA
Ca_Desi_Chr_5_12539353	Ca_desi_chr05	12539353	[C/A]	NA
Ca_Desi_Chr_5_12539354	Ca_desi_chr05	12539354	[C/G]	NA
Ca_Desi_Chr_5_12600166	Ca_desi_chr05	12600166	[T/G]	NA
Ca_Desi_Chr_5_12605174	Ca_desi_chr05	12605174	[T/G]	NA
Ca_Desi_Chr_5_12606408	Ca_desi_chr05	12606408	[A/G]	NA
Ca_Desi_Chr_5_12636617	Ca_desi_chr05	12636617	[A/C]	NA
Ca_Desi_Chr_5_12636623	Ca_desi_chr05	12636623	[A/C]	NA
Ca_Desi_Chr_5_12701963	Ca_desi_chr05	12701963	[A/C]	NA
Ca_Desi_Chr_5_12759422	Ca_desi_chr05	12759422	[T/G]	NA
Ca_Desi_Chr_5_12759567	Ca_desi_chr05	12759567	[T/G]	NA
Ca_Desi_Chr_5_12763953	Ca_desi_chr05	12763953	[T/C]	NA
Ca_Desi_Chr_5_12955174	Ca_desi_chr05	12955174	[T/C]	NA
Ca_Desi_Chr_5_12955233	Ca_desi_chr05	12955233	[A/C]	NA
Ca_Desi_Chr_5_12955235	Ca_desi_chr05	12955235	[C/A]	NA
Ca_Desi_Chr_5_12955239	Ca_desi_chr05	12955239	[A/G]	NA
Ca_Desi_Chr_5_12955246	Ca_desi_chr05	12955246	[A/C]	NA
Ca_Desi_Chr_5_12955254	Ca_desi_chr05	12955254	[A/C]	NA
Ca_Desi_Chr_5_12979164	Ca_desi_chr05	12979164	[T/C]	NA
Ca_Desi_Chr_5_12979391	Ca_desi_chr05	12979391	[T/C]	NA
Ca_Desi_Chr_5_12979407	Ca_desi_chr05	12979407	[T/C]	NA
Ca_Desi_Chr_5_12992831	Ca_desi_chr05	12992831	[C/G]	NA
Ca_Desi_Chr_5_13004532	Ca_desi_chr05	13004532	[C/T]	NA
Ca_Desi_Chr_5_13004548	Ca_desi_chr05	13004548	[A/C]	NA

SNP IDs	Chromosomes	Physical positions (bp)	SNPs	NCBI SNPdb accession IDs (NCBI_ss#)
Ca_Desi_Chr_5_13004551	Ca_desi_chr05	13004551	[A/G]	NA
Ca_Desi_Chr_5_13004552	Ca_desi_chr05	13004552	[A/T]	NA
Ca_Desi_Chr_5_13152770	Ca_desi_chr05	13152770	[A/C]	NA
Ca_Desi_Chr_5_13152811	Ca_desi_chr05	13152811	[T/C]	NA
Ca_Desi_Chr_5_13170683	Ca_desi_chr05	13170683	[T/C]	NA
Ca_Desi_Chr_5_13170686	Ca_desi_chr05	13170686	[A/G]	NA
Ca_Desi_Chr_5_13170698	Ca_desi_chr05	13170698	[T/G]	NA
Ca_Desi_Chr_5_13170708	Ca_desi_chr05	13170708	[T/A]	NA
Ca_Desi_Chr_5_13173580	Ca_desi_chr05	13173580	[A/G]	NA
Ca_Desi_Chr_5_13173613	Ca_desi_chr05	13173613	[T/C]	NA
Ca_Desi_Chr_5_13190974	Ca_desi_chr05	13190974	[G/A]	NA
Ca_Desi_Chr_5_13190975	Ca_desi_chr05	13190975	[G/C]	NA
Ca_Desi_Chr_5_13190987	Ca_desi_chr05	13190987	[C/T]	NA
Ca_Desi_Chr_5_13191923	Ca_desi_chr05	13191923	[G/A]	NA
Ca_Desi_Chr_5_13191966	Ca_desi_chr05	13191966	[C/T]	NA
Ca_Desi_Chr_5_13815426	Ca_desi_chr05	13815426	[T/G]	NA
Ca_Desi_Chr_5_13838359	Ca_desi_chr05	13838359	[T/G]	NA
Ca_Desi_Chr_5_13850044	Ca_desi_chr05	13850044	[T/G]	NA
Ca_Desi_Chr_5_13882651	Ca_desi_chr05	13882651	[G/C]	NA
Ca_Desi_Chr_5_13882652	Ca_desi_chr05	13882652	[G/T]	NA
Ca_Desi_Chr_5_13882678	Ca_desi_chr05	13882678	[A/C]	NA
Ca_Desi_Chr_5_13882695	Ca_desi_chr05	13882695	[G/A]	NA
Ca_Desi_Chr_5_13929239	Ca_desi_chr05	13929239	[A/G]	NA
Ca_Desi_Chr_5_14015931	Ca_desi_chr05	14015931	[T/C]	NA
Ca_Desi_Chr_5_14086022	Ca_desi_chr05	14086022	[T/A]	NA
Ca_Desi_Chr_5_14154057	Ca_desi_chr05	14154057	[T/C]	NA
Ca_Desi_Chr_5_14154083	Ca_desi_chr05	14154083	[T/G]	NA
Ca_Desi_Chr_5_14154088	Ca_desi_chr05	14154088	[A/G]	NA
Ca_Desi_Chr_5_14154115	Ca_desi_chr05	14154115	[A/G]	NA
Ca_Desi_Chr_5_14242265	Ca_desi_chr05	14242265	[G/A]	NA
Ca_Desi_Chr_5_14242294	Ca_desi_chr05	14242294	[T/C]	NA
Ca_Desi_Chr_5_14242311	Ca_desi_chr05	14242311	[G/A]	NA
Ca_Desi_Chr_5_14317110	Ca_desi_chr05	14317110	[T/G]	NA
Ca_Desi_Chr_5_14341079	Ca_desi_chr05	14341079	[A/C]	NA

SNP IDs	Chromosomes	Physical positions (bp)	SNPs	NCBI SNPdb accession IDs (NCBI_ss#)
Ca_Desi_Chr_5_14341106	Ca_desi_chr05	14341106	[A/G]	NA
Ca_Desi_Chr_5_14343497	Ca_desi_chr05	14343497	[G/T]	NA
Ca_Desi_Chr_5_14343498	Ca_desi_chr05	14343498	[G/C]	NA
Ca_Desi_Chr_5_14343499	Ca_desi_chr05	14343499	[T/C]	NA
Ca_Desi_Chr_5_14343506	Ca_desi_chr05	14343506	[A/G]	NA
Ca_Desi_Chr_5_14343509	Ca_desi_chr05	14343509	[A/G]	NA
Ca_Desi_Chr_5_14343512	Ca_desi_chr05	14343512	[T/C]	NA
Ca_Desi_Chr_5_14343514	Ca_desi_chr05	14343514	[A/C]	NA
Ca_Desi_Chr_5_14343527	Ca_desi_chr05	14343527	[T/C]	NA
Ca_Desi_Chr_5_14343530	Ca_desi_chr05	14343530	[A/G]	NA
Ca_Desi_Chr_5_14343536	Ca_desi_chr05	14343536	[T/C]	NA
Ca_Desi_Chr_5_14343542	Ca_desi_chr05	14343542	[T/C]	NA
Ca_Desi_Chr_5_14343547	Ca_desi_chr05	14343547	[A/G]	NA
Ca_Desi_Chr_5_14343551	Ca_desi_chr05	14343551	[A/G]	NA
Ca_Desi_Chr_5_14344076	Ca_desi_chr05	14344076	[T/C]	NA
Ca_Desi_Chr_5_14344085	Ca_desi_chr05	14344085	[A/G]	NA
Ca_Desi_Chr_5_14344088	Ca_desi_chr05	14344088	[T/C]	NA
Ca_Desi_Chr_5_14344091	Ca_desi_chr05	14344091	[A/T]	NA
Ca_Desi_Chr_5_14344094	Ca_desi_chr05	14344094	[T/G]	NA
Ca_Desi_Chr_5_14344101	Ca_desi_chr05	14344101	[A/G]	NA
Ca_Desi_Chr_5_14344111	Ca_desi_chr05	14344111	[A/G]	NA
Ca_Desi_Chr_5_14344115	Ca_desi_chr05	14344115	[A/G]	NA
Ca_Desi_Chr_5_14344117	Ca_desi_chr05	14344117	[T/C]	NA
Ca_Desi_Chr_5_14344125	Ca_desi_chr05	14344125	[T/C]	NA
Ca_Desi_Chr_5_14431431	Ca_desi_chr05	14431431	[A/C]	NA
Ca_Desi_Chr_5_14622404	Ca_desi_chr05	14622404	[A/C]	NA
Ca_Desi_Chr_5_14622415	Ca_desi_chr05	14622415	[T/C]	NA
Ca_Desi_Chr_5_14622433	Ca_desi_chr05	14622433	[T/C]	NA
Ca_Desi_Chr_5_14869781	Ca_desi_chr05	14869781	[T/C]	NA
Ca_Desi_Chr_5_15196890	Ca_desi_chr05	15196890	[A/G]	NA
Ca_Desi_Chr_5_15197106	Ca_desi_chr05	15197106	[C/G]	NA
Ca_Desi_Chr_5_15239781	Ca_desi_chr05	15239781	[G/C]	NA
Ca_Desi_Chr_5_15239787	Ca_desi_chr05	15239787	[T/C]	NA
Ca_Desi_Chr_5_15239788	Ca_desi_chr05	15239788	[T/C]	NA

SNP IDs	Chromosomes	Physical positions (bp)	SNPs	NCBI SNPdb accession IDs (NCBI_ss#)
Ca_Desi_Chr_5_15239790	Ca_desi_chr05	15239790	[G/C]	NA
Ca_Desi_Chr_5_15239793	Ca_desi_chr05	15239793	[A/C]	NA
Ca_Desi_Chr_5_15284586	Ca_desi_chr05	15284586	[A/T]	NA
Ca_Desi_Chr_5_15284589	Ca_desi_chr05	15284589	[A/G]	NA
Ca_Desi_Chr_5_15284591	Ca_desi_chr05	15284591	[C/T]	NA
Ca_Desi_Chr_5_15284592	Ca_desi_chr05	15284592	[G/T]	NA
Ca_Desi_Chr_5_15284594	Ca_desi_chr05	15284594	[T/C]	NA
Ca_Desi_Chr_5_15284599	Ca_desi_chr05	15284599	[A/G]	NA
Ca_Desi_Chr_5_15284602	Ca_desi_chr05	15284602	[A/G]	NA
Ca_Desi_Chr_5_15284605	Ca_desi_chr05	15284605	[A/G]	NA
Ca_Desi_Chr_5_15284608	Ca_desi_chr05	15284608	[G/C]	NA
Ca_Desi_Chr_5_15284611	Ca_desi_chr05	15284611	[A/G]	NA
Ca_Desi_Chr_5_15284613	Ca_desi_chr05	15284613	[A/C]	NA
Ca_Desi_Chr_5_15284617	Ca_desi_chr05	15284617	[T/C]	NA
Ca_Desi_Chr_5_15284621	Ca_desi_chr05	15284621	[T/C]	NA
Ca_Desi_Chr_5_15284624	Ca_desi_chr05	15284624	[T/C]	NA
Ca_Desi_Chr_5_15284625	Ca_desi_chr05	15284625	[G/T]	NA
Ca_Desi_Chr_5_15284637	Ca_desi_chr05	15284637	[T/A]	NA
Ca_Desi_Chr_5_15301225	Ca_desi_chr05	15301225	[G/T]	NA
Ca_Desi_Chr_5_15369158	Ca_desi_chr05	15369158	[A/C]	NA
Ca_Desi_Chr_5_15476922	Ca_desi_chr05	15476922	[T/G]	NA
Ca_Desi_Chr_5_15507393	Ca_desi_chr05	15507393	[T/G]	NA
Ca_Desi_Chr_5_15508663	Ca_desi_chr05	15508663	[A/T]	NA
Ca_Desi_Chr_5_15608973	Ca_desi_chr05	15608973	[T/A]	NA
Ca_Desi_Chr_5_15608977	Ca_desi_chr05	15608977	[A/G]	NA
Ca_Desi_Chr_5_15608985	Ca_desi_chr05	15608985	[A/G]	NA
Ca_Desi_Chr_5_15609025	Ca_desi_chr05	15609025	[T/G]	NA
Ca_Desi_Chr_5_15609054	Ca_desi_chr05	15609054	[A/G]	NA
Ca_Desi_Chr_5_15609057	Ca_desi_chr05	15609057	[T/G]	NA
Ca_Desi_Chr_5_15609066	Ca_desi_chr05	15609066	[T/G]	NA
Ca_Desi_Chr_5_15609083	Ca_desi_chr05	15609083	[A/C]	NA
Ca_Desi_Chr_5_15952944	Ca_desi_chr05	15952944	[A/C]	NA
Ca_Desi_Chr_5_16078290	Ca_desi_chr05	16078290	[T/A]	NA
Ca_Desi_Chr_5_16078300	Ca_desi_chr05	16078300	[T/G]	NA

SNP IDs	Chromosomes	Physical positions (bp)	SNPs	NCBI SNPdb accession IDs (NCBI_ss#)
Ca_Desi_Chr_5_16078351	Ca_desi_chr05	16078351	[G/A]	NA
Ca_Desi_Chr_5_16078357	Ca_desi_chr05	16078357	[A/T]	NA
Ca_Desi_Chr_5_16081057	Ca_desi_chr05	16081057	[A/C]	NA
Ca_Desi_Chr_5_16081058	Ca_desi_chr05	16081058	[T/G]	NA
Ca_Desi_Chr_5_16081061	Ca_desi_chr05	16081061	[T/C]	NA
Ca_Desi_Chr_5_16081062	Ca_desi_chr05	16081062	[G/T]	NA
Ca_Desi_Chr_5_16117325	Ca_desi_chr05	16117325	[G/T]	NA
Ca_Desi_Chr_5_16137743	Ca_desi_chr05	16137743	[T/C]	NA
Ca_Desi_Chr_5_16137753	Ca_desi_chr05	16137753	[G/A]	NA
Ca_Desi_Chr_5_16170834	Ca_desi_chr05	16170834	[T/G]	NA
Ca_Desi_Chr_5_16170915	Ca_desi_chr05	16170915	[G/T]	NA
Ca_Desi_Chr_6_641359	Ca_desi_chr06	641359	[G/C]	NA
Ca_Desi_Chr_6_641361	Ca_desi_chr06	641361	[C/A]	NA
Ca_Desi_Chr_6_641362	Ca_desi_chr06	641362	[A/T]	NA
Ca_Desi_Chr_6_641363	Ca_desi_chr06	641363	[T/C]	NA
Ca_Desi_Chr_6_641364	Ca_desi_chr06	641364	[G/T]	NA
Ca_Desi_Chr_6_646756	Ca_desi_chr06	646756	[T/C]	NA
Ca_Desi_Chr_6_646757	Ca_desi_chr06	646757	[G/T]	NA
Ca_Desi_Chr_6_711791	Ca_desi_chr06	711791	[A/G]	NA
Ca_Desi_Chr_6_760524	Ca_desi_chr06	760524	[T/G]	NA
Ca_Desi_Chr_6_767007	Ca_desi_chr06	767007	[A/C]	NA
Ca_Desi_Chr_6_767012	Ca_desi_chr06	767012	[T/C]	NA
Ca_Desi_Chr_6_767025	Ca_desi_chr06	767025	[A/C]	NA
Ca_Desi_Chr_6_767035	Ca_desi_chr06	767035	[A/C]	NA
Ca_Desi_Chr_6_785472	Ca_desi_chr06	785472	[A/C]	NA
Ca_Desi_Chr_6_785478	Ca_desi_chr06	785478	[A/C]	NA
Ca_Desi_Chr_6_785489	Ca_desi_chr06	785489	[T/C]	NA
Ca_Desi_Chr_6_785496	Ca_desi_chr06	785496	[A/G]	NA
Ca_Desi_Chr_6_785500	Ca_desi_chr06	785500	[T/G]	NA
Ca_Desi_Chr_6_785531	Ca_desi_chr06	785531	[A/C]	NA
Ca_Desi_Chr_6_809545	Ca_desi_chr06	809545	[A/C]	NA
Ca_Desi_Chr_6_816746	Ca_desi_chr06	816746	[T/G]	NA
Ca_Desi_Chr_6_816823	Ca_desi_chr06	816823	[A/C]	NA
Ca_Desi_Chr_6_834820	Ca_desi_chr06	834820	[A/C]	NA

SNP IDs	Chromosomes	Physical positions (bp)	SNPs	NCBI SNPdb accession IDs (NCBI_ss#)
Ca_Desi_Chr_6_834839	Ca_desi_chr06	834839	[T/G]	NA
Ca_Desi_Chr_6_837890	Ca_desi_chr06	837890	[A/G]	NA
Ca_Desi_Chr_6_837921	Ca_desi_chr06	837921	[G/A]	NA
Ca_Desi_Chr_6_872308	Ca_desi_chr06	872308	[T/A]	NA
Ca_Desi_Chr_6_1353076	Ca_desi_chr06	1353076	[T/G]	NA
Ca_Desi_Chr_6_1385751	Ca_desi_chr06	1385751	[T/C]	NA
Ca_Desi_Chr_6_1385759	Ca_desi_chr06	1385759	[G/A]	NA
Ca_Desi_Chr_6_1399383	Ca_desi_chr06	1399383	[A/C]	NA
Ca_Desi_Chr_6_1400111	Ca_desi_chr06	1400111	[T/C]	NA
Ca_Desi_Chr_6_1400124	Ca_desi_chr06	1400124	[T/C]	NA
Ca_Desi_Chr_6_1400152	Ca_desi_chr06	1400152	[A/G]	NA
Ca_Desi_Chr_6_1404688	Ca_desi_chr06	1404688	[T/C]	NA
Ca_Desi_Chr_6_1404695	Ca_desi_chr06	1404695	[A/G]	NA
Ca_Desi_Chr_6_1404699	Ca_desi_chr06	1404699	[T/C]	NA
Ca_Desi_Chr_6_1404721	Ca_desi_chr06	1404721	[A/G]	NA
Ca_Desi_Chr_6_1404723	Ca_desi_chr06	1404723	[A/G]	NA
Ca_Desi_Chr_6_1404734	Ca_desi_chr06	1404734	[A/G]	NA
Ca_Desi_Chr_6_1428933	Ca_desi_chr06	1428933	[T/G]	NA
Ca_Desi_Chr_6_1428953	Ca_desi_chr06	1428953	[A/C]	NA
Ca_Desi_Chr_6_1498051	Ca_desi_chr06	1498051	[T/G]	NA
Ca_Desi_Chr_6_1530870	Ca_desi_chr06	1530870	[A/T]	NA
Ca_Desi_Chr_6_1530874	Ca_desi_chr06	1530874	[T/C]	NA
Ca_Desi_Chr_6_1530883	Ca_desi_chr06	1530883	[T/C]	NA
Ca_Desi_Chr_6_1530895	Ca_desi_chr06	1530895	[T/G]	NA
Ca_Desi_Chr_6_1530897	Ca_desi_chr06	1530897	[A/C]	NA
Ca_Desi_Chr_6_1530910	Ca_desi_chr06	1530910	[T/C]	NA
Ca_Desi_Chr_6_1608546	Ca_desi_chr06	1608546	[T/G]	NA
Ca_Desi_Chr_6_1608564	Ca_desi_chr06	1608564	[A/G]	NA
Ca_Desi_Chr_6_1608570	Ca_desi_chr06	1608570	[T/C]	NA
Ca_Desi_Chr_6_1608584	Ca_desi_chr06	1608584	[T/A]	NA
Ca_Desi_Chr_6_1671674	Ca_desi_chr06	1671674	[A/G]	NA
Ca_Desi_Chr_6_1677536	Ca_desi_chr06	1677536	[A/C]	NA
Ca_Desi_Chr_6_1677550	Ca_desi_chr06	1677550	[A/C]	NA
Ca_Desi_Chr_6_1719311	Ca_desi_chr06	1719311	[T/G]	NA

SNP IDs	Chromosomes	Physical positions (bp)	SNPs	NCBI SNPdb accession IDs (NCBI_ss#)
Ca_Desi_Chr_6_1878679	Ca_desi_chr06	1878679	[T/C]	NA
Ca_Desi_Chr_6_1890600	Ca_desi_chr06	1890600	[C/A]	NA
Ca_Desi_Chr_6_1908568	Ca_desi_chr06	1908568	[A/G]	NA
Ca_Desi_Chr_6_1981937	Ca_desi_chr06	1981937	[T/C]	NA
Ca_Desi_Chr_6_1981943	Ca_desi_chr06	1981943	[A/G]	NA
Ca_Desi_Chr_6_1988583	Ca_desi_chr06	1988583	[T/G]	NA
Ca_Desi_Chr_6_1988635	Ca_desi_chr06	1988635	[A/C]	NA
Ca_Desi_Chr_6_1988649	Ca_desi_chr06	1988649	[T/G]	NA
Ca_Desi_Chr_6_1988650	Ca_desi_chr06	1988650	[A/T]	NA
Ca_Desi_Chr_6_1988665	Ca_desi_chr06	1988665	[A/G]	NA
Ca_Desi_Chr_6_2012657	Ca_desi_chr06	2012657	[T/C]	NA
Ca_Desi_Chr_6_2012663	Ca_desi_chr06	2012663	[T/C]	NA
Ca_Desi_Chr_6_2012691	Ca_desi_chr06	2012691	[T/C]	NA
Ca_Desi_Chr_6_2012704	Ca_desi_chr06	2012704	[A/C]	NA
Ca_Desi_Chr_6_2012711	Ca_desi_chr06	2012711	[T/C]	NA
Ca_Desi_Chr_6_2046940	Ca_desi_chr06	2046940	[T/G]	NA
Ca_Desi_Chr_6_2054992	Ca_desi_chr06	2054992	[T/G]	NA
Ca_Desi_Chr_6_2055044	Ca_desi_chr06	2055044	[A/C]	NA
Ca_Desi_Chr_6_2055051	Ca_desi_chr06	2055051	[A/C]	NA
Ca_Desi_Chr_6_2128596	Ca_desi_chr06	2128596	[T/C]	NA
Ca_Desi_Chr_6_2128602	Ca_desi_chr06	2128602	[T/G]	NA
Ca_Desi_Chr_6_2182019	Ca_desi_chr06	2182019	[T/C]	NA
Ca_Desi_Chr_6_2187070	Ca_desi_chr06	2187070	[T/A]	NA
Ca_Desi_Chr_6_2187091	Ca_desi_chr06	2187091	[A/C]	NA
Ca_Desi_Chr_6_2187115	Ca_desi_chr06	2187115	[T/C]	NA
Ca_Desi_Chr_6_2297822	Ca_desi_chr06	2297822	[T/C]	NA
Ca_Desi_Chr_6_2297831	Ca_desi_chr06	2297831	[T/C]	NA
Ca_Desi_Chr_6_2297835	Ca_desi_chr06	2297835	[G/C]	NA
Ca_Desi_Chr_6_2297853	Ca_desi_chr06	2297853	[T/C]	NA
Ca_Desi_Chr_6_2297861	Ca_desi_chr06	2297861	[A/G]	NA
Ca_Desi_Chr_6_2304316	Ca_desi_chr06	2304316	[A/C]	NA
Ca_Desi_Chr_6_2304317	Ca_desi_chr06	2304317	[G/C]	NA
Ca_Desi_Chr_6_2304318	Ca_desi_chr06	2304318	[A/G]	NA
Ca_Desi_Chr_6_2387723	Ca_desi_chr06	2387723	[T/G]	NA

SNP IDs	Chromosomes	Physical positions (bp)	SNPs	NCBI SNPdb accession IDs (NCBI_ss#)
Ca_Desi_Chr_6_2401739	Ca_desi_chr06	2401739	[T/A]	NA
Ca_Desi_Chr_6_2401789	Ca_desi_chr06	2401789	[T/G]	NA
Ca_Desi_Chr_6_2409341	Ca_desi_chr06	2409341	[A/G]	NA
Ca_Desi_Chr_6_2615000	Ca_desi_chr06	2615000	[C/T]	NA
Ca_Desi_Chr_6_2615001	Ca_desi_chr06	2615001	[T/C]	NA
Ca_Desi_Chr_6_2694591	Ca_desi_chr06	2694591	[A/G]	NA
Ca_Desi_Chr_6_2694596	Ca_desi_chr06	2694596	[A/G]	NA
Ca_Desi_Chr_6_2694630	Ca_desi_chr06	2694630	[T/G]	NA
Ca_Desi_Chr_6_2708655	Ca_desi_chr06	2708655	[A/T]	NA
Ca_Desi_Chr_6_2708683	Ca_desi_chr06	2708683	[C/T]	NA
Ca_Desi_Chr_6_2712681	Ca_desi_chr06	2712681	[A/C]	NA
Ca_Desi_Chr_6_2721172	Ca_desi_chr06	2721172	[A/G]	NA
Ca_Desi_Chr_6_2721181	Ca_desi_chr06	2721181	[C/A]	NA
Ca_Desi_Chr_6_2721251	Ca_desi_chr06	2721251	[A/C]	NA
Ca_Desi_Chr_6_2840091	Ca_desi_chr06	2840091	[T/C]	NA
Ca_Desi_Chr_6_2841114	Ca_desi_chr06	2841114	[A/T]	NA
Ca_Desi_Chr_6_2841126	Ca_desi_chr06	2841126	[T/C]	NA
Ca_Desi_Chr_6_2841130	Ca_desi_chr06	2841130	[A/G]	NA
Ca_Desi_Chr_6_2841153	Ca_desi_chr06	2841153	[T/C]	NA
Ca_Desi_Chr_6_2852350	Ca_desi_chr06	2852350	[A/G]	NA
Ca_Desi_Chr_6_2852369	Ca_desi_chr06	2852369	[T/C]	NA
Ca_Desi_Chr_6_2955294	Ca_desi_chr06	2955294	[A/C]	NA
Ca_Desi_Chr_6_2955297	Ca_desi_chr06	2955297	[C/T]	NA
Ca_Desi_Chr_6_2955308	Ca_desi_chr06	2955308	[T/A]	NA
Ca_Desi_Chr_6_2955310	Ca_desi_chr06	2955310	[A/T]	NA
Ca_Desi_Chr_6_2982105	Ca_desi_chr06	2982105	[T/C]	NA
Ca_Desi_Chr_6_3293123	Ca_desi_chr06	3293123	[T/C]	NA
Ca_Desi_Chr_6_3293147	Ca_desi_chr06	3293147	[A/C]	NA
Ca_Desi_Chr_6_3295359	Ca_desi_chr06	3295359	[A/C]	NA
Ca_Desi_Chr_6_3307383	Ca_desi_chr06	3307383	[T/C]	NA
Ca_Desi_Chr_6_3307385	Ca_desi_chr06	3307385	[A/C]	NA
Ca_Desi_Chr_6_3307395	Ca_desi_chr06	3307395	[T/G]	NA
Ca_Desi_Chr_6_3358488	Ca_desi_chr06	3358488	[T/G]	NA
Ca_Desi_Chr_6_3358575	Ca_desi_chr06	3358575	[T/G]	NA

SNP IDs	Chromosomes	Physical positions (bp)	SNPs	NCBI SNPdb accession IDs (NCBI_ss#)
Ca_Desi_Chr_6_3359184	Ca_desi_chr06	3359184	[T/C]	NA
Ca_Desi_Chr_6_3359206	Ca_desi_chr06	3359206	[A/C]	NA
Ca_Desi_Chr_6_3377407	Ca_desi_chr06	3377407	[T/G]	NA
Ca_Desi_Chr_6_3385734	Ca_desi_chr06	3385734	[T/A]	NA
Ca_Desi_Chr_6_3385743	Ca_desi_chr06	3385743	[T/G]	NA
Ca_Desi_Chr_6_3385756	Ca_desi_chr06	3385756	[T/G]	NA
Ca_Desi_Chr_6_3385770	Ca_desi_chr06	3385770	[A/G]	NA
Ca_Desi_Chr_6_3385772	Ca_desi_chr06	3385772	[T/G]	NA
Ca_Desi_Chr_6_3385798	Ca_desi_chr06	3385798	[A/T]	NA
Ca_Desi_Chr_6_3420559	Ca_desi_chr06	3420559	[T/C]	NA
Ca_Desi_Chr_6_3420684	Ca_desi_chr06	3420684	[A/G]	NA
Ca_Desi_Chr_6_3437528	Ca_desi_chr06	3437528	[T/A]	NA
Ca_Desi_Chr_6_3437537	Ca_desi_chr06	3437537	[T/G]	NA
Ca_Desi_Chr_6_3439456	Ca_desi_chr06	3439456	[T/G]	NA
Ca_Desi_Chr_6_3439480	Ca_desi_chr06	3439480	[T/C]	NA
Ca_Desi_Chr_6_3439501	Ca_desi_chr06	3439501	[T/C]	NA
Ca_Desi_Chr_6_3439510	Ca_desi_chr06	3439510	[T/G]	NA
Ca_Desi_Chr_6_3439522	Ca_desi_chr06	3439522	[A/G]	NA
Ca_Desi_Chr_6_3439525	Ca_desi_chr06	3439525	[A/G]	NA
Ca_Desi_Chr_6_3439531	Ca_desi_chr06	3439531	[T/G]	NA
Ca_Desi_Chr_6_3439534	Ca_desi_chr06	3439534	[A/G]	NA
Ca_Desi_Chr_6_3439550	Ca_desi_chr06	3439550	[A/G]	NA
Ca_Desi_Chr_6_3439555	Ca_desi_chr06	3439555	[A/G]	NA
Ca_Desi_Chr_6_3456474	Ca_desi_chr06	3456474	[A/T]	NA
Ca_Desi_Chr_6_3509278	Ca_desi_chr06	3509278	[A/G]	NA
Ca_Desi_Chr_6_3556652	Ca_desi_chr06	3556652	[T/C]	NA
Ca_Desi_Chr_6_3556692	Ca_desi_chr06	3556692	[A/G]	NA
Ca_Desi_Chr_6_3629315	Ca_desi_chr06	3629315	[T/A]	NA
Ca_Desi_Chr_6_3630697	Ca_desi_chr06	3630697	[A/G]	NA
Ca_Desi_Chr_6_3630704	Ca_desi_chr06	3630704	[A/G]	NA
Ca_Desi_Chr_6_3630726	Ca_desi_chr06	3630726	[A/G]	NA
Ca_Desi_Chr_6_3630748	Ca_desi_chr06	3630748	[T/G]	NA
Ca_Desi_Chr_6_3630750	Ca_desi_chr06	3630750	[T/A]	NA
Ca_Desi_Chr_6_3630753	Ca_desi_chr06	3630753	[A/G]	NA

SNP IDs	Chromosomes	Physical positions (bp)	SNPs	NCBI SNPdb accession IDs (NCBI_ss#)
Ca_Desi_Chr_6_3630805	Ca_desi_chr06	3630805	[T/G]	NA
Ca_Desi_Chr_6_3630810	Ca_desi_chr06	3630810	[T/G]	NA
Ca_Desi_Chr_6_3635393	Ca_desi_chr06	3635393	[T/C]	NA
Ca_Desi_Chr_6_3635445	Ca_desi_chr06	3635445	[T/A]	NA
Ca_Desi_Chr_6_3636833	Ca_desi_chr06	3636833	[T/G]	NA
Ca_Desi_Chr_6_3778024	Ca_desi_chr06	3778024	[A/G]	NA
Ca_Desi_Chr_6_3778078	Ca_desi_chr06	3778078	[T/C]	NA
Ca_Desi_Chr_6_3912449	Ca_desi_chr06	3912449	[A/G]	NA
Ca_Desi_Chr_6_3912452	Ca_desi_chr06	3912452	[T/G]	NA
Ca_Desi_Chr_6_3954796	Ca_desi_chr06	3954796	[T/C]	NA
Ca_Desi_Chr_6_3954808	Ca_desi_chr06	3954808	[T/C]	NA
Ca_Desi_Chr_6_3954811	Ca_desi_chr06	3954811	[A/G]	NA
Ca_Desi_Chr_6_3954826	Ca_desi_chr06	3954826	[T/C]	NA
Ca_Desi_Chr_6_3954829	Ca_desi_chr06	3954829	[A/G]	NA
Ca_Desi_Chr_6_3954836	Ca_desi_chr06	3954836	[A/C]	NA
Ca_Desi_Chr_6_3955583	Ca_desi_chr06	3955583	[T/G]	NA
Ca_Desi_Chr_6_3955615	Ca_desi_chr06	3955615	[T/G]	NA
Ca_Desi_Chr_6_3955632	Ca_desi_chr06	3955632	[T/C]	NA
Ca_Desi_Chr_6_3955665	Ca_desi_chr06	3955665	[T/G]	NA
Ca_Desi_Chr_6_3955706	Ca_desi_chr06	3955706	[A/G]	NA
Ca_Desi_Chr_6_3956720	Ca_desi_chr06	3956720	[T/G]	NA
Ca_Desi_Chr_6_3958734	Ca_desi_chr06	3958734	[T/C]	NA
Ca_Desi_Chr_6_3958754	Ca_desi_chr06	3958754	[T/G]	NA
Ca_Desi_Chr_6_3958764	Ca_desi_chr06	3958764	[A/C]	NA
Ca_Desi_Chr_6_3958797	Ca_desi_chr06	3958797	[A/C]	NA
Ca_Desi_Chr_6_3958860	Ca_desi_chr06	3958860	[A/G]	NA
Ca_Desi_Chr_6_3958872	Ca_desi_chr06	3958872	[T/G]	NA
Ca_Desi_Chr_6_4062416	Ca_desi_chr06	4062416	[G/A]	NA
Ca_Desi_Chr_6_4062428	Ca_desi_chr06	4062428	[C/A]	NA
Ca_Desi_Chr_6_4072744	Ca_desi_chr06	4072744	[T/C]	NA
Ca_Desi_Chr_6_4072760	Ca_desi_chr06	4072760	[A/G]	NA
Ca_Desi_Chr_6_4072763	Ca_desi_chr06	4072763	[A/C]	NA
Ca_Desi_Chr_6_4072775	Ca_desi_chr06	4072775	[T/C]	NA
Ca_Desi_Chr_6_4072793	Ca_desi_chr06	4072793	[T/C]	NA

SNP IDs	Chromosomes	Physical positions (bp)	SNPs	NCBI SNPdb accession IDs (NCBI_ss#)
Ca_Desi_Chr_6_4072800	Ca_desi_chr06	4072800	[T/C]	NA
Ca_Desi_Chr_6_4082183	Ca_desi_chr06	4082183	[T/C]	NA
Ca_Desi_Chr_6_4082195	Ca_desi_chr06	4082195	[T/G]	NA
Ca_Desi_Chr_6_4082209	Ca_desi_chr06	4082209	[T/G]	NA
Ca_Desi_Chr_6_4082221	Ca_desi_chr06	4082221	[A/G]	NA
Ca_Desi_Chr_6_4082239	Ca_desi_chr06	4082239	[C/G]	NA
Ca_Desi_Chr_6_4082241	Ca_desi_chr06	4082241	[T/C]	NA
Ca_Desi_Chr_6_4142557	Ca_desi_chr06	4142557	[T/G]	NA
Ca_Desi_Chr_6_4142665	Ca_desi_chr06	4142665	[T/A]	NA
Ca_Desi_Chr_6_4231783	Ca_desi_chr06	4231783	[A/C]	NA
Ca_Desi_Chr_6_4240873	Ca_desi_chr06	4240873	[A/G]	NA
Ca_Desi_Chr_6_4240884	Ca_desi_chr06	4240884	[T/G]	NA
Ca_Desi_Chr_6_4273801	Ca_desi_chr06	4273801	[A/G]	NA
Ca_Desi_Chr_6_4344336	Ca_desi_chr06	4344336	[T/G]	NA
Ca_Desi_Chr_6_4358576	Ca_desi_chr06	4358576	[A/G]	NA
Ca_Desi_Chr_6_4384783	Ca_desi_chr06	4384783	[T/C]	NA
Ca_Desi_Chr_6_4384784	Ca_desi_chr06	4384784	[G/T]	NA
Ca_Desi_Chr_6_4384803	Ca_desi_chr06	4384803	[A/C]	NA
Ca_Desi_Chr_6_4395405	Ca_desi_chr06	4395405	[A/G]	NA
Ca_Desi_Chr_6_4403586	Ca_desi_chr06	4403586	[A/G]	NA
Ca_Desi_Chr_6_4403587	Ca_desi_chr06	4403587	[T/G]	NA
Ca_Desi_Chr_6_4403588	Ca_desi_chr06	4403588	[C/T]	NA
Ca_Desi_Chr_6_4403589	Ca_desi_chr06	4403589	[T/G]	NA
Ca_Desi_Chr_6_4464430	Ca_desi_chr06	4464430	[T/G]	NA
Ca_Desi_Chr_6_4464525	Ca_desi_chr06	4464525	[A/T]	NA
Ca_Desi_Chr_6_4464527	Ca_desi_chr06	4464527	[A/C]	NA
Ca_Desi_Chr_6_4553112	Ca_desi_chr06	4553112	[T/G]	NA
Ca_Desi_Chr_6_4553440	Ca_desi_chr06	4553440	[A/G]	NA
Ca_Desi_Chr_6_4553465	Ca_desi_chr06	4553465	[A/G]	NA
Ca_Desi_Chr_6_4553475	Ca_desi_chr06	4553475	[T/C]	NA
Ca_Desi_Chr_6_4641663	Ca_desi_chr06	4641663	[T/C]	NA
Ca_Desi_Chr_6_4641674	Ca_desi_chr06	4641674	[A/C]	NA
Ca_Desi_Chr_6_4641708	Ca_desi_chr06	4641708	[T/G]	NA
Ca_Desi_Chr_6_4646376	Ca_desi_chr06	4646376	[G/A]	NA

SNP IDs	Chromosomes	Physical positions (bp)	SNPs	NCBI SNPdb accession IDs (NCBI_ss#)
Ca_Desi_Chr_6_4646391	Ca_desi_chr06	4646391	[A/C]	NA
Ca_Desi_Chr_6_4646393	Ca_desi_chr06	4646393	[A/C]	NA
Ca_Desi_Chr_6_4646799	Ca_desi_chr06	4646799	[A/T]	NA
Ca_Desi_Chr_6_4646807	Ca_desi_chr06	4646807	[A/G]	NA
Ca_Desi_Chr_6_4646819	Ca_desi_chr06	4646819	[A/T]	NA
Ca_Desi_Chr_6_4646848	Ca_desi_chr06	4646848	[A/C]	NA
Ca_Desi_Chr_6_4646858	Ca_desi_chr06	4646858	[T/C]	NA
Ca_Desi_Chr_6_4651071	Ca_desi_chr06	4651071	[A/C]	NA
Ca_Desi_Chr_6_4651164	Ca_desi_chr06	4651164	[T/C]	NA
Ca_Desi_Chr_6_4651190	Ca_desi_chr06	4651190	[A/C]	NA
Ca_Desi_Chr_6_4672196	Ca_desi_chr06	4672196	[T/G]	NA
Ca_Desi_Chr_6_4697911	Ca_desi_chr06	4697911	[T/G]	NA
Ca_Desi_Chr_6_4698856	Ca_desi_chr06	4698856	[A/C]	NA
Ca_Desi_Chr_6_4723758	Ca_desi_chr06	4723758	[C/A]	NA
Ca_Desi_Chr_6_4723761	Ca_desi_chr06	4723761	[T/G]	NA
Ca_Desi_Chr_6_4723762	Ca_desi_chr06	4723762	[C/A]	NA
Ca_Desi_Chr_6_4723763	Ca_desi_chr06	4723763	[T/G]	NA
Ca_Desi_Chr_6_4733449	Ca_desi_chr06	4733449	[T/G]	NA
Ca_Desi_Chr_6_4733514	Ca_desi_chr06	4733514	[A/C]	NA
Ca_Desi_Chr_6_4733525	Ca_desi_chr06	4733525	[T/C]	NA
Ca_Desi_Chr_6_4733551	Ca_desi_chr06	4733551	[A/C]	NA
Ca_Desi_Chr_6_4734361	Ca_desi_chr06	4734361	[C/A]	NA
Ca_Desi_Chr_6_4734362	Ca_desi_chr06	4734362	[T/G]	NA
Ca_Desi_Chr_6_4734363	Ca_desi_chr06	4734363	[T/A]	NA
Ca_Desi_Chr_6_4734364	Ca_desi_chr06	4734364	[C/T]	NA
Ca_Desi_Chr_6_4739968	Ca_desi_chr06	4739968	[T/A]	NA
Ca_Desi_Chr_6_4739978	Ca_desi_chr06	4739978	[T/G]	NA
Ca_Desi_Chr_6_4761059	Ca_desi_chr06	4761059	[T/A]	NA
Ca_Desi_Chr_6_4761060	Ca_desi_chr06	4761060	[A/G]	NA
Ca_Desi_Chr_6_4792757	Ca_desi_chr06	4792757	[T/G]	NA
Ca_Desi_Chr_6_4793027	Ca_desi_chr06	4793027	[A/G]	NA
Ca_Desi_Chr_6_4793035	Ca_desi_chr06	4793035	[A/G]	NA
Ca_Desi_Chr_6_4793041	Ca_desi_chr06	4793041	[A/G]	NA
Ca_Desi_Chr_6_4793047	Ca_desi_chr06	4793047	[A/G]	NA

SNP IDs	Chromosomes	Physical positions (bp)	SNPs	NCBI SNPdb accession IDs (NCBI_ss#)
Ca_Desi_Chr_6_4793051	Ca_desi_chr06	4793051	[A/C]	NA
Ca_Desi_Chr_6_4793056	Ca_desi_chr06	4793056	[A/G]	NA
Ca_Desi_Chr_6_4793062	Ca_desi_chr06	4793062	[T/C]	NA
Ca_Desi_Chr_6_4793064	Ca_desi_chr06	4793064	[T/C]	NA
Ca_Desi_Chr_6_4793084	Ca_desi_chr06	4793084	[A/G]	NA
Ca_Desi_Chr_6_4793088	Ca_desi_chr06	4793088	[T/A]	NA
Ca_Desi_Chr_6_4793106	Ca_desi_chr06	4793106	[T/C]	NA
Ca_Desi_Chr_6_4793120	Ca_desi_chr06	4793120	[T/G]	NA
Ca_Desi_Chr_6_4793124	Ca_desi_chr06	4793124	[A/G]	NA
Ca_Desi_Chr_6_4793126	Ca_desi_chr06	4793126	[A/G]	NA
Ca_Desi_Chr_6_4793128	Ca_desi_chr06	4793128	[A/G]	NA
Ca_Desi_Chr_6_4793130	Ca_desi_chr06	4793130	[T/G]	NA
Ca_Desi_Chr_6_4793137	Ca_desi_chr06	4793137	[A/G]	NA
Ca_Desi_Chr_6_4793141	Ca_desi_chr06	4793141	[T/G]	NA
Ca_Desi_Chr_6_4793145	Ca_desi_chr06	4793145	[T/C]	NA
Ca_Desi_Chr_6_4793538	Ca_desi_chr06	4793538	[T/A]	NA
Ca_Desi_Chr_6_4794434	Ca_desi_chr06	4794434	[C/A]	NA
Ca_Desi_Chr_6_4794780	Ca_desi_chr06	4794780	[T/C]	NA
Ca_Desi_Chr_6_4794803	Ca_desi_chr06	4794803	[T/C]	NA
Ca_Desi_Chr_6_4799876	Ca_desi_chr06	4799876	[A/T]	NA
Ca_Desi_Chr_6_4799887	Ca_desi_chr06	4799887	[A/T]	NA
Ca_Desi_Chr_6_4804469	Ca_desi_chr06	4804469	[T/C]	NA
Ca_Desi_Chr_6_4807031	Ca_desi_chr06	4807031	[T/G]	NA
Ca_Desi_Chr_6_4807042	Ca_desi_chr06	4807042	[T/A]	NA
Ca_Desi_Chr_6_4807094	Ca_desi_chr06	4807094	[A/T]	NA
Ca_Desi_Chr_6_4820438	Ca_desi_chr06	4820438	[T/C]	NA
Ca_Desi_Chr_6_4820440	Ca_desi_chr06	4820440	[A/C]	NA
Ca_Desi_Chr_6_4820445	Ca_desi_chr06	4820445	[A/C]	NA
Ca_Desi_Chr_6_4820457	Ca_desi_chr06	4820457	[A/C]	NA
Ca_Desi_Chr_6_4820463	Ca_desi_chr06	4820463	[T/C]	NA
Ca_Desi_Chr_6_4820465	Ca_desi_chr06	4820465	[T/C]	NA
Ca_Desi_Chr_6_4820472	Ca_desi_chr06	4820472	[A/C]	NA
Ca_Desi_Chr_6_4820478	Ca_desi_chr06	4820478	[T/C]	NA
Ca_Desi_Chr_6_4836612	Ca_desi_chr06	4836612	[T/C]	NA

SNP IDs	Chromosomes	Physical positions (bp)	SNPs	NCBI SNPdb accession IDs (NCBI_ss#)
Ca_Desi_Chr_6_4836614	Ca_desi_chr06	4836614	[A/C]	NA
Ca_Desi_Chr_6_4836623	Ca_desi_chr06	4836623	[T/C]	NA
Ca_Desi_Chr_6_4836631	Ca_desi_chr06	4836631	[A/G]	NA
Ca_Desi_Chr_6_4836649	Ca_desi_chr06	4836649	[A/G]	NA
Ca_Desi_Chr_6_4836668	Ca_desi_chr06	4836668	[A/G]	NA
Ca_Desi_Chr_6_4863652	Ca_desi_chr06	4863652	[A/T]	NA
Ca_Desi_Chr_6_4863685	Ca_desi_chr06	4863685	[C/A]	NA
Ca_Desi_Chr_6_4868317	Ca_desi_chr06	4868317	[A/C]	NA
Ca_Desi_Chr_6_4868402	Ca_desi_chr06	4868402	[A/T]	NA
Ca_Desi_Chr_6_4868416	Ca_desi_chr06	4868416	[T/G]	NA
Ca_Desi_Chr_6_4868422	Ca_desi_chr06	4868422	[T/C]	NA
Ca_Desi_Chr_6_4908422	Ca_desi_chr06	4908422	[T/C]	NA
Ca_Desi_Chr_6_4909247	Ca_desi_chr06	4909247	[T/G]	NA
Ca_Desi_Chr_6_4910031	Ca_desi_chr06	4910031	[G/A]	NA
Ca_Desi_Chr_6_4910422	Ca_desi_chr06	4910422	[C/T]	NA
Ca_Desi_Chr_6_4910434	Ca_desi_chr06	4910434	[T/C]	NA
Ca_Desi_Chr_6_4911436	Ca_desi_chr06	4911436	[C/T]	NA
Ca_Desi_Chr_6_4911475	Ca_desi_chr06	4911475	[T/C]	NA
Ca_Desi_Chr_6_4911716	Ca_desi_chr06	4911716	[G/T]	NA
Ca_Desi_Chr_6_4915651	Ca_desi_chr06	4915651	[T/A]	NA
Ca_Desi_Chr_6_4915652	Ca_desi_chr06	4915652	[G/A]	NA
Ca_Desi_Chr_6_4915673	Ca_desi_chr06	4915673	[T/G]	NA
Ca_Desi_Chr_6_4920326	Ca_desi_chr06	4920326	[G/T]	NA
Ca_Desi_Chr_6_4939703	Ca_desi_chr06	4939703	[A/G]	NA
Ca_Desi_Chr_6_4939711	Ca_desi_chr06	4939711	[C/T]	NA
Ca_Desi_Chr_6_4999533	Ca_desi_chr06	4999533	[T/G]	NA
Ca_Desi_Chr_6_5017058	Ca_desi_chr06	5017058	[A/C]	NA
Ca_Desi_Chr_6_5017065	Ca_desi_chr06	5017065	[A/G]	NA
Ca_Desi_Chr_6_5552713	Ca_desi_chr06	5552713	[C/G]	NA
Ca_Desi_Chr_6_5565255	Ca_desi_chr06	5565255	[A/T]	NA
Ca_Desi_Chr_6_5565258	Ca_desi_chr06	5565258	[G/C]	NA
Ca_Desi_Chr_6_5565294	Ca_desi_chr06	5565294	[T/G]	NA
Ca_Desi_Chr_6_5573898	Ca_desi_chr06	5573898	[T/C]	NA
Ca_Desi_Chr_6_5574091	Ca_desi_chr06	5574091	[C/T]	NA

SNP IDs	Chromosomes	Physical positions (bp)	SNPs	NCBI SNPdb accession IDs (NCBI_ss#)
Ca_Desi_Chr_6_5582291	Ca_desi_chr06	5582291	[T/C]	NA
Ca_Desi_Chr_6_5600377	Ca_desi_chr06	5600377	[C/A]	NA
Ca_Desi_Chr_6_5601147	Ca_desi_chr06	5601147	[G/C]	NA
Ca_Desi_Chr_6_5601304	Ca_desi_chr06	5601304	[G/A]	NA
Ca_Desi_Chr_6_5606330	Ca_desi_chr06	5606330	[A/C]	NA
Ca_Desi_Chr_6_5607979	Ca_desi_chr06	5607979	[A/G]	NA
Ca_Desi_Chr_6_5623892	Ca_desi_chr06	5623892	[T/C]	NA
Ca_Desi_Chr_6_5644572	Ca_desi_chr06	5644572	[T/C]	NA
Ca_Desi_Chr_6_5765527	Ca_desi_chr06	5765527	[T/G]	NA
Ca_Desi_Chr_6_5765536	Ca_desi_chr06	5765536	[T/G]	NA
Ca_Desi_Chr_6_5765551	Ca_desi_chr06	5765551	[G/A]	NA
Ca_Desi_Chr_6_5793661	Ca_desi_chr06	5793661	[T/C]	NA
Ca_Desi_Chr_6_5793689	Ca_desi_chr06	5793689	[A/C]	NA
Ca_Desi_Chr_6_5805145	Ca_desi_chr06	5805145	[G/T]	NA
Ca_Desi_Chr_6_5805148	Ca_desi_chr06	5805148	[T/C]	NA
Ca_Desi_Chr_6_5805150	Ca_desi_chr06	5805150	[T/C]	NA
Ca_Desi_Chr_6_5830309	Ca_desi_chr06	5830309	[C/T]	NA
Ca_Desi_Chr_6_5830337	Ca_desi_chr06	5830337	[G/T]	NA
Ca_Desi_Chr_6_5830357	Ca_desi_chr06	5830357	[T/C]	NA
Ca_Desi_Chr_6_5855179	Ca_desi_chr06	5855179	[G/A]	NA
Ca_Desi_Chr_6_5855793	Ca_desi_chr06	5855793	[T/A]	NA
Ca_Desi_Chr_6_5855826	Ca_desi_chr06	5855826	[C/T]	NA
Ca_Desi_Chr_6_5856372	Ca_desi_chr06	5856372	[T/A]	NA
Ca_Desi_Chr_6_5856391	Ca_desi_chr06	5856391	[T/G]	NA
Ca_Desi_Chr_6_5857703	Ca_desi_chr06	5857703	[A/G]	NA
Ca_Desi_Chr_6_5863791	Ca_desi_chr06	5863791	[T/G]	NA
Ca_Desi_Chr_6_5875894	Ca_desi_chr06	5875894	[T/C]	NA
Ca_Desi_Chr_6_5875912	Ca_desi_chr06	5875912	[A/C]	NA
Ca_Desi_Chr_6_5875935	Ca_desi_chr06	5875935	[A/G]	NA
Ca_Desi_Chr_6_5881201	Ca_desi_chr06	5881201	[C/T]	NA
Ca_Desi_Chr_6_5923519	Ca_desi_chr06	5923519	[T/C]	NA
Ca_Desi_Chr_6_5923536	Ca_desi_chr06	5923536	[A/G]	NA
Ca_Desi_Chr_6_5923545	Ca_desi_chr06	5923545	[A/G]	NA
Ca_Desi_Chr_6_5923566	Ca_desi_chr06	5923566	[T/C]	NA

SNP IDs	Chromosomes	Physical positions (bp)	SNPs	NCBI SNPdb accession IDs (NCBI_ss#)
Ca_Desi_Chr_6_5931952	Ca_desi_chr06	5931952	[C/G]	NA
Ca_Desi_Chr_6_5931987	Ca_desi_chr06	5931987	[C/T]	NA
Ca_Desi_Chr_6_5933120	Ca_desi_chr06	5933120	[C/T]	NA
Ca_Desi_Chr_6_5933446	Ca_desi_chr06	5933446	[A/G]	NA
Ca_Desi_Chr_6_5941908	Ca_desi_chr06	5941908	[T/C]	NA
Ca_Desi_Chr_6_5942035	Ca_desi_chr06	5942035	[T/C]	NA
Ca_Desi_Chr_6_5942107	Ca_desi_chr06	5942107	[C/A]	NA
Ca_Desi_Chr_6_5942108	Ca_desi_chr06	5942108	[A/T]	NA
Ca_Desi_Chr_6_5942109	Ca_desi_chr06	5942109	[A/C]	NA
Ca_Desi_Chr_6_5942110	Ca_desi_chr06	5942110	[G/T]	NA
Ca_Desi_Chr_6_5942121	Ca_desi_chr06	5942121	[G/A]	NA
Ca_Desi_Chr_6_5942213	Ca_desi_chr06	5942213	[T/G]	NA
Ca_Desi_Chr_6_5942263	Ca_desi_chr06	5942263	[T/G]	NA
Ca_Desi_Chr_6_5943258	Ca_desi_chr06	5943258	[A/T]	NA
Ca_Desi_Chr_6_5943278	Ca_desi_chr06	5943278	[T/G]	NA
Ca_Desi_Chr_6_5943369	Ca_desi_chr06	5943369	[C/T]	NA
Ca_Desi_Chr_6_5943489	Ca_desi_chr06	5943489	[T/C]	NA
Ca_Desi_Chr_6_5943564	Ca_desi_chr06	5943564	[C/T]	NA
Ca_Desi_Chr_6_5944029	Ca_desi_chr06	5944029	[A/G]	NA
Ca_Desi_Chr_6_5944054	Ca_desi_chr06	5944054	[A/G]	NA
Ca_Desi_Chr_6_5944178	Ca_desi_chr06	5944178	[C/T]	NA
Ca_Desi_Chr_6_5944184	Ca_desi_chr06	5944184	[C/T]	NA
Ca_Desi_Chr_6_5946300	Ca_desi_chr06	5946300	[T/C]	NA
Ca_Desi_Chr_6_5946308	Ca_desi_chr06	5946308	[T/C]	NA
Ca_Desi_Chr_6_5946749	Ca_desi_chr06	5946749	[C/T]	NA
Ca_Desi_Chr_6_5946974	Ca_desi_chr06	5946974	[T/C]	NA
Ca_Desi_Chr_6_5947020	Ca_desi_chr06	5947020	[T/G]	NA
Ca_Desi_Chr_6_5947049	Ca_desi_chr06	5947049	[G/T]	NA
Ca_Desi_Chr_6_5947076	Ca_desi_chr06	5947076	[A/G]	NA
Ca_Desi_Chr_6_5947130	Ca_desi_chr06	5947130	[G/A]	NA
Ca_Desi_Chr_6_5947178	Ca_desi_chr06	5947178	[T/A]	NA
Ca_Desi_Chr_6_5947488	Ca_desi_chr06	5947488	[A/C]	NA
Ca_Desi_Chr_6_5947664	Ca_desi_chr06	5947664	[G/A]	NA
Ca_Desi_Chr_6_5947773	Ca_desi_chr06	5947773	[C/A]	NA

SNP IDs	Chromosomes	Physical positions (bp)	SNPs	NCBI SNPdb accession IDs (NCBI_ss#)
Ca_Desi_Chr_6_5947774	Ca_desi_chr06	5947774	[A/G]	NA
Ca_Desi_Chr_6_5947913	Ca_desi_chr06	5947913	[A/G]	NA
Ca_Desi_Chr_6_5948138	Ca_desi_chr06	5948138	[T/A]	NA
Ca_Desi_Chr_6_5948987	Ca_desi_chr06	5948987	[T/G]	NA
Ca_Desi_Chr_6_5949006	Ca_desi_chr06	5949006	[T/C]	NA
Ca_Desi_Chr_6_5949037	Ca_desi_chr06	5949037	[C/T]	NA
Ca_Desi_Chr_6_5960706	Ca_desi_chr06	5960706	[T/G]	NA
Ca_Desi_Chr_6_5960810	Ca_desi_chr06	5960810	[C/G]	NA
Ca_Desi_Chr_6_5963675	Ca_desi_chr06	5963675	[T/C]	NA
Ca_Desi_Chr_6_5967285	Ca_desi_chr06	5967285	[C/G]	NA
Ca_Desi_Chr_6_5967982	Ca_desi_chr06	5967982	[T/G]	NA
Ca_Desi_Chr_6_5968766	Ca_desi_chr06	5968766	[G/A]	NA
Ca_Desi_Chr_6_5992325	Ca_desi_chr06	5992325	[A/C]	NA
Ca_Desi_Chr_6_5994534	Ca_desi_chr06	5994534	[T/C]	NA
Ca_Desi_Chr_6_5995400	Ca_desi_chr06	5995400	[A/C]	NA
Ca_Desi_Chr_6_5995429	Ca_desi_chr06	5995429	[A/C]	NA
Ca_Desi_Chr_6_5995441	Ca_desi_chr06	5995441	[A/G]	NA
Ca_Desi_Chr_6_5995447	Ca_desi_chr06	5995447	[T/A]	NA
Ca_Desi_Chr_6_6004431	Ca_desi_chr06	6004431	[G/C]	NA
Ca_Desi_Chr_6_6045978	Ca_desi_chr06	6045978	[C/A]	NA
Ca_Desi_Chr_6_6046044	Ca_desi_chr06	6046044	[T/C]	NA
Ca_Desi_Chr_6_6046096	Ca_desi_chr06	6046096	[G/A]	NA
Ca_Desi_Chr_6_6048304	Ca_desi_chr06	6048304	[C/T]	NA
Ca_Desi_Chr_6_6048641	Ca_desi_chr06	6048641	[G/A]	NA
Ca_Desi_Chr_6_6048799	Ca_desi_chr06	6048799	[T/G]	NA
Ca_Desi_Chr_6_6048824	Ca_desi_chr06	6048824	[C/T]	NA
Ca_Desi_Chr_6_6048899	Ca_desi_chr06	6048899	[C/T]	NA
Ca_Desi_Chr_6_6048900	Ca_desi_chr06	6048900	[A/G]	NA
Ca_Desi_Chr_6_6048909	Ca_desi_chr06	6048909	[T/C]	NA
Ca_Desi_Chr_6_6049168	Ca_desi_chr06	6049168	[A/C]	NA
Ca_Desi_Chr_6_6049189	Ca_desi_chr06	6049189	[C/T]	NA
Ca_Desi_Chr_6_6049208	Ca_desi_chr06	6049208	[C/A]	NA
Ca_Desi_Chr_6_6058595	Ca_desi_chr06	6058595	[T/G]	NA
Ca_Desi_Chr_6_6062805	Ca_desi_chr06	6062805	[T/G]	NA

SNP IDs	Chromosomes	Physical positions (bp)	SNPs	NCBI SNPdb accession IDs (NCBI_ss#)
Ca_Desi_Chr_6_6103720	Ca_desi_chr06	6103720	[G/T]	NA
Ca_Desi_Chr_6_6115682	Ca_desi_chr06	6115682	[C/T]	NA
Ca_Desi_Chr_6_6115721	Ca_desi_chr06	6115721	[A/T]	NA
Ca_Desi_Chr_6_6118896	Ca_desi_chr06	6118896	[C/T]	NA
Ca_Desi_Chr_6_6119010	Ca_desi_chr06	6119010	[T/C]	NA
Ca_Desi_Chr_6_6119042	Ca_desi_chr06	6119042	[A/C]	NA
Ca_Desi_Chr_6_6119060	Ca_desi_chr06	6119060	[A/G]	NA
Ca_Desi_Chr_6_6119061	Ca_desi_chr06	6119061	[A/G]	NA
Ca_Desi_Chr_6_6156756	Ca_desi_chr06	6156756	[T/G]	NA
Ca_Desi_Chr_6_6156757	Ca_desi_chr06	6156757	[C/T]	NA
Ca_Desi_Chr_6_6156758	Ca_desi_chr06	6156758	[T/G]	NA
Ca_Desi_Chr_6_6160317	Ca_desi_chr06	6160317	[T/G]	NA
Ca_Desi_Chr_6_6339018	Ca_desi_chr06	6339018	[A/C]	NA
Ca_Desi_Chr_6_6339058	Ca_desi_chr06	6339058	[A/T]	NA
Ca_Desi_Chr_6_6339065	Ca_desi_chr06	6339065	[A/G]	NA
Ca_Desi_Chr_6_6339067	Ca_desi_chr06	6339067	[A/G]	NA
Ca_Desi_Chr_6_6339069	Ca_desi_chr06	6339069	[T/C]	NA
Ca_Desi_Chr_6_6339071	Ca_desi_chr06	6339071	[C/T]	NA
Ca_Desi_Chr_6_6339077	Ca_desi_chr06	6339077	[T/C]	NA
Ca_Desi_Chr_6_6339080	Ca_desi_chr06	6339080	[T/C]	NA
Ca_Desi_Chr_6_6339092	Ca_desi_chr06	6339092	[T/C]	NA
Ca_Desi_Chr_6_6339098	Ca_desi_chr06	6339098	[T/C]	NA
Ca_Desi_Chr_6_6339107	Ca_desi_chr06	6339107	[A/G]	NA
Ca_Desi_Chr_6_6639714	Ca_desi_chr06	6639714	[G/T]	NA
Ca_Desi_Chr_6_6790124	Ca_desi_chr06	6790124	[T/G]	NA
Ca_Desi_Chr_6_6790130	Ca_desi_chr06	6790130	[A/G]	NA
Ca_Desi_Chr_6_6790134	Ca_desi_chr06	6790134	[T/G]	NA
Ca_Desi_Chr_6_6792785	Ca_desi_chr06	6792785	[C/A]	NA
Ca_Desi_Chr_6_6792786	Ca_desi_chr06	6792786	[T/C]	NA
Ca_Desi_Chr_6_6792795	Ca_desi_chr06	6792795	[A/G]	NA
Ca_Desi_Chr_6_6803689	Ca_desi_chr06	6803689	[T/G]	NA
Ca_Desi_Chr_6_6873851	Ca_desi_chr06	6873851	[C/T]	NA
Ca_Desi_Chr_6_6873868	Ca_desi_chr06	6873868	[A/G]	NA
Ca_Desi_Chr_6_6873877	Ca_desi_chr06	6873877	[A/G]	NA

SNP IDs	Chromosomes	Physical positions (bp)	SNPs	NCBI SNPdb accession IDs (NCBI_ss#)
Ca_Desi_Chr_6_6915571	Ca_desi_chr06	6915571	[T/G]	NA
Ca_Desi_Chr_6_6915580	Ca_desi_chr06	6915580	[C/G]	NA
Ca_Desi_Chr_6_6915583	Ca_desi_chr06	6915583	[A/G]	NA
Ca_Desi_Chr_6_6915592	Ca_desi_chr06	6915592	[T/G]	NA
Ca_Desi_Chr_6_6915595	Ca_desi_chr06	6915595	[T/C]	NA
Ca_Desi_Chr_6_6967871	Ca_desi_chr06	6967871	[T/C]	NA
Ca_Desi_Chr_6_6967897	Ca_desi_chr06	6967897	[T/C]	NA
Ca_Desi_Chr_6_6967906	Ca_desi_chr06	6967906	[A/C]	NA
Ca_Desi_Chr_6_6967913	Ca_desi_chr06	6967913	[A/T]	NA
Ca_Desi_Chr_6_6968565	Ca_desi_chr06	6968565	[T/C]	NA
Ca_Desi_Chr_6_6968621	Ca_desi_chr06	6968621	[T/G]	NA
Ca_Desi_Chr_6_6968622	Ca_desi_chr06	6968622	[T/G]	NA
Ca_Desi_Chr_6_6968653	Ca_desi_chr06	6968653	[A/G]	NA
Ca_Desi_Chr_6_6968674	Ca_desi_chr06	6968674	[A/G]	NA
Ca_Desi_Chr_6_6971912	Ca_desi_chr06	6971912	[A/C]	NA
Ca_Desi_Chr_6_6971913	Ca_desi_chr06	6971913	[G/C]	NA
Ca_Desi_Chr_6_6971915	Ca_desi_chr06	6971915	[T/A]	NA
Ca_Desi_Chr_6_6971916	Ca_desi_chr06	6971916	[C/A]	NA
Ca_Desi_Chr_6_6995741	Ca_desi_chr06	6995741	[T/C]	NA
Ca_Desi_Chr_6_6995761	Ca_desi_chr06	6995761	[A/C]	NA
Ca_Desi_Chr_6_6995777	Ca_desi_chr06	6995777	[A/C]	NA
Ca_Desi_Chr_6_6995786	Ca_desi_chr06	6995786	[A/G]	NA
Ca_Desi_Chr_6_6995797	Ca_desi_chr06	6995797	[T/C]	NA
Ca_Desi_Chr_6_7013550	Ca_desi_chr06	7013550	[T/G]	NA
Ca_Desi_Chr_6_7014087	Ca_desi_chr06	7014087	[A/C]	NA
Ca_Desi_Chr_6_7014121	Ca_desi_chr06	7014121	[T/C]	NA
Ca_Desi_Chr_6_7014125	Ca_desi_chr06	7014125	[A/G]	NA
Ca_Desi_Chr_6_7231208	Ca_desi_chr06	7231208	[T/C]	NA
Ca_Desi_Chr_6_7231232	Ca_desi_chr06	7231232	[A/C]	NA
Ca_Desi_Chr_6_7285638	Ca_desi_chr06	7285638	[T/A]	NA
Ca_Desi_Chr_6_7326383	Ca_desi_chr06	7326383	[T/C]	NA
Ca_Desi_Chr_6_7326401	Ca_desi_chr06	7326401	[T/C]	NA
Ca_Desi_Chr_6_7326410	Ca_desi_chr06	7326410	[A/G]	NA
Ca_Desi_Chr_6_7326413	Ca_desi_chr06	7326413	[T/G]	NA

SNP IDs	Chromosomes	Physical positions (bp)	SNPs	NCBI SNPdb accession IDs (NCBI_ss#)
Ca_Desi_Chr_6_7326416	Ca_desi_chr06	7326416	[A/G]	NA
Ca_Desi_Chr_6_7337712	Ca_desi_chr06	7337712	[T/G]	NA
Ca_Desi_Chr_6_7337756	Ca_desi_chr06	7337756	[T/G]	NA
Ca_Desi_Chr_6_7337815	Ca_desi_chr06	7337815	[T/A]	NA
Ca_Desi_Chr_6_7337822	Ca_desi_chr06	7337822	[T/G]	NA
Ca_Desi_Chr_6_7337860	Ca_desi_chr06	7337860	[T/C]	NA
Ca_Desi_Chr_6_7339767	Ca_desi_chr06	7339767	[T/G]	NA
Ca_Desi_Chr_6_7414765	Ca_desi_chr06	7414765	[A/G]	NA
Ca_Desi_Chr_6_7414789	Ca_desi_chr06	7414789	[A/G]	NA
Ca_Desi_Chr_6_7441444	Ca_desi_chr06	7441444	[T/C]	NA
Ca_Desi_Chr_6_7450018	Ca_desi_chr06	7450018	[G/A]	NA
Ca_Desi_Chr_6_7472934	Ca_desi_chr06	7472934	[C/T]	NA
Ca_Desi_Chr_6_7478424	Ca_desi_chr06	7478424	[T/C]	NA
Ca_Desi_Chr_6_7502284	Ca_desi_chr06	7502284	[G/A]	NA
Ca_Desi_Chr_6_7509650	Ca_desi_chr06	7509650	[G/A]	NA
Ca_Desi_Chr_6_7511058	Ca_desi_chr06	7511058	[C/G]	NA
Ca_Desi_Chr_6_7537055	Ca_desi_chr06	7537055	[G/T]	NA
Ca_Desi_Chr_6_7539901	Ca_desi_chr06	7539901	[T/A]	NA
Ca_Desi_Chr_6_7539911	Ca_desi_chr06	7539911	[A/G]	NA
Ca_Desi_Chr_6_7572539	Ca_desi_chr06	7572539	[T/C]	NA
Ca_Desi_Chr_6_7572553	Ca_desi_chr06	7572553	[A/G]	NA
Ca_Desi_Chr_6_7572558	Ca_desi_chr06	7572558	[T/C]	NA
Ca_Desi_Chr_6_7572571	Ca_desi_chr06	7572571	[T/G]	NA
Ca_Desi_Chr_6_7572577	Ca_desi_chr06	7572577	[T/A]	NA
Ca_Desi_Chr_6_7572600	Ca_desi_chr06	7572600	[A/C]	NA
Ca_Desi_Chr_6_7572619	Ca_desi_chr06	7572619	[A/T]	NA
Ca_Desi_Chr_6_7572707	Ca_desi_chr06	7572707	[A/C]	NA
Ca_Desi_Chr_6_7589557	Ca_desi_chr06	7589557	[G/A]	NA
Ca_Desi_Chr_6_7594832	Ca_desi_chr06	7594832	[T/C]	NA
Ca_Desi_Chr_6_7597632	Ca_desi_chr06	7597632	[T/G]	NA
Ca_Desi_Chr_6_7597654	Ca_desi_chr06	7597654	[G/A]	NA
Ca_Desi_Chr_6_7597902	Ca_desi_chr06	7597902	[A/G]	NA
Ca_Desi_Chr_6_7600172	Ca_desi_chr06	7600172	[T/C]	NA
Ca_Desi_Chr_6_7600182	Ca_desi_chr06	7600182	[T/C]	NA

SNP IDs	Chromosomes	Physical positions (bp)	SNPs	NCBI SNPdb accession IDs (NCBI_ss#)
Ca_Desi_Chr_6_7600190	Ca_desi_chr06	7600190	[A/G]	NA
Ca_Desi_Chr_6_7692170	Ca_desi_chr06	7692170	[A/C]	NA
Ca_Desi_Chr_6_7708553	Ca_desi_chr06	7708553	[T/C]	NA
Ca_Desi_Chr_6_7708557	Ca_desi_chr06	7708557	[A/C]	NA
Ca_Desi_Chr_6_7708561	Ca_desi_chr06	7708561	[T/C]	NA
Ca_Desi_Chr_6_7708581	Ca_desi_chr06	7708581	[T/C]	NA
Ca_Desi_Chr_6_7773260	Ca_desi_chr06	7773260	[T/C]	NA
Ca_Desi_Chr_6_7773315	Ca_desi_chr06	7773315	[A/C]	NA
Ca_Desi_Chr_6_7789032	Ca_desi_chr06	7789032	[G/T]	NA
Ca_Desi_Chr_6_7789059	Ca_desi_chr06	7789059	[G/A]	NA
Ca_Desi_Chr_6_7793211	Ca_desi_chr06	7793211	[T/A]	NA
Ca_Desi_Chr_6_7793288	Ca_desi_chr06	7793288	[G/A]	NA
Ca_Desi_Chr_6_7793325	Ca_desi_chr06	7793325	[A/G]	NA
Ca_Desi_Chr_6_7797752	Ca_desi_chr06	7797752	[T/G]	NA
Ca_Desi_Chr_6_7803317	Ca_desi_chr06	7803317	[C/A]	NA
Ca_Desi_Chr_6_7803318	Ca_desi_chr06	7803318	[T/G]	NA
Ca_Desi_Chr_6_7810436	Ca_desi_chr06	7810436	[A/G]	NA
Ca_Desi_Chr_6_7810471	Ca_desi_chr06	7810471	[A/T]	NA
Ca_Desi_Chr_6_7837771	Ca_desi_chr06	7837771	[T/G]	NA
Ca_Desi_Chr_6_7870799	Ca_desi_chr06	7870799	[A/G]	NA
Ca_Desi_Chr_6_7870804	Ca_desi_chr06	7870804	[T/G]	NA
Ca_Desi_Chr_6_7870807	Ca_desi_chr06	7870807	[T/G]	NA
Ca_Desi_Chr_6_7881974	Ca_desi_chr06	7881974	[C/T]	NA
Ca_Desi_Chr_6_7883724	Ca_desi_chr06	7883724	[T/C]	NA
Ca_Desi_Chr_6_7883744	Ca_desi_chr06	7883744	[T/G]	NA
Ca_Desi_Chr_6_7883764	Ca_desi_chr06	7883764	[G/A]	NA
Ca_Desi_Chr_6_7922447	Ca_desi_chr06	7922447	[A/G]	NA
Ca_Desi_Chr_6_7922471	Ca_desi_chr06	7922471	[T/C]	NA
Ca_Desi_Chr_6_7922480	Ca_desi_chr06	7922480	[A/G]	NA
Ca_Desi_Chr_6_7922498	Ca_desi_chr06	7922498	[T/C]	NA
Ca_Desi_Chr_6_7923548	Ca_desi_chr06	7923548	[T/C]	NA
Ca_Desi_Chr_6_7959093	Ca_desi_chr06	7959093	[T/C]	NA
Ca_Desi_Chr_6_7968802	Ca_desi_chr06	7968802	[C/T]	NA
Ca_Desi_Chr_6_7969718	Ca_desi_chr06	7969718	[G/A]	NA

SNP IDs	Chromosomes	Physical positions (bp)	SNPs	NCBI SNPdb accession IDs (NCBI_ss#)
Ca_Desi_Chr_6_7969721	Ca_desi_chr06	7969721	[T/G]	NA
Ca_Desi_Chr_6_7969725	Ca_desi_chr06	7969725	[A/T]	NA
Ca_Desi_Chr_6_8018663	Ca_desi_chr06	8018663	[A/C]	NA
Ca_Desi_Chr_6_8079590	Ca_desi_chr06	8079590	[G/T]	NA
Ca_Desi_Chr_6_8088093	Ca_desi_chr06	8088093	[T/G]	NA
Ca_Desi_Chr_6_8103858	Ca_desi_chr06	8103858	[A/G]	NA
Ca_Desi_Chr_6_8103869	Ca_desi_chr06	8103869	[A/G]	NA
Ca_Desi_Chr_6_8103873	Ca_desi_chr06	8103873	[A/C]	NA
Ca_Desi_Chr_6_8103884	Ca_desi_chr06	8103884	[A/C]	NA
Ca_Desi_Chr_6_8103906	Ca_desi_chr06	8103906	[A/G]	NA
Ca_Desi_Chr_6_8103908	Ca_desi_chr06	8103908	[A/G]	NA
Ca_Desi_Chr_6_8137948	Ca_desi_chr06	8137948	[A/C]	NA
Ca_Desi_Chr_6_8378481	Ca_desi_chr06	8378481	[T/G]	NA
Ca_Desi_Chr_6_8432509	Ca_desi_chr06	8432509	[A/C]	NA
Ca_Desi_Chr_6_8432517	Ca_desi_chr06	8432517	[T/C]	NA
Ca_Desi_Chr_6_8559933	Ca_desi_chr06	8559933	[A/C]	NA
Ca_Desi_Chr_6_8559941	Ca_desi_chr06	8559941	[A/G]	NA
Ca_Desi_Chr_6_8614241	Ca_desi_chr06	8614241	[A/C]	NA
Ca_Desi_Chr_6_8616283	Ca_desi_chr06	8616283	[T/G]	NA
Ca_Desi_Chr_6_8616287	Ca_desi_chr06	8616287	[T/G]	NA
Ca_Desi_Chr_6_8616299	Ca_desi_chr06	8616299	[T/G]	NA
Ca_Desi_Chr_6_8625356	Ca_desi_chr06	8625356	[T/G]	NA
Ca_Desi_Chr_6_8625462	Ca_desi_chr06	8625462	[A/G]	NA
Ca_Desi_Chr_6_8625560	Ca_desi_chr06	8625560	[T/G]	NA
Ca_Desi_Chr_6_8625563	Ca_desi_chr06	8625563	[T/G]	NA
Ca_Desi_Chr_6_8654704	Ca_desi_chr06	8654704	[T/C]	NA
Ca_Desi_Chr_6_8654755	Ca_desi_chr06	8654755	[A/G]	NA
Ca_Desi_Chr_6_8731132	Ca_desi_chr06	8731132	[T/G]	NA
Ca_Desi_Chr_6_8731136	Ca_desi_chr06	8731136	[C/T]	NA
Ca_Desi_Chr_6_8731142	Ca_desi_chr06	8731142	[C/T]	NA
Ca_Desi_Chr_6_8732387	Ca_desi_chr06	8732387	[G/A]	NA
Ca_Desi_Chr_6_8734991	Ca_desi_chr06	8734991	[A/T]	NA
Ca_Desi_Chr_6_8735019	Ca_desi_chr06	8735019	[A/C]	NA
Ca_Desi_Chr_6_8737776	Ca_desi_chr06	8737776	[A/C]	NA

SNP IDs	Chromosomes	Physical positions (bp)	SNPs	NCBI SNPdb accession IDs (NCBI_ss#)
Ca_Desi_Chr_6_8740748	Ca_desi_chr06	8740748	[T/A]	NA
Ca_Desi_Chr_6_8811914	Ca_desi_chr06	8811914	[C/A]	NA
Ca_Desi_Chr_6_8811915	Ca_desi_chr06	8811915	[C/G]	NA
Ca_Desi_Chr_6_8811916	Ca_desi_chr06	8811916	[T/A]	NA
Ca_Desi_Chr_6_9172228	Ca_desi_chr06	9172228	[T/G]	NA
Ca_Desi_Chr_6_9216531	Ca_desi_chr06	9216531	[A/C]	NA
Ca_Desi_Chr_6_9216534	Ca_desi_chr06	9216534	[C/T]	NA
Ca_Desi_Chr_6_9228751	Ca_desi_chr06	9228751	[T/G]	NA
Ca_Desi_Chr_6_9228852	Ca_desi_chr06	9228852	[A/T]	NA
Ca_Desi_Chr_6_9245293	Ca_desi_chr06	9245293	[T/G]	NA
Ca_Desi_Chr_6_9245317	Ca_desi_chr06	9245317	[T/G]	NA
Ca_Desi_Chr_6_9245321	Ca_desi_chr06	9245321	[A/C]	NA
Ca_Desi_Chr_6_9250216	Ca_desi_chr06	9250216	[A/G]	NA
Ca_Desi_Chr_6_9253782	Ca_desi_chr06	9253782	[T/A]	NA
Ca_Desi_Chr_6_9340323	Ca_desi_chr06	9340323	[A/C]	NA
Ca_Desi_Chr_6_9379394	Ca_desi_chr06	9379394	[A/C]	NA
Ca_Desi_Chr_6_9384087	Ca_desi_chr06	9384087	[A/C]	NA
Ca_Desi_Chr_6_9384090	Ca_desi_chr06	9384090	[A/C]	NA
Ca_Desi_Chr_6_9391584	Ca_desi_chr06	9391584	[A/G]	NA
Ca_Desi_Chr_6_9463915	Ca_desi_chr06	9463915	[G/A]	NA
Ca_Desi_Chr_6_9463929	Ca_desi_chr06	9463929	[G/A]	NA
Ca_Desi_Chr_6_9463931	Ca_desi_chr06	9463931	[A/G]	NA
Ca_Desi_Chr_6_9507956	Ca_desi_chr06	9507956	[T/C]	NA
Ca_Desi_Chr_6_9507961	Ca_desi_chr06	9507961	[G/A]	NA
Ca_Desi_Chr_6_9507965	Ca_desi_chr06	9507965	[T/C]	NA
Ca_Desi_Chr_6_9507984	Ca_desi_chr06	9507984	[A/T]	NA
Ca_Desi_Chr_6_9507988	Ca_desi_chr06	9507988	[T/C]	NA
Ca_Desi_Chr_6_9507994	Ca_desi_chr06	9507994	[T/C]	NA
Ca_Desi_Chr_6_9508004	Ca_desi_chr06	9508004	[A/G]	NA
Ca_Desi_Chr_6_9522938	Ca_desi_chr06	9522938	[A/C]	NA
Ca_Desi_Chr_6_9522973	Ca_desi_chr06	9522973	[A/C]	NA
Ca_Desi_Chr_6_9523000	Ca_desi_chr06	9523000	[A/T]	NA
Ca_Desi_Chr_6_9523032	Ca_desi_chr06	9523032	[T/C]	NA
Ca_Desi_Chr_6_9523046	Ca_desi_chr06	9523046	[T/C]	NA

SNP IDs	Chromosomes	Physical positions (bp)	SNPs	NCBI SNPdb accession IDs (NCBI_ss#)
Ca_Desi_Chr_6_9619281	Ca_desi_chr06	9619281	[C/A]	NA
Ca_Desi_Chr_6_9619282	Ca_desi_chr06	9619282	[C/G]	NA
Ca_Desi_Chr_6_9619283	Ca_desi_chr06	9619283	[C/A]	NA
Ca_Desi_Chr_6_9628721	Ca_desi_chr06	9628721	[T/C]	NA
Ca_Desi_Chr_6_9663889	Ca_desi_chr06	9663889	[A/C]	NA
Ca_Desi_Chr_6_9691297	Ca_desi_chr06	9691297	[A/G]	NA
Ca_Desi_Chr_6_9691307	Ca_desi_chr06	9691307	[A/G]	NA
Ca_Desi_Chr_6_9691317	Ca_desi_chr06	9691317	[T/G]	NA
Ca_Desi_Chr_6_9691346	Ca_desi_chr06	9691346	[A/G]	NA
Ca_Desi_Chr_6_9691349	Ca_desi_chr06	9691349	[T/G]	NA
Ca_Desi_Chr_6_9696444	Ca_desi_chr06	9696444	[T/C]	NA
Ca_Desi_Chr_6_9696459	Ca_desi_chr06	9696459	[A/C]	NA
Ca_Desi_Chr_6_9728076	Ca_desi_chr06	9728076	[A/G]	NA
Ca_Desi_Chr_6_9728107	Ca_desi_chr06	9728107	[A/G]	NA
Ca_Desi_Chr_6_9728112	Ca_desi_chr06	9728112	[T/G]	NA
Ca_Desi_Chr_6_9728119	Ca_desi_chr06	9728119	[A/G]	NA
Ca_Desi_Chr_6_9728131	Ca_desi_chr06	9728131	[A/G]	NA
Ca_Desi_Chr_6_9728137	Ca_desi_chr06	9728137	[A/G]	NA
Ca_Desi_Chr_6_9728147	Ca_desi_chr06	9728147	[A/G]	NA
Ca_Desi_Chr_6_9728153	Ca_desi_chr06	9728153	[A/G]	NA
Ca_Desi_Chr_6_9733693	Ca_desi_chr06	9733693	[T/G]	NA
Ca_Desi_Chr_6_9761314	Ca_desi_chr06	9761314	[T/G]	NA
Ca_Desi_Chr_6_9781707	Ca_desi_chr06	9781707	[A/G]	NA
Ca_Desi_Chr_6_9781713	Ca_desi_chr06	9781713	[T/C]	NA
Ca_Desi_Chr_6_9781724	Ca_desi_chr06	9781724	[T/C]	NA
Ca_Desi_Chr_6_9781737	Ca_desi_chr06	9781737	[T/C]	NA
Ca_Desi_Chr_6_9781748	Ca_desi_chr06	9781748	[T/C]	NA
Ca_Desi_Chr_6_9781757	Ca_desi_chr06	9781757	[A/G]	NA
Ca_Desi_Chr_6_9781761	Ca_desi_chr06	9781761	[T/C]	NA
Ca_Desi_Chr_6_9781778	Ca_desi_chr06	9781778	[T/C]	NA
Ca_Desi_Chr_6_9897705	Ca_desi_chr06	9897705	[T/A]	NA
Ca_Desi_Chr_6_9899089	Ca_desi_chr06	9899089	[A/G]	NA
Ca_Desi_Chr_6_9904506	Ca_desi_chr06	9904506	[A/C]	NA
Ca_Desi_Chr_6_9904514	Ca_desi_chr06	9904514	[G/T]	NA

SNP IDs	Chromosomes	Physical positions (bp)	SNPs	NCBI SNPdb accession IDs (NCBI_ss#)
Ca_Desi_Chr_6_9904519	Ca_desi_chr06	9904519	[A/C]	NA
Ca_Desi_Chr_6_9904522	Ca_desi_chr06	9904522	[T/C]	NA
Ca_Desi_Chr_6_9904525	Ca_desi_chr06	9904525	[C/T]	NA
Ca_Desi_Chr_6_9904526	Ca_desi_chr06	9904526	[A/C]	NA
Ca_Desi_Chr_6_9904529	Ca_desi_chr06	9904529	[G/T]	NA
Ca_Desi_Chr_6_9904537	Ca_desi_chr06	9904537	[G/T]	NA
Ca_Desi_Chr_6_9904548	Ca_desi_chr06	9904548	[A/G]	NA
Ca_Desi_Chr_6_9980886	Ca_desi_chr06	9980886	[A/G]	NA
Ca_Desi_Chr_6_9980900	Ca_desi_chr06	9980900	[T/A]	NA
Ca_Desi_Chr_6_9980909	Ca_desi_chr06	9980909	[T/G]	NA
Ca_Desi_Chr_6_9992247	Ca_desi_chr06	9992247	[A/C]	NA
Ca_Desi_Chr_6_10095710	Ca_desi_chr06	10095710	[T/G]	NA
Ca_Desi_Chr_6_10123319	Ca_desi_chr06	10123319	[A/C]	NA
Ca_Desi_Chr_6_10125156	Ca_desi_chr06	10125156	[A/G]	NA
Ca_Desi_Chr_6_10167624	Ca_desi_chr06	10167624	[T/G]	NA
Ca_Desi_Chr_6_10167633	Ca_desi_chr06	10167633	[A/C]	NA
Ca_Desi_Chr_6_10167658	Ca_desi_chr06	10167658	[A/G]	NA
Ca_Desi_Chr_6_10253611	Ca_desi_chr06	10253611	[A/G]	NA
Ca_Desi_Chr_6_10266783	Ca_desi_chr06	10266783	[A/G]	NA
Ca_Desi_Chr_6_10266790	Ca_desi_chr06	10266790	[A/G]	NA
Ca_Desi_Chr_6_10266792	Ca_desi_chr06	10266792	[A/C]	NA
Ca_Desi_Chr_6_10266794	Ca_desi_chr06	10266794	[T/C]	NA
Ca_Desi_Chr_6_10266813	Ca_desi_chr06	10266813	[A/G]	NA
Ca_Desi_Chr_6_10266814	Ca_desi_chr06	10266814	[T/A]	NA
Ca_Desi_Chr_6_10266828	Ca_desi_chr06	10266828	[A/G]	NA
Ca_Desi_Chr_6_10269558	Ca_desi_chr06	10269558	[A/G]	NA
Ca_Desi_Chr_6_10269568	Ca_desi_chr06	10269568	[A/G]	NA
Ca_Desi_Chr_6_10269573	Ca_desi_chr06	10269573	[A/C]	NA
Ca_Desi_Chr_6_10269582	Ca_desi_chr06	10269582	[A/G]	NA
Ca_Desi_Chr_6_10269591	Ca_desi_chr06	10269591	[A/G]	NA
Ca_Desi_Chr_6_10269598	Ca_desi_chr06	10269598	[A/G]	NA
Ca_Desi_Chr_6_10289062	Ca_desi_chr06	10289062	[T/C]	NA
Ca_Desi_Chr_6_10304722	Ca_desi_chr06	10304722	[C/A]	NA
Ca_Desi_Chr_6_10304723	Ca_desi_chr06	10304723	[T/G]	NA

SNP IDs	Chromosomes	Physical positions (bp)	SNPs	NCBI SNPdb accession IDs (NCBI_ss#)
Ca_Desi_Chr_6_10304724	Ca_desi_chr06	10304724	[G/A]	NA
Ca_Desi_Chr_6_10304725	Ca_desi_chr06	10304725	[A/T]	NA
Ca_Desi_Chr_6_10326027	Ca_desi_chr06	10326027	[T/C]	NA
Ca_Desi_Chr_6_10334639	Ca_desi_chr06	10334639	[A/C]	NA
Ca_Desi_Chr_6_10334716	Ca_desi_chr06	10334716	[A/C]	NA
Ca_Desi_Chr_6_10334724	Ca_desi_chr06	10334724	[A/C]	NA
Ca_Desi_Chr_6_10338751	Ca_desi_chr06	10338751	[T/C]	NA
Ca_Desi_Chr_6_10338768	Ca_desi_chr06	10338768	[A/C]	NA
Ca_Desi_Chr_6_10338779	Ca_desi_chr06	10338779	[A/T]	NA
Ca_Desi_Chr_6_10358300	Ca_desi_chr06	10358300	[G/A]	NA
Ca_Desi_Chr_6_10358317	Ca_desi_chr06	10358317	[A/G]	NA
Ca_Desi_Chr_6_10358319	Ca_desi_chr06	10358319	[G/T]	NA
Ca_Desi_Chr_6_10358320	Ca_desi_chr06	10358320	[A/G]	NA
Ca_Desi_Chr_6_10358350	Ca_desi_chr06	10358350	[G/A]	NA
Ca_Desi_Chr_6_10366655	Ca_desi_chr06	10366655	[G/T]	NA
Ca_Desi_Chr_6_10366663	Ca_desi_chr06	10366663	[T/C]	NA
Ca_Desi_Chr_6_10461640	Ca_desi_chr06	10461640	[A/T]	NA
Ca_Desi_Chr_6_10461644	Ca_desi_chr06	10461644	[A/G]	NA
Ca_Desi_Chr_6_10461649	Ca_desi_chr06	10461649	[A/G]	NA
Ca_Desi_Chr_6_10461651	Ca_desi_chr06	10461651	[T/G]	NA
Ca_Desi_Chr_6_10461656	Ca_desi_chr06	10461656	[T/G]	NA
Ca_Desi_Chr_6_10461657	Ca_desi_chr06	10461657	[A/C]	NA
Ca_Desi_Chr_6_10461666	Ca_desi_chr06	10461666	[T/C]	NA
Ca_Desi_Chr_6_10461673	Ca_desi_chr06	10461673	[T/C]	NA
Ca_Desi_Chr_6_10461678	Ca_desi_chr06	10461678	[T/C]	NA
Ca_Desi_Chr_6_10461680	Ca_desi_chr06	10461680	[T/C]	NA
Ca_Desi_Chr_6_10461683	Ca_desi_chr06	10461683	[T/C]	NA
Ca_Desi_Chr_6_10461693	Ca_desi_chr06	10461693	[C/G]	NA
Ca_Desi_Chr_6_10461696	Ca_desi_chr06	10461696	[T/A]	NA
Ca_Desi_Chr_6_10461741	Ca_desi_chr06	10461741	[T/G]	NA
Ca_Desi_Chr_6_10461749	Ca_desi_chr06	10461749	[T/G]	NA
Ca_Desi_Chr_6_10461760	Ca_desi_chr06	10461760	[A/G]	NA
Ca_Desi_Chr_6_10473079	Ca_desi_chr06	10473079	[T/G]	NA
Ca_Desi_Chr_6_10473090	Ca_desi_chr06	10473090	[A/G]	NA

SNP IDs	Chromosomes	Physical positions (bp)	SNPs	NCBI SNPdb accession IDs (NCBI_ss#)
Ca_Desi_Chr_6_10473095	Ca_desi_chr06	10473095	[T/G]	NA
Ca_Desi_Chr_6_10473106	Ca_desi_chr06	10473106	[A/G]	NA
Ca_Desi_Chr_6_10473110	Ca_desi_chr06	10473110	[T/G]	NA
Ca_Desi_Chr_6_10473112	Ca_desi_chr06	10473112	[T/G]	NA
Ca_Desi_Chr_6_10509047	Ca_desi_chr06	10509047	[A/T]	NA
Ca_Desi_Chr_6_10574749	Ca_desi_chr06	10574749	[T/G]	NA
Ca_Desi_Chr_6_10575305	Ca_desi_chr06	10575305	[T/A]	NA
Ca_Desi_Chr_6_10575316	Ca_desi_chr06	10575316	[T/C]	NA
Ca_Desi_Chr_6_10596426	Ca_desi_chr06	10596426	[A/G]	NA
Ca_Desi_Chr_6_10596432	Ca_desi_chr06	10596432	[A/C]	NA
Ca_Desi_Chr_6_10596451	Ca_desi_chr06	10596451	[T/C]	NA
Ca_Desi_Chr_6_10596455	Ca_desi_chr06	10596455	[T/A]	NA
Ca_Desi_Chr_6_10596504	Ca_desi_chr06	10596504	[T/G]	NA
Ca_Desi_Chr_6_10662694	Ca_desi_chr06	10662694	[A/C]	NA
Ca_Desi_Chr_6_10662695	Ca_desi_chr06	10662695	[C/A]	NA
Ca_Desi_Chr_6_10662697	Ca_desi_chr06	10662697	[T/G]	NA
Ca_Desi_Chr_6_10662707	Ca_desi_chr06	10662707	[A/G]	NA
Ca_Desi_Chr_6_10662716	Ca_desi_chr06	10662716	[T/C]	NA
Ca_Desi_Chr_6_10662891	Ca_desi_chr06	10662891	[T/C]	NA
Ca_Desi_Chr_6_10662892	Ca_desi_chr06	10662892	[G/T]	NA
Ca_Desi_Chr_6_10710378	Ca_desi_chr06	10710378	[A/C]	NA
Ca_Desi_Chr_6_10710381	Ca_desi_chr06	10710381	[T/C]	NA
Ca_Desi_Chr_6_10710384	Ca_desi_chr06	10710384	[A/C]	NA
Ca_Desi_Chr_6_10710396	Ca_desi_chr06	10710396	[A/T]	NA
Ca_Desi_Chr_6_10712288	Ca_desi_chr06	10712288	[T/G]	NA
Ca_Desi_Chr_6_10778151	Ca_desi_chr06	10778151	[A/G]	NA
Ca_Desi_Chr_6_10830011	Ca_desi_chr06	10830011	[A/C]	NA
Ca_Desi_Chr_6_10888437	Ca_desi_chr06	10888437	[A/G]	NA
Ca_Desi_Chr_6_10940503	Ca_desi_chr06	10940503	[T/C]	NA
Ca_Desi_Chr_6_10967359	Ca_desi_chr06	10967359	[T/G]	NA
Ca_Desi_Chr_6_11029177	Ca_desi_chr06	11029177	[A/G]	NA
Ca_Desi_Chr_6_11029213	Ca_desi_chr06	11029213	[T/A]	NA
Ca_Desi_Chr_6_11029244	Ca_desi_chr06	11029244	[T/G]	NA
Ca_Desi_Chr_6_11029257	Ca_desi_chr06	11029257	[A/C]	NA

SNP IDs	Chromosomes	Physical positions (bp)	SNPs	NCBI SNPdb accession IDs (NCBI_ss#)
Ca_Desi_Chr_6_11176033	Ca_desi_chr06	11176033	[A/T]	NA
Ca_Desi_Chr_6_11299905	Ca_desi_chr06	11299905	[A/G]	NA
Ca_Desi_Chr_6_11299919	Ca_desi_chr06	11299919	[T/C]	NA
Ca_Desi_Chr_6_11299937	Ca_desi_chr06	11299937	[T/G]	NA
Ca_Desi_Chr_6_11299947	Ca_desi_chr06	11299947	[A/T]	NA
Ca_Desi_Chr_6_11319790	Ca_desi_chr06	11319790	[T/G]	NA
Ca_Desi_Chr_6_11341974	Ca_desi_chr06	11341974	[G/T]	NA
Ca_Desi_Chr_6_11341975	Ca_desi_chr06	11341975	[T/G]	NA
Ca_Desi_Chr_6_11350097	Ca_desi_chr06	11350097	[A/C]	NA
Ca_Desi_Chr_6_11414655	Ca_desi_chr06	11414655	[A/C]	NA
Ca_Desi_Chr_6_11453287	Ca_desi_chr06	11453287	[T/C]	NA
Ca_Desi_Chr_6_11453341	Ca_desi_chr06	11453341	[T/G]	NA
Ca_Desi_Chr_6_11453369	Ca_desi_chr06	11453369	[A/G]	NA
Ca_Desi_Chr_6_11476473	Ca_desi_chr06	11476473	[A/C]	NA
Ca_Desi_Chr_6_11476765	Ca_desi_chr06	11476765	[A/C]	NA
Ca_Desi_Chr_6_11476768	Ca_desi_chr06	11476768	[T/C]	NA
Ca_Desi_Chr_6_11476770	Ca_desi_chr06	11476770	[T/C]	NA
Ca_Desi_Chr_6_11477655	Ca_desi_chr06	11477655	[A/G]	NA
Ca_Desi_Chr_6_11477693	Ca_desi_chr06	11477693	[T/C]	NA
Ca_Desi_Chr_6_11477727	Ca_desi_chr06	11477727	[T/C]	NA
Ca_Desi_Chr_6_11477753	Ca_desi_chr06	11477753	[T/C]	NA
Ca_Desi_Chr_7_2258	Ca_desi_chr07	2258	[T/G]	NA
Ca_Desi_Chr_7_62005	Ca_desi_chr07	62005	[T/C]	NA
Ca_Desi_Chr_7_62011	Ca_desi_chr07	62011	[A/C]	NA
Ca_Desi_Chr_7_62027	Ca_desi_chr07	62027	[T/C]	NA
Ca_Desi_Chr_7_62038	Ca_desi_chr07	62038	[T/C]	NA
Ca_Desi_Chr_7_62045	Ca_desi_chr07	62045	[A/G]	NA
Ca_Desi_Chr_7_62054	Ca_desi_chr07	62054	[T/G]	NA
Ca_Desi_Chr_7_62059	Ca_desi_chr07	62059	[T/C]	NA
Ca_Desi_Chr_7_174177	Ca_desi_chr07	174177	[T/C]	NA
Ca_Desi_Chr_7_174182	Ca_desi_chr07	174182	[T/C]	NA
Ca_Desi_Chr_7_174191	Ca_desi_chr07	174191	[T/C]	NA
Ca_Desi_Chr_7_174201	Ca_desi_chr07	174201	[A/G]	NA
Ca_Desi_Chr_7_174203	Ca_desi_chr07	174203	[C/G]	NA

SNP IDs	Chromosomes	Physical positions (bp)	SNPs	NCBI SNPdb accession IDs (NCBI_ss#)
Ca_Desi_Chr_7_174205	Ca_desi_chr07	174205	[T/A]	NA
Ca_Desi_Chr_7_174207	Ca_desi_chr07	174207	[G/T]	NA
Ca_Desi_Chr_7_174208	Ca_desi_chr07	174208	[C/G]	NA
Ca_Desi_Chr_7_174211	Ca_desi_chr07	174211	[A/G]	NA
Ca_Desi_Chr_7_174213	Ca_desi_chr07	174213	[T/C]	NA
Ca_Desi_Chr_7_174215	Ca_desi_chr07	174215	[G/T]	NA
Ca_Desi_Chr_7_174216	Ca_desi_chr07	174216	[A/G]	NA
Ca_Desi_Chr_7_174218	Ca_desi_chr07	174218	[T/C]	NA
Ca_Desi_Chr_7_174222	Ca_desi_chr07	174222	[A/G]	NA
Ca_Desi_Chr_7_174235	Ca_desi_chr07	174235	[A/G]	NA
Ca_Desi_Chr_7_174237	Ca_desi_chr07	174237	[A/G]	NA
Ca_Desi_Chr_7_174245	Ca_desi_chr07	174245	[A/G]	NA
Ca_Desi_Chr_7_174247	Ca_desi_chr07	174247	[A/G]	NA
Ca_Desi_Chr_7_175030	Ca_desi_chr07	175030	[A/G]	NA
Ca_Desi_Chr_7_175101	Ca_desi_chr07	175101	[A/C]	NA
Ca_Desi_Chr_7_178592	Ca_desi_chr07	178592	[A/C]	NA
Ca_Desi_Chr_7_182411	Ca_desi_chr07	182411	[T/C]	NA
Ca_Desi_Chr_7_182440	Ca_desi_chr07	182440	[T/G]	NA
Ca_Desi_Chr_7_218804	Ca_desi_chr07	218804	[A/G]	NA
Ca_Desi_Chr_7_327863	Ca_desi_chr07	327863	[T/C]	NA
Ca_Desi_Chr_7_339816	Ca_desi_chr07	339816	[A/C]	NA
Ca_Desi_Chr_7_339857	Ca_desi_chr07	339857	[T/G]	NA
Ca_Desi_Chr_7_350165	Ca_desi_chr07	350165	[A/C]	NA
Ca_Desi_Chr_7_360893	Ca_desi_chr07	360893	[A/C]	NA
Ca_Desi_Chr_7_386824	Ca_desi_chr07	386824	[A/C]	NA
Ca_Desi_Chr_7_398327	Ca_desi_chr07	398327	[G/T]	NA
Ca_Desi_Chr_7_413090	Ca_desi_chr07	413090	[A/C]	NA
Ca_Desi_Chr_7_428174	Ca_desi_chr07	428174	[A/T]	NA
Ca_Desi_Chr_7_428191	Ca_desi_chr07	428191	[G/A]	NA
Ca_Desi_Chr_7_429544	Ca_desi_chr07	429544	[A/C]	NA
Ca_Desi_Chr_7_463991	Ca_desi_chr07	463991	[G/A]	NA
Ca_Desi_Chr_7_463993	Ca_desi_chr07	463993	[A/C]	NA
Ca_Desi_Chr_7_463994	Ca_desi_chr07	463994	[G/T]	NA
Ca_Desi_Chr_7_575957	Ca_desi_chr07	575957	[T/A]	NA

SNP IDs	Chromosomes	Physical positions (bp)	SNPs	NCBI SNPdb accession IDs (NCBI_ss#)
Ca_Desi_Chr_7_640083	Ca_desi_chr07	640083	[T/G]	NA
Ca_Desi_Chr_7_694652	Ca_desi_chr07	694652	[A/T]	NA
Ca_Desi_Chr_7_694675	Ca_desi_chr07	694675	[G/T]	NA
Ca_Desi_Chr_7_735745	Ca_desi_chr07	735745	[T/G]	NA
Ca_Desi_Chr_7_735747	Ca_desi_chr07	735747	[T/G]	NA
Ca_Desi_Chr_7_747310	Ca_desi_chr07	747310	[A/C]	NA
Ca_Desi_Chr_7_754195	Ca_desi_chr07	754195	[G/A]	NA
Ca_Desi_Chr_7_754198	Ca_desi_chr07	754198	[A/G]	NA
Ca_Desi_Chr_7_754201	Ca_desi_chr07	754201	[G/T]	NA
Ca_Desi_Chr_7_770276	Ca_desi_chr07	770276	[A/G]	NA
Ca_Desi_Chr_7_770323	Ca_desi_chr07	770323	[C/G]	NA
Ca_Desi_Chr_7_789702	Ca_desi_chr07	789702	[A/G]	NA
Ca_Desi_Chr_7_789707	Ca_desi_chr07	789707	[A/G]	NA
Ca_Desi_Chr_7_789718	Ca_desi_chr07	789718	[T/G]	NA
Ca_Desi_Chr_7_789737	Ca_desi_chr07	789737	[A/G]	NA
Ca_Desi_Chr_7_789741	Ca_desi_chr07	789741	[T/C]	NA
Ca_Desi_Chr_7_789745	Ca_desi_chr07	789745	[A/C]	NA
Ca_Desi_Chr_7_802095	Ca_desi_chr07	802095	[G/C]	NA
Ca_Desi_Chr_7_984620	Ca_desi_chr07	984620	[A/C]	NA
Ca_Desi_Chr_7_984634	Ca_desi_chr07	984634	[T/C]	NA
Ca_Desi_Chr_7_1012489	Ca_desi_chr07	1012489	[T/C]	NA
Ca_Desi_Chr_7_1012502	Ca_desi_chr07	1012502	[A/C]	NA
Ca_Desi_Chr_7_1015717	Ca_desi_chr07	1015717	[T/G]	NA
Ca_Desi_Chr_7_1029913	Ca_desi_chr07	1029913	[A/T]	NA
Ca_Desi_Chr_7_1050537	Ca_desi_chr07	1050537	[G/T]	NA
Ca_Desi_Chr_7_1050538	Ca_desi_chr07	1050538	[C/T]	NA
Ca_Desi_Chr_7_1050539	Ca_desi_chr07	1050539	[A/T]	NA
Ca_Desi_Chr_7_1050542	Ca_desi_chr07	1050542	[A/T]	NA
Ca_Desi_Chr_7_1413096	Ca_desi_chr07	1413096	[A/C]	NA
Ca_Desi_Chr_7_1414414	Ca_desi_chr07	1414414	[T/G]	NA
Ca_Desi_Chr_7_1414430	Ca_desi_chr07	1414430	[A/C]	NA
Ca_Desi_Chr_7_1414960	Ca_desi_chr07	1414960	[T/C]	NA
Ca_Desi_Chr_7_1415415	Ca_desi_chr07	1415415	[A/T]	NA
Ca_Desi_Chr_7_1418438	Ca_desi_chr07	1418438	[A/C]	NA

SNP IDs	Chromosomes	Physical positions (bp)	SNPs	NCBI SNPdb accession IDs (NCBI_ss#)
Ca_Desi_Chr_7_1418441	Ca_desi_chr07	1418441	[A/C]	NA
Ca_Desi_Chr_7_1418447	Ca_desi_chr07	1418447	[T/C]	NA
Ca_Desi_Chr_7_1441106	Ca_desi_chr07	1441106	[C/A]	NA
Ca_Desi_Chr_7_1441778	Ca_desi_chr07	1441778	[G/A]	NA
Ca_Desi_Chr_7_1443635	Ca_desi_chr07	1443635	[A/C]	NA
Ca_Desi_Chr_7_1443656	Ca_desi_chr07	1443656	[T/C]	NA
Ca_Desi_Chr_7_1443801	Ca_desi_chr07	1443801	[A/T]	NA
Ca_Desi_Chr_7_1443823	Ca_desi_chr07	1443823	[A/G]	NA
Ca_Desi_Chr_7_1443839	Ca_desi_chr07	1443839	[T/C]	NA
Ca_Desi_Chr_7_1443864	Ca_desi_chr07	1443864	[C/A]	NA
Ca_Desi_Chr_7_1443867	Ca_desi_chr07	1443867	[A/T]	NA
Ca_Desi_Chr_7_1443869	Ca_desi_chr07	1443869	[G/C]	NA
Ca_Desi_Chr_7_1443872	Ca_desi_chr07	1443872	[A/C]	NA
Ca_Desi_Chr_7_1443875	Ca_desi_chr07	1443875	[G/C]	NA
Ca_Desi_Chr_7_1443877	Ca_desi_chr07	1443877	[A/T]	NA
Ca_Desi_Chr_7_1443880	Ca_desi_chr07	1443880	[T/G]	NA
Ca_Desi_Chr_7_1443882	Ca_desi_chr07	1443882	[G/C]	NA
Ca_Desi_Chr_7_1443904	Ca_desi_chr07	1443904	[T/G]	NA
Ca_Desi_Chr_7_1443906	Ca_desi_chr07	1443906	[C/T]	NA
Ca_Desi_Chr_7_1444385	Ca_desi_chr07	1444385	[G/A]	NA
Ca_Desi_Chr_7_1444388	Ca_desi_chr07	1444388	[C/T]	NA
Ca_Desi_Chr_7_1508084	Ca_desi_chr07	1508084	[T/C]	NA
Ca_Desi_Chr_7_1530481	Ca_desi_chr07	1530481	[A/C]	NA
Ca_Desi_Chr_7_1541580	Ca_desi_chr07	1541580	[A/T]	NA
Ca_Desi_Chr_7_1541639	Ca_desi_chr07	1541639	[T/G]	NA
Ca_Desi_Chr_7_1541646	Ca_desi_chr07	1541646	[C/G]	NA
Ca_Desi_Chr_7_1558730	Ca_desi_chr07	1558730	[T/G]	NA
Ca_Desi_Chr_7_1558732	Ca_desi_chr07	1558732	[T/G]	NA
Ca_Desi_Chr_7_1558734	Ca_desi_chr07	1558734	[T/G]	NA
Ca_Desi_Chr_7_1558737	Ca_desi_chr07	1558737	[T/A]	NA
Ca_Desi_Chr_7_1558739	Ca_desi_chr07	1558739	[A/T]	NA
Ca_Desi_Chr_7_1558747	Ca_desi_chr07	1558747	[T/G]	NA
Ca_Desi_Chr_7_1566076	Ca_desi_chr07	1566076	[T/G]	NA
Ca_Desi_Chr_7_1573638	Ca_desi_chr07	1573638	[T/C]	NA

SNP IDs	Chromosomes	Physical positions (bp)	SNPs	NCBI SNPdb accession IDs (NCBI_ss#)
Ca_Desi_Chr_7_1573654	Ca_desi_chr07	1573654	[A/G]	NA
Ca_Desi_Chr_7_1573659	Ca_desi_chr07	1573659	[T/G]	NA
Ca_Desi_Chr_7_1573745	Ca_desi_chr07	1573745	[T/G]	NA
Ca_Desi_Chr_7_1579057	Ca_desi_chr07	1579057	[A/C]	NA
Ca_Desi_Chr_7_1583764	Ca_desi_chr07	1583764	[T/C]	NA
Ca_Desi_Chr_7_1583771	Ca_desi_chr07	1583771	[T/G]	NA
Ca_Desi_Chr_7_1583778	Ca_desi_chr07	1583778	[T/G]	NA
Ca_Desi_Chr_7_1619995	Ca_desi_chr07	1619995	[A/C]	NA
Ca_Desi_Chr_7_1630848	Ca_desi_chr07	1630848	[A/G]	NA
Ca_Desi_Chr_7_1630861	Ca_desi_chr07	1630861	[C/A]	NA
Ca_Desi_Chr_7_1631320	Ca_desi_chr07	1631320	[G/A]	NA
Ca_Desi_Chr_7_1631428	Ca_desi_chr07	1631428	[T/C]	NA
Ca_Desi_Chr_7_1631890	Ca_desi_chr07	1631890	[T/C]	NA
Ca_Desi_Chr_7_1634725	Ca_desi_chr07	1634725	[T/G]	NA
Ca_Desi_Chr_7_1634726	Ca_desi_chr07	1634726	[C/G]	NA
Ca_Desi_Chr_7_1634727	Ca_desi_chr07	1634727	[T/C]	NA
Ca_Desi_Chr_7_1634728	Ca_desi_chr07	1634728	[T/C]	NA
Ca_Desi_Chr_7_1634729	Ca_desi_chr07	1634729	[T/A]	NA
Ca_Desi_Chr_7_1634730	Ca_desi_chr07	1634730	[A/T]	NA
Ca_Desi_Chr_7_1634790	Ca_desi_chr07	1634790	[A/G]	NA
Ca_Desi_Chr_7_1634992	Ca_desi_chr07	1634992	[T/G]	NA
Ca_Desi_Chr_7_1635022	Ca_desi_chr07	1635022	[A/G]	NA
Ca_Desi_Chr_7_1635054	Ca_desi_chr07	1635054	[A/G]	NA
Ca_Desi_Chr_7_1635068	Ca_desi_chr07	1635068	[C/T]	NA
Ca_Desi_Chr_7_1668089	Ca_desi_chr07	1668089	[T/C]	NA
Ca_Desi_Chr_7_1668112	Ca_desi_chr07	1668112	[C/T]	NA
Ca_Desi_Chr_7_1744061	Ca_desi_chr07	1744061	[A/C]	NA
Ca_Desi_Chr_7_1760104	Ca_desi_chr07	1760104	[T/G]	NA
Ca_Desi_Chr_7_1760157	Ca_desi_chr07	1760157	[A/G]	NA
Ca_Desi_Chr_7_1761375	Ca_desi_chr07	1761375	[G/A]	NA
Ca_Desi_Chr_7_1761414	Ca_desi_chr07	1761414	[G/A]	NA
Ca_Desi_Chr_7_1763162	Ca_desi_chr07	1763162	[A/C]	NA
Ca_Desi_Chr_7_1828880	Ca_desi_chr07	1828880	[T/G]	NA
Ca_Desi_Chr_7_1828967	Ca_desi_chr07	1828967	[A/T]	NA

SNP IDs	Chromosomes	Physical positions (bp)	SNPs	NCBI SNPdb accession IDs (NCBI_ss#)
Ca_Desi_Chr_7_1829019	Ca_desi_chr07	1829019	[A/T]	NA
Ca_Desi_Chr_7_1829034	Ca_desi_chr07	1829034	[T/C]	NA
Ca_Desi_Chr_7_1829055	Ca_desi_chr07	1829055	[A/G]	NA
Ca_Desi_Chr_7_1829061	Ca_desi_chr07	1829061	[A/G]	NA
Ca_Desi_Chr_7_1832858	Ca_desi_chr07	1832858	[A/T]	NA
Ca_Desi_Chr_7_1875982	Ca_desi_chr07	1875982	[T/G]	NA
Ca_Desi_Chr_7_1875998	Ca_desi_chr07	1875998	[A/C]	NA
Ca_Desi_Chr_7_1883010	Ca_desi_chr07	1883010	[T/A]	NA
Ca_Desi_Chr_7_2000373	Ca_desi_chr07	2000373	[A/G]	NA
Ca_Desi_Chr_7_2000399	Ca_desi_chr07	2000399	[T/G]	NA
Ca_Desi_Chr_7_2018106	Ca_desi_chr07	2018106	[T/C]	NA
Ca_Desi_Chr_7_2018747	Ca_desi_chr07	2018747	[T/G]	NA
Ca_Desi_Chr_7_2019144	Ca_desi_chr07	2019144	[T/A]	NA
Ca_Desi_Chr_7_2036771	Ca_desi_chr07	2036771	[C/T]	NA
Ca_Desi_Chr_7_2047543	Ca_desi_chr07	2047543	[G/A]	NA
Ca_Desi_Chr_7_2051090	Ca_desi_chr07	2051090	[T/G]	NA
Ca_Desi_Chr_7_2051208	Ca_desi_chr07	2051208	[A/C]	NA
Ca_Desi_Chr_7_2127195	Ca_desi_chr07	2127195	[C/A]	NA
Ca_Desi_Chr_7_2127196	Ca_desi_chr07	2127196	[C/G]	NA
Ca_Desi_Chr_7_2127197	Ca_desi_chr07	2127197	[G/A]	NA
Ca_Desi_Chr_7_2127198	Ca_desi_chr07	2127198	[G/T]	NA
Ca_Desi_Chr_7_2183490	Ca_desi_chr07	2183490	[T/C]	NA
Ca_Desi_Chr_7_2183574	Ca_desi_chr07	2183574	[A/C]	NA
Ca_Desi_Chr_7_2183597	Ca_desi_chr07	2183597	[A/C]	NA
Ca_Desi_Chr_7_2183627	Ca_desi_chr07	2183627	[A/T]	NA
Ca_Desi_Chr_7_2195618	Ca_desi_chr07	2195618	[T/C]	NA
Ca_Desi_Chr_7_2195622	Ca_desi_chr07	2195622	[G/A]	NA
Ca_Desi_Chr_7_2195623	Ca_desi_chr07	2195623	[G/T]	NA
Ca_Desi_Chr_7_2195792	Ca_desi_chr07	2195792	[A/G]	NA
Ca_Desi_Chr_7_2196288	Ca_desi_chr07	2196288	[T/A]	NA
Ca_Desi_Chr_7_2196542	Ca_desi_chr07	2196542	[T/C]	NA
Ca_Desi_Chr_7_2196580	Ca_desi_chr07	2196580	[A/G]	NA
Ca_Desi_Chr_7_2197057	Ca_desi_chr07	2197057	[G/A]	NA
Ca_Desi_Chr_7_2203200	Ca_desi_chr07	2203200	[T/C]	NA

SNP IDs	Chromosomes	Physical positions (bp)	SNPs	NCBI SNPdb accession IDs (NCBI_ss#)
Ca_Desi_Chr_7_2203203	Ca_desi_chr07	2203203	[T/C]	NA
Ca_Desi_Chr_7_2203210	Ca_desi_chr07	2203210	[T/C]	NA
Ca_Desi_Chr_7_2203215	Ca_desi_chr07	2203215	[T/C]	NA
Ca_Desi_Chr_7_2203225	Ca_desi_chr07	2203225	[A/G]	NA
Ca_Desi_Chr_7_2203249	Ca_desi_chr07	2203249	[A/G]	NA
Ca_Desi_Chr_7_2203291	Ca_desi_chr07	2203291	[T/C]	NA
Ca_Desi_Chr_7_2203320	Ca_desi_chr07	2203320	[A/C]	NA
Ca_Desi_Chr_7_2203348	Ca_desi_chr07	2203348	[A/T]	NA
Ca_Desi_Chr_7_2211434	Ca_desi_chr07	2211434	[T/A]	NA
Ca_Desi_Chr_7_2212373	Ca_desi_chr07	2212373	[A/C]	NA
Ca_Desi_Chr_7_2226248	Ca_desi_chr07	2226248	[A/G]	NA
Ca_Desi_Chr_7_2226279	Ca_desi_chr07	2226279	[C/T]	NA
Ca_Desi_Chr_7_2295278	Ca_desi_chr07	2295278	[T/C]	NA
Ca_Desi_Chr_7_2295281	Ca_desi_chr07	2295281	[T/C]	NA
Ca_Desi_Chr_7_2295317	Ca_desi_chr07	2295317	[A/C]	NA
Ca_Desi_Chr_7_2295320	Ca_desi_chr07	2295320	[T/C]	NA
Ca_Desi_Chr_7_2374483	Ca_desi_chr07	2374483	[A/C]	NA
Ca_Desi_Chr_7_2374492	Ca_desi_chr07	2374492	[A/C]	NA
Ca_Desi_Chr_7_2374502	Ca_desi_chr07	2374502	[G/A]	NA
Ca_Desi_Chr_7_2374524	Ca_desi_chr07	2374524	[A/G]	NA
Ca_Desi_Chr_7_2385449	Ca_desi_chr07	2385449	[A/G]	NA
Ca_Desi_Chr_7_2385453	Ca_desi_chr07	2385453	[A/G]	NA
Ca_Desi_Chr_7_2415032	Ca_desi_chr07	2415032	[T/C]	NA
Ca_Desi_Chr_7_2415035	Ca_desi_chr07	2415035	[T/C]	NA
Ca_Desi_Chr_7_2415045	Ca_desi_chr07	2415045	[A/C]	NA
Ca_Desi_Chr_7_2415050	Ca_desi_chr07	2415050	[A/C]	NA
Ca_Desi_Chr_7_2415055	Ca_desi_chr07	2415055	[T/C]	NA
Ca_Desi_Chr_7_2415067	Ca_desi_chr07	2415067	[A/G]	NA
Ca_Desi_Chr_7_2415068	Ca_desi_chr07	2415068	[A/G]	NA
Ca_Desi_Chr_7_2415070	Ca_desi_chr07	2415070	[T/A]	NA
Ca_Desi_Chr_7_2415075	Ca_desi_chr07	2415075	[A/G]	NA
Ca_Desi_Chr_7_2434160	Ca_desi_chr07	2434160	[G/T]	NA
Ca_Desi_Chr_7_2541486	Ca_desi_chr07	2541486	[T/G]	NA
Ca_Desi_Chr_7_2569346	Ca_desi_chr07	2569346	[A/G]	NA

SNP IDs	Chromosomes	Physical positions (bp)	SNPs	NCBI SNPdb accession IDs (NCBI_ss#)
Ca_Desi_Chr_7_2569348	Ca_desi_chr07	2569348	[T/A]	NA
Ca_Desi_Chr_7_2570445	Ca_desi_chr07	2570445	[C/T]	NA
Ca_Desi_Chr_7_2570459	Ca_desi_chr07	2570459	[A/C]	NA
Ca_Desi_Chr_7_2570483	Ca_desi_chr07	2570483	[G/T]	NA
Ca_Desi_Chr_7_2570489	Ca_desi_chr07	2570489	[C/T]	NA
Ca_Desi_Chr_7_2683264	Ca_desi_chr07	2683264	[T/C]	NA
Ca_Desi_Chr_7_2683272	Ca_desi_chr07	2683272	[A/C]	NA
Ca_Desi_Chr_7_2683275	Ca_desi_chr07	2683275	[A/G]	NA
Ca_Desi_Chr_7_2683284	Ca_desi_chr07	2683284	[T/A]	NA
Ca_Desi_Chr_7_2683308	Ca_desi_chr07	2683308	[A/G]	NA
Ca_Desi_Chr_7_2683825	Ca_desi_chr07	2683825	[A/C]	NA
Ca_Desi_Chr_7_2683837	Ca_desi_chr07	2683837	[A/C]	NA
Ca_Desi_Chr_7_2686437	Ca_desi_chr07	2686437	[A/C]	NA
Ca_Desi_Chr_7_2686440	Ca_desi_chr07	2686440	[A/C]	NA
Ca_Desi_Chr_7_2705539	Ca_desi_chr07	2705539	[A/C]	NA
Ca_Desi_Chr_7_2705542	Ca_desi_chr07	2705542	[A/C]	NA
Ca_Desi_Chr_7_2705550	Ca_desi_chr07	2705550	[G/A]	NA
Ca_Desi_Chr_7_2707626	Ca_desi_chr07	2707626	[T/C]	NA
Ca_Desi_Chr_7_2707632	Ca_desi_chr07	2707632	[T/C]	NA
Ca_Desi_Chr_7_2707637	Ca_desi_chr07	2707637	[A/C]	NA
Ca_Desi_Chr_7_2707640	Ca_desi_chr07	2707640	[A/C]	NA
Ca_Desi_Chr_7_2707647	Ca_desi_chr07	2707647	[T/C]	NA
Ca_Desi_Chr_7_2707674	Ca_desi_chr07	2707674	[T/C]	NA
Ca_Desi_Chr_7_2707708	Ca_desi_chr07	2707708	[T/C]	NA
Ca_Desi_Chr_7_2707720	Ca_desi_chr07	2707720	[G/T]	NA
Ca_Desi_Chr_7_2707722	Ca_desi_chr07	2707722	[G/T]	NA
Ca_Desi_Chr_7_2707735	Ca_desi_chr07	2707735	[A/G]	NA
Ca_Desi_Chr_7_2707763	Ca_desi_chr07	2707763	[A/G]	NA
Ca_Desi_Chr_7_2736407	Ca_desi_chr07	2736407	[T/C]	NA
Ca_Desi_Chr_7_2736428	Ca_desi_chr07	2736428	[A/C]	NA
Ca_Desi_Chr_7_2754623	Ca_desi_chr07	2754623	[A/C]	NA
Ca_Desi_Chr_7_2789427	Ca_desi_chr07	2789427	[A/G]	NA
Ca_Desi_Chr_7_2889402	Ca_desi_chr07	2889402	[A/C]	NA
Ca_Desi_Chr_7_2889547	Ca_desi_chr07	2889547	[A/C]	NA

SNP IDs	Chromosomes	Physical positions (bp)	SNPs	NCBI SNPdb accession IDs (NCBI_ss#)
Ca_Desi_Chr_7_2889767	Ca_desi_chr07	2889767	[A/G]	NA
Ca_Desi_Chr_7_2895784	Ca_desi_chr07	2895784	[T/G]	NA
Ca_Desi_Chr_7_2925738	Ca_desi_chr07	2925738	[T/G]	NA
Ca_Desi_Chr_7_2944464	Ca_desi_chr07	2944464	[T/G]	NA
Ca_Desi_Chr_7_2945921	Ca_desi_chr07	2945921	[T/C]	NA
Ca_Desi_Chr_7_2945963	Ca_desi_chr07	2945963	[C/T]	NA
Ca_Desi_Chr_7_2952198	Ca_desi_chr07	2952198	[C/T]	NA
Ca_Desi_Chr_7_2967020	Ca_desi_chr07	2967020	[T/C]	NA
Ca_Desi_Chr_7_2967057	Ca_desi_chr07	2967057	[T/C]	NA
Ca_Desi_Chr_7_2967079	Ca_desi_chr07	2967079	[A/G]	NA
Ca_Desi_Chr_7_2967086	Ca_desi_chr07	2967086	[A/C]	NA
Ca_Desi_Chr_7_3005751	Ca_desi_chr07	3005751	[A/G]	NA
Ca_Desi_Chr_7_3007471	Ca_desi_chr07	3007471	[A/T]	NA
Ca_Desi_Chr_7_3123550	Ca_desi_chr07	3123550	[T/A]	NA
Ca_Desi_Chr_7_3123632	Ca_desi_chr07	3123632	[A/C]	NA
Ca_Desi_Chr_7_3123729	Ca_desi_chr07	3123729	[T/G]	NA
Ca_Desi_Chr_7_3124015	Ca_desi_chr07	3124015	[A/C]	NA
Ca_Desi_Chr_7_3135128	Ca_desi_chr07	3135128	[A/G]	NA
Ca_Desi_Chr_7_3136837	Ca_desi_chr07	3136837	[T/G]	NA
Ca_Desi_Chr_7_3150114	Ca_desi_chr07	3150114	[A/T]	NA
Ca_Desi_Chr_7_3150159	Ca_desi_chr07	3150159	[A/C]	NA
Ca_Desi_Chr_7_3150165	Ca_desi_chr07	3150165	[A/G]	NA
Ca_Desi_Chr_7_3167829	Ca_desi_chr07	3167829	[T/A]	NA
Ca_Desi_Chr_7_3184049	Ca_desi_chr07	3184049	[T/A]	NA
Ca_Desi_Chr_7_3386851	Ca_desi_chr07	3386851	[T/G]	NA
Ca_Desi_Chr_7_3409216	Ca_desi_chr07	3409216	[T/C]	NA
Ca_Desi_Chr_7_3481205	Ca_desi_chr07	3481205	[C/A]	NA
Ca_Desi_Chr_7_3481220	Ca_desi_chr07	3481220	[T/C]	NA
Ca_Desi_Chr_7_3481243	Ca_desi_chr07	3481243	[T/C]	NA
Ca_Desi_Chr_7_3481247	Ca_desi_chr07	3481247	[T/G]	NA
Ca_Desi_Chr_7_3481267	Ca_desi_chr07	3481267	[T/A]	NA
Ca_Desi_Chr_7_3483183	Ca_desi_chr07	3483183	[A/G]	NA
Ca_Desi_Chr_7_3483311	Ca_desi_chr07	3483311	[A/C]	NA
Ca_Desi_Chr_7_3485064	Ca_desi_chr07	3485064	[A/G]	NA

SNP IDs	Chromosomes	Physical positions (bp)	SNPs	NCBI SNPdb accession IDs (NCBI_ss#)
Ca_Desi_Chr_7_3485566	Ca_desi_chr07	3485566	[T/C]	NA
Ca_Desi_Chr_7_3485583	Ca_desi_chr07	3485583	[A/G]	NA
Ca_Desi_Chr_7_3485586	Ca_desi_chr07	3485586	[A/G]	NA
Ca_Desi_Chr_7_3485592	Ca_desi_chr07	3485592	[T/A]	NA
Ca_Desi_Chr_7_3485594	Ca_desi_chr07	3485594	[T/C]	NA
Ca_Desi_Chr_7_3485626	Ca_desi_chr07	3485626	[G/A]	NA
Ca_Desi_Chr_7_3485664	Ca_desi_chr07	3485664	[T/C]	NA
Ca_Desi_Chr_7_3485666	Ca_desi_chr07	3485666	[A/G]	NA
Ca_Desi_Chr_7_3485674	Ca_desi_chr07	3485674	[T/C]	NA
Ca_Desi_Chr_7_3528194	Ca_desi_chr07	3528194	[A/G]	NA
Ca_Desi_Chr_7_3528202	Ca_desi_chr07	3528202	[A/G]	NA
Ca_Desi_Chr_7_3528208	Ca_desi_chr07	3528208	[A/G]	NA
Ca_Desi_Chr_7_3528212	Ca_desi_chr07	3528212	[T/C]	NA
Ca_Desi_Chr_7_3528229	Ca_desi_chr07	3528229	[A/C]	NA
Ca_Desi_Chr_7_3528235	Ca_desi_chr07	3528235	[T/C]	NA
Ca_Desi_Chr_7_3528237	Ca_desi_chr07	3528237	[A/G]	NA
Ca_Desi_Chr_7_3529252	Ca_desi_chr07	3529252	[T/C]	NA
Ca_Desi_Chr_7_3529337	Ca_desi_chr07	3529337	[A/C]	NA
Ca_Desi_Chr_7_3529364	Ca_desi_chr07	3529364	[A/G]	NA
Ca_Desi_Chr_7_3675235	Ca_desi_chr07	3675235	[T/G]	NA
Ca_Desi_Chr_7_3675434	Ca_desi_chr07	3675434	[G/C]	NA
Ca_Desi_Chr_7_3683741	Ca_desi_chr07	3683741	[C/T]	NA
Ca_Desi_Chr_7_3771421	Ca_desi_chr07	3771421	[A/C]	NA
Ca_Desi_Chr_7_3771425	Ca_desi_chr07	3771425	[G/A]	NA
Ca_Desi_Chr_7_3771426	Ca_desi_chr07	3771426	[G/A]	NA
Ca_Desi_Chr_7_3826692	Ca_desi_chr07	3826692	[T/C]	NA
Ca_Desi_Chr_7_3826722	Ca_desi_chr07	3826722	[A/C]	NA
Ca_Desi_Chr_7_3826724	Ca_desi_chr07	3826724	[A/C]	NA
Ca_Desi_Chr_7_3826751	Ca_desi_chr07	3826751	[A/C]	NA
Ca_Desi_Chr_7_3949766	Ca_desi_chr07	3949766	[T/C]	NA
Ca_Desi_Chr_7_3970206	Ca_desi_chr07	3970206	[A/G]	NA
Ca_Desi_Chr_7_4043690	Ca_desi_chr07	4043690	[A/G]	NA
Ca_Desi_Chr_7_4043692	Ca_desi_chr07	4043692	[T/G]	NA
Ca_Desi_Chr_7_4043704	Ca_desi_chr07	4043704	[A/G]	NA

SNP IDs	Chromosomes	Physical positions (bp)	SNPs	NCBI SNPdb accession IDs (NCBI_ss#)
Ca_Desi_Chr_7_4120102	Ca_desi_chr07	4120102	[T/G]	NA
Ca_Desi_Chr_7_4130618	Ca_desi_chr07	4130618	[A/C]	NA
Ca_Desi_Chr_7_4130746	Ca_desi_chr07	4130746	[G/A]	NA
Ca_Desi_Chr_7_4130747	Ca_desi_chr07	4130747	[A/T]	NA
Ca_Desi_Chr_7_4130748	Ca_desi_chr07	4130748	[G/C]	NA
Ca_Desi_Chr_7_4130749	Ca_desi_chr07	4130749	[G/T]	NA
Ca_Desi_Chr_7_4142662	Ca_desi_chr07	4142662	[A/C]	NA
Ca_Desi_Chr_7_4226398	Ca_desi_chr07	4226398	[T/G]	NA
Ca_Desi_Chr_7_4276890	Ca_desi_chr07	4276890	[A/C]	NA
Ca_Desi_Chr_7_4370417	Ca_desi_chr07	4370417	[T/G]	NA
Ca_Desi_Chr_7_4370418	Ca_desi_chr07	4370418	[G/A]	NA
Ca_Desi_Chr_7_4370420	Ca_desi_chr07	4370420	[A/C]	NA
Ca_Desi_Chr_7_4370421	Ca_desi_chr07	4370421	[C/G]	NA
Ca_Desi_Chr_7_4370423	Ca_desi_chr07	4370423	[C/A]	NA
Ca_Desi_Chr_7_4479869	Ca_desi_chr07	4479869	[A/G]	NA
Ca_Desi_Chr_7_4479871	Ca_desi_chr07	4479871	[A/C]	NA
Ca_Desi_Chr_7_4597378	Ca_desi_chr07	4597378	[T/C]	NA
Ca_Desi_Chr_7_4597380	Ca_desi_chr07	4597380	[T/C]	NA
Ca_Desi_Chr_7_4597385	Ca_desi_chr07	4597385	[A/G]	NA
Ca_Desi_Chr_7_4597392	Ca_desi_chr07	4597392	[A/G]	NA
Ca_Desi_Chr_7_4597399	Ca_desi_chr07	4597399	[T/G]	NA
Ca_Desi_Chr_7_4597406	Ca_desi_chr07	4597406	[A/G]	NA
Ca_Desi_Chr_7_4597417	Ca_desi_chr07	4597417	[A/G]	NA
Ca_Desi_Chr_7_4729239	Ca_desi_chr07	4729239	[T/C]	NA
Ca_Desi_Chr_7_4729243	Ca_desi_chr07	4729243	[T/C]	NA
Ca_Desi_Chr_7_4729251	Ca_desi_chr07	4729251	[T/C]	NA
Ca_Desi_Chr_7_4734260	Ca_desi_chr07	4734260	[A/T]	NA
Ca_Desi_Chr_7_4734264	Ca_desi_chr07	4734264	[G/C]	NA
Ca_Desi_Chr_7_4734265	Ca_desi_chr07	4734265	[A/T]	NA
Ca_Desi_Chr_7_4734268	Ca_desi_chr07	4734268	[T/C]	NA
Ca_Desi_Chr_7_4734278	Ca_desi_chr07	4734278	[T/C]	NA
Ca_Desi_Chr_7_4734291	Ca_desi_chr07	4734291	[T/C]	NA
Ca_Desi_Chr_7_4734292	Ca_desi_chr07	4734292	[C/T]	NA
Ca_Desi_Chr_7_4734296	Ca_desi_chr07	4734296	[T/C]	NA

SNP IDs	Chromosomes	Physical positions (bp)	SNPs	NCBI SNPdb accession IDs (NCBI_ss#)
Ca_Desi_Chr_7_4734305	Ca_desi_chr07	4734305	[T/A]	NA
Ca_Desi_Chr_7_4940454	Ca_desi_chr07	4940454	[G/A]	NA
Ca_Desi_Chr_7_4940521	Ca_desi_chr07	4940521	[T/G]	NA
Ca_Desi_Chr_7_4997761	Ca_desi_chr07	4997761	[T/C]	NA
Ca_Desi_Chr_7_4997784	Ca_desi_chr07	4997784	[T/C]	NA
Ca_Desi_Chr_7_4997800	Ca_desi_chr07	4997800	[T/G]	NA
Ca_Desi_Chr_7_4997818	Ca_desi_chr07	4997818	[T/C]	NA
Ca_Desi_Chr_7_5036733	Ca_desi_chr07	5036733	[C/A]	NA
Ca_Desi_Chr_7_5036746	Ca_desi_chr07	5036746	[T/G]	NA
Ca_Desi_Chr_7_5036756	Ca_desi_chr07	5036756	[A/G]	NA
Ca_Desi_Chr_7_5036766	Ca_desi_chr07	5036766	[T/G]	NA
Ca_Desi_Chr_7_5036769	Ca_desi_chr07	5036769	[A/G]	NA
Ca_Desi_Chr_7_5036776	Ca_desi_chr07	5036776	[T/G]	NA
Ca_Desi_Chr_7_5036781	Ca_desi_chr07	5036781	[T/G]	NA
Ca_Desi_Chr_7_5091350	Ca_desi_chr07	5091350	[A/G]	NA
Ca_Desi_Chr_7_5097661	Ca_desi_chr07	5097661	[T/A]	NA
Ca_Desi_Chr_7_5097710	Ca_desi_chr07	5097710	[T/C]	NA
Ca_Desi_Chr_7_5097721	Ca_desi_chr07	5097721	[T/C]	NA
Ca_Desi_Chr_7_5097740	Ca_desi_chr07	5097740	[T/C]	NA
Ca_Desi_Chr_7_5097753	Ca_desi_chr07	5097753	[T/C]	NA
Ca_Desi_Chr_7_5097764	Ca_desi_chr07	5097764	[T/C]	NA
Ca_Desi_Chr_7_5097778	Ca_desi_chr07	5097778	[A/G]	NA
Ca_Desi_Chr_7_5097780	Ca_desi_chr07	5097780	[A/G]	NA
Ca_Desi_Chr_7_5097782	Ca_desi_chr07	5097782	[A/C]	NA
Ca_Desi_Chr_7_5137817	Ca_desi_chr07	5137817	[A/C]	NA
Ca_Desi_Chr_7_5137821	Ca_desi_chr07	5137821	[C/T]	NA
Ca_Desi_Chr_7_5138627	Ca_desi_chr07	5138627	[G/A]	NA
Ca_Desi_Chr_7_5139249	Ca_desi_chr07	5139249	[A/G]	NA
Ca_Desi_Chr_7_5139263	Ca_desi_chr07	5139263	[A/G]	NA
Ca_Desi_Chr_7_5139269	Ca_desi_chr07	5139269	[G/A]	NA
Ca_Desi_Chr_7_5230967	Ca_desi_chr07	5230967	[T/C]	NA
Ca_Desi_Chr_7_5230983	Ca_desi_chr07	5230983	[A/G]	NA
Ca_Desi_Chr_7_5231212	Ca_desi_chr07	5231212	[T/A]	NA
Ca_Desi_Chr_7_5332132	Ca_desi_chr07	5332132	[T/G]	NA

SNP IDs	Chromosomes	Physical positions (bp)	SNPs	NCBI SNPdb accession IDs (NCBI_ss#)
Ca_Desi_Chr_7_5338941	Ca_desi_chr07	5338941	[A/G]	NA
Ca_Desi_Chr_7_5338942	Ca_desi_chr07	5338942	[G/A]	NA
Ca_Desi_Chr_7_5338944	Ca_desi_chr07	5338944	[T/C]	NA
Ca_Desi_Chr_7_5338945	Ca_desi_chr07	5338945	[G/T]	NA
Ca_Desi_Chr_7_5353889	Ca_desi_chr07	5353889	[C/A]	NA
Ca_Desi_Chr_7_5584192	Ca_desi_chr07	5584192	[T/A]	NA
Ca_Desi_Chr_7_5601847	Ca_desi_chr07	5601847	[T/G]	NA
Ca_Desi_Chr_7_5601857	Ca_desi_chr07	5601857	[T/G]	NA
Ca_Desi_Chr_7_5601864	Ca_desi_chr07	5601864	[T/G]	NA
Ca_Desi_Chr_7_5601880	Ca_desi_chr07	5601880	[T/G]	NA
Ca_Desi_Chr_7_5617700	Ca_desi_chr07	5617700	[T/C]	NA
Ca_Desi_Chr_7_5617723	Ca_desi_chr07	5617723	[A/G]	NA
Ca_Desi_Chr_7_5729985	Ca_desi_chr07	5729985	[A/C]	NA
Ca_Desi_Chr_7_5729987	Ca_desi_chr07	5729987	[A/G]	NA
Ca_Desi_Chr_7_5768131	Ca_desi_chr07	5768131	[C/T]	NA
Ca_Desi_Chr_7_5804084	Ca_desi_chr07	5804084	[A/C]	NA
Ca_Desi_Chr_7_5882433	Ca_desi_chr07	5882433	[C/A]	NA
Ca_Desi_Chr_7_5882434	Ca_desi_chr07	5882434	[T/G]	NA
Ca_Desi_Chr_7_5882437	Ca_desi_chr07	5882437	[T/G]	NA
Ca_Desi_Chr_7_5885434	Ca_desi_chr07	5885434	[T/G]	NA
Ca_Desi_Chr_7_5885449	Ca_desi_chr07	5885449	[T/C]	NA
Ca_Desi_Chr_7_5885453	Ca_desi_chr07	5885453	[A/C]	NA
Ca_Desi_Chr_7_5885486	Ca_desi_chr07	5885486	[C/T]	NA
Ca_Desi_Chr_7_6078085	Ca_desi_chr07	6078085	[T/C]	NA
Ca_Desi_Chr_7_6078114	Ca_desi_chr07	6078114	[A/C]	NA
Ca_Desi_Chr_7_6093837	Ca_desi_chr07	6093837	[A/G]	NA
Ca_Desi_Chr_7_6093841	Ca_desi_chr07	6093841	[T/C]	NA
Ca_Desi_Chr_7_6093852	Ca_desi_chr07	6093852	[T/C]	NA
Ca_Desi_Chr_7_6093864	Ca_desi_chr07	6093864	[T/G]	NA
Ca_Desi_Chr_7_6093868	Ca_desi_chr07	6093868	[T/C]	NA
Ca_Desi_Chr_7_6093872	Ca_desi_chr07	6093872	[A/C]	NA
Ca_Desi_Chr_7_6093873	Ca_desi_chr07	6093873	[T/C]	NA
Ca_Desi_Chr_7_6093890	Ca_desi_chr07	6093890	[A/G]	NA
Ca_Desi_Chr_7_6093896	Ca_desi_chr07	6093896	[C/G]	NA

SNP IDs	Chromosomes	Physical positions (bp)	SNPs	NCBI SNPdb accession IDs (NCBI_ss#)
Ca_Desi_Chr_7_6099565	Ca_desi_chr07	6099565	[T/C]	NA
Ca_Desi_Chr_7_6099577	Ca_desi_chr07	6099577	[T/C]	NA
Ca_Desi_Chr_7_6099580	Ca_desi_chr07	6099580	[T/G]	NA
Ca_Desi_Chr_7_6099586	Ca_desi_chr07	6099586	[A/G]	NA
Ca_Desi_Chr_7_6099594	Ca_desi_chr07	6099594	[A/G]	NA
Ca_Desi_Chr_7_6099600	Ca_desi_chr07	6099600	[A/G]	NA
Ca_Desi_Chr_7_6099605	Ca_desi_chr07	6099605	[T/C]	NA
Ca_Desi_Chr_7_6099611	Ca_desi_chr07	6099611	[A/G]	NA
Ca_Desi_Chr_7_6099632	Ca_desi_chr07	6099632	[A/G]	NA
Ca_Desi_Chr_7_6136674	Ca_desi_chr07	6136674	[T/A]	NA
Ca_Desi_Chr_7_6179102	Ca_desi_chr07	6179102	[A/C]	NA
Ca_Desi_Chr_7_6252936	Ca_desi_chr07	6252936	[T/C]	NA
Ca_Desi_Chr_7_6252937	Ca_desi_chr07	6252937	[C/A]	NA
Ca_Desi_Chr_7_6252943	Ca_desi_chr07	6252943	[G/A]	NA
Ca_Desi_Chr_7_6439899	Ca_desi_chr07	6439899	[T/G]	NA
Ca_Desi_Chr_7_6441048	Ca_desi_chr07	6441048	[A/G]	NA
Ca_Desi_Chr_7_6441056	Ca_desi_chr07	6441056	[A/G]	NA
Ca_Desi_Chr_7_6441060	Ca_desi_chr07	6441060	[T/A]	NA
Ca_Desi_Chr_7_6441072	Ca_desi_chr07	6441072	[A/G]	NA
Ca_Desi_Chr_7_6441074	Ca_desi_chr07	6441074	[T/G]	NA
Ca_Desi_Chr_7_6441081	Ca_desi_chr07	6441081	[T/C]	NA
Ca_Desi_Chr_7_6441099	Ca_desi_chr07	6441099	[A/G]	NA
Ca_Desi_Chr_7_6441103	Ca_desi_chr07	6441103	[T/C]	NA
Ca_Desi_Chr_7_6441109	Ca_desi_chr07	6441109	[T/C]	NA
Ca_Desi_Chr_7_6450367	Ca_desi_chr07	6450367	[G/A]	NA
Ca_Desi_Chr_7_6483361	Ca_desi_chr07	6483361	[A/C]	NA
Ca_Desi_Chr_7_6484390	Ca_desi_chr07	6484390	[A/C]	NA
Ca_Desi_Chr_7_6484394	Ca_desi_chr07	6484394	[G/T]	NA
Ca_Desi_Chr_7_6529630	Ca_desi_chr07	6529630	[T/G]	NA
Ca_Desi_Chr_7_6537171	Ca_desi_chr07	6537171	[A/T]	NA
Ca_Desi_Chr_7_6537181	Ca_desi_chr07	6537181	[A/C]	NA
Ca_Desi_Chr_7_6537183	Ca_desi_chr07	6537183	[T/C]	NA
Ca_Desi_Chr_7_6537185	Ca_desi_chr07	6537185	[C/T]	NA
Ca_Desi_Chr_7_6537200	Ca_desi_chr07	6537200	[A/G]	NA

SNP IDs	Chromosomes	Physical positions (bp)	SNPs	NCBI SNPdb accession IDs (NCBI_ss#)
Ca_Desi_Chr_7_6537202	Ca_desi_chr07	6537202	[A/G]	NA
Ca_Desi_Chr_7_6537214	Ca_desi_chr07	6537214	[A/G]	NA
Ca_Desi_Chr_7_6537215	Ca_desi_chr07	6537215	[C/G]	NA
Ca_Desi_Chr_7_6605141	Ca_desi_chr07	6605141	[T/C]	NA
Ca_Desi_Chr_7_6605148	Ca_desi_chr07	6605148	[T/C]	NA
Ca_Desi_Chr_7_6605183	Ca_desi_chr07	6605183	[A/C]	NA
Ca_Desi_Chr_7_6605214	Ca_desi_chr07	6605214	[A/G]	NA
Ca_Desi_Chr_7_6627849	Ca_desi_chr07	6627849	[T/G]	NA
Ca_Desi_Chr_7_6627877	Ca_desi_chr07	6627877	[T/A]	NA
Ca_Desi_Chr_7_6678171	Ca_desi_chr07	6678171	[T/C]	NA
Ca_Desi_Chr_7_6730241	Ca_desi_chr07	6730241	[T/A]	NA
Ca_Desi_Chr_7_6736128	Ca_desi_chr07	6736128	[T/C]	NA
Ca_Desi_Chr_7_6950270	Ca_desi_chr07	6950270	[A/C]	NA
Ca_Desi_Chr_7_6950273	Ca_desi_chr07	6950273	[A/C]	NA
Ca_Desi_Chr_7_6950276	Ca_desi_chr07	6950276	[A/C]	NA
Ca_Desi_Chr_7_6950285	Ca_desi_chr07	6950285	[A/C]	NA
Ca_Desi_Chr_7_6950288	Ca_desi_chr07	6950288	[A/C]	NA
Ca_Desi_Chr_7_7078062	Ca_desi_chr07	7078062	[T/G]	NA
Ca_Desi_Chr_7_7078065	Ca_desi_chr07	7078065	[T/G]	NA
Ca_Desi_Chr_7_7078111	Ca_desi_chr07	7078111	[T/C]	NA
Ca_Desi_Chr_7_7078115	Ca_desi_chr07	7078115	[A/C]	NA
Ca_Desi_Chr_7_7078120	Ca_desi_chr07	7078120	[A/G]	NA
Ca_Desi_Chr_7_7083489	Ca_desi_chr07	7083489	[T/G]	NA
Ca_Desi_Chr_7_7176145	Ca_desi_chr07	7176145	[T/G]	NA
Ca_Desi_Chr_7_7214111	Ca_desi_chr07	7214111	[T/C]	NA
Ca_Desi_Chr_7_7222600	Ca_desi_chr07	7222600	[T/A]	NA
Ca_Desi_Chr_7_7232958	Ca_desi_chr07	7232958	[T/G]	NA
Ca_Desi_Chr_7_7234448	Ca_desi_chr07	7234448	[C/G]	NA
Ca_Desi_Chr_7_7266110	Ca_desi_chr07	7266110	[T/C]	NA
Ca_Desi_Chr_7_7266125	Ca_desi_chr07	7266125	[T/C]	NA
Ca_Desi_Chr_7_7266154	Ca_desi_chr07	7266154	[A/T]	NA
Ca_Desi_Chr_7_7266160	Ca_desi_chr07	7266160	[A/G]	NA
Ca_Desi_Chr_7_7266162	Ca_desi_chr07	7266162	[T/A]	NA
Ca_Desi_Chr_7_7266164	Ca_desi_chr07	7266164	[A/G]	NA

SNP IDs	Chromosomes	Physical positions (bp)	SNPs	NCBI SNPdb accession IDs (NCBI_ss#)
Ca_Desi_Chr_7_7266167	Ca_desi_chr07	7266167	[A/G]	NA
Ca_Desi_Chr_7_7266168	Ca_desi_chr07	7266168	[T/G]	NA
Ca_Desi_Chr_7_7266186	Ca_desi_chr07	7266186	[A/G]	NA
Ca_Desi_Chr_7_7266189	Ca_desi_chr07	7266189	[C/G]	NA
Ca_Desi_Chr_7_7266192	Ca_desi_chr07	7266192	[G/A]	NA
Ca_Desi_Chr_7_7266193	Ca_desi_chr07	7266193	[A/G]	NA
Ca_Desi_Chr_7_7266194	Ca_desi_chr07	7266194	[G/A]	NA
Ca_Desi_Chr_7_7266198	Ca_desi_chr07	7266198	[G/C]	NA
Ca_Desi_Chr_7_7266205	Ca_desi_chr07	7266205	[T/C]	NA
Ca_Desi_Chr_7_7266207	Ca_desi_chr07	7266207	[A/G]	NA
Ca_Desi_Chr_7_7266210	Ca_desi_chr07	7266210	[C/G]	NA
Ca_Desi_Chr_7_7488833	Ca_desi_chr07	7488833	[T/C]	NA
Ca_Desi_Chr_7_7488842	Ca_desi_chr07	7488842	[T/A]	NA
Ca_Desi_Chr_7_7497833	Ca_desi_chr07	7497833	[T/C]	NA
Ca_Desi_Chr_7_7497865	Ca_desi_chr07	7497865	[A/G]	NA
Ca_Desi_Chr_7_7497869	Ca_desi_chr07	7497869	[G/A]	NA
Ca_Desi_Chr_7_7688659	Ca_desi_chr07	7688659	[A/T]	NA
Ca_Desi_Chr_7_7688705	Ca_desi_chr07	7688705	[A/G]	NA
Ca_Desi_Chr_7_7688714	Ca_desi_chr07	7688714	[A/C]	NA
Ca_Desi_Chr_7_7688717	Ca_desi_chr07	7688717	[A/G]	NA
Ca_Desi_Chr_7_7688756	Ca_desi_chr07	7688756	[T/C]	NA
Ca_Desi_Chr_7_7688758	Ca_desi_chr07	7688758	[T/C]	NA
Ca_Desi_Chr_7_7688765	Ca_desi_chr07	7688765	[T/C]	NA
Ca_Desi_Chr_7_7750219	Ca_desi_chr07	7750219	[A/G]	NA
Ca_Desi_Chr_7_7750254	Ca_desi_chr07	7750254	[T/C]	NA
Ca_Desi_Chr_7_7750272	Ca_desi_chr07	7750272	[T/C]	NA
Ca_Desi_Chr_7_7750981	Ca_desi_chr07	7750981	[A/G]	NA
Ca_Desi_Chr_7_7751000	Ca_desi_chr07	7751000	[T/G]	NA
Ca_Desi_Chr_7_7751102	Ca_desi_chr07	7751102	[T/G]	NA
Ca_Desi_Chr_7_7751105	Ca_desi_chr07	7751105	[A/G]	NA
Ca_Desi_Chr_7_7751107	Ca_desi_chr07	7751107	[T/G]	NA
Ca_Desi_Chr_7_7751119	Ca_desi_chr07	7751119	[T/G]	NA
Ca_Desi_Chr_7_7796833	Ca_desi_chr07	7796833	[A/T]	NA
Ca_Desi_Chr_7_7796875	Ca_desi_chr07	7796875	[T/C]	NA

SNP IDs	Chromosomes	Physical positions (bp)	SNPs	NCBI SNPdb accession IDs (NCBI_ss#)
Ca_Desi_Chr_7_7796939	Ca_desi_chr07	7796939	[A/C]	NA
Ca_Desi_Chr_7_7796940	Ca_desi_chr07	7796940	[G/A]	NA
Ca_Desi_Chr_7_7810668	Ca_desi_chr07	7810668	[C/T]	NA
Ca_Desi_Chr_7_7829116	Ca_desi_chr07	7829116	[G/T]	NA
Ca_Desi_Chr_7_7869801	Ca_desi_chr07	7869801	[A/C]	NA
Ca_Desi_Chr_7_7895111	Ca_desi_chr07	7895111	[T/G]	NA
Ca_Desi_Chr_7_7895150	Ca_desi_chr07	7895150	[A/C]	NA
Ca_Desi_Chr_7_7895159	Ca_desi_chr07	7895159	[A/G]	NA
Ca_Desi_Chr_7_7895792	Ca_desi_chr07	7895792	[T/C]	NA
Ca_Desi_Chr_7_7895806	Ca_desi_chr07	7895806	[G/A]	NA
Ca_Desi_Chr_7_7895821	Ca_desi_chr07	7895821	[T/C]	NA
Ca_Desi_Chr_7_7895840	Ca_desi_chr07	7895840	[A/G]	NA
Ca_Desi_Chr_7_7895865	Ca_desi_chr07	7895865	[A/G]	NA
Ca_Desi_Chr_7_7895868	Ca_desi_chr07	7895868	[T/C]	NA
Ca_Desi_Chr_7_7913009	Ca_desi_chr07	7913009	[C/T]	NA
Ca_Desi_Chr_7_7945493	Ca_desi_chr07	7945493	[T/G]	NA
Ca_Desi_Chr_7_7945586	Ca_desi_chr07	7945586	[A/T]	NA
Ca_Desi_Chr_7_7947269	Ca_desi_chr07	7947269	[A/G]	NA
Ca_Desi_Chr_7_7947274	Ca_desi_chr07	7947274	[T/A]	NA
Ca_Desi_Chr_7_7947292	Ca_desi_chr07	7947292	[A/C]	NA
Ca_Desi_Chr_7_7947303	Ca_desi_chr07	7947303	[T/C]	NA
Ca_Desi_Chr_7_7980565	Ca_desi_chr07	7980565	[T/G]	NA
Ca_Desi_Chr_7_8015487	Ca_desi_chr07	8015487	[G/C]	NA
Ca_Desi_Chr_7_8016944	Ca_desi_chr07	8016944	[G/A]	NA
Ca_Desi_Chr_7_8021380	Ca_desi_chr07	8021380	[C/T]	NA
Ca_Desi_Chr_7_8021391	Ca_desi_chr07	8021391	[A/T]	NA
Ca_Desi_Chr_7_8097527	Ca_desi_chr07	8097527	[A/C]	NA
Ca_Desi_Chr_7_8113292	Ca_desi_chr07	8113292	[T/G]	NA
Ca_Desi_Chr_7_8180284	Ca_desi_chr07	8180284	[T/A]	NA
Ca_Desi_Chr_7_8180333	Ca_desi_chr07	8180333	[A/C]	NA
Ca_Desi_Chr_7_8181793	Ca_desi_chr07	8181793	[T/G]	NA
Ca_Desi_Chr_7_8181801	Ca_desi_chr07	8181801	[T/G]	NA
Ca_Desi_Chr_7_8181821	Ca_desi_chr07	8181821	[T/C]	NA
Ca_Desi_Chr_7_8187265	Ca_desi_chr07	8187265	[T/C]	NA

SNP IDs	Chromosomes	Physical positions (bp)	SNPs	NCBI SNPdb accession IDs (NCBI_ss#)
Ca_Desi_Chr_7_8206435	Ca_desi_chr07	8206435	[T/C]	NA
Ca_Desi_Chr_7_8206437	Ca_desi_chr07	8206437	[A/G]	NA
Ca_Desi_Chr_7_8206439	Ca_desi_chr07	8206439	[A/G]	NA
Ca_Desi_Chr_7_8206440	Ca_desi_chr07	8206440	[T/C]	NA
Ca_Desi_Chr_7_8206467	Ca_desi_chr07	8206467	[A/G]	NA
Ca_Desi_Chr_7_8206468	Ca_desi_chr07	8206468	[T/C]	NA
Ca_Desi_Chr_7_8206470	Ca_desi_chr07	8206470	[T/C]	NA
Ca_Desi_Chr_7_8206471	Ca_desi_chr07	8206471	[C/A]	NA
Ca_Desi_Chr_7_8206472	Ca_desi_chr07	8206472	[T/C]	NA
Ca_Desi_Chr_7_8209362	Ca_desi_chr07	8209362	[T/A]	NA
Ca_Desi_Chr_7_8209367	Ca_desi_chr07	8209367	[T/C]	NA
Ca_Desi_Chr_7_8209406	Ca_desi_chr07	8209406	[T/G]	NA
Ca_Desi_Chr_7_8209410	Ca_desi_chr07	8209410	[T/G]	NA
Ca_Desi_Chr_7_8209428	Ca_desi_chr07	8209428	[A/G]	NA
Ca_Desi_Chr_7_8209430	Ca_desi_chr07	8209430	[T/G]	NA
Ca_Desi_Chr_7_8209434	Ca_desi_chr07	8209434	[T/G]	NA
Ca_Desi_Chr_7_8209456	Ca_desi_chr07	8209456	[A/C]	NA
Ca_Desi_Chr_7_8210859	Ca_desi_chr07	8210859	[A/C]	NA
Ca_Desi_Chr_7_8343153	Ca_desi_chr07	8343153	[T/G]	NA
Ca_Desi_Chr_7_8343174	Ca_desi_chr07	8343174	[A/C]	NA
Ca_Desi_Chr_7_8369401	Ca_desi_chr07	8369401	[T/C]	NA
Ca_Desi_Chr_7_8369407	Ca_desi_chr07	8369407	[A/C]	NA
Ca_Desi_Chr_7_8369433	Ca_desi_chr07	8369433	[T/C]	NA
Ca_Desi_Chr_7_8369436	Ca_desi_chr07	8369436	[T/G]	NA
Ca_Desi_Chr_7_8369448	Ca_desi_chr07	8369448	[C/A]	NA
Ca_Desi_Chr_7_8369552	Ca_desi_chr07	8369552	[T/A]	NA
Ca_Desi_Chr_7_8369564	Ca_desi_chr07	8369564	[A/G]	NA
Ca_Desi_Chr_7_8387214	Ca_desi_chr07	8387214	[A/G]	NA
Ca_Desi_Chr_7_8387450	Ca_desi_chr07	8387450	[T/C]	NA
Ca_Desi_Chr_7_8387455	Ca_desi_chr07	8387455	[A/G]	NA
Ca_Desi_Chr_7_8387457	Ca_desi_chr07	8387457	[T/C]	NA
Ca_Desi_Chr_7_8387460	Ca_desi_chr07	8387460	[T/G]	NA
Ca_Desi_Chr_7_8387462	Ca_desi_chr07	8387462	[A/G]	NA
Ca_Desi_Chr_7_8387485	Ca_desi_chr07	8387485	[T/A]	NA

SNP IDs	Chromosomes	Physical positions (bp)	SNPs	NCBI SNPdb accession IDs (NCBI_ss#)
Ca_Desi_Chr_7_8409779	Ca_desi_chr07	8409779	[A/C]	NA
Ca_Desi_Chr_7_8413417	Ca_desi_chr07	8413417	[T/C]	NA
Ca_Desi_Chr_7_8429520	Ca_desi_chr07	8429520	[T/G]	NA
Ca_Desi_Chr_7_8441794	Ca_desi_chr07	8441794	[T/G]	NA
Ca_Desi_Chr_7_8441798	Ca_desi_chr07	8441798	[T/G]	NA
Ca_Desi_Chr_7_8441806	Ca_desi_chr07	8441806	[A/C]	NA
Ca_Desi_Chr_7_8452768	Ca_desi_chr07	8452768	[A/C]	NA
Ca_Desi_Chr_7_8452792	Ca_desi_chr07	8452792	[T/C]	NA
Ca_Desi_Chr_7_8452795	Ca_desi_chr07	8452795	[C/T]	NA
Ca_Desi_Chr_7_8452801	Ca_desi_chr07	8452801	[T/C]	NA
Ca_Desi_Chr_7_8452805	Ca_desi_chr07	8452805	[T/C]	NA
Ca_Desi_Chr_7_8452815	Ca_desi_chr07	8452815	[T/C]	NA
Ca_Desi_Chr_7_8452819	Ca_desi_chr07	8452819	[T/C]	NA
Ca_Desi_Chr_7_8452822	Ca_desi_chr07	8452822	[T/C]	NA
Ca_Desi_Chr_7_8454777	Ca_desi_chr07	8454777	[T/C]	NA
Ca_Desi_Chr_7_8454791	Ca_desi_chr07	8454791	[A/G]	NA
Ca_Desi_Chr_7_8454808	Ca_desi_chr07	8454808	[T/C]	NA
Ca_Desi_Chr_7_8454812	Ca_desi_chr07	8454812	[T/C]	NA
Ca_Desi_Chr_7_8454819	Ca_desi_chr07	8454819	[T/C]	NA
Ca_Desi_Chr_8_32673	Ca_desi_chr08	32673	[T/G]	NA
Ca_Desi_Chr_8_65238	Ca_desi_chr08	65238	[T/C]	NA
Ca_Desi_Chr_8_65261	Ca_desi_chr08	65261	[T/G]	NA
Ca_Desi_Chr_8_90332	Ca_desi_chr08	90332	[G/A]	NA
Ca_Desi_Chr_8_90334	Ca_desi_chr08	90334	[T/A]	NA
Ca_Desi_Chr_8_115690	Ca_desi_chr08	115690	[A/C]	NA
Ca_Desi_Chr_8_122422	Ca_desi_chr08	122422	[T/G]	NA
Ca_Desi_Chr_8_134344	Ca_desi_chr08	134344	[T/C]	NA
Ca_Desi_Chr_8_134410	Ca_desi_chr08	134410	[A/C]	NA
Ca_Desi_Chr_8_145480	Ca_desi_chr08	145480	[T/C]	NA
Ca_Desi_Chr_8_157344	Ca_desi_chr08	157344	[A/C]	NA
Ca_Desi_Chr_8_201411	Ca_desi_chr08	201411	[C/A]	NA
Ca_Desi_Chr_8_201422	Ca_desi_chr08	201422	[C/T]	NA
Ca_Desi_Chr_8_202026	Ca_desi_chr08	202026	[G/C]	NA
Ca_Desi_Chr_8_202036	Ca_desi_chr08	202036	[G/T]	NA

SNP IDs	Chromosomes	Physical positions (bp)	SNPs	NCBI SNPdb accession IDs (NCBI_ss#)
Ca_Desi_Chr_8_210526	Ca_desi_chr08	210526	[C/A]	NA
Ca_Desi_Chr_8_210623	Ca_desi_chr08	210623	[A/C]	NA
Ca_Desi_Chr_8_252816	Ca_desi_chr08	252816	[A/T]	NA
Ca_Desi_Chr_8_277065	Ca_desi_chr08	277065	[T/C]	NA
Ca_Desi_Chr_8_298428	Ca_desi_chr08	298428	[A/G]	NA
Ca_Desi_Chr_8_298454	Ca_desi_chr08	298454	[T/A]	NA
Ca_Desi_Chr_8_332253	Ca_desi_chr08	332253	[A/T]	NA
Ca_Desi_Chr_8_344209	Ca_desi_chr08	344209	[T/G]	NA
Ca_Desi_Chr_8_344217	Ca_desi_chr08	344217	[C/T]	NA
Ca_Desi_Chr_8_344219	Ca_desi_chr08	344219	[C/T]	NA
Ca_Desi_Chr_8_344221	Ca_desi_chr08	344221	[C/T]	NA
Ca_Desi_Chr_8_344229	Ca_desi_chr08	344229	[C/A]	NA
Ca_Desi_Chr_8_344231	Ca_desi_chr08	344231	[A/C]	NA
Ca_Desi_Chr_8_344237	Ca_desi_chr08	344237	[G/A]	NA
Ca_Desi_Chr_8_344981	Ca_desi_chr08	344981	[T/A]	NA
Ca_Desi_Chr_8_355973	Ca_desi_chr08	355973	[A/T]	NA
Ca_Desi_Chr_8_355984	Ca_desi_chr08	355984	[A/G]	NA
Ca_Desi_Chr_8_355989	Ca_desi_chr08	355989	[A/C]	NA
Ca_Desi_Chr_8_356004	Ca_desi_chr08	356004	[T/C]	NA
Ca_Desi_Chr_8_357356	Ca_desi_chr08	357356	[T/G]	NA
Ca_Desi_Chr_8_357383	Ca_desi_chr08	357383	[T/G]	NA
Ca_Desi_Chr_8_357386	Ca_desi_chr08	357386	[T/G]	NA
Ca_Desi_Chr_8_357399	Ca_desi_chr08	357399	[T/G]	NA
Ca_Desi_Chr_8_371628	Ca_desi_chr08	371628	[A/T]	NA
Ca_Desi_Chr_8_379518	Ca_desi_chr08	379518	[T/C]	NA
Ca_Desi_Chr_8_379528	Ca_desi_chr08	379528	[T/C]	NA
Ca_Desi_Chr_8_380499	Ca_desi_chr08	380499	[T/G]	NA
Ca_Desi_Chr_8_393113	Ca_desi_chr08	393113	[T/A]	NA
Ca_Desi_Chr_8_393118	Ca_desi_chr08	393118	[T/A]	NA
Ca_Desi_Chr_8_393178	Ca_desi_chr08	393178	[A/T]	NA
Ca_Desi_Chr_8_393196	Ca_desi_chr08	393196	[T/G]	NA
Ca_Desi_Chr_8_393218	Ca_desi_chr08	393218	[A/G]	NA
Ca_Desi_Chr_8_393236	Ca_desi_chr08	393236	[T/C]	NA
Ca_Desi_Chr_8_397447	Ca_desi_chr08	397447	[A/C]	NA

SNP IDs	Chromosomes	Physical positions (bp)	SNPs	NCBI SNPdb accession IDs (NCBI_ss#)
Ca_Desi_Chr_8_421949	Ca_desi_chr08	421949	[T/G]	NA
Ca_Desi_Chr_8_422053	Ca_desi_chr08	422053	[A/G]	NA
Ca_Desi_Chr_8_422120	Ca_desi_chr08	422120	[T/G]	NA
Ca_Desi_Chr_8_422131	Ca_desi_chr08	422131	[T/G]	NA
Ca_Desi_Chr_8_422154	Ca_desi_chr08	422154	[A/G]	NA
Ca_Desi_Chr_8_427208	Ca_desi_chr08	427208	[A/G]	NA
Ca_Desi_Chr_8_427213	Ca_desi_chr08	427213	[G/T]	NA
Ca_Desi_Chr_8_427216	Ca_desi_chr08	427216	[G/T]	NA
Ca_Desi_Chr_8_427219	Ca_desi_chr08	427219	[A/G]	NA
Ca_Desi_Chr_8_427220	Ca_desi_chr08	427220	[T/C]	NA
Ca_Desi_Chr_8_427223	Ca_desi_chr08	427223	[C/T]	NA
Ca_Desi_Chr_8_427224	Ca_desi_chr08	427224	[T/C]	NA
Ca_Desi_Chr_8_427225	Ca_desi_chr08	427225	[G/T]	NA
Ca_Desi_Chr_8_427230	Ca_desi_chr08	427230	[A/G]	NA
Ca_Desi_Chr_8_427231	Ca_desi_chr08	427231	[C/A]	NA
Ca_Desi_Chr_8_427233	Ca_desi_chr08	427233	[A/C]	NA
Ca_Desi_Chr_8_427238	Ca_desi_chr08	427238	[A/G]	NA
Ca_Desi_Chr_8_427241	Ca_desi_chr08	427241	[A/G]	NA
Ca_Desi_Chr_8_427250	Ca_desi_chr08	427250	[C/G]	NA
Ca_Desi_Chr_8_427257	Ca_desi_chr08	427257	[A/G]	NA
Ca_Desi_Chr_8_427271	Ca_desi_chr08	427271	[A/G]	NA
Ca_Desi_Chr_8_517136	Ca_desi_chr08	517136	[T/C]	NA
Ca_Desi_Chr_8_525562	Ca_desi_chr08	525562	[T/C]	NA
Ca_Desi_Chr_8_525585	Ca_desi_chr08	525585	[T/C]	NA
Ca_Desi_Chr_8_525623	Ca_desi_chr08	525623	[A/C]	NA
Ca_Desi_Chr_8_527253	Ca_desi_chr08	527253	[T/G]	NA
Ca_Desi_Chr_8_527260	Ca_desi_chr08	527260	[T/C]	NA
Ca_Desi_Chr_8_527283	Ca_desi_chr08	527283	[T/C]	NA
Ca_Desi_Chr_8_538485	Ca_desi_chr08	538485	[T/C]	NA
Ca_Desi_Chr_8_538946	Ca_desi_chr08	538946	[T/G]	NA
Ca_Desi_Chr_8_538976	Ca_desi_chr08	538976	[T/G]	NA
Ca_Desi_Chr_8_538979	Ca_desi_chr08	538979	[T/G]	NA
Ca_Desi_Chr_8_553709	Ca_desi_chr08	553709	[A/C]	NA
Ca_Desi_Chr_8_553712	Ca_desi_chr08	553712	[A/T]	NA

SNP IDs	Chromosomes	Physical positions (bp)	SNPs	NCBI SNPdb accession IDs (NCBI_ss#)
Ca_Desi_Chr_8_553714	Ca_desi_chr08	553714	[T/C]	NA
Ca_Desi_Chr_8_553721	Ca_desi_chr08	553721	[A/C]	NA
Ca_Desi_Chr_8_553742	Ca_desi_chr08	553742	[T/G]	NA
Ca_Desi_Chr_8_553752	Ca_desi_chr08	553752	[T/A]	NA
Ca_Desi_Chr_8_556309	Ca_desi_chr08	556309	[A/G]	NA
Ca_Desi_Chr_8_556313	Ca_desi_chr08	556313	[C/T]	NA
Ca_Desi_Chr_8_556314	Ca_desi_chr08	556314	[A/G]	NA
Ca_Desi_Chr_8_556326	Ca_desi_chr08	556326	[A/G]	NA
Ca_Desi_Chr_8_556330	Ca_desi_chr08	556330	[A/G]	NA
Ca_Desi_Chr_8_556338	Ca_desi_chr08	556338	[T/C]	NA
Ca_Desi_Chr_8_562208	Ca_desi_chr08	562208	[A/G]	NA
Ca_Desi_Chr_8_562209	Ca_desi_chr08	562209	[G/A]	NA
Ca_Desi_Chr_8_574607	Ca_desi_chr08	574607	[T/A]	NA
Ca_Desi_Chr_8_574899	Ca_desi_chr08	574899	[A/T]	NA
Ca_Desi_Chr_8_588730	Ca_desi_chr08	588730	[T/C]	NA
Ca_Desi_Chr_8_588738	Ca_desi_chr08	588738	[A/G]	NA
Ca_Desi_Chr_8_588746	Ca_desi_chr08	588746	[A/G]	NA
Ca_Desi_Chr_8_588748	Ca_desi_chr08	588748	[A/G]	NA
Ca_Desi_Chr_8_589887	Ca_desi_chr08	589887	[A/C]	NA
Ca_Desi_Chr_8_595440	Ca_desi_chr08	595440	[A/C]	NA
Ca_Desi_Chr_8_598381	Ca_desi_chr08	598381	[A/C]	NA
Ca_Desi_Chr_8_678765	Ca_desi_chr08	678765	[T/G]	NA
Ca_Desi_Chr_8_684980	Ca_desi_chr08	684980	[A/T]	NA
Ca_Desi_Chr_8_705748	Ca_desi_chr08	705748	[T/C]	NA
Ca_Desi_Chr_8_758815	Ca_desi_chr08	758815	[G/A]	NA
Ca_Desi_Chr_8_758826	Ca_desi_chr08	758826	[T/G]	NA
Ca_Desi_Chr_8_760810	Ca_desi_chr08	760810	[A/G]	NA
Ca_Desi_Chr_8_764969	Ca_desi_chr08	764969	[A/G]	NA
Ca_Desi_Chr_8_764978	Ca_desi_chr08	764978	[T/C]	NA
Ca_Desi_Chr_8_827352	Ca_desi_chr08	827352	[T/C]	NA
Ca_Desi_Chr_8_827358	Ca_desi_chr08	827358	[T/C]	NA
Ca_Desi_Chr_8_827371	Ca_desi_chr08	827371	[T/G]	NA
Ca_Desi_Chr_8_827389	Ca_desi_chr08	827389	[T/C]	NA
Ca_Desi_Chr_8_827393	Ca_desi_chr08	827393	[T/A]	NA

SNP IDs	Chromosomes	Physical positions (bp)	SNPs	NCBI SNPdb accession IDs (NCBI_ss#)
Ca_Desi_Chr_8_839314	Ca_desi_chr08	839314	[A/G]	NA
Ca_Desi_Chr_8_839333	Ca_desi_chr08	839333	[A/G]	NA
Ca_Desi_Chr_8_863059	Ca_desi_chr08	863059	[T/G]	NA
Ca_Desi_Chr_8_863067	Ca_desi_chr08	863067	[T/G]	NA
Ca_Desi_Chr_8_868438	Ca_desi_chr08	868438	[A/G]	NA
Ca_Desi_Chr_8_894731	Ca_desi_chr08	894731	[A/G]	NA
Ca_Desi_Chr_8_935366	Ca_desi_chr08	935366	[T/G]	NA
Ca_Desi_Chr_8_935383	Ca_desi_chr08	935383	[A/C]	NA
Ca_Desi_Chr_8_935388	Ca_desi_chr08	935388	[T/C]	NA
Ca_Desi_Chr_8_984898	Ca_desi_chr08	984898	[A/C]	NA
Ca_Desi_Chr_8_984926	Ca_desi_chr08	984926	[T/A]	NA
Ca_Desi_Chr_8_1017345	Ca_desi_chr08	1017345	[A/T]	NA
Ca_Desi_Chr_8_1041386	Ca_desi_chr08	1041386	[G/C]	NA
Ca_Desi_Chr_8_1056729	Ca_desi_chr08	1056729	[A/G]	NA
Ca_Desi_Chr_8_1056734	Ca_desi_chr08	1056734	[A/C]	NA
Ca_Desi_Chr_8_1056737	Ca_desi_chr08	1056737	[T/C]	NA
Ca_Desi_Chr_8_1056750	Ca_desi_chr08	1056750	[T/C]	NA
Ca_Desi_Chr_8_1073619	Ca_desi_chr08	1073619	[A/G]	NA
Ca_Desi_Chr_8_1073628	Ca_desi_chr08	1073628	[A/G]	NA
Ca_Desi_Chr_8_1073644	Ca_desi_chr08	1073644	[T/C]	NA
Ca_Desi_Chr_8_1073652	Ca_desi_chr08	1073652	[T/A]	NA
Ca_Desi_Chr_8_1073655	Ca_desi_chr08	1073655	[T/G]	NA
Ca_Desi_Chr_8_1073669	Ca_desi_chr08	1073669	[A/T]	NA
Ca_Desi_Chr_8_1073679	Ca_desi_chr08	1073679	[T/C]	NA
Ca_Desi_Chr_8_1352236	Ca_desi_chr08	1352236	[A/C]	NA
Ca_Desi_Chr_8_1352393	Ca_desi_chr08	1352393	[A/C]	NA
Ca_Desi_Chr_8_1365491	Ca_desi_chr08	1365491	[A/C]	NA
Ca_Desi_Chr_8_1365492	Ca_desi_chr08	1365492	[G/T]	NA
Ca_Desi_Chr_8_1365835	Ca_desi_chr08	1365835	[T/G]	NA
Ca_Desi_Chr_8_1386349	Ca_desi_chr08	1386349	[G/T]	NA
Ca_Desi_Chr_8_1386350	Ca_desi_chr08	1386350	[G/A]	NA
Ca_Desi_Chr_8_1386352	Ca_desi_chr08	1386352	[A/C]	NA
Ca_Desi_Chr_8_1386356	Ca_desi_chr08	1386356	[C/A]	NA
Ca_Desi_Chr_8_1386469	Ca_desi_chr08	1386469	[T/G]	NA

SNP IDs	Chromosomes	Physical positions (bp)	SNPs	NCBI SNPdb accession IDs (NCBI_ss#)
Ca_Desi_Chr_8_1388732	Ca_desi_chr08	1388732	[C/T]	NA
Ca_Desi_Chr_8_1388750	Ca_desi_chr08	1388750	[G/A]	NA
Ca_Desi_Chr_8_1399524	Ca_desi_chr08	1399524	[T/G]	NA
Ca_Desi_Chr_8_1399529	Ca_desi_chr08	1399529	[T/G]	NA
Ca_Desi_Chr_8_1399543	Ca_desi_chr08	1399543	[T/G]	NA
Ca_Desi_Chr_8_1399548	Ca_desi_chr08	1399548	[T/G]	NA
Ca_Desi_Chr_8_1399551	Ca_desi_chr08	1399551	[T/G]	NA
Ca_Desi_Chr_8_1399581	Ca_desi_chr08	1399581	[G/A]	NA
Ca_Desi_Chr_8_1399582	Ca_desi_chr08	1399582	[A/G]	NA
Ca_Desi_Chr_8_1399583	Ca_desi_chr08	1399583	[G/T]	NA
Ca_Desi_Chr_8_1413520	Ca_desi_chr08	1413520	[T/C]	NA
Ca_Desi_Chr_8_1413540	Ca_desi_chr08	1413540	[T/C]	NA
Ca_Desi_Chr_8_1413559	Ca_desi_chr08	1413559	[T/C]	NA
Ca_Desi_Chr_8_1428523	Ca_desi_chr08	1428523	[T/A]	NA
Ca_Desi_Chr_8_1430284	Ca_desi_chr08	1430284	[T/G]	NA
Ca_Desi_Chr_8_1430285	Ca_desi_chr08	1430285	[T/A]	NA
Ca_Desi_Chr_8_1430287	Ca_desi_chr08	1430287	[T/C]	NA
Ca_Desi_Chr_8_1430288	Ca_desi_chr08	1430288	[G/T]	NA
Ca_Desi_Chr_8_1431844	Ca_desi_chr08	1431844	[T/C]	NA
Ca_Desi_Chr_8_1431889	Ca_desi_chr08	1431889	[T/G]	NA
Ca_Desi_Chr_8_1431896	Ca_desi_chr08	1431896	[A/G]	NA
Ca_Desi_Chr_8_1431906	Ca_desi_chr08	1431906	[A/C]	NA
Ca_Desi_Chr_8_1443357	Ca_desi_chr08	1443357	[A/C]	NA
Ca_Desi_Chr_8_1474785	Ca_desi_chr08	1474785	[A/C]	NA
Ca_Desi_Chr_8_1474791	Ca_desi_chr08	1474791	[A/C]	NA
Ca_Desi_Chr_8_1474794	Ca_desi_chr08	1474794	[A/C]	NA
Ca_Desi_Chr_8_1474816	Ca_desi_chr08	1474816	[A/C]	NA
Ca_Desi_Chr_8_1474819	Ca_desi_chr08	1474819	[A/C]	NA
Ca_Desi_Chr_8_1516022	Ca_desi_chr08	1516022	[T/G]	NA
Ca_Desi_Chr_8_1516028	Ca_desi_chr08	1516028	[T/C]	NA
Ca_Desi_Chr_8_1516062	Ca_desi_chr08	1516062	[A/C]	NA
Ca_Desi_Chr_8_1526250	Ca_desi_chr08	1526250	[T/C]	NA
Ca_Desi_Chr_8_1526256	Ca_desi_chr08	1526256	[A/T]	NA
Ca_Desi_Chr_8_1526805	Ca_desi_chr08	1526805	[T/A]	NA

SNP IDs	Chromosomes	Physical positions (bp)	SNPs	NCBI SNPdb accession IDs (NCBI_ss#)
Ca_Desi_Chr_8_1557071	Ca_desi_chr08	1557071	[A/G]	NA
Ca_Desi_Chr_8_1557075	Ca_desi_chr08	1557075	[A/G]	NA
Ca_Desi_Chr_8_1557085	Ca_desi_chr08	1557085	[T/G]	NA
Ca_Desi_Chr_8_1557092	Ca_desi_chr08	1557092	[T/G]	NA
Ca_Desi_Chr_8_1557099	Ca_desi_chr08	1557099	[A/G]	NA
Ca_Desi_Chr_8_1561842	Ca_desi_chr08	1561842	[A/G]	NA
Ca_Desi_Chr_8_1561855	Ca_desi_chr08	1561855	[A/C]	NA
Ca_Desi_Chr_8_1575192	Ca_desi_chr08	1575192	[A/G]	NA
Ca_Desi_Chr_8_3412263	Ca_desi_chr08	3412263	[A/G]	NA
Ca_Desi_Chr_8_3482952	Ca_desi_chr08	3482952	[T/G]	NA
Ca_Desi_Chr_8_3482955	Ca_desi_chr08	3482955	[A/G]	NA
Ca_Desi_Chr_8_3495217	Ca_desi_chr08	3495217	[T/C]	NA
Ca_Desi_Chr_8_3497507	Ca_desi_chr08	3497507	[A/C]	NA
Ca_Desi_Chr_8_3498620	Ca_desi_chr08	3498620	[A/T]	NA
Ca_Desi_Chr_8_3499933	Ca_desi_chr08	3499933	[T/G]	NA
Ca_Desi_Chr_8_3499952	Ca_desi_chr08	3499952	[T/G]	NA
Ca_Desi_Chr_8_3499956	Ca_desi_chr08	3499956	[T/G]	NA
Ca_Desi_Chr_8_3500433	Ca_desi_chr08	3500433	[G/T]	NA
Ca_Desi_Chr_8_3500526	Ca_desi_chr08	3500526	[T/C]	NA
Ca_Desi_Chr_8_3500530	Ca_desi_chr08	3500530	[T/G]	NA
Ca_Desi_Chr_8_3500538	Ca_desi_chr08	3500538	[A/G]	NA
Ca_Desi_Chr_8_3607588	Ca_desi_chr08	3607588	[A/C]	NA
Ca_Desi_Chr_8_3649424	Ca_desi_chr08	3649424	[T/C]	NA
Ca_Desi_Chr_8_3649440	Ca_desi_chr08	3649440	[T/G]	NA
Ca_Desi_Chr_8_3657904	Ca_desi_chr08	3657904	[A/C]	NA
Ca_Desi_Chr_8_3659314	Ca_desi_chr08	3659314	[T/G]	NA
Ca_Desi_Chr_8_3659459	Ca_desi_chr08	3659459	[G/A]	NA
Ca_Desi_Chr_8_3660064	Ca_desi_chr08	3660064	[T/C]	NA
Ca_Desi_Chr_8_3677264	Ca_desi_chr08	3677264	[T/C]	NA
Ca_Desi_Chr_8_3677281	Ca_desi_chr08	3677281	[T/A]	NA
Ca_Desi_Chr_8_3692660	Ca_desi_chr08	3692660	[T/C]	NA
Ca_Desi_Chr_8_3692696	Ca_desi_chr08	3692696	[A/T]	NA
Ca_Desi_Chr_8_3692701	Ca_desi_chr08	3692701	[T/C]	NA
Ca_Desi_Chr_8_3692827	Ca_desi_chr08	3692827	[G/C]	NA

SNP IDs	Chromosomes	Physical positions (bp)	SNPs	NCBI SNPdb accession IDs (NCBI_ss#)
Ca_Desi_Chr_8_3697226	Ca_desi_chr08	3697226	[T/C]	NA
Ca_Desi_Chr_8_3719263	Ca_desi_chr08	3719263	[A/G]	NA
Ca_Desi_Chr_8_3738606	Ca_desi_chr08	3738606	[T/A]	NA
Ca_Desi_Chr_8_3741971	Ca_desi_chr08	3741971	[C/T]	NA
Ca_Desi_Chr_8_3741978	Ca_desi_chr08	3741978	[T/G]	NA
Ca_Desi_Chr_8_3742132	Ca_desi_chr08	3742132	[A/C]	NA
Ca_Desi_Chr_8_3747504	Ca_desi_chr08	3747504	[A/C]	NA
Ca_Desi_Chr_8_3747520	Ca_desi_chr08	3747520	[T/C]	NA
Ca_Desi_Chr_8_3747523	Ca_desi_chr08	3747523	[T/C]	NA
Ca_Desi_Chr_8_3747528	Ca_desi_chr08	3747528	[T/C]	NA
Ca_Desi_Chr_8_3747533	Ca_desi_chr08	3747533	[A/T]	NA
Ca_Desi_Chr_8_3804398	Ca_desi_chr08	3804398	[G/T]	NA
Ca_Desi_Chr_8_3804399	Ca_desi_chr08	3804399	[T/C]	NA
Ca_Desi_Chr_8_3804401	Ca_desi_chr08	3804401	[G/C]	NA
Ca_Desi_Chr_8_3804424	Ca_desi_chr08	3804424	[A/C]	NA
Ca_Desi_Chr_8_3804436	Ca_desi_chr08	3804436	[C/T]	NA
Ca_Desi_Chr_8_3804439	Ca_desi_chr08	3804439	[A/G]	NA
Ca_Desi_Chr_8_3810941	Ca_desi_chr08	3810941	[T/C]	NA
Ca_Desi_Chr_8_3810985	Ca_desi_chr08	3810985	[G/A]	NA
Ca_Desi_Chr_8_3877064	Ca_desi_chr08	3877064	[A/G]	NA
Ca_Desi_Chr_8_3889433	Ca_desi_chr08	3889433	[A/G]	NA
Ca_Desi_Chr_8_3936097	Ca_desi_chr08	3936097	[T/C]	NA
Ca_Desi_Chr_8_3946044	Ca_desi_chr08	3946044	[A/C]	NA
Ca_Desi_Chr_8_3959925	Ca_desi_chr08	3959925	[C/T]	NA
Ca_Desi_Chr_8_3959929	Ca_desi_chr08	3959929	[C/T]	NA
Ca_Desi_Chr_8_3959944	Ca_desi_chr08	3959944	[G/T]	NA
Ca_Desi_Chr_8_3960569	Ca_desi_chr08	3960569	[G/T]	NA
Ca_Desi_Chr_8_3960571	Ca_desi_chr08	3960571	[A/G]	NA
Ca_Desi_Chr_8_3960575	Ca_desi_chr08	3960575	[T/G]	NA
Ca_Desi_Chr_8_3972122	Ca_desi_chr08	3972122	[T/A]	NA
Ca_Desi_Chr_8_3972123	Ca_desi_chr08	3972123	[A/G]	NA
Ca_Desi_Chr_8_3972130	Ca_desi_chr08	3972130	[A/G]	NA
Ca_Desi_Chr_8_3972845	Ca_desi_chr08	3972845	[A/G]	NA
Ca_Desi_Chr_8_3986472	Ca_desi_chr08	3986472	[G/T]	NA

SNP IDs	Chromosomes	Physical positions (bp)	SNPs	NCBI SNPdb accession IDs (NCBI_ss#)
Ca_Desi_Chr_8_4010181	Ca_desi_chr08	4010181	[A/G]	NA
Ca_Desi_Chr_8_4010224	Ca_desi_chr08	4010224	[T/G]	NA
Ca_Desi_Chr_8_4028253	Ca_desi_chr08	4028253	[A/G]	NA
Ca_Desi_Chr_8_4060876	Ca_desi_chr08	4060876	[T/G]	NA
Ca_Desi_Chr_8_4066100	Ca_desi_chr08	4066100	[A/G]	NA
Ca_Desi_Chr_8_4069032	Ca_desi_chr08	4069032	[A/C]	NA
Ca_Desi_Chr_8_4069054	Ca_desi_chr08	4069054	[A/G]	NA
Ca_Desi_Chr_8_4115400	Ca_desi_chr08	4115400	[T/C]	NA
Ca_Desi_Chr_8_4126442	Ca_desi_chr08	4126442	[G/T]	NA
Ca_Desi_Chr_8_4126447	Ca_desi_chr08	4126447	[A/C]	NA
Ca_Desi_Chr_8_4180080	Ca_desi_chr08	4180080	[A/C]	NA
Ca_Desi_Chr_8_4180097	Ca_desi_chr08	4180097	[T/C]	NA
Ca_Desi_Chr_8_4185464	Ca_desi_chr08	4185464	[T/C]	NA
Ca_Desi_Chr_8_4187093	Ca_desi_chr08	4187093	[T/A]	NA
Ca_Desi_Chr_8_4187120	Ca_desi_chr08	4187120	[T/A]	NA
Ca_Desi_Chr_8_4208540	Ca_desi_chr08	4208540	[T/G]	NA
Ca_Desi_Chr_8_4299995	Ca_desi_chr08	4299995	[A/G]	NA
Ca_Desi_Chr_8_4300014	Ca_desi_chr08	4300014	[A/C]	NA
Ca_Desi_Chr_8_4300040	Ca_desi_chr08	4300040	[A/C]	NA
Ca_Desi_Chr_8_4302163	Ca_desi_chr08	4302163	[T/G]	NA
Ca_Desi_Chr_8_4310626	Ca_desi_chr08	4310626	[A/C]	NA
Ca_Desi_Chr_8_4341367	Ca_desi_chr08	4341367	[A/C]	NA
Ca_Desi_Chr_8_4341447	Ca_desi_chr08	4341447	[T/G]	NA
Ca_Desi_Chr_8_4341450	Ca_desi_chr08	4341450	[A/G]	NA
Ca_Desi_Chr_8_4341453	Ca_desi_chr08	4341453	[T/G]	NA
Ca_Desi_Chr_8_4341459	Ca_desi_chr08	4341459	[A/G]	NA
Ca_Desi_Chr_8_4341462	Ca_desi_chr08	4341462	[T/G]	NA
Ca_Desi_Chr_8_4385556	Ca_desi_chr08	4385556	[A/G]	NA
Ca_Desi_Chr_8_4385557	Ca_desi_chr08	4385557	[T/C]	NA
Ca_Desi_Chr_8_4385592	Ca_desi_chr08	4385592	[T/C]	NA
Ca_Desi_Chr_8_4385596	Ca_desi_chr08	4385596	[T/C]	NA
Ca_Desi_Chr_8_4448684	Ca_desi_chr08	4448684	[T/G]	NA
Ca_Desi_Chr_8_4448872	Ca_desi_chr08	4448872	[A/G]	NA
Ca_Desi_Chr_8_4448893	Ca_desi_chr08	4448893	[A/G]	NA

SNP IDs	Chromosomes	Physical positions (bp)	SNPs	NCBI SNPdb accession IDs (NCBI_ss#)
Ca_Desi_Chr_8_4448895	Ca_desi_chr08	4448895	[T/C]	NA
Ca_Desi_Chr_8_4448897	Ca_desi_chr08	4448897	[T/C]	NA
Ca_Desi_Chr_8_4448917	Ca_desi_chr08	4448917	[T/G]	NA
Ca_Desi_Chr_8_4448919	Ca_desi_chr08	4448919	[T/C]	NA
Ca_Desi_Chr_8_4461931	Ca_desi_chr08	4461931	[C/T]	NA
Ca_Desi_Chr_8_4574066	Ca_desi_chr08	4574066	[A/C]	NA
Ca_Desi_Chr_8_4597741	Ca_desi_chr08	4597741	[T/G]	NA
Ca_Desi_Chr_8_4597752	Ca_desi_chr08	4597752	[A/T]	NA
Ca_Desi_Chr_8_4650466	Ca_desi_chr08	4650466	[A/G]	NA
Ca_Desi_Chr_8_4650495	Ca_desi_chr08	4650495	[A/G]	NA
Ca_Desi_Chr_8_4650612	Ca_desi_chr08	4650612	[A/C]	NA
Ca_Desi_Chr_8_4650623	Ca_desi_chr08	4650623	[T/C]	NA
Ca_Desi_Chr_8_4651359	Ca_desi_chr08	4651359	[A/C]	NA
Ca_Desi_Chr_8_4664540	Ca_desi_chr08	4664540	[T/C]	NA
Ca_Desi_Chr_8_4664543	Ca_desi_chr08	4664543	[T/C]	NA
Ca_Desi_Chr_8_4670682	Ca_desi_chr08	4670682	[T/C]	NA
Ca_Desi_Chr_8_4722955	Ca_desi_chr08	4722955	[T/C]	NA
Ca_Desi_Chr_8_4722969	Ca_desi_chr08	4722969	[T/C]	NA
Ca_Desi_Chr_8_4722989	Ca_desi_chr08	4722989	[A/G]	NA
Ca_Desi_Chr_8_4750082	Ca_desi_chr08	4750082	[T/G]	NA
Ca_Desi_Chr_8_4750168	Ca_desi_chr08	4750168	[G/T]	NA
Ca_Desi_Chr_8_4750169	Ca_desi_chr08	4750169	[T/G]	NA
Ca_Desi_Chr_8_4750175	Ca_desi_chr08	4750175	[T/A]	NA
Ca_Desi_Chr_8_4802091	Ca_desi_chr08	4802091	[T/G]	NA
Ca_Desi_Chr_8_4802122	Ca_desi_chr08	4802122	[A/C]	NA
Ca_Desi_Chr_8_4822795	Ca_desi_chr08	4822795	[T/C]	NA
Ca_Desi_Chr_8_4822822	Ca_desi_chr08	4822822	[A/C]	NA
Ca_Desi_Chr_8_4822827	Ca_desi_chr08	4822827	[A/G]	NA
Ca_Desi_Chr_8_4822836	Ca_desi_chr08	4822836	[T/C]	NA
Ca_Desi_Chr_8_4845385	Ca_desi_chr08	4845385	[T/G]	NA
Ca_Desi_Chr_8_4845433	Ca_desi_chr08	4845433	[A/C]	NA
Ca_Desi_Chr_8_4845448	Ca_desi_chr08	4845448	[A/C]	NA
Ca_Desi_Chr_8_4848496	Ca_desi_chr08	4848496	[A/G]	NA
Ca_Desi_Chr_8_4848498	Ca_desi_chr08	4848498	[C/G]	NA

SNP IDs	Chromosomes	Physical positions (bp)	SNPs	NCBI SNPdb accession IDs (NCBI_ss#)
Ca_Desi_Chr_8_4848499	Ca_desi_chr08	4848499	[T/G]	NA
Ca_Desi_Chr_8_4852080	Ca_desi_chr08	4852080	[T/C]	NA
Ca_Desi_Chr_8_4867801	Ca_desi_chr08	4867801	[T/G]	NA
Ca_Desi_Chr_8_4867807	Ca_desi_chr08	4867807	[T/G]	NA
Ca_Desi_Chr_8_4867819	Ca_desi_chr08	4867819	[A/G]	NA
Ca_Desi_Chr_8_4867830	Ca_desi_chr08	4867830	[A/G]	NA
Ca_Desi_Chr_8_4867842	Ca_desi_chr08	4867842	[A/G]	NA
Ca_Desi_Chr_8_4867847	Ca_desi_chr08	4867847	[A/G]	NA
Ca_Desi_Chr_8_4954441	Ca_desi_chr08	4954441	[A/G]	NA
Ca_Desi_Chr_8_4954444	Ca_desi_chr08	4954444	[A/C]	NA
Ca_Desi_Chr_8_4954449	Ca_desi_chr08	4954449	[G/C]	NA
Ca_Desi_Chr_8_4954451	Ca_desi_chr08	4954451	[C/A]	NA
Ca_Desi_Chr_8_4954457	Ca_desi_chr08	4954457	[A/G]	NA
Ca_Desi_Chr_8_4954461	Ca_desi_chr08	4954461	[T/C]	NA
Ca_Desi_Chr_8_4954474	Ca_desi_chr08	4954474	[T/C]	NA
Ca_Desi_Chr_8_4954478	Ca_desi_chr08	4954478	[A/G]	NA
Ca_Desi_Chr_8_4954480	Ca_desi_chr08	4954480	[T/G]	NA
Ca_Desi_Chr_8_4954484	Ca_desi_chr08	4954484	[T/C]	NA
Ca_Desi_Chr_8_4954493	Ca_desi_chr08	4954493	[A/C]	NA
Ca_Desi_Chr_8_4954518	Ca_desi_chr08	4954518	[T/G]	NA
Ca_Desi_Chr_8_4954521	Ca_desi_chr08	4954521	[T/C]	NA
Ca_Desi_Chr_8_4971006	Ca_desi_chr08	4971006	[T/G]	NA
Ca_Desi_Chr_8_4971053	Ca_desi_chr08	4971053	[T/C]	NA
Ca_Desi_Chr_8_4982805	Ca_desi_chr08	4982805	[C/A]	NA
Ca_Desi_Chr_8_5040587	Ca_desi_chr08	5040587	[T/G]	NA
Ca_Desi_Chr_8_5113580	Ca_desi_chr08	5113580	[A/G]	NA
Ca_Desi_Chr_8_5159223	Ca_desi_chr08	5159223	[T/A]	NA
Ca_Desi_Chr_8_5159291	Ca_desi_chr08	5159291	[A/C]	NA
Ca_Desi_Chr_8_5174546	Ca_desi_chr08	5174546	[A/C]	NA
Ca_Desi_Chr_8_5174548	Ca_desi_chr08	5174548	[T/G]	NA
Ca_Desi_Chr_8_5174551	Ca_desi_chr08	5174551	[C/G]	NA
Ca_Desi_Chr_8_5177806	Ca_desi_chr08	5177806	[G/T]	NA
Ca_Desi_Chr_8_5185902	Ca_desi_chr08	5185902	[A/C]	NA
Ca_Desi_Chr_8_5186128	Ca_desi_chr08	5186128	[G/T]	NA

SNP IDs	Chromosomes	Physical positions (bp)	SNPs	NCBI SNPdb accession IDs (NCBI_ss#)
Ca_Desi_Chr_8_5205654	Ca_desi_chr08	5205654	[A/G]	NA
Ca_Desi_Chr_8_5205656	Ca_desi_chr08	5205656	[T/C]	NA
Ca_Desi_Chr_8_5205673	Ca_desi_chr08	5205673	[A/C]	NA
Ca_Desi_Chr_8_5232882	Ca_desi_chr08	5232882	[T/G]	NA
Ca_Desi_Chr_8_5248835	Ca_desi_chr08	5248835	[T/C]	NA
Ca_Desi_Chr_8_5253411	Ca_desi_chr08	5253411	[T/C]	NA
Ca_Desi_Chr_8_5253422	Ca_desi_chr08	5253422	[A/C]	NA
Ca_Desi_Chr_8_5253455	Ca_desi_chr08	5253455	[T/C]	NA
Ca_Desi_Chr_8_5253507	Ca_desi_chr08	5253507	[T/C]	NA
Ca_Desi_Chr_8_5253530	Ca_desi_chr08	5253530	[A/C]	NA
Ca_Desi_Chr_8_5253562	Ca_desi_chr08	5253562	[T/C]	NA
Ca_Desi_Chr_8_5271323	Ca_desi_chr08	5271323	[C/A]	NA
Ca_Desi_Chr_8_5294107	Ca_desi_chr08	5294107	[T/C]	NA
Ca_Desi_Chr_8_5294111	Ca_desi_chr08	5294111	[T/G]	NA
Ca_Desi_Chr_8_5319656	Ca_desi_chr08	5319656	[T/G]	NA
Ca_Desi_Chr_8_5319672	Ca_desi_chr08	5319672	[A/G]	NA
Ca_Desi_Chr_8_5319682	Ca_desi_chr08	5319682	[T/A]	NA
Ca_Desi_Chr_8_5325886	Ca_desi_chr08	5325886	[G/T]	NA
Ca_Desi_Chr_8_5390674	Ca_desi_chr08	5390674	[C/A]	NA
Ca_Desi_Chr_8_5390676	Ca_desi_chr08	5390676	[A/T]	NA
Ca_Desi_Chr_8_5406458	Ca_desi_chr08	5406458	[A/G]	NA
Ca_Desi_Chr_8_5407243	Ca_desi_chr08	5407243	[A/G]	NA
Ca_Desi_Chr_8_5412174	Ca_desi_chr08	5412174	[T/A]	NA
Ca_Desi_Chr_8_5412183	Ca_desi_chr08	5412183	[A/G]	NA
Ca_Desi_Chr_8_5412187	Ca_desi_chr08	5412187	[A/G]	NA
Ca_Desi_Chr_8_5412191	Ca_desi_chr08	5412191	[A/T]	NA
Ca_Desi_Chr_8_5412194	Ca_desi_chr08	5412194	[T/C]	NA
Ca_Desi_Chr_8_5412203	Ca_desi_chr08	5412203	[T/C]	NA
Ca_Desi_Chr_8_5412208	Ca_desi_chr08	5412208	[T/C]	NA
Ca_Desi_Chr_8_5412214	Ca_desi_chr08	5412214	[T/C]	NA
Ca_Desi_Chr_8_5412232	Ca_desi_chr08	5412232	[T/C]	NA
Ca_Desi_Chr_8_5430497	Ca_desi_chr08	5430497	[C/T]	NA
Ca_Desi_Chr_8_5430519	Ca_desi_chr08	5430519	[T/C]	NA
Ca_Desi_Chr_8_5430520	Ca_desi_chr08	5430520	[G/C]	NA

SNP IDs	Chromosomes	Physical positions (bp)	SNPs	NCBI SNPdb accession IDs (NCBI_ss#)
Ca_Desi_Chr_8_5431975	Ca_desi_chr08	5431975	[T/G]	NA
Ca_Desi_Chr_8_5432005	Ca_desi_chr08	5432005	[C/G]	NA
Ca_Desi_Chr_8_5478976	Ca_desi_chr08	5478976	[A/C]	NA
Ca_Desi_Chr_8_5478985	Ca_desi_chr08	5478985	[T/C]	NA
Ca_Desi_Chr_8_5479023	Ca_desi_chr08	5479023	[T/G]	NA
Ca_Desi_Chr_8_5560962	Ca_desi_chr08	5560962	[A/G]	NA
Ca_Desi_Chr_8_5577324	Ca_desi_chr08	5577324	[A/C]	NA
Ca_Desi_Chr_8_5598364	Ca_desi_chr08	5598364	[A/C]	NA
Ca_Desi_Chr_8_5598366	Ca_desi_chr08	5598366	[T/C]	NA
Ca_Desi_Chr_8_5598369	Ca_desi_chr08	5598369	[T/C]	NA
Ca_Desi_Chr_8_5644307	Ca_desi_chr08	5644307	[C/T]	NA
Ca_Desi_Chr_8_5760999	Ca_desi_chr08	5760999	[A/C]	NA
Ca_Desi_Chr_8_5761000	Ca_desi_chr08	5761000	[G/T]	NA
Ca_Desi_Chr_8_5779231	Ca_desi_chr08	5779231	[A/C]	NA
Ca_Desi_Chr_8_5834461	Ca_desi_chr08	5834461	[G/A]	NA
Ca_Desi_Chr_8_5857914	Ca_desi_chr08	5857914	[G/A]	NA
Ca_Desi_Chr_8_5859625	Ca_desi_chr08	5859625	[T/A]	NA
Ca_Desi_Chr_8_5859659	Ca_desi_chr08	5859659	[G/T]	NA
Ca_Desi_Chr_8_5859673	Ca_desi_chr08	5859673	[G/A]	NA
Ca_Desi_Chr_8_5860040	Ca_desi_chr08	5860040	[T/C]	NA
Ca_Desi_Chr_8_5860057	Ca_desi_chr08	5860057	[A/G]	NA
Ca_Desi_Chr_8_5860839	Ca_desi_chr08	5860839	[A/T]	NA
Ca_Desi_Chr_8_5860849	Ca_desi_chr08	5860849	[A/C]	NA
Ca_Desi_Chr_8_5860864	Ca_desi_chr08	5860864	[T/C]	NA
Ca_Desi_Chr_8_5861157	Ca_desi_chr08	5861157	[A/C]	NA
Ca_Desi_Chr_8_5873045	Ca_desi_chr08	5873045	[T/G]	NA
Ca_Desi_Chr_8_5873056	Ca_desi_chr08	5873056	[T/G]	NA
Ca_Desi_Chr_8_5893720	Ca_desi_chr08	5893720	[T/C]	NA
Ca_Desi_Chr_8_5893729	Ca_desi_chr08	5893729	[A/C]	NA
Ca_Desi_Chr_8_5893731	Ca_desi_chr08	5893731	[T/C]	NA
Ca_Desi_Chr_8_5893744	Ca_desi_chr08	5893744	[A/C]	NA
Ca_Desi_Chr_8_5893757	Ca_desi_chr08	5893757	[A/C]	NA
Ca_Desi_Chr_8_5894433	Ca_desi_chr08	5894433	[T/A]	NA
Ca_Desi_Chr_8_5896265	Ca_desi_chr08	5896265	[A/T]	NA

SNP IDs	Chromosomes	Physical positions (bp)	SNPs	NCBI SNPdb accession IDs (NCBI_ss#)
Ca_Desi_Chr_8_5898197	Ca_desi_chr08	5898197	[C/T]	NA
Ca_Desi_Chr_8_5898863	Ca_desi_chr08	5898863	[G/A]	NA
Ca_Desi_Chr_8_5902520	Ca_desi_chr08	5902520	[T/C]	NA
Ca_Desi_Chr_8_5927786	Ca_desi_chr08	5927786	[C/T]	NA
Ca_Desi_Chr_8_5937879	Ca_desi_chr08	5937879	[A/C]	NA
Ca_Desi_Chr_8_5937896	Ca_desi_chr08	5937896	[T/C]	NA
Ca_Desi_Chr_8_5937899	Ca_desi_chr08	5937899	[T/C]	NA
Ca_Desi_Chr_8_5937905	Ca_desi_chr08	5937905	[A/C]	NA
Ca_Desi_Chr_8_5937930	Ca_desi_chr08	5937930	[T/C]	NA
Ca_Desi_Chr_8_5937981	Ca_desi_chr08	5937981	[T/G]	NA
Ca_Desi_Chr_8_5937986	Ca_desi_chr08	5937986	[T/C]	NA
Ca_Desi_Chr_8_5955310	Ca_desi_chr08	5955310	[T/G]	NA
Ca_Desi_Chr_8_5955394	Ca_desi_chr08	5955394	[T/G]	NA
Ca_Desi_Chr_8_5997400	Ca_desi_chr08	5997400	[A/C]	NA
Ca_Desi_Chr_8_5997410	Ca_desi_chr08	5997410	[T/C]	NA
Ca_Desi_Chr_8_5997415	Ca_desi_chr08	5997415	[A/C]	NA
Ca_Desi_Chr_8_5997422	Ca_desi_chr08	5997422	[A/C]	NA
Ca_Desi_Chr_8_5997427	Ca_desi_chr08	5997427	[T/C]	NA
Ca_Desi_Chr_8_6011013	Ca_desi_chr08	6011013	[T/A]	NA
Ca_Desi_Chr_8_6011053	Ca_desi_chr08	6011053	[G/A]	NA
Ca_Desi_Chr_8_6017379	Ca_desi_chr08	6017379	[T/C]	NA
Ca_Desi_Chr_8_6018400	Ca_desi_chr08	6018400	[T/A]	NA
Ca_Desi_Chr_8_6018477	Ca_desi_chr08	6018477	[T/A]	NA
Ca_Desi_Chr_8_6018492	Ca_desi_chr08	6018492	[A/G]	NA
Ca_Desi_Chr_8_6019979	Ca_desi_chr08	6019979	[C/T]	NA
Ca_Desi_Chr_8_6021387	Ca_desi_chr08	6021387	[G/C]	NA
Ca_Desi_Chr_8_6034557	Ca_desi_chr08	6034557	[G/A]	NA
Ca_Desi_Chr_8_6042338	Ca_desi_chr08	6042338	[G/A]	NA
Ca_Desi_Chr_8_6058202	Ca_desi_chr08	6058202	[T/C]	NA
Ca_Desi_Chr_8_6058211	Ca_desi_chr08	6058211	[A/G]	NA
Ca_Desi_Chr_8_6058218	Ca_desi_chr08	6058218	[T/G]	NA
Ca_Desi_Chr_8_6058226	Ca_desi_chr08	6058226	[T/G]	NA
Ca_Desi_Chr_8_6058228	Ca_desi_chr08	6058228	[A/G]	NA
Ca_Desi_Chr_8_6058232	Ca_desi_chr08	6058232	[A/G]	NA

SNP IDs	Chromosomes	Physical positions (bp)	SNPs	NCBI SNPdb accession IDs (NCBI_ss#)
Ca_Desi_Chr_8_6058235	Ca_desi_chr08	6058235	[A/G]	NA
Ca_Desi_Chr_8_6122011	Ca_desi_chr08	6122011	[A/C]	NA
Ca_Desi_Chr_8_6122025	Ca_desi_chr08	6122025	[T/C]	NA
Ca_Desi_Chr_8_6122035	Ca_desi_chr08	6122035	[A/G]	NA
Ca_Desi_Chr_8_6122050	Ca_desi_chr08	6122050	[T/C]	NA
Ca_Desi_Chr_8_6122079	Ca_desi_chr08	6122079	[C/T]	NA
Ca_Desi_Chr_8_6122094	Ca_desi_chr08	6122094	[C/G]	NA
Ca_Desi_Chr_8_6125494	Ca_desi_chr08	6125494	[C/T]	NA
Ca_Desi_Chr_8_6135840	Ca_desi_chr08	6135840	[A/T]	NA
Ca_Desi_Chr_8_6138235	Ca_desi_chr08	6138235	[A/G]	NA
Ca_Desi_Chr_8_6180472	Ca_desi_chr08	6180472	[T/C]	NA
Ca_Desi_Chr_8_6180476	Ca_desi_chr08	6180476	[A/G]	NA
Ca_Desi_Chr_8_6180483	Ca_desi_chr08	6180483	[A/G]	NA
Ca_Desi_Chr_8_6180491	Ca_desi_chr08	6180491	[A/G]	NA
Ca_Desi_Chr_8_6212269	Ca_desi_chr08	6212269	[A/C]	NA
Ca_Desi_Chr_8_6212326	Ca_desi_chr08	6212326	[T/G]	NA
Ca_Desi_Chr_8_6212366	Ca_desi_chr08	6212366	[T/C]	NA
Ca_Desi_Chr_8_6212369	Ca_desi_chr08	6212369	[A/G]	NA
Ca_Desi_Chr_8_6218931	Ca_desi_chr08	6218931	[A/G]	NA
Ca_Desi_Chr_8_6222453	Ca_desi_chr08	6222453	[A/G]	NA
Ca_Desi_Chr_8_6222489	Ca_desi_chr08	6222489	[T/A]	NA
Ca_Desi_Chr_8_6222492	Ca_desi_chr08	6222492	[A/G]	NA
Ca_Desi_Chr_8_6222513	Ca_desi_chr08	6222513	[T/A]	NA
Ca_Desi_Chr_8_6222609	Ca_desi_chr08	6222609	[A/G]	NA
Ca_Desi_Chr_8_6222627	Ca_desi_chr08	6222627	[A/G]	NA
Ca_Desi_Chr_8_6222631	Ca_desi_chr08	6222631	[G/A]	NA
Ca_Desi_Chr_8_6222792	Ca_desi_chr08	6222792	[A/C]	NA
Ca_Desi_Chr_8_6224375	Ca_desi_chr08	6224375	[T/C]	NA
Ca_Desi_Chr_8_6224381	Ca_desi_chr08	6224381	[A/G]	NA
[truncated: 554,929 more chars]
